# A unique eukaryotic lineage of composite-like DNA transposons encoding a DDD/E transposase and a His-Me finger homing endonuclease

**DOI:** 10.1186/s13100-022-00281-3

**Published:** 2022-10-22

**Authors:** Kenji K. Kojima, Weidong Bao

**Affiliations:** Genetic Information Research Institute, Cupertino, CA 95014 USA

**Keywords:** DNA transposon, *Kolobok*, *KolobokP*, Long terminal direct repeats (LTDRs), Terminal inverted repeats (TIRs), His-Me finger nuclease, Homing endonuclease (HE)

## Abstract

**Background:**

DNA transposons are ubiquitous components of eukaryotic genomes. A major group of them encode a DDD/E transposase and contain terminal inverted repeats (TIRs) of varying lengths. The *Kolobok* superfamily of DNA transposons has been found in a wide spectrum of organisms.

**Results:**

Here we report a new *Kolobok* lineage, designated *KolobokP*. They were identified in 7 animal phyla (Mollusca, Phoronida, Annelida, Nemertea, Bryozoa, Chordata, and Echinodermata), and are especially rich in bivalves. Unlike other *Kolobok* families, *KolobokP* adopts a composite-like architecture: an internal region (INT) flanked by two long terminal direct repeats (LTDRs), which exhibit their own short terminal inverted repeats ranging up to 18 bps. The excision of LTDRs was strongly suggested. The LTDR lengths seem to be constrained to be either around 450-bp or around 660-bp. The internal region encodes a DDD/E transposase and a small His-Me finger nuclease, which likely originated from the homing endonuclease encoded by a group I intron from a eukaryotic species. The architecture of *KolobokP* resembles composite DNA transposons, usually observed in bacterial genomes, and long terminal repeat (LTR) retrotransposons. In addition to this monomeric LTDR-INT-LTDR structure, plenty of solo LTDRs and multimers represented as (LTDR-INT)_n_-LTDR are also observed. Our structural and phylogenetic analysis supported the birth of *KolobokP* in the late stage of the *Kolobok* evolution. We propose *KolobokP* families propagate themselves in two ways: the canonical transposition catalyzed by their transposase and the sequence-specific cleavage by their endonuclease followed by the multimerization through the unequal crossover.

**Conclusions:**

The presence of homing endonuclease and long terminal direct repeats of *KolobokP* families suggest their unique dual replication mechanisms: transposition and induced unequal crossover.

**Supplementary Information:**

The online version contains supplementary material available at 10.1186/s13100-022-00281-3.

## Background

Transposable elements (TEs), also known as transposons or mobile DNA, include a wide variety of DNA segments that can, in a process called transposition, move or duplicate themselves from one location in the genome to another [1]. Eukaryotic TEs are traditionally divided into two classes: Class I and Class II [2]. Class I TEs, also called retrotransposons, include all TEs that transpose via RNA intermediates. Autonomous retrotransposons encode a reverse transcriptase (RT) for their reverse transcription. Retrotransposons are further divided into several categories, long terminal repeat (LTR) retrotransposons, non-LTR retrotransposons, tyrosine recombinase (YR) retrotransposons (or *DIRS* retrotransposons), and *Penelope*-like elements (PLEs) [3, 4].

Class II TEs are all TEs that are not mobilized with an RNA intermediate, and are also called DNA transposons. DNA transposons known to date encode one of several types of “transposase” proteins, which do not share origins or catalytic mechanisms. The DDD/E (also called DDE) transposase is the most common transposase for both eukaryotic and prokaryotic DNA transposons [5, 6]. DDD/E transposases constitute RNase H fold and contain 3 acidic residues (DDD or DDE) for its catalytic reaction of DNA strand transfer [5]. The integrase domain of LTR retrotransposons and retroviruses is a type of DDD/E transposases. In bacterial genomes, the simplest DNA transposons are called insertion sequences (ISs). Despite their common DDD/E motif, different IS families use different transposition pathways such as “cut-out-paste-in”, “copy-out, paste-in”, and “peel-off, copy-in” [1]. A composite/compound transposon can occasionally be formed out of an internal region and two flanking ISs, being transposable as a discrete unit [7] or dying out due to the excision of flanking ISs [8].

Eukaryotic DNA transposons are classified into 20 or so superfamilies [4, 9]. In the classification system incorporated in Repbase, 20 superfamilies (*Academ*, *Dada*, *EnSpm*, *Ginger1*, *Ginger2*, *Harbinger*, *hAT*, *IS3EU*, *ISL2EU*, *Kolobok*, *Mariner*, *Merlin*, *MuDR*, *Novosib*, *P*, *piggyBac*, *Sola*, *Transib*, *Zator*, *Zisupton*) encode a DDD/E transposase. Some superfamilies share conserved residues in addition to the catalytic DDD/E residues [10]. These conserved sequences are called “signature.” The 5 superfamilies (*hAT*, *P*, *MuDR*, *Kolobok*, and *Dada*) share the motif C/DxxH between the second D and the last E residues [10, 11].

*Kolobok* was first described by Kapitonov and Jurka [12]. *Kolobok* generates TTAA-specific 4-bp target site duplications (TSDs) upon integration. Unlike *piggyBac* transposons, which also generate TTAA-specific TSDs, the termini of *Kolobok* are 5’-RR and YY-3’. *Kolobok* is known to be distributed among animals (chordates, hemichordates, arthropods, nematodes, mollusks, annelids, cnidarians, sponges), fungi, plants (chlorophytes (*Micromonas commoda*), rhodophytes) and protists (*Naegleria*, diatoms (*Fragilariopsis cylindrus*), trichomonads) [9]. The originally reported *Kolobok* families, called the Kol0 group represented by *Kolobok-1_XT*, encode 2 proteins. In addition to the DDD/E transposase, the other protein is designated KolX. *Kolobok* includes several lineages encoding different accessory proteins: *KolobokE* encodes a PD-D/ExK lambda exonuclease-like nuclease downstream of the transposase domain; *KolobokH* encodes a RecQ helicase downstream of the transposase domain and has been found only from fungi. *Kolobok-1_CCri* from a rhodophyte also encodes a RecQ helicase in the complementary strand.

Here we report a new lineage of the *Kolobok* superfamily of DNA transposons and designated it *KolobokP*. Unlike any other DNA transposons, *KolobokP* contains long terminal direct repeats (LTDRs) at both termini, and LTDR has its own terminal inverted repeats (TIRs), resembling the structure of a composite DNA transposon. In its internal region, *KolobokP* encodes a DDD/E transposase and a downstream *I-PpoI*-like His-Me finger nuclease. *I-PpoI* is encoded in a group I intron in the slime mold *Physarum polycephalum* [13], and related homing endonucleases (HEs) are found in group I self-splicing introns inside the nuclear ribosomal RNA genes of fungi and protists [14]. The group of *I-PpoI* is also specifically termed as His-Cys box HEs to reflect its diverged structural and functional features from canonical HNH HEs. Eight conserved histidines (H) and cysteines (C) constitute two zinc-binding motifs functioning in stabilizing the folded protein structure [15, 16]. *KolobokP*-derived solo LTDRs are frequent in the genome and are likely mobilized as a nonautonomous unit. Solo LTDRs could also be generated analogously to solo LTR out of LTR retrotransposons. *KolobokP* also shows tandem arrays in which two neighboring copies share an LTDR. The mechanism for LTDR generation and the function of His-Me finger nucleases in the replication are discussed.

## Results

### A new lineage of Kolobok DNA transposons with long terminal direct repeats (LTDRs)

During the characterization of TEs from the genome of Pacific oyster *Crassostrea gigas*, we found a group of *Kolobok* superfamily of DNA transposons with unusual features. This *Kolobok* group has long terminal direct repeats (LTDRs) at both ends, which themselves contain short (11–18 bp) perfect or imperfect terminal inverted repeats (TIRs). The terminal 2 nucleotides conform with the feature of *Kolobok* families: 5’-RR and YY-3’. We designated them *KolobokP* for “*Kolobok* with Paired ends”. In total, we were able to reconstruct the consensus sequences of 8 autonomous *KolobokP* families which propagated sometime in the past in the Pacific oyster genome (Additional file 1: Table S1). We note that a “family” here represents a group of sequences originated by the propagation of one active TE copy or several closely-related active TE copies in the past. Besides, we found 2 more autonomous families, but could not generate consensus sequences for these families (*KolobokP-9_CGi* and *KolobokP-10_CGi*) due to the lack of enough sequence information. Besides them, 8 nonautonomous *KolobokP* families were also identified. Autonomous *KolobokP* families are between 4,022 bp and 4,748 bp in length, while nonautonomous families are between 1,738 bp and 3,261 bp. We also found another incomplete *KolobokP* family (*KolobokP-11DR_CGi*), only the LTDR part of which we could characterize.

TBLASTN searches against the available genomes with the proteins encoded by *KolobokP* families as queries at the NCBI BLAST server (https://blast.ncbi.nlm.nih.gov/Blast.cgi) and subsequence analysis of respective genomes revealed a wide, but patchy distribution of *KolobokP* families among animals (Table 1 and Additional file 1: Table S1). The sequence identities of transposases among some distant *KolobokP* families are as low as 21%. In total, 7 animal phyla contain at least 1 genome which retains *KolobokP*. The most widely distributed is Mollusca. Almost all analyzed genomes of bivalves contain at least 1 *KolobokP* family. Two early-branched gastropod lineages, Patellogastropoda (true limpets) and Vetigastropoda (abalones) also contain *KolobokP*. Besides Mollusca, *KolobokP* is distributed in Annelida, Phoronida, Nemertea, and Bryozoa among Lophotrochozoa. In Chordata, we found *KolobokP* only from three species of *Branchiostoma.* In Echinodermata, *KolobokP* is distributed in 3 classes. We could find no *KolobokP* family from arthropods or vertebrates although many of their genomes have been sequenced. In total, 228 *KolobokP* families were characterized (Additional file 1: Table S1; Additional file 3: Data S1; Additional file 4: Data S2).

**Table 1:** The distribution of *KolobokP* families

Phylum	Class	Order	Family	Species
Mollusca	Bivalvia	Ostreida	Ostreidae	*Crassostrea gigas* (Pacific oyster)
				*Crassostrea virginica* (eastern oyster)
				*Crassostrea hongkongensis* (Hong Kong oyster)
				*Saccostrea glomerate* (Sydney rock oyster)
		Pteriida	Pteriidae	*Pinctada imbricate* (Akoya pearl oyster)
		Pectinida	Pectinidae	*Pecten maximus* (great scallop)
				*Mizuhopecten yessoensis* (Yesso scallop)
				*Pinna nobilis* (noble pen shell)
		Mytilida	Mytilidae	*Mytilus galloprovincialis* (Mediterranean mussel)
				*Mytilus coruscus* (Korean mussel)
				*Modiolus philippinarum* (Philippine horse mussel)
				*Bathymodiolus platifrons*
				*Perna viridis* (Asian green mussel)
		Arcida	Arcidae	*Tegillarca granosa* (blood clam)
		Adapedonta	Pharidae	*Sinonovacula constricta* (Chinese razor clam)
		Venerida	Veneridae	*Cyclina sinensis* (Chinese venus)
				*Mercenaria mercenaria* (northern quinog)
		Unionida	Unionidae	*Potamilus streckersoni*
				*Margaritifera margaritifera*
				*Megalonaias nervosa*
		Myida	Dreissenidae	*Dreissena rostriformis* (quagga mussel)
				*Dreissena polymorpha* (zebra mussel)
		Adapedonta	Hiatellidae	*Panopea generosa* (Pacific geoduck)
	Gastropoda		Lottiidae	*Lottia gigantea* (owl limpet)
			Peltospiridae	*Gigantopelta aegis*
		Lepetellida	Haliotidae	*Haliotis rubra* (blacklip abalone)
				*Haliotis rufescens* (red abalone)
Phoronida			Phoronidae	*Phoronis australis*
Annelida	Polychaeta	Sabellida	Oweniidae	*Owenia fusiformis*
			Siboglinidae	*Paraescarpia echinospica*
Nemertea	Pilidiophora	Heteronemertea	Lineidae	*Lineus longissimus* (bootlace worm)
Bryozoa	Gymnolaemata	Cheilostomatida	Membraniporidae	*Membranipora membranacea* (Kelp encrusting bryozoan)
Chordata	Leptocardii	Amphioxiformes	Branchiostomidae	*Branchiostoma belcheri* (Belcher’s lancelet)
				*Branchiostoma floridae* (Florida lancelet)
				*Branchiostoma japonicum* (Japanese lancelet)
Echinodermata	Asteroidea	Valvatida	Asterinidae	*Patiria miniata* (bat star)
	Echinoidea	Temnopleuroida	Toxopneustidae	*Lytechinus pictus* (painted urchin)
	Holothuroidea	Aspidochirotida	Stichopodidae	*Apostichopus japonicus* (Japanese sea cucumber)

### The binary distribution of the LTDR lengths

**Fig. 1:**
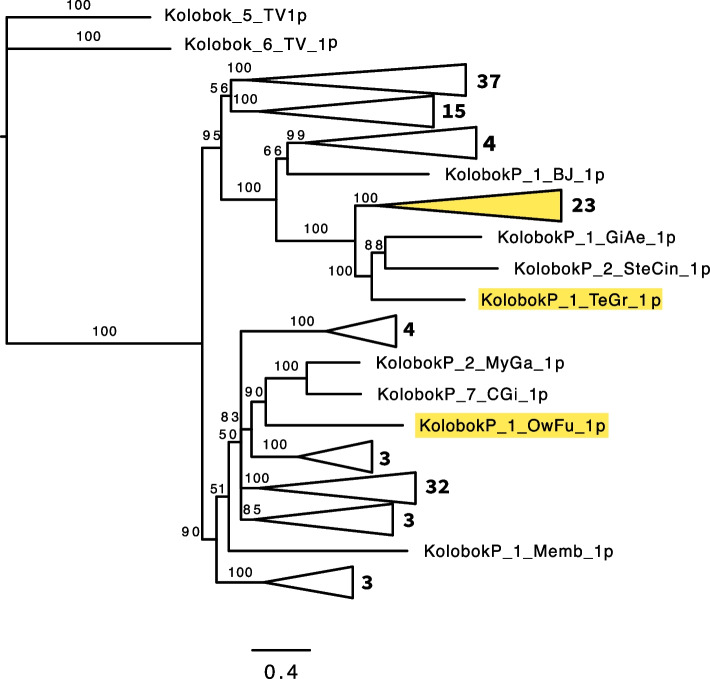
The phylogeny of *KolobokP* DDD/E transposases. The families with ~ 660-bp LTDRs are highlighted in yellow. Well-supported branches are collapsed. The number of the families is indicated on the right of each triangle. The tree is built by MrBayes based on the alignment of the entire sequences of transposases. The transposases from *Kolobok-5_TV* and *Kolobok-6_TV* are used as the outgroup. The sequence alignment and the compete phylogenetic tree are provided as Additional file 3: Data S3 and Additional file 2: Figure S1

Among the 228 autonomous and nonautonomous *KolobokP* families identified from 7 taxonomic groups so far, the length of LTDRs seems to be evolutionally constrained; it is either around 450 bp long or around 660 bp long (Additional file 1: Table S1). Phylogenetic analysis based on the *KolobokP* transposases indicates that ~ 450-bp LTDR, the major type, is the ancestral type, and almost all of those with the ~ 660-bp LTDR are from a single lineage (Fig. 1 and Additional file 2: Figure S1). The singular case of *KolobokP-1_OwFu* may represent a separate case of LTDR shifting from ~ 450-bp to ~ 660-bp. Possibly *KolobokP-1_TeGr* is another separate case of LTDR shifting, as it clustered with 2 *KolobokP* families with ~ 450-bp LTDRs, *KolobokP-1_GiAe* (473-bp LTDRs) and *KolobokP-2_SteCin* (480-bp LTDRs), but it is also possible that *KolobokP-1_GiAe* and *KolobokP-2_SteCin* have shifted their LTDRs from ~ 660-bp to ~ 450-bp.

### Solo LTDRs and multimers are present

A typical *KolobokP* monomer structure can be formulated as LTDR-INT-LTDR, in which INT represents the internal region sandwiched by two LTDRs. However, we found that a substantial number of *KolobokP* insertions are either a solo LTDR or a tandem cluster of monomers (multimer). Both are usually flanked by TSDs. To get the clues of insertion mechanisms of such multimers, we chose 3 genomes of mollusks to analyze the insertion patterns of *KolobokP*, based on the completeness of genome sequencing and the number of *KolobokP* families: *Mytilus coruscus* (Korean mussel), *Mercenaria mercenaria* (northern quahog), and *Gigantopelta aegis*.

Table 2 shows the summary of the structures of *KolobokP* insertions. As expected as an analogy to LTR retrotransposons, solo LTDRs were observed for all analyzed *KolobokP* families. They are mainly flanked by TSDs of 3–4 or 4–5 bp in length, depending on the families. (Additional file 2: Figures S2-S4). Solo LTDR can be derived by eliminating the internal portion of *KolobokP* through the recombination between two LTDRs or unequal crossover between two sister chromatids during replication. Another possibility is that LTDR alone can be excised or copied out and transposed as a discrete nonautonomous transposon, as observed in bacterial composite transposons.

**Table 2:** Patterns of insertions of *KolobokP* families from 3 species of mollusks

TE family	LTDR-INT-LTDR	Solo LTDR	(LTDR-INT)_n_-LTDR	LTDR-(INT)_n_-LTDR
*Mytilus coruscus* (Korean mussel)				
*KolobokP-1_MyCo*	3	2	0	0
*KolobokP-2_MyCo*	7	2	0	0
*KolobokP-3_MyCo*	4	1	0	0
*KolobokP-4_MyCo*	2	2	1	1
*KolobokP-5_MyCo*	2	12	1	0
*Mercenaria mercenaria* (northern quahog)				
*KolobokP-1_MeMe*	50 (39)	96	12 (8)	4 (4)
*KolobokP-2_MeMe*	2	6	3	0
*KolobokP-3_MeMe*	10	9	2	0
*KolobokP-4_MeMe*	3	3	0	0
*KolobokP-5_MeMe*	1	1	0	0
*KolobokP-6_MeMe*	2	7	2	0
*KolobokP-7_MeMe*	4	9	1	1
*Gigantopelta aegis*				
*KolobokP-1_GiAe*	4	7	1	0
*KolobokP-2_GiAe*	3	16	0	0
*KolobokP-3_GiAe*	3	6	1	0
*KolobokP-4_GiAe*	13	23	0	0
*KolobokP-5_GiAe*	7	7	0	0
*KolobokP-6_GiAe*	37	57	0	0
*KolobokP-7_GiAe*	6	30	2	0

Only the insertions with recognizable TSDs are counted. Numbers in parentheses in the line *KolobokP-1_MeMe* is the numbers of insertions with a deletion of the sequence 539–1748 of the internal portion. All insertions counted are shown in Additional file 2: Figures S2-4

Multimers of *KolobokP* are also common. Prototypically, they can be represented as (LTDR-INT)_n_-LTDR, consecutive monomers sharing abutted LTDRs. LTDR is sometimes missing between two consecutive INTs. Compared with the INT sequence from a typical monomer, micro-deletion of 3 or 4 bp is almost always observed at the junction of two abutted INTs, occurring at either tip of the original INT. For example, four insertions of *KolobokP-1_MeMe* showed the structure of LTDR-(INT)_2_-LTDR (2 cases), LTDR-(INT)_2_-LTDR-INT-LTDR, and LTDR-(INT)_4_-LTDR (Additional file 2: Figure S3). The INT of *KolobokP-1_MeMe* starts with TATA and ends with TATA, and in these insertions, only one TATA is observed at the junction of two INTs. One insertion of *KolobokP-4_MyCo* can be represented as LTDR-(INT)_2_-LTDR-INT-LTDR (Additional file 2: Figure S2). One insertion of *KolobokP-7_MeMe* shows the structure of LTDR-(INT)_2_-LTDR-(INT)_2_-LTDR (Additional file 2: Figure S3). The directly abutted INTs (INT-INT) substructures can be explained by an excision event of a solo LTDR or an LTDR-INT-LTDR monomer inside of (LTDR-INT)_n_-LTDR. It is noteworthy that because only insertions with TSDs are counted in the table and long repetitive sequences are not always well assembled, the occurrence of tandem insertions is higher than reflected by the table.

### Solo LTDRs can be excised as nonautonomous transposons

**Fig. 2:**
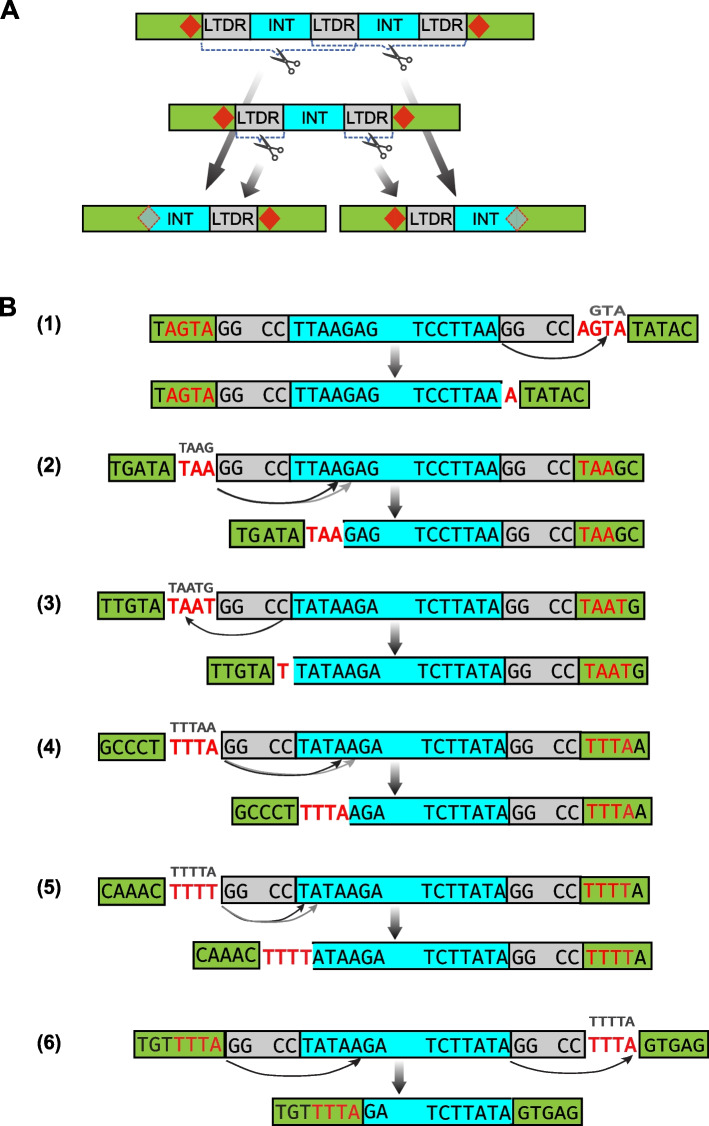
The excision of solo LTDRs. **A** Schematic illustrations of the excision from a dimer (top) or a monomer (middle), which gives rise to the LTDR-INT/INT-LTDR structure (bottom). TSDs are symbolized by red diamonds. **B** Nucleotide “footprint” left in 5 cases of LTDR-INT/INT-LTDR in *KolobokP-7N2_CorFlu* (1–2) and *KolobokP-4N1_CorFlu* (3–5), and one case of “solo INT” in *KolobokP-4N1_CorFlu* (6). Red-colored, unboxed base pairs represent the inferred likely TSDs; other alternative TSDs (gray, smaller font) are marked above. Sequences in green boxes represent the target sequences prior to the insertion of *KolobokP*. The raw sequences of these 6 cases are provided in Additional file 2: Figure S5

To explore the possibility that the INT-INT structure is derived from the excision of an LTDR or a monomer, we focused on two nonautonomous families, *KolobokP-4N1_CorFlu* and *KolobokP-7N2_CorFlu*. The two families are from the genome of Asian clam *Corbicula fluminea*, the assembly of which seems close to being complete [17]. They are relatively young based on their average family sequence diversities (2.3% or 1.3%, Table 3). Each family contains adequate members to enable a comparison of the family components (Table 3). They have similar monomer lengths (2637-bp and 2453-bp, respectively), but their LTDR lengths are distinct (473-bp and 664-bp, respectively). Unexpectedly, in the *KolobokP-4N1_CorFlu* family, 40 cases of INT-LTDR (INT and the right LTDR) and 6 cases of LTDR-INT (the left LTDR and INT) were identified (Table 3). In contrast, only one LTDR-INT and one INT-LTDR occur in the *KolobokP-7N2_CorFlu* family. The two most likely models leading to LTDR-INT and INT-LTDR are the excision of either LTDR of a pre-existed monomer or the excision of a monomer on either side of a dimer (Fig. 2A). The difference between the two models lies in that the latter, but not the former, would degrade a dimer and reduce the percentage of dimer in the multimer group. In contrast, the other types of multimers, containing trimers or above (*n* > 3), are likely more resistant to such degradation in effect, because, theoretically, excision could occur to any two TIRs from the multimer, and if assuming the likelihood is inversely related to the excision lengths. Compared with *KolobokP-7N2_CorFlu*, *KolobokP-4N1_CorFlu* exhibits a large number of LTDR-INT/INT-LTDR (Table 3), the observed percentage of dimers in multimers, however, is quite similar between the two families: 21% (5/24) and 23% (7/30) in *KolobokP-4N1_CorFlu* and *KolobokP-7N2_CorFlu* families, respectively. This data argues in favor of the model that LTDR-INT/INT-LTDR are mainly generated by the excision of a single LTDR from a monomer. From another perspective, this also means that not all solo LTDRs are generated by homologous recombination. Some LTDRs could potentially undergo a “cut-out” transposition process, probably by the activity of transposases. Notably, we identified 3 cases of “solo INT” insertions of *KolobokP-4N1_CorFlu* elements (Table 3 and Fig. 2B), which again probably arose from the sequential removal of LTDRs from a monomer. If these solo INT insertions had been generated by the sequential removal of two monomers from a trimer (LTDR-INT-LTDR-INT-LTDR-INT-LTDR), there should be more transitional copies such as LTDR-INT-LTDR-INT or INT-LTDR-INT-LTDR. The rarity of such structures suggests the transposition of solo LTDRs.

**Table 3:** Comparison of the two nonautonomous *KolobokP* families in the Asian clam *Corbicula fluminea* genome

Family	Diversity	Monomer	Solo LTDR	Multimer*	LTDR-INT	INT-LTDR	INT
*KolobokP-4N1_CorFlu*	2.3%	90	290	24 (5)	6	40	3
*KolobokP-7N2_CorFlu*	1.3%	118	34	30 (7)	1	1	0

^*^Dimer (*n* = 2) is deemed as a multimer (*n* > = 2). The numbers of dimers are indicated in parenthesis

In several cases of LTDR-INT/INT-LTDR, we were able to restore the empty, prior-insertion sequences (Fig. 2B and Additional file 2: Figure S5) since the insertions occurred inside another family of repetitive sequences. By inspecting the sequence at the junctions on both sides, we concluded that the excision event of the *KolobokP* family barely leaves its flank unaffected. Instead, together with the LTDR, 1–5 flanking base pairs on either side of LTDR could be deleted, affecting only one side per event (Fig. 2B). This phenomenon is reminiscent of the transposition pathway and excision in some bacterial IS families, such as IS*3* and IS*256* [18–21]. These IS families use the so-called “copy-out-paste-in” pathway in which a circular DNA intermediate is formed, containing a few extra base pairs from either side of the original transposon at the donor site, spacing the two abutted TIRs. The length of the spacer nucleotides is typically the TSD length of the families [18]. Another remarkable phenomenon observed in the *KolobokP-4N1_CorFlu* family is the far outnumbering of INT-LTDR against LTDR-INT (40 vs 6; Table 3). The mechanism underlying this asymmetrical transposition is unknown. It may involve sequence substitutions at one side of the LTDR in the transposase-binding region, altering the protein binding efficiency.

### KolobokP encodes a DDD/E transposase and a His-Me finger nuclease

Most *KolobokP* families encode two proteins: a DDD/E transposase encoded on the direct strand and a downstream His-Me finger nuclease (also called HNH nuclease) on the complementary strand. Some *KolobokP* families, such as *KolobokP-1_LG* and *KolobokP-1_SaGl*, do not appear to encode a His-Me finger nuclease, but we could not exclude the possibility that it is due to the accumulation of mutations since transposition.

**Fig. 3:**
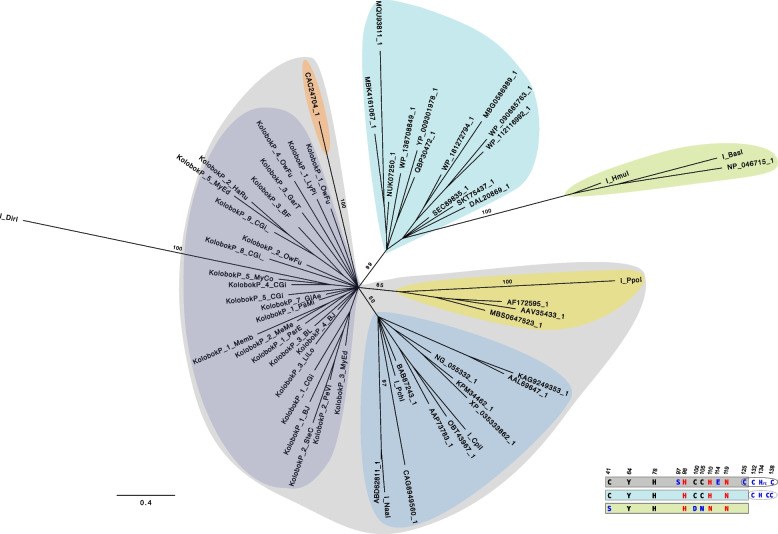
The unrooted Bayesian phylogeny of His-Me finger nucleases. The schematic sequence signatures (bottom-right) of each major group, and the lineages on the tree, are highlighted by the same color code. The positions of the conserved amino acids are indicated above the uppermost sequence bar, according to the sequence of the *I-PpoI* nuclease. The two catalytic residues (H98, N119) and H110 of the His-Me finger motif are marked with red font. The tree is based on the range from H40 to P128 of *I-PpoI*. Most of the C-terminal zinc-binding region is excluded because of the extreme sequence variability. The numbers on the tree indicate the posterior probabilities of the marked branches. The scale bar indicates one substitution/site. The sequence alignment is provided as Additional file 3: Data S4

The HHpred analysis (https://toolkit.tuebingen.mpg.de/tools/hhpred) revealed the His-Me finger nuclease encoded by *KolobokP* families shows the structural similarity to the His-Cys box HEs, *I-PpoI* (1A73) from a slime mold *Physarum polycephalum* and the HNH homing endonuclease *I-HmuI* (1U3E) from the *Bacillus* phage SPO. These 2 HEs represent 2 distinct groups (groups 2 and 12) of His-Me finger nucleases [22]. The group of *I-HmuI* (group 2) is widely distributed among organisms and especially abundant in bacteria and viruses, although the group of *I-PpoI* (group 12) is found only in eukaryotes. The sequence alignment and phylogenetic analysis indicate that *KolobokP* His-Me finger nucleases and other 2 or 3 eukaryotic group I intron-encoded HEs belong to the same major group (Fig. 3 and Additional file 3: Data S3). As reflected by the term “His-Cys box homing endonucleases” applied to *I-PpoI*, they all contain eight conserved histidines (H) and cysteines (C) which form two zinc-binding motifs [15, 16]. In addition to the catalytic motif residues (H98, H110, and N119), S97 and E114 are also conserved in this group (Fig. 3 and Additional file 3: Data S3). The long branch of *I-DirI*, a eukaryotic HE from *Didymium iridis*, probably represents another distant lineage in which several “key” residues were altered, such as Y64F and E114V (Additional file 3: Data S3). Admittedly, the short length of the His-Me finger motif makes it difficult to get a deeper glimpse of the relationship among these eukaryotic HE lineages. Still, this analysis highly suggests a similar function of the *KolobokP*-encoded His-Me finger nucleases as these group I intron-encoded HEs.

### KolobokP is a distinct lineage inside the Kolobok superfamily

**Fig. 4:**
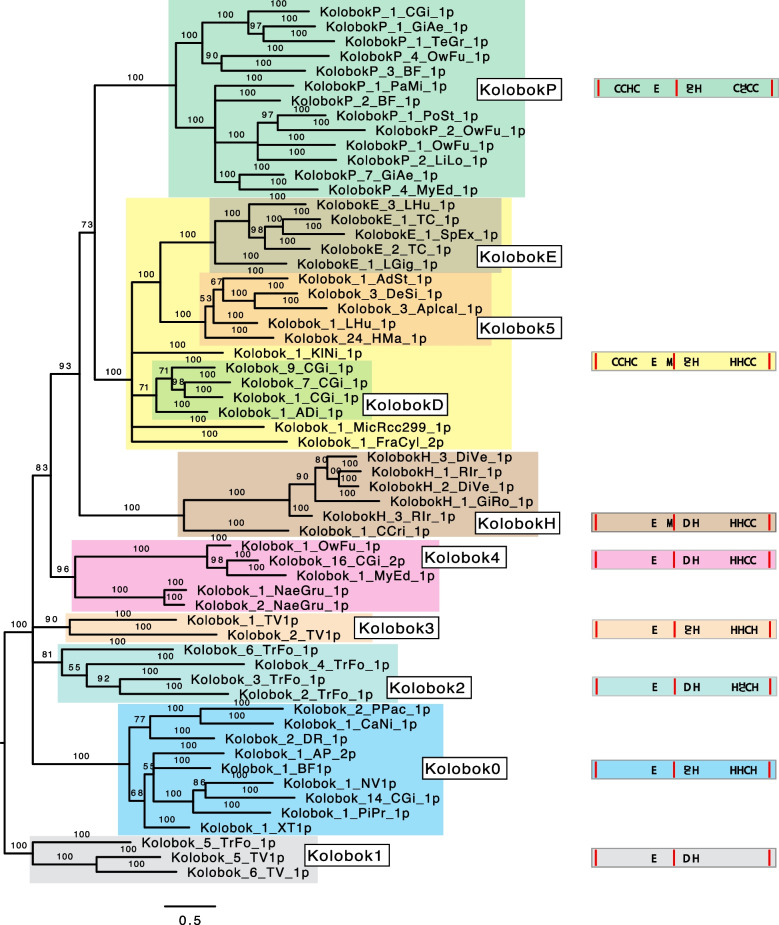
The phylogeny of *Kolobok* families. This Bayesian tree is based on the alignment of DDD/E transposases spanning from the first D to the last catalytic E, inferred using the LG model. The sequence alignment is provided in Additional file 3: Data S5. The root is placed in the *Kolobok1* lineage for its ancestral features: the lack of a zinc finger motif between the C/DxxH motif and the last catalytic residue (E). Some established or obvious lineages are color-marked on the tree. The representative signature strings of each lineage or group are shown on right. Three red vertical lines inside the bar represent the positions of the DDE triad

The phylogenetic analysis based on the DDD/E core region reveals that the *Kolobok* superfamily consists of many diverse lineages (Fig. 4). While some lineages encode only a transposase, several are associated with distinct accessory proteins. Specifically, DEDDh (DnaQ) exonuclease, PD-D/ExK lambda exonuclease-like nuclease, His-Me finger nuclease, RecQ helicase, and KolX protein are encoded in *KolobokD*, *KolobokE*, *KolobokP*, *KolobokH*, and *Kolobok0* lineages, respectively. The DEDDh (DnaQ) exonuclease and the KolX protein are elaborated separately in the following sections.

Sequence alignment and phylogenetic analysis of diverse *Kolobok* members indicate that *Kolobok* transposases had acquired two different zinc finger motifs in the past. The first-acquired zinc finger motif is located between the second (D) and the third catalytic residues (E), but downstream of the conserved C/DxxH signature. This zinc finger occurs in almost all *Kolobok* transposases except the *Kolobok1* lineage (Fig. 4). The third-positioned C of the zinc finger is extremely conserved, but the C/H on the other 3 positions exhibit some variability. The second zinc finger (CCHC) is located downstream of the first catalytic residue (D), and only appears in a few lineages, including *KolobokP*, *KolobokE*, *KolobokD*, *Kolobok5*, and a few solitary families (Fig. 4). Unlike the first-acquired zinc finger motif, the second one barely displays any amino acid variation. The three protozoan members of the *Kolobok1* lineage have neither zinc finger motifs. In other superfamilies closely-related to *Kolobok*, such as *MuDR*, *P*, and *hAT* [10] a zinc finger is also absent between the second and the third catalytic residues. Based on these facts, *Kolobok1* is assumed to be a root in the phylogenetic tree, if the lack of zinc finger is not due to being lost (Fig. 4).

### KolobokD is a new lineage encoding a DEDDh (DnaQ) nuclease

During the search of *KolobokP* from the genomes of mollusks and arthropods, we found another group of *Kolobok* families, which encode a DEDDh (also called DnaQ) exonuclease, and designated them as *KolobokD*. *KolobokD* was found in mollusks, arthropods, and cnidarians. We have not done an extensive survey and thus, the distribution of *KolobokD* would be broader than the present knowledge. The BLASTP and HHpred searches revealed that the DEDDh exonucleases encoded by *KolobokD* families show similarity to maternal protein exuperantia, three prime repair exonuclease (TREX), and ERI1 exoribonuclease. TREX families are 3’-5’ DNA exonucleases, and are known to degrade the *L1* retrotransposition intermediates and inhibit autoimmunity [23]. The DEDDh exonuclease of *KolobokD* would degrade DNA intermediates from their 3’ end during transposition.

### The KolX protein of the Kol0 lineage adopts the cytoplasmic ballast fold

Homology search of KolX proteins encoded by the Kol0 lineage of *Kolobok* families revealed that KolX proteins show clear sequence similarity to the C-terminal part (the region 476–595 in the human protein) of P2X7 purinoceptor (purinergic receptor) (Additional file 2: Figure S6). The region shows a unique structural fold, called cytoplasmic ballast, including 2 zinc ions surrounded by 7 conserved C residues [24]. Our search revealed that a few *piggyBac* families of DNA transposons also encode a protein similar to the C-terminal part of KolX, but they do not have most of the conserved 7 C residues. (Additional file 3: Data S6).

## Discussion

Here, we report *KolobokP*, a unique lineage of *Kolobok* DNA transposons. *KolobokP* contains long terminal direct repeats (LTDRs) at both ends, which contain short inverted repeats in themselves. Our analysis of the *Kolobobk-4N1_CorFlu* family strongly suggests that the LTDR region can be excised and probably be transposable as a nonautonomous transposon. These *KolobokP* features resemble those of so-called composite/compound DNA transposon identified in bacterial genomes. LTR retrotransposons also carry long direct repeats at both ends, but the underlying mechanism differs. Besides, the LTR region has no terminal inverted repeats, except the common TG..CA at the extreme termini.

### The composite nature of KolobokP families

Composite transposons, delineated by identical or similar IS elements in either inverted or direct orientation, are commonly observed in bacterial genomes [7] and are generally viewed as an opportunistic creation. Transposase is encoded by either or both of the ISs, and antibiotic resistance genes are usually found in the internal region. The latter genes further boost the spread of composite transposons into distant genomes under selective pressures. Compared with these typical bacterial composite transposons, *KolobokP* families encode only a DDD/E transposase and a His-Me finger nuclease in the internal region. The sequence diversity of *KolobokP* transposases is considerably large, and elements permeated into genomes from 7 phyla. These data suggest *KolobokP* has been a sustainable lineage of mobile elements. It is tempting to speculate that some mechanisms could be functioning through which the integrity of the composite-like architecture is maintained, either by inhibiting the excision of solo LTDRs, or by some pathways to restore the composite organization if excision occurs. It has been known that the bacterial IS*6* family members, such as IS*26*, had a particular transitional tendency to form new composite-like, or called “pseudo-composite”, organizations [25, 26]. This process is proved driven by the IS-encoded DDD/E transposase, likely through a circular structure termed “translocatable unit (TU)” [27]. However, the exact molecular pathway of this “targeted integration” is still unclear [28]. The molecular pathway used by *KolobokP* families is also unknown. It is to be determined whether a circular intermediate is involved as implied by the LTDR excision footprint. Nevertheless, the encoded HE seems to add another layer of complexity to this mechanism. Solo LTDRs and multimers are very common in various *KolobokP* families. *KolobokP-7N2_CorFlu* family exhibits a much lower LTDR excision frequency than *KolobokP-4N1_CorFlu*, but still has a considerably large number of solo LTDRs (Table 3). These solo LTDRs could be generated through the homologous recombination process, which analogously gives rise to the solo LTRs of various LTR retrotransposon families.

Our phylogenetic analysis indicates that *KolobokP* sits inside the *Kolobok* superfamily, among a few lineages which seem to have acquired a CCHC zinc finger motif at a relatively late stage. Furthermore, the encoded endonuclease is likely acquired from one eukaryotic His-Cys box HE. Taken together, how *KolobokP* families emerged with their novel features unseen in other *Kolobok* families would be an intriguing question.

### Possible mechanisms to generate multimers

One conspicuous feature of *KolobokP* is the presence of multimers. In the cases of LTR retrotransposons, tandem arrays of LTR retrotransposons have been observed for a group of LTR retrotransposons, called Terminal-repeat retrotransposons in miniature or TRIMs [29, 30]. TRIMs are nonautonomous LTR retrotransposons, and most families are shorter than 1 kb in length [29, 31]. The absence of multimers of autonomous LTR retrotransposons, as well as the frequent occurrence of solo LTRs, would be explained by the decreasing forces on the genome size [32]. Compared with TRIMs, *KolobokP* families are much longer; autonomous *KolobokP* families are in general longer than 4 kb (Additional file 1: Table S1). The presence of TSDs at both extreme ends of the multimer excludes the possibility of recombination between *KolobokP* copies at different loci.

**Fig. 5:**
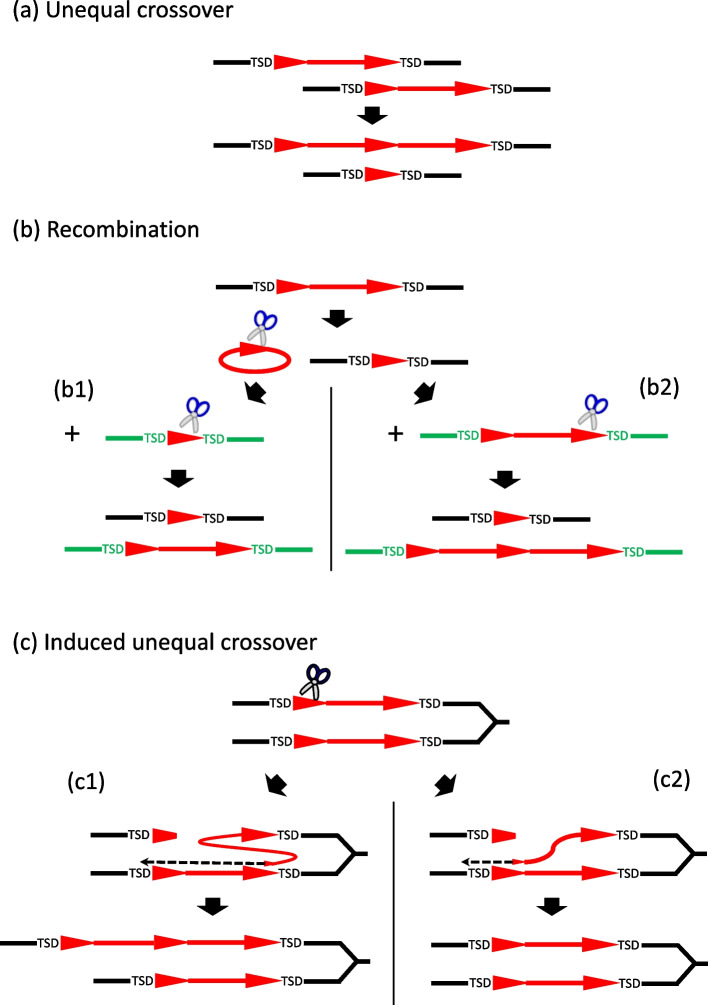
The models of propagation of *KolobokP* families. Red triangles represent LTDRs while red horizontal bars represent internal portions (INTs) of *KolobokP*. Scissors indicate His-Me finger nucleases. **a** Unequal crossover: the unequal crossover between the LTDRs on the sister chromatids results in the generation of a multimer and a solo LTDR. **b** Recombination may excise an extrachromosomal circular DNA composed of one LTDR and one INT. It would be integrated into a solo LTDR to generate a full-length copy (**b1**) or a full-length *KolobokP* copy to generate a multimer (**b2**). **c** During the DNA replication, the His-Me finger nuclease cleaves one DNA inside of an LTDR farther from the replication fork. If the LTDR closer to the replication fork is used by chance as a template, one LTDR-INT portion is duplicated (**c1**). If the LTDR farther from the replication fork is used as a template, the original *KolobokP* insertion is reinstated (**c2**)

As *KolobokP* is a type of DNA transposon, template switching during reverse transcription, proposed as a mechanism generating multimers of LTR retrotransposons [30] can be safely excluded. There are two possible mechanisms to generate *KolobokP* multimers. One is the unequal crossover between two allelic LTDRs of a single *KolobokP* insertion (Fig. 5a). The unequal crossover would generate both a solo LTDR and an (LTDR-INT)_2_-LTDR insertion. Another possible mechanism is the insertion through a circular intermediate resembling a “translocatable unit” [26], which is composed of 1 LTDR and 1 INT (Fig. 5b). Such a circular intermediate would be generated via the recombination between 2 LTDRs, along with a solo LTDR that remained on the genome. If the circular intermediate targets specifically at a solo LTDR (Fig. 5b1) or the LTDR of a full-length *KolobokP* (Fig. 5b2), either through recombination or another unidentified mechanism, the resultant is a single unit (LTDR-INT-LTDR) or a dimer (LTDR-INT)_2_-LTDR. In all these cases, TSDs remain unaltered. Once a multimer is generated, the unequal crossover could generate variations in the number of units.

The His-Me finger nucleases encoded by *KolobokP* families are closely similar to the group of eukaryotic His-Cys box HEs embedded in group I self-splicing introns (Fig. 3). The His-Cys domains and the active site are well aligned (Additional file 3: Data S3). One of the most-studied His-Cys box HEs is *I-PpoI*, which recognizes a 15-bp semi-palindromic homing site but shows tolerance to sequence variations. It binds the target DNA as a homodimer and generates staggered double-strand cuts with four-base 3’-overhangs (TTAA) [15, 33]. The sequence specificity is determined by 4 residues (61R, 63Q, 65 K, and 74 T) at the major DNA-binding surface. *KolobokP* His-Me finger nucleases likely have a comparable DNA-binding ability to a semi-palindromic site. *KolobokP* does not show a strong target sequence specificity or preference (Additional file 2: Figures S2-S4). Therefore, His-Me finger nucleases seem not to determine the integration site. Interestingly, the left and right TIRs, as suggested, are abutted on the circular intermediate and could potentially constitute a symmetric or semi-palindromic target. Of course, this does not exclude the possibility in the internal LTDR region occurs a homing site. It is possible that *KolobokP* His-Me finger nucleases are able to cleave DNA inside of LTDR or at the termini of the TIRs on the circular intermediates. The activity to cleave DNA inside or at the termini of LTDRs may induce the integration of an extrachromosomal *KolobokP* circle into the genome (Fig. 5b).

Except for its potential roles in transposition, the activities of His-Me finger nucleases could also provide a framework to explain the unusual features observed in *KolobokP* families: the abundance of multimers. Canonical HEs act as a selfish genetic element and are inherited in a non-Mendelian fashion [34]. HEs cleave DNA strands at the homing site and initiate a gene conversion process using the allele containing the HE gene as a template. This is accomplished by the host DNA break repair system.

During the DNA replication, the His-Me finger nuclease encoded by *KolobokP* families could cleave one sister chromatid inside of the LTDR farther from the replication fork (Fig. 5c). The DNA can be repaired using the sister chromatid as a template. If the LTDR closer to the replication fork is used by chance as a template, one LTDR-INT portion could be added (Fig. 5c1). If the LTDR farther from the replication fork is used as a template, the original *KolobokP* insertion could be reinstated (Fig. 5c2). This “induced unequal crossover” model can explain the frequent presence of *KolobokP* multimers. Although it is speculative, the induced unequal crossover is able to counterbalance the disruption of the composite-like structure of *KolobokP* families. The transposition of solo LTDRs results in the decrease of functional *KolobokP* copies. On the other hand, the copy number of *KolobokP* can be increased by the induced unequal crossover. A multimer longer than trimer (LTDR-I-LTDR-I-LTDR-I-LTDR) includes more than 2 full-length monomers, both of which can be mobilized. The excision of a circular DNA of LTDR-INT can occur from multimers, and the circular DNA can be recombined with a solo LTDR so as to restore a functional *KolobokP* copy (Fig. 5b1).

### The KolX protein of the Kol0 lineage is likely the origin of cytoplasmic ballast fold domain

The KolX protein of the Kol0 lineage was revealed a cytoplasmic ballast fold domain. It was reported that the proteins showing similarity to the cytoplasmic ballast are found in diverse animals [35]; however, it has not been pointed out that they are a part of *Kolobok* families of DNA transposons. The Kol0 lineage is found in diverse animals including vertebrates, annelids, cnidarians, and sponges [9]. It was proposed that a P2X gene, similar to the P2X4 gene, captured the C-terminal domain in the common ancestor of teleost to give birth to P2X7 gene [35]. Given the fact that the C-terminal domain is seen as a protein encoded by *Kolobok* DNA transposons, it is now clear that P2X7 gene originated by the fusion between a P2X4-like ancestral gene and the KolX protein encoded by a Kol0 lineage of *Kolobok* DNA transposon. Nanor, a protein expressed in the midblastula transition in zebrafish, also shows similarities to KolX proteins [35, 36]. The entire protein of Nanor is well aligned with KolX proteins and thus, it is likely that Nanor is also a domesticated KolX protein.

## Conclusion

We characterized a new, derived lineage of the *Kolobok* superfamily of DNA transposons, designated *KolobokP*, which is distributed patchily but widely in animals. A unique characteristic of *KolobokP* is its long direct repeats (LTDRs) at both ends, which makes *KolobokP* resemble prokaryotic composite/compound DNA transposons. The copies of *KolobokP* with an unusual structure suggest the transposition of solo LTDRs. *KolobokP* encodes a DDD/E transposase and a His-Me finger endonuclease that likely originated from an HE encoded by a group I self-splicing intron. We propose a model where His-Me finger nuclease contributes to the multiplication of *KolobokP* by inducing unequal crossover, which waits for experimental validation.

## Methods

### Discovery of KolobokP TEs from the Pacific oyster genome

RepeatModeler (https://www.repeatmasker.org/RepeatModeler/) was used for the initial screening of TEs from the genome of Pacific oyster *Crassostrea gigas*. Censor [37] searches were performed against the genome with the consensus sequences of repeats generated by RepeatModeler. Up to 10 Censor hits were extracted with 5,000-bp flanking sequences at both sides. Consensus sequences were regenerated to be elongated to reach both termini. The termini were determined based on the terminal RR..YY signatures, TIRs and TSDs. If a consensus sequence was well generated, it would be identical to the sequence of ancient active TE copy. The characterization of *Kolobok* was done based on the sequence homology to the reported *Kolobok* families in Repbase [9].

### Characterization of KolobokP and other Kolobok

TBLASTN was performed at the NCBI BLAST server (https://blast.ncbi.nlm.nih.gov/Blast.cgi) with the protein sequences of characterized *KolobokP* families from the Pacific oyster genome as queries. With the aim of discovering as many distant families as possible, hits with E-value up to 1e-04 were subject to further inspections given that the fragments are above 300 aa. The final transposon sequences from these protein leads were all verified by the existence of transposon termini and LTDRs in the genomic sequences. Such process was performed multiple rounds, each with a different protein as query from different major lineages, until no new distant relatives were found. In this process, a guiding phylogenetic tree was updated once new distant relatives were found. The genomes which contain sequences similar to *KolobokP* and the genomes of their phylogenetic relatives were downloaded from the NCBI Assembly (https://www.ncbi.nlm.nih.gov/assembly) and figshare (https://figshare.com/). The genomes used in this study are shown in Additional file 1: Table S1. Censor [37] searches were performed against the genomes with the protein sequences of characterized *KolobokP* families, or with the nucleotide sequences of characterized LTDRs as queries. Censor hits were extracted and clustered with BLASTCLUST 2.2.25 in the NCBI BLAST package with the thresholds at 75% length coverage and 75% sequence identity. The consensus sequence for each cluster was generated with the 50% majority rule applied with the help of homemade scripts. Censor searches were performed with the consensus sequence of each cluster against the genome. Up to 10 Censor hits were extracted with 5,000-bp flanking sequences at both sides. Consensus sequences were regenerated to be elongated to reach both termini. The termini were determined based on the terminal RR..YY signatures, TIRs and TSDs.

The consensus sequences of *KolobokP* families characterized in this study are available as Additional file 3: Data S1. All contig coordinates are shown in Additional file 4: Data S2.

### Phylogenetic analysis

Protein sequences predicted from the consensus or representative sequences for *KolobokP* families were aligned with the help of MAFFT v.7.407 [38] with the linsi option. Any fragmented or partial protein sequences caused by the incorrect prediction of protein-coding sequences, errors in sequencing or consensus-building were removed from the further analysis. Representative sequences were chosen based on the phylogenetic distances and the status of characterization. Protein sequence alignments used for the phylogenetic analyses are available as Additional file 3: Data S3 (DDD/E transposases of *KolobokP*), S4 (His-Me finger nucleases of *KolobokP*), and S5 (DDD/E transposases of entire *Kolobok*). The Bayesian inference tree was generated from MrBayes 3.2.7a [39] with parameters as follows: LG or rtREV amino acids replacement matrix.

## Supplementary Information

**Additional file 1: Table S1.** Characteristics of *KolobokP* families.

**Additional file 2: Figure S1.** The complete phylogenetic tree of *KolobokP* DDD/E transposases. The families with ~660-bp LTDRs are highlighted in orange. The numbers at branches indicate the posterior probabilities. *Kolobok-5_TV* and *Kolobok-6_TV* were used as the outgroup. The sequence alignment is provided as Additional file 3: Data S3. **Figure S2.** Complete, solo LTDR, and tandem insertions of *KolobokP* families in the genome of *Mytilus corusus*. LTDRs are highlighted in yellow, while internal portions are in cyan. TSDs are colored in red. **Figure S3.** Complete, solo LTDR, and tandem insertions of *KolobokP* families in the genome of *Mercenaria mercenaria*. LTDRs are highlighted in yellow, while internal portions are in cyan. TSDs are colored in red. **Figure S4.** Complete, solo LTDR, and tandem insertions of *KolobokP* families in the genome of *Gigantopelta aegis*. LTDRs are highlighted in yellow, while internal portions are in cyan. TSDs are colored in red. **Figure S5.** Junction sequences of solo LTDR excision. The inferred likely TSDs are highlighted in yellow. The internal sequences of *KolobokP* families are omitted. **Figure S6.** Protein alignment of the cytoplasmic ballast domains of P2X7 purinoceptors, Nanor from zebrafish, and KolX proteins of *Kolobok* families.

**Additional file 3: Data S1.** Consensus sequences of *KolobokP* families characterized in this study. The LTDR and internal portion (I) are divided (fasta format). **Data S3.** Multiple alignment of *KolobokP* DDD/E transposases (fasta format). **Data S4.** Multiple alignment of *KolobokP* HNH nucleases and related homing endonucleases  (fasta format). **Data S5.** Multiple alignment of *Kolobok* DDD/E transposases (fasta format). **Data S6.** KolX protein sequences encoded by *piggyBac* DNA transposons (fasta format).

**Additional file 4: Data S2.** Coordinates of all complete copies of *KolobokP* families. *KolobokP* families were divided into LTDR and internal sequence (I). Only the coordinates with >99% length of and >90% identity to the consensus are shown. 

## Data Availability

All data generated or analyzed during this study are included in this published article and its supplementary information files. All *Kolobok* sequences characterized in this study are also submitted to Repbase (http://www.girinst.org/repbase/).
