# Supplementary material for: A unique eukaryotic lineage of composite-like DNA transposons encoding a DDD/E transposase and a His-Me finger homing endonuclease
Source: Mob DNA. 2022 Oct 22;13:24. doi: 10.1186/s13100-022-00281-3 (PMC9587614; doi:10.1186/s13100-022-00281-3)
Supplement: Supplementary file 2 — Additional file 2: Figure S1. The complete phylogenetic tree of KolobokP DDD/E transposases. The families with ~660-bp LTDRs are highlighted in orange. The numbers at branches indicate the posterior probabilities. Kolobok-5_TV and Kolobok-6_TV were used as the outgroup. The sequence alignment is provided as Additional file 3: Data S3. Figure S2. Complete, solo LTDR, and tandem insertions of KolobokP families in the genome of Mytilus corusus. LTDRs are highlighted in yellow, while internal portions are in cyan. TSDs are colored in red. Figure S3. Complete, solo LTDR, and tandem insertions of KolobokP families in the genome of Mercenaria mercenaria. LTDRs are highlighted in yellow, while internal portions are in cyan. TSDs are colored in red. Figure S4. Complete, solo LTDR, and tandem insertions of KolobokP families in the genome of Gigantopelta aegis. LTDRs are highlighted in yellow, while internal portions are in cyan. TSDs are colored in red. Figure S5. Junction sequences of solo LTDR excision. The inferred likely TSDs are highlighted in yellow. The internal sequences of KolobokP families are omitted. Figure S6. Protein alignment of the cytoplasmic ballast domains of P2X7 purinoceptors, Nanor from zebrafish, and KolX proteins of Kolobok families. [file 13100_2022_281_MOESM2_ESM.pdf]

Figure S1. The complete phylogenetic tree of *KolobokP* DDD/E transposases. The families with ~660-bp LTDRs are highlighted in orange. The numbers at branches indicate the posterior probabilities. *Kolobok-5_TV* and *Kolobok-6_TV* were used as the outgroup. The sequence alignment is provided as **Additional file 3: Data S3**.

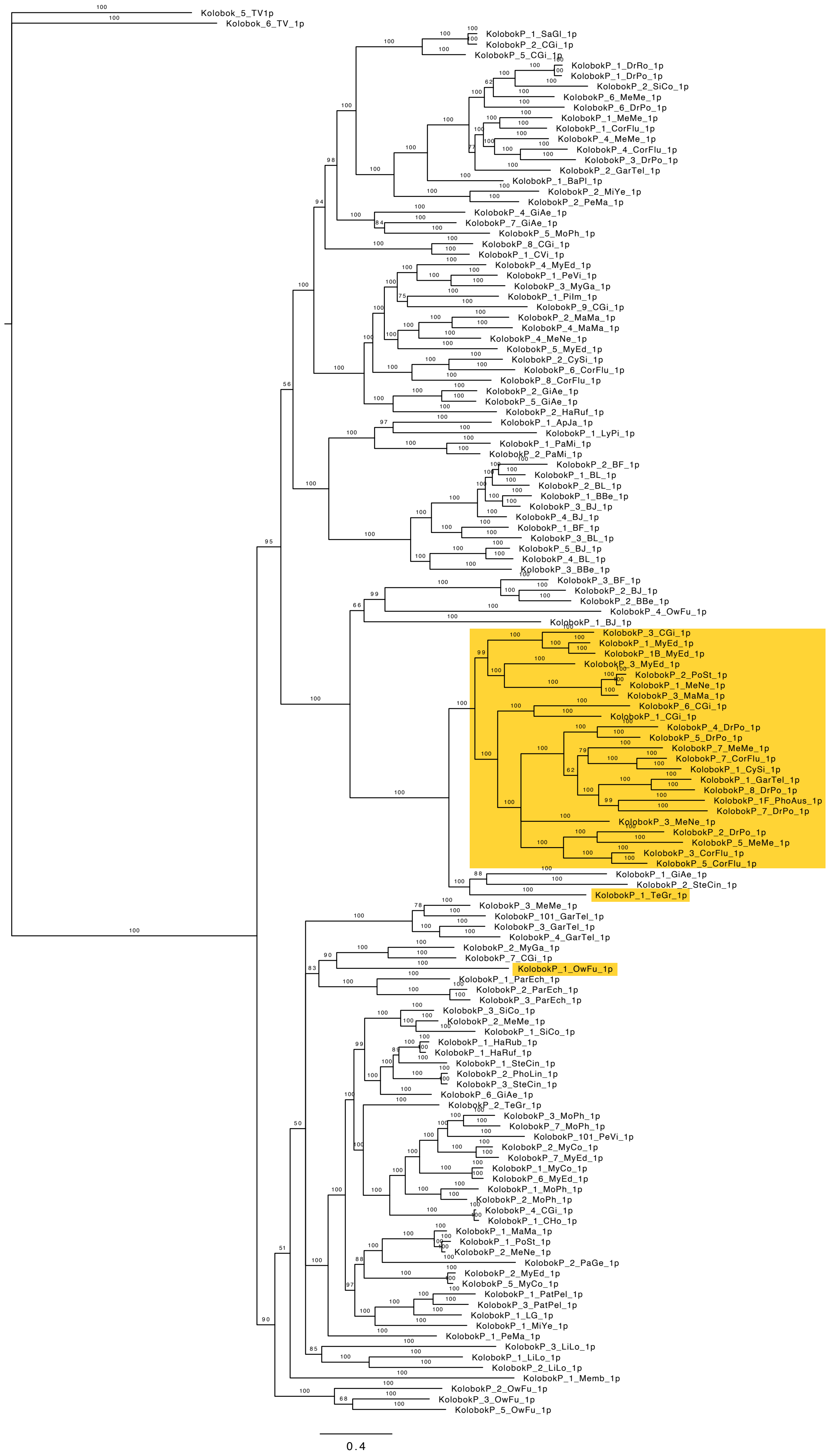
Kolobok_5_TV1p
Kolobok_6_TV_1p
KolobokP_1_SaGl_1p
KolobokP_2_CGi_1p
KolobokP_5_CGi_1p
KolobokP_1_DrRo_1p
KolobokP_1_DrPo_1p
KolobokP_2_SiCo_1p
KolobokP_6_MeMe_1p
KolobokP_6_DrPo_1p
KolobokP_1_MeMe_1p
KolobokP_1_CorFlu_1p
KolobokP_4_MeMe_1p
KolobokP_4_CorFlu_1p
KolobokP_3_DrPo_1p
KolobokP_2_GarTel_1p
KolobokP_1_BaPl_1p
KolobokP_2_MiYe_1p
KolobokP_2_PeMa_1p
KolobokP_4_GiAe_1p
KolobokP_7_GiAe_1p
KolobokP_5_MoPh_1p
KolobokP_8_CGi_1p
KolobokP_1_CVi_1p
KolobokP_4_MyEd_1p
KolobokP_1_PeVi_1p
KolobokP_3_MyGa_1p
KolobokP_1_PiIm_1p
KolobokP_9_CGi_1p
KolobokP_2_MaMa_1p
KolobokP_4_MaMa_1p
KolobokP_4_MeNe_1p
KolobokP_5_MyEd_1p
KolobokP_2_CySi_1p
KolobokP_6_CorFlu_1p
KolobokP_8_CorFlu_1p
KolobokP_2_GiAe_1p
KolobokP_5_GiAe_1p
KolobokP_2_HaRuf_1p
KolobokP_1_ApJa_1p
KolobokP_1_LyPi_1p
KolobokP_1_PaMi_1p
KolobokP_2_PaMi_1p
KolobokP_2_BF_1p
KolobokP_1_BL_1p
KolobokP_2_BL_1p
KolobokP_1_BBe_1p
KolobokP_3_BJ_1p
KolobokP_4_BJ_1p
KolobokP_1_BF_1p
KolobokP_3_BL_1p
KolobokP_5_BJ_1p
KolobokP_4_BL_1p
KolobokP_3_BBe_1p
KolobokP_3_BF_1p
KolobokP_2_BJ_1p
KolobokP_2_BBe_1p
KolobokP_4_OwFu_1p
KolobokP_1_BJ_1p
KolobokP_3_CGi_1p
KolobokP_1_MyEd_1p
KolobokP_1B_MyEd_1p
KolobokP_3_MyEd_1p
KolobokP_2_PoSt_1p
KolobokP_1_MeNe_1p
KolobokP_3_MaMa_1p
KolobokP_6_CGi_1p
KolobokP_1_CGi_1p
KolobokP_4_DrPo_1p
KolobokP_5_DrPo_1p
KolobokP_7_MeMe_1p
KolobokP_7_CorFlu_1p
KolobokP_1_CySi_1p
KolobokP_1_GarTel_1p
KolobokP_8_DrPo_1p
KolobokP_1F_PhoAus_1p
KolobokP_7_DrPo_1p
KolobokP_3_MeNe_1p
KolobokP_2_DrPo_1p
KolobokP_5_MeMe_1p
KolobokP_3_CorFlu_1p
KolobokP_5_CorFlu_1p
KolobokP_1_GiAe_1p
KolobokP_2_SteCin_1p
KolobokP_1_TeGr_1p
KolobokP_3_MeMe_1p
KolobokP_101_GarTel_1p
KolobokP_3_GarTel_1p
KolobokP_4_GarTel_1p
KolobokP_2_MyGa_1p
KolobokP_7_CGi_1p
KolobokP_1_OwFu_1p
KolobokP_1_ParEch_1p
KolobokP_2_ParEch_1p
KolobokP_3_ParEch_1p
KolobokP_3_SiCo_1p
KolobokP_2_MeMe_1p
KolobokP_1_SiCo_1p
KolobokP_1_HaRub_1p
KolobokP_1_HaRuf_1p
KolobokP_1_SteCin_1p
KolobokP_2_PhoLin_1p
KolobokP_3_SteCin_1p
KolobokP_6_GiAe_1p
KolobokP_2_TeGr_1p
KolobokP_3_MoPh_1p
KolobokP_7_MoPh_1p
KolobokP_101_PeVi_1p
KolobokP_2_MyCo_1p
KolobokP_7_MyEd_1p
KolobokP_1_MyCo_1p
KolobokP_6_MyEd_1p
KolobokP_1_MoPh_1p
KolobokP_2_MoPh_1p
KolobokP_4_CGi_1p
KolobokP_1_CHo_1p
KolobokP_1_MaMa_1p
KolobokP_1_PoSt_1p
KolobokP_2_MeNe_1p
KolobokP_2_PaGe_1p
KolobokP_2_MyEd_1p
KolobokP_5_MyCo_1p
KolobokP_1_PatPel_1p
KolobokP_3_PatPel_1p
KolobokP_1_LG_1p
KolobokP_1_MiYe_1p
KolobokP_1_PeMa_1p
KolobokP_3_LiLo_1p
KolobokP_1_LiLo_1p
KolobokP_2_LiLo_1p
KolobokP_1_Memb_1p
KolobokP_2_OwFu_1p
KolobokP_3_OwFu_1p
KolobokP_5_OwFu_1p
0.4

Figure S2. Complete, soloLTDR, and tandem insertions of *KolobokP* families in the genome of *Mytilus corusus*. LTDRs are highlighted in yellow, while internal portions are in cyan. TSDs are colored in red.

KolobokP-1_MyCo

```
CAGCCTCTTAGGCCCGAGAC//GTCTTGGGCCAGTTCATCCG
TGCACGGTCTGGCCTGAGAC//GTCTTGGGCCGTCTTTCATG
AAGTAATTGTGGCCCGAGAC//ATCTTGGGCCTAGTAAAACT
GTAGCTTGTAGGCCCGAGAC//GTCTTTGGCCTGTATATTCA
TAGCCTACATGGCCCGAGAC//GTCTTGGGCCACGAACGGCA
TCACGTAAGAGGCCCGAGAC//GTCTTGGGCCTATTAGACCC//CAGCCTCTTAGGCCCGAGAC//GTCTTGGGCCAAGAGGTTAA
TCACATATACGGCCCGAGAC//GTCTTGGGCCTATTAGACCC//CAGCCTCTTAGGCCCGAGAC//GTCTTGGGCCATACAAATTG
TCACATATACGGACCGAGAC//GTCTTGGGCCTATTAGACCC//CAGCCTCTTAGGCCCGAGAC//GTCTTGGGCCATACAAATTG
```

KolobokP-2_MyCo

```
TAACTGTACTGGCCTGAGAA//TCCTCGGACCTAATAGGACC//CAGCCTCTTAGGCCCGAGAA//TCCTCGGACCTACTGATGAC
ATTATAAAGTGGCCTGAGAA//TCCTCGGACCTAATAGGACC//CAGCCTCTTAGGCCCGAGAA//TCCTCGGACCAAGTACGTGG//
GACAACACTAGGCC-GAGAA//TCCTCGGACCTAATAGGACC//CAGCCTCTTAGGCCCGAGAA//TCCTCGGACCACTAGTGTAA
AGTGACAGTCGGCCCGAGAA//TCCTCGGACCAGTCTACTAA
TCTGTCAAGTGGCCCGAGAA//TCCTCGGACCAAGCATGTGT
GACCCTATACGGCCCGAGAA//TCCTCGGACCTAATAGGACC//CAGCCTCTTAGGCCCGAGAA//TCCTCGGACCATACGTATCG
CGATTGTACTGGCC-GAGAA//TCCTCGGACCTAATAGGACC//CAGCCTCTTAGGCCTGAGAA//TCCTCGGACCTACTCCCGGT
CTTTTTATATGGCCCGAGAA//TCCTCGGACCATATTGTATG
ATCTTTATATGGCCCGAGAA//TCCTCGGACCTAATAGGACC//CAGCCTCTTTGGCCCGAGAA//TCCTCGGACCATATGAGGAA
ACCTCTAACTGGCCCGAGAA//TCCTCGGACCTAATAGGACC//CAGCCTCTTAGGCCCGAGAA//TCCTCGGACCAACTGGCTAA
```

KolobokP-3_MyCo

```
GCGTCTTGTAGGTTGCACTT//AAGTGCAACCTATACTGTAC//TTACCTTATAGGTTGCACTT//AAGTGCAACCTGTAATGTCT
AGCTTCTGTAGGTTGCACTT//AAGTGCAACCTATACTGTAC//TTACCTTATAGGTTGCACTT//AAGTGCAACCTGTATGAGTT
GAGTATAATAGGTTGCACTT//AAGTGCAACCTAATAACCAA
ACCTATTGTAGGTTGCACTT//AAGTGCAACCTATACTGTAC//TTACCTTATAGGTTGCACTT//AAGTGCAACCTGTATTTGTT
AGCTTCTGTAGGTTGCACTT//AAGTGCAACCTATACTGTAC//TTACCTTATAGGTTGCACTT//AAGTGCAACCTGTATGAGTT
```

KolobokP-4_MyCo

```
AACCATATTAGGGTAAATTC//TGAATTTACCCTATAATAGA//TTTCTCTATAGGGTAAATTCA//GAATTTACCCCTATAATAGA//CAAATTTTCTCTATAATAGA//TTTCTCTATAGGGTAAATTCA//GA
ATTTACCCCATTAGTCA
ACTCGTGTAGGGGTAAATTC//TGAATTTACCCTATAATAGA//TTCTCTATAGGGGTAAATTC//GAATTTACCCCTGTAGTATT
TTACATTGTTGGTAAATTCA//GAATTTACCCCTGTTGCTAG
TCAATCTATAGGGTAAATTC//GAATTTACCCCTATAATAGA//TTCTCTATAGGGTAAATTCA//GAATTTACCCTTAAATAATT
TGCATTATAGGGGTAAATTC//GAATTTACCCCTATAAGAGA//CTCCCTATAGGGGTAAATTC//GAATTTACCCCTATATAATT
CTTTCTGAAGGGGTAAATTC//TGAATTTACCCTATAATAGA//TTCTCTATAGGGTAAATTCA//TGAATTTACCCTATAATAGA//TTCTCTATAGGGTAAATTCA//TGAATTTACCCTATAATAGA//
TTCTCTATAGGGTAAATTCA//TGAATTTACCCTATAATAGA//TTCTCTATAGGGGTAAATTC//GAATTTACCCCTGAAACGCT
ACTGCTATCGGGGTAAATTC//GAATTTACCCCTATATAGCC
AATGCATAAAGGGTAAATTC//TGAATTTACCCTATAATAGA//TTCTCTATAGGGGTAAATTC//GAATTTACCCTATAGTAAAA
```

KolobokP-5_MyCo

```
TAGATCATGTGGAACGGGGA//ATCCTATTCCTGTCAGACTT
GACTGAAACTGGAACGGGGA//ATCCTATTCCAACTAATAGC
GATAAGTAATGGAACGGGGA//ATCCTATTCCAGTAATGAGC
TCTTTTATATGGAACAGGGA//ATCCTATTCCATATGCTGAA
GATGGAATATGGAACGGGGA//ATCTTATTCCATATGCTCGT
CCCTCTGACAGGAACGGGGA//ATCCTATTCCTGTCTGACTT
CCCTCTGACAGGAACGGGGA//ATCCTATTCCTGCTGTTTCA
CCCTCTGACAGGAACGGGGA//ATCCTATTCCTGTCACGTGT
TTATATGTATGGAACGGGGA//ATCCTATTCCTGTCAGACTT
CCCTCTGACAGGAACGGGGA//ATCCTATTCCATTTTCACTT
ATTTGTAAATGGAACGGGGA//ATCCTATTCCAAATCAAAAG
CTCATCAACAGGAACGGGGA//ATCCTATTCCAACAGGCAAC
ACTAATAGCTGGAACGGGGA//ATCCTATTCCCGCTCACACC
CCTGCCAACTGGAACGGGGA//ATCCTATTCCAACTAGCACG
AATATCATCTGGAACGGGGA//ATCCTATTCCTGTCAGACTT
CCCTCTGACAGGAACGGGGA//ATCCTATTCCTGTCAGACTT
TCAATAAGTTGGAACGGGGA//ATCCTATTCCTGTCAGACTT//CCCTCTGACAGGAACGGGGA//ATCCTGTTCCAGTTGAAATG
CCCTCTGACAGGAACGGGAC//ATCCTATTCCACCTGCTGTG
TAACAAACGTGGAACGGGGA//ATCCTATTCCTGTCAGACTT
CCCTCTGACAGGAACGGGGA//ATCCTATTCCATATATACTC
CTATCAATCTGGAACGGGGA//ATCCTATTCCATCTTTCAAA
ATACAGATATGGAACGGGGA//ATCCTATTCCATATGTCTCA
AGTACAACTTGGAACGGGGA//ATCCTATTCCTGTCAGACTT//CCCTCTGACAGGAACGGGGA//ATCCTATTCCACTTGTTTCA
GTTGAATAGTGGAACGGGGA//ATCCTATTCCAGTCCTTTTA
CCCTCTGACAGGAACGGGGA//ATCCTATTCCAGACTATAAC
TTATAAGACTGGAACGGGGA//ATCCTATTCCTGTCAGACTT
CCCTCTGACAGGAACGGGGA//ATCCTATTCCAACTCTTGCT
CCCTCTGACAGGAACGGGGA//ATCCTATTCCTGTCAGACTT
CTCCTTAGTTGGAACGGGGA//ATCCTATTCCTGTCAGACTT//CCCTCTGACAGGAACGGGGA//ATCCTATTCCTGTCAGACTT//CCCTCTGACAGGAACGGGGA//ATCCTATTCCAGTTCTAGTT
CGTGTGATATGGAACGGGGA//ATCCTATTCCTGTCAGACTT
CCCTCTGACAGGAACGGGGA//ATCCTATTCCTGTCAGACTT
CCCTCTGACAGGAACGGGGA//ATCCTATTCCTGTCAGACTT
CCCTCTGACAGGAACGGGGA//ATCCTATTCCTGTCAGACTT
CCCTCTGACAGGAACGGGGA//ATCCTATTCCTGTCAGACTT
CCCTCTGACAGGAACGGGGA//ATCCTATTCCTGTCAGACTT
CCCTCTGACAGGAACGGGGA//ATCCTATTCCAATGCTGTCA
CCCTCTGACAGGAACGGGGA//ATCCTATTCCTGTCAGACTT
CCCTCTGACAGGAACGGGGA//ATCCTATTCCTGTCAGACTT
GTACTTATGTGGAACGGGGA//ATCCTATTCCATGTATTCCG
CCCTCTGACAGGAACGGGGA//ATCCTATTCCTGTCAGACTT
CCCTCTGACAGGAACAGGGA//ATCCTATTCCTGTCAGACTT
CCCTCTGACAGGAACGGGAC//ATCCTATTCCTGTCAGACTT
CCCTCTGACAGGAACGGGAC//ATCCTATTCCTGTCAGACTT
```

Figure S3. Complete, soloLTDR, and tandem insertions of *KolobokP* families in the genome of *Mercenaria mercenaria*.

LTDRs are highlighted in yellow, while internal portions are in cyan. TSDs are colored in red.

KolobokP-1_MeMe

```
AATATATTTAGGTTATAACA//TCTGTATGCCTATAAGGAAA
ATACACTTGAGGTTATAACA//TCTGTATGCCTTGACAATTT
GCCCTAATAAGGTTATAACA//TCTGTATGCCATAAACCGAA
AGTCTCTGTAGGTTATAACA//TCTGTATGCCTATAAGGAAA//AATTCTTATAGGTTATAACA//TCTGTATGCCTATAAGGAAA//AATTCTTATAGGTTATAACA//TCTGTATGCCTATATAAGTA
ATTTATTAAAGGTTATAACA//TCTGTATGCCTAAAGAAATC
TCTCGTTTAAGGTTATAACA//TCTGTATGCCTATAAGGAAA//AATTCTTATAGGTTATAACA//TCTGTATGCCTTAACGGTGC
AAACGCTATAGGTTATAACA//TCTGTATGCCTATAAGGAAA//AATTCTTATAGGTTATAACA//TCTGTATGCCTATACATGAG
ATTGGCTTTAGGTTATAACA//TCTGTATGCCTTTAAAGGCG
CAAGCTTGGTGGTTATAACA//TCTGTATGCCTGGTAAGCCT
TCTCATTTTAGGTTATAACA//TCTGTATGCCTATAGGTTAT
AAATACTCAAGGTTATAACA//TCTGTATGCCTCAAACAGAT
GTTGCGTAGAGGTTATAACA//TCTGTATGCCTATAAGAAAA//AATTCTTATAGGTTATAACA//TCTGTATGCCATTATTACAA
TAATCGTAAAGGTTATAACA//TCTGTATGCCTAAAGGCGTA
AAATGGTGAAGGTTATAACA//TCTGTATGCCTGAAAGGAGG
AGTACTTAAAGGTTATAACA//TCTGTATGCCTAAAGACAGC
ATGCCTATGAGGTTATAACA//TCTGTATGCCTATGAAGCCA
TTACTGTAAGGGTTATAACA//TCTGTATGCCTAAAGACGCA
CGGGTTTGTAGGTTATAACA//TCTGTATGCCTATAAGGAAA//AATTCTTATAAGGAAATATG//AATTATTATAGGTTATAACA//TCTGTATGCCTATAAGGAAA//AATTCTTATAGGTTATAACA//TCTG
TATGCCTATAATCTAC
TCTTCTTCTAGGTTATAACA//TCTGTATGCCTTCTATCGCT
TGTTAGTGAAGGTTATAACA//TCTGTATGCCTGAATAGGTT
CACTGTTTTAGGTTATAACA//TCTGTATGCCTTTAGTTAGA
ATTTATATAAGGTTATAACA//TCTGTATGCCTTTCTTAACA
TGTGCCTCAAGGTTATAACA//TCTGTATGCCTCAAGGCTCA
GCTCACTGTAGGTTATAACA//TCTGTATGCCTGTAGTGTTG
CCATATTATAGGTTATAACA//TCTGTATGCCTATAAGGAAA//AATTCTTATAGGTTATAACA//TCTGTATGCCTATAGCCATG
CGTGTAACAAGGTTATAACA//CCTGTATGCCTATAAGGAAA//AATTCTTACAGGTTATGACA//TCTGTATGCCACAACTATCC
TTACTGTAAGGGTTTTAACA//TCTGTATACCTAAAGACACA
CCATCGTGTAGGTTATAACA//TCTGTATGCCTATAAGGAAA//AATTCTTATAGGTTATAACA//TCTGTATGCCTGTAACTAGT
GGTCAGTACAGGTTATAACA//TCTGTATGCCTACACTCAGT
AATTCCTTTAGGTTATAACA//TCTGTATGCCTTTATTATTG
TCCCTTGATAGGTTATAACA//TCTGTATGCCTATAAGGAAA//AATTCTTATAGGTTATAACA//TCTGTATGCCAATTGTCCGC
CGTTAATTTAGGTTATAACA//TCTGTATGCCTATAAGGAAA//AATTCTTATAGGTTATAACA//TCTGTATGCCTTTATTTATG
TATACTAGAAGGTTATAACA//TCTGTATGCCTATAGGAAAT//AATTCTTATAGGTTATAACA//TCTGTATGCCTATAAGAGCT
CTGAAATTCAGGTTATAACA//TCTGTATGCCTATAAGGAAA//AATTCTTATAGGTTATAACA//TCTGTATGCCTTCAGCCCAA
TGGGCTAGTAGGTTATAACA//TCTGTATGCCTATAAGGAAA//AATTCTTATAGGTTATAACA//TCTGTATGCCTAGTACTTTA
CCCCTTTTAAGGTTATAACA//TCTGTATGCCTATAAGGAAA//AATTCTTATAGGTTATAACA//TCTGTATGCCTATAAGGAAA//AATTCTTATAGGTTATAACA//TCTGTATGCCTTAAGGTATT
GACTGTTCCAGGTTATAACA//TCTGTATGCCTCCAGTGTTC
TAAAAGTTTTGGTTATAACA//TCTGTATGCCTTTTTGTCAG
TAAGTTTTATGGTTATAACA//TCTGTATGCCTATAAGGAAA//AATTCTTATAGGTTATAACA//TCTGTATGCCTATAAGGAAA//AATTCTTATAGGTTATAACA//TCTGTATGCCTATAAGGAAA//AATT
CTTATAGGTTATAACA//TCTGTATGCCTTATCCTAAC
CTGTCTTAGAGGTTATAACA//TCTGTATGCCTAGAACCAAT
ATCAATTGTAGGTTATAACA//TCTGTATGCCTTGTAGCGCA
TATTTATATAGGTTATAACA//TCTGTATGCCTATATAATGA
ATTGTGTGAAGGTTATAACA//TCTGTATGCCTATAAGGAAA//--------------------//TCTGTATGCCTGAAATTAAT
AGCGACTAGTGGTTATAACA//TCTGTATGCCTATAAGGAAA//AATTCTTATAGGTTATAACA//TCTGTATGCCTAGTGGCTAA
ATTACTATTAGGTTATAACA//TCTGTATGCCTATAACTGCA
AAGAGCTTCTGGTTATAACA//TCTGTATGCCTATAAGGAAA//AATTCTTATAGGTTATAACA//TCTGTATGCCTTCTGTGTCT
TTCGCATTAAGGTTATAACA//TCTGTATGCCTATAAGGAAA//AATTCTTAT-CCACCACTAT//TCTGTATGCCTTAAGGTTTA
TCCTTTTGAAGGTTATAACG//TCTGTATGCCTTGAAGAGCG
AGGGAATATAGGTTATAACA//TCTGTATGCCTATATGGTAA
TGCTTCTCTAGGTTATAACA//TCTGTATGCCTATAAGGAAA//AATTCTTATAGGTTATAACA//TCTGTATGCCTCTATAAATA
ATACTTTCAAGGTTATAACA//TCTGTATGCCTATAAGGAAA//AATTCTTATAGGTTATAACA//TCTGTATGCCTATAAGGAAA//AATTCTTATAGGTTATAACA//TCTGTATGCCTTCAACAGAA
GACATATAATGGTTATAACA//TCTGTATGCCTAATTCAGAG
AAGAGCAATAGGTTATAACA//TCTGTATGCCTATAAGGAAT//AATTCTTATAGGTTATAACA//TCTGTATGCCAACAGCATTT
AATACCTTAAGGTTATAACA//TCTGTATGCCTTAATACAAA
AAGTGATGTAGGTTATAACA//TCTGTATGCCTGTAGCTGAA
TGTTCTTTATGGTTATAACA//TCTGTATGCCTTATTCATAT
ATAACCTTTAGGTTATAACA//TCTGTATGCCTATAAGGAAA//AATTCTTATAGGTTATAACA//TCTGTATGCCTTTACCTTTG
CAAGCGTCTAGGTTATAACA//TCTGTATGCCTATAAGGAAA//AATTCTTATAAGGAAATATG//AATTCTTATAGGTTATAACA//TCTGTATGCCTCTAAGCATT
TATGCCTTAAGGTTATAACA//TCTGTATGCCTTAATGGAAT
ACCCCCTCTAGGTTATAACA//TCTGTATGCCTCTAGCTTGT
ACAAAATATAGGTTATAACA//TCTGTATGCCTATAGCAAAA
TTTGTTTTAAGGTTATAACA//TTTGTATGCCTTAATTTGTC
GATTTATATAGGTTATAACA//TCTGTATGCCTATAAGGAAA//AATTCTTATAGGTTATAACA//TCTGTATGCCTATATTGTAT
ATTGCCTTAAGGTTATAACA//TCTGTATGCCTTAAGAGATT
GATTTATATAGGTTATAACA//TCTGTATGCCTATAAGGAAA//AATTCTTATAGGTTATAACA//TCTGTATGCCTATATAAGCT
GTTGGTTATAGGTTATAACA//TCTGAATGCCTATAAGGAAA//AATTCTTATAGGTTATAACA//TCTGTATGCCTGTTGGTACA
GAATAATATAGGTTATAACA//TCTGTATGCCATAATGAAAT
TTCATTACAAGGTTATAACA//TCTGTATGCCACAAATAGCC
ATTCATTACAGGTTATAACA//TCTGTATGACTATAAGGA--//AATTCTTATAGGTTATAACA//TCTGTATGCCTTACAAATAG
TTTCGATGTAGGTTATAACA//TCTGTATGCCTATAAGGAAA//--------------------//TCTGTATGCCTGTATCTCAC
TAGCTCACTAGGTTATAACA//TCTGTATGCCTATAAGGAAA//AATTCTTATAGGTTATAACA//TCTGTATGCCTATAAGGAAA//AATTCTTATAGGTTATAACA//TCTGTATGCCTCACTACAGA
ATTCTTTATAGGTTATAACA//TCTGTATGCCTTAAATGCAC
CGATGAATAAGGTTATAACA//TCTGTATGCCATAATTGCTT
TAGCCCTATAGGTTATAACA//TCTGTATGCCTATACGGAAA//AATTCTTATATACGGAAATA//AATTCTTATATACGGAAATA//AATTCTTATATACGGAAATA//AATTCTTATAGGTTATAACA//TCTG
TATGCCTATATGTGTT
CCACTGTAAAGGTTATAACA//TCTGTATGCCTAAAGTTCAG
TTATCTATAAGGTTATAACA//TCTGTATGCCTCGAACATAA
CACAGATTTAGGTTATAACA//TCTGTATGCCTTTACCATAC
TTTTGCTACAGGTTATAACA//TCTGTATGCCTATAAGGAAA//AATTCTTATAGGTTATAACA//TCTGTATGCCTACATACCAA
CGGGATTCTAGGTTATAACA//TCTGTATGCCTCTACAGATG
AATCATTTAAGGTTATAACA//TCTGTATGCCTTAAGGTATT
CTTTGTTATAGGTTATAACA//TCTGTATGCCTATATATGAA
TGACGTTATAGGTTATAACA//TCTGTATGCCTATAAGGAAA//AATTCTTATAGGTTATAACA//TCTGTATGCCTTATAGTTCT
AATGTCTCGAGGTTATAACA//TCTGTATGCCTCGAAAAGAG
TGGGAGAATAGGTTATAACA//TCTGTATGCCAATAATCAAC
TTCTCTTTATGGTTATAACA//TCTGTATGCCTATAAGGAAA//AATTCTTATAGGTTATAACA//TCTGTATGCCTTATGTCAAC
GGTTGCTTTAGGTTATAACA//TCTGTATGCCTATAACGAAA//AATTCTTATAGGTTATAACA//TCTGTATGCCTTTATATGAG
CTTCCTTACAGGTTATAACA//TCTGTATGCCTACACTGTTA
GAACACTTTAGGTTATAACA//TCTGTATGCCTATAAGGAAA//AATTCTTATAGGTTATAACA//TCTGTATGCCTTTAAGCCAG
TCGCTGTAAAGGTTATAACA//--------------------//AATTCTTATAGGTTATAACA//TCTGTATGCCAAAACAGTTA
TACGTTTCTAGGTTATAACA//TCTGTATGCCTTCTATCAGT
AAAATGTAAAGGTTATAACA//TCTGTATGCCTATAAGGAAA//AATTCTTATAGGTTATAACA//TCTGTATGCCTAAAGAGGTT
CTTGTATTTTGGTTATAACA//TCTGTATGCCTTTTTTCGAG
CTCCCTTTAAGGTTATAACA//TCTGTATGCCTATAAGGAAA//AATTCTTATAGGTTATAACA//TCTGTATGCCTATAAGGAAA//AATTCTTATAGGTTATAACA//TCTGTATGCCTTAAGTGGAG
AGTACTTATAGGTTATAACA//TCTGTATGCCTATAGTATAG
TTTGTCTATAGGTTATAACA//TCTGTATGCCTATAATCTAA
AGTAAGATTAGGTTATAACA//TCTGTATGCCTATAAGGAAA//AATTCTTATAGGTTATAACA//TCTGTATGCCATTAGAACAT
GAAGCATATAGGTTATAACA//--------------------//AATTCTTATAGGTTATAACA//TCTGTATGCCTATACGGGAC
GAAGTTTCAAGGTTATAACA//TCTGTATGCCTCAATGCAAT
TTATTGTATAGGTTATAACA//TCTGTACGCCTATAAGGAAA//TATTCTTATAGGTTATAACA//TCTGTATGCCTATAGGTTTA
```

AATGCTTAAAGGTTATAACA//TCTGTATGCCTAAACAGTGG
GGGCTTTTTAGGTTATAACA//TCTGTATGCCTATAATTATC
CATTTCAAAAGGTTATAACA//TCTGTATGCCTATAAGGAAA//AATTCTTATAAGTTATAACA//TCTGTATGCCAAAACATATG
CTTCATTATAGGTTATAACA//TCTGTATGCCTATATTGATA
ATTGCGTAAAGGTTATAACA//TCTGTATGCCTATAAGGAAA//AATTCTTATAGGTTATAACA//TCTGTATGCCTATAAGGAAA//AATTCTTATAGGTTATAACA//TCTGTATGCCTAAATCGGCT
AACCTCTTGAGGTTATAACA//TCTGTATGCCTATAAGGAAA//AATTCTTATAAGTTATAACA//TCTGTATGCCTTAAGTGGAT
AACTCCTAATGGTTATAACA//TCTGTATGCCTAATACGAGA
TACCATTTGAGGTTATAACA//TCTGTATGCCTTGAAAAAGA
TTACTGTAAAGGTTATAACA//TCTGTATGCCTAAAGACGCA
CGTGTATATAGGTTATAACA//TCTGTATGCCTATAAGGAAA//AATTCTTATAGGTTATAACA//TCTGTATGCCTATATACGCG
CTAGAATTTAGGTTATAACA//TCTGTATGCCTATAAGGAAA//AATTCTTATAGGTTATAACA//TCTGTATGCCTTTAAGGTTA
ACCGATTATAGGTTATAACA//TCTGTATGCCTATAAAAATA
GTGTTTTTAAGGTTATAACA//TCTGTATGCCTATAAGGAAA//AATTCTTATAGGTTATAACA//TCTGTATGCCTATAAGGAAA//AATTCTTATAGGTTATAACA//TCTGTATGCCTATAAGGAAA//AATT
CTTATAGGTTATAACA//TCTGTATGCCTATAAGGAAA//AATTCTTATAGGTTATAACA//TCTGTATGCCTATAAGGAAA//AATTCTTATAGGTTATAACA//TCTGTATGCCTTAATTACTG
AGGGCAAAAAGGTTATAACA//TCTGTATGCCAAAAGACATG
CTGGCCAGAAGGTTATAACA//TCTGTATGCCAGAACCGGAT
GTTATTTATAGGTTATAACA//TCTGTATGCCTATAAGGAAA//AATTCTTATAGGTTATAACA//TCTGTATGCCTTATACCAAC
CGGCCATCTAGGTTATAACA//TCTGTATGCCTATAAGGAAA//AATTCTTATAGGTTATAACA//TCTGTATGCCTATAAGGAAA//AATTCTTATAGGTTATAACA//TCTGTATGCCTCTAGAGAGT
TTCCTTAGAAGGTTATAACA//TCTGTATGCCTATAAGGAAA//AATTCTTATAGGTTATAACA//TCTGTATGCCTTAGAATGAA
AGTACGAAAAGGTTATAACA//TCTGTATGCCAAAATATGAG
AACGGATTTAGGTTATAACA//TCTGTATGCCATTTAAGGTA
TACTCGTAAAGGTTACAACA//TCTGTATGCCTATAAGGAAA//AATTCTTATAGGTTATAACA//TCTGTATGCCTAAATACTAT
ACCTAAATAAGGTTATAACA//TCTGTATGCCATAAATCTAA
AAATTAATTAGGTTATAACA//TCTGTATGCCTATAAGGAAA//AATTCTTATAGGTTATAACA//TCTGTATGCCATTATCACCG
TACCATTATAGGTTATAACA//TCTGTATGCCTATACTTTAC
AATTCTTATAGGTTATAACA//TCTGTATGCCTATAAGGAAA//AATTCT**TATA**GGAAATATGA//AATTCTTATAGGTTATAACA//TCTGTATGCCTATATTCTAT
GACTTTTTTAGGTTACAACA//TCTGTATGCCTAGGGACTTT
GTTACTTGTAGGTTATAACA//TCTGTATGCCTATAAGGAAA//AATTCTTATAGGTTATAACA//TCTGTA-GCCTATAAGGAAT//AAATTCTAGAGGTTGATAAC//TCTGTATGCCTATAAGGAAA//
AATTCTTATAGGTTATAACA//TCTGTATGCCTGTAGGCTGA (CM025877.1 83675160-83667018)
GGTCGCTTACGGTTATAACA//TCTGTATGCCTATAAGGAAA//AATTCTTATAGGTTATAACA//TCTGTATGCCTTACGCATAA
TATTCCTTAAGGTTATAACA//TCTGTATGCCTTAATCAATA
CGGTTATAAAGGTTATAACA//TCTGTATGCCTATAAGGAAA//AATTCTTATAGGTTATAACA//TCTGTATGCCTAAATGAATA
AGGTGGTATAGGTTATAACA//TCTGTATGCCTATATATATA
ATGTATTTTAGGTTATAACA//TCTGTATGCCTTTTAGTACC
TGCACGTTTAGGTTATAACA//TCTGTATGCCTTTATAAACC
TTACCTATATGGTTATAACT//TCTGTATGCCATATTTCAGC
TGTGTTGTTAGGTTATAACA//ACTGTATGCCTATAAGGAAA//AATTCTTATAGGTTATAACA//TCTGTATGCCTGTTATTCAT
GTGTCATATAGGTTATAACG//TCTGTATGCCTATAACGGTT
TAGGCCTTCTGGTTATAACA//TCTGTATGCCTATAAGGAAA//AATTCTTATAGGTTATAACA//TCTGTATGC-TTCTGGTTTA
AACTGCTCATGGTTATAACA//TCTGTATGCCTCATGAGTAA
ATCGATTAGAGGTTATAACA//TCTGTATGCCTTAGAAACAC
AATCGATTAAGGTTATAACA//TCTGTATGTCTTAAGAAGCC
AAAGTTTAAAGGTTATAACA//TCTGTATGCCTAAAGAGTGT
TTTAGTTTAAGGTTATAACA//TCTGTATGCCTTAACACAGC
ATGCCCTAAAGGTTATAACA//TCTGTATGCCTATAAGGAAA//AATTCTTATAGGTTATAACA//TCTGTATGCCTAAATACATT
ACATACTTAAGGTTATAACA//TCTGTATGCCTATAACTTAA
GTTTCTAATAGGTTATAACA//TCTGTATGCCTATAAGGAAA//AATTCT**TATA**AGGAAATATA//AATTCT**TATA**AGGAAATATA//AATTCTTATAGGTTATAACA//TCTGTATGCCTCTAATCGAT
TCTCGTGTTAGGTTATAACA//TCTGTATGCCTGTTAGCGTG
GTTCGATCAAGGTTATAACA//TCTGTATGCCTCAAAATAAT
TCAATCTAGAGGTTATAACA//TCTGTATGCCTATAAGGAAA//AATTCTTATAGGTTATAACA//TCTGTATGCCTAGACGCATA
GTGGGCTACAGGTTATAACA//TCTGTATGCCTATAAGGAAA//AATTCTTATAGGTTATAACA//TCTGTATGCCTACATCGAAA
ATAGTCAAACGGTTATAACA//TCTGTATGCCAAAAATGCAC
TTGAGGTATAGGTTGTAACA//TCTGTATGCCTATAGCTGTA
TCGATTTATAGGTTATAACA//TCTGTATGCCTATAAGGAAA//AATTCTTATAGGTTATAACA//TCTGTATGCCTATAAACACA
CCCGGATATAGGTTATAACA//TCTGTATGCCTATATATGAC
GCCGTCTAGAGGTTATAACA//TCTGTATGCCTAGAGGCAGG
ACGCTATAAAGGTTATAACA//TCTGTATGCCTATAAGGAAA//AATTCTTATAGGTTATAACA//TCTGTATGCCTATAAGGAAA//AATTCTTATAGGTTATAACA//TCTGTATGCCTAAAGGCGCA
TCGGCATATAGGTTATAACA//TCTGTATGCCTATAACGGCT
TCGCCTTCAAGGTTATAACA//TCTGTATGCCTATAAGGAAA//AATTCTTATAGGTTATAACA//TCTGTATGCCTCAACACGCG
ATAGATTAAAGGTTATAACA//TCTGTATGCCTATAAGGAAA//AATTCTTATAGGTTATAAAA//TCTGTATGCCTATAAGGAAA//AATTCTTATAGGTTATAAAA//TCTGTATGCCTAAACAAATA
GGCGGTTTAAGGCTATAACA//TCTGTATGCCTTAATTGGCA
ATCGATTAGAGGTTGTAACA//TCTGTATGCCTATAAGGAAA//AATTCTTATAGGTTATAACA//TCTGTATGCCTAAGAACACA
GGCCCTTTTAGGTTATAACA//TCTGTATGCCTAGAAGGAAA//AATTCTTATAGGTTATAACA//TCTGTATGCCTTTAGGGGCC
AGTTAATGTAGGTTATAACA//TCTGTATGCCTATAAGGAAA//AATTCTTATAGGTTATAACA//TCTGTATGCCTGTATCAAAT
CGAATGTTTAGGTTATAACA//TCTGTATGCCTTTATATTTG
GCGTGCTTGAGGTTATAACA//TCTGTATGCCTTGAAACGAA
TTACACTTGTGGTTATAACA//TCTGTATGCCTTGTATACTA
AACGACTGTTGGTTATAACA//TCTGTATGCCTGTTGCCTGC
TTTCGTTAAAGGTTATAACA//TCTGTATGCCTATAAATTAA
TTACTGTATAGGTTATAACA//TCTGTATGCCTATATATGTC
TGGTTCTTTAGGTTATAACA//TCTGTATGCCTTTAGGGTGC
ATCACATTTAGGTTATAACA//TCTGTATGCCTTATAGGTTA
GTCAGGAAAAGGTTATAACA//TCTGTATGCCTATAAGGAAA//TGACAATTCT**TATA**AGGAAA//AATTCTTATAGGTTATAACA//TCTGTATGCCTTACATAGCC
CCTTATCCAAGGTTATAACA//TCTGTATGCCTCCAAGTAGA
TACTCTTATAGGTTATAACA//TCTGTATGCCTGTTAAACAG
CAGCATCACAGGTTATAACA//TCTGTATGCCTATAAGGAAA//AATTCTTATAGGTTATAACA//TCTGTATGCCTATAAGGAAA//AATTCTTATAGGTTATAACA//TCTGTATGCCTCACACACTG
CTTGTATTGAGGTTATAACA//TCTGTATGCCTTGAACACTT
GGAGCATATAGGTTATAACA//TCTGTATGCCTATACGCACC
GTACCTTATAGGTTATAACA//TCTGTATGCCTTTATAAACG
ATGTACATTAGGTTATAACA//TCTGTATGCCATTAGCGAAT
CGAGTCTTTAGGTTATAACA//TCTGTATATTTAGTGCCTATAAGGAAA//-------------//TCTGTATGCCTTTATATAAC
CAGTACTATAGGTTATAACA//TCTGTATGCCTATATAGCTA
CACGTTACAAGGTTATAACA//TCTGTATGCCTATAATGACA
AAACAATAAAGGTTATAACA//TCTGTATGCCTAAACCTATG
ATTCCTTATAGGTTATAACA//TCTGTATGCCTATAAGGAAA//AATTCTTATAGGTTATAACA//TCTGTATGCCTATAAGAATA
GTACTATTGTGGTTATAACA//TCTGTATGCCTTGTACCTAC
ACTGCTAAAAGGTTATAACA//-------------------//AATTCTTATAGGTTATAACA//TCTGTATGCCAAAAGACATA
ATTTCATGCAGGTTATAACA//TCTGTATGCCTGCAGCCACA
TTTCCTTGATGGTTATAACA//TCTGTATGCCTGATCACAAA
AGGCCTTTAAGGTTATAACA//TCTGTATGCCTATAAGGAAA//AATTCTTATAGGTTATAACA//TCTGTATGCCTTAACGTGAA
CTGCTTATTAGGTTATAACA//TCTGTATGCCTTATTAAAGA

KolobokP-2_MeMe

~~AAGGCAGTCTGGCCAAAGAC//ATCTTGTGCCTAGTTAGTAC~~
ACGAGGAGTTGGCCAAAGAC//GTCTTGTGCCTAGTTAGTAC//CGCTATATCAGGCCAAAGAC//GTCTTGTGCCTAGTTAGTAC//CGCTATATCAGGCCAAAGAC//GTCTTGTGCCTAGTTAGTAC//CGCT
ATATCAGGCCAAAGAC//GTCTTGTGCCTACTGACGGT
GCTACCTTATGGCCAAAGAC//GTCTTGTGCCTTATAGCGCG
TGCATATCAAGCCAAAGACC//ATATTGTGCCTAGTTAACGT
TCAGTGTACTGGCCAAAGAC//GTCTTGTGCCTAGTTAGTAC//CGCTATATCAGGCCAAAGAC//GTCTTGTGCCTACTGCTGAA
AATAAAATCTGGCCAAAGAC//GTCTTGTGCCTAGTTAGTAC//CGCTATATCAGGCCAAAGAC//GTCTTGTGCCTAGTTAGTAC//-------------------//GTCTTGTGCCATCTCCAGCT
AATAAGAAGTGGCCAAAGAC//GTCTTGTGCCAAGTGCTTGA
TCCAGTAGACGGCCAAAGAC//GTCTTGTGCCAGACACCCTA
AAGGCAGCCTGGCCAAAGAC//ATCTTGTGCCTAGTTAGTAC
AACATTCTATGGCCAAAGAC//GTCTTGTGCCTAGTTAGTAC//CGCTATATCAGGCCAAAGAC//GTCTTGTGCCCTATGCACCG
ATGCGCCCTGGGCCAAAGAC//GTCTTGTGCCTAGTTAGTAC//CGCTATATCAGGCCAAAGAC//GTCTTGTGCCTAGTGTGTGG
TTTACAAGACGACCAAAGAC//-------------------//CGCTATAGACGGCCAAAGAC//-------------------//CGCTATATCAGGCCAAAGAC//GTCTTGTGTCAGACCTGAAT
TCTCTCAAGTGGCCAAAGAC//GTCTTGTGCCAAGTCCAGCG
AACGATACCTGGCCAAAGAC//GTCTTGTGCCTAGTTAGTAT//CGCTATATCAAGCCAAAGAC//GTCTTGTGCCTAGTTATCAC
ACACTGTACTGGCCAAAGAC//GTCTTGTGCCAACTGTCAAC
CACAAAAGGAGGCCAAAGAC//GTCTTGTGCCAGGACGATAA
ACCCGTAACGGGCCAAAGAC//GTCTTGTGCCTAGTTAGTAC//CGCTATATCAGGCCAAAGAC//GTCTTGTGCCTAGTTAGTAC//CGCTATATCAGGCCAAAGAC//GTCTTGTGCCAACGGGATAA

```
ATTGATATCTGGCCAAAGAC//GTCTTGTGCCTAGTTAGTAC//CGCTATATCAGGCCAAAGAC//GTCTTGTGCCTAGTTGTGTG
TGGCGCCAGTGGCCAAAGAC//GTCTTGTGCCTAGTTAGTAC//CGCTATATCAGGCCAAAGAC//GTCTTGTGCCATATCGATAT
AGGAATATCAGGCCAAAGAC//GTCTTGTGCCTAGTTAGTAC//CGCTATATCAGGCCAAAGAC//GTCTTGTGCCTATAAGGTCA
TCATTCTTATGGCCAAAGAC//GTCTTGTGCCTAGTTAGTAC//CGCTATATCAGGCCAAAGAC//GTCTTGTGCCTAGTTATGCT
TTTTGGTAGTGGCCAAAGAC//ATCTTGTACCTAGTGTTGTT
```

KolobokP-3_MeMe

```
GTTTGCTGAAGGTGTCGGCC//GGGTGACACCTAAATCTAAC//CCATATATATGGTGTCGGCC//GGGTGACACCTGAAGGTATA
TGAGCCGTACGGTGTCGGCC//GGGTGACACCAAAATCTAAC//TTCCCATATAGGTGTCGGCC//GGGTGACACCGTACCTAAAA
ATAAGCTAATGGTGTCGGTC//GGGTGACACCTAAATCTAAC//TTCCCATACA----------//GGGTGACACCTCATAGAGTT
CACCAGATCTGGTGTCGGCC//GGGTGACACCATCTGTTTTT
ACATGAATTTGGTGTCGGCC//GGGTGACACCTAAATCTAAC//TTCCCATATAGGTGTCGGCC//GGGTGACACCTAAATCTAAC//TTCCCATATAGGTGTCGGCC//GGGTGACACCATTTTGGAGA
AACGAAGAATGGTGTCGGCC//GGGTGACACCGAATATAGCC
CCCATGTTTAGGTGTCGGCC//GGGTGACACCTTTAATAGTT
ACTAAATATAGGTGTCGGCC//GGGTGACACCTCTTATCCGC
AAAGGTCTATGGTGTCGGCC//GGGTGACACCCTATACGAAG
TTTCAAATATGGTGTCGGCC//GGGTGACACCTAAATCTAAC//TTCCCATATAGGTGTCGGCC//GGGTGACACCTAAATCTAAC//TTCCCATATAGGTGTCGGCC//GGGTGACACCATATGAGTGT
CGGGGTGTATGGTGTCGGCC//GGGTGACACCTAAATCTAAC//TTCCCATATAGGTGTCGGCC//GGGTGACACCGTATATTACA
CTCAATGCAGGGTGTCGGCC//GGGTGACACCTAAATCTAAC//TTCCCATATAGGTGTCGGCC//GGGTGACACCGCAGAGTTGG
TATTCCTTCAGGTGTCGGCC//GGGTGACACCTAAATCTAAC//TTCCCATATAGGTGTCGGCC//GGGTGACACCTTCAGGTAAC
GTACCTTAACGGTGTCGGCC//GGGTGACACCTAAATCTAAC//TTCCCATATAGGTGTCGGCC//GGGTGACACCTAACGTAGTA
CTGGGTGTAAGGTGTCGGCC//GGGTGACACCGTAAGATAAG
ACGTTTCTATGGTGTCGGCC//GGGTGACACCTAAATCTAAC//TTCCCATATAGGAGTCGGCC//GGGTGACACCTTCTATCCGT
GTTTCTATCCGGTGTCGGCC//GGGTGACACCTAAATCTAAC//TTCCCATATAGGTGTCGGCC//GGGTGACACCTAAATCCGTG
ATATATATATGGTGTCGGCC//GGGTGACACCATATATAGAA
GTACACAAAAGGTGTCGGCC//GGGTGACACCAAAAAATATA
TTGCACTGATGGTGTCGGCC//GGGTGACACCTAAATCTAAC//TTCCCATATAGGTGTCGGCC//GGGTGACACCTGATATAACA
ACGGCCATTAGGTGTCGGCC//GGGTGACACCATTAGAAGAC
TGTCTCATCAGGTGTCGGCC//GGGTGACACCTAAACCTAAC
TTTCAAATATGGTGTCGGCC//GGGTGACACCTAAATCTAAC//TTCCCATATAGGTGTCGGCC//GGGTGACACCATATGCGTGT
CATCTCATAAGGTGTCGGCC//GGGTGACACCTAAACCTAAC
```

KolobokP-4_MeMe

```
CACTCCTTTAGGCTGAACAC//GTGATCAGCCTATAAGAGAA//TACTCTTATAGGCTGAACAC//GTGATCAGCCTTTAGCTCCG
TATAATTATAGGCTGAACAC//GTGATCAGCCTATAAGAGAA//TACTCTTATAGGCTGAACAC//GTGATCAGCCCTATAACACA
TAAGTCTTAAGGCTGAACAC//GTGATCAGCCTAATTAAGAA
GTGAGCTACAGGCTGAACAC//GTGATCAGCCTATAAGAGAA//TACTCTTATAGGCTGAACAC//GTGATCAGCCTACATCGAAA
AATTTCTAGAGGCTCAGAAC//GTGATCAGCCTAGACACATA
TGTGTCTTAAGGCTGAACAC//GTGATCAGCCTATAAGAGAA//TACTCTTATAGGCTGAACAC//GTGATCAGCCTTAACTTGTT
TGTACATAGAGGCTGAACAC//GTGATCAGCCTAGAGCGCCT
-TTCGGATGAAGCTGAACAC//GTGATCAGCCTATAAGAGAA//TACTCTTATAGGCTGAACAC//GTGATCAGCCATAAGCGTTT
```

KolobokP-5_MeMe

```
GAAGTGTGAAGGTGGCAGGG//CGCTGCCACCTTAAGTTTAC
-TAAAATAGGAGTGGCAGGG//CCCTGCCACCTTAAGTTTTT//ATTTCCTTAAGGTGGCAGGA//CCCTGCCACCTTAAGTTTTT//ATTTCCTTAAGGTGGCAGGG//CCCTGCCACCGAAGCGTAGT
CTTTTTAAAAGGTGGCAGGG//CCCTGCCACCTTAAGTTTTT//ATTTCCTTAAGGTGGCAGGG//CCCTGCCACCAAAGATTTTC
TAAACATAACGGTGGCAGGG//CCCTGCCACCAACGCCCGTG
```

KolobokP-6_MeMe

```
AATTCTTGCAGGTTACACTC//TAATCAGTCCTGCAACATTT
TTAATCTATAGGTTACGCTC//TAATCAGTCCTTTAAGGAAC//TGCTCTTCTAGGTTCACTCT//TAATCAGTCCTTTAAGGAAC//TGCTCTTCTAGGTTTCACTC//TAATCAGTCCTATATCTGTG
TTCTTATCTAGGTTACACTC//TAATCAGTCCATTTAACGAT
TGATTCAACAGGTTACACTC//TAATAAGTCCAACAGTTAAA
GCGTGCTTATGGTTACACTC//TAATCAGTCCTTTAAGGAAC//TACTCTTCTAGGTTACACTC//TTATCAGTCCTTATCTTCCT
GTTTTATCTAGGTTACACTC//TAATCAGTCCTCTATATCCA
TGTCCAAGAAGGTTACACTC//TAATCAGTCCTTTAAGGAAC//TACTCTTCTAGGTTACACTC//TAATCAGTCCTTTAAGGAAC//TACTCTTCTAGGTTACACTC//TAATCAGTCCAAGAAAATTA
TGTGCGTATAGGTTACACTC//TAATCAGTCCTATATAAATG
TCAGCCTTCAGGTTACACTC//TAATCAGTCCTTCAGACCAA
GAATCATCGAGGTTACACTC//TAATCAGTCCTCGAAGCGCT
TTTATCATTAGGTTACACTC//TAATCAGTCCATTAGGCGAA
CGTTGTAAAAGGTTACACTC//TAATCAGTCCTTTAAGGTTA
ATCTGTTGTTGGTTACACTC//TAATCAGTCCTTTAAGGAAC//TACTCTTCTAGGTTACACTC//TAATCAGTCCTTGTTGAAGG
TTACGAGATAGGTTACACTC//TAATCAGTCCTATACTTAAA
```

KolobokP-7_MeMe

```
TCGGGCTTTAGGTAGCAGAT//AACTGCTACCTTAAGGACCA//GATGTCTTAAGGTAGCAGAT//AACTGCTACCTTATCGTATG
CCCGCCCTTAGGTAGCAGAT//AACTGCTACCTTAGCGTATC
AAGTCCATTAGGTAGCAGAT//AACTGCTACCTTAAGGACCA//GATGTCTTAAGGTAGCAGAT//AACTGCTACCTTAAGGACCA//GATGTCTTAAGGTAGCAGAT//AACTACTACCTTATATTAAC
TGCATGTTTTGGTAGCAGAT//AACTGCTACCTTAAGGACCA----------------------//AACTGCTACCTTTGTAAACT
CGCACACTTAGGTAGCAGAT//AACTGCTACCTTAAGGACCA//GATGTCTTAAGGACCAAGAC//GATGTCTTATGGTAGCAGAT//AACTGCTACCTTAAGGACCA//
//GATGTCTTAAGGACCAAGAC//GATGTCTTAAGGTAGCAGAT//AACTGCTACCTTAGACTGGT
TGAGCTATTGGGTAGCAGAT//AACTGCTACCTTAAGGACCA
AGTGTCTTAAGGTAGCAGAT//AACTGCTACCTTGATTATTT
ATGAACATAAGGTAGCAGAT//AACTGCTACCTAAAGGAGGT
GATGTATTTAGGTAGCAGAT//AACTGCTACCTTAAGGACCA//GATGTCTTAAGGTAGCAGAT//AACTGCTACCTTAATGATGC
AATGTCTTAAGGTAGCAGAT//AACTGCTACCTTAAGGACCA
GGAAGGCTTAGGTAGCAGAT//AACTGCTACCTTATATTTTA
TAAGAATTAAGGTAGCAGAT//AACTGCTACCTTAAGGACCA
AGTGTCTTAAGGTAGCAGAT//AACTGCTACCTTTAATGTCT
TGTGTGCTTAGGTAGCAGAT//AACTGCTACCTTATTAAGTT----------------------//AACTGCTACCTTAGTTGTTT
AAATCTCGAATCTGTGGCCG//AACTGCTACCTAAACTGGAG
AACTGCTACCTTAAGGACCA//AAATCTCGAATCTGTGGCCG
CAGAGAGTAAGGTAGCAGAT//AACTGCTACCTTAAGGACCA
AATGTCTTAAGGTAGCAGAT//AACTGCTACCTTAAGGACCA
AACTATCTTAGGTAGCAGAT//AACTGCTACCTTAACAAAAA
TCCCGCTTTCGGTAGCAGAT//AACTGCTACATTCGCACGC
```

Figure S4. Complete, soloLTDR, and tandem insertions of *KolobokP* families in the genome of *Gigantopelta aegis*. LTDRs are highlighted in yellow, while internal portions are in cyan. TSDs are colored in red.

KolobokP-1_GiAe
```
AGCACATTATGGTCTCACTA//TTGTGAGACCTATGTTCAAA
CAGTGGCTTAGGTCTCACTA//TAGTGAGACCTTACCCTAAA
ATTTAACTTAGGTCTCACTA//TTGTGAGACCTTACACACCA
CACCAGCATAGGTCTCACTA//TAGTGAGACCTTAAACAAAA//GTTACATTATGGTCTCACTA//TAGTGAGACCATAGATGAAT
TTCCCTCTGAGGTCTCACTA//TTGTGAGACCTTAAACAAAA//GTTACATTATGGTCTCACTA//TTGTGAGACCTTAAACAAAA//GTTACATTATGGTCTCACTA//TTGTGAGACCTGATACCGAT
CTTGAATTTAGGTCTCACTA//TAGTGAGACCTTAAGGAGAT
TGGCAGTTAAGGTCTCACTA//TAGTGAGACCTTAAACAATA
ACCGACTATAGGTCTCACTA//TTGTGAGACCTTAAACAAAA//GTTACATTATGGTCTCACTA//TTGTGAGACCATAGACTAAA
CTGCCTCTTAGGTCTCACTA//TAGTGAGACCTTAAACGTTT
TGCAAGGTTAGGTCTCACTA//TTGTGAGACCTTAGAAAATT
TGAGACCTTAGGTCTCACTA//TTGTGAGACCTTAATTAAAC//GTTACATTATGGTCTCACTA//TTGTGAGACCTTAATTAAAC
TCACCACTATGGTCTCACTA//TTGTGAGACCTTAAACAAAA//GTTACATTATGGTCTCACTA//TTGTGAGACCTATAGTGCTT
```

KolobokP-2_GiAe
```
TAAAATAATAGGTTGGACTA//GAGTACTACCAATACAGTCA
ATCCTGATCAGGTTAGACTA//GAGTACTACCATCAGGAGCA
AATACTACTAGATTGGACTA//GAGTACTACCAGTGGTATCA
TGTCGTATATGGTTCGACTA//GAGTACTATCACATACAGCC
CTCATGTATAGGTTGGACTA//GAGTACTACCTATAAGCAGA//TTTCTCTATAGGTTGGACTA//GAGTACTACCTTAAATATTA
GATTATATCAGGTTGGACTA//GAGTACTACCTATAAGCAGA//TTTCTCTATAGGTTGGACTA//GAGTACTACCATCACCAACG
TGACCTTGTTGGTTGGACTA//GAGTACTACCTATAAGCAGA//TTTCTCTATAGGTTGGACTA//GAGTACTACCTGTTATGGAA
TACTACTAATGGTCGGACTA//GAGTACTACCTAATAGGCAG
TTTCTCTATAGGTTGGACCA//GAGTACTACCTGTAGGCGTC
GATACTTTCAGGTTGGACTA//GAGTACTACCTATAAGCAGA//TTTCTCTATAGGTTGGACTA//GAGTACTACCTTCAGTTTTA
AGTAGTTAAAGGTTGGACTA//GAGTACTACCTAAAGGAAAA
TACTATTAACGGTTGGACTA//GAGTACTACCTATAAAAATC
ACACGTTTATGGTTGGACTA//GAGTACTACCTATAAGCAGA
TTCTTCTGTAGGTTGGACTA//GAGTACTACCTATAAGCAGA
TTCTTCTGTAGGTTGGACTA//GAGTACTACCTATAAGCAGA
AGCATGACACGGTTGGACTA//GAGTACTACCTATAAGCAGA//TTTCTCTCTAGGTAGGACTA//GAGTACTACCAGACAGGGGC
CGACCATATAGGTTGGACTA//GAGTACTACCTATAGCTATA
GTTTACTATAGGTTGGATTT//GAGTACTACCTATAGTCCGC
ACGTCGAACGGGTTGGACTA//GAGTACTACCAACTCTGACA
AACAAATATAGGTTGGACTA//GAGTACTACCTATAGGATGA
GAGTTCTTAAGGTTGGACTA//GAGTACTACCTTAACGACTC
ATAGCTTATAGGTTGGACTA//GAGTACTACCTATAAGCAGA//TTTCTCTATAGGTTGGACTA//GTATACTACCTTAAATTATT
CAATGATGTTGGTTGGACTA//GAGTACTACCTGTAATATCA
GGCGGTTTCTGGTTGGACTA//GAGTACTACCTTCTGGGTTA
TACATGAGTAGGTTGGACTA//GAGTACTACCAGTAAGTGCA
TAACAATGTAGGTTGGACTA//GAGTACTACCTGTAACCTGA
CTGGATTATAGGTTGGACTA//GAGTACTACCTATATGTGCC
TTCCCTTTAAGGTTGGGCTA//GAGTACTACCTTAAGAGCTC
TTCCCTTTAAGGTTGGGCTA//GAGTACTACCTTAAGAGCTC
TTTCTCTATAGGTTGGACTA//GAGTACTACCTATAAGCAGA
```

KolobokP-3_GiAe
```
TAAGTTTTCAGGGTCCATTA//CATTACAACCTTCAGTTTTG
CACTTGTTTAGGTTCCATTA//CATTACAACCTATATTGAAA//TTGCTGTATAGGTTCCATTA//CATTACAACCTTTAGTGGTT
GATGGTTATAGGTTCCATTA//CATTACAACCTCTACGTCCT
GGGTGTTGAAGGTTCCATTA//CATTACAACCTGAACGACCC
ACAGTCTGGAGGTTCCATTA//CATTACAACCTATATTGAAA//TTGCTCTATAGGTTCCATTA//CATTACAACCTATATTGAAA//TTGCTCTATAGGTTCCATTA//CATTACAACCTGGAGATGGT
TATGGATAGAGGTTCCATTA//CATTACAACCTAGATGTGTT
CACCACTCATGGTTCCATTA//CATTACAACCTATAATGAAA
CTGTACTACAGGTTCCATTA//CATTACAACCTATATTGAAA//TTGCTGTATAGGTTCCATTA//CATTACAACCTACACAACAT
ACCCTCTTCTGGTTCCATTA//CATTACAACCTTCTCTCTCA
CAGACCAATAGGTTCCATTA//CATTACAACCTATATTGAAA//TTGCTGTATAGGTTCCATTA//CATTACAACCTATACACAAT
CGAGGTTGTAGGTTCCATTA//CATTACAACCTCTAGCTTGT
TGGCACAGCAGGTTCCATTA//CATTACAACCTATATTGAAA//TTGCTGTATAGGTTCCATTA//CATTACAACCAGCAGATGTA
GCTCCGAGTAGGTTCCATTA//CATTACAACCAGTAGGCACG
CGGTGGTATAGGTTCCATTA//CATTATAACCTATAGCTTAG
```

KolobokP-4_GiAe
```
ACACACTGAAGGTATCTCAC//GTGTGATCCCTGAATAGGAA
ATGTGCTGCAGGTATCTCAC//GTGTGATCCCTTAAGAGGGG//ATAGTGTTTAGGTATCTCAC//GTGTGATCCCTGCAACCAGC
ACGGGTTTCAGGTATCTCAC//GTGTGATCCCTTAAGAGGGG//ATAGTGTTTAGGTATCTCAC//GTGTGATCCCTTCAACACAC
TTCCCTTGTAGGTATCTCAC//GTGCGATCCCTGTACTACTC
GCAGCCTTCAGGTATCTCAC//GTGTGATCCCTTCAGAACTC
CTCGCCTGATGGTATCTCAC//GTTTGATCCCTGATGCGTGG
GAGCATATAAGGTATCTCAC//GTGTGATCCCATAAAGGATC
ACAGTGTTAAGGTATCTCAC//GTGTGATCCCTTAACATAAT
TTGTTCTGGAGGTATCTCAC//GTGTGATCCCTGGACAGACA
TGTACCACCAGGTATCTCAC//GTGTGATCCCTTAAGAGGGG//--------------------//GTGTGATCCCACCAGCCTCA
ATTTCGTTTAGGTATCTCAC//GTGTGATCCCTTCAATCAGG
ATTTCGTTCAGGTATCTCAC//GTGTGATCCCTTCAATCAGG
TGGAAGTACAGGTATCTCAC//GTGTGATCCCTATACATATT
GGTTAGTTATGGTATCTCAC//GTGTGATCCCTTATAGTTTA
AAGAGGTTCAGGTATCTCAC//GTGTGATCCCTTAAGGCTGG
GTCCATTTCAGGTATCTCAC//GTGTGATCCCTTCACATAGA
TGTCAGTGTAGGTATCTCAC//GTGTGATCCCTGTAGCGTGT
GTTCTGCAATGGTATCTCAC//GTGTGATCTCTAATAATATA
ATACATTTTAGGTATCTCAC//GTGTGATCCCTTAAGAGGGG//ATAGTGTTTAGGTATCTCAC//GTGTGATCCCTTAACATTTC
TATACGTTGAGGTATCTCAC//GTGTGATCCCTTGAGTGTAG
AAAAACTTAAGGTATCTCAC//GTGTGATCCCTTAAAGACTT
AAACTGTTAAGGTATCTCAC//GTGTGATCCCTTAAATAACT
AAGCATTGTAGGTATCTCAC//GTGTGATCCCTGTAGTCACA
ATTACGTGCAGGTATCTCAC//GTGTGATCCCTTAAGAGGGG//ATAGTGTTTAGGTATCTAAC//GTGTGATCCCTGCACATGTC
TTAAACTTTAGGTATCTCAC//GTGTGATCCCTTAAGTATTT
TAGCAGTTACGGTATCTCAC//GTGTGATCCCTTAAGGTCGA
GAAATGTGCAGGTATCTCAC//GTGTGATCCCTGCAGGTTTT
GAACGCTGGAGGTATTTACC//GTGTGTTCCCTGGAACGTTT
CCAGCCTGATGGTATCTCAC//GTGTGATCCCTTAAGAGGGG//ATAGTGTTTAGGTATCTCAC//GTGTGATCCCTGATGGGTCT
TGCTATACAAGGTATCTCAC//GTGTGATCCCTTAAGAGGGG//ATAGTGTTTAGGTATCTCAC//GTGTGATCCCACAAGGACAG
TTGAACTTTAGGTATCTCAC//GTGTGATCCCTTAAGAGGGG//ATAGTGTTTAGGTATCTCAC//GTGTGATCCCTTAATGACGA
GCAATATACAGGTATCTCAC//GTGTGATCCCTTAAGAGGGG//ATAGTGTTTAGGTATCTCAC//GTGTGATCCCTACACTGAAG
GGTGGATTAAGGTATCTCAC//GTGTGATCCCTTAAGAGGGG//ATAGTGTTTAGGTATCTCAC//GTGTGATCCCGTAAGTTAGA
TGTGTATTTAGGTATCTCAC//GTGTGATCCCTGTTTGTGTG
GGCCACAGTAGGTATCTCAC//GTGTGATCCCTTAAGAGGGG//ATAGTGTTTAGGTATCTCAC//GTGTGATCCCAGTAAGCTTT
ATGTGTTACAGGTATCTCAC//GTGTGATCCCTACACACCAA
TACGCATGCAGGCATCTCAC//GTGTGATCCCTGCACATGGC
TAACACTGAAGGTATCTCAC//GTGTGATCCCTGAACATCAT
```

TGATCTTATAGGTATCTCAC//GTGTGATCCCTTAAGAGGGG//ATAGTGTTTAGGTATCTCAC//GTGTGATCCCTATATCTGCC
TAAGTAATTTGGTATCTCAC//GTGTGATCCCTTAAAACGGT
CCGATGTGCAGGTATCTCAC//GTGTGATCCCTTAAGAGGGG---------------------//GTGTGATCTCTGCACCCAAA
GCAACCTTAAGGTATCTCAC//GTGTGATCCCTTAATTTTTG
TATCAGTTTAGGTATCTCAC//GTGTGATCCCTTAAGAGGGG//ATAGTGTTTAGGTATCTCAC//GTGTGATCCCTTAACTCGAA
GTCCATTTCAGGTATCTCAC//GTGTGATCCCTTCACATAGA

KolobokP-5_GiAe
GTATATTATAGGGTGTATAA//GACTACTACCTTACAGCAAT
TAAAAGTAGTGGGTTACAAC//GACTACTACCTAGTAGTAGT
TTACATTATAGGGTGTATAA//GACTACTACCTATACTGCTA//TTCCTGTATAGGGTGTATAA//GACTACTACCTATAAAAGTC
CTCTGCTATAGGGTGTATAA//GACTACTACCTGCAGAACGA
TTCCTGTATAGGGTGTATAA//GACTACTACCAGTAATGGAG
CGGTCCTATAGGGTGTATAA//GACTACTACCTTTAAAGAAA
TTTTGTATTAGGGTGTATAA//GACTACTACCATTAATATGC
CCCAATATAAGGGTGTATAA//GACTACTACCTATACTGCTA//TTCCTGTATAGGGTGTATAA//GACTACTACCATAAACAAGC
TAAGGCTTCAGGGTGTATAA//GACTACTACCTTCAGATCAA
ATAACCTTTAGGGTGTATAA//GACTACTACCTATACTGCTA//TTCCTGTATAGGGTGTATAA//GACTACTACCTTTAAGATAT

AAGTGTTATAGGGTGTATAA//GACTACTACCTATACTGCTA//TTCCTGTATACAGTATAGGGTGTATAA//GACTACTACCTATACTGTTA

CCTCTCTTTAGGGTGTATAA//GACTACTACCTATACTGCTA//TTCCTGTATAGGGTGTATAA//GACTACTACCTTTAGGTTCC
GATGTCTATAGGGTGTATAA//GACTACTACCTATAGCATTA
TAACTCTTAAGGGTGTATAA//GACTACTACCTATACTGCTA//TTCCTGTATAGGGTGTATAG//GACTACTACCTTAAATGTTT
TTCCTGTATAGGGTGTGTAA//GACTACTACCTATGCGCTGG
GGCCACTAAAGGGTGTATAA//GACTACTACCTATACGCTCG
AATCCCTATAGGGTGTATAA//GACTACTACCTATAGACAAG
AATGTCTATAGGGTGTATAA//GACTACTACCTATACACAGA
AGATAGTATAGGGTGTATAA//GACTACTACCTATACTGATA//TTCCTGTATAGAGTGTATAA//GACTACTACCTATATGAATA
AAGCACTTCAGGGTGTATAA//GACTACTACCTTCAGGTGAA

KolobokP-6_GiAe
CGCTCTATCTGGCCTCAGAC//GTCTGATGCCATCTTGCGAG
CTACAAAGCTGGCCTCAGAC//--------------------//TACTATATCAGGCCTCAGAC//GTCTAATGCCAGCTTACATG
TACCGGAGTAGGCCTCAGAC//GTCTGATGCCTATCAGACCA//TACTATATCAGGCCTCAGAC//GTCTGATGCCAGTAATGTGC
GACGTCAGCTGGCCTCAGAC//GTCTGAGGCCAGCTCCTGAC
GTGTTCATCTGGCCTCAGAC//GTCTGAGGCCTATCAGACCA//TACTATATCAGGCCTCAGAC//GTCTGA----ATGAATGAAT
TGTGAGAACTGGCCTCAGAC//GTCTAATGCCAACTCGTCAG
TTTCATATATGGCCTCAGAC//GTCTGAGGCCATATCATAGC
GACAACAGGTGGCCTCAGAC//GTCTGAGGCCAGGTTGTCAT
TGGACCAGCTGGCCTCAGAC//GTCTGAGGCCAGCTTGCCCA
TCAAACGTATGGCCTCAGAC//GTCTGAAGCCTATCAGACCA//TACTATATCAGGCCTCAGAC//GTCTGAGGCCGTATACGCTT
TTTACTAAATGGCCTCAGAC//GTCTGAGGCCTATCAGACCA//TACTATATCAGGCCTCAGAC//GTCTGAGGCCAAATAAGTAT
AAATGGAGCTGGCCTCAGAC//GTCTGAGGCCAGCTGCTTGA
CGCCAGAGACGGCCTCAGAC//GTCTGAGGCCTATCAGACCA//TACTATATCAGGCCTCAGAC//GTCTGAGGCCAGACCGGTGT
TACAATATTTGGCCTCAGAC//GTCTGAGGCCTATCTTGGGG
GCGTCGTGTTGGCCTCAGAC//GTCTGAGGCCTGTTGCCATT
CATACCATATGGCCTCAGAT//GTCTGATGCCATATTTACTC//TACTATATCAGGCCTCAGAC//GTCTGATGCCATATTTACTC
ATTAGGCTCAGGCCTCAGAC//GTCTGAGACCTATCAGACCA//TACTATATCAGGCCTCAGAC//GTCTGAGGCCAGGCTAACAG
TGCCATAACTGGCCTCAGAC//GTCTGATGCCAACTTGGTGC
CTCTGAATCTGGCCTCAGAC//GTCTGAGGCCTATCAGACCA//TACTATATCAGGCCTCAGAC//GTCTGAGGCCATCTGGGCAC
TTGTTTAGATGGCTTCAGAC//GTCTGATGCCAGATTATCCA
TTTTACAGCCGGCCTCAGAC//GTCTGAGGCCAGCCGTTCCA
TCAGACAGTTGGCCTCAGAC//GTCTGAGGCCTATCAGACCA//TACTATATCAGGCCTCAGAC//GTCTGAGGCCAGTTCGGGGT
GTCGTGATATGGCCTCAGAC//GTCTGAGGCCATATGGAGAT
GACGTAAGTTGGCCTCAGAC//GTCTGAGGCCAGTTAGTTGT
ACCGCGAAGTGGCCTCAGAC//GTCTGAGGCCTATCAGACCA//TACTATATCAGGCCTCAGAC//GTCTGAGGCCAAGTCGTACA
ACGGATATCAGGCCTCAGAC//GTCTGAGGCCAGAACCAAAT
TCTGGAGTATGGCCTCAGAC//GTCTGATGCCTATCAGACCA//--------------------//GTCTGATGCCGTATGTCGCT
GCTGCCAGCTGGCCTCAGAC//GTCTGAGGCCTATCAGACCA//TACTATATCAGGCCTCAGAC//GTCTGAGGCCAGCTGCCAGG
GACCACAGTTGGCCTCAGAC//GTCTGAGGCCAGTTGTTTGT
TGTTGAATATGGCCTCAGAC//GTCTGAGGCCATATAGTACA
TTTCACACCTGGCCTCAAAC//GTCTGATGCCACCTGACCTG
ACTACGAGTTGGCCTCAGAC//GTCTGAGGCCTATCAGACCA//TACTATATCAGGCCTCAGAC//GTCTGAGGCCAGTTAAAAAT
TTGGGTACCTGGCCTCAGAC//GTCTGAGGCCACCTCCCTAT
CCTACTGTCTGGCCTCAGAC//GTCTGAGGCCGTCTGCCAGT
GACTGCAGGTGGCCTCAGAC//GTCTGAGGCCAGGTGTCGTT
GATTGACACGGGCCTCAGAC//GTCTGAGGCCTATCAGACCA//TACTATATCAGGCCTCAGAC//GTCTGAGGCCACGTCAAACA
CGGCGCGGATGGCCTCAGAC//GTCTGAGGCCTATCAGCCAA//TACTATATCAGGCCTCAGAC//GTCTGAGGCCGGATGGTTGA
TACTGTAGATGGCCTCAGAC//GTCTGAGGCCTATCAGACCA//TACTATATCAGGCCTCAGAC//GTCTGAGGCCAGATGAAATA
TATTACATGTGGCCTCAGAC//GTCTGAGGCCATGTGGATCT
TGGTTCATATGGCCTCAGAC//GTCTGAGGCCATATAAGCGT
ACCTGGATGTGGCCTCGGAC//GTCTGAGGCCTATCAGACCA//GTATCAAAGTTATATCAATG//GTCTGAGGCCATGTAGCGAT
GACTTGAGCTGGCCTCAGAC//GTCTGATGCCTATCAGACCA//TACTATATCAGGCCTCAGAC//GTCTGATGCCAGCTATGGAC
CAATCCACCTGGCCTCAGAC//GTCTGAGGCCAACTGCGGAA
GGTGTCGTCTGGCCTCAGAC//GTCTGAGGCCGTCTGCCTCT
ACGAGCAGATGGCCTCAGAC//GTCTGAGGCCTATCAGACCA//TACTATATCAGGCCTCAGAC//GTCTGAGGCCAGACTATGTG
GTCTCGAAATGGCCTCAGAC//GTCTGAGGCCAAATAGGTCG
TCAGCTATATGGCCTCAGAC//GTCTGAGGCCTATCAGACCA//TACTATATCAGGCCTCAGAC//GTCTGAGGCCATATAGACTG
CCTAAGAGATGGCCTCAGAC//GAATGAGGCCTATCAGACCA//TACTATATCAGGCCTCAGAC//GTCTGAGGCCAGATCATATG
TTCTGCTGATGGCCTCAGAC//GTCTGAGGCCTGATCATGTA
GGGGGCAGATGGCCTCAGAC//GTCTGAGGCCAGATGGAGTA
TCGCATAGAGGGCCTCAGAC//GTCTGAGGCCAGAGGTTGCA
GTGAGGAACTGGCCTCAGAC//GTCTGAGGCCAACTGATTGA
GGTTTGTGATGACCTCAGAC//GTCTGAAGCCTATCAGACCA//TACTATATCAGGCCTCAGAC//GTCTGAGGCCAGATTGTTTA
TATAGCACATGGCCTCAGAC//GTCTGAGGCCACATATGACT
TGCGACTGCTGGCCTCAGAC//GTCTGAGGCCTGCTCACTGC
CGCACTAAGTGGCCTCAGAC//GTCTGAGGCCTATCAGACCC//TACTATATCAGGCCTCAGAC//GTCTGAGGCCAAGTGATGCA
TGAACAATCTGGCCTCAGAC//GTCTGAGGCCATCTAGAAGT
CCCCAAACCTGGCCTCAGAC//GTCTGATGCCACCTAAGACG
AGTGTAATATGGCCTCAGAC//GTCTGATGCCATATAATGAA
CATGCTAACTGGCCTCAGAC//GTCTGAGGCCTATCAGACCA//TACTATATCAGGCCTCAGAC//GTCTGAGGCCAACTACCTAT
TGGCATGACTGGCCTCAGAC//GTCTGAGGCCTATAAGACCA//TACTATATCAGGCCTCAGAC//GTCTGAGGCCGACTGGTCGC
CGATTCACCAGGCCTCAGAC//GTCTGAGGCCTATCAGACCA//TACTATATCAGGCCTCAGAC//GTCTGAGGCCACCAGCCGGG
TTCAACATTGGGCCTCAGAC//GTCTGAGGCCATTGCAGCTT
TGTTTTAAATGGCCTCAGAC//GTCTGAGGCCAAATGTGACA
ACCGTGAAACGGCCTCAGAC//GTCTGATGCCAAACATTGAT
GATATCATCTGGCCTCAGAC//GTCTGAGGCCTATCAGACCA//TACTATATCAGGCCTCGGAC//GTCTGAGGCCATCTTCGGTT
ACGTTGTGGTGGCCTCAGAC//GTCTGAGGCCTATCAGACCA//TACTATATCAGGCCTCGGAC//GTCTGAGGCCTGGTGACCGA
GCTGGCACCTGGCCTCAGAC//GTCTGAGGCCTATCAGACCA//TACTATATCAGGCCTCAGAC//GTCTGAGGCCACCTGGTTCT
TGGAACAGTTGGCCTCAGAC//GTCTGAGGCCAGTTAATAAT
CTTGACAGTAGGCCTCGGAC//GTCTGAGGCCAGTAAATAAA
CACTACAAGTGGCCTCAGAC//GTCTGATGCCTATCAGACCA//TACTATATCAGGCCTCAGAC//GTCTGAGGCCATGTCTGTTT
CAAAACATGTGGCCTCAGAC//GTCTGAGGCCTATCAGACCA//TACTATATCAGGCCTCAGAC//GTCTGAGGCCATGTGATTAA
TGTTACTACTGGCCTCAGAC//GTCTGAGGCCTACTCAGTGG
GGACGGAGCTGGCCTCAGAC//GTCTGAGGCCAGCTGACGAC
TGCTACATCCGGCCTCAGAC//GTCTGAGGCCATCCAGTAAA
TCGGACATGTGGCCTCAGAC//GTCTGAGGCCTATCATGTCG
GTGATGAGATGGCCTCAGAC//GTCTGAGGCCTATCAGACCA//TACTATATCAGGCCTCAGAC//GTCTGAGGCCATTTGCTAAC
GCATATATCAGGCCTCAGAC//GTCTGAGGCCTATCAGACCA//TACTATATCAGGCCTCCGAC//GTCTGAGGCCACATTCTTGG
ATGTCTATGTGGCCTCAGAC//----------TATCAGACCA//TACTATATCAGGCCTCAGAC//GTCTGATGCCATGTGTGTTT

CCGCATATCAGGCCTCAGAC//GTCTGAGGCCTATCAGACCA//TACTATATCAGGCCTCAGAC//GTTTGAGGCCATACCAAGGC
ACCGATAACTGGCCTCAGAC//GTCTGAGGCCAACTGCTGGC
CAGTCAAGCTGGCCTCAGAC//GTCTGAGGCCAGCTTGGGGC
GTTAGCAGACGGCCTCAGAC//GTCTGAGGCCAGACGACAAA
TTTGGCATGTGGCCTCAGAC//GTCTGATGCCTATCAGACCA//TACTATATCAGGCCTCAGAC//GTCTGATGCCATGTTTGAAA
TTACACATATGGCCTCAGAC//GTCTGATGCCATATCAAGTG
ATATATATCAGGCCTCAGAC//GTCTGAGGCCTATCAGACCC//TACTATATCAGGCCTCAGAC//GTCTGAGGCCGTATGTCCTT
AAAAACAGTTGGCCTCAGAC//GTCTGATGCCAGTTCATCGT
GCCGCCATATGGCCTCAGAC//GTCTGAGGCCTATCAGACCA//TACTATATCAGGCCTCAGAC//GTCTGAGGCCATATGGCACA
TGAACTTCCTGGCCTCAGAC//GTCTGAGGCCTATCAGACCA//TACTATATCAGGCCTCAGAC//GTCTGAGGCCTCCTAGAATA
TAGCCTACATGGCCTCAGAC//GTCTGAGGCCTATCAGACCA//TACTATATCAGGCCTCAGAC//GTCTGAGGCCACATGTATGT
ACCGACTTCTGGCCTCAGAC//GTCTGAGGCCTTCTACCAGG
GCTAATATCAGGCCTCAGAC//GTCTGAGGCCTATCAGACCA//TACTATATCAGGCCTCACCA//GTCTGAGGCCATTAAGGCAA
TGCGTCATGGGGCCTCAGAC//GTCTGAGGCCATGGATGTAT
CTATGGAACTGGCCTCAGAC//GTCTGAGGCCTATCAGACCA//TACTATATCAGGCCTCAGAC//GTCTGAGGCCAACTGCTACA
GATACCATTTGGCCTCAGAC//GTCTGAGGCCATTTATCATA
ACACGCATTAGGCCTCAGAC//GTCTGAGGCCATTACAAGAT
TGACGGTAATGGCCTCAGAA//GTCTGAGGCCTAATGAGGGC
TTCATATATTGGCCTCAGAC//GTCTGAGGCCTATCAGACCA//TACTATATCAGGCCTCAGAC//GTCTGAGGCCTATTGTTCAT
GAGACGTTATGACCTCAGAC//GTCTGAGGCCTTATGGCCCT
AGATATATCAGGCCTCAGAC//GTCTGATGCCTATCAGACCA//TACTATATCAGGCCTCAGAC//GTCTGATGCCAAATGTTGCT
GCCCATATCAGGCCTCAGAC//GTCTGAGGCCATAGGCATTG
GTCGAGAACTGGCCTCAGAC//GTCTGATGCCAACTGTTGAA
ACCACATGCTGGCCTCAGAC//GTCTGACGTCTGCTGCTGGA
TAGCTCTTCTGGCCTCAGAC//GTCTGAGGCCTATCAGACCA//TACTCTATCAGGCCTCAGAC//GTCTGAGGCCTTCTTGTTTA
TAGCTCTTCTGGCCTCAGAC//GTCTTAGGCCTATCATACCA
GACTACATGTGGCCTCAGAC//GTCTGAGGCCATGTATCAAG
AACGAGAGATGGCCTCAGAC//GTCTGAGGCCTAAGCCAAAT
TACTATATCAGGCCTCAGAC//GTCTGATGCCGTATCAAACC
GTGATTAACTGGCCTCAGAC//GTCTGAGGCCAACTGATACT
ACAGTGATATGGCCTCAGAC//GTCTGAGGCCATATGCGACA
CACGTAATATGGCCTCAGAC//GTCTGAGTCCACATGCAGCC
TCGTGTATTTGGCCTCAGAC//GTCTGAGGCCATTTTACAGC
CGGCTTATATGGCCTCAGAC//GTCTGAGGCCTATCAGACCA//TACTATATCAGGCCTCAGAC//GTCTGAGGCCATATCAGGGA
AAATAGACCTGGCCTCAGAC//GTCTGAGGCCTATCAGACCA//TACTATATCAGGCCTCAGAC//GTCTGAGGCTACCTGTGGA
GACCGTTTCTGGCCTCAGAC//GTCTGAGGCCTTCTGCTTTC

KolobokP-7_GiAe
TCGTCGTCTAGGTAGCAGCG//CTCTGCAACCTCTAGTGTGT
CAGTGGTAAAGGTAGCAGAG//CTCTGCAACCCAAAGCGCTC
CTATGCTCCAGGTAGCAGCG//CTCTGCAACCTCCATATGGA
GGAGGCTAATGGTAACAGCG//CTCTGCAACCTAATGATAGC
GAAGCCTTCAGGTAGCAGCG//CTCTGCAACCTTCAGTGGTT
CGCCTCTAAAGGTAGCAGCG//CTCTGCAACCTAAAGCACAT
ACAGGTTTTTGGTAGCAGCG//CTCTGCACCCTTTTATGGGC
TTCGTCCCCAGGTAGCAGTG//CTCTGCAACCCCCAGGCAAC
GGTCTTTTGTGGTAGCAGTG//CTCTGCAACCTTGTGCACCA
CTAAGCTATAGGTAGCAGCG//CTCTGCAACCTATAGTACCG
CACAAGTACAGGTAGCAGCG//CTCTGCAACCTACAGGTTGT
TGTCCCTATTGGTAGCAGAG//CTCTGCAACCTTAAGAACAA//AGTGTCTTCAGGTAGCAGAG//CTCTGCAACCTATTGCTCTA
GGCATGTTATGGTAGCAGCG//TTCTGCAACCTTATCACAGT
CTCCTGTCTTGGTAGCAGAG//CTCTGCAACCTCTTCTCCCA
CCAAGCTATAGGTAGCAGAG//CTCTGCAACCTATAGGCCTA
AGCGGTACCAGGTAGCAACG//CTCTGCAACCACCAGATGAA
AGAAGCTGTTGGTAGCAGAG//CTCTGCAACCTTAATGTTGT
CCCTGTTCCAGGTAGCAGCG//CTCTGCAACCTTAAGAACAA//AGTGTCTTCAGGTAGCAGCG//CTCTGCAACCTCCAAGCTAC
GCTGCTAGAAGGTAGCAGCG//CTCTGCAACCAGAATACTCT
ACTACGTAATGGTAGCAGCG//CTCTGCAACCTAATTAGAAC
ATGATATAAAGGTAGCAGCG//CTCTGCAACCTAAATAGATA
TTTTTGTTTAGGTAGCAGAG//CTCTGCAACCTTAAGAACAC
CCTGCCTTCAGGTAGCAGCG//CTCTGCAACCATCAGCTCCT
CGTCATTTCAGGTAGCAGAG//CTCTGCAACCTACAGTGAGA
CATACATACAGGTAGCAGCG//CTCTGCAACCTACATACACA
ACACTGTTCAGGTAGCAGCG//CTCTGCATCCTTCAATTATG
AGCTGTTGTAGGTAGCAGCG//CTCTGCAACCTGTAGCGTAC
CGGACCAGATGGTAGCAGCG//CTCTGCAACCAGATAAATGT
AAAGGTTAAAGGTAGCAGAG//CTCTGCAACCTAAACAGTCG
TTAATGTGCAGGTAGCAGAG//CTCTGCAACCTTAAGAACAA//AGTGTCTTCAGGTAGCAGAG//CTCTGCAACCTGCAGTGGAT
CCATCCTGCAGGTAGCAGCG//GTCTGCAACCTGCAGAACGC
CATGGCTGAAGGTAGCAGCG//CTCTGCAACCTTAAGAACAA//AGTGTCTTCAGGTAGCAGCG//CTCTGCAACCTTAAGAACAA//AGTGTCTTCAGGTAGCAGCG//CTCTGCAACCTGAAGAGTAG
AGCGACTTCAGGTAGCAGAG//CTCTGCAACCTTAAGAACAA
TATATATTATGGTAGCAGCG//CTCTCCAACCTTATATAGGC
AGACATAGAAGGTAGCAGCG//CTCTGCAACCTTAAGAACAA
ATTATATACAGGTAGCAGCG//CTCTGCAACCTACAGACGCT
AGGCTTTACAGGTAGCAGAG//CTCTGCAACCTACAGACGCT
ACATCGTAACGGTAGCAGCG//CTCTGCAACCGTAAGAACAA//AATGTCTTCAGGTAGCAGCG//TTCTGCAACCTAACCAACAC
GTTCGTTTATGGTAGCAGAG//CTCTGCAACCTTAAGAACAA//AGTGTCTTCAGGTAGCAGAG//CTCTGCAACCTTAAGAACAA//AGTGTCTTCAGGTAGCAGAG//CTCTGCAACCTTAAGAACAA//AGTG
TCTTCAGGTAGCAGAG//CTCTGCAACCTTATCGTCTG
ACATTGTATTGGTAGCAGCA//CTCTGCAACCTATTACTTTA
GTCCTCTTCAGGTAGCAGCG//CTCTGCAACCTTTAGCACTC
TGCTGCTTTTGGTAGCAGAG//CTCTGCAACCTTAAGAACAA//AGTGTCTTCAGGTAGCAGAG//CTCTGCAACCTTTTCCATCC
CGGCTCTATAGGTAGCAGAG//CTCTGCAACCTATATACCTA
AGTATCTTCAGGTAGCAGCA//CTCTGCAACCTTAAGAACAA//AGTGTCTTCAGGTAGCAGCG//CTCTGCAACCTTCTGTGTTG
CCTTGATCTTGGTAGCAGCG//CTCTGCAACCTGATTGTTCT
GTTATATTCAGGTAGCAGCG//CTCTGCAACCTTTATGGGAA
GCCCCCTGAAGGTAGCAGCG//CTCTGCCACCTGAAGCGCGG
GGTGCCTGAAGGTAGCAGTG//CTCTGCAACCTTAAGAACAA
ACTCTCTTTTGGTAGCAGCG//CTCTGCAACCTTTTATCCCA
AGTGTCTTCAGGTAGCAGAG//CTCTGCAAC-TTAAGAACAA//AGTGTCTTCAGGTAGCAGAG//CTCTGCAACCTTCATCGGAG

Figure S5. Junction sequences of solo LTDR excision. The inferred likely TSDs are highlighted in yellow. The internal sequences of *KolobokP* families are omitted.

Case_1, LTDR-INT, KolobokP-7N2_CorFlu

```
>LG08  14733476 -> 14735329
>LG02  47074796 -> 47074865


                                               KolobokP-7N2_CorFlu LTDR-INT
                                      GGCGGCAGTAAAC//ACAAATTTCAGCGATATCCTTAA
                                      |||||||||||||  |||||||||||||||||||||||
GAAAGAGGTCAAATCTATTTTTAGCTATCAGTGTAGTAGGCGGCAGTAAAC//ACAAATTTCAGCGATATCCTTAAATATATGAACCAAGTGAAGGCAATCCAAATCAG
||||||||||||||||||||||||||:|||||||||||                                       ||||||||||||||||||||||||||||||||
GAAAGAGGTCAAATCTATTTTTAGCTGTCAGTGTAGTA---------------------------------------TATACGAACCAAGTGAAGGCAATCCAAATCAG
```

; FRAGMENT 14733476 -> 14735329
LG08
GAAAGAGGTCAAATCTATTTTTAGCTATCAGTGTAGTAGGCGGCAGTAAACACTTATTTTGAATATTTTG
GACTTTCAACCCGACGCCAAACACTTGCCACTCTTGCACTGTCTAACAACTTGAACCTGAAATAAAAAGA
TGATATAACACATACAATTCAGTATTTAAAAACAATAATCATATTGTTAATAAACCATGACTTCAAATTT
TCTTAAGAATTCAATTTTGGGGCTTGGTCGCGAAATTCAATGGCAATTTAGAGTCGGTTTGCACTTTTTT
TAACAGTCATAGCTTTCTTATTGCCAACGACTGATACTTGAAACCTGTAAAATGCTTTTTCTCATTTCCT
AGTGATAACAAGCAGCTAAAACAAATATGTTCATAACCGATAAGATTTTGCAGTATTCCAGCAAATCTGT
ATGCCCCTGAAAAACTTGCCGGTAAAATGGCTTCCGAGCAGACGTTTGCATCACTTCCGTTTCCACTTCC
TCAAAATGATGTAAAGCTAGCAAGTTCAAGATATTGTGCATTTTCTTATTTCTCAAACTTCATATTTGGC
AGCAAGAAATCAAATTTTGATAAAACAATTACTAGTTAGTCAACAAGCTACCTAAATCTGCAACTTACCT
CTTATCTTATATTTTGACCGGGGCCAATTCGCATCTATGGCTAAAAAAGTCTTTACTAGTCCCTTAAGAG
CAGAGAGATGTTTTCCTTTAAGCCATGAACTCATCTCTGTTCAAATTTAGAACCATTTCTGAGCAAATTA
AGACCAAAACCAAGCCAGTATCATCATCCTTGCTGGTTTTAAGAGCAAATGACTACCACCACTCACTTTC
TAAAAATGGCCAATTTTGCCTCCATTTACAGTCAAGGAAATTTATACAGTGCTTCTAAGAAATGCTATTA
AACAGACAATGGAACCAGAATACCTGACATTCATTTACCACATAAAAGAAGACAAGACAGAAGATGATCA
ATGAAAAAATGCTGTTACAAGCACTCATTTTTGTAATAAAATAGGTCAGGCAAAGCAAATTGTATCAGTC
AAACAGGCAAAACTGGCCATTTCTCACTTTCATCACTCACCTACCATGTTGCCATCATGTTTCACTTGCT
CATATGGACCATGTGCATACCTTTCTGCTAATGAACTGCCACTTTTCAACAAATACACACATCACACTTT
ATGTTTATGCTTGCTCTAATGTCACCAAAATAATGATTGAATCAAAAAATGGCCAAGAACAAATAGCAAA
TGATGAAATTCTTACCTCTGGCTGCAGAAAGGAATAAACAGCTAAGCTAACATAACAGACTACAGCTGAA
GGCCAAATCCTGCCTATCTGGGCTAAAATACCTACTAAACTGATAACAGTGTTCATGGCAAACCCTACAC
AGTTCTAGAACTTGATTTTCAATGGTCGACCAGAATTTACATCTCGGTTCGCAAAGCTCGGACTGTGGAA
ATCTGCTTGCCGAGCTTAATTTTTCTTAATTTATTTTTATCGGCTTGATTTTCTGGCAGATCTTATGCCA
AATGCTCTTTCGTAATCATCTGAGAGCTTTTCATGATGTATTTGATGGCCTTTCAATAAAAATCTGCATT
TCTGACATTTCACGACTCGTTTTTATGGCTCGATGGAGGATTTTTTTTCTAATAAACTGTAATTACCTCC
CTTTATTTGTAAACCTCAGCTTGTAAGCAAGTATGTAACAGCATAAAATGATGTCATTTTGCAAATATAG
CAGTCCAATACAGCATTCTAGACCAAAATTATGACATAATGAACACTTACAAATTTCAGCGATATCCTTA
AATATATGAACCAAGTGAAGGCAATCCAAATCAG

; FRAGMENT 47074796 -> 47074865
LG02
GAAAGAGGTCAAATCTATTTTTAGCTGTCAGTGTAGTATATACGAACCAAGTGAAGGCAATCCAAATCAG

Case_2, INT-LTDR, KolobokP-7N2_CorFlu

```
>LG07  61998071 -> 61999928
>LG07  62512633 -> 62512564


                                          KolobokP-7N2_CorFlu INT-LTDR
                                   TTAAGAGCAGAGAGATGTTT//AAAAGTCTTTACTAGTCCC
                                       ||||||||||||||||  |||||||||||||||||||
CTTTGCAGAGCAGAACACACAATTTTATCATTGATATAAGAGCAGAGAGATGTTT//AAAAGTCTTTACTAGTCCCTAAGCTTTAAATAACCCGTCTTACATCTAAACTC
|||||||||||||||||||||||||||||||||:||                                        ||||||||||||||||||||||||||||||||||
CTTTGCAGAGCAGAACACACAATTTTATCATTGTTA----------------------------------------TAAGCTTTAAATAACCCGTCTAACATCTAAACTC
```

; 61998071 -> 61999928
LG07
CTTTGCAGAGCAGAACACACAATTTTATCATTGATATAAGAGCAGAGAGATGTTTTCCTTTAAGCCATGA
ACTCATCTCTGTTCAAATTTAGAACCATTTCTGAGCAAATGAAGACCAAAACCAAGCCAGTATCATCATC
CTTGCTGGTTTTAAGAGCAAATGACTACCACCACTCACTTTCTAAAAATGGCCAATTTTGCCTCCATTTA
CAGTCAAGGAAATTTATACAGTGCTTCTAAGAAATGCTATAAAACAGACAATGGAACCAGAATACATGAC
ATTCATTTACCACATAAAAGCTCACAGAAGATGATCAATGAAAAATATGCTGTTACAAGCACTCATTTTT
GTAATAAAATAGGTCAGACAAAGCAAATTGTATCAGTCAAACAGGCAAAACTGGCCATTTCTCACTTTCA
TCACTCACCTACCATGTTGACATCATGTTTCACTTGCTCATATGGACCATGTGCATACCTTTCTGCTAAT
GAACTGCCACTTTTCAACAAATACACACATCACACTTTATGTTTATGCTTGCTCTAATGTCACCAAAATA
ATGATTTAATCAAAAAATGGCCAAGAACAAATAGCAAATGATGAAATTCTTACCTCTGGCTGCAGAAAGG
AATAAACAGCTAAGATAACATAACAGACTACAGCTGAAGGCCAAATCCTGCCTATCTGGGCTAAAATACC
TACTAAACTGATAACAGTGTTCATGGCAAAACCCTACACAGTTCTAGAACTTGATTTTCATTGGTCGACC
AGAATTTACATCTCGGTTCGCAAAGCTCGGACTGTGGAAATCTGCTTGCCGAGCTTAATTTTTCTTAATT
TATTTTTATCGGCTTGATTTTCTGGCAGATCTTATGCCAAATGCTCTTTCGTAATCATCTGAGAGCTTTT
CATGATGTATTTGATGGCCTTTCTATAAAAATCTGCATTTCTGACATTTCACGACTCGTTTTTATGGCTC
GATGGAGGATTTTTTTCTAAAAAACTGTAATTACCTCCCTTTATTTGTAAACCTCAGCTTGTAAGCAAGT
ATGTAACAGCATAAAATGATGTCATTTTGCAAATATAGCAGTCCAATACAGCATTCTAGACCAAAATTAT
GACATAATGAACACTTACAAATTTCAGCGATATCCTTAAGGCGGCAGTAAACACTTATTTTGAATATTTT

GGACTTTCAACCCGACGCCAAACACTTGCCACTCTGGCACTGTCTAACAACTTGAACCTGAAATAAAAAG
ATGCTTTCTTGATATAACACATACAATTCAGTATTTAAAAACAATAATCATATTGTTAATAAACCATGAC
TTCATATTTTCTTAAGAATTCAATTTTGGGGCTGGGTCGCGAAATTCAATGACAATTTAGAGTCAGTTTG
CACTTTTTTTTAACAGTCATAGCTTTCTTATTGCCAACGACTGATACTTGAAACCTGTAAAATGCTTTTT
CTCATTACCTAGTGATAACAAGCAGCTAAAACAAATATGTTCATAACCGATAAGATTTTGCAGTATTCCA
GCAAATCTGTATGCCCCTGAAAAACTTACCGGTAAAATGGCTTCCGAGCAGACATTTGCATCACTTCCGT
TTCCACTTCCTCAAAATGATGTAAAGCTAGCAAGTTCAAGATATTGTGCATTTTCTTATTTCTCAAACTT
CATATTTGGCAGCAAGAAATCAAATTTTGATAAAACAATTACTAGTTAGTCAAACAAGCTACTTAAATCT
GCAACTTACCTCTTATCTTATATTTTGACCGGGGCCAATTCGCATCTATGACTAAAAAAGTCTTTACTAG
TCCCTAAGCTTTAAATAACCCGTCTTACATCTAAACTC

; 62512633 -> 62512564
LG07
CTTTGCAGAGCAGAACACACAATTTTATCATTGTTATAAGCTTTAAATAACCCGTCTAACATCTAAACTC

Case_3, INT-LTDR, KolobokP-4N1_CorFlu

>Contig4291   14634 -> 16855
>LG01_1   76171507 -> 76171573

```
                                      KolobokP-4N1_CorFlu INT-LTDR
                                  TATAAGAGAACTT//AGTTTAATACAACC
                                  |||||||||||||  ||||||||||||||
TGTAGAAGGTACCTTCTGCGTTGTTAACTTGTATTATAAGAGAACTT//AGTTTAATACAACCTAATGACTTGTACTGTATTTAAGACACGCATACC
|||||||||||||||||||||:|||||||||||                              ||||||||||||||||||||||||||||||||||
TGTAGAAGGTACCTTTTGCGTTGTTAACTTGTA------------------------------TAATGACTTGTACTGTATTTAAGACACGCATACC
```

; FRAGMENT 14634 -> 16855
Contig4291
TGTAGAAGGTACCTTCTGCGTTGTTAACTTGTATTATAAGAGAACTTAATTTTTTTTAAATATCTCTATA
TTTGTTCTACACATAGCTGCACCTTTTGTAAGAAATGTTTTAACAATTTTAAAGCACTCAGACTACCCAT
TCCATGAAAAACACCATATCTATACAGCTAGTGTCCATGGCCTCGGCCAAGCGATTTTAGGCTCATTTTA
TATATCGCAACATTTTGGCTGCCATTTTAAAAACGCGTTACTTTTTCATGTCATATTGACTTTTTTGATT
GCATAAGAAGTTACTTTATACGATTACACTACAATAGTCTGTTTATTTTACCAAAGGCAGCCAGGCAAGT
GCAATTACTTCAATTTTTTTCTAAAATTGCTGACTTATTTTTTTCAATTTTCACATTCACTTCAAACTTT
TTAACCCAAACATCACAAAATACTGACTAAAACTCACTTATTTTATCAAAAATAATATTATTACAACACA
CTTTGATCAGTAGATTTAATAATAAGCTGATTTGAGTGGCTAACCCAGTCAATTAAAGGCCTACAGTGCT
GTTAACTGCCATTGACAGCACCTTAACACCTATGTGTGATTGCAGCACCTTAACATAACAAGTTGCCTAT
AAAAAAGTCAGCTAAATGTTCCCAAGGTGTGTGTGTATTCCCTAAGCCACACTAGTGTATGTTTAACTCA
CAAATATGGTGTCTTATAGGAAACGAGAAAAAACATAGGCGCTAAAACTGGGTTTGAAAAAGGCCAGCCG
AGTGCTATGAAAGGGAAGTCATTTGAAAATGAGGAAACTACCTCTGAATCCAAATTTACATGCTTGCCCA
AACAAACTTACTATGATCAAATAGTAGAGGATGGAGGAATTCTTACTTTTGAAGATGTTGATGGTTCAAA
TACCAATGTGAAACCACTCTGCAGTCTGTCAATCCAAAACTCTGGTTGAGGAGTGCACACACCCTAAAGT
TGACTCTGGTTACTACCACCCTGACTTGCTTGTTAACATGATTTACAGGCTTTTGGATGTCTGTCTAATG
TTCAACTCTGAAATGAGGAAGCATATGTCTTTAAAGCCTCCGTTGCAATGGATATATCATGATTGATGCT
GCCAACTCAAAGCATATTCTGACTTGTCGGACTGTTAGTTTTCACCGACAGAATGCTGACAAATGAATTG
TTGTATATACTGTACATAGTGTAAATATGTTATAGTTGTTCTTATCCTATTTTAAACATCTAGTTATTTA
AATAAAAATTAAATACATACCAAAATGTACACTTTGGCAACATCTCCCCATTTATAATATGCCCCATACA
ACCAGCAGGTCTGCCTTTGATTGTTCACCACCTGAGGCTCTACAGTCAAATAACTGAAATTGCAACATAA
AGAATAATGACACAGATGTGACACATGCATCCATCTGGTAGTCTTTCCATGTTCTGGTGTATTTGATAGA
CTAGCCTTTGAACATAATAGTATCTGCTAACTGAGTCCTCTGGAAATTTAACTCTCATTCTGCCATACCT
CATGCCCTTTTGGTCCCTAAGTGTCCCACCCTATCTAATACACACCCTGTTGCCAAATACTTCACATTTA
GATTCTATTCTTCTTTGCAATGCTGATAAAAAAGTGATTTAAAGTCATTCTGACTGCAGACGACCAAAGT
AGACAGGTAACCGGATGTTGTCATTTTTTCTTATAGGTTGAACCAAACTAAAACAATTTCCATAGACGCT
AATGTTAAATAAATGATTTTCAATTGCATCTTGTCATTTTATTTATGTCTTAATCCCAGCAAAACCTGGT
ATCAGTTTGAAGGTCTATCTATCCTGTTTTGTCTAAAATAAAGCAAATGGCATGTTAACTTACTTGTAAG
AAAATCCCAGTGCACCGAATGACGTCACCGTCAATTTGTCTAGTATCCATACGCGGGATTTCCTCAACTT
ACCTACTTTCACTTTCCAATTAACTGATCATCCATTTTCTGTCTGATTTTCTTATTTTATGTCTCAAATG
ATTCCTAAACGATTGCTTGTATCATAATGAACACAAATAACCACATTCTGAAGTATTTTATGTCTTTACA
TACAAAAGGAAAAATGAACTATTTAGCAATTCGCACTACATTCGAATGGACCACGGAGGAATACTGGGTG
ATTTAGTTTAATACAACCTAATGACTTGTACTGTATTTAAGACACGCATACC

; FRAGMENT 76171507 -> 76171573
LG01
TGTAGAAGGTACCTTTTGCGTTGTTAACTTGTATAATGACTTGTACTGTATTTAAGACACGCATACC

Case_4, INT-LTDR, KolobokP-4N1_CorFlu

>LG11   25854663 -> 25856886
>LG01   101591680 -> 101591620

```
                                    KolobokP-4N1_CorFlu INT-LTDR
                               TATAAGAGAACTT//AGTTTAATACAACC
                                   |||||||||  ||||||||||||||
TTAATTAGTTTTGTGCGCTAGAATTGGCCCTTTTAAGAGAACTT//AGTTTAATACAACCTTTAATCAACGGGGGATAACTGGTAAAAT
|||||||||||||||||||||||||||||||                             |||||||||||||||||||||||||||||
TTAATTAGTTTTGTGCGCTAGAATTGGCCCT-----------------------------TTTAATCAACGGGGGATAACTGGTAAAAT
```

; FRAGMENT 25854663 -> 25856886
LG11
TTAATTAGTTTTGTGCGCTAGAATTGGCCCTTTTAAGAGAACTTAATTTTTTTTAAATATCTCTATATTT
GTTCTACACATAGCTGCACCTTTTGTAAGAAATGTTTTAACAATTTTAAAGCACTCAGACTACCCATTCC
ATGAAAAACACCATATCTATACAGCTAGTGTCCATGGCCTCGGCCAAGCGATTTTAGCCGCATTTTATAT
ATCGCAACATTTTGGCTGCCATTTTAAAAACGCGTTACTTTTTCATGTCATATTGACTTTTTTGATTGCA
TAAGAAGTTACTTTATATGATTACACTACAATAGTCTGCTTATTTTACCAAAGGCAGCCAGGCAAGTGCA
ATTAATTCAATTGTTTTCTAAAATTGCTGACTTATTTTTTCAATTTTCACATTCACTTCAAACTTTTTAA
CCCAAACATCACAAAATACTGACTAAAACTCACTTATTTTATCAAAAATAATATTATTACAACACACTTT
GATCAGTAGCTTTAATAATAAGCTGATTTGAGTGGCTAACCCAGTCAATTAAAGGCCTACAGTGCTGTTA
ACTGCCATTGACAGCACCTTAACACCTATGTGTGATTGCAGCACCTTAACATAACAAGTTGCCTACAAAA
AAGTCAGCTAAATGTTCCCAAGGTGTGTGTGTATTCCCTAAGCCACACTAGTGTATGTTTAACTCACAAA
TATGGTGTCTTATAGGAAACGAGAAAAAACATAGGCGCTAAAACAGGGTTCGAAAAAGGCCAGCCGAGTG
CTATGAAAGGGAAGTCATTTGAAAATGAGGAAACTACCTCTGAATCCAAATTTACATGCTTGCCCAAACA
AACTTACTATGATCAAATAGTAGAGGATGGAGGAATTCTTACTTTTGAAGATGTTGATGGTTCAAATACC
AATGTGAAACCACTCTGCAGTCTGTCAATCCAAAACTCTGGTTGAGGAGTGCACACACCCTAAAGTTGAC
TCTGGTTACTACCACCCTGACTTGCTTGTTAACATGATTTACAGGCTTTTGGATGTCTGTCTAATATTCA
ACTCTGAAATGAGGAAGCACATGTCTTTAAAGCCTCCGTTGCAATGGATATATCATGATTGATGCTGCCA
ACTCAAAGCATATTCTGACTTGTCGGACTGTTAGTTTTCACCGACAGAATGCTGACAAATGAATTGTTGT
ATATACTGTACATACCGTAAATATGTTATAGTTGTTTTTATCATATTTTAAACATCTAGTTATTTAAATA
AAAATTAAATACATACCAAAATATACACTTTGGCAACATCTCCCCATTTATGACATGCCCCATACAACCA
GCTCTATCCCTGCAGGTCTGCCGTTGATTGTTCACCACCTGAGGCTCTAAAGTCAAATAACTGAAATTGC
AACATAAAGAATAATGACACAGATGTGACACATGCATCCATCTGGTAGTCTTTCCATGTTCTGGTGTATT
TGATAGACTAGCCTTTGAACATAATAGTATCTGCTAACTGAGTCCTCTGGAAATTTAACCCTCATTCTGC
CATACCTCATGCCCTTTTGGTCCCTAAGTGTCCCACCCCATCTAATACACACCCTGTTGCCAAATACTTC
ACATTTAGATTCTATTCTTCTTTGCAATGCTGATAAAAAGTGATTTAAAGACATTCTGACTGCAGACGAC
CAAAATAGACAGGTAACCGGATGTTGTCATTTTTTCTTATAGGTTGAACCAAACTAAAACAATTTCCATA
GACGTTAATGTTAAATAAATGATTTTCAATTGCATCTTGTCATTTTATTTATGTCTTAATCCCAGCAAAA
CCTGGTATCAGTTTGAAGTTCTATCTATCCTCTTTTGTCTAAAATAAAGCAAATGGCATGTTAACTTACT
TGTAAGAAAATCCCAGTGCACCGAATGACGTCACCCTCAATTTGTCTAGTATCCATACGCGGGATTTCCT
CAACTTACCTACTTTCACTTTCCAATTAACTGATCATCCATTTTCTGTCTGATTTTCTTATTTTATGTCT
CAAATGATTCCTAAATGATTGCTTGTATCATAATGAACACAAATAACCACATTCTGAAGTATTTTATGTC
TTTACATACAGAAGGAAAAATGAACTATTTAGCAATTCGCACTACATTCGAATGGACCACGGAGGAATAC
TGGGTGATTTAGTTTAATACAACCTTTAATCAACGGGGGATAACTGGTAAAAAT

; FRAGMENT 101591680 -> 101591620
LG01
TTAATTAGTTTTGTGCGCTAGAATTGGCCCTTTTAATCAACGGGGGATAACTGGTAAAAAT

Case_5, INT-LTDR, KolobokP-4N1_CorFlu

>Contig4074 75588 -> 73366
>Contig4074 54690 -> 54637

```
                                    KolobokP-4N1_CorFlu INT-LTDR
                              TATAAGAGAACTT//AGTTTAATACAACC
                               |||||||||||||  ||||||||||||||
ATCAGGTTCGATTTAAAGAGGCCAAACTTTTATAAGAAAACTT//AGTTTAATACAACCTTTTAGCTAGATGAAACTTCCATCCCG
|||||||||||||||||||||||||||                                |||||||||||||||||||||||||||
ATCAGGTTCGTTTTAAAGAGGCCAAAC--------------------------------TTTTAGTTAGATGAAACTTCCATCCCG
```

; FRAGMENT 75588 -> 73366
Contig4074_1
ATCAGGTTCGATTTAAAGAGGCCAAACTTTTATAAGAAAACTTAATTTTTTTTAAATATCTCTATATTTG
TTCTACACATAGCTGCACCTTTTGTAAGAAATGTTTTAACAATTTTAAAGCAATCAGACTACCCATTCCA
TGAAAAACACCATATCTATACAGCCAGTGTCCATGGCCTCGGCCAAGCGATTTTAGGCTCATTTTATATA
TCGCAACATTTTGGCTGCCATTTTAATAACGCGTTACTTTTTCATGTCATATTAACTTTTTTGATTGCAT
AAGAAGTTACTTTATATGATTACACTACAATAGTCTGATTATTTTACCAAAGGCAGCCAGGCAAGTGCAA
TTAATTCAATTTTTTTTCTAAAATTGCTGACTTATTTTTTCAATTTTCACATTCACTTCAAACTTTTTAA
CCCAAACATCACAAAATACTGACTAAAACTCACTTATTTTATCAAAAATAATATTATTACAACACACTTT
GATCAGTAGCTTTAATAATAAGCTGATTTGAGTGGCTAACCCAGTCAATTAAAGGCCTACAGTGCTGTTA
ACTGCCATTGACAGCACCTTAACACCTATGTGTGATTGCAGCACCTTAACATAACAAGTTGCCTATAAAA
AAAGTCAGCTAAATGTTCCCAAGGTGTGTGTGTATTCCCTAAGCCACACTAGTGTATGTTTAACTCACAG
ATATGGTGTCTTATAGGAAACGAGAAAAAACATAGGCGCTAAAACTGGGTTCGAAAAAGGCCAGCCGAGT
GCTATGAAAGGGAAGTCATTTGAAAATGAGGAAACTACCTCTGAATCCAAATTTACATGCTTGCCCAAAC
AAACTTACTATGATCAAATAGTAGAGGAAGGAGGAATTCTTACTTTTGAAGATGTTGATGGTTCAAATAC
CAATGTGAAACCACTCTGCAGTCTGTCAATCCAAAACTCTGGTTGAGGAGTGCACACACCCTAAAGTTGA
CTCTGGTTACTACCACCCTGACTTGCTTGTTAACATGATTTACAGGCTTTTGGATGTCTGTCTAATGTTC
AACTCTGAAATGAGGAAGCACATGTCTTTAAAGCCTCAATGCAATGGATATATCATGATTGATGCTGCCA
ACTCAAAGCATATTCCGACTTGACTTGTCGGACTGTTAGTTTTTTTCACCGACAGAATGCTGACAAATGA
ATTGTTGTATATAGTGTACATAGTGTAAATATGTTACAGTTGTTTTTATCATATTTTAAACATCTAGTTA
TTTAAATAAAAATTAAATACATACCAAAATATACACTTTGGCAACATCTCCCCATTTATGACATGCCCCA
TACAACCAGCTCTATCCCTGCAGGTCTGCTTTTGATTGTTCACCACCTGAGGCTCTCAAGTCAAATGACT
GAAATTGCAACATAAAGAATAATGACACAGATGTGACACATGCATGCCATCTGGTAGTCTTTCCATGTTC
TGGTGTATTTGACAGACTAGCCTGTGAACATGGCTAATAGTATCTGCTAACTGGAAATTTAACTCATTCT
GCCATACCTCATGCCCTTTTGGTCCCTAAGTGTCCCACCCCATCTAATACACACCCTGTTGCCAAATACT
TCACATTTAGATTCTATTCTTCTTTGCAATGCTGATAAAAAGTGATTTAAAGTCATTCTTACTGCAGACG
ACCAAAGTAGACAGGTACCCGGATGTTGTCATTTTTTCTTATAGGTTGAACCAAACTAAAACAATTTCCA
TAGACCCTAATGTTAAATAAATGATTTTCAATTGCATCTTGTCATTTTATTTATGTCTTAATCCCAGCAA
AACCTGGTATCAGTTTGAAGGTCTATCTATCCTCTTTTGTCTAAAATAAAGCAAATGGCATGTTAACTTA
CTTGTAAGAAAATCCCAGTGCACCGAATGACGTCACCCTCAATTTGTCTAGTATTCATACGCGGGATTTC
CTCAACTTACCTACTTTCACTTTCCAATTAACTGATCATCCATTTTCTGTCTGATTTTCTTATTTTATGT
CTCAAATGATTCCTAAATGATTGCTTGTATCATAATGAACACAAATAACCACATTCTGAAGTATTTCATG
TCTTTACATACAGAAGGAAAAATGAACTATTTAGCAATTCGCACTACATTCGAATGGACCACGGAGGAAT
ACTGGGTGATTTAGTTTAATACAACCTTTTAGCTAGATGAAACTTCCATCCCG

```
; FRAGMENT 54690 -> 54637
Contig4074
ATCAGGTTCGTTTTAAAGAGGCCAAACTTTTAGTTAGATGAAACTTCCATCCCG
```

Case_6, Solo INT, KolobokP-4N1_CorFlu

```
>LG03   76820672 -> 76818934
>LG03   77366061 -> 77366121
```

```
                                     KolobokP-4N1_CorFlu INT
                          TATAAGAGAACTTAATT//TCATTTTTTCTTATA
                               ||||||||||||  |||||||||||||||
ATCACGAATTCTTCTCATAATTTCTGTTTTAGAGAACTTAATT//TCATTTTTTCTTATAGTGAGAGTATTTATGTGTGTAAATATAAGG
|||||||||||||||||||||||||||||||                             ||||||||||||||||:|||||||||||||
ATCACGAATTCTTCTCATAATTTCTGTTTTA-----------------------------GTGAGAGTATTTATGTATGTAAATATAAGG
```

```
; FRAGMENT 76820672 -> 76818934
LG03
ATCACGAATTCTTCTCATAATTTCTGTTTTAGAGAACTTAATTTTTTTAAGTATATTTGTTCTACACATA
GCTGCACCTTTTGTAAGAAATGTTTTAACAAATTTAAAGCACTCAGACTACCCATTCCATGAAAAACACC
ATATGTATACAGCCAGTGTCCATGGCCTCGGCCAAGCGATTTTAGGCTCATTTTATATATCGCAATATTT
TGGCTGCCATTTTAAAAACGCGTTACTTGTTCATGTCATATTGGCTTTTTTGATTGCATAAGAAGTTACT
TTATATGATTACACTACAATAGTCTGATTATTTTACCAAAGGCAGCCACGCAAGTGCAATTAATTCAATT
TTTTTCTAAAATTGCTGACTTATTTTTTCAATTTTCACATTCACTTCAAACTTTTTAACCCTAACATCAC
AAAATACTGACTAAAACTCACTTATTTTATCAAAAATAATATTATTACAACATACTTTGATCAGTAGCTT
TAATAATAAGCTGATTTGAGTGGCTAACCCAGTCAATTAAAGGCCTACAGTGCTGTTAAGTGCCATTGAC
AGCACCTTAACAGCTATGTGTGATTGCAGCACCTTAACATAACAAGTTGCCTATAAAAAAGTCAGCTAAA
TGTTCCCAAGGTGTGTGTGTATTCCCTAAGCCACACTAGTGTATGTTTAACTCACAAATATGGTGTCTTA
TAGGCAAAGAGAAAAAACATAGGCGCTAAAACTGGGTTCGAAAAAGGCCAGCCGAGTGCTATGAAAGGGA
AGTCATTTGAAAATGAGGAAACTACCTCTGAATCCAAATTTACATGCTTGCCCAAACAAACTTACTATGA
GCAAATAGTAGAGGAAGGAGGAATTCTTACTTTTGAAGATGTTGATGGTTCAAATACCAATGTGAAACCA
CTCTGCAGTCTGTCAATCCAAAACTCTGGTTGAGGATTGCACACACCCTAACGTTGACTCTGGTTACTAC
CACCCTGACTTGCTTGTTAACATGATTTACAGGCTTTTGGATGTCTGTCTAATGTTCAACTCTGAAATGA
GGAAGCACATGTCTTTAAAGCCTCAATGCAATGGATATATCATGATTGATGCAGCCAACTCAAAGCATAT
TCTGACTTGTCGGACTGTTAGTTTTCACCGACAGAATGCTGACAAATGAATTGTTGTGTATACTGTACAT
AGTGTAAATATGTTATAGTTGTTTTTATCATATTTTAAACATCTTGTTATTTAAATAAAAATTAAATACA
TACCAAAATATACACTTTGGCAACATCTCCCCATTTATGACATGTCCCACACAACCAGCTCTATCCCTGC
AGGTCTGCCTTTGATTGTTCACCACCTGAGGCTCTAAAGTCAAATAACTGAAATTGCAACATAAAGAATA
ATGACACAGATGTGACACATGCATGCGATCTGGTAGTCTTTCTATGTTCTGGTGTATTTGATAGACTAGC
CTGTAAACATAATAGTATCTGCTAACTGAGTCCTCTGGAAATTTAACTCTCATTCTGCCATACCTCATGC
CCTTTTGGTCCCTAAGTGTCCCACCTCATCTAATACACACCCTGTTGCCAAATACTTCACATTTAGATTC
TATTCTTCTTTGCAATGCTGATAAAAAGTGATTTAAAGTCATTCTGACTTCAGACGACCAAAGTAGACAG
GTACCCGGATGTTGTCATTTTTTCTTATAGTGAGAGTATTTATGTGTGTAAATATAAGG
```

```
; FRAGMENT 77366061 -> 77366121
LG03
ATCACGAATTCTTCTCATAATTTCTGTTTTAGTGAGAGTATTTATGTATGTAAATATAAGG
```

Figure S6. Protein alignment of the cytoplasmic ballast domains of P2X7 purinoceptors, *Nanor* from zebrafish, and KolX proteins of *Kolobok* families.

```
                     1
P2RX7_Human          PVWCQCGSCL PSQ--LPESH RCLEELCCR- ---------- --KKPGACIT TSELFRKLVL
P2RX7_Opossum        LKWCCCGHCR PSQ--LPEGT RCLEELCCR- ---------- --RKGGPCIT TSALFEELVL
P2RX7_Platypus       PHWCHCGNCS QSQ--LPKGN RCLEELCCR- ---------- --EKKGPCIT ISALFQELVL
P2RX7_Chicken        PKWCCCGRCQ VAQ--KHH-- ---EQLCCR- ---------- --KKEGQCIT TTYWFAQLVL
P2RX7_Fugu           PAWCKCACCV LTS--VPH-- ---EELCCR- ---------- --RSDGACIT SSPLFELLVL
P2XR7_Polypterus     PSWCHCGHCS SAP--TNH-- ---EQLCCR- ---------- --RSNGQCIT TSELFSRLVL
Kolobok-1_NV2p       PDWCKCGNCR EMP--QQI-- ---ENVCCG- ---------- -KR---NCES TKARFGKLCL
Kolobok-1_SKow_2p    PPWCKCGQCI NMP--TQI-- ---ENKCCV- ---------- -VRQGQECLT GSWLFQHLVL
Kolobok-1_AP_1p      KDWCLCNKCI RFT--TDQ-- ---ECICCFE LEKL-HK-LL PQSNKKTCIT EISSFSKIIL
Kolobok-1_BF2p       FLWCRCTNCR PMT--TVR-- ---ECVCCHD LTEAETK-GV GQWDGIHCLR DHPDFSAVVL
Kolobok-1_CTe_2p     NTWCRCGMCE SETLGDEK-- ---EAICCLE VPNCVKMIAA EASADKQCIT LHPDFDVLCL
Kolobok-1_XT2p       TDWCQCSNCS PMP--TVI-- ---ECVCCHE EPLI----KA LIPDDASCII EWHRFKSDII
Kolobok-1_AQu_2p     TTWCQCGHCV PMP--TCM-- ---ECVCCCE IQQVVAK-KN HLQSPVVCIT LHPGFHNVCL
Nanor_Zebrafish      SQRCSCGNCR LTP--E---- ---ENVCCRD IPQVKHK--- CGQVNVCCIT NHPGFELVTL

                    61
P2RX7_Human          SRHVL----- QFLLLYQEPL LALD-VDST- --NSRLRHCA YRCYATWR-- FGSQDMADFA
P2RX7_Opossum        SRATL----- RFILLYQEPL LEMD-AATL- --NNRLRRCA YERYIDWR-- FGSEDMAGFA
P2RX7_Platypus       SRPTL----- QFMLFYRDPL MTLD-SDAL- --TRELRHCA YKRYIDWR-- FGSEDMVDFA
P2RX7_Chicken        SRDTL----- NKALLYEDPF LDLT-GHSS- --NSQLRRIA YKQYIHWR-- FGSFELEDRA
P2RX7_Fugu           RRSTL----- EAALLYRDPL DPPT-GPGQ- --TTTLRHCA YRQYIFWR-- FGEQADGSHP
P2XR7_Polypterus     DRRTL----- ELTLLYENPL LDLS-NENI- --ICKLRHCA YRQYVEWR-- FGSISPGGIA
Kolobok-1_NV2p       DVEVL----- SLGIRSSADI RNDW-HDSS- --ARAFRKAG YRNYILDK-- HGYLGKGRRR
Kolobok-1_SKow_2p    DGDVL----- EVAMRVVADV YAEN-PLRD- --NACFRHYA YRQFIYWQ-- YGRLGKGNRR
Kolobok-1_AP_1p      DEEVLNITRQ QIIVKSKNKS KKKT-LCSSQ PTNKMWRYIC YKQFTHWINS WNSIGKGNRI
Kolobok-1_BF2p       NKAVL----- DAALNFRVDI KLEP-LRDE- YPPRTYRLQA YRQCTAWL-- HQRLGRKIRR
Kolobok-1_CTe_2p     HTTVL----- RNVLVSLNHT RMDD-WNISN LDNRSYRWAA YRQFTWWV-- YAVLGARIRR
Kolobok-1_XT2p       DPERA----- DCALKLTNSK KKKK-PNTAA AYMRAIRKAA YRCFTVWV-- YGYLGTGVRK
Kolobok-1_AQu_2p     DMWVL----- QAASYVYR-- ---------- --QHQMRHTA YRQLVSWC-- WQWLGRNNRV
Nanor_Zebrafish      NPDVL----- QVSYCRYQDV YGKT-LPDL- --NSRNRHLA CLNFIFLC-- WSDVGQQTRA

                   121
P2RX7_Human          ILPSCCRWRI RKEFPKSE-G QYSGFK
P2RX7_Opossum        ILPSCCRWMI RDHFPKQD-G KYTGFK
P2RX7_Platypus       ILPSCCRWRI RKEFPKPG-G QYSGYK
P2RX7_Chicken        IIPSCCRRLI RSTYPKEN-G NYTGFN
P2RX7_Fugu           VIPSCCVWRI REEYPSLD-R RYSGFR
P2XR7_Polypterus     VVPRCCVNKV RHKFPNAS-G HYKGLC
Kolobok-1_NV2p       VAPSCIVWQI RHHYPTRT-G IYMGFR
Kolobok-1_SKow_2p    VVPSCCVWAV RRRFPSPN-N VYVGFK
Kolobok-1_AP_1p      VIPSCVVNKI RQKYPEQD-G CYVGYK
Kolobok-1_BF2p       VLPSCAVWAI REAYPEPAGG NYRGFL
Kolobok-1_CTe_2p     VVPACAVNAI RKAFPEVS-G DYTGYQ
Kolobok-1_XT2p       VIPACVVTAV REAFPDPK-G KYVGFL
Kolobok-1_AQu_2p     VLPACAVAKI RETFPS-N-G NYVGFE
Nanor_Zebrafish      VIPSCVAGRM RQKLPEGN-E NYNGLF
```
