# Supplementary material for: A unique eukaryotic lineage of composite-like DNA transposons encoding a DDD/E transposase and a His-Me finger homing endonuclease
Source: Mob DNA. 2022 Oct 22;13:24. doi: 10.1186/s13100-022-00281-3 (PMC9587614; doi:10.1186/s13100-022-00281-3)
Supplement: Supplementary file 3 — Additional file 3: Data S1. Consensus sequences of KolobokP families characterized in this study. The LTDR and internal portion (I) are divided (fasta format). Data S3. Multiple alignment of KolobokP DDD/E transposases (fasta format). Data S4. Multiple alignment of KolobokP HNH nucleases and related homing endonucleases (fasta format). Data S5. Multiple alignment of Kolobok DDD/E transposases (fasta format). Data S6. KolX protein sequences encoded by piggyBac DNA transposons (fasta format). [file 13100_2022_281_MOESM3_ESM.pdf]

Supplemental Data S1. Consensus sequences of KolobokP families characterized in this study.
The LTDR and internal portion (I) are divided. (fasta format)

>KolobokP-1_CGi-LTDR FRAGMENT 1 -> 651
ggtagcaggggacagtttttttgatgcatgaaattgtggggtgggggtacccccatttagcactatatt
gcgggaaatatataatttgcacctggatcaaaatgacttgtaactacctgttagacttaaggaaggacat
taaggttaaaaatttacccaaaatgacacccatattttttctggtcaaataaaatgtctaattgacccc
cctgtgtaatgtcaaaattttacccattttggcccatttagaccccaccctaccgtattccctgcagaga
gatgacccccgggacagggtgcaacctgtagtctaacatgtcccctccctgccagtctgtaccgccccac
ctcccatccatgtcattttcacactatttcacttaccgtctgccctactttcttcttgcatgaaaagtga
gcatacatgtcaacagccgccgtttcgcatctgactcccaagcacccactgtaacactatgggacctaca
tgctttacatcaaaatacaggaatgactccctgctaccataaaattatcacctataatcaattcactaat
tttcatagtacatacaggccttcaaatacagtaacactcatttttactaaaatattgacgggtactttat
gcgaaactgtcccttgctacc
>KolobokP-2_CGi-LTDR FRAGMENT 1 -> 461
ggtctcacacaaggaattaatttcactatatgttactatatagctttaattcagttaattaattaactat
actctcagcatataattcatgaaagagaaattttagttaaatgcttcaagaaaaattttagatcagaaaa
ataacgccttaagcggtttctaagagagataattgagagtaaacatacctacccccaaatacacgtgggt
tccacatgttgacaaacaggctcagcaccatccaggaagttgcatcatgcgcatctaaaaaaatagttcc
actgaaattagtgtgacaattcccctaaagtaataattctcctagccactgagtccaaaatcaatactat
aggcctaaaataaggtgccaaaaaaggaagaaagaaaagaaattgtctctatatatatggaactactatt
gactaagttcagtttgattggctatttccttgcgtaagacc
>KolobokP-3_CGi-LTDR FRAGMENT 1 -> 653
ggatgcaaaacacagttttctatatgacgttatcgcgacgtcattacatgagggacaagcaaaactgacc
cctataaagaaatgttgttaaaatgtgttacatatatatctatatatcaaaagaaagataaaatgatgaa
ttttgtaaatattaaaagataacacatttatcaataaataataaggtgtattagacgatggaaatatggg
gtaaatatggagataatgccccaaattgctctacacccattcattttagcacccctaacacaaaattgca
accaaatcagccatttcaagttctaagcatttcacagagcaagtcttaaggtcttgttttcatgttattt
tagggtacattcgccagctttaaagagctatattacctgctactcaagacatgaaattctgcaactgtaa
aatccggatgtttatcgagttacctcctctatatcagagactcacaaaattaattttaaacattttgcat
atttttacatctattctaaagataagactatattctatgcaactgaaaataaaagaaattatttcatgta
taccgcacaaaaatagcacgaaaatccctacccatcatgctattcactggggaaaaacccccctggatttt
atacgaaactgtgtttagctacc
>KolobokP-4_CGi-LTDR FRAGMENT 1 -> 451
ggacacagaccacaattatttttccctcttattcctttataaaacaaaaattcatataaaaaattccacc
ggctcaaatctattcaaatttggcataatgacagacaaacattagtccaacaacatttccaaaggacagg
aaaatcaatcgagaggggggctgagatatggcccctacaataacccctaccctgcaatatccactttcact
tcggaatatttgtaaacattggcctctttacacccttcctattgcgcctttaaagatcgattttcccaat
tatttttagctagtatttttttttcaaaccttcttctttccatccatgccctaaaaggcgccaaattcgcc
catttagcacccctgggaatttttggaaaaaaacatacatttttagaatagaactacttgactgatcaat
gagcacggggtaaaaatagtggtatgtgacc
>KolobokP-4N1_CGi-LTDR FRAGMENT 1 -> 451
ggacacagaccacaattatttttccctcttattcctttatacaaaaataaatcatataaaaaattccacc
ggctcaaatgagttcaaatacagcatacctatagaatgacactagtccaacaacatatccaaactacagg
aaaatcaatcgagaggggactgagatatggcccctaaaataacccctaccatgaaaaattcactttcact
ttggaatttgtgtaaacattggcctctttacaccctttctattgcgcctgtaaagataatttttctaaat
tttttcctgcctgtatttttttttcaaaccttcttctttccatccatgccataaaaggaaccaaattcgac
catttagtacccctaggaatttttgaaataatacatacatttttagaatggaactacttgcgtggtcaat
gagcacggggtaaaaatagtggtatgtgacc
>KolobokP-5_CGi-LTDR FRAGMENT 1 -> 461
ggtatcacaccggtaattgttttcgctatataattctatgtaaggaaaaaaatcgtaaaaaatcgaaata
ttttttttgtgagaaataaaatacatagcaatgttctatgataccttactcattatctaacatcagaaaa
tgggcatgcgatactgcattttctcaagttttctagctgcgaacatgcctacccccaaaaccacgtgttt
tcaagctgtttgtaaacagactgcgcgcaccccaggaagtcggcctatatgccctttaagaaatagttcc
cgttaaaagtgaaatcaaatttacaaagtacagaaatttccctaatcatagatatccattttattaaaat
cgaatatgggaaaatgcaaaatgcgggcaaaagaaaaaaatattccttataaatgtatggaactacaatt
gactaagttcattttcattggccgattaccggtgtgatacc
>KolobokP-6_CGi-LTDR FRAGMENT 1 -> 651
ggtggcaggggacagtttttttgagtgcctaacatgtaccgagggggtagacatatacttgaggtcaatg
taaataaataagggggggacagtttcatgagggctggttaattacacttcatatacccctagcttaaataa
tagacccacccagagcatgtaatctacatattgcagtgcagatgcactaaaaacacactgggggccctgc
aagattttggcttgatttttcaaaatttgggcctcatccgcacccctacagcccctcccagtcccgagcg
agggagataaattacaaagcatttggtagataattgtatgccctgatgaaatttcaccaattgatctggg
gtcaatcaccttcgttctgagatacagaccttctcctaaaccctactgcttaccagcggaatttcctcag
catctcacagcatgtattagtttcgcatttgcatctatgtctatcctaagcatcatagtgacacctacat
attgcacatcatttttctcagcaagcactctgctatacaggaaatgtatctgataattacttcattcatt
tttattcataaaatatctcatcaaacagcacctatcactaattttaagaaatttgaccgggtctttagt
acgaaactatcccctgccacc
>KolobokP-7_CGi-LTDR FRAGMENT 1 -> 456
gggtatagtccactaatacattttccatagccggtaatgttaacataatgtcacacagaattctccctaa
ttttcgttcagcaacgagtgatccctcaaactgaagctacacaagttgtccatcttcagtccgagtttcg
tggtttgatgtcagattcgttttcccgcaaaatccgtctgtttacgtcaacccctgaaaaagaaagtgaa
agtagtaaactcctgcctatcagtgaagcatcaaataacacaaaatagttccatacactttcaggttaaa
tcacattttgcttaatatacttacaggaaaaatgatcctcacttacaagtatgatgttcataataataca
tagcttcattatttttttatgtagtctgctaataaaggaacttttaattttaatggcacttctttgttgt
cctaccgattctaaaaatagttgaaggataaaccc
>KolobokP-8_CGi-LTDR FRAGMENT 1 -> 448

```
ggttgcacaacactattaaattcccctatataatcttaccaaacccactcacctttagactgctctcact
caggtacaacaaacttcccagacgtttccctttcacatgttaaagtactattcttactgtacatttcagt
accaaaaaatacctacccatccacaaaggcgtgagactttgtttacatccaagtcaagcaggtgcttgaa
aaccatgcctggagccaaatcaccccactctgtatgaaaaaaaattaaaaatatttcttggcctcctcaa
tttttttaagcatatcatctgaagcaaaatacttgaatttacacctcataaaaaaattgggcacccacc
tcttaggaaaaaccctcaaatcaaagggaaaaactgggttctatattctgtccgccatgttttgacgtga
accttcagagaaatagtgttgtgatacc
>KolobokP-9_CGi-LTDR FRAGMENT 1 -> 448
gggaaaaggctaccaattatttcccatagaactctatacaaaccttaggacctctcagtactttactat
gacaatagcagccaaatctttggtatcatttgaaacttcattttaagcactttcagaaatttccaaaaaa
aatacatggtcccgtggcgtttataggtcaaattcgcacttgtttacaacttacagcgatcagcagtaaa
tggcaccctgcataaaaattaaccccactcctacaatctcctctggcatttctgtattttctgcaatatt
ccctcatcccatcatttctttcaaacatttatattttggctttaaaatgaaaataattacatacctgaat
tcagccaatcgcctgacgcctatccatttattccgtgttacaatgttctataactttttctctgatgtca
acatccggtgcattggtactcttattcc
>KolobokP-10_CGi-LTDR FRAGMENT 1 -> 449
gaaaaaagagtaccaattaatttcccatagaactcaatgttaacctaaacacccctagctactttgctat
attacttacagacatatccttggtaaaatctgaaagttcattccaagcactgacagaaaatcacaagaaa
tatatatggtcccatggtgtttataggtcaaaattgaacttgtttacaacttacagtgatccgcagcaaa
ttaacccaagtctctaaaatgacacccacctcctactttaacccctctaatttctttactctctgcagta
cttcctcatttcatcatttaatttaaacactggtaattttactttaaaatgatcattatttcaacaactt
attcagccaatcacctaagccctaccttttctgctaggctcaatcttgtattaacaatggtctgatgtc
aacatccggtgcattggtactctcattcc
>KolobokP-11DR_CGi-LTDR FRAGMENT 1 -> 450
gggaaaagcctaccaattatttcccatagaactctatacaaaccttaggacctctcagtactttactat
gacaatagcagccaaatctttggtatcatttgaaacttcattttaagcactttcagaaatttccaaaaaa
aatacatggtcccgtggcgtttataggtcaaatttgcacttgtttacaacttacagcgatcagcagtaaa
tggcaccctgcataaaaaattaaccccccactcctacaatctcctctggcatttctgtattttctgcaata
ttccctcatcccatcatttctttcaaacatttatattttggctttaaaatgaaaataattacatacctga
attcagccaatcgcctgacgcctatccatttattccgtgttacaatgttctataactttttctctgatgt
caacatccggtgcattggtactcttattcc
>KolobokP-N1_CGi-LTDR FRAGMENT 1 -> 455
gggtatagtccactaatgcattttccataggcggtaatgttaacgttatggttcatagctccggccctaa
ttttcgttcagcaacgagggacctcttgaatgaaagctcatcaaattgtctctttcctgttaaaatttcg
tggtttgaagtcagattcgttttccggcaaaatgcgtctgtattgaaaaactctgaaaatgaaagtaaaa
gtagggaatttctcagttttggaaagggtgtacatcaaacaatttcaattcttttcttcaaatgataatt
caattttgacacttgttttaaaaattgcaaatcctcttttctgacatgtgtcaagcattctgatttaaaa
tgtcccaataaatgtatatacactctgcaagtatagggactattttgcttaaaggcactactttgttgtc
ctaccgattctaaaaatagttagagggatataccc
>KolobokP-N2_CGi-LTDR FRAGMENT 1 -> 648
ggtggcaggggatagtttcgaaggaaagacccggtcaaaattttgaaataaagggagaacctggggtttg
gctggttatttgaggggtataaattggtagaatatttattgcattcattttgtggatagaggagctcatg
ccaatttcattgatatatgacactttaatactggtagcacttttcatcagatatggaatgaaaatgcgaa
actgtggccaaaattttcactttcggttttgtgcttgaaaagtagggtgggttcagaacacgaaatgtca
ctcggaaagaaggtgattgaccccccatcaattggtgattatttgcatgggtatacattaagctaccaat
cctatggtagtttatggcagttgctcgggactggagcgtggttatggacccgtgaaaatgtgaaaaaagg
gcaatttttcagaaaattttgagaccgtccctggctattctttatagtgctatatgtaagttgtacttgt
tttattctgaaatgtttaaaaattctcaagagtcgggaccatgtgtccctgcatgcaaaccaattttagc
aaatttaaaatccactgaaaaattaaatcacatatatgagcactatctgcctcacatttcctcgaccaaa
aaactatcccctgccacc
>KolobokP-N3_CGi-LTDR FRAGMENT 1 -> 460
ggtatcacaccggtaatcggccaatcaaaatgaacttagtcaattgtagttccatatatacttcagaatt
atttttttccttgactgcattttacattttcctttactcagttttaaaatttttgtaccactgagtag
gatatttcatgtattttgttaggtgatgattttttttcacagggactacttctttcagggctcatgcagc
agcttcctggggagtgtgcagtctgtttacatacaaatggaaaacacgtggttttgagggtagacctgtt
tggagctagatattttgagaaaatgcagtatcgcttgcccttttatggtgttagctgatgagataggta
acaaataacatttcctccgaatattttgtcatgaaaaatacaaactgattttaagaattttttcccctc
tatagtgttatatagtgaaaacaattaccggtgtgatacc
>KolobokP-N4_CGi-LTDR FRAGMENT 1 -> 650
ggtggcaggggacagtttttttgatacaattcattgtagccaagggatgcatgtcttcggaactctgtaa
ctagaatttcctcagttcccattcagcacaaatacctttaattcactctttataaggccaatcaccaatt
tctgaagaaaattactaatcaaaacctgtaaaattctctacatactggtttttatgagattttgaccctt
tcatattttgctaatttttcatgatttttcagctttttagacctcccccagctaattccctgcagagggt
tgaccttccgtaccgcgtgcatgttgcactatcacatgtcagaaacttaccggtgtatagtttacaatgt
cccatccacctacttttcgtgttattttacctcccgtctgtaacttgcaatttcactgtgtaaagtcagc
acaacagtcatagccgcaggtttcgcattttacttctgattctcatgtacctaacataaggggcctacat
attgcatatcaatttcaacaagagacacccctctaactaatgataatcattaaaaatcatttcatgaatt
ttagcaacactcataagccttcaagtacagcaactctcaattttacaaaaatattgacgggtactttatt
cgaaactgtcccttgctacc
>KolobokP-N5_CGi-LTDR FRAGMENT 1 -> 445
ggttgcactacagtattaaattctactatatattcttaccaaaattgcacaactttacatacagattaaa
aattcaaaacgaacttctggaaaaaatcctcttgacatctttttaaacaacgcttcatttacaagtttct
agcaaaatacacacgtgcatccagcaaggcgtgcgactttgtttacatcgaagttacacatgtgcttgaa
aatcaagcccgggacttttcaccccccctccagaaacaacgcgcatcaaaaattcaaatgacctcgcatta
ttacaaaactgggatagttagacctcttacacattgtttttcatctcatacaaaaaatcagctcctgtct
cgagcggaaccgccccgaattcaaaggaaaattcgggaattattttggctcagccatgttttgacgtgaa
ccttcagtgaaatactgttatgaca
```

>KolobokP-N7_CGi-LTDR FRAGMENT 1 -> 449
gggaaaagagtaccaattcatttcccatagggctcaatgttaacctaaacccacctggctagtttgctat
tatacttacagacatatccttggtcacatttgaaagttcattccaagcactaacacaaaatcaatagaaa
tacatagggtcccatggcgtttataggtcaaaattgagcttgtttacaacttacagcgatccgcagcaaa
taaatccaagtcccaaaattgacacccacccccctatttcaacccctcttatttcttcactttttgcagta
cttcttcaatccatcattcattttaaacagtgataaatttactttgaaataatcattatttcagcaatta
attcagccaatcaccttagccataccttttttctgcgcggctcaatcttgtattaacacttctctgatgtc
aacatccggtgcattggtactctcattcc
>KolobokP-1_CVi-LTDR FRAGMENT 1 -> 451
ggttgcaccactaaattaatttcccctatatattcttaccaaaatcacctaacttaaccctggtcctact
catttaaagtgaacttctaaaatgtaatacttacacatcctgtagtactgtccttaatgttcattttat
gcaataaaaaacatacccatccagaaaggcgtgagagtttgtttccatcaaagtcaattaggtgctttat
cactacaacaaaggcctgttcaccccctccacgagaaaaaaaattaaaaatactaaatggcccccctaaa
ttttttkgctttacattttctgaaactacatactctattttatatctcataaaaaatttggacacctacc
tctcagggaaagtccccaaatcaggagaaatttttgggaattatatcctgtcggccatgatttgacgtga
acctcaaggctaatatagtggtgacacctgc
>KolobokP-1_CHo-LTDR FRAGMENT 1 -> 451
ggacacagaccacaattattttccctmttattcctttataaaacaaaaattcatatgaaaaattccgcc
ggctcaaatctattcaaatktggcataataacagacaaacatcmscccaacaacatatccaaagtacagg
aaaatcaatcgagaggggctgagatatggcccctamaataacccctaccctgmaatatccactttcact
tcggaatatttgtaaacattggcccctttacacccttcctattgcgcctttmaagatagattttcccamt
tttttttagctagtattttttttcaaaccttcttctttccatctataccctaaaaggaaccaaattcgsc
cattcagcacccctgggaatttttgggaaaaaacatgcatttttakaawggaactacttgcgtggtcaat
gagcacggggtaaaaatagtggtatgtgacc
>KolobokP-2_CHo-LTDR FRAGMENT 1 -> 449
gggaaaagagtaccaattcatttcccatagaactcaatgctaacctaaacacccctggctagtttgctat
tatacttacagacatattcttggtaacagctgaaagttcattccaagcgctaacagaaaatccatagaaa
tacatagggtcccattgcgtttataggtcaaatttgtacttgtttacaacttacagcgatccgcagcaaa
ttcttcctagtctcttaaatatcacccaccccttattttaacccctctcatttctttactttctgcagta
cttcctcattccctcattaaatttaatcattgataattttactctgaaatgatcattatttgggcaatta
attcagccaatcacataagccctaccttttctgctcggctcaatcttgtattaacacttctctgatatc
aacatccggtgcattggtactctcattcc
>KolobokP-1_SaGl-LTDR FRAGMENT 1 -> 461
ggtctcacacaaggaaataatttcactatatattactataaaacttgaattaaattatttaatttattag
actctcagcagaaaattcatgaaagagaaaataaagattaatgcgtaaacaaaaatttgagatcagaaaa
aatatgccttatgcggcttttaagaaagaaaatcgacagtaaacatacctaccccgaaatgcacgtggga
ttcacatgttgacaaacagcctacacaccatccaggaagttgcatcatgcgcacctgaaaaaagagttcc
ttttaattcagtgggataattctgctaaagaaatgatattcctagtacatgagtccaattttaatactgt
aggcctaaaacaaattgccaaaaatgaaaaaagaaaaaaaaaatttctctatatatatggaacttcaatt
gactaagttcattttgattggctatttccttgcgtaagacc
>KolobokP-1_PiIm-LTDR FRAGMENT 1 -> 450
ggtatacaggtagtaattattttccatagacttacatgttaacgtttccccaaataacctacatttatag
agtgatttggcaaaattattttctttcatttgaaagattggtccatgttgcttaaaatattggaagaaaa
aaaggggggttaacgttgcttttctatctcgaattttcgaatctttaaaacttgtgtctagtatgcactaa
atgaacaactagatttttttttgcccccctcgtttaaattactccatataaaatttatgagtgtaaaattc
agaaactttttttatttatgcagaaagtacattgaatgcactttaagaaaaaatgcatttcttcactgt
ttatccaatcactgaccagatatctattaattccgtgtcaaactttgggtggcctcccacgtgaccaagc
tagctcacaggatgttggtctatgtttacc
>KolobokP-1_PeMa-LTDR FRAGMENT 1 -> 460
ggcctcagaccacaattaatttccctctatgttataccaaagaaaaataaaaataaaaatatttcaaaac
attgcaaaaaatctagttttcatatatgttgtagaactgttcaatctgaatctgcagtgagctggaaagt
acctcatgaccggcatttaagaaagatatttttatttaaatgccaccacccctctatattatgggaaaat
ccggaaaattacaaaatatcactactcaaaccttataactccctgtgtccttcctcacaaacaaacatgc
aaatggtgtcaaaacaaaattctcccactagaatagatattgcactacaaatatatgttaaaattatgaa
catatctgcaatagctaagtcaggaaaaaatatttagtaacatacctgaaatttacttatgtactacatt
cgtgtacaacgacccggaagtgtaactgtggtctgaggcc
>KolobokP-2_PeMa-LTDR FRAGMENT 1 -> 459
ggtctcacgcaagtaattcagcccctatatttattaccaatgaattaaagctataattaattcatttga
agtttaattcaagtcagcaatggctgaagttatagatacaaacttgttacacagtttgaaacccaaatgt
tgacaggttttaagccttctaaggatctgacaggtccatgtgctgtattaccggatgtttacatacacaa
ccggtgctattttgggaaaatacctggtcattttgcacctatgtgaatacgccagtatgtacatctcagc
acagcaaaatcagtatattctgacagtttggaatatgtacttcaaataaatatcagatatctcaacatca
cacctaaacatatcaatcaaaataatatttaaatgtggaactttattttcttaccagactactttcgcat
acaacacttgctgattggttgatttgcttgcgtgagacc
>KolobokP-1_MiYe-LTDR FRAGMENT 1 -> 452
ggactaagaccacaattaattttactatatgtttcattgtaacatttaatttcataacaaattcccaata
tccgatagccaactttccatctaacctggtcaagagcaacatcttgggagtaaaatatcaaaatttcaac
atagcatgttggcctttttcttgagataatcgtctgcatgtgtcaccaatgtcaaaaataaacttccggg
tcaggggtagaggtaaacacaggacttttcacacagcaccctgcacaaaaaattaatgcacaatataaaa
atttgacacagaaattgttagattgatatctccacagtattttaacaccaaacttttacacaaacaccaa
gttctaagaggcatagtatagtttttactaaagtacctatatttctaagggacttcattcgtgggtactg
aacccggaagcatttaattgtgacctcagtcc
>KolobokP-2_MiYe-LTDR FRAGMENT 1 -> 460
ggactcacacaactaaatcatttactatatgttattaccaaacattttagctaataactttaatatttga
agttcaacaccagtgacaactgtctagattcataaatacataccttgcaaaggttacaaaacgacttttt
tgataggtttagtggcatattagagtgtcacagcgtccagaagttgacaaccggaagtttacaagcataa
ccggtgacccaaaacagaaacgcccccatacattttgcccctgtgcaaatccgcacacagacatggaaat

```
atatcaaaagtatacatttttcaaaagttaaatacctgaacatttcatttatctaaaaatctgtcatcag
ttccttattaaacttcctcagtggcccagtaattagccggaactaattttagatagggactacaaacgta
tgaaacacttgctgattggctgatttagttgtgcgagtcc
>KolobokP-1DR_PiNo-LTDR FRAGMENT 1 -> 448
ggtcagacaccactaaatggttttccataggcgacagtgttaacgtttggtcaaatattctatgtttccg
acgttccaacgcatataaactatataccgatttgtaggccgtcccttccccaattctcgaataaactcaa
aaccatgggttgaccttctggattttcgctacatgtccatgattggggccaccccccatttcggcacaaa
atgtacagaaaattttgacactgggttacacctaattcaaggggcccaaaatttcacttttttcaacaatc
tagtcattctacatatttttcaacactttactgcccactttataatattcaattgaaaataatagcatta
tacaccaatcagagaacatctaacgtcaaaatagcaggtcccctctccctacaccttcttaaccaaccaa
attgccccttatttagtggtgtctgacc
>KolobokP-1_MyGa-LTDR FRAGMENT 1 -> 456
gggtcaacatttttaattaattctccataggcggtaatggtaacgttttcctccgttgctcagtccatat
cgtttacttgaacatgtatgatggctcatatcgaagctgatacatttatacatgtgtggttaaattttcg
gggtggcaagtgagattactttttttcgcaaattgctggaccccggaagatggtgaaaaatgtcactctcc
acatgaaataatcccaaaattctgatcataaaattcaataaaaaaattgtcctttctaataatttatttc
aggtcaaatctacatatttctttttctcatcacatcagctctataaaacagttcttattgttcatttatg
tccatttttaaaatcacagcatccacaattgtatggcggactaatattgtttttttaattacgtcatctga
gttcctcatctcaaggtctattagggatcctgaccc
>KolobokP-2_MyGa-LTDR FRAGMENT 1 -> 455
gggtcaatatgcgcattagattctccataggccataatgttaacgttttcttctgttgctcaggccatat
cgtaaacttggatatgtatgatagctcgttttgaagctgagacacttggacacatgtggtgcgattttcg
gggtggcaagtgtgattcgttttcccgtcaagtgcccaaccccgaaagatagtgaaaaatgtcactctcc
acaggaaataatcccaaaattctgaccataaaaatcattaaaaaatatgtcctttgtaatttttttgatca
ggtttattttgtatacttttttcacatcacatcagctctataaaacaccagtcctagttcatttatgt
caatttttaaaatcacagctcccacatatgcatggcggactaatttttttaatgagagtcaaaaatctgtg
ttcctcatctcaaggtcctttaggggtcttgtccc
>KolobokP-4_MyGa-LTDR FRAGMENT 1 -> 449
gksccgagaccacaattaattcctctattagttctttataactttttgtctcattaaaaaaaattkacat
attctttttgkcagatcttttgtgtgtgtaatgtacagtacaagtccaaaaatatgtccaagwttcagaa
agattgcacatatacaacttacaaatttagccaatacacatccatacccctatggacaagtcaagagatg
ttccgaaaactgtaaatatcaccttttttgcacctgacctaatcactctttccatatcaaaatcagttaaa
atacaacataaaaatttatttcacctctcttctttcatttccaccccataaaagctaagaaatgwwtgag
aaaggsaaagaaaaaaawtwaagaagaaatacactttaattaaagggaactatattcgtgaacaacgaaa
agtagggtcattaattgtggtcttgggcc
>KolobokP-1_MyCo-LTDR FRAGMENT 1 -> 449
ggcccgagaccacaattaattcctctatcagttctttataacgttttgtctcattaaaaaaaaatgacct
attctttttggcagatctttcgtgtgtgtaatgtacagtacaagtccaaaaatatatccaagtttcagaa
aaattgcatgtttacaacttacaaatttagccaatacacatccatacccctatggacaagtcaagagatg
ttccgaaaactgtaactattaccttttgcacctggcctaatcactctttccatatcaaaatcagttaaaa
taaaacatctaaaatttatttcagccctcttctttcatttccaccttataaaaactaagaaatgtttgag
aaaaggaaagaaaaaaaattaagaagaaatacactttaattaaagggaactatattcgtgaacaacgaaa
agtggggtcattaattgtggtcttgggcc
>KolobokP-2_MyCo-LTDR FRAGMENT 1 -> 453
ggcccgagaacacagttattttcccctatttgtctattaaaacataatggcaattaaaaaaaattccacc
tgctctattctaataagatttggtgtgcataaagtttgacttttatcccaaaacatatcaaaaattcaga
aaagtctaccagctcattctctcctggtaaccccaaacataaggacaccggtaaggatgaagccagagag
cttccgagaagaggaaattccacctttatttacctgaccaaatcactcatttctacagaaaatttcaaaa
agattttttcagacagtatatttgaagtcttatctttcacctaaatgcaaacaaataatgaaaaattcc
aaaaaaagacaaagaaaaaaaataagaaaaaaaacactgaaattaaagggaactacatttgtggacaacg
aaaattgaaggtagataactgtgtcctcggacc
>KolobokP-3_MyCo-LTDR FRAGMENT 1 -> 458
ggttgcacttgtgtaaaaccagcaatgcaattttccataggaactgcctaaaatgcatatgcagtcacac
ttcattcaggaagttttgaggagaatgttatatttcccttgaaaggagacatgcactactttttttgca
ccaataaaaacatatagctgtgcggcatattttcaagttttactggcctccatttataaacattgcagga
tcgcactgaatttttccattaaatttgactcaacacccaaatgtcttccagtcctcctgaaaaataagtt
aaataaatagacatttttatttatgtccatttaattctccaatcaagtcttaattaaaaaaaaatagttt
gcatccatggcaacaactatgtaaacagtagcagtattgatatgcatattcaatttctccccaaagaggc
gtaacttgcaaatcacacgtgatttacaaagtgcaacc
>KolobokP-4_MyCo-LTDR FRAGMENT 1 -> 447
gggtaaattcagtaaaamatctcccattaacttcaatgtaaacctacatgttcaattaaatttataactt
ttttacctttagaaattagaagctttcatatttcattcattaaagtacaaatgtagccccatcttttgca
accatcaaaacatagggtcatgcggcatttttgggccgtccacatggctttgtttacaaacactgtagtt
tcgcacttaattcctacaatgaccccatcacttctcaatcctgttttaaaaaaaattaaatgcattcagc
attatttaattatttttttaaccttagttttgatcaatctaatcaaaaaaaattattgaaaactttatc
taccaatcattgcagcacaggtcttcaattttatactgaaagctctcccccaaatgggcggtccctgatg
acgtatatttatttactgaatttaccc
>KolobokP-5_MyCo-LTDR FRAGMENT 1 -> 464
ggaacggggacccagttatttgccccattgaaacgcattgaaagtcatagtaaataaatggggtgctgtt
cattaaataattttgatagccaccttgggacttctgtttcatgttaggcattagtttccaagtttctaag
tacaatctcagtgcctcatgaccggcattccgttgcaaaattttgttttgattgacaacagggtcactca
gtctacttccggggaagaataaaggctagaatttaggcaatttactctatgcactgacgccagattatat
ttaaatcaatcaaaatttttacagaaattcaaactagaaagcctaccctttaaaataaatctacattcag
ttacagaacttgtgtaataataacactacaccattttagataaaagttatattttttttttaagaactac
attcgtgggtactggactggttgccgtaactgggatcctattcc
>KolobokP-1_MyEd-LTDR FRAGMENT 1 -> 656
gggtgcaatcaacagtttttttacgtgacgtcattctgtaacagggcatgtaatagtggacccatcatgca
```

gaagtcagatattcatagttatatagatatctatacatcaatggaaagataattctatgaactttctgaa
taaataaaaaaaaatccaacatctcttgtttaaacatgcaaattggtacaagttatcagctggaaattag
gcattttcccccctaaaaaaccttaaaaacagaccacctttggacacacggatcagtaacctgtgcatata
tttgagatttcttataatatgcatttcgaagagcttgtccggctgatttaagaaaatgtagtttcagcct
acgttcgcctgctccgaaaggagctatcttacctgacaaatcaaagtgctttttgcaacaggtgaaaatc
agctgtttatggagttgcctcccatatagtaggaagtcacaaaaacatttttttttttttaaatcttgac
aaaagtggcattttaattgaacttcaatatatgtttttcacattgaacataaaaaacaatcaactttttc
atttagtggtatataaatagcagggaattccatcagtatgtaaatctcactggggaaatacccctagttt
ttagtacgaaactgtttatagcaccc
>KolobokP-1B_MyEd-LTDR FRAGMENT 1 -> 655
gggtgcaatcaatagtttttttcatgacgtcactacgtaacagggcatgamatwttggaccgatcataka
gaagtcaagtattcatagttatatagatatctatacatcaacggaaagataattccatgaatttttcgaa
taaataaaaatatatccaacattwttgggttgaaaatgcatattggtacaagttataagctgcaaatgag
gcatttttcacgtaaaaaccttaaaaacatacgacctttgggcccatggatcaataacctgggcacatat
ttcagttttccaataaggtgcatttcgaagagcttgtccggctgattttagataatgaagtttcagggta
cgctttcctgctccgagaggcgctatcttacctgacaaataaaagtgctttttgcaacaggtgaaaatca
gctgtttatggagttgtctcccatatagtaggaagtcacaaaaacattttatttttttaaaatctttaca
aaagtggcattttaattgaacttcaatatatgtttttcacattgaacataaaaaacaatcaaattttcca
ttcagtggtatataaatagcagggtattccatcagtatgtaaatctcactggggaaatacccctagtttt
ttatacgaaactgtttatagcaccc
>KolobokP-2_MyEd-LTDR FRAGMENT 1 -> 464
ggaacggggacccagttatttgccccattggaacgcattgaaagtcatagtaaataaatggggtgctgtt
cgttaaataattttgatagccaccttgggacttctgtttcatgttaggcattaggttccaagtttctgag
tgcaatctcagtgcctcatgaccggcattccgttgcaaaatttttgttttgattgacaacagggtcactca
gtctacttccggtgaagaataaaggctagaatttagacaatttactccatgcactgacgccagattttat
ttaaatcaatcattttttttacagaaattcaaactagaaagcctaccctttcaaataaatctacattcag
tttcagaacttgtgtaataatgacactacaccattttagagaaaagttatagttttttttttaagaactac
attcgtgggtaccggactggttaccgtaactgggatcctattcc
>KolobokP-3_MyEd-LTDR FRAGMENT 1 -> 687
ggtagcaatacacagttagcaaatgaagctgaaatttggtctttaagttcaagaacatgtgcatgcattt
tgaaatggatataggcaatgctacaagaatacccaaacatgtatacagatttagaatcctatttctcagc
agatttcaaatctgtaaacaaacacttaaaacaaaattgtacaagttcagtatccatggaaacatttggt
gtttgttttctatttttttcttcaaaaattgctgttggacacccccatacaggcttaaaaatatcctttt
tataaaatttcaatgaatatcagacatacattgtttcaacactgaatgatagccctctcaaaaaaaacaa
aaaaaattgggggtcaatctgctcctttttccgcaaattacctttcaaaagatcgaggcaaattttcacc
tttgaaaaatagtcaacaaatgctgatatagttttagagtcattggtggactggatatgaatttagaccc
gaaaactacatttctactgagcaaaccaaggttcaatctcttctctggaatcatacctttcaattaaaac
cactctcaaagcatcaaattttatttttcttcattttcaggtacttataacttgtctttctttttttatct
taaaaattcacaggggccaaaatgcgaaactaacacttctaactgtgtattgctacc
>KolobokP-4_MyEd-LTDR FRAGMENT 1 -> 447
gaatgtaaaaaacaattgatttttccattgccgacaatagtaatgtttcttgctgtactgatcttattga
gttgatttttattataaaacctttttttaaaataaagaatatktattgtgttaacatatttcataaaaaaa
tgatatggtcgcgttgcttttctaggtgtaattkacagttgtcaagggtgaccgtcaatctcgcacaaaa
ataacagtaaaatttatgcattcaacaacaggaagaaaaaaaattctcaactatttctgaaggaggaatca
aattttattttgtcattgctacaaccaagaatcaataaggtttcaggaaaaaaaatattatttgtggcat
actccacccaatcaaaatcwgccatgtttttgtttctgaatactatgttatgctcattttcgactggtcc
caaaggagctaatggtagattataacc
>KolobokP-5_MyEd-LTDR FRAGMENT 1 -> 459
ggttgcacttgtgtaaaaccagcaatgcagttttccataggaactgcctaaattgcatatgcagtcactc
ttcattcaggaagttttgaggagaatgttatatttccctggaaaggagacatgcactactttttttttgca
ctaataaaaacatatagctgtgcggcatattttcaagttttactgccctccatttataaacattgcaggt
tcgcactgaatttttccattaaatttgactcaacacccaaatgtcttccagtcctcctgaaaaataagtt
aaataaatagacattttttatttatatccatttaattctccaatcaagtcttaattaaaaaaaaaattactt
tgcatccatggcaacaactatgtaaacagtagcagtatttaatgcacattcaatttctccccaaaagagg
cgtgacttgcaaatcacacgtgatttacaaagtgcaacc
>KolobokP-6_MyEd-LTDR FRAGMENT 1 -> 451
ggccacagaccacaattaattcctctattagttctttataacttttttatcacattaaaaaatttgcacca
tgcacttttgtccaatcttttgtgtgtgtaatgtatagtacctgtccaaaagtatatccaaaattcagaa
agattgaagcaatcacttttacaaaattagccaatacacatccctacccctattgacaagtcaagagatg
ttccgaaaactgtaaatatcacctttttgcacttagcctaatcactctttccatatcaaaatmagttaaa
atacaacatccaaaagtttatttcacctmtcttctttcatttccaacccataaaaactaaataatcaatg
agaaaggcaaagaaaaaaaataaagaaagaatacaccttaattaaagggaactatattcgtgaacaacga
aaagtagggtcattaattgtggtcttgggcc
>KolobokP-7_MyEd-LTDR FRAGMENT 1 -> 454
ggtccgagaacacaattaatttcccctatctgtctattataacataatgattaataaaaaaaatcccacc
tgcgcttttctaacaagatttggtgtacataaagtttggcattaaccacaaaacatatacaaaaaattag
aaaacttcatcgcctcatttctcttggcaacacaaaacacaaggacaccagtatgggttaagccagaga
gcttccgaaaaggggaaatcccacctatgtttacctaaccaaatcactcatttctgctcaaaatatcaaa
aagaaattttacagaaattctcttagatgaccttacattcacattaaagcaaaaaaaggggaaaaaattc
aaaggaaaggcaaaaaaaaaaatttaagaaaaaatatactgaaattaaagggaactatatttgtggacaac
gaaaattgaaggkggataattgtgttcccggacc
>KolobokP-8_MyEd-LTDR FRAGMENT 1 -> 448
ggggtaaattcagtaaaaaatctcccattaacttcaatgtaaacctacatgttcaattaaattcataact
tttttacctttacaaattagaagctttcatatttcattcattaaagtacaaatatagccccatttttttgc
aaccatcaaaacatagggtcatgcggcattttcgggccttccacatggctttgtttacaaacactgtagt
ttcgcacttaattcctacaatgaccccatcacttttcaatcctgttttaaaaaaaaawaaatgcattcag
cattatttaattattttttttaacctcagatttgatccatctaatgaaaaaaatattattgaaaacttta

tctaccaatcattgcagcacaggtcttcaattctatactgaaagctctcccctaaatgggcggtccctga
tgacgtatattatttactgaatttaccc
>KolobokP-H1_MyEd-LTDR FRAGMENT 1 -> 455
gggtcaatatgcgcattagattctccataggccataatgttaacgttttcttctgttgctcaggccatat
cgtaaacttggatatgtatgatagctcgttttgaagctgagacacttggacacatgtggtgcgattttcg
gggtggcaagtgtgattcgttttcccgtcaagtgcccaaccccgaaagatagtgaaaaatgtcactctcc
acaggaaataatcccaaaattctgaccataaaaatcattaaaaaatatgtcctttgtaatttttttgatca
ggtttattttgtatacttttttcacatcacatcagctctataaaacaccagtcctagttcatttatgt
caattttaaaatcacagctcccacatatgcatggcggactaattttttaatgagagtcaaaaatctgtg
ttcctcatctcaaggtcctttaggggtcttgtccc
>KolobokP-N1_MyEd-LTDR FRAGMENT 1 -> 663
ggtagcaataaacagttaggaaaaaatactaaaatttgcccttatgaattttaccatccccttttcattg
ctttcttgcaatatcaagcttactgtttgtgtcatatatgggcatatgtatgtaatcagaatcttccata
gattttatatccagtatataaaatggtaaaatataatttctttttggtgtatccatggcaacaatctgca
tttatttgttataaaatattgcaaaaaggggggaaagatgtcatatatttccccaaaatttggctaaaa
gtagaatttcaatcgcttgaagtaataccttttctgaaactgtgtacatatatcattcagcaaatccaat
gataatcatatgtctttcatctcatttttttgtaaaattgcgtcaaaaacttcgcgtagaaaaagagtgt
ttgtaaacagtacattaatttctggaccgatggccggtctagctatgaaaatacggattgtcaaacagaa
aacagcccataaaacatgtaaaaaataatcttgatgaacttataaaccatctttgaaggctaaattagca
ctcatctgtctaaaaatgaattttattacacaataactttcctattttaaaaatccacaggggccaaaat
gcgaaactaaactcctaactgtgtattgctacc
>KolobokP-1_MoPh-LTDR FRAGMENT 1 -> 453
ggcctcagaccacaattattttgcctgtgctttctttgtaaaaataaagacatattaaaaaaatcgcacc
ggcattattgaaatgacactcgcaccacataaagtttgatgtcatcagagtaacatatcaaaaaatcaga
aaaatccatcgctgtttttttccgtggcaacatcataaacaaccatggggtatgaccagtgaccaaaatc
tcaaaataaacacaattttaacctttgaacttttagccaaagcacaacttcctttttaaaaattctgat
aaatatggtgtttaatattaagttcaatcattctactatcatattaacaagaaaaaatatcaagattttc
aacacaagtcacaggaaaaaaatcagaaaaaaaaattaaaatttattacttggaactatattcgtatacc
acgagtaaggggaattaattgtggtctgatgtc
>KolobokP-2_MoPh-LTDR FRAGMENT 1 -> 454
ggccacagaccccaattattttcacagtgcattctaaccaaaagtaatgtcaaataaaaaaaaaaatcgca
ccattttaaacgacatgacacttggcagacatgaagttcaaccttacccgataaaaatactggatgctca
gaaaaatcatccactggtttttttgaatgacggcccaatacacaattgtcatgtgtcaacatccgcaaaag
tgaaagtaacaaccaaaataatgacctatgaacttaagccaatttactacttccttttccaaatattatc
tattagaagcctgaaaacaatttttcaaacactctgtaataaaaaacaactaaaaaacattcatattttt
caccagaaagtgcaccaaaaaaatttgaaaaaaatacacaattttcaatagagaactatattcgtagac
cacgagcaaagggaaataattgggatctgtagcc
>KolobokP-3_MoPh-LTDR FRAGMENT 1 -> 451
ggcacgagaccaccattaatttcccctatttgttctttataacaaaattccaaattwaaaaaatcgcacc
acctcaaaccacatgagatttggctcactcacagwgwagcccaagacacaaaaaatatcaaaaaattaga
aaatcctaccgtkggaatttttttacagctccacaaagatatccaccatcacaaaaaggcggaggagtt
ccaaatactgaaaacttgacccttttgaccttagcctaactacagctaaaacatggaattttatttcaac
tgtatttttactagtttttcaacatctcttctttcaattaaacacaaaaaataaaagcaaaagaaaaca
agaagggacagaaaaaaaaaatatgaaagaaaaccccccaatttactaagaactatattcgtacacaacgaa
aattgaggggaggtaatagtggtctcgtacc
>KolobokP-4_MoPh-LTDR FRAGMENT 1 -> 458
ggccagactccactaaatattttccatagacgacaatagtaacatttctcaatgtactgattttacctta
acttcactttaagtgacagtgccatttcctgaaagggtcatgtcttcgctacaaaatatctctctggcag
gccaagtttaaagtcaaataagaaatttctgacatgtttacaatttgacgtatcacctggagcatgtatt
tccggtggcgtccctagaaactggaccaggtaaaaaattcctgtttaagaaaaagtttctcaacaaaaat
aactgtttttgggatcaaaatttacctgaaacttttacctaccacatcaatgtaattttaatcaaatttg
gacaagcatacggctttgcgcagtaatataacacagctatttttattttgataagtacttttttttacta
tgccagcatccgattggttgatttagtagagtctaacc
>KolobokP-5_MoPh-LTDR FRAGMENT 1 -> 459
ggaggcactccacttgaaaatttcatgtgcatttcaaccaaattaaaaaaatcataaaaaaatcaaaatga
attttcctacaaggatgaccagcaatttcaagatatttagggccttgcccaaatttacaacacaaaaaac
caccaacttcattaccaaataagaaaaatttcagctgttgaacacttccggtgtaaacatgccttattac
aagtactgagaaaatgctccactaggtgcgcaagaatttgtcttgtaattttcctgtatgcagttgtcac
taaacaaaacctatggaaaaatgctctggatagaccatacaaacaaactatatacaggtttactgcaaaa
tgtatccattcaacaccataaaaatttttttgaaaaccttactactttgttaaagtactactttcgagggc
tgtctgtatccggtttggctaaataagtgaagtgccccc
>KolobokP-6_MoPh-LTDR FRAGMENT 1 -> 452
ggcctcagaccacaattattttgcctgtgctttctttgtaaaaataaagacatattaaaaaaatcgcacc
ggcattattgaaatgacactcgcaccacataaagtttgatgtcatcagagtaacatatcaaaaaatcaga
aaaatccatcgctgtttttttccgtggcaacatcataaacaaccatggggtatgaccagtgaccaaaatct
caaaataaacacaattttaacctttgaacttttagccaaagcacaacttcctttttaaaaattctgata
aatatggtgtttaatattaagttcaatcattctactatcatattaacaagaaaaaatatcaagattttca
acacaagtcacaggaaaaaaatcagaaaaaaaaattaaaatttattacttggaactatattcgtatacca
cgagtaaggggaattaattgtggtctgatgtc
>KolobokP-7_MoPh-LTDR FRAGMENT 1 -> 450
ggcccgagaacacaattaatttcccctatctgttcattataactaattgggagataaaaaaaattgcacc
tgctcaatcttcatgaaatttggcatgcttacagaataatcttagcttgataaaatataggaaaactgga
aaaattccccatttcgtttttttgttgacacttccataaacatatacaccatcatcgaaaaagtcagaggt
cttccaaacaatgaaaatttaaccttttttgaccccagccaaatcacaccttgtggatgaattttttttca
tctttgatttttttattgtatttcaatgtctcctctttcatttagactaaaaaaatacccatttttcaaaa
gctgaggcatagaaaaaaaattaaaagaaatacactaaattagtgggaactaaattcgtggacaacgatt
cagaaagggacgtaattgtgttctcgtatc

```
>KolobokP-1_BaPl-LTDR FRAGMENT 1 -> 470
ggtgtgaaacctataattcatttctttaacattttctactaaatttgtgtttttttcgagcctacctggcc
attttatcttaagtttaacctttcgaagaccatacatttcctgaaacatccatgattcctctacgttctg
tcaaaatatcgggccatgtttaaagccttgcagtgaaatgccaggctatggaaaatacctactctaaaaa
tagctccgtcaacccccagaaattccgtatttataggtcatgcatcaaatcctccatatccatattaaaa
tacaactaaatcaatcaaatttgggctcaaaataatccttatacatttaactttaattatcaattttaat
tattccattccattgagtatttttcacattacaacaaagtgaaaggaactttttttctttaaaagggacta
cttttgtatatgttcttagctctgattggttgaattactggtttgacacc
>KolobokP-1_PeVi-LTDR FRAGMENT 1 -> 461
ggatacaagagcgcaattatttgtaatgcattttccatagggtacctatgtagtttgaaaattaatttca
agaaaaccacacaaattagcagaaaatgagtttcagttcctaaaactagaaaagcatactttttatttgc
acaaaaaaaaagtacatggtcatgcggcaaaagttgacctaaatttcagttgttatggactcttgaaaat
tcagcacaaaatacactgcaaaaaaaaatgaagcacctagatttcatttatgagaaatcatttcaaccct
ataacattttattaattattaccaaaataaaatcaatcataaatgggtaagctttgaaatgaaaataaat
ttcaagccatatccaaccaatcaggtaccaccttgtattcagtactcagtaccatgttggggtaaaaaag
ttcccggtagaatgtaaacaaataattgcgcatttgtatcc
>KolobokP-2_PeVi-LTDR FRAGMENT 1 -> 456
ggtctcactaaccacttctgacgttcccattactttacatgttaaagttgacattttcgaagtttcactg
tgaattcscaggacaaatggkaccttaatwtggcataccaatacgaagctttaaaaaagaggatattcwt
actggtgtataaatggtacggtcatagtctaaatttttcaccaaattaccttgaakacaaaatactcaga
aaaccacatgttttcattcacaaaaaatggcagcatggggcaacttacagwgtcatgtcagcagtattca
tacgcactagttaaawataatgcatatattctaaawatagactctcgctttaatttgatagcaatatcta
ttaatagaatgaaataaaacactcataataaatagacttacagttactttgtttttggtactacataggg
gggtggccaattgacccggaagtggtaagtgagacc
>KolobokP-1_TeGr-LTDR FRAGMENT 1 -> 688
ggtagaagacctcagtcgtgaaatgaagatcagaaatgcccaaaattacagctacaattttcttatttac
agtgccagcatctaaaaattctactaggaagctatacatatgcatatttttttcaaatcaggagtatcagg
gcattcattctatcatatttgaaagaccaaaatggccatgtatatgatttaagcaatcattcagtttcca
tgacgatacttaagaaatttggcaattttggccttaaaagggctagttttcatcctatcagttggtcgac
ataccccgaactacgtataatgagacctatgtctcctgactacttaacttgtccccttttgaaaaattgg
gaaaatcgagatacttgtctgattcatttttttcacaaatttgaagtgtttctcttacctgtgaaaagcac
atgtgggattttccctccaggaagtgatatatacacaggaagtgttcatcttaacataccttcttacgcc
aaagatatagtacctagctcggtccaatgggtgttaattttaagcacatctctgcagctttcaagattgt
attatcacagaacaagtcttgaaaacgtgtgctgactaggcagggagaatggaaaaagtactttaagttt
tatatgcactacttttgaccgtcccacttccggcatatcacgactgaggccttctacc
>KolobokP-1_SiCo-LTDR FRAGMENT 1 -> 453
ggcctcagaccacagttattttttactatatgttctatatagacattgtggacactggtcatgttggtaac
tctccaatcaaatcagagtcacttgctgtcagtagtatcatccaagagaagaaattctagcagcagaaag
ttgggggtcaagctgcttccggtcccataatttccagtcaaagtacaaccatgcaccattcggctgtaat
ccgccggaaccacgtgaatttggccatgaacacaatcactgaaaatagacaaattgacataaagtcttca
tcaatgatttcatttaccagcttattaaggtccactctgttgaaaaatgtccatgttgccatctctttca
ttcatcaacttatttatatccgactttcaacacatgcactatttactatgtaaacacttccttattgtgg
tcaaccaagggcaaataactgtagtctgattcc
>KolobokP-2_SiCo-LTDR FRAGMENT 1 -> 470
ggttagacgacactaaataagtttgttatatattctatagtaagtcgcgaaactttggaaccaaattgca
ccatgaccaagctatgacagacccccataaccatatataggtgaaaagagctatttgttgtctatcagaa
agacccagaaataagtcaacttttgcaacttgtaagaaggtcctgcttcatacagtgcacataccatcaa
attgccagtcggtaaccctcagatttcaatgaaatatgtacttccgctcaaggtggatcagtccattatt
gtgcatgggagtgcaacattttttgcttgattttaattctacatggtttcagctttcttttgataccaaaa
ggttcagattaccttcaaaataggaaatatgatgacaaattatagcaacagactgttattaaagagaaaa
agtccatgtcacacattctacttccggtggctatttagtgtaatcacccc
>KolobokP-3_SiCo-LTDR FRAGMENT 1 -> 453
ggcctaagaacacagtttttttcactatatgtttcatataagcatctgtcaacagaacatttttgtttac
ttccagaacagaaaaatcttacctgaactttgtaatactatatattgagacattttctagccgcaggaaa
ttgcatgttgaccagtagtctagaccaataatttcaaaagtatatccggtgacttgacaggtcaataatc
tcggcggtaaacaggccaaagacatccctgttcaatagctgatttgcaacaatctatcattaaacttcca
tcaaaactgacatttactgactcatttacacatactttgtacaaaaatgccatttaatcagtcttcttca
ttcaacttttaatcagtattcgtcagcagaaacagggactcttttgtcttgttctgtttcctgttcttgg
gtaacgaagggcataaaactgtgttctgacgcc
>KolobokP-1_CySi-LTDR FRAGMENT 1 -> 650
ggtggcacaccacactttcgacaggtttcttcggmttgaktaccgtctcactcaaaaaaaaacctctctgt
acacagacaaacaacaacctgaaaaasatatgatttaaactgtagtcacaagaccataamstttcaataa
acatcaactagaacctatgaaaacttcaaagtckttcacagtaaamaaatttctcggcctctcctcgcca
tttttcaaggtttgccgagccagaattggccaaatctgaaccgctgcaaaamctcacaggaaaattack
gatatawaaggcctacttckccttgctcttctcaaacmgaaggtctgaaaaatcataaaattaatagctg
tcatagcttccctttttccacaaaatccgtttgactgtctgcagctcttttccgactgctcgcatcagct
aaatcattcaaattgactagtttcgaattccaagctcctgcataacactgatgaacattaatawtagtgt
acctggcmaaacgcaaataattatgatcactctctttcctcaaaatgagaaatcttagaatttaaattat
tttagatctcagaaatgacactagagttcaaaattaccttctcgtacagaagttctacatkgccgattcg
catttakggctacaaaagtg
>KolobokP-2_CySi-LTDR FRAGMENT 1 -> 461
ggtcagagactactaattattttcccatagggttacattgtaaccttttgctctgccactaattccctta
aattcattcagtaccaagacccacttgcttgcatagaacaatcctagacacaaacttccatgcactttttt
atatggggttcaccagcatttaagagagctagccggtctcaatgaccttcaccctcaaaaatgctaaaaa
tacagcagcaaattttgtcataatacctcattcaataaagtccatcaaaaataccttgaatatgctttga
agtctttataaaaatgtttgttttattaaataagcatagtccttcaatattaataacaaatatcaccaaa
agaataactaccaatcaaaatacagcttacaagacatctaagttagcccgttattccctaaacaaaatgt
```

gccaatcacgagggccctaccacaattagtagwctctgacc
>KolobokP-3_CySi-LTDR FRAGMENT 1 -> 461
ggtgtgaaaccaccaaatagattccttctttattctatataaaattcataaagttcacagggctgtaaaa
catagcaaaacctttttaaatagctttaacttacatattcttgtagatctatatctgtactcatgaaaa
atagcaagaaaagtgggggttgtgtttcaaacaagaaagatatttctgctcatgtaaacttacccataaa
attaacaaaaaatctgagctaattttcagattggggtatgtgaaacaatttcacattgtgaattctattc
tgaatgcatatgagcataaaattgctaccacactatcagaaatagtcaaacaaaatgattccagcaaaaa
aaatacacagattagggcaaaaatgttaaaaatagccaagaaaactgagaactatttctagccgccatta
tgcgggtaccatttttgagctgaattggtggtttcatacc
>KolobokP-4DR_CySi-LTDR FRAGMENT 1 -> 459
ggtcagagtaaactaaatggttttccataggcctacattataagatctaaccctctggagattagtttaa
atgattattttcaaaaactccaactttttgcagagacacatcctaggggagtctaaaattgctcagcaa
atagggtcttcaccaattcaatctcacacaattttgaaccggaagtggtcaccccagaaactgacactaa
tctccgagccaaaaataaaaaaataccaacatcmtttaactgcctcaaattgccaattcawatgcactga
atgttccttttcattttaaattatgcaggtaagccttatctgcagaaatatgtccaaatctttattttg
atcattacactttcaaccaatcagactctgtagactctgtaagcccgaatcgtccctagacaaaattagc
cwatccgagaacctagtttctatttagtgcactctgacc
>KolobokP-1_MeMe-LTDR FRAGMENT 1 -> 456
ggttataacacactaaatagccaccactatatattctatataaaatgacaagttttcacaatgctgcaat
acacacctagacccattttaaatttctttaacttacattttcttgtagaataacatttatactgtaaaaa
tatccaaagaaaagtaggggtcatgtttgwtgcaagaaaaatatttctggtcaaacggacacaccgtaat
ttttacactgaaatgacaatcacaatttagggtaggggtgtatgaataaatttcacatgttaaatcagtt
tggagtctttttcaacatgcaaatgctaccagtatatcaagtataggcatgcaatatgatttcaaccaa
tatatagcaatgatttcaaggaaaaatccttcttacagcaaaaaaaaaactgagaactatttgaatccgc
cattatgcgactaccatttttcaaatgtgctcagg
>KolobokP-2_MeMe-LTDR FRAGMENT 1 -> 453
ggccaaagaccacagttatttttactatatgttctatatagacactgtacaatattgcaattttgtctgc
attccaatcaaaaaaaaattacctgccctgaatagaactatccaagagaaaaaattccagccacaagaaa
ttatgggttgaccggctcctttttcagtatgtttacccaaatcatacgcctatggtaaatagtcttttg
acggctgcaaacaggcaaaatctgtcctcttcaagtgaccggttttgcatattcagacaatttaatccta
tcaaaattcacatctaactattatttcacatctcctctcttaaattatgccttttagtgcatgtctttca
ttcaatttttcaccaatattcaccatcaaacagaggaactattttccgagtaagcacttccttattgtgg
tcaaccaagggcaagtaactgtggtcttgtgcc
>KolobokP-3_MeMe-LTDR FRAGMENT 1 -> 470
ggtgtcggcccagtacaatttcttcttctttgctgtatagtaaaacaagcaacttctgacatggattgtg
actttcacaacaaaattgcagcattgacagtcctgtacttctgaagaatgaccctgttccagtcaatata
gcttagaaaatcactatcatcaacgatgaactttttttgctacacatcaaagttttaagtccatgtttata
tttccaatacacatctgtcatgtctaccagtctggagtagtttttttaatccatccgcacaggaatatagt
cctgaaaaacacagacaaaacaactgttaaagacatcagaaatggcacaaaatgtacacagtgtcagtaa
atacatttaaaatataacatgtcaaagtagggatggctttaaattgggaacttcaaaatttcaaagaact
acttcctaggatacaacgacccggaagtacttcaataccggggtgacacc
>KolobokP-4_MeMe-LTDR FRAGMENT 1 -> 471
ggctgaacactactaaatgcagcccctatataattctatataaaccacacttcaaaaccagctctgcaaa
tctatttcctaatatatcaggtcttggcagcctaccatctttcaaaagctaaatgttaatgcaacaaaaa
aatcaaaagaaaatagggtgttaacttttatataagaaaactgtgagcagctgaaaactcaccatccaaa
atacagcaacttttacaccggttccggcttgttgacagtggtattcttgcaccacttcctgttttcataa
ttgacaattaaacttacccaaagtgaatttatgtcatctacacaacctatattacttgtaacaatcaaca
tcagtggcatcaatcagtgtccaatgaaaaaaaataagggaaaaaaattgcactatattttaattgggtc
tacttgtgactagacctcggaggcttacactgtatttggtagtgatcagcc
>KolobokP-5_MeMe-LTDR FRAGMENT 1 -> 648
ggtggcaggggagcgcttttcggctatattttaagcaaatccacaattctcaamtgtttttgtttatata
ttacaagtgcaaaaagagcattacttctkctttacattgtgatattgcactaaggtaaccttcaaaatga
aaccattaattctgacccatgtggtcaactttaagaggagcagacatgaaagcctgcccttaagcaccct
taaaatgaagaaaatctgagcatattttccttactttcaacacatgacatatactcgtttgtgtaatttc
atacacttacaccaggttttcatataaatcaacccttaagttgtaaaaccatgctaagcaaagtgtttcc
caaccaccaacttttaggagaaaaagcaggtcaaagacacttactgtccgttttgctgcagaaaacacta
gatttgtttaaatctgcctccacaaataatgactcacaaaatttatggatgtaggtgtagcaaaatattc
gcatctgctatttatagctacatgtaagtacacattgatttcttgtagaataaactttcaataattaaaa
cattaatattaatacactttaaacaaacctaccctaaatatttaaaaaatccaccggggccataattcga
aactgctcccctgccacc
>KolobokP-6_MeMe-LTDR FRAGMENT 1 -> 475
ggttacactctaccaattcgattccttctttatttcatatagggaattaaattttcagacaccattttct
gtactttagagcatttcaaaggtatggaaaccatatatagccttttagtataacatgtctacttaccaga
aataaaaaaatattgacatgttatgttgaatataagaaaaataatttgttcagaaaaacaacaccgtctg
aaaatgcttccatgaaaatgctaatcgaacaagtacgcgtaggtagacactttaatcaaaaaaatatact
aaaacatggaaatgcactgtgacagttgcttagatcttttctcaatataatctgcaatcaaactaaacca
aaaatgtaactgtcacatcatatttcacttgtttctgttatattttatcaacagcatggaactatttctt
ggactacttcacatgtaacactatgtagtcacttccggtttggtataatcagtcc
>KolobokP-7_MeMe-LTDR FRAGMENT 1 -> 661
ggtagcagatgccggtttttttctsctacttcggacttccgagcaatgtaccaaagtcggacggcttctga
tttcttacgagatgcagacctgaaaataaaaagatacctgtttacatcttacttgaagaagaaataaaat
acatagtaaacttcatacaaagcactgattttaaatccacaagccacctgaaagataatttcagcccgc
gaaaatatgtgacatttcagcccaaattagccatttctaaaagtggccttaaaatgttagggaaaatgat
agagccaaatggccacaagcattctgtacccagtttcaagtattacttgcagacctggacaaaaatacct
gtcaaccgattactttttttcactattttcaactgtgtgaggcttatatgatatttgacagttcacgagac
gctcaatgctgatagtggtatatacgcatttcaagcagttatatgaaaatgatgccatcaagcttacatt
cgcatctgccagaatttctgcaacataccattcttaaacttgttaatcaawaactcacatcaacaaatat

```
ttcaaacatcaaaacagccttcacttcaaggttttacctttaatgacagattttgaccctggccaattcg
aatctgtggctggaaactaccaactgctacc
>KolobokP-1_CorFlu-LTDR FRAGMENT 1 -> 473
ggtgtaagacctgtaaatagattcccctatataagctatagtaaacatgacatgcttcaaaaggctggca
atcatagctggtaccatttgataaggggttaacttacatgcttttaaagctaagtgtttgwattatccat
ttccagcaagaaaagtaggggtcatgtatcaattaacagagatctgacatcttgaatccggtggtctgtt
tttttcactacggtgtgaacttcaaaaatgttttacatatgccgtcttaaaatcagggccctttttttata
cattgcatttccaaacataaatttggtatcagtgtgtcagtaatagaatgccagttactaaacatgtccc
ttttagcccatactggccaattaatgcccccaaaatgcaaaaaaaatgggactatttagtgtcagccatt
gtctgactagcccgagtaggaacattctgtggactatttacaagtaattagcc
>KolobokP-2_CorFlu-LTDR FRAGMENT 1 -> 454
ggcctaagaacacagtttttttccctatatgtttcatataagcatttgtgaacagaacatttttattaac
gttccgaatagaaaaaactcacctgaactttgtaatactatccaatgcgacatattctagtcacaagaaa
tggtaccttgaccggctactttgcgcaataatttcatgagtgagacggctggtttgacaggtaaacattc
tcggcagtaaacaggccaaaataacccatgttcaaaggctgatttgcaataatctatcattacaattcac
tcaaaattgacatttacttactgcttcatacctcctctgtacaaaaatgccatttaatcagtcttcttca
ttcaattttcaatcagtattcatccccaaaaagaggaactctttactgtgtaagtacttccttattgtgg
gtaaccagagggctaaaaactgtgttctgaggcc
>KolobokP-2N1_CorFlu-LTDR FRAGMENT 1 -> 454
ggcctaagaacacagtttttttccctatatgtttcatataagcatttgtgaacataacatttttattaac
gtcccgaatggaaaaatctcacctgaactttgtaaaacaatccaatgcgacatattctagcagcaagaaa
tggtaccttgaccggctactttgcgcaataatttcaaaagtgtctcggctggtttgacaggtaaacaatc
taagcagtaaacaggccaaaataaccccctgttcaaaggctgatttacaacaatctagcattaaaattcac
tcaaaactgacatttacctactgtttcagacctactctgtacaaaaatgccatttaatcagtattcttca
ttcaaatttcaatcagtattcatcatcaaaaagaggaactctttactgtgtaagtacttccttattgtgg
gtaaccagagggctaaaaactgtgttctgaggcc
>KolobokP-3_CorFlu-LTDR FRAGMENT 1 -> 648
gggcgcagggtacagtttccgagcagttttccgagctttgccagtgcgtgcaatgttattctgtttgaaa
aaaatacaattgcattaataaaatgttgttattatattttctgtgttgcataagaatagtggctttcaaa
ttattggcaactgtactaaaatacacatccttcagaaaatatgcagatatttaagttaaccccccaccccc
tgattttcacatgaaaatctatcaaaattcatgcatgtttgaaagcctgcatttttttttcctgcaggaag
ttgactcatctaacccctttctggtcaatatttcaactatccctaataacccaagcaccaggaaagtgtc
tcccatccagctactttttttcagtatttgtcactgaattacctgattatcaagtttttcacgtgctcgtc
tctgaaagctgttttcccatccgacaaatgctcaacttacaacatgcaaagctagcatatacatgttgta
gcagtcagcatcccatttactatcatgtaaactatataattttaaacaaaatctttcatctttatttcaa
acattaattataatgcattttattctggggtaccccaaatatctaaaaaatccaccgggaccaaagtgcg
aaactataccctgcccac
>KolobokP-4_CorFlu-LTDR FRAGMENT 1 -> 473
ggttgaaccaaactaaaaaaatttccatagacactaatgttaaataaatgattttcaattgcatcttgtg
gttttatttatgtcttaatctcagcaaaacctggtatcagcttgaaggtctgcccatcctctaatgtgta
aattaaagcaaatggcatgttaacttacttgtaagaaaatcccagtccaccgaatgacgtcaccctcatt
tagtctagtatccatacgcggaatttccctaacctacctactttcactttccaattaactgatcatccat
tttctatctgattttcttattttatgtctcaaatgatttctaaatgattgcttgtatcataatgaacaca
aataaccacattcttaagtagttcatgtctttacatacagaaggaaaaatgaactatttagcaatttgca
ctacattcgaatggaccacggaggaatactgggtgatttggtttaatacaacc
>KolobokP-4N1_CorFlu-LTDR FRAGMENT 1 -> 473
ggttgaaccaaactaaaacaatttccatagacactaatgttaaataaatgattttcaattgcatcttgtc
attttatttatgtcttaatcccagcaaaacctggtatcagtttgaaggtctatctatcctcttttgtcta
aaataaagcaaatggcatgttaacttacttgtaagaaaatcccagtgcaccgaatgacgtcaccctcaat
ttgtctagtatccatacgcgggatttcctcaacttacctactttcactttccaattaactgatcatccat
tttctgtctgattttcttattttatgtctcaaatgattcctaaatgattgcttgtatcataatgaacaca
aataaccacattctgaagtatttcatgtctttacatacagaaggaaaaatgaactatttagcaattcgca
ctacattcgaatggaccacggaggaatactgggtgatttagtttaatacaacc
>KolobokP-5_CorFlu-LTDR FRAGMENT 1 -> 647
gggcgcaggggatagtttcagaggggtcttctggactacgcgcctgcctgcgaacttaattgttctgaaa
aaataaaaactgttgcactaaaattatgttgttgttgtttctgtgttacatggatctggctccatgcaaa
ttatgtcccactgtaccaaaatacttatcattccgaaactatgaggcatttaagttcaagtcgcggtcct
catttttcttaagaaaatgtgtctaaatccgaacatatccagagacctgcaaaaaatttcctgcagagatt
ttccatatgcaaccctgtttctgttaatatttcaactgtgcccaatttcccaggcaactggaaagtgtct
cccatccgtctcgtttttttcagcatttttatttgaaatacctcacctccaaattctacaattgctcaccc
ttgtaaactgtattttcatccgacttctgttcaacttgcaacatgcaaagctatcagccatgcaattgag
ccaccagaatcaaatatttcaatgtattatctgtcccaaattaaattaaaatattgcatttttaattcaa
acaataatataaatgcactttattcttgggtaccctaaatatttaaaaaatcgaccgggaccaaagtgcg
aaactatcccctgctcc
>KolobokP-5N1_CorFlu-LTDR FRAGMENT 1 -> 647
gggcgcaggggacagtttccgaggctacctccagcgtctgggaccccgtgcaagcttacattgactgaaa
ataaaaactttgcacagctaaaattttgttgttgtactttctgtgttgcaggggtctagctttatgcaaa
ccacaaccaactcttctacagaaccaatcattcatgatttatacaggcatttaaagtcaccccaagtcct
gattttccaatgcaaatttggccgaaattctacacatttgaaaacctgcaaaatatttcctgcaacgatt
tttcaaataaaacccctttcatgctaatattttatgtgtcccttatttcccaagcagctggaaagtgtct
cccacccgtctcgatttttcagcatttgacattgaaatacctcacccccaaattattctcaaattcagcc
cggcagactgcgttttcatccggcaactgttcaactcagaacatacaaagctagcatcaatgcatctgag
ccaccgtggtcagatttcatattacatatactatacaatattaaaaaagaatttgacatctataattcaa
acaatgattttaatgcattttattcacgggtaccccaaatacttagaaaatcgaccgggaccaaagtgcg
aaactatcccctgcccc
>KolobokP-5N2_CorFlu-LTDR FRAGMENT 1 -> 648
gggcgcagggtacagtttccgaggcggttttcggagtctgggactgcgtgcgaactcttctttactgaaa
```

atacaaattttgtactgctaaaattgtgttgttgtattttctgtgttgtaggggtgtggcttcatgcaaa
ccacagccaactgttctacagtaattatccttcaggattttaataagacatacaagttcaccctacccct
gtttttccagtgcaaatctggcaaaaatcctacatatttgaaaacctgcaaaatatttcctgcaaaaatt
tcataaataaaacccatttcctgttaatattttatctgtgcccaattccccaagcagatggaaagtgtct
cccatccatcaatatttttcagcatttgtcattgaaatacctcaccgtcaaattattctccgattcagca
gggcagactgtgttttcatccgattactattcaactctgagcatacaaagctaccatccatgcatttgag
ccaccatgatcagatttcttattgcacaaactatacaatattaaaaagaatctctcatttttttattcaa
acaatgattttaatgcatttaattccagggtaccccaaatatctaaaaaatcgaccgggaccaaagtgcg
aaactgtaccctgcccac
>KolobokP-6_CorFlu-LTDR FRAGMENT 1 -> 461
ggtcagagaccactaaatagtttcccatagagttacattataagatttggtcctcctgtgatttagttta
cacgacttgttccaaaactaccacagatttgtatagtggcactcttgggaaatgcaaaaatgcccaggaa
acggtatcttcaccaactcattctccagcattttgaaccggatgtaatcaccccccagaatgtaaacaaat
ctctaacccaaaatcagaaaagtaccaacttcataaaaatgccacaaatcacctttcaaacagtgtttta
aaaaatcattttcattttaaagtatctatttaaagtcattttacatttttgaaaacaaaacattattttt
attctttatagttttggccaatgaaactttgaaaataaagtaagcccgaatcgtccctagacaaaattgt
ccaatccgctacaggcttttctatttagtgtactctgacct
>KolobokP-6N1_CorFlu-LTDR FRAGMENT 1 -> 461
ggtcagagaccactaaatagtttcccatagagttacattataagatttggtcctcctgtgatttagttta
cacgacttgttccaaaactaccacagatttgcatagtggcactcttgggaaatgcaacaatgcccaggaa
tcggtatcttcaccaattcattttcttagcatttgaaaccgaaagtaatcaccccccagaatgtaaacaaa
tctctaacccaaaatcagaaaagtaccaacttcataaaaatgccacaaatcacctttcaaacagtgtttt
aaagaatcattttcattttaaagtatctatttaaagtcattttacatttttgaaaacaaaacattatttt
tattctttatagttttggccaatgaaactttgaaaataaagtaagcccgaatcgtccctagacaaaattg
tccaatccgcgacaggcttttctatttagtgtactctgacc
>KolobokP-7_CorFlu-LTDR FRAGMENT 1 -> 662
ggcagctgacctcagttttaagtcgtattttcacacttgccgacacacactctgtaaatggtctatgaaa
tcaatccacaacaacaacctgaaacagaaatctgtgtttaatgtaaacatatggctatgaacttctcagg
caacacaaatttcaggctatgaacaactcctgacaaaaaaacaaggaacactgaatctcaacccaagcat
cacttttgcacagtcggccgagccaaaaatcaccatacctaaaatcttgcaaaaaattaatgtccaaccg
acaacttcaaaaaattgttgctttcagtccagctgaaacagcagattcagaaaacaatatagccaatagc
tgtcatccctaccgattttctgcaaaatcaatccaaacacatgcaactcaatttctgcagcggaaacagc
tgactttacaggatttccctagtttcgaatccaagcccctgaatcacactgattatcacgtggctgcttt
tggtttctaacaattattaaacaaatatattctcaatactctgaaaacacaaaacctgtatgtataatta
atttaggcttgataaatgccaaaatatcttcataatatcctctatttcataatttctacatggccagttc
gagtaggtggctatcaaactgctatgcgctac
>KolobokP-7N1_CorFlu-LTDR FRAGMENT 1 -> 663
ggcagctgaccacagttttcagtcgtaatttcaaacttgccgacacacactctgtaactggtctatgaaa
tcaatccacaacaacaacctgaaacagaaatcagtgtttactgtaaacatatggctatgaacttctcagg
caacacaaatttcaggctatgaacaactcctgaaaaaaaaacaaggaacactgaatctcagcccaagcat
cacttttgcacagtcggccgaggcaaaaatcaccatacctaaaatcttgcaaaaaattaatgtccaaccg
acaacttccaaaaattgttgctttcaatccagctgaaacagcagattcagaaaacaatatagccaatagc
tgtcatccctaccgattttctgcaaaatcaatccaaacacatgcaactcaatttctgcagcggaaacagc
ttactttacaggatttccctagtttcgaatccaagcccctgaatcacactgattatcacgtggctgcttt
tggtttctaaaatttactaaacaaatatattctcaatactctgaaaacacaaaacctgtttgtataatta
atttaggcttgataaatgccaaaatatcttcataatatcctctatttcataatttctacatggccagttc
gagtaggtggctataaaactgctatgtgctacc
>KolobokP-7N2_CorFlu-LTDR FRAGMENT 1 -> 664
ggcggcagtaaacacttattttgaatattttggactttcaacccgacgccaaacacttgccactctggca
ctgtctaacaacttgaacctgaaataaaaagatgctttcttgatataacacatacaattcagtatttaaa
aacaataatcatattgttaataaaccatgacttcatattttcttaagaattcaattttggggcttggtcg
cgaaattcaatgacaatttagagtcagtttgcactttttttaacagtcatagctttcttattgccaacga
ctgatacttgaaacctgtaaaatgctttttctcattacctagtgataacaagcagctaaaacaaatatgc
tcataaccgataagattttgcagtattccagcaaatctgtatgcccctgaaaaacttgccggtaaaatgg
cttccgagcagacgtttgcatcacttccgtttccacttcctcaaaatgatgtaaagctagcaagttcaag
atattgtgcatttttcttatttctcaaacttcatatttggcagcaagaaatcaaattttgataaaacaatt
actagttagtcaaacaagctacttaaatctgcaacttacctcttatcttatatttttgaccggggccaatt
cgcatctatggctaaaaaactctttactagtccc
>KolobokP-7N3_CorFlu-LTDR FRAGMENT 1 -> 664
gggggcagtaaagggttttctgatatatcgccaaaccgaagaatcacagccgacacgacaagtccacttg
ctccctactagcacatacctgaaacagaaagggcacataactgacatcacaatgacattcagtttatgaa
aacaaagcaaaataatattcaaaacatatcaattgaattctaagcaccattaactttcaaattctggtcg
cgaaaactggtcgtccgacgagacaaaattccaaacattcatcggtccacagctttcttattgccaacaa
ccaaaataaatgcttgccacttacacatggtaaattccctactgtttcattaaatcatactcaaaatata
ccataaccattaagattttgcactactcacacttgtttgtatgccccttgagaattgcaagttaaactga
aggcggagccagaaatgtattagtttcgcatttccaagcacctgcctcacactgaaagtgtcaaggtcaa
aaagagcatgtcacacaattttttggggatcctagctacacatgtgaaactgcaaatatagcatctaaaaa
tattttaaagtgccatgaagcttacagtttgcccacttacctcttatcttcaaaatcgaccggggccaat
tcgcatctaaggctaaaaagtctttactgcaccc
>KolobokP-7DR01_CorFlu-LTDR FRAGMENT 1 -> 662
ggtggcggacgccagtaagcgacagcaatcacacagtcgtcgacacactatctgtaaccggtcgattaat
atgacttacggcaacaacctgaaacaggaagcattgtttattataaacatatggagatcagctactgagg
caacacaaattccaagcaattaacaactcctgaaaaattccaaggaacattgaaactcagcccaagcatc
acttttgcgcagtcggccgaggcaaaattcaccgaatctgaaatcctgcataaaatcaatgtccaaccga
aagctctccaaaatcaatgcttttgattcatcttgtacaggagatttagaaaagactatagccaatagct
gccatccctaccgattttccacaaaattcatccgacaacatgcagctaaatttctgccgcagaaataatt
tactttacccgacttccccagtttcaaattccaagctcctgcatcacactgaatagatatggctgctttt

tattttcaataactaataagaaaatatacactgattactctgaaaacacaaaacctgtaattataattat
tttagacctcaaaattgccaaaatatttccataataccacttatttcataatttcaccgggccagttcgc
atatttcgcctatacaactggtatgtgctacc
>KolobokP-8_CorFlu-LTDR FRAGMENT 1 -> 459
ggttacataccactaattcaatttccatagacttccattataacttttcatcaagttctgaatttactat
tgggtcaagcccttaaaatcatacagttctcaagagaaccaacctagagacaccgtaaaacaccaattcc
accatagcatgaccgataaactttttcggcattttagccagaagtgtatcaccccattctctgcggaact
tttgtttacattctgtcaaaaatgatagctatttttaaagttgaatttcactttttctttcattattaat
ttcatatattttgtcttcaaattactataaaatcattatattttctcaaaataattttctttttaaaata
tttattttcattttctaccaatcaggcttgacttcctgttactggtccgcaaattccctgcacaaaatta
tttagaggcctgaaccccaatatttagtggtgtataacc
>KolobokP-8N1_CorFlu-LTDR FRAGMENT 1 -> 458
ggttacataccactaattcaatttccatagacttccattataacttttcatcaagttctgaatttactat
tgggtcaagcccttaaaatcatacagttctcaagagaaccaacctagagacaccgtaaaacaccaattcc
accatagcatgaccgataaactttttcggcattttagccagaagtgtatcaccccagtctctgcggaact
tttatttacattctgtcaaaaatgatagctatttttaaagttgaatttcttttttttttcattatcaatt
tcatataatttgtcttcaaattactacaaaatcattatatttctcaatataattttcttttcaaaatat
ttattttcattttctaccaatcaggcttgacttcctgttactagtccgcaaattccctgcacaaaattat
ttagaggcctgaatcccaatatttagtggtgtataacc
>KolobokP-8N1b_CorFlu-LTDR FRAGMENT 1 -> 458
ggctatagaccaccaattaaatttccatagacttccattataacctttcatcaagttctgaatttactat
tgagtcaagcccttaaaatcacacagttctcaagagaaccaacctagagacaccgtaaaataccaattcc
accatagcatgaccaataaactttttcggcattttagccagaagtgtatcaccccagtctctgcggattt
tttgtttacattctgtcaaaaatgatagctatttttaaagttgaatttcactttttttttcattatcaatt
tcatataatttgtcttcaaattactacaaaatcattatttttctcagaataattttctttttagaatat
ttattttcattttctaccaatcagacttgacttcctgttactggtccgcaaattccctgcacaaaattat
ttagaggcctgatcaccaatatttagtggtctataacc
>KolobokP-8N2_CorFlu-LTDR FRAGMENT 1 -> 458
ggttacactacagtaattcatttcccatagactcccagcatagcttcatctcccattctgaattagctat
tgggtcaaggccttaaaatcttacttttgcaaataaaactaacctagagacatcataaaaacacaacaaa
aatatagcgtaaccagtaaactttttcgcgacatacactggaataccaccaccctcagctacgcgccact
ttcagtttcactttgtccaaaatattacccattttaaaagataaaaattttcatacactcaaaatataaa
tgcaaatgttttgtttcaacttcttctatacactgtgcattttcatatgtctatttaatatttttttattc
tgcttttgcattttcaaccaatcaccgagtgactttatctactggtccgcacagaccctagacaaaattg
actggttcccgatccacctgaattactgtagtgtaacc
>KolobokP-8DR01_CorFlu-LTDR FRAGMENT 1 -> 458
ggctatagaccactaattcaaatcccatagacttccactatagcatttcatcaaattctgaatttactat
tgggtcaagcccttaaaatcttacatttctgaatagaaccaacttagagacacattaaaatcccaattcc
atcatagcatgaccgataaactttttcggcattttagccagaagtgtatcaccccagtctctgcggaact
tttgtttacaatctgtcaaaaatacctgctacttttaaacgcggaaaatttagaaaataagtaaccaatt
tcacagttattttctttggcttactactcaaaccctatatttctcaaaatcattttctttttttaaattt
caattttcattttccaccaatcaattctgacttcctgttataggcccgcaaattccctgcacaaaattgt
ttacatgccggatcaacattaattagtggtctgtaacc
>KolobokP-9DR_CorFlu-LTDR FRAGMENT 1 -> 472
ggttgaaccaaagtaaatccaattcccatagacctcaatgttaaattcccaatattcaatgtacattaaa
catgatttctatcatcttaatttctagacaaccatatattggtgtgaagatctacatttcatctattcaa
aaacacacaggaaattggggggttaacttacttgtaagaaaattactgatctctaaccgaacagaccctat
tttaaacgcggtatcgcaaatacggtgccaaaaatatacatccgctgttccggaactagtccgcctctat
caaacgagatataggcatgaaattgctttcgtttcttttcctgacatgttatacctgcaaacaagccaca
aacaggccgatttctaagggttaagaggcccccagatgaagagaaaaaaatatcatatattaaaacggac
ttcattcgaatggaccacggaggaatactgcgttatttggtgttatgcaacc
>KolobokP-N1_CorFlu-LTDR FRAGMENT 1 -> 473
ggtgtaagacctgtaaatagacttccctatataaagtatagtaaacatgacatgtttcaaaaggctggca
atcatagctggtaccatttgataaagggctaacttacatgcttttgaagctaactgtttctactgtccat
ttccagcaagaaaagtaggggtcatgtatcaattaacagagatctgacaccttgaaaccggtgctctgtt
tttttcacttctgtgtgaacttcaaaactgttttacatatgccatcctaaaaccagggccttttttcata
tattgcatttccaaacataaatttgctatcagtgtgtcagtaaaagcatgtcatttactaaacatgttcc
tttcagaccatactagccatttaatgccaccaaaaggccaaaaaattgggactatttagtgtcagccatt
gtctgactagcccgagtaggaacatctggtggactatttacaagtaattagcc
>KolobokP-1_GarTel-LTDR FRAGMENT 1 -> 665
ggctccagaatacagtaccggttacagtcttgcacgccctcacaaccaccgctttcaaaagattcaaaca
aagcctcaataaccaacctgaaataacataaaccatttaaatataacaatgtagcccagaactgtaaaat
gaatggaattaatcacatgtttactccagaaattcgtaaaatttaacaaacgccaacattcggcatggcc
gcgaaaacaggtgaaaaagtcagtcaatttagccttaacccacaatatagcagattccttatgccagctc
atagcctataaatggcatgcttttctgaaacttcaatcaatatgcacattagggagaggcaaaaacatac
caggttacctatgaacattttcacaaaatcttactgtgtttagtctgctccaattcagcatacagagcta
gcctcattccgcacacaagccgacttttagtccctaccctctaaaataggcataaagtgtggagaggtca
tgacctttacattgcacaattgctgaaagtctccttttcacaattttacctcatttaggggtacagcagc
tactcttatcttcaaatttgaggtcagaactgatattttcataatatttgtaaatttgaccatggccaat
tcgaacatttccccccagtactgtatcctggctcc
>KolobokP-2_GarTel-LTDR FRAGMENT 1 -> 472
gggcatgggtcaccaaaaccacctccctatataatgcatataaaatcatcttactttcggttcgcaaacg
gtcaacttttattccaacagcatatgattgacacatatcactttgaagatctatccaaatacaatgggaa
aacaatgaaaaaatggggggtcatgtggcatataagaaaaatttcattcaatatttaccatgactgcact
tctgcaacctcggctgacaggcacagcctaaaactgggacctctcaaatccggttccacaagttctaata
tttggggtaaataacagtctaaatgcatgaaaagtattgtctggagtctcagtaactgacaaatagcaaa
aataaagatgaaatcactcaaaaaatgctttttagagtaaaaaacttggaactatttctttgccggact

cctttcgactacaccatgaagcacttccggttgtttttggtgtccctatacc
>KolobokP-3_GarTel-LTDR FRAGMENT 1 -> 468
ggtgtgacaccggtgaattttcccccattcagcctatgtaaattagcctcatggccaactccacaacac
ttttcacagagttagttcagctttaagattatatttccatcatctgcatgcttagaacagtcttttaagc
accataaacatattccaagtacagtgaacttttccaatatccagcgcagaatgtaacaaccggccacttt
ctctttcaaaaaggtcaatgtcacccaagtacacccacatcttcaagccaccactgttgaaaatagtcct
gcacaaaaaggggaaaaaaacctctcaaatcacagaatacacacaggagttatacacagtcaagcaatac
aacatgtgaaaggtgttttaggctactcacaaacccttcatttgtataactggtaattttaagaactact
tccattgtacaacgacctccggaagtaaaggttcactggtgtcacacc
>KolobokP-4_GarTel-LTDR FRAGMENT 1 -> 466
ggtgtgactccggtctaatttgaccttctttcttctttgtaaaacagtcattttccacatcgcttggatt
ctcccaagaatagatttgggcttaactcatttaacccattgatatttggtccaagcactgcctctccatc
actagaaaactagggcaagattggctgcttttttttgctactgagaaaagtatttatgcaccgtctacttt
cagtttaaatttgaccatttccgacctatcaccctcactttccccaacagccacaagaggaagtagttct
ccaaaaagaaacagaaaattgccacaatgggcagagaatacacaaaattacttgaacatatgacattaca
aggcatgccaggtctaatagacacttttagatccatatttggacagatggcaattttaaagatctacttt
ttactgtactacgacctcaggaagcacttcctcaccggtgtcacac
>KolobokP-5_GarTel-LTDR FRAGMENT 1 -> 467
ggtgtcggcctggtctaatttcgccctatataatctatagtaaaatgcctaactttcagagccaacagct
ccctcaaaaacaggatctaagctataaaagacatacccttcaattctatagacatttccctgttgattga
taccagtcaacctataacatcggcaaatacattttttgctatcaattattttctaaggtcactgtcaaaat
aaaggtaaacaactttaaccttcaacccattcactgtgtttcaatatgcatccgcctggggaaaaagtcc
tgaaatgagatccctaaaacaatgccatttggtggaaatgaccacaaattaccagttttcagcaagaaaa
ccattcaaactcacccacaggcaacactaaacattgccaatcttggaaatgtatttttcaaggtactact
tgcaagaccacaacgacccggaagtgattccagaccaggccgacacc
>KolobokP-1_DrRo-LTDR FRAGMENT 1 -> 451
ggttacgcatgcgcaattaatttccttctatattacatataaaatagttgaagttcgatatcaattttta
ccatgatcaagcgtatagacactatatcatcatacatcattggaaagataaatgcataatcttttgaaaa
aaatgcatgtcattaaattctatgtgttgtaactcaaaatattcaccttagaataggcacaccggttttg
acagctgtggaggcggccattttgggctcaaatagtatgacctactttcaaagccgggaaccatcaacag
aaaaagtagagcacaaaaatggcttttttttcttattgcttacaaatataccttcaatttgataccaaaac
ataccatttgcaatgtattttacaaatcctaggcagcatgcactgaacaatgtgtgctaagtatcaagtt
gactgcatgtaacccttcaatcaggagttcc
>KolobokP-1_DrPo-LTDR FRAGMENT 1 -> 470
ggttacgcatgcgcaattaatttccttctatattacatataaaatagttgwagttcgatatcaattttta
ccatgatcaagcgcwtacccactatatcatcatacatcattggaaagataaatgcataatctttwaaaac
aaatgcatgtcattaaattctatacgckktaactcaaaatatttaccttagaataggcataccggttttg
acagctgtgcaggcgaccattttgggctcaaatagtatgacctactttcaaagccgggaaccatcaacas
aaaaagtagagcacaaaaatggcttttttttcttattgcttacaaatataccttcaagttgataccaamaw
caaccatttgcaatgtwtttkacaaatcctakgcagcatgcactgaacaatgtgtgctwagtatcaaact
gactgcatgtaaccctaaaaacaggagttccggtttggggttacgtgacc
>KolobokP-2_DrPo-LTDR FRAGMENT 1 -> 649
gggggcaggggatatattttttgaccaaatttcgagttccgaaaggccgcaaacagtcaaatccatttcat
caaaacaaaaagtgacacatgaaagtctttttgtgtcagtgtcataacataggcaacatacattgaaaat
acagtcaaacacaaggctggtttcttcacttgtgaaactatggcccttgatctttccccccacccacctc
atttttaatgaaaacttaagaaatttgacccatttcatggcatgcacagtacattcaaacagaatgcaa
tcctagttttggctacattactcattaatttcatccctgtgctgtgtttattcacatagaaaagtgtctc
ccatacgtttagattttcacaatttacctccaaagttctgttctgtttctgcagcgtgaaatattgact
tccgccaaaagtgaaaacctccttttgaagattcaacatttacttgctagggagctgtcacgagtttca
atgtttttttccaaaattttgagacattctttctcaaacctaacacaagaaatacatcttataaataccc
aattgattttaaagaccatagcttacccctaccctaaagatatgtaattttcatggacccccctttcac
tgatatatcccctgccacc
>KolobokP-3_DrPo-LTDR FRAGMENT 1 -> 476
ggttatattacactaattcggattcccatagggtcccattataaaactgacaactttcaacgcatttttc
ttgtctttccgacgtatcaatttttccgaacctggtatcattttaaagatggatgtgtcctcttccaata
attgcattcaaaaacataccgtctcgcaacttctaagaaagtaaatcgctgttgaatgcacccaccgtag
aaaaacctgttttgcaagcctaatttcgacaaatgccccacacaatagaaaagacacccccaaaagttca
tcttttcagttagctwccaatccaaggcatcaaagtactccagacacatacaggatattacaaatatcca
caaaagacatgtstcacattgwtaaatggcttttattcaagaagagaaaaggaactctttagtaataggc
actacactttcgaatggaccacggagggatacacaatgatttagtgtaatgtaacc
>KolobokP-3N1_DrPo-LTDR FRAGMENT 1 -> 474
ggttatattacactaattccgattcccatagggtcccattataaaactgacaactttcacagcatttttc
ttgcctttccgacgtatcaatttttccgaacctggtatcattttaaagatggatctgtcatcttccaata
attgcaatcaaaaacataccgtctcgcaacttctaagaaagtaaatcgctgtcgaatgaacccaccgtag
aaaaacgcgtttcggaatcctaatttcgacaaaagccccacacaatagaaaacacaccctcaaaagttca
tcttttaagttagcttccaatccaaggcatcaaagtactccagacacatacaggatattacaaataacca
caaaagacatgtctcacattgttaaatggcttatattcaagaagagaaaaggaactctttagaaataggc
actactttcgaatggaccacggagggatacacaatgatttagtgtaatgtaacc
>KolobokP-4_DrPo-LTDR FRAGMENT 1 -> 664
ggtggcaatatacagtttttttagtctcggccaatctcatacacaagcaggagttaaccactgtccgaaa
cctggtcacctcgcatatctgaaaataaaccaagcacatttccagaatggttgagggtgtgccatctgaa
aaatcagtaacattcaaatcaactgagttggggcaaaatgctttagtattgattatttcaaataaaaaca
acaaaattatgtgtgtttccgagccttttttaagccagtttcaacaacaaactgccactttcctgcggaag
caaggtgcctggagaccatgtttttacattggtaacttaccaagtactaaacagcaatggagaaaattgg
gggtcaaaggattagatttttttgtaatagttcaatgtccgtacgccctttcaaatttccaacagaaacac
tgaccggagccagcattacacctgttttcgaattgcattaattctacttcctgtatgcagctatagttac
aatacctatgttttggtaacttacaaacatccaagaacaatcaccacaactcaaacctgacattcattat

tatttagacactcaaaatggtgcttacttgagcattgttctctttattttgaaatttctcccttgccaat
gcgcattggtggctaaaaagtgtggtgtgcagcc
>KolobokP-4N1_DrPo-LTDR FRAGMENT 1 -> 664
ggtggcacactacagttttttttagtctcggccaatcgcgtacacaagcaggagcgaaccaatgtctggaa
actggtcacctcgcaaatctgaaaataaaccaagcacattgccagaatggttgaggctgtgccttctgaa
aaatcagtaacattcaaatcaaatgagttggggcaaaatgttttagtattgattatttcaaatcaaaaca
tcagaattatgtgtgtttccgagccttttttaagccagtttcaacaacaaactgccactttcctgcagaag
caaggtgcctggaaaccatgtttttacattggtaacttaccaagtactaaacagcaatggagaaaattgg
gggtcaaaggattagatttttttgtaatagttcaatgtccgtacgccctttcaaatttccaacagaaatac
tgaccggagccagcattacacctgttttcgaattgcattaattctacttcctgtatgcagctatagttaa
aataccaatgttttggtaacttacaaacatccaagaaaaatcaccacaactcaaacctgacattcattat
tattaagacactcaaaatggtgcttacttgagcaatgttctctttatttagaaatttgaccggggccaat
tcgcattggtggctaaaaagtgtggtgtgcagcc
>KolobokP-5_DrPo-LTDR FRAGMENT 1 -> 665
gggggcacactacagtttttcacaaatcggctactggctgccggggtgggggcactgtccgtcctcacag
ctaacacaacatctacacctgaaatcatacaaaacatatcttcagtattgttgggggtatgtcttccaaa
aaatacatggatagccatggtgcacccacttgctgtatatttttatcatggaaaatcagccaatttaggg
gtctgaatgtcacctgttttcgaggccatttctgtgattttcaacacaaatttaccactaaactgcaaat
gccagttcattaaacactttctgttttactgcatcactaaccagatatttgaccgacaccgaagaatcct
agggtcatccacgcggttttttgcagaaattcagttgagtggaggcacctttaaaattcatgcaaaatta
gccttcggagctagaaaccagcatgtacgtatttcatgacattgactgcccagcagacacactggctagg
cactggacacattttcgaattcactaagcatgcatttcaaaccaccaccactcaaatctgatactttaaa
cttgtaaaacactattattgctgctcagttttattgtatccctatttattagaaatttctcccctgccaa
ttcgacactaaggctcaaaactgtatagtgctacc
>KolobokP-5N1_DrPo-LTDR FRAGMENT 1 -> 665
gggggcacactacagtttttcacaaatcggctactggctgccggggtgggggcactgtccgtcctcacag
ctaacacaacgtctacacctgaaatcatacaaaacatatcttcagtattgttgggggtatgtcttccaga
aaatacatggatagccatggtgcacccacttgctgtatatttttatcatggaaaatcagccaatttaggg
gtctgaatgtcacctgttttcgaggccatttctgtgattttcaacacaaatttaccactaaactgcaaat
gccagttcattaaacactttctgctttactgcatcactaaccagatatttgaccgacaccgaagaatcct
agggtcatccacgcggttttttgcagaaattcagttgagtggaggcacctttaaaattcatgcaaaatta
gccttcggagctagaaaccagcatgtacgtatttcatgaccttgactgcccagcagacacactggctagg
cactggacacattttcgaattcactaagcatgcatttcaaaccaccaccactcaaatctgatactttaaa
cttgtaaaacactattattgctgctcagttttattgtatccctatttattagaaatttctcccctgccaa
ttcgacactaaggctcaaaactgtatagtgctacc
>KolobokP-6_DrPo-LTDR FRAGMENT 1 -> 469
ggtcacaatataccaaaagatttccttaacaaattcaatgtaaaagcaataactttaacacaaaataaag
tcaaactttgcgcgtagacaccctcataaccatacctttcttaaagagcattcaaagacaaattgatt
taagcaataaaaaagataggtcatccaatatgtaagaaagaactcaatgcgaatcagcggtcactgtttt
atggccatctttttaccggcttttctccagttgatgatggtttcccgccaactgtcaaagtctcatgtag
tctccaacctaaaagcaaaacagtgaatttgttgtgtacaacaatgttttccctaccacatgaacacaga
aatattcaaaaatacagtcatggcaagtgcccatacagagaattgtgtcttcaattccatgaagattttt
tttagagggtatagcattgaacaccaaaagtatttagtgtattgtaacc
>KolobokP-7_DrPo-LTDR FRAGMENT 1 -> 664
gggggcagtctacagtttttctggaaaatccccaggccgacaagcactggtctgcaaaacaccctcaaaa
caaggccttcagtcacaacctgaaaatatattcagaccatattcattgcggcccgtcaacagcttgaaaa
taaagcacaaaacaatgcagaaaatgctctttaaatttatatctggggtaaaatagaccttttcgggggt
ctcggaaattgcccataaaaaccccatttttacataaacccggcaatcacatttaactttccgggtaaga
atacgcacacagtcgacaacatgctttgcactatcaaagcaaatgctttcaaactaacccaaacaaaatg
ggtcattaccattgaactttttttgcactgctaacttatttaaacccaccgttgaaatctgtcaaatgccg
gcwgtttcaagcagaaagtacatgtgtttacttctcactcagcatgttgttgttgttgactaaaattcag
gtcacttagtttcgaattactaaaaacatgtgaaattactagaatattgaccatttatacttacatttca
gctcctggagtcaaaaaacaatttcttttaggccatacaccttaaattcaggaaattgaccggggccaat
gcgcagataaggctaaaaaactgtagactgcctc
>KolobokP-8_DrPo-LTDR FRAGMENT 1 -> 667
ggtagcagaacacgatagaaacacactatctccgagccgcggacgccttcttgatctccaccaagcttaa
ttcagaaaacaacgaacctgcaatcataaaaacacattcactgcacacaagtagtctgaaacttacattt
aagataaaaaaaaatagcatatggaacctgtacaggtgatactattgacaaaaattaaagaatgacaaggc
cgcaaacaggggctacaaaataaactttttaccatgtgcgaaagtggctggggacgaatgaaatggaaat
gatagcctgcttttgtaatgatatttttacacgcaaaacatagcacaacccatcaaaccatgaataaata
ccacgtaaccaggtggttttcgcgcaatacttcacagcgtgaccctactagcgattccagcttaaaaaga
acccaaaatacaatatgcctaccgcagttttccttcctgagtaacaaagaacacaacaacaacaagaggt
catgaccccacagtttcgcatttgaaaatggtcatattcttaggattttatgcaatttgggggtacaata
agacctattaccctcaaatttgacattgaaacctacccttttaccaatatttcactttttgaccgggccca
attcgcatatttctgcttcctactgtgtactgctacc
>KolobokP-8N1_DrPo-LTDR FRAGMENT 1 -> 667
ggtagcagaccacagtagacacacactctctcggagccgcggacgccttcttgatctccaccacgctcaa
ttcagaaaacaatgaacctgcaatcataaaaacacattcactgcacacaagtagtctgaaacttacattt
aagataaaaaaaagagcatatggaacctgtacaggtgatactattgacaaaagtgaaagaattgcaaggc
cgcaaacagtggcttcaaaataaacgttttaccatgtacgaaagtggctggggacgaatgaaatggaaat
gatagcctgcttttggaatgatatttttacacgcaaaacatagcacaacccatmaaaccatgaataaata
ccacgtaaccaggtggttttcgcgcaatacttcacagcgtgaccctactagcgattccagcttaaaaaga
acccaaaatacaatatgcctaccgcagttttccttcctgagtaacaaagaacacaacaacaacaasaggt
catgaccccacagtttcgcatttgaaaatggtcatattctcaggatttaatgcaatttggggatacaata
agacctattaccctcaaatttgacatggaaacctaccctttcccaatatttcactttttgaccggggcca
attcgcatatttctgcttcctaccgtgtactgctacc
>KolobokP-1_PaGe-LTDR FRAGMENT 1 -> 471

ggtgtaaccccaccattgatttttttgttctatattctatagcaaataataaaagtttgaaggcttgtca
ttcatagagagacccaaatgaaagaagtgaaacttacatattcttgtttgccaacctctgcactaccaaa
atatgatgaaaaagtaggggtcatgtgtcatataagagatttttttatgtcaagtgtaaacaccgttgaa
aaaacggaagtaaacttccaaaatattgtattcatacccccccatctcaaaaactttttttttttcttctaa
attcacaattcaaacatgacaatgcttctgaaatgttccaaatagattaacaatgccacccaaaccgaaa
aatgacaataaaaaatgtgaaaatgcaattttagagaaaaagaaactaggattatttatggcagccatca
tgcgactacctcacggaagcacttcctgttttgcaatggtggcactatgcc
>KolobokP-2_PaGe-LTDR FRAGMENT 1 -> 462
gaagctggaccaccattaatttcacatagaccgtaatattagcacamtagactgtcacacttacagttgt
actccaaactcattgagaatttccagaattcatattcaattcaagcagaacacaaaaaacagttaaaaac
atgggcattaactgcttccaatttcacaattttgctcaaagcgaaagtaggtgtccctatttcacataca
gaggtcaaatcagccaaattatgcaaaattcttaccgtctaagaaatagtcccatctgtgtgttatagct
accagtccactcaattttgcaccattctctgggaaaatatattcccttcacaaattcacattgcaatagt
acctgtcaagtgcattttcacactcacaccatcttgaatgaccactgtcaattttctatggaactacttt
cgggtactaccattttgacccccctggttcctggtctaagtcc
>KolobokP-1_PoSt-LTDR FRAGMENT 1 -> 452
ggccatagaacacaattaatttgactatatattctctacattcgtttgtcagtactgaactctcccctgt
gtgacaatcaatacaaatcatatattaatctaaaataccttcaaagatgacttcacccttaagttctcgt
caagggttgacctgcatttttttgagaggctgaattgtaaacaaaacaccatgcaatggaacactgattt
cacttccggttcatcaccaaggccttgtcactcactgagcactgccgcggcaatacacacagacaaccac
accataagctcacattctaaagattaaacaattacctttaaaccctaccaaaaatgcatcaataaaact
cttctatcaccaacataatgtgagagcaaaaaatgttctaaaaatagataagaactacattcgtgggtac
tgaactggaagtactaactgtgtaccgattcc
>KolobokP-2_PoSt-LTDR FRAGMENT 1 -> 654
ggtggcgctcaccagttattatgtacttttgagagagtgatgtacgtaaggtgaagttgtttgcattttt
aacatgcacagtggagagaatgttgccatacctctatgatactttgatgtaatgtggatgacatttcttt
ggatactgtacttgtgaaaattgtacatgatctaagcatacagtataacaaaatggatgcacccatgtgc
tcgacaaattaacctgaattagtgaaaagtacactaactttagcagttattaaaatagggaaaacgaatt
ttcctaaagtaggcaatagcagtttctgaaagtgcaatgaacaaattacaaaatatctgaaaaacaaaaa
aagttatcggctctttttttctacaacttacctctgaagtacaggaagtgaaaatgaccaagcggagcta
gcagtttttttaacatgagcacacctcaagtgagcccaagctgtatactctctaacccaaattacaccca
tcaatcatacaccaaaataaagggaattaaaagttcttttaacaacacctaagatatacaaagtctttat
tatatacccagtacaaccagcgaggaaaacctgggaaatgctttttctttaaatttttaaccctatgcta
aatacgaaactgtagagcgccacc
>KolobokP-1_MaMa-LTDR FRAGMENT 1 -> 448
ggcctcagaccacaattaattttcctatatagtctctacatttgtttgtcagtactgaactttccactgt
gtccaaatcctaataaatcatatgttgctgtaaagtatcttcaaagatgaactcacgctgaagttcttgt
aaaggttaaccggcattcttatgaatggctggactgtaaacaaagcaacgtgcaatgagtcacagatttc
acttccggatcagcaccaatgcctcattactcactgtgcactgtcgccaaaatacacacagacaaccaca
ctataatgctcatattctaaacaataaccaatttcctttaaatcaatactaagaatgactaaatctaatg
catacatcaccaccaaaatgagagaggaaaaaatgatctaaaaatagactcggactacattcgtgggtac
tgaaccggaagtattaattgtgkcctga
>KolobokP-2_MaMa-LTDR FRAGMENT 1 -> 473
ggactcacggtactaattcaactcccatagtaaacctatggaaacaccatgatattaaaaattcaataac
tttaaatttctaaatgctcatgatcaaacctatatttatcagtatagacccatccttgatgcaagcttct
gtgttaagaaaaccacatgagccgtggcatattttggatctacatgtacatgaaacatgtgttatccatt
ctgcacctaatgaatcaggtgctactgttgttattgattgttcctaagtcaagtctataaaaatgctaaa
atgcatcaatagaatcttgtctctatttttttttaggtacagataggataagctctcaaaatgattttt
actaaaatttactttcattattaaccaattatggaccaccaaacccgtatatactctattgtatcttc
acccatttctagactggttcacagttgttccatataattagtaccgtgagtcc
>KolobokP-3_MaMa-LTDR FRAGMENT 1 -> 650
ggaggcgctctacagataatatgtacttttgagaaattgacaggtgtaaggtgaagtggtttgaatttgc
aacatgaacaatgaagagaatgttggcatacctgtatgttattttgatgtactgtggctgcagtttcttt
acatactgtacttgtgtaaattggacatgatgtaaacacacagctgacaaaaatggacgctgccatgtgc
tcgaaaaatcagcctaaagtagtcgaaagtgcactattttcagcagttattaaaatagggaaacgaattt
tgccagagcagacaatgccagcctctgaaagtactatgaatgaattacaaaatatctaaaaagtaaaaaa
agttatcggctcttttttttttaatttacctttgaagtccaggaagtgaaaatgaccaagcggagctagca
gtttttggagaggtgcacatctgaagtaagaccaagctgtatactctataacccaaattgcacccatcaa
tcatacatcaaaataaagggaattaaaagttctttcaataatacctaagatatacaaggtctttttaaa
tagccaacataaccagtgaggaaaacctgggaaattatttttctttaaaaatctaaccctatgcaaagta
cgaaactggagagcgccacc
>KolobokP-4_MaMa-LTDR FRAGMENT 1 -> 461
gggatcggaccactaattcatttcccatagaacttaatgttaatatattacaaaataaaaataaattctt
cactagcttggcatacaaaccacacatgtatccattcattacagttagagaattatttttcagcctcaaa
aaacaggtgcttcgcagcacattttcgagtctcgtgatactatcagggggctccctggtctacacctaa
taacatagcctcacaccaagagtccatccgctattttgtcagtaggaaattaaatacaggatatcaaaat
aaaagtaaatatttttggtcagctatagtacatagattgaagtttgtgaaattaataagggaacaaaatt
gaacccatttattacccaatggatgcatctcaaaagatttagtactctatttttttattcccccaccagtg
gactggttccctggctataaaataattagtggtccgatacc
>KolobokP-1_MeNe-LTDR FRAGMENT 1 -> 652
gggggcgctcaccagttattctgtacttttgagagagtgacgtatgtaaggtgaagttgtttgcatttgt
aacatgcacaatggagagaatgttgccatacctctatgctactttgatgtaatgtggctgacatttcttt
ggatattgtacttgtggaaattgtacatgatctaagcacacagtataacaaaatggatgctcccatgtgc
ttgacaaattaacctgaattagtgaaaagtacactaactttagcagttattaaaatagggaaaacgaatt
ttgctaaagtaggcaatagcagcttctgaaagtgcaatgaacgaattacaaaatatctgaaaaacaaaaa
aagttatcggctctttttttctacaatttacctctgaagtccaggaagtgaaaatgaccaagcagagctag
cagtttttttatacgagcacatctgaagtgagcccaagctgtatactctagaacccaaattaccccaatc

aatcatacaccaaaataaagggaattaaaagttcttttaaagacacctaagatatacaaagtctttatta
atatagccagtataaccagtgaggaaagccggggaaattctttttctttaaaattctaaccctatgcaaa
atacgaaactgtagagcgccac
>KolobokP-2_MeNe-LTDR FRAGMENT 1 -> 452
ggccataggacacaattaattttactatatattctctacatttgtttctcagtactgaactctccactgt
gtgacaatcaatacaaatcatatattaatgtaaaataccttcaaagatgacttcacggtgaagttcttgt
caagggttgacctgcttatttttgagaggctgaattgtaaacaaaacaccatgcaatggaacacagattt
cacttccggatcatcaccaaggccttattactcactgtgcactgccgcccaaatacacacagacaaccac
accataagctcatattctcaagaataaacaattacctttaaatccctaccaaaaatgcaccaataaaact
cttctatcaccaccaaaatgagagaggaaaaaatgttctaaaaatagactagaactacattcgtgggtac
tgaactggaagtactaattgtgacccgattcc
>KolobokP-3_MeNe-LTDR FRAGMENT 1 -> 648
ggtctcactggacagtkktttkggcaaattttcatccgacgatgacctattgcagaaaaggataataata
ttacattaaagatacaaataaaaactaattaatgatttttgttgaaaggatacttgtaagattctgactg
aggtattaaggctaattccttatacaactggtggcttttatactgaaccgatactascaccaacccctct
tttttacattcattttgacagaaaagagctattttgaacattttcctgagacaaggcggctaagagaat
cagtatgtgccttatkcatswwtamagttcaccagtactataacaaattaacatacctttcwtcacccct
caaccatctagattttagatatttacctctgtagcagacacatgtaaatacgtaagccmagtcgkctgc
tcatttgcagcccaagttacgcatttgatctccaatgctagtctaactgtgacaggtagtcaccaacaag
ccaacccattttcaagcctaactcaatgtaaatgtaaaccacattacagatttagattgtgtgtgaattt
ataaatgctagggttgtgataattaggctcatgccttctaccttaaaaattagggggggtgtcaaaatacg
aaactatccagtgagacc
>KolobokP-4_MeNe-LTDR FRAGMENT 1 -> 463
ggtatcacggtaccaattgtttttccagagacctcaatgctaatgttttgtgaaataaatatactttcta
tcctacctcagcatgcatgtcacccactgtcgcagagagcaatgttagattgtcaatttgacaaggacag
aaccaggtactccattgcatttaagagagataactctgctggaactagacaggggtatgagttatctagt
cacaacagacacattttcagtcatgtgtaccacattttcaaaaaatcataaaaaatacaaaatatgatct
agataggaattaacataatctcacatgttgccaagaggctcaactttttttaagcctaacagagagtac
aactcttacataatgcaccaatcacagctagtgttgttgtttatttctgaatacaatgttccctacacct
taagctccacccacctggacttgcgcaattggtaccgttatac
>KolobokP-1_LG-LTDR FRAGMENT 1 -> 466
ggactcactccactaattcatttgccatctcttcccatgttaaaacacgaaattcaaccttctataactt
tttccagaaatctcgttgaaagctttccaatatgtcgaatgaaagcctgaccaattctcaatgtctccta
aaaaattcattatatgatgaccatgaaaattttgagctactaaaattgaaagtagactactaccggttat
actcatccgctagagtttggcacttcacaaaacgactatttttagaattagccttccgcctttaagtctc
tcatcatctcaccaaatctgaaagataatgcaatttcagacatcaaattccaaatctagaccaaatttta
cccaaaaataggcaggcaaaaaccccaaaagggagagagaaagaagatttctaaaaatagcctggactac
attcctatacaatcatttgcataaaatgttttagtggagtgagtcc
>KolobokP-1B_LG-LTDR FRAGMENT 1 -> 466
ggactcactccactaattcatttgccatctcttcccatgttaaaacacgaaattcaaccttctataactt
tttccagaaatctcgttgaaagctttccaatatgtcgaatgaaagcctgaccaattctcaatgtctccta
aaaaattcattatatgatgaccatgaaaattttgagctactaaaattgaaagtagactactaccggttat
actcatccgctagagtttggcacttcacaaaacgactatttttagaattagccttccgcctttaagtctc
tcatcatctcaccaaatctgaaagataatgcaatttcagacatcaaattccaaatctagaccaaatttta
cccaaaaataggcaggcaaaaaccccaaaagggagagagaaagaagatttctaaaaatagcctggactac
attcctatacaatcatttgcataaaatgttttagtggagtgagtcc
>KolobokP-1_PatPel-LTDR FRAGMENT 1 -> 465
ggaggcagaccaccattaatttctccattgactccaatgttaaatcagtggtgttctgcgtgctgtagcc
cattccctaaatttcgttgagaacttctctttgtagcacagcaagtatgacatcatgggatcatcagagt
actaaatttgtatgaggttgaccaccaaaatttcaattcactgaaattcctagcagactggggtcacaat
atcatgtccgtgttagtgcaaaaacaaaacaaagcctctttactcattgtattcttccgccttaaaagtc
tcaatctgacaccaaaccaggcagaaaatagaaattcaatgcctcttcatcaattctacaccaaaaagtc
aatacaaataggcatgcaaaatggcaggaaaggagggagaaaaagatatctaaaaatagccggcactaca
atttgtgggtaccgagatatggaaaattaatggtggtctgcctcc
>KolobokP-2DR_PatPel-LTDR FRAGMENT 1 -> 461
ggccacaggtcaccaattattgccccattggcttcaatgttatttcacgctcatgtcacaagctgtaact
ctttccacaatattcgttgagctaattccttcacaaggcataaagtacaacacctggcagtcatctcctc
cgaagcacgtatgaggttgaccatgaaaatctcaaatcagtaaaagttaaagtagactggggtcaattct
ttacttccggttcaatgtgagacgtctttcaagcccttttacccatgttattcttccgcataaatatcct
ctttttccacccaaactggcaaaatattaaaaattcaaaaccttcccaacaattccacatctaaacaaaa
ccccaaaaacgcctgaaaaagggagaaaaaaattaagggaaaattaggctatttttagaccagaactaca
tttgggtaaccaaagtaggaatttttttggtgacctgtgtcc
>KolobokP-3_PatPel-LTDR FRAGMENT 1 -> 465
ggactcagactagtaattatttgcccattgacttcaatgttaaatgggtgtaacatcacaggctgtaact
ttttgctcaacattccgttggttccaatctttttaggcacaatggggaccacatttggcaatcacctcat
cataaacttgtaggggctttatggtttggatttttttgctacagggatttgaagttgcctggggtgaaaat
ctcacttcctatacatttgattgctccgcagaaagacattttacaaccccatccctccgctgtaatatca
ccagtattacccaaaactgctataaaattgcaaatttgatccctttttctcctattctagaccaaaaagtc
aacccaaaaatgcaggaagaggggagaaaaaaattaagggaaaaattctctattaatagctcgcactaca
ttcgtccatcatgaaaactaggcaaataattactagtcttagtcc
>KolobokP-N1_PatPel-LTDR FRAGMENT 1 -> 462
ggaggcagaccaccattaatttccccattgacttcaatgttaattctagcctctttgcctacctccaact
ttttcccaaattttcgttgggctgaatgcttttcactatagacagaggaacaaatgaccatgtctttgca
ctaaaatttgtatgacgttgaccaccaaaatcttgagatacaccaagttgaagaagtatacccaaaacat
gtacctagttgccgtacatatcaccgtagccaacctgataactcagttatccttccgcctcaaaatccgg
aatatttaccaaaacctggcagaaaatattaattcaaaccctcttcttccattctagaccacaaaatcaa
caaaaatgtccatgcaaaggggggagaaaagagaaggagagagaattgctataaatagacgaactacaatt

tgtgggtaccgagagtgtgaaatttaatggtggtctgcctcc
>KolobokP-N2_PatPel-LTDR FRAGMENT 1 -> 462
ggaggcagaccaccattaatttccccattgacttcaatgttaattctggcctctttgcccacctccaact
tttccccaaattttcgttgggctgaatgcttttcactatagacagaggaacaaatgaccatatctttgca
ctaaaatttgtatgaggttgaccaccaaaattttgagatacaccaacttgaagaagcatacccaaaacat
gtaactagttgccgtacttatcaccgtagccaacctgataactcagttatccttccgcctcaaaatcatg
aatatttaccaaaacctggcagaaaatattaattcaaaccctcttcttccattctagaccaacaaatcaa
caaaaatgtccatgcaaaggggggagaaaagagaaggagagagaattgctataaatagacgaactacaatt
tgtgggtaccgagagtgtgaaatttaatggtggtctgcctcc
>KolobokP-N3_PatPel-LTDR FRAGMENT 1 -> 452
ggccacaggtcactaattctttccccattgactctaatgttaattctcgtctcttagaccggctctaact
ttttcccaaattttcgttgggtaaaacgcttttgccacagatggagcaacatgtggcaaactcttgccc
aaaagcatgtatgaggttgaccatgaaaaaattgagatacacaagctggaaatagggacaagaaaacatg
caatttcaggccctacatttcaacgtaaccaacctcacaactcttgtatccttccgcctcaaaatcatga
atattcaccaaaacctggcagaaaatgtattctcaaaccctttttttcgattctacaccaaaaagtcaac
aaaaatcgccatgcaaaaggggaaaaagagaaagggagagagagactaaaaataactttcgccctacat
tcgtgggtgacggcccaggaatatttggtgac
>KolobokP-N4_PatPel-LTDR FRAGMENT 1 -> 465
ggactcagactagtaattatttgcccattgacttcaatgttaaatgggtgtaacatcacaggctgtaact
ttttgctcaacattccgttggttccaatctttttaggcacaatggggaccacatttggcaatcacctcat
cataaacttgtaggggctctatggtttggatttttttgctacagggatttgaagttgcctggggtgaaaat
ctcacttcctatacatttgattactccgcagaaagacattttacaaccccatccctccgctgtaatatca
ccagtattacccaaaactgctataaaattgcaaatttgatccctttctcctattctagaccaaaaagtc
aacccaaaaatgcaggaagaggggagaaaaaaattaagggaaaaattgtctattaataactcgcactaca
ttcgtccatcatgaaaactaggcaaataattactagtcttagtcc
>KolobokP-1_GiAe-LTDR FRAGMENT 1 -> 473
ggtctcactacacagatgctatatgccattgaaacctatgttaaactgcccaacttcaaatccaaattgc
taacagaatgggttctcgttgacactaatgacatataccattttaaaggtttttttatacctttatttatt
ttggctccaaaattgacaacatttccgtctcagtttttttgctacatggcccactgtgacagtaccgtgtg
tttttaggcatggcagagtaaccggatcactccgggcacctggtcttcttcatactcatgtgtgcaaaag
taaatgcataatatttcaaaacctcatgggcaaaatgcagccagaggacctggcatctgaaataaccagt
ccctttaattcctctgcaagtactaacagtgaacatgtacaccacaacatgtgtcacaattaggaactat
agtggaccgtgatgaatgacctttcccccggaagtgaaccgtgttgtgagacc
>KolobokP-2_GiAe-LTDR FRAGMENT 1 -> 452
ggttggactataccaattaaaatcccattggtgacagtgttaagatattggcctgaaactactatacaca
acgtatcaaccaaaatttgaaccatagatatgaatactacaacacctcacccaaagtagtgactgagtta
aatggcacctttcatggctcattttcgagataacaggatgttgttacaagccccgtcaattccgcacaaa
atggtagtgcttagttttacaggcaccccgtgtatgtttcaagcacaagtgtgcctgacacactggtact
gcacaaaataaaattgcatgcatttattacctacataaaactgtacttgatgcaagcaaccactgtcaca
tttatcaccaatggcaggactcacggctttaactcctccattaaaagttcgggtcacttcgaactttgac
ccagccgagatgctatttggtggagtactacc
>KolobokP-3_GiAe-LTDR FRAGMENT 1 -> 451
ggttccattagaccaaataaattcctattgacgaccatagtaacaattcttaataaaactaacagaagca
acgtttcaaaccwccgggtcagtatatacactgacagtgtggtacctcccattgataattattcgaagaa
actatactgtcacgtatcgttttaagcacttacaggaagtttacagtacccaccgtctggtccgcacaaa
atgccagtggaaatttttattcccaccccagttactgacaagtacacaatgtcactagtgtaataattgt
tcacgtaataaaacatgtggaactgatggggcatttgtcatattatttgcacaaataaccagagtcacat
atatccaccaatcacggccatagttacggtttactccttaaacatttgacggtgcacttcgaactctgac
ctgacaatcacatgattggtacattacaacc
>KolobokP-4_GiAe-LTDR FRAGMENT 1 -> 456
ggtatctcaccactattgattatacattggtgacagtacaaatataaagccaagcactgactaaattatt
aacagtaggtgctatatactacctgacatgaagagaacagcctctgtgacaggatttaagtaaataaaat
tgatacttcgccacctgcaattttaattatccactctcctaagcacatgggctaatggaggggaggctac
tctcgttaaatatatgtaccacaggcatagcaaacaatggtcttgcttgtagctaagcagatatgcatct
gtcagtttgatatgccaaaatcaagaggcaatgttacccttccaatattccataatcatactgtcaagtc
tgttcagacaccaacataataaaataaagaaaagaggtactctttttcatggaactaaattcgtcgccta
cacgtcgctcattggctgaaatagcggtgtgatccc
>KolobokP-5_GiAe-LTDR FRAGMENT 1 -> 451
gggtgtataacaccaaataaaatcccatagacgaccatagtaacatatggggggtataacagttagatgca
acgtatcaatcaacatacacacaacatatataaatactagaacacctcacccttgaaataaatcacagaa
aatgggggtcaagctgctagtttctgagataacaggaagcgtccttcacaaccgctgtcccgcacaaaa
attgacaagcttatttttattacatcccacctatctttcaagcatcacagtatgtggtacactaacactg
gttcaaatcaaatcatagcaagtttttggtcacataagacaacactttattacaacaagtcctgtcacat
atgtcaccaatggcattcgaggaaccatgaagtgctgtatcaaaagttgggtgcacctcgaactctgacc
tggacgagatgctatttggtagactactacc
>KolobokP-6_GiAe-LTDR FRAGMENT 1 -> 462
ggcctcagaccacaattaatttcactatgtattctcatgtagccccagtggctgtagcacttttattaat
ttcaccaggccattagcatttcacgggaaacattacctgcctgggcccattgaacactcgaaaggacgac
atctgttgtccagctcttttccgcgttatatcaatgtaaacaaacacacgtgggctaagggaccgaatca
cacccatttctctgcattttacacaaattttgcatgacttcaatgtgctctgggggacattatgcagtat
ccagtgtaaaccaagtgcacatattcactaactgcatcctctcacctgtcaaacccagtgtaacaatcca
gtatactaagcagctccccagccattaatcacatcacaagaaaactatatttctcgcattaacttccgt
attttggacaaccaaatgggtggataactctagtctgaggcc
>KolobokP-7_GiAe-LTDR FRAGMENT 1 -> 459
ggtagcagagaagtaaatcataacacactgtccactatattaacataaatttaaaaaaatagaaaaagag
aattttcccaggtgaatcacacatgtagtgatgatggccagtccttgttttgtgcagcaagtatgtctga
actgggtgatgtcaacctaaatctggaaataatctcttattttggaggcaccccccatttgccatttc

acggctgctaggaagtctctccaccccgaccatgtattcatactcggtatgtacaaatcactcatggaaa
gtgagatctcattcaacacaactgtgcaccataaatgtaggtgcaattcctgtcaaagatccaatttctt
acctattttcaaactcataaattattttattcaaataggggtgtattttaaaagcactatcttcgtggg
caacacgacgctcattggtcaatttacttctctgcaacc
>KolobokP-1_HaRub-LTDR FRAGMENT 1 -> 452
ggcctcagaccacaattattttccttctttgctatctacagtgacatgccagtgcaccataattaatatc
ggtcgtaggctgttctcctgttgcatgctcaccaacctgccacttgtcacttaatcggcaaaatatgagc
ctggatcgaccatgtcgattttgctgtatccactgtaaacaatggtcaccggaagttacgagccgaaaca
caccagacagccttgaacttggggtcaaatcaaggaaggctggtgtcaataccatgagtagattgtggac
caaaaaamttttgcaataaagtacaatgtcttacctttcatttaagctatcacaagtcaccctgaaaaaa
tcagcacaagcagaacagcccatttaaacaaggcactatattctcatattttacttccgggtcatggtaa
aagttaaggggggtaactgtggtctgaggcc
>KolobokP-1_HaRuf-LTDR FRAGMENT 1 -> 452
ggcctcagaccacaattaatttccttctttgctatctacagtgacatgacagcccaccataattattatc
agtcgtaggctgttctcctgttgcatgctgaccaacctgccacttgtcacttaaaacgcaaacttacgtc
caggatccaccaccttgctttcgcagtaaccactgtcaacaatgctcaccggaagtttcgagccgaaaca
cacacagcggccttcaacttggggtcaaatcatgggaagctggaatcaataccttcacagattggggcta
acaaaaaattgacagcaaactacaatgtcttatctttcatttaagctatcacaagtcactccaaaaaaat
tgccacaggctgaacagcccatttmaacwgggtactatattctcctattttccttccggttcatggtaaa
acttagggggataactgtggtctgaggccta
>KolobokP-2_HaRuf-LTDR FRAGMENT 1 -> 448
ggttacacacaaccaaatgttttcccatagacttctatagtaacgttatagctttttaaaaaacaactag
gcattcaacagccataacatttacatatgtgcacagtctggccttaaagctgcaataaaaacacaaattt
tggctagtttacccagctccttttccgagtacaggaagctgaacacaccaaccccagttccgcaccaaat
atcacctgcttgaaaatatctgtgctcacataaagcacaagtcatcttgttcaaaaagtcagcaccaaat
attgaaaaatatgggcagttttaattactggatggatgtgcatttaaaagacataaatatcacacaatat
catccaatcacagcctgcatatgtctcttgatctgtactgaaggctccctacgaaaaccgcactgtcaca
accagttctgatttagtgtagtagaacc
>KolobokP-1_SteCin-LTDR FRAGMENT 1 -> 451
ggccacagaccacaattattttacagtagttttatcatcattgtctttctaatacagccttttccctata
tttcgttgggcgttcagcttttgcacactgacttacctgccatagaccacttttcagtcaaaaattgagc
acaccttttgcggcagcgaaggccggtagagagtgtaaacaaatatgaccccttcgtcaaggccgttcag
catgacatgaccttcaacttggggtcactttgcctgctcacaccaacactttagcagtaattccaaatcc
cagaaaaatttacacacatactagagagtcagatctttcatttgcaccatcacatgtcacaataaagtgt
ctggaagtatttttttaaaaattatccactagggactactttgtctctgaattgcttccgggttgaggtaa
aagttagtcgatgtaattgtgggctgtgtcc
>KolobokP-2_SteCin-LTDR FRAGMENT 1 -> 480
ggtctcgttatacagtttttttccccatagactccaatgttaaattgccacccgtcattggcatgtcagt
cccttacgactgaataagcccactattgaccaataccggcagtaagatgagcaaatttctgataaaaaaa
tcaattaaaaagtataccttcgccttgcaggtaatgagatacagccaattaaatatacacctacagaaaa
ccatgcaaggaaatccatgtgattttggacccggaagcgaccgaacagcttcccttgagtgggtcacttg
accttccgcaattggcgtagaaatatatgagtgttgcaggctgacatccaagtatataggctatttctaa
aagaaagacttactctaatacctcaaataacttttcttttaggggtaaaacactgtcagacctctatttg
ctatctgaactgggaactctttggcccccgttcacaatgaaaaaactgtataacgagacc
>KolobokP-3_SteCin-LTDR FRAGMENT 1 -> 451
ggcctcagtccacaattaatttctagtgcatttatcattagtgatattggtcgctacaaagctacctgac
attgcgggactgggggacttgttttgaaatgaaaatacatactctcgacactacaaaatcgcagactaaa
cttggtgttgaccatgccgattttgaaatatgaatgaaaaatgtcaatatacactgacatgtcagagaaa
gcacatgaaaatgttcaaaatatgcagaacctatgacccccaccaatccaaaaatattcactccaaattt
ttattaaaattaccactgaaaggaccacatcttacctctccatgaaatccggaaacatatgtctccaatg
taaacaaacctagaaaataatatcccacaacaccctactatttcatcatttccttctcgcccacaaccac
taccgggtcaaaataactgtgacctgaggcc
>KolobokP-1_PhoLin-LTDR FRAGMENT 1 -> 450
ggccatagaccacaattatttccaagtagttctatcaccattgtgtttctaacacagccacttccctata
tctggtaggctattgagcttttgcacactgacttacctgccatagagcacttttcagtaaaaaaatgagc
acacattttgcggctccgaagaccggtaggcagggtaaacaaatatcaccccaatgtcaaggccggtcag
tcagactcggccttcaacttggggtcacattgcctgctcacamcascaccataacagtaaattaaaaatc
tagaaaaattcacacacamactacagtatcagacctttcatttgcaccatcacatgtwacaaaatgawaa
ttacwagtcacagaaaaaaattctcaactagggcctactttgtctctgaactgcttccgggttgtggtaa
aagttaggcttggaattgtgggctgtggcc
>KolobokP-2_PhoLin-LTDR FRAGMENT 1 -> 451
ggcctcagtccacaattaatttccagtgcatttatcattagttgcatgggtcactgataagctacctgac
attgcgcgactgggggacttgttttgaagtgaaaatacatactctcgacactacaaaatcgcagactaaa
cttggtgttgaccaggccgattttgaaatatgaatgaaaactgtcaatatacactggcatgccagcaaaa
gcacatgaaactgcccaaaatatgcaaattctatgacccacacccattaaaaaatactcactcaaaaaat
ttagaaaaattacagatgaaactaccagttcttacctctcaatgaaatccagaaacatatgtctccaatg
taaacaaacctagaaaatgatatcccacaatacactaatatttcatcatttcctcctctcccacaaccac
taccgggtcaaaattactgtgacctgaggcc
>KolobokP-1_OwFu-LTDR FRAGMENT 1 -> 655
ggtaggactccaccattgatttgctgatttactatctgcaacactggaccgaatgactgtgtatcatcct
agacccgttttccaccaaactttgcaaaaccatcaccttatagataagcttcaaaaatcaagttgtcttg
gctaccgcgatctcatggtaaccatagcaaccatgcaaattccttaaaaaattggggtaaaaaaagcacc
aaacctaccaaaaaagcacttatttttcaaatatttcctgacatagaatttttttttaaacttaaatatca
tctagagccatccaagacaacaaacatatcaaaaattgagggtaggtttatcacttcaattttttgctag
ggatggggaaaggaaatctacaaaaaatgacccattttcgctttcaattcaccaattatcgcccaaattc
acatgtttttctaaggtcaaggtcacgatactaaacttagaataactacagtgtacgtacctttctgaca
ttctctgatagctctgcttcaccatgaaacaaacacaattggtctggatcagccatttccatcatgtaat

```
agatgtgaaacctgaactatgtcttaaatggatgagaaactttatgccaagcagtaaaaatgccattttc
gcactacttttggcgtaataatacc
>KolobokP-1N1_OwFu-LTDR FRAGMENT 1 -> 658
ggtatgactccaccattgttttgccgttttactacatgccacaatcaacctcactgccagcatatcaaac
caggcccattttccatcaaacttggcccaactactatcaggtggatgtccttcaaaaatcaagttggcat
ggttaccaaagcctaatagttgccatggcaaccgtagaaatggggtaaaaaatgagagcaaaaaagcacc
caaacccaccatattggcactaatttataaaatatttctagacatacaaattttcttaaactttgatatc
atctaggacacttctagacaacaaaaatatcaaaaattgggggtaggtttaccacttcgatttttttcta
ggcttggtcaaagtaaaactacgaaaaaagcactcattttcacttttgaatcacctattatcgcccaaat
ggctatatttttttcaaggtcaaggtcgccatgttaaaagtagaattacggagataaatctgctcttcaga
caatgtttgatggcttttcttcaccacggaacaaagatctttgcgctggtatgcccatttccatgagata
atatacgtgaaagaggaactatgtctttaaatgtcatcaaaactttatgccaacacgtgaaaaggccatt
tccgcactacttttggtgtaataattcc
>KolobokP-2_OwFu-LTDR FRAGMENT 1 -> 459
ggttgaagatcacagtagaaattcccataggtggccattataacattttaccctatatcttattaaatat
tagtatccaggcctagtgtaacataccacactgtccgggagagacttacctagcttgctgtaacaatatt
accatgggttgtccaccgaatttttgagataaattgatttgaagacactaaccccctctatagtcaggtt
tttcagaaaaattctggtaaaatcaccaaagtcaaaatcactccgccgccaacttaaaatgagatttccc
actcaactttgacatgtttttagataaatgaggacctgccttataaatattgagccacgattcatttacc
aaacgccagatggcgctctgatggggtagggaaggagaaaaatgacttttttatacattaaaccaaatcac
taacggtaaccgctagtatccttactcaggactatattc
>KolobokP-2N1_OwFu-LTDR FRAGMENT 1 -> 458
ggttgaagaccacagtagagtttcccataggtggccattataacattttaccctgtatcttattaacgat
tagtatccaagcctagtgcaacataccacagtgttcgggagagactcgcctagactgctataacaatctt
accatgggttgtccaccgaatttttgagataaataaacttgaagacactaaccccctctatcgtcagatt
tctcagagaattctggtaaaatcaccaaggtcaaaatcactccgccgccagcttaaaatgagatttccca
ctgaactttggcatgttttcagataaatcaggacctaccttataaatatttagtcccaattcacttccca
aacgccaaatggcgctccaatggggtaggaaaggagaaaaatgactttttatacagaaaaccaaatcact
aacggtaaccgctagtgtccttactcaggactatatcc
>KolobokP-2N2_OwFu-LTDR FRAGMENT 1 -> 455
gggtcaagattgcagtaaaaaatcccattgacgaccattataacgtttcatttcataatatattcatggg
cacagatctagctcagcgaaacagtgcccagtgttcattggagtcttgtctagcttgccgttacaaaata
agcatacggtgaccacaccgtttttgagataactgaatctggagagagctaccaccgtaaagctacctgt
ttcagaaaattattctgctctacatagtctcaaaaatcactccgccatctataaaaattctgtttctaac
caaacttgtaaatgtttcctgatagattagtacttaccctatcgagaataaaccttgattcatttatcaa
acactagatggcgctgcgacgggacagaaaaggagaaagagctttttatacagaaaaccaaataaccaac
ggtaaccgctagtatccctactccggactatattc
>KolobokP-3_OwFu-LTDR FRAGMENT 1 -> 470
ggtagaaaacaacttacttttctgacataggataacatggtgaattgacaaagtttgagagaatattaca
aaaatcctagcccatcgatttcactcatattaacagacaataaagagatatccttaaacttacctcagaa
aaagtcttgaaaaaaatatcccacgaaaatcaaaagaaccgctggattgttcacacgcgtagtgattttt
caatggaaaattaacatggcccagccaacacacccccctacgtcatcaatatttaccaaaaaataatccaa
tttttttcatgaaagttttctgctatcatccaatgcctaagagtctgtacttacttattaaccagcaaag
aggtacattgcatcctaatatgtcccatttatacaccatccaacacccaaacagttttcaaaatggacta
cttttcctatacgatgagtaaacaaaaggccacgtgagttgtattgtacc
>KolobokP-4_OwFu-LTDR FRAGMENT 1 -> 469
ggtacgagtaccgtaatctaacacgaattttgaaaattccttccaatatttgggacaaatggcgttcgtt
acctactatgaaacgcttaccccccatcatgttatatatcaaaatgtacctgtttatatagggattcgat
taatctacatcaaattatagagagtcaggttacttttcgagatatcagcgttcatttattgcaactccca
tttgctaacatttggacaatacggaaatgacgtgcacacgcgtagtaaacacgatcacgtcagaactatt
tttgacctacatcgctctttttgtacttaaaatgagagttgaactcatcctctttattttgataccaaat
aatggcatactaggtatcctagaagccatccatggccttatttgcaacaaatcgcaggtttttttaaatgg
actatataggggtcataccttttggcggatggatatggaacctcatacc
>KolobokP-4N1_OwFu-LTDR FRAGMENT 1 -> 469
ggtatgagtaccgtaatccaacaccaaattcgaaaattcataacaatactttgcacatatggcgttcgtg
accaactataaaacgcttacccccccatagtgttatatatcaaaatgtacctgttcatatagggggttcgat
tgatctacatcaaattatagggagtcaggttactttttgagatatcagcgtccatttattgtgactcgcg
tttgctaccacttcctcaattctgatatgatgggcaaacgcgtagtaagcatgctcacgtcggaactatt
tttgacctacatcgctattttcgtaattcaaatgaaagttgaactcctcctctttattttgataccaaat
agtggcatactaggtaccctagaagccaactatggcgttatttagaacaataccacgagtttttaaatgg
actatataggggtcataccttttggcggatggatatggaacctcatacc
>KolobokP-5_OwFu-LTDR FRAGMENT 1 -> 458
ggggtaactccactgaaagttcttccataggcctacatattaacatttggcttgattctgaataaactag
gattttcggtatatagtcacatataccatagtgtagctctatatgtcccctttcaaacaagaaaagaaaa
acagtagaacacccatggaattaaaaagttcttaccagttttccccgccatctattgaaaatttgcaaat
ttctcactagatttccaactctagaccaccacaacaagaactaaaatattttttgttctgccataaaatgt
cgacataatttttattaaaatatgaacatataacataaataaagtttcatgccagatctcgacttttttca
ctcataatggcactgatttacattgattttcggcaaccatcatcccaattttaaatatagatttttttgtc
ttctacctttttcacagcgccatctgtggagttatccc
>KolobokP-5N1_OwFu-LTDR FRAGMENT 1 -> 468
gggacaattccacagataaatgctgtaatgtaatccaatgtgaaaatattgaaatctttggattgattat
gaaaaaacgaggattttcggtacataaccccatataccacgggatagctccatatgtcccctttcaaatc
aaaacaaaaattgcatagaacacccattgagaaaaaaagatatgactgcttttttccgccatctatccgaa
ttttaaacaattttcaatagatttcaaactcaagaccaccgtcacaaaaagtcaaatatttgaagtgaac
cgaatcatttcgccatacttttttgtaaaattatatacacacgagataaacaaaataaaatataaaatctg
tacattttcactggtgattgcactgataaatattgatttcaatcaaccatcatcccgattttaaatttag
atttttgtcttctacctttcactagcgccatctgtggagttatccc
```

```
>KolobokP-1_ParEch-LTDR FRAGMENT 1 -> 454
ggggcgagaccacattagatttccccataggcggccattataacgtttcctacatttcctgtaaatttta
cccaaaagttttctttaaatttctacaccatatttaccctctgttcatacacatgctgtgcaaaattcgt
aaaattctcatacctcaattttccataaaccactttcaaacctgacacccccttcatttctgctagaacca
gacagtgatttgccctaatgacatcatacagccaaatgacacctgtgtctatacgcagaaaagcacaatt
tgacaactgtcataatttgacagctctggtaccacgctgcagaaaaacataatttctactcaatcaacac
tatacaagtactctcagaaattcacacaacctgcataatgccatttctattgggaactctctcggagggt
aatggcggacgaatccttaatgtggtcttacccc
>KolobokP-2_ParEch-LTDR FRAGMENT 1 -> 454
gggctcagcacactgtcagttttcccattggcgaccatgttaacgttttgtgtgattataagaaaacgtg
acctataaagtttcttaaacttctcacctgctgttattatcatctcattgacatgctggtcgagtttagt
caaaatctaatatctctagctcttgcaaaatgcctgtatgtatgacacccgtcaatatttacaactggat
tcaggcagtaatccggctaaagtgtcaactagccaaattgctgccagatacccttccgcttagtaaaatc
agctaaaaagacacaattttactgtcaacagcttctacaatcacatctagaaatctgactactcaaatgc
acttatgtcagagcaagtgaacattgtaaaaaatcatttctcaaaaatatactgcactttttttcgtctg
taatcaagcaggaagtccaacagtgtgctgaccc
>KolobokP-3_ParEch-LTDR FRAGMENT 1 -> 451
ggggtcagcacactgttggttttcccataggcgaccatgttaacgttttgtctgattataagaaaacgtg
acctataaaatgtcttaaacttctcacctgctgttatcatcatctcattgacatgctgatggattaaaaa
aattgcatacttccagctctcgtaaaatgcctgtatgtgtgacacccgtcaatatttacaaatgccggca
agaattactccgcaaacaggtcacacaggcaaattgctgccaaatatccttccgcttattcaaatcatct
agaaatccaccattttacagtcaacaccttctacaatcacacatgaaaatctccctactcaaatgcactt
atgtcacagcatgtgaattttggaaaaaaatcatttctcaaaaatacactgcacttttcttcgtgtgcaa
tgaacaggaagtctaactagaacatgacccc
>KolobokP-N1_ParEch-LTDR FRAGMENT 1 -> 455
gggctcagcacactgtcagttttcccattggcgaccatgttaatgttttgtgtgattataagaaaacgtg
acctataaagtttcttaaacttcccacctgctgttattatcatctcattgacatgctggtcgattttagt
caaaatctaatatctctagctcttgcaaaatgcctgtatgtatgacacccgtcaatatttacaaatgcat
gcaggcagtaatccggctaaagtgtcaactagccaaattgctgccagatacccttccgcttagtaaaatc
agctaaaaagacaccattttactgtcaacagctkctacaatcacatctagaaatctgactactcaaatgc
acttatgtcagagcaagtgaacattgtaaaaaatcatttctcaaaaatatamtgcactttttttcgtctg
taatcaagcaggaagtccaacagtgtgctgacccc
>KolobokP-N2_ParEch-LTDR FRAGMENT 1 -> 455
ggggtaagaccacatttaaattttacataggcggccattataacgtttccttcatttcctgctaaatttg
acgaacaattttcttgaaaatttcacagcatatttaacctccattgacacaagtgctgtggaaaactcgt
aaaattctcatacctcagttttcagaaaaatccacccaaacctgacacccctcaatttcgccccaatctg
gtgacggattttgaaatattggtgcgttctgcaaaatagccactgtgcgaatacgcgagcgactagcgta
tgtttattgctacctcgtgaaagatctaatgtagtagtacattcagtattcactggactgacatgtgcca
tgccaaagtgctcgaacaaaattcacacatgacaccaaaacggtattttcaagggcactctttcggtggg
taatggcggacgaatccttaatgtggtcttacccc
>KolobokP-1_LiLo-LTDR FRAGMENT 1 -> 444
gggacaacaccggtgattatttgcaatgcattttctattaaacttaaaaatctgccaaaaataggaaacc
gtgtttttttacaaaccgccacaaggggttaatatccaaggtctgggacatccctcactgcaatggcgta
tagggttcaccacgaaaatctagagaaaatcctatttttgtaaaacagttctcagattctcactttccaa
gctgcttgtctccagaaatgaagcccttactggtgctgccctcctgaaaatacccgggggaccatagaac
tgaaatcaatattttatgaaagattgaatactactctataattcaaatcaatatttatcttgattacctt
ggtattaacccacgccatcccagggtcaactttttgtttttattcttcaataatttgacgcccataccga
tgtaggtgtcactggtgttgtgcc
>KolobokP-2_LiLo-LTDR FRAGMENT 1 -> 432
gagacaagaccacaattgattttccaagccatttccagttatttaaaatgcccgccatcaaagaatgcgt
ggagctgtgaaactgaaattcggatatgatatacgggttagtcttgcgagtaactggtgcgatggaaatt
ggtgattgaccacggaaatttttttttaaaattgacaagtttgacacaagtggacatttttacccatttcg
gccattttcactccaaatttgaaccacccccccctatggccaggttgtcaaaattcaaaattattttttt
accaaacaatttatttatatctgtagacttgccacagcaaatatttttttcaatacctctccaaatatgta
cttgagagtaaaaaacatgcactcgtctaatagataggcctggaccctccaacctatgaatattcattag
tggtcttgtctc
>KolobokP-3_LiLo-LTDR FRAGMENT 1 -> 467
ggccagagtcctctattaaaccttgtgtacattacatgtacattgttgtgcatatgttttggtaaaaaaa
gtacaccttggaccaatcaatctgcacaaaattttatacatgtcaagaagaaccctttgccaatagcata
ataaagtttaaaaaaattccaataccgattgcctgagaaaaaaagatttccgtacaccatgtccatcaaa
acgtcactggtgcattgatatggccctgtcaaagtacaagttctaaactgaccagtgagaccacattttc
ttaaatatattatcaaaaagcatatttctgtgatggcctgactctgtagatttagaatcaaccacagatt
gagaattaattccactttggtccatcccagccatgtatttaccacaacttgacattttgataagctctat
gtgactgtgccacacttccggtcgcggatgaatacaggtctctgccc
>KolobokP-3N1_LiLo-LTDR FRAGMENT 1 -> 467
ggccagagtcctctattaaaacatgtgtacattacgtgtacattgttgtgcataggttttggtaaaaaaa
gtactcattggaccaatcaatctgcacaaaattttatatgtgtcaagaagaaccctttgccaatagcata
ataaagttttaaaaaattccaataccgattgcctgagaaaaaaagatttccgtacacaatatccatcaaa
acgtcactggcgcattgatatggccctgtcaaagtacaagttctaaactgaccagtgagaccacattttc
ttaaatatattatcaaaaagcatatttctgtgatggcctgactctgtagattaagaatcaaccacagatt
gagaattaattccactttggtccatcccagccatgtatttaccacaacttgacattttgataagctctat
gtgactgtgccacacttccggtcgtggatgaatacaggtctctgccc
>KolobokP-4DR_LiLo-LTDR FRAGMENT 1 -> 467
ggccagagacctctattagccagcgtgtacattctgtgtacattttaatgaatataggttggtgaaaaaa
gtacaccaagggtgctttaattcccaccaaattttatatgtgttatgaagaggcttttatcgaaataata
gtaaaaattcaaaacattccaataccgattgcctgagaaaaattgatttgtgtacagcattgccatcaaa
tcgtcactcgcggactgatatgggcctattagaatacaagttctaaactggccagtgagacaatatttcc
```

ttaaattaattatcaaaaagtatatccctgttatggcctggctctgtagattaagaatccaccaaagatt
gaagaataaatccactttcacccatcacagtcatgtatttaccacagcttcacagttcagtacggactat
gtgtcagtgccacacttccgcctgtggatgaatacatgtctctgccc
>KolobokP-5DR_LiLo-LTDR FRAGMENT 1 -> 446
ggcacaacaccggtatcgtttttaagtcattttccagttgtttcaaaaatcagcgaaaaatatcaaatc
cgggtttcaaaccaaaccgctttaggggtcaatatacatcatccgggccacttaccctgaaaattttgc
gtggggttcaccacggaaattttgagaaatattgatttttaaaaatgtaccctcagatttcacacgctca
gtcaaactgtctactggtacagaagctctcctctgtgctgccccctggcattagttggacgaaacccctg
cccaaaatgaattttattctaaagcttgaatccagtttaatatttcaaatccctatttttctacttcagc
taatcattaacccccttctattccagtgtctaaattatgtttttgatattcaacaatttgacacccattcc
attacaggtggctctggtgttgtgcc
>KolobokP-N1_LiLo-LTDR FRAGMENT 1 -> 444
gagacaagaccacaattgatttttttaagccttttagcagttaaattgtcaatgtttgaccgcgacgcatc
aaagaatgcgtggtgttgtgaatctgaaatttagattatgttattccggacagtcgggcaagtaaatggt
gaaaaataaaatggtgattgaccacggaaatttttttgataatcgacaaattagacagactttgatatttc
cactcttttcagccaactggcagaaaaaactcaaaacacgcccccctattacccaattagcaaaattcgaa
attaatttttcatcaaataatttctttatatctgtagacttgccacaacaaatatttttttcaatacctc
tccaaatatgtacttgagagttaaaaacatgcactcgtctaattgataggcctcgaccctccaacctatg
aatattcattagtggtcttgtctc
>KolobokP-1_Memb-LTDR FRAGMENT 1 -> 469
ggtccaacacctctatttatttccccattgctttacatgcaaattatgttgacttctcacctccaaaagc
ctgcttcagcttctttcaattctttaaccagaccactcaaaacgatggtctgcttctgctctgacatact
tgagagtattgaaagaaaaacggttgtcaaatttttttggcgctcaatcaaacggagcaagtgcattttt
acaggttttccagccaattttgcctcaactgacttgtctgcataaagcgccccaaattgattttgctaaa
attatttaaaacctcaaatcaccaaaatttcaattgcaaatacaacaggcaacattttcatgcctcagta
ttactgactgcagtactatcaacttgcctgggtcctgatttgtcttcagcatactagtcattttattttg
gaggatgggtgcaacaagttgaccttaacctaattacaggtgtattacc
>KolobokP-1_PhoAus-LTDR FRAGMENT 1 -> 660
ggaggcacagaacagttctcactgggttttgcctagcaaaacacacatcacttgatttgtaacctttttct
tcactctgtcaaaaacaggatgcaaagaatggcatgttttcaattatttcacataaaaagcccagcagat
gagcacagaaactttattctttcttgtatttttgaccatttcattcccactgaaaaccaagctagactaa
gaaaaataagggcaaaaaaatgcaaaatcggcacttttcacccgaaaaacaccacttccctgccgatgca
cgactaaaaccaacattagagtctgaaagcaaatttcctaccgattcmgaaaatgtggtccaaaagtact
gtcaaccaactagtttttttggcaattcttgcttgtttgagggctacctaaaatcacagccaatttctcgg
tcgctacccaacaatgcacaattgcagccgggggccgccatcttgaagaagagtgcagctacagccaaga
aagtggcctttttttattcacagcatgttcacaaatacaaacaaaccacattcactgagcctacgtgcaat
gcccacctacaaaatgcccccaaacttggcctaccccaaaatttctatattttggcagtgcaaattggcc
gcggccgcagcctaactattcagtgccacc
>KolobokP-1BDR_PhoAus-LTDR FRAGMENT 1 -> 661
ggaggcactgaacagttctcacagggatttgcctatcagaacacacgtcaatggactcttcaccctttct
tcactctgtcaaaaactgcatgcaaataaagaaaagttttgagtcatttcacatcacaagccctacagat
gagcacagaaactttgtactctcttgtatttttgaccctttcactcccagtggaaaccaagctgaactaa
gaaaaataagggcaaaaaatgcaaaatcggcacttttcaccctaaaatcaccacttccctgccgatgctc
gactaaagccaacattagattgcgaaagcaaatttcctaccgattccaaaaatgtggtacaaaagtactg
tcaaccaactagtttttttttgcaaatctggcttgtttgagggctacctgaaatcgcggccaatttctcag
ttgctacacagcaatgcacaattgcaacagggggccaccatcttgaagaagagtgcagctacagccgaga
aagtggcctttttttcattctcagcatgttcacaaatacaaacaaaccacattcactgagcctacatgcaa
tgcccacctacaaaatgcccccaaacttggcctaccccaaaattttaatattttggcagtgcaaattggc
cgcggccgcagcctaactattcagtgccacc
>KolobokP-1CDR_PhoAus-LTDR FRAGMENT 1 -> 633
ggaggcacagaacagttctcacaggggtttgcctatcagaacacacgtcaatggactcttcaccctttct
tcactctgtcaaaaactgcatgcaaataaagaaaagttttgagtcatttcacatcacaagccctacagat
gagcacagaaactttattctctcttgtatttttgaccctttcactcccactgaaaaccaagctagactaa
gaaaaataaggacaaaaaatgcaaaatcggcacttttcaccccaaaaccaccacttccctgccgatgctc
gactaaagccaacatattcaaaaatgtggtccaaaagtactgtcaaccaactagtttttttgcaaatctg
gcttgtttgagggctacctgaaatcgcggccaatttctcagttgctacacagcaatgcacaattgcaaca
gggggccaccatcttgaagaagagtgcagctacagccgagaaagtgtggcctttttttattctcagcatgt
tcacaaatacaaacaaaccacattcacttggcctacatgcaatacctaactaaaaaatgcccccaagcct
ggcctaccccctagattttaatattttgggagtgcaaattggccgcggccgcagcctaactgttcagtgcc
acc
>KolobokP-1F_PhoAus-LTDR FRAGMENT 1 -> 652
ggaggcacagaacagttctcactgctgggcaaaacccacatcacttgatttgtcaccttttcttcactct
gtcaaaaacaggatgcaaagaatgacatgttttcagttatttcacataaaaagccctgcagatgagcgca
gaaactttattctctcttgtatttttgaccctttcaatcccactgaaaaccaagctagactaagaaaaat
aggggcaaaaaatgcaaaatcggcacttttcaccccaaaaacaccacttccctgccgatgcacgactaaa
accaacaatagattctgaaagcaaatttcctacagattccgaaaatgtggtccaaaagtactgtcaacca
actagtttttggcaattctggcttgtttgagggctacctaaaatcgcagccaatttctcagtcgctacc
cagcaatgcacaattgcaaccgggggccgccatcttgaagaagagtgcagctacagccgagaaagtggcc
tttttattctcagcatgttcacaaatgcaaacaaaccacattcactgagcctacgtgcaatgcccacct
acaaaatgcccccaaacttggcctaccccaaaattttaatattttgggagtgcaaattggccgcggccgc
agcctaactattcagtgccacc
>KolobokP-1_BBe-LTDR FRAGMENT 1 -> 459
ggcacagcctagaaatagaaaataggcatctaatacatgtaaatcatgacagaaaagcggcatcaaaagt
cacctacctgagttttaggccatccatatcacacattgactgggtagcacatcatctaaactgtccattt
agcccaaaatttcaaaatcctacgcacctaaacatcgttaaaatccttgtttacaatgtagggtgacctc
tgacctcccatggacagcacagcaatgcatgtaaacatatatcttcttttctttgctggacatctcaaac
atggctgaaagtctgttttctgaacaaaataaaaattcaagaacccttacctgtaacatccagctccaaa

aaggcaacatttcccacaaaaaagactttttacatttggttttgtattgccaatctctatatactaaaac
tggccccaatacacatgcgcggattcctaacctttgccc
>KolobokP-2_BBe-LTDR FRAGMENT 1 -> 476
ggtagcagaacacagttttcgcgctatggggatttctatggttaaggactgcaaggtggcccacttcaac
gcggaaaaaattataatagggtgcacttcagaagtacgaaaaacgaatatgtttgagatgaaggtataac
ctacaaaatatctaaaaacaaaaaaatcctgtcggcgtattgtcttgtgacgccctctttgaaatcgccc
tctagcggggtcaagtagactgcagccggatcactcggaaggccagttgcgtaatctggcgtatttatac
acgaacgggaaaaactacacaatccaaatcatacagtctgacagccgacatggttcggtacctcacactg
tatttgacatttggctgcctctagcacaaaacctgctacccagtcttcaagaagtttgtacgcactcttt
tctatacccgcgtcatagaccccgggggtcgcccattaatactgtgttctgctacc
>KolobokP-3_BBe-LTDR FRAGMENT 1 -> 481
gggcagacagtactattagatcttgccataggaacacatgcttaaatacactatttcaacacctcatgaa
agtacttacccacactccaatctcaaaaattcttctgcaccaggtagccacaagggtctgcttcccacat
atagaaagaaaacagggggtcgaccacttcaaattcccaccacagctgatgatagtaaacaaacctcaac
tttgacctactttttccttggtatccatccgcctggccagacaagacctaaccactcacatctctcacta
cgcccactaaactggtgaaataaacaaaacttgaccaatgtaggaaagtttagaccctacctgtgccttc
cagcccaaaaatctccaaaatggaccaaaattttcaagagaaaaaaggattttaaaaggtattaactagg
cactacaatcgtgttcccatttttttggcctccactgcgcagcgcaattgtgacgtctgccc
>KolobokP-1_BJ-LTDR FRAGMENT 1 -> 475
gggggcacaccccagtttctcggccccattgaaacctatagttaagacacgtttttcatccacgccgtac
ttgaagaaaaaaatttcccgcataccacatctatataggaaggaaacatgtaccatagtctctaacata
tctgattttwaaaaaaattctagtactgcagttatcacaataatggattttcggaggtatgtcgagcgcc
catagctgtgacgtcatcggcctacccgctcggggcgcttcactgtttgccgcctcggagcgatacccgg
aagcgaagattcgccgacaaaaacatatccaaaacagcctataggggtcgaactttatacagacatcgac
ctacatccatcacatggattttactctaagtaactaggcacagagcggacactaaaaaacccttactcca
aaataagatgggactaccacggaccccggtcgcggaagaactggggtgtgcaccg
>KolobokP-2_BJ-LTDR FRAGMENT 1 -> 476
ggtggcagaacacagtttccgccctatggggatttctatagttaaggactccaaggtgactcacttcgac
gcagtaaaaattatagtagggtgcactagaggaacacgaccaactaatatgttgaaatatacggtataat
ctacaaaataactgaaaacaaaaaaagttatcgtttttttaacattttgacgccctcttttatttcgccta
ctgcggaggtcaacggaactgcagccgactcaccctggcaggcagctgcgtaagtcacgcgcatttatcc
actgaaagagaaaacttcacaatcctactcatgcgatcgaaaagtcgactcattatactaccatacagta
aagctcatgcctcgctggctttcatgttaaagtgtttacaaacgctacaagatttcgtccgcactatttt
ttgtaccctacgtaatagactccgggggtcgcggaaaattactgtgttctgcgacc
>KolobokP-4_BJ-LTDR FRAGMENT 1 -> 461
gaggggacagcccaccattccaccgtcccattacaatacatagttaagataccaaaaacaagctatcaaa
aaccacctacctgaatgttcaactttggaaaagtgacattggcagggtagtccaacctccaaattgtcca
tttagtaaataaatcccaacttttattcggcgaaaacaggttggatacatgtaaacagaacttggatgac
ctttgacccccctccacggcatcaaaacaaaacaacattgcatgacttttagagctctaccaggcaggcaa
aacaagcttaaaagccagtttttttcaacaaaatacaaaaagcaataactctccagctggaaaatgccagc
atttatccacacataccaagaaaaaaggctgttttcggttgatttggatgctatcttcaaagggtactca
gaaagccatctatgcgcctgcgtgaatagtaacctttgccc
>KolobokP-5_BJ-LTDR FRAGMENT 1 -> 478
ggggagggaccagcaaatccccamaccacacgtttcctatacaaaaacaggcatattcaaggcttcaaaa
tacacttaccattaatttcaaactccgaaagctcctctccactggatagctaaacatgtctacttaccgt
ttgtggaaaaacccacgcttcgactccggcgttttaaagagctacagctgatttcttgtaaacaaccact
gacctttgaacctccagcttggactcgcctggctgaggggtgaaaacatactcccaaaagatgtaatata
acaccactatacaacactgaggtcacaacttctgtctagatttgaaaagtaaacatacctcttgtatacc
tggcagaaaactggcacaatctgaccaaaattggccccaggaaaaaattctataaaataataacagaaaa
ataatcgtgggtgtacaaaccagcctctctgcgcaggcccagtttctggcccctcccc
>KolobokP-6DR_BJ-LTDR FRAGMENT 1 -> 476
ggtggcagaacacagtttccgccctatggggatttctatagttaaggactcgaaggtgactcacttcgac
gcggtaaaaattatagtagggtgcactagagggacacgaccaactaatatgctgaaatttacggtataat
ctacaaaataactgaaaacaagaaaaagttatcgtttttttaacaatttgctgacctcttttatatcgcct
cctgcggaggtcaactgaactgcaggcggctcgccctggaacgctgttgcgtaatgcggcgcatttatac
acgaacaggaaaaacttcacaatccaaatcctacagtctaaaatctgacatgtttctataccacacagcg
tagtttactcatggctacctctcacataagccgttctaaccaggcttcaagaatttcgtccgcactactt
tgtatacccacgtcatagactccgggggtcgcccaagaatactgtgttctgcgacc
>KolobokP-7DR_BJ-LTDR FRAGMENT 1 -> 478
ggtagcagtacacagttttcgccctatggggatttctatagttaagaagcccaaagtgagaaggttcgac
gctgcaaaatttatagtagggtgcacaagcaagctacaaaaatttagtatgtttgggttgagggtacaac
ctacaaaatatgtgaaaatcagctcaagtttgtcggctcgttcctttgtaaaacccacttttaaaagcgc
tatgcgggggtcatgtgaactgcagccgacgcacagtactaggcggtaacaagatccaggcgccagacat
acgccggccgaacaaacatcataattcaaattatacggtcggaaacctgacgcattcctctagcattcag
cacagttttcacctcgctgtcgtatacagaagaatctctgcagctttctctagtattgtgccgcactact
ttgtgcccgtactacttttgactacggggttcgcccatgaatactgtgtactgcgacc
>KolobokP-8DR_BJ-LTDR FRAGMENT 1 -> 452
gggcacagcccaccatttcgagctcccattgaaaagtatagtaaacatctaagaaatgagctgtcaaaaa
tcacctccttgagtgttcaactattgaaaactcacattgacaggatagcttggcctccaaattgtccatt
tagcccaaaattcttattttttgaatcggctcaaactccttgtacgatggccgcaatgacctctgaccccc
ctgaacaccactaaacacaacaacatagcatcacttctagagctctacccagcatcaaaaacaagcctga
aggccagtttttgcaaggaaattaaaaaagaaagaaaacccaccagctgcaaaataccggcaaatagcaa
gatatgccaagaaaaaggctgtttacagttggtttggatgctctcaccaatggtcactcaaaatggcctt
tctgcgcgtgctcgtaatggtgacctttgccc
>KolobokP-9DR_BJ-LTDR FRAGMENT 1 -> 477
ggtagcagtacacagttttcgccctatggggatttctatagttaagaagcccaaattgggaaggttcgac
gctacaaaatctatagtagggtgcacaagcaagctacaaaaatttagtatgtttgggttgagggtacaac

```
ctacaaaatatgtgaaaatcagctcaagtttgtcggctcgttcctttgtaaaacccacttttaaaagcgc
tatgcggaggtcaattgaactgcagccgactcacccggaaagccagttgcgtaaatacggcgcatttata
cacgtactgcaaaaccttcacaattcaaatcacacagtctcaaacccgatacattcctataccacacagc
acaatttacatctgactggcgctaacatcaggctttctacccaggcttcaagaatattctccgtactatt
ttgtatacccgcgtcatagaccacgggggtcgcccatgaatactgtgttctgctacc
>KolobokP-10DR_BJ-LTDR FRAGMENT 1 -> 458
gggcacagcccactatttccaggtcccattagaatacatagttaagataccaaaaacaagctatcaaaaa
ccacctacctgagtgttcaactatggaaaagtgacattggcagggtagtccaacctccaaattgtccatt
tagtaaataaatcccaatttttattcggcggaaacaggtagaatggatgtaaacagaacttggatgacct
ttgacccctccacagcaagaaaacacaacaacattgcatgacttttagagtgctaccaggcaggcaaaa
caagcctaaaagccagtttttcaacaaaatagaaagagcaataactctccagctggaaaatgccagcat
ttatccacacataccaagaaaaaggctgttttcggttggtttggatgctgtcaccaaagggtactcagaa
ggccatctaggcgcctgcgtgaatagtgacctttgccc
>KolobokP-11DR_BJ-LTDR FRAGMENT 1 -> 478
ggtagcagaacacacttatctcccaatgggcattcgtatggttaaggaccccaaggtgactcacttcgac
taggtgaaaattatagtagggtgcactagagaagcacgacaaacttattcattcaaaattacggtataat
ctacaaaattcatgggaaaacaaaaaaagttatggtttttaacaatttgacgaccactttgatataccc
ctctgcggaggtcaacggaactgcagccgtctcacccaggcggtcagctgcatgcatcgcgcgcatttat
acactgaacgggcagacttcacaattctaatcatacgatctaaaagccgacttatcatactacaatattg
ttgagttcatgcctccctgcctttcacgttaacgcctttacaaacgcttcaagattgcgtccgcactacg
tttttacccctacgtcacagaggacgggggtcgcggaagaatactgtgttctgctacc
>KolobokP-N1_BJ-LTDR FRAGMENT 1 -> 448
ggccacagcccactatttccaggtcccattgaaaagcatagtaaacatctaagaaatgagctgtcaaaga
tcacctttttgagtgttcaactattgaaaactcacattgacaggatagcttggcctccaaattgtccatt
tagtaaaaaaatccaaaatttattcagcgaaataacgtctgattgacgtctgatgacctttgacccccct
ccacagcaccaaaacacaacaacattgcatgacttttagagctctaccaggcaggcaaaacaagcctaaa
agccagtttttcaacaaaacagaaacagcaataactctccagctggaaaatgccagcatttatccacac
ataccaagaaaaaggctgttttcggttggtttggatgctgtcaccaaagggtactcaaaaggccatctat
gcgcctgcgtgaatagtaacctttgccc
>KolobokP-N2_BJ-LTDR FRAGMENT 1 -> 661
gggggcacaccccagtttttttcgggcttccattcsagtctgcacaccctcgagtctagcgaccgtctct
tccagggaataattcggaagccaaataactgtatatgggatggaaggatatcccttcagctacaagactg
actgagttttgtttcttcaaactctttaattttgtgaaattaacagaaaaatcatgatttttatgccaa
aattaatgcacaaaattgggacttttcgcgtaggataaaccaggacaaaaagaattttccggcagaaacg
actggtgttggcagaaagatacaaacctcgagtatacttgattcaattataaagaaaatcctagtactgc
agtttttgtaatcatccwcggcgcgaggggtcccaaatccgcccacaccacgcataagtggagaatctgg
gtcaaatgggcggatcacgctcggggcgcaagaatacccggaagtgaggatccaggccgaaatcattctt
caccccaaaaacaccacttcttatacttttcagccatattagtatctggtatcatttcacagcagattag
gaaaaatgtgaactcaaacttgaccactcaaaaccctttcgtcactttttgaccgcggactaccacggac
cccggtcgcggaagaactggggtgtgcaccg
>KolobokP-N3_BJ-LTDR FRAGMENT 1 -> 661
gggggcacaccccagtttattttgggactcaattcgattctgcacaccctcaagtctagcgaccgtctct
ttcaggaaataattttgaagaaaaaacactgtgtatggaaatgaaggatatctctctagctacaataaca
acagaatttcactttctttaacctttaaattttgataaattttagggggaaaaggattttcctatgaa
aaatcatgcgaaaaattggccattttcacttggaattaaacccatcacccataatttagctacccaaacg
actgatattgtcggaaagagaaaacctttgtgtataagcaggaaaagtataaaggaaatcctaacactga
gattttcgcaatacctcgtcgcgcgaggcatagcaagagcacccacagaacgcatgaaacgtaaatctgg
gtcaaatcgccgcaccacattcaaggcggagggatacccggaagtaagcctcggatccaaaagcaaattt
cacccaaaacacaccacttcttatacttttcagccatcttactagttaatgccatttcgcagcagattat
gagaaatgtgaactcaaacctgaaccctcgaaaccctttcgtcaatttttttaccgcgcactaccacggac
ccctgtcgcggaagaattggtgcgtgcaccg
>KolobokP-N4_BJ-LTDR FRAGMENT 1 -> 459
gggcacagcctactaattggctttcctatgttaatatatgggaattatgtcagaactaagtggtcaaaag
tcagatacctgagttttagaccttgaaaaacacacattggcagggcacaccaacctctcaagtgtccaaa
actccaaaatcagaattttctatacagtggaacctgtcggaacccttgcccgaacctccaaaagtgacat
ttgacccctctggacagctgatgaccgcatggaaacctatatcttctctagtgaactagacaacacaaac
atggctcaaagtctgtttttttcaacaaaataaaaacactacaaccctaacctataacatacacctccaaa
caaacaatatttcccaagaaaaaggcttctttacatttgctttcgtatgccatctgtatagactagaact
gggccctctgcgcctgcgtgtaattacctgcctttgccc
>KolobokP-1_BF-LTDR FRAGMENT 1 -> 459
ggggagacgcaggccaagccttggcacattgaaatgtgtacataaaggcacagaaagacagtgccaataa
ctcaactttctgacattactggcatacaaacttaccctcatatgaaagcccatcatctcagctgtgataa
aatcccactgtcattagaaatcatacggcataatttgcagaaaaatacgcccaaaccctaccaccccctc
aaactcataacaaccaaggaaacagacccttacacaaccaggaaatagtcccaacttcatatcccctgat
aaccacaaaaactcagcttttgggccataatcagaaggtagaaaccctccccagtcacattgacctcaaa
actgccagaatttctacccatttttccaagaaaaaaaatcttaaagttttaggccctgaactacattcga
ccaccatcaatttgcatgccgaatcgctggcggatcccc
>KolobokP-2_BF-LTDR FRAGMENT 1 -> 468
ggctacaatacggcatttcctatgcatatgcttaacaacagggatcatgggagaaagaggctgtcaaaaa
tcacctaccggacttttagaacatgaaaaactcacattggcatggttgtcagccctctaaagattaattt
gacggaaaaaaaaaggaaaaatttacggtggaatcatgtaatacgatatcaaaacataggtggtggcctt
ttcagcccttgtgactgcctgaaaacaacaccaaaacacctaacttctaaagcctactgcatccacaaat
attgctacaaatctggtttttcaacacaatatagaagctacaacccttacctataagatgcacctccaaa
atccaaaaatatcccaaggaaaacatttctacagccacttttcccaagcctacctaagcattctgagggc
actaaaactgggacatctgcgcaagtccggaaataccgaaatttgccc
>KolobokP-2N1_BF-LTDR FRAGMENT 1 -> 469
ggctaaatttcaatatttcctatgcatattcttaacaatgggaatcatgggagaaagaggctgtcaaaaa
```

```
tcacctacctgacttttagaacatgaaaaactcacattggcaaggttgtccgacctctgaagagtaattt
aacgacaaaaaagggaaaaaatttacggcggaattatattatacgccatgaaaacataggtgggagacct
tttcacccccttttcactgcatgtaaacaacaccaaaacacctaacttctaaggcctactgcaaccacaaa
tatcactaaaaatctgtttttttcaacacaatatagaagctacaacccttgcctataagatgcacccccaa
aatccaaacatatcccaagaaaaacaattttacagccacttttcccaagcctacctaagcattctgaggg
cactaaaactgggtcatctgcgcaagtccggaaatactgacatttgccc
>KolobokP-3_BF-LTDR FRAGMENT 1 -> 476
ggtagcagaacacagtttttctcctatggggatttctatagttaaggaccccaaagtgaggtggttcgac
gactcaaaagatatatcagggtgcaaaagtaattaacaagaattcaatatgttggagttcagggtagaat
ctacaacatatgtgaaaatgagcataagtttgtcggctcgttttcccagaaagaacactttgaaatgacc
cccagcggaggtcaccatactgcagccgacacacggaagtagcgggaaccggattcgtgcgcgaaattct
acccagccaaaaaagcatcgtaacccaactcatacagcctcaaaacttacgtattcctctactatccacc
acagtttccgactcgctgacgtgcacagaaaaatttctatggcttttcaagaactgcgacgacctatttt
gtgcccggcctatttatgtccacgggggtcgcccataaatactgtgttatgcgacc
>KolobokP-3N1_BF-LTDR FRAGMENT 1 -> 476
ggtagcagaacacagtttttcgcctatggggatttctatagttaaggaccccaaagtgaggtggttcgac
gactcaaaaaatatatcagggtgcaaaagtaattaacaagaattcgatatgttggagttgagggtataat
ctacaacatatktgaaaatgagatgaagtttgcckkctcgttttcccagaaagaacactttgaaatgacc
cccagcggaggtcaccatactgcagccgacacacggaagtagcgggaaccggattcgtgcgcgaaattct
acccagccaaacaaacatcgtaacccaactcatacggcctcaaaacttacacattcctctactatccacc
acagtttccgactcgctgacgtgcacagaaaaatttctatggcttttcaagaactgcgacgccctatttt
gtgcccgacctacttatgtccacgggggtcgcccataaatactgtgttatgcgacc
>KolobokP-4DR_BF-LTDR FRAGMENT 1 -> 467
gggcatgctttaccaaatatgttctagtatggtactctactcaaataaccacagaaagagggtgccaaaa
tctcacctctatgactaaaagtccatgaaaactcacctcgacaggatagcccacctcctgaagtgtctaa
aagccagtttctgacaatttccacacggctctttaaaatttaaacctccataaacagacggaaacctgac
ctttgacctcaagcatccaagaattaacctgatcacacctcttgggactacttctcctagtggtctccaa
accctttcaaaacacagttttgggccaaaattattgactagtaatcctaacctataacattaacctcaaa
aatgacaaaaaaggcaccacatcttcaaagaaaaagaagttttattgttatataccctgtactacattca
cactcgccgggcaccttgcacgcatgcgccaatagaaatccctgccc
>KolobokP-N1_BF-LTDR FRAGMENT 1 -> 476
ggtagcagaacacagttttcggcctatggggatttctatagttaagggcctcaaggtgactagcttcgac
gcaatgaaaaatatagtagggtgcacttttggaataccaaaatcggatatgtttgagttgaaggtacaag
ccacaatatattaaaaaacaaaaaaatactgtcggcgtatcgtcttctcacgccctctttgaaatgcccc
tctgcggaggtcacagaactgcagccggctcacgctagaaaggcagttgcgtaatggagcgcatttatac
acaaaatagaaaactttcacaatccaaaccatgcagtctcagagtcgacaccttccgtgaccacgtagca
caggttatatctggctgcccctcaaataaggcttttttacctctccttcaacaagtttatccgtactattt
tatgtacccgcgtcatacaccccggggttcgcccactaatactgtgttctgctacc
>KolobokP-N2_BF-LTDR FRAGMENT 1 -> 661
gggggcacacaccagtttatttggggttsaaatggaggcggcacaccctcaagtctagcgaccatctct
tccagggaattatcctgaaggaaaataactgtatatggggtgagaggatataccttcagctwcaagacca
actgagtttcgatccttcaacttctctaattttgaaaaattatagggaaawawcattattttcatgtcaa
aaatgatgcacaaaatcgggcattttcgcattggataaaacagacgaagaagatttttccggcaagcacg
actgatactgccagaaagaggaaaatctaaagactaatccagccaattataaagaaaattctagtactac
agttttttttaatcatccacggcgcgcggcattgcaacttcgcttgacacagcgcttcagagktaaatcta
ggtcacsggcctctttacattcaggcgtagggatatcgcggaagcaacgcgcggaccaaaacacaatttg
caccaaaaacacaccatttcctacgcttttcagccatgtaaccgtttaatgtcatttcgcaggaaataat
gagaaatgtcaactcaaacatgggccctcggaagcctttcgtcactttttggtcgcggactaccacggac
cccggtcgcggaagaactggggtgtgcaccg
>KolobokP-N3_BF-LTDR FRAGMENT 1 -> 469
gggtatccgcaggtgattcaatttgcagcctctaaacaatgggttcctatacaaaaaagaggagaaagca
actgttagtaactcaactatctgacgttactggcacacaaacataccctcatatgaaagcccgacatctc
agctatccgaaaaaccacatttcggcatgaatctaacggtgagaacttccgggaaactgaaagattgacc
catgacctcaaaaccgcaccacaacccagtgaggaggccatgtgctaggacaaaaaagtcccaactttat
accccctgaaaaccacacaaactcagctttttggccctaatcacaaagtagaaaccctcccctgtcacac
ccacctcaaaactgccaggatttctacccatttgaccaggaaaaaaatcttgaagttttaggccctgaac
tataatcgaccaccatcaatttgcatggcggattgacctgcggatcccc
>KolobokP-N4_BF-LTDR FRAGMENT 1 -> 662
cggtgcacaccccagtttatttggggtctcaaatcgaggctgcgcacccccaggtcgagaggccgtctct
tcaagaaaatagatctaaagcagaaaagccgtatatgggaatgacgggtatcactccagctttaagaaca
gccgggtatcagtttcttcactctcactacttttcataattttggggggagaaaagtttttttcacgcaa
aaaagtatgcacaaaaaggggaattttgacgtggaaataaacccatcacctatagtttagtcatgaaaat
gactgatattgtcggaaagagaatacacctaagattaatcaggcaaaaaataaagaaaatcgtaacactg
agatttttacaataacccgtcgcgcgaggcataccaacacggctcacagaccgcatacggacgggaaatc
tgggtcaacgacgaatcacttccaacgcgtagaaatacaaggaagtgaccctcgggcccaaacccaattt
gcaccaaaaacacatcacttcttacgctttttagccatgccagtattaaatgccacttcgcagcagatta
tgagaaatttgaactcaaacatggaccctcgagaccctttcgtcacatttttgtcgcgcactatctcgga
cccctgtcgcggaagaattggtgtgtgcaccg
>KolobokP-N5_BF-LTDR FRAGMENT 1 -> 457
gagcagtgaccaccaatcatgtcttccctatccttataatgcatttaatggcaaatagaagctgtcaaaa
gtcacctacctgacgtttcgttctcgaaaaactaacattgagagggtagcccggcttctaaaatgtccat
tctcaacttttcggaactttctgaccactggaacacatgtgatttgcatccaaacgcaactcggtgacct
ttgaccccatctggcactacaatgacagctacataacaaacttctttctagtgtccaggcacccaaaaca
ggcttgaaagtctgtttttttcgacataataaaaaacctacaacccttacctgtagaatacagctccaag
atgccaacatttccaaagaaaaacacatcttctgatacttttaaaaatcaatcctgtggggactcaccgg
ggaccttctgcgcagccgtcaattagggtcattgccc
>KolobokP-N6_BF-LTDR FRAGMENT 1 -> 476
```

```
ggtctcagaacacagtttttcgcctatggggatttacatggttaaggaccccaaagtgaggtggttcgac
gactcaaaacctatagaaaggtgcagatacaatttacaagaatttagtatgttgaagtcgagggtacgac
ctacaaaatatctgaaaatgagcatagatttgcctgctcgtttttccataaaagacactttgatttgacc
cccagcggaggtcatggaactgcaggcgacgaacaggaagtgccggtaacagtatcaaggcgcaaattcc
cgctggccgaaggagcaacgtaatccaacttataccgtatcaacaacgatatattcctctaccattcacc
acagtttccacctcgctgttgtacacggaagaataactacggcctttgcaagtactgcggtgcactattt
taacccgaactacttttggggacgggggtcgcgkakaaatactgtgttctgagacc
>KolobokP-1_BL-LTDR FRAGMENT 1 -> 467
ggccacagaacagtaatgaagcttcctatcttaatatatggcaatcatgtaagaaagaagctgtcaaaag
tcacctaccagagttttagtccatgaaaaacatgcattggcagggctgtcaggcctccaaagtttcaatc
tagagcaaaatttcaactttctaatcggcggaaccactacgaatccgggcatgtttaccaaagttgacct
ctgacccccttctccacctgataatcacacaaaaacagcaaactttgcaggcctacagacaccacaaaca
tggatgaaagtctgatttttcaacttaataagaaggtagaaacccttacctgtaaaatccagctccaaaa
tatcaacatatcctggaaaaaagcccctttacagctacctttaccaagggtaccaaacctatttccatgg
gctaaaagttggccttctgcgcctgcgcgaaatactggagtatcccc
>KolobokP-2_BL-LTDR FRAGMENT 1 -> 468
ggggaaagagtaccaattgctggcccctatactttacatggtagacatataaggaaagggctgtcaaaag
tcacctacctgaagtttaggtcatggaaaactcacattggcagcgtagtctgacctctaaagtgtcaatc
tactgagtttcgccgattttccaccggcgcaaagtggtgaaattgtatcaaaacatcagcagatgacctt
tgaccccccttcacaagacctgatcacacccaaactacacctatttcaaagtgtaccggacacttcaaac
acggctaaaaccctgtttttctaactaaatgaaatagccacaacccttacctgtcaattccagctcaaaa
acaccaacatttccaacaaagaggcctcctgcagctacttttaatagcatgtacatgcggtttctgtctg
gcattaaaatggaccctctgcgcatgactataatggtgaactttgccc
>KolobokP-3_BL-LTDR FRAGMENT 1 -> 478
ggggagactaaagggaaacgcttaagcacaccagtctatgagaaaataggccaaaaaagggcatcatatc
tcacctttcagacattccggtgacacaaactcaccttcatgtggtagattacttcttaatttgtccgtac
ttgtcgtcacaaggaatttgatacggctattttctagctacaggccattttgtaaacaaacctgaacttt
gacccctgagccaggcctacaacaggcgaacagccagctgggagtctctggcatcatgtatccaataacg
acaccatatatggacaaaattctgtttttttgccaaaacattttcttaaaaccctatactatgccatca
gccagcaaaatgccacaatttcaccaaattgcccctcaaaaaaggttttcaaagttctaataagaaaaga
atmttgaggtctctcatcgtgcatgctacgcgcatgtcccattccctttagtctcccc
>KolobokP-4_BL-LTDR FRAGMENT 1 -> 477
ggggagggagtgctgaaatcagatcccattcagaacctgtataaacaaggaacaaaaacaggcataaaac
tacacttaccagtatttcaatgtcccaaaacttccacccaatgaacagccaaacatgtctagttgctgtt
tttgcaaaaagcagaggttcgactcctgcattctgaagcgatattggccatcgaacgtaaacaaccactg
acctttgaaccggcagctgggagacactggtctggcccgggaaaacccccctcccagatgatgtaattcta
aaccaccacaagtggcaaaggtcacaaatttttttctggatttgaaaagtaaacatatgtctggtccccct
gcctgaaaactggccaaaagcgaccaaagatttcaccaggaaaaaattctcaaaaataataacaagaaaa
taaacgtggaccttcaaactgggcttcctgcgcatgtctatcaagaggtccctcccc
>KolobokP-N1_BL-LTDR FRAGMENT 1 -> 477
ggggagactaaagggatacaataaatggcagtaatgcatgtagccctatggggaagttaagaaaaagaag
gtgtcattatctcaggaatcaaaacatcagactacaataacttaccctcaaatactaaccagttatgtca
actgtcatcagattcatccctgacaaattctatacggctattctccagataaccccccctaatcagagcc
cttggataggccctacaggcttcaaacacccagttgaagaccatcccactatatcaggggggtgccaaac
cccacaaggtagagaaagaccatttatttcaactaattcagcaaatacagagtctatcctaccacagcct
atcctatattgcccagatttcacaaaaaatcccaccaaaaagaattttcaatggtttaaaccatgttttt
tacttggagtctctcatcgtgcggtctgggcgcaggcgcaatccctttagtctcccc
>KolobokP-N2_BL-LTDR FRAGMENT 1 -> 477
ggggggattaggacattccaatttgggccaataatacatgcatccctatggggaagttgacagaaattag
gtgtcattatctcaggaaccaaagatcctgaccaagaaaacttaccctcaaatttttaccagttctgtag
actgtcatctatcagaattacgagaacttctatacggctagtctccggggaaacacccccgagcgagagc
acttgagtcagtccatttcccttataagggccgatcgcgaccatcacactatctcccgggggtccagaag
acaaaaagatgaagaaaggtcgtttttgtcaacaaattctaaaattatgaagtctgaactgtcataccca
ccccagacttgccaagagttcaccaaaaagcccaccaagaaaggctttcaatgttttaaagccctgttta
ttcttgagctctctcaccggagggtctaggcgcctgcgccaataccatcatcccccc
>KolobokP-1_PaMi-LTDR FRAGMENT 1 -> 479
gggtgcgcgcctgcaaataaattcccatagacactagtgctaaatgtccatacttcaaaattcatttaaa
aatactcagaactctcacaggtattcaagctgcacagaaaaatgatgatcaacatgtcatctttcatttt
cttttcaattttgggtgggttgaccattgactttttgagatattctcacccaaataagaacacccctaaa
acgaatgatgtttcccgaactggaaaagtagccattttgaaaaagaatccctccacaacatggctaaata
gcgccactttcgggccagattgcatccaaaatgctcagaattggaagctccaagtccaggccagaggttc
agtgccacatgtcttcaattccccaattccatggttctcagaactctctaatatagaatattttgagggt
ataacttttatttatgagctcggcggacggattttggtctatttacaggcgtgcaccc
>KolobokP-1BDR_PaMi-LTDR FRAGMENT 1 -> 428
gggtgcaacactgcaaataaattcccatagacactagtgctaaatgcccatacttcaaaattcttttaaa
aatactcagaactctcacaggtttttaagctgcacagaaaaatgatggtcgacatgtcgtcttttcgaga
aattctcgcccaaagaagacgacccctattttccccaaagcggaacggcagccatcttgaaaaacgatcc
ctccacaacatggcaaaatagcgccactttcgagcccaattgtatccaaaatgctcagaattggaagccc
cgagtccgggccagaggttcattgccatatctcttcaattccccaatgttatggctctcagaactctcta
atatagaatattttgagggtataacttttattcatgagctcggcggatggatttaggcctatttaccggt
gtgcaccc
>KolobokP-2_PaMi-LTDR FRAGMENT 1 -> 470
ggtagcagaccagtaaataaacttccatagaccccaatggtaaatgctcccacttcagaaattcgggaaa
aattcttacaactctccaaggtgttcgagacacacatcaaattaaagcttattttatcacctttcatttt
cttttcaattttcgggggtcaaccgatgacttttaggagaaatttccatccgaagatgatcacccctaa
aaatagtcctgaagcccatcgccattttgaaaaacgatggctacactccattgcaaaatagagcgctttt
agggtccaaattacatccaaattacacacaactgcaagctcccagtcctgtgcacggcttccacccatat
```

tttttacaaaccctcaagttcatggcccacaatactctcaaatactgaagattttgagggcatgcaaatc
gtgatttggctggcgtaggcaatttggggcctatttactggtctgcaacc
>KolobokP-2N1_PaMi-LTDR FRAGMENT 1 -> 470
ggtagcagaccagtaaataaacttccatagaccccaatggtaaatgctcccacttcaaaaattcgggaaa
aatactcacaactctccaaggtgttcgagacacacatcaaattaaagcttatttcatcatctttcatttt
cttttcaattttcgggggtcaactgatgacttttaggagaaatttccatctgaagatgatcacccctga
aaatagtcctgaagcccatcgccattttgaaaaacgatgcctacactccattgcaaaatagagcgctttt
agggtccaaattacatcaaaattacacagaactgcaagctcccagtcctgtgcacggcttccacccatat
tttttacaaaccctcaagttcatggcccacagcatgctcaaataccgaagattttgagggcatgcaaatc
gtgatttggccggcgcaggcaatttcggaccgatttactggtctgcaccc
>KolobokP-2BDR_PaMi-LTDR FRAGMENT 1 -> 438
ggttgcagaccagtaaataaacttccatagaccccaatggtaaatgctcccacttcagaaattcgggaaa
aatactcacaactctccaaggtgttcgagacacacatcaaattaaagcttatttcatcatctttcatttt
cttttcaatttttgggggggtcaactgatgacttttaggagaaatttccatccgaagatgatcacccctg
aaaatagtcctgaagcccatcgccattttgaaaaacgatgcctacactccattgcaaaatagagcgcttt
aagggccccaatttcatccaaacggcttccacccatatttttttacaaaccctcaagttcatggcccacag
catgctcaaatactgaagattttgagggcatgcaaatcgtgatttggccggcgtaggcaatttcggaccg
atttactggtctgcaacc
>KolobokP-3DR_PaMi-LTDR FRAGMENT 1 -> 483
gggtgcactgaaccaattcaagccccattgaaaacagtgtaaaatcaacaaagttcagggccaaataaca
tgcaagccgacatcactggggtttctcaaacaaaaattaaattattgtcttgtacttcatcttttcattt
cttttcaattttgggtagattagccgattacatttttaaatattcacaccaaaacaaaatcacctcccga
aaagtgatagttcccaagcctgcatgtagccgttttacaagtggcatccttccactttctcaggcaaata
ggacccattttttcaaccgtaattttatcaagcctgccctcagatggaagccccaaaaccagggagttgat
ccttggctacatttacacaattccaccgtttttaaagccaaacggtctccgttttcaaaagaaactgtgg
gggtcactttttatgctaatcagctcgcgtagaaattctcacttgaattagttcactgcaccc
>KolobokP-1_LyPi-LTDR FRAGMENT 1 -> 466
ggtgtcacgcgcgtaaaacattccccatagacttgtgtgttataatggcacttttcaaaaattcataaaa
aataaatgtgaaattagagggtcgaatgtttactaccattggataggatatatatctgacattccatatc
tgaccaggaaagggtaccgtagccagctcatttttaaaaataaatcgccatttatgaagataactatgaat
ggatcaaattcatcaactgaaacagtaaaatggcccttattcttccgccagtcaaaaaatagttccaagt
cacttttgtatctccccaatttgggcaaaggaagttcagtagttaccatttctagaatcagtgttaaatt
ttcaatcatattcatgcattttcgtcaatcagctatatcaaattgaaaaaaaaattgaatgggttgggtc
gaatgaatatcttatgccttcccgatgtaattaggctcgtggcacc
>KolobokP-N1_LyPi-LTDR FRAGMENT 1 -> 464
ggtctcacgcgcgtaaaacattccccatagacttgtgtgttaaaatggcacttaagaaaaattcataaaa
aataaatgtgaaattagagggtcgaatgtttactacctttggataggatatatatctgacattccatatc
tgaccagagaagggtatcgtagccagctcatttttaaaaataaatcgccatttatgaagataactatgatg
ggatcaaattcatcaactgaaacagtaaaatagccccaattcttccgccagtcaaaaaatagttccaaat
cattgatatatcttcccaatttgggcaaaggaagttcagtagttaccatttctagaatcagtgttagatt
ttgaatcatattcatacacttttgtcaatcagcaatatcaaattgaaaaaattcgaatgggttgggtcga
acgaatatctttagccctcctgatgtaattaggctcatggcacc
>KolobokP-N2_LyPi-LTDR FRAGMENT 1 -> 465
ggtgtcacgcgcgtaaaacattccccatagacttgtgtgttaaaatggcacttttgaaaaattcataaaa
aataaatgtgaaattagagggtggaatgtttactaccaatggataggatatatatctgacattccaaatc
tgaccaggaaagggtaccgtagccagctcatttttaaaaataaatcgccatttatgaagataactatgaat
cgatcgaattcatcaactgaaacagtaaaatagccctaattattccgccagtcaaaaaatagttccaagt
cacttatttatcttcccaatttgggcgaagtaagttcagtagttaccatttctagaatcagtgttagatt
ttgaatcacattcatacattttttgtcaatcagcaatatcaaattgacaaaaattcgaatgggttgggtcg
aacgaatatctttagccctcctgatgtaattaggctcgtgggacc
>KolobokP-N2BDR_LyPi-LTDR FRAGMENT 1 -> 461
ggtgtcacgcgcgtaaaacattccccatagacttgtgtgttaaaatggcacttttgaaaaattcataaaa
aataaatgtgaaattagagggtcgaatgtttactacctttggataggatatatatctgacattccatatc
tgaccagagaagggtatcgtagccagctcatttttaaaaataaatcgccatttatgaagataactatgatg
ggatcaaattcatcaactgaaacagtaaaatagccccaattcttccgccagtcaaaaaatagttccaaat
cattgatatatcttcccaatttggcaagaagttcagtagttaccatttctagaatcagtgttagattttg
aatcatattcatacacttttgtcaatcagcaatatcaaattgaaaaaatttgaatgggttgggtcgaacg
aatatctttagccctcctgatgtaattaggctcatggcacc
>KolobokP-1_ApJa-LTDR FRAGMENT 1 -> 469
ggtagcacgtacgcccattaaaagccccattgacttacacactaaatcacaaaagtaatgtcttctctaa
gcctagatagaagttttcacttgctaaacgctacccatcactggatagctgacaagttggcctttcatgg
caccatcaattttccgtaccatgaccgtgagatttttttgctacgagagccggaacttttcgtcacttgta
aacaaacctctccactcagtsgtaaacaawcttcstacgctgctccttggaggsctaaackggtctaaaa
ttgcctcaatttkacccaatttttttaaccacatgtgcttccattcatgttctttaacatcmaagccacaa
aaaagcacatcctaattgataacatagcaaaaaagttgttctcctgctcctaattggcacttttcctgaa
ggagaacatttgccgcggattgatatagacgctaataggagtatgccac
>KolobokP-1_CGi-I
tttaactttgagtgaacatccttaaatagggggtgaaactgccttaaaatctacccaaggctatgacggag
taaacttgacaaaattttgaagatgggtactatggggaatgaaatataccagcggaacaaaacatgtaga
ttttgaataaaaaagctctttccgagaaagtatgagttgtcatttgttaagggtccaaactacaaaaact
caaaaagactgaagacgactaagaaatagaagaatactacagagcctatagattctatatcaacagattt
tacccaactctgacttattgcatccaggtgaacacttacatattagccaaaatggtctataattttgaat
tttccctacattttttattaaattaaatacaattaaaacacataaaacatcttaaaatactgaaatttgat
acaaaggatctccaattagcctacacgggtcacatatcccctcaatgactgtttatctaaactcagcaag
tgataatgtgcagttagtcttaaatccagggggagataatctgaagacatgatctgtgaccaccactga
cagcatccatcaattcaccctttataatcctaacaaagcctcatcagaggaaaattccttatcagatac
tggttacactcttaatttaaacaagcacattagtgataaaaggatggggatacaccaagtgctgcaacta

tatgtagcaggtacccaaaggcaggccacattcaaacatgccccgctccaagagaacccaatacaaaata
aaccacggaaaacgcaaagggtattttcaaaagagtcacccctatttcagttcaaaagtagtacctgttg
gcggttgccatgacgacgacgttggagatgctgatgacagttcccggtcagtctcggccagtgaccagtg
gatgcgtccgccccaggaagcctatcgagatgccctttcatccatgtcctcttcatgccaacagtccggt
gcggttctgccatctcgactccgtcctgtgaagcctacaacagtcaaaattgcaagtgaggatcggtctg
acggagaaaacattatagtcaacgtacaatcactgaccatgctactgcaagaaacagcagtccaccatta
ctgcagtagccctaaaatgaactttagcatttccaagagacaaggactttgcataacaggccagtccaag
tgtcttacctgtcggttcagctctagtcaggtgcagctgtacgaaacacacaagaaatctagaggaccgg
agactggaaccctcaacgaggcccttctacttggctgcctaaaaacaaaggttggagtgtcagacactag
cttcattctcagctgccttaatgttaaggcacctgatacaagggggaatgcagcgaaaactgaactcact
ctgtgaccgagtggaagaaattaactctgagagcatgcgagaaaaccaacgatacgtgaggagggtgaac
cgcttggcaggaagggaggagacggaagtcgacttggagactgacacgtcatataacaacagaccacagg
ccggcttcgaggctgggactcagagcttctcaccccttgtagagaacaacactccccggaagctggtgct
ggtgctacagactgccaacaagctctgtagtaaaagatcctgtgaccaccggaactgcaagaaaaattat
gagagtgatgagaccattgcatcaagtgaagctacccttgtgaggagaaacttagattccctgcgcgagg
agaacattctcaatgtcagatcagtaaccagtgactcctctgcacaacttgataagacactcagagacta
ttcagcagcgactggcactaaaatcatacagtacaagtgctttattcataagctaaggaacctgcaaaag
aacatcaggagcataaagctaaagtccccccctcccacccggtagcaacagagatgcttttattcagagac
tggcaacatgtgtgcgagcacgagttcgatatgaactggtacacattagaaaactgtgcaagtctgatga
acttttcatacataaggctgagcaggccatatccaacattctgccctgcttttccggagtccatgaccac
tgtcgcagaatgtcgtttacgtgcaatgcccacctacctacttacagcgccaaattcctaccttatggaa
ggcacctggacctggaacctgaagacatttccagcatacagagggtcatcaataagcagatactgggcca
aaacttaccaaaaatctgccgactctcgactactaataaatgtgagagtctccatcagacagtgttcact
tatgcaccgaagaataccacttgggccagaaacttcaccgcactgtgtcactccgcagttcatacctcta
ctcacggcactggatcttcctcatctctcattgccaacaaggtcggtgtgcgctgggaccatgcagggcc
attccaccggaagatgactcagctcgatcgcctgaggcagtaccacagcctacgaaagaaaacaatcaag
tacaagaccactcgacactttgggaggaagaagaaaatgaaccgaaaactccgagaactctccctctaca
ataacgaaagagaaaacagggcaaatgagcatgcgtgaggaattaactgctaaaccttaacagtaacatc
atactaccttatggaaacagacactgcggatatggttcatgacccatgcatctccttaacgacttacatg
ttttccgtgaattgtttacagactgcggctcaatagacaaatgctgtgtgtttatacaaattttacagtg
acacagatgagaaacatgtaaatttttgtcaatatgatgtccttgtataaaaaaataaaatctgtgcact
aatactttgtgttgtttactgtttatgacggggcgaaaaaccccataccctgactcgttaaggctgccta
accaaaaatgacaaaaattgktcctatcgaccttcctttcaattttagatatgaattgagctttctgctc
ttccgttaaattgatttcgtaaactgcagacatgattacattagagatgcagaggtaaaaaccaaatatg
gtcaagtacctgtaattaacaataaggaaagatcaatgggccattttaattgataatttaattaatattc
tgcttaattaattaataaaatgaagtgatggggcagcataagcaaaaaatattgttaccaagagccccca
gtgtctgcaggtccgtccattgtgctattcaccaggcttagtaatcactgcctattcctagccaaatatt
agagttatctttccttggctacaaagccaaaaatgagtgtaaacagatacaacagaggacaaaatctgac
caattggaactaacagcatcttatgcaacacagatatgtcactaaaactagttttggggcaattcataat
tttgacctatgcttgaa
>KolobokP-2_CGi-I
tttaagacaactaaaagtttatgaaatttaattaaatttgcagaaaatgtctagtaggtatctatcacct
atatgctagtgtatgaaaatctacttttgaccctaagggattaaaaagtaggagataactcttcttaaca
gcttgctgaattctggagtaaatttctgttaaaattcatctacagttcatcttttcaaaactcaataaaa
wttaattagtgagatattgagaaaagagcttggaatgaaatgaaacctgaaatgttagagtttatgtgta
tgtgctttaaaaaaaattcatctctaattaatagacattaattagctaagaaacaggaaaatgacttaaa
tattggaattaattagcaaaaagttgttaattttaattctaagacaaagacagagactggaaaaaatggt
gccagaagagtcttcaataatgtaccttggagctttgataacggtggtcactgattaatggctagtgatt
aagataagaggttcttaagcaatgggggtgatgaggcttttccatcagtcatcaaagatgggggttcct
cttagaagacatggcagctctaaaataaataaacagtatgaaagtttacaatacttaaaaatttgcacca
cacacaacatcatattattagaaaaagtctacaagtgtacagttcaatataaatcatgccaaagaaagga
ccaaagtggctgcatcaacatcagtacaaaaaaggaaacattcctcacaacaagggccagaaaagtacag
ggaccaccagcccagtacagtgtctatgtcctataagaaggctgagtaaagaagagtttcaggacgtcgt
acagcagcagaaggatggaatattcacaataagcgatgctgagcgtagaggcagtcaaatgaagatttg
cgacccgggccgagtgaagtgcagctggtagactcctacacggatgcagagtcaccgaaccctaattcca
gactttacaaagtgctccatcaagaaaaaactgctaaactatggaaccatgcatttaaagagcactcaca
gaaattcccggactgtgacggttcactggactggaaccagcatggggaggaaaagagaggcctggcatgg
atcctttccattcgatgtaagaagtgccattacaccagtcaccgtgaaaagctgtacgaggaagtcagtc
gatcaggaaggggacggagagccgcacagcttaatgttgccatggctgtcggccacttgggtaccagcct
tacaacagagggtatgcgtggtatgcttcttggcgcaaacatcgaaccagcatcagccaccaacattcat
cagacctgcaacaaagtaggcaaaatcatcactgatgtgaacaaaaacagcatgaagaaaataaggcagg
acattcaagagcttaacgagaaatgtggacttgccaatacacctataagagcggaaggagatgcccggta
taacaatgccaccttcagtgcgatagggaaaacaccattccaagctgccacacaagtgacatacacttta
agtgaaaatgttaccaagaagaagaatgttgtggcagtgttttgtggcaataaactgtgcaagaaaggca
cccatcttagagccaagggaaaggaagttacctgccccggacatgaaggctgcactgcaaccattcctcc
agagaccaccattggagacgaaaagagatgggctgcggaatgcatcagtgagctgcaaagtgatgaccgt
ccactcgtcatttcccatttcaccagtgatggagatagtgctgctgcttccggtgcatctgaaaagcaag
ggcatatgattgaaaacctcaaagacctgcgccacttctttgactctcagagaaaacagactgctaaggc
tccattcagcagtcacatgtttcctggaagaacaaaagctatgagagaatccatgcagaggagatttgcc
ctggacctgaagcttagatgcaggaccgagtacgaaaactgctacaaacactactccggtgacttacctt
tgatgaaacgggccatgtcaactgctgtccgttccatcatcagctgctaccaaggacagtgcggaaagtc
ctgccaacttcattccttctcctgtcgcggactgaaaaacaacaagtggaactcttcagtgttgcctcgt
gactttgaattaagcatgacagaagaagacatgtgcttgttaaaagactgtataaatctgactttgggac
ctaataatcttgatagaattaaatatcacacttgtacacaaaaatctgaatctgttaatagggcatattt
aaaatcaaatccaaaatgtattacaagtagtagaaattttgaaagtagaatacataaagttgtgcactca
ctcaatcggggtcatggaaattcaacattggaactttgtgaatctgtaggggcacctgttgtaaaaggaa
gtagagtggtccatcatctaaaacaacagcaaaataggcaaaattactttaaaatgaaacaaaaactatt

aaaatataaattccaagaatgatctaaaagatatcagctctacgataaaaagtctaaagacacttatagt
aagtcactaacagatccaaagttaaacaggaagtagctaagtgtgaaggggggggtgttatcatgggta
ggtggggggggggtgtagttagcatgtatgaaatgtttgttatattatacctgtcatgtgaattttgtat
tacatttaatgattgctatgttttaataatgtgcactgaatgtatggtaatgtgtgaatgatgagagtgt
gtaaaatgtgacatgtccttttatgaggtgctctgctcatgtaaatatgttatatttctcattgtttatc
ttattgaaatgttgcaaacaattatcttgattttcttacctgtctggtgtacacaacaaagaggactgag
acgattggtagaaatacgggtatacaggtgtcaaatttcaccgttggtgacaagacagcatcccgcattg
tttatccgccattgttgatctttaacaagggagacaactcctgaaattagaaaaaatgcagtttaaatga
aagttacatgtttctcttta
>KolobokP-3_CGi-I
ttaaaggaaataaaacatttctgtatgctactttgtaatgcttagatgcaaagaggacaaaattacaaac
attttggtgtatgcagcattcaatatagtcaagaaatggccgagaaatgtccaagacattgactgaccca
acaaacaagactgaacagtcaagaattcagaactgaccatcaactcacaaattctcaatttatctaatat
aaagaacatcgtgaattgaactgcatgtatgaatattgatgacatgcttcagtacatattaaaaaattcc
tccaactacacattctgttgttttagatttacatttgttgaattatgaacacatgagtgatgattctatg
tgaaattgtgttgaaataagagaatgcctcaaatgtccatagagtgtcatcacacagttacatgtatata
cacatacatgtatatacatgtacatatatatatatatatatatatatatatatatatatatatatatata
tatatatatatatatatatatatattgatgacatgctatgacgtcacaatgcaaatgcattgatgatgtc
acaatatacatgatgtcataatcacatacatgtatcatcacaatgtaaacaatcatgaatgagggactat
gctgttaaatgattttcaacatgcctagaactgatggacgcaggcaaggcaaaaagaagaaaacacgcaa
gactacatttctaaagggacacctaccttggaataaggatcagcagcctatcagcagtgttccttgcacg
gagtccacctctgtcaatatccctgtgtgtcgtccggagaaagcaatctatgacgcttcaattctaaagt
atgagggtcctccaaatacccttccaggaaagctaagacctaaacatgtaagaaaagaggatgaaccgtt
agacaattcatcagaaaatataattgttaacttctgtaagttaaaagaattcattaaatcaagcattagc
cataactgcaggcgacaagacagtttcaatgtggcaattgctaagcgaaaaggattgtgtgtatgtctgc
aggcgagctgcacaaactgtggttttgataccggtgtgcataaactgtacaccgagtatacacgcaacaa
aagaggtcccgcggcaggctgcttaaatgaaggcctttctatggccaccgttaaatccaaaatgggagga
accgacgttcagatgttaatggcttgtcttgatattcgtgctcccagtttaactataatcaacaacaaaa
tcaataagcaatgtgaaaaaatgatagaactaaatgaaaaatccatggttgagaatcagaagtttgccgg
ccaagtggcaaccttcatcggagagggaaacaacattgatgtcgaaacggacacgtgcttcaacaaccgt
aaccaaactgggtacgaagccggcacgcaatcattctgtccggtgattgagaagacaactggtcttaatc
tcccaattgcaatggccactgcgagcaaactgtgttcaaaaataaacatgtgtacccatgagaacaacaa
atattcactgacctacaaccctgatgaatcaatctcctccagtgaatcaaaactgaccactaaaaacttg
caaaagattcatgatctcaaaattgtatccataaaatccttaactagtgatgcaagctctcaaatcagca
aggttgttagagaagttgggaaaaaaaatgaaatcgttatcaagtacttccaatgttttgttcaccgact
acgcacaattcagaaaaaagtgaaatccatacagttcttaaaactcccggccggttacgatcaagacatc
tttcgccaaaaattggcaacatgtatccggtcgagagtgagactcgaactagtaaggattaaaaagaagt
acccagcagaggggctttgtgaccagggcactgatggctatgaagaacattgtgacatgcttcagtgg
atcccacacttattgtgcagagagatccctggcctgcaatgctcacctggagtccctcagtactcagtat
cttccatatggaagttacttaagtctggtcacagaagaccgccgcaagctggaatcggttatgaataagt
ccctgacaactgaaattttgaataaaattgctaatttatatacaaccaaccaatgtgagagcttgcatca
cagagttttacatatgcaccaaaatctgtgttgttcacaagaaattttaatggactatgtcactcagca
tgccattcatccacatttggaactgggaagtcatccgtactgttagcaaaatcccttggtcttaatttct
caaaatgcgcccctttttccgttacatgactcgaaaggatatattagctttgtaccattcgcaaaggaa
aaagacaccaaagtacaaaatacagcggtatcttgcaaaatgtagaaaaagtaatcgaaaaattagacaa
gggtccttgtactcaaatgcatgtaatgaactggtacaaattcacaactatgcaataaatgtcaacaaac
actaaatgttaataaaacagaaaaaaaaaacaaaaacagagttcatttttttgtgttctactgacctaaaa
atacaattttaaaaccatcatgcccgcagcatgtctttgactgtctgcagctattgcgctggacattaa
tttctcctggctccagagataaatgctgaatattcatacatttttttgttatgacataagtgggaaacatg
atatgatatgttatgaaaatgacaatcattttccaaataatacataagacggtgaacggtaaaaatttgt
cttttcagacggaaagttggtcttatgatgccatatccatcagagtctgtcgcaccgtaccatatatgac
aaccatgtaattcattaaatttatacaactttgagtataaaaattgtttctcttctgttgtgaaggggt
ttcatatgccttgaacatgatagatttttttaaaatatgtactttaaatccagcggtctgaaacaatatca
tagaaaacacagtcatgatgttgtgtcaaaagcaactgacactgaaatgggctgtaagtaaatgacatat
ttaacatgtacaacacactggcgtccgatgcaatattgagagaaaaaacctaatttccaaaatcatgaaa
atcctaatccgtggggagtaatcctatacctatacttccaaaacaaaacatctttcccggccataacatt
tttttaccccaaatcatgaaaatccttatccgctgggcgtggcgggatgagtatacctatagttccaaaa
aaaaatatgtgggggagtatacctttgactccaacttctcaatctttcaaacgtacattacggaaaaatt
atgtttcatgcgaagaaaaagtggggggctgaaccctctattctgctatgtgccaaatgattaggtctaa
ccaggggggctaagcctatgcaacacatgtaaaaacatgtacttatgctttaatgtgacatgaaaccaa
tactgtatgatttttaataaaaaaaataagtcacatcaaccactttgcagcctgagcatgcagaacaatg
aagaaaacgttatttcatgtgtgtcccttaa
>KolobokP-4_CGi-I
taataatttcacatacctttattgattttgatgaaacttggtaggttggcacctgtgggtatttccaatg
tctcctagaagtttcggtgaaatccttctccttacaccagagttatgggcccctgaaaaaaggtacagga
atagccgaaattgcatgccaacaccagcatctcacacaaaattcacttcactaaagtactcacaatgacc
ttattttataatcaatttccttgaaactttaatatgtcatatttcaacttattgcccaaagacatctgac
atttttctcatcaagattaatattatgcaattttaataatttttttgaaattataccccaaataaggcta
taaatccccagaaaagcccaaaaataaaatatgtagacacagatttttcagtaggaaaaaatatgtaaaa
tttctacatttaatatactatagaaatcagacatgctagtaaataaacagcaagtaatccctttgttgta
agcagataatcaaacatggccactgcaaccaacttgaggaagtggcacttacaggtcaaaatggattagc
tctgggtacagtttagatgacatggataggtataaaatgccatttgaagtactgtcaaacatacaagact
ataaatatgatagaaataaaggaatccatcacaccatctttgatacagcaagcagaaagtacttacagaa
gttatcaaaatgtttcagaaaaaccaaaggccatggaacaaagggagggaactccgccaaaggaagccgc
ccagcaggaggtttttttcggcaaggtaatactaacagatccaaggcttcatcagaggaccaagagacaac
tcgtaaacgctcctttgtccggctatccgctggggaccatgccctggtaacacgaccaagtttagactgc
caatcctacataactcccgactgtgatggggagagtggatcctcgtgtattcttcggccacggtcagaaa

gctgcagatacctacaaaatgccaacagcaatgaatatgaaggggacaacgtgtagtgagtgaggagaa
aatgatagatatgatgacgagcacagcccatattcatcagactacttcaagcacctgccccatgccaaat
tttgctgtactcgaggcaagaaagctaggcatctcttggaagtacaagataaaatgcgcaaattgtccgt
ttacctctccgacattcaacctgtatcatgagattcatcaagagggaccaggccgtaatccatcagcaac
caacatcgctctcgcatatgccattcaagacactgccatcggggccgaaaagataattgaactcctcacc
tgtttggacatacctgctcccagcaaatcattcatgcagacactcctgtgcaaaacaagcagaaaaatga
cagaacttaacaaactggacatgaatgacaagatacaaaaagtcaaagaggttaacgttactcgaggaca
ccctgaaaatatcatcaacatttcaacagatgcaaggtacaacagcagcgtcatgtttagtcggaagacc
cccggccagaatgccagtcaagccttttctctggcagtagaaacaaatacagatagaaagtacatacttg
cctgtgctgtacagaacaaactctgctggactggtgcatggctgcgcggcaaaggcatggaggtggactg
tcctggcggtcatgcggaatgtactgcaaacctgtctccggctgctccgttctcggaatacgagatggga
aaggatatcggcactcagctcggcctgcaagatgttctagtccggtatgttaccacagacggcgatgccc
aaggtgctaaaggagtggacgatgccatgcgggctcttcatcccttgtggaaagtagagcgcctagcaga
ccatgtccatcttgggcaatcacagttccgtgccagcaaccgagcagtatacagcgatgaaatgttccat
tctaagacaaaagaagaaaagaaagagttacaaagggtgtttagcaatgacttaaaatccagatgttcca
tgatagtgaaaaagctattcaccaagtatgacagagacctggagaaaatgagcagtttcttacccaaagt
cctggaggcaactgttgcatgttacgacggagactgttctatgtgtgccgcctattccttggtctgtgag
gggagtggtctccaactggtggaactttagccgcaacttggccatctataggatcaccgccctgcaaatg
aatgacaacgacctaagaattgtgacagaagttctgaaaatgaagttgagcgtggaggctatctcngcca
tgaaacttctgactgacaccaatgccaacgagtcctaccatagatctgcaagtgctaacatgcccaagaa
tgtgaaattctcaagaacaatggaaggccgtctgcactccatgattcacaggaggaataacttaccagga
acctcaaccagactcaagtgtgaatctgccggtgtccaatactcagactccggcaagcgacgactagcca
ggattgaccagcagttcctctacagacaagaataccataaaaaggaaaagattaagaccagaagaaacaa
acagcagggtaggaaaatggaggaacaccatctatatcgacaggaacatcggacaaagtcagactaccgg
aagggacaattggatatgaacctacctgcagcacgtgatcatccctaccacaggagaggagcatgccaat
atcatctgaggcctcgcccaggatgtgactgaatgactttatacccattttatgaatgatactgactttt
actaaccttaaagcttaattgaactgaatccatcatgtgccatcaggcatatactaaaaccattattgtg
aggagaaatgcatgactctccaatgtttagcggtgtaacattctaataaatatgataaacttacctatat
tctttgttttctcaattcatgtcaataacgaggtcaagtctgcaaggcaggtacggaaagtggcccaggc
aggtgcccctagctcggcaggtctgtcgctggttgttaataccatgtggttccagagtgatgtgctccgg
aacaacacacagcctgttgtggcacaagtgtgatgcaacaatggatgcatccaactcatgataaccatga
tgtagcatgacactgagtctatgtacatgcatggtaacccacttgttctttactggatgcttgtagcata
caacaccatatttgttgttgctgcttctagcaccagtccagatgcgacactgaccgttcaaactaggagc
agagcccgagcgcactttttctctgtattttgtcagaaattcgtccatagccacgaaatgaaagttgcag
acgacaaaaatagtgccgacgcatggttacatagaaatacgacttacctcatttgcatatgcgggtcata
ctaatcacccaacaataaaaccacccccacccccaatttttcctctattttggtgaaattaaattttca
taattgaacaaaaggcccaatttctccataaattaccttaaacgattgatatttgttaagaaatgggga
tagaaccctaaataagcaatttcacttttgacctgata
>KolobokP-4N1_CGi-I
taataattacacaattcttctttgattttgatgaaacttggtaggtagacacttatacccataatacacc
tccctgtgaaatttggcgggaattgtgtattctgcatcagagttataggcctttgaaatctggtccagga
aggctgaaataacatgcaaaatctagcacgttacacacaatattcaaatttcaagagctacatttaacat
taattacttgtcaaaagtttcattaatctattacattcattaacctaacatgtctacatgatgatgctaa
tgatgatttaattcttttacacctggaactatttttggaaatttttgaaaaaaataccccaaatatggc
taaatttttcagaaaatgccaaaaatgaaacatttaatcacatgctatgagcttgaaaaaatatttaaaa
aattctgcattaagtttgctatagaaatcaggtattttgataaatatacagtggctaatccgtttacatt
aagcagataatcacatagaaccattcacacattttcaggaaatggcacttgctgaccaaagtagattaac
tcagagtacacagggctgatatggataggcataaaatgccttttgaaacaaaactaacaatttagaagaa
tttagaatgaattctatttgtccattcattacatatacactgaataaattacttaaatgtaccctaattc
ataaggagccttggttcatgaaaactgtactcttaaaacaaagggagataactctacacattaaaatcca
aagtagggatttttttacaaacattaacgagaaactatttcagaaattacaagtccatcaccttctttcag
cattatctggtgtgattaatcttgaggagatcataccttttgtagcgaggattcaccattcaaagtcatg
ggtttctacatacattgtgcatttagaggtgaaaatgtctggtatgttaccatgaacaatgatgaccaag
actcaaattaatgatgagagtgccaaaaagatattactacagtattacaaatagtaaagcttctagcaga
cctcatatatcttgggcaatcactgttcaatgccagcaaccaaactcatacacatgctacatttgttggt
agttacatttctaggacacaaaagaggggggaaaactgacataataagactttaaagcagtaatttaaat
aaaaktttgcaaggcattgataatttagatgctgaaatgactatttctaaccagtaaaatgtatatgtct
catataaatatctatacatgaaggttwacatattctaaagtactgaggaaaatgttgtgatgcctactca
ccatcagatttcaggtgttaaattgtaagaaaaatatgaataaatgaataaaatcaacttaccatttgtt
gttttcttcattatttctctgtcaaaatggtgttaactggggggggggggggggggcagcaggtgcccc
tttaccagcttgtatgtcctgtcatgcaatctgctaggcagtgaaatatgtcatcattcataggaatcat
aaagtaacataagccctactttcctccttataatttaatcaaaataatattaaaggaaatatatatatac
ttttacatgtaatatgattaatatgagagagagagagagagagagagagagagagagagagagagagagg
gtatatttaaggctttataacttaatgacaaatcgaccaattcttttatgatttgggtgataagtaaagt
taacattagaaagtgtttttagacattttgcaagtacttacgtcccctgtttgactcattgatgacacca
tgctgttcacaccccgttcaaatccatttcacacaatttttaccgttattttttcatattatctgccaaa
attgaaaaagaaccccacaaaatgtcattattcatcctaattgtttgaaaaataacgaaaaattagactt
aagttgataaacgtggattttgacctttgacctgata
>KolobokP-5_CGi-I
ttgaagacaactaatatgtctttaatcaatgccaaattagcacagcaggtctgctaattaataatgaaca
tcatatcactagcaattacgaataggagcagataagccggagaatatgaggattgatctcccttggggaa
tagcttgcattgctctccatttcttagtaaaatatgaccaattagtggtacaagttaaactgtcataatt
tggtaacggtgggagatatttacttgaaattacctacattagttagaccgatatttgttccgataatttc
aataaaaaactgattctgaattgaatcagaaaaattaatatattgctaattgcttcgaaaaaacgtatcg
tgaaacatgaattttaactaaaaaatgaaatttgaaagtgttaatagaatctgcatctggtaaatttgg
tgccaaaataaaacccattaattacattttacagtttgaccacggacgggacccacttgctattagtgat
taagataatggcttctttatcggctaggggtgttgttccttgtgttgtcttcaaagccaggtctggtcgt

ttgaaaaggtcaaatgctggttaaaacaaataaataagacctgagtctaggtgagtagtattcagggtta
aatgttcatagaaccatactgcaattatcgatataactgttcacagtacatgtaaaatgggtaaagaaaa
cttaaatggctgtccaattttcaattcagaaaagggagtgtgccacacaacaagggaaagaaatctcaag
ggagcaattatcctgaatcgaagaaaccacagacagttaggctaagtagtgagctatatgatgaattggt
tacaattactaacaatggccagttaatgactgttaatgacgcagagggcagggacagcaacatgatgatt
ctaagaccccgggggagagagcccaggaccattgatttatacggttacagtgaagctgcaggaagcccag
aaagtgaaacttatagacttttacacaataaaagacttccgagttatggaacgattcatttaaggatcat
cgatccctgaaaccaacctgtgagggagtctagattggaactactctcgcgaccaaaaacgaggtctggc
atggataacatctctccgctgcgacaactgcgggtaccagagtcctccacgtaagctgtatgaggaggta
aagtccccaggtccagggaggaaagcggcacgcctcaacttagccatgtcggtgggtcgtatgggcacct
gtctgacgaccgaggggatgcgcggaatgctcctctcagccaatatccacccagggtcagccagtagcat
gcaggaaacggcgaacaaagtcggggaaaaaatactggaaacaaatcaacaaagtatgagaggtattcgt
caaaaaattcaagaaaacactacaaaatgcgggcttcctgcaacctcaactatacgagcggagggggacg
caagatacaacaattcaacattcagcgcagtcggtaaaactccttttcaggcagcaactcaagtgacttg
cactatgtgtgaaaacgtaaccagtaaaaaaaagtcattgcagtgttttgtggaaacaaactgtgtaaaa
aaggagaggtcctgcggaggagaggggcgaatgttacctgtccagaccacccagactgtactgcaaacgt
agctgcagataccaacatcggaaacgaaaaaaggtgggcgggagagtgcatccgtgaaatccagcgggac
ccccattctcttaccgtttcacacctcacaacggatggagacagcgccgccgcaacaggggcttctgaaa
tacaaggaaaacctatagagaacctgaaggacttgagacatttctttgaatcgcaaaggaaacagactag
caaagcgccgtttagcgcctcgatgtttcctggcagaaccaagactgaccgtgaatctgtacaaaaacgt
tttgcacaggatttaaaaatgcgttgcagaagtgaatacgaaaatgcatacgatcactatggtggtgata
tcaggaagttgacgcgcgcaatgtcctatgccgtagactctattctcacctgctaccgcggggaatgtgg
ccgaccttgccagaaacattcatttgcttgtaagggtttgaaaaagagccactggaaatcctcagtgtta
ccaaaaaactttaaattgaacatgaatgacaatgatgaacatttattaagagactgtgttaatttaactc
tagggccaaaaaatttgtgcaggataagatttcatacttccacgcaaaaatgtgaatctgttaacagggc
atacctaaggtcaaattcaaaatgtataacaagtagtagaaactttgaacctaaaatccatagagttgtt
catagcttaaatgagggatatgggaattctaccttaaaactgtgtgaagctgtaggggcacctgttgtta
agggtgggaaggttgtgcatctcctgaaacagcaagaaaaaagacaggcctattttagacaaagacaaca
atctaagttgtataaaagtaaacgaaaatattatagaaaggttagatatcagctgtatgatgagagaaat
gaaaataaatattgcaatcattaactgatcctaaattacccaagaaacgtaactcacgcgcagcacgtg
tagagcacagttatcatagaaaatgatattatgtcaaaatatattcaccgtgattagaaattaaaataca
cagtcagggtatgcaccatgtcctaaacatgtcttaggctgtctaattttgcatctctgtctattattgt
ttacatgctgaggttcgtatgagaggtgttgaatattaatgcaatgtgtgttgtgacagaggtgtgaaac
atgcatgtgtcgattaaaaatgtcaaaattgcgtgtatgtacaatgtaggaaagtctgtgaacgtatatt
cgggttttccgggcggctccgggcawtaaaactgttgtttgaatgtatttagaaaatgtgttgttgtgat
tgaagatttgacactgtgtattatgtaatgtttcagaattgtgtaataaaaaacgcatttgttcatcaaa
ccacgtattgtcaactgcagcagccattttacgaagatgaaaaggggaaaacccctttaaaaccggaaat
cttaaaatatcacttatcttcaa
>KolobokP-6_CGi-I
aacccctttaaaatggccctttaccccctgttttaagaaattcagtccattccgagtaaagaaaaaccaaa
acatcattcctactgccattcgtatttttttaataaaaactctaaaaatcacctatttttgtgtgttttg
tgttatgttctgtgcacagggggtggggttacattatcagctctcagagatacaaatagcataacatgta
aattacatgtagtgtatatgttacctaatatgtagtgttacatactgcacatgtaatctgaagatgcttt
aaagaggaaggagaccagaaaggaggaaaacgacaggctgaaccaaaaaaatggagctaatttattactt
tttcttcataaattttcatctaatgactccaatggagcaaataaaagagtccctgttaagcaaacaactc
tctgacattgttctatcaacaaattatgacctaaatctcctccctggtcctttttgatcgagaatactac
agatgtgtgaagaacaactacaggtcaaagggaggtaatttaaacaatcaccatgacaagactaatctct
gaaactgaaatggcttatctccttaatgcccaaattaagggaaatgtgttttgaaaaggacaagcaatga
acatcctatctgaatcaagctcagttcttatcttagaataactaatattaatgtccacacatccaacccc
agcctcacaagcatccccctaacagaatcgacattgtaaatgaccatttcacacctctaacaatgaagac
taacagttacaagaaaaatcagtccaggaaaaagaaagtcctatttaaggcgggacatacctatttacct
cctcgggtctcagcctctggaaacgaagaccaagcaagtactgcatcaggcggcaaagaagtacaccagc
cacagtggcttcgtccaagctatgaagagtactgggatgcggtgcatatgtcagcggatgacgactccct
ccgtcccaccaaactcaggccagcaaagtctgcaaattttaggttagaagaagacataataagttgtgaa
gagaacattattgtgaacatacagtcactagggtcactgttacaaaactctagtgatcatcattgtgaaa
atccagcaatgaatttttacatcacaaagagacagggcctatgcattactggcagagcagagtgctccta
ctgccatttcaagtccacagaagtgaagcttcatacaacatttaagaaaaagagaggcctgaaaccggta
ccttgaatgacggactcgctctggccctgacgaaatccaaactgggagttgcagacgcgaaactggtgat
gagctgcctaaacatcaacccacctgacggccgtggtctgcagcgcaagttgaaccagatgtgtgaccgg
gtggaggcgatcaacgaagcatccatggtggaaaaccagcagtacgtacggcgggtaaacacactcaggg
gtgaaggggacgccgtggacctggagacggacacctcatacaacaaccggccccaggccggatttgaagc
agcgacgcagagctttagtcccatggtggaggccagcacacctaggaaactggttgtgtccctgcagact
gcaaacaagctatgctgcaagaggaaatgtgaaaatcacaacaactgtaaaaataattactacaccgagg
actccatttcctcgagcgaagcaaaactgctgcggaagaatctggacttcattcaaacaagaatatcttg
accccccgctcagttaccagtgatgcctcggcccaagtgaaaaaaccatcagagactattcggaggacca
caacctgaacataaagcactaccactgcttcatccacaagctgaggaccctccagaagaatctccgaagc
attcgactcaaaaccagactggctgcgggacaagaccgagaagcctttatccagcgactagccacctgca
tccgtgccaggatcaggtataagctggtgaagattcgcagactgtgcagggtggatgctacattcctacg
tcatgcacagaatgctgtcctcaatgttttaccctgcttttcgggcatacacgacaagtgtaggagaagt
tcatttacctgttgtgcccatcttcctggctacactaccttccgtatggaaaacacctggacctggactt
tgccgacatggatgccattcagtccgtcatctgcaagcatttccgcgatgatggtctccggaaagtctgc
aagctggcaaccactaacaggtgtgagaacctccaccagcgtgtcttcacgtacgcgcccaagaacacca
cgtgggccaggaacttcacagccatgtgccactctgcagtccactcctcctcgcatggcaccggcaagtc
catggtgatcctagcgggaaaatggggcctgaaatggaagaagacaaaagaaccattttacaggaatatg
ctgcagaaagaccaacttgcaagctaccaccgtcaacgaaaacagtcctccatctacagaacatctcgat
acctgtcgagaaagaagaaaacaaccaaaaaactgagagaactgtccctgtacaacaatgcagggccctc
cacctcaactgaacactcatacggagtgaatcccctaccttagggaggaaagatatatatatatacatgt

atatatatatatggaatggcgaaaacgtttgcggttaataggatcaatttgtgattttattctggatatt
tattattttaaattatatatatatatatatatatatatatatatatatatatatatatatatatatatat
atatatatatatatatatatatatatagtaaactgtgcaaaaatacacgtaaacttttactcacttttaaaa
gtgacccaagactataagtctggatttccttagtgtggtctaaattgaggggcaagccacagttcccaca
ggcactcagtccttttgctggcacatctaactcaactattcgtctccctagtctccagtctttgcattcc
ttaatggtatgaaggttactagactccacaacttcttcctctctttctacatgtgataaatctggaccta
caatatatcagacaaatttaatgatatgtattaattaaccagtaactatgattacaattaatgaaatctt
aggaattttgattgtaatgcctagggctttcagaacatcaattgaatgaattctttaatgaatgtgaagc
aatcattttgatttttttaagcttaataaatttataaacaatttaaacagtgtcgttgtacatgaagatta
aagttatgttacactaggtaatgaacaaaacagctggtacattaccacaactcaaagccaaaagctttaa
atacataaactcagtgctatcattcctgcatactttgagtatctatatcataccatacaaaacaatgttg
attttggtgatcttaaaaatgtatatcttgcattcaatatctgcacaagtgctttcattttcatctgccc
tacgaaakgaattaatgactmataatattagccttgctcaatttttttttttcaattataagtgaattaaa
ttcaatttgagaattacatagcaattaatwagatctactaaacaatatactttttttaagttacaacttc
aaagtaattttttttaaaattaacatttcagatttcatgcctaaaactttgaacagctattgtggtttta
tgatcatgtacatacattgttcatttcggtgtgttaaaaatggagaaaaatataactgaatatgtcttct
ttat
>KolobokP-7_CGi-I
aatatgtaaacccatttatcaatctaatgatttttcaacatatcattatctattatacatcaccacatcc
atactaccaagtttcgtgaaattctgataaatagtaaaagttttacagactaataaaaattacatgtacc
cctccgtcattacatgtaaatatagctgttttcagacttgtcttttgattgacttatacacacaaaaata
ttaagttataccatgatttttttagtttaaattgcttcttagaactagatttaagaatataaatcaaaata
ttgagagtaaactgttaacttacataaaaaaatcagtatattaagttaaaaaagtacacaatttttctcat
tactggatacatttttactcaaatcaagaaaaattgcatcacaaacaccacgaataagggaattgcaaga
aagaagaggatatgtcgtttctaaaaaggtgctacatgtgaaatttggtatgttagaaatggagttatta
aacattcaatgtgtaaacctatcagtctaacctaactacatttttacgccaaaacccaacgtcatgatgt
catagacagctgaggcagccatattgaaatgtgatgtcatcataacaaggtaatttgaacttgtgtatgc
attgaaaatgagaggtgagagaaaaaagcacaagtttgtctgtggaaacagtataagaaaggggagagtg
ggtcacggccactctaaacatgaggtatttaaatctgtttggttaccacggttaacagctaatcaattta
acttaattactaaagtatcacctgacggtaagtcgtacactttgtccggagctgaaaaaagtctaggtaa
tgttaaggttttgcgcccccgcccaagtgatcccaacccttactcaagagtcaccagactcgacagccgg
gaaatgagggttgtagacaagggtctgacccttgacatgataaacgacctgtgtgtatcacatgctaggt
gtaaatgccaacttgctgattttgagataaaatccgaaactaagtggggctaggctggtcatggacatt
cgggtgcaagaggtgcaattttatttcgaaaccatataaattgtatcatgaggttccatctgagcactgt
ggcaggaaagctgcagaaatgaatctaggtatacaaacaggtatctaccagaccccattaggtaatgacc
aggccagacttattctggcatgtaccggcattccacccccagcaaggtctgcaatgtataaatcaggtaa
taaagtgggtgagaaaattgttgaaattgcggaacacgatatgaatgaaaaattgaaacaacttaagaaa
aagaatgaaatgttaggtcttccatcctcgcatccaatcaatattcaaatggatgcctcttatcaatcaa
ggggtatcaccagcaggcataaaatgggccagggagcctcacaagtaattggtgtagcctgtgaggatga
gacagaccagcataatgtgataagctatcatatggtaaataagctttgctgggttggagcatggctccga
ggagagggatatgatgttagctgcccaaatgggcatgtcggttgtacagcaaataaaaatcctgccgaac
cattgtccgagagggagacgggatacaaaattggggaaaaactggcaaaacatgacctactggttaaata
tgtgaccactgatggtgatgccaccagttgtgcggggttagaaaccgcccttcaaaacactctgtccccc
ctgtggaaaactagccgactagccgataggattcataggggggcaaagtctgtttcgtcaaggtattaaag
ccgaattcagtcccaaaatgtttcctgcacacactaaaactcaaaagtccgatttgcagaaaatgtttgc
caatgacatcaaagaacgatgccacggcatttttcaggcattgtttaaaaaacataacggtgacttgaac
aaaatttcaaaatgcctacctagagtcgtagacagggtcatcaagtgctacagtgggggttgcggtgaag
gttgcagatggagtgtaactctctgtaagggtggtaagaaaaccagctggtggcacaaatctgttggtct
acgggcacataatctagaaccaggtgacctaaagttaaatcagaaagacaaggttattcttaaatcatta
ctcggaatggttctctcgatctctgccttgaatgaaatgaaactgaacacaactacaaacaagtgcgagt
cagtcaatcgaaccatatctgtcagtcttcccaaaaacagaacttacagtagaaatgcaaaatcccgtgc
tctctcgggtctgttacgagctaacaatggggtagacatggcggtgtgtaaaacccttgcttccctaggt
gctccccttggtgcccagtctcagtcactgaaagctctgcaaagaataaaacaaatatcagagtatagca
taaagcatgcaaaatcactgaatcagaaaaagaagcgagcatgggctcgatgtagaaatgcaatacagta
tgtcaaacaaaaactaaatcggcctgtaaagactgactaccgaaagaatcagctagaaccgtcactctcc
ggtctggcaaaacaaaggtgtgagaatttagagaatcccgatcccgatcaacctggttgcagtcattggt
gaaaaatgaactgatatcaacttttaaactcttgtgttcttcaaatgtgtgtttaaaaaatacaaactga
tttgcaatgagtatgcaataaaaattatgtgcaatacttttaaacataaaatttgtaaagaagaaaaaaa
attgtagaccactgggggggaggggttggagatggcctccagtgctctgcatatttttttttttatatctt
tataaaattacagaatacataaatttaaagtgaattaaatctataaaatacataccattactcatttaaa
tgaagatttacaagacatgggggataattcacatgctgtctgcacacaccttcagacatacagttgaccc
gctgcatattaaccccatgtggttcaagtgagagatgatctgggtttatacataagcggttgtggcataa
atgtgaacaatcaagagatggagggggaataaatctgagtttaatcattaaagataatttagcgggggaa
taactatgccattccaggttcggaagtttaacttttatctgaccatatgactttgacctctgaccattat
gccacagtctacactggagattggccgatacatagctttttttcacgtaatttctcagagtatttagccac
aaaagcatccatgttgatgaagaagatgaacaggaagtggataaaataaaaacacacaccgttgaatttt
atatttcagataaaaaataaaaaataaaaaaaatcagatatttttgctacttacttaatat
>KolobokP-8_CGi-I
tgcaacaaaataaagaatttttttaagaattttataaaatgctgttttaggaacttgaaacatatctgtaa
ttgatattgtatatgaatttttgctttaaaggctaacaaagcactacaggccaaatgaaaaatgtgtatg
ttttgaagtgagtagtgttcaagtggacatttttttaagcattgcataggtgtttgtaaatttatgaattt
aataacttgtactttatgaaagagtatgaaattttcacagattgttgttgagatattaaacttgtatttg
cctgtgtttcatgtgattctttgagcagtgtatgtattttttgaacatttcattgtgtaaatattgctta
tgatatgataaaattatgaaatagatttattaaaccaaaacagtgtgttgtgtttttaattcaaaacatg
aatttaaacaaagataaataccttggcttccattttactgcaaagacattaaggatctgccactctgcca
ctctaataaaccatgaaggtccaatttggcatggtgctcacacaagagccaagcactggaggctgaagaa
acataaattagtcatggtcaattcataagatcctgacaacaaataaaagcagctgaatgcttgaccacaa

gacattctactgagtattaccagaaagcaagcagcaaattagaaaatgaagggacagagcaaaagtaaga
gtgcgcaattcaaagccaaggatgggcgtcgcagaggtcctggccataggggatggggtaaaaagaggca
gccctccaagtatttgagacctcaaacaagaacacagatcagcaagacaggaaaatgcattctgaaggat
gtggcctcagccatagagaccacaggacccatcatgcaactccgcccgagaactgcatgcacagtacctg
agaaggacaaccctagcaaacaggagcagaactcctacagaatgctccacatggggaagacgtgcgagat
gttcaacactgcctaccagcagcacaaactacagagtcctaaatgccccacctaccttgagtttgacttt
gacgctgaacaacaaaagggagtctgctggaaggagacccttaaatgccggtactgcaatttccgttcag
gacaaaataacctgtacgaagaaatgcagacagacagccggggtccaaagactgctaaaccaaacataag
cctatgggtagccttaatggacaatccaataatggggaccagcctacaagaaatattcctggccctcaac
tgtccagccccatcctacactggactgcaacgcaatgggggtaaggttggtcccaggatggtggagatgg
taaagaagacttgagacgtgaaagaacctacctgaaggacactctggaaaactgcggatacccacgcctc
acacccataccagttgaaggtgacggccgctacaacaacccactgtactggtctcgggacaggaatccat
tccagcctgcaacacagagtacttacaccataagtgaaaatgttacctctgataaaaaaatcataggtgt
caccgcaaaaaacaagctttgccgtaagcgtacaaaggggagcaggtgcccagagcatccaggaaaatgc
tcagcgtccctaaccatggatgcacccattggctgtgaacacctcgccaccgaggaaatctgtcatgact
tccttacagacagagaacccaccctcattagccatatgactacagacggagacagcgctgcattccgcgg
tgtgcagaaagcaatgagggaacatggacagaccgtggaggccctgagagatacccggcaccttgcccag
tcccagaagaaggcggcggacaatgcaaaatttagccagaacatgttccccgggaggaccgccactgaga
ggcaggccacaaaaagaaaattttcagtggacctaatgaagaggtgcacagctgagtatgatatagcaca
gaaaaaattctgtggggattcagaaaagctgatccacaccctaagctttgcaacagatgccatagttgag
tgctactcgggccgctgcggcggtacttgcacagaacactcactcgtgtgcagcggcctacctacagact
gctggccaaaggagtacctaccaccaaactcaaggacccttaaccccaccctggaggatgaagagaccat
acgccgcctcgtggacttccggtttagcaggagtaccatcaccactacccgatatggtacaaatactcag
aagtccgaggcacttcatagaggatactctaaatccaatccaaagaatgttacctgtgctgtgagctttg
agccccaaatctattctgccatccacaggatgaaccatggtcccgggaaatccaccgtcctaaagtgtgc
tgcccttggtgctcccctgcctgaagatacccgggtgacgagacagctagaaagaaaacaggaaatatac
ataacagacaagaagaggaaaaaatcatccgtgtaccgcacaagaagacgggccttcgtcaaggagaaat
tcgagatgtacttccaaaaaaaaactggtaccgaggctggatattccaagggaatgagtgatccccccat
accaaagagtgtcactaagaacaaaagtgaacactcgtacaacaagcaacaaaagtgacttacctctacc
gctcaaaaagacaatcaggatagccagcatgttttaagcagacttttgggactaaattcttacatagttg
tctctcattattgacatgtgcttcctctagacttaagtgtaatggatttatgcagagtgaattgtgacat
atgtgtgaaatctgtttatcatgtggaatatgtaatgtatttgtgtgcaacatgtacataaatctgtgtg
ccaggaaaactttttcttgtgttgtcaggtaatgtwattttgaccctaccatactttggcccctgattatt
ccccctcctccgacaatttgtagctaaactgttatcaaaaaaatcacaccccaaactattcacccttccc
ctatcaactttttctaataaaacttctaatttctctcgtctccattcagcatccatgatttttgtaaaaa
atccctttaaggacccagactccctatagatgctaaaattatcccatgaaactaaggtaaacataaacac
ttacctcccgagttttctctcttca
>KolobokP-9_CGi-I
tatacttatattgttacctacctcagaattagtccattttacaattttcaaagactttacacctacctgt
gtatgtgtatgaaatttgagcctcgggccttcagtgtcacggattctgtgaccatttcaatgatgccatc
caactgccaacatctgtgtaatatggagtgatgaactttacaaactctgttcctatatggatgaaaaaag
tgactgtaatgaataaaacagttttcaaatgtcttgtttttgttttatgatgttaataaaatgatataaa
cccatgtttgtgtcaatttcttcataagggtggattctacccagaatttgagagtagcctcaaaaaatca
aagaaatttaggaattaatgttaaaaatggaggtttttaatttaccaattagcaaaatttttgatgtata
tgagaggaaaattatccctgaccctaatgtagctagtgattcctatctggaccttcctgctgattaagca
tgtgtgagatgtatcaccacccccaaatctaatgcagcttcaagagatagcaggtgccccatcatcttt
gtttacaattaacttagacaagagaaagtccatcattgttgaaaacacattagagttcagacactggagc
ttcactctacacaaaaattaagggagacttttgaaatttaagactgaaaaaggattaatttagagaaatg
ccaaaattcaaaaggagaaacaattgtggctttaaggcaagaaatgtaccccacaataagggggtaaca
agtgaaaagttgcaaaattcccaaggacagtacaggagactgcctgaggatatggaccaaatggttcgag
actgtccgtcagcctccgaacaagaggaaatgctcctccggcccaactgctaccatctcctgaggccgag
gaagcttagggcaaaagatcatgaaagttgcctagatggaaatgaatcagagcagtaagtcaaattttca
aaaaacgagtaagacaggagatttttttccccaattttcttacatttaccccattcaaatatctgctat
atggttaattatccaagaaaatgaccttataagggtggggttcatgtatttaaaaattacatgtaattac
atgtaggtacatgagaagtcaagtgtgatacatgcagctaaagggcatttcagtaaattgagacaaaaac
aagaagcttaggttgcctaaaactgaaattacatcaatttgaaattgtacatgtcaagcctgttagtcta
agggaagtaactctaataactgctctcatgctctgcatactggtgactgtttcaagtaaacaaatcattg
tgactttacctaggctggctgatcaaaattcacatagactcctccacctgggaaaaacagctgagatgtg
gaatgaggttctccgacttcaccagcaggagacacctgaatgcactggacttctgtcctgggacttggcg
tcggaagagaggcggggacttggaagcagaatggggctccgctgcaccaagtgtacctactcgtctaggc
gttacaacctgtatgaggaggtagataccaaatccccgggtagaaaggcagctaaaatcaattatggtct
gcaagttgggctcagtcaaactcctgtgggtaatgatgggatgcggaaaattcttctctgtactaatact
cctcctcctgctcgtaggtccctacaaaagcttccaataaagttttgcccttgatccaaaacatcaacga
ggctgacatgagtgacaggtgtcgccggctggtagatgtgaacaccctgagggaaatgaatcccacatgc
catccctgtacaatgcgatggcatgtacaataacccccctctactcgggggtgggtgagacacccttccag
ccggcgacacaaactgtgtactcatttgctgagaatgtcacctcaaaacaccaaataattaaaatggtga
ctaaaaataagatttgctcaaaacatggtcacttacttgatgagggctctcgtgaccatcaatgcaaccc
cggggtctgtggggcaaaccttccgatgcatcatacgataggggatgagttcacgtgggccagggagggt
atggcggagctactctgtgaagatggcctggaggtaagggaagtcaccaccgatccagacagctctgcag
gtcgagctgcagactccctctataaggatggtctgctggaaaataagccatgtcactatattgacactcg
tcacctgtctgagagcataagaaaagcaatcaaaagagaccagaaactcttggagatcatgccgaaaaga
accaaggcagaaaaaaccaaacttttgcacaattttgcccttgatgctgttgacaggtgtacagccgaga
taaatcaagcgcatgcactatatgcgggtaacgcagataaaataaaaaacaagctctcttacaccaaaca
tgcagtggttaagtgttacacaggggatcatgccttatgcaaaaacactcgttagtgtgcaaaggagggt
aaataataactgggtacgcaagagttcttacctaggtcaaaacttttcgataccgtcctccgacgagaat
gaaacaatgctccatgcttgtttctcaaagcggttgtctccagctgtcttggacaagacaatcaaaacag
taattctcagaaggtcgaaagcttcaacagaactctgcgacgatcactgcctcgcaatgtcaccttcaca

agaaactttgctggccgggctcacagtgctgctcactcctgtaacaacggtcccggcagctcctccgagg
tctctgcgcaggggttggagctcccataccctctggtggccaagtggaccagaccctagataaaatacaa
acagattatgagttaaataaatcatatcaaaagtctttatcttacaaagcaaaaagagtaacaaaacgaa
agaaaatgtacaaactttatgagaaacatcaggaggaaattaaatataaaaaaaacatgatgttgactga
atctcgagcccgatcatgcaaaaagactcatacagaacacaattattccaaaccagatcaataattccat
gattatattttaaaaacatcaaattacactaaaatatgcatcttggatggggatggtgtgccccactgca
taccccctgctgcatgcaatggatcctctcctgattcgtggcatgaggctccagtgagaggtgcatcggg
ttgacacacagcttctcatggcagagatggctgacctccaggtttccacctggaaactgtgacctcgtca
gacgcatctccaccataagagccacccgatgcactctctcaagcttgctcccctcatctggccataaaac
ccttatatacccataaccatttctcccaacccccccttccataagacacaacccccccagacccccaacc
ctttcacttttgcttccaatttgattctcaatttgttgaaaacgtcctccatcatggattctgtaacta
aaaataactactttcttctttaaagtcacatgacatgtcaaaaactgggtctctata
>KolobokP-10_CGi-I
tatactcataattttactcacctcagaattaggcaaatttgtgtttttcatgaaccttatacatacctct
tattgtgtgtgaaacatgagtcctggatctgcagagacatgtatgctgtgctcatccgagtacgaggccc
tctctctcggcaaatgctgtgatgagagtggaccgactgtgaaatccagaatgaatcaactgctttttga
gtaaaatttgaatgaatgcgtttgttcgtttgtacttaagggtgttatgaacaaattggatatgattttt
caataagggtggattctacccagatttaaagaaatgatgctgaaatttcaatttaagatgcaaaattaag
aattttacattgagtaaattagtaaaaaattatattcttatgaactgtttactatttggaaagctgatga
gcaaatcatcccttatctctgacccccctgcagttaatataggagataaaacttttaccacccccctacatg
gcagagcttaaaataatagccggtcccccatcttttgctacataaatcattcacagataaacaatagccc
aattttcggatacagtataacagcttaatctccctagtgcgaaacagcatcaacaattaattaggaattt
agaatttgcattcagaaaaggcttaattttcaccatgccaaaatttcaaaaaggacattcctatggattc
aaagcccaaaatctccctcataacaaggggaaaaaaagtcaccaggaggcttacataccagctacatata
aaaggttgtcttctgagatgacagatctggttaacaacccccccatctgcatcagagagggaggaaatggt
ccagcgtccgggaaactaccatctcctgagaccaaggggggatgcctgtaaatgtgaagtggaaaatcat
caagaccagaattttgagcagtaagtcatcttttgtcagctaaaatgaatagaaatcacaattttactta
aacttttgtgaaattaacatttttttttacaaattagaccctttatggccaataaaatggggctctcggac
agtaggtccatttttttcaggttttacatgtggctacatgtcactacatgtaatctggggtgataaaacat
gtatttataattcatttacctcacgtgtatcagaaaaattggtttcagagcagtttatgttaaattacat
tattttcaaaaatctacaaaatttacaactagtctaagggggggtaactctaaaataaattttccccatct
tcacaacacacacagttacacactatgtaaacatcagttacaattattgtagagcggagacagattctaa
cagattactgcacttggggaagaccggtgcattgtggaaccaagcctttcggagccatcaggagcagaat
ccaggctgcaatggatccctgacctggaacctggccaccgaggaaaggcgagggcttgccacaaggatgg
ggctcaaatgcggcgtgtgtcccttcacatcgaagagacacaacctctttacagaagtagagaccagtgc
acccggcagaaaaccagcttctcttaattactcccttcaggtcgggctgagccaaacaccccttggtaat
gatggaatgaggaaaatactgctttccactaacactcctgctccttcccgtaagtccctgcaaaagacca
gtaacaaagttttggctaaaattgagaccctaaatacagctgacatgagtcgcaggtgtcgccagttggt
tgacgtcaacaccattaggggacaagagtcgccgcagtctatagacgtgcagtgcgacggcatgtacaac
aaccccttgtactcgggggtcggtgagacgccattccagccggcaacacagactgtctactctgtcgctg
aaaatgtaactgcaaaacatcaaattatcaaattgataacgaaaataaaatttgctcaaagcacggtcac
ttacggatgcggcatctgctaaccattcttgtagtccgggggagtgcggcgctaacctccccatgcaga
atactataggggacgagtttacgtgggctagggaagggatggccgaacttctttgtgaagacggtatcga
ggttaagggggtaacaactgacccagacagctcagctggtcgggctgcagactccctcttcaaggatggg
ctgtacaaggtcaagccaatgcattacctggatactcggcatctctccaaaagtatcaggaaatctataa
aaagagatgaaaagctcttacaagtcatgccctgcagaacgaaagctgagaaaaccaaacttttgcataa
ttttgccttcgatgcagtagatagatgcacggccgagatcaatcaagcgtatgacttgtatgctggtgat
agccataagatgaaaaacaagctctcatatgctgtggatgcaattgtgcagtgttacatgggtgatcatg
ctctatgcaggaaacattctttagtgtgtaatggggaatagttaattgggtaagcaaaagtacatatct
aggttcaacttttgaaatacctcactcttcggagaacgagaacttgcttcgtgcttgcatatcataaagg
ttgtctccagctgttctggacaagaacatcaactctcagaaggtcgaaagcttcaatcgcactcttcggc
gatctttacctcgtaatgtcacctttactcgcaactttgcgggtagggctcatagtgctgctcactcttc
aaataatgggccaggtgactccatccgcagcttatgtgcaggtgtgggttgtgccatccccagtggaggc
tcggtggacaagtctcttcataacattcaaaataaccatgaaaccagtaaaaaaatatcaaaaatcaact
ctatacaaagcaacaaagggtggttaaacgtaaaaaaaaatctacaagttatatgaaaagcaccagaaaa
atatgatgctctctaaatcccgagcccgaggcaaaatgaagaggcattctgagcataattattcgaaatg
tgattaaattgtgtattacaatacatattaactaattacatggtagaatgcaatggggatggtgggccct
gcaacagaacccctgtgctttgcagtggtttctctcctggttcacttcatgcgtctccaggacgaggtgg
gcggcgttgacgcaaagcttcttatggcacagatggctgacctccaggtttccacctggaaactgtgatc
gggtcaagcgcatctgtgccataagaatagccctatgaaccctctcaaactttggtccctcctctggcca
tctcactcttaacactccatacccatgggcagtggtggcgcctttccatacaaaacaccccccctggtcc
cccctgaatttcactttttttccaaaattctaagcctcaactgttcaaagaaatcttccatgatcaaatat
caacagaactaaacttcctatggaactatttatccttaaaaagtcacacgacaaggggaaaataggctct
ctata
>KolobokP-N1_CGi-I
aatataaaacagtcattgtggttatttacaattcttagcttatatattcactctagacactatacagaac
catgattccaaatttggaaaaattcaatacataactatggattttgcagcattgatacacatgcatgaaa
tttgaagaaacagtccagtcataatttacctaaacaacatatagctcattggcactcgatgctcttcaaa
ttgcataaattgaggaaactttaatctcaaggattaaaatacatgtaaaacataaacattcaatatttta
ccaaatttattttgttcatattcttatgaaaaagctcaattatgtctgattttataaaattgttgtcgta
aaaatcatgagtttttctttagttacaaaaaagaattgttttcaaacaccacgaaaaaggtaaaatagaa
aagtataggaatttccctatccatcaaggtattacatgtaggtattggtttataaataataaagttatat
gactctataatgtgaggccctcaattcagtttttgctccattttgatgctagcttgagattacctcacct
atgacgtcataatgacgtcatagtgatgagttgtttttactcatataagtgtaatatttgctgaactttt
atttaagcctaatttcggaacaaatgcaccatgacttaactcattaaatatatgtcatagggaaaagaaa
cttgctaataaggatttttttcttttactcttgtatattgttaccatggtaacaaatttaacaaaaattat
caaaagacatatcctgtggaagagagtctacatttaactaaagttcaaaatccagaagaacaagaaatac

ccacaaagtctgtgaaatatgtataatcaatgtaaagtttcccagtatctataatattctaaattatctg
catgtaaaatactggccatgcagggtatatagtttttataggttccttcttttaaacttggacgagtgaa
gttacaaaaataaattttacttacatcaagtgtggctcattgttgtgttttattctttgaagaagacttc
catttcaatttacatgttatctaaaatgaaatgttttgaattaaattctacattatccaacactattccc
aacatttgcaattccatgtcaaaagcagccatatgttgagagagagagagagagagagagagagagagag
agagagagagagagagagacagagacacagacagacagagagaattagagagaggggaaagatacagaa
agagagaacagataggcaacaataactacatcttcatgcacaacacatctatacagcctaaagaccaacc
cccccccccccccaataacattcaatatcaacccccaccccaacatattgatctatttttcaatagt
atgaataaataaatactagtaaatatctacttaaaatgattactgataatttcattcaattttgattatg
ataaacaaagataggttttgtggattaagtactaaccaacatggtaggcttctacatgttaaagagtgca
tacctacatcttcggtacatagtcatgtcacatgcatgacaaattaatttactccaatttaatgcaaatc
tatcatcataaatatagaattggaacctaccggacactaatacaattcccttacaatgcaaactaatctt
agatagctggacactgctagaatgaagtattttcctcaccggaaactttctgcttttctactttcactt
ttgctcgcttgaaatgatcgcactaaaaacccgaagtgatatttggtctcaaaatttataaaaatgtctt
ctaaactcatatataattagaaacacacctacattttcatttttttaatgtaaaaactcaaaataaatta
cattcaagttaaaatcacaatttctttaatttaaattggtttttgaaaatcccgggggggggggggggg
ggggggggtgttgtgatggatattttcttggaaaatatgctattaaaatgattaaacttgttgaacttgt
tggaaatttagtttattcacataaatgagaagatgtacaaaagaaaaatgcacgtttatcataataattg
caaataaacaagaaatatgccttaccagcatggagctcaatat
>KolobokP-N2_CGi-I
ttcaagaaaatgtcaaattttaataggctttaagtttaacaacatgggaacagaaaagatgttcaatgtg
tgtaaaatgggaaactggctgaatttttatgattgctcaaccctgtgatgagatttaggggccaaaatgt
agcaggggtggtccttagaaaagtgcaattttctagtattttgggtttgtccttatattaaaaccctta
taggcatattattcctattgaaataagatgttgggttgaatttggattaactgaataatattgtaccatt
ttcatgaaattttcttgaaattaattgacagccatgcagttttatgaagttttaaacccttagatttgat
aagaaattgtaaaatattgttctttctcaagctaaaactgacaaaaagttgattttagggctaaatatat
attttaaatgtgctacatgtaatttacatgtaataacatgtaattctaaatagttaatatctgcatttta
agtcattataaacaattttaaaatgtctgattgtcaaaaaagcacccccccccttcaatttcttcaccg
acaaccaaaaatagcctttatttcacatttcttttgataaaatctcatgcatacataaaatttctctcat
ttaaaacaaaacacacatgtttggtttcttctttattgaaaatataccaagaaaaattgaatttgtgtgc
atttttaaccatttgtcatttgcatgtattcccacctcattatggggtgtggtcattatgttcaaattt
ggtatttctttgtatgaaaaacatgtatttcaatctttataacaaataaatctagatcttttggttttgg
ttaaagaataaacaaagaaattaggtttcatcatcatccgttcagcatatttgggaaattactcctcggg
taagatgcctgattgccattttctaggtacctgtcccttttgaaaagatgaaagtgaaggttttgtgttc
caaataggttgcttttgtgttccaatagagtttttaaacctgttagtaccatttagaaacaaaaaagttg
attgcaaggagtaataatgtgtttttttcttattcccatttgcaggggggataggtgatacatatgagttga
attctcctatatgttgttactaaatgtgaatatttcaatgtttttacatcagatagggataaaggtttct
tgttatgactgattttaaagaaaaatgtcttttggattgaaagaagtcatgatgtatcttaaaaacctt
acagaaacaggttaaggtgattcagccggaaaggggtaatacttaaacagccataactttttattttc
aatgaaatcagaccaaatttgaaaggttttactcagaattgcatgaactttctgatttaaagtgttttgg
ttagagagaaacaaactctaaaagttaaa
>KolobokP-N3_CGi-I
ttgaagggctgtgtgtcaaattgaaaaaatgcatgcaggtgccagttttcagtattttatgtcaaatctt
gttaacagaaattagtttctgtagtctgaagatagaaaaatgcttttaaaattttgtaatgaaaaaaata
tgaaaaatgatgatttttggtgtggaaaacatgtaatttttaatatataataataaaaacctttaaaaat
ggatgatttgtagtcactggagcttagaatattggtttctgtgttctagatgctgtaaaagtgtgcagat
cagttgtcgcagtcatatttgatgccctcagactttgtcgtaagagcacgcagcgactagtttttcatcg
aatgaccaattttgagtttacgtgcaagtctcagaattaagcgacaagtgcgaaacattgcgaagaagaa
catgaacagtctatcgaaggattattttgacaaaaactttaatttctttgatttctgctcaccttatgtg
agttttaatttcatttcctacatagtcctgattgaaggaattaagggatttgggcaaatatgtgctatgt
ttatgtttattttacactgaattacccatttgtaatagtttaaggcaaaaacatgctttaacaccattta
caagatcacacagatgcatattttgtgtagaaaagaggtttcatgcatttagttgagaaggtcattatga
gtcaagtatatataatatcatcatgattgaatctaggagcttttcatggttttcatccttaaaaactgat
gaatttcagtttaccagaaaatgtttttatgcattatagccagaaaatcagtttcttcccacattttaag
ggagttccttatttacaaaatacaaatactgatatttatgtttgaatgacctaagatattgaaacattag
tcattgttaaaatgtttagaaggagatattactatttttttatatggtaacctcagcacacctgtcctatt
ctaagtaatgaaaatcaaaatttgaatgaaatcatatcatgtaacatgtatgggtacaatatagatgccc
cacccttatccacaactagkggggttggtatgattctttttttcagtattgtgatatggcatgcacttatg
tgatttctcttgtaaatatgcatcagaaatgcattgccttattgaattactwcatgtatatgcttattgc
tcttgctgtaaattatctaacatgtgtcaatcatcattgatcagtaattctcttcaaattctatagggat
ggcctgatgaggggtataattaagtgtgtccaattgaaatttcatggctacttttgctctacagatagaa
tgttacataaatcacagaatgaagttgcatcccagttgtttcttttacatttcttatcatcaaagcaaga
tttatggttgtcagagcagatctgagctccattacatgctgatatcatttgggctgtcctgtgttaccat
gctgcttttagataaaagtgagtatggcagtgtgtagacaagtaaacaggctttacctgatatataaatt
taccagatacagatttcatttttgacctgataattcacaatattgttatgaaatgtgtgaacaatagggg
taaatttcttctttgttatgcaatgattgcataatgaagtgactctaatggaatatttttattttgatgt
aaagaaaaatgtatatgctctgatttggtaccaagatgattttgtatcttgttgttttaaggatgaaaat
tggcttcaggtcatgcatggtgtgaggctccaaatgtgtttcatctgtgctattttcttcagtctgagta
agggagataaatcctcaatagctaatgaaatattgggagcttggggacagggttttagggatttggtgac
agttgattgggaattctcaggtgcaaatttgaaagggatctgacatctttagagtctcttca
>KolobokP-N4_CGi-I
tttaacatacactaacattctctgaaaagtcatcttgtcttaaaattaaaaagcttatgctattctgaaa
aaaagtacaccacatttttgccaattccattgaggtttaagaaaaatatggccatttaagtgaacccacca
tttccactttcctctatttaacacagattcacaggctctccataaataaagcatctttacctaatcttt
tagaggtcaactgttgttcaacctccttaaataagaaaccttattcaatactacatacatctgcttgata
ttatcatgataaaagcatcacatcacttagaaatccctttacatgtataccctcccccctatcttttgatt
ttcacaagaagcattagtttgaacactttaacctaatattatgaaacttgtggcagtttccttgagtcat

ttcttacacatatttgaaaacaaccatcactgatatttaaaattgatctttttggtaaatggatgattt
gtagcatttttattcacaaaaagtcatgtcgccctacatgtgatttcttgttttcatgaaaaactaagcc
tataatcatatttagtggatatcttatatattctggtttctagtctccttttaactttgctaaattagta
agacctacaagtgtgatgtgtgcgtttaattaaaatgaaattcaaacacacccgggtacctggggacgga
tgagaattccatgaaaaatgttcaaattttacatgtttttctacatttttgatgcatatatacaataaaa
gggtaaaaatatgttgtttttttgttcatttcaagagtaattatcatagcagatgttatttcaaggtcaaa
tgtacatttacatatcctacatgtaatggtaatggagtccattggaaagagggggtggctttttcaattc
tgtaaatacatctaaaataccatttttttgataggaaggagcaaaaacttgagtttttttgacatattgtat
actttgataactgcatttgtctacatgtaaagttcatttgaaataaaaatatgctgttttaaaaaaattg
ggtgatataagtcaggtggmttaatgttatttcataaaatcagacagcaaaatggctgcaaattcataaa
tttaatgatttaattgataaaaactgttttaaagtatttattcttatctcagtaattaacttaagcctat
taattgtcatcaggataggaaatgcctactctctaagccaatttactctagtggtggtcattctacacat
ttaagagggagttactccccttgaatattttagctaataaatcatgtcatgacatctatagccaagaaaa
tcatccattttcacaaaatgtattacttatggtacaaaatccactcaaaattacacttaaaggagaaata
tgaaaataccatgtagtcttgaa
>KolobokP-N5B_CGi-I
tgcaagtaatctattattttacactcgaaaaataaaatctgaaaacacaaaacatttattgtggaacaca
tgtggaaagttttactgaattctgtagctatatcaaaagttatgagcgattattaaaaccatggtgctcg
cggtggcctctgatttatgaaaatatgttaaaagttatactgtatgtaatttcacggcgtgttaattttg
aatgattttcttggttctaatttgtgcaaatatgcatgtctattcacaagacaatgcaaacttgtttata
tcatgacgacccgagaatatatacatgtacaagtttagcgaaccctgcctttgggggaggggttgaaaac
tgccgttatattattcaattttaaagaattggcgtgcattttcatataaattagcattgtcaggttctaa
taaaactaatgcgggtgttgaatcatagctaaattcatattttactttaatattggcgttttatgggcat
aattttatagattctgtataccatacaagcgtctaaataactcatttacacaaaattcgtattacataa
aagctttttggtttacgacgataaaaccaactttctgataagctgctgtataattttggggcagtttgga
cggtccgatacactataatcgggtcgctgtttgatgttctacgaaatttcagaggaaatctgggctaatt
ttgtatgcgccattgacagcaaaataagagaggtgccggccacaaaggctcattgttggtgatttctaat
ctacacacatcgatagaacgccaaaaactccactttatgacttaaaacacatcataacaattttcctcca
agtaatctattattttacactcgcaaaaataaaatctgaaaacacaaaaacatttattgtggaacacatg
tggaaagttttactgaattctgtagctatatcaaaagttatgagcgattattaaaaccatggtgctcgcg
gtggcctctgatttatgaaaatatgttaaaagttatactgtatgtaatttcacggcgtgttaattttgaa
tgattttcttggttctaatttgtgcaaatatgcatgtctattcacaagacaatgcaaacttgtttatatc
atgacgacccgagaatatatacatgtacaagtttagcgaaccctgcctttggggaggttgaaaaactgcc
gttatattattcaattttaaagaattggcgtgcattttcatataaattagcattgtcaggttctaataaa
actaatgcgggtgttgaatcatagctaaattcatattttactttaatattggcgttttatgggcataatt
ttatagattctgtataccatacaagcgtctaaataactcatttacacaaaattcgtattacataaaagc
tttttggtttacgacgataaaaccaactttcttgggataagctgctgtataattttggggcagtttgga
cggtccgatacactataatcgggtcgctgtttgatgttctacgaaatttcagaggaaatctgggctaatt
ttgtatgcgccattgacagcaaaataagagaggtgccggccacaaaggctcattgttggtgatttctaat
ctacacacatcgatagaacgccaaaaaactccactttatgacttaaaacacatcataacaattttcctgc
aagtaatctattattttacactcgcaaaaataaaatctgaaaacacaaaaacatttattgtggaacacat
gtggaaagttttactgaattctgtagctatatcaaaagttatgagcgattattaaaaccatggtgctcgc
ggtggcctctgatttatgaaaatatgttaaaagttatactgtatgtaatttcacggcgtgttaattttga
atgattttcttggttctaatttgtgcaaatatgcatgtctattcacaagacaatgcaaacttgtttatat
catgacgacccgagaatatatacatgtacaagtttagcgaaccctgcctttggggaggggttgaaaactg
ccgttatattattcaattttaaagaactggcgtgcattttcatataaattagcattgtcaggttctaata
aaactaatgcgggtgttgaatcatagctaaattcatattttactttaatattggcgttttatgggcataa
ttttatagattctgtataccatacaagcgtctaaataactcatttacacaaaattcgtattacataaaa
gctttttggtttacgacgataaaaccaactttcttgataagctgctgtataattttggggcagtttggac
ggtccgatacactataatcgggtcgctgtttgatgttctacgaaatttcagaggaaatctgggctaattt
tgtatgcgccattgacagcaaaataagagaggtgccggccacaaaggctcattgttggtgatttctaatc
tacacacatcgatagaacgccaaaaaactccactttatgacttaaaacacatcataacaattttccttca
cc
>KolobokP-N5_CGi-I
tgcaatataactcatttttgttatcgcaaaatcaacattaataaattgattaatactcattgtgttgtgt
gactaaaaaaaattatgaagatatctactcatttcagaaagttatgagcgatttttaaacaccacggtgt
acacggtggccactgatttatgaaaatatgatcaaagttatactgtatgcatttacacggtgttttgata
ttcagtgatttttttggttttaaattgacaagtgatgtatgaaaatgtataaaatgacgcaaattaattaa
tatcatgtggaccccagaaattatgtcggccaaaaattcaatgaccctgcctctgggggtggggtagaga
aaacgtcattatttcatacaatttatgaaaatggtgagaacctttgtccaaaatagcatggtattgttct
aatctaactaacatgagtgatacatctcagctgttttccgctttcgtatcaatatttctcaattttggac
ataaattgagcatttcttactgtaacacaagggcctaaacaaccgattttatttgatattcacaccccgt
agaatattttgttgtttacgacgatgaatactacatcgtatttgatctgctaaaaattttccggacagtt
tagacggtccgatctactataaatggctcattgttttgaggcagtagaacatttcagaggaaatctgggc
taattttgtatgcatcattaaaagcaaaataagcgtgctgctggctacaaaaccccattgtttgtcattt
ttcgtctaaacacactgatttaacacctaaaacacaggtaaaacaacttttaacatagcaaacccgtttt
cctgca
>KolobokP-N7_CGi-I
tatactcataattttactcacctcagaattaggcaaatttgtgtttttttatgaaccttatacatacctct
aattgtgtgtgaaacttgagtcctggatcttgatttatatttatgctgtgctcgtctgagtatcaggctc
tctccctcggcagatgctgtaatgagagtgggctcacagtgagactcaagatgaattgactgctttttaa
tgactttaacaaataaaatacatggaaaaacggattgaatgtgtttgtttaaatgtattaaagggtatca
tgaacaaatttgataatttttttttaaataagggtggattctacccagaatttgagaaaacctgttaaaat
agatgaaaacctggtgaattatacaaattaaacaggctaaatattgacttwtatgtaaaaaatattcaca
tcatgaaaatttaatcattattgactctgattagccaaataatcctaatctggcctttcctgttgattta
gcaggtgcgaacaatatcaccaccccccagtctaatgcagcttagagaactagcaggtgccccctgtcat
tttgtattacagtaattttgaaaaaggagaggtacatatttttgacattatattacagctcagtcattgt

agtaataaacagaactgataaattgtaaaatttttggaatttacacctagaaaaagagaatttttttgaca
atgttaaaaaatctagaagggtaattgttctggtttgataccacaaatatgcctcacagtaaagagacag
agactagtatactaataattttacctgcatcatgaagaaaaatatctgtcatgatggatcagatggatac
ggactctctgtcagcgttataaagkgacaaaatgactccgmcgccctggtgccaccatctccgaagacag
aggaggcaaaaaaatgaaaatcctcctaagtgcacgaatgagagaaaatcagtgttacaagttattcatt
gcacaaaccaatgtttatacatcaatttttatcatacatgccttacaaatatacatatatatcattatta
cacacataaatacaaaattaaatgattattcaggggtggggtacataaaaagcacaattacatgaaatta
catgtaattacatgtattaccacatgtgatttatgcagtcagaataacattcaagtaataaatttaaaaa
taagatgcagctagtctaaaaaaaaagaaattgcatcatgttgaaattctgcaacactgaccagtaagcca
aagggaagtaactctaaaaactgctgtcttgttctctacactgttgacattttcaagcatttgaaccatt
aaaacttcacaaaaagtccccaccaaactcataaaatattatcatgcaaataaagtatattccatattca
gttgacatgagtcccaggtgtcaccccaagactagaaagtggtccccattctgtttcatgtgtaatgatc
atgtaaattaaacattccctttccmaaccctgggggggcactcattctacattccagactagacatattc
ccaaactctaaactgtattccttctagcccaaaaactccaaatcattgaactggggataaaaaaaaatgaa
acatggcaacctaccagatgctgacactgttgacagtggatgtagtctcagggtgtgtgacgcaaacctt
cttttacatcaacctcattgcacgagaccacttcaggcttgaaagaagactgcggaactcctctgcaagg
atggctgaaaagtatgggaggcagtatttgatccgaatcactctttcgatcaagctgcgcaatccatgta
caataaatggtctgctgaaaaataactcagatgaactaaattggcattggctagcatcatttaatctatt
taaaaagactatagacacttctgagaagtcaatggtaatatggagcgcacaaaaataacagaatatcaga
aaaaagtctgagttaaacctaagtttggtccagattctatattgaacagatggtcagtcaagatttatct
caaaaatcttgtacaattaaaataaatatacaagcaaagaactgcaataatggagtgcaacacaggtgat
caacaattattttatccaatttaccataaaccccacatttttttgcttttattcttgcctggtcaccctt
ccattaccctttacactcattcagtacccccccttattttgaaaatgacagccactcccaccctcaaattt
acatttacagtattaattttacttctactgttttaaaatgttatatctgctcttttcaatatgaaataat
taacctttttcaatttttaagctacaaaacatttaaaaaatagggtctctata
>KolobokP-1_CVi-I
ataaatggaataatttttgttatttttctcaaagtgtaaaaataagaagttgagatatatctgtatttga
ctttaaaatagtttttgaaattttcatgtattacatgtaattcaggccatgtgaaaaatgtgactgttta
ccagattttgtgtgtaaaatgggcagttttgaggatgtgggaggggtattgaacttttgatttttttgtgt
acttactgtttatcaaggaaaatgaaatttttgtatgatgtagctgaaatgttgaacttgtgtgtatgtt
tgtttttaattctataaagctatgtatttgtgtgctatagatgttttcattataaattttgtgtttgaaa
catgtatacttcattaattaaacttattaaacttaaaaatgtgttgtgtttttgattcaaaataatgttg
ttgggaaagagaaataccttgtctttctttgttctgaaaaacttgtcaagatctgccacaataatcagca
tgacttcatgattatccatggtctgccaaggagagctggctaaggggagactgtcgaaataaagattaat
gtttaagcattcaaagtggattacatcaattgggtacataaactacatcactacacttctgagagcatta
agagaaagatcagagtattatacaaacaaattaattagaattcatcagcaaaaatgagaggaaaaagcaa
gtcaaaaaggggggatttttgcaccaggcgatggccgacccaggggatcaggccacgccggatggctaaaa
atgcagaaaaagagcaagtacattagaggggaggctgtaaagaaggtcagcaagatgggggcaagcatac
tgagagaccatgctaggagtgaaggcaccaccatgcaactgcggccaaggagcatctcaacccagtccga
mctgacccacaaccccccaaactgaatcaaactcctaccgatttttccatgcggggaaagtatgcgagctg
ttcaacctagcctaccaccaacacctactaaccagccctaactgcccaacacgcctggagtttgactaca
ccagagagcaacaggtgggagtctgttggaccgagactctgaaatgcacgtactgccatttccgaagtga
aaaaacaaagctgtacgaggaggcaagcactggaaaaagaggaaaaaagccggccaaacccaaccttgcc
ctatggacagccttaatggacaaccccatcatgagcaaagggctgcaagagattttccaagctctaaact
gtccagcaccatctaccactggattacagcacaatgccaacaaagtggggcccatgatagtccagatggt
tcaagaggacctgaaaagagagagagctcacctgaaggacattgtggagggttgcggttatcccagggat
acaccaatacctgtcgagggtgacggccggtataacaaccccctgtactggtcccgggataggaacccct
tccagccagcaacccaggcctcctacaccatctctgaaaatataacagaggagaaaaaaatcattggctg
taccacacgaaacaaactatgcagcaaaaggaacagaggtgaaaactgtacagagcacccaggggaatgt
gccgccaccttaccctaccagcatcccataggcagagaggaccaggctgttgaggaaatctgccgggaat
tcctcagtgatcccgagcccaccttcattagccacatcactacagacggagacagcaccgcattccgtgg
ggcccagagggcaatggctgagcgggagcagacggtggaggccctgagggacactcgtcacctagcccag
tcccagaagaaggcggcagacaatgccaagttcagccttcagatgtttcccggcagaacagctgcagaca
ggcaggccacaaagaggaaattctctgtggactttatgaagaggtgcacggccgagtacgaccaggccat
aaagaaataccggggcgacacagagaagcttgtcaacaccctgagcttcgccactgatgccataatcgac
tgctactcaggcagatgcggacgtacctgtgcacagcattccctggtatgcagtggtgctccggaaaagt
gttggccaaaagagtacctcccacctcaaagcaggacactacatcccacagatgatgacgaggatgtcct
gcgtcaactgataaacttccgcttcagccgagcaacactgacctccacccggtacgggacgaatacccag
aaatcagaagccactcaccgagggtactctaaatccaaccccaagaacgttacctgtgccaggaatttcc
aaccgcagatcttttccgcaatccaccgcataaaccacggaccagggaaatcagctgccatgaagtgtgc
tgctcttggagccccaatcactgaaggtacccgagtatgccgtcaactacaaaggaaacaaaagaaacat
gagcaggaccgagcaaggaaaaggtcgtccaaatacagggccaagagacaaactctggtgaagcagaagt
tccaaatgtacttccagagggcagagaatgagacccaagcaggctacctgaagggaatgagtaatccccc
cttacctgattccaagaagcagactggagagcattcatacagtaaaatgaagaaagtgaagtgacttacc
gttttcaaaatagacagtccggaaaaccagcatgcttcaagcaaactttagggaccatatttttgcataa
ttgtctgttattgtttacatctggtggttctgcacttaagtgtaaaggattgatgcaaagtgactggtga
caaatgtgtgaaatgtgtatatcatgtggaatatgtagtgtgtttgtgtgaagcatgtacatgagtctgt
gtgccaataaaccttcttacttccatcaggtaatgtaaccttaaccctcccataaactggcccatgatt
tatccccctacgtctaatatttgtcgctaaattaatgtcataaaaatcacaccccaacccattgacccta
cccctatccactttttctaataaaactgctaatttctcccgcctccactcagcatccatgatgatgatga
taaaaatctctctaatgacccagactctccatccgtgttacaaaaaccccgggtacttgatgtaaacatg
tgtacttacctcccgagtttcctcgctgca
>KolobokP-1_CHo-I
taataacttcacatacctttattgattttgagaaacttggtaggttggcacctgtgggtatttccaatgt
stccgagaagtttcgttgaaatcctcctccacacaccagagttatgcactcctgaaaaaaggtacaggaa
tagctgaaattgcatgccaacactagcatctcacacaaaattcacttcactaaaatastcawwatgaccc
cattttacaatcaatttccttgaaattttaatatgtcatatttcaactctttgcccacagatttctgaca

tttttctcatcaagattaatattatgtaattttaatatwttttkgaaataataccccaaataaggcta
taaakccccagaaaagccccaaaataaatatttagacacagatatttcagtgggaaaaaacatgtaaaaa
ttctacatttaatatactatagaaatcagacatgctagtaaataaacagcgagtaatccctttgttgtaa
gcagataatcaaacatggccactgcaaccaacttgaggaagtggcacttwcaggtcaaaatggattagct
ctgggtacagtagagatgacatkgataggtataaaatgccacttgaagtactgtcaaacatacaagacta
tawatatgatggaaataaagcaatccatcacaccatctttgatacagcaagcagaaagtacttacagaag
ttatcaaaatgtttcagaagaaccaaaggccatggaacaaagggaggcaactccgccaaaggaagctkcc
cagcaggaggtttttttcagcaaggcaatactaacagatccaaggcttcatcagaggtccaagggacaact
cgtaaacgcttctttgtccggctatccgctggggaccatgccctggtaacacgaccaagtttagactgcc
aatcctacataactcctgactgtgatggggagagtggatcctcgtgtattcttcgcccacagtcagaaag
ctgcagatacctacaaaatccaaacagcaatgagtatgaagggggacaacgtatagtgagtgaggagaaa
atgattgatatgatgacgagcacagcccatcttcatcagactacttccagcacctgccccatgccaaatt
ttgctgtactcgaggcaagaaagttaggcatctcttggaagtacaggatgaaatgcgtaaattgtccgtt
tacctctccgacattcaacctgtatcatgagattcatcaagagggaccaggccgtaatccatcagcaacc
aacatcgctctcgcatatgccattcaagacactgccatcggggccgagaagctgattgaactcctcacct
gtttggacatacctgctcccagcaaatcatttatgcagacactcctgtgcaaaacaagcagaaaaatgac
agaactaaacaaattggacatgaatgacaagatacaaaaggtcaaagaagttaattaacattactcgagg
acaccctgaaaatatcatcaacatttcaacagatgcaaggtacaacagcagcgtcatgtttagtcggaag
acccccggccagaatgccagtcaagcgttttctctggcagtagaaacaaacacagatagaaagtacatac
ttgcctgtgctgtacagaacaaactctgctggactggtgcatggctgcgtggcaaaggaatggaagtgga
ctgtcctggcggtcatgcggaatgtactgcaaacctgtctccggctgctccgttctcggaatacgagatg
gggaaggatatcggcactcagcttggcctgmaagatgttctagtccggtatgttaccacggacggcgatg
cccaaggtgccaaaggagtggacgatgccatgcgagctcttcatcccttgtggaaggtagagcgcctagc
agatcctgtccatcttgggcaatcacagttccgtgccagcaaccgagcagtatacagtgatgaaatgttc
cattctaagacaaaagaagaaaaaaaagagttacaaagggtgtttagcaatgacttaaaatccagatgtt
ccatgatagtgaaaaagctattcaccaagtatgacagagacctggagaaaatgagcagtttcttaccgaa
agtcctggaggcaacagttgcatgttatgacggagactgttcgatgtgtgcggcctattccttggtctgt
gacgggggagtggtctccaactggtggaactttagccgcaacctggccatctataggatcactgccctgc
aaatgaatgacaatgacctaagaattgtgaccgaagttctaaaaatgaagttgagtgtagaggctatctc
tgccatgaaacttctgactgacactaatgccaacgagtcctaccatagatctgcaagtgctaacatgccc
aagaatgtgaaattctctagaacaatggagggccgtctgcactcaatgattcacaggaggaataacttac
caggaacctcaaccagactcaagtgtgaatctgccggtgtccaatactcggactctggcaagcgacgact
agccaggattgaccagcagttcctctacagacaagaatacaataaaaaggaaaagactaagaccagaaga
aacaaacagcagggaaggaaaatggaggaacaccatctatatcgtcaggaacatcggatgaagtcagact
accagaagggacaattagatatgaacctacccgcagcacgagatcatccctaccacaggagaggagcatg
ccaatatcgtctgaggcctcgcccgggatgtgactaaatgactttatacctaaatgacactgactattac
twaccttaaagcttgaactgaatccatatactaaaaccwttattgtgaggagagatgcatgactctccaa
tgttcagcggtgtaacattctaataaatatgataaacttacctattctttgtattctcaattcatgtcaa
ccattagatcaagtctgcaaggcaggtagggaaagtggcccaggcaggtgcccctagctcggcaggtctg
tcgctggttgtttataccatgtggttccwgagtgatgtgctccggaacaacacacagtgtgttgtggcac
aagtgtgacgcaacaatggatgcatccaactcatcktgatgtagcatgacastgagtctatgtacatgca
cggtaacccactwsttctttactggatgcttgtagcacwcaacaccatatttgttgttgctgcttctagc
gccagtscagatgcgacactgaccattcaaaccaggagcagagcccgagcgcactttttcactgtatttt
gcaagaaattcgtccatagccacaaaatgaaagttgcagacgacggaaatagtgccagcgtgtggttaaa
tataagtasgagctacctgatttgcatatccgggtcatactaatcacccaacaataaaaccaccccaccc
ccawttttccctctattttggtgaaattaaattttcataatttaaaaaawggccgaattttctcataaat
taccttaaacaattgatatttattmaaaamtgggggatagaacccttaataagcaatttcacttttgacc
tgata
>KolobokP-2_CHo-I
tatactcatcattttactcacctctgaatttgtcaaatttgggtttttcgtaaaccttaaacatacctct
cattgtgtgtgaaatatgagtcctggaatttgagcgataagtatgctgtgctcatccaagtatgaggccc
gctccctcagcaaatgcagtgatgagagtagactgacagtgagatccgggaggaattgactgtttaatgt
tgactgtaacaaataaaatacatgtaaaacttgaatgaacgtgtttgtttttatgtacttaagggtgtta
tgaacaaattggatgagatttttcaataagggtggattctacccagattttaagtaatggtactgaaaga
actgaaatgtcaatttaagatgcaaaattaagaattatagattgagtaaattagcaaaaacttgtactct
tatgaattgttaactattaggaaagctgataatcaaaccatcgcttatctctcacccccctgcagttaata
tatgggataaaacttttaccacccccctacatggcagagcttaaaataatagcaggtgccccccctctttt
gctacataaatcattcaaagataaacaatagcccaatttttggatacaccacaacagcttagtctcccta
gtgtgaaacatcatcagcaattagttagaaaattggaactggcattcaaaaaaaggtcctatttaaatca
tgccaaggtttaagaaaggacattcttgtggattcaaaccaaaatatgtacctcataacaaggggaaaaa
gggttgccagctgactggcttacctggtaaatataaaaggttgtctcctgaaatgaaagaactggtcaac
aacccaccgtctgcatctgagaggaaggaaatggtcctgcggccggggagctaccatctcctaagaccaa
ggcagggtgtccttaaaagcaatatggaaactcagcaagatcagacctcggagcagtaagtgatttttca
tagactagaatacataagaaacacattttaacattagctttggccaatggaacatcttattatacattta
atcactttaatatcaatttaattagactttctaacactacatcaattttgtcaggttttacatgtaatta
catgcaattttatgtaaccaggagtgatgaaacatgcattcataaatcatgcacctcaactttatcaaat
aattggaattgaagcagtatatgtaaaaatacattgatattaaaaaatcaacaaaatgtacatcttgttt
aagggaggtaactctaaaatcagttttcaccatcctcacaacacagacattcacatgcttagtgaacagt
agttacaaatcttgtagagcagaggaagattcccacagactgctccacttgggcaagaccgctgccatgt
ggaaccaagttttccgaagccaccaggagcagagtccggactgcaatggaagcctgacctgggacttggc
caacgaggaaaggcggggacttgccacaagaatgggcctaaagtgcgatgcgtgtcccttcatgtccaag
agacacaacttgttttaggaagtagagaccagtgcacctggtagaaaatctgcaactcttaactactcac
ttcaggtcgggctgagccaaacaccccttggtaatgatggaatgaggaaaatactgctttccactaacac
tcctgctccatcccgcaagtccctgcaaaaaaccagtaacaaagttttggccaaaattgagactctaaat
acaactgacatgagtcgcaggtgtcgccagctggttgacgtcaacaccgttaggggacatgagtcgccgc
agtctatagacgtgcagtgtgacggcatgtacaacaaccccttgtactcgggggtcggtgagacgccatt
ccagccggcgacacaaactgtgtactctgttgctgaaaatgtaacaaaaaaacaccaaatcattaaacta

gtaacgaaaaataaaatctgctcgaagcacagtcacttacgggatgaggaatctgctaaccactgttgta
gtccaggggaatgcggggcaaacctcccaatgcagcatactatagggggatgagtttacctgggctaggga
ggggatggccgaacttctctgtgaagacggcattgaagttcaggcagttaccactgatccagatagctca
gctggtcgggctgccgagtccctcttcaaggatgggctttacaaggtcgagccgacacattacctggata
cacgacatttgtcggaaagtatcaggaaatctataaaaagagatgaaaaaactgttaaaggccatgccct
gcagaactaaagctgagaaaaccaaacttttgcataattttgctctagatgcagttgatagatgcacagc
cgagatcaatcaagcctacgacttgtgtgcaggcgatagccaaaagatgaaaaacaagctctcctatgct
gtagatgccattgtgcagtgttacatgggtaatcatgcactgtgcaggagacaatctctagtatgtaatg
gggggacagttaattgggttagcaaaagtacatatttaggtccaaactttgaaatacctcactccttaga
gaacgagaacttgctccgtgcttgcatctcaaagaggttgtctccagctgttcttgacaaaaccatcaag
aacagcaactcgcagaaggttgaaagcttcaatcgcactctgcgacgatctttacctcgtaacatcacct
ttactcgcaacttctcaggtagggctcatagtgctgctcactcttcaaacaatggtccaggcgactccat
ccgcagcttatgtgcaggagtgggttgtgccatccccagtggaggctcagtggacaagtctctacaagac
attaaaaaaaaccatgaaactagtaaaaaatatcaaaaatcaactttgtacaaaacaaaaagggtggtta
aacgtagaaaactgtacaaactttatgaaaaacatcaagaggaagttaagtacaagaagaacattatgct
ctctgaggcccgagcccgaagtaaaatgaaaaaaaattctgagcatagttattcaaaataggtagaaatt
gtattttagcaacatctcaactatataattacacataggaatgcaatagggatggtgggccctgcaacat
aacccctggactctacaatggtttctctcctgattgatctcatgcgtctccaggacaaggtgggctgggt
tgacgcagagcttcttatggcacagatggctgacctccaggtgcccacccggaaactgtgaccgggtcaa
gcgcatctgtgccataagagcaacccgatgaactctctccatcttcggtccctcctccggccatctaacc
ctcattactccatacccgtggacagtggtggcacctttccatataaacaccccccctggtccccccctgaa
tttcaaatttttcaaagagtttcatctgtaatttctcgaatatatctgccatgttcacgattttactgaa
aattcactttttctagaactacttaaatattaagtcacatgacaggtcgtaaataggctttctata
>KolobokP-1_SaGl-I
tttaagacaattaaaagttaattaaattaaatgaaatttgcacaaaatgtctccgagttatttatctttc
atatactagtgcgtcaaaatttatctttgtccttaagggatgaaaaataggagataactcttcctaacag
attattgaattttaggctaaatttctgtacaaattcatctttagcatattaatttcaaaacttgaaaaaa
tttaatgagtgaattattgagaaaaaaaaaatttaatgaattgaaagctgaaatattgtagtttttggcca
tatgctaaaaaataatttcaactctaattaatgagctctaattagctaagaaacaggaagttaactgatt
tttaaggaattaattagcaaaaaagtgaaaatcttaattctaaaacaaagtcagagactggaaaaaatgg
tttcaaaagagacttcaataatgtaccttggagctttgataacagtgatcactgattaatggctggtgat
taagataaggggttcttaagcagtggggggtgatgaggcttttccatcagtcatcaaagaatggagtttc
tcttagaagaccaggcagctctaaaataaataaacaacacaaaagtttacaatacttaaaaatgtgcacc
acatacaatagcatattcttagaaaaagtctacaagtatacagttaaataattaccatgccaaagaaagg
accaaaatggttgcatcaacatcaatacaaaaaaggaagtattcctcacaacaaaggccagaagaaaata
gggaccaccagcccagtccaatctctagttcccataagaagactaagtaaagaagagtttcaggacattg
tacggccacagaaaaatggggtgtttacagttatcgatgccaagagccgaagcagcgagatgaagttttt
acgacccgggccaagtgaggtccagccagtggacacctacatggaatctgtgtcagatgacccaaattca
aaattatacaaggtacttcaccaagaaaagactgctaaattgtggaacaaggcattcaaagaacacttac
aaaagtttcctgactgtgatggtccactggactggaatcagcatggagaggaaaaacgaggcctggcatg
gattctttccattcgatgtaagaagtgccactataccagtcaccgagaaaagctttacgaagaggtgaga
agatctggaaggggacgaagagctgcacagctgaatgtggccatggctgtcggtcacctgggtaccagtc
ttacaacagagggtatgcgtggtatgcttcttggtgcaaatattgaacctgcgtcagccgccaacatgca
acagacctgcaacaaagtggggaaaattataaccgatgtaaataaacaaagtatgaagaaaattcggcag
aatattcaagaacttaatgaaaaatgtggacttccggataccccccatcagagcggaaggagatgctcggt
acaataatgccaccttcagtgccattgggaaaaccccattccaagctgccacccaagtgacgtataccgt
aagtgaaaatgtaactcagaggaagaatgtggtagcagttttctgtgggaataaactatgtaagaaaggc
gcacacctacgcagcaaaggaaaagaggttacctgcccaggacatgaagactgcactgcaaccattcctc
cggagaccaccattggagacgagaagagatgggccgctaattgtgttggtgagttacagagtgatgaccg
gcctctagtaatatcccattttaccagtgatggagacagtgccgctgcatctggtgcatctgaaaaacaa
ggacatgtgattgaaaaccttaaggacttgcgtcacttctttgactctcaaagaaaacagactgctaagg
ctccattcagcagtcacatgtttccgggaagaaccaaggccataagagaatccatgcagcgaagatttgc
cttggacctgaagcttcgatgcaggacagagtacgagaattgctacaaacactactccggcgacttacct
ttgatgaagcgtgctatgtctactgcggttggttccctcataaactgctaccagggacagtgcgggaagt
cctgtcaactctattccttttcctgtcgtggactgaaaagcaataagtggaaatcttcagtgttgcctag
tgactttgaattaaacatgacagaaaaagacaaatgtttgttaaaagactgtataaatttgactttagga
caaagtaatcttgatagaatcaaatatcatacttgtacacaaaaatctgaatctgtaaatagggcatatt
taaaatcaaatccaaaatgtattacaagtagtagaaattttgaaagtagaatacataaagttgtacactc
actgaataggggtcatggaaactcaacactagaactttgtgaatctgtaggggcacctgttgtaaaagga
agtagagtggtccatcatctaaaacaacaacaaaatagacaaaattactttaaaatgaaacaaaaactgt
taaaatataaatttcaaagaaaatactacagatctaaaagatatcagctgtacgataaaaaatctaaaga
cacttacagtaaatcattaacagatccaaaattaaaaaggaagttgtaagactgtgaaggggggggggga
ttgatgatatgtacatgcatttcaaatgtataaaatgtgtatttaaaatatattttgtgaactatcttac
atgtaataattgtttttatggttcattatgcattgattattttcaactgtgtaaatggggaaaatgtgga
catttgaataatgtattattgtatgtgtaaaatgtgacattttgttatacgagagagtctgtacatgtaa
aaatgttatattctttattatttagctcattaaaattatttgaattaaaaatcttgtttttcttacctgt
gtgatgaacacaacaaaggtttcagaggtatttggtagaaatatgggtatacgggtgtcaaaactgtccg
ttggtgacaagactgcatgccgcattgtttatccgccattattgatcttttcaaggggagacaactcctg
aaatcagagaaattttagtgaaaatgagagttacctgtttctcttta
>KolobokP-1_PiIm-I
taaaaaagaattttatcaaacagtggttttggcaaatgagataccaaattaatcatgaatgtttcttctt
ttaaaatatgtgctttgaatttgaaaagattacaattcttatttaagaaaggtgttcaaaaccccctacc
ctcatggtttaatgaaatgaaatgttcataaagcatatatgtctttgaaatgtttatgaatattaaacat
gaaactttgaatcatattgagtcttcttttatttatttgtaatggtgatatatagtactgtaagaaaaa
ggtctttatacatgtcaacatttagaaggggagatcactccatgcatgaattgtacatctatgaaattga
agcaaaaggtgtctttatctctatcaaattcccactttaaagtgatataggtaataaattattacagtat
actgtacaggcgcgctttaccagcaagttaatctacgattaattgcctagtgtcgagcgttccaattata

tggataattgatacttagttagtgtaaatatcccatgaggaaaaaataggagggcaccgttatttatggc
ttgatgctgaacaaatgtcaaagattagaagaaagaaaacatacataaaatgcagcagcttaaccatcta
aattttagtcatagtataaaactgaacacatattgttttacaagagaaatatctataaaatcatgcctag
acattttaagaaaggaaatacttcagggttcaagaaaggttgtaaacctcataatgcaagtgacaatcaa
acaggtgccataagtgatatgaataacgaaagaatatatatcaggccaagcgaagaaacctataacttag
catttgacataccagagtatggactgaagcctcaagaacctgaccagtccagatccgttactgtgctgcg
gccaagacgatataaggaacaagaacctgtaacagaaattgatgatgaattatttgaaaggtatagccga
gttttagttctataaaaagtacagatttatatattaaatcatgcccgtttatagaaaacatttcaatggg
taaaaatcaagaatacattacgaaatctaccaaaaaaagggggggtgtgaatattattttttttccttcat
gaatatcatcgacatcccatcaattatttttttttctgtataaaagaacatctaatattgcgaacaaaaat
tattttaagttctttcgttcacataaaataccataatgatatgttttatattaaatttctaatttttttca
tatgtaaggagggataactctaactcaaaagtatgcatgtactttgaaaattgaaaaagtaaacaaaacc
catttttatctaaattaggctgaattcccaaggttatagagttgtccacatacaaaagaaccaggaaatg
tggtacaatgtatacaaagagcacagtcaaacctcaccaaactgtgatggcatgctggtctggcatctgg
cttctgaggaaaggcgtggtttagggtgtagagaggtagccaagtgtacaaaatgcaagtacatgtccag
catgcacacactctacactgaggttcaaacacccagtcaaggcaggaagtgtgctgatcttaacttggga
ttacaagttggtctttgtctttcaccaattgggcccacttccctaaggaggatatgtctgtccacaaata
tgcctgctccatcaagtaaaggactacagcttgctgcaaataaagttggcaaagctattgttgaagaaaa
taagaaagatatgcgaagaagaagccaaagattaaaacaaataaacatattaagggggcatccaaacacct
gagaacattcatgtgcaaagtgatgcagtgtacaataatccattgtattctggggttggtaaaacacctt
ttcaaccagccacacaagttgtgtacactgttgctgaaaatgaaacaaaaaagcatgaaatcattagtct
tgtaactaaaaacaaattatgttctaagaataaacatctggacaaagaccatgatacatgcagcccctct
tgctcagctaatattccttttgaacatagtataggagatgaatattcatgggcaaaagaagcttataagg
atcttgagaaagagggtttgcatgtatcatatctttgtacagatccagatagttctgcctttaaagctgc
tgaagatttggccattgaaagcaatcatctatttcaagtacaacatttcattgacacaagacacatgtct
gacaatcatagaaagaacataaaaaagaatgaaaatcttatgaaaattgtaaatggcagaacaaaagctg
aaaaaaagaaagtggtaaataatttagctattgacattgcagacagatgtcaagctgagatcaaccaagc
atacaaatcacacacaggggatgtacaaatcatgcaaagaaaactgacctatgcaactgatgcaatcata
cagtgttatcaaggttgccatgctttatgtagaaaaaagtcatttgtctgtaaaggagggaaaacaaata
actggttattaaaatccaactttctaggagagtcatttacattactgagagggaaaaatacaaatgagat
attgtcagattgcttaaagttcagattttctcttgctgcattgaagaaaaccattattattgccaacaca
caaaaagtagaaggttttaatcgctctattaggagatcacttcccaaaactagtacattctgtaaaaatt
ttacaggcagggcacatgctgctgcctatgcagtaaacaatggagaggggggatgccatatctaatttgtg
taatgcagtgggctgtattataccaaagggagggattgtttcaaaagctttacaaaaaaatcaggaactt
gaccagatgcgaaaagctaacatgaagaaaattgaatttaaacgcagaaaatgtcaaaaaaagagaaaat
tattcaaactgtatgaagaggcaagtgaaaaagctgaatatgagaagggtaaactgttgaaatcattgca
aatcaaagattattcagaccacacatattctaagaaaaagaaggtaaatcataatagataagaaactttta
cctctttcttatgattacaagagacataaaggaggatcatgactctcaggataacaactgttttgcatat
ggcatgaaattcttccctggttggatgtatgagtctcaagtattatatgtgatggatttatacatgtttt
tagatgacataaatgactgcactctaagacattattattctcatcaattcttggaatttgcataaatgtg
attttatgttgtagcatatatgccatcctgtgagctttctccactttacggtcttttaaacctggccact
tcactttcaaaatgccatacccaccccccttttgtgtgatttcacccttgaatgcaatgcacccatgtac
attccctacaggctcagacagcctgtctaatcttttactaatatcttcccaaaaagcctccattttataa
tcacatgacatggtttcattatttttaagaattaagaagtaagaaaagaaaaaaggaaatttcatgattt
tccttta
>KolobokP-1_PeMa-I
tgtatatatgtatttttttagagatacaattttctggttatatcaccacatttggcatacaataacacatg
ttattgcaaattattgtgaaatttacttcataaatgaaaaagttatatcattttcagttaccctacccccc
atcctctgtaaaactctactctgccaatgagacttctgagagacccaatatgtaaatggctaattgggcc
atcaatattcaagtaatatatcaacatcatatatcaaattaaagcttattcaatacttttctgaaattta
tatgttgtttttcaattttcacaaaaatgaactttataaggaatttttaataatccatgtaaaaaaatac
acttttcaacacaattttttttcagtaaaaatgaaacagttttttttttcaaaaatcacagcctgtctaact
ttataccactgtatacaggaagtttccccctataaaatgagtgtaattgaaactttatagcccaattaat
taattagtaaacacatgacaaacacacccaggtaggcaggtgccctctcttcattggtcagcaccagggg
gatttgggtatgagatatcccccttcctgtgggcttgtaatccttattaagtggtgtatatatttagacca
tatgtgttttatgtaagttcaggtaaaataaagggttttggatatataggggtcaattttagagggatat
ttggcctgacagcaccatgaaagggaggaagaaaagactaaaatctgctttccagtatggtaacacacca
cataataagggtaaaacaactgcaggacatggggtaagtaaggttttaactaaaaacgaatcaggcacct
ggaaacgattgagtcccaatgtcttttctttagttacagtaacgcgccctggtagcaactttcccagcac
ttctgacaccgaaggtaaccctggtaatgtgaaactccttaggcccagaaaaaccaaagcgactgatcta
gaggctttagacctacatgaaaacatgaataaaaagcctagattacaaactcgtatcagaaatcagggta
atcgattaataaatacagagaaaattggagacctcatgcaaattatctcctcacacaggtgtgacaaccc
agatgttgatatttacaatgagatcaaaataggagtaacatgtaaatttcaagtctcatgtaaaacatgt
ggttgggtgtctgatacacataagatgtacaatgaagttcaatccccaaaaaggggaccaaaccctgcag
taccaaatctgacaatgcacgctggcctccaagaaacacctatggggccagcaggtgcacggcgtttgtt
taattcaatgaacttggttccccccctgccgtagtggtatgcagagaaatgctaatatggtgggggagaaa
ataatatcactaaataaagaggatatggctaataaagcaaaagaggtaagggagatatgcgagttgaggg
gagaccccaacatggttaacgtacaagttgacggaagatacaataggtcaacaataacctgccgtaacaa
gcctggtacaagtgctgtacaattagcactaatagcgcgtgaggacattacagacaaaaattatatcttg
ggaatagccgtccaatcacagatatgctggaagggtaaatggttacggctcaaagggtttgatgctaaat
gtcctggtggccacattgactgtagtgctacaataggggaggggacaccgctatccgagttcgaagctgc
taagcttgtgggtgagcaattgtaccaacaggatcttattgtacggtacgcaacatctgatggagacgcg
agatctgtcgcaggtctagatgcggcatataaaaactttatacccatgtggaaagtccaacgtctagctg
atcccacccatttaggacaagctcaatttagaaaatgtatacgagcacatttcagctcagacatgttccc
cggaatgagcactagagagggaaaaactgaatcacaaaaagttctgagcagggacataaaagctcgatgt
agtttgatttgtaaagagctcacaaaagattgtaccggggaaaccaaaattatgcgtgaaaaactacccc
atgtgctagatgctaccctcaggtgctacagtggggattgttcccggtgcaaaagatactcctatgtgtg

caatggtggagttggtagtggtacttggtggaacaggtccatattcctgtctaaacataaactatttagc
ttgaacccagatgaaaatgacaaacagctattgctcgagatactgaaaatgaaattaacagatgaatgta
tcacaggaatgtctcttggagcaaatacccaaagatgcgaggctttttttcaggctgcttaacagccgctt
accaaaaaatgtgctatatagcaagaatatgaatgcacgattcaatggagctgtccacacagcaaataat
ggggtgggaaattccctcatgaaaaaaatggagtgcctcggtgcagcagtagcacctgaagtgaggcgct
ctctgcgacaaatgcagtcagagcaaaattaccaccaagagtaccaaaaaaccccagcttttaagaaaag
gactctagaactgaaagcagaagctattcagaggcacattaaaaataagggagctagatctgattataaa
aagggacagcttgacacatatcctgacctcagcaccagtagactgagctccaacaatgatgaacacaact
atcacaaatagttatatataaatatatatgtgtgtacataaaatgaaatcatcaaaaacattaaaaaaa
caaaataaaacaaaaacaaaacaaaaaccaatagaaataaccatgattgagatgtatgagatgagccgag
cttgagccgtgctctgtgcaaaatgtacataaaatgttgatgcatatatttcagataatgtacctgtata
tattctaataaacaatctggatattccccatgtcctgtgcaattattacagtttttacaatgaattctgt
tgttgttaataacatgtggctccaaactgatatggtcaacagttgtgcataaattattatgacacaggtg
tgatgcatcaaagtgtgcaggtaattctaaaacttccttatccagcataacacagaaacggtgtgcggta
accgttttccagacacctgtgtccgggtgccggtatcttacaatgccatatttttttgttgttagacagtg
gtcccatccataccctgcacctgctgttcgcagaaatcacagatttctgggttaccatttccctatattt
cacaagaaaattttccattttcctgtatctaattaaattttagatgttcatgaaagtgaaagtacgtata
ctgaagtaaacatgtgctttgctgacaacccggctcaatatataagactttatcaaattttaacatgaaa
ttttgctaaaatcatagaaaaagtgatgtaataagacaaataaaatataaattcattcataatttacact
gacacctagtacaaaccttcaacttcaatatcccagcctgtcaaatgtctacttcaaacgtgccgactac
agcggaagttttagacggggtataactctagcctatgca
>KolobokP-2_PeMa-I
ttaaagcaaataaatattgacttgtattgactctttcacctgtatattgaaagttcagataattcccaat
aaagttttgcagctacagtgttgactggagtccattgtctcatgcaatatggagttatccccccctggtgt
cagataggagtaccaaactccatatgactgattttaccttagaaaacaaaaatatattcattcacagaca
gcacagtttttttacagatttatttcatatttagcacacatgtataatatatataagtctatcataccaat
aaatttcatgatcatatgactttcaattaatgagttataaggattatactaaacatgttaattattttttt
ttgtttttgttttaaaatcaagaaactctgttacatggagcagtgatagcaatcgacaactgagactgga
cacttgacaggctgacaatacacagcatcaatatctcctggggtctctatacactacaggccctagtagg
gggtaggggtaagattatccccctgacagccacacaggggctatataaacaatacagctaggttacttca
tcacagaattagtttctacacctaagtatactatactgacaacctggatttatatacatattgaaaaaat
gggcagaaaacgacgacttttatcgaaaaatcatggttttcagaaaggaaatgtaccggccaacaaaggt
gaagtgtcaccaaggaaaccagtacctaagtctgtgcctgtgaacagactgacccaagatgtgtttgatt
cgagggtaaagattgatgaaaagggtgaaatgagcacatcaaatgctgctgggattccaagcgatatgaa
aatcctgagacccaagcctgcccagactgtcatcaatacctgcatggagactactgtacctgatcctgac
aactacacatacaagttactacggccaattgatgtggggagactgtggaacaatgctattctggaacatc
aagattgcacaggatatctgaagtttgattcccagaactcaaagcagtggggatggggttggtcagagag
actgtgttgtaatacctgccaatatgtcagcggtcactacaagttgtatgaggaggaggagacaaagtcg
aggggatgaaaaagatcaactctgaatactgccatgcagactgcactgatgggagcctctgtgtcaaatg
cagctatgatcgagatatgccttaccgccaacattatcccaccttcccacactggtctccaaaacacagc
aaaccatgttggagaaaagatagtggaaatgaacacagagagtatgaacaatatcagaagaaatctggtc
gaggagaaccggtctctcggactacaggacccagagtgtgtgaatactgaagccgacgggaggtacaaca
atccactgtttagtggagagaggacaccttaccaaggagccactcaggtaacttacccaatatgtgagca
gctctctaccaaaaagaaaatcctgtctgttttcacgggtaataagctgtgcaggaaagcagaaatacta
agacgacaaggccgacacatcaaatgtccagatcacgagggcctgtgcacagccaatgttcctgaggaag
ctgctattggcaacgaggagacctgggccactgtagttggagaagacatctcggattctctaaaggtact
tcaccatacatctgacggagacagtaaatccttctctggactcaggaagtctcatccgggctgtgctagc
ctgaaagatacaagacatttagcaaagggaataaaaaagaaaatgctgagtcagaaattcagtccaaaca
tgttccgtcgacacaagggatatacctacggaacactgaagtcaaggctggcaaatgatgtgaaggctag
atgtagggcagaactgatacaggcccacaaattacacaatggagacatggacaaaatacagaagcatatg
ccggatacaatagaagctatgctgatgtgctacaaagggtactgtggtgacatgtgtcagcgctacagct
actgctgtaaaggtgagagacaggaccactgggaaagaacatttctaccagaggggacagtactatctat
gacagaagaggatgagaaatactttaaagtgtgtgtggacacattcctgagcacagagtgtttggaacaa
acaaaactgttgaccgacactcaaaagactgaagctttcaatcggaaactccaaaagacaaacccaaaaa
gtgttacctggtacaggaactttccatcatgggtgcacactgctgtgcacatgtccaaccatggctttgc
tgattccaccatccttcgactgagatcagttggatcacatattacatcaggatcatcagttgtcaggaga
ctggtttctgtaaacagaaggcagatatgtcaccggctcagacagaaaaaactagcatacaaaaagcgtc
gaggccaactaaggattaggagatacctgtgtcattcgaggaaccgggcccctgtgccaacttaccgttc
aggaatggacaatccaccaagtgtcagtgaccatacttacagcaaataactgtgctaatgtgaatctgtg
aaccctgaactgtgctaaaatgaactgtgcttttatacttaatggaaatatttctgttatattattattg
tgtatatataatattaaattctaattaaaaatttaaacttatatttaatctttgttttatcttacctact
gaaaaatacaatttctataaggcccatggcctttacatgttggtggtagcatgtgtttgcagatctgtct
gctgttattcacatggcctggctcaagactgaggtgacctgacttgacacatgaccgactgtgacacaaa
tgagaaatctgtaactctgggttcaattcaatttggtgaattgaaatcataaaagacaatctatgtgtat
aaattgtcttagatttccggccatggccaggaaatctaactctggttacaccgtaaccatgtgcgtttct
agcacccatccagaatatacaaccatttcctgtaataaggaacttttttgaatttttttctgagtaatac
tcaaaaaaattattaaatgaagtctcttggactgccatttggaataaattcagaaaactggacggaaaac
aaacttcaaaagacagagttcatacgtcagactgcacgtgctataatcctgttaaa
>KolobokP-1_MiYe-I
tattataggactcaatttttttttgatttttttaagtaaatttttcctgaaactgtaagcaatttggaact
tgttggtgaaaaccgcgtaattttaagacatataaaacagaagttatgatgaatttaaaaaactctatca
tactgtgattttgtgctgagtttgacagacattttctacatatatttctaactacaagggtcagggaaaa
tatcatatcttctgaaaaaaatatgatatttcaattctgtaaagtgatttatgaagcagaataatatctc
cacttactcaaataagttgtttacattataatcttcaacttaatttttttttaagaatttaaatttaaat
ttttgctttaaaatttaaactctttcattaaatgaatatttaaacagtgccaaaaccatcattcagtctc
cataactacacaaaatactctagagacaattacaattatcataaagataagttgcacgcatggacgcacc
cacagccacctgcatacacacatatagacagatagccagtgccaaaggcagggggggtgggtggggggtg

tgtgtgaaagggacctatctctttgatgttaaggctctcctgttttctaaatcatcatctttaaagttga
taacaaacaaactacataagaatgagtagacaagtggtcagcaccactctgattttggattctcaacaca
actgaccactgaccaccactactactattactactatgaagggagatactaagaggaggaaaaatcattt
tcaacctggaaatattcccaagaataagggccagacacttatgcctgtagaagatcaagctggacaccca
aatgaccggactgtatttaccagaaggctaactacagaggagttcaagctggtgaccaaacctagtcatg
atggtcgatcacttatagcctcggacactgaagacagaccatgtccagctcggatacttcgcccatcaac
agttcctcaggaagactcagactctgaaccggacaatatcgagggaaacagaattattgatgtactctta
atggtaaaaatgtggaattctgtattcagactccatgcagaaaaacaccatgagacatgcaaaattccag
agtttgaacttgacaccgagagtaaacgggccctgggatggagttgttccgtgcggtgtgctaagtgcag
ctaccggtctccactgcacaagctctacagagaagcggacacaaataagccgggaccaaagccagcactt
gtgaacaggtacctaccatcagcactatgtggccagtctgtcagcaccaaaggagccagactgcttctcg
gacacctcaacatccctgccggagcaaaaagtgggatgcagcgacaagctaacctggtgtccaaggagat
aacagcactcaacaaaatagacatggccgagaagaccagacaggtagtggaagttaaccacctgagaggg
gatgctaatccgagcactatcggaatagcactggacgggcgatacaacagcacatcatttgggagtgcta
agaaacctggcatgaatgcctcacagggaatgagtctgggcattgagacatcaactcctaagaagtggat
agtagcacgcacacttcagaacaagctgtgctggactggtgcttggctgaggggaaaaggatatgacgtc
aactgtcctggcggccaccctgactgcacagccaacactgaccgatacgacggcctgtcagagcatgcca
tgggaaaaaacatcggagatcagctgaccagtcaaggtattttggtcaagtacgccacgactgacgggga
cggtactggagccaaaggcattgaggaggccatgcaagcgctagatcccatgtggaaggtggagagactg
gccgaccctacacatttaggcaaggcacagttccgaaagtctaacagtgccaagttcagtgagtccatgt
ttcctggccgaaccagactgatgagagcgcattcccagaaaatattttctcaggacctgaaagcccggag
cagtctgatcttcaaggacctgatgaaacttcacaatggaaacatggacattataacaaaacgtttaccc
gctgtgcttgatgcgactgttgcgtgctactccggtgactgctccaaatgcaaacagcattctgtcgtgt
gctctgggggtgacagtaataactggtggacacgctcaatgttcctaagtgccaacaaaattcacggcct
gcagatgacatcagatgatgagatgttagtgctggaactcctgaagatgaaactcagcaccgagacggtc
caatcaatgcgactcagtacatctacccaaaagaatgagggatgtcatcggacgctgaatgtttccttgc
caaagtaccagaatttcggaagaaatgctgagggacggttggacaacacaattctaaccataaataatac
cgcaggcaaggccactcagcagaaagtagatcatcttggtggaactctgagtaaggaggttcgacatcag
ctggaccaaataagctccgaggaaatgtaccagcgccagcgtcagtacaaacccgatgtcaagaagagaa
atcttgagttgttggtggaacagcacagggagcatgcaaacaggtcaaagtctgccaggaagtccgacta
caggaaaggtcaacttgatccaacactgcatcgaaccaggggagacaatcaacctcaggaccacctgtat
tgagagaaaaatgacatttaatttgaggactttttttgaaatcaaagacaaaattccaaattcaagaaata
tgtaacaaatgaaataaaagttacatatatatgactacctaaagagggaacctccacgtggtggtagtcg
gacaatgttgtatctgtgtatacaatagctaataccctcaaaaatagttgtttgttgtttttttcaatcaa
gccactttcaattccaacatacaatttctaaaacctacatgaccatagcataaatttgaatttaagcaat
attgtctgttattattaatgaaatgatgctctaaactaatgtgatttacatttacacacaaactattgtg
gcacaaatgcgagcaatccatatcacttctaatatctattttttttttcaaaaaccgtaaatgacagtcta
tgagcatggtttttttttccatttcaaatccctgggatctcggaaactaatcagtccatattttccattct
ttgttaatggtccctgccagattctacatgttccattttctgcactgataactgaattttctaatattct
gtattgatacaattcaaagaattcctccatttttacttttaaaaatcaggtgtaggaatacataaaacca
cataatatgcaaatttaagtgtctatatttaaacactctgccattaaaaaaataattaaaaaattaaaat
taaaatgaatttaaactttaatttttaatcaaatttaactttttttataaaaactatttgattcaaaaaat
caaggatgcagtatagaataatcatgccaaattttatcgaaatcggtcaaaaaatacggatctgagaaat
aaaaacatgcatttttagaccagtcatata
>KolobokP-2_MiYe-I
ttaaaggaaccttcaatttttttgaaatgttaaaataaagttattttctgaaagtatattcaacaccaagt
aaaattgtgaagtttggtgggtataacactattggttatggagatatggcaagttatgtcccctggtgtc
ccaatgctctttacagtcagaagcaagacattttcaatttaggtacaggtaattaacctgtcatagatca
actaatttatccttatagaccttcatattttgcatgtatataaaacacacatttacctcatggactgtgg
tcttcaaattgaaataaatcaactactgtgttttatacagaattttaactaattgatttgtttatttaat
gaaattttgttaaaaaagtataatttgacaatctttcagtgtacaatattgttcaggatttctaacagga
ccttggccataaatggttatttgatatgtacctacactgattagctgctacttagacaagcttagtgaca
ggccctcaaacacccctggggagccaccaccactgggagagctatgggggtagaggaaagataatgccat
aaactgtcaattgtctgtgtaaacaatcaacaataacacactgtcccatcagcacagatgtatatctagt
agtatataatgacaaaacatctcaacacccactattcatttcaaaaatggggagaaagcaggctaaaatt
tccaaaacaaatggatttcagagagggcatgttccacacaacaagggaaagacacagacacctgtttcca
caccattgccaggacaaatcaacaggttaccccaggatgtatttgaatccagggtacaaactgacagcac
gggaagaatggcaatatctaatgctgagggcttgccgtccaacatgaaacttctacgtcccaaaccaaca
aaggcagctatagattcctgcatggattctgaagtacagcacccgaacagctacacatacaagctatacc
ggcccatagatttggagaagttatggaattcagcaatccgtcagcacaaccgtcagcacaagcactgcac
aggctatctaaggtttgactcaaagaactcaagacagtggggctggggatggacggagcagctttactgc
gaaacatgcggatatgtaagtgaaagctgcaagatgtatgaggacgaaaagacggacaacgacacccccta
agagaggccggcgacggagtcaactgaacttgggtatacaaacagcactcatggctgcatcagtatccaa
ttcagcaatgattgaaatatgcctgagtgccaacatcatccccccctcttatactggattacagaaaacag
ccaatcatgttggggagaaaattgtcactatgagcaaggccagtatggaaagtctgagagagaaaattgt
tgcagataatacaacttgtggactggagaagccagacctcatcaatgcagagatggatgggaggtacaac
aacccacttttcagtggagaccggacaccctatcaaggagccacccaggtcaccatgaccctgtgcgaac
aaatgacatcggataagaaaatcctaagtgtgttcacaggcagcaaactgtgtaagcgtgctgagtattt
gagaaggtgtggggtacaggtcacctgtcctaaccacacaggcatttgtacagctaatgtggctgaggat
gctgccatagggaatgaggaagcctgggccacaattgttggagaggagattgcagacactcttacaatca
actacctcaccacagatggagacagtaaaacacacaccggtatgaagaagtctcacccggcatgtaaaaa
tttgaaagatcctcgacacctggtgaaatcagtgaagagagagttttggaagagcacattcagtgctgac
ctgttccagaaattccgaggtctaccatatggaacgctgaagtcacgactggccactgatgtaagaaaca
ggtgtgttaccgagctggaaagggcacacattgttcacaaaggaaaccttgaccagataaagaagaatat
gccaatgaccatagaggccatgatacagtgctacaaaggaaactgtgcagaaatgtgtgctgagttcagt
tattgctgtcaaggattaccagacaaccactggagcaagacatttctgccacaggggatagtgctatgcc
tcagtgctaaagatgaacatcattttaaacaatgtgtgctaaagtacctcagcaccgagtgtctggacag

caccaaactactaacaagcactcagaagtgtgaatcttttaacagagtgcttcagaaaacaaacccgaag
atgatgacttgttaccgcaactttccagccagaattcacactgctattcatctgtgcaactttggcatag
cggactctacagtgctgaggctacgtgcagtcggtagtcctttggtgagtggctcgcgtgtagtcagtcg
catcacaaacctgcgaaagagacaattataccatcagcaacgtcaaaggaatatcatgttcaaactaaaa
agaggatatcatagaatcagacggtatcgcatgcatgcaaaggcgcgtgttcccacagtaacatataaat
ccggagtggacgacccaaagactatatgctaagaagatgtgctaaaaacagactgtgccaaaaaaaaatc
tgatgagaaaatatgtgttaagatgactgtgcttttgaaaatgtgtttatttgtgtgctttttttgaatt
tcaatgtaaataactgtttaattattaaattttaaaacttactttaattgttggttatttctactagtca
aaaaagatacaatttggatgagggtcatgacctaaacaagtctgaggcagcctcagcttgcaggattgtc
tgctattattgatgtaacctggctcaagattcaggtgttcaaacttggtacagaggccattgtggcacaa
atgagacacctgcaatgtacgaggcaaggtaaattgtcttgtagaaatcatatactgtaatctatggaca
tatattgtccgagacctggtgcagccaggaaacttaacacggacaagtccataatcacccctcgagccac
ccctggctccgagccatactacacagcctgtagcgggtgtaatttcagatttttttgctaattttgcctg
ataaagtcctagccaagagtttagagacatgatgacagaactgtcattcgaaaattaggtcataaacaac
agagaatcaaggaaaggtgagggtcacctgagcacgtgctatgcatccttaaa
>KolobokP-1_MyGa-I
atattagatcagcaaatgtcagattccacttgtattgcagtatgaattccttcggtacaccttttcgaac
aattctgtgaagtttttttccgcatctgaaataaaataagttaatttttagtatttttaagtaagcatgc
ttattactgttccttatatcttttagtagaaaaaaagcacaaaatccatatgttatcaattccacaattt
agacattgattaagacttttcatagcttaaagtgccatttcaacaaaccttcattaccttactgacttta
taaaaatcatcatgtttagatgatgaatcatgaaaatattgtgtttttttactgtaaataaggtagattta
aggatttttttcactcagaaaataatgtttttgatatttgtttctttcaaagaaggaatagaacattttta
tttttttataactgttaagatcattcacagaccattctaaaacaggttttagttttaaaatccatcaagt
aataaccattttagagttctttttgtgtgagtgttcaaatagggaaaaatggtgcttttctttggtgaag
ataacattgtgacgtcatcgcttttgtgacgtcatgactttatgatgtcataatttagaattgtacaaac
ataacagagtgtgtatttattcaataatgaaagggaaaaggaaaagaaaccaatataaggttggtaacca
agcccatctattgcgttttgtatccctaacaacagatcaaggattgtggtgttaataatggtgttgctcc
atctacagctccatctacacctccatctacacaggttttaaaaaatttatctatgcaagtttcacctgca
ccacctaaacttaaattttctaattccactacagatttgacaactaaaaacaccacagaatctgtctggt
taccccgccttacagaaaaggaattcaagctgtactcaaaagagagttacgataaaaaatcttatgtagc
cccccaaactgaaaaatgtgcgactacagtcaaactactaagaccgcataaattgtcagatgattattta
gatgaatatttagaggagaaaagtactgaaaatagaacagtagatagagatcttaatattttaatgatca
atagtcttataatatgtcatttaaataaaagtccaaagtgtgttgttccacattttgaactggtacaaga
gactcaatggggtcttggttggatttggaaaatgaagtgtaccaagtgtaaatttatttcagaaaaatat
aaactatttagagaaattgacacaggacgtgctggtcgcaagagtgctacaattaactatgggtttcaga
ctggcgtgataaactgtaatattggtaatgatagcgcgcgactacttttaactagttcatgtattcctcc
tatgtcaaaaggaacaatgcagagaataacaaatactgtttgtgacaaggttgttaaaacttgcggaaaa
tgaaaccgaacaagtggttgagagtttcagaaagagaaatgaaaccttagggttaaataaaaatagtcct
ataaaattacaaatggatggtacctatcagagtgtgcatataaaaagtaggcataaaatgggacaaaatg
catcacaagtcattggtattgcctgtgaaaatgaaactgacaatcatgatatcattggttttcaccttgt
aaacaaactttgctgggttggtgcatggttacgcggtaaaggttatgacattgaatgtcctaatcacgag
tactgcacatcaaatacaaataggcatgatcctttgagtgagaaggatcttgggtatgaaataggtaaaa
aaaattgctaaacttgatttacttgtggactattgtactactgatggggatgcaaaaagtgtacaaggtt
tacaagaagcaatgcaagaaatatttgaccctttatggtcagtaaatagactggcagatactatacaccg
gggtcaaagtcagttccgtgaggtattgcgagcaaaatttagtgtcggtatgttccctggagctacaaag
gcccagaggaatgatattaaaatagcttttgccaatgatttaaaattacgttcacgcatggtataatgaa
atgtttatttgcaaaatataatggtgaccgacaacaaatttcaaaatgtttgccacgcattgttaagtct
gtcgtgaattgttatagtggtaattgtagcgatacgtgtagatggagcataacactatgtaatggtggta
taaaaactagttggtggtacaaatctattaaccttagcagtcatggtttacaaaatgggtctttgaaacc
aaataagactgacaaattattaatagaatcattgttagaaatgaaactgtctcagaccgcattaaatcag
atgcaatttttcagcaatacaaataagtgtgagtccgtcaataggacaatttctacatacctacctaaga
ataagaatttttctcgcaatgccttaggccgtgcatctgcagcagtcttgaaagtcaataataaacggga
tgttgcattggctaagaccctaaaagctgtaggttgtggtctgggtagaaagtctcgtgctgtagtggct
ctgaaaaaaatacgtaaacgtgaaatatatgattgtgcatatcaaaaaagtttacgtgtcaaattaaaca
ggttaaaagctcgtaaaaaacaggctatcgatttcttgttaaacaagagagtgcgaaacaggttgattag
tggatataaaaaaacatcagttagaaccaaagctatgtcagaattcaagtccgaaagaattcgttccaca
gccaggttgtagttcatggccagattaacatttatattaaaaggttacacaatatggttttctttcagcc
tttcaggaagcatatgtagttatttatttatttacagagaatactaaacatcttatattatattaaaaca
aacactttttcaggaatatatgagcatgatatgagttagttgtgttttttttttaaattaaaaagtttgtt
tattttaagtttgttatgaaaatgggataatggtccaagacactgaaataaaccttgtatcagacaaaaa
gaccacttcatatctcccaatgcatgcatgcatccagccacagtacatgcatttattggaaaacacatac
cttgaaacaaaattataaacaaaataaaaccatgtaaaatgaatattacaacattaggtcattcacagtt
tgcctttgtaactcgatgtcatatttggatttttttcctttctgttatttttttcttttgtttttttgttaa
cttatttaattaatgactgtcatactttgttcattgatttgtctctatgtttcactttcatttagttctc
taccgcatgtttacttcctgtgatcacctgttgattgaatgacccagttttctgacacagtaatgaaacc
atcaagagttactccccttgcactgtaaaaattagtaaacactggcgaaaaaaaccacataaatgtttga
ctgagcttataatcttgcataaacatgtttgccatgtttgtttcagtgtcttgggggtatgagggtgggc
tttttttcccattttcgtttttttacttgtgttaaaaacttgtattttcagagatgattaatgtggaggta
aaatgtgcaaggtgcatatgtttactacaaaatcataacaaaccacaatttcagattcagcatgcagtta
gtatagtttgtgccatgcacaatatgtgtggtgcattggtgtgcagctacacatatttttctttgattgt
taatctcccgaggttctaaaacaatatggtctgtattcacacacaatgcattatgacaaaggtgtgaagc
atctaaatttttggctaaatcagtagatttcaattgcaaaatttttgctaaacgtgcaacattcatttta
gtacgtttggcatttgggtatgtgacattgatcatccctagaatataattatttctcctctgagtggccc
cagtccaaataatgcatgccccattttctgcctcttcactgtttataagtataaaattggtatatttttg
ataaaaatcwaaattattggwagccattttgtattctttttaatcaaatagcactaaaagagtgtaactg
acctcgatttcagcagctcccatccgaaattgtgatcattgaaccatgaaacactccaacacctttgaaa
taatagttcacaattt

>KolobokP-2_MyGa-I
atattagatcagctaatgtcagattccacttattttgcagtttgtatcccttcagtacacatttctgaac
aattctgtgaagttttagccatatctgaaataaaataaggtcattttaatacttttaggtagcaatgttt
ataactgtttaatatcattttaagaaagaaaaaaacacaataagcatatgctataaattttcattttaa
acatttgttttaactttttttggtataactgcaattcataagcctcttttaccttacagactttctacaa
atcatctttttttgttgataaatcatgaaaatatagtgtttttcacttaaaaactatcagatttaagaaa
tttttccaccctgagagtcttgttttctattatattttttttattgaataaaaagctcttatatctttg
cattactgtaaagattagtcatagacctttccaaaactagtcttgtttttaaaatccatcaagtaataag
aattttagaggttttctttgtatgtgtcatgaaagtgggaaaaatgggttattttttttggtgaagagtac
attgtgacgtcatggtttttgtgacgtcattgcattatgacatcataatttagagttgtacacaacttac
agagtgtagtaattttgaaaaatgaaaggcaaaaagaaacgaaacaaatttaaggttggtaaccaagcat
ttttatcacgttatgattccctagcaacaaatcaagattgcaatggtgttgctccatctacagctccatc
tacacaggttttaaaaaattcatctatgcaagtatcatctgcatcatctaaaagtgaatttactaattca
actacaaatgtattgccaaaaaaaacccactgaatccctctggttaccccgccttactgaaaaggatttt
aaattgtattctaaagaaagttatgataagaagtcatatgtagcacctcagactgaaaagtgtgctacta
ccgtgaaactccttcgtccacataaagtgtctgatgactatttggatgaatatttaggggatacaaatac
agaaaatagaacagtggatagagatttaaatattttaatgatgaataatctaataatttgtcatataaat
gaaagtccacattgtaatgttccacattttgaacttgtgcaagagactcagtggggtctcggttggattt
gggaattaaaatgtacaaagtgtaagtttatatcagaaaaatataaactgtttagagaaattgagactgg
gcgtcctggccgcaagagtgccagcataaactatgggtttcaaactggtgtaataaactctaacataggt
aatgatagcgcgcgcgagtacttttgaccagttcgtgtattcctcctatggcaagaacaacaatgcaaag
aataaccaataatgtatgtgacaaggttgttaaacttgctaatgaggaaactaaaaaggtggttgagaat
tttaaaaggagaaatgaaaccttagggttaaataaaaatagtccaataaaattacaaatggatggtactt
accagagtgtttatattaaaagtagacataaaatgggtcaaaatgcctcacaagcaattggcattgcctg
tgagaatgaaactgacagtcatgatgttataggacttcacttaataaacaaactatgctgggtcggtgca
tggttacggggtaaaggttatgatgttgaatgtcctaaccatgagcattgtacagcaaataccaataggt
atgatcctttgagtgaaaaggatctagggtatgaaataggtaaaaaaattgccacaaatgagttacttgt
agattattgcactactgatggtgatgcaaaaagtgtaaaaggtttacaagaggccatgcaagaggtattt
ggtcccttatggtcggttaacagacttgccgatacaattcataggggtcaaagtcagttccgtgaaggaa
ttcgagcaaattttagtgttggtatgttccctggtgtaacaaaagctcagaaaaatgatataaaaacagc
ttttgcaaatgatataaaactaaggtcgcatggtataatgaaatcattatttatgaagtacaatggtgac
cgacaacaaatttcaaaatgcttgcctggtattgttcggtctgtggtaaattgctacagtggtaattgcg
gtgatacgtgtagatggagcataacactttgtaatggtggtaaaaaaacaagctggtggtataaatcaat
aaacctaaatagccatggcttacaaaatgggtctctgataccaactgacagagacaaattattaatagaa
tcattgctagagatgaaactctcgcagagtgcgttagatcaaatgaaatttttcagtaacactaacaagt
gtgagtctgtaaataggactatttccacatacctaccaaaaaataagaatttctcccgcaatgccatagg
tcgtgcttctgcagcagtcttgaaagtaaataataatagagatgttgccttggttaaaactctaaaagct
gttggctgcacaatgggtaaaaagtctcgtgctgcagtagctctgaaaaaaatacgtaaacttgaaatgt
atgatagtgcatatcaaaaaagttcaaaagtaaaatatcgaaggttacaggctcgtaaaaggcaggcgat
taattttctgcttcacaaaagtaaccgaaatgacgggtacaaaaaacatcagttggaaccaaagcttagt
cagttaactaactgcaaagagtttgttccgcagcctggttgtagtttttggccagattaacaaggtcaca
attcccatttctctttcagctatttcaggaaatagtacatgtacaaatgtattaatttatttagctattt
atttatttttatgattacagggactaataaatgttatataaattattgtgatgataaatcaaaatcttt
ttcagtaatatgcatgtgcatgttgttttgagttgagttatttctatttctataataaagagtttgtttc
attttaactgttttgaaaatgggataaaggtccaagacattgaaacataccacacacagcatcatacatg
caaatttagacacatgtatatattgcataaaaatttgtgtttcctatactgcacacattagttgaaactg
aacatacaaggaaataaaataaaataaaataaaataaaatcatgtccatatttacttccattctgtaatt
ttgcaactattttagttctgtaggctttttcttttactttttgatatcctaattttataattttgtagtt
tttgtttgatttgtttggtttttatattgaggatttcttttcactcgtgcttacttcagttggttcatgt
ggtttgattgtcccaatttcctgacacaggtaaaaatacatttgtaaacaacaagagttactccccttc
tatgtaaagtaaaatgtaaacactcgtgaaaccagccgtaaatgttttacctcaggttaataaacatgca
tgtttgtttcagtgtcttggagggtaagagggagggcttttatcccattttcatttttttgagcagtgta
agaaacttgtatttgagtaaatgattcgtgcataatgaaatgtgcagttgtcacctcggggagacttaaa
ttttagaattaaaaatataacaaaccacagtttaagatgcagcatgcagttaggatatgttgtgccatgc
agttcatgagtggtgcatttttgtgaagctacacagaccttacggtggttatttaaatcatggggctcaa
aaattatatggtcagcattaacacacaaggcattatgacagagatgggaggcatctaaatttctatcaat
atcaattgtttttagctgcaacatttttgacaagcgtgcaacattcatttttgtcctttttccatttgga
taagccacatttagcatccctaacatataatttcttttcctctgaatggcccctgtccagattttacacg
acccattttctgcatcaatgcaatttaacaacagaatattggtatatttttgataaaaatcaaaagtttg
agaagccatttttgtattgttttgattaattagcactaaaagagtgtaactgaccttgatttcagcagctt
tcatcctaatattgtagtcactgagccatgaaacactccatcacctttgaaattaaaggtcacaagat
>KolobokP-3_MyGa-I
tatagaaagaataaaaagttttcaaatgagactttatttttgcctgcatatacaactatctatgtctatc
acacacaccaaattaggaagtgatctgtgaagtcttctttgagaaatggacatttctatacttcatccca
tgtttgcggccagtagaagatttagtcagcaataccaactgaaacagaagtttaaatgaaaaattcaagc
attaaaattctacaaaaaaatacaaatgttgtttttcatgtacatataagcaatgtttctttgtgtatg
tataagttgatttcaaattttattcagggtggaatatacccaagatttattgaaaatagtgaaaaacagt
ctttttcatatattttcaaaacttttgtccaaaaaaaagtgtactgaagaggcactttgaatcattaac
ctctatcatttattaccaatgataagagaaataaagatgatatgcattaaatctggctgtcctcacaga
aactataaaaatatcaaaacattatcttgttgtctatttagaaattctgtaaaaatttaaggggtcatct
gtttcttttcaatttcttctataaattagtgagcatttgatttcaacttatattgcaaaaagaaattata
agaacattattcaaaatcagcatataaaaaaaaaagacagtgtagaatataagtgaaaattctgttgttt
tactaaatatggtattccaaaagggaaatacacacaaattcaaaggtggaaatgttccttataacaagaa
aagaaagactgtgaaacaaaataaaagtgactcttacacatctaagtacattagacttaccaaagaaaa
cacaatttagtagtgaatgatccgtatgctaatccacaggagaaatccaaaagggtttgccgtgctgcaa
gattactccgtccaatgagtgatgaggtgcttcttaccaaaccaattaggaaaaatgctaaaaaggacaa
aaggtaagctaacaattgaataatatttactcataaacagtttaagtgaaatatttttaccaaaaaagac

aataatttggaaataatagagtaaaaagtacaaaaaattgcccattttgagccactatgcacctttctta
attaaaaaagaacagtataatcatataaactgttatttttatgaatttaacaacaaatatattaataaaa
agaagcatcttcatacatattgcaagatttaaaagtctatgtaccatctaaaatcacacagagaaaaata
tctgtcaactaaggggcaggtaactctattttcaaaattagtaaataggcatatgtactttgctaagtct
aatttttatttttttgatatttttagaccggattacaattcaaacagagcaatatgtatatacaagat
ggaggaaatgtggaatgcagcatttaaagaacatcaattgagaaatccacagtgtgaaggtattctgcta
tgcgatttagaaaatgaagagaaaagaggctttggaacgcgacaacagctaatatgcacaaagtgtgatt
acaaatcaaagcggtacaccctgtatgaagaattggaaagtgggaaacctgggagaaaagcatcaaaact
tgacactgcaattcatgtcggcctgagtcaatcacccattgcttattctggtatacaaaaaatcttctta
agtggtaacatcttggctccaacaacatctagcctacaaagaagagcaaatacagtcatgaaacaaattg
aacaaattaataaacaggatatgaaaagaagaagaaatgatattgtagaaataaataagttaagaggcaa
ggaaaatccacatgctataagtgttcaaatggatggaatgtacaataatccactgtattctggtgtaggc
cgcactccatttcaaccggcaacacaaacaatttatacggcagctgaaaatgaaacatcaaaacacaata
tattagcattaaatatcaaaaacaagctctgttccaaacattctagtcttgacgtagacaatgactcagg
ccgacttcatgaagattgcacagatgattgcagtgcaaatatacccatggtaaaaagtataggagatgag
tatacctgggccagggaatgcctccttgatttaaaggaagaccaacttgaaattgagcatttagttactg
atgcagacagttctgcctataaagctgccatggaccttcacaatgagggaataaatactgttgagccaga
aaacttttttagacactagacatctttccgaccatgtacgtaaaggagcaaagtcagataaaactcttta
aaagtgatgccagctacaacaaaactaaaaagacaaaaacttttgaataatttctctgttgatctgactg
agagatgtaacagagaactattactggcctataaattttatgctggagacttttgcaaagtaaaaaataa
aatttctcacactgtggatgccatagcttactgctacatgggagatcatgccaggtgcagaaaaaaactcc
tttgcctgtaaaggatttcagggatcgtggttaaaaggccgtcctttcttaccaaattcttttaaaataa
gtagctgcaatgagaatttagactcgcttcggaaacaaataaataagcgacttggttccaaagtgcttga
aaagacacggttaaacatgaacaccaattttgttgagggatttaataggtgtttaagaagaatcattacc
ttcaaatgttactttcaagaaaaacatgtctggtcgtgcacatgcagcagctcacagcgttaataatggt
ccaggggagtctatattagaactctgtagtgctctacactgtgatattccagtaggcagtagtgcataca
aggctctgaaaaatattcaaaaaactgacattttgcagaaaaaacacaaacaaagtgtaaattataaagt
attttcgaggtgaaaaaaggtgcaagttatacaaattatatgaaaaactcagcgaaataatccaatatga
gaagaatatgctaatgaaagctgaccgcggcataaaatcatcaaaatcaaaacatgaagaccacccgtat
gttaagaacaagaagcagaagaagatttctattcgaaaataaataaatttagtacttttaaattaaaca
atatggtttgtgactctttgaacatagtccctggtttttacagtgcagtcggtccaagttaatagcatga
ggttctaacacaaggtggtttacatttacacatgatgcattgtgacaaatatggctacactctattgtat
tattgttttcatccacccttggcatatcatatctggtcaatttatgcctaatcatatatgccactctatg
tgcagcctcccttttttcctcccccctgtggccatttcacaacctgtatgccataaccagattttcccttt
tttgtgcccgaccactcaatacacccctttccattgacacttccctgggtttttttgttgaatttgtgtt
gtagttctctccaaaagtcatccatattgattaaaattcaatcaaaatcaattgaatgtttgattcagag
tcagattttctgaaaatccaatatcgaaagtgaaagtagagaaattctatattttcagtcaacctattac
cacaaaaaaaatatttccacagagaatactaaatcaatcattcc
>KolobokP-4_MyGa-I
tattagacccttwwtcttcaatataatgccccaaaaaatggcacgcattaagtttattctatatcsaact
agcataccaaatatcaaatcaatccgacaagttttatggcagttctgatagttttaataacactaccctc
akgttgataattgtaggtattttctggcwtgaaattwtaaaaacatgwaactactaggaaaagtaaaagt
ttaataactttcttgttaaacatgatatcttaaaaaagtaaaatgtctatagtagctaaatggttatatt
tctagaatctgaaagaaaatttataaaaaawtatgtttttttcaatwgaaattaattttttttcttaaattc
ttttwwwtttagctaacataccaacactagatataaaaaawtccattwaaaatatttaaaaagaaaattt
acwttcaaaacttatcttaaaaaattgcttatcttatctgtaataagtaaaatataacataaacactaat
tagwcawagttcacaggtacttggcctatttacctgaggtaccaatattcaagtgtaacatcaggtcaga
ttacatgtaaagamaatttcaaactwgtgtaatttctttattccttgaaatatcmcwctaaaataaactt
tttagtgaagttttccktttttgtttatccttatmaataaagagawtttaattttctaamagatttctgta
agaaaaaaaagttattaaaaawtatcctgaattttgasaacawaaattatctaaaamattaataaaaaaa
atmaatatgcttctttgttmaattgatcwctcaataaatkattaagtaacmtatttcmatagatttkgat
maaacaatcttwatctggacccctgatagtacawtaattggctcagctggatactgctaagaaaacwctg
ttaattcaaagttgcctctgaaggtggtctctkgaamwccatgccacagactgcatcaaaggacaaacat
gtacatcaaagcagatatcgtacaccacttgattataaatataaggatgtccaaagtttatgccattagt
ccaagttgtgatattactgatccagttaaattttaaaatgttcactaaaggttataccccatatscaaa
ggcaagaaaaggtcaaagaaagacaactctccaagaaatcctggatggttccaagcaggcaaaccaagtg
gtagaagcagaagccggacgtcaatacagatccctgattcagctagtgcaagtacttcctatgtgcggca
aactttacaatctcctgactgcaaaaaggcaactggtcacatctgtactcttcgaccgcaagctaatgct
gacctgaaaagagaagaaaacaaagcattacattctggtatgagatggatagacagtcaactaacaatca
gcttgatcaacactaatattatgataaacacaaagtggactcaccatcctgtcctaagtttattgctgac
tatcatgaacagaaaaagtggggaacttgctggcagtataccttgaaatgcaaaaacctgtggttttatt
ggagacagaatgaagatgtacaaggaaatagctaatggcaaaccaggacccaacccaggacaaccaaatg
tagccctagcatctgctttgcaggactgcccaattggaaatacaacagtgcagcaactacttgcaggaat
ggacacaccaccaccatgtagaagcagtatgcaacgcacatccagcaaagtagcagctgaaatggtcaag
ctgaacaaaacagacatggcacagaaactggaactagtaaaagaagagaacaggaggcgaggagtcccag
aaaatgaaattaacataactgtggatgctagacgatacaacagcaacacaatagttagtaaaaagaagcc
tggtcagaatgcgactcaagtttttgcactgggaatagaaacaatkacagatagaaagtttattgtagct
gctgttgtacagaataagatgtgttggagaggggcttggcttagagggaaaggctttccaatagaatgtc
ctggtcatgaagaatgtacagcaaatttatacagagctgctgccctgtcagagtatgagctgggaaaaga
gatggggaaccagataggactccagaaatgtctggtacggtatgtgaccacagatggagatggacgttca
gctaaaggtattgaagaagcccttacagctgtggaaccaatgtggaaagtagaaagactggcagaccatg
tacatttgggacagtctcaattcagggcctcttttcgagctcaatacagcccgggaatgttccatggtaa
aacmaaagaggaaaatagacaactwaagacagtgtttagcaaagatmtwaagtgcagatgctccatgata
wtwaakaaactgatggagaagtacgacaaaaacattmacgawatgagcaaagacttaccaaaagtkctmg
atgcwaccctccgctgctatgatggtgactgcacccwgtgtcakgaacattccattgtctgcaaaggtga
tgcatgcactcaactggtggagtcgttcmcattacttgtccacataccagatcactgccctgcaaatgga
taaaacagacaaatttttactacaagaaatcctgaaaatgaaactaacaaaagcagctgttgaaagtatg

```
aaactgtatgataatacaaataaaaatgaaggagttcacagagcaatgagtgtaaatttgccaaaaaatg
taatttactccaaaaatatggaaggacgacttgcctcaggagtgcatcgaaataacaatatgccaggaac
ttcaacccaactgaaatgtaagaatttaggagtagacctttcaactagaagtcaactttacctgaagaaa
atggacaccaacttcaaatacagtcaggattatgaactgagaccagcagttcaagaaaggagattacagc
agagtgcagaaaagttagccgagcacaaaacatggagggaaatgaataaaaagcacgattattactgcaa
agaccaattagatccacaacctgccttacatttccaggaccatgacagctactgtaaaccgaagctcaat
ccaagacctgctgctgactgagataccagctgatccacctaaagaccaacagcaaacagaatcataaaag
gtccttttaagggccacacaaatattaaaaaaagaatctgaatttgttgtacatcagtcttgaaatttct
tttctttttctccctcatttcaatttttttgccagcccttcccttggatagttataaaaataaatgtacc
caattaatattttacccaaattattattatccattttcaactccactctacagggagggaggggttctgg
gtggcccaagcaggaacaggttgaaacgcatgactgtcggctgttgttgaagccatgaggttccaacgac
agatgcccgacagtaatacattttagaattgtggcacaggtgcgaaacatcataagatttatcaataaaa
atattctgatgatgcaccatgtaggcaagcctgtgcacatgcatttgtctccaccctctggcatctggaa
atttacaatttataaccccatatttgccattaggtgtacataaacctgtccaaatttgacactgcccatt
atagccaggtgcagagctatcattcagtttttgtctatatttttgtaaaaatgcatccattttgaagata
ttgttttagaaagacataagagtagtaatatattctaaaaataaaaagggtccaacagaattgacagaaa
gggctgaaacaggggtcaaaggtttttcagtgcatcaaaatggccaaaaatacccccaaagtgcccatta
tcccagaatcatgatatatataagttaaaaatacaataatcaactagtatttatcatttgaagtgacttt
ctgccaataaagttggtcaattctttattttcaagattttgggaatcagcctctta
>KolobokP-1_MyCo-I
tattagacccttttcttcaatataataccccaaaatggcacgcattaagcttgttctatatcgaactag
cataccatatatcaaatcaatccgacaagttttatggcagttctgatagttttaataacactaccctcat
gttgataattctaggtattttctggcatgaaactataaaaacatgaaactactaggaaaagtaaaagtgt
aataactttcttgttaaacatgatatcttaaaaaagtaaaatgtctatagtagctaaatggttacatttc
tagaatctgaaagaaaatttataaaaattatgtttttaattgaaattaatttttctttaattcttta
atttagttaatataccaacagtggatataaaaaatccatttaaaatatttaaaagaaaattcactttcaa
aacttatcttaaaaattgcttatcttatctgtaataagtaaaatataacataaacactcattagtcaaat
ttcacaggtacttggcctgtttacctgaggtaccaatattcatgtgtaacatcaggtcagattacatgta
aagaaaatttcaaactagtgtaatttctttattccttgaaatattcctctaaaataaacttttagtgaa
gttttctgttttgtttatccttatagataaatagaatttaattttataacagatttctgtaagaaaaaca
gttattaaaaaatatactgatttttgataaataaattatctaaaaaattaataaaaaaaaataaatatgct
tctttgttaaattaatctcttaataaatgattaagtaacctatttcaatacatttggataaaacaatctt
tatctggaccccctaataatacattaattggcttagttggatactgctaagaaaactctgttaattcagag
ttgcctctgaaggtggtctcttgaaatccatgtcacagactgcatcaaaggaaaaaatgtacatcaaagc
agatatcatacagtgcttgattataaatataaggatgtccaaagtttatgccatcagtccaagttgtgat
attacggatccagttaaattttaaaatgttcactaaaggttataccccatatagcaaaggcaagaagagg
tcaaagaaagacaactctccaagaaatcctggatggttccctgtaggcataggtggtagaagtagaagcc
ggacgtcaatacagatccctgattcagctagtgcaagtacttcctatgtgcggcaaactttacaatctcc
tgactgtgaaaaggctactggtcacatctgtaatcttcgaccgcaagcgaatgttaacctgaaaatagaa
gaaaacaacgcattacattctggtatgagatggatagacagtcaactgacactcagcttgatcaacacta
cttatgataaacatcaagtggagtcaccatcctgtcctaggtttaatgtggactatcatgcacagaaaaa
gtggggaacttgctggcagtataccctaaaatgcataacctgtggttttattggagacagaatgaagatg
tataaagaaatcgctaatggcaaaccaggacccaacccaggacaaccaaatgtagcccttgcatccgctt
tgcaggattgcccaattggaaataccacggtgcagcaactacttgcaggagtggacacaccaccaccatg
ccgaagcagtatgcaacgcacatctaacagagtagcagctgaaatggtcaaactcaacaaaacagacatg
gcacagaaacttgaacaagtaaaagaagtgaacagaaagagaggtgtaccagaaaatgaaatcaacataa
ctgtggatgctagatacaacagcaacacaattgttagtaaaaagaagccgggtcagaatgccactcaagc
attcgcacttggtattgaaacaatgacagacagaaagtttattgtagctgcagttgtgcagaataagatg
tgctggaaaggggcttggcttagagggaaaggctttccaatagaatgtccaggccatgaagaatgtacag
caaatttatacagagctgctgccctgtcagagtatgagctgggaaaagagatgggaaaccagctaggact
acagaaatttcttgtacggtatgtgaccacagatggagatggacgttcagctagaggtattgaagatgcc
attaaagctctggaaccaatgtgggaggtagaaagactggcagatccagtacatttggggcagtctcaat
tcagggcctctaatcgtgctcaatacagtgcgggaatgttccatggtaaaacaaaagaggaaaatagaca
actaaagacagtgtttagcaaagatatcaagtgcagatgctccatgataataaataaactgatggaaaag
tacgacaaaaacatttatgatatgagtaaagacttaccaaaagttctagatgcaactcttcgctgctatg
atggtgactgcaccctgtgtcaggaacattccattgtctgcaaaggtgatgctgcacttaactggtggag
tcgttcccattacttgtccacataccaaatcactgccctgcaaatggataaaacagacaaatttttacta
caagaaatcctgaaaatgaaactaacaaaagcagctgttgaaagtatgaaactgtatgacaatacaaaca
aaaatgaaggagttcacagagcaatgagtgtaaatttgcccaaaaatgtaatttactccaaaaatatgga
aggacgacttgcttcaggagtacatagaaataacaacatgccgggaacttcaacccaactgaaatgtaag
aatctaggagtagacctttcaacacgaagtcaactttacctgaagaaaatggacactaacttcaaataca
gtcaggattatgaacttagaccagcagttcaagaaaggagattaaaacagaatgcagaaaagctagctga
gcacaagacatggagagaaatgaataaaaagcatgaccattactgcaaaggccaattagatccacagcct
gccttacagttccaggaccatgacagctactgcaaaccaaagcttccaagaccggctgctgactgatata
cctgctgatccaccaatagaccaacagcaaacagaaacataaaaggtcctttttaggaccacacaaatat
taaaaagaatctgaagttgttgtacatcagttttgaaatttcttttcttttctccctcattctgatatca
atttttgccagcccttgaataataataaaaataaatgtacccaattaatattttacccaaattattatt
gtccattttcaactccactctgcagggagggaggggatctgggtggcccaagcaggaaccggttgaaacg
catgactgtcgactgttgttgaaaccatgaggttccaacgacagatgttcggcagtaatgcatttggaat
tgtggcacaggtgggaaacatcataagatttatcaataaaaatattatgatgatgcaccatgtaggcaag
cctgtgcacatgcatttgtctccaccctctggcatctggaaatttacaattaataaccccatatttgcca
ttaggtgtacataaacctgtccaaatttgacactgcccattatagccaggtgcagagctatcattcagtt
taagtctatatttttggaaaaatgcagccattttgaagatatactgtttagaaagacataagagtagtaa
tacatacttaaaataaaaaaggggtccaacagaattgacagaaagggctgaaacaggggtcaaaggctt
ttggtgcattaaaatggccaaaaatacccaaaaaggccccattatcacagaatcatgatttatatcagtt
aaaattacaataaacaattagtatttatcatttgaaatgactttctgccaataaagttggtccgttcttt
attttcaagattttgggaatcagcctctta
```

>KolobokP-2_MyCo-I
taataggacccttccagtagtccaaatgaaataaaatttacatacattaattttaatatatatcaagac
tacataccaaatttcagaccaatcagacaatgcatgcaccagcaaatgcatttcttgcacccctaccttc
acacccaaattttatcatttttctggtatggaattttcaatttcctcaaaataccttctttattcaaacg
ctcataactttttttattagtgcaagttttatgaaaaactaaagtgtttgttaaagtagaaacattgataa
aatttaatcaatgataaaattttccaaaattagaacgcataattgtgtaactaaggttatttttctaaat
tcatttcaaatcacgaaaattccaaaccagaatatttaaaaaaatcaatgaaaaattcaaaactcaacct
acagttttcagaattgcattaaaaaattctatagtcatactacaatacacaaaatttaactttatggggg
tcttgaataaacttttggcttatttacctgtatattcaacacccccctttacaccatctgcccgaggcaaa
atcaagatgtcataaaaactttaaaatgctgtaaaatccttattttttgcaattttgagatgaaataaag
tgttttggaaaggttaagatgccaaaaattcatttcaattaaaaaaagtaacaaattcaacagatttta
aaattaacttttttttttcaaaatcaaacaaaaatagaaccaaaaaattaacaataaattacaaaatcact
tttcaatggcaaaacaactttctaaaatttggaaaattaacgtttgtaaaatctgaataggaaaagaatt
tttatttcacaaataaaggccaaaataaggaattttgtttgaactggaccaaaccctttgaagccagagt
tggtcatttctttaatctgcagctaaaaatagatcaaaagaattcacagaattgattgacagcatcactt
agccaattaaaaatctgaatcaaaagttacataatgacaaaaagtataaaaactaacacttctatggtct
gtcatccaagtttctttcgacgatttacactgcaacaccaaaatatttgtctcaacatgtatccaaaagg
ttactggccattcaacaaaggcttagctggacggctgcagagaaggagagttgcacctgttggtagattc
cagaatggcagaattccctggaatgttcctgttcctgtccctgtactagagttgtccactacaccagacc
aagatatcaataatgctcctgttgttgaaatgaatgttgaacagaaggacattaaagatgaacctacaat
cacttctacttcagctgtacacaatgatggcataaagactcgttcccagctactggtatcatcctatgac
ctgaaaacagaagaagaagattggagttcactttctggtatgaggttcgtcgatcatgaagagatgatga
agatgctgaacattgttataaaggctcacacaaaagaattcaagtactgcaaaaatccagaaatactggc
acacagacaagttaaatggggagtttgctggaaatatacccttagatgtaagaactgcacctacatgagc
ccagttataaagttatacaaagaagttgtcaccaataaacctggtaccaaccctggagcacccaatgttg
cagtagctgcagtcctacatgactgtcccatcggtagtacaaagttccaggagctgtttgctagaatgaa
ctgtcctcctccatccagaacaagcatgcagagaatgtcacataatgttggaaaggagctagtaaaactt
aacaggacagacatgtcagaaaaactggagattgttaagtcagtaaatcgagagagggggctacaagaaa
atgtgattaatgttacagtcgacggaaggtacaacagtcagaccattaccagccgcaagaaaccgggact
gaatgccacccaagctttcacacttgccattgagacaatgacagagaggaaatatattgttgcatcattt
gcccaaaatcagatgtgctggaaaggagcctggctgagggggaaaggttttgatgttaattgtcccaatg
gccacgaagactgcactgcaaacttatacagagcagctccagtgtcagagtaccagatgggtaaagaaat
tggttcacagctggcacttcaagacatccttgtaaagaatgccacaacagatggagacggacgggctgcc
aaagggatagatgacgcaaccagagccctacatcccatgtggaaggtcgaaaggctagctgactatgtcc
accttggccagtcacagttccgagcatccctgatggctcagttcagcgaaggcatgttctatgggcgtac
aaaagagataaagaaagaaatgaagaaagcttttagtcaggatgtcaaatgtaggagttcgatgatagtg
ggacagctaatggaacagtacaaaagaaatacagatgacgtatgtaaagacttacccaaggctctgcagg
cgacaatcaggtgttatgatggtgactgctccatgtgtaaacagcactcagtggtatgtgcaggtgatga
gagctacaactggtggacccgctccaagtatcttggctgctataacattactatcctgcaaatggacgaa
aaagacaagttgttactgcaagagatcttgaaaatgaagcttagtgaagatgcgattagtagtatgaagc
tttacaacacaacaaacaagaatgaagctgtgcacagatccttgagtgtgaatttacccaagaatgtaat
cttctcaagaggcatggaggccagattagcatctggaattcacagaaacaacaacatgccaggaacctca
gcaaagcaaaagtgtgaacatctgggggttaatttatctgattccagtttaaagttcctcgacagtatgg
acaaacagtttacctacaagcaagagtatgaaaaaaaaacctgaagttaaacgtagacgtctcacacagag
tggtgaaaaaatagtagaacatagagaggccaaggtaaaaagtaaagagcgagatgtctacagaaaaggg
cagttggacccagtgcctgccttacttagccatagtacctactgcaagagaaaactaccaccagcaccat
agatgagacagcaacccagcaacacagtaactcaaccaaaggtccttttaaggaccacccaaatctttca
aaaagatatctgaaattgttgtttacatcaataaaataaaataaagtaaatatttaatttataaatccta
aaacatgaataataccaaggttattgtttgccatctttaagtccaacttgcaaggtggcaatggagctgg
atgacccaagcaatggccacatgaaatgcaagtttttctactgttgttgaagccgtgtggctccaacacc
agatgatcaacattaatgcattttgtgttgtggcacagatgcgagacatcaaaattaggtaaatgaaaat
ttagatgccttatcatgtacgcaagtctgtgcacatgcatttgtctccacccgccggcattgggatgctt
gaaatttatcaccccatatttgccattaggcgtacaagcccctgtccaaatcagacagtgcccattttgg
ccaggggcagatttttcttgtaattttgctaaatatttttgaataaatccctccatttaaacttcaaaga
actatttcttatcagaatgagtaggaatttgctcaaaatagaaaaattatgagcccctgttttgagattt
gccccaaaagtttcaacatataaataacaatccaaaaatatggcaaaataggattttttttggcatttaa
aaatattttccaattatttactattaattaacaatgaaatatctttacatttcaattttgaggcaaattg
tgcaaatcaggggaccaaaatgtccgttttggggtacagtgggttcagcctctta
>KolobokP-3_MyCo-I
tatactgtactttcaaaccccttttagggtcaaaattttcttatattcttgttgttatttatgtaagtaa
tagaaaatactaagttacaatggtagtttttcattattattttttttttagtacttctagtaactataccc
agtttgcggcaaggtgtgtttttttccttttcactagatttttttttttttgtatgcaatattttattttt
ttttgtcttcttaataaactcattatatatttgtgtttttcttgtgccttgatgtaatagctatttgaat
catggttagtttttctacaatctgtctattttgtagaaattttgtcttctttatgtactataccataaaa
tgcataaaaagttgcatacaagcttcaaaaaactatccctataacaaaaacatacaccttatctttcctt
tgtgtttatgttatagcacacaagcctgtcagcactgataaaaatataaaatgaagagaggtcaaattaa
ataaacttgtcaaattacaaaataatgtgattggccatcatgatgattttatatgatttatataaaaaa
tcttacatctggggtcaccttcagtaccatcagatataaacaaaaacttagtcattcatgattgtttagt
tgtaaggtcctatatttgatgtttaaatattgaaatactggaataaagtgcattttaaacaattcttaca
ataagaatgggtctaatcccatggtgtctaaggaaatacagatttcagaaaggaaacaagccatataata
aggggaaaaaatacagaaataagactttacctgctgaaaaaggcaaatttgtgagactaccaaaagactt
gtatgacttagtaactaaatctgttcataaaaagaatgttcaagtaattcctgcctctacaaagtcagct
cgctttctaaggccgaagcctccgacaaaaacagaagttgaaaaatgtgctgaaaatgaaacaaaaacag
ggtaagaatactttttttgttattaagctttcaaaataattaaacttgcttaaaaagccacttattaaca
actatgaagaaataggcatgtgtgttatatgccaattcttaaaatgtgacatctgaagaaaatgcaagct
aggattatacttattttaaaatttctagtcctatatatatttttacaagctaaattgtgcatggttttat
atattcaatatataaaacacagacaaagaaaaaatattcactttgaaagtaaattttgtcaaaaagctt

atttaaagaaaacaccattgtaagggaggtaattcaaaatagaattattttcacattttccaaacattag
ttttgaccaagaaaactaattcatttcaattttggcaacatttttcaacttcaggtcagagaaaatagtt
ttcgactccttcgcatcaatgaactggaacagatgtggaatgaggttttcatagaacacagacaaatatc
ccctatgtgcactgggttcatatcatgggatttaagtgcagagcagcaaagaggagctgcctggagggaa
cgggcaagctgcaatgagtgttcttaccattcaaaaatgttcaacctttacaatgaggtggtcgctaaga
aacgtggtcgtcgaacagcagctattaatctatccatacaagtcgcacttaatcacatagctatcagcac
aactggtctacagaaattatttcttggttctaatataccagctccttccacttcaagtatgcaacacagt
gctaatgttgtttctgaaattatagaagagtacaatcaaaaggatttggctcagaaaagaaaattattaa
aggaaataaacatattgagaggcgataatcctaatattattaacattcaggccgacggaatgtataataa
ccctatttattccggaatgggtaaaacaccatttcaaccagctactcaatgtacatacaacatgttagaa
aataatacttataagcacagtattgtaagcacaagacaagtttctaagctttgctcaaataagagtgaac
ataaaacaaamacaaaagttaaatctcacatgggccactgttccgcaaacattgaaatgcatgacagcat
aggcagtgaggagcgatgggctaaagactgcttgttagacctgaaaaatgatggtcttgaagttcaagaa
attactactgaccctgacagcagtgcatttagggctgcagagtctctttttaatgctggtactaccaata
ctcaaccaattcatttctagatacaagacatgtgtctgcaaatcatagaaattttattaaaaaaatgtc
aggtctgaaggaaataatgccggcacgattagtttcagagaaagataaagtgctaaaacggtttgcatta
gatatggcagcacgttgtcaggcagaatacaatgctgcctttgaaagattcaatatggactcggtacgga
taaaagttcacctatcatacgcatgtcatgctatagtttcttgctatcaggagatcataatgggtgtaca
aaatattcccttgtttgttcaaataggaatagtttgaagacatggactgaaaaatcagcttatctgaaaa
ataacttcaaattaaataaatcagagaacacagcagcattgttacgtgaatgcgtaaaatatcgacttgg
acctaccatgctaaaccgtacatgcaaaaacactaacacacaaaaagctgaagcaactaaccgtgcaata
cgtgcaactgtacctagcaatgtaacatttaccagaaactacaaaggaagggtacatacagctatacaca
atgtaaataatggccctggagaatccatcgttaaactctgtaaagcggctggggttcccattgaaccagg
aagtcgtgctgcacgaggtctgaaaaatattcaacgacacaatgaaaaacataaattatacaaacagtca
aagcgatacacagaccagagatgctcaaaaagcatgaactctatgaaatatatgatgaatatcaagagga
aaaagactatgagaaaacaaacttttacagacaaaacgattggccgcaaaacagcctcaagagtatttgt
ttgccaaaaaactcagatcaagacatgttaaaaataaataaattacctgcatcagataagacaatacggt
aaatgactttttgtacactgttttatccgtctacagaaaattctatctatgtttatactgtgcggctcta
aaatcaggtgctccggatttatacatttagggttatggcaaaggtgtgaaatatctaaatgatcaccgtt
tggagagtaacggggaagatttcttttaatattcttttatctagcatataagataatctgtgagctctt
tcaagagttctagtgccatcaggccaattaacaactttttcccatagaccccatgctccaaagctccta
tccacactgtacagtcataatttccataattttttcacttttcttatacaaagctaaagccaatgcatt
ccaaaatgattccatgttgtttctgcaaaatgaaaacccaaaaggggtccaacatttggttcccagggag
gctgagtgatgaattcccattatttaccttata
>KolobokP-4_MyCo-I
ctataatagacaattcttcctcaaatcatacttttcttgtattaattatgtaatttagcctattttcaag
ttgaagattcaatacaagatctgtaaaggcttaattgttaaagtttaaggaaaatttgtactatagccca
tattgcggccaatgtatattttgtacataaagttgtatattgaatgttgtataataaatgatgtaaaata
tattgtttaataaatatgaactgtttttgtgtttgtttgaacatatattgccagttaatcttagaaacat
aagtcatgttatattctgagattaatgtctccatatatggaagttttaatgagtataccctgaaaatacc
acaaaagttgtttcaaaggcttttaaatgagtgtcatacaaaaaaaaatcaacaataatatttcctacac
taaatctgtgttttaatatcttgtatagatcatttaaagtgcacacagataactatagggtcatttacta
tcaaagatgagaatgtcttatcatactggtagagtcagagaaaaacagacttttcttcaactgtcaacca
taaaatgatatcttagmaataaaatttgtcctgtttctgcactttccttcttcttacttttgttaaatga
aatcagagagtgacaaatattgttataagaataattatttcatcaaaaamttactaaaaatgggacgtta
tgctgtgcacttaaaaacacagatttaagaaggaaatattccacataacagaggacttaaattggaaaat
gttaaaatacctgtattccctgtggttaaatttgaaagactatccaaggatgactttgatcttgtgacaa
aacctgtaaatatcaaacactccgctgaaattcctatgcactcaacatcagggcggcttctgcgtccaaa
acaggcagccaagactgaagtagaaaatgtgcagagaatgaaaataagactgggtaagatcatactttgc
attacaattgataaagcaactatttacagtatttaatagaaaattttaagcatcacattagaaaaacaaa
cattttatcaacagtttatagatgaagggtatctaatactgaaaagttctacagaatatttttgaattga
agaaacgcattgaatgatgaaataaatatattatttctacataccaaataattattcgaatgctaaaata
cttgaaaagtgcttatttacaacaatatctgcatcacagccttaaaaacactgctctgaaaaatacagtc
tcaaagggagataattctaattttacacatgatactacataatactacataatataaaaaagaaaaataa
gcaacatttgctactcagtgatgtaaaatagggagttaattaaagtctttcattaatttttttacctccag
atctgaaaaatacaccaacagaacagtaacaatcagcaaaaacacagaaatgtggaatgaggtgttcmga
gagcatcagaatgtatccccacattgtaatggcatccttgaatgggacttgtcaatggaagagaagtggg
atctgcttggagagtgcgcaaaatgtacaaaatgcacatacagatctaaaatgtttaacttgtacgaaga
ggttgcaagtataaaaaggggacgacgcgcagcaaaaataaatcttggtttacaagttggtctacatcat
actcccattagtacagcaagttaccgtaaaatctgtatggcctcaaatattcctcctccatctgtctctg
gtatgcaacacactgctaatgcaatatctgaaaaggtagaagaagaaaacatgagagatttacaacgaca
aagggagaaaataaaaaggataaaaaagatacgcggggaaaatcctgatgttgttaacattcaatctgac
tgtgtatacaataatgctatttattctggcattgggaggacccctttttcaaccagcaacccagtgtgctt
acacagtagctgaaaatgaaacatataaacatagcattataaacacattaccaaagtctaaactctgttc
gaagagaaagcatacagttagtacgaatgtcggtgaacatgctggtccgtgcacggcaaatattaacatg
caagacagtataggtaatgaagagcggtgggcaagagagtgctttgaggaaccaaagaggatggattaga
agtaaatgaagtaacaacagacccagacagcagtgcatacagagcagcagagagtttatttcaggaagga
ctaactacaacaaccccccatccatttcttgacaccagacatgtagcatctaaccatagaaaatttgtaa
ataatctatccacagtttgtgacattatgccggctcggttaaaggtgcaaagaacccggctacaaagttt
gtttgcttctgacatggctgcccgttgtcaggcagaattcgtacaggctaatattagatatcacaaatcc
cctgatgtaatgaaatcaaaactttcatatgtttcagatgccatagtgggctgttactgtggagatcaca
cagattgttccctatattcatttgtttgttctatatctagaaaatctcaatcatggattgacaaaagtgc
atatttaaagaggcataattttgaaattgagctaaatgagaacagtgaaaatattctgcgacaatgtgtc
aattaccgtctgggtccaggcatgcttgctaaaacagctaaaagtgcaaattcacaaaaagttgaagccc
taaatcgatccatacgtagcactgtaccagttaatgttacctatgctagaaactttacaggccgggtaca
tacggcatgtcacaaagtcaatcatggtacaggcaattctatcgtcatcttgtgcgaggctgctggaagt
ccaatacaaccaggtacaaaagtggcaaaatctctaaaaaaattagaagaccatagtgatcaaatgagaa

attgtaaatactcagaaaacagtatcaatcatagaaatgcacgaaagcatgctctgtacgatatacatgc
acggcatcaggaggaaaagattatacaaaaataaactttaccggtcactaaaacagtcctatgtacccc
aaagaccactcatatggcagactccgcaagtctaccaggctttcaaataaataatattcaccttaattta
taataaacaatatggatggtgatcctttgtacatamatttctcattctacaccscattctatccatattg
agagagtgtggctcaagaattaaatgttccggcttcagacagatggggttatggcatatgtgcgagacat
ctaaccgctccccctgtaaatttttatttggcattgcatccctcaaaatatgatgtgcatgcatgtagga
cagcctgtgaaccctttctatttttctttccatcaggccacacaactgacttcttcccatagaccccat
gctctctaccaccagtccactcagtacacccatgccgaccgcaattaaaaatagacttttgcattaaaga
tagtgctatttgattccaaaakgcttccattgcattaatatttaccagaaaagaactttggggttctcag
gtggccttgaaaatgtacagtgacagagtgcaaattttctctatag
>KolobokP-5_MyCo-I
tgtcagactttaatttttgttccagacttaaaactgcacttttaaaataaagatatacatctcagtagtt
attgtgcaaagtttcaataaatttgatccatcagttacagagttattgatccttatctataacatctcaa
ataccagaaaattggtcggataaaaatataaattcatgaagtaaatatgcttaaaatcagcaaaactttt
ttatttcaaaagatatttacattctgtaaattgcacatggtagataaatttattctttgtatgaaagtct
agtaaaaattaaatttcaatgaaaaaaaatttaattaaaatcatttttttaattaaaaaaaaaaatcttt
ctttttttataggaattacatatgcaaagaaggtaaaagttaaaaacttacaagaaatgtatatttatg
aaggcaaataagtaccttatcatttatgaaaagcagtggtttacatggtaatgcaattagtggtaatcca
ataaaatgtaagccctactgtacacacacatgatatcttagaccgcgttgtccagcacctgtaaatcccc
ctaagagtgtcactgtcagcaattagtggcaaaattgtgtcatggaagctgacagcatcttcgaatgata
taaataatgcaggaaaatatagtacttcataaattatcagtcaatggtactgtaaattaacatgaaaggt
gaaaaaaatcgacgtaaatcttgcttcaaaccgggtaacataccccataacaaggggataaaatatgaaa
cacagtcttcaaacagtcagctaagttcatctaatatacaatggaagaggctagatgcagagaagttctc
acttgtagctaaaaccagtcaagacggcacctcctacagtttaccagatgtcgagggaaatcctggatct
gctaagttattacgtccacatccaaaatctttgaagattctgaaaaaagaagctatatttgagtctcatg
atgaaatacatgttaaagatgacacatatagagctgttaagatgaagaaaattactctattgataaatgt
agtggttaatcttcacaatttacaagacattaaatgtgaaaatccaaactttgaactgacaaaggtaata
aagtatggatctggtgtaaagtgcatatataaatgtacaaaatgcaagtttacatcaccacgtgttaacc
tgttcgatgaaattaaaactttgaaacctggacctaaccctggagagttaacccgcatgtttgtatctgc
cttacaggagactcctatgggaataaaaagaggaagatttctgatggctgcaggactaaatataccacca
cccactaaaagaacactgcagaggcattctaatgttgttgcaaatgagattaaagagttaaatgacaacg
atatgaagaaaaaacttgaaactgtgaaagatgttaacagaattcgtggtgtaaaagagccgtcacatat
tcctgtagcaatcgacacacgttataacagtatgcatatagtgagcacaaaaaaacccggacaaaatgct
tcccaggcaatatcattggcgtgtgagcaagtgactgaccataagttcattgttgcatcagtttttcaca
ataaactttgctggacaggtgcttggcttaaagggaaaggacttgatgtaacctgtccagatggacatgc
tggtacatgtacagccaatgttaagccaaccacgcctttaagtgaatatctcatgggtaaagaaattgga
cttcaggttgataggcaaaatgtgctgataaagtatgcagttacagatggagatggacgtggagcagaag
gtataaatgatgcactaaaagttttacacccgttgtggaaagttgaacgccaagctgacccaattcatct
aggacgatcacagtttcgacaaagtaattctgcaaattttagtttgaacatgttttctggttcaacacgt
gaaaagaaaaaacaaggacagaaagttttaagtcaagatttgaaagccagatgtagcttagtgttcaatg
aaatgtggaaagaaaactgtggaaatttggaaaagattaaaaaatgcttacctagagtgcttgatgctac
tgtgcgttgttatactggtgactgctctaaatgtcccactagttcatacgtgtgtggtggtggacacaca
aactgttggtggctgagatccatgtacttgggtgcgaacaagttaaccaacctgaatatggataataatg
acaagttaataatgcaggaacttttaaaaatgaaacttagcgaggaggcattaatgaaaatgaaatttaa
tttcaacactcaaaaatgtgaagccgttaacagagcactaagtgtatccttaccaaaaaatgtgaacttt
tctagaaattttgaagggagggcatcagcaataattcacactttgaataatggtataggtaactcctgga
gacaaaaagcagaatgtatcggttttaaaatatcgcaaggttcattaaaacacttggaccaaatgcaaag
ggagtgtgaacggaacaaagtataccaaaatactccagaaaggaaaaagaaaaatttacaacaaaccgga
cagaaaattaaggatcatgtggaagaaaaggaacaaagtaaatttacagacagatatcaaaaaggacaac
ttgatccaaaacttgatattgcatttgtaaaaagcataaaggacgatcattcatatgggagagtaatgcc
agctaagaagaatatacctaccagaagctgtacttcaaaagttccaaagtcaaagaagatatagctgcat
tcatctacagctgaaaaagataaagacacaattaactaaaggtccttttgaggaccaccaaatctttaaa
aaagagtctaaaattgttgtacattcagtcaaaattctttatataagctatagccctaccccttaagtat
cttccaaaaataaatccaggcggcaatctggaaaattggcatggccctggcaagagtttctgtgtttgca
ataaattctgttgttatttatgtgatgcggctctaaattaatgtgagccgcattcacacacaaactattg
tgacacaaatggctggcgtcaaatgttttatttaatactaaataattattgaaaaccataaaactgaatc
tatgggctttttttcttttccaggtcccagatataggatccaggaaagaaataagcccatattttccatc
ttttgttgtctcctgatcccatattaaacatttgtcatttcttgccaggtggctcttttctagtattttt
tgcttatatttttcaaaaaatccctccattttaacttacaagtgtatgaatacatattaccaacaaaatg
accttactgtgacccctttttaagctttcaaatgaaaaaaaaactttttttaaatttcaaaaatttaaat
ttcaatttaattttaaaagtttaattcaaattttaaaaaaaataacaccattgttttccttatttaattt
tgaagttccttgcaaaatttcatccatgtgactcgaaaaataagcttacagtagcagattgaagtgttca
atatatgccctctgaca
>KolobokP-1_MyEd-I
ttaagcaattagcatgtcttatattaaccaatttgtgcatctagttgcaaaaccagtatcatttcgagca
ttttggtgtcttgatcttttatttctatgcagatttggccgagttattaacattttagtatacactgcgt
gaaaccttgaatgacaataaaaaaaataaaaaatcagaatattcaatttgtatagacatgtttattgcaa
ctcatttttctgttgaactaaataaagaacacaatcaacagagactttctcttctagaaaagttcgaaca
taatgtattatttgccatataattgtactgtatggttaaaatggtgtacacactaaccagaattctttac
ataagaaaactcaaaacagcataaaattcaatgtctaacaattaacaactaaatatagaatgttaacctg
ctaaggaaattctaatatgacatattgaaccatgaatatctaactttttttcatttcatatcatcacaaaa
taaaaaaaaatgtatgtttctatagcacatagttcatttgtggtcaaggggggagataatcaaagcattaa
ttacaaatttacaaattcaaacaccaaaaaaatgcagaaattcaaataacaaatttaacaatagtgcagtt
gcacttgacaattttattaaacatatttttctgcgtataaaataaaatatattgctattgtctattcaca
tctttaaaacaatcatgacgtcatagcaacatttgtgacgtcatcaataccagatcagatcatttcttgt
actacagtaaaaattcagtgttcaaaatgtctagctatgataggagaaaaaacagaatacaaagaagaag
aaaaagtaaatttgtcaaagggtacacaccttggaatgacaagttggaatatattacatcagatactgag

gcagaacatcaatatattaacaacaggatctgtaggcctgaaatgaagatatataaaactgcagtgtcac
agcatactaaggaagataatacattgccaacaaaacttcgacctcgtcattgcttcatggaggaagagga
aactgaagcgcaagaggacaccacagaaaatatcattgttaatttttgtaagttgaatgattttataaaa
aagtgcctaaaacatcgatgcaggaacaaatctagcatgaatattaacatttcaaaccgcatgggtatat
gcatttctttacgagcaaattgtgcgaaatgccaatttgatacaaattcatgtaaggtatataccgaatg
tgaaaaaaagagcagaggaccagctgcaggctacctccatgaagcaatgacactagcagtattaaaaaca
aaaatgggaggatcagacttacaaatgacccttgcatgcctaaacatcagagtgccaagtctacagctga
tctcaaataatatcaataaacagtgtgataagataatacagctaaatgaggaggccatgatagaaaatca
gagatttgtcaaagaatttaatacctgtattggtaaagacaatgaggtagatgtggagacagatacagct
tataacaatagaaaccaagttgggtatgaagctggtactcagtctttctgcccacttattgaacaaaaca
ctggacttaatttacctatttctatgtcaacagctaacaaactatgcagcaggaaaaaaaattgtaatca
cagtgacaatcaaagctgtaagaaaaattacaatactgatgataccattgcttccagtgaaggaaaactt
gcagaaaaaaatttgcaaaaagttaatgatcaaaatattttaaaagttaaatctgtgaccagtgatgcaa
gcgctcaattgagtaaagttgtgagagaatttggtaaaaagtctggcaactccattcggtactatcaatg
ttttgtacataggatgcgcactgttcaaaaatatataaaaaatttaaatttttctaaattaccacctggt
tatgacaaagactttttttcggcaaagattagctacttgtattcgatctagggtaaggctcgagttagtgc
gcattcacaaaattagtacagagcatacttttggaactttagcacagtcagcaatcaaaaatattgtttc
ttgttttagtggaaatcatgcaaattgcaaagaaaaatctgaagcctgctgtgcacatttagacactttt
aatactaaatatttgccgtatggagtttatttaaatcttaacgtgatagacaaaaaaaagatagaatctg
cattaaataagtctctttctttagatgttttaaataaaatttctagtcttcacacaacaaaccaatgtga
gagcttgcatcaccgtgtctttacatatgctccaaaatcagtactgcataagagaaattttaatggttta
tgtcactctgcatgtcactcgtctacatttggaactggaaaatcttctatcttattggctaaatctttag
gacttaacttttcaaaatttgctccgttctttaaatttatgatacgaaaagacattatcgcactttatca
ttctcagagaaaaaaaacagcaaaatacaaaattcaaaaattcttggctaagtgtaaaaaacaaaacaga
agagttagacaagggtctatgtatagtggcggcagtactgatgtaaatgaaatccataactatggaatca
atgaaaacaattaaaatgtgaaaccaactgaactgttttattcttttcttacctcaaaatatacagtttt
taaaaccattatgtccctgacatgcacctaaaggtttacactggtttctttcaatatttatatcaaccgg
ttctaatgataaatgatcaatatttaaacatttttttttatggcataaatgtgaaacatggtgagtaata
ttagaaaatgtacaattatttgccaaaaagtattgaaggcggtgtacagtgaaattttgatgtttgcctc
taaacgatggccttataatgccatatccatctgcattggttgcaccataccaaatctgacagccattata
tatattaaatctaaaaagttttgactttaaatattgctgttcttcatctgaaaactcagtttcatacact
ttggacatgtcatgaaaaaaatgggaataaaactatatgtgactataaatctgaaaaacatagcaaattt
gcctatcaaattgaactcaagaaaaattggatgcttcacaactgtttgtgcataaaataaacaaagacta
acctgcaattgagcataatggttacaaaaatgacaattgatatgtacatgctgaaaataacctgcacctg
agctgtcttgataaattttaaacatgaaaattagaatatttgggcatatttctaatgccttaa
>KolobokP-1B_MyEd-I
ttaaccagtttgcatgtcttttaataacccatttgtgcatctagttgcaaacccagtgctatttcaagca
ttttggtgccttgaacttgtattttggtgtaaaaatggctgagatattgtcattttagtatgcaatgcat
caaaccgtctatgaggctataaaatgaaaaatctgaatattgaatctgagaagacattatttattgcatc
tcattcttccggtgaatgaaatagataaaatgatccacagagactttcaacttctcgaaaaggtcaatca
aaatgaattatggccatatattagcactgtatgtctaaaagggtgtatacataaagcaggattttaaaca
taataaaacataaaacaatgaaaaaaacacaccaatatcaaacaataaatgacagtcaaacctagtaaag
aaattcttatatcacatattgaaccatgaataatcaactttttttcttattttatcatcaacaaaataaa
aaatacacatctctatattatagagttcatttgtggtcaagggggagataatccattaatttcaaattt
gcaaaatcacacacaatgaagaaagtaaaataaaaagattaaacaatatttcatttacacttggcaattc
tataaaatttttctgaacatgtataaataaaataaatttgctattgtctattaacatcattgaaacaatc
ttgacgtcatagcgacatttgtgacgtcatcaatagaaaaaaaatcattagaaacaaacagttaaaattc
agagttcaaaatgcctggccaagatagaaggaaaaaaaaaaaaactatggagaaaaagaaataacttcaac
aaaggttacacaccttggaacaataaggtacagtttgaattgggtgttgaagcagattcttcaaatgctg
caaacatcaaagtcagtagacctaaaatagaaatttatgaagctgcactgacacagcataatggtgcagc
caatacattgccaacaagactgcggccccgtcgttcctctcataaggagaaaactgaagaacaggaggac
tgttctgaaaatattatagtaaatttttctaaattaaaggattttataaaaaagtgcttaaaacacaggt
gcaggaacaattcaaaagtagatgtggctattgcaaagcgcatgggattatgtatttctcttcaagccaa
ttgtgaaaaatgtaattttgacaccagagcctgtaaagtgtatactgaatgtgagaaaaaaagcagagga
ccagctgctggttacctccatgaggcaatgactcttgccatactaaaaaccaaaatgggagggacagact
tacaaatggctctagcttgtctcaacatcaaggtacctagtttacaattaatatcaacaaatgtcaacaa
acaatgtgacaaaatgatagaactgaatgaggcggcaatggtaaaaaatcagcaatttgttcaagaattt
aatcaatgcataggaaaagataatgaggtggatgtggagaccgacacggcctacaataacagaaatcaag
tcggatatgaggccggcacacagtccttttgtccactaattgaaaaaaatactggacttaatttactaat
gaccatgtcaactgctaacaaactttgcaacaagaaaaatgagtgttgtcatactgaatgtaagaaaact
tatagttctgaagaaaccatttcttcaagtgaaggcaaacttgccgaaaaaaatttacaaaaaatgaatg
atcttaaaattttaaaagtgaaatctgtgaccagtgatgcaagcgctcaattgagtaaagttgtgagaga
atttggtaaaaagtccggcaactccatccggtactatcaatgttttgtacatcgaatgcgcactgttcaa
aagtatataaaaaatttaactttttctaaattacaaactggttatgacagagactttttccggcaaagat
tggctacttgcattagatcaagggttaggctcgagttagtgcgcattcacaaaattagtacacaacttaa
ttttgcaactttagcacagtcagcaatcaaaaatattgtctcttgtttcagtggaaatcatgcaaattgc
aaagaaaattctgtcgcctgctgtgcacatttggacacttataatactaaattcttgccatatggagttt
atttgaatcttagtgtggtagacaaaaaaaaatagaatctgcgttaaataagtctctttctttggatgtt
ttaaataaaatttctagtcttcacacaacaaatcaatgtgagagcttgcatcaccgtgtcttcacatatg
ctccaaaatctgtactacataaaagaaattttcatggtttatgtcattctgcatgccactcctctacatt
tggaactggaaaatcttctatcttattggctaaatctcttggacttaatttttctaaatgtgctccgttc
tttaaatttatgattcgaaaagatataatcgctctttactattctcaaagaaaaaaaaaacagcaaaata
taaaattcaaaaatttttggcaaagtgtaagcgacagaacagaagagtaagactaggttcaatgtatagt
agtggctgcaatgatataaatgaaatccataactatggaatcaatgtaaataaataaaataattgaaacc
aactgaactgtgtttttttatatatctttcctcaccttaaaatatacagtttttaaacctttatgttctt
gacatgtaccaaagcgtttacactggtttctttcaatatttactataacaggttccaaggataaatgatc
aatatttaaacatttctttgtgtggcataaatgtgaaacatggtaactgatatcagaaaatttgcaatca

tttgccaaaaaatattgcaggcggtgtactgtgaaattttgtttttttcctcgaaaagttggtcttatta
tgccatatccatctgcgtttgatgtaccataccaaatatgacagccattaatattattaaaacggaaaag
tttagacttcaaatattgctgttcttcatctgcaaattcagtttcatacactttggacatgtcatgacac
aaatggtaataaaattatatgcgagtattattctgaaaaaaattataaaaagcatggtaaaattgccttt
ctcaatcgactccataacatgaaatacttcacaacagttaatgcataaaataaaccagagtaacttgcaa
ttgagcataaatggtcacaaaaaggacacatagtatgtgtaggctgaaaaaaaactgcacctgagctgtc
ttgaaacatcttgaacagaaaaattagatttttttgtgcatatttcagaggccttaa
>KolobokP-2_MyEd-I
tgtcagactttaacttttgttccagacttaaaactttacttttaaaataaagatatacatctcagtagtt
attctgaaaagttttaataaatttcatcgaacagttacagagttattgatccttatctataacgtctcat
ttctcctcaaatatcagaaaattggtcggataaaatgatatattcatgaagtaaatttgcttaaaatcag
cacaacatttttatttgaaaagatatttacattctgtaaattgcacatggtagataaattcattcattgt
atgaaaatttagtaaaaattaaattttaatgaatttttttttaattaaaatcatttttttaattaaaaaaa
taaaaaatccttatttttttttataggaattacaaaaggaaagaagttaaaagtgaaaaacttacaaaaaa
tgtatatctatgaaggcaaataagttccttatcatatatgaaaagcagtagtttacatggtaatgcaatt
agtggtaatccaataaaatgtaagccctactgtacacacacatgatctcttagaatacactgtccagcac
ctgtaaatcctcataaaagtgtcactgtcagcaattagtggcaaaattgtgtcaaggaagctgacagcat
cttccagtgatataaataatgcaagaaaatgtagaacatcataaattatcagtcaatggtactgtaaatt
accatgaaaggtggcaaaaatcgacgtaaatcttgcttcaaagcgggtaacagaccccataacaagggaa
taaagcatgaaacacagtcttcaaacagtcagccaagttcatctaatacacaatggaagaggctagatgc
agagaagttctcacttgtagctaaatccggtcaagacggcacctcctacagtttaccagatgtcgaggga
aatcctggatctgccaagttattacgtccacagccaaaatgtttgaaggttctgaaagaagaagctaaat
ctgagtctcatgatgaaacacatgttacagatgagtcatatagagttgttaagatgaataaagttactct
gctacttaatgtagttgttaaatttcacaatttacaagacattaaatgtgataacccaaactttgaactg
acaaagttaattaagtatggatcttgtgtaaagtgcatatataaatgtacagaatgcaagtttacatcac
catgtgttaacctgttcgatgaaattaaaactccaaaacgtggacctaaccctggagagttaacccgcat
gcttgtatctgccttacaggagactcctattggaataaaaagaggaagatttctgatggccgcaggacta
aatataccaccacccactaaaagaacacagcagaggcattctaattttgttgcgaatgagattaaagagt
taaatgacaatgatatgaagaaaaaacttgaaactgtgaaagaagttaacagaattcgtggtgtaaaaga
gccgtcacatattcctgtagcaatcgacacgcgttataacagtatgcatatagtaagcacaaaaaaaccc
ggacaaaatgcttcccaggcaatatcattggcatgtgagcaagtgactgaccataagttcattgttgcat
cagtttttcacaataaactttgctggacaggttcttggcttaaagggaagggacttaatgtaacctgtcc
agatggacatgctggtacatgtacagccaatgttaagccaaccaaccctttaagtgaatatctcatgggt
aaagaaattggacttcagattgataggcaaaatgtgctggtaaagtatgcagttacagatggagacggac
gtggagcagaaggtataaatgatgcactcaaagttttacacccgttgtggaaagttgaacgccaagctga
cccaattcatctcggaagatcacagtttcgacaaagtaattctgcaaattttagtttgaacatgttttat
ggttcgacacgtgaaaagaaaaaacaaggacagaaagttttaagtcaagatttgaaagccagatgtagct
tagttttcaatgaaatgtggaaagaaaactgtggaaatttggaaaagattaaaaaatgcttacctagagt
gcttgatgctactgtgcggtgttataccggcgactgctctaaatgtcccactagctcatacgtgtgtggt
ggtggtcacacaaactgttggtggctgagatccatgtacttgggtgcaaacaagttaaccaacctgaata
tggatgacaatgacaaattaataatgcaggaacttttaaaaatgaaacttagtgaggaagcattaatgaa
aatgaaatttaatttcaacactcaaaaatgtgaagccgttaacagagcactaagtgtatccttaccaaaa
aatgtgaacttttctagaaattttgaagggagggcatcagcaataattcacactttgaataatggtatag
ctaattcctggaaacaaaaagcagaatgtattggttttaaaatatcacaaggttcatttaaacacttaga
gcagatgcaaagggagtgtgaaaggaacaaagtataccaaaatactccagaaaggaaaaagaaaaattta
caacaaaccggacagaaaattaaggatcatgtggaagaaaaggaacaaagtaaatttaaagaccgatatc
aaaaaggacaacttgatcccaaacttaatacggcatttgttcaaagcacaaaggacgatcattcatatgg
gagagtaatgcctgctaagaagaatatacctaccagaagctgtacttcaaaaattccaaagtcaaagaag
acatagctgcatccatctacatctgaaaaagataaagacaaaattaaaggttcttttaaggaccaccaaa
tctttcaaaaagagtctaaaattgttgtacatttagtcaaaattctttatatataagctatagccctacc
ccttaaatatcttctaaaaataaatccaggcggcaatctggataattggcatggccctggcaagaattgc
ggtgcttacaataaatcctgttgttatttatgtgatgcggctctaaattaatgtgagccgcattcacaca
caaactattatgacacaaatgactggcatcaaatattttatttaactctaaataattgttataaaccatg
taactgaatctatgggctttttttctttttccaggtaccagaaataggatctagaaaagaaataagcccat
attttccatcttttgttgttccctgatcccataaaaagcattcatgatttcttgccaggtggctcttttc
tattatttttttgcctatatttttcaaaaaatccctccattttaacttacaagtgtatgaatttaaatttc
caacaaatgacctcactgtgacctctttttaaactttcaaatgaaaaaaaaactttttttaaaatttcata
aatttaaatttcaatttaatttcatccatgttactcgaaaaataagcttacagttgtagattgaagtgtt
caatatatgccttctgaca
>KolobokP-3_MyEd-I
ttaaggataaaatataatacacttttcagtaaaatccctatatgttgacaaagtttaccatatttcaagc
attttcgcctaaatttttccaagatcggatgaaaataaccaaagatacagccgtgcaaagttataatggt
atttaagaaaatcattaaaattttctatgaaaaagtgtcaaattttggattactggtgacaagtatgaaa
tgtgaaataaaagtcaattgataaaataaacacccttttgaggtatctggtaaccatgctaaatatatga
acacacttttgtaaacatttttcagctgaaaaaatatgttgctatgggcaacaaaacaaaagaaataaaa
aaatctacaaaaaaatcttcatgattttattataattcaactaataattattctaagattacattttcct
tctctttgagagttaattttatgttttggttcattcttaattgaagatttacatcattttcatcttatag
tgtactttgactggaatacattacactttaaagatttatggtcaaaggggagataagcctcaaaattcat
gaattttaacagttaatttttatgaaaatttcatgttgtagagatcatttaatttgcattggaatcagtg
ttaaaatgaaatttaatatcttttaaggtgtgttttgactttagactatatttaaatatctgcactttta
attatgacgtcatacaggttatgatgacgtcataaaggtaatcatgacgtcatgacgtcataaatactgg
ttagcaacatcaaagtataaaataactaactatcaactttgtaaaatggttaatactaaaaagtcagcat
gcaattggttattaaaaccaggatgcataccttggaacaaaagtatggatactgggcatgatggaacaac
atcaacagatggaactcttagtactgacacagactatactgccaagaataattctgccttaaaagtttct
ggtgtttcatgtgtttccttcaacaatctgtcactcttatcttcttctgattcttccgttgcttctgatt
tttctgattctcctaatcttcatcctaattctgagccaattaaattttccagaccggataaaactgtgta
tgatgaagcccttaaatcggaggctaaatgggctggctgtaaaccaggtgagatcacttttcttccgaca

aaactcagacccaaaaaaatggagaaccattttcatgatgaaactgaaattgatttagctgatgatgaaa
atgaaaacttaattgttagtgtcaataagctttcggcagctatttctgcatttctaccacattttcaggt
gagaaaaaaaatgcaagtcaccaaatccgacaattcaagttacagagagaaacggtatctgtgtttctata
acagtgtcgtgtagaaactgtacatttaagtcgtcacaaattgaaatgttcaaaagaattgtaaacaata
acgctcccggtccgaatcctggtgagcttaatgaagcccttgcaataccggtgatgaaaaccaaatgtgg
gccaggtgatgttgtatttctgctttcatgcctaaatattaggccaccatctcttagccttattaatcgt
aaagtaaataaaacgtgtgataaaatggtgtcattgaatgaaaactccatgatagaaaaccagcagtatg
ttaaaaaggtcaacaatttgcttggccacgacaacttcatagacgtcgaaacagatacgtcttacaataa
tagaccccaagcaggtggtgaatcagctacccagtcctttacaccattggttgagttgaacactacaaaa
aaattaacaattgcattggatgtaaaaaataaactatgtaaaaaaaggaaatgttgtcatgaaaatgatt
attgtaaaaaaaattatgctaccgaggagtctatttcttcaagtgaagctaaatctgcaaaaataaatct
tcaaaaggtaaattctggggtattctaaaataaattctgtcacctgtgatgctagcgctcaacttgca
aaaacaattcgcgaaacatctgaaaactttggttacaccatacggcactatacatgttttattcacaaga
tgcgcacaattcaaaaaaatgtaaaaaatttaaaattgactcgcactcctgcaaacactaacagagacat
atttttgcaacgattggctacttgcgttcgagcaagagtgcgattagaaatagtgcgcatcaagaaaaac
tttcctctcgaggacactttttgcgataaagcgatggcagcaatcgaaaatatagtttcatgttttagcg
gtaggcatacaaattgtaaaaaaaattcaatagtctgcatcgcacatttggctatgtacaatacaaattt
cttgccgtatgggagacatttagaattatgcacagaagacgagctgaaattaaaaaatgctatacaaaag
tctctatctttggaaaccatgcagaaaatttctcgtcttctgaatacaaataaatcagagagcctacatc
acagggttttcacttatgcaccaaaaaatacagtatggagcagaaatttttcggcactttgtcactctgc
agtacattcggcttctattggcacaggcagatctgctcttttgattgcaaagcaaacgggaattaaatat
actaaattaaatacattttttaggtacatggtaaaacgggatgacagtgaccgttatcaccttgaaaaga
aacagactccttggtacaaaaaaaatcgtttcataaacagaaaaagaaaattcatgcgcacacctttggc
aaatgaaattaacatttaataaactataacatagaaaatttatgtcttaccacatgttaatataaaatgc
atttcctatagcttccatgaccagtacattcaccattattttacaaatttttcgtgaatcatttatata
tttaggctcatatgataaatgttcaaatttaagacataactttgtatgacaaagatgtgaaacatgaaaa
gtttgacataaatcatgattaactttcatgaaaaacgacaaacggtgaacagagaaacgtttccttttcc
ctctaaaaacaattctaaaagtaccatatccatcagccgttacatttccacctgtccatatatgacagtt
tgattttacttcaaaattggttaatttttttttctattttcaaccgttcacgaactgttaacttacatgaa
tatatttctgacatactgatttaaagaagctatattaagtacagtttatattatatctaaaagaaattat
ggaatattattgtcaataaatttaaatatatagttcacatgctttcttttctttgtacggaattaaatgg
attgaccattgcattaaaaattgtattgctttcaccttcacatctgacgacgtcaaaaaacaccatggca
aagtcaagcaactatgagccttaaacagtcatttgaaaaggaaaataatggcttgttagacaaaatgtca
agaaaattaaaggatttcatatcacatacaatgtctttaactagattagccaatattcttgcccaaaatt
gagtattttccccttttatgatgtgtatattaacttaattcacattaccataa
>KolobokP-4_MyEd-I
tatagaatcaaaaaaagggcccagaatctagcaaattttaggtcaaatgaatacttgttatatctagtaa
tggaaaagttgtaggaaaaagatgtcgatcacttacaagcgaagaaaaaagaaaagttcaacatgtaccc
caaatgacggcagaggaaatgtgaagctgagagttgatttttggacaaataaaacttatttattttctt
attaaatatacaaaagcaataagtctgtgtgttttatcagtagactagtggataggactttcacataaaa
cactaacatcagctgtcagataaatggggtagattctacccaagaattgaaaactacaggaaaaaatgac
aatttcttaattttgaagtattaaaagtttaatatgaaacaacttaatgcactaaaaaaccataacctt
acaccaaacagataagacattgaccagagatgatgaatcaattatcttatcatatctgtcactgcagctg
taagaattaaaaatttatatctgtactgctatctctctctgtttatgagaagtttttatgagagttctgt
ttggtgcctttatttctaataaaagatgcaaattttaaccaaatcttcaatataaaagtaaagacagaat
accaaactttcacaaactttttctttaacagttacattcactgattaaaaataaattctacatataaaa
atgaccaaatttagaaagggaaataattcaggttttccaactggaactgtgccttacaatagacaaagaa
aaactgtaaaaccaagtaaaagtgacacaacacaaacagtttataaaagacttaccagaaaaatgacttc
tcttgtgcaaaacattccatataaggataccgatactcaatgtgtaaatgacaccaaacctgctgttctg
ttgcgaccaagggatactgctcctaaaattccaaaaacaaagaaatcagcaaagaaaaatcaaaggtagg
ttaaatgataattaaagtaataacataaaatatcaaaataccagttaatatgaacataggtattaattac
ctctattttaaatcatttttctaaaagcggattgagaatgcctatcagttcattaaatgtacaaatttct
cttgaaataatcctatgttgttggtgtaccttcttgaatttataaaatatatgcttaacttcaagaaaat
aatgacactttaacttgcttagcttcataataccaagaaattccattttatatttgtgccaaaaaaaaat
tgtgaccaaagggaggtaattcacaaatattttgaagtacatttagaaacttctgttacaggtagaaaca
cactctcttatggcatttttttctattaaattttaggtctgaagattcatctaatagaacagttaatgtg
agactgaatgcaataatgtggaactctgtatttagagaacaccaaagacttcatcccaactgtaatggat
ttttacagtggaatatggaacgagaagaaaagtttggatttgttaatcgtgaggaggcaatgtgtgataa
atgcaactatcaatcaagaaagttcaaactgtacgaagaagtccaaaccaaaaaaccaggaaggaaggca
gccaagataaatgtatcagctcaagcagcattaagtcaaacaccacttggatatactggtttgaggaaaa
tagtacttggctgtaacatgccagctccatccgcttcaggacttcaaaagagggcaaataaagttctacc
tgaaataataaatataaataaggaagacatgaaagcgcgacgcaaacagctgattgaaattaacacactc
agagggagaaaagatcctggttctgtaagtctacaggctgacggtgcatacaacaatgcaatatattccg
gcattggaaaaaccccattccagcctgctacacaagtggtgtactctgttgcagaagctgaaacagaaga
taaaagtataataggagtggtttgcaaaaataaactttgctcaattcatccaatcaagtctggtgaaaag
tgtacatcttcatgtagttcaaatttaacatttatgaaaagcataggagatgagtacacctgggcaaaag
aagctcttcaagatcttgcaagtgatggcattgaggctaaacatttaacaacagatccagatagctctgc
ttatagagctgcagatgacttgtatcttgaaaatacaacatctacagaaccagaacactttctagatacc
cggcatttttttaaataatcacagaaaaaacatcaagaataataaagaattgggagaaataatgcctggta
gaactaaaaaagaccgtgaaaagcttttgaacaattttgctttggatttagctgagagatgtcagtcgga
gtttacccaggccatggaaaaatatggtggtgactttaccaaagtgaagaataaaatctctttcaccgtt
gatgccatacccgcatgttacactggcaatcatgaactttgtcgtagacattcctttgtttgcaagggtg
gaaaaaagttctggttgtccaacagagctttcttaccaaatagtttcaaaatcagaaaattggatgaaaa
tttgaatgccataagaaagtgtgtgttatatcgtctcagtccgagcgctttaaaaaagacgagattgaac
ctaaatactcaaaaagttgagggatttaacagatcactgcgtagatcactaccaaaaaatgttacctata
caaaaaactttgaaggtcgtgtccattcagctattcatagtgtaaatcttggtcctggtgagtctctcct
agtgatctgtaagcaacttggagcagaaatcagtcctgggtctgctgctgaaaaagaacttaaagctata

caaaaaacagacagaatgcagaaagcttataagaaatcaatgaaatataaaaaggagaggtctaatacta
ggaaacatttgtataaaatttatgaaaaacatcaggaagaaaaatgttatgagaaaaacaagcttatgcg
gccagtaaaacgtaccaaaacgcaagccgaggaccatccctacgccaaaaaaccacgagttgcagtgaca
aagaagtgttaatttgtaaataaagcattcttttatataatacagtgtggttcatgacattttgcacata
aattttgccctttacaatgaatacgttcctggtttgtagcatggggctcaagtttaatatgctctgaatt
gacacaggtcttattatggcacaagtgactacattctatgcgattgttgttttcatcaagtcttggcata
tcattgtggcttattctttccttaatcatatatgctagtctatgtgctctttcttttttgcaccctccc
ttggccagtttacattcatgacaccgtatcccaatttatcaacatagccgtcccatgctacacaaccatg
aatattgcccaatttttttgttttcttaagaaatctctcctctaaatctttataaaagtcctccatgatg
ctgacacaatcagaaaaacgttttaataaaagtctgattcagcaacttgcttttctaaaatgaaagtgaa
agtaaatttcatcccaatata
>KolobokP-5_MyEd-I
tatactgtactttcaaacccctttagtatcaaaatattcttatattcttgttgttatttatgtaagtaa
tagaaaataataagttacaatggtagtttttttcattattgtatttttatagtacttctagtaactatacc
ctgtttgcggcaagatgtgtttttttcctgttcactatttttttttttttattcaaattattattttttt
tgtctacttaataaactcattatatatttgtgtttttcttgtgcctttgtgtaatagctatttgaatcat
ggttagttttactacaatctgtttattttgtagaatttttgtcttctttatgtactataccataaaatgc
ataaaaagttgtcatacaagcttcaaaaaactatccctataacaaaaacatacaccttatctttactttc
tgtttatgttatagcacacaagcctgtcagcactgataaaaaaattaaatgaagagaggtcaaattatat
gaacttgtcaaattacaaaataatgagattagccatcatgatgatttttatatgatttatataaaaaatc
ttacatctggggtcaccttcagttccatcagatataaacaaaaaacttagtcattcatgattgtttagtt
gtaaggtcctatatttgatgtttaaatattaaaatactggaataaagtgcatttcaaacaattcttacaa
taacaatgggtctaatcccatggcgtctaaggaaatacacatttcagaaaggaaacaagccatataataa
ggggaaaaaatacagaaataagactttacctgctgaaaaaggcaaatttgtgagactaccaaaagacttg
tatgacttagtgactaaatctgttcataaaaagaatgttcaactaattcctgcctctacaaagtcagctc
gctttctaaggccgaagcctccgacaaaaacagaagttgaaaaatgtgctgaaaatgaaacaaaaacagg
gtaagaatactttttttgttattaagctttcaaaaataattaaacttgcttaaaaaagccatttataaac
aactatgaagaaaaaggcatgtgtcttatatgccaattctttaaatgtgacatctgaagaaaatgcaagc
taggattatacttattttaaaatttctagtcctatatatattttcaagagctaaatagtgcatggttttt
atatataatatatataaaaacacaggcaaagaaaaaaatatgcacttttgagagtacatttgatcaaaaa
gcttatttaaagaaaacactattgtaagggaggtaattcaaaatagaattattttcacattttccaaact
tagtttaacaaataaaaaactaattcacttcaattttggcaacatttttcaacttcaggtcagagaaaaa
tagttttcgactccttcggatcaatgaacttgaacagatgtggaatgaggttttcatagaacacagacaa
atatcccctatgtgtacagggttcatatcatgggatttaagtgcagagcagcaaagaggggctgcctgga
gggaaaaggcaagctgcaatgagtgttcttaccattcaaaaatgttcaacctttacaatgaggtgatcgc
taagaaacgtggtcgtcgaacagcagctattaatctatccatacaagtcgcacttaatcacatagctatc
agcacaactggtctacagaaattatttcttggttctaatataccagctccttccacttcaagtatgcaac
acagtgctaatgttgtttctgaaattatagaagagtacaatcaaaaggatttggttcagaaaagaaaatt
aattaaggaaataaacatattaagaggtgataatcctaatattattaacattcaggccgacggaatgtat
aataaccctatttattccggaatgggtaaaacaccatttcaaccagctacccaatgtacatataacatgt
tagaaaataatacttataagcacagtattgtaagcacaagacaagtgtctaagctttgctcaaataagag
tgaacataaaacaaaaacaaaagttaaatctcacaaaggtcactgttccgcaaacattgaaatgcatgac
agcataggcagtgaggagcgatgggctaaagactgcttgttagacctgaaaaatgatggtctggaagttc
aagaaattaccactgaccctgacagcagtgcatttagagctgcagagtctcttttttaaagctggtacaac
caatactcagccaattcattttctagatacaagacatgtgtccgcaaatcatagaaattttattaaaaaa
atgtcaggtctgaaggaaataatgccggcacgattagtttcagagaaagataaaatgctaaaaaggtttg
cattagatatggcagcacgttgtcaggcagagtacaatgctgcctttgataaattcaatatggactctgt
acgggtaaaagttcacctatcatatgtatgtcatgctatagtttcttgctatcagggtgatcataacggg
tgtacaaaatattcccttgtttgttcaaataggaacagtttgaagacatggactgaaaaatcagcttatc
tgaaaaataactttaaattaaataaatcagagaacacagcagcattgttacgtgaatgcgtaaaatatcg
ccttggacctaccatgctaaaccgtacatgcaaaaacactaacacacaaaaagctgaagcaactaaccgt
gcaatacgtgcaactgtacctagcaatgtaacatttaccagaaactataaaggaagggtacatacagcta
tacacaatgtaaataatggccctggagaatccatcgttaaactctgtaaagcggctggggttcccatcga
accaggaagtcgtgctgcccgaggtctgaaaaatattcaacaacacaatgaaaaacataaattatataag
cagtcaaagcagtacacagatcagagatgctcaaaaaagcatgaactatataaaatttatgatgagtatc
aagaggaaaaagactatgagaaaaacaaactttaccgacaaaacggctggccgcaaaacagcctcaaga
gtattcgtttgccaaaaaactccgatcaagacatgttaaaaataaataaattacctgcatcagataagac
aacacggtaaatgattatttgtacactgttttgtccgtctacagaaaattctctctatgtttatactgtg
cgactctaaaatcaggtgctccggtttaatacatttggggttatggcaaaggtgtgaaatatctaaaaga
tcaccgtttggagagaaacggggaagattttcttttaatattcttttatctagcatataagataatctgt
gagctctttcaagagttctagtgccatcaggccaattcataactttttttcccatagaccccatgctccaa
agctccaacccacactgtacagtcataatttccataattttttttcactttttcttagacaaagctaaagcc
aatgcattccaaaatgattccatgttgtttctgtaaaatgaaaacccaaaaggggtccgaaattgggatc
ccakggaggctgaggggtgaattcccattatttaccttatataa
>KolobokP-6_MyEd-I
tattagacccttaatcttcaattaaatgccaaaaaaattagcatgcattaagtttattctatatcgaacc
agcatacaaaatttcaaatcaatccgacaagttttatggtagtcctgattcttttaataacactaccctc
atgttaataattacaggaattttatggcatgaaatttacaaaacatgtatctactaggaaaagttaaagt
ttaataactttcttgttaaacaagatattttaaaaaagtaaggtgtctgtagcagctaaatggctaaatt
tcaagaatttgaaagaaaatttataaaaaatcatgtttttttgaaataaaaattgggtttttttcaaaaatt
cttgtattttttagctaatataccaagattaaatataaataaaattcctttaaaaatacttaaatacaaaa
ctcactttcaaaacttatcttaaaaataagcttatcttatctttaataagtaaaatataactcaaacaat
aattagacaaaattcacaggtacttgacctgtttacctgaggtaccaatattcatgtgaaacaccaggtc
atgttacatgtaataaaaatttcaaactagtgtaatttctttattccttgaaatatcacattcaaataaa
ctttttagtgaagtttaactatttgtttatttttatcaataaagagatttaaatattataaaagatttct
gtaagaataaatatttatttaaaaaatatataagmcacatgaccaaatttgacaacaaaaattatttaaa
aaattaattgaaaacatccaatatgctttcttgttaaattgattactcaataaattattaagaaacatat

tttcatagattttgatcaaacaatcttaatctggacccctgaaaatgaaataattggctcaactggatac
tgttaagaaaacactgttaatttagagttgcctcagaaggtggtctctgtaacaccataccacatattgc
atcaaagaacaaatatgtacatcaaagcagatattgtacaccattttattataaatatcagggtgtccaa
attttatgccattagtccaagttgggatataactgatccaatttaatttttcaaaatgttcactaaaggt
cataccccatattccaaaggcaagaaatggtcaaagaaagacaactctccaagaaatcctggatgcttcc
aagcaggcaaacccagtggtagaagcagaagccagacgtcaatacagatccctgaatcagctagtgcaag
tacttcctatgtgcggcaaacttttaaatcttctgactgcaaaatggagacgactggtaacatctgtaca
cttcgaccgctcgataatgctgacctgaaaagagaagaaaataaagaattacattctggcatgaggtgga
tagacagtcaactaaatataagcttgatcaacactaatattaataagcaccaagtggactcaccatcctg
tcctatgtttattgctgactattatgaacagaaaaagtggggaacttgctggcagtttaccttaaaatgc
atagcctgtggttttattggagacagaatgaagatgtacaaggaaatagctaatggcaaaccaggaccca
acccaggacaaccaaatgtagccctagcatctgctttgcaggactgtccaattggaaatacaacagtgca
gcaactacttgcaggaatagacacaccaccaccatgtagaagcagtatgcaacgcacatccagcagagta
gcagctgaaatggtcaagctgaacaaaacagacatggcacagaaactggaacttgtaaaagaagtgaaca
ggaggcgaggtgtcccagaaaatgaaattaacataactgtggatgcacgatacaacagcaacacaatagt
tagtaaaaagaagcctggtcagaatgcgactcaagtttttgcattgggaatagaaacaataacagataga
aagtttattgtagctgctgttgtacagaataagatgtgttggaaaggggcatggcttagagggaaaggct
ttccaatagaatgtcctggtcatgaagaatgtacagcaaatttatacagagctgctgccctgtcagagta
tgagctgggaaaagagatgggaaaccagataggactccagaaatgtctggtacggtatgtgaccacagat
ggagatggacgttcagctaaaggtattgaagaagcccttgcagctgtggaaccaatgtggaaagtagaaa
gactggcagaccatgtacatttgggacagtctcaattcagggcctctttgcgagctcaatacagcccggg
aatgttccatggtaaaaccaaagaggaaaataaacaacttaagacggtatttagcaaagatctaaagtgc
agatgctccatgatactaaagaagctgatggagaaatatgacaaaaacattaatgaaataagcaaagact
taccaaaagtgctcgatgctaccctccgctgctatgatggtgattgcactcagtgtcctgaatattccat
tgtctgcaaaggtgatgatgcactcaactggtggagtcgttcacattacttgtcaacataccagatcact
gccctgcaaatggataaaacagataaatttttacttcaagaaatcctgaaaatgaaattaactacagcag
ctgttgaaagcatgaaactttatgacaataccaacaaaaatgaaggagttcatagagcactgagtgtaaa
tttgcccaaaaatgttatttactccaaaaatatggaaggacgacttgcttcaggagtacatagaaacaac
aacatgccgggaacctcaacacaacttaaatgtacgaatctaggagtagaactttcagaacgaagtaaac
gttacctcaagaaaatggacactgactttacatataaccaagattatatactaagaccagaagttcaaaa
aaggagattacagcagaatgcagaaaggcttgcagagcacaagacatggagagaactgaataaaaagcat
gactattactgcaaaggtcaactagatccacagcctgccttacagttccaggaccatgacagctactgca
aaccaaaacttccaagacctgctgcagactgagataccagaaccaccagccaacagaatcataaaaggtc
cttttaagggccacccaaatattaaaaaaagaatctaaagttgttgtacagtagtttccaaatttctttt
cctttttcctcccattctgataacaaaatatattttttttgcccgccccagaaaaaaaaagaaggaaat
taaaaatatagaagtacccgataatcattttacccaaattattattgtccattttcaactccactctgca
tggagggaggggatctgggtggcccaagcaggaactggttgaaacgcatgactgccggctgttgttgaaa
ccatgaggttccaacgacagatgttcgacagtaatgcatttcgaattgtggcacaggtgcgaaacatcat
cagttttatcaataatgatattctgatgatgcaccatgtaggcaagcctgtgcacatgcatttgtctcca
ccccctggcatctggaaatttacaattaataactccatatttgccattaggtgtacataaacctgtccaa
atttgacactgcccattatagccaggggcagagctttctgttaatttctgactatatttcaaataaaatg
catccattttgaagatgtgatgttttgtaagacaatagagtagtaatatattattaaaatataaaaaggg
tccaacattattgacagaaagggctgaaactggggtcaaagggcttttagtgcatcaaaatggccaaaaa
tgccttaaattgcataatattgccagtttaaaaattaaaattgattaaaattacaataaaacaataatat
ttatcatttgaagtgactttctgccaaaaaagttggccctttcttcattttcaagaatttgggtatcagc
ctctta
>KolobokP-7_MyEd-I
taataggtactttctagtggtccaaatgggaaaaaaattagtttcattaactttaacatatatcaagctt
gcataccaaaaatcagaccaatccgacaatacttgcataagcattgacatttcttaaacacctaccctca
tacccaaatttctacagttttcttagcatgaatttgtcatttccctaaagaccccccactttatcaaaaga
ttcataactttttttattagtgcaggtatctttaaaaattaaagtgattctgaaaggtgagtccttatata
aatttaatagaaaataaaaatttctaaaatctgaacttttcaaagtgtaattgaagttaaattttcattg
tttttaaatcagtgaaatcccaactgaaaatactttcaaaattcaattaaaatttcaaatttcatccct
gaattgtgagaattgcattaaaaaattccaaattcattctagaataaacaaaatgaaactttatggggt
attgaataacctttaggctaaattacctgtatattcaacacccccttacatcatctgcccaagactaaa
tatgaatgtaataaaaaagttaaaatgatataaaatccttatttctttgaatattgagatgaaataaagt
gttttggaaagggtaagaagtgaggaaataattgcaattgaaaaatttaaccaaaagtacaggttttttt
ttcattgattttttttttaatttgaaaaaaattcaacaataaattcaactaaaattcacaaaatcaaatt
ttctgaaccaaataagttttaacatttgcctaaattaatgttcttttgaaatctaaacaaggggggatttt
tttttttgtaaaaaaggccaaacaaaggcattttctttgaagcagacaaaaccctttgaagttaaaaatc
actttctttcattacacagctaaaaatagatcaaaacaataaacaaaaatgattgacagcatcattatgc
caatcaataatcagcatcaaagttacataatgcaaaaaagtataaaaactaacaattttaaggtctgtca
tcacagttccattcgacgctttacactgttccagaatattctatttatcatcaaaatgtttccaaagggt
tcccgtccattcaacaagggccttattggactgcagagagggagagttgcaccaggttacaagtatcagc
ataacagaacaccatggaataaagtccctgtctcacagctgtccacatcactagatcctgaagaaaacca
tgttcctgctgttgaaatgaatgttgaaccaccgaatgctatccttgatgaatccccatgcacttcttct
acatcacctgcagacaatgacagaaaacagactcgttcccagctactggttccatcagctgacctgaaaa
cagaagaagaatggtgttcaacatctggtatgaggtttgttgaccatgaagagatgatgaagatgctcaa
tacagttataactatccatacaaagaagtttaggcactgtaaaaatcctgaaatactggcacacaaacag
gaaaaatggggagtctgttggaaatatactcttagatgcaagaactgtacctacatgagtccacttatga
agctttataaggagattgttacaactaaacctggtaccaatcctggagcacccaatgtagcagtagcagc
agccatacaagactgtccaatggggagtactaaactccaggagctacttgccagaatgaactgtcctcct
ccttcaagaactagcatgcacagaatgacccacaatgttggaaaagagctggtacaacttaatactagag
atatggcagaaaaactggagattgtaaaggcaaaaaatcaagaaaggggacttgcagagaatgaaatcaa
tgtcacagtcgacggaaggtacaacagtcagacaataaccagtcgaaagaaaccaggactgaatgctacc
caaggttttgcacttgccatggagacaacaacagaaaataaatatattgttgcatcatttgcccagaatc
agatgtgctggaaaggggcctggttgagaggaaaaggatttaaagtggaatgtcccaatggtcatgaaga

ctgcactgcaaacctacacagagcagctccattgtcagagtatgagatgggaaaggaaattggtactcag
cttgctcttcaagatatcctggtgaagaacgcccacaactgatggagacggacgggcagctaaaggaatag
acgatgcaactagagccctgcatcctatgtggaaggtggaacgactagccgactatgtacaccttggaca
gtcacagtttcgggcatcactgagggctaagttcagtgaatctatgttctatgggcgtacgaaagagata
aggaaggaaatgaagaaagcgttcagccaagatgtgaagtgtaggagctcaatgataattggacagctaa
tggagaaacacaacaaagtaacagaagatgtgtgtaaagacttacccaaagtcctggaggctacactcag
atgttatgatggtgactgctccatgtgtaaacagtactctgtagtttgtaccggtgacgagggttataac
tggtggactcggtccaagtatctaggatgctacaacatcactgtcctgcaaatggatgaaaaagacaaat
tattattacaagaaattttgaagatgaagctaagtgaacaggcattaaacagcatgaagttatatgacac
caccaataagaatgaaggtgttcatagagccctcagtgtcaacttaccaaagaacgttattcattcaaga
ggcatgcaagcaagattggcatccggaatacaccgtaacaacaacaagccaggaacatcagccaaaatga
agtgtgaacaccttggggttaatttgtcggagagcagtttacagtttttgagcaaaatggacagcgacta
tacttacaaacaagaatatgaaaaaaaaccagaagttaaacgtagacggctaattcagactggtgaaaaa
atattggaacacagagaagctaagcaaagccgaaagaagtgtgatgtttacagaaaaggacagttagacc
cggttcctgcattgtgtaaaagggcacctccacctgcaccttagacgacacccacccagccagcagcaca
tgatattaaccaaaggtcctttaaggaccaaccatatctttcaaaaagaagtctgaaattgttgtctaa
ataaataaataaataaataaataaataaataatagatcttaaaaatataaataataccaatattattgtt
tgccatcttcaaatgtaccatgcaaggtggcaaagggtctgggtgaccaagacaatgaccacatgaaata
catgattttctgctattgttgaagccgtgtggctctaacacaagatgttcaacattaatacattttgtgt
tgtggcacaggtgcgagacatcaaaattaggtagatgaaaattaagatgcctgatcatgaatgccagcct
gtgcacatgcatttgtttccatcccccggcattgggaagcttgcagtttatcaccccatatttkccattg
ggcgtacaagccccagtccaaattagacagtgtccattttggccaggggctgaattttctgacaattttc
ctgaatatttttgaattattccctccatttaactttacagaactatttttacatttgtatgtgtagtwat
ttgccccaaaaatagaaaaaatgatgagcccctgtcagcaaaattgaataaaagggtcaacaggtaaaga
aatttcaaccagatggcccaaaattggcttttctttgcaattwaaaattatttcttcatgttttctataa
attaacactaaactatctttgtatttcatgtttgagacaaattgtgcaaatcagggggccacaaagtcca
ttttcagaaaaaaagtgtgctctgcctctta
>KolobokP-8_MyEd-I
ctataagagacaattcttcctcaaatcatactttttttatattaattatgtaatttagcctattctaaag
ttccagattcaatacaagatctgtagtggctaaattgtcaaagtttaaggaaaatttgtactatagccca
tattgcggccaatgtatattttgtacaaaaagttgtaaattgaatgttgtataaaaaaatgatgtaaaata
tattctttaataaatatgaactgttttttgtgtttgtttgaacaaatagtaccagttaatcttagaaaca
tcagtcatattatattctgagattaatatctccatatatggaagttttaatgagtataccatgaaaatac
cacaaaaaagttgtttccaagtcttttaaatgagtgtcatacaaaaaaatcaacattaatatttccctaca
ctaaatctgtgttttcatatcttgtatagatcatttaaagtgcacacagataagctatagggtcattctc
tatcaaagatgagaatgtcttatcatactggtagagtcagagaaaaaacagacttttcttcaactgtcat
caaccataaaaattatatcttagcaataaaatttgtcctgtttctgcactttccttcttcttactttgt
taaatgaaatcagagagtgacaaatattgttataagaataattatttcataaaaatttttaactaaaaaat
gggacgttatgctgtacacttaaaaaaacacagatttaagaagggaaatattccacataacagaggactt
aaattgaaaaattctaaaatacctgtattccctgtgattaaatttgaaagactatccaaggatgactttg
atcttgtgacaaaatctgttaatatcaaacactatgctgaaattcctatgcacccaacatcagggcggct
tctgcgtccaaaacaggcatccaagactgaagtacaaaaatgtgcagaaaatgaaaataagactgggtaa
gatcatatattgcattacaattgataaagcaactatttacagtatttactagaaaatttcaaacatcaca
ttagagaaacaaacattttatcaacagtttatagatgcagggtatgtaatactgaaaaagttatacagaa
tatttttgaattgaagaaacgcattgaatgatgaaataaatatattatttctacataccaaataattatt
cgaatgtaaaaatacttgaaaagtgcttatttacaacattatctgcatcacagccttaataacactgctc
tgaaaaatactggctcaaagggagataattctaatttaaatcttgatactacataatataaaaaagacaa
ataagctacatttgctacacagtgatgcaagatagggagttaattaatgtctttcattaatttttttacct
ccagatctgaaaaatacaccaacagaacagtaacaataagcaaaaacacagaaatgtggaatgaggtatt
cagggaacatcagaatctatccccacactgtaatggcaaccttgaatgggacttgtcaatggaagagaag
tggggatcagcttggagagagtgtgcaaaatgtacacaatgcgcatacaaatcaaaaatgtttaacttgt
acgaagaggttgccagtgtaaaaaggggacgacgcgcagcaaaaataaatcttggattacaagttggtct
acaccatactccaattagtacagcaagttaccgtaaaatctgtatggcgtcaaatattcctcctccatct
gtatctggtatgcaacacactgctaatgcaatatctgaaaaaagatagaagaagaaaacatgagagattt
acaacgacaaagggagaaaattaaaaggataaaaaagatacgggggggaaaaatcctgatgttgttaacat
tcaatctgactgtgtatacaataatgctatttattctggaattgggaagaccccttttcaaccagcaacc
cagtgcgcgtatacagttgctgaaaatgaaacttataaacacagcattataaatacattaccaaagtcta
aactttgttccaagagaaagcatacagttagtacaaatgtaggcgaacatgctggtccgtgcacggcaaa
tatcaacatgcaaagacagtataggaaatgaagagcggtgggcaagagagtgttttgaggagctaaaaga
ggatggattagaaataaatgaagttacaactgacccagacagcagtgcgtacagggcggcagagtgttta
tatcaggagggactaacatcaacaaccccccatccattttcttgacactagacatgtagcatctaaccata
gaaaatttgtaaataatttatccacagtttgtgacatcatgccagctcggttaaaggtgcaaagaacccg
actacaaagtttgtttgcttctgacatggcagcccgctgtcaggcagaattcgtacaggctaatattaga
tatcacaaatccccttgatgtaatgaaatccaaactttcatatgtttcagatgccatagtgggctgttac
tgtggagatcacacagattgttccctatattcatttgtttgttctatatctagaaaatctcaatcatgga
ttgacaaaagtgcatatttaaagaggcataattttgaaattgagctaaatgaaaacagtgaaaatattct
gcgccaatgtgtcaattaccgtctgggtccaggcattgcttgctaaaacagctaaaagtgcaaatacaca
aaaagttgaagccctaaatcgatccatacgtagcactgtaccagttaatgttacctatgctagaaacttt
accggccgggtacatacggcatgtcacaaagtcaatcatggtacaggcaattctatcgtcatcttgtgtg
aggctgctggaagtccaatacaaccaggtacaaaagtggcaaaatctctaaaaaaaaattagaggaccata
gtgatcaaatgagaaattgtaaatactcagaaaacagtatcaatcacagaaatgcaagaaagcatgctct
gtatgatatacatgcacggcatcaggaggaaaaagattatacgaaaaataaactttttaccggtcactaaa
aacagtcctatgtacccaaaagaccactcatatggcagactccgcaaggctctactaggctttcaaataa
ataatattcaccttaatctataataaacaatgtggatggtgatcctttgtacataaatttctcattctac
accccattctatccatattgagagagtgtggctcaagaattaaatgttccggcttaagacagatggggtt
atggcatatgtgcgagacatctaaccgctccccctgtgaattttttatttggcatttcatccctcaaaata
tgatgtgcatgcatgtaggagagcctgtgaaccctttctatttttttctttccatcaggccacacaactg

acttcttcccatagaccccatgctctctaccaccagtccactcagtacaccccatgccgaccgcaattaa
aaatagacttttgcattaaaagatagtgctatttgattccaaaaggcttccattgcattaatatttacaa
gaaaagaactttggggttctcaggtgtccttgaaaaagtacagtgacagggtgcaaattctccctata
>KolobokP-H1_MyEd-I
atattagatcagctaatgtcagattccacttattttgcagtttgtatcccttcagtacacatttctgaac
aattctgtgaagttttagccatatctgaaataaaataaggtcattttaatacttttaggtagcaatgttt
ataactgtttaatatcattttaagaaagaaaaaaacacaataagcatatgttataaattttcattttaa
acatttgttttaactttttttggtataactgcaattcataagcctcttttgccttacagactttctacaa
atcatctttttttgttgataaatcatgaaaatatagtgtttttcacttaaaaactatcagatttaagaaa
tttgtccaccctgagagtcttgttttctattatattttttttttattgaataaaaagctcttatatctttg
cattactgtaaagattagtcatagacctttccaaaactagtcttgtttttaaaatccatcaagtaataag
aattttagaggttttctttgtatgtgtcatgaaagtgggaaaaatgggttattttttttggtgaagagtac
attgtgacgtcatggtttttgtgacgtcattgcattatgacatcataatttagagttgtacacacagcat
catacaatgcaaatttagacacatgtatatattgcataaaaatttgtgtttcctatactgcacacattag
ttgaaactgaacatacaaggaaataaaataaaataaaataaaataaaatcatgtccatatttacttccat
tctgttttgcaactattttagttctgtaggctttttcttttactttttgatatcctaattttataatttt
gtagtttttgtttgatttgtttggtttttatattgaggatttcttttcactcgtgcttacttcagttggt
tcatgtggtttgattgtcccaatttcctgacacaggtaaaaatacatttgtaaacaacaagagttactcc
cccttctatgtaaagtaaaatgtaaacactcgtgaaaccagccgtaaatgttttacctcaggttattaaa
catgcatgtttgtttcagtgtcttggagggtaagagggagggcttttatcccattttcatttttttgagc
agtgtaagaaacttgtatttgagtaaatgattcgtgcataatgaaatgtgcagttgtcacctcggggaga
cttaaattttagaattaaaaatataacaaaccacagtttaagatgcagcatgcagttaggatatgttgtg
ccatgcagttcatgagtggtgcatttttgtgaagctacacagaccttacggtggttatttaaatcatggg
gctcaaaaattatatggtcagcattaacacacaaggcattatgacagagatgggaggcatctaaatttct
atcaatatcaattgtttttagctgtaacatttttgacaagcgtgcaacattcatttttgtcctttttcca
tttggataagccacatttagcatccctaacatataatttcttttcctctgaatggcccctgtccagattt
tacacgacccatttctgcatcaatgcaatttaacaacagaatattggtatatttttgataaaaatcaaa
agtttgagaagccattttgtattgttttgattaattagcactaaaagagtgtaactgaccttgatttcag
cagctttcatcctaatattgtagtcactgagccatgaaacactccatcacctttgaaattaaaggtcaca
agat
>KolobokP-N1_MyEd-I
ttaatagtaaatatttttttcaaattctcccttttaacttattttctgagttctgctagctaatacgagt
aaaatggccttttatttttgaaaattggttgagtaatgaatattttatgaaggttagcagttgacactga
aacgcgacaaaaactgattttaaagaaaggtgccaagtcaaagtgtaaatcttgtctctacctcaatttt
tagccaaatcttaaaaactgtacctcttttgtcagtttttttaatgttgaatatcttaataagatctctga
tgatgcaacattgtataaaatttagcaatttcaagcataaaaatgttaatctttatgcttattgcatacc
aaagacttgattaaaaaacttggtgatagggaatcaatttcttacatctgatttaactatacatttaata
tatgccaaaatgtaacatgaaatcttgataaaaacaataatttgatatattcgcaagaaaaaaaccaacg
cgttcaatacttaggctactacatgcagcccaatccatcagaagcaagagttaagccgatagagtacaaa
tataagccttgtgtcaaaatgtctaattttggttgctaaggactgtaaacaataaaattgacatatattg
tcattcttaaacacttaatttactcagaaattgattaagaatctaaatatcaatagatttgtggcatgaa
aagtcttaaattatacaattctgagcttggtaagtatgccataa
>KolobokP-1_MoPh-I
tattagatgtctaatttttcaaataatgcagaaaaaattgacatcagtaagaaaaatgtattacaaagaa
tgctaccaagtttcaaaacaattggacacataataaacaagttacagcaactttcatatgataacccata
ctgtctgaaaacttatctgtgcaaagcaatacagagaaatttctggtatagacttcaatcttaaaaagtg
aagaactttcttatttcttggagtttttacatgaaataaaatttaccatgcttcagagagaagcagctat
tttttggagtgaagaaatcacataaacatttaatagatttgctttaaaaaataaaaaatgttaaaaacatt
aaaacaatacaagttttatttttcaaaaatcaaataaaattttaaatttacttattttgtgaaataattt
cttcacaaaaatccattcatgacctggcactataataaaagtggaaataatgtgctttcataaacttgat
tgaaatacagagtcttctttaaagtactacctattcaaacaatttccatgacttttcattcactttacaa
gcaagtttccaaactggaatttattatttcagaataaagtatggattttaaatatttgaatataaaaagt
cttatgtattttcaatttttttagcataaaaagtaaattatagaaactttttgtttggaatttgttcagca
atttaaaaaaataaacttttttaaaaaagatattaactttttaaattaaaataaaaattcaaacaaaatct
gtatcgcaaactctttattttaaaaaattgcagtaatatgtaacacataaaatattagtagcagatacaa
aaatgagatttggtaaacaacataaatctcctcaataacagaccaccccctgcacagattgcagttaagt
acattcaactgaggtcatttgactgtctaattaatccaaattaaagcatcaatcatcaccttaagtaaat
tacataagaagtctataaaatgaagtgtatctttacccaactctttcattctaaatcattattgctttga
aataataaaagaaaaatcatgtttaaaaagggaggaactccatggaataaaggccgcactggattgatca
gcatgaaaattcctaaaaagtcattgtttgtaaaaaattccacaccatggaataaaggaactgaagtacc
ttgggacaaatccgagccagaaaattcataccagcgtatggaatccaaagagtatgaactagttgtcaag
ccaaaaatggatggaaaactactgtcaacacctgactgtgatggtaaatctggtccgcttcatctgctgc
gtccaaaagctccgcccaaacaggatctgaaagtagacatgaaaaacttgtgtgaagggatgagttatgt
agacaatgaagaacttatgacaatgattaatagtgcaatacaattacacataagtactgaatcaacatct
tgccaagttccatgcttcaactactacaatatccagaaatggggaatttgctggaagtgtcagttatatt
gtgaaaactgtaacctcattacaccagtatataaactgtataaaactattccgtcaaataaacctggacc
agaccctgccgcacctaatgtcggttttgccttgggattgcaagagaccgctataggaaacacaaaagcg
caacatcttctttcaaatattaatgtgccgccaccatcaagaagtagcatgcagcgaacatccaatgctg
ttagtttgaagacaattgagatgaataaaagagacatgtctgagaaacttgaaatagtaaaatctgtcaa
taggaaaagaggggtgccagaaaatgagataaatgtaacagtggatgctcgttattccagtaacaccatc
attagtaaaaagaaagcaggacaaaatgcgaatcaagccattgctctaggaatagagacagttacggaca
ggaagtatgtggttgctactgcatctttaaaccgcatgtgctggacmggagcatggttgagaggaaaggg
atttgacatcgagtgtccaggaggacatgaagaatgcacagcaaatctgtatcgtgcagcgccgttgtca
gaatatgaacttggcaaacaaatcggaacgcaattagctgttcagggaatattagtaaagtacgccacaa
cagatggagacggaaggtccgcaagtggaattaatgatgctattcaagcacttaatccgttatggacagt
agaacgacttgccgatccagttcatcttggaaatagccagttccgatctgcttatcatgcttgttttagt
gaaaatatgttctacagcagaactaaagaacaaaaaaaaggaaatgcaaaaggcattcagcctagatatt

```
agatcaaggtgcagcttaattgtgaataaactaatgcaaatgcatggacattgtatgcaggagttatcta
agtacttaccaaaagcattagaagctacaataaactgttatagtggggactgttcaatgtgctcttctca
ttcagtcgtatgtgctgggactgatagcaggaactggtggaacaagtcaatttatctccctacatacaac
attacaagcctgcaaatgaatgagaatgataaacatttgttaaatgaaattttgaaaatgaagttgagca
cacaggcaattgatagtatgaaactgtatgacaacacaaacaaaaatgaagcgttacaccgatccgtcag
tgcaaacttgccaaaaaatgttgcattcagtagaaatatggaaggaagattagctgctacagtgcaccgt
agtaataacagtccggggacctcaacaaagatgaagtgtgacagtttaggcattgatttgtccaacagaa
ctaaacagttccttgacaaactcgatcacgataccgaatattttaagcaatatcagaaaagagacaatgt
caaaatgcgcatacttgctaagcgggcacagcaactaagtgatcacaaaacattcaaagacgctaacaaa
gtttccagtgattacaagaaaggccagctggatcctgttttagaccatgaccatggtcaactgtgtaaga
aaagactaccataactttctgacttgagataaacattccagacattaagacgtgtaattgccattaatac
gtaaatgaaaaaacctctaaatttcatgacagtttaaataaatgtgatctcaatattttctaggatgaga
ttcamwggaaactgtgatatckgaaaaatatatattgtcgcgtwaaccctagtatggttgtaaaaatatt
caaaatagtaaattttattatttacggatattattgtttggcatttttagctccagtttgcatggtggta
acggctctggatgcccaaaacattttcctgctgacacacaagtcattcggctgttgttgtagccatgtgg
ttcaagtgataagtgttgcgaattgacacataaagtattgtgacaaagatgagacacatcataattttgt
aaatttatgtgtcgattcgcaaccatatawgacaatctatggacatatagtctggtccacwctcttgagt
ttggcagtttgcagtttattgtgccatattttccatttggggtgcaactccctgtccatatkaaacaggc
ttcattttgcccaactctactttttttctctkagtttgcctccatatttttcaaaaaatgcctccattta
actttattatgtcaaaggtcatatatgtgtatgaatatgtacaccaaaaatgactttgtgaccttatttg
tatcaaatactgcaattatttcttttaatgctcaaatttttatttttttattaaaaaataaaaaaattaa
aattaattttggtaaaaakttgacactgtgaaatagccaatgtcattwkgattctaaatctaccattaca
tttacagtattgcaacaaataaggtcagaatgtcatttttttgtgtcaaatgggaatcggtctatta
>KolobokP-2_MoPh-I
tattagatgcatttttttcaaataaaatttctgttatttcatacacatggaggaaaatgaatatcaagca
agtctacaaagtcttatgtcatttattgatgtagaaaaaaagttacagcacaaattaagaaccaccttac
attacatagagagcttatcagattatcacaaatatgaatagataagaataaagacatctatcttcaaacc
ttaataactttctcatttttttggagtatcttcatgaaataaaatttggcatattcatgacaaaatgaaga
ttataaaactttttttggaattatcttttaactataatacttgtatttttaaaaattgacagaaagaaata
tgttaaagaaatacagattttgaatttcaaaattcaattaaaattagttatcaactttttttcatgagaaa
aaataattttaaaaattagtctaacatgtcattaacatttatcattaaaccaaaatttcaaaacattgtc
tcaaacatagtaaaaaatctttaaagaggggtatcgtgtaatctcatactgtgagatttcctcacataat
ccattatcattcatgagaacagaaaaacatagaaattaaaactctgcttcaggaataagatatttttatg
gtgaaataactagtgcactaatttttagtgaaaaaaaaaaattaagtttaatcatttatttctttgaaaat
tatccaaatttaaaaaatagggtgaaatttaaaatttttatttttctttaaaattcaaattaaaattattga
atgacttgacatttaaaaaaatggagctgtacgtgatatttcagaatattgtaaaaaaaaattaatcaat
gtaaaggagactacacatctggtttagatgtgaatatagtctataagaaggtacccccctgcatggacctt
aagatgaatttccactattatggatgtttatccctttattaatgtaaacagtatataagtcacaacttaa
acttacatttaatgaaaacccttcataaattattgcattcttacaaaaaaaatttgtatgaatcattgat
ttttcaaagcttttcaactgcaattcttaaaaatgtttcaaaaaggtcgaaccgcatggaataaaggtgt
gtctaacttaacaaagagtcgcaaaaaacataacagttctcatttcaaaaaagggtccactccatggaat
aaggattctaaagttgcttgggatactaccaagcctgacactacctatgaacgcatggaatctgcagagt
atgatcttgtagtaaagccaaaaatggatggtatgctgttaacaactcctgactgcgatggaaaccttgg
ttcgatgtgtgtcttgcgtccaaaaagttccccaactgttgatctgaaagcaaaaataaacatgaattta
gctgcaggaatgagctatgttgacaatgaggagcttatgtctatgataaacagtgcagtgcaacaacatg
ctatgaagtctatgagttgtagtattccctcatttcagtactacaacatgaaaaagtggggagtgtgttg
gaaatatcaactatattgtgtacattgccaatttgtgacaccggaatataatttgtataaatcaattcca
tccaaaaagccaggtccaaatcctgctgcaccaaatatcggcttagcattggggttacaagagactgcaa
taggaaatacaagagctcagcaactgctttcaaacatgaactgtagtcctccaacaaagaaatctatgca
aagaatgtcaaaccaagtggctaaaagaacagtggagttgaacaaaaatgacatggcagaaaagttagaa
ctggtgaaagcagtaaacagaaagagaggagcacctgaaaacattatcaatttgacagtcgatgcgagat
actcaagtaacagtattgttagcaaaaagaaaccttgtcaaaatccgaaccaagcaattgcattaggaat
agagactgtaaccgatcgaaaatatattgtcgcaaccgcagcattaaatcaaatgtgctggacgggagcc
tggttgcgtggcaaaggatttgaggtccaatgcccagacggacatgaagagtgcactgccaatcttcatc
gcaccaaacccttatccgagtatgatttgggaaagcaaattggatctcagttagctgtacaaggaattct
tgttaaatatgcaacaacagatggggatggaagatctgcatcaggaataaatgatgcaatcaaggcattc
catccaatgtgggaagtcgaaagattggcggatccgattcatttaggccaagctcagtttcgtgcatcaa
tgcgtgcaaactttagcactggaatgttccatggtaaaaccaaagaacagaggaagaggctacaaaaaat
attcagtcaagatttaaaagcacgatcatcactggtcattgacaaccttatgaaaaaccatggtaatcaa
atacaaaaggtggcaaaagacttaccaaaagcactggaatccacaatgagatgttacgatggagactgtt
ctatgtgtgcttcgcattctgttgtctgtgcaggtggcgaaacaaaaaactggtggaaccgatcaatatt
tctgacatctcatcaaatcacatcactgcaaatggatgaaaatgacaaaaaattattacttgaaattctc
aaaatgaaactcagcttaagtgcacttgaaaccatgaaactatttgacaatacaaataaaaatgaagctg
tacacagagcaataagtgctagtttaccaaaaaaatgtataccatagtaggaatatggagggccgtcttg
ctgcgacaattcacagaaataataataatcctggcacgtctactagaatgaagtgtgactattttggaat
tgaattatccgaacgcacaaataaattcttagacaaaatggatcgagaatgtgaatatcaaaggaatat
caaaagcgacatagtgttcaacaccacagagttcaacaaagagcacaaaaacttatagaaagtaaaactg
ttaaagactgcggttacaaaaaggatcaactagatgttatgccacttgaccatgctgtgtatgcaaagaa
aaaacttatatgatttggacaaagagttgtgagtaggatttttctgttatagtgccattttttccagaccc
tttatttgttgcgaatttttttaattaaatataatatcttttatttgtaaaaaaagagagaatgaattgct
gatataaaatagaagcactataattcaaccgatatgatatgcacaagataaaagtttgtaatggtccaaa
tcacataagtttatgttaaaggttttatatttactaaggttattatttgccattttcagatcaagacgac
atgcagggagaggaaaggggtgtgtcaggcatttcccagcagaaacgcatgtcatgcgactgttgttaaa
accatgcggttcatgcgttaagtgctcaacatttacacacttagtattgtggcacagatgagatacgtcc
aaacctttttaaatctaaatcaggatgaacaatcatatatgctaatctgtgcacatttattttactccatc
ctgtggcatttggaaatttacaatttaaaaccccatatttgccattagatgtgcatgtccctgtccatat
tttgcactccccattttgccctaaaacagtattttcatgtatcttttctgtgcatctctcaaaaaatccc
```

```
tccattttaactttaaaaaagtcaaaggtcaaacttgagataacaggcgtatgaatttgagcatgtccca
tcattttatgacctcaattttgacagttttaaaaaattttaaatttaaaaaaaaaaaattaatttaaattt
taaatttagctttgtcaataataccatctcaaagcatttgtcataacccctctgccaattttcaacaacc
tgctatgaaaattatgcttgcaatgccatttttggtattcagcaaccccccctctctta
>KolobokP-3_MoPh-I
ttataatcccagaaaaattgttcaaacatgataaaaatttgtgttcttctttttttgttatatttcaaact
aatttgccaagtttcacattgatctgtcaatttctggctttgcaatctgggttcaaatacacctaccctt
tgttgtgaaataaagacattttcctgtgttaatttttctgcctcctgaactgacccctttttgctaaaatg
gctgtaactttccggggactaagacaaaaataagcgaatgcttcaaaatataatttgtatagccgttgac
tacacaacacacgaatttgttacacacgtgttgcatatatcatcgtcaggtcatttcttacgtaatttaa
gcacaggtgaaaatcgtttattggcacaatcaacatcaggggtggctgtcaaagggtcatactgtcaaag
ggtcataaaacaactgaaccataaacaatctactttgtaataatcccagaaaaattgttcaaacatgata
aaaatttgtgttcttctttttttgttatatttcaaactaatttgccaagtttcacattgatctgtcaattt
ctggctttgcaatctgggttcaaatacacctaccctttgttgtgaaataaagacattttcctgtgttaat
ttttctgcctcctgaactggcccctttcgctaaaatggctgtaactttcttgtttttccatatttctttg
aaaaccaaacaccatctgaaagtagagaggttcaagttaatatatactgcaaatattatgacaaaaaagt
actttttagaacacttttaactgttggaatttgactttttgaaaaaacaagcaaataatctgaatttaat
tttgaaacattcataaaaaattgaaatttgaacatagaaatttacaaaatatttcaaaaaattgcaaatt
catttttctattcatgcatgtatatwacatgtatgtattaattacattaaacttacaataaattgtttat
tgcaggtacccccatcaaggcttcccccagtcaacatgggagataactacaaaaatttgatggttgcaaa
tttcttctttttataaagtttttcaacaaaaaaggactttttttggaaagaatattgtcttaacaataaat
tctcatgaaataaacacttaaaagcccaaatttattttttgtacagatttttttttctttttaatcattt
tttcaggaaaaaaaaatttcatttattgaaaaaatattttttttggactttaaaactcttccaatgtttaaa
attaaactttgaaatattgaatcattcatatttttcctaaaataaatattttggaacagcaaaacaccat
gtttttgatctaaacatgatgtaaacaaggggtggagctaatgagcatattcatctgatccaaattcact
tgcaataaacaatctaccattatgtaacttacagctgtcaatcataatggcttgattgattttaaaaagt
atataaactgcaaactctaagggttgggcatcacttcgagagttgatgctgtagtactgttccacagata
ctgcaagatgttccaagttggtcatgttccagttaacaaagatgctgttctgcagaaaaggaagatccct
aggcctggaaggttccaaaaaggcaacactgcaaagagagctgcactggataactcaacaaatgaagaat
atgctgctagtacctccaacaccagtaccatacatgcatcactagagactcataactacatcgaagatgt
aacgccagagatgcacgatgatacagatgagtggaagctcaaactcagtcatcgtcgttgtccaggatca
gtcaacagcaatctgaaaaatgaagaagaatggactgtactttcaggaatgcggttcatagacagcgagg
agatgatgaaaatgatgaacactgcctacaaaaaccatagagcgacatcaacagtatgctgtgaacccca
aatggaggcctaccgacagaagaaatggggagtctgctggacttactacttgagatgtctcaaatgtaca
tactggagtcaacctttcaagctgtataaagaagttgtgaacaacaagcctggtccaaaccctggtgcac
caaatgtagcacttgcaactgcattgcaagattcaccaattgggaacacaaagacacaagagattttagc
cagaatgaattgtccgccacccagtcgcagtggtatgaataaaatgtccaatactgttgggaagaagact
gttgccttaaacaagaaggatatgggtgaaaagctggaaatggtaaaggcagtaaacaaacagagaggaa
ctccagagaatcagataaatgtgacagtagatggaagatacaacagtcagacaattgtgagtaagaagaa
gcctggtcagaatgcatcccaagcattttccctggccattgagacagttacagatatgaagtttattgtt
gcagcagtatggcagaaccagctttgttggacaggggcttggttgcgaggtcaaggttttgatgtcagtt
gtccaggcggccatgaggaatgtactgctaaccatcatagagcagccccactttctgagtacacaatggg
gaaagagattggttctcagcttgccctacagaatgcattggtgaaatatgcaacaacagatggagatggg
agatctgcagctggtattgatgatgccatgagggcccttcatcccatgtggaaagttaacaggctggcag
atcatgttcatcttggacagacacagtttaggacagctctgagagcacgttttagtgaacagatgttcca
tggtaaaaccaaggaagaaaagaaagaactgcaaaagatactaagcaaggatgtgaaatgcagaagtgca
atggtactcagtaacctgatgacaaaatacaagaagaatatagaggaaatatgtaaagacctacctgcgg
tacttgaggcaacacttctatgctacagtggagattgcagtctttgtagtcaacactccttggtatgtga
tggcactgatacttcaaactggtggctaaggtccaaatatctgtgtagctacaacatcactgtgctgcaa
atggaaaaagaagatatgttactactacaagagatactaaaaatgaaactcagtgaagakgcaattagaa
gtatgaagctttatgacaccaccaataaaaacgaagcagttcatcgatccctgagtgtgaatcttccaaa
gaatgttaatttctcaagaaatatggattcaagattatcgtcaggagttcatcgcaacaacaacagacct
gggacctcagctctactgaaatctcagcatcttggtgtgaacctctgtgaccacagtacagcttacctta
aaaaaatggacagagattttgagtacaaaaggaggtacgagaagaaacccgaggtgaaaaaacgacgact
acagcttagtgcagaaaggctacaggagcacaaagcatacaaagagctccacaaacaacacccagagtac
aagaagggccagcttgatcaagttcctgccatagtacttcatcaagactactgcaagaagaagatgcctc
cagatgcaccatgatgtggaaaacaaacaaaaaggcccttttaagggccaccaaatttctcaaaaagaaa
ttaatgttgttattatcaaataaagttatcttgaagcccacagaatacctccccgtcaaaatcatacttc
cggcaatgcccatccacacccctccccctccccttcccatcaaaatgattgaaaaaaaaatggaagaatt
ctcccattattgttttgggaagtaattgaaagatcttgtgatttaatttgaaccaaaattggttggaaat
ataccctattcccatacaaaatggccgctcaagtaaaaaawttcaaaaatgacaaaaacgggctaaaaag
gggggattctgaaaacattctatcatataaacatttgattccacacaattcttccaaattttaccttgtc
cacacatttaatagccagaaatttccaaaaattgtaaaaaaattgatttcatgtaccccactacta
>KolobokP-4_MoPh-I
ttaataaccacttaaattttacaaaaatcaatgaaactttctgatatgatacatacatctattctaaaca
tttttgccaagtttcactgtgatttatcgactgtgacactcgtattttgatttttttcaaagtatactctc
atgcctggggaaagcagatttcttcaatgaaaatagcctgttgaacgccaatactttggcttatgctaca
aaaaattgtttcaatttatttgaataatttttttcgctattcgaatctactgttataatactttaaatccg
gggaaaattcacatgtcaaaaattcaacatttttgaaaaaaattaaatttcattcaaaattttgatttaa
aaaatgtagattttcttcaaaaactgctatttttttacccattaataaaggcaaaatttgcattagtttgc
aaattaatttaaacaaaatatcagagagtatatcttgatagcaagtattgacaatccaattagttttaag
tatacatatggcctttacaaccatggggtgttgcgaagacagtgcatatttgtctgtgagggggggattaa
aaggattagctcaaatttcactttatttaatcaataagaagtaaatcatataaaattgggtagctaacta
tattttttaacattatgggagctgtcggagtggaatttttaacctatttctccaaaaatgtccagaaaac
gaaagaacttatcaagaaaacatggctttcagaaaggcaacacccagtggttgaaaggtaaagaatttaa
ttttccagacaaaatttcacatgttagaagcacaagattgtctaaaattgtttttgactctaggactgtc
caaagtagtaatggcacccttacaatatttgacgtaaatggttcagaaagcctcatgaaggtgttgcgtc
```

ctaagccatccatccctgaggttgtagatctgtacatgcctgattgtaatcctgatgttgataattacac
atacaaactgtatcaacctgtaaaaattgaagaactattcaatatcgcaattagagaacataaaaaatgc
tcaggcaagttgatttttgatggtaaatatgctcaaaaagggggtaacatggagagaaagactatatt
gccctaaatgtgactataaaacacaaccccacagcctttatgaagaggttgtctcttcaagaccaggtag
aagagctgctatgccaaatatcagacttcaaaatgctctttgtaccactcctatttcaaatacaagtctc
ataaatatccttgtgcaaattggtgctatacccccctcttacagtggtatgcaaaagacaactaataatg
taaacaaacaactcgtacaaatcaatactgagagtatgaaaaatattcgggagacaatatatcaagaaaa
caaggactgtgggctattaaatccagaacttgtacgtgtagagagtgacggtcgctttaacaatccaatg
tttaagaacgacacaccgttccaagcaggaacacaagttacttatgtaatgattgagaataattcaaatt
taaaaagaataatatctgtatttgcaggcaataaactctgtattaaagcctctatgttaagaaatcaggg
gcaaactgtcatatgtccccaccatgaaggcatgtgcacagctaatatagctgaagatgtatcaatagga
aatgaatctgcatggagtgctgagtgtgcaaaagaaataaatgatacattagttatttcgcacttgacca
ctgatggtgacagcaaggctatcattggtgtgcgcaatactcaaaaaagaggatgttgaatcattaagag
atattcgacatctttcacaaactatgaaaagacatatcatgaatctcccattcaaccccaatatgttttg
tggtccccgtcgcttaaatttgaaaaggcgttttgcccaagaagtgactttaagatgtgttgctgaactt
aaatcaattcattccatgcttaacaatcacatagaatatatacagtcttgtatgcccgatactataaatg
caattgtactgtgtttaaaaggtatatgcggacgaacttgtagcaagttttcatttgtatgtaaaggtca
aaaatatgaccattggcaaaataggtttatgcctaacagatcagtattgaaaatgacatgtgatgatgaa
ttgaaactgtattcagctattgaaattttgcttagtactgaaagtctgcatcgtactagatttcttacct
cgacacaaaaaagtgaggctttcaatagggttctccagcgtgttaatccgaaaacatctacttggcccgt
gaacttcccaggcagagtgcacactgctgtccacatgacaaatcatggttttgttgagtcggcattactt
cgcactgagttgttgcatgtaccactcaagaaaggcacatcagttataaaatacctgaaatcaagatcaa
aatatgacaaagtaaaaaggaaagcacatcttttaaaatcctctatgcaaaagcggtgtgcaacaaggaa
ccgtaaattcaaactgcatttggaaattcagtgtgaagaatacaaaaagggtacatctgatcctgtccct
gctaatgtaactagtacagttagaggggaaaatgtatgatttaatgaggtgactgtccatttaatgggtt
atggacatggacttgtgcaaagtgtttaaaaaaaatggggaggggggaattattgtgcatgtttagatgta
aatatttctaaatttggtgtaaatattttaaaccgggtgttgtaaatagaaaaaaaaataaataaaaata
cactttggatatattccatgtccacaacattcagctggtactcttccagtgcagttctttctcctgttgt
taacatcctggggctcaagtgacaagtgctcaggcttgatacacaaagagttgtgacataaatgagacac
atgtaaacccacaggtatgtgaatagttctgcgcgaacacatatatgatagtcggtgcacataaacattc
ttagaccgcgacatatgtggaaattttacacgaattgtcccatatttatgtgctgggggacctacacccc
ccttttttccatatctggcagtcaaaaacccccttcaggttcagagtttttggcaagtcttgctttctggtg
gttaaaccatgcggtattatccattttcagttgtttttttatgcaaaaattttgaaaatcmgtgtcaaaat
tatcattacacaggtcaaggactcacgtgcgatttcccacttaa
>KolobokP-5_MoPh-I
ttaagacagccttattttcccccgttattgaaatggattttttttctagcaacatggatgtcttacctaat
gacggatattagttttttaaaaaaaaaaccacagaattaagggagataatcaagaaaaatagaacacacc
ttgttcaaggaaaaaagaaaattctgagagaaatcacaaatttcatatgtttgatttaaaagccattaca
tgtaaaaaggtcaaatttacaatatgacaatcatataactccatgaaacacagtttgaaaaatgctaaaa
aagacattaaaaatacagagatgctgagttggattttgccaatttttttkgtgcaagacttcagttttc
aacaagctacataagagaaatttgtaaatattacaaaaataaaaaagaagttggaggtcaatttaatatg
tttgcaaagtaaaatcattaggctattagataaattaattgcttcacacagattgttttaamaggtgaca
ctagaggccatttaaaatcagcacccctctccatggatgaaatttcaattagctttatggcccctttaat
gcctttgatttagtggctaattaatttcccagtaaaccaaagtacttataacagttagaatttatgataa
agtcccaaaaaaaagtatgccaaagaagggtcgacaaaagaaaagggtctattttttttgaagatggccat
ccakgttacaagaaaagaacaccatcagatgaggccgtgttggatgtacagtcacaaagattaccttcaa
acacttttcaggatgctgtccagatttctgctgatggaaatcacttcaccgttacagattccgatggaaa
tccaggaaacatgcgcttctttcgtcccattcctgaggataccaatgaagatcatctagacctggaaaat
gacagaacagatctgtatggctacaaaatattgcattgtggtaaactgatggacatgattaatgactttt
acagtgaacatttaaaaaacagtccgagatgcaaacctttacttagatttaatccacaagactgtacaaa
atggggattttgttggcggattgctcttaaatgtgagaactgtaacttttctaccgagaaaaagaaactg
tatgaggaaagtgaaaaacagacaccgcgaggacagagatattcaactgtcaacctgggtattcatgtgg
ggttacagagcaattcaattggaattgacggattaagaacattaatgctaagtgctggcatccctgtgcc
ctcatgcagtggcatgcataaagctggaatatatgtgagtgacactactacaaagttaaatgaaagtgac
atgcaaaagagacgtgaaaaactaactgatttacacaaaatacgtggctttgatgcatctcatccaacct
ctgtatctggagactgtcgctacaacaaccgtttggatagcagtgtgggtcacaagcttggacaggctgg
tacgcaatccgtatactgtatatctgaaaatgagacaaagtcaaaagatataatttcagtgaacattgac
aataaaatttgtgtaactggtacaagattaaatgcgcggtgtccggatcatcctggaatatgtacagcaa
acttggacaaagatgcagtcattggagacgaaggtcgttctgcggagcgttgtgcacttacgatggcaca
agaccgagtacctgttattgttggacagtttacatcagatggagactcagctgctagcacaggatttgaa
aggggacagaaaacacacagtaatgttccggtggaaaacttgcgtgattcaagacattttggtgaatctc
agaggaaagtgctgaaaaacattaagtttagtaagacaatgtttcccggcagaaacaaggcaactaggga
taaagtacaacagcgattttcaattgaagtgactaagcgatgtaatgcggagaaaaatttagcacataaa
caatatgcgggtgacattaataaaataattcgtaaactaagctatactgtggacagcgtattacaatgtg
taacaggggactgtggacaagcatgtaagaaacattcgtttgtatgttccggaatgaaaaagactgcatg
gaagccaaaatttctagaaactggaactatctttaatccaacagacaatgatttaagactgttgcgtgac
tgcatagtattaagactaggtgctaaagggattagaaagactcgtttgaatacaaacacacagaagaacg
aaagtatcaaccgtacatttacaaagactaacccgaagtcaacaaattactgtaggacacttagtggtcg
aattcacggtgcagtccatatgcgtaacaatggaagatccaattcaatttgtgaaaggctcagggcagtg
ggatcaagtgttgattcatcaactttagtgctgagacaattgaaaaatattcaaagcaaagacacgtatc
acaaggctagaaagaactcaacaaaatataaagcaagacaatcttatttgcgtcgtgtccgctttaataa
atctgcagttgaaccacacaatacagggacttaccgcaaaaatatcttggatccagaaatgaacgatcat
actagtctttacgcaaagaagaacaggcgttacagaatgaaggtggaataatttaaagtgtgcaatctgg
atatggactgtgagactgtgtacatttcttctccgagtaacaatgttgtcgtgcattattgatatgttgc
ggttcaaaaactaaatgtgaaatattgatacaatctttagtatgacataaatgtgacacatgatagtcag
aaggatttagttttaagacacggttatgaatcatataaacaagtctgtgtacgtgtatcggtgaatttct
ttgtgaatttgggaaacggacatcaattacaccatatcctgtctttctatgtcttccaataaaatgtata

caagtatttttttcatctttttcagagtttttatcaattctagacatgagatttgatgccatttgttcac
tcattgtgaaatcaggtgctctggccatagtttaagtatttttctgcaaataattaatttattgccaaat
atggtaaattaggggtttatctaagatggctgacaaaaattacccagcatccgtacctgttacataggtt
atctccccgaaatcatacgtccctatgtaacggttaa
>KolobokP-6_MoPh-I
tattagatgtctaattttttcaaataatgcagaaaaaaattgacatcagtaagaaaaatgtattacaaaga
atgctaccaagtttcaaaacaattggacacataataaacaagttacagcaactttcatatgataacccat
actgtctgaaaacttatctgtgcaaagcaatacagagaaatttctggtatagacttcaatcttaaaaagt
gaagaactttcttatttcttggagtttttacatgaaataaaatttaccatgcttcagagagaagcagcta
ttttttggagtgaagaaatcacataaacatttaatagatttgctttaaaaaataaaaatgttaaaaacat
taaaacaatacaagttttatttttcaaaaatcaaataaaattttaaatttacttattttgtgaaataatt
tcttcacaaaaatccattcatgacctggcactataataaaagtggaaataatgtgctttcataaacttga
ttgaaatacagagtcttctttaaagtactacctattcaaacaatttccatgacttttcattcactttaca
agcaagtttccaaactggaatttattatttcagaataaagtatggattttaaatatttgaatataaaaag
tcttatgtattttcaattttttagcataaaaagtaaattatagaaactttttgtttggaatttgttcagc
aatttaaaaaaaataaactttttaaaaaagatattaacttttttaaattaaaataaaaattcaaacaaaat
ctgtatcgcaaactctttattttaaaaaattgcagtaatatgtaacacataaaatattagtagcagatac
aaaaatgagatttggtaaacaacataaatctcctcaataacagaccaccccctgcacagattgcagttaa
gtacattcaactgaggtcatttgactgtctaattaatccaaattaaagcatcaatcatcaccttaagtaa
attacataagaagtctataaaatgaagtgtatctttacccaactctttcattctaaatcattattgcttt
gaaataataaaagaaaaatcatgtttaaaaagggaggaactccatggaataaaggccgcactggattgat
cagcatgaaaattcctaaaaagtcattgtttgtaaaaaattccacaccatggaataaaggaactgaagta
ccttgggacaaatccgagccagaaaattcataccagcgtatggaatccaaagagtatgaactagttgtca
agccaaaaatggatggaaaactactgtcaacacctgactgtgatggtaaatctggtccgcttcatctgct
gcgtccaaaagctccgcccaaacaggatctgaaagtagacatgaaaaacttgtgtgaagggatgagttat
gtagacaatgaagaacttatgacaatgattaatagtgcaatacaattacacataagtactgaatcaacat
cttgccaagttccatgcttcaactactacaatatccagaaatggggaatttgctggaagtgtcagttata
ttgtgaaaactgtaacctcattacaccagtatataaactgtataaaactattccgtcaaataaacctgga
ccagaccctgccgcacctaatgtcggttttgccttgggattgcaagagaccgctataggaaacacaaaag
cgcaacatcttctttcaaatattaatgtgccgccaccatcaagaagtagcatgcagcgaacatccaatgc
tgttagtttgaagacaattgagatgaataaaagagacatgtctgagaaacttgaaatagtaaaatctgtc
aataggaaaagaggggtgccagaaaatgagataaatgtaacagtggatgctcgttattccagtaacacca
tcattagtaaaaagaaagcaggacaaaatgcgaatcaagccattgctctaggaatagagacagttacgga
taggaagtatgtggttgctactgcatctttaaaccgcatgtgctggacaggagcatggttgagaggaaag
ggatttgacatcgagtgtccaggaggacatgaagaatgcacagcaaatctgtatcgtgcagcgccgttgt
ccgaatatgaacttggcaaacaaatcggaacgcaattagctgtacagggaatattagtaaagtacgccac
aacagatggagacggaaggtccgcaagtggaattaatgatgctattcaagcacttaatccgttatggaca
gtagaacgacttgccgatccagttcatcttggaaatagccagttccgatctgcttatcatgcttgtttta
gtgaaaatatgttctacagcagaactaaagaacaaaaaaaaggaaatgcaaaaggcattcagcctagata
ttagatcaaggtgcagcttaattgtgaataaactaatgcaaatgcatggacattgtatgcaggagttatc
taagtacttaccaaaagcattagaagctacaataaactgttatagtggggactgttcaatgtgctcttct
cattcagtcgtatgtgctgggactgatagcaggaactggtggaacaagtcaatttatctccctacataca
acattacaagcctgcaaatgaatgagaatgataaacatttgttaaatgaaattttgaaaatgaagttgag
cacacaggcaattgatagtatgaaactgtatgacaacacaaacaaaaatgaagcgttacaccgatccgtc
agtgcaaacttgcmaaaaaatgttgcattcagtagaaatatggaaggaagattagctgctacagtgcacc
gtagtaataacagtccggggacctcaacaaagatgaagtgtgacagtttaggcattgatttgtccaacag
aactaaacagttccttgacaaactcgatcacgataccgaatattttaagcaatatcagaaaagagacaat
gtcaaaatgcgcatacttgctaagcgggcacagcaactaagtgatcacaaaacattcaaagacgctaaca
aagtttccagtgattacaagaaaggccagctggatcctgttttagaccatgaccatggtcaactgtgtaa
gaaaagactaccataactttctgacttgagaaaacattccagacattaagacgtgtaattgccattaata
cgtaaatgaaaaacctctaaatttcatgacagtttaaataaatgtgatctcaatattttttaggatgaga
ttcaatggaaactgtgatatctgaaaaatatatattgtcgcgtaaaccctaatatggttgtaaaaatatt
caaaatagtaaattttattatttacggatattattgtttggcatttttagctccagtttgcatggtggta
amggmtctggatgcccaaaacattttcctgctgacacacaagtcattcggctgttgttgtagmsagkggt
tcaagkgataagtgttgcgaattgacacataaagtattgtgacacagatgagacacatcataattttgta
aatttatgtgtcgattcgcaaccatatatgataatctatggacatatagtctggtccacactcttgagtt
tggtagtttgcagtttattgtgccatattttccatttggggtgcaactccctgtccatatgaaacaggct
tcattttgtccaactctactttttttctctgagtttgcctccatatttttcaaaaaatgcctccattttaa
ctttattatgtcaaaggtcatatatgtgtatgaatatgtacaccaaaaatgactttgtgaccttatttgt
atcaaatactgcaattatttcttttaatgctcaaatttttatttttttattaaaaaataaaaaattaaaa
ttaattttggtaaaaagttgacactgtgaaatagtcaatgtcattatgattctaaatctaccattacatt
tacagtattgcaacaaataaggtcagaatgtcatttttttgtgtcaaatgggaatcggtctatta
>KolobokP-7_MoPh-I
tattagtcactttatacatgatatatcattaaaataaaacatgatttatattttgtaccttatctttgat
taatctgccaagtttcatggtgatccgtcttttaattggctggaaatctgcgatcaaacacaactaccca
tactctggatttttcatgaatttgtcagcctgaattttccattttcctgatctgacccctctcactaaaa
tggctgtaactttgttgttgtttcatgcatcttcaagaaataaacatcatctgaaagaggaataaataat
ctaaaataatattgaaaaacatttagtaaaactgtgcttattcaggcctaaattacaggttgatttcaaa
aaattaaaatttttcaaaaaatttaaattttcaactttttaatttcaaataactttcaaaccgtcgacca
aaaaaatgtcagaaattagcagaaaatatcttaaatatattgcaaatctgattctgttgcacttcggtat
attttttcatacaataatagtgtaaataacatttactttctataaccttttttttcaaaatttcaaaaatt
ccagaaataatggagttcaactacagatttcaacagcttgtgaactgtttatttttttttaaattkcaagga
caatttgaggtttttttgtgaagaaatattcataaattaatgaaattcactgaaaaattacacaaataaac
ccttccaaatgttatttgaagagcccgcataccccgggtacatttttctgggatttttcttttttttttct
ttttttaatgtgtttaaaatttcttaattttgaaaaacaatgcataatataatggaaaaacaatttaat
taaccaaaacaatgcacagttcaataagaataaggacccagacattgtttgtttgatgtaaacaaaggga
ccctgtttgcacacatggacactgtctcttactgtgaaatcaaagaagtcaatttcctattgaagagttg

ttaattacatgtaattaacatcccctcctcccttataaaatatgtaactgtcaatcaatatttaaaaaag
tataaaaatccaaagttttgtggactggacatcattttaagtcacggtttctgaaaaaaaaagatgttt
accaaaggttgcgttccgttcaacaaaggtgtaactggacttcagaaaagaaagattccaccagctggcc
ggttcaccaagaatcacactagatggaagggttctcaaaaaacaatgacagttactgcagaggatgaaag
ttccaacaccagtaccatatatgcaagtccggtgcttcatgaacaaagctcacatgtaacaccaagcatc
cacagagatactgatgaatggaagtctaggctgcgttctggtactcctgctaatctgaagagagaagaag
aagaagaagaatgtactgtattaaagggaatgagatttgtggacagtgaagaaatgatgcacatgatgaa
tacagtaatgaagcaacacagaaagacatcaatgacatgcgatgaacctgaaatggaggcatgtacccag
aaaaagtggggagtctgctggacataccagctaaggtgtgtaaactgttccttccggagtgagcagttca
agttgtacaaagaagtaatgaacagtaagcgtggtcctaaccctgcagcaccaaatgtagcattggcaac
tgtacttcaggacagtcccattggtaacaccaagacacaggaaatattggccagaatgaattgtccaccg
ccaagtcgtaccagcatgaaccgaatgtccaatacagtgggaaagatgacagttgacctgaatgtcaaag
acatggcgaacaaggtggagattgtaaaagaagtaaacaggcagagaggtgctccaatcaatgagataga
tgtgaccgtcgatggcagatacaatagccaaaccatttccagcaggaagaaaccaggccttaatgcatca
caggcattctccttagccatcgagacagtgaccgacatgaaatacatcatatcagctgtctcacaaaacc
aactatgctggaagggggcttggttaagaggacaaggatttgaagttaaatgtccaggaggccatgaaga
gtgcactgcaaaccactacagggctgcaccactgtcagagtataccatgggaaaagaaataggaaatcaa
cttgccctgcaaaatgtcttggtkagatatgctacaacagatggagatggaagatcagctgcaggaatag
atgatgctacaagagctcttcacccattgtggaaagtaaacagactggcagatcatgtacacctgggtca
gacacaatttagggcctcaatgcgagcacaatacagtgatggcatgttctcgggcaaaacaaaagacgag
aaacaagaactgaagaaaatatttgcctggatgtgaaatgccgcagttccatggttgtagataaactta
tggagagatataacagagataccgagaagatatgcaaatgcttacctgaagtcctggatgcaacactgag
atgctatgatggtgactgcagtcaatgtggtcagcactccattgtctgtgatggcactgattcttcaaac
tggtggttwcgttccaaatatctgtcagtctataatattactgccctgcgaatggatcaaaaagacattg
tattgttacaagagatactgaagatgaagttgagtgaaaatgcmatcacaaatatgaaactatacaactc
tacaaacaaaaatgaagcagtacaccgtgccatcagtgttaacctgccaaagaatgtaaacttttcaaga
aacatgaatgccagactatcatcaggtgttcacagaaacaacaacaaacctgggacttctgctttgaaaa
aaagtgaacattttggtattgacttgtgtgatgagagttgtaactttctgaagaaaatggatcaaacctt
catctacaaacagaactacgagaaaagaccagaagtgaagaaacgtccctccaacaaagtgtcgagaaac
tgcaagagcacaagacatacaaagaaatgaacaaaaccatgcctgactacaaaaaagaccagctgaacta
tacctgccctacagtttcatggagactattgtaagaagaaatgtcatcttgaaccagctgaagaacaaca
ataaaaggcccttttcagggccacctaaatcattagaaaagagtcttagttgtgttgcacaaataaaaaa
gaaacaaaaaataaataaataaataaatgaatgaatgaatgaatgaatgaatgaatgaaacccatccccc
aaaatagcaaaaaataaaatactaatgttattatctgccatctttaagtcaatcatgcacggcctgagag
gggcggcatggcctaaacatctgccacacgaaacacatgtctgtctactgttgttgaagccatgcggctc
caacaccagatgatcaacattcatgcatttcgtgttgtggcacaaatgcgacacatcaccatcaaccaac
tgaataatgcgatgttcaagcatgtacgcaagtctgtgcacatgtatttgtctccaccctctggctcctg
ggaatttacaattgataaccccatatttgccattgggtgtacaagcccctatccaaataatgcactctcc
gttcaagcctggggctgctcggccakccagttttgctctatatttgttaataaattcctccatttttctc
tagagaactttttcaaggactgggagtatgaatttgcccctgaaaattaaactcggsaacccctgttttc
aagaaactgacaactcttatgtcaatttataaaatttccaaaaaaattgaaataatggataaaaaatgcc
aaggtgcataaaatttgaattaatttcatatatttgtcctcaaattgaagtttacctgaagtttcaagac
aatacattggaaaaawgtccactttctacaacttgacccccccccccatta
>KolobokP-1_BaPl-I
taaagagcaactttttgggcttcaaatttgctaaaacttgctgcaaacttagtcatataaatttcgagta
aaattctagaattttggcattctccgtgcagggctttcctaaaaattacaaaaatgtaaagccttataa
tgaactgttaggagatttttttttcaaatatgtgtcataacgcttctttcggaaaatggaaatttcaag
tcctcaaacaatatcagtttataattttcttagtgaaatgcattaaacacatattagacaagatcaaaac
atcaatacgcggtagaaatcaaatttaatttttagctaaatgaaatttaaaattaaaattttnattttta
agagattaaatttgatgaatttcattaattttgatcaatcatcttttatatttttggatagaaaatatga
aaacctgtaaataaacattatcaaagtattttgttcacatattgtctccaaatctaattagcatcttcct
ccagggcacttctggctcagcaggtataatacctgcaattcatataattttcagagggggtccaataaa
tggctgttattaattagatatgataaattttctataaaagtttggttttcaataaaaattaaacattcct
gcacattttggaagaaataacatcatatccacagttttataagcaaatttgaaggcagaaatggaagaa
aaaggaaacaactttcaaaaatatttggatttaaggaagggaacacacccagtaataggggtaagaaatt
agaatatgaacaaaattctagttccgaaccttttatgaggttgaaaggtggtgtttttgagtctagagtc
acacaagagagtgacggagtcctgacaattctggatgttgaccaatctccttgtcctccaatgttgttac
gtcctcgccccaagagtcctgaagttctcgatgattatcttgaatcaacagtctctgatcctgacaatca
cacatacaaacattatgttccttcacttgtgagtgttctttggaattctacaataaaagaacatgcaatt
gagagaaatggttgtgatggagaactagagtttgactccatttcatccaaaaagtggggatttgcatgga
aagagaggttaaaatgtaaaaagtgtgactttgttggtcaatatcacaaattgtactatgaagtagacac
ctctggtccaggtagaaaggctgccaccattaatatcggcttacaggcaggcttaatgactacccctata
tctaacaagagttttcgggatatcacaataaactgtaatattattcccgcaagtttgtccagcatgcaac
atcttgcaaacaaggctggttctgtcattgtaaaacataaccgtgatgatatgaaagaaataagagaaac
cattgttgaagaaaatgaaatgtgtggctttgaaaatccacaccttgtaaatgttgagtcggacgcgaga
tataacaatccaatatataatagtggtgtaaccccatttcaagctggaacccaagttgttcaaacaatgt
gtgagaataatactaaaaataaaaaaatagtctcagcattccttggtaataaactctgcaatgttgcatc
acgactgcgaaataaaggtattgcggtgaaatgtccccaccataatggacattgcactgcaaatttagca
gaggatgctgtaatagggaatgagacagagtacagtaggcaatgcactactgaattaaacgatacactta
aaatagccaatataaccaccgatggagacagtaaatccttcaacggtgtaaataatgctcagggtaaagg
tgctacacagttacgagacatacgtcatctgtccaatagcatgaaaagggctgttcaaaattgtacgttt
agtttgtcaatgtttgcgggtaaaaataaaaataatatgaaaagcaggttcgcaatggacctcaaagcac
ggtgtgtggcagagctccatcaagcttttaaagcacacaaaggtcaattaattgaagttaaaaagcacat
gccaaatgtcattaaaacaattgtgatgtgctacaaaggttattgtggtatacactgccaaattaactcc
tatgtgtgtgcagggttaacctcaaatcactggcataaagaatttattccaggtaatacatctttgaaaa
tgacacctgatgatgaaatgtcagttgagaagtgtattggtgtactattagggtcaaaaagtttagaact
tgtaaggtttcttacctccacccaaaagtgtgaagcattcaatcgtacacttcagcggtgtaatcccaaa

aatgtcacacactcaagaaacttccctggtcgtactcatacagctatacacttgcgaaatcattggtttg
gtaactctacgctactgcgaactaaagtcttgggtgcggctttgacaccagggtcatctgtcatcaagca
cctaaaacaagagcagtatttagaagtgtataggagcaagcgtaaacagttaaaagagtcaaagcaaata
cggtctatgactcgacaamgaaagtatgaaatgcatgctgcgatatgttataaatcaccgatacattatc
gcaaaggtatagcagatccaaaaagttcctaacttatgggtcatgctcatggttatgtaaaataaacagt
ttttttttactatgattatctccctttgatatgcttgtaaatattgtacatatttgaaataatttgtata
tggaatgtttaatattttctttttattgttacctaaatcataacaaacagtctttgtaaggtgcatgcga
tttacatctagtgggaaatataccacggcaaatttgcctgtctttgttgacacttgccggttcatatgac
aggtgatctgtgtttatgcaccgcctgttgtgacaccggtgcgatacctgcatgtcactggaaaggtcga
agcattgtacgtgaagcatgtaagcaaagcgatgacatgaaactgtctttttctttttactccaaggaaa
agtgacggtcaatgttgaataggccgtctgccctccccccaatttgcataacaaacaaccaaattcccca
ttggcctctgaattttgtaaaagtctctgtttttgggcttctatccaatcatccatattaattttaaaat
aaaatgtctgctaaaagagtaaaaaattacaacatagtatatttttggggtcaaggctgcacgtgcagaa
ttttcttaa
>KolobokP-1_PeVi-I
tatagaaaacatgaagcggttaagaatcagtactcaagttcgcattcataaagcacatcttattacaagt
acagaaaccaagttaggtgaaaatccgttaaagtttagtcgagatattccctttttagtttggagtgcga
gtttgcggccagtgatgaaaaatttccacagaaagcaaaaataaaaaattaaaaatggaaaaatttctta
ttaaaatatcttagaaattattatgacttatgttttccaatactttaagcaatactccttttatgcatgt
acaagttgaaatctaaatttattaagggtagaatctacccttctaatagtaaaaatacaaaaaaatggcc
aaaagttggaaattttccttaaaactttctaaattaagtgaaatgaagatgcactttctaactttatcat
gtaatttttaattgttggtgataagaggtaccagaatgacagcaacaaattttgcttgtcatcatgataa
ttatagaagagccataagatcatcaaatggcatctttgaagaattttatatgtttgaaggggggcacct
gttgtttatcctgtaaatagtgcaaagactgaacttttcacatcaacttgtactgcaaaacaagggtata
aaataaacttgcatcagagcacaccaacacttatcaaagctaaattaatagagtcaacatttcaaattta
taaaatgtcaggattcaaaaaaggaaataaatacacatttaaatctgggaatttacctcacaacaaagca
aggactaaactttcaacagtgaaaagtgacttagaaacacctaaatacattagacttacaaggaaaactc
atgaactggttgttaatgatccatacactgatccagaggagaaggcagagcggtccagtcgtgcggccaa
gttactgcgaccaaaatctaaatctgttcctgttgctaggaaatctaagaaaggagctaaaaaagaaaaa
aggtaggacatgtaataataaataacataattaacttacaatcaaacagtttattaatgtagattttgaa
aaaaaaaaaaatatatgccatatgtgcacaattttcaaactatttaggatgtttttttccttaaaacttgc
aaatttatttccaagaacacaaattcttgcactttgtatagtaattaatctttatttaagttataaagta
ggaaaaaatcaactagatatcagcccaatatacataaatgctccattaaaaaaaatattaaaattaaggg
ggagataactctaaattgcatttgaatatagaaataactatagtgttagtaggacattaaatatggtttc
ttttctaattttcaggttgaattctgattcccacagagtgctgtgcctttacaagtcagaaaacatgtgg
aattgcgcatttagagagcatatgatgaaaagtccagactgtgatggtattttactatacgatttggaca
aagaagaaaaaagaggtttctcatcaagatttcgacttatttgcgcatcgtgtaaatacttttcgaagat
gtatacacagtatgaggaacttgaaaccggcagaccgggtagaaaaccctcaaaactaacaactggtgta
ctagttggcctgagtcagactccaattggtcctacaagcttgcgcaaaatctttctaagtggcaatattt
caactccagcagcctctagcctgcaaagaaggacaaatcaagtatataaaaacattgtacaagttgtaaa
aaaagacatgaaaagaagaagggaagaattagtcgaaataaaccgcctccgaggaaagaaaaatccaaga
tgtattagtgttcaaatggatggcatgtacagcaacagaccctactcaggaattggacgcacaccgttcc
aaccagctaatcaggttgtgtatacatcagctgaaaatgtaacaaaaaaacataatattgttaatttggt
tgacaaaaataagctctgttcagagcattcttcactagatgttgattttgactcaggccaactccactct
gaatgtacagatgaatgcagtgctaatattccaatggtgaagagcattggtgatgaatatgcatgggcca
gggagtgccttgaagacctttaaaagacaacatagaagtagactatttagttactgacccagacagttc
agcttacagagctgcagttgatttgtatgaggagggaaaatcaaaaactgagcctcagcatttcattgat
accaggcatttgtcggaaaatatgcggaaaagtgtgaagaaagacaaaagtctcctaaaaatcatgccgg
cacggacacaaaagcaacgccaaaagctactgaataactttgcaacagacttaactgaaaggtgcaattc
tgaacttgctgtatgtactaagcaatttggaggaaattttaaaagagttaaatcaaagatgtctcataca
gttgatgcagttatccggtgctacatggcaaaacatgacttatgcaagaaatattcttttgtatgtggca
agagtgagtggctcaagaggagaagtctactaccaaagacattcaaaatcatgcatacccatgaaaacat
ggattttattcgtatcgcagtaaataagcgcctcggaccaaaagcacttgagaagaccaggcttaatatg
aacactaactttgtcgagggtgtgaatcgtagcataaggcgttccttaccttcaaatgtaacgtacaaaa
agaactattctggaagggcacactcagccatccatagtgtaaatcttggtccaggtgaatccatccttga
gttgtgcagtgccttacactgtgacattccagttgggagtacagcttaccaggctctgaaaagtgtgcag
aaattagacattttacaaaagcagcataagcaaacaatggaatacaagaaatatagaagtgaaaaacgtg
aaaaactgtacaaactgtatgaaaaattaagtgaaattattgcgtacgagaaaaacttgctactgaaaag
tgaaaaagaacagaaaccttcttctcgaaacaaacacacagaccattcctacagtgtaaaaagcaagaaa
aggagaatttctgttagaaaatagtttttatattaataaaattacatattaaacaatatggtttgtgtga
ttttgagcagaggccttgacttttgcagtgaattctctcctggttaaactcatgttgttctatggctatg
tggtctggattgacacacagtttctcatggcagatatggctgcactctttgtcattaaaagatttttgaa
agtcatcccttgtgatcctatgccttatcatgtaggaaagcctgtgtgcagtctccctttttgccctctcc
atcaggccatttcacaacttggaccccataacctgacttggacttccgtgcccctatccattcaatgcat
cccttaccattgcccattaaaatagcctttttgaaaaactttttctcaatatcagcccaaaaagcatcca
ttttgaaaaaaagaatgatcaaagtcaccacagagtttgcaggaaagctaagcaccaaaaaacagtgatg
aaggaaaaaagtgaatgtgaaagtacacattctctata
>KolobokP-2_PeVi-I
ttaaggttttggtgtaccttacaaagtkwctcttttgcattttcataaagtatagctcattccgaataaa
acaagcctcamacttgawtgtacaaggctgcattccaactcaatgccatttttaaaaagtatggtgttgt
wgaagaaagttaagattgtaacgtcaaataatttacaaacgttactaaattctataagkgtattcaactg
tttttttatttttgataaaacaaaagctttattccaaatacttattttaattgtatttgatcagacat
ggtggcatggtcattacaagtataaaaaagtgcatattttgcaaaaatatagaaaaaatgccattttgag
waaatttcttatacgaaaaatgsacctaataatwagctaaatttattctacatcataatcaatcattcag
taccaacatacatatatatatactctgtcatgctacttaaccttaagcaatgatagtggtcaaagggcag
ataactctwattactacagaatagtattattattwttwtttttaacaatcagacttttgcgagagaaaa
aaaacatggtattttattcctgcgttkgccttgttaaaacattgttaaatatgaataaaattaactgacc

aataatataattataaaaaaataatgcactcctgctaaatgccacaattatgacgtcataatgcatgacg
ttagaaacacagtggatgcaaggatgcccagatcaagaatttctcacgactttacaaaatttagaaaacc
tggttcaaggaaaagaagctgtttttatacaagtgacaaccwaccttcaaaaaagttgaagtcttctmaa
acagatgaacaggaagatttccaatgcgcagattttccacagctcawaacacgtcttccatcamatacaa
ccaatttagtccatacagataagagatcgactgtaattccgttcaaactccgtccgtatcagcaacaacc
taaaaaaaasaagaaaaaactaaaagacagaaggaaacatttgatacacatgaatatggtattgtaaaat
tctctaagattagtgatttattcaataattttatagttcaacatacggagaccggttgcatgaaaccagt
aatwgaaatatttwaaaaaatcccatcttggaatttgcatatcagcatatatacaatgcaaataktgcat
gttcgaatcaacaccggttgacckttttgacagatcaaatwcagattccaaccatggatatccatccggt
agtttaaatgatgccmtaggkctggcatgtttaaaaacaaaaatgggaatgacwgatatacagtttatat
tatcgcttttgtctatwaaaccaccttcaataactatgttacaaaggaaagtcaacaaaatttcagatgt
aatgataaaaattaatgaagaatcaatggttgaaaaccaaaagattgtaaaggaaataaatgcattaaaa
tcatcaaattcaagctcaatttcagttgagwcagatgttagttataatwaccggccccgtcttggatacg
amgcaggaacacaagcsttctcgscaatgatagaaaatataacaacaaaaaattaaccctctcattggat
tatgtcaacaaattatgcagtcacaaagttaaaaactgtacgaaggccaactgccgaaaaaactatccgg
accaaaaaactgtggcctcatcagagggatatatgctacaacataatttagaaaaaattgagaatactgg
aattgtatcagttgacacggtcacaagcgatgcttgtaatcagatcaacaaagtctgtaaattcaacaga
aaaccagtacgccatttaacttgctttttacataaaatgagaacatttcaaaaacatatacatttcatga
aattagaaacatcatcaaacaaatttggtttagaaagttcacagtatataaaacaactagctttagcatt
tcgtaagcggatacagaaggaattaggacgcattaaaagaaaatgtaaacaagagtcagaattcgtaaaa
ttttcttcaatatgtataagaaatattatcaattgttttaccaacaaccatagtatgtgtggaacattgt
ccactgtatgtcaagcatacaaggagtcttttcgaccaacatttttaccttgcggtaaatatttaaattt
atgtcagacggatattaataaaattaccgcccatcttagcaaggatttttcacatgctaatttacatgaa
ctatacggattaacaaatactaacaaatctgaaagcatgcataaccgccttatgacttatatcacaaaac
gagtgacatggagtcgaaattttggagctatgtgccattcagcagttcactccgatacacatggcacagg
ccattcaacattaataattgcaaaagaaattggaatatcttactgtgaagacagtccattttatgatcac
atgatcaatattgatactagaaatattttccataaagaacgacaaaaaagtgaactttatcgacaggakc
gatactttaggaaattacaccaagtcaatcgaaagctcttagagaactctatgtacaatacgccatcggt
atcaactgaactttataactttgcaataaaaccaaaataaaaaaattcaaatagtaacattctgtctttt
agcatacttgccttatattattcatataatacagtgcttgtagccttgatgcccaaaacatttattttct
tcataacataaattcctctgacaattaatggcattaggttcatagcttaaatgttcaatattaatgcaca
gcttctcatgacacaagtggctcacatgcattgaagagtttaatggttctgcattatttaataaataaaa
agtgagacgatgtactctaaggcgtatcctcctgcctcgaaaggttactcttaaagatgggtatcctccc
atcacttcaccacggtaaatgaaacagtttctgtcatcgacagtataaaaattgtcaaatttactgaaaa
gaaatgactgttcttcagaactaattttgtcgttaaaaatgacactcatggctacaagatagtattatat
aactggaaactctgtaatattttatgtaagaatggagtaacatgacaaatacaatacttcaaaaaaaaaa
caaaggcaaaaaaatggaatctgccagacccttactgtgtaaaatgctttacatacagcacaaactaagc
atacatttgtagtttatttaataattttaatacttttggagcagaactgaacttctacacaaacttacta
ccttgccagtcatctgaatgggaagatattaacacaaaaaaacccataagtggcttaaaatttgcaacaa
agtcagttatagtgaatttcagctggtgacgtttat
>KolobokP-3_PeVi-I
tattagatgcgctaaagttaaaatattgtaataaaaaaccatcttctgttatttcatcatatttccaatc
attctgcaaagtttcgatcaaatcggtccattatcatgtccacaaaccagcttcaaagacagctacccaa
aactgttttcagctcttctttcttatctgaaaaattcaaaaagttagatatgaccacttttatcaatatt
gcaataactttttttactttttgagatattttcacaaactaaagtgtttttttctcttggataattatagt
atttgaattacaaattttcttttatcaaaatagttttaaagtggtttaattttttttttcaaattccaaat
atggaaaaaaaatagaattgaatcaaatgaagctaaaattaaaaatacttctacttcataaaaaaaaataga
aaaaaaatattataaattactttaatgattcagaaaacagccccaatttacagattcttgataagttgaa
aagtatttgtgtaaataattattttctacaggtgccgatatgaccaataatctaaaggctcagggttgca
tactcatacataaagttcttcagataagaaaatttttgaataattacacaggtattttaggaaattttca
tcacagcaagtttttttcttcacattttaaagaaaacaaaattgatttatcacatttgggtctcaaagaa
cgaaaattataatatagtggtgcaaaaatggtcaatttcatttttttttttttctaaaggcattttatttt
ttttactagtatctacttaaaaastacaaaaacaatcccaaatcaaatatttgttatcaataatacggaa
ttaaaccaccatattggctttcttttgatgattctttgaaaaccccccatcactcagggggcgatctagt
atcctattaggatcaaattatctgatctaatgagtaaaagactgcaccctgagccttattagaactaagt
aaacattgtcagcatctagaaattctcacctaaactatcaaaagaaccaaaaagtataaatagtagccct
tttgagactaaaatcataccaaattttgttgcggaaccatacaagaagaagattcaaatatgtttactga
tggtaatactccacacaacaaaggcaaaactggtctagcaggaagccacaaggttagtaatgctggacgg
ttcaagaaaggtcatactccatggtcgaaaggtctcgtttttgagaatgcagtttcatcagatgaagaag
agtcaaaagcttacatccgaatgaatctgcaagatcaccaacaagacgacatcaaagctgattccgtatc
caggactgacagctcacaggtggtaaaagaagatggaagtggtgtgattaaatccagtatgatcctgcgg
ccacggttcctgaaagcagacaaagaaaaggtgacatcaaataattctcatattattgagagtgaaaaga
tgtttgacatgttcaacttggcgtaccaacagcacagggaaacaccaaaaacttgcaagaggccatggat
ggagccacatagtgacagaaaatggggtgcctgtatcatgtacagagttcggtgtgcaaactgttggttt
gcgactccaacactagcaaagttgtactcagaagttgaaaacaacaagccagggccaaatccagccggta
ctaacattgcactagtttcagcaggactagatagtccaataggaagcaccaagacccaggagattttagc
gcgtgtaagtgttccacctccatgcaagagtggtatgcagagaaacaccgaccgaagttatggtaaagca
gaacaaggacgacatgaaagataaggttgaacaagtgaaggcatacaacaaaaagaagggacaaccagaa
aatgtgatagatgtgaccttagatggcaggtacccaactgcaacaaggtccagcaggaagaaaccagggt
taaatgcatcacaggcacagatgcttgcgattgaacaaaatactccatggaagtttgtagtcgcagcatc
ttcactaaaccagctatgttggaagggagcttggctcagaggaaagggatttgatgcagagtgcccaggc
catgaaggctgtactgctacccttcatagaactataccaatatcagaatatgccatgggaaaagaaattg
gaaatcagcttgcactgcaaggtatgcttatccgttatgccacaacagatggagatgcacgttccgctaa
aggagtagaagatgccatacatgtactgtatcctatgtgggaagtgcagagactggctgacccagtacat
ctaagccaacatcaattccgagcgggaatgcgagctgagtttagtgagaccatgtttcatggacaaacta
aagaagagaacaaagacttgaagaaaatgtttagcctggacttgaagtgtcgcagttccatgatactgaa
ccagttaatgaatgaatataacaaggacacagaaaaagtwtgtaaaaaattacctaccatcctttcaact

acagtccagtgttatggtggagactgttcatcttgccttaaggagtctgtggtatgcaaaggtacagtca
gtgataactggtggaccagatccttttacctaagcacttacaacatcactgtgctgaaaatagatgaaat
tgatgaaattatactgctagaactactgaaaatgatacttagtgaggaagcagtcaatagcatgagaatg
ttccacacaacaaataaaaatgaaggagttcatcgtgcattgagtgtgagtttaccaaaaaatgtaactc
atggaaaaaacatggaaggcagacttcattctgcaatacacaggaccaacaacagacctggtacatcagc
caagatgaaaagcaaagagttgaacattaaagtgtctgaagactgtgagaccttctttgataaaatggac
agagagtatacttacaaaaggacttatgagcagaaacctgaagtaaagacaaggaggttacaacaggcag
ctgtcagactggaggaacacaagattcataaagagctccacaaaaagagaccagactatgtaaaagggca
actagatcctgtaccagatgagctgatgcatgatgcatactgcaagaagaagatgcctaagtaaaagaca
gacagcttaaaaaaatcaaaggtccttttaaggaccacccaaatctttcaaaaagatttcttatattgat
acgaataaaatgaaatttaatgaataaatgaacactttcccctgcccattttcttttaaatccattaaaa
tacctaaattattatctggcatcttaaggtgcactaaacagggcggaaaaggggcaatgtgacctataca
ttcccctttcgacacacacgtttgcctactgttgttgaaaccatgtggttccaacaccagatgggctaca
tttatgcatttggtattgtggcacaggtgtgatacatcaacagctgctaactgtgcagccctatgctgta
tcatgtatgcaagcctgtgcacatataataatttccacccaatggcaccaggaaatctacaatttatgac
cccatatttgccatttggtgtacatgaccctgtccaaataaaacactctccatttagcccaagggcacat
ttttcctctagcttttgatggtacttctgaaaaaaggcatccatttgtaaagttactttttcaagcaag
attgagtaggaatatttctcaaaaagaggatttggtacccctcaaaaccaaaaaaaaagtccaaatttga
gatatccaacccctatcttttgaaaaatg
>KolobokP-1_TeGr-I
ataaaactgaggcaacattttaaacatcctttttttttgttgataatatgaaaaaagacattatttcaaac
aaaatgatatatcacatgatgtatctgttcatgttaactgtgtttaggagcctatttacattgcatgtct
gacctggtaattttttgcatgtttttgacaaaagcttatacttgaaattatcaactttacacatgcagtgc
atatgtttgcaatgaaaatgaacacaaaatatcatcaaaaattggaaagagatgggcagaaactgattca
gttcatctgtttatattctcaaataacaatcacagtgacttttctctttgaaaagatcttattaagtaaa
aagtatgtaaaattataagattgcattatgtatttgacactgttcaaaatgcttagttatagtaaacatt
aattaaaggtaaaaaaccatggcaaatcttaactacaagtcattactgattactaaaaataatgaaattt
acatgaaattttcatcattattagttaatatgcatttcaattcatttcatttctactacagtaaggggag
ataatgtttgtcaaccacagcttcaaatctgatgacatcataaaacaacatgatgtcatcagcttttgtg
taatgcacaaaactgtgttcttttcaaaattatgtcatcaatttttgtttttacttgaaataaacattta
taaacatttcgccttgtcaaaaccagtagcagcaactgcagtgatgccaaaaaaaagtaaacattggtat
tatttcaagattaatcaatttaagggtactagaagacaccagtttaaaataggtaacaaattatggaatc
gacgtaccccaaataatcactcggttgtgattcctgataatgttgaacccagttcttcaagcaaccaatg
ggttcgcccaacccaatccctatatgaagctgcaatgtacccaggcaatagctgtgcaccaaccaaactt
cgaccaattcctgaaatgaaagagactgaaaatcttcagagatttgaacccaactctcttctaggaaatg
gaataatacaatttaacaaactcccccaacttttcaataattttgtaagctttcacagtcacaatactga
atgtcaaaaaatgcacatcgacattttcaaggttgttcgcaaaggcctatgtatagaattgcaaatttac
tgtgtaacatgtggcataaaatcagagtcgattaaaatgtatgatgaggtccataagaaactcagctcat
atggcccaaaacctggctcactcaatatagcactgtgcttgtccattctaaaaacaaaagttggcataac
tgatttacaatttatcatgtcttgtcttgacataaagccacccaccaggtcttctatgcaacggttagtt
aaccaattgtcaaataaagtggcaagaatgaacagggattctatgctagacaaccaaaagtttgtaaaac
ttgttacagaaataagaggtgaccccccctacagtagatgttgaaacagacgtgtcatacaacaacaggct
tgctttgggttttcaaagtggtacccaagcattctgtcctttagtggaaaatacaacaaacaaaaaactt
gtcctaaactgccaaatagcaaacaaactaaactcatgcacacaaaatgttgcaccaaacacagttctta
caacacatgaaaaaactatgacaattcgcaatgttgaggaagtgaatgccaacaactttgtaaccgtgtc
aagtataactactgatgggtgcccccagatggatagtgtggcaagtgcaataggtgttgcatgtggtaag
cccccattaagacattttacatgttttgttcacaagatgcgcagagtttataaaaacattactagccttc
ctttgtcactacctggatatacgactgaaagaagagccacatttaagaaaaaattggcaatagcaacacg
caacaggctgactagagaactgactaggttaaaaaataatgtctctgtagaaaggcaatttgttgaacaa
gcagttaggataagagaaaatgtacttgattgtttcagtggcaaccacagtcagtgcaagaagatttcat
taatatgcaagccacacttgccaccaatacaccttccatttggcaggtggctcaacctctgtgattcaga
taaagacagatatacttgcacccttagaaaaatcttaaataacaaaaaactaagcgaaatgacacgtctg
ttcaacaccaacaggtctgaaagcatacaccacagggtgttcacatatgcaaccaaacaagtaacttgga
gaagaaatttccctggattgtgccattcagctatacattctgacacccatggcactgggaagtcaaccgt
gttaattggtaaagatgttggtctaaactttcacattcaaggccccccttatgacccaaatggttaaagtg
gacacagttgctaagtaccagtcaagatacaaagcaatgctagcaaacaagatcaaacgatattatgcac
gttatcgaaataaccaccaatccattttaaataactcaatgtatactgctaatggcacaaatcagggaaa
tgagcacaactatgcaattaacttaacataacttaacataacataacataacattcagaacagtgttttc
aataccttacagcttgcagttttcatatttactatgcccaaaacattgcctttcacttacacaaattttt
ctagcattatttacatgggctggctcataggacaaatgttcaaagtacacacaagctttattgtggcaca
gatgactaatgtgcatatctggtgacaaagtatctagcactgctaaaaagtaaactaagcgtggtacacg
taaagtgtaccgcctttgcctaaatgtacttttcaacagtgggtatccataacgatcaaactgcccaccc
caaattatgcactccgtgtcattatgtgttataactttttttcataattagcatatttatatggtctcttt
cagaataagtaatgttcagcttgaaaaaagccatgttttgggaagaaatatataaactaatatataaaat
tctgaaattataagcatattaagcaattacattggtctatataatatatataacatttttatatatataa
aaaaaaaaaaaaaaaaaaaaaaaaaaataataataataataatttaaaggggaaaagggaaataataa
tgtggaggaagtggaggcaaagagggggggaatgggggaaattcatcataaatttttcaatggtgtcaac
tttgcaacaaatgtcttgcatttttttacataaaggggaatgatgtaactcagttgttgctatggagatg
gcagggtgaaatttatggtgattgggggtgggaggggggggtcaaatttcatttcaataagaaaatgttc
aactttaaacacttataaataattaacatacttataaataataaacttgatgatttccaatgaaatcaaa
tacattaagataaaaaaatccatttgaattgcaggaaatgttggtgtaatgatcacagtgtatatattga
cctttgtgcactaaaatgaccttatgacctcaaagttgtaaacattagtcaaatatagtttttaagcagt
attataatgcttacattcatggttcacaacaaaaatatcataaaacatccattttaatcatttctgggtc
attttcaaaaaatattactgtctgcttat
>KolobokP-2_TeGr-I
tgtcagattttcagttttcaaccgaatcaattcaaattttcacacgttatttagatgtgtttatacattt
gttaatgaaaatttcaagtgattctattgtcaaatgactttttttatagtctgttttccacaccacctgta

aactgactaagatagaaaaattggttcaaatctcactaatggggctaaattgatattgggcaaaaaatta
atactttttccaacaaaccaacttgatttatgtgtgatctttagcacaaataaagtctcttttattcaca
ccattcaaattctgaatttgtacaatttcatattttcacttatttttaataatttttaatattttttaa
atattaattttttttttcaaactttaaccaatcatatctcaaaactgaaatgactgtgcctgtcaaatga
attaacatacagtagcccagtcttaagtgtcttgacttgtatattacataacattcctctagctgtccaa
ttaacaacagctgagtctagacagtcaaggggacaaaactctaccttgacacatgccagtgtccaggtta
tgtatattggctctgacttagccatgaagcagaggatggcagttggtgtgtgacaatttacaggagatac
ctatggcttactgacaatacgcaaccacaacagttaaatagaggaaatgtggggaggctggtcattcaag
ttttgagactgcaacatgaagggtgataaagggcgtaaagttacacagtttaagaagaaccagactggtc
ctaatagaagatacatagcaaacctagatgtcatggagactgattctgaaccagttccaagtacatcatc
tggatccactgggtggaaaaggctatcttcagatatgtactccatggtaaccaaaacaggattggatggt
gaatgtgttactgttccagactgtgaaggaaacactggtactgctaagatactgcgacccaagtcttctt
cctgtgatcctgacctgaaacagatgaaaaaagaagatgagataaaaggcatgagacttgtagataatga
agaattactaaagatgatgaatgatgtctacagataccatacagatgaagcaagtgactgtagcaaccca
gatttcaccatagccaagcagaccaaatggggaatatgctggaagtacacactgatgtgtaaaacttgta
agttccagtcagctgagtacaaactctacaaggaaatcaagacaaacctaccagggagcaacccagctgc
accaaactattccttagctattggacttcaagacacaccaataggaaacactaaggccagactactactt
gcttcaatggatgtaccacccccagccatacaaagcatgcagactacatcaaacaaagttagcagggcca
caacagaattgaaccagagggacatgtcagcaaagattcaacaagtgaaagaaatcaatgtggaacgagg
agttgaagatccgtctcaactaaacatttcaatggatgtaagatacaactctatacagatcacaagtaga
agaaaaccaggacaaaatgcatctcaagccattggactagcagtagagacaaccagtgacaagaagtaca
tcattggtgcagctatacagaataaactgtgctggaagggacaatggttgaggaatagggatatgaagt
tacctgtccggatggccatgaagactgtacatcaaacttatcaaagccagttccactctcagaatatgaa
ataggaaaaacacttgctgaacagtttttaatggatgaaatccacatcaagtttgccacaacagattcag
atggcaggggagcagagggactaaatgatgtattcagtgaagtaaatcaagactggagtgtggagagact
tgctgatccaacacatgtggcacaaggacagttcagaaaatgtcaatcagctaaatttagtgaccagatg
tttgcctggaaaagaacaaaggcaggacagagagaagcacagttagaccttagtaaggatgtaaaatcaa
gatgcagtatgatttataagaagatgatgcatgattatgcaggcaacatgactgaaataaagaagaagtt
accaaaagtaatggaagccactttaagatgctatgcaggggactgttctatgtgtagaagacatgccata
gtatgtgaaggaggagaccaaaagaactggtggaatagatcttttcacttggcaaccagcaaaatcaatc
aactaaatatgaccgacaaagataaagacctgatgttggagattttaaagatgcggttgagtgaggaggc
cttcatggacatgagattgaacacagacactcaaaaaaatgaagcagttaacagatcactcagtgtttca
ttgccaaagaatgtgaacttttcaagaaacctaccaggtagagtggcttcaaccatccacagagtcaata
acacaacaggaaaatcaacaattgaaaaatgtaccaatgttggagtggacctctcaccattagccaaaca
ctcacttatacagatggaaaaagcagaggaatacaagagagagtacaacaaaagacctgaggtgaaaaac
agaagaataaaacaacttgggagagacatccatgcccacaagagatataaagaatctggtggaattgaaa
cagactacaagaaaggacagctagatcatacctactccagatgaaagacaagacaagacaagcaaggcaa
ggcaagacaaggcaagacaagacaagacaacagaacaccaaaccaaacaaaaaccgcccttataagggcg
atccatgttcttcgaaaagatctaagttgtaatcatatcataagggagggtgggaaaaccagacggttga
acggacggaaggacagacggacmgacggacagacggacggacggacagacagacagacagacaggtgttt
atacaaacttacctcaggaaatgtgaaggtcaaggcgacaatctggctccccactgttgtctctggtaca
atatccaaggttcttgcacttcagtctgtaattatttacaatatgcctctctaaattgatgtgatctgga
ttaatacatctacaattatggcatagatgggaacagtcaaacccccctgggcagatctacaaaatggtggt
gcaccatgtaagataatctgctgggcctgcaatggttgacccatctccccgtgcctgggtgtttaaaaca
aacattcccatatttatgcaaggggtcaaggtcaacccacaatctacattctccattcggtgctggaatg
gaattttcagtgagttttctttgatatttttgaacaaatgcctccattttaacttttcatttgaaaatga
ggctgaaaggggggccccagggataccccatcctaaaaaaaattgaaaaaaattaaatttaaataaattta
aatattttaaaaatattaaaaatttaaatttttaaaaaagtaaataataatattaaagttcattgtctag
tgcacatattgaccaaatttcagggttctacatgaacaaacaagaaagatatggcccctcattgacaggt
acccccccccccgcatctgaca
>KolobokP-1_SiCo-I
tatttagccagtatttttcattcaaatttggtaaaactttgcatacattatcccaaacatatatgtagaa
gacctggaaagtttaaagtcattatcttgtaaactgatgacatgacagaatattatgtaccataggtcta
tttacctttgtcgccatgtgtcggctcattgaagtagaaatgcaccaaaactaaaaatgctgtaactttc
ttaaaagtaaagatttttaatcaaataaaaagatttttgctgctctgttcaatatttacttgaaacaaa
atgtaattttcacatagatgaccaagttgaatttaaaaaaaaaagtggactaaatatcataaaaattagtc
atattacataacattgtaactgaattatataatttttaaagatttcagttttgtcaaattcattacttta
taaaaggcatacatttaacctttaaaatgatgggttgcattttatcttaagtcatatggtttaaaagata
tggccaccagacaggactgccactgtgtccaggaacacaggtggctggctggccgcctggctgcacagtg
gggggtggggtgcttagtgcaggttcactgcacatgcttctgcatcaaaaacatatgccagcatatggga
ggtctgtttttttcagtttaaaagacttcaaacagatatctggattcccaaactgaaaattttcaatataa
aaggggccccagttcctgatttcatcacaagaagattaatcaccttcatgctattaacaactgctgtcc
aagtatgaggggcgatcgaaagaagaaggcgttcggtttcaagaagggatgctcaccttggaataaaggc
ttgaagtctgacagtcatttccatgttgatgctgaacctaagagatctgtcatcaggctgactgaagatg
aatttccagtgtctaccagacctactgcagatggaagcgagctccgcggagatgtcatgctgctgcggcc
aagaacatcaagaagtcaacctggagcaaaacaagaagaaaacagtaactgtcacggcatgagacttgtt
gacaagcaagagatggcgagttcaataaatgtggcacttgaaagtcatcggcagaaatcgactgaatgcc
atcagcctaatctgattgtggccgatgaaagaaagatgggactgtgctggaagatgtcattcaagtgcaa
caactgcagctttattacaccattattgaagctatacaaggaggcagagtcatctaagaggggaccaaag
ccagcaacaacaaatctgagtcttgctgtaggactacaagatgcacctatggggaactcaaaattccggt
acatactagcaagtatggacattccaccgccagctaaaagtagcatgcagaaaacatccaacactgtggg
cagtgcaatcaaagaactgaacgaccgagacatgtcaaagaagatttgcctggtgaaggatatcagcaga
aaacgaggaaatcctgggaactctattaatattgctgtggacgggcgatacaattcgagtgttatctcga
gccggaagaagtcaggccagaatgcatcacagtctatcgcagtagcctgtgagacaatgacagaaaggca
gtatatcttggctgcagcagtgcaaaataagctttgctggacaggtgcctggctgcggggaaaaggatac
catgtggaatgccccggcggtcatgcaggctgtacagcaaatacatatcgacaggcaccactctcagaat
atgagctcggaaaatctattggccagacactagcagctgaaagtgctcttgttcggtatgtgacaacaga

tggcgactccagatcagctgccggtgtgactgaggccatgcgattactcgatcctatgtggaaagtagaa
cgccaggcagacccaattcatctcggtcaggctcagttccggtactgttacaatgctaactttagtaacg
gtatgttcccaggcgcaaagaccagagaccaaagacaggagcaacagaagggtctcagccaggacattaa
ggctagatgcagctgtatcctgaaggaaatgataaagagacatgctggagacatgacatttataaaaaaa
acagttaccccatgttcttgatgccacagtgaactgttatgccggggactgctccaagtgcagacgctac
tcatacgtatgttccggtggtgtaacaaacaactggtggaaccggtctgttttcctgggcagttacagga
tcacatctctcaatctagacaaaaatgacaagaaactgttaaatgacattgtaaaaatgagaataggtgt
aagtgctatagaagaagttaaatctggtaccagcacacagaaatgtgaggcagtgaaccgcgccatcagt
gtctccctgcccaaaaacaatgacttttctagaaacattcatggacggctgagctctacaattcacaggc
taaataacggtttggcggacagcttggtcgcgaagctggatcgagtcggagtacgtctttccaagaagac
ctcacaatccctacaccaaatgcaacgggaagagacgtaccagagacattattcaaagaaacctgacacc
attaagcgcaagctacaatcgaaaggtagacaattgcaggaacatttaaaattcaagaaaacccacactg
tacagtcagattacagaagaggccagctagaccctaaaccaagcacatcaaagggagacaacagtgacca
tgcttactcacaactcaactgagttaactctcattataaacagtgttgatgtgttaaataaaatgtcagt
gacatcataatataactctacctatcaaattagttttcttagcagtgagagacaatatacatgtatttaa
gtgtgacttcatgtgggttctatatatatttcaatactatttcaatttgcatacatgcccttcaattgaa
cttgttttaatttgctaccatttctgtggctggttttgattaaataaatgttactatgaatttatgcact
gtttttattcatttttacaatagtaaagtgttttcagtactgtttaacaagttctcatggtctatggctg
ttgacgagcaattatttcacgctatgcacatgtgaactaactcatcgtactgaaaatgcactgctattgt
tcataatataaatcaattatagtgccaacatactgactaaaaataaaataaacaacaacaaaaagacaat
acaataactagcacacacaatttgttcattaaaagacaaatacatctttatgaaccttattaaattaata
aataaataaataaagtaaataataaaaaaccttctgtcgacatgtatgacatctcgagatgtcttttcaa
caaaccaaatactaaccttgcttaaataaacagtctggatagactatgtggcctgagcatgtgccacggt
ttgtacatttaagcctatctgtgttgacagtatgaggttcgaggctgagatgttccgcgcggacacaaag
tgccacatgacacagatgcgaaacttctagacctggttctacactcaatttttatggtgaaccaggtaa
agaagtttgtgcacatgcatggtgtgccactttgagtcaggaaatttagcctttattacaccatatccca
ctggcccctttttaacacaaccctgccaaatggcacaaccatgactgttgggcagagacttttcagttat
ctttgtcatatatttttcaaaaaatgcctccattatgatttgaaagtgacagtgaggctttgacctacac
aggaaatgacctcttggctatttttagatgttattttatactgaaagtagatgttttcatttttttttcaa
aaatagtatatattattatataatattatataatataatgtaataacatcttaaaggttgacatttaatt
ataaattctgattttacattcttcatctaccttagggtcaagtttgaacaaaattgttcaagaaataaa
aaagttattaccattcaaacaccatgacccccagtcaatggatgctcctaatca
>KolobokP-2_SiCo-I
tttaaggcaaactttttttacattttctgtttgaaaactatatatacatttaggttattgtattctgaac
attatctccaaattttagtaaattctgaccagaaatagtgacaaaaacccatttttttccagacccatcta
tactgggcctgagggcagaaaatataatgaaaatgtcagtaaaaagttgatggtcatatatgttcttctt
ttctcacagaaacactagtgttcttgcagggatggcactttttttgtggcaaaatgatccagtaagactgg
agtaactaacaatgggtgttatgatgtacctttggttaattttgggtgaaaaaatcatgtttttatgtaa
aaaaaatagaaaaaaatgaaaaaaaaattagaaaaaaaatgtaaaaaaatcattttctatgactgttattt
ttagtttaaagctttccattcatagtagaaacatctgccagattaagcatggggtcctgttaataacact
tactagttggggtctaattgagagccaccagctccatcaggtgtccttaattaacaaatgcatatcaatc
accttatggctggtcctggccagtggcttccacttgttgagccctatataggtttactgcatttgacaat
ctatcatggcatccctgatgctagatccagaggaggtgattgtatttccttcaatgttgtgattctaaat
agaatttaactgcattttgtgtcaaaaatggtgctttcaaggcagagggcgcataaccgcagagtaggat
tcaccaagggccacaagagttgcttaaaaggtaaatcgtttcattttgccaagccggaaactccagttcc
atacgtaagattgccagaaaatgaatttgctgatcgaatttattgccacgatggtgttctaacctttaga
gacacagacgggcgatcgacaaatgttaagccactgagaccccggccggtccctgttgattctgttgacc
aggtcctttgcgacacgtgtcccgactcccccctttcctgtctctaattgccacaaggtatatgatttgga
taaggtgtcacatctgtatgactctggaaacagaaaacatgggttgtgtagcaaaggttgtgacggaagg
cttttgattgatagtgacaattccaaaaagtggggtctcgtatggagcgagcggttaaaatgcaccaagt
gttctttcgtaagtgactattgcaaactgtacgaagaggttgaaggaaaatctaaacgaggcagaaaagc
tgcaacggttaatgttggcttgcagttggggctgagcacaaccccaatcagtaacactggtatatgccgc
gttttaaacaacgcaaacattgttgcccccagcataagctctatgcagaggcttagtaacaaggtgctag
aggaagtaatttcactcaaccaaattgatatgcaagacaaagtaagggctgtagttaagacaaataccct
gattggtcaaaccaacccccctaaacattaatgttgaaggtgatacttgctacaataacaaaatctttaat
gcggacacgactccttttcaagccggaacaatagccaccactaccttctgcgaaaacaacaccaggtcta
aaaggataatcggtgtgaatattgccagcaaactgtgcttggtggcctcacgcatgcgcaacaaaggtga
agaggttacatgtccccatcatgccggttactgtacagccgacataagcgagagctctgttataggtaat
gagggcacatggtcagaaaaggtggccaaaaatctgaccggtatcaatattgcaggttatactggtgatg
gtgactcgacgagtcatgccggtgtgcagaaggcacaaccgtgtagggtaaacatgttcaaagatgttcg
acatcttggaaactcagttcgtcgagctgtctatagcaccaaattcagctcatcaatgttcagtggtccc
tcaagatgtaacctgaagaacaggtttgccctgtcgattaagcaaaggtgtcatgcagagctcaagcagg
cccataagaaatacaatggtcatttgcccacaattaagttcaaaatgcccatggttatcgacacaattgt
gttgtgtttcaatggttactgcggtcaatcttgcaaagacaacagcctcgtctgctcgggtaactatcga
ctagcaaaaaactttatgccagctaatgtaaaggttagaatgacagaaaaagaccaagacctgcttaaac
agtgcataggcattatgctgggaattgaggcccttgaaaaaactaaactcttgacatccacccaaaaatg
tgaggctgttaaccggtcgtaccaggcagttaatcccaagatggtaacgaacccaagaacatttaaggga
agaatacatgggcagatccataaattgaacaatgggtacactaggtcaatggtcatgaaagccaggtatc
ttggcgccccaataaaaaaccctgcaataatccgtcaaatgatggctgtcgataagcgagcagcgcgcac
caaagatcctggggccatcgagcgcagaaaggtctgtcggtattccgccagcaggcgtcggtacaacctg
catgagaaagaacattacagcccaggtttaacagatggaaaaccagctgctagcagttctaaacacttga
aagatcacaattatgtgtaggtggccctttcaagtgacaagaaatggagtaatttcaatgtaatatagcc
tgtatatagtttgttttgattttgtgcatgccttatattgtcataatttctcaggttttgctgcaaatgc
tcatgtttttttaactaaataatgttcagactgatggaaaaaacaataaacagtacttacttttgagggtt
tttgctcttaatagactagtttcaaatcaaatgatacattctggcaaccctacatgtccactgcactttt
tcacttttgcacacacttttctatcattattaacagacatgggttccaatgacatatggtggacattaca
acaaagacttttgtggcataaatgtgacaaaaataaggaacttgttaaaaaagtattgtatgtaaactga

tatatgaccctatgcacataatggttgcgagaggggtatccggtgggcattttacccgtatccttccat
atttacatacaaaacctccactagtggccccagtccatagcaaacaacctttattattatcaaaaatgca
cctggacttgattctatccaatagttttcttctataagcttcctggtttgattctgacatgttaacaact
ttatttgcagacaacaggatgtgaggggttttccgttaaa
>KolobokP-3_SiCo-I
tatttaaccaaagatttgtcttcaaatcataaaaataattcatctacaccatgttcaatgtatgttgagc
aaatgtgccaagtttgagcaagaatgcatttgaaacaaattaatgacagtctatgttttagagcatgtgt
gtgcagtcgtttcaatggcaaatggccgacccgttttgtaaaaatccaaaacgcaaaatgtaataacttt
tttatttatgggaatttttttcatgaaacaaaaagctctgaaatgtactcaaagagacctaccagaaaata
tgttaaaataatcagtttggcattaaacttaatttttttttaatttttacaagtttaagttaaaaaaat
ataaaaaatatacaatatttaacttaattcaaaaagtatctttaacctcaacctgtcaaatttactcaat
gttttaagaacattttaagaccattaaaatgatgtatcacttagaagttttgcaaaattctgtgcagaga
tatgacagtaaaaggaatgccactgtatgtccccggccgaacaaatgtataaaatggccaaaactagaat
tccaatatctttcttattttcatgaatttttaaccaaataaaaaaatagtttcttctagtaataggtaaa
aaatagaaaatcccaccaaaaactaagatttcctacaattaaagtatttacatgttttctttattttta
tttcaataatttattaaaaaatagtgtatatgcagcttacagttatttatgaaacaaattatttactgaa
aaaatcttctttaaccagaaataagatgggcctcacttaacagaacacagtaatttaatccagaggtggc
atatggctgtcacaacagtttcttgagtggacaaaaatccacaagaggtccacatgtggcataatttgca
taattatttgcataatgaagcaacccaagagactgcctcccagcagctgtggatttctttgtatgccaaa
acaggtataaaactggagttacatacagcattattttaaaacaamtcttactctctgmttgttcacatca
cagcttatgaacagcaatgaaaggcgacaaggcaaggaagtccaccatgttcaaggcacgtcatgttcca
tggaatamaggcttgaagatggccagttccagctctgaaggtwccaggcagcaacctattattacwacaa
gattgactgcagatgagttctccatggtatccaaacccagtgcagatggaagaggcttcactactccaga
ctgtgagggcaactctggcaatatccgtctgctgcgaccaaagtccgagaagacatctgacctggcgtcg
aaagaagacaatgaatcaattaccggcatgagatttatcgacatagaaaagaactcagctgtattcaacg
aggtcttccaatgtcaccagcatgaatctaaagactgtatccaggcmaatatgactgttgcagaagagcg
caaagttggcgtctgtgtcaagttttctctaaaatgtacaaattgtgattacatttcaccagtaaagaaa
ctgtacaaggaggcatcctcgtcaaaacgtggtccaaaaccagcagcagccaacawcgcacttgccacag
ctctacaggacacgcctatgagcaacacgaaatgccggtatctgctggcaaatatggatgtaccgccacc
atctcgaacgagtatgcagaggacatcaaaccgagtcagcaaagccatcaagaccctaaatgacagagac
atgtctgcgaaagttcagcatgtaaaggatgtcaacaaaaagcgagggcatgatgagaacataatcaatg
ttggcattgatggacgatacaactcggcatgtataaccagtcggaagaaggcagggcaaaatgcttcaca
gtctataggcattgcctgcgaatcagttactgggaagcagtatataatagcctcagcattacaaaacaag
ctttgctggtctggtgcctggctgagaaaccagggctttaaagtgacatgccccggtggacatgaagact
gcactgccaatattcaccctcaagccccactgtcggagtatgagcttggacgagaaattggggagcagct
tggcgtccaaggtgtcttcattaaatatgcaaccacagatggagattccaggtcagcagctggggtagca
caggcaatgaaagtactggacccaatgtggaaagttgagaggctagcagacccaacacatcttggtcagg
cacagttcaggcagtgctacagtgccaagtttagcagtgaaatgttctcaggcaagactagggaaaagag
acaagaacaacaaagggtcttgagccaagatgttaaagctcggtgcagtcttgttcttaaggaactaatg
aaagttttagctggagacgtaagacgaataaaagtggagttaccgaatgtccttcgtgcaacggtgagct
gctatgatggagattgctcaagctgcaggcgtcattcttatgtgtgctcgggtggggtttcaaacagctg
gtggaccagatccatgtttcttgccactcacagaatcagcaatctgcaaatgaatgacaatgatagacat
ttacttgaggagatacttaaaatgaaattgagtatcagcgcgttggaggagatgaagttgggaaccagta
cacagaaatgcgaagcagttaaccgstccatttcagcgtccctgccaaagaatgtcaacttttcacgaaa
cgtgcatggcagactctcgtcaactattcatcgactgaataatggtctttgcaacagtgcagtcgagaag
ctgggctgtatgggggttaccttgtctccaaagccgcttagatccctgaagcggatgcaacaggaacagg
agtaccagaaaatgtatgctaaaagaccagaaaccactaagagaagacttttatgtaaaggccgtcaact
gcgtgaacatctgcaatacaagaaaataagcaaactgacatctgactacagaaaaggacgcctggatcca
gttccttcaacatctagggcagaaaacaatgaacattcttactctcagtgaacagcagtcatacaacagc
agctacaggtaaaaatccttattgaaaaagctttttccagtacaaatatgacaagttctcatcataagtg
gcaaaacttacaccagtcatacatgcaaactaagccattttaccagaaaagcatgggctgacatgcctac
attttaatacatccacataagatactttaaactgatgtttaagggacttgtgacattttaacgaaagacg
actaacattatttattgatataatcattgacgcgacaggatggcattatatcattaccggtcagttttgc
tggggtagtcgacaaagcgagaatggctcatgccccccccccccacatttacctcggttaaaacacttg
agcctatcggtgttaaccgaatgtggctckaggcttaaatggctgaattcaacacacttgggctcatgac
aaaggtgggaaacctctaaacctttgtctaacaccaatgtctggctacaaactaaatattgtagcctatg
tgcagtagtagtatgccagccatctggaaatttgacattaastacaccatatggcactgggcctttcttt
tggcagccatgccatatcctacaaccgttttcaakaattgtagaattttcatttaattttaagcgatatt
tgttcaaaaagccctccattttaacagtttaattcaaagtgaggctgaacaacttcaaaccttgacctct
tgkttattttttagaccaaaaatgaaagtgacaacagccattttccagatttgaaaaaaatcatatataat
attgtaaaataatatattctatatataataatatacataatataacaatattactctacacactaattac
atggcctcatatctatcacctgtccaattttcaacaaaatatcttcataaatgatgaaaatattgacatt
ttagtttgggcacccccccccacccacattcacattatatca
>KolobokP-1_CySi-I
ttaaagactgcaaacatgttatgcataagcaataaagctgackwttcaaataatttagcctatttccatc
aggaaatcagtcaataaaagcttccgtagtgctttctttctgtcctggaggcaaacgaaaaaggtgccta
aattgcccaaatgctsagaaatgtatggccwtctatccgtgtcatccttaagggatttgacccttctgt
aagacttaccttacaattttcactaaactaacagcactgcactkctttcataagtaccgctgaacagtct
taatccaasaatgtacttactaaacgttgaaataagactccgcgacttcaaaaatgccctcggacacagt
catttcttgctttatttaccttacagacctgaaagtttatgataaatgcctaaaatgttatacttacct
tgcactgaagaatatcaggtattcwwwattttcaaaatgwccaaaatacaacatctgttaagctcaaaga
ctaaaaaactttgataaawaaacatcaactgaacagaaaaagggaggcaactcttgaaggtacctaataa
aaaaagtgcccaagaaggatgattaattawctttctctaccaacagtggtwatatgagactctctctggt
aagcccagtccacctaggtaaaaaacacacacamatttttctctcwwccataaaatagcattcattata
cagctaaaaaaacttatatgcagaaactggcttcctccatccgagcaagagttcgacttgaattacagtc
acttcaagctaggaaaagaaatgaaaatgactacattggtcgctgcatcttagcattagaaaacatagtt
ccttgttttagtgggcagcacactacatgcaaactgaagtccaccacctgccggagtcatttagagacat

acacaaccaaattcttaccatggggtacccatctggagctgaattcaacagacaaaaggctactgacaga
tgaaattttgaaggtttataatacagaagcactgaagaaaacagctatgcacttcaatacaaatatgtgt
gaaagttttcactctacagtatttacatatgcaactaagagagtttgctgggcaagaaactttgctgcta
tgtgccactcagcagctcattcgagaactcttggacgaggcaaggcaacgattcagcttgcaaaagcagc
aagcatcaaagtaccacccaaaagcagaatggcaaaccaactacacagcatggataagagaaaccaatat
cattcaaaaagaaaaagcagtgacaaataccgtgaaaagtcgatattttaacagaaagaaatcatcaaac
cgagctctttatgaaaattctctttacagcactgatgccgtaccatccacttcatcctctgatcacaatt
atggactgcatttgtaaatttttaaatacataatcaacataaacataactgaaaacaagaaataaatcc
gtataaatgaaacttgcaaagtgtttaaacttcataaacaagtaaacatccagtgatagtacctgacaca
gtctacttcagtaagcaacacccgtagccatgatgtccgtgacattctccattaagcacgcatgttttgc
gactattgttaacaaattttgactcatacgacaagtgacttatatttacacaaagctttgtgtgacacaa
atgagatacatgcactgcagacttcagtgcaaattttggatttgctaaatggaaaactaaacggtgaact
ttaagacggagtcktttttgctttwattgtgacacgcaaaaatccataacctaacttatcatgctcaccat
tccaaagaagacaatttccagatggaactattttktcttgaataagattactcagtttgaaatgtaatga
agattctgtatgcgtgttcgcaaaaaaatgcctcggacatattccttkggacactgaaagaagtcataat
tgaatagcagttaaaaactagactgtatatagacattcatatatatatatattattgttcctcttacaca
caaaagaagacttgcmatcttaacawgttagaaaatatatacaattattacatacctgctcgagttgttg
ttctggtaccgaattaccccccttttgttctgagtgacttttagccatgtmtgcatcaaaaaaatcttacg
ctactacctgtatgttgctctaaatgagtaactacgactatccaacacataaacttttcmatcctagcta
cwtagaaaaatgactgtcttaccttaa
>KolobokP-2_CySi-I
ttaaggaaagtttctttttgcatttttttaaaaaccatgcaatcatagaaaacaagttacatttccgcat
aaaaccacaggtcacttttagaaatattccatgaaacgtagtcgcaatagcatctcaaatgtgacagccc
tgatctgaattctgcggcttttatatatatcattataggcaaaatgaacagggataggtacaggaattaa
atacttataacttctacatttaagcagataaaaggctgaaatttggagggcatatcagcaacaccttaac
atttcataaaatgcaatatttaataattttgttactaaattattgccaaatatggtaattgaaatttgaa
acctatgattaaaaaaaaaaaatttttttaaatcatcatttttttttttgttttcatgaattgtgaagtgt
tttattttctctctgcagactcatcagaactgtgtcatagaaaaacataaatcaaacatggcaattagca
ccattaattaccactggaaaggacctagtcttggtcatacctacatcagaatgataaaatgcagatacac
agggctagtttggaagttttctattacacattcagcttttcatcaacattctttgggagttatatctaca
gagaaatatttttagttttattttcattgtgtttttatatacaaagaggaatatttcaataataaaaatgc
caaaaggtgataggcaaagaaggaaaacgcgcttccaaaagggacatgtcccttacaataagggttacaa
gtttgaagaaaaacccaaaaaggattcaataaaggtgaaatatgtaaggccatcatgcagtgaatacaaa
gctgcagaagaaaggcctatattcatcggcgcactggcatctgactgcacagccgacactgatgcaggac
cgtcagaaataacttcactggaaaacaatgtgctttgtaagccattgagaccagtttctgaccagtttgg
ttccactgatacagaagatatgtcacaaaataaccaaaggtaagcaatttgttaaaaaaaaatatgcatg
ccaacccttatcagttgcaataatgaactgatctattaaaagttgcataatatactaaaagaccattaaa
gccccattaaagcaatgaccatggctcatcactgactattttaggagtgaatatttcctaaaagatttaa
agagaataatttcatgtgaaaaaactagaaaaacttgccaaaaaatccataaatattaagtaagatttct
gaaaactaactttttaatagtccaagggagacaactcaaataggtttatccaactatacactttctactg
atttttttatctgtcactctgactgatgcaaagttaaactgaaaattacatctccatggatttctgttct
ttcagtgaaggctatagaacatttcacactggaaaattgtgggaaatgttcaacaaagtgaccgcagagc
attacaatcatgccggaagagtatgtcatggaaaccttggttttgtaaaggaagccgaacaaaaatgggg
atttgggtggagggaaaaagttcagtgtgacctatgcagctattcgtccggcatgttcaatctatatgag
gaagtaaagaatcaaaaagcagggagaaaggcagctacaattaatatgggaataaatatagccatgacac
aaacccctacaggacccactagcataagaaaaattttccatggtggtaatattcctgctccatccagggc
tggtatgcagaaaacagcaagaaaagtctcaaagattctggtccaggcaaatatggatgatatgaaggaa
agacgagaaaaattaaaacggattaagcgtctgcgaaacagtcctgagcacgaaatagccgtccagtcag
acggcatatataataacgcattaaactctgctgcgacaagaaatccatttcagccagcaacacaagtagt
ttacactgtagctgaaaatgagaccctccagcatgagattctggcagtagaaacagttaacaaatgctgc
tccaaatatggctttcatgagcaggaagatgaagagtgtgacatgaagtgcgcaccatgctcttccactg
cttgcatggaagtaaatataggagatgagcaaagatgggccaagtatgcctttgaatcactgaaagagga
tggccttgaggttaaatatataactactgatccagatacatcagcccatagagcattggaagaaatgtac
tctagtggtgtaactaaaacaaagcccgagtaccaagttgacacacggcatttgtcacgaaaccatggga
agtttgttcggcaaagtgagaaagttctacaaatgatgcctgctaggcttaaatctgttcgagtaaagtt
aagagccaggtttgcattagatataagtcatagatgccatgctgaaattcaaaaaattcacttggaagaa
cgaggtatattttcaaaaatttctgcacgcgtagccaagtgtatagatgttatggtgaagtgttacggtg
gggatcacggttgctgtgctgccaattcaacaatgtgccatggtacatcaagtgacaactggctagttaa
gaatgtctatttgcaaagtaattttaaaatagacatttcaaaacatgaagagactctgcgggattgtatc
aactatcgcctcggaccagaaaggcttaagctcacacgactcaataccaacagtcaaaaagtggaaggaa
ctaatcgtgctattaaacgaagcttacctaaagatgtcacgtacactcgtaactttgaaggcagagcaca
cagtgcaattcacagtgtcaacaatggtcctggtcagtcactaatcacacttctcgataaggctggttgt
tcaattcctcggggaggccaggtctgtcgagcacttgccagtgaacaacgtttctctgaagcaaaaaaga
gacgtgaaaaatcaattgacagcaaatgtaaacgcaaaatgagacggctaaaattattcaatttatacag
aagacaccaggaaaagaggaattatatcaagggtttgctcctaagaagtaaacagtctacgaaaagggcc
cgaaaagtgctaacccgtactccgcaagcttcaagatcttccattgatcataactattcaaagtaattta
accttaatatacatgctggtggctctgggcaaacacacaagcctcgtaacacgcagtgacgtctcatcat
gttctgagtgtgtgtctctagaactagatggtttatttgaatacaagatttgttatgacaccgatggcta
acatctaaactagacccatttgtatgcaatctcgggatgtcatctctcaacagtttatgatgaaacatgt
acgccagcctatggacccgttccagagactgggaaccattcggccaactgacacgtttaactccatatcc
atagcggtcctttgcaccggtccaatctaaacaatcttctacagaatccccagatggggtatgtacacta
agtttttctagagttctccccagcttctcaaaaaatttcttggttttatcacttgaaaacattgtaattc
ttgtatcaaaacaatttcagtcaaagaattaaagcagacgacagaaaaaaaaaaattcccggactttttc
aaaaaatacttactttcacttta
>KolobokP-3_CySi-I
tataagaaaactttttttttctgatactgtgatttttttggtttctacatcaagagtaactaatttcaact
taagaaaatcaaatttcagtccagaaatactagtacttttcataaaatgaagcttttaaaatgactcaaa

atgtctgtgcggactgcgaacaagagcagcaaaatgaaagaaagtgtctgtggtgaaatttctctgttat
tttttaaaacgcgttatatatttgaaccaaatctgtctcagcttatgtgtgaatacttaatttatccaat
tttatcaaatttagggttacacattagtagaaaatacataatattcttagttgcatgaagatttttttggg
aaaaaaagcatatttttcaaattatttgactttcaagtcatattttgtcctaataagcatgactgaact
gcaattctctatcttatatgtgaaaaggcatcacactagattatttcaagaggttactgttatacctcca
ggtcaaaaatcagcaaatagatttcaaaagctatggtccttctgtctgccaaaaaagatcaaaatttctc
tggtccttctctgtcttgtatccccatctgtctcaaatgatagtttatgctgtccatgccttctttaaac
ctaagaactgaagtatttatatacaatataacagaataaatgctacatttttcttctttcatgccaatta
cacctctttgaaggaggaaattttaacaattttaattcagaaaagtccaggaacaccacctgaatgttag
acatgtattcaacatacaaaattttattgttcatctattatatactgtctttacactgatataaattgtt
ctttttacttcttgagcaatgtttaactgtaagtatgaatgaaaatgactgtttacttactaaaaatggc
aatgatagatcttcaacatgggctccacctctatggccagtgtgtagtcatgtgagtacatctgatatgt
gcaaaaggcaaagcaatccttgagtcctaaacacagatacaaccaaaggaagggatttttctaactgatt
agaagaatatttcactctgaaaatagaaaatgcatatattggtagctactgcatgtaacatatacccaca
aatttcaaactttacttttctacctaattagcatgttccctttcaagatcacttcataccccttacaatat
agcataaacagtcaagcaaatgttataaattgtatgaaattgatgtggaacacagttttggctaaagcaa
tgtagctgcagtggatgctgctttgttaatgggtttccagaaaatagcattgctttagcctgaaaatttc
caaatacaagtactgttcctccatccataccttgtatgcaaactgtcaaactagaataatagcactggtt
atgtgaaaatactgtagcctaacaaagatgaaagcttacacaataaaagtattaacagaatcagaaaaca
gatcccaagagtaatattattttgttagctttgaggtacctgtctttgtgaaccagccacaaatattata
ccactgtctgcttaagacttcccaaagcctcaactacacactatcttaatcaaatatcatgaaaacttga
aagaagcttaatgcactttcttcttatccataaacagactaatataaaacataaacagaagctcaatgac
cataagaaacatttttttgactgaattaagtcctactgtgcagtaaaacctgacattaccaaacttcttgt
tctcatcatacacatgaattttgaactcagctaactgtttcatgttcactaaataagctaaactacacta
aatggctcttgtgagcactctggcagcttacaaaacttactccttgccaaaaatatcaggtttgtgtaca
caaaaattctgtttacagttttacaaggacaattttcctgcaattttaccataatggcttaactgcagta
tgaaaagtttgaaaactcactaaatccactcctggcatacttctttacagcattttagcaataaaaacat
caaaattctacattctggcaagtttaaaagttttcatatgcagacaacaggaaatgaaatttttcttata
>KolobokP-4_CySi-I
actttaaggaaaattcaatctgctttttttaatttttagacttgaatttgtagaagagacatttctgata
ttatgtaaacataaacaaactaaattttactaaacaaaacagagacacagcatttcagtgtggtaggagt
cggccagattctatgatattccatatcagagaaactgaaaaaaaagatccctatagtctgtaaaaggctg
tcaaatacttatttgctaaacaatctttccaaaaattggcaccaaaacagtacaattataatactttaca
aatatgtattttctgaaccttaagttttaaatttaatggtattttaatttaaattgtaaaatttaaaaag
atgattcaacttttctttagaatgcttcagaacagaggcaattttttaagctttaatataagtgaaatat
gaaatctttatgtctaattaataaaaactatcatctgggctcttgacatgtttatttgttttacagataa
agactttgcaatacacagaactgaccaaaattaagaggttttatacagacagttactacactgtgactca
cagtgctaaaggacatcaggcacactggctcaaaatgacactggacaccgagcctctggcatacgtcaaa
tacagagtgctaagatcgaggaaagacccgggctttaagaaagctgtggattaaatctgcccaactttag
acaggtttgcacattcagcatgttgcatgctaagttgaactgatttcttttctaagtaaaggtgtctgca
ttggcttgaaagatcatgcaaaaggcagtcatagcaaaagtgcctaaaacctcgaccggctgctaagtca
gtgttaatttctgagaaaattaggggggaaatgcttaaactggtattatgtactttaatcacaaacttagt
aggcaaacaaacactctcccaccctttccagtaatataagaagacaaggatgaatgaaaaaagttgtcct
tagtgtaagtcaaagggaagcaactcacgaaactgtttttttacataaattatttttttgcatgctgacttc
tacagaactgtaaaaaaaaggtataatttacatttaaatttaaagacaaatacattttctatttccagggc
tgaaaatgaaggttactgaacttccatgtaacaaaactaatgaacatgatgaatgagttattcaaagatc
accaaaactactgcaatggtcctggttcatgtcctggtccatttcaactggatttaacagcagaggtgaa
aaggggatttgcatgaacagagatagtgaaatgtgaaaaatgcaaatatacttccagaaggtataagctg
ttcgaagaagttgacacaagcggtcgcggctgaaatgcagctacaattaatgtcggtatatctatggcaa
taacacagacaccggttgggcccaccagtctgggccatatattttatgggggtaacatacctgcagcatc
aatagccgcaatgcataaaacagcaaaaaaggaatcgcacacaatcatagaggccattcagaaggatatg
aagcttaggctcaatacaacaaaaagtgtttcaaatgcgcaaccggtcggagagtgaactgcaagctcaa
agtgacagtgtttacaacaacagtctgtatattgaagtaggtaagaatccttttctagctgggacacagg
cagtttacactgtggcagagaatataactaaaaatcctgatataattgaagttgaaacagtaaataagtt
gtgttctaaagatggttttcatgaccatgaaagcatgacaagctgcagccttaaacacggccaatgttct
gccactcaccccatggaagataacattggggatgagaagaaatgggcgaaaaagacctttcaacgtttaa
aagaacaagggtcagttaccatcaacatcaaacaacagatcctgatacatcagcattaaaggcagcagaa
gagatgtatttagacggcggaaccaaaacaaagcctcttcatcaaatcgatacacgtcatttataaaaga
atcacggaaatacataagaaataatgaaaatgtccttaaaatgatgccagatggaacttctaaattaaga
gcagagcagagaaatatttctgcatttgaattaacaacaaaagatgtcttgcaaaatttcaacaaattca
catgcaggaaagaggcaacttaaacaaacttcaaaaacgtgtccagaaatctattttggctatattaaag
tgctattcaaccattccgagtgtaaaatacatagtgctctttgccgtggcaaaaataaatacaaaaactg
gttctgtccaagcagtaattatctaactccaagctttaaacttgaaacagaactgcacccagatacgttt
ttggactgtgtttattacagacttgggccagaaagacaaaaactaacaaaactgaactaaacagtcaaac
ggctgaggcaacaaacagggctttaaagctatccttacctacaaatgtgacgttcacacgttacattcaa
tgccaagctcacagtgctatccaccgcgtcaaaatgggccaggtaagtcactaacaaaacctattgccag
ggcaggttgtccaatatcgttcaaagtgggtcagggtctgctgtctgaacaacagttttcaacagtgaaa
acaaaaaacaacataaaaaacactggcttatgcacagacacgcaaacaacacataatgaaaatctacaat
ttgtatcagagaagaagagaagtcaaaaactaccagtatggaatgcttcttagaatgaaagctgcatgac
gatccgcaacaaaaattttgaacaggggggaaaagaccccgacattctgaagatcataactaatcacggaa
tgttctggtacatgaggtggctaacttaaatatacacgactcaacccggttcacaggacgggaacaaaac
cctgcaccaacacaaagatgtcgttgattatttctggaatggcactctaatacgagataatctggcctga
tacaggactttgtgtggcaccggtgactgacctccaggtcctccccattcccaattttacggacccatca
ggccagttaatacggttaatgccatacccgtacttgtctatggcacctgtccataatacacaggcttcgg
gactgtatcccattatagaagagtgcttatctaatgtttctaatttctcaaaacaattttccctatttaa
ttgaaatattcaaaaatatagtacaggccatcactatgcatgaaaactatgtaaagaacttaaaaataaa
aagaatacctgcacgcgctgtatccaaagtctactttcacttta

>KolobokP-1_MeMe-I
tataaggaaatatgaaattttttttttcagctttttttgctctctatgtgaagtttcatctatattcagct
tataaaacaagtttcagcccaaacaaaccagtagttttctcaaaatcactatttttgtacagccatcatt
ttgtccaaagactgcggtttaagccgttaaatttggctgaatcacatatagtcattaaacgctgtttttt
caaaaaacccgtaagatattacatcaaatataccaccagcaagtgcggaatagacaactacactgaaaaa
aacttataattatgcaactgtgataaaatatagaacaaaattagtgtttagtgaattttttttggttaaa
aaaaagcttattttttcagtaaaaaatgggaaaattgtctttcttttcatcacaaatgcaacaatactgca
tatttttgcattctatagctaaagtcatatctcatggccatggtattcaagttctctccaacaattcatg
tcaaaatgtaactaaaaaggtgttgatactgcacacctggtctctgctcaaaaatgtgtgaaattcctca
ggttcagtactgtcagacacaggaatcctcctgaaatgttagttwagtttctgcatgccttgttactaac
acatataatttagaaaataaaatagaaaaaaaaaaggtttagtgtaaataatcatttttttaagaaataag
ccgctcttcaagtgagatgccacggaaattttcccgtaaaagtcataacagacttgtcggatttaaaaag
ggggtaaatcatgaaggtaaaactaacatttctttacttagtcagacaagtgttactgcaaaaaagtatg
ttagacttacaaaagagacttttgagtcaagggttaatgataacgaaggtttacttacactaaatgatgt
aaatggctctgaaacgggtgttgcacctctgcgatcactaaacagctgtcctaagttggcggatgagtac
agtacaccaggaaacactgaggctcaccctgatctgttaactcacaaactgtactgtcctaagctgctac
aggaaatgtttaactctgaaataaaaaggcattgcactggtgaaaacatatgtgatggcaatctgaattt
tgatgctgaatcttctcgtaaatggggattagcttgggaagagcggcttaaatgctcaaaatgtagctat
attagtaaatattataagctttatgatgaggttgaaaaacaaggaaggggtagaaaagctgctaaaatca
atgttggcttccagttaggtgtaatgacaaccccaataggtaatacaggtgcagctagattactggccca
tgctgagataattccaccctgtgttactggtatgcaaagattgtcaaataaagttggtgaaaacattgaa
acccttaacaaacagaatatgcatgaagtaagacagtcacttaaacaggaaaatgcaaaatgtggtaatt
caaatagctcagaagtcttaaccgaaggggactcctgctacaataaccccttgtttaactccgaaagtac
gcccttttcaagctggcaccattgtcgcaacaactatttgtgaaaataatacacatagaaagtcaattat
tggtgcatttgttggtgttaaagcatgtacagctgcttctaaattaagaaatcaagggtataaaaatgta
aagtgccccaatcatccaggccactgtacagcaaatttagatgaggcccaatccattggtgatgaagcca
aatggaatgaaatggtatctaaggatattactaaagatcttcagatttcacaccacacaggtgatggaga
ttctaaaggtcattctggtgtagacaaaggtcaaggaactaaaactgtgcacttgaaagatgtgaggcac
ctagcaaattctctaaaaaggcaaattaaccgggcaccatttagtgacaaaatgttccgtggtaaacaga
agtctaatctaaaaaacagatttgctctatctattaaatctaggtgtgtttcagagctatcaagtgcaca
taagaagtacaaaagcaatttgaaagtaataaaacacaaaatgcctaatataatttcagcaatagtacta
tgtttcaaaggttactgtggtacctcatgttctaaatacagcttggtttgttctggcaactacagaaaag
caaaaaactatatgccagacaatgtcaggcttaaaatgactgatgatgatgagactttactcaggaaatg
tattgaagttctccttggtccagaaagcatagaaaaaactaaatttttaacatctacacagaaatgtgaa
gcagtgaacagatcctataatgcatgcttgcccaaaaatgttacatttctccgcaactgccatggccgta
tccatggccagattctaagactaaatcatgggcttgctgacactgttatccttaaagctagggtaaatgg
tgtaaaactgtcacatggctcttctgttatcagacaccttttaagaacagagtactacgacaagttgcgt
aaaactactaggtacatatcgcgagcaagacgcagtcgttttgcatcacgccagtataggtacaaattac
atacagatttacactattcaaagggtctcactgaccccaagcctgacttttctagcaccccacacctaaa
agatcatgcttatgcatagagtgtgatattaggatgaaataaagatggttataagtaaaattattgtgct
gccttgtggccagctgagccggcactgcacacatataacaagttatattaaccgcacaaatgtaaataag
ccttgtaaaagttgtgtatataatagtgctatatttatcaaaaatattatacaaaaatagtaaactactg
tataccatagaatacagtccttgaaagaaaactccctaaacttatggcctgagcattggtccccacattt
ctgcctttcattatttatgaactggggttcaatgctcaaatgttccgggttcacaaacagagaaaagtgg
catatatgcgacacgtgtaggccttcgggaattttaaaatatttctagtggcctgacacataagtctgt
gcacataatattgttttctgacagtttctccaggcagttttgttgttatttttccatagctaggatttt
attcactgtcgtacaaccaagccatttccgacacccttttcccccaccggtatagaattatcatttatt
ttttgcttaattctttcaaaaatcaattgtctgggttgatgtgccatgtttacaatttttttttttatttg
cagacgacagaaaattttagtaattctaccgggtgacaattcttata
>KolobokP-2_MeMe-I
tagttagtacagatttgtcggtgaattcaaataatattctagcaaaataaaatatagggtatttcgaaca
aatcctgatgttttcaggtcattctatcatttaatttgaaacttacagcctttttcctatgacaggtgta
tacagaactctgaccaaactgcagccatagaattgtgtgaaattccaaaactagaattgctgtattttt
tcatttcttgcaattttttcatcaaataaaaaatttttttgttcagtttataaatgtctatctgaaaatgt
attcaaattgcaccagaagtmagtagtttaaaaaaaawttttttttttaatttttttaagagttttttttatc
tttttaaatatcttaaattaaaaaaaaaaattaacaaaaattctgaacctgtcaaatttttactttttct
aaaaggctataatttaacaaacaaaatgataggtaatttacaattttatgacctgtggttccaaagatat
ctcatgctgaaatgtgccactgtctggtccagcctctggcaggcttgtatattgtccaatacacaacatt
ccagtagagacaagcttgacttctgtgtcacttctgtggaatccaggaatttctggggttgttgacaggc
attttccgcattctttgaatgccaagtttaatataaataggaatcaaatttttggtaatcctcagaaaaa
cacttacttttatacaattcacttcaactgcacaactatgaaaggagaccgcaatagaaagtcttctaga
ttttctcctggcagcatcccatggaacaaaggtctacagctgagtagcatgaactgtggagacactgaac
agaagccctctacctcaaggatgactgcagaagaattctctctagtggctagacctactgctgatggagg
ggaaacctctggaaatgtccgggtgctacgaccaaaagtgactgagacgtcagccctgaaaaaagaagat
acaacatcatgttcaggcatgcgacttgtagactgtgaaaagatgtctgaagccttcaatgaggccttcc
agattcaccaacaagaggagttagactgcgaacaggccaatatcatagtggccgaggagagaaaagttgg
cgtttgttggaagttctccctaaaatgtacaaactgtaattatgttacaccactgaagaagctatatcaa
gaggcaccatctccgaggcgaggaccgaacccagctgcaccaaatcttggattggcagctggcctgcaag
acactccacttgggaacaccagatgtagatacctactggcaaacctcgacatccctccaccctcaaaaag
tggcatgcagaagacagccaacaaggtgggctctgctattaaagaactgaatgacagtgatatgtctgaa
aaagtagagatgttaaaggaggtgaacagaagacgtggtgatccagaaaattcaatcaacatagcaatgg
atggccggtataattcaaccacaatatccagtcgaaagaaagccggacagaatgcttcccagtctattgg
tattgcctgtgaaacaatgaccgagaagcagtatatcattgctgcagcagttcagaacaagctctgctgg
acaggtgcctggcttcgaaacaaaggctacgatgtcagatgcccagatggccatgcagactgtacagcat
ctcttcatcgtaatgcaccactgtctgagtaccaacttgggaaatcaattggggaacagttaggggtaca
agaagcatatattaagtatgttaccactgatggggatgcgcgatcagccgctggagtaggagaagctatg
cgcttactggacccgatgtggaaggttgaacgtcaggcggaccctactcacctcggacaagctcagtttc

gacactgttaccaggcgaatttcagtgccggaatgttcacagctaaaaccagggagaaaagacaagaaca
gcagaaagtgttcagtcaagacatcaaggctaggtgtagcctagttttcaaagaacttatgagaatacat
gctggggacattacacaaatcaagaaagagttacctagtgtcctaactgccactgtaaactgctatgccg
gggactgttcaagctgtaggcgtcactcatacgtctgtggtggaggtgttaccaactcttggtggaaccg
ttctatgtatctcgccagtcataaaatcaccaacctgtttatggaagaaaatgacagacatttagtacag
gagataattaaaatgaaactaagtgactctgctgtagaacaaatgaaactagggaccagtacacaaaaat
gtgaggctgttaaccgctctatcagtgtgtctctcccaaagaatgtcaacttctcaaggaatgtacatgg
ccggttagcatcaacaattcaccgacttaacaatggcaattgcggatagtgctgaaaagaaactggaaca
cttgggagtacacctgtcaagcggtactcggtattctctacggcaaatgcaggtggaagaggaataccag
aagaagtacgcaaagcaacctgaaaaagtgaaaaggaggttactatccaaggggcggcagctagaagatc
acctaaaataccaggcagcaaaaccttcaaggtcagactaccgcaagggacagctagacccagtgccttc
aacatcaagggtagggaactctgctgaccatagctattcacagtgacacctctagttatctcccttgtgc
tggagaaaaatagactatagcaagaaaaaaaatcttatgtgggatatggacaaataaataaataaataaa
taaataaataaataaatatcttcagcaatcactgtgttgtacagacatctttacctgaaacttacaaaat
atgttgtttagtatataattatgactgttgttgttgttgttgctgttgttsttgttgttgtaactgtata
ttctgtaataaaatgctattttgtaaatattaaaaagtatgaactgcaaccaaatgagtttgtttttaaa
ttataacaattgsagtctcttttttattgaaattaaacaagttttcacggcagatgtcaatgaaaaaaata
aaagtatcttgtgatgactaccgtgcaaactgacatatttcaatggaaagacactgccattgtattagta
ataccgtaaawaaaaaawatmaccaaaagtagctaccgtaaaacccataataaatgcccatcttcaaaca
aatgccaacactatgtcctttaaaacattgaaaaagaacatcatgaaatgccggtactatttcaataaaa
atagtaagaaaaataaccattgmcacgacaaatactgaccgataaactatatcagctatttaaatctggc
tctgctttaattcctatacatttagagtaaaaaataatagccgatccatgtctggtgcwaaataaaaaaa
aatcaccagggcatttatttggaaatttacggtaattacttatgtggttattttattaggaattttacgg
tacacaaaaaaattacaatatgtaaacagagaaaatttggagaaatacccagtcttaacatctgaaatac
atgaatttagtctatcaaaataagtacaaaatagctcggcagatctcgacagcacggcagcaggcattac
aacaagacctcggaaggctagaaggaaatatatactaacactatagtaaccgacaagttgcataaggccc
atgcttcgaacagaagccccggttaacacacttctgcctctcagtatttackctatgaggttcaaggctc
aagtgttccaggttcaggcagagtggttcgtgacataggtgagatacatctaaacccggctccagagaca
gaatgcgtctactgatcatgtaccgcaacctgtgggcatgcatagtatgccagtcaccatccggaaactt
ggcctttatgaccccatagcctatggctcctttttttaacacagccctgccaaatatgacagccatgcaca
ttttgctgtatttttctgtctattatttcagtgtatttttcaaaaaatgcctccatttttaactccagaa
atggaaatgaggctgtagactgtgtatcaaaaaatatctgggctataattagccaaacattcaaagtgaa
agtagaatctgtcacattttctggaaaaattgcatattaaaatatataatgattatataatattaaataa
tacaaataaaaattagaatttcttgaaatcaatgaaatgtcaggtttccacatcatataactgtcacctg
tgaaagtttcatccaaatacaagcaaaactgaaaaagatattaaattttaaagtgggtcaccctctctcc
atttcacgctatatca
>KolobokP-3_MeMe-I
taaatctaaccaaaatttgaattaagccaaatttttttacccatattctagtaaattagggtattgcaaga
aatactcaacaaggcattctcaccacaaactaaattctgtaccgtagtaacactttaaacatgaggctta
ctctgcggacaaattggctcggaaaccggtatttcaagtgaaaatagacacattaggaaaattgtcatat
cttgaaaaatttttgatggattcttatgaaataaagtttggtgtcaccggcttgtcttgtagaatctttt
atataacagtatgccttattttttctgtaaacttatatatcaaataagaaattactaaaaaaaataagac
atttttatttaaaaaatacagaatttgctaactaaaatgaaaataaattgatattttaaagatttaaagg
acaaaattccatgactgagaccaattaggattactgcaactaatatctgaaagtaaaacacacccataca
ccacactatttactaataatatcaattaagatcaaaagtgattatctaagacttaacacctcttaatgat
gcacttcaaacagactttctgatagctggattagcatatattatccttgaaaaaacaacctgaatgtgtt
aataatggcacaaaaaagacttctacttacaatactgccagtctttaagcagacagcatcaaaatcttac
atcaactgtatagaattcaacatggcaaagggcaaacttaagcgccgtgctgctggcggacatttcaagc
caggaaataagaaatcacctgcttaccttcttccttctaaggctgcaaccgatcctggatgtagttcagc
tgtaaatgaagaccagctggataggggctgcccaaagaaacccgaacttcgaccacgacagctggatctg
aaaatggaatgtgaaccattagggaacagggttgtgaatctggaccaactaatttaccttatgaacagtg
tgtatcagcaacatagttctggaaagtgtaaacagttaaatatctcactgcacaaggaagagaaatatgg
actaggctcaaaactgcagttcaagtgtaattcctgccatttcctctcaaataaaatggacacatataaa
aaacgtcgggacggcaaaggagcagcaattaacatgttacttgcttctgcccttcaggatactgccatcg
gaatagaaaaagtgaacttgctcttaacatctatggacataccaccaccagctagaagcaacatgcaaac
tttactgaatgctgccagcctcaacactgtcaagctgaatgaggaggacatggcagcaaaaaggcagcta
gtcattcagcataatctggaacaaggacatcagaacccgcaacttctagacctgtcctttgatggccgct
acaatgcaacaagaatggtgtcttcttacaaacctggacaggcagcttcacaagcctatggagttgccat
tgaaaaccatacaagctacaagtatatagtgggactagctgtacaaaataaactctgctggacaggggcc
tatctgaggaacaaggggttcgacataacttgtcctggtggccatgctgaatgcacagcaacaatatcat
acatgcagcctcattctgaaagaaaaatggcatatggcattgctgagcagttgtcaaatgaagacctact
tgtccgcacactgacaactgatggagatacagcagctcatctgggaatgaatgacttctatgataagcta
ggctatgcctggagtgtcaaccgccaggcagatccgcatcatcttggcagcaggcaagtgagaaaagccc
ggacttccaactggagccagacagtattcgaaggcaagaaactgacaggcagtgccagacaacaagccat
ttccgctctgggtaaagatatcaaggccagatgtagcgccatcatagagaaacttcgaaccgatggaaat
ggactgacagaagaagtagtaaatcgcttaccggcaataagagcagctactatacagtgctacgctggaa
actgcagcttctgtccacacgactcgatagtttgctctggtctaggggtgttggtgactggtggtacca
ttctaagtttctaccaacccatggaattcatcacctgcaattgaacaaaaatgaccgtgagctgttgtca
acaattcttgagataagactcagtgagtgtgccatcatgagtgtgaaaaacaacacttcaacacagaaat
gtgaggggttcaacagggcagtactatctactatgccgaaagatattaacctgtccagaaactttgctgg
tagtctggcatcaaaaacacttcagctgaacaattcattgcaagcttctgtggaaaaaaaagtcaaaagc
attaccggactggctctttcaccacaggcaaaaaggtatctgaaaacaacttcaaagaggtcacagagac
acagaaatcatcaaaaaacagccaagttcaagacccgcagaaaaaataatcgagcaaagctggaatatgc
ttatcatcaagcccgcagttcaggccagtattcggacgagtacgtgaaaggacaacttgatgaggctcca
tcagcaaaacagtgtttctaaaatttaatgccttagaaacatggcggttgttcacctacaaggaaaagaa
acagaaaaaaagggaaagagcaccgtcacattcatggcagaatgtctgatgatggcctgatgccggccga
aacggaaagagctcttgaacatttactgaaactaaaataatacataaacaacaataacaacaacaagaac

caaaacaacaggcataactagtatccatctcaaattcattgtaatacaaacattgccctgtaaattaaaa
aaaataacagaaaattaaaactatagtcaatgtgttgataaatgcactagacagtctgctaagcggtgcc
ctgccacatcaaaatgcccataacacctagaatgagaaaaacaattttttccttaaattatttgtactgtt
tccctcaaaaacaaggtgtttaacatttatgcacagtttattattgcacaaatgtgatgcctgttgactg
cctgaaatatcaaaatctctataatgaaccattaatgcaacacggtgactggttcgtgttctataaccgg
cagacggtgggtctcttggatctctgtaacgaaactggccgtagccaaatttgttaatacaaccagtcca
aaatatacattctccattttcatgcaagtcactgttgatctgcagtttgttgcggcaatgttccagtagg
tcatccatcctaaaaaaactgttcgaaaaactgatacacaaaaatagtgattttttttaaaaagtaaaca
aattaattttctctattttttttttaaattaataaaatctgtctgattttctcttatatagcccggtta
atagtcttttcttcttgaaagtctttgcgctattgcagacgactgaataatcaaaggagatgactgtt
acttaatttaaatgaaaatttaactattaaatttttaaaaattcaataaaattaaaatttcacaaacctt
gataaaatcctttagccactggtttttattgttatcagctactagtgtgtgaagtttcatagctgtaaa
ccctttggttgctgagatattcaaaattaactttttcccatata
>KolobokP-4_MeMe-I
tataagagaaatttttttaattattttcacaaaaactgcctttacctattaaaacaatccaattgcaat
aaacataccaagtttcaggcccaagaaatgagtacttatgaaaatatttccagttttgtacggtcatgta
aaaggcgccaaattcaaacaaaaactggtgtccgagggcaaattcagtgtttcacttttttaagctttat
tcgcagtttcgcgttaaatctttcaaaatgactggttgtttcatacccgaatgataacagtgtatctaaa
aaataggcttttacatatattatggctaaataatctcccttttattatgaagtcatacaaattttctgaa
aaaaaaactcctttttttctgaaatttttttctgttttagcttatttttttggcacttgaagcaataagta
ccagtaaaaaatgcattttagatgttctttgatgtccaaatcagaattcagcactaattaagtgtcaata
agtttgtctgtcttggtgcaacaaagaataatggagacttaccttcaagataaaaaggtgtgaaaatcac
catgtccaattagccacctgcagatctagcttaacaatatcacatactataaatacttcaaacctacctt
ttatatatgttattccaaggtctgacagagctgttgaagcattattgaggtatgaaatgttaatttttat
gtcagtaaagtatgccccctgccagcaaaaggaaaaaaataggccgacagcatgggtttcagaaaggtat
gccaaatttgagtataaaaggtaaaaatttctcttatgtacagaatactggcagaaagtttgttcggttg
cagtcagacacctttcaggctagaattcatgaaggtaatgacatacttacattcagagatgttgatggtt
ctgaaacaaatactactccactcagacccttgtctaggactcagaccttggctgaagaatatgcacactc
tgactcagaaggtatcaaaaatcaccctgatctcctcacaaacaagctgtacaggccccttgaactgtct
gttatgtttaactctgaaataaagtaccacatgctaacttataagtcatgtaatggtgatttatacattg
acactgccgcctctaggaaatggggtcttggttggtacgagcgccttatatgtaccgaatgtggatattg
tagcaagttttaccgcttatttgaatctgtcccaactggcaaaaaaggtagagaagctgccaaaagtaat
gtaggactgcaaataggtcttatgtccactcctattagtaacacaggtgctattagaattttggcaaata
ctgacattataccacctagcccagcaggtatgcaaaaacaatcaaatatggtaggggaagctattgaaag
actaaatatagaaaaaatgcatgaagttagggaaaatataaaagctgaaaatgagcaattaggttataaa
aaccccagacttgtccgtgccgagggtgattcctgctacaataaccctctatttaacagtgagagcacgc
cttttcaggccgggacaattgttacctcaacaatttgtgaaaataactcaaaagataagaaaatcatagg
tgtttatgtaggtaacaaactctgtaagacagcctcaaaattacaaaatcaaggacatgacattaagtgc
ccaaatcataaaggtaaatgcacttcaaacttggctgaatcagattccattggtaatgaaaacaaatgga
gccagaatgttgtccgggatattaatcaagatcttgacattgccttctttactggggatggggactctaa
aagtcatgctggtgtaagtgcagtacagaaaacccatgtcaatcaccttaaggacttaagacatatgagc
aactccctaaagagagaattaaataaggtcccatttagcaagaccatgtttaccggccaatttagagcta
actacaggaacagattctccctatctgtccgggcccggtgtgtggctgagctgaaaaaagcccacactat
ttatagaggagacctcagccttataaaacagaaaatgccaaacataatatcttcaattattatgtgtttt
aagggctactgcggagacttctgtaaaaagcacagtttagtttgcaccggtaattaccgcagagtaaaat
actttctcccagctaataaaaaactgaaaatgactaagtctgatgaagaaatgctaagggcatgtcttca
gtcattcctaggcccttcaagtttagaacaaactagattcctaacatcaacccaaaaatgtgaagcagta
aacaaatcatatcagacttgtatgcccaaaaatcaaacattttctcggaactgtttcggtagaatacatg
gtcagattcttaaactgaactatggttttgctgattctgtactgattaaatgtaagtctttgggtgcttc
tctagctaagggtacttctgtgattaaacatttgctgaggaccgactctgttcatcgcagacgtaagaca
tcacactataagctaaaggcaaaattgtcccggtatgcaagccgtactaggcgttacaaagtacatgaac
agttgcattattccaagggattaactgacataaagcctgattttagcaatatccctcatttatcagacca
ttcatatgcatgctaagtgatgagtaacttgttaaccacttttagatgtgtcagctgtgcagcaatagtg
ctatttcatatatcccccacaccacactccccccagccttttaagtaactgttttgtatatatgtatata
tgtatatatagtcaaatcaacatgtataaatacaaattaagagtaataagtaccaaattatacagtctgg
gtatgagaggccattaaaattatgcccgaaacaccgctcctcacacctacgcctttcattgtttatgttt
tgtggttcaaggcttaaatgtgtcacattacaacataacgaaaatttacataaatgcgacacatgcagtc
catccggtagttcgtaagagtttacagaacacatatatataagtcggtgcacatagcggtatttagaact
ggggtcctgggggaatttgaccctcaggcgcccgtaagcaggaaaatttgcgttggaccgagtgcctgtc
cactttaaacagcctttttggccaaaattttctgttttactcagaattctggcttgcaaagcctgaaaat
gttgatttgcattcattgtttacaatataaacaaatttcaactgcagacgacaaaaatttttttacagag
tgttactcttata
>KolobokP-5_MeMe-I
ttaagttttttttatgtgtcagttttaaatgttcaatatgttatattttaaaaatttgttctatttcgaat
gtaataaaagaaataaaacttgtaaaactgtattttgtctatttttatatacaatttaaaattgtgacta
ccatcaagggaaataacagcagcttttctgactgatgttttaaaaatggtaccattattttctaatttaa
ataatggaatggtacctattcattaaagtatgcctgtcaaaagcaaggaccagcttcagcatacaatgca
attaatgactggacttggtggaggggttctgctaaaagcaatgagctgtagaacatctccagcccccaaa
aaggcaatcaaaacttagcaacagcctttatgtcagtagaaataaatatcagttttattaaatttgacca
tttacatttaaaacaatggttaaggtatcaatttaaagcatttacagctttgcagtaaagacatccacag
tgtctgtgccatgatagtcactcatctctatagataaaaatatcactatgatacaagggaggcaattcag
aaaccaaaagtattgttcacatcctctccaggtaagtagatagaaaacacagccagttaatgaaacaatg
cctgtcatctgacaccaaattccttagattagcacacacttcatgataatatccagtattctgtgcaagt
ttttgtctgaattaaaagactgtggttaaaactgtcctaaaaatgaggagtactaaaaaaaaatctgaac
gcaagtacaaaactgtgagtagaaatcaaagggcaggattctttaaaaagggccataagccctattattt
atatacaaacactcagtgtttaagtgaggaaagtgtagttgctggatgttcatttgatcactcacaccaa
gggggtgtttcaactgtaacaagactcagtgcctctgttgctgcagatgttctgaatgctaccagtactg

atgacacggatttgactgttccattcaagttgagacctgctgctgcacccaagaagccacctgagcctgt
caataaaaacattcatctaaatctaacaagaatgagacgcaaaacaatgaaaatattatcataaatctgg
atatgttaaaatgtgcattcgagatggtgtcaaggcatacctgcccagtgccggacattactctaggacg
agcaaattggaaaggtttgctggtagagattgatattgtgtgtagaaactgcgatttccataccggttta
atacctgtgacagtccccaccacagttatgaattccagcttaataaacacctctctactgctgcctgtaa
tgctgtcaaagctgggcataaatgatctacaacttgtgctcgctgcattaaacattcacgctccggacaa
aagagggatgcaaagagggctgaattttctggcagatgaaatacaaagactttcgcgaaaacaactaatt
aagaatcaggaatacgtaaaacagattgaaaaacactccggcaagacagatgggacagacgtggaatacg
acgtatcgtacaccagtcgaccgaaggccggcagtgacacagcaagtcaatgctttgcgcctttacttga
aaagaccactaggcagcacttaccggtagatattcaaataggcaacaagctatgcccaaaaagaaactgc
ggacataaatctgacacatgtaaaaagaatttcgacagtgatacttctataacaaatgcagaaaaaattc
tattaaaaagttcagtaaagtctgtagataaatccaatatcattacgctaaacaccataacaacagacgg
gagtcaacaaattagtaaagctttacgagaaataaataccaggcggaagaaaaatatcacacattacagg
tgctttgtgcacaacatgaggaactttcatcgtcatctgaaaaatgctaagatcaaagcaccaagcggac
aaaatcgaaaagtatttaccatgaaacttgcttccagtctgcgtatgagaattcggttggagctaaaacg
tctgaaaacagtctgcaaaggtacagctagtttcatccataaagctcatgcctctgtaaacaatatactg
gattgcttccacggtaaccacacactctgtcgaaagaaatctgtagtgtgtactgcacatctaaaaaaaa
gattcaccatgtcacatttaccatatggcaagtaactcaaactttcatgtaaagataagcagatagtaat
gtcatatattgaaaaatactgcggtttagatcagctgaagcactctgcagaattatatactacaaatagt
tgtgaaaatatgcatagcagattgttttcatatgcaccaaaaactactgtttggagtaggaactttccag
gtctatgccactctgtttcacttggagcaagtattggccgtggaaagtctttactccaaattgtacaaca
tctaggtataccgttctaaattcaggatcctatatacaaatatgcgcttaaagctgaaagagtcgcgatg
tacaatcgcagccgaaaaaggtcttacaaatacaaaacttctaggtatgtaaagcgacgaaaatattcaa
atagaattattcttgaaaattccatttaccgcagtagaactaccgcaagtaaagaacacacatacgctat
agacatgctaaaatgaacaaaaaaatgaagtaacagaacagaacagaacaaaaaacttgcaaaaactgga
ataccatgaagtgaaactgtagaaaacagtgtcagatgtactttacctcacaacttgcacctagggtaag
cataatggccactacactctccttcatttttacattttaaccggttattattaatttcttgcggttcata
acttaaatggtccaagcgtacacataacttattatggcaaagatgtgacacatgcatttttgtgcttaaa
agcatgtcacatgacaagaagtaaactagacggtgtactctgacttttactcttctcccccgaaatgtaa
agcggcacaatccatagccatctgccgtacacaagccgttccaattataacagttttcactggaatttgt
gcggcttttaacaaaggcgggtattttaagcmtttcgtatgcttctaaagatgtttgaaatatgtatgcg
gccattttgcttaaagacaacagataataatgaagaaataagtactttctcaagcataaaattatccaat
ttgtcattaaatatatgctaaatgtgcttatccagttgtatatcatgttaattgtatcactaaatggttt
gaaatggacaacagtcacttgccctggtgtatgaaagggttgcgtactattactgaaaatttcatcgtta
gttaattacctcccttttttatctagaaattctcagccgaggtttcwcgcaggaactaaaaattcttcgca
caatttcaagacaatgtttgtgtgatacatttatccgagaaaattgtaaattcgctaactttttttcattt
tcaaatttccttaa
>KolobokP-6_MeMe-I
tttaaggaactttttttggtggacaaaatttgaaattgatacactgacctttaggtccaccttttactaca
aacatgttaaaatttgaaaggaatccatgcatgcattctctcaaaatatgcatttttaaatgatgatatt
tttcaggctcggccaattgctattttctgatttttttacagaaatacctcttataggaaaatgtatatatt
ttttgtatttcttaaaactgaagactctaactgctattggctattactgggatgacatttgaataataaa
aagtaaaaaaatttcacatatgatcatatttcctacttttaatatggcttgaaacatactttttttataaaa
aaatgcaccatattttccaaattttgcaattaagatggtatacaaatgactttctataaccaaagaacat
gttaaatttggttatttgataaaaagcaaagtgtcatttagtgattacaataatttagagacaagataat
ccatgctaggggctttattaaggtatattgccatgtctgtgcatctggctgtgaaggtgtgaaattcctc
aggtgttccacagggttctacactatctcctgttttgttcatagtcacttaacctgttgttttgtataga
ttaagatgttaaaatttaacagtatatgcatgtgttagttattagttataaaatattatctatttttttt
tgtcactaaattgcataaaatggttcgtttgaagaaacaagtaaaacactgccgcagtgttgggttcaag
aaaggtgctcctagcacaactaaaggtatgaaactttgttataacactgataagttagaatataggtatg
ttaggttagaaaaacaggttcacaatgcaacagttcatgagaactctgaagtactaacctttaaagatgt
tgatggcaatgccacaatgacaaaaccactcaggccaaaaccagatagtgttgggattatcgataagtat
ggtactgctcaaactgattctgttcacccggacttgcgaactaacaaactgtatcttcccgtgaatgtac
aggctatgttcaattctgaaatgagaaatcacagggctgtacacagtgaatgtgaaggtgatcttgtgtt
tgatggtgaggcgtcacaaaagtgggggtagtgtggcgtgaaagactcaaatgtacaaaatgcatgttt
ttgagtcagtttcacaatctttatgatgaggtggatactggtaaacgtggcagaaaggctgccacagcta
atataggtttgcaattaggattatacaccaccccaataagtaatactggcgcttgccggatactaaataa
cgcaaacataatttcaccgtgtgtagcttctatgcacaaaactgcattaaaagttggccagggtttacag
gtactaaatgaacaaagcatgtgtgatattcaaacaaatcttaaaacagtaaatgaacaaataggtcaaa
aagagcctgatttagtgaatgttgaaggagactcttgttataataatcctatatttaactcggattcaac
cccgtttcaggcgggtacaatcgttgcgactactttatgcgagaacaataccaaatcaaaaaaaatcata
ggggttcatgttggcagtaaactttgtgtagtagctgccagacttcgcaataatggtagaaatgttgaat
gtccaaatcatgaaggacactgtagtgctaatgttcctgaaactgattcaattggtgatgagggtagatg
gagtcaaaaggtagcaagtaaactgtcaatgcgcatagcttcctacactggagatggtgactcaaagagt
catgccggtattgaaaaatctcaaaacaataaagtaacacacttaaaagatgtcaggcatcttagcaatt
caatgcggcgtgaattgtacaaggccccattcagtagctcaatgtttaaatgcggtagtaaacaaaaatc
aaatctaaaaaataggtttgctatttctgtaaagctgagatgtgttgctgagctcagaaaagctcacaaa
gtacataatggtgacataagtaaaataaaaaaaaaatatgccaaaagtaatatctgccattgttatgtgtt
ttaaagggtactgcggacaaacatgtaaaaatgatagcctggtttgttgcggtaacttcaaacatgctta
cagttatatgccggcaaatgtcaaggtacgaatgactgaatgcgatgaaaatgttttgcgaaagtgcatt
aatattctccttggtcctgacagcatcagtaaaaccaaacttatgacatcaacacaaaaatgtgaagccg
tcaatcgttcatatcaagcagtcattcccaaacagacaacatttgcccgttcctcgtcaggacgcataca
cgggcaaattactaagttaaaccatggttatgctgaatctgtcttactaaaaactgagttctttaaggct
aacatttcaaaaggttcaaaagtagtaaaacaaattgtaagaactgaaaaaatcagatgtacccggcttc
ggtcggacatgaaacagaaagcgaaaagagctcggtatgctgctcgggctcggcggtacaaacttcatga
aaaaattcactactcgtcaggccttacagatcctaaaccaaattatagtgcagtgaaacacttgcaagac
cactgctatgcataatttagtgcctaaaattgcatgatttagtgataagactgcatgataagtgatacag

aagcttagatgtatatacagaaaaaatgtgtgacaaatgtatataagaagttatgctgttggttcaaata
aagtgctttttctgtatattttctacatatttcttatgttttcagtggtctagcttgtacatactaacaa
aacaaacaagacactgtcacaaaaattagatttaagtcacattttctttcagggatttttttcaggctcta
aataatacaatttgggtagccggtatgcccaagacatctgcctctggcaacacaagtcttcctgtcatga
ttaatttccatgggttctagggataggtgacttgtgttaaaacacaaagagtggccacacaggtgtgaga
gaggctggttagatgtcaaaatcaacttaaaatgaagcatatatgccaaacggtgaacaaaatgtacttt
agaaggggcatcttctggaaatttagccctaatcttgccatatttaggccctaaatgtccagaacatgcc
cctgtccaaacaatacagcccttttcaggttctagcatgcagtttctttccatttttctttcaatttctc
tgtaattttcaccttccatgattttgtagcagacgatatgttacatccgaatactcttcta
>KolobokP-7_MeMe-I
ttaaggaccaagmcatgcatctccatkgacwgtttggcacctgattctaagaaaatgatctatgacaaat
aaaatcttaccaatatctaaacaatgctttgttcttcttgcaaattgcacatgtccaaacaatgtctaca
acaktgtggatwtggccmcattttacacaccmtatttcttgctgcatctatctttctgacattaaaaaaa
gacttacctactawtacatgatggccataaatgtwctgtattagcattgtgcacagccctgcatacttga
ggaacagaattaaaagcagcacagcttacttacccaagactaacagtacttcaagtccgcaaggttacaa
aaagccgcccctgtttacaagtactttttcatattaccttacatacaatgagtatatatkacttatctga
tatataatactaattaaaaatacaattaatatcagcaaatgtaatgtataaatgaaaactgtacctcttc
agtgaaccaaaggatgccagttcctacataggtgaaacatagtcctcagtgaaagggagacaacttgtgc
aaatttcttttacctacctgcaaaagtccattcttgatgagagacatatgcccctttccagtttgtcatt
aaggctgaaatctgatatccaacaagctgataagtacataccttttctcctgcctggtcatttctgaaca
gacattgcaacagaaagtgtgtaaaattaaaaaaaaaatcatatacttgccttaatatttctggataatc
acagtttagccatcagtttgtgttaagcaatttggattatatgctgaacagtcatgcttaaaggatctac
aagtaagagaagctacawacatgcccataagagaaggaaaagctacttcaagataggcaatcaactatac
aaaaacagacacaaaccttgtgaacaggcagaggtgttaacttctggacaagaccaggtagagactttta
cacagcgcttaagcwactcagatgcacaggaaatactgcatatatctagagatgacaatcctgacttgac
actaccatacagactgagaccaaaacctgaaaaagacaatgataacaatakaaataaacttgatgaaaat
gtaattataaatatttcaaatctacaagaacttgtaaaatccttgaacggacacatatgtgatcaaccac
acctagaaacacaaataaccgacagaactggactttgcataaccctgtcagtgaattgtcaccactgtca
cttctcatccggagagattaacatgtccgagaaagtaaagaagtcacgtggtcccccggccggggcactg
aatgaaatgcttctactgcccgtcttgaagtcacgaatgggactatcagatgttcttcttgtgctatcat
gtctaaatatcaaaacaccgaatcgaactaccctacagagtaaattaaatgttctaagtgacgcagcaac
acagataaacaagaaacaaatgacatcaaatcaaagataccttgcaagaatcacatctcttgcaggaaaa
gagaaatctgttgacacccagtatgacacttctttctctagtagacctcaggccggaagtgagaaggccc
agcaaagtttttctccactcattgagcatacaagtttaaagaaacatgtcctcagtattgcagttgcaaa
taagcactgcatgaaaaagaactgtaatcaccaaagctgcaagaaaaactatcccagcgacgcttcaatt
tcagccagtgaaagtactctccttcaccaaaaccttaacaacatttccgaagcaggtcacatcaaagttc
ggtctgttactactgactcaagctcccaagttgcaaaggcgttaagggaatacaatgcagagaagaaatc
aaacattaaacattacaaatgttttatccacaaattaaggactttaaataagcacgtaaaaaatmtcaaa
ctcagcagtataccgaaaaaacacgacaaaaacacatatacccaaaaactggctacttcagtgcgcacaa
gggtgcgaatggaactacaaaatctccgtggactgaaaccgagtaccgagaaatttgtagaaaagggcag
cttatctctaaaaaatattgttccctgcttcagtggacatcataaaaattgtagacaacactcaacagtt
tgctcaagtcacctaaagagctacaataccaatttcttgccatttggtgttcatctgtcattaagtggaa
gtgaccaggacaaaataacaaaagaaataacaactgttctagataaaaatgagctaagaagcctttccca
cttgtataacaccaacctctgtgaaaacctacactccacagtctacacctatgctccaaaattcacctgt
tggagcaggaactttccagcactatgccactcggccacacattcaagaactatgggaagcggtctagcaa
ctatacttctagctaaatcagtcggtatcaaaacacgtggcagaagccgcatgtgcctacagctaaaaaa
gtatgatgaacaacgtgaataccaccaacgaagaaaaagaacaacaaaatataaacaaacaagattcttc
tcccgtaaacgcaaactattccgcactgtatatctgaaatccatgtacactgatctcccatcaacatcat
caatgaacmaggaccacaactacggtttacgtgtgtgaacattamaaaatwatggtgaaagcaaacccgc
taaaataaggaaacatgaactaaagacggagtaaaaaaactgaaacaaaaaataaacatagtgaaggatt
aaactgtmaaatacaactcataccttctagtgcaggatacatcttggataccctctgtgacctgtgcact
ctccttcagctgcacatacttgcctactgttattaacagcaccaggctcatatgaaagatgtgttgcctt
cacacaaagtttttatggcataaatgtgacacatgaatttcctgtgataatgtaattcctggatttgas
agccagtatgctaaacgatgtgcagacaatctgacacgtttaccggaaaacataaatctataatatccgt
aaccataacggtcaacatcaccagtccaaagcatacagtgttcagcttgaacagacttttgcgaaagaac
taaaagtaaactttctcgagttgcggcattatgagtagccgagaaaaatgcacaagccattatataaccg
acagaatctgaaaaaaagaggtgttaatactttccactattctaaagttattaatatacatgcaagaaaa
tagctgtaaataaatactttacaatgcacttttgttgttgttgttgttgccaccaccacttacctat
ttcaatgaattcgagcaatctcccagtacatctccgagaaaccatatcttcccttaattaaaaacaaata
tctgcaaaagttacctggaccttaaatgcacccaagatgtaaccactctttacactgttcctctgtcatc
atgtcctgacactgataaagccacaatagaaatttgtaagaactaaaatcatacctgccacccgcgaaat
atcttcgaaagaaatatcaccataaaatccagttccgascttgtgaaacagccatgtatttacaaaatg
ccagaaagttgcatttatatttcctttaaatagtccttcttcctttaaatttgatataaactacaaaaaa
atgtgtttcccattgaaaattttaacaaaaccctacactacaaacagaaataagatttacgtacctggta
aaatcagagttacctccccttaacttctaccatatcaaacaacacctgtctaagctatgaatccattcag
ctcggatttataaataacacatgctggagagcttgaacaacacaaaagaaagcatgaaatagtacttaca
aaaattgacagatgtcttaa
>KolobokP-1_CorFlu-I
tataagaaaaaactgtttttcaacatattgtttttttcctcattttcattaacaataagatgtccttgaaa
aatatttcaagtttcagcttaaaataatgaaaaataaagtcgcaatgatgaattttctaacaccatgtat
tctcgtgcaagtttgcggcaaaagcggctaaaattgagattttatgccttttgtacaaacatgcataaaa
attaaaacgcgtaaaatatctcaaataaaatgtcattaatacatgtaagacatctcaaatgtgtaaaata
attcacttttttgagcttacatatgcatctttaagtgttaagggtgttttatgtagcatttttttaaggcaa
aaacaacatttttttctgttttgaagctgattttggagtaaataattttaattttactgatgtttacact
ttttgatgtgaaagcattcatgcaacaagtatattcctcaagtttaagtcaggaaatgccatgttgtata
tgccaatttaagccagacagacttagtgatgacccctaccataaactcaaaaggtgaggaatttcacagg
ttggttcaccaggcctcataaaactctatataagatgtaaaatcattatgaatggcactactgcattcat

gctattgttggtataatatataaaactaaactaaatcaaaagtgttcataaaacaaaacaatttaaagat
gggtagaacttggtcgcgccgcaatctttgcaaacggattggatttaaaaaaggtgttgcaagtgtaaac
aaaggtacaaaatttgtttttgagcaacattcaacaagtagaaaatatgtcagactgtacaaagaacagt
ttcaaaacagaatcataacaagtgaaggtactctcacctttaaagatgtagatggtactgacacaggtgc
tgtacccttgcggcctctcactgaggaaactaaaccaatagatcactatgttgaaccggaagacactaat
gtacaccctgatttgctgactatgaaaccgtattgccccaaactggttcaagatatgttcaattctgaaa
tacaatcacacaatgattccaataaaagctgtactggcagattagaatttgatggtacgggttctcgcca
aaggactcttgcctttgccgaacgtctaaaatgtacaaaatgtacttatgtcagtaaatattacaaattg
tatgatgaaattgtaactggtaaaagaggtaggagggctgcaaaaattaatgtaggtctacagttgggtt
taatgacaactcccatcagcaacactggtgctatccgaatcctagctcatgctaacatgataccgcctca
tagaacatcaatgcaaactatgtcaaacaaagtttctgctagtgtagaaaccttaaataagcaaaatatg
catgatattagacagaatcttagggaacaaaatgagaaatgtggaaataaaaatgcctctaaagttaacg
ttgaaggggactcttgttataacaaccctctattcaattctgacagcacgccttttcaggccggcacaat
tgttactacaaccatgtgtgaaaataacactaaagacaaaaaagtcataggtgtttttgttggtgtaaaa
ctgtgtaaaacagcctctaaacttaaaaatctaggtaatagtgtacaatgtccaaatcatcctggtacct
gtacggcaaatattgaccaaacccaatcaattggcaatgaagcaaaatggaatgaacatgtaactaaaga
tataaatgcagagctcagcatctccagctatactggggatggtgactccaaaggtcatagtggggttgac
aaagggcaaaatgactctgttatccaccttaaagatataagacatttgggaaataccttaaagaggcatc
tcaataaagcaccatttagcaatacaatgttcagcggaaaactaaaatctaacctgaaaaacagatttgc
aatgtccattaaagcaaggtgtctttctgaacttaagatggcccataaacaatacaaaggggatgtaaac
aaaataaacactaaaatgccagatgtcatcagtacaattgtgctgtgctttaagggttactgcggtgaat
catgccgcaaacatagtttagtgtgctatggaaattaccgtcaagtcaagtattatttgccgcaaaacta
taagttaaaaatgactgcttcggatgaaaatatactgtatgaatgcattaaagtgtttcttggcccacaa
agtgttatgaatactagatttctgtcatccactcaaaaatgtgaggctgtcaatagggcatatcaagcgt
gtatgccaaagtcagtaacattctcccgaaactgccatggccgtattcacggtcaaatactgcgtcttaa
ccatggctttgccaactcaacagtgttaaaaacaagtgccctaagatgtgcattaacaaaaggttcatcg
gtcataaaacgccttcttaaaattgacgaacaatacaaacgcattaaaagtgagcggtacattaataaac
aaaaagctacaaggttcgcataccgcatgcgaaactatcaagttcattccgaggtacactataaaaaggg
tatttctgatccaaagccagattttcacaaatcaatcatttaaaagatcatgcatatgtatgattaaca
tggccatacatactaaaatagtgctggttagtgtaagtaaaaaccaaacaagcccatcagtggccaggag
ccgggcagtttgtgttatacagcttgttctgtacataatgtaaaatactgtaaatttaagtggtttttc
aattatttaataaatcataaataaaaagtaccaaagaatgcagtctggaaactgggtatcatttattta
tggccgctacagatgtcaccacacatctgcctctcattgttgagagactgaggttctaaacttaaatgct
caaagttcacacacaggctaaaatgacacctatgtgacacatgaagcccatttgggaggtcccacatatc
agacccaagcatgtaggctagcctatgggtataatacttcttgctgtatggttcattagggaattttaca
ttaagacggccatactgtggggattttgmcccagaccgacccccaacccacattatacaaccctttcggc
caaattcaaccgagttgttttgaatttttaatttaagatttgctttaaattggtcaaaatccatttctag
ctgaaaataaaattgcagacgaccaaaataatcatgtgctgccctctaccggtacttattata
>KolobokP-2_CorFlu-I
tatttaacaaagaaatgttttcaaaacactaaaagaattcatctacacaatattcaatgtatactgagct
tggggccaagttggagcatgattgcctatgaattatatgaatgacagccttttttctagagcatgtgtt
tacagaattttcagtataaaattgccggccgaatctgtaaaactccaaaactcattctgtcataactttt
ttatttattggaatttttttcatgaaataaaaagcaatgaaatgtactcaatagcacctatcagaaaatgt
gtttcaataatgtgtttggccttcaattttttttttgttttaaattaaaattttaattaaaaaatttaaa
taaaatttaaaatatttcatttaatttcagaaatatttcaaacctagacctgtcaaatttacttcatatt
taaagaacattttataaccattaaaatgaggtgtcacttataagttttgaaaaatttcatgcagaaatat
gacagcttaaggaataccactgtatgtcctcggccgggcaaaagtattataatgccaaaactaaaatgtt
aatatctttcttatttatttgattttttttcaccaaataagaaacagtttctcttatacaatagtaaaaat
cagaaaatgacaccaaaaacaaattttgccccaaaataatttatttacaaatttttcttgttatttttat
ttaaataatgtatttaaaaattaattttatatgctatataaagttattttttgaaataaattattaaatca
aaaatttaagtcattatcttctttaacaagattaaatgggggcttacttaacagtatacaataaactaat
caagagataaggctgtcaaaacagtgtctggggtccaaatatacagggtcccaatatacagcattccatt
gcaccaccccaatggactgcataccagtgactgtggcagtcttttgatgtcttacttcaaataaattcct
tggaagcttaaaatgccaacacaggaataaaaatggagctgcatgcagcatttccttcaaacaacactta
ctgtcagtctttgcacatcatatgaccagcaatgaaaggagacaagtcaaggaaggcttccaggttcaag
gcacataatgttccatggaatagaggcttgaagatggccagttacagctctgaagataccaggcagcaac
tttctactaccagaatgactgcagatgagttctccatggtatccaaacccagtgcagatggaagaagctt
taccactccagactttgagggcaaccctgggaatgttcgtctgctgcgaccgaagtccaagaagacatct
gacctggcatcaaaagaagatgacacattaattaccggcatgagatttatagacattgaaaagaactcgg
ctgttttcaatgaggtctttcaaagtcaccagcaccaatctaaagactgtatccaggctaatatgactgt
tgccgaagagcataaagtgggcgtctgcgtcaagttttctctgaaatgtacaaattgtgattacatttca
ccagtaaagaaattgtacaaggaggcaccatcatcgaaacgtggtccaaaaccagcagcagccaacatcg
cgcttgccacagccctacaggacactcctatgggaaacacaaaatgccggtatctgctagcaaacatgga
tgtaccgccaccatctagaacgagcatgcagaggacatcaaacagagtcagcaaagccatcaaaaccctg
aatgacagagacatgtctgcgaaagttcagcttataaaagatgtcaataacaagcgagggcatgccgaga
acataatcaatgttgccattgatgggcgatacaactcgacaagtataactagtcggaagaaggcagggca
aaatgcttcacagtccataggcatcgcctgcgaaacagttactgggaagcagtatattatagcctcagca
ctgcaaaacaagctttgctggtctggtgcctggctaagaaaccagggttttgaagtgacatgtcccggag
gacatgaagactgcactgccaatattcaccctcaagccccattgtctgcttggaagagaaattggagagc
agcttagagtccaaggggccttcattaaatatgcaaccacagatggcgattccaggtcagcagccggggt
agcacaggcaatgaaaatactggacccagtgtggaaagttgagaggctagcagacccaacacatcttggt
caggcacagttcagacaatgctacagtgccaagttcagcagtgaaatgttctcaggcaagactagggaga
agagacaggaacaacaaagggccttgagccaagatgtcaaagctcgaagcagtcttgttctcaaggaact
cacgaaaacgtatgcgggagacataagaaaaatcaaaatggagttaccgaatgtgctgcgtgcaacggtg
agctgctatgctggagactgttcaagctgcaggcgacactcttacgtatgctctggtggggcttcaaaca
actggtggaacagatccatgtttcttgccactcacaaaatcacaaatctgcaaatgaatgacaaagataa
acatctacttcaagaaatactgaaaatgaaactgagtataagtgccttggatgagatgaaactgggaacc

agcacacagaaatgcgaagcagttaaccggtcaatatccgtgtccctgccgaagaatgtgaattattcac
gtaatgttcacggtcgactctcatcaaccattcatcggcttaacaatggactttgtaacagtgcagtcga
gaaacttagctgtatgggtgttaccttgtcaccgaagccgctaagatccctgaaaaagatggaacaggaa
caagagtaccaaaagtcctatgctaaaagacctgaaacagtgaagagaaggctattgtgtaagggccaac
aactgcgggaacatctgaaatataagaaaacaaacaaaacaacctctgactacagcaaaggctgtttgga
cccggtaccctcaacatcaaggggaggaaactgtgaacattcctactctcagtgacagcaagttatcacc
catggactttttgaaaaacataaaatgaaacaaaaatcaaagtaaatgatactattactggacaaaatgg
acttataacatgaataagtgagattatagtgaatcaaaaactattaaaatcaagaaagtatcatctatct
tgcatctgaccattaattgttgaataactttttgtttatgtttacagcttttctgattttgttaaaaatt
gaataaaatgactgaaattcatgtattactgttgttatttcatgcataaatccttaatgtccaagcaata
ccagtaaaaatgcgacaagttctcttcgtcaaaacttagaccggttacaactggcaaacccggttacatg
agaactaaccgattttacgacaaaggtatggtaatggttactttcaaggttgacatggtcatggtcactt
tatgtttttttagtgacatgatgaccatgccaactttaaaatgaccatgacatgccaccatttcaagatga
catttaagggtctttcgacatcttgacgacaaacgacttaaattatttattcgtataaacaatggcacga
tggcacgacaggatgactactctttaaaccacatatcattacctatcaattttgaggtaggcgacaaagc
gcaaatggatcatgcccccacaattgccccggtttaaacacttgagtctctctgtgttgacagaatgtg
gctccaggctcaaatgctcaaatgcaatgcacaggggcttgtgacaaaggtgggaaacctcatagccttg
ttccaaggtcaaggtcttactagacacaaggtatctaagcctatgggcatgtacagtatgccatccatct
gggaatttggcctttattacaccataaccaataggacctttcttttgacagccctgccaaattctgcagc
cattttcaacaacaacagattttcattcaatttagattcatattttttaaaaagccctccattttaac
tgttaaatccaaagtgaggtttaccaatttcaaaccttgaccccttggtttatatttagaccaaaaatga
aagtagaatagccattttccagatttgaacaaaaataatatataatgttatataataatatatatattat
ataataatatacataataaagcaatcttactgcactccttgatttcatggcctcatatctatcacatgtc
caaatttcatcaaaatatctttataaatgatgaagatatttacattttagttcaggcacccccccacccac
tttcacagtatatca
>KolobokP-2N1_CorFlu-I
tatttaaccaaaaaatgttttcaaaacttaaaaagaattcatctacacaattgtcaatgtatgctgagca
aagggacaaagtttgagcatgactgcataaaaatcaaatgaatgacagcctattttctagagcatgtgtg
cacagaattttcagtaacaaatggccaggccatttttgtaaaaatctaaaactcattatgtcataactttt
ttatttattagaatttttttcatgaaataaaaagcactgtaatgtactaaatagcatctaccagaaaatga
gtgtgaataatgagtttggccagaaattttacttttttttaattttaaaattttaattaaaaaaattaaa
taaaaattaaaataattaatttaatttcataaatatttaaaatctgtacctgtcaaatttactgaatatg
taaaggatattttataaccatgaaaatgaggtatcacttgaaagtttatcttaattgtatgcaaagatat
gataacataaagaatgcccctgtatgtctccggccgagcaaatgtattaaaaagccaaaactcaaatctc
aatatctctcttatttgttaacatttttttcaccaaatcaaaaatattctcattcattaaatagtaaaaat
tggataatgacatcaagaattaattttgacccccaaaaaattattttcatgtttttcttttttttattta
aataatttattgcaaaattcaattcatatgcaatatgaagctgattttaaaataaattatttatttaaaa
acaataaatgactatcttaattagcaatgtataaaatagatggcttagttaacaattttcactactctga
tcaagatataagaccaagaaaacatctgctactgtagagtttctggggggctaataacctagcattcaatt
gcaacacaccaggtgactgcattatcagtggtgttctataatgtcttcttcaaatacattccttggaagc
ttaaaatgccaatctaggcataaaaatgaagttcattgcagaatttatttatttcatcacattattcgca
acttatgcccagcaatgagagacaacaagttccgaaaggcttcaaggttccacgcacaaaatgatccatg
aaataaaggtgtgaagatatccagttctacctttgaagatactgtgcagcaacctcctgatactggaaag
actgcagatgagttctctttgttatcatatccaaagcaatagtggatggaagaagcttcacaactccaga
ctgtgagggcagttgttggactgtttgtatgctgcgacagaagtcatctcaggaatcctacctgacatca
aaagaagacaacacattagttattggcattacattttagacactaaaaagaactgtgttgttatcaatc
aggtcctccatagacaccatcatgtatctaaagactgcatcctgaccaatgtgactgtcattgaacagca
taaaccactcctactcccagtgaacagtagatatatcagtcccattgagtttctaaaatcatcaaatatc
acaaaatttttaatatatgctacaattactgctcaaaattgatttatgtcatgaactggtaagataaaag
tgatccgaacgaatgaaaattaaatcggaagtatcaaatctatcgcctcggacaattacttatggaatag
taagctttgtgtgtatttgtctcttttaagtttgacagaatttgttaaagcttgaatacaatgactaaaa
ttgatatattattgtttttattaattaataaaccccaaatgatccatcactttcagtaaaaatgtgatag
ttactcaatatgcttgtcaaaactaaggccctttttacatgttatatgcaaaccgacccattttactgaa
ataacatggtcatgaaccatcactttaaaggttggcatggtcatggttactttaaataaagcaaaaagag
ttcttttgagtggccatgagcatgccaacctgtaaagaggaccacgacagtgcaaaccattcaagatgac
tacaatgggacttgtcgcgttttcacgacagacgacttactgtatactgtttaatgatataaacatagac
aaggcaggcggactttctgtaaaaccattcatccttacttatcaacttaccccgggtagacaacaaagca
caaatggctaaagatccccacatttgccccagtttaaactcttttgtctttctatgttgactgaatgtgg
ctccatacaaaaaatggtctatggctatatagtagcccaaggcaaacaaggtccaacactggaaacatac
aagatagacctaccttagtctatgtgcaggcattgtgtaccatccatcagggaatctggccttgattaca
ctaatttacaccatgggtccttccctttggcagccctacaaaaaagtatagcaatgttcattcattggtg
gtggagttttcactcaaattagtggcatatctctcttaaatcccttcattctaactatttaattgaaatt
gaggatacaaaacttaatacctgaccccttgtttacatttagaccaaaaatgaaagtaaaaatagccact
tttcaggtttgaactaaaataatatataatataacaaaataatatatattattgtataatattatataca
gaaaatcaaaataacaccactgtctgatttcatggccttatatctgtcacatgttcaattttcatcaaaa
tatctccaaaaatgatgaagatattgacatttgagtttgggcacaccccacccacatttacattatatca
>KolobokP-3_CorFlu-I
ttaagaacttgggtcttgcttaattattgaatatttaacttttctttttcaaatttgatctttttccaat
tgaaataacagcttgaatcatgtatccaatgtcaaatgtagtcagtagagcatatttacaactatactca
ttaccaagggaaataacaagaattttactgatttcggtagtgttgctgcatgaatgttttgctaattctg
tgactttcaaactatttacaaataccattatccactgtaatcaagctcagatatcatttaaatattgagc
tgtgcaaagaaaacacatcaagaagtgtatttaatttcaattttgtttaaaaaaagagaacatttatcaa
tttctattatgtttttcattaaaatgttgagtttcatgtaagaaaatatggtaatatacatttgaaaatg
attaaaacataatttctgaatcaaacagtgtgcattttgttcttttcttacacagaagactaccatctct
caaataatatggtacaatgtgtctttacaaaactcccttgtcaatacaagggagataatccaaatataaa
atgaatgaccattactatggctaattaaaaagcaaactgtatctatccagacaattcattttggcatgt
tcttgataagaaaaacatccagagcagataatgaagtattttgtcactattttagaggtctttgtagcaa

aaagaaacaaaatcttacaacaaatactaacttagtgaaaatgcggactaattcttctaaaagtagactt
tcttacaaaaattcacttaaacggagaaaaacccaatttaaaaagtgccataaagtgtggaataaatcat
taaaaagtgataaaaatgaaataaatgagccaggaccatcccataaacaggacttaccaaactatgcaga
acctgtggttagacttgaagcttcagttgctgaagatgtttgcaatgctcaacttggatactctacactc
tacaattccccgtaaaacttagacctaaaaagaagaaagaaagctcaaaagatgtaaatgaaccgcaac
atgttggcgaaaatgaaaacattattgtgaactttaaacagcttgaaagtctattccaaaagtttgcagc
acatacatgtgacaatccaaatataaaattaacactcgaagacagacaaggtctatgtgtatcgctaaaa
gttaaatgcagcaattgtaaatttatcacggatacttcaaaactatttaacagcataaagtcaacaaggg
ggccggatgctggctgcttaaacagtgcacttcttctaccggttttactgtcaaaagttggcatcaatga
cattcttcttatgttatcggcactaaatattcaagcaccaaacaagcgtggcttacaaaaaaaacttaac
accctatccgacaaaattgaaaaccttggaaaagaacaaatgatacaaaatcaacattatgttaaaaaaa
tccagtcactatccggcacatcagggactgatgttgagtttgatgttgcttataccagcaggccacagtc
tggatgtgaaacatcaagtcaaagttttgctcccatcattgagcagacaacatccagacatttacctata
tatttgtcaacagctaataaattgtgtttaaagcaaaagtgtaaccataataaccgaaattgcaaaaagt
cgtatagcactgaagattccatcgcaagtacagaggctgtgtttataaagtcagcgatcgagtcggtgga
gtcttcgaaaatactcaagatccgttcagttacaacagacgcctccctccagattgcaaagtcattacgg
gaaataaatagtgaaaggaattctaaaattaatcattataagtgttttgtgcataatttaaggaacatga
acaaacatttaaaggccattaaatttaataaaattcctccaggtcaaaacaaaaaatattttgcatcaaa
attagcttcaagtattcgcgtcagagtccgcttagaacttacaagacttaaaatttgctccaaaacatat
gaagaatttctacaaaaagctcaggtatcaattgaaaatatactgccatgtttcaagggcctgcatatca
actgtcgaagagactctgttgtttgtaaatctcatctaaaatgtttcaaactcagctacttgccatatgg
caaatatctcaaactgcctgattctgacataagcaaaattaaatccataatatcaaaatactgtggtcca
gcaatgatcaaagatataagccagctgaaaacaaccaaccaatgtgaaagttttcacaaccgggtatttt
cacacgtaccaaaaaacacagtatggagtcgcaacttcagcggactttgtcactcagctaccatccgagc
aagtataggcagaggaagtaccttgttaaaaatagcccaaaaccttggattgccagtatcggaccatgac
ccattacagcggtatgcacttgataccgacaaaaatgacaaatatcatatgaacagaaaaaaatctttca
agtataagacattccttcatttgaaacgaaaaagaaattcaaaccgcagcataatctcccagtcggctta
ttcggacgccacagatttaacagaagagcacccatatgcgttaaatccaaactaacactgaactcaaatt
gtaatggaaagagaagaaaacacaacagtacttttaactagatcttacctcacagaaacagcttggaca
tgcaaaatgaccatggcaataaccgtcatttctacatttcattctattgttattgattctttggctttca
tatgataaatgtgtgtatgttatgcaatttttaacatgacaaatatgcgatacatgcatttctgggctta
aatatcttgattcatgcaagaaaaatacaagccgatgaactgcaacttttatccgttttgcacggaacat
aaatcttatttgaccgtagccgtcccggctcatctggccagtccatataaaacaatgaccatcaaaccga
gtgtgttttcttacagaacgctcaattttagacttttcactaggatttaatgaacaacggaaaaattcca
tggacattttaacacaatacactgaaaaacattatttaaattgtgaaaaaacattgttcagtatgacaag
tgcacctgcatatatgcatactttatatgatgtgataatgttcatataccgtaaaatggttcagtgtttt
ttgtagtttttttactcaaatttcttaaaattaacccaaaatatttatctgctattttacgcatatacaa
aatctgcaactgtaactgctatcaacatcaattcagtgagtaccatgggaacatttacgtatttttgatg
ttcataaattcatccccatcgcgaaaaaataccatcttacggtattaaatttgtgtaaaaaaacagcaaa
taatatgtccaaattaattaaaacaccaagaaagatgtaatattcactaaaataaaatcaattttcccaa
ataagaaaatattgttttcgccttaa
>KolobokP-4_CorFlu-I
tataagagaacttaacttttaaaaaaatctctatatttgttctacacatagctgcaccttttctaagaa
atgtctaaaagttttaaagcattctgatcatccattccatgaaaaacaccatttctatacagccactgtt
catggcctcggctaagcgtttttaggctcatatttgctgattttatatatcgcaacattttgggtgctat
tttaaaaacgcgttactttttcatgtcatattgactttttgattgcataaataattactttatatgatt
acactgcaatagtctgattattttaccattagtagccaggcaagtggaattaattcaagtttttttaaa
aaaatggctgtttttttttcaaattttcacattcacctcaaacttattaacccaaaaatcacaaaatactt
aataaaattcacttattttatcaaatcaatataattacaacaaactttgatcagtagcttcaataattag
ctgatttgagtgcccaatccagtcaattaaaggcaattagctaattagtgctgttaattgcaattgacag
caccttaacaccaatttgtgattgtagcaccttaatataacaagttgcctatataatgtcagctaaatgt
tcctaaggtgtgtgtgtactccctaagctacactagtgtatgtttgactctcaaatatggtgtcttatag
gaaaagaaaaagcataggggctaaaactgggttcaagaaaggccagccgagtgctatgaaagggaagtca
tatgaatatgtggaaactacctctgaatccaaatttgcacgactggctgaacaaacttactatgacagaa
tagttgaggaaggagaaattcttactttcaaggatgttgatggctcaaatacgaatgtgaaaccactccg
cagtctgtcaaaagccaaaactctgcttgaggagtacacacaccatacagttgactctggttactaccac
ccggacttgcttgttaacaagatttacaggcttttggatgtctgtcttatgttcaattctgaaatgagaa
agcacatgtctttaaagcctcaatgcaatggatatctcatgattgatgctgccaactcaaaggcttgggg
ctgggctgccattgaacgcctattctgtaaggaatgctcatatgtcagcccatactacaagctttatgag
gaggttgaaactgataaaagaggcaggaaagcagcacaaattaatgttggaatacaactgggacttatga
caacaccattatccaacacaggtttaagccggatattggccaatgccaatattccgcctccaaatctgac
ttcgctccaaaaaatggcaactcatgtctcaacctcagtaacagagcttaatgaacaaagcatgtcacaa
ataagacagcaaattaaagcagataataaactgtgtggcttgtcagacccatccttggtcaatactgagg
gtgacagttgttacaataatcctctgttcagctgtgacagtacacctttccaaggaggcactattgctgt
cactactatgtgtgacaacaacacaagatcaaagcctgtcattggggtccatgtggccagtaagctatgt
cttgtggcaagcagacttaaaaacaaaggcatcaatgttgaatgcccaaatcatgctggccactgtacag
ccacaatcaaagagtcagactccattggcaatgaggcaaggtggaatgaggctgtcacccggcaaataaa
ccaggacttaaatatagcctgtttcactggagatggtgactccaaaggccatagtggagtagacagaggc
caaataaagcctgtcacacatctgaaagacatcaggcatctggcaaattctatgaaaagagccattaata
aggcaccattcagtaaaggcatgttcaacgcaccgccttcacataagactaacatgcgcaatcggtttgc
tttgtcaatccgctcaaggtgtaaagctgagcttaaacagtgtcacaaaaaatacaagggtgacatacac
aaagtaaaaaaggatatgcctaaaatagttagtgccattataatgtgctacaaaggctactgtggctcat
catgtcaaaagtacagtcttgtatgcaatggacagtttcacaatgcaaaagcctacatgcctgccaactg
caaaattaagatgactgtttcagatgaggctgtactgaaatcatgcataaacatgctattaggcccagac
agcattgaaaaaactaaactgctgacatcaacacaaaaatgtgaggcagtgaatagggcatatcaatttg
ctacccccaaacttatgaactttgcccgcaatagcactggtcgcatccatagcaccattctaaaactaaa
tcttggctatgctgactcagtattgcttaagtcagaaaagaccggagcaccactgaaaagaggctcatca

gttatccggttcttgcgaagaaccgagcaaagagctgcacgactgaaatctcagcaatacaagcttcgtg
cgaggcagtcgcgatatgctacacgaaagcggcgattcagcctccactcaaatttgcactatgccaaggg
tatggcagatccaaaaccagattttagtgctataccacacttaagtgaccactgttactccagttaacag
tgtgcttcacaaacaaatgtgcttgtattcatttgattggcatattctgacttgtcggactggcagttct
caccaacagaatatgccgacaaatgaattgttgtatattgtgtaaatagtgtaaatagtgtaaatatttt
atagttgtttttatcatattttaaacatttaaataaagaaataaatacttaccaaaatatacactttggc
aactcctccccatttatgacatgccccaaacaaacagctctatccctgcaggtctgcctttggttgttca
ccacctgaggctctaaagtcaaatgactgaaattgcaacataaagaataatgacacagatgtgacacatg
catgccatctggtagtctttccatgttctggtgtatttgatagactagtctgtgaacataatagtatctg
ctaactgtgtcccctggaaatttaactctcattctgccatacctcatgcccttttggtccctaagtgtcc
caccccatctaatacacccccctgttaccaaatacttcacatttagattcaattcttctttgcaatgctga
taaaaagtgatttaaagacattctgactgcagacgaccaaaatagacaggttcccggatgttgtcatttt
ttcttata
>KolobokP-4N1_CorFlu-I
tataagagaacttaatttttttttaaatatctctatatttgttctacacatagctgcacctttttgtaagaa
atgttttaacaattttaaagcactcagactacccattccatgaaaaacaccatatctatacagccagtgt
ccatggcctcggccaagcgattttaggctcattttatatatcgcaacattttggctgccattttaaaaac
gcgttactttttcatgtcatattgactttttttgattgcataagaagttactttatatgattacactacaa
tagtctgattattttaccaaaggcagccaggcaagtgcaattaattcaatttttttctaaaattgctgac
ttatttttcaattttcacattcacttcaaacttttaacccaaacatcacaaaatactgactaaaactc
acttattttatcaaaaataatattattacaacacactttgatcagtagctttaataataagctgatttga
gtggctaacccagtcaattaaaggcctacagtgctgttaactgccattgacagcaccttaacacctatgt
gtgattgcagcaccttaacataacaagttgcctataaaaaagtcagctaaatgttcccaaggtgtgtgtg
tattccctaagccacactagtgtatgtttaactcacaaatatggtgtcttataggaaacgagaaaaaaca
taggcgctaaaactgggttcgaaaaaggccagccgagtgctatgaaagggaagtcatttgaaaatgagga
aactacctctgaatccaaatttacatgcttgcccaaacaaacttactatgatcaaatagtagaggaagga
ggaattcttacttttgaagatgttgatggttcaaataccaatgtgaaaccactctgcagtctgtcaatcc
aaaactctggttgaggagtgcacacaccctaaagttgactctggttactaccaccctgacttgcttgtta
acatgatttacaggcttttggatgtctgtctaatgttcaactctgaaatgaggaagcacatgtctttaaa
gcctccaatgcaatggatatatcatgattgatgctgccaactcaaagcatattctgacttgtcggactgt
tagttttcaccgacagaatgctgacaaatgaattgttgtatatastgtacatagtgtaaatatgttatag
ttgtttttatcatattttaaacatctagttatttaaataaaaattaaatacataccaaaatatacacttt
ggcaacatctccccatttatgacatgccccatacaaccagctctatccctgcaggtctgcctttgattgt
tcaccacctgaggctctaaagtcaaatgactgaaattgcaacataaagaataatgacacagatgtgacac
atgcatgccatctggtagtctttccatgttctggtgtatttgatagactagcctgtgaacataatagtat
ctgctaactgagtcctctggaaatttaactctcattctgccatacctcatgcccttttggtccctaagtg
tcccaccccatctaatacacaccctgttgccaaatacttcacatttagattctattcttctttgcaatgc
tgataaaaagtgatttaaagtcattctgactgcagacgaccaaagtagacaggtacccggatgttgtcat
tttttcttata
>KolobokP-5_CorFlu-I
tttaaggtttttttttcttactttcaaattgcatatatagctcttattcttttaatttcatcttttcctgg
ttcaacaaacaacttaaaacatgtataaaatgcacaatttgttcaaaacagctatttccaaagacatatc
atattaaagggaaataacaagaattttactcttttaagcagtgtggtagtttgtatgtttagtatcttat
atctctttaaaaatatacgaaaacatattcttttccactgaaaacaattacaaatgtaattgcgcggtta
aatatttcaaacgatccccaaaagatgtaaataaaataattattgagtagaaaaatccttatcagttgtc
tattttcaatagagattgaattcaaatataagttatatgtatgaaaaatggtatactagccctaaaaatt
attccagcaagtaattcctacttttagatataatcttttgttaatttaaaacatggacatctgcttaagt
caactgagtgaggtgctctgtgtcttttgaaaatccctgtgtcaatgcaggggagattatccaaatacaa
aatgaatgaccattactctggccaaggaaacaagcaaacaatatgatcccacagaaatctgatatggcat
cttcttgataagaacaacatacagagcagttaataatgaccacttgtcacttttatcttactattgtagc
aaaaagaaacaaatacttacagaattaacactattttttgtaaaaatgcggactagaagttctaaaagtag
gaattgttataaaaactcacttaagagaagggaaacaagttttaagaaatttaataaggcatgcaaaaaa
acgtgtaacagtggacaaaatgaatcaaatgtacagggaccttcaaatgagtatgaacagatcttacctg
acaactcaaaatctgtgttcaccagacttgatgcttcggagtttaatgacgtctgcaatgctaaccaaag
tgatgctggatgttacacttcccccatcaaactgagaccaaagaagaggaaagagagctcaccagtagta
cagcaacctggaagtgttggggaaaatgagaactttattgtcagcttgaaaaagcttgaaagtatttttc
aggcatttgcatttcacagctgtgacaacccaaatgtaactctttcacttgaagacagacaaggtctttg
tatttccattaaggttgtgtgtagaaaatgtaaattctctactgacgtaacaaaaatgtatgatgtcatc
aagccttcaaggggaccaagtggtggatgcttaaatagtgcaattttaatgccagtgatgctctcgaaaa
ttggcatcagtgacatttacttgtgttgtcagcattgaatattagggcaccagacaagcgcggcttaca
acgaaaactcaaccatttatccgacaacatagaaaacatcggtcgcgagcaaatgctacaaaaccaacag
tatgtgcagcgcatccagtctctagccggtatgtcgggtacagaagtcgaatttgatgtggcatatacaa
gtaggccgaagtcgggctgtgaaacatctagccagagttttgccccactaattgaacagacaacaacgag
acacctgcctattgccatttcaacagcaaacaaattatgctcgaaaccagcatgtgcccacaacaatgct
caatgcaaaaaatcttatgatgcggatgtgtcaataggtagaactgaggcaacattcataaagtcagcaa
tcgattcggtggagtcaagcaatataataaaaatacgctcagtgaccactgatgcatctctgcaaattgc
gaaagctttaaaggaggtgaatagtgcaaggagcatacaaattaagcattacaagtgttttgttcacaac
cttagaaatatgaacaagcatttgaaagcaattaaattcagtaaacttccgcaaggacaagacaaaaaat
tcttcgcattaaaattagcatccagtattcgagtacgggttcgactagaattaacacgactgaaaaatag
ctccaaatacaatgaagtgttcttacaaaaagcacctatgtcaattgacaatatattgcaatgttttcaa
ggaaagcatattaactgtcgcaagcactctttagtttgtaaagctcacctgaaatgttttaagctgaatt
acttgccctatggaaagtacctgtcacttccagattcagatatttctaaaattaagtcagtgctcgcgaa
atactgtggtccagtcatactgaaagacctatgtcaactgaaaacaacaaatcagtgcgaaagtttccac
aatcgtgtttttttcatttgcaccgaaaaacactgtttggagtcgcaacttcagcggactgtgccattcgg
taacaattgcagcaagtgtcggtcgtggcatgacactgcttaaagttgcacaaaaacttggcttgcctgt
aacgattagtgacccgctatatcattatgcaattaatacagacaaaaaggagaaataccatatgaaaaga
aaaaaatcatttaaatataagacatttttgcatttaaagcgcaaaagaatatcacatcacgctatcattt

ctcagtcggcatattcggatgacactgatttaatggaagaacacccatatgcaataaatccaaattaatt
tatattcaactccaaagaaaaaacaacaaaacaacaacaacaacttaaatattataatgcaaaataaca
ataactggatcgtgtaaagccagtatatgctaaatgttgatagtattcttcaaatatttatttgttttg
tattgttaatgtatctcgaatagctagtatttatatcttaacagtaaataatagtctcatttaaatgtaa
aatgtgtaatattaaacacagcgatgaaatgtaatcaatacataagacggtaccaaatttatgtttttc
tatttgatttttagtgatttacctccctttattctgactccctacattgctgtaaatgtttttaaacagt
agcaaaatcctgtccaagttaaccaaaaagacaccagaattgttatttacactgatcccaagtcaacttt
tacacaacaaaaatcgaagtttttcttaa
>KolobokP-5N1_CorFlu-I
tttaagatttttkttcttaccctcaaattgcataaaaagctcttcttttctgattttgatctatttcaga
cttaacaaacaactctaaacatgtattgcaggcaaaccgcggtcaaaagagctagtttgacatatacaca
gtgttaaaggaaaataacaagaatattaccctcctgggcagggtgacagtatttctgtcacacgtgcact
atatccctttcaaaacaaggaaaactcgccgatttctattcaaaccaaagtccagatattacatttgcat
gttttcaaagtcaactttcgcagattaaaaatattcagttttgccttaa
>KolobokP-5N2_CorFlu-I
ttaagaacttgcgtctcaatctcaaatctcatatatccccctttatctcaagtttgatcctttttgggt
tcaacaaaccaatctaaacatgttttactggccaaatgaggtaattagagccagtctgacacctacaccc
taataaagggaaataagcagaatatcacccccttgtgcagggcgacagtatttctgttacacatgctcca
tctcccttctaaactgagaaaaatgactgttttcaactcaaactatgttcagatactgcatttgcatttc
ttcgaagtcaactttcgcatatgaaaaaatacgcgtttttaccttaa
>KolobokP-5B_CorFlu-I
aagcattacaagtgttttgttcacaaccttagaaatatgaacaagcatttgaaagcaataaaattcagta
aacttccgcaagggcaagacaaaaacaatttcgcattaaaattagcatccagtattcgagtacgggttcg
actagaactaacacgactaaaaatggctccaaaaacaatgaagtgttcttacaaaaagcacataagtcta
ttgacaatatattgcagtgtttccaaggaaagcatattaactgtcgcaagcactctttagtttgcaaagc
tcacctgaagtgttttaagctgaattacttgccctatggaaagtacctgtcacttccagatttctaaaat
taagtcagtgctcgcgaaatactgtggtccagtcatactcaaagacatatgtcaactgaaaacaacaaat
cagtgcgaaagtttccacaatcgtgtttttcatttgcaccaaaaaacactgtttggagtcgcaacttca
gcggactgtgccattcggtaacaattgcagcaagtgtcggtcgtggcatgagactgcttaaagttgcaca
aaaacttggactatcagtgacccactatatcattacgcaattaatacagacaaaaaggagaaataccata
tgaaaagaaaaaagacatttaaatataagactttttgcatttaaagcgcaaaagaatatcacatcgcgc
tatcatttctcaatcggtgtattcggatgacactggtttaatggaagaacacccgtatgcaaataaatac
aaattaatttataaacaacttcaaagaaaaacaacaacaacaacttaaatattatattgcacagtaacaa
taactgtatccgtgttaacttgatcttacctcaaaagaaacagtttggatgtgcaaagtgaccataacaa
taaccttcatttttacatttaatccaatgattattaacggtttgactctcatatgacaaatgttcatatg
tgatacactttttttcatggcacaggtgtgacacatgcatttctgggctcagatgctttgaacagtgcag
gaaaaattcaagtctgtgaactgacgttttgatccgtttgccccgaaacatgaatcttatctggccatag
ccatcccgactctcctggccggtccaaattaaacagtgaccattataacgtgtatatttcttatagata
actcaatttttgattttcctgaagcatcaagtaaactatggaagaatgccatacacattttgcacgcaat
acccaaatctgtgaaatgaaatatttgttaagccagtatgataatattcttttaatatttatttgtttt
gtaatgttaatgtatctagaatagctagtatttatatcttaacagtaaataatagtctcatttaaattta
aaatgtgtcatattaaagacatcgatgaaatgtaattaatacataagacggtaccaaatgcatgcttttc
tatttgatgtttagcgatttacctccctttattccgactccctatattgctgtaaatgtttctaaacaga
aaagaaaaacatgtccaagttaactaaaaagacaccagaattgttatttacactgatcccaagtcaaatt
ttacacaacaaaaatctaagatttttcttaat
>KolobokP-6_CorFlu-I
aaaggaagttttaattacttatttttttttgaaaaagaatcccttaatataaagaacatatatttatcctt
taatcaacactgaatttttttgtttttgaacaaagcattaaaaaattacacccttttttaatagaagagggg
taggccaagatattgcactttttatgcatgtaaaagaaggggaaaaaatttgagataccaccagtctaaa
gaaggctgtaaaaccattatttgatgtccaatctatacaaaatttgacatgaagtgaatatgtaaatcct
tcttaccaaatacattaatttgaaaactcaaaaatgaaattttgaatgattttaattaaatttaaatttt
aaaatcattatttttttggtaaaaatctgaaaatattgtcattttctcacaacagtatttcacattttga
tacattttatagaagaaaagtaatttttttgaggactaattaatataaaatcataaaaaatgctttatctt
ggcaatctgggatgtttatttgcttacagatatagattatcaagaatgacagaagtgaccaaaatgaaga
ggttttatacacagagttgctacaaagcaacataccacattataattgaccaggcatataaagtatatga
agcagcagtgtgcttatactggagttttaaacaaaaataatataccaaacaaagtgaatattgtgaaatt
taatgtgattttaataacaaacatggggaaaattaggaaaggtagagctattaggaaaggtttgcaagca
aggaaggcaacccaattcaaaaagggtcatatctctcctatgaaaggtgtaaacatggaaagagaggcat
ttccagatgaaaaggtggtctatctgagaccatcaagttcagagtatgctgcagctaccagtgagcctat
tgaaactgaggcactggcactggatgacccctcagaaaccactactgcaacatccaccaagtacaaattg
ttgcggccgactaaggataagaagcctacaactgatgatgtagatgaagagttctcaaatttggaatggt
aaatatatgtaactgatattaaatgataatttggatacattttaatgcaataaaagtaatatattttgtt
aaaaggcagtgcaatatagataaataaggttcaggggtgcggtgctgctgtatacataatttaaagaata
aattttgaaaagtaaacataaaaatacaataacaaaaacaataaataaataaataaataaataaataaat
aaataaataaataaataaataaataaaattaataaaataaataaataaaataaaataaataataat
aaaaatctttataattaatacacatacagttatatgttagtgaaagggaagtaactgcaaccaaatattg
gtgactgcaaatttctactcagttcaccatgcatttagatttttcttcttaatatctacattgagaaaca
atttatgtattttagggccattaaagaaggatacagaacgtttcacaaaaataaagttatggacatgatg
aatgaagtgtttagagaacacaacaactatggagcagctccctgtcctggtgctttgcaatttgacacaa
gtgaagagataaaatggggtttcgcatggttcgagaaggcaaagtgtgaaaaatgccactactcatcaaa
agtctacaaactgtttgaagaagtaaaaactggaaaacgcggcagaagagcggcaactatcaatgtggga
atatctatagcaatgacacaaacacccattggcccgacaagtttgagacacatatttcacggaggcaata
ttccagccccatcaaggtcaggcatgcagcaaaccgcaaaaagggtagcttctacaataataaagacaaa
caaagccgatatgaaatgtcgcactgaaaaaacaaagagaattctccgtatgcgtaaccggccggacaat
gagtttgcagtacaaagtgacagtgtctacaacaacaacttgttttctgctattggaaaaagtcctttc
aagctggcactcaagctgtttacactgttgcagagaatgtaacttgtaaccatgatataatagaaataga
aactgttaataaactatgctcaaaagaaggataccatgatagcaacagcatgggaaaatgcaacatcctt

catagcagttgttctgccactcatccgatggaggacaacattggagatgagaaaaggtgggccaaaagta
cattccaacgactaaaagaacaggcggttgaagtaaaatatctgacaacagaccccgacaccgctgcgtt
taaagcggttgaagagatgcatcgcagcggtgaaacaacaacaaaaccaatacaccaaattgatacacgg
cacttatcaaaaaatcacagaaaattcatcagaaatagtgaaaaagttcttaaaatgatgcctggacata
cagtaaaattaagaaaggaacaaagaaatggatttgcatttgatttgagtcaaagatgttatgctgaaat
taatcaaatacaccagcaacaaaaaggacaatttgacaaaatcagaatgaaagttgaaaaatgtattcag
gctatgcttaagtgttatgctggcaaccacagtgactgtaaagagaacagtgctgtttgcaaaggtgaat
caaataataactggttctgtccaagcaccatttacctaaatgctagcttcaaaattaatacagacattca
caaacaaacattttttagactgtgtaaattatcgtcttggaccagaaagacttatactcacacgtcttaat
acaaacagtcaaaaagttgaaggaacaaatagggcaataaagcgatccttacctccaaatgtgactttta
ctcgttgctttgaaagccgtgcacacagtgctattcacctggtaaataatgggccgggcaggtctctagc
gaaacttcttgttagtgctggttgtccgatttgtccgaccggaaaggttgcacgggctctgaactatgag
caacttggcactgaaaaaaagaaagaccgtgaaaaaactctggaatataaacaaaagcgtaaactacgta
aagtaaaaatctataatttgtacaaagctaggaaagaaataaagaactaccaacggggaatgttactcca
catgaaagctgctaaacgctcagcgaccaacattttaaagaaatgtaaaaaacccagggatgaccacagt
tattcgcgaaaaccaccaaccgataacaaagctacctttaatatgcaagcagcagcaccaggacaggaac
aggatcctgatctaaaacatagctgcctctgattattgatagaatgtggctctaaaatgagatgatccgg
ccttatgcatttcttcatatgacaccggtgactgacatcaagatccgtcccatcaaaatgaaggcgcgta
acctggtggcgcagaagccgccgtgcagccatatatgccaacctatgaacccgttcaagagatactgttc
catctggccaggtaacccgcttgctgccatacccatatctatccatggcaccagtccattcaatgcatga
atctgggccatccccccaccactctgcagtgctttcttaaagattttccaattttggaaaaatttattc
atttttgatgaaatacttaacaaaatacagtattcaatggtttttggatgatctggagccaaaagtatga
aaatagaaagaatacctgcacgcgcttgtagtccagttctactttcacttta
>KolobokP-6N1_CorFlu-I
taaaggggattttcaaattaaagaatttcattttcttttagtttcaattcatacccagccatagcagctc
acaattagaatgtttacttttatgttggtttataaacatgggcactatgaccagacaagtaatgcctatc
acacttgttcttaaaaaattgcatgattttcagttttagaaatgactaaccagaaaatgttctctcacca
caagttatttgcatataaaataataaatatattcaccacatgatgtgtttttttgttgcctttcaagaaaa
atgcagttttccaccaattttataatattaaaacaattatttatgggtttcatttaccccaccccccttag
aatacaacattttcccccaatttcaacaaaataacattgtttacaagatatataattaaataaatcaatt
tattttaccatagatttcatgaaaaacatattatgtattgcaaggaatgattaaaatgagcctgtttttt
acatttcatgacacatacaggctaggtctggtacaggagaacttaactttggaacctttttgacatgacc
agtcatctttgagcctcattttctggaatttcttactgattatgctcatgatttgacataaatcatgtaa
agagtgtttcattctgtgatataaacctattacttgaaatataattcatgattcactgagaaatttcaca
atttatttgaaaaatataccatattttgactaatttgaagaaaataaaatataaaaaaataataataatg
ctgaaataccccaaattatgcatttaattgactaaacacacttcaaggagctcattccaattaaaaatca
ccatatcatggctactttcagtgaataaaagcaacaaatttaagaatttaatcaattttgcatattcaag
ggaggtaatcagaataatggaaagccagttatctgttgtttatcaattcattttagttcagatccttgct
aatttcagtgcttttaaacattacaactgaaatacagctaaaatctgatttagtttactatatttcaaga
aagagacaaaaatctaatcactggtgaggtgacaaatacattataaaaacactacacccctgcattcttt
caagttgcatcattttaaaagagatcagggaccaccaaaactaatacaaataaagctggaagcagttttt
tggttttaaactattatgaaagatgttttggggcctaaatgctgcaaagtttacaaaaaagttgcctcag
atattcaaggcgacttgttgggaaaaactttccaaacgcatcttgtcgcgacaaaatatttatcgcaaga
aagtaataaaatgatttcgacaattacttttcgtacaaaatgcattccaaaacattcgcaaagaatgcca
gaaacattttttacatgagaaagtttgaacagattggaaactataaaactgtatgtgatgtagaccactat
tatttgtttggcaccaaaattcctaccaacatgatacttatattactgaaggtgcatttgtatggaaact
ttatgtaatacatgagacattttcactgaaatatggttcatgttgtattaatctacaagttttaagaaat
aaaatatgcagaaaacacttactaatgctcattctagtactaatcacgctttgtttacatccaggcttta
>KolobokP-7_CorFlu-I
ttaattaatggcaacagcactgtcaaccaaaaagagaactattagtttttttaaataatgacttcattgt
gactaataaaataaccaatagatacttctggctgtttgttatcaattttggcaggccattgaaatagatg
ctttaatcacaaaattatgaagagattttttgcaatagaattccatttgaagcataacagattatacctga
cagtactgttttctgcacactgttcagcaaaagaaacacttatttccttgcaagatgtattgctaaatct
tttccaatactgaaacagttggaaaaatgtaagaattagtaactgtttgcttcaaaatgccttccgccgc
cccaattccatgcattttcctttaaacaaaaacatgctaaactgagagatgtcaataaatattccaataa
cacattaaaataaacataaacaaagactcaattttctaaaataacaaaattaatcaaaaaaaaaattaaaa
taaccaaggaagatgagcaataaggtatgacaacataatctaagggagattactctgcaaggtacctata
aaagagaaatacctgggaaagttattaattgactgtttctacaataattgtaatatgatacctgaaatga
taagcccagtccacctggagataaaatacatgcactaacaagctgtctttgcagaaaaacacaacttta
tagaattctctgaaacagtacctcaaattttgctgactttcattcataacacctattattgtttggatt
taattaaaagtgataaaaatgggatataaaaagaaaagttataaatattctaatgctaaaaaggccactt
ggtttaagaaaggacacaatgtcagaaaacaaataaaactgcacttaccaaatgaagaatttctaaaccc
ggcagtgggaagtacagatattaaatcgaaacggctaacactatcggaagcctgtgaagtacaacacttg
atggaacaccaaacgcaaccaaacactctaccatacagactcagatcaacagctgaaagtgaacaaaatt
cagatttaatgtgtaattcagatgaaaatataattgtcaactttaagaaactgcaggaattcacaaagat
gattcacagcaaatgctgtacaagaccacagatagaactcagtatgtccagaacaggactgtgtgtttcc
ttaacagcatactgcagtcgctgtcaatatcgaagcccatccatggatatgtcggaaaagattcggaaag
cccgtggtcctgcgggaggtgccctaaatgaaatgttgcttctaccagtaatgaagtccaaaatgggcat
tgatgatgttttaatggttatggcatgcatgaacataaaagcacccagccggtcggtacttcaaaagaag
cttaattccctgtctgacctgatgataaacatgaatgaagctcaaatgatacaaaatcaggaggaggtga
aacatctcactgaacaagctgggctgccatgtgaagtcgatgtacagacagacactgcctttgcgagccg
tccgcagagcagccatgaaaaggctcaacaaagtgtcgctgctacaattgagcacacaaccaaaaaaaaa
cgcatcattgcactatcagttgtaaataaacattgcaatattaaagactgcaatcaccaaaattgtcaca
aaaacttttccaaagaaaaatcaataacttcaagtgaaagaactcttctccatgaaaacatgagtaaact
gaaaaaaacaaaaatagttaaagtcaaatcgattacaactgatggtagcagtcaactcgccaaagcagtt
agagacttcaattctgcaaagtctgaaaatattatacattaccaatgtttcatacacaagcaaagaacat
tagaaaaacacataaagaatctgtcgctgaaaccataccttccagatacaacaaatcaacatacatgca

aaaactagctagctgtatccgaacacgtgtacgattagaattacaatctcttcacgcactgaaaaagaac
actaacaattatattggatgttgcattcttgcactggaaaacatagtgccatgtttcagtggcaaccatg
tcaactgcaaagtaaaatcaactgtttgtaaaagccatttagaatcatacacaacaaaatttttgccttg
gggtgttcatctcgaaattgatgggactgatcgaaaatctgtaaaagatgaaattatgaaagtttacaac
atagatgccttaaaaaaaactgttaaactgttcaatacaaacatgtgtgaaagcttccacagtaccgtgt
ttagctatgcctccaagtcaatttgctgggcaagaaactttgctggaatgtgtcattctgcagcccatac
cagaactcttggtcgcggcatgtcaacagttgatctagcaagagctgttggcataagagtttcaaagcac
agcagcatgatgcaacaattagtgaaaatggataagcgcaataagtacgattcgcacagaaagagcacaa
aggaataccgtgaaaaccgatactttctgagaaaaaagaaatcaaaccgattgctataccaagactcact
ttacagtgcagaaccgtcaacttcatcattggaacataactatgggttgtctttctaaagagcaagaaca
atgaaataacagtgctcaaacgaactagaacctgtaaaaaagacaatactgtgtgtaaacaaactggaac
ctggaaagaacctggaaaatatcaatactaactggaaactgatatacctatttcactaagtctcttccta
taaacaagcagggcttgaagccgtggtgcccgtggcattctccatcaaacatgcatcttttgcgagaatt
gttgacagtttttgtctcatatgacagatgacttacatttaaacacagctttgtatgacataaatgcgaa
acatgcattgcgtgatgtaaagctaaatcgacattggctaggtgaaatgctaatctatgggttcgcaatc
gcagtcttttacccttatgcgtaacacgtaaatagccgtaacctaaacgatctttttcaccggtccaaat
taaacattcatagcgagatacagacttttcaagtaataaattactaagcgtattatgtaattctgcatca
atatgcgtgtctgaaaaaaatgcctcggacatgtcctcggacaatctgaaaagagcaggatttactaaaa
atgagcgaaaagcacgcacaactttaaaaccaaagcatgctcctactaaaatgacgatgtttaatgaaca
agtttagcaaatgcatgctatttttagctaagaaatacacttattataagaaactcgccttttttcatttt
ttgagctcttcgcttatctccctttatttgaaggctactgcaagcctgtcaagtgcccgaagatgggagt
ttgatgcatatctgtcaccgacaatacatggcagctatgaatagcatactaaaaacaatttgtgagagtg
tttgtaaaactgcatatttaccttaa
>KolobokP-7N1_CorFlu-I
ttaatagcagagagatgttttcctttaagccatgaatcttctctgttcaaatttagaaccatttctgagc
aatataagaccaaaaccaagccagtatcatcattcttgctggtgttatgagcaaataactaccaccactc
actttccaaaaatggctaattttgcctccagttacagtcaaggaaattaatacagtgcttctaagaatgc
tataaaacagacaatggaaccagaatacctgacattcattgaccacataaaagcacatagaagatgaaca
atgaaaaatatgctgttacaagcactcatttttgtaaaaaaatatgtcttgcaaagcaaattgtttcagt
caaacaggcaaaactggtcatttctcactttcatcactcacctaccatgttaccatcatgtttcagttgc
tcatatggaccatgtgcatacttttctgccaatcaactgccacttttcaacaaataaacacatcaaactt
tatgtttatgcttgctctaatgtcaccaaaataataattgaatcaaaaaatggccatgaatgaatagcaa
attatgaaattcttacctctggctgcagaaaggaaaaaacagctaaggtaacataacagactacagctga
aggccaaatcctgcctatctggtctaaaatacctgctaaactgataacagtgttcatggcaagccctaaa
caattctagaacttgattttcattggtcgaccagaatttacgtctcagttcgcaaagctcggactgtgga
aatccgcttgccgagcttaatttttcttaatttatttttatcggcttgattttctggcagatattatggc
aaatgcgctttcgtaatcatttgagaccttttcatgatgtatttgatggcctttctataaaaatctacat
ttctgacatttcgcgactcgtttttatggctcgatgcaggatttttttccctcaaaaactgtaattacct
ccctttattttgaaccgccagcttgtaagcaagtatttaacagcttaaaatggtgtcattttgcaaatat
ggcaggtcaatacagcattctagaccaaaattatgacataatgaacacttacaaattttcagaaaattcc
ttaa
>KolobokP-7N2_CorFlu-I
ttaagagcagagagatgttttcctttaagccatgaactcatctctgttcaaatttagaaccatttctgag
caaattaagaccaaaaccaagccagtatcatcatccttgctggttttaagagcaaatgactaccaccact
cactttctaaaaatggccaattttgcctccatttacagtcaaggaaatttatacagtgcttctaagaaat
gctattaaacagacaatggaaccagaatacctgacattcatttaccacataaaagctcacagaagatgat
caatgaaaaatatgctgttacaagcactcatttttgtaataaaataggtcaggcaaagcaaattgtatca
gtcaaacaggcaaaactggccatttctcactttcatcactcacctaccatgttgccatcatgtttcactt
gctcatatggaccatgtgcatacctttctgctaatgaactgccacttttcaacaaatacacacatcacac
tttatgtttatgcttgctctaatgtcaccaaaataatgattgaatcaaaaaatggccaagaacaaatagc
aaatgatgaaattcttacctctggctgcagaaaggaataaacagctaagctaacataacagactacagct
gaaggccaaatcctgcctatctgggctaaaatacctactaaactgataacagtgttcatggcaaaccctа
cacagttctagaacttgattttcattggtcgaccagaatttacatctcggttcgcaaagctcggactgtg
gaaatctgcttgccgagcttaatttttcttaatttatttttatcggcttgattttctggcagatcttatg
ccaaatgctctttcgtaatcatctgagagcttttcatgatgtatttgatggcctttcaataaaaatctgc
atttctgacatttcacgactcgtttttatggctcgatggaggattttttttctaaaaactgtaattac
ctccctttatttgtaaacctcagcttgtaagcaagtatgtaacagcataaaatgatgtcattttgcaaat
atagcagtccaatacagcattctagaccaaaattatgacataatgaacacttacaaatttcagcgatatc
cttaa
>KolobokP-7N3_CorFlu-I
ttaagagcagagagatgttttcctttaagccatgaactcatctctgttcaaatttagaaccatttctgag
caaattaagaccaaaaccaagccagtatcatcatccttgctggttttaagagcaaatgactaccaccact
cactttctaaaaatggccaattttgcctccatttacagtcaaggaaattaatacagtgcttctaagaaat
gctataaaacagacaatggaaccagaatacctgacattcatttaccacataaaagctcacagaagatgat
caatgaaaaatatgctgttagaagcactcatttttgtaataaaataggtcaggcaaagcaaattgtatca
gtcaaacaggcaaaactggccatttctcactttcatcactcacctaccatgttgccatcatgtttcagtt
gctcatatggaccatgtgcatacctttctgccaatgaactgccacttttcaacaaatacacacatcacac
tttatgtttatgcttgctctaatgtcaccaaaataatgattgaatcaaaaaatggccatgaacaaatagc
aaatgatgaaattcttacctctggctgcagaaaggaataaacagctaaggtaacataacagactacagct
gaaggccaaatcctgcctatctgggctaaaatacctgctaaactgataacagtgttcatggcaagccctа
cacagttctagaacttgattttcattggtcaaccagaatttacatctcagttcgcaaagctcggactgtg
gaaatctgcttgccgagcttaatttttcttaatttatttttatcggcttgattttctggcagatattatg
ccaaatgctctttcgtaatcatctgagagcttttcatgatgtatttgatggcctttctataaaaatctgc
atttctgacatttcgcgactcgtttttatggctcgatgcaggaatttttttctcaaaaactgtaattacc
tccctttattttaaacgtcagcttgtaagcaagtatttaacagcataaaatgatgtcattttgcaaata
tggcagtccaatacagcattctagaccaaaattatgacataatgaacacttacaaatttcagcgaaatcc
ttaa

>KolobokP-8_CorFlu-I
taaagaagccccttaacatatatgaatcaatgtgttttttaattttccattaaagcaaacatttaccaa
cattttcatatatttcctttatgttttgtcccaaaaatgacctttctgaagcagttctaagaaacaaggt
gatttcagatttcagtcatattaaaagcaagaaaacaacagtccaaatgtcaaagtagcagctccttaaa
aattttatttttttatattaataaacaaaactaattgtaatagtttgcaaacatattgtacaatatgttg
actttccaaacacacacatgtcactagtttacccccattttggtgaaatttgaggggtttaacattaccc
cacccacttaatgtttttgttaatttttgtatttgtttggttttttttttcagttaatattgcaaatggaa
ttgctagtttggtgatttaatgcagtaaatacacagatataccctggtctgtagtaagctgcttatcagc
cattatcaaaatatacctgatatcacttctatttagtccccagtaaagctatgtctatggctcttaaaag
agagttacagcattgtgacactatatacaaaggtgctctcaaactgttttatctggacaactcaagttac
cacagaaatgcacctaattgtccatttctacaacctcagtatcatttggatgctatacttagtgaaaaac
agtttcttaaagccaaaagaaagatgggtagaaataaaggattctttaaacggagaagaacaacattcaa
acatggaaattctgccacaatcagaagagttcaggattgcatttctaatgcagaagaaaccgaacctgta
aaatatgtgagactggatgccgaacagcaagctatggtggaaaacaaccctatattgcctgctgctgtag
ccaaacacagtgcctcggacaaaatggtgccgactttcaagttcctacggccatgcagagctaacccagc
tgacgttaagccaaagctttcaaaacagcaaatacaaaggtaggttacattgcttaacctatactagctt
tttttatttcatatttatatataaaaacttaaaaaataattataaagaataaaaaactwaataawatcta
gtttttwaatcaaatttttcactttagaatgaaaacaaacagtttttttttctgtattctttcagcaaag
tatccataatatcagctatctggtatcattaagatcattttcagttaaaatcagtgccaactttaacatg
atttcatwaaaaagtcacttaatggacaatttggtataggcactgcaattcatataatgggccaagggag
acaactactctaaccatgtaatgtttactaatcccctacagacaacttttttgagtgaaaattgtgtctta
tatggacaccaacccaaaggaaagacaggtttctcattctatttacagcattgaggcagattcctacagg
gtactgcactgtggccagctgtggaccatgattaatgaagtgaccagggagcacctagataggaacccca
gatgcaaaggtaacctctcctttgactttgagaatgaaatgcagtgggaattgtgctggcgagaaagtgt
catttgtgataaatgccagtacaagtccaaaatatacaacttgtacacagaggtagaaactggccggcca
ggaaggaaagcagccacagccaatgtaggtttgcacattgccatgagtcaaacccctgtaggtcctacca
gtgtcagaaaattattgctagctagtaatattccagcaccatgtatatcaggaatgcagaaaacatcaaa
taaagtaaataaattaatagaaaatacaaacaaagaagatatgcaacagagacgggagaacctatctgct
atcaatcaattgcgtggcgcccccaaaatgaaatagccattcaaagcgatggggtgtacaacaacagtc
tgtggtctggtgtgacaaaaacaccttaccagccggctacgcaaatggcctatattgttgcagaaaatgt
caccggcaaacatcaaattataaatgtggaactagttaataaaatctgctctaaacatggatatcatacc
atggaagatgatgaatgtgacatcaaatctggtgagtgttcggctacagcaagcatggaaacttccattg
gggatgagaaacggtgtgcaaaacttgttttagagggactccttgaagatggtcttagtgtcaagtacat
aacaacagaccaggataccgccgcataccttgcagctactgaactttatcatgaaaataaaaccgagact
gaacctgaacatcaaatcgacactcgacatttggcatgtaatcaccgcaaacaaattaagaacagttcag
atgttcaagctatgatgccgggcaataccatacagtacagacagtacctgcagggcagatttgccattga
cataagcaaaagatgccacaaggagtatgccaccattttcaaggaggaagctggggacttcaaagcttta
tcagacagaattaacctggccattggtgcaattaaaagatgctatggggggtgaccacagtagatgccaaa
gattttctactgcctgtaagggtgaaagcactaataactggatattaagaagtgcatatttaccaagaaa
tttcaaaattaatattcaaaatgaacacaatcaagaagttttgacccaatgtattgaatacagactcgga
caaccgattctcgaaaagacccggctgaacacaaatagccaaaaatgtgaagcaaccaacagatcagtaa
gaagatccttacccaagaacactctgtttggtcgtaactttccaggtagagctcacagtgcaattcacag
tgtcaataatggtcctggtgactcgattaagaagttatgcttgcgtgccggttgtcctattccttctggc
agtaaagtagctcaacggttacaaaaagaacagcagctttctgaaaaatacaaagaaagagctcggtcaa
ttacagcgaagtccaaacgaatagcaagagccaacaagctatacaaacttcatgaaaaaaamcgtgagaa
aaaccgatacatacgagcacaaatctctaaacaactaactgttaaaaacaaaaagaaaacagacgtctca
aagtgtaacacaccgaggccaagcacatcagaccatcactataacagactgtcaagtcaacggaagaaaa
tacaaggcggwttatgacctcccatgcaccgtcccaatgattcgcagtgcctgcgctcacagtttatctc
atgtgactccagaacaagatggtcagggcggacacagactttactatggcacaaatgacttatgtcaagt
tggcccgacaaatcatgatgtggcacttcatagggtagtaacttgtgatggaccatataagccaccctgt
gcgcactttccagggtaacagatttatccggccaggtgactctctttcggccatagccatatctgtccaa
acaccccacccactcaatgcagcctcggccattttcaaccatggcagtctttttttgtcaaagatttgcac
aatttttcaaaaaattcatccatattgattttcaaatgcttgcagcagactatggtttgaaatctggcat
aacaaaacaagtttgaattgcttacttgcgtcacatgcacgtgcagtttcccaaacttggtgcctattta
>KolobokP-8N1_CorFlu-I
taaatatgcatgtttgactttttgttacgactattttctattttatccaaataaacaaataatgctgaac
acaaaaataccattttttgtctgtcattatccccaattcagcccattggcttaactcttatacaagcactc
actgctcatttgcatgcaataaaagcactatagatttgaatagtgaaaaatggtatctaaaaattcatta
aaattttaattttctcacagatgccttaaaacttgcacaaataagatcttaacatgtgtatcacaagttg
cactaatattttctgaattggccttattcttcttttatacagagcaatttttgttaccccacccccctaca
aaaaaggtgaaatttcctacatttatcaccatttttatgctcttgtaatattgccttactgaatatccaa
ctggatataacttcaaaacctcaataaatcacatttatatatgacaaaaagctaataatgtgtacttctg
caacaatgaacatcagccatgccttaaatatgagcctagacttaaccaaaaacagaccaaaatggagcta
ccaggcaaatttggaaccattatttgcctgttctaatacaccaaagtaagaaaatagctggtaattttgc
atgaaatatgtacaaatagtcaaaaataggcaacaatttatatggtagactcatccatttgcctatggtt
acatctaagtctggaagatccttcagtaatgagctttatttgtgtagtgctggaatccaaacccttaact
ctacccattactgctttaaaacccttctaaatgaaattatgtcttgaaataccacaaaaatgcttaaaa
ccatggtgcttttttagtgtcagatagtcaacagacctctgagaggtgtctgaatatccattcttgacaat
tatcaatcaaagggagataactcctagacccaatatttctatattttacatattatgggtaatatttaca
actttttggtaaattttcatcagattttattaaaaactgtgatttatgactaaaatgacaaaatctgaga
cttggttagacacttttagacatacagatttacatttttcttcaacattggtgtcattcataaatctaaa
acagaactgtgggttccacaaggtcactaagttaacccaaaacagcaatcaactcatttaaatgtgttct
gatatccactataggtatattttttttcaaaaaaaagctgactttcatggctaaagtgaaattagctactt
acaaatttgcatcttatatggccatattagccaaaatttccttcatcttgaccaaattctaaattgttac
aatgaagccgacaactaactttgagccaaatattgccagctttacgcagaaataaggaacaaatatctat
tcgcgacatatttctcacacaaatatgccacatgtaggcttacgatgaaataaaataaaacaaacagtc
actctctgtattgcaaccccccacctctcactttatggatatgcaaactgtacttatttcagtgttccctc

agatcagatgatccaaatcaagacttattacagttgtttacctatctaattgtgcagaaactctccactt
ttgtccccaaactgacagcatatacaattttaaaaaattctatttcaaatatggcgccttgagaagatgg
tccattactttcgctttcatattta
>KolobokP-8N1b_CorFlu-I
taaatatgcatgtttgacttttgttatgactattttctattttatccaaataaacaaatattgctgaac
acaaaaataccattttgtctgtcattatccccaattcagcccataggctcaaatcttatacaagcactc
actgctcatttgcatgcaataaaagtactatagatttgaatagtgaaaaatggtacctaaaaattcatta
aaattttaattttctcacagatgctttataacttgcacacataagatcttaacatgtgtatcacaagttg
cactaatatttttggcttggccttattcttcctttatacagagcaattttgttaccccacccccctaca
aaaacttgaaattttcaacacttatcaccattttactgatcttgtaatactgccttaatgaatatgcaac
tggatttaacttcaaaccctcaataaatcacatttatagatgacaaaaagctatttgtatgaacttctgc
aaaatgaacaacagccatgccttaaataggaggctagacctaaccaaaaagaggccaaaatggagctacc
aggcaaatttagtaccataatttgcctattttatgacaccaaagtaataaaatagctgataattttgcat
ggaaagtatacaatagtcaaaaatatgcaaaaaatcttctggcagagacatccattagcctatggttaca
tctgagtctggaaaatcctacagtaatgagttttatttgaatattgctggaatccaacccattaaatcta
cccattactgctctaaaccctttctaaatgacaattatgtctagaaataccacaaaaatgcttaaaacc
atggtgcttctagtgtcagatagtcaacagacctctgggaggtgtctgaatatccattcctgacaattat
caatcaaagggagataactcctggacccaatattgctatattttacagattatgtgtaatatttacaagt
ttttggtaaattttacaccagagttttgtaaaaatggagattatttactaaaatgaccaaatctgagact
cagttaggcacttttagacattcagattcacattttccatcaaaatttgtgtcattcataaatctaaaac
agaactgtgggttccacaaggccactaagttaacctagaatagcaataaacacattgaaatgtattctga
tacacactacaggtaaattttctcaaataaagctgactatcatggctgaaatgaaataagccacttacaa
atttgcatcttataaggtcatattggccaaaatttccctcatcttgactaacttcttattgttacaataa
agccgacaactaactttgagccaaaacatgccggctttacgcagaaataaggaacaaatatttattcgcg
acatatttttcacacaaatatgccatatgcaggcttaaaataaaataaaataaaaaagaagtccctctc
tgtattgcaaccccaacttctcacttcatggatatgcaaactgttccctatgcaggtattcccttagatc
agatgatccaaatcaatacttattacagttgtttacctatctgattgtgcagaaactctccacttgtgtc
cccaaactgacagcctatacaattttataaaattctatttcaaatatggcgccttgtgaagatggactt
ttactttcattttcatattta
>KolobokP-8N2_CorFlu-I
taaagatgtatagcttacttttataatgactttattctgttttatccaaataaatagataattttgaac
agtgaaatcactttttttgtctttcattatcccatgttttacccacaaacttaaatcaaatacaagctgat
agtgcccagtttcctgcaataaattttctgtgattttagttatgaaaagtggtacacaaaaattcatgaa
aattataattctcaaacaaatgctatacaacttctacaattaagatgtcaacatgtgtgtcacataatgc
atcaacattttcttgcttggccttattagtctttaacacagagctaattttgtttccccacccccctcttc
aaaatcaagaagtttaacatttttaatattttcctgctcctgtagtactgccttagttattatccaact
gaatatgacttcaagattacatgtaaacacatttatatgtgaaaacaatactttttaagtctacatctgc
aacacaagaggacagtccttgcaaaaatggaaagctagactaaggcaaaacaggctaaaatttacctata
gggcacttttggcaccacaatttggatattttatgatcctaaaatgtcagaagatgtacaattttgcaca
aattatgtaaaaatgtcctgaaaataagcaaatttcacatcacagacacagccatttgcttataatcaca
ctactgtctacaaaatccttcagaaatgagctttatttgagtcatccttgactcaaaatcttatattgta
cccattattgcttaataaccctgttaaaatgccatttatgtctagaaataacataaaatacttataaaca
gggcatttttcagtgtaatttagtcaaaagacttctgagagctgtctgaacatccactgttgactattta
ttataaagggaggtaattcataaaatctgaattgccatattttacaggttctttcagtaatttacaaggt
tttggttattttgacatgagatttttgttaaaaatgatgatttgtaactaaaatgacccaatttgatgat
caattagacagtttttgacctccagattcacattttcttttaagttttttgtaattcataattctgaaac
acagctctaagtttcataaggccatagtgaacccatcagcaacattatgctgatttaacaggctttttca
caggcactgcagtattatctatgtgaataaagcagatattcatgcttaaaaagatataagctacctcaaa
atttgcatcttaaatggtaataatggcaaaaatgccatacttctttactaaccgctaaacggtgaacttc
agccgacaacctactttctgcgaaaacctgccaatgttacgcaggaaaataccatacgtatcaatacgcg
acaaatttctatgcacaaaatattcctagtccaagctgtaattgaactataacaaaataaaagtgtttct
ctgccatgctaccaacacctcccattggatgaatatgcaaaatgtacctgtttcagtatcccatggccaa
aattatgccttctgacaacatttacctatgtgattccataaaattgttcagaaacactccaattttgtct
agaaactgacagcataccaactgatgacaatacctatttcaaatatagcatcttatgaagacagtccatg
actttcagtttgtctattta
>KolobokP-N1_CorFlu-I
tataagaaaacttgtgtttttcaatatcttatattttctgtttttatttaataatatgatgtttgcaata
tttctaccaagtttcagcttccagttctcaaaaatgaactcaatatgatgaattttccaacaccacctat
tctggaacgcgtttgcggtcaaagcggctaaaactaagaattcatttcttgcaaataaaacttgaaaaac
atcaaatttcttaaataaatggttatgaataacagtaaaacatgtcaactacttaaacaaactcactttt
taagctgatatatgcataatgtgccataaagggtattaattgtggcatttttaaggcaaaaatagttaaa
attgaccccaaacctagttaaaaccccattttgcctcatgttacacagttattttgatttttgtctttca
aatttgcttaaaattggtcagcaatcaattttagtgaaaaataatattgtagactctattaatattcaaa
tgctgccctccacttgtacttcttata
>KolobokP-1_GarTel-I
ttaaggaccatttttttttttcctgacaatttgaaacactacatttcagcattgcgcactaaatttgac
tgttttaggtcattggcatgacactaaatgacttaaaactgtactaaaatggattttgaacccctatgca
aaacatccaattttaacccattttagtgaagaaatgtgcacacagtccgcaactgagagaaacattaact
ctgcactataacaacttgttggcacttacttgcctgctccaaacactggatacatgttctagaacatctt
ttcattcctaatcaaagattattccatgttccagtacactttcaagtcaaaaatgacgtctgctttactt
tcactatgcaaaattatctgcaaattttgttaattttgttaatttttcctcaacgaaagcataactgctt
gaaaatgaaggctctcacaactagccttacattaagtaaggaagtacaaataaaacacactcttaaaata
tctcccatagttacattacacttcatactgtaagatatctccaatgcaagggaggtaattcaaattgtct
taccaaaatttcattctctaacactaacatttactatcccagcctcccaaatggtctaattatgggtatt
gctaggtaacctgatataccagttgataaatccacatatgaaagagcctaattggacattttatctgata
tgctatgaagaagaccaaaacatatgtacaaactcttactttctgtgtggacttagtctaaccaagaaga
tatttcacaaagagattggaattacttatacaaaaatggaaaaaaggtgtaaaaggcgtgacagaaccat

ggtaagctgcaataattctcgaaaaaagagagctacccagttcaagccgggtagatccgcggcaacaact
gttgaagtcttgcctgaaacaacaccccaggccgtccgttcaacatgtggaggcaatgaatccataagaa
gtcgtttgacgagtcaggaggccaaagatttagacgcaatttcttcttcccccaatcttccatacactct
ccgaccaaaggcggttatggacgaatgcccgggcattgaaaatttggaaatggacgagaatgttatcgtc
aacatgagaaaagtcaatgagctggttaatcgtgttcacggattaacatgcaaaaaccctagggttagtg
ttggcattgtggacagaaaaggtctatgtgtatcttttgaagtgaaatgtagtgcatgtcgactcaggct
acatcccgtgcaaatgtcggaaacggtgaaaaaggccaaaggtccgccagctggtgcactcaatgaaatg
gctgtcttacctgcagtaaagacaaaagtgggcttggcggatatcacaacagtgctgacatgcttaaaca
ttaaagctccttgcaaacgtgccatgcagtcaaaattcaattcaatgtgtgataaaatgactcaaatgaa
tgaaaggcagatggaaagaaaccaagaatatgtgcagcatgtgacatcacttattccgacagaagcggga
attgatgcccagtatgacgtttcatacacaagtcggccgcaaggtggcagtgaaaaggcggaacagagct
ttgctgcaataattgagcataccactaccaggaagctccccattgcagctggtattgccaacaaacattg
cacaataaaggactgtgaccatactcaagcacggtgtaaaaaaagctatagctcggacgaaaccattgct
tccagtgagcgagcactgctacacaaaacattggatgacgtaaaaagaacaggtcttattacacttcgct
cggtgaccactgatgccagtacacagcttgcgagagccctaaaggactacaacaagaaagacgtcaattc
aaacatagcccactataactgtttcattcacaaaggccgaacgtttgagaagcatgtcagagcacttcaa
ttacgaagcataccgaaacggcacaccaacaaaccaaagtaccgacagaaactggcaagctgtatcagga
gccgaattagaatagagcttaaaagagcacgagcaataacgcgaacagatgactctttcatacgtgccgg
ttcggcagcaacacacaatataacccaatgcttttccggtcaccaccgtctttgccgagatgtttcccat
gtttgcaaaagccacctaactgcatatactacagatcatttaccatatggagtacacttggagctaagtg
agacagatctgtgcttggtccagaaggaaatagacaaaatgtttgatgaaaatggcctgaaaagtattag
taaactttttaacacgaacatggtcgagagcctacattccgcagtatatagactggcgcccaagtcgact
tgttggaagcggaattttccagccctctgtcattcagccgtgcattccagctcaataggaccaagtcggt
caacaatatatcttgcgcaagcagccggcataaagatttacaaaacttctcgtctatatagccagctgaa
attaaaggaccgccaaaggcagtatgactcgaagagaagcgcttcatctaaatttaaacagagacgatac
tttttgcggaaaagaaaattattctggcctcttttcaccaattctctctacagcactggaaatgaagcct
ctcaatcagctagtgagcacaactatggccttttgtcttaaaaaagaaacagaaaaactaatgagaatga
ccaatttcctcaaaaaaccaacatacaacaacaacaacaacaacaacaacaacaacccagaaaacaaatttttt
tggcaaataactattctttaccatgttgtggaagagacatcgtctgaatcctctatgaccagtgcattcg
ccgttatgttgacaaacttttctcgcattgtttactgaagaactttcataggacagatgatcaacattta
tgcacaacttattatggcataagtgagacacatgcattttgctacataattgaattggactattctcgac
aaaataagcaagtcggtgaacaagtagcttcaaccgtttaccatcaacagtaacgcgatgtattccatag
ccatatacatcggtggtgccggcccaaactacacattcaccgccactgggcgcagacttgagaattaaag
aattccgcaaagtttcagctctttgagcacacaatggggaatcaaagaactcactggccatagggtatgt
ggaatcttattgctgtaatatcaggaaaaccaaattagcatacaaatacctaaattctttcaaatttctg
aaatcattcaaaacctgtttggattacctcccttgtttaaagtcattattgacaacagaaaatagtcttt
ggagcatttcaaagaccagtttctgactagaaatacacaaaattactcaaacaaatccattttagtgtca
aattgctatgattgaaaaatgtctatttacctattaa
>KolobokP-2_GarTel-I
tatgagagatggtttttttcaattaaatccccagattactctattattcaaaagtatagtctattctgat
acagtttaccaattttcacatgtctaacataattatttttacgtctagagccattttcataacatgcatg
ttagcccgcccgaaagctgaaaattgagccgtcagccacctattctcctgtcagtctgaaatcgctgtct
gagacaaaacgcgtcatttagaaacatttcactggttcagttgactgcgaataaacttaattaagctaag
gaaactttaatttgacgcgcaggatggaaaaaaatcaatttacaccattgattttacaaaaatttcgaca
atttttcggaattttttttttttttgttaaaaaatggatttttcagttttattttagttttaaatggcct
gaaatataattctaagagtaaagtatgtatttcaagagtgaaaccaagtgtttcaaagagcctgacacct
gtcaattagcagataaccaaaatacaccagtcagagaaaggtgttaatttcagagtaaggtattgcagtg
gccacaggttgacaccttggcttactctcaagcatgcatattatgtgtgaatcttgcagtaattcataag
taggtgatctcttaagtagaacagttcaaattaaagcttagtttttctctgaaaatgctattctgagcca
aagcaatgaagaaaaaaactcgcaaccgcctgcttcgaaagataggctttaaaacaggtacatctaatcc
aagcgtcaaaggtaagagccgtaatattttcactgaaactgaaccaggacaatttattagaactttcaaa
gaaacatttgattcacgcatagatgaaaaggaaaatggctcttttggttttgttgatgtagacggtcagg
atactgcagttaggcctttgcggccgttgaaaactaagccaaaactcttagatcagtatgaaaacaagtc
agtgtcaaatcttcatcctgatctgtttgtaaataaattgtactgtccattttatgttcaagggatgttt
aactctgagataaagaaacatgtacagtcaaaccccacttgcaatggggatttagtcattgacgtaaaac
attcaatgaaatggggcctttgctggcaagagcgtttagtttgttcaaaatgtacatttaccagtggttt
tcacaaactgtatgaagaagtagaaagtaaaactaggggtaggaaatctgccaaatgtaatgttggtctg
caattggggctaatgtccactacaattagtaatacgggtgcatgcagggtgctagcaaatactaatataa
ttccgccaagtagaaatagcatgcgcaaacagtcaaacaaagttggtgaggcattagttaatcttaacaa
taagtctatgcgaaaggtacgacaagagttagttgaagaaaatagactgtgtggcaataagaatgccaaa
tctgtacacattgaaggtgattcttgctacaacaatcctatttttaattctgagagtacaccattccaag
caggtactattgtaacaagtactttttgtgaaaacaattcaaaaagtaagaaaatcattggtgtttttgt
aggtaataagttgtgtcctattggctctagactgaaaaattcaggacaaaatgttaaatgtccaaatcac
aagggtaaatgcacggcaaatgttgaagagtcagagccaataggcaatgaagagaaatggaatagtgcag
tgtgttcaagtataaatggagacattgaaattgcctcatttacaggggatggtgattccaaggggcacaa
gggtgttgtcaaaactgtaggatataacattgaacaattgaaagatttgagacatttagggaactcagtg
aaaaggcaaataaacaagacaccatttagttcgaacatgtttacgggcgcaaagcgagttaatctgaaga
accgctttgccatgtcagtaaaagctagatgtattgcagaactgaaaagggcacacactgtatatagggg
tgatttagataaaattgcccaaaaaatgccatatgtaaaaaacaccattctcttatgtttcaatggctac
tgcggtcaagcgtgttctaaacacagtttggtatgtggcggtaattaccgaagggaaaaatattttctac
cgccaaatattaaactgagaatgactgaatctgatgagcagttgcttttgaaatcaatggacatacttct
tggtactttaaatttgccactcacaaaaagattaagttctacacagaagtgtgaggctgtgaatagagct
tatcaatcagtgaacccaaagcattccacttttccccgaaattgcaccggtcgaatccatggtcagattt
tgaaacttaacatgggctcggccgagtccatgcttctcaaatgtgagtcacttggtgcgccacttacaaa
gggttcatctgtaattaaacatttagccaaactgcatgcaagagacttatataggccatccaaaagtagc
aaagcgcgcaatagactgcggcgttataagactcttgcgcgaaagtatgaaatgcatgcaagcattcatt
atagcaaaggtttaacagaccctagaccagacttttctgtacatgatcacctacaagatcacaaatactg

tacttaaactgaattcagtcaaacttaatgctaagaacacatatatctactcactgtgtagcccaggcta
gatgctgtgttaaaatagcaagtgcttctttcaccatgtaaatatgtaccatacatgtagtctgtagtct
tcttaagagctcaaagatgaatatttttttctatgtttacatgtaaatacaaactgaatgtgctttttttac
tcccttgattatttcctttctatataagacactttgggtagttgttgccagatagagaatggccttgac
atctatctgtgcaagactgtcgactattgttgacagcttgcggctctaaattcaaatgtaaagggttgca
gcacagactgaatttgcataaatgggacacatgcagatgctgtggtaactgaagcatgtcccagtggacc
ataaacatgagtctgtgcacataataatactttgactgtcttacccctggtagtttggcacgaatcttcc
catattttagccttggacctcccaatgttaccccgtccacctaaggcaaccctttcaccctcatttac
acattttgaccagattctttcctttgtaaccattctaaattgatataaatccattttggagtcaattttg
atactgcagacgacataaaaatataaacaggaaatgacctcaaagttcgggaaatctctctta
>KolobokP-3_GarTel-I
aaaataaaaccccgggcttcatacaaccattaaattagactttctgctagcttttagggtagtttccatt
aaaaatacactactttgaagataatagatacatttctcagtgatttatactcatataaacagggtagggg
tataagcaaaaaacatggctaaatttctcaaccttgccgcaccccaagtctgtataaaaacttgctgtta
ttcacttatttctggatggattttgataaaataaaaagtaatggatgcccctcatcccagacctttacat
ctggcatattttgtgcacttgtggctaatctaggtatatgcagggttcaattttatttgaaaatgcgtca
aattttacgagaaaatcatcattccgatgaatcaaaatctatatagaagcatttttttcttcaaatttgt
cttataatttatattacatttcatatatggatataaacaatcacttaaaaataactagacaggtaatcta
agaccccagtacaccagaaaccataacagaagcagctgaccctgataagcccagcaggagggtgcctgga
atgcttcttgaatatgtctggggtctgtctgttggaggtctgcagtaacatctgcttaaaattcaagcct
ctcctagcctatataagcatcccaattccaaaccactgtcattccttagctctgaagaagaaatttactt
actttcacctgaacttctatccagtatggcgaagggcgaccgaaagaaacgtgccgcaggtatcctctac
aagaaaaaccatccaagtacacgtcatgccgttgaagaacaccaccaacagcctggacaaagatttgcga
cagaagttcaaccagaggcaccggccaagatcatttcagagtcacgccctagaactcggggtctgctgaa
aatggaaaaaagcaatgaaagtctgggaaatcgcatagtttctctcaacaagatgatatgcctaatgaat
accgtttgccgggatcatcaacagtttcctgcccactgtgaaacaatgaatttacagcttcacagtgaga
agaagattggacttggctcagatctgaagtttgcctgcaaaacatgctgttttgtatcgcagacatgtca
aacttacacgccatgtgctggaaagacgaggggagctgcgatcaacatgttacttgcctctgccttacaa
gacatgtctattggggtgggaaaggctaacattctgttaagctcaatggacattccaccgccttcaaaca
gtcatttgcagaccctggccattaaagcatcagagaacacggtacagctgaatgaacgcgacatggccga
gaaacgccaattagtcatccaacataaccaagcaaagggtgctacagacccaagacagttagatgtgtca
tttgactgcagatacaatgccaaccgaatggtctcttcatataaacctggccaggcggcatcacagacct
acggagtggctattgaaaaccataccagcttcaagtatattgtcggcctggctattgaaaacaagctgtg
ctggtatggcgcatacctacgaaaccagggatttgagggtatcgagtgtcccgacaccgacaacagtcat
gagtgtacggcaaccctggattacatggaaccccattctgaaagacgtatggcatatgacgtagccagtc
agatgcacagggaagacataattatacggaccttgaccacagatggcgacacaaagtcttaccttggaat
gcaagacttttatgatcagcttggagaagcctggagtgtatctagacaggcagatccttaccatttgggc
agcacacaggtcagacgggtgcggaaagcaaagtggagtaccggtatgtttcccagctgtgcaaaccaac
agaccagacaacaagcaatgtcggcatttgcaaaagacgtaaagtcaaggagctccaaagtggtagagag
attacgagctctcggagatggagacattaccaaacatattaaaagattacctggtgtgtgtgctgccact
gtcgattgctattcaggaaattgctcactctgcccacatgaatctctcgtttgttccggtgttcgtggcc
agggtgactggtggtccacttcagagttcctctcgacacatgagatcgaccgtctgagaatgacagaaaa
cgacaaatgcttgttaaagacaattctggaagtcagacttagcgaacaggcagtctacaatgtttcgtca
aataccagcacccagaagtgtgaagcatttaaccgcggcgcactggcttctttgccgaaagaagttaatt
ttggaaaaacttttcctggcagattagcttccaagaccctgcaactgaataacagtatacagacttctgt
gcaagctaaggtgacagccatcactggtcagaaactgtcgtccaggaccagtcgctacttagatttctgc
gcaagacgtacagagagtagaaaacagcgtcaacggtcaataaggttcaaagctaagcggcgacataaca
gggcccgcctagaacttgcataccacagtgcaagatctgggtgtaaaaaacaatctggatctaatgaata
tagcaaaggtcagctagactaaaggatactgaaagacgggcgtcataggcaaagaaacatgcaaagcaag
aaaaaaaacaaaaaaaacccacccgtcaacatcatggcagatgttctgatgatggtctgatgccgaccga
aacgggaaaaaacatgcaattaaaatcaaagacccccccccccccttaacaagattcagaacaccccca
cccccccccccccccccaataaaaacaaaacaaaacaaaaacacattcaggctgagatgagactcacat
aaccttacctgttcaaagagttcaataaggcacataggggctggagatccatcactgttgacatggctac
tgcattttgaaagtctgaaacatgtccgtcgctggcagttggtggagctggactcgaacacaatatgctc
ggcattgacacatatcttgttgttgcagagatgggatgcctgttgctttgctgaaacatcaaaatttga
aatttagccataagtgcgactcggtgagctgtacgagtcttatgatctgccgacgagaggtcccttgggt
ctttatatctaaactgaccataaccctgtttgcttatacagcctgtccaaaacatgcacttgccattgga
atgaagcacactgtttgctgacagcttctgttcacattctcggaaaaaaatgtccatattaaggcaatta
aatgtcaacaatacagcaaaatggtgaaaaaaaacagtgttaaaatatccgaaaaaagctaaaattcaag
atttttgttatttttagctataaaaattgcatgaaagcacttatctgtctagtcatatgcttgcaaatg
taaagcagacgacataagaggcaaatccgacaagggagaatactccaacacagcttgacaccaattttca
aagtcatttttttaaaaatttaataaataaaaaaataatcatacctgggataaatcctttcttgtccaa
gcttctgatgtactaggctacttgcagaaaaaatttcaggatcctattccaagaattgacgaagttatta
aaattttagtttttccatata
>KolobokP-4_GarTel-I
cagtatttgtcccaaatcttttaagaaacaatgtttaatagtctcctccaacttttgatacatttctgag
taaagtaaaagcaatcacaagtggatccaaccattttaagggagctgggctaaattgaaaaacttactg
tctcggccaatctcacagctcggccgcaatgttttgagggtactgacagcttctacggaaacttgaataa
gtcttggaattgtcactggattctggtcaaataaatagttttgtagttcaatgttcatgtattacctgga
aaaaataaaaccaaatacaccagtctaccctttacacctttaacctgctgataaacatgaaaatccttta
aaatttgctttaaattcttacatttcaaaggtttctttaactttaaagggggaaaaaattattatttca
tcagaaaaatgcatctgcttgactcttttaacaaattgacacaggaccaggaagtaaagtcagtccccac
tgatgttgagcctctccagcctgtacttaacaacatcacttgagatgcacactttaaaaatgaccaaatc
cagacgccctcccaaggaccttgtctgcctggctgatgtacactgcaagtgtaactgcatataaacaga
gccatcaatgggccatttcattagcagccagattctgagagttacactatactctaccagtgtttgctcc
aattccaagcgccatgtctaagaaaggaaaactgaaacgccgacgggccggacatctcttcggcagcaac
aacaagcggacgtctcgtgtcaccaaacacacttcagctgaaaaagtcacacctgtgcacattcctgttc

cagttcttcacataaacgatggacaacaggagaatctggggccatgtcttcgtccgaggggaaacctgga
aaacacgggcaacctgggcaaccgaataatcagcctagatcagctgctagaggtcataagtactgtttcg
aatgaccacggcaaccatccggtcaactgcgagtccatgaaactgagacttctcagcgaaaggaaacttg
gcttaggctcggatctaacatttgcctgttctacatgccagtttgtgtccaagccttgccaaacgtatag
gccgagagccgggaaaagacgaggggctgccgtcaacatgctgctagcatcaggcctacaagatacctgc
attggtgtggagaaaggaaatatactgctctcttcaatggacataccacctccatcaaaaagccacctgc
agagtttggtcaatgaaatgtcagcatcaaccatttccttaaatgaagatgacatgtcaaagaaaagaaa
ctggattattcagcagaacgaagctaaaggtgcaaatgatccgagacagcttgatctttcctttgactgt
cggtacaatgcgacccggatggtttcttcatacaagccaggacaatcaggatcccaggcatatggtgtgg
ctatagagaaccacagtgagtataagtttgtgcttgctcttgcgattgagaacaaactttgcttgacggg
agcactgttaaagaggaagcataagtacgaggaggtgaaatgtcctggaaaacatgaaggttgcactgcc
aacatcccatacatgcagccccattctgagagacgcatggcgcatgatgtcgctcagcaactatgggagg
acgacatagtggttcgaaccctgacgacagatggcgatacgaggtcatttttgggcatgcaggacttta
ccgagaactaggagaagcttgggatgttacgcggcaagcagacccacaccatttgggaagcactgtagtc
agacactcgagaaaagcaaactggagtagagacatgttccctaacaaagtgacacgactgaccaagcagc
aagccatagcagcttttgcaaaagacgtcaagtcacgatgctctaaaataatcagtaaactgcggattct
tggagatggggacctcaggcgaacattacctttacttccgagtgtttgttctgctacggttgaatgctat
tctggcaactgtggtctatgtcctcatagatcactggtctgcaatggagttgggggccaaggcgactggt
ggttccactcagagtttttgccgacgcacaacataaacagcctgaaaatgaccgaacaggacaaggaact
actcggaagcattctgcagataaggctgagtgagcaggctataatcaatgtggcctctaatacagacaca
cagaaatgcgaggcattcaaccgtggtgtgctgtcaattttgccgaaggaagtgaattactgtcggaact
ttgctggaaaactagcctctaaaacgcttcagctaaacaactctctagttacagctattgaagccaaagt
gagtttgataactggtgaaagcctctctgcgaaaccaaagagctacttacgacaaaggtctcgtgtgtct
gcagcacgaaaaagacttcagggaaccactgcattcaaggccagaagacgtaagcggagagctgagctgg
agcacacctatcacacagctcgtacaggtgcgactgctgaaagacaagacgaatatgtcaaaggactgtt
agacaaaatgggctgctgacatgctaccacactgtgacacagtgacacagtgggcggggtggatggacag
agctagaattgagttacgattgttttggagaaaaaaaatcccccgtctgattcaatggcagaatcactga
tgagacctggtgccggtcgaaacgggaacttttacagaccgccgaaataaaatgttgaaaaaataccccc
ccacccccccccccccccaccccccaccccaccccccccaattaaatatgtaatacacacacacactt
acacacaggcacaattgaaaacaattcaattctcacttaaatacttgaatatttgatctaaatgatatga
aacataaacattcaaagaactgttactattatttcaggttaagctctatcagacagtccggtgcacggtt
tcctgcatccagcatgtgaccgatgcacttagacattcgaaaacatgtacgcctctggcaatttatccga
ctttgctcaaaaacaagatgatctgtgttcacacacagcttgttattgcacaaatgagaggcatgataat
gtcgggaaacgtccaaacttctcattttgaccatgagagctgcacgattcgcagcacaggtcttatgcga
agccagaaaacccgtctgggatctttatatctaaattggccatagccatttactcctattgatccagtc
cacattaggcactggccatttgcatgcaagcaactgtttgctgtcaatttctcagtgcaaagcttccaaa
actcttccattttgagttgaaactgaaattcttgacttaaaaaagtccaaatactgagcaaaaaggtgct
taaaaaacagggaatacacaaaaaatcaacttatatttgaaattcaagcaatgtcactgaagagctcctg
ttagcagacgatagtttgttgtaaacagtgcaagggagacaactcatccttatctaaaacaatgatttt
ttaaattctcaataaatcaaaaaaaactcaaatcagtgcctagtgtctcctgttataagcttctgtagct
aaaaataacttgcatgacaagtttcaggcctgtaaaccttaccgttttgaagattttccattttgacaga
tttcagatta
>KolobokP-5_GarTel-I
taaatatcaccgaaatgttgaaaaattctagcgaaaactatttttcctaggtatttcatatgcggtaact
gccaaaaaacattcaaaacatggctcaatgattttatatcgaaacagtaggggttcaaacacaagacccc
tttgtgtgtttcagctgcctcgggaggtacaaacttgcctccactacctctgtcactaaaaatgctattt
ctggaaaaccactggatggatttgcatgaaataaagtataaaacatctgtctgatcttgttgtattttt
catctacaaattttatgtctttaaagccagcttcaatgcttaaattaaaaaaaaatgctaaagtttgaat
ttaaaatttaaaaaatcctgaaattatcacattttaaakccattcwaaaggataaaaagcatttttcag
tacaaaattgtaaaacaaataccattcwtatacccacagaaktatcttagaggcaatttgtctgtatgtt
tgaacaagagatttactgtacccagccactacagaacagcttaggcaggcagkctgtcawgataatcccw
tccaagaggcccaaagacaatgctactaawtwgcatatmtgsaaatagatacagcatgcacacccatgta
tatatgagcccttatgtcagtgcatcaaactggacatacacakcactttcaaagatctggmgagaagcat
ttcaagctacmatggtcaagggaaaactaaaaagcagggctgctggaagccaatttaaaagtttaaagga
agaaaagtttaaaacactaaagcaagcattcgtacgaagagtcattaagccgtcctcgtcaaaaattaat
actgcaaccgggcttcaggaatctagggcatcgcgtctgggaccgactttaagacctcggaaagaagaca
tccagcaatctcttgggaatcgcatcattaacttggacatgatattgataatgatgaataacctctttaa
atcccatcgaaacagttcaaaacattgtgaccagctgacattgggatcgcgaatggagcagaaacacggc
cttggctcaaggctacagttccagtgtgaaacctgccactttgtaagcgatagctacaaaacctatcagc
cttgcgcgaatagccgaggggctgccataaacaaaatgcttgcatcagccctcatggacatgccgatagg
agtagaaaaggcaaatctgctgttaacttgcttagacatacctccaccatgcagaagttacttgcaggag
cttgtgaataaagcaagtactgatgtcgagcaactaaaccagtctgacatggccgagaaacgacagttgg
ttgtcaagcataaccgtgaacgtggacttcgaaatccaaaacacttggatatatcctttgatggccgcta
caatgcaactcgtatggtttcatcctacaagccaggacaggcagcctcccaggcctatggagtcgccatt
gagaaccacacaagctaccagtacattgtggcattggctgtggaaaacaaactgtgttggactggggcaa
atctgaagaatagaaattttagagttaaatgccctggtccccatgagggaagcacagctaccaaagagta
ctttgctccacattctgaaaggagtatggctttcagcatagccgagcagctgtcgcaagaagacctgctt
gttcggaccctaacaacagacggggatgcaaaggctttcttgggaatgacagatttctacaacaagggcc
actatggccctaaatcgctcacctggtaaccatgtaaccatgtacaccgtgtgacctaggtatttgacck
ggcatgaccctcattctaacttgacctcgattkcaatgagacaaacattttgttcaagtttcatgaagat
ccaatgaattttgtagtctctatagagtgttaacaaggtttttcgatgatttgacctagtgacctagttt
ttgaccggacataacccacattcgaacttggcctagatttcattgaggcaatcatattgatgaagtttca
tgaaatccaatgaaatttgtagtctctagagtgttaacaaggtttttcgatgatttgacctagtgaccta
gtttttgaccggacatgacccacattcgcacttggcctagatttcattgaggcaacatatcgatgaagtt
tcatsaagatcmaatgaaatttgtagcctctagagtgttaacaaggtttttcgataatttgacctagtga
cctagtttttaaccggacatgacccacattcgaacttggcctagattcattgaggcaaacattttgatga
agtttcatgaagatccaatgaaatttgtagtctctagagtgttaacaaggtttttcgaagatttgaccta

gtgacctagtttttgaccggacatgacccacattcgaacttggcctagatttcattggggcaatcatatt
gatgaagtttcatgaagatccaatgaaatttgtagcctctagagtgttaacaaggtttttagataatttg
acctagtgacctagtttttgaccggacatgacccacattcgaacttgcctagatttcatgaggcaaacat
tgtgaccaagtttcatcaagatccaatgagatttgtagctgctagagtgttaacgaacaattgtggacgg
acagacgacggacgacggacatccagcgatcctaatggaaggtcasccgacaatctgacccataccatct
tgccagtcgacaataccgtaaagcacgcagtgctcagttcagcmagaatatgtttcccaaaaggaaaaac
aaagaagccagagggattgcccgagcacagccctggcaagggacataaaggcccggtgctcagctgtcct
tgaccgactgcgggaaattggacatggagatgtttcgcagcagatagaacgcttacctgcaatctgttct
gcgacagttgagtgctattcaggcaattgcagtttctgtccacatgattcactcgtctgctctggactaa
gaagaaaaggtgactggtggtacaaatcagcctttctacaacctcatggaattaacagtttgaaaatgac
aacaaatgaccgagagttgwtgtsgaccattttagagatacgactcagcgaacaagccgtattgagcgtg
aaaagtggcacctcaacacaaaagtgtgaagcstttaatmgggcaaccttgtcaactatgccgaaggaaa
taaatatgtcaagaaactttgcaggtgcattggcatcgaagacattacagttgaacaactcacttaaggc
ttccgttcaaaagaaagtactcggtataacaggcagagaactgtctccaagagcttcccaatacctaaca
gccacatccagaagagcaaacagtcacagaaattatcagaagacattaacttacaagagaaggcggcggt
acaggcgagcaaggcttgaaaaccggtaccgtctaatgagaaataagggtctaattgtagaggagtacgt
caaggagaagacagacggcacctatgaaaaaatgtaatgcttccttacattttaaatgacaggacagagt
gaaagtgcagccccggacatgataatacagatgatcaaaaactctgtcacattcaaaggatgtctgatga
ggtctgaagctgaccgaaacagaggcttataatgaaacacacagacaccccccccccacccctttatcct
gcaatatgcaacaacaacaacaactatgaacccccccccccccccaaaaaaaaaaacaaaacaacaaca
ttttccagatgcacacatacacatggtaggtgcaggcatgacaagaattaaaataaagatttgttaccta
cccactgttgatgtatctgtctgtctctatatcctatttcaagtcaacaaggcagtctggcagtctgttc
ccattctcatccatatggccattgcacctagaatttttaaaacataatttcctgagattgttggttttgc
ttttctcaaaagtcagatgctctacatttatacatattttattatcacacagatgtgacgcctccatttc
tggaggaatgtcaaagtccctgaacttcaccataagtgccacacggtgggcggctcgagtcttgtggcca
gcggacatagggtctctagggtctctgtacctgaacacgccataaccatacttattcttacagcctgtcc
aaatgcgacattgtgtattaggtgcaagaagactgtttgcttgtagtttctgtttacattcttcaaagag
cctgtccatgttaaacagtatcaaaacacaaaatataaggcagaaagacaaatacagggttaaattcaac
ttttaaaaaacagatttgcaattcaaatttataataaaataatgaactatccttctctctggtagattta
gtctcagcttaggttagcagccgacaaaaactgttgcagtcaaagggagactactctgccgggcatgatt
cagtcaagtgcataatgttgaaaatttaaaaaacatcataaaatgaaaattcaagcttggatcttcaaat
gcttttcaaatggtgatttatattgccaaacgctacctgcatgtgaagtttaaagtagatctgaccagta
cttttgaaaatggaaaatttcatata
>KolobokP-1_DrRo-I
ggtttagtgttatgagacctttaagagaacccaaccctttctgatttcaataaaaccatatttttaata
agtatgatgtattttcagaaagaatataaagtactgtcaagctcaagtgaggatttattggaaaaacaag
gcattggaaataacacatgcaaatgcattttgagttggaaattaagatcagaaaaaactgtttgggtaca
tgttgaaaacatagttttaatccaaaaataagcatggctttgagtttcttaaggcatagaagtaacataa
tatgagaacagagttaacaacactcttcagcggtattttaaattgaacaaaatggtactttaggctcttt
ttcacatttttttttttatttttttttcacatttttttggggtgattagtagattttagtagcatttcagat
gtcatttgtccccataaaagttttttttttacatattcattaacagacattcacttctggcacatacaga
cacaaatctgaccataaagtgtcaataccgggtattaatacacagtctgatgtgacactagagcctccag
ggcagctgtgaaatccttcttggccagtgtacagtactacccagactgtctcaggtactctctgctgtgc
aatctgtgaaaatagaaggtttttatatgcattttgatcaaatataggattatttagtgtaagtacagta
tttgtttctcacactgtacactgtgtaggttgttttattaagtaatatagtcagtaactatgaggctgtc
taagcgacgagctcatttacgaaaaagtggcttcaaaaaggggtgtgtgagtccaaaaaagggtcaaact
gtagcttttcctactcttgctgacaataagtttgtcaggttggaacagaaagcctttgatagtagagtac
acaccagtaacaatgtactaacctttaaagatactgatggtacggacactacggtgtcaccgttgcgacc
ccgaccgaatgcgtctaatgtagttgacgagtatagtggctgtgatgacgagtcaattcatccagacttg
tttaccaataagttgatggttcaggctaaggtccaggctcttttttaattctgcattcaaagaacatagac
atgataaaccaaactgtgaggggggatctgaattttgatgctgctcacgcaattaagtggggtgcaggttg
gcgtgagcggctaaaatgtacaaagtgttcatacgtgagcgcttatcacaaactgtatgaggaggttgag
aataaaaaaggtccgggtaggcgtgccgcaaaggtaaacattggccttcagcttggtctgtgtactacac
caataagtaatactggtttgcaaagaatttttaataatgcaaacatcatagccccaaacctgacctccat
gcaaagattaagtaataaagtaaatgaaaagatacaaagtgtcaatataagggacatgcgtgagcaaaga
gtcactcttgttaaagaaaatgccatgataggtcaaaaaaaatgcccaaactgtaaatgtggagggtgact
cttgctacaataaccctatgttcaattccgattcaaccccatttcaggcggggtccattgcgacaaccac
attttgtgaaaataacactaaagacaagaaaataatagggggtacacattgccaataaactttgcacgatt
ggagctagattgcgaaacaaggggaacgatgtcgtttgtcctaatcatagtggtcattgctcggcaaacg
tgtccgagtcagatccaataggtaacgaaggtagatggtctgaaattgtggcccgtaacatatcaaatga
ggttcaagttactggttatactggtgacggtgactccaggagtcatgcaggggtgagcaaagcaagtaaa
catacaattctacactttaaagacataaggcacttgggaaattcactaaagcgtgctgtgtacgcagcca
actttagttcaaatatgttcaaagggtcgtcaaacacaaacctaaagaatcgctttgctctgtcaattaa
acagcggtgtgtagcagagttgaaaatggcccacaaatcttataacggtgacataaacaaaataaaacaa
tgtatgccaaaggtagccgatgcaattgttgcttgctttggcgggtattgtggtgagccctgcaaagcat
ctagcttagtttgctgcggtaactatagacaggccaaaaactatatgccgatgaacgtaaaactaagaat
gacagagggtgacaaggtgttgcttttaaaatgcatggacatcatgctgtctcccacagcccttgataaa
acaaaattattgacatctacgcaaaagtgtgaggccgtgaatcgatcctaccaaactgtaaacccaaaaa
tgatcactaacccaagaaactttgctggcagaattcatggccaggttcataaacttaacaatgggtatgc
agtatcagtgctgataaaaaccaaatcactgggggcaaatcttacaaaaggttcttcggtcatacgtcaa
atacggacgaccgatgccatatcaaaaaagccaaacaaagatgttatccaaagacgcaaaattactaggt
atgcggcacgacagcgccgttaccatttacatgaacgtttgcattacaataaaggtctcactgacactgt
gcatggtatggaaggtttcggacagttacgagaccacaattacgtataatataatatgtcggccacatat
ttaagtagttattgcccccgacatgttatttgacatgccaacgagttacttatgttgtcatttgctgcct
tgtgtttttatgcatcaataaatagcaaaaaaaaactattttaggccaatattgtattattacaaaaaaa
aatggtgttaaatatgattagtttagtttgcaaagaaagaaaatggctaacttttagacaaggcagtcag
ggcggtcaacatgccctgtgcatctgccgattcgggcgcaaactttacgagcattattgaccgaaactgg

ttctaaaactaaatgatagggactgcaacacaaggttttatgacataaatgagataaatgctgagaggga
ttcaaatttaagcaaaagtggaactgaaaaacaatgcgatgtacatagtgggtcttggatgggtgctcag
gtggacattttgctttgattttcccatatgtaacccccctttgtacactggagcacccatcccacataat
acaccctttaactgggtccaaatgacatcttttatagatttttttccaaaactgcctgcttataagcatcc
attttgtcttttttttgcagacgataaataattccggatatgcttaaa
>KolobokP-1_DrPo-I
tttaagagaacccaaccctttcwgatwtcaataaaacmatattttttaataagcatgatgtatwttcaga
aagaatatwgagtactgtcaakctcaagtgaggatttattggaaaaacaaggcattggaaataacacatg
caaatgcattttkagttggaaactaagagcagaaaaaactttttgggtwcatgttgaaaacatagtttta
atccaaaaataagcatggctttgagtttcctaakgcatagaaggtggcawawatgagaacagagttaaca
acactcttcagcggtattctaaattgaacaaaatggtamtttaggmtctttcgagcattttttttccgca
tttttggggggtgatttgaagatttaagtagcattgaagatgtcatttgtccccagaaaaggttttttttac
atatgcattaacagacattcacttctggcatctacagacacaaatctgaccccaaagtgtcaataccggg
tgttcatacacagtctgatgtgacactagagcctgcagggcaggtgtgaaatccttctgggccagtgttc
agtactccccagactgtctcaggtactctctgcagtgcaatctgtgaaaataaaaggtttttatatgcat
tttgatcaaatataggattatttagtgtaagtctagtatatttttctcacactgtaaactgtgtaggttg
ttttattaagtaatagagtcagtaactatggggctgtctaagcgacgagctcatttacgsaaagtttggt
tcaaaaaggggagtgtgagtccaaaaaagggtcaaactgtagtttttcctactgttgctgacaataagta
tgtcaggttggaaaagaaagcctttgagagtagagtacacatcaataacaatgtactaacctttaaagat
actgatggtacggacacaacagtgtcagcgttgcgaccccgaccgaacgcgtctaatgtagttgacgagt
atagtggctgtggtgacgagtcacttcatccagacttgtataccaataagttgatggttcaggctaaggt
ccaggctctttttaattcttccttcaaaaaacatagacatgataagccgaactgtgaggggggatctgaat
tttgatgccgctaactcagttaggtggggtatgggttggcgtgagcggctaaaatgtacaaagtgttcat
acatgagcgattatcacaatctgtatgaggaggttgagaataaaaaaggtccgggtaggcgtgccgcaaa
ggtaaacattggccttcagcttggtctgtgtactacaccaattagcaatacaggtgtacgcagaattttt
aataatgcaaacatcatagccccaaacctagccgccatgcaaagattaagtaataaagtaaatgaaaaga
tacaaagtgtcaatattagggacatgcatgagcaaagagtcactcttgtaaaagaaaatgccataatagg
tcaaaaaaatgccaaaactgtaaatgtggagggtgactcttgctacaataaccctatgttcaattccgat
tcaaccccatttcaggcggggtccattgcgacaaccacattttgtgaaaataacactaaaaacaagaaaa
taataggggtacacattgccaataaactttgcacgataggagctagattgcgaaacaaggggaacaatgt
tgtttgccctaatcataagggtcattgctcggcaaacatgtccgagtcagatccaataggtgacgaaggt
agatggtctgaaattgtggcccgtaacatatcaaatgaggttcaagttactggttatactggtgatggtg
actccaggagtcatgcaggggtgagcaaagcaagtgaacacacaattctacactttaaagacataaggca
cttgggaaattcactgaagcgtgctgtgtacgcagccaactttagttcaaatatgttcaaagggtcgtca
aacgcaaacctaaagaatcgctttgctctgtcaattaaacaccggtgtatagcagagttgaatatggccc
acaaatcttataacggtgacctaaacaaaattaaacaatgtatgccaaaggtagccgatgcaattgttgc
ttgctttggcgggtattgtggtgagccctgcaaagcatctagcttagtttgctgcggtaactatagacag
gccaaaaactatatgcccatgaccgtaaaactaagaatgactgagggtgacaaggtgttgcttttaaaat
gcatggacatcatgctgtctcccatagcccttgataaaacaaaattattgacatctacgcaaaagtgtga
ggccgtgaatcgatcctaccaagctgtaaacccaaaaatgatcactaacccaaggaactttgcgggaagg
attcatggccaggttcttaaacttaacaatgggtatgcagtatcagtgctgataaaaaccaaatcactgg
gggcaactcttacaaaaggttcttcggtcatacgtcaaataaggaagaccgatgccatatcaaaaaggcc
aaacaatgatgttatccaaaaacgcaaaattactaggtatgcggcacgacagcgccgttaccatttacat
gaacgcttgcattacaataaaggtctcactgacactgtacatggtattagaggtgtcggacagttacaag
accacaattacgtataatttaatacgtcggccacatattaagcagttatttcatcggacatgttatttaa
caagccgacgagttacttatgttgtcgtttgctgccttgtgtttttatgcatcaatacatatgaaaaaag
ttattttaggccaattttgtatgattaaatcaattaaacacatgattacaaaaaacaaatggtgttaaat
aggattagtttagtttgcaaagacaaaaaatggctaacttttagacaaggcaggcagggtggtcaacatg
acctgtgcatctgccgctttgggcgcaaacttttcgagcattattgaccgaaaccggttctataactaaa
tgatggggactgcaacacaaggttttatgacataaatgggataaatgctgagagggatccaaattaatgc
caaagtgaaactgaaaaactatgcgatgtacatagtgggtcttggatgggtgctcgggtggacattttgc
tttgattttcccatatctaacccccctatgttcactggagcacccatcccacataatacaccctttaact
gggtccaaatgacatctgccatatatttttttccaaaacagcctgcttatatgcctccattttgtcttttt
ttgcagacgacaaattattccggatgtgcttaaa
>KolobokP-2_DrPo-I
ttaagtttttttaatatatgcatgataacacaaaaagcttttgttatattgaaatataatgtttaataaat
taaatgcaacatttcttaactgtatactgtgttttgtttgtattttacagtaattccaaaatttcacaca
attcaagggaaacaaccagatcagaaataatccaacaaatagtgttcctattcacaacatttgactctat
ttattagagaagcacaagttattggtcaaatgtttaaaatgatattgacaatatcaatcagtcttacaga
ggtatattccmctatgaatacatagtacatgcaccaaatattgacattcatggtcaatgccgagagaaag
tacagtctcaaaagttatttgaacctataattgtgcacagattacaaaaagacactggataaaataccca
tactccatctggagtagcacatacttagattcatagatcatgaccaaacatagtcccaaagctactggcc
cttcagtgttttaaagccaaaatgcagctttattgaattctgttttcactgtacacaagggagctaaatc
ctatgtccatatacaagtttgactcctctgtatttgcatgtgtactaattggtacaataaaacaaacact
atctggacacctgactctgctgcactcattatgtagataaaaaagaccagaatttgctaaaacaatgaag
tggccttattgtttacaagtctatagtgttgagcagatgtatcttttaaaacaccttggataaaatacca
ttcaaaatgagatcacttcataagaaatcacagcacagttataaaaacaatcgcgctaggaaaaacaaag
gtgcacttttcaaaaaaggagtatttccaaactataacagcagtcatgtgcgctgtgtagagaaacaaga
tcaagattccattccgttgaatttagaccctgacctggaaagtgtcgtgcgattgggtgattctgtggct
aacgatgtcttgaatgcgtgtaaccgttctcgagatccactcgttctcccttttaaactgcgaccacgtc
aaacagtaccacagcctgagaaacaaagtgcaaatatatctgacaatgagaacatcattgtaaacataag
tcagtttggaaacattttcaagtgcatagggaatcacagctgtgatcaacctagcctggattacaatatc
acagacagaaaaggactatgtgtggatattaaggtgttatgccacaactgtgacttttgctcagatgaaa
ttccattatacaccagcatcccacagaagcatggcaagccactaggagtcctgaatgtcagcttgcttct
tccagtgctgtgctcaaaattgggcatatcagacatacagttactgctggcctgcctcaatatacaggct
ccagacaagaggggcatgcagaggaagctgaacactgttgctgaccaagtggaagacatgggacgtcagc
aactagtgaaaatcaggaatatgtgaggcgtatttcaaaccttgctgggtgttccggtgaaacagatgt

ggagtttgatgtcgcgtatacaagcagaccacaagctggatgtgacaccgcaactcaagttttcgcacct
gtaattgagaaaacaacatctaaacacctgcctgtagatcttcacattggaaataaactttgcagcaaac
ccaactgtaatcatttagacagatcttgcaaaaagaattattgtgacgaaacatcaattaatcaggcaga
ggcaatatttcttaaaaaatctttggaaaatatcaaaaatcaaaatattctaaaagttaagtctgtgacc
actgatggtagtgcttcacttgccaaagctatgagggagcacaatgccaaggtacaggagaaagtccaac
atttcaagtgctttattcataatatgcgaaatctacacaagcacttgcgttccgcatgcataaagcagcc
aaagggaatggataggctcctgtactgcaagaaattagctacagctatccgtaccagagtacggctcgaa
cttaccagactgaggaagcttctgcgtggggacgagttgtaccttgcccgggctcgagaagcagtcacta
atgttctagattgtttctcggggtcacacgatagctgcaagcacaaatctgttgtctgcactgcacatct
gaaaggacacagtacaaggtatttgccatatggcaagaatgttgtgttgtcagcagcagacaaacaaaaa
ttgcagaaggtcttggacaagtactgtggagtacaacagctaagggacactgtgcaactacacaatacta
acagatgtgaaaacatgcacagtaggctgttttcatatgctcccaagagcatggtctggaaaagatcatt
tacagctctatgctactccgcaacactcggtgcaagcgtgggccgtggcttatccttacttcaacttgct
gggcgctgtggcattaatatcgaggcaagcgatccgatgtacagatatgctatgttcacacagaacatag
cacggtaccacagtgatcgcaaacaaaaacatgaatacaagacatctcgatatttaagccacagaaaaag
ggcgaatagggctgtactttcccagtcattgtacaaggctccaagtaaagttacaaaagaacatgactat
ggaatcaatcttggaaattaaaatgtgtaaatagaaccaaaacaatataacaatttaactgtgtagcagc
aatatttgtaacattaattttacaacagtgacaggtgtccaaactatttatattacctcaaatcttgcaa
cctgaatagccataatgaccatgacataaaccttcatgagcacatatttgtctgttgttattaatgtttt
gaggctcataacttaagtgcttgatgttgacacaaagtttattatggcataaatgcgacacatgcatttt
tgatgacatggcatgtttagtttcaagaaaatatagtgcgcggtgagctttaagccttagccttttgccg
cggaaactacaccgcagaacaccatagccgttcggagtgcatgccccttccagatgtggcattcattta
ttatttggtatttattatcaaataaatgcttcaatcttagtctttcttcatctgttaggtgtgccatgaa
gaaatatgttgccatgatgactttaaataagaagaaaaaataatttatgtttgcggcatgttacaaacca
ctattttgcatggttttcctattttaacaactttttctacaaaactgcatacttatcttgcttattttag
tattcgagctcagaaaatgtgcaaatgtagatgctagtataaaaatatgagtttaaacattgttttccag
tgcaaaaccagcacacgtgaaattttcctaaaataagcaatgtttttaacattattacctccctttgtt
ttccataatctaaaccatagcggttaaaagatatatgctaaattatgttcgatttgttaccgcaacacat
ttgtggaatgtttgtatcaatttttgtatgataaacagaattttaaaattttgcattttgctattaa
>KolobokP-3_DrPo-I
tttaagggaactcaaccttgtcctacctttttcaaactttctccacatgagatttgaactatttgtacaa
tgaatatgcaatttgagttcattcggatgggcataaaggccttaaaatggcgattaaaagaggcgactaa
attggccgcakcagtgtcgaaatgcacgattttwagtcttagtcacgacagctgatttttttgtccccaat
aattattacgcgtaactttaatggcaaaaamtggtaccattgcatgcgaaattaatcaatacatcaggaa
aaggccccaataaatgtattaattgtgaatcagtacacttttcttcatgaaaaatgagattttttttaaa
ttttaagcctttttttttcaatgaaaaactcacttacagcctataaaakcatgtttttttaagaaaattatt
attattwgctttaatgacaattaaaacattcaaactgcagttaaaagaatgaatggaatgatgataagag
gcaaaatttgcagctttcaagccaatgcaagcctacaacccaaaattatcactcaaaaaggtgctaattg
cttcttcgacagcttacgatgcaatgtgatgtttttttgcacatccagatagtctctttcctatataaagg
tcttattagctacatttcattcagtgttctctctcaaacccagtggtgatagcttaagttgtagcaaaaa
tgcccagagtctctaagcaaaaagcatgtttgctaaagaattcctatgcgaagggtcacactagtgtcat
gatgggaaagtcatttgggtatgaatcctctgtagttcagcgttcattcgttcgactacctggggatgac
tatcaggatcgaatttcccagcttgatgaagtgttgacctttcaggatgttgatggatcgtccacaagcg
ctgcaccactgcggccgcggtccaggacacggagtaggattgaggagtacacaaacccggagtcaggtaa
ggttcatccagacctattaacaaataaaatttataggcccctggaacttggcattatgttcaatactgaa
ataaggaaacatatagaggagacatcatgccatggctttctcatgattgacacggcaaactcgaggaact
ggggacttggctctagtgagcggctaaaatgtgactcgtgctcgtatgtaagcccgcactataggctgta
tgaggagttggaaaccggcaaacggggcagaaaacctgccaaaatcaatgtcggcttacaaacaggtctc
atgaccactcccatatccaatactggcatgagtcgcattctggcacacgctaatattgcccccccccccc
aaatgtatccgcgatgcatcgggctgccggtaaagtgtctgaggcaatggtagccctcaatgagaaagac
atgcatgacataagggaaaagatcaagcaggacaataggctgtgtggattaaaggatggaaccaaagtga
atgtggagggtgacacttgctataacaacccactgttcaactctggtggacacaccccgttccaaggtgg
tacgattgcggttacaaccatgtgcgaaaacaacacccggtccaaaaggatcattggtgttcatgtcgct
aacaagttatgcatggttgccagccgccttcgtaaccaaggaattgcagtggattgtccaaatcatgacg
gaaaatgtacggcaaacatgtctgagacgggcgttattgggaacgaagaaaagtggaacgagcaggttgc
ccggaaaattaacacagaccttaacatagcctcattcactggggatggtgactccaagggccacagtggg
gttgataaggctcaggtccagcaaacagttcacttcaaagatttgaggcacttaggtaactctctgaaga
gagctatcaataaggctcaatttagcagtggaatgtttgctggacccgcttcgaagagagcaaactttca
aaacaggtttgctctgtcaatccgagcacgatgcatgtctgagttgacaagagcacacaaaaaatacaaa
ggtaatataaaggaaattaaaaatcatatgcccaaagtaatttcaagtattattttatgctacaagggct
actgtggagcatactgctcaaagcatagtttggcctgtcgtggttctgctggcggcaagaacaaagcaaa
gttatatctgccagagaactgtaagctcaagatagcaatatctgatgaagctctgcttaagacatgcatc
caaattgtacttggtcctgaaagcattgacagtacaaggcttcagacctcaacccaaaagtgtgaggctg
tcaatcgcgcttaccagaccgcaatgcccaagacagtgactttctcccgtaactgcacaggccgcatcca
cagcacaatccttaagctcaatcatggtctggctgactctgcaatagtaaaatctgaatttacaggtgct
catttgtctaaaggttctagggttattgcctatcttcttaaaagcaaacataatgacatgctaaagaaga
catgtgcatttcagagaagacgtaaggctgcccggtacttggctaggaaaaggcgctatgctcttcactc
tgaaattcattattctaagggcttgacagatccaaagccagatttcagtgacatctcacagctaaatgac
cacagctacagctaaatgtgcttcataaatagaaaagaaagttacttttctgaagaactagtgctactaa
ctcatcggctatgttgctccactggccgcaagtctgtaaatagtgtacatatgtaaataggtagtctact
gcatgcaccgcacccatatatccactgtaaatacacaacttagtaagtgcttttaccagaaaacagaaat
tcatgtttaaattaaacaatcaggataatgatgcccatttaaatcatgtccaatgcaccggtcagagcaa
gacagccgctgattgttcactatctgcggctcaagggacaaatggtcaggcctacaacaaagagaaaaat
ggcataaatgagatacatgcatgtcatttggcatatatggcatcttgtgttccacctgaaacttcaatct
atggacataataatatttactaacaagatcagttggaaatttaacccttatccttccataccgagtcccc
ttcccatcccgaacagtcccaaaccacctaaaacacccctttggaccaaaatattggcatctttcatcta
ttcttgcacccaaatcatccaaaaaatcattaaaactcattttctacttatcttgcctgcagacgaatct

ctgtgaccttgacattgcagtaaaagtgctttttaaa
>KolobokP-3N1_DrPo-I
tttaagggaactcaactttgtcccatctttttcaaactttctccacatgagatttcaactatttccacaa
tgaatatgcaatttcagtgcattcggatgggggtaaaggccttaaaatggccatttaaagcggtgactaa
attggccgcagtcaagtcggaatgcatgatttttagtctaagtgacgacagctgattttgtgtctccaat
tattgttacgcgtaacttttatggcaaaaactggtatcattgcatgcgaaattcaccaatatatcagcaa
aaggcccaaataaatgtattgattgtgaatcagtatacttttcttgatgaaataggagactttttttaaa
ttttaagcactttttttcaataaaaaactcactgacagcatataaaatcatgtttttttaagaaaattatta
ttaaaagcttcacttacaatctaaacatacacattgcaattacaaaaattaatgctattatgatgagagg
cataatttgcagctttcaagccgatacaagccggctacccaaaattaacacttaaaaaggcgcaaatcgc
tccttcaacagcttacgacacaaagtgacacttaatgcgcatccagatagtctctctcatataaataagt
cctgttagcgacattttattaagtattctctttaaaatccagtgttgaaagcgtaagttttgcaaaaatg
cccacagtcaccatgcaaaagagcacgtttgctaaggaattcctacgcgagtggccacactaatgtcgtg
gcaggaaagtcatttgggtgtgattcctctgtagttcagtgtacattcattccactacctggggatgact
atcaggatcaattttcccagcttggtgtagtgttgacctttcaggatgttgatggatcgtccacaagcgc
tgcaccactgcggcctctgttcaggacacagagtcggattgaggtgtacacaaacccgcagtcaggcaag
gatcatccagacctttaataaaataaaatgtttaggcccctgaaactggcaattatgttcaacactgaga
taaggaaacacatagaggagacatcatgccatggctttctcattatcgacacggcaaactcgagcagctg
gggacttggctctaaacaaaaacaggtatgctccatcaatgtgagcaagatggcatgtcagagttaaaaa
gggcaaacaaacatacaaaagagatataaaggaaattagaaattaaatgccagaagtaattttaagtatt
gtttttctgtctgcaagggttgctgtggaaaatagtgcttaacgcatagtttggcctgtagggattctgc
tagcaagaacaaatggaagtcatatctgcccgcgaactgtaagcttgagataaccatatctgatgaagta
ctgctttgagaatgcattctgattgtacttgaaccagatcgcattgacagcacaaggcttcagaccccccc
aaaagtgtgaggctttcaatagtgcttacctgataccagaccgcattgcccaagacagtgactttctccc
ggaactgcacaggctgcatccgcagcacaatccttaagctcaatcatggtctggctgactctgaaatagt
gaaatctgaatttacagatgctcatttgtctaaaggttatgagtttattgcctatcttattaaaagcaaa
cacaatgacatgcttaagaagacatgtgcatttctgaaaagacatacagctgcccgatacttgactagga
aaaggcgctatggtcttcactcttaaattcattactctaagggcttaacagaacaaaagccagatcttac
agctaaatggccccacatacagctaaatatactttataggttgaaaataaagttatttgtctgaagaact
agtgctattaactcatcagctatgttgctccactggccgcaattctgtaaacatagtgtatttatggaaa
tacctagtctaccacctgctgcgcaccactacatccactgtaaatacacagctaagttagtactttaacg
aacaaactgaaattcatgtgttaattaaacaatcagtatgatgactcacattgaaatcatgtccagtgca
ccagtcagagcaagacagctagcggattgttaaccaactacggctcaaggacaaacggtcaggcctacaa
acaaagagaaaaatggcataaatgggctgcatgcatgtaatttggcatatttttcatcttgctctccacc
tgaaacttcaatctatggacataataatatttacatttaagatttgttggaaatgttacccttacttgtc
cataccaggtcccccacccatccggaacagtccaaaaccacctaaaacatccatttggaccaaaatattg
acatctctcatctattttagcacccaaatcatcaaaacattcattaaaattcattttctacttgtctcgc
ttgcagacgaatccatgtgaccttgacattgcagtaaatgtgctttttaaa
>KolobokP-4_DrPo-I
ttaagggccagcatagtcttagaaactgctgaaaatgacttgttttcttaaaatttggacaaaaacaagc
aaatataatacatttgtaaccaattctgaaggcttgtctgttatttataaagcgtttaatccaacccctg
ttttctctttttttctatgccattttctgggttactgtagaagtcatggtaatgaaaagctacctcacag
aaacatattgcaacaataaaatgaagtcttaatcacattgaaatgataaatggtagtgcaggtgactagg
taagcctctaaatcataactttattacagaagcaacacaaaagaacacagtttggggccaataaatactt
tatgttgctaatttttttacatctctcccaataaatgcacatgtttttgcttagaataataaaaatata
aattaattgcatgcttagcatgaacttgaaaggcaagttaggcatttggacctttaatctgctgtaaaaa
cacaacattaaaggccccagtgtactaatcccttaaacaaacacatgcaagggaggtaacccaatcatag
cataaatatctggggctgacatattgaggaaataccccctgcgataacactaacaactgcctaaaaattga
catctgacataaatatcagcccaaatatcttggaaacagaaaattaaaatttctagtaagctcagatcat
tgtaaaaaggcatacaataaagtgaggcatttaccagagtcacttcttcacaataacttcttatttacat
ataaataacatcagttgaattgcgcacaatggccaaaagtaattcaaggcgcagttatagattaaataaa
gcacgcaaggcattttggtttagaccaggaaacacattccgacaaacaggccaaaatttacctagtccca
ctgaagatatcgctgttccatcagtacctgcaacggagcgtctttctgctgaagaggctgttgacgtact
tcacgcttcttcttcagggactaccttgccttacaagctacgaccaaaagctgaaataaaagacattcca
gactgcactagtgttgatgagaatgtcattgtcaatatgcagtgcttaagaaacattatacaacaggtac
atcagcaaaactgcaagaattcactggtttccgtgagtgttgtgcaccgacgtggactagctatttctat
ttgtgcggaatgcaggggctgccattataagtcaacacctatggagctatcaaatacaataaagaaacct
agggggccaggtgctggtgtcctaaatgaaatgattttgctgccagtgatgaaatccaaaatggggatgg
ctgatgtgtcactggtgttgtcatgcttgaacatcaagcctccaagtgactcacttatgcaaaagaaaat
gaacttgatgtctgacaaggcaacaacagtcaacgaagacgaaatgtgtaataaccagcattatgtcagt
cgcattctaacactgtcaggaaaagacaactctgctgatgtacagtttgacacatcgttttcatgcagac
cgcaaggtggtgcggaaaaagcaaagcagagttttgctcctcttatcgagcacaatacgtcaaagaaatt
tgtcctggcagcatcaatttccagcaaattttgcaagaaaaaagcctgcacacatgataattgctcaaaa
acactggctacagaccaatcgatatcgtcgaccgaacgaagttccctacacactaacctgaacagtgtcc
tgaagcgacgcgttttgaatattcgatctgtgacgacagacgcaagcagtcaagtggcgaaagctctgag
agactacaatacagagaataaaacaaacatgaaacattaccattgttttattcacaaactcagaactttg
gagaaaaaaatcaggaatatcaacttaaattcaaaacttaaggctgggcaaaacaagacaattttcctga
aatcacttgcaagtggaattcgtacccgtgcccgcatggagctgacaaaccttaaatccgtaagaagtaa
tgaggccgagttcattgagcgtagcacaaaagctcttgaaaacatagttccctgtttcgcggactcacat
gttgcatgctctaagcaatcgactgtctgccaacatcatcttgtgcactacggaaagtacagttataaac
atctaccctacggacaacaccttaccctgtcaaaagatgatcaaactactcttaaagatgccatcatgaa
cacgttcaacacagaaactttaagatcagttaaagaactctacagcacaaatcaatgcgaaagcatgcac
tcgacaattttcaattacgctccaaagtttacatgctggacgcgaaacttctcaggactatgtcactcag
caacacattcgcgaacactgggtcgaggacgggctacattgatactagcaagggctgttggcattaatgt
gaagaaaaactcacaaatgtacatgaatttgaatcgaatcgaccaaaaaagccagtatcattccaggcga
aagaagtcgcaagcatacaaacaatctcgatacttcctacggaaaagagtatcaaatcgaccgctgttac
aggagtccctgtactcatgtgagccgtccacttcaagccaggaccattcatatggcattacatactaaat

gtgttattaaatgcctttgcatttgccgggaacaatgttaacaagatgtttatgtgccagggaacaatgt
taacaagattgtaatgtgcaataggccctaaacagtagtaaatgctgcagaacggccattttgactcatt
attttgattgtattcttatattgtttgctgtatttctgacagtatatgaaaataaagttattcttcacca
cagagtagtaaattttccaacccggctacaaatttaacacatacttttacacagagctgttcgagaattg
cagatatcggtaaacatgattccgaattacctcccttgatttacaatcagtattggtcatacaatggtgc
tatggggcaaacactctgtttagaaacaaaaccttagggataaataattaagcaaaagcatatttcatgg
gtgtttgatcattttcataatttgatgttttaccttaa
>KolobokP-4N1_DrPo-I
ttaagggccagcaaagtcttagaactgcaaaatatggacttgttttcttgaaatttggacaaaaacaagc
aaatataatacatttttgaccaagtctgaaggcttgtttgttatttataaagcgtttaatccgacccctg
ttttcccttttttttctatgccattttcagggtcattgtagaagtcatggtagtgaaaagctacctcaca
gaaacatattgcaacaattaaatgaagtcttaatcacattgagatgacaaatggtagtgcaggtgacttg
gtcagcctttaaatcataactttatcaaagaagcaacacaaaaacacgcagttttgggccaataaatact
tggcgttgcttagatttttacagctctcacaacaaatgcacatgtttttgcttaaagtaataaaaatata
aataaattgcatgcttagcatgatcttgaaaggcaagttaagcatttgtacctttaatctgttgtaaaaa
cacaccatttaaggcccctgtgaagtaatcccttaaacaggcccatgcaagggagataacccaatcatag
cataaataattggggctgacatattgaggaaatacccctgatatgacactaatgattgcctaaaaattga
catctgagataaatattaatccatatttcttggaaacagaaaaagtaaaacttctagtaagctcagatca
ttgttaaaaggcatacaacaatgtgaggcacttgcaagagtcacttcttcacaataacttttttatttgct
tataaatatcatcagttgaattgcgcacaatggcgaaaagtaagttatggcgcagttatagattaaacaa
agcacgcaggacatttcggtatagaccaggaaacacattccgacaaacatgccaacatttacctagccca
ctgaagatgtcgttttaccttcagtwactgcaacggagtgtctttccgctgacaaggctgttgacgtatt
ttagttcttctccagggaccatcttgccttacaagcaaacgactaaaacctgaaataaatgacattccac
actgatttcaatgcgaaagcatgcacaatgttaacatggaaacaatgttaacaagattgttaccaggcca
taggtccttaaccgtactaaattctgcccaaacggccattttgaatcattgttttgattggattcttata
ttgtttgctataattttttgacattatatgaatataaagcccttcttcaccacagagtagtaagttgtcca
taccggctacaaattgaacacattcttcatacagaactgtttgagaattgcagaaaccagtaaacatgat
tccgaattacctcccttgatttacgaacagtattggtcatacaatagtgttatgggcaaacactctgtta
aaaaacaaaaccttaggaatatataattaagcaaaagcatgtttcatgggtgtttgatcattttggaaat
ttgtcgttttaccttaa
>KolobokP-5_DrPo-I
ttaagaaacaccatactatggtagaatcaggaaagttgactattttcatgaggtttactcatatatgagt
gtgtacaataaattttaaaatcctgggacgactcgtgtattttttatgccttgttttctacacatgataa
tttggtcattttcagcactgtttcccactatctgcagactgcactactgagccaggaattgcattatgtt
tgcaagcaaatttcaacaaacactgcatttttgtacccaatcatttaaaaaatgatgtccaaatgataat
taacagccaaacacttctgatcaatgcattggtgctgaaaaatcctcaaaaagcatagcaaaaaggttaa
tttttgaaagtttttacatattttcattaaaaaaagcccatttaattacatgatttacctttttcttatg
caaacctaccactgaattttcttctacaaacaagcacagtactttgagtaatgtaaacatttgattaaac
atggctttggctgccacaatggactaaagtgacaatatctcccaatgaaagggaggtaatccaaaagtta
tagagatgaccactggccaatcttaaaaattaacaaaaaatagatacagcacagggaaaaacaagaacct
tggcacttatcagatgggattaaacaaacttgtgcacatacaaagaaagtattttcatatcatgtgagaa
tcaaaggcttaaacaacaactggttatgtgcctgcagtggtcatttgtgaacttccgagttctagcgctg
acttattgttcaagagagagcttgtttaagaataaatgtgctcttttgctagagagatgacattttaggc
tgtgcttatcagctgttcatgttgtgaatgttgataagtgtgggtctgtttggtgaaagtggacacaatt
gtagcttaataacatgtgtctggaaagagtttagtctgttagaattcaggtttaaggtggtaacctcaaa
acatcatttattgtggatttacagttgagttattcaaaataacatcttttgtgatatatagttaaattaa
aaggtaaaataaactttacatcaaactgtgcattatcgcttactccaatgtagtgtgataaattaattta
ctgtccgaaacattaaatacaaaaccggaatacatcgctaaacttaccaaccggcaaaaacattaaaatg
gctaaccataaggttcgaagaagttacaaaaaatcaaggggggaaaagtcagtcatggttcaaacggggac
atgtcggctttagtagacagaccccacaagaaatacaaaatatgtcgacaagtgccgctgaagattcgca
aactaaacggctatctgtacgtgaggcagaagacgtacgacacgccgccgaggatataaatggagccact
ttgccgtatcaactgcgacctcaaccccaaaatgaagaaagggaagacatggcagaattagatgaaaaca
tcatagttaacatcgactgtttacacaacctggtgcgagaagtacacaaacagtcatgcagcaaaccatg
tgttaaagtcgcagtcacgaaacgagcaggcctttgtgtaaccgttcagatgaagtgcagcagctgcaag
tatcaggcgccgcctgttaacctcactgacacaatgaagaagtcgcgcggtccagcagctggtgtcctca
atgacatgcttgttctgccagtactcaagtccaaacttggcctgatggatgtatcccttgttctctcctg
cttaaacataaaggctccaactcaagcattgctacagaggaaattaaacaacatgtctaacacagtttct
cggttaaatgaagaccaaatgatgcacaaccagcaatatgtccacagagttctgcagctttccggtaaag
aggccgcagcagacgtacagtttgatacatcatatgcttcaagaccccagggagggtttgaaaaggctaa
acaaagctttgcagccgttatcgagcacaataccaagcagaaacttgtgctgtcaacagcgatcgcaaac
aaacactgtgccagcaaaacgtgcgctcataacaactgctcaaagaatttccctagtcaacaatctatag
catccagcgaacggcatttactccttgaaaatctaaacaacttggacaagaagaaaattctcaagatacg
ttcgatcaccacagacgcaagtgctcaaattggcaaagcgcttagggatttcaacagtaaaaaatcatca
aatataaaacattaccactgcttcatacacagacttagaacattagagaaaaaaataagaaacattaaac
tggattcaaaaatgttgggcacacaggacaaaacaaactacataaaaatgcttgccagtggtgttcgaac
acgtgtgcgaatggaacttcaaaatttaaagacaataaaatcaaacgatggcaattactttgtaacaaga
agttcacaggctcttgctaacattattccgtgtttcagcaacgatcattcaaagtgctcaaaagcgtcta
ccgtgtgccagcaccacatgtcaagcattggaaagtacagcgtaaaacatttgccatacggacagcacct
tcatctttcgaatacggatagcagccgtctcaaagacactatatcaagtacttttgataccgaaacactt
caatatgtaagcaatctgtacaatacaaacagatgtgagagtttgcactcgacaattttcaactatgccc
caaaattcacatgttggaaaaggaatttttccggcctatgccattcagcaacccattctcgaacacttgg
aaaaggcagagccacggttattttggcacgcgctttcggtattaaagtaaaacagcattcacagatgtac
aggaatttacaacatattgacaaacggagcacttatcattcacgaagaaaggaatcctttcaatataagc
aaaccagatacttttttgcgaaaacgggtttcaaaccttccactactgaaagactcactgtattcatctca
gccatcatcttcagctggggaccatagttatggaataagttattagatctgtttgcaatgtgcagattga
caaagaacaatcttaactagaaacaaaaacaagaaatcagagatttaaaacaatgtcaaccgtgatcaac
aaaccagttacctttcacagaatacacttcgggtaaccatgatgaccagtgcattctcttgtcgccaagc

acttttttcgagaattattcaccgattggggctcatacgacaaacgttccaccttaatgcactttttgtt
gtgacatatatgtgaaacattcattgtatctatcaaaatcattgtacccaagaagtgcaaaaagaacgca
agccggtgtgcgaggaaatgtatgcgcttgccggccactgtggctcgcagcaccgcatatccatagcggt
catgctcacccacccataaccggcaagcacctgctgcacctgcacctccagacttttgttctacaaggtt
ttttagccgttgaactgtgtgcaaagaagggacacttgcataaaatgcactggccataagctatgactga
aaatgcaaacaagtatggatattaatgctgtttttgtccatttttcccccggaaaaaccttacatttcac
tattggactttcgctttagcacaataatctttgtaagcagttgtaaattggttaatatggtcaataaaac
ttgtaaagatccattaatgtgcattaataacaaattgccaataccagacattgagttacctccctttgtt
tgagcccttagtatgctggtgttagtgttttcccattggaaatattgtcgaaaataaaatgctgacatgt
tcagcaacatttttaaatgcaattaaatgtttttgacaggacttgaaaaattgacactttaccttaa
>KolobokP-5N1_DrPo-I
ttaagaaacaccatactatggtagaatcaggaaagttgactattttcatgaggtttactcatatatgagt
gtgtacaataaattttaaaatcctgggacgactcgtgtatttttttatgccttgttttctacacatgataa
tttggtcattttcagtactgtttcccactatctgcagactgtactactgagccaggaattgcattatgtt
tgcaagcaaatttcaacaaacactgcatttttgtacccaatcatttaaaaaatgatgtccaaatgataat
taacagccaaacacttctgatcaatgcattggtgctgaaaaatcctcaaaaagcatagcaaaaaggttaa
ttttttgaaagttttacatattttcatttaaaaaagcccatttaattacatgatttacctttttcttatg
caaacctaccactgaattttcttctacaaacaagcacagtactttgagtaatgtaaacatttgattaaac
atggctttggctgccacaatggactaaaatgacaatatctcccaatgaaagggaggtaatccaaaagtta
tagagatgaccactggccaatcttaaaaattaacaaaaatagatacagcacagggaaaaacaagaacctt
ggcacttatcagatgggattaaacaaacctttgcacatacaaagaaagtattttctggattaaactggat
taaaaatgttgggcacacaggacaaaacaaactacataaaaatgcttgccagtggtgttcgaacacgtgt
gcgaatggaacttcaaaatttaaagacaataaaatcaaacgatagcaattactttgtaacaagaagttca
caggctcttgctaacataattccgtgtttcagcaacgatcattcaaagtgctcaaaagcgtctaccgtgt
gccagcaccacatgtcaagcattcgaaagtacagcgtaaaacatttgccatacggacagcaccttcatct
ttcgaatacggatagcagccgtctcaaagacactatatcaagtacttttgataccgaaacacttcaatat
gtaagcaatctgtacaatgcaaacagatgtgagagtttgcactcgacaattttcaactatgccccaaaat
tcacatgttggaaaaggaatttttccggcctatgccattcagcaacccattctcgaacgcwtggaaaagg
cagagccacgtttattttggctcgcactgtcggtattaaagtaaaacagcattcacagatgtacaggaat
ttacaacatattgacaaacggagcacttatcattcacgaagaaaggaatcctttcaatataagcaaacca
gatactttttgcgaaaacgggtttcaaaccttccactactgaaagactcactgtattcatctcagccatc
atcttcagctggggaccatagttatggaataagttattagatctgtttgcaatgtgcagattgacaaaga
acaatcttmactagaaacaaaaacaagaaatcagagatttaaaacaatgtaaacagtgatcaacaaacca
gttacctttcacataatacacttcgggtaaccatgatgaccagtgcattctcttgtcgccaagcactttt
ttcgagaatwattcaccgattggggctcatacgacaaatgttccaccttaatgcacgttttgttgcgaca
tatatgtgaaacattcattgtgtctgtcawaatcattgtacccaagaagtgcaaaaagaacgcaagccgg
tgtgcgaggaaatgtatgcgcttgccggccactgtggctcgcagcaccccatatccatagcggtcatgct
cacccacccataaccgacaagcacctgctgcacctgcacctccagacttwtgttctacaaggtttttaa
ccgttgaactgtgtgaaaagaagggacacttgcataaaatgcactggccataagctatgactgaaaatgc
aaacaagtatggatattaatgctgtttttgtccatttttccccctggaaaaaccttacatttcactattg
gactctcgctttagcacaataatctttgtaagcagttgtaaattggtaaatatggtcaataaaacttgta
aagatccattaatgtgcataaataaaaaattgccaataccagacattgagttacctccctttgtttgagc
ccttagtatgctggtgtttagtgttttccgattggaaatattgtcgaaaataaaatgctgacatgttcag
caacatttttaaatgcaattaaatgtttttgacaggacttgaaaaattgacactttaccttaa
>KolobokP-6_DrPo-I
tataagagaaatttattttgactaaaatgtgtttttcttgcatattaataccttcccatgcatattgcac
caatatattaagtttggctggtttatctgtccatttggccatttcactccatattttcaaacgcaggtga
ttttggcctgcggaaggccattttgagcctcttaaagcttaactgtacctttaagatttaaagctggtgc
cttcaatatctgggacaaaataccaaaacaaaccaaataaacatgcacatggggcttatgtggggtgagg
aaagaatgcctttgttagttattttcaatttaagccatatttttttatggctttttgtgtatttttttwtat
tttttaccccaaaaatgtcaattccatcacacacagaaatcaaatttcatccaagaccattatacaatt
agcaaaatgtggagatcaaagatactttgaagtgtagaaatcaatcagctttcattaattgcttgtttca
caccaataaagtgtgaaatcttgaaagcacattgtttgttgcagccagcccctttaattacaggccccaa
tggtacattctagtatataatgtcatgggtgcctttaaactgcagcaggtgatcaatttgaattgcctgc
tcacatttaaaggttaaagggattgtaaaatagcatattttgggtgcaaatcaagttctttgactctttt
ttaagctaacaacctggtcaaaatggggtttcttaccaagcgtaagaaaaagttgctcaatacagccttt
aagcccggtggacttagtctgaataagggtagaaaatatcattatgaaagaaggtcaaactccaacacaa
cctatgtaaggttagaaaggtcatcctttgaagcaagggtagcttacaatgacaatattctcacttttcg
ggatgtagacggctctaaaaccccagctaagcccttgcgtccgcgagtgaacaggactcgctacgtagat
atgttacgggttcccgctccatcaaaagtccatccagacttactaacgaacaaggtctatgtccccgccc
tactccagtctatgatgtcctctgaaaatagaaagcataggttaagaagtgctaaatgtaagggtgactt
ggtaattgatggcagtgcttccataaagtggggcttaggctggaaggaaaggttacattgtacaagatgc
agatatgtaggtgagcattataagctgtacaatgaggtcccatcaagtacaaggggcagaaaggcagctc
agattaatgtagggtcccaaataggtgttgccagcacttctattgggaacacaggtttccgcagaattct
taactctacaaatattatcgccccgtctccacaggccatgcaaaaacaagcaaacaaggttaatactgcc
ctcaagtcccttaatgagagaagtatgtgccagataagaaaggaattggtggctgaaaatgccaaaatag
gtcaaaaagatccctstttggttaatgtggaaggggacacttgctacaataacccmattttttaagtctga
cgcgacaccctttcaagctggaaccatagccgtaagcacaatgtgcgaaaacaatactaaaaagaagcaa
attgtaggggttctggtggcaaataaactctgtaggtcttcatcaatgctaagaaataaaggtcaatctg
ttcaatgccccaatcataggggcattgttctgcaaatgtttctgagcgggaaccgataggtgatgaagg
aaaatacaataaggtgttgggagacctgatctcacaggacatcaaaatttcaaatttcacttgcgatggg
gacagtagggcatttcaaggtgtgcgtaactcacacggtcacaatgttggtcaccttagggatctaaggc
atttaggcaactcgctgaagcgcgagctaaacaaagcacctttctcgaagggcatgcttaagggtcattc
caagactaatatcaggaatcgattcgctctttcggtcaaggctaggtgtatggctgagcttaaagctgcc
cacaataaataccagggtgacatcaaagttctaaagaaggtcatggtagatgtaatctcaaccataattc
tttgcttcaatggttactgcggtactagttgtgcaaagtacagttatgtctgtgctggtaccaataggca
agcgaagaacttcatgccaaacaatgttaaggttagaatggttgacagtgaccaacaagttcttaaaaaa

tgtcttgaaatggttttgggtccagctgctcttgacgcaaccaaactgttaaccacaactcaaaaatgcg
aagcagcaaacaggtcataccaagccgtaaatcctaagtctgtgacgttctcccgcaactgtgtaggtcg
cattcatggacaggttcacaaattaaacaatagctatgctgactctgtcattgcaaaaaccatggaactt
catgcaaacctcacgcagggctcaaaggttataaagcagttagcgtacgaggaccgcaatgaacttaagc
gaaagaggttatccgctaccatcaaagcacgagctttacgtgccaggacgcggaactatcgttacaagtt
acatgaagaattgcattatggtaagggcataagtgaccccaaacctgattttgacagtctcccacacctt
aaacatcataaatatgcttagttaattgtgtgattataataatacagcttgactactaccagctctggca
ctccttggcaaaaggtctctatgcaatggctcccgtctcagcctcaggcttccttccagctaaaaaaagc
aaaaataccttagaaacatagatataaatgaagaaaacaagcatttatgtactttaagtgtatttctatg
aataaatggatgccaggaaacttgtggtaatcatataagtaccaaatatgcaggcaggctcagttccatg
gccgttacaatgtccgaactgtaaacatgtcctccgttccatgttggtggaatggggctctatgtttaaa
tgttcaggcttaacacacaagctattgttgcacaagtggctcacagagtccctggcatttagtgttgact
tcatgtagcacattaacgctacacggtgaaccaagtatgcttttgatttttcatcagaaggtaatttagc
cctgattcggccataattgggtggggaactgcttgtaatgccaccattccatatcagacattcagaatta
aggcatttaatacacttatctgtgatttttttgccaagaagtgcccaaaaatccactttatcagccattt
tttcaacttgcagacgataatttattttaacccaaagttagttta
>KolobokP-7_DrPo-I
cttaagatagcagtcaaaagttttaaacccatctaaatttgaattatttgtatacttcagtcaattctgg
atgaaactgggtggttttctttgtgaaataactacaatgtacaattgcatactatttttagtgagcatat
cagcggcggcattttcacgcaactttttggtcatttttkkggttttctacccaagtctaaaaatcactca
gtttgcacagatatgaaatgtaggtataatcatattgccacaaatgactcctaatgagtktcatctttsa
tcctgagtaaaaaattgttcacaacactggtcaaagttaaaaaataaaatgaattttgatgatttttttcc
ataaaatcagtgtgttttactcacttttcatcaaagttgatgcacatttaggggtttaaatggtttgaaa
ggcactaaatgttgtataaatagttgatttaagtcaacttggtattacttgcataaacaactaagcctat
ggcacacagaaacagagactgcagctctaaagcagatgtgcktacaatgcaagggaggtaattcctatag
ccatatttgaccatgaagttccaagtcttaatacactcacatttttttggtctaaatatttgtcttatggt
aatatgatgccattgaagcaagattagagccccaaaacaatgacctggtgatattttgcagataaaacct
tcatttgtgggagtctttataggccagggtatcatttttgtgaaagttgctattcagtcaacatctattt
ttctactttggacatacatttgtacttagtgtgaaaatgccttttaattacagaaaaagcgcggggaata
ttagcacacgcaggagcagaggcaagaaagccacccagtttaagctgggacacaagccaaaatggactac
atccactgaaatacaccaacctggagtttccagtgcaagttccgaggcgccgaaagcttctaaacgactg
gtacggctgacagacaaggaagccagggacagtcaattcgtcagaaccacagcagggttagcacaggcag
acactctggcatacactctcagacctaaacctgaaaaggaagaaaaaacagattgtgagtgtgaccgcaa
tgaaaatttcattgtgaataatgagaaaatgctggcacttattaaagatgtacatgcaagtggttgtaag
aagccaaacatgaaaatgaaagttacacgtgctggtctgtgtgtctacatctgtgtaaaatgtagttact
gtggatttaattcgcaaacattgccaatgtcggacacaatccagaccgcaagaggccccccctgcaggatt
tttgaactatttagtagcaatgccagtgctaaaatctaaaattggaatggatgatgtagctactgtatta
acatggcttaatattaaggcgccatgcaagcaaacatttcaaaaaaagtttaatgacttgggacaagcac
aaacagaactgagcgacaaacaattggaactaaatcaggactatgtcgcaaatgtcatgaaaaaagccgg
cttcgaacctgmagttgacatccaatttgacgtcgcctacacatgtcgaccccagggggggatgcgaaaag
tctacacaaagctttggggccctggtagaacacaatacaggtcaaaaactcccacttgccatagccatgg
ccaacaagcattgcagaaaaaggtactgcaggcatgataactgctcaaaaacctttgcagctgaggcctc
cattgcaagcagtgagcgagtattacttcacaggtcattggataaagtgaaagatggtcctttggcagta
aggtcaataaccaccgactctggtacgcaatcggcaaaggctatacgggattactatgagcttaaaaagc
aaacaaatcacttaaaaaagcagacggccacccactacaagtgctttgttcacaagctgagagctctcga
aagacatgtgcgtgccgcaaagtcagacatcaaatccattccaaaaaagtacaacaaagacgaatacatg
cgtaagctggccagctgcctccgctgccgagtgcgcattgaactggaaagactgcgaaaacagagctgca
gtgatgagctttttcttcgatcggccaaatttgctatagaaaacatcatgcattgcttctccggagatca
caagatgtgcaaggaacgctcacgagtgtgcacctatcgagtgaccagctcgtacaaacatttgccctat
ggagagccactagcactccaggagagtgacaagaaaattgttttaggaaacattaataaaacatttgatg
ccacggggttgaaagaagtggcaaagctctttaacacaaatgcttgtgagagcttaaatgcatctgtgtt
tcattatgcscccaaaacttcattctatgctagaaactttgcagctctgtgtcattctgcagttcataca
aggtctttggggcccagcaaatcatcaatgaaggtggcagaaaaggtgacagggaaaaaaatcagtttac
atagcatgatatacaaccaactgaaggccaaggatcgcagacgggaatatgacagccgcaggaaggcctc
agtgcggtacaaaatagtcagattctaccttagaaagcggcacgcaaacagagccctgtaccgggacggt
ctatatgcctctgaaagtatgccatctacaagtgctgctgaccatagctatggacttaaagtgtaaaaca
gaaaamcttgtgatgtacagtacatgcaaactgcgaacggcaacataacaataacctaacttgtatattt
accgcagaccacagacagcattccctgaagcccctgtgacccgagcattgaccatttgtaaggcacactt
tgcgggaattattaacagttactggctcatacgataaatgctctatattaatgcaaagcttattgtggca
gaggtgtgagatatggaaattgcgctgcagtgcactgtgtccgggatcacacagatagtacttgagacgg
tggacttttagcttgatgcgcttgcccgatacaacaacacggtgcacaccatagccgtacctatctgttt
caccagtccacaggcaacacagaccaaccaactcactcttttcacccagaacagcttccagtttcactac
aagctgatccgggggaaaactcgaaaaaaaagcagcggccatgagtcacagggtcttaagcacagatgaa
tctgaagaaaagtgaagtaaatactcttaattttgtagcatcacattctattatgcctatttagaagatt
tcactctctgaaaagtaaatttaaatcacctatgataagtatagtggttaaactgtcatgcaaagtaggg
ttttattcaaaaatcaggcttacctcccccctactgaaatgttaatatctctgctcaaattggtcagaatt
gaatgggaaaactgttttcaggtgttgatgattatattttatcaaaaaatgtcacatttgactatgckgc
sttgggtgtgaaatttggcatttttttaccttaa
>KolobokP-8_DrPo-I
ttaaggacaccaattattctttttcattactgcaaaagcatctttttctagtattttagactattcctga
cagaaacagactgttttcgcttaactgtattgcaaaaaagactgtctttcttcatatcaaacacttaacc
caaagtgcacactttactcctatttaactgagaattcatgcattcagagttctgacagagacactcttta
aatgtcttctggaaccttttggacatctttccaacctttttaaacactattctcatgcttatatacatct
tatatgcacagcagatacctttttccaacatgtctggacctaatttttaagaaaaatagttgatttttcag
cccccggagtgccaatttctttgatttttgtctattttgctccctttcctacattaatacaactgtttta
gcatacataagacactaagaacatgagaaacactcttataacatgcaacagtaagaataacctgataaaa
taataacaaactggcaaaaacatgcactcaatactggtcaaagggaggtaattgaaattgtcatcttagg

gtttgcatgcaagtgcatcactaacaaacccataccaaccaaaataatgctttggctggattttatatgt
gaaacctgatactgcagctgataaatccacatatttgagggtctaattggtgatttcatctccaaaacta
acagaatggccagggaaaacacagaatctctcacttttagtgttgacttagtcaaagcaagaggtttatt
caacaaatataacggattaatagtgccaagatgtataaaagagctgacaaaattagctcaaataattcct
acaaaaaaaggaaaacacagtttaaaaagggattcggtgtgatgaaatcagatgatgcgaatgcaaaatc
tgtgcatttatcagaaccacttccttcaacatctcagtgcagtgatacagttgatgcagttgattcagta
aaaagtcgcctttagaatcaggaggctgcagatttgaaatacatgtcttgtaccgaatacctgccgtata
cactccggccaaaggctcaggaaagccctgcgtgtgaaaatgacgttgtattggaaaacgtgattgtcaa
cgttggaaaagtagcccaacttgtaagccgtgttcacgaggcaagctgccaagacccgagggtcaaagtc
gatgttgttaacagaatggggttatgtgtttcctttgctgtcaaatgtaattcctgcaatttcagcctag
aacccattcagatgtctgacacagtggagaaggctaaaggaccaccagctggtgcacttaatgaaatggc
agttatgcctgcagtaaagacaaaaatgggcctaacagacataacaacagtcctcacatgcctcaatata
aaagctcccagtaagacagccatgcaaacaaaatttaatgctctgtgtgacaaaatggcagcagtcaatg
agaggcagttgataaaaaatcaagaatacctgcggaaagttacgtcacttcttcctgccatatcgggaat
tgaaacgcaatatgacgtgtcatattcaagtcggccgcaaggtggtagtgagaaggctcaacagagcttt
gcagacattattgagcatacgacaacgagaaaactccctattgcagctgctgtggccaacaaacactgca
gaagaaaggactgtgaccatagacaagacaaatgccgaaaaacatacagctccgaaaagtcaatagccgc
cagtgagcgctctcttctcaatgaaacattaaacaatgtgagaacagcaggcctcattactctacgctct
gtcaccacagatgccagcacgcagcttgctaaagctcttcgggaatacaaagctgaaaataacttgaatt
tttcacattacaaatgcttcatacatagagggaggacacttgagaagcacataagagcacttcaactgtg
cagcatacctaaaacatgtgacaggcaagcctatatgcagaaactggccagttgtcttagaggtagagtc
cgcattgagctcaagaatgcccgagctctgataaaaggagacgggtccttcatacgcgctggagctgcag
cagtgcgcgtcataactaaatgcttgtctggtgatcaccgccattgtaaaggaacttcatttgtatgcag
aggtcatctgctcacctacagtacaaattgtttgccatatggaatgcatttgcaacttaataataaagac
cagagcctgattgagaaagagttggagaaaatgtttaatgaagacggcctaaaagacatgtgcaagcttt
tcaatactaatatggttgaaagcttgcactcaacagtttaccgacttgcaccgaagagcttatgttacag
ccgcaacttttctgctctatgtcattctgctgttcactctacttccgtgggaccgagcttgtcaacctta
cagttggcgcaggcggctgggctaaacgtaaaagaaaattcccagatgtataagcagctgcgattaaagg
accggcaaagaaaatatgactcaaaaagacgagcttcagcggactataaacaaagaagatactatttacg
aaaaaagaagtcaaaccgttcgttattctcaaattctttgtacagcattggccaagaagcttctcctatg
gctcaagaacacaactacggccttacatcctagttatctcaatccacacaccaccaccaccaccaccacc
accaccaccaccaacaacaacaacaacaactaaaaacctaataatatcagcatttcttttttttccacga
ctgaggtaaaatttgcacctcttaaagccacgatgaccgatacatcatctgtttagcagacatatcttcc
tcgaatttttttattattgttcgtttcatatgacaagtgatccacattgatgcacagcttcatgaatggg
aaacatacatgtcttttgctacagagttgcattggactcttatgaaggaaataagtcaaataacggacaa
gtacttcaaccttttaccatacatagtgattcagtgtacaccgtagccatatgtatcggtcggaaccggt
ccaagatacgcattcaccgatgcagaccacagacttgtcggttggggcagtacgcagactttcggctgtt
tccagacttaatggaggacagaagaatgtactggccacttaatggtatgtagatttactgctgaagtatc
agaaatgcatgcaaacaaaggatgaactagcataaatctcaaatatctttgcttagaattcaacaattca
gtctctcagacagtcaaatcactgttagagctttttttcgaattcaagatgaaaatagcctttttttaatat
ttgaattacctcccttgtttagatcagttatttacatccaaattatgaaaactaagctcatcaaagccca
ttttctgactaaacattggtgtgaaagcttaaaaaaatacatttcacaggcaggctgtgtaaaaaaaaaa
atgtcatttaccacttaa
>KolobokP-8N1_DrPo-I
ttaaggacaccaattattctttttcattactgcaaaagcagctttttcaagtattgtagactattcctga
caaaaacacactgttttcactcaactgtactgcaaaatagactgtctttcttcatatcaaacacttaacc
caaagtgcacactttactcctatttcacggagaattcatgcattcagagttctgagtgagacactcttta
aatctctcctggaaccttttagacacctttcaaacatgtttcctttwtataaacactctactcatgctta
tatacatcttacttgcacagcagatacctttttccaacatgtctggacctaatttttgagaaaaatagttg
atttttcagcccgcgaagtgccaatttccttgacttttgtctattttgctcctttctcctacattaatac
aactgttttagcatacataagacactaagaacatgagaaasactcaaataacatgaaacagtaagaataa
cctgataaaataataacaaactcgatgtcggacagccatatcactgttacgagctttttttctgattccag
ctgaaacaagatgtgtttgtgaaacactatgtacacccaccacatatatatgacctttgtacttgacctt
tgaccttgacggatgaccttgacctttcaccactcaaaatgtgcagctccatgaggtacacatgcatgcc
aaatatcaagttgctatcttcaatattgcaaaatttgacctttgaccttgaactttgaccttgaaggatg
actttgaccttgaccttttaccactcaaaatgtgcagctccatgagatacacatgcatgccaaatatcaa
gttgctatctttaatattgcataagttatggttaatgtaaaggtttgacgctaacaaacaaaccaacgaa
caaaccaacagacagggcaaacacaatatgtcccccagtatagtctggggaacataaacagccattttttc
aatatttgaatcatctcccttatttagatcagttatttacatccaaattatgaaaactaagctcatccaa
gcccatttttttgactggacggtggtgaaaaatctttaaaaaaatacatttcacaggccgactgtgtaaatt
gtttttgtcattwaccaattaaggacaccaagtactctttttcataactgttaaagcatctatttctagt
attttagacaattcctaataaaaacagactgttttcaatcaactgttttgcaaaaaaaactgtctttctt
cacgccaaacacttaacccaaagtgacactttactcctatttcactggtaattcatgctttcagagttcc
aagaaagacacacwttaaatgtctcctggaaccttttggaaaccatttaaacactctgctcatgcttata
tacatcttacttgcaccgccgatacctttcccaacatgtctggacctaatttttgagaaaaatagttgat
ttttcagccttcggggtgctaatttccttgactttttgtctatttggctcctttttcctacattaatacaa
ctgtttaagtatacataagacaataagaacatgagaaacactcaaataacatgttacagtaagagtaacg
tgataaaataataacaaactggatgtcggacagcgaaatcactgtaagagctttgttcggattccagctg
aaaatagccatttttttaatatttgaattacctccctttttcagatcagttatttacatccaaattatgaa
aactaagctcatcaaagcccattttcacactgcacgttmgtgcaaaagcttaaaaaaatacatttcacag
gcaggctgtgtaaacaaaaatttgtcatttaccacttaa
>KolobokP-1_PaGe-I
tataaggattttttttatatataaattggttttaacctcctgtatggaaaacaataccaatttccagca
atattgccaacttacaggcatttaacactgaaaatttggtcaaaatatcattttctagatggtcatagtg
ccagtcggcagctgtcggccaaaactgtattttaggcattttcagtgagttaaccattttaattcatgga
catcaaaacccgtcatttttttacattataatagtctcatgacattttcaacatatgtgttgactaaagg
aattcaaaacaaaaaaatcacaaaaactgtgacttcaatttctgtaataaaatatcaattaatgctgttt

aattaaggaggtgttwcttatttaacatttcagaccataacagtgtaaaaaaaaagttatttcaccaatt
tttaccaatcatcaggtcaatggtgcattactkaaaatggtgtgaaaaacagtctccagaaggcagacca
aactttagcaaacttgaaccaaaatttcatatcataaggtgttaaactagggtttgaataggaatttaaa
cacattaaagtaggttataatcatcaaacataaattttgcaactttttttacatttttatgcttttat
gggtgacaaaatgcgaaaaaagaaattctctcgagagaaacactgtagaaatataggatttaagaaaggt
gttcaaagtggtcaaaaaggtaaaatttattcattctgtaatgaaagtaatgataagaaatatgtaagac
tcttgaaagagtcatttgatgaaagagttgtggtaaataagggaaagcttacctttagagatatcgatgg
ttccgaaaccaatgtcgctccgctacgtcccgtggttaagcgtaacacactgctcgatgcatacgccagt
gctgacaaaactgaggttcaccccgactcgttcactaacaaactatatagtcaggcacgtgtacaggcaa
tgttcaacactgaaataaaaaaaaacattgtttgtgtggtaatgcatgtgaaggtgaattaaatattgat
gccacggcatcaaacaaatggggtctagcttggacagagcgactaaaatgttcaaaatgcccctacataa
gtaaatattataaactgtatgatgagataaaaacaaacaaaagaggcaggaaagctgcacaggtaaatgt
agatttccaaatgggtctaatgactactaccatcagtaatactggggcctccagaatacttgtaaactca
aatatcataccaccttcaagaaatgctatgcaaaaacttgcaaacaaggttggtaaggatgtacaaactc
taaaccaaattaacatgactgaaataagggaaaaaataaaagctgacaacaaactttgtggtaataaaaa
tgaaaatgaggtaatttgtgaaggtgacagttgttacaataacccgttgttcaattctgagagtacgcct
tttcaggcaggcacaatagtcactgctaccatgtgtgaaaataacacaagaaacaaacaaataataggtg
cttatgttggttgtaaaatatgtccagtttcaacaaaattaaaaaacaatggtagcaaagttcagtgtcc
taatcatccgggtcactgtacagcaaatttgaatgaaaccgaatcaatcggaaatgaagcaaaatggaat
gcattggtcaccaatgaagtgaataaacaacttaaaattacacattacactggggatggtgactcaaaag
ctcacagtggtgttgataaagaacagggtactcatgtaacacacttaaaaaagatatcagacacctgagt
aactcccttaaaagggaaataaacaaagcaccgtttagtaaaaatatgtttagcggtgcaaagaaaataa
atttaaaaaacagatttgctttgtcagtgaaagctagatgtttggcagagcttaagaaagctcatgtggt
gtttgacagcaatatasaactaattaaaagtaaaatgccagctgcaataggagctatagtgctatgtttt
aaaggttattgtggcgactmttgtgctaaacacagctttgtgtgtcctggtgatttcagacgcactaaaa
ttatttaccatcaaacatcaaactaaaaatgactggcactgatgaacaaatacttaataagtgtataatg
atgtttctggggcctgacagtataatgaaaacccagcttttaacttctactaaaaaaactgaggcagtaa
atagagcatatcaagcatgcttgccaaaagctgttacatttgcacgaaattgtcatggtagaataaatgg
ccaaattctacgcataaatcatggctatgcagagtcagtattaattaagtgccaggcacttggcgtaaaa
tttcagcatggttcttctgtaattaaacacttaattcaaactgacctacgagaaatacggagtaaaagtc
agaggtatatggttcgagccaagacatcgcgatatgctactcgcttgcgacgatacaaaatgcactcaga
gttgcactattcaaagggtatgactgatcgaaaaccaaacttctcaaacttacaacaactcaatgatcac
cactatgcataaaatgatagagctctataaaatataaacaaaaataagcttaccataatatatactgtgg
ctgaatcagtctgttgtggtctgggctgtctcaaacactgaactgcgagaagttacacaaacactgaaaa
ataaaaaacaaaaactaacagttaactgaaggcataaaactgtaaatatgtacataatgatttgagtttc
tatttttttgtgttattttaattatgttttaagaactaacaacataaaatggtacataaaagctataaaa
tgcagtcaggaaactgaaaatttgacaaactgtgtgatgagcactggtcgccacactgctggcgttcatt
atttatgcattgtggctctaagcttaaatgtaatggattaacacacaaactgaaatgacataaatgtgat
acatgtagaccctctggtagctcttgaaaaccattatttaacatatatataactctatgtacataaaagt
gctttgaatgtggttgattaggaaatttwacacgaattttcccatattttgccaccccccctcccacatgt
actccctaaccacttaatacaccctttgccccccatgatttatgctattttcatgtattctttgtttagtt
gtattaataaaatgatcctgatccattgttgttgctgaaaatcttgcagacgacagttttaaaaatgtaa
acaattactggtaatctcccttata
>KolobokP-2_PaGe-I
tatatgaaactgttccattaattctgacttttaaaaacatatggctgtgctctcctaccttagggttagt
ggtaagagtagattccatggctgtatctcaaaaactgtccttcacattcatgtgccagttgaacctatga
tattgcgtcaaattcaggggaaaatccaaagatctggcaatccgcacaaactgaaaacagtaatacaact
attttaggctagataacataccactacctgcatatatttatccgtatacatttactctgtctgaaaatta
taaaattaaggaatgccaaatgaagatataaaaaaaaaataatttttagatgactgtatagaaattggcg
ccatttttcagagatttctaaagaaaacaaattcccagaagaaaatctggatttgaaaattgtcaaatta
atatgcacaaaaatttcccatatattactaatgtaaccatggtaaaacatcttgaaaaaaagataagatt
tttttttacagcctttcaacttttgctggtattttgacaaattgaccaaaatggccaaattctggaaca
attcagaaattctaaaaatttaataactttttttattcatgattcaaatttgatgaaacttgctaggtact
aagtcaatagtatttgtaatctgaaagtgaaactagtttctctgtcatacaagtaagagaaaatttataa
aaaaaattagatgtcggctaccgaccaaaacattgaaattctgactaaatctctacatttattttacata
attatttttttactctaaataaaatgtttattttactatttatttattgttctaatgttattaaatcatt
ttgaataaaataagttcaacagaaagaagcaggcttccattccaggcaagttgcatattaaacatcaaag
aaggcatcctgctgtgctaaacaacacaggtctgaaaacagaagccaaaacaggtaaccagattgccaaa
acaggtaaccagatgctcatgtccactcctcccatgtttatattctgctatataaggcactgtagaaact
gggatattactattccaccatgaaaggtgaaagaaagaggaaacatgctatgttcaagaaaaactgcaca
ccttataataagggcctcgtcagattatcatcagcatcatcatccaacagtagcgacaacatggcagctg
aaaggtcgataaggcggtctactacttcactttcactctgcagtcagacgtccacatctaccagtcaaga
agctgatgtgctagtcttgcgtcacaggcctgaaacagtcaacaaaacacaggacatcaatcgagaggca
gaacatgatggcaatcgaatcatagacaatgacaaaatgatggaggcattcaacgactgtactcagagtc
atcaggaatctgcaacctgtacaaaacctcattttgctatccacagcaatgaaaagcagggcctggcttg
gaaatacacattcagatgtgtaaattgtacctacaaatcaaagaaatacaagttcttcaaggaaatagtg
acccgcctgcctggtccaaatgccgctgcgataaatgtatcattccaagctggacttcaagacacaccaa
ttggcgtcacacggtctcgtcttcttattgcgtcaatggacattccgccaccaactaagaatgcaatgca
aaagacagctaataaggtggcacgagaaacagtcaaagtgacaaagttagatatggaggagaagttacaa
ctgcttcaaacaactctgtctgatctcggtctaagcggagaaatccacggttcgtttgacggtcgataca
actcgcttacaattgtaagctgtacgaaacctggccacaactcatcccaggcaatcggtgtgtttgccga
gtctatgacggaaaatcatttcatactggattatgaaatcgaaaacaaactgtgctatgttggtgcgcat
ttacgatcaaagggctatgatgtgaattgcccaggacacgcagactgcacagcgaccttgccaaatgtag
ctccacattcagagtatgagatggcaaaatcaatagctaccagacttgggcaaaaaggagtgaaagtaag
catactaacaacagaccaggacgcccaagctcaaaaaggttttcaagaaatttatgaaaccatgtttcca
ggtcacaaaatcctgcagcaagctgatccagcacacataggaaggtcacaagtgcgaaaatgtatttctg
caacatttagcaaagacatgtttgttggctgcagcacacgagaagcatctaaaaaagctcagacgatatt

tgcactggatgtaaaatcaagatgcagtttgattgtgaagaaagtgctagagttatactgtggtgactta
ccttccatgatcaaagctgtgaagaaagcgttgaaatccacaatacagtgctatgatggtgactgcacaa
cgtgtcgaagaaactctttcgtatgtgggggaggccaaaagtcgtgctggttcgaacgctctgcattctt
ggctccacacaagatcaccacattgagaatggctgaaagtgataaagaactgttagaagatatactcaaa
atacgcttatgtgaaaaagcaatagaaagcacacagtttaattataacacacaatcagtggagtcattta
atgctacactatcaaaaagtgctcctaagaatgtgaacttctcccgtaactatggtggaagatgtgcatc
agctgttctccggcacaataatggacaagctactgctttaattaaaaaattggagtacatgggtatttat
ctgtcaaagagaacaggacaggcagtttcaaaaattggtgcggatatgagaagtcatcgaaagtacagga
aagacaagcagtacaacagacgtataattttgcgtcgtggtgaaatggagtctcgccatcggtggtatag
acgtctcaaaaacaatagacaatctgatacctaaaaaaaaggagtgaccataactaccagacacctgaat
aaaatgctggaactgtgataaatagagacagaaaataataaattctaatgataaaatgtatatagaacag
aacagaacagaatagaatatgtttatatctatatgtgagtgagtgagtgagtgagtgagtgagtgagtga
gtgagtgagtgagtgagtgagtgtgtgtgtgaccattgtattatattgttttgataattatattgtgcaa
tgcatacaagaaaacatgaacaaattaaaaattaaactttacctacctgtgttcttctcattaaaccttc
cagaaacagtagctaaatakacttcacctgaattaaactaaatcacactaagcatgttccactatgtgga
cactaacctacactacacagctataccatgctaattgatatgtaaaaataattgcaacaaaccattaaaa
cttgagctccacaagacaggccttgaactgcccatgtccacggcacgatccagcagacacacacgactcc
ctctcattgtttgttcctagtggctcatgggaaaggtggtcctgatttatgcacaatttattgtgacata
aatggctgacacaccaaccctttggtcccagttcatgctgttgatcttaagcacatggcaagctgtgtac
taaatatggactgctacgcctgcaccaattggcccgagccatcgacactgtccatatccattgtgggttt
gaccactagtccaaattacacacacaccattctcacctaaaacactgcgcttctccaggttcgttgtaat
gtactgtttgtaagctgcatccatgttgctgaggctgacaaatagtctctaaccaaaaaatagcctcaaa
actgcattcatggcccgagaaaacacataaacttacctcttatttgcaactgcatcatttaataatcgtt
attaactgccaataaacacatgtatatgtgacttatttaaaaataataattatatcaacctacctgcaga
aaatattaccaaaaattccaatgcaccgttcacaacagccatctccataca
>KolobokP-1_PoSt-I
tgaaagggtcagaaaatttttcttatatggacaaaacatccttttgtaatcaaagtaatgtctcttaaat
actgctgcaaagttttaagggaattcattcaggaataggacagctacaggcttttatctgacaccactgt
aacacaagcaaaagccaattccaaatggccgaatgtcaaaaatgtactaaacttaaagttttactgtgat
tttactaatcaagataaatacttcaaataaactgtaattaccttttaatcctaacaccaatctggaaagt
actaaaaattaaacttgacagttaaacttttctagaaaatttaattttaaaaaattatacatatttctat
ataaaaatttaaatttaacttcaattttaatgaaaatttaaaatcggatgttgacaaattgtattctgtg
acaaagtaactccattcctttatcatgaacatcaatggtttttcttgtctgctattatgcaattcaacca
ggacctgttgatcaaaggtaacatcttaatacacaagatattcactgttctggccaagtacatgacatca
tttccacccatgaaggggtggatccctcatatgttaggcttccaataggattgaagagttagtgtagctc
tctaaaactaagctagcatgcattgtactaggtatataagtgacagagagatgatacatcttgtattaaa
tttccatgcagttattgtcacagaaaccatgaaaggctataagaaaaggaagacaactttttttaaacct
ggatatattccacataataaaggagtcagatatctgactgatgatactctaccaacttccagtgctgcat
ctgttccagtgaccagaagaatggatgcagtcaagttctctctcatggcaaagacaaagccgacaaatgg
ggatctccagtcagttcaggcactagactgtaatggcacacctgggtctgcaaacattctcagaccatca
tacaaagcaaaactggacctgaaagaaacagaattgaatgaacatgatgggacacgtatcatctgcatga
aaaaaaatgttgagatgtggaatgacatcattcatcgccatggtaatcaagttaacatgtgtggcagacc
agagtttcaaattgcaaatgaaagacaatggggacttgcttggaagcatactatgatgtgtaaaaactgc
agttttaagtcaccagagtacaaattgtatgatgagataaaaacaaataaacctgggccaaatcctgctg
ctgtaaatatgatgtttcaagtgggactacaagatacaccaatgggcaacaccagggctagatacttgct
tgcagccactgacattccaccaccaagcagaacatctatgcaaattgcctcccacactgtaggtaaagca
actgtgcaactcaatgaaatggacatgatagacaagctgaaactggtcagacaggtgaatgtcatgcgtg
gtgttcagcatccggagcaaataaatgttgcttttgacgcaagatacaatgccattacatttggacatga
aaaaaaacctggtcaaagttcatcacaggctgttggaattgcctgtgaaactcttacagagaagcaatac
attgtggggactgcagtagaaaacaagctgtgttggactggagcatggctgaaggggaaaggttatgatg
tgcaatgcccaggaggtcatgttgactgcacagcaaatctgtcaccagttgcaccacattcagagcttga
aatggcaagagaaatagggaacaagtttgtgctgcaagagattctcattagacatgcaacaacagatgga
gatgcacaggctgtggcaggcttccaagctgcttgcaacattttacatccaatgtggaaagtggaaagac
agtcagaccccattcatttgggtcgtttacagttcaaacgctgtaattctgctgtatttagtgaagacat
gtttccaggaataagaacaagagaaggcaaaaagctgaaacagaaaattttcagccaagacattaaagca
cgctgcagtttgatattcaaacaactgatggaggacaatagcggtgatgttacaaaaataaaatgtcaac
taccccaagtactggaagccactctgcgctgttattcaggggactgctcaaaatgtcggcatcattcaaa
ggtctgtgggggaggaagtactaacagctggtggatgcgatcagcatacctgggccctcatcaagtgact
catcttaacatgacagaagatgataagaatattctgctagaagttctaaaaatgaaactatcctgtgatg
ctatagaaagactgcgactgaacacaaacacacagaaatgtgaggctgtgaacagatcaatcagtgtttc
attacctaaaaatgtgaactactctagaaactttgaggctagagtgcattcaacaatacacagactcaat
aacagcctgggatcatcactgaaaacaaaagtgacacacttgggagggaaactttcagcaaggactctga
gatcacttaagataatggataaagactgcacttaccataagttgtatcgaaagcgtccacaaactaggca
acgtattctagccagacgaggaagaatgatacaggcccatctgaggtacaggactcagccaggaaatagg
aagtctgactactccaaaggacagctagacaccaagggcgatcactcctatgcaaatgcttagcaggagg
caatggatactgcaaatagaaaagaatatcttaattccatgtgcaaaatgacagtactcaaagacatcct
gaaaaagacatggtacagaagggaaaactgtagtggggaaatccgtaggtaacacaatgcatgttataag
cactgtgttacttactaccacctgatgagacctatcgagaggtcgaaacagttttctctcaatatgaaga
aacaaataaagaaattacaaaataaaattataaatccgaaaaaaatgctaaagtaaaataacttgctgaa
tgaatgaatgaatgaatgaatgaatgaatgaatgaatgaatgaatgaatgaatgaatgaataaaggaagg
aagaaagaaaggaaggaatgtatttaagacagcactgtcaccagcatggtctactaaaataaagaccaca
ctctaatattcttcatccatccatccatccatccatccatccaccatccatccatccatccatmcatcca
cccacccacccatccatccatccatcatccatcatccatccatccatccatccatccatccatccatcca
tccatccatccatccatccatccatccatccatccatccatccatccatccatccatccatccaatctca
tgatacctggattatttccatgttacaatgtaaactttatcatgcagtctggaaataccccatgtccaat
acaatgcccataacttttgcagcactgtctattattgttaatggaatgaggttctaaagttaggtggtcg
acattgacgcacaaactgttgtggcataaatgtgatgcatcaagctcctttggaatattcagacttctca

attttatcattaatgccagtctgtggacatgtttttcttccaagcatttgtttctggatccctatatga
taatagaccataaagtttattggctgttacagttcctgtccatattaaacaattattattgtgtactggc
actgaatttaaagaaattttcctagagaggctttcaaaaaaatcttccattttaactttcatatggagta
ggaatacatttctgcaaataataataggtgtcatctccaattttaccatccctgtgaatttatggccaa
aaaaaaattaatttaaatttttttaaaaaaaattatttaaatttttttaatttaaaaaaaaaataaattcta
attatattttctaatttttaaagtcaatatacacaatgtgtgaaaatttcatgtgtctatcattattaat
aaaaaaattatatcaatttaaagtttagtcagtaatgccctgaca
>KolobokP-2_PoSt-I
ttaaagaaccactgaagatgtgttgtcaacttattgaaaagctagtcatattctgtagtcaatttcaaat
attttgatatattgtacatggtatttacttccaaaatgataacgacatagttaatttactacacccctct
gaagtagcttgaatgtagcttcacgacgtccaaaatgtactctaacgtcattgggagatgctatatttct
gacatgaaagaaagacatccatgagtaaatgattatcaagtatttgttttatttttttaatttaaacatta
aataatgcattctgatcattttccagcttgtgtactatactttggtcatgtttactttgaaatcaagaga
gaaaagtggggaaaatttcataaacattgaggcaatagtacaataattgccctgcattggatttagaaaa
ataagtccttgatgctgtaactttataacagcagaaaaaaatatatcttttgtacataattactttttttt
ttggtactagtaacctaaactgaatctgtactaatgcttgtatgccaaagaattgaaataaaaagtcaag
tggaattaattcctggtgagaccattccatggacagggaatgtgattggtgattacagacataatttatt
acaacaaaaatgaaataaattgactgcaatatatggtcaattaaataaaaaaattaaaacatgcagactt
ttatacaccaaggtgtggggtttgcttcaagacccagcctgactatgatgtcacatcctgtctaagatcc
atcacaaccaggaagaattggtaaccatgacgtcatttatactacatccacctacatcctaagaaaacat
ggctgcaggcagacctaggaaaagagcgaagcgtccacaagaatttacacaacacagtcaaccatggaac
tatggtgtaaaagtatccaagagtgaccaaccaggatctagtttgcaatctggctctgcccctccactaa
taaagcggccaagatttgatgtctatcaagaatcactatcttcctgtggcccaaatgaagtgccctccaa
gcttcgccctgaaaaggagagacaaaataaagaaaagttctttgttgggtcacgtgaggcaacatgtgtt
aataacwtaagtgaaaatgtcattataaacatctcaaaactaaatatgatattggaaaacttgcgcttaa
tgcatgacagacttggaaattgcaagcgatactgtccgcagataaataaaagcagaactcaaggactatg
tgcctttttaacagctacatgtaccagctgcaacctgaaggaatcagaacaggctgcattttcagaaaag
tgccaaggacagacaaggagaggaccagtcacaggaaatttaaatgagatgttggcactttcttgttaca
agacaaaagttggagtgactgatctaagattcattatatcctgcctgaacattttacctccttcttcggg
ctatttatacaagaaactaaacgaactctctagtactatcatatctgttaatgaatgtgttttagttgaa
aatcaaaaatttgttcaagaggttaacatgctaacatacaaaggcattcatgtacagacagacacagcgt
acaaccacagaccacagagtggggggaatctgccacacagtctgtggcgatagtcatggaacataccac
aagcagaaaacttccactctgtgtgtccattgcaaacaaactatgtagcaagaaaacatgcatacactta
aactgcaagaaaaaccatcgcacagaagactcgatgtcctccacagaaactaagttagcaaaggaaaatt
taaataagcttgaaatgttaaacatactaaaaattgattctttaacttgtgatgcaagcgctcagcttga
aaagactgtcagggaacattcggctaaggttcaaagacccatacgacaatatacatgctacgtgcatgca
atgcgcacacttcaaaaacatgtcagaaatgtagatttaactggtcctttcctggtaaggacaaacatc
tcttccagcaaaaattggcaactgctatcaggagaagagtacattttgagcttgtacgcattcaacgact
tttcagttctgaggaagtatttttggcaaaggcaaaggcagcggtccaaaatattattcaatgtttcagt
aatgagcatggaaattgccgaaaacattccttagtctgccttgcccatttagcatcctattcatcgaaat
ttctaccatatgctaaacatttaaatctttgttccgaagatactgaaaagcttgaatcagtactaagaaa
ttttgtctcagatgtcaagctaagtaaaatttcacgccttctaaacacaaataaatgtgagagcttgcac
agtaggattttttacatatgcaccgaagacatcagtatggagcagaaattttgatgcgttatgtcattcag
cccttcatagctctgtatttggtacaggaaagtctacactcatattggctgagtctcgtggaattgtttg
taatcagtctgaaccagtatttcagcacatggttgaacgagatcaaagaacagcaaaagatagggagagg
aaatcgagtcttccatacaaatatacacgctacatcaaacgcaaacaaaaatgcaacagaacacttattc
aaaattcagtttttgccaactcagtaaataattttgtaacgaatgaacattcatacactttaaatccttt
aaattaacaccatggaacaattatgaaatataccttaatatcacagtatacaacttccatagcccctatg
gccttggcattcaccattggctttacatacttttctaaccatgtttactttctttggttcaaaagataaa
tgataagtattaatacatgtctttgtatggcatctatgcgatacctcacactgagatggtagtgggtttg
ccatagtttctatatagaactttagtcggtgagccaaaactatcttttttttcccacgaaaagttacata
aactattccataaccatgtcggtcatgtgagcctttccaaatgtgacacttgctagtgacttgtgtatgt
cgagctaaagctcttttatatgtcattaattcttgagctgttaatttattttgaaatatttctgacatag
cttaataaccaagaccatatgatactatagaaatttttatctgaaaataaaaaaaattaaataccatgac
aatgatttttctgatttttttttcctctttgcccataaaatttaatttgtcaataaaaaaaaatcaaatta
ttaccagttcaaagaagaagtaataacatcttccttgaaaatggtgaaatgaatgcgaaagtttttaaaa
aaaaacaactacacatgtttctaattgacactagtcaatgctgtgttgtttacaattacaagggacataa
ctcttctcaaacctacctgcagccgcttaaagcagattttagaatgatgagacatatttaagttctaaaa
tgtagtgaatataaactacatgagccaatattaatacaaaaaacatcataaatataactctatagtgtaa
acccaaatgctctttat
>KolobokP-1_MaMa-I
tatcagggtcagaaattttcacctgaatggaaaaaaactaaaataaatgtgtctgaaatactgctgcaaa
gtttcaatgaatttgatgcatgcatataaattttatctgacaccattgtattacaaacagtatgaatttc
aaaatggccgactgtgaacaatgcacaaaacttaaagttgaatggtgaatttattaattaagataaacac
ttgaaataaactgtagttagtttttagtcctcatacaaatctggaaattactagaaattaaacctggctg
ctaaactctcagtggagagtccattttaaaatatgatacatatttttatttaaaaatttgaatttaaatt
aaattttcatgaaaatttaaaaactcaagttgacaaattttgttcttttgcaacatgtctccattccttt
atcacaaacatcaatggttttccttatcaggtgtgaagtaattaaaccagggcctgtcaataataggtag
cacctgaaatctggtagcatctgaatacgctagttaggggtagtaactaaggtagtgatgtaattttcac
cccttaaggggaggagctcttagatgttakgcttccaatggtactgaagagttcgtgaagttctatgata
ccagggcagcacacctttttcccagttatataaggggggagagaggataattcttgtacttttacatgcagt
tattttaactgaaaccatgaaagggtacaccaagaagaagaaaactttttttaaaccaggaaatattcca
cataacaaaggggtcagatttctgactgatgatactttaccaagttccagtgctgcatctgctcaagtga
ccagaagaatggatgcagacaagttctctctcatggcagtgacgaagccgacaggtgggggtctccagtc
agttgtggcaccagactgtaatggcacacctgggtctgctagaattctacggccatcatccaaagcaaga
ctgaccctgaaagagacagaagtgaatcagcatgaggggacacgtatgatctgtatgaataaaaatgtgg
aaatgtggaatgatatcatccatcgtcatggaaatcaagacaacagatgtgagagacctgatttcaaaat

tgctaatgaaacaaaatggggacttgcttggaagcatactatgatgtgtaaaaactgcagttttaagtca
ccagagtataagctgtatgacgagataaaaacagctaaacctgggccaaatcctgctgctgtaaatatga
cgtttcaagctggactacaggatacaccaatgggcaacaccagggctagatacctacttgcagccwctga
cattccaccacctagtagaacatctatgcaaagggcttccaacattgtaggtcaagctactgtacagcta
aatgaaatggatatggcagacaagctgaaactagtccgacaagttaatgtcatgcgtggtgtgcaggatc
ctgagcaaataaatgtcgcgtttgatgcaagatacaatgccattagctttggacatgagaagaaacctgg
tcaaagttcatcccaagctgttggaattgtctgtgaaactgttacagagaaacaatatattgtaggagct
acagtagaaaataaattgtgctggactggagcatggttcaaaggccaaggtttggatgtgcaatgcccag
gaggccatgttgattgcacagcaaatctgtcaccagttgcaccacattcagagcatgcaatggcaagaga
aataggaaacaagtttgcgctgcaagaaattctcattagacatgcaacaacagatggggatgctcaggct
gctgctggctttcaggatgcttacagcatcctatatccaatgtggaaagtasaaagattgtcggacccca
ctcatttgggtcgttcccagttcaaacggtgcaattcggcaacttttagtgaaagcatgtttccgggaat
aagaacaagagaaggaaaaaagctgaaacagaaaattctcagccaagatgttaaagcccgctgcagtttg
atcttcaaacgaatgatggaggacaccggtggtgatgttagacttatccaatgtcaacttccccaagtac
tggatgccactctgcggtgttattctggggactgcaacaaatgtcggcattattcgaaggtttgtggggg
aggaagtactaacagttggtggatacgatctgcatttctgggccctcatcaagtgactcatctaaacatg
acagaaagtgataagaatattctcctggaagtgttgaaaatgaaactttcctgtgatgttgtgcaaagac
tgcgacttaacacaaacacgcagaaatgtgaagctgtgaacagatcaattagtgtttccttacctaaaaa
tgttaactattccagaaactttgaggcaagagtgcattcaacaatacacaggctcaataacagacttgga
tcatcactgaaaacaaaagtaaaacacttgggaggtatactttcaccaaggactctgagatcactgaatg
aaatggatcaaacctgtacttaccatcagttgtatcaaaagcgtccggaaaccaggaaacgaatactggc
aagacggggaaatatgatacaggctcatcttaggtatagaactcagcccggcaacaggaagtctgattac
tgcaaaggcttattggacagtcattccaagggtgatcactcctatgcacaggtttagcaggagccaagga
cactgcagacagaaaagatgatcttagtccatgtacaaaatgacagtgcccaaagacacaatgaaaaaca
cataatacagaaaggaaaactgtcgtggggaaatccgtaggtaacacaacgcatgttatgtgcagttgtg
ttacctactaccacctgatgagacctatggagaggtcgaaacagttttctcgaaacatgaagacataaat
atagaaataaaatttttaaaaaaataataawtaaataataacatattctaacataacatgaactgactga
cctgactgactgactgactgactgaatgaatgaatgtatgaatgtaatgaatgaataaataaatgagtga
atgtattatgtaagataaagctgtcatgccagcatggtcaaataaaataaagaccagttcctctttcttt
cttctatcctccttactcaaccaaacaatcatccatccatcmatcaatcaatcaatcaactaatcccatt
ttatccatgcaaccagcaagccagccatttcatgctaatatacatttctatatggatgtgggtgggtggg
tgtgcagggcatattattatacctgcaattattccatatcacaatttcaactgtagcatacagtttggaa
attcaccatgtccaaaacaattcccatatcctttgcagtactgcctattattgttgatacgatggggttc
caggctgaggtggtccgcattaacacacaaactattgtggcataaatgcgatgcatctaactcttttgga
atgttcagacttctcatctttatcattaatgccagtctgtggacatgtttctttttccaaacattagtct
cagggtccctatatgacaatagcccatataacctatttgatgttacatttcctgtccagagtatgcaagt
actgttaggtgcaggcacagaattgaacaagattttctcagacaaaatttcaaaaatatccgccattgca
acttgtataaggtgtaggaatacatacatgctgataataatgagtatcaaccccacagggtgcatttaaa
aaaaaaattgaaaatttaaattttatttaaaaattaatttaaatttttaaaaatatttaaatttttttaat
ttttaatttcatagttctatttttaaaggtaatatacacaaaatgtccaaatttcatgcatctaccttga
ataataaactcaggataacaatttaaacttttataaccttcctactgaca
>KolobokP-2_MaMa-I
tataaatagatctgaatttttttacaaagagatgtattttactcattgatgttggagcatggttgtaataa
agaattggccactctcgtgcaaagatattagtgtaacggcatttgatgtgataccactatctatagctaa
attgtatccgcataacattttgctgtattgtattgtacatattatgatttattcttgttattaaattcat
tgttttctgtatatgtatcttcatgttatatttctatggcatgcatggtacacaatagaaacacaaacat
aacatgcagacatcagcaagtattatataactgggtatcaaattacatgacacattatattttcatctct
cactggctataagacaagttttaatagcttatacaattcaaaatgaccttttgtttcctgactcacacag
aaataaagtacagtgtatccctattcccatctttcaaatcaaactaataagtgtgccagatgacagagga
taatgataaggggtcattgcccttctgggactaagagaaacaaacacacccatgaaacaacaagtgatgc
tgacattccaaaattatctmaactgtcaacacctgtgatttttcacccccaaatctgctcagtaatgtat
aaaaagccaaacaagtaccataagttttattctattgaaaaattaaatagttgagtgctacttttttatta
taaaactatgggaaagagtaaggaaacacagaatagacaaagtaatttcttcaaacctggtaatgttccc
cacaacaaggggaaaaaatgtacatcaaccagaaaaacaaagccactcgaggtatgttacataaggacaa
caagagaaaaattggaaaaagctattcaaagttcctataatggtgaaaccgatgttggagacagcaggcc
agtgtgtaaattgctgcggcccaagccaagaagtaagctggaggtggagaaatatatggaaaactttgcg
atgccaaggtaaatacatgtttcaatacaatatacacacaatgatataatttatgactgcataagatact
cagtaaatttaagcaataggattcaattacatgttaaagttctccgatacataaatctatacctactttt
agcaaaagatcataaaaagttaagaaaaagcccccaaatcactattgtgccaactttctactacaagcag
tgttaaattacttatctatcaagataaagctggttaaaatacataaattatagagaaacagagaaataaa
gataatcctgcaatcactcagttacagtcaaagggagaggtaatataccttaatttggcataccaagtag
caaacttgaatttacagttaaatgagtctatactaccattgatactaagtgtagagcaattttcctttc
taaatagctcttctttccaaatctgagcacacatttctgacatgaaacatgttttcttctattcacatca
acagatctggagaagaaaacacatatcgcttagtgcacacagggaaaaatgcacagatgtggaataccat
gttccatgaacaccagttcaaacacccgcagtgtaatggttttctctcctgggatctaagacatgaagag
aaaagaggtctagtgtggcgggagagagcaatatgttcgacatgcagttatgcatcaaaaatgtttaatc
tgtatgatgaggtacagaagaccagcacgaaaggaagaaaggcagcctgtgtaaatagctccatacagat
aggactttcacagacttctattggaaatgaaagtttaaggactatacttctaagtgcaaacatccctgct
ccctctactaaagggatgcagaaaagtgcaaatagtgtttgtaaagaaattgaaggtaccaatattagag
acatggctgaaattaggaagggtgtcaaggaatttaattttatgagaggaaatgatacaaatataattga
tatggaatgtgattctacatataataatgcaatttatagtggggtgggtaggacaccatttcaaccagct
acccagtgcacatatattcaagttgaagatactactccagagaaaaaggtgataaatataatcacaaaga
acaaactgtgcacatttggtaaaatgcacacttcagatgatgatgccaagctgtgtcactgttcagcaaa
tataagtttacaaagaaatattggtgatgaatacagttggtgtaaggaagggcttatgtatttgaaaaaa
tcagacaatctagaggtacacaatattacaactgatccagacactgctagtttccgggctagcctggacc
ttaggagggagggggtaacaaaaacagtgccagaaaatcatattgatacacgccatctgggtcaaaatca
tacaaagtttataaagaaacagagcaaattaattgatttgatgccgggaagaactaaaaagcggagggaa

aagatgcggaacaactttgcacatgatttgagtaagaggtgccaggctgaatttgaaaaatcattccaaa
aatgtggtaaatgtacaccaaaattaattagtaatttatcctatgcaacagatgcaatagttaaatgcta
tcaaggtgatcatagtctttgttacaagcaatctaatgtgtgctgggtagatggttgcaactgggtatat
agaagcacattcttaccaagttatttcaagctgaaaatatccagtgagagtgaagagtctacattacgca
tgtgcattgagtatcggttaggtaaatctacagtgctgaaaacaaaactgaacaagaacacccagaaagt
ggaatcagtcaatcgaaggattaaacgtagcttacctaaaaatataaccttcaagagaaacttccatggt
agggctcatagtgctgttcataactgtaaccatggacctggagaatctttggtgaagctatgtaaatcta
tcggttgtccggtttcagcttgtagttctgtggcacaacatctgaaaaccaaacaagatgtatatctcag
agataaaatgtacaaatcaagtaaagtatataagaaaaaaagatctgagaaaagggcatttctttacaaa
ctgtatgagaaatatcaagaacaaaagaattatgacaaaggcaaattgctgtgtgctatggctagaaaaa
taaaagaaaaatatgaacactcttaccataagccacctccatttagcactttttttacaggccgatgcaa
tgtggcacgtgcagttttgtgcacagtccttgattttacaatgctccctgctttgatttattgaatgtg
tttctaaagttaaatgatcagtattgacacataagcgattgtgacatatgtggcttacgtttaaaatctc
tccccccccccccttgtgggaatcgttgcatacgttaaatggtattkagccatataagccaatctgtgga
caccaacatatgaaattctgccatctggccatactattctctttttttccatagcctgagccatctaccgc
ccctgtccattgcatacaaccctttccatcagtgctttttacagatttgttctctaaaatcatatgcaaa
ttatgaaaaaaagactccattgtgattttcataaccaaaaaccaccaaaacaataccctggatccacgtg
gtaaccatggaaacaacaggctcttggtatggtatagtgcccctata
>KolobokP-3_MaMa-I
ttaaacagtcactgaagatgctttgtcaacattttgaagaagctgacatattctgtagtcaatttcaaac
attttcatatatgatacatggtatttacttccaaaatgaccattacatagtcaatttactccaccccact
gaagcagcttgaatgtagcttgatgacgtcaagaatgtagtgcaacgtcattggaacatgctataatttg
gtcttgaaagaatgacatwcaagagtaaatgcctatgaagtatttgtttaatttttcaattcatacatta
aataacatattctgagcattttctgagttgtctactatcatttggccatgtctacagttaaaasaagaga
gcaaagagggaataattccttaaattttgaagtaatagtgcaataattgtcttgcattgcatttagaaaa
gcaagttcttgatgctataatttcattgctgaagaaaaaaatatatttgttgtaaaaaataactttttc
ttctgtactggtaacctaatatgaatctgtactaatgattttatacctatgtgtttaagtgatgtcaagg
ggaattaactcttgttgagaccattcagtgcacaatgaaaggtgattgataattacatccataataatgg
attaccaagaaaatgaaacaacaagtagactgcattttatggtcaaataaataaaaacctcataagttgc
cactttgtaaaaaatgggggaagtggttttcataatagtttcaagcccctgcctgtctgtgacatcact
tcctgtctaagatccatcacaaccaggaacaattggtaaccatgacgtcattagtactacatcaacctac
atctccccaaagaaaacatggctgcaggaagacataagaaaagacctaggcgccatcaacagtttgtaca
gcacagcataccatggaaccgcggtgtagaggtatccacaagtgacccacctagttcaagctggcaatct
ggctctgtccctccacttatcaagcgcccaaggtttgatgtctaccaagaatctctgtcttcctgtggcc
aaaatgaagtsccatccaagctacggcctgcaaaaaakagaaaaaaatgaagaaatgttcctcagtgggtc
acatgaggtcaacggtgttgacgacacaaatgaaaatgtgattctaaatatagcmaaagtcaatattata
ctgsaagacttacaccaaatgcatgaagaaattggaaattgtaaaagatacagtccacagatggtcgtaa
gcaaaactcaaggactgtgtgcttttgtaacagtgaaatgtgtcaagtgcaatcttgtgaatgtggaaca
gcctgtctttaaagagcaaggtcaagagtggaggaagagaggcccagtcacaggaaacttgaacgagatg
ttggcatttcttgtttcaaaacaaaagtcggagtgactgacctcagatttatcctgtcctgcctgaaca
ttcaacctccttcttctggtttttatataagaaagttaatgaactctctagtagtataatatctgtcaa
tgaatctgttttactagaaaatcaaaaatttgtttatgaggttaaccaactaacacacacaggcattcat
gtacagacagacaccgcatacaaccacagaccacagagtgggggggaggctgccacacagtctgtggcta
ttgtcacggaacactccacgagcagaaaactcccactctgtgtgtcaattgcaaacaaactctgcagaaa
gaaagcatgcacacacttcaactgcaaaaaaaatcatcgcactgaagactcgatgtcctcgacagaaacc
aaattagcaaatgaaaatttaacaaagctcgaaactgcaaacatactgaaaattgattctttaacttgtg
atgcaagtgctcagcttgaaaaaactgtcagggagcattcggctaagtttaagcgacccatacggcaata
tacatgctttgtgcatgtgatgcgtacacttcaaaaacatgtaaaaaatgtcaatttcattggtcctttc
aagggtaaggacagacatctctttcagcaaaaattggcaactgctattaggagaagagtgtatttagagc
ttgtatgcatacaacgatatttcaattctgaagaaatatttttggcaaaggcaaaggcagcagtccaaaa
tgttattccatgtttcagcaataggcatgaacactgccgacaaaattcccttgtctgccttgcccatttg
gcatcctattcaccaaaatttctgccgtatggtaaacatttgaatctttgtcatgaagacagtgaaaagc
ttatattggtcttaagaaatttttctccgatgtcaaactaggtaaaatctcacgtcttctcaacacaaa
taagtgtgagagcttgcacaataggattttcacatatgcacctaaaacatcagtatggagcagaaatttt
gatgctttatgtcattcagcccttcatagttctgtgtttggtacaggaaagtctatgctcctactagctc
aatctcatggaattgtttctaatgaatctgatccagtatttcagcacatggtagaacgagatcgaagaac
agtaaaagatagggagaggaaatcgggtcttcagtacaagtatacacgctacatcaaacgcaaacaaaaa
tgcaacagaacacttatccaaaattcagtatttgccaattcaacaaataatgttgtaaccaatgaacatt
catacagtttaaatcctttaaattaaaaccatataacagttattaaatgtacctgaatatcataggatgc
aactcccatagcccctatggccttggcattcaccattggctttacatgattttctagccatgtttactct
ctttggttcaagagataaatgatcaggattgatgcaaatttttgtatggcatctatgtgatacctcgtaa
tgagatggtagtgagtttgccatagtttgtaaataaattgttagtcggtgaaccaaaacaatctttcttt
tcccacgaaaggttacatacagtattccataaccataacgatcgtgtgagcctatccaaatgtgacactt
gtttgtaacttgtgtatgccgagctaaagctcttttatatttcattacttcttgatctgttaatttagtt
ttaaatatttctgacattgctgtatgaaccaagaccatatgatactatagaaatgttatctgaaaatgga
aaaatattaaacttcatgacaatgtaatctttcttgtgttttccttacccatacatttttatttgtcaat
gcaaaagagaaaataccctaaatattatcaattcaaagaagaactgatattatattccttgaggaaattg
acattcatgcaaacatttgtaataaaaatgtctatcattacacacaggtttctattttcgacagtaatta
ttaccgtgttgtgtacaatgacaagggacataactcttctcaagcctacctgcagcctcttagttcaaat
tgatttttggaatgaagagacacatttaagttctaaaatgcagtcaatatgaactaaatgagccaatatc
aatacatacaacacataatttaactccatgtgtacacatcctaatgccctataa
>KolobokP-4_MaMa-I
tataccgaacatacatttttggtatttttgtttattgacaaccttaggcaccaggtatgtactatactaa
cttgggcgatattctaagcgcactaaatgacaataatgaacacttatgttatgccgacagatgataagaa
tgaattttggaattcaaggatgtttaccatgttgatacattatagatgtagataactgaataaatacagt
gattgtaatagtttgtctcatgtttttctttatgtcttgtgtgtctgctattcataattgcatgcatgaa
atatccacctataagcattatgtaataacaggtatcaaattacgcaacccccctgttttcatcaatttctg

gtacaaagatcatattttgsttgcctaaaatttccttcattggagattttgttatcttgaatcacagatt
aasaagtgttaatggaaccaaacaggttaattttatttttcatcacttgtcacatgacaaattgatatat
ttagatatctggaacttgaggccaaatgtgagtctggccagactagaaactgatactagcttccattggt
gcaaataattaatataattaatgaaaacaatgcagaatgtagttctaacagcactaaacagatacttaat
tcacactttgaaactcaaagcctaaactacagttatattacagtgtcaaaaatgccaaaagtaaagagaa
aaaccaaaggttgtcttaatagaaaaaagtttcacttcaaaccaggaaatattcctcataacaagggtgt
aacaagagagaaaagtgtagagagggatgtcacaaaaacactgtataaaagactttcagaggaaaagtac
aatcttgtcacaaaaagaccaagttcacactctggtattgataaagaggaagtttcagtgcaatacaagt
tcctacggccaattaaaaccaaagatttacaggctgatctgcatgccagtgactccgaacaaaagaggta
aatagaaattcatcattattcaatgttaaaaaagattatcaatcaatttggtttcctgattttcattaca
taattcatgcaggaaagtaatcgcaagaaaaaatataacaatgattaacaggtaaaaatggcattttaaa
aaaatgttgcagacaaaatatgtgcacaaatagcatttattctgagaggcaggtctcttcgcataattcc
taactgtaataaaaaaaaaaaaaaaattaccatttgagtgttaaagttcacattctgctactaagagatg
tgttagcccatgctcaaacaatgcagtaggggaggtaagctatccaaggaaactaataccttgacacaga
gagttgaaaataagaaattaatgcatttagaaagcacctggcaatataaaatatgataaatgtaagattc
tatgtgacaaaaactgtaatatattcataattttcatatcaggtctggtgaaaagagtacatacaggctg
gtacagatcaagaaaaatgaagaaatgtggaattcagtgttcacagaacacaagagaaaatatcccatgt
gcaatgggaacctagaatgggagatggaaagtgaggagaagcgtggtctcgcatggcgagaaagggcaaa
atgcaaattatgcacctacaggtctgatatgttttctttgtatgaagaggcacccaccaaataatcccgg
tcgaaaagctgctatgataaacaagggcattcagattggcctttcacaaacatctattggaaattccagc
ctaagatcaatccttttgagtgccaatgttccagcaccatcaaaaagtggcatgcagaaggcatcaaatt
ctgcagcacatgccatagaggccgagaacaggaaagatatggacaaaatcagagaagaaataaaagaagt
gaatttgttgcgaggcaatacagcaggtattattgacattcaatgtgatggtatgtacaataatgcaatg
tattcaggtgtcggaaaaacaccattccgccctgcaactcagtgcagttacattcaggtagaaaatataa
ctcacacaaacaaggtgattaatgtggtcaccaaaaacaagctctgcaattatggtaaatttcatacatc
tcaggaagaagttgataactgtacctgtagtgcaaatataactgttcaaagggatattggtgatgaaaaa
tcatggtcaaaagagggtcttctagaactgaaacttcaatcagacatggaggtaaaaaatatgacaacgg
atccagatagtagtagctaccgggcagctgtcgagcttcatgtggctggtacgacgaacactttccctaa
gaattttattgatacaaggcacctcggacagaatcacagaaagttcattaaaaagtctaatgtacttaca
aatctgatgccaggaaaaaacaaagttgaacgcgagagaatgaaaaacaatttttcaatagacctgagca
agcgttgtcatgcagagttcaatgctgcctttgagaaatactgtggagacacttctatgataatgagggc
aatgtcaagagcagtagatgccatagtgctctgttatcagggtgatcatgtcctttgcaagactaagtca
tatgtttgcacttgtgaaaatgcaaactgggtagttaaaagctcttatttgcctggcacatttagaatag
tgaagtctacacatactgaatcaatgatacggatgcttgttaactaccggctagggaaaatgacaattga
gaagacatatctcaacactaacacacaaaagtctgaatgtgtaaataaagtcattcgaagaagtttacct
cgcaatgtaacattcaagaggaatttccatgcacggtgtcatagcgcagtacattacagcaacaatggcc
ctggggagtctatagtgaaactgtgccaagcttctgggtgtcccatagcagcaaactccagtgtggctat
gcaactgaaaagaagacaaaaagaacatatcattgataagacattttacaagaaaaagaaagcatacaaa
cataataaatgtaaaaagagggcacttttattcagactctatgagaaatatcaggaggagaggaattatg
caaaaggcagattgttatgtaatattggaaagaaactaagagctaaatatgacaagagtgttaaagattt
accttgaataagatctgttatattaaacagatgggtttgtggcattttgtacatagtccctggtttttac
agtgatctctctctttattgatagcatggggttccaaaactaaatggtcaatgtttacgcacatttttc
atgacacaagtgactaacatcgacaggctgatttttctcatcaaatctwggcattaagtcaggtgtaagt
cttctttgtatcatatatgagaatctgtgtgcacttaaataggaaatggtgccatcaacccatctcactt
tatgcttcccatagccagatttgtcaggtttcccaatccagatcacacaaccatgcttgttttccataat
tctttgctttttcaatagaatctgctctaaattactccaaaaatgatccattttactataattacgctga
aatcaatcaatgatactcaaaacggatgctgagatgcacgtggttgtcatggatacacacactaccccaa
ccctata
>KolobokP-1_MeNe-I
ttaaagaaccactgaagatgtgttgtcaacttattgaaaaacttgtcatattctgtaktcagtttcaaag
attttgatatattatacatggtatttacttccaaaatgacaatcacatagttaatttactacacccctct
gaagtagcttgaatgtagcttcatgacgtcaaaaatgtattctaacgtcattgggatatgctataatttt
gacatgaaagaatggcattcaatagtaaatgcctatcaagtatttgttttatttttcaattcaaacatta
aataatgtattctgatcatttcccaaggtgtgtactatactttggtcatgtttacatttaaatcaagaga
gaaaagtggggaaaattccataaaatttgaggcaatagtgcaaaaattgtccagcattggatttagaaaa
ataagtccttgatgctgtaacttcataacagtagaccaaaatatatcttttgtaaataattacattttt
ctttggtactactaacctaatttgaatctgtactaakgcttgtaagccaaagaactgaaatgaaaagtca
aggggaatcaactcctggtgagaccattccatggacaaggaatgtgattggtgattacatacataattga
ttacaatgaaaatgaaataaattgactgcattatatggacaaataaataaaagcctccaaacatgcagac
ttttatacattaagggtgggggggtgcttcctaacaatttcaagacccagcctgactatgatgtaacat
cctgtctaagatccatcmcaaccaggaacaattggtaaccatgacgtcactaatactacatccacctaca
ttagccacttcctaagaaaacatggctgcaggaagacctaagaaaatagctaagcgtccacaagagttca
cacaacacagtcaaccatggaactatggtgtacggggatccaagagtgaccaaccgggatctagcttgca
atctggctctgcatatccaccaataaagcgcccaagacttgatgtatatgaagaatcactatcttcctgt
ggcccaaatcaagtgccatccaagcttcgccctgaaaaggagagaaaaaatgaagaaaagttcttggctg
ggtcacatgaggcaacaggtgttgacaacatcaatgagaatgtaattatcaacatatcgaaactaaatat
gatactggaaacactgcgcataatgcatgacagagttggaaattgcaagcgatactgtccacagatgtat
gcaagcaaaactcaaggactgtgtgccttttttaacagttaaatgtaccaactgcaatctaaaggaagcag
aacaggctgcctttcctgaaaagtgccaagggcagagaaagagaggcccagtcacaggaaatttaaatga
gatgttggcactttcttgttacaagaccaaagttggagtgactgacctaagatttattatatcctgcctc
aacattcgacctccttcttctggctatttatacaagaaactgaatgaactctctagcactattatatctg
tcaatgaatcagttttagtggaaaatcaaaaatttgttcaagaggttaaccagctaacatacaaaggcat
tcatgtacagacagacactgcatacaaccacagaccacacagtgggggggagtctgccacgcagtctgtg
gctatagtcatggaacattgcacaagcagaaaacttccactctgtgtgtccattgcaaacaaactatgtg
gaaagaaaacatgcatacacttaaactgcaaaaaaaatcatcgcacagaagactcgatgtcctccacaga
aactaaattagcaaaggaaaatttaaataagcttgaaatgttaaacatactaaagattgattctttaact
tgtgatgcaagcgctcagcttgaaaaaactgtcagggaacattcggctaaggttcagagacccatacgac

```
aatatacatgctacgtgcatgcaatgcgtacacttcaaaaacatgttaaaaatgtagatttaattggtcc
ctttcctggtaaggacaaacatctctttcagcaaaaattggcaactgctatcaggagaagagtacatttt
gagcttgtacgcatacaacgacatttcagttctgaggaagtatttttggcaaaggcaaaggcagcagtcc
aaaatattattcaatgtttcagtaataagcatggaaattgccgaaaaaaattccttagtctgccttgccca
tttagcatcctattcatcaaaatttctgccatatggtaaacatttaaatctttgttctgaagatattgaa
aagcttgaatgtgtactaagaaattttgtctcagatgtcaagctaagtaaaatctcacgccttctaaaca
caaacaagtgcgagagcttgcacaataggatttttacatatgcacctaagacatcagtatggagcagaaa
ttttgatgcgttatgtcattcagcccttcatagctctgtgtttggtacaggaaagtctacactcctactg
gccgagtctcgtggaattgtttgtaatcagtctgaaccagtatttcagcacatggtagaacgagatcaaa
gaacagtaaaagatagggagaggaaatcgagtcttccatacaaatatacacgctacatcaaacgcaaaca
aaaatgcaacagaacacttatacaaaattcagtgtttgccaattcaataaataactttgtaaccaatgaa
cattcatacacattaaatcctttaaattaacaccatgtaacaattattaaatgtacctgattatcacagt
atacacctcccatagcccctatggccttggcattcaccattggctttacatgatttctagccatattta
ctctctttggttcaaaagataaatgatcaggatttatgcatgtttttgtatggcatctatgtgatacctc
acactgagatggtagcgggtttgccatagtttctacatagaactttagtcggtgagccaaaacaatcttt
cttttcccacgaaaggttacataaattattccataaccatgtcggtcatgtgagcctgtccaaatgtgac
acttgctggtgacttgtgtatgtcgagctaaagctcttttatatgtcatcaattcttgagctgttaattt
agtttgaaatatttctgacatagcgtaataaaccaagaccatatgatactatagaaatttttatctgaaa
ataaaaaaatattaaacaccatgacaatgaattttttttttcttctttgtccataaattttttatttgtc
aataaaaaaagagaaaatatccaaattattaccagttcaaagaagaactgatatcatcttccttgagaaa
ggtgaaattaatgcaacagttttacaaaaacaactacacacacctttctagttgccactagtcaatgctg
tgttgcgtacaatgacaagggacataactcttctcaagcctacctgcagccgcttaattaaagcagattt
tggaatgaagagacacatttaagttctaaaatgcagtgaatataaactaaatgagccaatattaatacaa
acaacattaaatataactccatgggtatacaccctaatgctctttat
>KolobokP-2_MeNe-I
tgacagggtcagacaatttcacctccatggacaaaacctcgttccgtgatcaaagcaatgtctcttaaat
actgctgcaaagttttaaggaatttcatccaggaataggacagctacaggcttttatctgacaccactgt
aacacaagcacaagccaattccaaatggccgaatgtcaaaaatgcacaaaacttaaagtttaattgtgat
tttgctaatcaagataaacacttcaaataaactgtaattagtttttagtcctaacaccaatctggaaagt
actagaaattaaacttgactgttaaactttcctagaaaattcaattttaaaaaatgatacatatttttat
atgaaaatttaaatttaacttaaattttaatgaaaatgtaaaatctgaagttgacaaattttattctgtg
gcaaagtctctccattcctttatcatgaacatcaatggttttccttatgtggtatgatgcaattcaacca
gggcctgtcaatcaaaggtaacatcttaatacgctagctattcgctgtactaaccaagtacatgacatca
tttccacccatgaaggggaggagccctcatatgttaggcttccaatagtattgaagagttggtgtagctc
tctgaaaccaagctagcatgcattttcctaggcatataagtgagagagagagacgatacttcttgtattt
tacatgcagttattgtaacggaaaccatgaaagggtacaagaagaggaagacaactttttttaaacctgg
atatattccacataacaaaggagtcagacatctgactgatgatactctaccaagttccagtgctgcatct
gttccagtgaccagaagaatggatgcagacaagttctctctcatagcagtgacaaagccgacaaatgggg
gtctccagtcagttcaggcaccagactgtaatggcacacctgggtctgcaaaaaattctacgaccatcatc
caaagcaaaactggacctgaaagagacagaattgaatgagcacgatgggacacgtatcatctgcatgaaa
aaaaatgttgagatgtggaatgacatcattcatcgccatggtaatcaagtcaacatatgtgacagaccag
agttccaaattgctaatgaaagaaaatggggacttgcttggaagcatactatgatgtgtaaaaactgcag
ctttaagtcaccagagtataagttgtatgaggagataaaaacaaataaacctgggccaaatcctgctgct
gtaaatatgatgtttcaagctggactacaagatacaccaatgggcaacaccagggctagatacttgcttg
cagccactgacattccaccacctagcagaacatctatgcgaagggcctccaacactgtaggtaaagcaac
tgtggagctcaatgaaatggacatgatagacaagctgaaactagtcagacaggttaatgtcatgcgtggt
gttcagcatccggagcaaataaatgttgcttttgacgcaagatacaatgccattacatttggacatgaaa
agaaacctggtcaaagttcatcgcaagctgttggaattgcctgtgaaactcttacagagaagcaatacat
tgtggggactacagtagaaaacaagctgtgttggactggagcatggctgaaggggaaaggttttgatgtg
caatgtccaggaggccatgttgactgcacagcaaatctgtcaccagctgcaccacattcagagcttgaaa
tggcaagagaaatagggaaaaagtttgtgctgcaagagattctcatcagacatgcaacaacagatggaga
tgcacaggctgctgctggcttccaacatgcttacaacattctgcatccaatgtggaaagtagaaagactg
tcggaccccatccatttgggtcgtttacagttcaaacgctgtaattctgcaatattgagtgaaaacatgt
ttccaggaataagaacaagagaaggaaaaaggctcaaacagaaaattttcagccaagacattaaagcacg
ctgcagtttgatcttcaagcaactgatggaggacaatagtggtgatgttacaaaaattaaatgtcaactt
ccccaagtactggatgccactctgcgctgttattcaggggactgctcaaaatgccggcattattcaaagg
tctgtgggggaggaagtactaacagctggtggatgcgatcagcatacctgggccctcatcaagtgactca
tcttaacatgacagaaaatgataagaatatcctgctagaagttctgaaaatgaaactctcctgcgatgtt
gtagaaaagactgcgactgaacacgaacacacagaaatgtgaggctgtgaacagatcaatcagtgtttcat
tacctaaaaatgtgaactattccagaaactttgaggcaagagtgcattcaacaatacacagactcaataa
cagcctgggatcatcactgaaaacaaaagtgacacacttgggagggaaactttcaacaaggactctgaga
tcactggaggcaatggataaagactgcacttaccatcagttgtatcaaaagcgtccacaaaccaggcaac
gtattctagcaagacgaggaaatatgatacaggcccatctgaggtacaggactcagcaaggcaataggaa
gtctgactactccaaaggactgctagacaccaagggcgatcactcctatgcacaggtttagcaggagcca
atggacactgcagatagaaaagattatcttaattccatgtgcaaaatgacagtgctcaaagacaccctga
agaacacatggtacagaagggaaaactgtagtggggaaatccgtaggtaacacaatgcatgttataagca
ttgtgttacttactaccacctgatgagacctatggagaggtcgaaacagttttctctgaatatgaagaca
caaataaagaaataaaaaaataaaaaaataaatcttaaaaaattctaaaataaaataacatgaaatgact
tactagctgaatgaatgaatgaatgaatgaatgaatgaatgaatgaatgaggaaggaaggaaggaaggaa
ggaaggaaggagagagggaggaaggaaggaaggaaggaaggaaggaaggaaggaaggaatgtatttaaga
cagcactgtcatgccagcatggtctactaaaataaagaccacactctttcttttctccttcttactcaac
acatccatccatccatccatccatccatccatccatgccatacatgccatacatgccatatcatgatacctgga
ttattccatgttacaatttcaactttatcatgcagtctggaaagtcaccatgcccaatgcagtgcccata
acctttgcagcactgcctattattgttaatggaatgaggttctagaattaggtgatcgacattcacgcac
aaactgttgtggcataaatgggatgcatcaagctcctttggaatattcagacttctcaactttaccatta
atgccagtctgtggacatgctttttcttccatgtgtttgtctctggatccctatatgacaataggccata
taatctattggatgttacatttcctgtccatattctacaatcactattgtgtgctggcacagaatttaaa
```

gagattttccctgacaggctttcaaaaaaatcctccattgcaacttgtatatggtgtaggaatacattgt
tgctaataataataagtgtcatctccaacagataccatttgtgtgaatttatggccaaaaaattcatttt
taattttttaataaaaaaaattaattaaaatttttaaattaatttttttaaaattttaattatattt
tctaattttacagtcaatatacacaatgtgtgaaaatttcatgtgtctatcattaataataaaaatttt
atagcaatttaaagtttagtcaatactgccctgaca
>KolobokP-3_MeNe-I
ttaagaatttaaatagcagtctgactaagggaaaatcttcttattttcaaaactttgggcaattctaaat
aaaacgcaaccttaacatgtgtaaaattcaagtttgtttgtttcctatggtttattgcttttatcgatga
ccaaaagccatcaaacagagcaacagctatgagacagcttcaaacttcatctattactttccccactgaa
acataccatttccttataacatatcagtttcatcatttttcataaccaatacaccctgtattgttagtgc
ctggtgatatcagagatatstgacagcasaaataatacaactcttcatgaaatctgaaatagtagtgaat
aaaagatgcacaagaagccaagctgaatctcttgtaaaataactgcagaatgtacatgctaagtacttga
aagaataataacgatgcacagcatatataaactgaccagtattctctcactgatttaataaagaaggtca
aatctatgttacaatacagaaatccgaggtgaagtataagggaggcaactcctcaggtaaccccaaattg
actgaccaatgtttgtaaacagaaccaattcatttgggaaacttcaacagtgaaattctatacccagact
cagcacatctgctgagtagacaatatgtatggaaaatgtagtttcacacctctaagaaggtcacctttgg
gtcattttcacactggccagctcatattgttgctgaagctgctgcttttcagacaagtttttttcctag
ttattgtaacatgcctacactcaactctaaagtaaaaagaaaatcatgcaaaagaaatcaggggaggaga
aaaacacagtttaaaatgggacatccatttcttgcaagttaccaaaactccagtgaagttgatgcagata
cacctgttcctgcaaaagccccacaacaactgatgcggctgactcccgatgaaacctttgatgtcatcca
ggctagcaatgtctcttcagcaagtgtgctaccatacaccttgaggcctagaagaaatgaaaaccctgaa
cttcaagaagaatgcagtcagtcatcagaagaatcagatgaaaatgtgattattaattttaaaaaattaa
cacatttgtttgaagcttttataccccatatgtgtgatgttccgaaaccacaagttatcattgacaagcg
aatgggactgtgcattacagctgcagtgacttgtacaaactgcagtttcaaatctatgggtgtggaactg
tatgaaacagtaaaaaagtcacgcgggccttctgctggtgtgcttaatgaatccctggttttaccagttt
taaaatctaaagttggtatagcagatgtacttttagttctatcatgtatgaacattaaggttccagatgc
acatggattacagagaaaacttaatatcctatctgacaaaatggtggatctaaatgaaggacatatggaa
aaaaaccagcagtatgtgcactgcattaatctcattgctgggagcattgatgacgaagcagtggatacag
aatatgatgtcttctatgcaagtaggccacaggctgggtgtgaggcatccacacagagttttgcaccatt
aattgagagaaacacccggaagaaattagtaatttccctgcaagttggcaacaaactgtgcagaaaactt
tagtgtgatcacaagaacaataaatgccaaaataaattgaaaagtgaagaatcaattgcctcagccgaat
caaaatttctaaagagaaatttagaaaacattcagtctcgtaaaatattaagggtgcggtcagtcacaag
tgatgcaagctctcagatagcaaaagccttatgccagtattcaacttcacaaaatcaccctattgtacac
tacagatgttttgttcatagattacaaataatatacaagcaaatcaaaaacattaaattaactagttgcc
cgaaagcatatgataaaagattgtactgtcaaaaattagtatcatgtattcaggccaggattcggttaga
acttgtcagacagcgatctcggtgtacaactgatgatgcagttgttcaatattccaaaactgctattgac
aatatattatattgcttccggggtcagcatacaatgtgttgatctagattaatggtctgttgttcacacc
ttgcatcttacagtcctaaatttcttccctatgggaaacatcttcagctcagtatgtcagatattaagaa
gttacaggctaaaatagataatctttgcagccaagaaaatttaaagcaaatgtcaaaactgtcaatcact
aacaaagctgagagcctacatcaccatgttttcacttatgctccaaaaaatacagtttggagctgaaatt
tttctggtctgtgtaattcagccgctcattcatcttcattggggtctggccagtctaccattcatttggc
aaaacatatgggaattgaagttaaatctcaagatccattttatatgcaattaaacaaaattgacatgaag
gcaaaatatgacagtaagaggcaaatgacttttcactacaaaagaaataggtactttcttcacaagaaga
aaaacaatcaaacaatatgtaagagctctgttttcactgggcttcaggagattgctgatgaacatgagta
cagcttaaatccagtatcatgaagagaacaaactataaaatggaaaaactgcaaataatttgtaattatt
accttacaatatgcactttggaaagccataatgtcctgtacactcacccatgtttcgacaaatatgtcga
ttattattaattgatgcagtttccaaagataaatgtgaaatttctatgcatgctttattgtgacatatat
gtgacacgtgtagatttttttgacatggtcacattaggatggtgcaagaaaaaaaagtaaacgatgtgctag
aagatttattctcttccctctgaaatgtgtacagatcatcgcatatccagctgatgtttttggtccaagc
caaattgtacatgtgtccaattttacagttttcttacttaaaatctcatgaaattgctgcttttcaacag
gtgttaaatgggtagtgtatatataggtcattataactgaaaatgtctgtaaataagatgaaatctgtgt
tgaaacattacatcatgagcatataggaatgtaaatgcataagattaagtcaattagcattaaattataa
aaatgtagaaagtatgtattgtaaatattgaagaaaaaaatgaataagtaagtgaaatacttcaattaaa
ctacattgtatcaaacttataaggtctttgttgttcattagaagattggatcagaaacaacacctgttaa
ctctgacagtgctgagttagctcccttcttctactatgatttactactatcaaatctataggaagtgtat
gttttggaatacctaactatgtcagattcaagacatggaggtaataaggaaatgatttatggcatagcta
atcacatttgacaagactgaaaaaatgacatgggtccttaa
>KolobokP-4_MeNe-I
tatagcaaaccttaatttttccaataatatgtgaaatagttcagaactgtataagtaacaaatgtactta
gtcctacttgtcaaaaacaattttatgaggtactaagctatgtcttatatgaatatgtatagaaatgtca
ttgagctgtttgaactcttgttttcatatttttgcttcactagaaaaatcatcttttgaaataaaatgaa
ttcttttacctatgagttgtgttttattcatagcagcacttatctaacttcactttggctatatgggaca
aatcacattttgtcatattatgtaatagcaaggtcaaaggtcaatgcactagttagtatgtacctgttct
actcttaaaaggacattttgatctaaaataaaggactttatatttgtctcataacacagaacttaacatc
agggacaggtggacatgacccaatcttaaaagagagtctaggctatcaacacctttcactgcctctaaat
gaatgaggtcaattaccccagtcctttcacacagttcagcctcccccatcccttctgtaacataaacaa
caaactttcctcccttaaatagtgactaatattcatgtaatgagccactctgtgatcataccttgtacaa
tacaacttgtaagcactttgttttggatttttgaataactttggggaatcaagaacaggaaaatgccaag
aaaaactaataagtctagcaattccaagggagttaaaaacaggaggacagtgtgtttccaaaagggacat
gtaccttacaataaaggaatcacatatgaaaacaacaatgaacagaaaacagagaacagaaagtttatta
gacttacattgaatcaatatgtggaggctgtaccaaaacataacaatgcaaatccattggcaccagtact
gctacacccccctaccagcatcaaaacttcaggtggaacagtgtgcatatccatctgaaataaaagggtaa
ataatttaaagcatacaaacacatgcaatttgtattggtcctacacattaagttaatattaattggcttc
aattaaaccggtgaaagaaataatttaaaaaacaaaacaaacaaacaaacagtaagctttgctctgacaa
attatgaaagcaattttactgtaatttgcataattaattagtgatactgtaatattgcattattaatctt
ctcattattgacataaaaagtactgaaaatgtaacttttacttcttgaaaagatatgttctatgaaggg
agataactgagtgagatacctacacatgtgtagtaattttctttccatgtcttaattctccatgtaatac

aagtcagttcacataaagcgatttaagttatatgcattaaatcaaagaaaaatacaatattagaatgaga
gtgcttacataatcagagatctatacatttatctttatataagtatagcagagagcctgagggtagttat
tttttaatttttttgtcagaaatgaggagtcatccactaatcgcatcataaatgtgcaccggaatgaagt
aatgtggaacaccgtcttccgggaacatgcgcaaatatccccaaagtgtttggggtttttgcaatgggac
caactgtcagaggaaaaatggggccttggttggagggaacgggcaatatgtaacagatgcatttataaat
cagaaatgttcaatctctttgaagaaatttataatcaaaatcctggacgcaaagcagccaatataaatag
agggctacaagtggggctaacacaagtgtccatgggaaatgctggcctaaggaaattactcttgtctgcc
agtattcctgccccttccacaaaggggatgcaaaaggtttcaaacaaaatttgtaaagagatcatacaag
aaaatattttggatatgagaagtcgaagacaaaacctaagagaaattaatatcgcaaggggaaatccacc
tgatatcattgatgtacaaggagatggttcatataataatcctttgtattctggtgttggaaaaactcca
tttcagccagcaacacaagtctgctacttacaggctgaaaatgtaacatcaaagaatgacattattgcct
taacaactaaaaataaattgtgttctcatcacatacaacaccaatcagatgaacttaatatgacaaacaa
gacctgtgactgcactgcaaatataagtatggaaactaatattggtgatgaggaaacttgggcctcggaa
tgtcttctcaacctaaaagaagataattttgaagtgcgaaacataacaactgatccagatacaagcagct
atagggctgcagtcaacctttacaatgaggggctcactcagactatccctaaaaactttatcgacaccag
acatttctctgaaaaccataggaaatatataaaaaggtgcccaaatttggttcacatgatgccagggctt
accaaagctgctagacaaaaaatgcgtgacaggtttgccattgacctgactcagcggtgtcaggtggagt
ttgaaaatgcatttaataaacttaacggggatactgacagagtcaagggttgcctatcatacagtgtaga
tgctattgtacagtgttaccagggagatcatagtctgtgtactgataattcatatgcatgcaaaggtaac
tggttggagaaaagtccatacttgccatttagttttaaagtgcatgtaacaaaacagtctgagggcatat
tgcgggattgtattaattatcgtttgggaccattaaatttggaaaaaacaaaatttaacactaatactca
aaaggttgaggcagcaaacagggttttaaggcgttcattgccaagaaatataacatggacaagaaatttt
cctggtagagcacacagtgcagtgcactctttaaataatgggcctggggaatccatactaaaactatgca
atgcagttggttgtagtatttcatctggaactagggttgctcaaacactagctcaggagcagaaactttt
tacacgatataaagcttacaaaaagtcaaaggcatttaaaagaagaagatgtgagaaaagaacttatctg
tataaattatatgaagaatttcaagagaaaaaaaaattacaagaaagggttattgttgccaaatgttaaa
aagaaaaggggaaaagaagaccatatatataataaattaccaaagatcacttaatccagcatttcctata
tgacacaaagcggtctgtgacaaccggaacaccttccttgagccctacagtgttctctatcctggtttat
ggcatgttgttctagaactagatgtttactatttatacatgatttattatgacatatatgtgaaatatct
aataggtgaccacaatcatccatacgtggtatattgtgtttaaataaattattttgtcgcatgtatgaca
atctatgagcagactccactgatactgagccatctggccatacaactctctgtctgccatagccatcttt
atcaaatgccccccctccaaagccagcatgtgatttttttctgaagattttatacattttctatgtaaaagt
ctgtcaatattagaccaaaacttctcaatatccattgttttaccaaatgacactgtgacagtttaaaaat
ggcgcctaagtatacttgttactatggacacactgtgaagctggtattacctttta
>KolobokP-1_LG-I
tatatatgtagctaattttttttttcttgctttaatttgttctataacatctgtggaacaacctgacccaa
aattatgcaaataaacctaaaagtaaaccagtggtagtgtttttacttttgccccctctttcctataata
agctatatatgcactgtataagcttgtatacgtacaattctgaattggtcagaactttttttatttatgaa
gataattcaattctgtaaaaagattaaggaacgttgaccaaaaccaagcctgaaaaacataaattacaca
gaataagggtcaaatttattacgtcacatttacttcgcacataattcagcctgtttctacttcaaaaacc
acagtacttcttaaattaatgacaattgcagaaatagaaataacatggaaccaaatgttttcaagaatga
atcttaaaagaccatatatccattaggtagtgtgttaaagttacaatcattacataactcattaaggttt
atgatccctaaaccccagaaaccaaaatcagtccacatagtaaatagaaataaatgatttctctgtctat
tcattctgtcttcaatatatcattctgctgatattttcacctccctcaagtctcaaaataatatatattt
agcaaaaatgagaaattcactcatttccattcaaaactctcaatatgaaaggcgacagaaaacgaaaaag
ttcacattttttacctggttatgtaaagaaagttagtgaggtgacacaaactgactacgactatgatctt
ccatctacctctgcagtgtatgaagaccaagaattgagctatgtgaatattgaactagactctccaggta
gtcacagggttttacgaccaaggcctgaaaaaaaaagaaagcagttttgatcaaaatgaaatctcaggca
acaggataatcaatgtggaaaaaatggtagaaatgtggaatatagccattcagactcatgcagacgaaga
ggccgaggtgtgtgcagtaccacaatttaagatcaacacagaagaaaaatggggaattggatggaaatat
tcactgaaatgcctcaattgtggatacatctctcctgtggtaaaattatacaaggaaatcacgactggca
aaagaggtaggaaagcggctgcagtaaacatcaacttacaagctggattgctagacatgcctttaggcaa
ttcaaggactaggcttctactggcagatttagacattcccccaccagctgaaagtggtatgtccaagtta
tccagttatgtgagttcaaagacaactgacttgaacaatgaaagcatgagaactaaggtggaagaggtga
aaaatgtcaatagacgaagaggtgcttccaaccctaacgtgataaatgtggctttggatggccggtacaa
ttgtctgaccataggacacagcaagaaacccggacaaggagcatcccaatcaatcggcattgcttgtgag
acaaacacagataagaaatatatcataagtgctgtgttgcaaaataaactttgttggactggagcgtggc
taagaaacacaggcattactgtcaactgtccaggacacgagggatgcacagcaaatctgacaagacatgc
acctttatctgagagagaaatggggagaataattggtgaagatttatctattcaaggggtgctcatcaag
tttgccacaacagacggagattcaacgtcagctaccggaattgaagaggcaatgagaactttacatccta
tgtggaaagttgaaagactagctgacccagcccatcttagttcatctcagtttcgccaatgttttaaggc
taattttagcgacggaatgttcagaggcaaaacaaaagttgaaaaaacaaatcaaaagaaaactttgagc
caggacataaaatctaggtgtagccttgtgatcaaagagatgtactctacttactgcggagatgtggaca
aactgaaaaaacagctaccgaaaatactacaagcaactgttatgtgctacgatggagactgttccaagtg
ccgttccaactctgtggtctgtagtggcggtcacagcaacaactggtggaatcgatccatgtttctggct
tcaaacaacttaacgagcctggaaatgaatgacaatgataagtttctgttaaatgaacttttgaaaataa
gattaagtgaggaagccattgacaaaacccgttataagactgacactcaaaaatgtgagtcaataaatcg
atccataaacatttcgcttccaaagagtgtcaaatatggaagaaatgctatggggcggctatcatcgact
attcttcgatcaaatattgggataaaggaggcaacacagacaaaagcagagtttttaggggcaaaattgt
cccctaagagtttacaagcactaagcagtattcagaaaaaatcagtgttccatagagattatgaaacaag
accagaagttaagagaagaaagcttctcgctcgaggaagaaatatccatgaatttgcaaaatataaaaaa
ttcaattcaactgacagtgggtacagaaaaggacaattagacaaaattttacctagtgctttgcagcagt
tggatgatgatcattcttattcacattcgaaatgatatttaaatgtataatttgccttgaaatgaaaaaa
gaaaagaaaawtcagtctttttcataataacgtgttctcttttatactacaactctamaatctggcaaca
gaagtgtcaaagccagcactaaactaaccctaaccctacttttttgaaaaatatcaattttaaaaaataaa
tgaccttaccagaca
>KolobokP-1B_LG-I

tatatatgtagctaatttttttttcttgctttaatttgttctataacatctgtggaacaacctgaccmaa
aattatgcaaataaacctaaaagtaaaccagtggtagtgtttttacttttgccccctctttcctataata
agctatatatgcactgtataagcttgtatacgtacaattctgaattggtcagaactttttttatttatgaa
gataattcaattctgtaaaaagattaaggaacgttgaccaaaaccaagcctgaaaaacataaattacaca
gaataagggtcaaatttattacgtcacatttacttcgcacataattcagcctgtttctacttcaaaaacc
acagtacttcttaaattaatgacaattgcagaaatagaaataacatggaaccaaatgttttcaagaatga
atcttaaaagaccatatatccattaggtagtgtgttaaagttacaatcattacataactcattaaggttt
atgatccctaaaccccagaaaccaaaatcagtccacatagtaaatagaaataaatgatttctctgtctat
tcattctgtcttcaatatatcattctgctgatattttcacctccctcaagtctcaaaataatatatattt
agcaaaaatgagaaattcactcatttccattcaaaactctcaatatgaaaggcgacagaaaacgaaaaag
ttcacatttttttacctggttatgtaaagaaagttagtgaggtgacacaaactgactacgactatgatctt
ccatctacctctgcagtgtatgaagaccaagaattgagctatgtgaatattgaactagactctccaggta
gtcacagggttttacgaccaaggcctgaaaaaaaaagaaagcagttttgatcaaaatgaaatctcaggcaa
caggataatcaatgtggaaaaaatggtagaaatgtggaatatagccattcagactcatgcagacgaagag
gccgaggtgtgtgcagtaccacaatttaagatcaacacagaagaaaaatggggaattggatggaaatatt
cactgaaatgcctcaattgtggatacatctctcctgtggtaaaattatacaaggaaatcacgactggcaa
aagaggtaggaaagcggctgcagtaaacatcaacttacaagctggattgctagacatgcctttaggcaat
tcaaggactaggcttctactggcagatttagacattcccccaccagctgaaagtggtatgtccaagttat
ccagttatgtgagttcaaagacaactgacttgaacaatgaaagcatgagaactaaggtggaagaggtgaa
aaatgtcaatagacgaagaggtgcttccaaccctaacgtgataaatgtggctttggatggccggtacaat
tgtctgaccataggacacagcaagaaacccggacaaggagcatcccaatcaatcggcattgcttgtgaga
caaacacagataagaaatatatcataagtgctgtgttgcaaaataaactttgttggactggagcgtggct
aagaaacacaggcattactgtcaactgtccaggacacgagggatgcacagcaaatctgacaagacatgca
cctttatctgagagagaaatggggagaataattggtgaagatttatctattcaaggggtgctcatcaagt
ttgccacaacagacggagattcaacgtcagctaccggaattgaagaggcaatgagaactttacatcctat
gtggaaagttgaaagactagctgacccagcccatcttagttcatctcagtttcgccaatgttttaaggct
aattttagcgacggaatgttcagaggcaaaacaaaagttgaaaaaacaaatcaaaagaaaactttgagcc
aggacataaaatctaggtgtagccttgtgatcaaagagatgtactctacttactgcggagatgtggacaa
actgaaaaaacagctaccgaaaatactacaagcaactgttatgtgctacgatggagactgttccaagtgc
cgttccaactctgtggtctgtagtggcggtcacagcaacaactggtggaatcgatccatgtttctggctt
caaacaacttaacgagcctggaaatgaatgacaatgataagtttctgttaaatgaacttttgaaaataag
attaagtgaggaagccattgacaaaacccgttataagactgacactcaaaaatgtgagtcaataaatcga
tccataaacatttcgcttccaaagagtgtcaaatatggaagaaatgctatggggcggctatcatcgacta
ttcttcgatcaaatattgggataaaggaggcaacacagacaaaagcagagtttttaggggcaaaattgtc
ccctaagagtttacaagcactaagcagtattcagaaaaaatcagtgttccatagagattatgaaacaaga
ccagaagttaagagaagaaagcttctcgctcgaggaagaaatatccatgaatttgcaaaatataaaaaat
tcaattcaactgacagtgggtacagaaaaggacaattagacaaaatttacctagtgctttgcagcagtt
ggatgatgatcattcttattcacattcgmaatgatatttaaatgtataatttttgccttgaaatgaaaaa
gaaaagaaaaatcagtctttttcataataacgtgttctcttttatactacaactccaaaatctggcaaca
gaagtgtcaaagccaacactaaactgccccaacccccccccccccccctcactcgttttggcattacaaa
atattcataaacatgaaagccaatctatgcacattcatggtctttcttctatgactgatgggacattgaa
tagatattaccccatacttcccatttgatgacaaaaattaagccccctgccatatcctgcaagaggaatt
tatagccaaaaaagatgaattttgcagcctggcactagtgcatgtgttaaaggcatccattttgtgtgaa
aacatgtgaagaaaaacaaataattacaattttgatacattgctgcaaagttgacaggtcgaaatttggc
tgaaatggcgtttttcaataaaagtggccaaaattcaaattttggacaaaaaaaatatgcaaatgtagcca
taaaaagtaatttagtctttattctacctattctgaaaatttcatacaaaatcattcacaactttttgaa
aaatatcaattttaaaaaataaatgaccttaccagaca
>KolobokP-1_PatPel-I
tatatagaatcaatttctctgttttcttgagcattaagtctgaattataaataaaaacatatttggagaa
aaattatcacaggatttttaaaaaaataaaaatttagatgatcagttgcctttttgctgatgggcacatt
taagctatttaagtgcggaaaactggagttagatttacaaaacaaaaaacttcaattaagcataactttt
ttatttttgataataaagttattctgtaaactgattttgcaattacaatgtaaaaagaatctagatgggt
aatattttaagaaaaatatctgaaatattttttttttcaagtttttcaaaaaaacaccactgatttca
tgtcgttcaaggaagaaatcacaggatatgtacaatttaaaatttgatggaacatatatggctattattt
tcagaaatgtctttcagtgggcaacattagcacaggccaattagatttagataatgctattatgtaacac
agcaacaggtttattatgcccacgggtcttctcgatatggaatcctacaaatggctgaccaatcacaggt
ctattttcttttcaataaaaagaaatgatttattctttctgtgtcgtcttgagtttccctggctaagaga
gaggcagaaatgcaaacacaagtgtctcttcaccatttttctaaaaatgaagggggagagaaagcgcca
ggctggacgttttctaagtggtcacaaggttctcttcaaaaggcagtgcttacaggcaacatctgggaca
cagactgactacaatgttccatctacatctaaatgtcacacggaagctgctgggttcaatatacatccat
tatttgaccaagacagccctggtagtcatcgaaaactgagaccaagatctgaaaaaaagggagacaatta
tcatattgaggctttaccaatcgaaggcaataggattgtagatattgtcaaaatgttggaaatgtggaat
tctgtcataagagggcatgtcaagcagaagccaaaaagctgttcgttgccaaagtttgtgttggctacgg
aggagaaatggggagcagcttggaaatgctcggtcaagtgtacgaaatgtggattcatttcccctgtcca
caaattatactcagaagttcataccggcaaaaggggtcccaagcctgcgtctgtcaatcttaccctacag
tctggtctcatggatacaccaataagtaacagcagggccaggctacttatggctgatttagatattcctc
ctccagccaggaattccatgcagaaactcagcaattacgtcaacaccaaaaccacacggttaaacacatt
aagtatgagggaaaagattaatgaagtcaaatcaattaatgcaagaggaggagccgtagacccgaatgtc
ataaatgttgcagtagatggcaggtacaattgtataacaattgggcattccaaaaagcctggacagggtg
cgtctcaagcaattgcactggcttgcgaaacaaataccgaacataagtatattctcgccgctgtcatgca
gaataaactgtgttggatcggggcctggatgaaagggaaaggcatgcaagtggaatgtcctggcggacat
gaaggctgcactgcaaatttagtgacagaagcccctttgtccgagctggacatgggtaaagcacttggta
cagattttgcacttcaaaatgttctaatcaagtttgccacaacagatggagacggcagagcagctcaagg
catagaagaaagccttcaacacctacatcctatgtggaaagttgaacgacttgcggatccaacacatctg
gctgcttcccagtttaggcaatgcagcaatgcgaaattcagtgaaaatatgttcccaggaaaaacaaagc
tcgaaaagggaaagtttaaaaaaatcttaagtcaggatgtaaaagcgagatgcagccttatcgtcaaaaa
tttattgaaggaccactgtggaaatgttcaagaattgaagaaacgcttacccaaaatacttcaagctacc

attatgtgctatgatggagactgctcaaaatgcaaagttcactctgtcgtttgtaatggtaatctgagga
gtaactggtggataagatccatgttcctagctgctaacaaatttaccggcctgcaaatggaagataatga
taaattcatattgaatgaaatattaaagataagactgagtgaagaagcaataataaaaacacgcttcaac
acaaacacacaaaaatgtgaagctctcaatcggtctctcagtgtttctctcccaaagaatgtgaattatg
gaagaaatgcaatgggccgattatcttccaccattctccgcaacaacattgggataaagcgctccacaga
agggaaagctaactatctgggagctaagctatcttctaaaactagacactctcttacccgaatggagaat
gaacgcaagtactattcacaatacaaaaacagaccagctgtgaagaagcgaaacctatttactcgaggaa
gaaaactgtattcatttgcaaagtacaagcagagtcattccgtagaatcgggttataagaaaggccagct
agattttcacagtagggacatgacagacaattctctggaagatcatagttactctcagtaaacattttt
gacaacaacctaatcttaatcataacctcaaacaaatgacatacatgtcaccccccccccaccaatacc
ttatttcaaacagtacacaaaaccacaattaaacacaaatgcagcagattccctttaaatacctaaaaca
aaaacaaaaaaacatggtcggaaacaagggaggcctccacgcggcttcgactggaaataggaatatttt
tgaatatttctagccaacatccctttaattaaaatctgaaatataaaaaaaaacggctaagccttggagc
tctagccttataattgtaaatgtagcagacagtctctgtattcataatggccatggcagaaaccacatgt
tttacagtgtttcctgttattgttaatgtaatggggttcaagactgatgtgcgatgtttcgatacacaaa
cagttatgacacaaatgggagcaatctagttttgatgacaattcaaaattcataaccacagacatcatga
aagacagtctgtgaaccttagttttagaccaaaggttggtaattgggttcttggtgctaattataccata
cttcccatttgtggttctagccccttgccatatacgacacacactatttgcagcaatggttgaacgattt
tgtaatttctcgtaataaaactggtatatttcagccattttttaaaaagaaaaggcttcatatgacgtaat
aataatattaacaaaattttaatacaatgttgcataccacatcaacacctgctcaatgttgtatgtaaac
aacatgtaaaattcgaggcctcgactcccaaaaaaatgcacatgagagaaaaaaaattaaaaaaagcataa
atgctggcaaaaaagtaattcataatgttcactctacctgttctgaaagtttaaggccaaaatattcaca
aatcttcacacagcagacaaatcaaaatttccgccattgccaaatata
>KolobokP-3_PatPel-I
tatatacctcacatgttcttgctgtttgaacaaatctgttgtattctaataaataaacacatatgcaatg
ccctggccaaagattatgaaaatccatgcattttcagaccagtggtgcagcattctccaaacactctaat
acagcagcaaactgcggattttcagaaataaatttaaaagcacatgaattttgatttccacagaacattt
ttatttttgaacataaatgcattctgtaaaaagataacagaaatgaaatataaatcaaacaagaaaatcc
tttaaaacagtaattatcatgcaatattagatttttaacattttcgtcgaaaagcacatagttttttttc
tgttaatgcagtttcaagtgccaaattgtggtcaaatctaagatgttgaaacatgcagtgcctataaatt
ttaaaatgtctcctatatagctgtatttacataggacaattaagttcagataatctgattatgtaacact
acaacagttttatgatccccacccccctagtatggaatctgatacacagctgaccaatcatagttctcact
gttcattctggcatgtcaaatgatttacaatggggatggtacctacaactttcttgaccaagagaaatgc
aaaacagtcaacatacaatcaatttatgttaccttccaatatgagaggccaaaaaaaaacgcaacgctttc
tttttcaagctcggaaatcaagtcaaccccaaaaagcaacgtttacaggggtcctcaactgcaacccaaa
ccgattacgatttgccctcaacttccacaggtggcacagagatcagggatgaaattaatatgcatcatct
ttctactcaatccagtccggaaagtcatcgggttttgagaagccgacctgaaatgttaaaattggatgag
actgagaattctgaggcaaatgctatccagggaaacaggattatagacatccagaaaatgttcgaaatgt
ggaattctgtcattcggttacatacccaggggcagaggaaggcctgtgcagtaccaaatttccagcctac
tagtgagcagaagtggggagcgggttggatatactctgtaaagtgtacaaaatgtacatttatctcacct
gtctacaagttgtataaagagattgcaactggtaaaccaggaagaaaggcagcagctattaatctgaccc
tacaatctggactgttagacatgccagttggaaacaccagagctcggctgctgttgacagatttggatat
tccaccgccctctcggagtggcatgcagactctttctaaccaagtcagtgctaaaatgacaaggttgaac
actatgaacatgagggaaaaaatagaagcagttaaaactttaaatcagaaggcaggggcagtaaacccaa
acatcataaatgttgcagtagatgggcgctacaacagcataaccataggccacagtaaaaagccagggca
agcagcctcgcaagccattggaattgcatgtgagacaaatacaaagcataaatatatcctgagtgctgtg
atgcaaaataaactgtgctggcaaggtgcctggctcaagggaaagggttttacagtgaactgccctgggg
ggcacgaaggctgtactgcaaatttaccagtccacgcaccattatctgagtatgatatggggaaaagcat
tggaacagaactagctctccaaaacgtcctcatcaaatatgcaacaacagatggagacggccgatcagct
gctggaattgaagacagtttaaaattttttgcatccgatgtggagtgtggagcgactagctgacccttcgc
acctggctgccactcaatttagacattgctataatgctaaattcagtgacgagatgtttcctggtaaaac
cagactggataagggcaaggtaaaaaaaaatactgagccaagatgtaaaggctagatgcagtcttatcatg
agtaccttgttcgaaaaccactgtggtaatactgaggagatgagaaaacgcctaccaaaaatcctagaat
ccactatcatgtgttacgatggtgactgctcaaagtgccgccctcattctgttgtgtgccggggtgggat
aacaaataactggtggaaccgttccatgtatctggcctcaaataaagttgattccctgaatatgaatgaa
aatgacaaatcaattctaagtgaaattctaaaaatccgaataagtgagtcagctttagaaaaaaacgaaat
tccaaacaaacacacagaaatgtgaggcaatcaatcgctcgttgagtgtttcacttccaaagaatgtcaa
ttacggaagaaatgcgatgggtcgactttcctcaacaatattgagaagcaatgatggaataaagagtgcc
acagagcagaaagctaaactccttggggcccctctttctccaaagacaagatattccttgcggcaaatag
aaagagaaagtctgtaccacagagaataccaaaccagacctgaagtgcgaagacaagctcttctgctgcg
cggaagaaagctttacgatttctgtgagtacaagaattaccgtgcagttgaatccggctacaaaaaaggc
catttagaccagccatcaaaagcagacgactcagaccagccatcaaaagcagacgacttagaccagccat
caacagcagacgacactttacaggatcacagctatagtaaataagcaagccattgtcatataggcctacc
caccttttgccttaatacttgtgttgaggtcaatcattccactcttgcacagcacatcacagtgtcagta
acctgaaatgatacaaagatgtcaataaatatgcaataaaaaataaaaagtaagaaaataataataaaat
aaaaaaaaaatgtggttgaataaaagggagacctcctcgcggtttcaactggaaatagaaataatatgcc
tctttatttctagctaacatccctgtaattaattaacaatcaatcgatcaataaaaaaagtaaatctaag
gcagcctcaaatgtaggagacacctgctgtagttgccatggccaaagcaatggccattggatttacagta
ctgtctgttgttgttgatatggtgcggttctaaactaatgtggctggcattcgtgcacagactgttctgg
cagacatgtgaacaatccatgtctgagggcaaatcaaaatttaatgacgtggtcaccataaaggacagtc
tgtggacaccggtggacttccaaaccccctgttagtgggcacttaaaagacattctcccatatctcgaatc
agccgacttcaaaccctgccatattctgcattccccattttcactcaataaagatgaattttctatttta
tcataaatgcgacggtagaaatatgccattttgaaaagaacacgtgccactagttatctagaaacattgt
aatacaattcagtgttttgatgacgtacatcatgcaacaaaatgtaaacattaaatatgacgactaaggc
cttccccaaattcacactctgcaaacggaaacccaaaataatggctaaaaaatgccttctgagcaataaa
aaaacagtattcttacctttctgacacttctaattgaacaattaatgaaataagtggttcagaacaaat
ctctttcggccatatttagtatcataa

>KolobokP-N1_PatPel-I
tatatggttttaaaagttgatatatcatcaaaattcttttctttatagcttcacatttatttttgaaaaa
ttattataatacagaattaaaaaaaaaaaattacagaaaaaatggtgaccctttaaagttatgtactgtt
ttgtataccaggttgcagcccaagtctatgggccttttgttcaagaattgaagattctttcttaattttg
tcaaaatgtaagttctgcaaaaagatttaaagagtttcaaacaagtctcatcaacaatattcaaaatatt
gggatatttttcaaaaattgtatttttttttacctttctacaaaatggcccatttttttacactttgtat
gtctcaaaatgccaatatttcccaatttcttggaaaatctgaggtaaaaggcatttatttcaagaaacct
tggccaatatacaagaagacaagctgcaaattggttgcattgaaggtaactgtatggaactataatgaaa
cttgtaagcccacccctgaaaaatgtttctgtggtaccaaatccaacttgtgtccacataagtactactg
ttgatggaatatggctaaaagccatgttcttctatccctggcttatatacatacacacaaacacatgtac
ttgtaagaagtatgaataatttattaaaatatggattcataaatgtaaaagaaatgctgtattcaggtga
taaccagccactctaataaagggtcacaaaacactcttttttttttaaaaaatgggaatatttcagaatt
taaaacattttaacaatcagaggtatccttgaaattacatagtgtaaaaattttgtggaatcatgttgtt
ttaattttcaaatcaaattcctgacaaaatgaggtggaaaaagtgagttttgacataattcatagcataa
aaggagtatgaaaaagctattaattaatattttattgttctgtaaatgccacattttaaaaaatcaccac
aaataatagatttacacccaaaatgtaaaacattaagctgcaaaagacaaatctacaacacatcaatttt
ggggtgtggactcatttggaaaaactgtcataggattacatgtttggccataaacaaaaagtcttagaaa
tccttgaaatcttatatgtaactatttgtttgtgcaaaataaaatgcccggttacaattcaagcacaaaa
tccaattactgtaaatttggagtcaatatatgttcaattggatatccaaatatcatgttcaagaaaaggg
gtggggtggggagggtgagccagtctgaatgaaatgaagacagagaaatttccctgcaaacacatagatt
ttattttctcgagaagggaaatgcaacaatttcaggttttaaaagattaaaattgtgttaagtcagagga
acactgcagcaaaatgcagttcactcatgatttggaaattctaacatacctcagcagtacataattagac
caaatttcttaattttatatttctgacaaacacatggatatttttttcaatatctgacaaacacatgaat
catttccaatttatttaacaaaggaaataaaaacatgcaacacaatttgttgcattttacaacagatgaa
taattaaaattttgaccgcagtattgggggtaatctgaagaatttcaggcaaattttcatgattttttagg
caaaattgagtgaaattattgtactttcttccattctaacatttttgatgcataaaaagtaaaaattata
cattaaaatatttttcactaattttgaaaagtaaataaacactgaaaatcaccaaatttggactaaaatc
aaaaatgttcctcacaaatctgtcaaatattttttgaaattttaccatttcactttttttacactcaaaat
gccatatatcacattttctggcaatttagagatcatatcatcaaattcaggcctttcgtctactttcagg
ttgtttacatata
>KolobokP-N2_PatPel-I
tatatggttttaaatgttcatatatcatcaaaattcttttctttatagcttcacatttatttttgcaaaa
ttattataatacagaattaaaaaaaaaaattacagaagaaatggtgtccctttaaagttatgtactgtttt
gtataccaggttgcaggtcatgcccatgggccttttgttctagaattgaagattctttcttaattttgtc
aaaatgtaagttctgcaaaaagatttaaagagtttcaaacaagtcccatcaaaaatattcaaaatattgg
aatatttttcaaaaattgtatttttttttaccttttctacaaaatggcccatttttttacactttgtatg
tctcaaaatgccaatatttccctatttcttggaaagtctgaggtgaaaggcatttatttcaagaaacctt
ggccaatctaaaagaaaacaagctgcaaattggttgcattgaaggtaactgtatggaactataatgaaac
ttgtaagcccacccccgaaaaatgttgctgtggtaccaaatccaacttgtgtccacataagtaatactgt
tgatggaatatggctaatagccatgctcttctatccctggcttgtatacatacacacaaacacatgtact
tgtaagaagtatgaatcatttttttaaaatatggattcataaatgtgaaagaaatgctgtattcaggggat
aaccagccactctaataaagggtcacaaaacacttttctttctcaaaaaatgggaatatttcagcattt
aaataattttgacaatcagaggtatccttgaaattacatagtgtaaawattttgtggaagcatgttgttt
taatttccaaatcaaattcctgacaaaatgaggtggaaatggggagttttgacataattcttagcataaa
aggagtatgaaaaggctaataattaatattctattttctgcaagtgccacattttaaaagatcaccacaa
wtaatagatttatacccaaaatgtaaaacattaagctgcawaagacaaatctacaacacatcaattttgg
ggtgtggactcatttggaaaaactgtcataggattacatgtttggccataaacaaaawgtcttagaaatc
cttgaaatcttatatgtaactatttgcttgtggaaaataaaatgcccggttacaattcaagcacaaaatc
caattactgtmaatctggagtcaatatatgttcaattggatatccaaatatcatgttccagaaaaggggt
ggggtggggtggggagggtgagccagtctgaatgaaatgaagacagagaaatttccctgcaaacacatag
attttattttctcgagaagggaaatgcaacaatttcaggttttaaaagattaaagttgtgttaaggaaca
cagcagcaamatgcacttcactcawggttkggaaattctaacatacctcagcagtacatwattagactaa
atttcwtmattttttagatttatgacaaacacatggataccttttcaatatcttccaagttatttaacaag
ggaaawaaaawgcaacacaatttgttgcattttacaacagatgaataattaaaattttgacagcagtatt
gggggtaatctgcagattttcaggcaaattttcatgatttttaggcaaaatcgagtgaaattatcgtact
ttcttcccgtctaacatttatggtgcataaaaagtaaaaattatacattaaaatattttccactaatttt
caaaagtaaataaacactgaaaatcacmaaatttkgactaaaatcacaaaatttcctcacaaatttgtca
aatatttttgaaattttaccatttcacttttwttattatcaaaatgtcatatatcacattttctggcaat
ttagagatcatatcaaatccaggcctttcgtccactgtcagattgttgacatata
>KolobokP-N3_PatPel-I
tatataggggaggaacttcaggtatcaccaaagggccctttttatttgcttcctatgtgttctggcaaca
tgccsswgaaaggaaatcaaaatmaaaaaataactgcaaaaatgacggccctttaagtatacatgctgct
ttatgtacagtggtgtatctgaggtccatgggccttttgtgctaaaatttagaaatctttctaattggag
gcagtatttgagttctgtgaaaaaaatagwcaagttgcaaacaaactacatagaaaacactcatgatttg
gatttttttttcaaaaaaatatttttttactgccawgtcatgaaaaatgtaccaaatttacatgwagcatc
ttacaaaataccaatagttttccactttttttaaatatccagactggcaagaaatatatktttagaatctg
tkgacaawatgcaaacagaaaaattgcaaatcagttgcattgattgtaatattagagatttatactgaaa
catgtatccctacccccctgaccatgccacagatagaatcaatctaatctcttggttgcagaattaacatt
atcaaaggaatctgtctaaagagccatgttttaccatccctggctaacatatgtgcatagawactaaaat
aggtgcaataaatatgaattatggtttaaaataggccttgatgaatgaaaaaaatgcaatatttagtcta
tgatcactcctccctaacaaatgggtcacagaatacacatttttcacaaatttgggaatatttcaatgtt
tccaacatctcaaacctcagaggcatcatacaaaktttatcgcaatccttgtgctgaagcaagatgtct
ggacctttacatcaatttcctgmaaaaatgaagtcaaaaaggtggttatgacagaattgacaacaaaaag
gaggcctkaaagggatcaaaatttacatattattgttctataagcaacatgaatktaacaatcaccacaa
atcctagattttgatccaaaaatattgaacattaagctgatwtttacagatatacaccagttttctacca
gggtgtggtctcattggcctgacatgtctcatggttctaagttggactttaaactaaaaaggttagaaat
ccttgaaatgtacaaaataacaatttattggtgcaaaataacattcctggccataattcaactacaaatt

ccaataactatcatttaattgtcaacacaggtgcaattgaatctctmaaaacatattaataaaaaagggt
ggggtggggcattttagcctctctaaaacagaatgatggagtaaaactgcccagaaagactgagatatt
gcttcctacatgaagggaatkctagaatttaatgtttcaaagaagaaaaaaatgctcattctgggtaaaa
tagtggcaaaawggagccaacccctattttgaacattttgtacatatccatctgctgtacattattaact
gagtgtgaccaaatttcatttttttgtaggaaagctggagttattttctaaacctgacaaacttatagat
cccatctaagttatattagcaatgaaaataagggtatttttagacattttgactcatttgagtagtcatg
taagcattaatttctacaccgaaatkggagaatctgtagattttctgtgaattttcctgatttttagaca
aaattaagtgaaattataatacttttcactttatctgtgctttgtgaggctgagtaagccaaaattggac
attwaaatgtttttctwactgttagaaattaaataaaagctgaaaatkgagaaatttgccttaaagtcac
aatwtttcgacaaaattcaataawtctttttttwaattttacatttttttaaaatttttctgtactcaaaat
atttatacatatttctgataatttagtcttcatattttcaaaactaaggcctgtcagtcaaagccttg
tcctacacagaca
>KolobokP-N4_PatPel-I
tatatacctcacatgttcttgctgtttgaacaaatctgtggttatctaataaacaaacatatatgcaatg
ccctggccaaagagtatgaaaatccatgcatttgcagatgcgcagtgcagcattctacaatcactatagt
gcatcagaaaactgcggaaattcwgatagaaatttgaaagcacgtaaattttgaattccacacaacatct
ttatttttgaacataaatgcattatgtaaaaagatgagataaattcaatataattccatcacggaaatac
tttaaaacagtaatcatgatgcaatattagactttttatcactttcatcgaaaaccacagtttttttctct
gttaacgtagctgcaaatgttaaattgtggccaaatttaagatgttgaaacaagcagtgcttatcaattt
taaaatatctcctatatagctgtatttccatgggataagtaagttgaaataatctgataatgtaacacta
caagagtttcttcatccccacccccccagtatggaatctggtacattgttgaccaatcatgggtctccctg
ttcactctggcctgtcaaatggtttacaagggcaatggtgcctacaactttcctgaccaagacatatgca
caaccgtcaacacacaagccatttatgttatcttctaatatgagaggccatgataaacacatgggtgtcc
tttatcgggctcggaaatgaagtcatcaccaaaaagcaaagtttacagaggtcctcggttggagcccaaa
ctgaattcgatttgccctccacttccaccacaggtgtcatagagatccgggatgaaattaatatgcattg
agatcatcgttctactgaagccagtccaggaagtcatcagggtttgcgatgtagacctgaaatgttaaat
ttggaagagagtgagaattctggggagaatactatccagggaaatggacctttgacacccagaaaatggt
caaaatgtgcaattctgtcattcagttacatgccaaggggagtagaaaggcctgtgcagtaccaaatccc
cccccccccgttagtgagcagaagtggggatcaggttggctgcacttcgaaaagtggtgtatgaaatcca
catttatctcacctgttttgcttcttttttttaaatacttgtgtttgctgaggtcaatcaatccactcta
tgaaatattacccatggcacctgaaatgatagakagctggggataaatatgcaataaaaaaacagtgaga
aaatacgaaagaaaaaaaaatggttgcaactggaaataaaaataaacctctttatttctagggaacatcc
aawcactggctctccaacaagaaaccagtaaacagtcagaaaagtcagtaaaaaaatgaaaaaaaaatga
gtgtcacacaagtctaagccaatctcaaaggcagataccatgtttttgaggttcagtctaaatattaagg
ccagctgcaggtatggtcaaaagtcacacgggagtgaattttggagtgtacatgttaacatttcattgta
tgatgtcaaaacatgtcatgtctaaatattaaggccaactgcagacttggttcaaggtcacatgtaaata
gggttgtggccacatgatttttttggcaagattgtgggttggggctagggtcaggactagctgtcaggact
agctgtcttggcataggcctacgccaaatgcttttttgacgagaatattcgattaaatcataaatgcgaca
gtagaaatatgcctttttgaaaaaaaaacacgtgccactagttaactagtactaaactagaaacattgta
atacaattcagtgttttgatgacgtacatgcaacaaaatgtaaacattaaggcctacggtccctgaattc
acactctatcaacggaaaccccaaataatggctattttgatatcagacagtcagaaaaatgccttgtgag
caataaaaaaacagtattcttacctttttctgacactccttattgaaaaattcatgaaataagtggtggag
aacaaatctctttcggccatatttagtatcataa
>KolobokP-1_GiAe-I
ttaaacaaaacctgtcatattcataaaacacaatttaggtgttttcatataatttacaccatttggagta
aaatggacccacacatgacccacataacacaaaagataatgaagttatggcccgaaaagaggccatgtac
aacattgttgtgacagtacattatatactcagatatgtcatacacaaaaaatatgtctaacattaattgt
ggctaatgttgcataataggtgttcttttttcatgtctaaataaaggtaatagcattcctaggtaagatt
caatgaatggaattgtcacatgacaagttcttagttactgttgccatgggtatccaggtactgtccatga
acaattggaaaatggcagctcccccaaaaaatgctagaaacactaaatttagcaggaaacgacgaaagaa
aggcacatcattgttccaaaagggcaattgtaagtggagaaaaacatcagagcagcctcagacaagggac
aagggacaggagaaggaacaaattcagcctatgaaaacagacacacagagagttaaacgcatcgagattg
agatttttcagtcagtcagtacatctggaaacacactgccatccaagttaagacccaagataaagacagt
ctcatgtaagtcagcacctaggcttaataattttactggaaatgtcatagtaaacattaataacttgcca
ctgcttgtaaacaagttttgttctattcactcggcacactcaaatgaatgcaaaagtccccattgtgttt
tacgttccatcaagtatgttggactatgtgtcagtgtttctctaggatgtcagagctgcaatatgaatat
caccatggacttaactgataaactgcccaaacctaaaaacaaaccaggtcctccaccatccactctgaat
aaggcattggcactgtcggtattaaagacgaaagctggtcccagtgacgtcagatacatcctgtcactac
tggacataaagcctccatccatgacacaagtacagaaggatttgaaactattaagcaatgaaacagtcca
cataaataatgtatcaatgaagaaaaatcaagagttcatcaacgaagttgccaagatgcttaaccaagta
acatccaattcgcgtggcctcattcccgtcgaagccgattgctcgtacaacaacaggtgccagagtggtt
acgaagctgggacccaggccttctgtccactggtggagcagctgaccaaaagaaagctggtgttaggctt
gtcaactgcaaataaattgtgtagaaaacatcaatgcagtcatacaaaatgtgcacgcacttataatcca
acacattcaatggcatctgtagaaaagaagttaatatcagaaaatattacaaatgtagagtcccaaaatg
ttgtaaaaatctcaagcctaacaactgatgcatgtgcacaattaggaaatataatgcaaacttcaaatcc
tatccactgttcctctcattccaatatgttccctcttaagtatgtgtgttttgtgcaccgaatgcgaaga
gtttttataaatacaaaagttgcagcactgaagcttcaatacaagcctataggtttaacaaaagatgctt
ttgcaaataaactggcagtgggaatgcggtgtcgactgactagagaactatcccaattacataaacaatg
tcatggggtcaaagcatttgtttctaagggtgcgtcaataagagacaacattgtgaactgtttctgtgga
gaacacacacattgtaaaacagcatcattagtgtgcaatgcaaaaagcagtaaatacttcaagccagtat
tcctaccaaatggacaatatctgcagttaaataatacagatagacatagcttaatgcacacactaaacaa
aactgtatcaaatataatgctttctcagttagtatgtttgtataataccaacaaatctgaaagtttacat
cacactgtgtttacatatgctcccaaaatgagcttatacagttgtaatttccatgggctatgtcattcag
cagtccacaccaatacccatggcagagggaattctacacttatcattgcaaaaaagataggtataaaata
ttccaattatggtccaatgtgtgactatatgaaaaaaactgatgagaaatttcaatatgactacaagagg
caaaacacacatagatataagcgtgacaggtatttttctctgctgaacaggtgtcacagaaactttaca
tgcactcttcatacagtagtggaagtaaacacataaataggcaacacaattatgccattaacttgaatca

ctggcaacagcaacagcaacaccagcattagcattagcaaactgaattatgaacacacagcaactgtaac
attaataaaactctttaacagtaaactgtaacagacaatgaaacataattcacatggggtaaataaaacc
ccaaaacacaactggaaatacaaaatgtaccttgaagttggaaaaacacagttcttatacacagaatgcc
caaaacatttattggcatagacacaactctgcctctgcatgtttatgcaagcaggttcataagaaagatg
atcaaaatttaagcacaatttattgtgacataaatgactaacatgtatttttggatcaagtttgtgcaag
ccacttttcataaaaaaaagaagcctgtgtgctcgaactcggaccctctgtgtccgaaaagtggcttgca
caatgggataaccatctttttcagtcgcggcacaccaaatgtggcattcaccacttatgtcaattttttgg
gtcaattatttcttgaaagtattgaatttcagtttctgttaataaatcattgaacactttagacattctg
taatatcctgatttaaaaaaaaaaaaggagaacaaataaatttgtttactaaaaagcacacctgtatttgt
tttatgttattagataaatgttaaaaaaagtgtatgtcaaaggatgattcatgtgagggagtgtgagggt
gagggtgagggtgagggtgagggtgagggtgagaccatgacagtgctcacactgaggtggaggattttgt
ttcacaataattaatgtgtcaagtacatgtatgtgtatgtgtatgtatgaccctttcctgtatcgccaga
acactagctctctcagggcgacagtaaatcatggcagtagtcacaacttctgtcttgtacagagatacat
tttttgtgtgataagggttttgtttacattttgtttcaagcaaggaggaataacccaaaaataagtgaag
attcactaatattatgatattgtgtaaaataaatgggtgaaactgtcaaatattgtaaaatataggttta
tttacattaacacaatatattacatattataatgtcattaaaacttaacattatatattattacaactat
actggtatttgtttaatgacatacatgtgctgctcatgtaaacactgacaacaacacatgaatatttaca
cagacatcatcacacgtctacatgtgctcatcgtcacatgtacacttgttacctaaacattaacctatca
acccttaatacaataggtaaaaacacaaaatataccatagatacacactcaaaattgtattcagaaaccc
ctacaaccacaatttccctcacaaacaaaaaaaacccactcactttcgtttcaaacagcacatgtgtaa
atagtgcaggttacattat
>KolobokP-2_GiAe-I
tataagcagaactgcactgtttcaaaaagtgtcgaatcacctgtttttggtgaatggcaggttcacatgt
tttataacataaagtattaagttgactgcaatgtcctatttggagtcaagcgcttatgaaagtgcatatg
acaatgttcgtaactacataataaaataatatgaaatgtaatgttgtgttcttcttttgtgtgttcaagg
agtacagttgaagataataacctatgtttgagtatgcatattatgtgtgatatacttgttaatcccagca
ctatgaatgcaccatgtcacctacatacagagactagtgaactattttctctcgtaacctaaaaatttga
aaattctgctacgggcaggtctcattaaccaacatttatcagaaagtagtccagtgccatgggtcttttc
aaatgtgccaagttgtacaagaccaagcattgcaggaatgtaaacaaagttaagaaacaagctttatcac
acctctaggtcagagkttactgttgtgaggagtcaaactgtcgaacaattaactcacagatgggtgtccc
tctcaacatgaatttaaatcactggtaattaggtcagctgcttaaactatattaaaagcaagcatgctgg
tacagtcatttcacaattgtcacttgactaaaaataatcacccattcctattagaatatttaacaaattt
taaaatgccgccagtgagaaaaagtgcaaaatgtggatttaagaaaggggacattccacacaataaatcc
aggaagtgtgcgaaagtggatacaggtgtgtacgaaatgagacggacggccagactaaccaagtccatga
caaaaaaagtcttggacactgaggctcatggtgtccaagttggacaattgccgacgacagcgccatacag
actattgcgatctgtggcagacccactgactgaaactgagcagtgtttgaacagcaccataggttcaagg
taaatgatgctaatgtacttaatctgaataccctacatattctaattgacttcttacaatagttcttaca
tgcatgtatcagggcagagataggttggaaaggggaaaaatgagaaaagagcaattaaattataccttt
tatatgaaagttacacattacataccttgtagcatgcaaatcacattttgttggataaatacactattt
atgtgattacaaacacaatcacaaagagtcagatttgaaaaatatggcttgcaaacaatgtcaacttggc
atggtagcactggtgaaaacagtccaacagtccaagggagataatctatcaatgaaaatgctagatacaa
gaaaacattaagaaatgaggtagtgctagtatttatgggcttctatttgggttttatcaacagatcagag
gccgacacatacagggtcctccacatgggcaaactggagcaaatgatgaatagaacacacaacgaccacc
gcactttggctccagaatgccatggtcaactgatgtgggacttcccggcggaagaaagaaggggtgtatg
cactcggcagcgtttgtgctgtaatatttgtacataccactctggtatgtacaacttgtacacagaggtg
gacaatccagagcgaaagggtcgaggtcgtaaggcagctcagccaaatgtgtcagtgcagatcggtctga
cgaaaatgcccattggggttcaaaacttccggctcctgtgtctctgcatgaacataccagcaccgtgtgt
tgcggggtgtcaaaaaactgcaaacaaagtatgtgacactattataaacagtaacaaaaccgacatgcag
gctaggagaaacaatcttaaaacaataaacactatcaggggttgtcaaccaaatgcagtaaatttacaat
gtgatggcatgtacaacaatccattatattctggggttggaaagactcctttccaacctgccactcaggt
ggtctacacagcaacagaacacaacacatcacagaagcaagtgttggctattgctacaaaaaataaatta
tgctccaaacgtcacggcattagtcaatcacatacatgtctgaatgatccaacatgctctcgcaatatcc
caatgacaatgtctattggagatgaaaaaacttgggctaaggactgtttaactgatttaaaaaatgacgg
tcttgacgtacattacatgacgacagacccggatgctaaaggtttcaaaggtgctgaagagttataccag
caaaatgttactgacatcgaaccacaaatgttaatcgacacacgacatgtttcggaaaatcatcgcaagt
ttgttaaaggtgcaaaatttgttgaaaatatgatgccaggacggacaatatctgacaaaaagaaattaca
gatcaaattctccaatgacattgcttttaggtgccaggcagagtatgaacaggcttatagaatacacaca
ggaaacattcctaaattcatgcatgcactgtcatacacatccgatgccatcgcagcctgttatcaaggca
atcacaaaaaatgtatgaaacattcatttgtctgcaaatctgcaagtaaaaactggctggttaagagtac
ctttctcaattcgcatttcaaaattgccactaacaatgacaccgacttgaggaagtgcatagactatcgt
ctcggacccaaaatgttacaaatgacaaaatttaatacaaacacacaaaaatctgaggctgtcaataaaa
gcattagaaaatcattaccaagacacacaactttcagcagaaacttctcagggagggcacacagtgcagt
gcacagcatcaataatgggcctgtagagtcattacttaagttgtcaagggatatgggatgttcattctca
tcgggtagtaaggtcatttcagctctgcacaaagtcgaacacaacagaactctgcaactaaatagaaaaa
aaaacaatgccatataaaattaaaagatgtcagaaaagaaaggttctgtatgcactgtatgaaaaacacc
aagaagatgaaaaatatgaaaaaaattacatcttaaaaaatcacgcttcgacctcgaaagtcgaacataa
ttacgcaaaatctaaaaaatatccccacaagttacaataacacacacaattaagaaaacaaccacagact
aacaatacctcacaacagacagtgtggtgtatgtccctcgagacacaggccctggtgtttgcaattaatt
ctatcaatgttatttgcatgcgtttctaatgtcaaatgttcggcatttatgcactgcctattgtgacaca
aatgagagatttcaagcactcctttatgaactgccatatcagatggccatttcttgtttcttatcatgta
tgccagtctgtgagctttctctattttgggtgcaggggtgttaggccaatgcactttctggcggccatat
ccatcagcattcatggaacccagccacatgctacatgcatgttcacctacattaaattcagtttttttgt
ctaaaagatattgtacatgttcaaaaaatgactccatgattactaattaattcaaaatggcagttacaca
tcaattaaaaaccggaatttatgataattaggacataccaaaaaatgtcattttctctata
>KolobokP-3_GiAe-I
tatattgaaaaagttccaatttcaaaaggtgtccaatcacatatttttaagaaggttattttcttgtgta
ctccagtatgaaatgatacagccagcagagtaagtggaactacagagcagcgtctttaatagtacctacc

aaaatggatccacagcataataaatatacatgggaatgaggtacatcatattttatccttgtctggtagg
tagcacagctgaataatataacacccatgtgggtttgtatactgtatagggtacatactccaaactgagc
tattcagaataccccacactagaagaactttacatggagatgctatgtactgagtgaaccacagaaatca
ggaaaataatcctcataacatcaaagtcgcacccaattagtgaccaaaccactgatgaggcagctgtaac
tagcacccaattaatcactggaagcagtgaaagactaatgtccattgtggcaaagtgtacaatatacaca
gggagaatgagtcaaaaatgaagtgacaacgtgtcgacattacgaaatctggctaatttggggggtgaca
aatgtgtttgcatgtatcaacacatgggaaataaattgccttcttttaatattactatccatgcaccagc
aaagtttgtcatttaatttctgaaatcagactgacaatcatcatagttttaagcaaaccattacacattg
attacaatgccagcagtgaaaaaatcattacaatttggtttcaagaaaggacatgtttcataccacaaga
acggaagaccagtcacaaggggggtggaccacatcaaaagtgagagagacagtcagacttacaaagtcaat
gactgaaagggtcttgcacaccagtgctcatggtgtacaggtgacacaagttcaggctgcggattcctgt
cggcttttgcgtggactgcggaataccccaacacagctggaactgtgttctgtggctcccgctggtgcaa
ggtaaacaattaccactgcatgacacattgattacagaaaataaaatcactcactgaacatacatgtagc
atattaaagccctttgagggctattttcaccaaaagttaactaaaatcattacacagaccacgttatctg
gcctaagaataaatcgctgaggtttttcgggcttaacatcaccaatttcagattaagtggataagtgtct
gcatgaaatttacatctgcaagtatcaaactatgataaaacatcaaagagtaggaatgttgcactataca
aaatacacactacagtgtaaggggagataatcttcatgaaaaactacatgagaataaatgcataaaagtg
tagggaatacagtaacagtggttacttacactgaagttaaatgagggtacctttgattaacaggcctgag
gcagactcctacaggctgattcacatgggcaagctggaggacatgatgaacagggtttggagagaacatg
ccagtgccgttcccaactgcaattcaggtcgactagaattcaactttgccgcagaagagcaaagaggtgt
ctgcacccgccagcaagtccactgcacatcatgcatgtacaaatcaccaacatttaatctgtacacagaa
acacagacacacacacggaggggcaggggcagacgacaagcacaacctaatgtcacagtacaagtaggcc
tcacaaagatgccaataagtacgaccaattttcggttgctgtgtacctgcttaaatatcccaccaccttc
acataagggctgtcagaaaaatagcaataaagtagctgacacaatcacacaagtaaatcaagaagactgt
atgccaaacgggaggaacttaaagtaattaacacagtcaggggtacacaatccaacattatcaacattca
atgtgacggcatgtataacaatgccctgtattctggcataggcaaaaccccgttccaacctgccacgcaa
gtggtatatactgctgtagaacacactacaaaagacaaaaaggttatagcaatttccacaaaaaaataaat
tatgttcaaaacggcacagtataaatgattcgcaaaaatgtgcggaaaatatgtcatgttcgtgtgacat
tcccatggcaatgtcgataggaaatgaaaaggaatgggctaaagattgtttactacaaattaaaagtgag
aacaatttagaagtacaatacctgacgacagatccggacacaaagggtttcaaagctgccactgaactgt
atcagaaaaaatgtcaccaaaaccgaaccgcattttcttctcgacaccaggcatctttctgaaaaccaaag
aaaatttgtcaaaaattctaaatcagtagaaacaatgatgccaggtagaacaaaaacagagagagttaaa
ttacaatctaggttttccaatgacattgccatgagatgtcaggctgaatatgatcaagcctttaaaatgc
atgcgggaaatatttctaaattcatgcgtgcaatatcctacactcgcgaggccattgtgtcctgctacca
gggcttacacgacaagtgtaaaagatactcacatgtctgtagaaatattacacacaactggatctctaag
agttgtttcctaaataataactttcaaatagacactacagaagacaacaccaacactttaagagcatgtg
tcgactaccgcctaggcccaaagatactgcaacacaccaaattcaacacgaatacacagaagtgtgaggc
cgtaaacagatgcctaagacattctttaccgagacacacaacattctctagaacttttgccggcagagct
cacagcgcagtacacagcatcaacaacgggcccgctgaatctgtaatacagctgtcacagcgtatgggtt
gcagcttctcacctggaagtaaagttgtaaaatctttttaaaaaatgcaagagtacaaatttggacagcg
gcttaaaaagaaatctattgagtataaaagaaaaagatgtcaaactagaaagtctctgtatgcactgtat
gagattcatcaggaacaacaaacatatcagaaaggcattatgctacaacaaagcaccaaacacaaaaaag
ccgagcataattatgccaaaaatattttttttcagacaaataaaaattaaatactaaaagaaaacattga
ttttaaaagcagttcacctcacagaagacagtgtggtacatgctgttggttacaaaagccttgcagtttg
cattcttttctaccattattagcaagcctggattccaaaaccaggtgacttgcatttacacaacagcgaa
catgacataaatggcttatttcaaaaatcccatggtacatagccatgtctgtgggaagtaatttgttctc
tgccatgtaggccagccgatgggccctctcaacttttgctcctggggaagaaggccacacaattctctgt
cttccatacccatcgggtgtgacacttccctgccagacagtgcatttgaattgtccaacattaaatttac
ttttcttatcaagcagatattttaaatggtgccaaaagtcatccattgtgaatactaatagactggtacc
agtatatgctaattaacagacaaacctgaatattaataacaaatttaattkttcatggaaattgctstat
a
>KolobokP-4_GiAe-I
ttaagaggggggtttggaatgggatagctagtcagttttgacacatgacatactatgacgcatacctagta
ggaaaacaccaaatttggtcatgtcttctaatatgcattaacgcaagaggccatgaagactggcccgggg
gttagcaacactgggctgaaataatagaaaattaagcttctcccacacctcaagttcacctgaccaaagtt
ataacatggaggtgacattccaaatgtttgctgttgtataatggggggtactgacagcagaaccttatga
aatggtcacattaaaattacaagaccatactgggtactggcatattttaatatgtcatatgcatgaactt
ttattaaaaatttaatttaaatattggatttggcaaattcaagtgagccacaaaaaggttattttgggac
agtatgagagggaagtatgcacactggcagctgtcaacgggacacatgggcaatttcaaactgggagtga
actgaagcctaaagtatctcatggctgagagaactgaccacaatcataaacaaattcaaaacagaacacg
catcagtttactgcatgagagtatttattaacataacacacaccacaggttccattccgctgaagactgc
tacatgctaagagatacaaaagttcacaactttttccttatactaagagaatataccacacccagtgtgct
aacaatgaagccaaagaaaaatccaacagaaaacctgcacacaagggtgcctttcaaaagggacaccaa
tgtctgttcaaaagaactataggtgttactaaaacaaagtgtcttaagcctgaaggtgacaacataccag
ttaccaagagacttagtcaagaagagttcaacgctgtggtacaccattgtgatagacagtcatacatcgt
caaggatgcggatggaattcccagtgacattagacttctccgaccaacacggatctcaactggaggtggt
acacaggcaggcgagttcctgaaagcaaaaatggacatgaatgattccaaaattatacactgtggtatgc
ttctgaagatgtttaatgacactaatgaagacccacagactagcttgtccagggtgcactggcaagtacac
atggaatctagacttgtgtcagaaatggggcctctgcacccgtgcaggactgagatgtcaaaagtgttca
ttcacaagtaaaaaaatacaaactgtatgaagagacaacacagaatactaggaggggaccaaaatcagcaa
agccaaatgtcggtgtacacgtaggactacagagcacatctgtcggtgttaccggtttccggggaataat
gctgtgtgctggagtcacacccccagctaccagcggaatgcagaatactgccaacgaagtgggacgagtg
acaaccagtctcaataaacttgacatgtccacacgcagagcagacttagtacaactgaacattaataggg
gattttcagctgaccatcccattgccatagaaggagactgccggtacaacaatcctctttcaagtgggtg
cggttcaacaccatttcaaccggctacacaagcaacttacactatggtggaaaatgagactggcaagaaa
caaataataggactttacacaggaaataaactctgcaaggctcgtgagcttcaagcagcgagagaaaata
gactaatactctgccccgatcatacaggtacctgtacagcaaatctccggcttgacgacacaataggaga

cgaagaacattctgcagctcagtgttcaagtgacttctcatcagacgtctctccactcacgattggattc
ttcacgactgatggcgattcccgtgccgtgtccggattacagtcatcccagagcaaaatatctaaagtga
agataaaaaatctgagagacacccgtcatttatccgaatcacaaaggaagtttacagaaagggtcaagtt
cagtgacaacatgttccctggaaacacaaaagcggaacgagaaaaggttaaaaaaagatttgctatcgac
ttgagacggcgctgccacagtgaatatacatcggcacacaaccaacttgccggcaacatgaacaagttag
taagcaaactgtcttacactgctacgagtatactatattgtgtgactgggaaatgtggagcaacatgcag
aaaacattcctttgtgtgctcaggggtgaaaaagggacgatcaaacatgacattcatgcctaccggtaca
aagttaaatgtcacggtagaggacgaggcactgattaaacaatgcatagacataagactgggtgtaaaag
ccattgagaagactaaactgaacacaaatactcaaaagtgtgagagcgtcaacagagcgtacagcagaac
aaatccaaaaactgttacataccatagaaatttcaccagccgcatacacagtgccgtgcatatgctgaac
agtgggttttcaagttcggtcattcaaagatgtacagctgtgggagctggggtacgtgacaaaacatcgg
tcatatgccacttgaagagcatcgagcggatagccaggagacatgcgagtcgtcaacgaacacagcaatt
ccaagccaggagggcatctctaagaagaaaaagatacaatatgtatgatagacttcatcggacaataaca
tattcaaaaaacttactcgaccccgaactgaactataactctgttggcaacaaactgtgaacactcggtg
aattctttccgatatatatatatatatatatatatatatatatatatatatatatatttgtatgtatgta
tttatttatttacctgaactattataaaatgcaatctggatatataccatggcctgaacatttctttgga
accatatttctacagtgctgcctttgattattgattacatgtggctctagacttaagtggtgtatattga
cacacaaactgtggtgacataagtgagaaacatcaaaagattttggtaaagtgactgatttgacatgaat
catgtatcgaagcctgtgaacattaattggcttgtgtttatcctgacctggcagtctaatattcatgaca
ccatatgccatatttttataatgtctgatatatccattccataatacacaattatttacattactctgtc
ttgtgtttctattaattttatgcaaagtggtggctatccacagtgcattcatgctatatttgcttaatta
ttatgctaattagtgctgattaaaatatccaccacacttcaaaaaacaaaaaatgtgtagcctagtgtgt
gccgatgctctgagaacacgaaagcacatggctggtgaagtttgcaccaattacaacatggtcacggccg
ggccggtgctatgcactggtaaaatagtgttta
>KolobokP-5_GiAe-I
tatactgctaccacacactttcagaaagtgtccagtgggctacatttgagaaggacagccttacatgtcc
taaaatatacaaacaaataattgatcagaattgttttaaatgcagggtgacagtttgaaatgtatatagg
acaatatttgtaaataaaaattaaacatgtgatatataacacatcttgccttttattttatktataatga
gtacaatttatcacactcatctcaatttagttgtgtgtactgtgtatcacttacatgttagaacaagcat
actgaatgcactacattacctcctaacacagaacagagtcgaatttactctcgtaacccagaaatgagaa
aaatcagcaaaaatggatcaaattaacctatattaatcaaacatttaatccagtgtcaaacatcacttct
cttgtgctgactcgtacaagaccaagtactgctggaatgcacactaaattgagaaacaatgtctgtttca
aacatgcctggcagattgttgtcatcagcaggataagtttgaaaacactgamccagtaatggctgcccct
gtccacattaattataatcactagtaattaggtcagctgctctaagtttatcagttgtaagcaggtaacc
ccagtcatttcactaagagtacttgattgaaaacaactgcatccatattgtcttaaaattaaagtaattt
aaaaatgccgccacttactacgaaaggaaaatgtggtttcaaaaagggatatctcccaaacaacaaacac
agaacttgtaggaaactaacaamacctaatgctaaaagtagcaggacagtcagactgtgcaaatcaatga
ctgaaaaggttatcaactgccatgcccatggagtacaagtgagtgaaatgtcgactggtgagccgtatcg
gcttctacgttccgtaccagacccaccaactgaattggaaaaaaattcaaagaaagtacccggctcaagg
taactgatttcatgccttagagtagacacactgcaaaatttgacatacctgatgtaatagtacttattta
cattctatgggtgaaaattgctaaaaaatgaacaaaatgatggggaaatttataccgaaatgtaacattt
tgcccaagaaattatttgtaaggcctgtcacatgcagtttgtttctttgtgacaactacactttctctgt
aaccataaacaaacacgcaacaagtgtcactcaaatatttgtcactgaagtcagattacagtaacatgct
actaatctagcaagtggccaaagggagataatctagtttgacaatgcaatgtacattaaaatacatttag
cactgcaggaaaaaaaagcwattttttttaatttttttatatttaacagacctgaagcaaacacctacagag
tactgcatatggggaggttggaggagcttattaacaccagtcaggtcaaacatgccgactcgtcccctaa
ttgctgcggtgtgtttcgcttcgactttgatagggaagaacggaggggcatctgtacacggttgaatgtt
ttctgtgagaaatgcagttacctctccaatacatttaccttgtatgacgaagtggagaaaactgaagtaa
gatccaggggggcggagggcagctcagccaaatgtgacagttcaggttgggctmacaaaaatgcccatagg
tgccgaaaacttccgtctattgtgtttgtgcatgaatatccctgcaccatcagccagtggatgccaaaga
actgcaaacaaagtttgtaaaaacatagtaacatcaaacactgcagacatgaaagccaggagagagacac
taagggacataaacaaattaaggggctccaaacctaacactgtcaacctacaatgcgatggcatgtacaa
caatccactgtactcaggggttggtaagacaccttatcaacctgcgacccaggtcgtgtatacagcagtc
gaacacaacacacacaaaaagcaggtgttagcaatttctaccaaaaacaaattatgctctaaacatcaca
acattagtacttctgaaacatgcctgcaagacccaacatgctctcgtaacatccccatgtcaatgtctat
tggagatgagaaaaaatgggctaaggactgtttggcacaattaaaaaatgattctctcgaagtacaatac
atgacaacggacccagatgccaagggatttaaaggagctctcgagatgtacaacgaaaacaatagcgaga
taaaaccccaaatgttactagacacccgacatctgtccgaaaatcacagaaaatttgtaaaaaattctaa
atttgtcgaaaacatgatgccaggacggacagtgtctaatagaaagaaaatgcaaataagattctccaat
gacattgccatgaggtgccaagcagagtttgaacaagcttacaaacaacatgctggaaacactaaaaaaa
ttatgcatgcattaaggtacattagcgatgccatcgcagcctgttatcaaggcgtacacaacaaatgtaa
gagacactcatttgtttgcaaaaagaaaaactggctcgcaaagagtagctttctaaattctgatttcaaa
attcccaaatccatacaaaacaacamaaaccttaggaagtgcattgattatcgtctcggcccaaaaatgt
tgcaccttacaaaatttaacacaaacacacaaaaatgtgaagctgtgaacaaatgcattcgaaaatcttt
acccagacacaccaccttcagcaggaatttcgcagctagggcccacagtgcagtccataccattaacaat
gggcctgcagaatctttactcttactttcaaaagacatgggctgtacgttttctgccggcagtaaagttg
ttgcagatctctctaaagttgaaacactgcgaactcagcaactgaatagaaagaaaaaaaacacktacaa
aattaaacgatgtgaaaagagaaaggccctctatgaaatgtatgaaagacaccaaaacgatgaactgtat
gaaaaaaatagccttatgcgaaatctagttccgaaaaccgcacaaaatagtacgaaaaaaaaatgtaata
ataaaaaataaaataaataatgtacaatcttacatgtacatacaatggaaatagtataatttagtatttc
acagaagacagtgtggcctgtgccctttgtgacaccaccccctggtctttacaatgtaccctgtcactgt
tacttgcatgactctccaatactaagtgctcaggattaatgcaccgccgattatggcacagatgggaaac
ttctaaaacaccggcatatgcagccatgtcagaaggaagtagcttgttacagatcatataggcaagtctg
tgagctttctctatttttggtttgtccattttaggccaattaactttctgtctgccataaccaacttcat
tcacagatcctaaccatactgtacagtcatattttccgacatgaaaatcacttttcctgtccaaaagata
ttgcaagtgttgaaaaaaagtttccataataactgacacaaacaatgttgcttcaagtgaaaagcaaaac
cagaataaattattataattatgtaccgcaacaaaatgtgaaattcctgtata

>KolobokP-6_GiAe-I
tatcagaccaaaattgttttcaccatgacactcaaactcacatatggcatttaaatacatatgtcaagtt
gcatacaggcaataaaaagaatatttgataccaaatatggctattggagcctgtgaaatgggggactgcc
tgtgccgcaaaaggcggcaatctgcgtcaaattattcccaatagtggactagtaattatggaataacttt
aaaagttacagacattttctgaacaacaaaaagaaacttgctccttattgtgttctctgcaattgaaat
atagcagttggcaggaatatgatttttcaaaaaaaaaatcaatttaatttaaattgatacattttcaaa
aaaaaaattcccacaaagttctctttttaaacaaaatttaatctttaaaactgtaaaattttggatactg
cacacagtcaatatatgccaattacaaattaggactgctcgagtgtcattctggtgagggtaattagcaa
ggtgtctgcacaccgtaacaggtcacacctgaatgcagaggacagggcaccagtctgcaaagatgcagta
ccgaatacacagtgaacaaaaaaaagctgtcaactgccactgcactgaaatgttttaatgcatcagaact
gaaagtgacatcacacatgaacatatatataagggtggacatgcataccacaataagtatttcactgttt
tatacactacgagtcacatccatggaaggcaatacaagacgaaagacacagttcaagaagggacatgcac
cttggaataagggtgcaactctgctgcatgaacatgcaagtccagaatctcacactgagaagttgcgacc
agtagtccggatgaatatagacgagtttgccttggtcacgaagtcgagttccacttcagccaccatatcc
actccagattgcgaaggtgcgtcacagccagtccgcctgctacgtcccattacgagtacatgtactgcgt
ctgaaatgaaagaagaacaacagtgtaaagacaaagaaggcatgagaataatggacaatgacagaatggt
agatgcatggaatgcagctttcagataccactcaacagcttcccctgagtgcgacgagcctgaaatgcaa
atattaaaagaggttaagtggggtctttgttggaaagtcacactacactgtgtgaattgtgacttcactg
ctcctgaaagcaaattgtacaatgaggtgaagacaaataaacctggcccaaatgcagcaaaaactaatgt
gggtcttgctatcggtctccaagatactcccgtcggcaatacgagagcgagagtgctattggcaggcatg
gacattccaccaccgtgtcggagcagtatgcagagaacatcatacaaggtatcaacggctgtaacagatc
taaatgaaaaagatatggcacaaaaagttgagctactgaaagacattaacatgaaacgtggaaatgaacg
tgatgaaattaacatagccatggatgggagatacaactccacgacaataacaagtcggaagaaaccgggc
cagaatgcatcacaagctataggacttgcgtgtgaaacaatgactgaaagaaaattcattgtaagtgcct
gcttccagaataaactttgttggaccggagcctggttaagaaacaaaggtattcatgtgacgtgtcccgg
tggtcatcctgactgcacagcaaacttgccgccatttgctccgctgtcggaatacgagatgggaaaagat
atcggaacagacttggctctacaaggtattctcataaaatacgtcacaacagatggagattccagatcat
ctgctggagttggtagtgctttgaaacttctacatccaatgtggaaagtgcagcggctagccgatccaac
ccacttaggtcagagtcagtttcgtaagtgttacaaagctaacttcagtcccgacatgtttactgggtac
acccgtgaacaaaagcgtgaagcacagaaggtattcagccaggatatgaaagcaaggtgcagtttaattc
ttaaagaactgatgaaactacacactggtaatatcggtgtactgaaacgaaacttaccaaaggtcctgca
atcgacactttcgtgctacggtggtgactgttcaaagtgttcgcggtactcggtcgtctgcagtggagga
ataacgaacaactggtggcaccgatccatgttcctgggtacacacagaatcacagagctgaatatgaatg
aaaatgacaaaatgttagtactggaacttctgaagattaaactaagtatttcagctgtagagcaaatgaa
actgtatacagacacacagaaatgtgaggctgcgaaccgttctctcagtgtttccttacctaagaatgtg
aactattcacgaacgatgactgggagagctgcatccaccatccacaggctgaacaacgccccgggaacat
ccatgatagaaaagtgcaagtactgtggcatcgaactgtcagctcgggtgaggcgttcactgcgtcagat
ggacaaagagtcggactatcaaaaaagatatcaaaacaatccacaggtgattaaaaggcacttagtccag
caaggaagtaaaattagagatcacttacgatataaacatttgcacagagatcaacaagctgactacaaga
aaggattattagatattgacagtcaaagaacagacaactcctgtgaccacagttattcaaagtaatttt
gtcacaaaccttcaagtgatacatataatataggaactatcagtttgcatgatagcatttctgctgtgga
tatgaaccctagacattgaagactgactttaaacaataaaaaatagtaatgtgaataaatgtatgctttt
gaataaatgtatcatgtgatatttatatgtgatatttatatgtgattttatgacataaaatatttcttta
aattaaactttgttattgttttacattcttatatgttatgcattctgagatgcaagaggcagggagggta
ttgtccatgtccctgacagacatcccggttcacacattgtatgcgcgagctgttaattgcatgtggctca
gcagataagtgatctttaaacatacaaagacggttatggcacaggtgactgatgtcactgccatctatgt
ccatctccttattaaagaccatgaaacttaatctgtgagcatttactgtacgatactggcctgtgattgg
tgatttgaacttaatagcaccatatccatttggtctgggaggcccattccacattatacattcaccattt
ggcccaggatcacattttgaaaaaattaccctctggtatttttgataaaaagcctccattttcacttgtt
caattagctcaattactcaaagtgaggccctcacagggtgcagggatacattctgattgaaaagaatgac
ctatgacctgataggggtacaatgcatgctgaattatgtcataatttgtgaaattacaaattttgaaaat
ataaatataataaaatttaatttaatttaaagaaataaaatattcatcacccaaactgataactttcaga
tttccacctatctgcacaattatcatgtacattttgagtgctgtaactggcataattacctcacaatttg
gtatcaaagtgtgtcaccctgtgtcaaacatcattgaagaaatcctccatattgacttatttgaccttaa
ccttcaaagtgaggctctcatagggtgtagggatacagcctgattgaaaagaatgacctatgacctgata
ggggtacaatgcatgctgaattatgtcataatttgtgaaattacaaattttgaaaatataaatataataa
aatttaatttaattaaacaaaataaaatattcaccacccaaactgattagtttcagatttccacctatct
ggacaattagcatgtacattttgagtgctgtaactgacattgttacctcacaattaggtatcaaagtgtg
tcaccctgtgtcaaacatcctactatatca
>KolobokP-7_GiAe-I
ttaagaacaaaaaatatttttcggtttataaattttattgctccaacatgaatactaacatttkgcactt
cacgaaagtcacttggttatatgttgacggctggtcaagggggataactctttgaggagtgtcactgtg
ttggtctgatttggcctgtaaaaaatgggggaaaaattattagtgtgatacactggaattgttttgttta
aataaagttttctattttaggccaagtctgactgattgctaaacctggtaatatcatactgtatacaggg
gggtattttcaggccacctcacctgtccctcaatttttacatttgattttaaagttgaaaattgtggaat
gtgggaaacattttctaattacttcagtgattatcacaaaactgttgctctatttggagtatctttggca
tattgggtaaccactagcagccatacgtccaccatgacacaacagcatgttgtttcagtgtgttgttact
acataccaggacaaagtattcagtgggggtattctgcggacctggcaaatgttttcccagaggcaggat
tatttgtcagtcagataattttgaaaagtgagaacatgtttttgtcacactggggcagactacagaaaat
taaatttatatactgacattgagaaattattatggggaaaccccctgccaagaaaaaaaaagttttgggt
agacccccctaacagaaaaaatgtatatgtaattggtcacaaattccatcaccaaaaagaaagtacactgc
tcccaaatgccaccaaatcacctacctgtaccaggctgtccagggaagactttgatgcagtggctaagcc
gtcaacgagtcgaggtgcatcttcatatgtggtcaccgattcacaggggacgactggcaatatgcggtta
cttcggcctcacccagctgttcccgatgccttggaagatttcctgaaacaaaacaatcaagacttagatg
gctataaacttcttcattgtggtagagtgctcaatctattcaatgaatcttatcgagaacatagaaataa
cttcccaacatgtgatggtgatctgaagtatgacgaacagtcctatcaacaatggggtatgtgttggcgc
ctaggtttaaaatgtgagctatgtcattttagatcgaagaagcacaaattgtacgaagaagtcactttac

ccactgcacgaggtcagaaagctgctcagcctaatattgggatacatgttggccttatgaatacatcagt
tggaattacaggatttcgcgattccttatccgcagctggtatccctgttggaagcaagcgtggaatgcag
aattcagccaattatgttggatccaaaacagtgtacataaacagacaggatatgaaagaaagacggtcta
atttggttcgactaaacaaactaaaaggttttaccagtgatcacaagataagtgttgagggagactgccg
ttacaacaacagacttttcggtgggaactcgagcacacccctccagcctgcaactcaagcggtctacacc
attgctgagaacgaaacacctaaaaaggaaattattgggatatacactgcaaacaaactttgcaaaaaag
ccgaaatcctcagatctaagggagagaaaattacatgtccagatcacggtggaaaatgtacagccaattt
gaagacaacagattccatcggcaatgagggcttatatgcagaaaaatgcgtgaaagaatttcaagtggac
gaagacaaactcaatattggttatttcacgactgatggcgattcacatgctactgacggtgctacccggg
cgcagacaggttccattgaaaatttacgtgacactagacatttttctaaaagtcagcaaaaggcgattga
aaaagtttcatttagcttaaccatgtttccgggggaaaataaatcagaccgacagaaagtacagcgacgt
tttgccatcgacatcaagcaacgttgtcacctcgaacttgacatcgcccaccgtcaacttgcaggagata
tcactaaaatagttagaaaaatgacgtacaccatcgattccatcgtggcctgtgtgagtggcaactgttc
aacactgtgtcaaaggcattcatttgtgtgttcgggcaggcggatcaaggcatggaaacctgcctattta
cctgcagattcagttctgaaaatgaaagaaggcgataaagaattgttaagaaaatgcctagaacttcgac
tcggcaaaactgcacttcttaaaacaaaactcaatacaaacactcaaaaagtagaaagtgtcaaccgggc
ttatactagaacaaatccgaaacaagtgacattctatcgcaactttgaagcaagaatacacagtgcagcc
cacatgctcaacaaaggaattctaaagtctactgttacaagatgtcaactagtgggagccccactgactg
gaagaatacacgccctcgcagaactgaaagctcgggaacgtgaagaccggtatcatgctactaggaaaaa
aactactcggtacagatcccgaagaaattatctccgcaaggttcgataccagcaatatgacgacaaacac
aacagtactgcaagaacaacctacaagaagaatgtacttgactccgaagttgtaccaccatgtctaacag
atcatacgtacctgtccccaagaagaaaaaaacagtctctataacctggatggccgatgcagtgtttcgg
gacaatgtttagacagtgttgacggttgttgttaacataatgtggttcgaggctcaagtgttcaatgtta
acacagagggagtggtgacataaatgcgatacatccatgctacgaggtatgtcaaagtgattgtggtgga
gcatatactggagtcggtgtactttaaaagtttggtgttttggacgaccaggtatttttgcatgaataac
tccatatttgttataatcctttcttttgtcccattttggcagcctgtccaaattacacatcccatattt
ggcatcttttcagtcttggccaatatacgatttttcatagtcaatacccagtttgagttgacctcaacct
taacatttgttgttgttgtggtcatattacatcagtgattatgtaacaamttataaaaattaaagcagcc
aatttctggctccattaaaattatgaaatatacaaatacattgttattattaccttggwaaccagtgtgg
tgtgaattccagtggtatgtgtcaagagtgtcttca
>KolobokP-1_HaRub-I
tatcaggacaccaaaaagtctgcaaattaaataaaacttmatcaatactaagcccagacactatgtaagg
ctcacagaaaatttgaaagccctaactcatacacaacttgacttatactccttttactccatgctgtatc
tctggcatttgagtgttaattttgggcatgaattaatttcaaacacaattctaatttgtctataactcca
taaccaggtggaaattgacaaaaacatcaagtgttcctcttttgttattgcatgtgctatcaaaggacaa
tggtttcagtccattaaagcatttttttcatttttttctctatttttatatacatacatgcattttatac
taaaataattccattaaagttcatatttttcaacaaaaatgaaaattgaaactgttaaatttttattcat
tagaaagccctatctttcctcgttaaattgattggctgcacaagtagctgagtacagccaatcaagtgca
gcgagtcaccaagcatgtccactctcacctgacacctggccaatacgcgacaaagtgtcagtaggtgtgt
atggctcaactgcgatttacaccagtggcacaagacatgtgtatgtcttgagtttccaatacattttcta
atacatatacctgcttgtcagtgccagtgacaacacacagagatgggatatataagtacatgctgtgctc
agagctcattagttctattcgacacaatcacgatgaaaggcgaccgcaagagaatcaagtcacagttcag
caaaggccatactccctggaacaaaggtctcacccttgaatcggaagagtcggaaagtgcctctccaccc
cagatacccaggacagtcactaggttgagtgcagaagacttctccctggtcaccaagtcaaggctggatg
gcactgggctgtcaacacctgactgcgatggcatctctacttcagtgaggctgctgcgtcccacttcaac
gagcaagcatgacctgaaacaagagtcaagcaaagatgacttggagggtatgcgactagtcgacaatgaa
aaggtggccgaagcatggaacacagctctgcgactgcatcggtcaacatccagtgactgtgacgaccctc
agctgcagatagccaaggagagaaaatggggagcatgctggaaaataacactgaaatgtgtcacatgtga
ctttgtagcacctgaaatgaagttatacaaagaaatcgagacgggtaaacctgggccaaacccagcagca
cccaacgtgggacttgccttgggtctacaggacacacctataggaaatacgagagcgaggttactgatgg
ccaacatggacattccacccccatgcaggagcagtatgcagaggacctccaacaaggtggctaaggctgt
aactgaacttaacactaaggatatggcagaaaaggtggagttagtgaaggaagttaatcgaaagaggggt
actgagccgtgtgagatgaacattgctatggatggccgatacaactcgacaacaatagcaagcaggaaga
agccagggcagaatgcatcacaagcaatcggcatcgcctgcgaaactgttactgacaagaagttcatcgt
cggtgcgtcattccagaataagctctgctggactggtgcctggctgaaaggaaaaggcatggatgtgaaa
tgtccagggggggcacccagactgtactgcgaacatgtcggccttcactccattctctgagttcaaaatg
ggagaggacattggtaatcagctcgcactacagggtgtactcataaagtatgccacaaccgatggggatt
cgaagtctgcagatggagtagacaaagctctcaaagtgttggacccaatgtggaaggtgcaaaggctagc
tgacccaacccacttggctcaaggacagttccgacaatgttacagagctgattttaacccagatatgttc
cccggcactactcgtacacataaaatggaagcaaagaaggtgctgagccaggatgtcaaagcacggtgca
gcctgatactaaaagaactcatgaaagatcatgcaggtgacatgaaaacacttcgtagtatcttaccaag
agtgctcgaagcaaccctcaagtgctacagcggcgactgctccaggtgtgcccggtactcagttgtgtgt
agaggaggtgtaacaaacagctggtggcagcgatccatgttcctcggctgcaacagtatcactcatttca
atatggatgacaatgataaacacttgcttaatgaaattctcaaaatgaagttaagtgtagaagtggtgga
acaaatgaaactgtacacagacacacagaaatgtgaagctgccaatcgatcactgagtatttctttgcca
aagaatgtaaacttttcaaggaacatgattgggagggcttcttccacaattcacaggctcaacaatggcc
ctgggacatcagcaatcgacaagtgcgaccattctggggtgaaactttcaagccgagctgtgcggtcact
tgaacaaatggacaaggaggctacctatcacaaagactatcagaaaagccccagtgttgtgaaaaggaaa
cttacacagcatggaagcaagatctgtgaacatttgaagtctaaacagactgacaagcagagtggggact
acagaaagggtcagttagaccctgtaccgtgtttcagcaaaaaccatgaacattcttattcaaaatattg
aaaatgtgcagtgatgctgcagaactgtatgtgctaattacttgctaaagacagatactgtgtaactta
agtttaaatacatcataatgtctctaaagctatgtgttgtgaataaatgctatgtcacaactaactgtga
ctgtggtgaaaatcaggaaatatgaataaaattttactatattatatgatatagtatttttcatatactt
tgcctttgtttttctgttctcctataacttcaatgccaaaaggcaatctgggaaaggtgcatggccttta
cacacacccctgtttacacaggccactctgtctgcatttatgctgtgtggttcagcagacaggtgttcga
cataggtgcatttactgttgtggcacaaatggctgatgtcgtaatcacctatgtcgaagttttccctgtg
tactataaaactcagcctgtgagcatgagtagtacgccactggtaggctatggggcaaaaataccgcaac

accccatacccactgtgcttgcatgctccagtccacatatgacatatgccattttccacactgaggactg
aattcttcacaaacttggctctatatctttggaaaaaggcttgaatattagccattttaggtatcacatg
catctataaggcacgtgcggtgtagtgatacatacattcaccataattgaaccaatctgtctaaatatga
tgcgtccgctcactcactaaaacaatatgcaatcagaaaactgaaataaattcataaaaaatgtaaaaaa
aaatattttcagttgaaaacacatgtatacatatttcaaatgctcctggcagtaaatatgcaaagtttca
agtgaatattttgaaaaataacagacatatagcaaattaaacacttgcactaccttcaccatatca
>KolobokP-1_HaRuf-I
tcaggacagaaaaaagtatgcaattcaaatacaacttcatcaatactaagcccatacactgttgaaggct
cacagaaaatttggaatccctaactcatacacagcttgacttatagtcatttgaaatcatgctgcatcca
tagcacttgagagtacatttttggcatgaattcatttcaaatgcaattctaatttgtccataactccata
accaaaaggaaattgacaaaacaagcaagtgttcctgtctcgtcatgacatatactatcaacagacatca
atttcagtccggtatatcaattctttctttttttggcctatttttaggtacatgcatgcatttttttaaca
aaacaattgaattaaagttcatatttttcaacaaaaatgaaaattgaaactgttaaatttttattcacat
gaaagccctgtctttactcattaaaatgattggtgggacaggtagctgtgtacagccaatcaagtgcagc
gagtcaccaggtgtgaccactctcagctgacactcggccaatacacgccaaggtgtcaaactgcgtgtac
agctcaactgcaatttggactgctggcacaagacatatgtatgtcttgagagtttctaatacatttacac
gcttgtcattgccattgacattacacagtcatggggtatataggtacatcttgtgctcagagctcattag
ttctgtccgagacaatcacaatgaaaggcgatcgcaagagaatcaagtcacagttcagcaagggccacac
tccctggaacaaaggtctcacccttgagtctgaagagacagaaagtgactctccaccccagatacccagg
actgtcactaggatgagtgctgaagacttctcactggttaccaagtctaggctggatggcactgggctgt
cgacacctgactgtgagggcatctctacctctgtgaggctgttgcggcctactccggccagcaagcaaaa
cctgaaacaagagtcaagcaaagatgacttggagggtatgcgactagtcgacaatgaaaaggtggccgaa
gcatggaacacagctctgcgactgcatcgttcgacatccactgactgcgacgaccctcagctgcagatag
ccaaggagagaaaatggggagcatgctggaaagtgacactgaaatgtgttgtatgtgacttcacagcacc
tgaaatgaagttgtataaagaaatcaagacaggcaaacctggaccaaacccagcagcccctaacgtagga
cttgccttgggtcttcaagacaccccctattgggaacacaagagccaggttactgatggcgaacatggaca
ttccgccccatgcaggagcagtatgcagaggacctccaacaaggtggctattgctgttactgaactgaa
tacacaggatatggctgaaaaagttgaattggtaaaggaagttaatcggaagaggggtactgagccatgc
gagatgaatattgccatggatggccgatacaactcaacgacaatagcaagcaggaagaagccagggcaga
atgcatcacaagcaatcggcatagcctgcgaaactattaccgataagaagttcatcatcggcgcgtcatt
ccagaataagctgtgctggactggtgcttggctgaaaggaaaaggaatggatgtgaagtgtccaggggg
cacccagactgtactgcaaacatgtcggccttcgctccattctccgagttcaatatgggagaggacattg
gaaatcagcttgcgctacagggtgtgctaattaagtatgctactaccgatggggattctaagtctgcaga
tggtatagacaaggctctgaaagtgttagaccctatgtggaaggtgcagaggctagctgatcccacccac
ttggctcaaggacagtttcggcaatgctacagagctgatttcaacccagatatgttccccggcactactc
gtacacagaaattggaggctaagaaggtgctaagtcaggatgtaaaggcacggtgcagcctgatactgaa
agaactcatgaaggatcatgcaggcgacatgaaaacacttcgtagtatcttacctagagtgctcgaagca
accctcaagtgctacagcggcgactgctccaggtgtgcccggtactctgttgtgtgtcgaggcggtgtaa
caaacagctggtggcagcggtccatgttcctcggctgcaacaaaatcactcatttcaatatggatgacaa
tgataaacatttgatgaatgaaattctcaagatgaagctgagtgtcgaagttgtcgaacaaatgaaactg
tacacagacacacagaaatgtgaagccgtgaaccgatcaatgagtatttctttgccaaagaacgtgaact
tttcaagaaatatgattgggagggcttcatcgactattcataggctcaacaatggccccgggacatcagc
aatcgacaagtgccaaaactctggtgttgaactatcaagtcgagcagtacggtcacttgaacaaatggac
aaggaagctgcctatcacaaggaataccagaaaagtcccagtgttgtgaaaagaaagctcgtacagcatg
ggagcaaaattagtgaacatttaaagtcaaaactgagtgacaaacagagggggactacaaaaagggtca
acttgaccctgtgccatgttttagcaaacaagctgaccattcatattcaaagaaatgaacatgtgcagta
atactgtaggtaatgactagcttatgactgactttgtgtaaacaattgagttgaaataggtaacagttgc
tgtacagctatgctttttgtatatgcaatgctaacaatcagctgtcactgtgctgaacatcagttaatga
ataaaatcttaatcatttactttgcttgttgttgtctcttttcatagcttcaaagccaaaaggcaatctg
catatggcgcatggcctttgcatgcacccctgttcacacaggctactctttctgcattgatgctatgagg
ctcagcagaaagatgttcgacataggtgcatttggaattatggcacaaatggctgatgtccaaatcacct
atgtcgaaatcttccctatgcacaatgtagctgagtctatgggcatttatggtgcgccacttgttggata
tggggcaatagtatctcaaaaccccatatccactgggcttacatgccctagaccacagctgacattggcc
gttttgacaggtgccgtgttggtcagttttggctgaatgccgagcaaataactctctgtatttcttaaaa
aaggcattgatgttggccatattaaaagtgtccaagtgcaaacgaggcacgacaggtgtagtgatacatg
attctaccatattggaactcatatgacttattatgatgctggcattcactcatttcaacaacatgcataa
aatcgcacgttcgaaaaagtacacaaaatcattaaaaaaaataaaaaaatatttcaacctgaatacacat
gtacacagatttcaacacttactgtgaatatatgtgccaagtttcaagtcaaaatcttgcataataatag
agatatagtgaatcaaacatatgcactagcctcaccatatca
>KolobokP-2_HaRuf-I
tgtagacacaactgtctaaaactgcacttgtcaaatttgtgatgtattcattgtaaatagatgtagcttc
ttggaaacaaatggaaattgtttcaagttcaatactttttcctatctgacaggtctatatgacacccata
tacacatatctgatgtaccaaaagagctgtgagggttaaattatttagattttgtcaactgattacaaaa
tgaacttctgttttaataaagaactttctatgacagtacacaggcctttattgtttattcttgtttcaca
caaggatgggttcacaggtcaattccatggttgccagacgctgcttcgaacatatcccccccaaaagtatc
ttttcgaccccgtatgatagacaatacttggaaattcaaagcaaattaggctttcatatttttaatttat
taatggtgagttgtttgctgacctttgctgataacaactgaggatagcaaagtctggtacataacatgga
atttggttgtgacactaataagcttaagtcataaaagaagctggactcagtgagcttgctggaactggta
cccctgccttgtttacataggccaatatataaatgacaggtccatatttggtattttaactatgggaggt
caagaaccaatgttgatgctttcattccaaagaaaaattcatagtgtcaacatcttgaacctactcttga
aacatggggagacatcgaaggaattcgataacatcttttagaatgggacataaagcttggaattcaggca
aaaaattcaccagtgaaaagcaaagcaactttaaagtgccaaaaactgtcaggttgacaactgagctcca
caagaaagtgtgcagtgtaggggtggctggtgtccagcaagttcaaacactgacaagtgaaccgtaccgt
ctgttaaggccaaggaaggaaaacacccctgaggaggataagagttccctgaaaacaaaaagtgaaaggt
aataacactttaaactatttattcctttggcctagactttgaaatacttgtagggaatacaagatgctca
ctgctaaatgatatgggtggataaacttaccaagttcaagtatttgtcctttaacgagatcacttaataa
aagtttaatataatggacagttttcagttattaggtacagcaaggatcaaattctcatacatatatgag

tggtttgttcaataccatataaaaaacagtgttgttacaccctaattaagggcttgaggggatagaaat
agaatatgcatttgatctgccaggggagataattcagaaatgtcgaacactatcacatacttaaaaggct
tatttagaagttttcaaagtagtcaatgggttttctttaatctgcaggactgctgctgatgtttaccacc
tgttgcatgttggagagactgacaaactgtacaatgaggcattccgccagcacgccaagacttcgcctat
gtgtaaggggacattgtcatggacatagacaatgaacagcagtggggacttaactggatagagagactt
aaatgcactgactgtacatacctgtcagagtaccataaactgtacaaggaggtagagggtagtggaatga
gaaggggacgtcgatcttccactgccaatactggacttcagataggcctctcaaaatgtcccattggaag
tgagagtttccgtgtcctgtgcatgagtgccgatatacctccaccatcccattcagggatgcagaaagct
gcgaacagagtttgtgataatataaaatctcagaacacaaaggatatgagagccaggagggtggaaataa
gggaaataaataaatacaggggaacaaatttaaataacattagtttatcaactgatgcaatgtacaacaa
tccactttgttcaggtgttgggaaaaccccttttcagccagccactcaggtaacctatgtggccactgag
aaccaaactagtgatcatcaaattttggctttaaacacaaaatcaaagctctgttcaaagcacagtttag
ttgacaacatttcttgtgtcaacaacccagactgcacatctgatttgcacatgtcagaatcaattggtaa
tgaaaaacagttagctgcagagtgtttcagggacttaaagaaagatcaaattgaggtagaatttttgaca
acagatgctgacagtgcagctcacacagctctctctgaaatgtatgaagcaggtgatatggatgtttccc
cacaaacttttctcgatacacggcatctctcggccagccatagaaagtttattaaaaattctaaatttgt
tgtggatatgatgcctgctagaaccaaaaaagacaaacaaagtttggcaaatacatttctttagactgt
gcatccagatgtgcagctgagttctcagctgcttactcaagtcatgcaggtaacctgacaaagttgaaga
catctctttcatgtacttctgatgctattgcaaactgttacatgggtgatcacagaaaatgtagaaggca
gtcatttgtttgcaaggggagaagaaaaattggttattgcaaagtaaatatttagataaaaacttcaaa
attggaaacaatgaaactaactttcaaaatttgagaaaatgcactgattttcgtctcggcccgaacatga
tcgaaaagacgcaattcaacctcaatagtcaaaagtgtgaggcaacaaacaaggcattgcgccggtcatt
acctcgcgataccacctttgctcgtaacttccaaggtagagctcacagtgctgttcatagcgtcaactgc
cagagtccggtaaagtcaattcgcaatcttcgattagggcttggctgtgctgtcactgcaaaaggaaaaa
tagatgatgagataagcaaaagtcataataaagtacttaaagctaaaacatacaagaaatcacagaaaag
taaggaccgccgtgcaaggaaaaagttagaactattcaaactccatcattgtagaaaagttcattattcg
aaagacatgttattgaaaagaaatgcatatttgagaaatttacacccaaaataggataatccatcttaaa
aattgccccaaacagaaagatctaatgatcagatacataacttacaccaaacagtagggtttatgacttt
tggtgcacaagccctgttgtcgacagtgaagtctctccatgttaatttcatgggactctactttcaaatg
ctcaacattcacacaagaggcgttatgacagagatggctcacttcaactttgttgccaaagttgtcagtg
ttgctaaaaccatctggaaggcatttatgcacaatgcagagggctattctgtgagctctctcctctgtcc
gcgcctttgacggccacaaaaagaagcaacgaccatagccctgcttatcacggcagcccaaccattcctt
acaaccccatttcccactcgcttcacccattttgcgttcaaatttgcctctacaattatcccaaaaggtc
tccatggcaatagatatacagaaaaacatataaattaggaaatgctgaaatatgcacttaccctaca
>KolobokP-1_SteCin-I
tatcagagctactatttttgtcgaaatccaataaaattttacaggtagtaagtacatccaattacctatt
aatgcaccaaagctcgggtctcagttgctaacacaagcaacttgacaggggtcttgcttcaggctctctc
atctgctttcaaaggccagttgatctgcaagatttgtcgtctgcagggtagcgcatcactcataactctg
aaatgagacaagatatattcaaaaagtaacattttcctacaaatatgactcaaatactttcaaataaaca
acacttgaaaaaattaggaccaaaactaatatttttttacaatttttttaaaaattcatcgattttcggta
caatttttcaaaatgttcatcaaatttcaatttaaagataaaaattgaaactgctaaatttggtaccact
tgaaagacattttatcagtggttaaaatgagaccactctcagttgattgctaattgcctcttgattggta
ggtgctgtttagtgctacctgtcacaccttgcagttagccaatacgcgtataggtgtcaagtaggtgatc
tctcctgccggacaatatacatttgcctggtgacaagtataggttccaagtggcattgtatacactaaat
tgtcagtggagagacaaatcacaggtatggtgtataaaatgagctgctgccagcagtggtgcatcatttc
aactttgctatcgaacatgagaggagacaagaagcggcgcactcaaggcttccagaagggcaacattcca
tggaatcaagggctaacttttgattctgatgaagaatcagacctccaacagcaaacacaaccaaagcttg
tcaccaggatgagtgctgagcagtttgaactggttgccaggtcaaggtctgaagactctcttctccctac
accagactgtgagggtatctctagctcggttcgtctgcttcgtcctttgccggctgagaagtccaagtca
caggacggcagttctcaagtctctgagggtatgcggctggttgacagtgaagaaatggccaaatgctgga
atgagagtatccgccttcaccactcgacgtcacccgactgcgaaaacccaaaccttgttatacataagga
gaggaaatggggtgtctgctggaaggtagtcctgaaatgcactgcatgtgactttaaaacacctgagatg
aagctctacaaggaggtgaagcaagacaagccaggacccaatccagcagcagctaacttagccttggctg
tggggttagaagatacaccgatgagcacaacaagagcaaggcttctcatggctacgatggacatagtccc
cccggctaaaagcagcatgcagagaacagccaatcaagttggaaaggctgtgactgaactcaacacgaag
gatatggctgacaaggttcaactcatcaagcaagtcaacagcgatagaggagtcgagatgagtgaggtga
acattgccatggacggccgatacaattccacaactataaccagcaggaagaagcctggtcaaaatgcctc
ccaagccatcggcctcgcctgtgaaacagtgactgacaaaaagtacatcatcggtgcctcctttcaaaac
aagctgtgttggactggagcctggctgagggggaagggactggaagtgacctgtcctggagggcatgcag
agtgtacatcaactatgtcaacatttgctcccttctcagagaagaatatgggaaaagacataggtgctca
actggccctacagggtatgctgatcaagtatgtcaccactgatggagattcgagatccgctgagggtgta
gacagtgccatgaagacagttgaccctatgtggagagtcgaacgacttgctgacccgacacatttggctc
aaggacaattccgccactgctacaaggctagtttcagtcctgagatgtttgcgggcaccacccgagagca
gaggaagcagcaacagcgagttttaagtcaggatgtgaaggcccggtgcagtctcattcttaaggaactg
atgaagactcatgctggtgacatgaatgcacttaggaatatcttacctagggtcctcgaggcaacacttc
tgtgctattctggggactgctcccgatgtgcccgtcactctgtggtttgcagtggcggtgtttcccgtag
ctggtggactaggtccatgcacctctgcaagaacaagatttcaagtttcaacatgaatgacagtgataaa
catctgctacaagaaatcttgaagatgaaactaagtgtggaagttgtagaacagatgaaattttatacgg
acactcaaaaatgtgaagcagcaaaccgatctctgagtgtatctctacctaagaatgtgaatttctctag
aaattacatgggacgggcatcgtcaaccatccacagactcaacaatggacctggcacatctgcgattgag
aaaactgagtgtgtcggtgtacacctctctgcgggatctgttcgcgctttgcaccaaatggatgagcaga
cagaatatcagaaaaagtacaataaagacagtgctgttgttaagagaagattattacagcaaggagagca
tattggcagttacattaaggccaaagcatccatgaaggacaaatgtgattacagaaagggtcagcttgat
cctaaaccatctaccagtcaggacagagatcattcctattcaaagtgatgctttgtattaacaatgttta
attgtgaaatgaggactgggaattctacttcattgttctaccatgcaaacaaactgactctaattcatga
tttctacaaattaatgcttgcttcatgtatattttctatccacatgagctacctgaaactgtgttgtgat
agtattattgaaatgtgatgaatggaagaattgtacaaatgtttacaaaaatcaataaaaacaaaattat

catcatatatttgcctgttattctttgtgatattggagaagacagtcctgatatgcaccatgaccagtgc
agacatgcctatttacacatttgcacctatcggcattgacactgggtggttctgcggataggtgcgtaat
caaaatacatttcttattgtgacataaatggctgacatggagatgatcttctataaacttttctttaaag
acaatgtagctcagtctatgagcatttactgtatgccatctatttgtaacaggagatgagtatcttatga
tgccatagccagagggctttagggctctttgccaaatcatacactcatcttctttcctactattttccag
aatctttgcttgatacttattaaaaaaagcctccatcttgaacatgtgcaataattgctatagtgggtgt
agtaatacagaaatattccatatttagaagtacctaatatgtctttttttgacctgctaagaaaatctaat
gatattatagacatttaaaaattatcaatattttaaaaaaaatttaaaaattcaaaatgaaaattctagt
aaaaaattattatactctctgatttatatgtgtatactgaataagtgtaccaaatttcaagcaaaaagat
ttaaaactgacaaagttattagcaatcaaacacatctacccccccttttggtgctttccatatca
>KolobokP-2_SteCin-I
ttaaggaaaaaccaactctcaccaaaacaaatttatagttatttttcttgaccgtcaaggtttttcgaat
aaaacggtatatgagaaaaggtttttagatggaaaaaaatgcccaactacatgattttgctatgagccac
ccaaaagcctcaaagagagctcacagcatcaggtttaagacctcccagtcaaagaacaaacacataatct
atgcaacaaagacaatatactataacattttagagcttttctcaatcattagggtctaaagtaatagaaa
atagccaaaaaagcacagaagatctgtatatttcccatcaatcatggttttaactacttctggcatgaat
tacatactaaaaatggatcaaatctgtggtaaagaatacaaattttgagtatttacaaacattttcctct
ttacaatgtatataactcttctttgacattaaaacattattttctgctgttaaatggtgctaaaggccct
aaaacctataaactataggcaaaactagtgaggatactgaaaaattaaggggagataactcttgtataaa
taatcacaaaaaggagtctctagagccactgagttccacgcggcattgtttaccagttccaaaagaacaa
acatcgtcatagcgatgttgtatgatgtcattaggcattgtttgggcctatcagctttcataactgccat
cctcctttagcaatggagtcctgtttttatatgtaccttcatcttaatactacaaagtgatattttgagt
tttaagtaccttaaaattaccataataatgaagaaatgtggaaaatcaagcgggtgcaaggggcgataca
actatccagggagatttaggcctggagtcaagcacaaacctaaagtgagtgtcctagttgagtcgtatga
ttctgcggatgaagatgccgtcaccgacaatctgcgcacgaggttgcgtcaagatgtggttacctgtgca
gaggatagaaaatctgttttaaaaccatccagactgaggccagcaaaaattgaaggaaatcaacaacaac
caagtgcacccaaggctttgaatggacttgttttacttcaatcaattccagaactatttaataatttcat
cagtattcataatactgctgacattaaatgtcaaaacatgttaatggaaatttttgacattgcatatgtg
ggacttgggattatactcacagttcagtgcgcaaattgtaaaatgcatctttcgcataaaatgtataaaa
tgtgtgagagaattaatcaaagacgtggaccccgccagcccgaaatgaatcgacaagtcgggatggcatt
gttcaagacaaagatggggatttctgacattcaattcttattttcttgcatggatgtacacccaccttgt
agtgcaactcttcaaagggtgtcaaatcttataggagagactgttgctgatctcaatgctgagtctatgg
ttgaaaatcaagtcttcataagggatgtgatcaatctgcagaaacaaacatatggagatgacaaaaagtt
aattcttgatgttgaaactgatgtggcgtacaacaaccgaccatcaggggggcggtgagagtggaacgcag
gcttttgcacctataattgagactgtaactaataagcatttagttttaggcttttctactgcacaaaagg
tgtgcactagaggacttacatgtagccataagaattgtgaagttaactatgatatgaaaaaaagcatttc
ttcagcagaagcagttttagccattgaaaatattgtaaatgttaacaaaaatggcattgtaaaagtggaa
tctatcacagcagatgcttgtaatcaaatagacaaagttgctgataagttaaagctcaatagaaatactt
gtgttgtgcatagaatgcgcgttctccaacggcggattcaatcattagatttaagccgtcacactggttc
gaataagaatgaaagaaacttgtatataagaaagttatcacgtgcagtgagaaacagaattagatctgaa
ttgagtgcattaaaatgtaaaagaattagtcaaactgaatatgtgaagatagctaaaggcataaggggaa
atgtattgaactgtttttctggaaatcatgcagtatgtacatattcaggggggttgcaaggccagaaaaaa
ctcatcatttaaacccgtctttttaccacatggttcctaccttgatcttactgaaagtgacaggtctaag
ataaatgcagttctaaacagcgttttatcagagcgtcttttgcaacaaatgtggcgcatgaaaacaacta
atcattgtgagtcacttaatcatcaagtatttacctatgccccaaaaatgtcactctatgccaaaaattt
caaaaatctgtgtcactctgctgttcattcaaatacgcatagtgtagggcagagtacatgtttactggct
aaaaaattgaaaatcaaatatgctaaacaaggatgtttcaaacattttatgttgggtaaggaccgcaggg
cactttatgacagaactaggcaagctggaaaaccatttcttatcagacggcacttacgacgaaaaattcg
agcctatgacaaaacattgcaaagttctctctacctttcatcgtctaatgtcagcctttcagatgaacat
aattatgccaaaaacatattttaatctaattccaaatgattatgtggaacaaatcataatgaaggaaaag
tgataatgagaactaagtaaacccaataatttgtcatgcttaagttacctcttataatgcaatctggctc
gttggggtgagaaaaacattgtttctcttgaaaacaagctttcctttgtgagttagtacatgatgtctct
tgattgagatgttgaatattcatgcaaattttggtgttacacaaatgacttatttctaaacttgaatgaa
aactttcggaattcatcatataatagaatgcacgatgcacccgcaacctgacacgtctttttttgtaatgt
tgtggtcaaaacaggataaccatccttatctgtggaaccttcccacatatggcaatcctgtcctgaccca
ctattgtcaattctacttcttaatttgcttagcaaattgtttcgttcaacatcagttaattttaatgagt
ataaaatattcattctttgttctttcacccaaaactgtgaatagcacacctgaaaacataggattactat
aattattgaattattgatttatattttgatgaggaggatggcagttatcacatgtacataagttgtgtta
ataaaattagtgtttttttaaacatttgtatttgttttgatttagagtgtgcctacaagaaatgatctcat
ccaaggatagacactatttggacacaaaattaaaccaatttaagtgaacttttagtcaaatacaacagaa
acaacattaataacaaattatctctcatttcaactttcatttagcccccaaaaaatactcaaattacccc
aaattaccaaaaaaaaggtcaagaaagatatttttgacccatttttgtactatatgcaagtttccgtggca
acggccataaactgtgtccacattttgtctatatttggatttgtctttgatcagtagttatgaaaatcca
ataactggtcccaaaaggcttgattcattcccagaaagacagaaaatgtcacatttaggtttttccttaa
>KolobokP-3_SteCin-I
tataagacctcaacatatgcttagatatggttgaaatttacctatatagtacatcaatgtatggggaatc
accattccaaataccaaatcagtcacttcacctacagccaaatgacagtcattcttcaatacataccta
tgaccaatttgggcttcaggaaaggccggccgtccaagatttcgcacatgcgcaattcatcacagcagtg
tctcctgttggaattaagaaacaaagtaaagtttttataaacagtggagtatcactctgttacagcatac
aagttttatagaaataaacattaaatttgatatttaagtaaacaaaattcaaaactagtcatttttttatc
agaatttttttatttaatacaaaattaccaaaaatcttagattctcaacctgcaaaatttctatttgtga
taaagatgccttttcagggattctaatgagctattacaccatggcacctggtcactgggtatctcttaac
gaggccctaaattcttcctcctcacaggacagtatacaaacaaacagcatggcaagggaggagacactct
gttatggcaggcaggggtcagatttgacagacacgggggtgttttcaaaacattacctacccctgggac
tcaaataactataaaagagaggtggaaaacttgagttatcattcccttccatgacttaccaccatgaaag
gcgataagcgaagaaaagcatcacagatgaagaaaggccatccacagttcaagaagagtcaagattctaa
ctcagaagaacccatgccacctccaagcgaccaggtagaacggcagtacactagacttagtccagaagaa

tctgaaatggtcagcacatccacttcgggaggcgccagtctatcagcggcaaacattgacggcaaagtct
catccatttgcctgctgcgtccaaggtctggtaatcacccagaaaggcaagaaacatccgggcagagtga
acttgagggcaacagagtaatagatgtcaccaagatgaccgaggccgtcaactctgccctagaaatgcac
cacacatcatcacccgactgccgagttccgcaactcgttgtgcaccgagagagaaaatggggtctcgcct
ggaagattacgctgaaatgcactgagtgtcatctaatttcaccagaaatgaagatgtataaggaggttga
gacaaaccaacctggtccaaatgcagcagcgcagaatgtcggccttgccgtagggttgcaagacaccccc
atagggaacagcagagccagactcctgttggctaacatggacatccctcctccctgtcacagtgccatgc
agagacaatccaataaggtatccaaagctgtgacagagctcaacactcaagacatggcagagaaagtgaa
gattgtaaaaaatatcagtgaaaagatgggaagcaatcctaacgaaatgaacattgccatggacggtcgg
tacaattcaactacaattgtgagtcggaagaagccggggcagaatgcatcacaagctattggtttagcct
gcgaaacgatgacagaaaagaagtatatagtggctgcaagtttccaaaacaagctatgttggacaggggc
ctggttacgaggcaaggggatagaagttagatgtccggacggccatccagagtgcaccgccaacatctca
gcatttgctccactctctgagctgaacatggggacggatatcggcaaccaacttacaagacaggggattc
tcatcaagtatgctacaactgatggagatgcaagatctgcagagggcttagacaaggcaatgaagctcct
agatcccatgtggaaggttcaaagacaagcagaccccacccatctagcccagggacaattccgacaatgt
tacaaggcgcagttcagtgctgacatgtttccgggggccactaccagggatcagaagatggctgcaaaga
ggacgttaagtcaggacgtaaaagctagatgcagtcttgtcctcaaagagttaatgaaagaacacgctgg
ggacatgaaatcgctctctattatcctacctaaggtgctggagacaacactcaagtgttatgatggggac
tgctcacaatgtggccgtcactcggtggtgtgtggaggtggcgtcactaacagctggtggcagcgctcca
tgttcctcgcctgcaatggaattgaacaattaaacatgaatgacagtgacagacatcttctccttgaaat
tcttaagatgaaacttagtgtacaagttgttgagagcatgaaactttatacaaacactcagaagtgtgag
gcttctaatagatcattaagtgtgtctgtgccaaaaaatgtgaactttccgagaaatatggttggtcgtg
cttcatcggccatccatagactaaataacaatcagggagcatcaacaatagccaaatgtgaggctatggg
tgtcgatctctctccacggtgtttgcgttcactgtccaagatggacaaagaccaagagtatcagaagcga
tatcaaaagaacccatccaatgttaaaagaaagttaataaagcatggcagggacattgttaaacattgtg
aaascaggaaaaacaacggggaaaagtctgactatcagaaaggacaactagatccgaaaccttctcacag
ccgagatcatccctactcccgatgagtctaaacaatattgtaaatatccattaatatattgtataattta
tatatcataatacattttgtgacatttattgagaaaagcaaacattgatgttgtaaaagagcaaaactgt
tcatgtgaatgattgtagttcctgaatgaaatgcaataaaataatatatatcatgctatttttttttgtta
tttattggttgtgacgtatgtcattggaaggaatcaaagtatagtgaagtataagtaatgaactgcttac
aaaaggttgaactgatttacctaacacttgtattgcaatagacaaggtgggtggggcccatgggttgagc
agtcatttcgattaacacaccgcaccctatcagcgtttatggcggcggcctctgcggacaagtgttgtgg
gttgacacacaactgattgtggcataggtgacttacatggagaccagcaaggttgtcagacctgctatct
accatgtaagccagcctatgagcatgagttgtgtgccatcctgtcactggatgcttatatttgacaacac
catagccatctttacttaaagcccttgtccaaaatctgcactgcccatgtggcatggaaaattgagcaat
ttttacagagtatttaagatagaactcctccatgatgtcatcaagctgagtaataatacactaagagagt
attccagtgctaactctggtatgaaagggcatttaactatcaaaatttacaaattcacaattttagagt
gaaaatgcaataaaacatccaaaatatcaaaaaatatgtgatttaaattaaaataaagtgcatttcctag
taatataaagtgtaacagacttacatgcaaaattttggcaatgtagttccattagttcttgagttattgc
aaaaatagaatttacatagccagttctatatca
>KolobokP-1_PhoLin-I
tgtcagacaacctatttttgtctaaattaaataaaacttcacaggtagtaagtacatccaattaccaatt
aatgcaccgaagctgaagtctcaggtgcagaaacgagcaactctacaggcgacttgcatcaggctctatc
acctgctcttaagggccagtacatcaggagaattggtcgtctgcagggtggcgcaagtctcataactctg
aaatgagacacgatatcattaaagagagactttttatggcaaatttcaatcaaacaccttcaaataaaca
actttttaaaaaaataaaataaaaagaaatatttttacaatttttgaaaatttcatcaattttagcta
ctaattttggcaattttcatcaaatctctctttgaaaataaaaattgaaactgctaaatttgatgtttct
aaaaagacattttatcactggtcaaaatgagtccacactcaattgactgctaattagcaattgattgcta
tgagctattaagtgtcagagctcagacctggccattagccaatacgcgtagtggtgtcaagtaggtaatc
tgtcctggcagccattacacctctgcctggtgacatttatcaattccaagaggcagcatatacaataagt
tgtcagtgcagtgacaaatcacaggtatggtgtataaaatgtgcagctaccagcagtgctgtatcgtttc
aactttgctatcgaacatgagaggagacaaaaagcggcgcagtcaaggcttccagaagggcaacattcca
tggaatcaaggtctaacttttgattctgatgatggattggatctccctcatcaaacccaacaaaagtctg
tcaccaggatgagtgctgagcagttcaaactggttgcaatatccaggtctgatgcctctattctctctac
accagactgtgagggtacatcaagctctgtacgtctgcttcgtcctttgccgcctaagcagtccaagtca
gaagacaaagctagttctcaaaactctgagggcatgcgactggtggacagtgcagaaatggacaaatcct
ggaacgagggtaaccgccttcactgtttgacgtctccggactgcggcgacacgaaccttgtgatacataa
ggagaaaaaatggggtgtctgttggaagatagtgctaaaatgtgtgacatgtgactttgaaacacctgag
atgaagctctacaaggaggtgaaacaagacaagccaggaccaaacccagcagcagctaacttagccctgg
ctgttggacttcaagatacaccaatcaacaccaccagagcgaggctactcatggctacaatggacatggt
cccccagctagaagcagcatgcagagaacagccaatcaggttggaaaggcagtgactgaactgaataat
ggaagacatggctgacaaggttcaactcgttaagcaggtcaacagcgacagaggcgtcgagttgaatgaa
gtaaacatcgccatggatggccgctacaattccacaactatagccagtagaaaaaagccaggtcaaaatg
cctccatagcaatcggcctcacttgtgaaactgtgactgataagaaatatattatcggtgcatcattcca
aaacaagttgtgttggactggagcatggctgagagggaaaggactggaagtgacctgtccaggtggacat
gcagagtgtacggcaaatatgtcaacatttgctccattttcagaaatgagaatggggaaggacattggtg
accaactggccctacagggaatacttatcaagcatgtgacgacggatggtgattcaaggtcagcggatgg
ggtggacagggcaatgaaggccattgatcccatgtggaaagtggaacgacttgctgacccaacacacctg
gctcaaggacaattccgcaggtgctacaaggccagtttcagtgctgagatgtttgaaggcaacacccgtg
acaagcgtaagcagcaacagcgggttcttagtcaggatgtcaaggcacggtgcagtcttattcttaagga
tctcatgaaaacacatgctggcgacatgaatkcactgaggaatatcttacctagggtcctcgaggcgacg
cttatgtgttataccggggattgttctagatgtgcccgtcactccgtggtctgcaccggtggcgtgtccc
ggaactggtggactcggtccatgcacctctgcaagaacaagatatcwagtttcaacatgaacgacagtga
caaacatctcctacatgaaatcttgaagatgaaactaagtgttgaagttgtagaacaaatgaaactttat
actgacactcaaaaatgtgaggcagcaaacagatctctgagtgtatctctacccaaaaatgtcaacttct
caagaaattacattggccgggcatcktcaaccatccacagactgaataatggccctgggacctccgcaat
tgaaaaaaacaagtgtctaggcatcaacttatctgcgaaatcagttcggtctttacacaagatggatgag

gatgctgactatcaaaagatgtacaataaggacagtggtgttgctaagagaagattgatwcaacaaggag
agcagatttctggttacattaaggccaaaacatccatgaaggaaaaatgtgattacagaaagggtcagct
ggatcctaaaccatctaccagtcatgactgggatcattcctattcaaagtaaccacatgcaaaataatat
ctcaatgtgaaatgagggctgggaatacttctttactatccwgccatgacagcaaactgacttttttca
tgatttctacaatttgatgawtgttttatgagtattttatatccccattggccacctgacatggtattat
ccgagtattactgaaatgtgatcaactgttatgtgatcaactgttatgactgattctatmtcacaaatac
ttatatatwtacatgtcatatggaattaaaacaaakttaacatgatatatttgcctgttattcttctgaa
catgcaagaagacaatccatatatggtttatgtccatgacaakttccgcggtttaaacatttgcatctat
cggcattgacactaggtggttctgccgacagatgcgcaaacaaaacacatmtcttgttatgacataaatg
gctgacatgtaggtkctcatcaatgaaattttcactgaaaacaatgtagctcagtctatgagcaactgta
tgccatctatttgtgacaggagatgagtatctaatgataccatagccagagggcttcagagccctctgcc
atatcaaacatttgttcactttgtagctgttttcaacaattttggcttgatattttacaaaaaatgcctc
cattttcttaacatgtgcatacactaaccttcagggtgtagtaatacattaatgttcaatatttagaagt
agtttaactcacacatggctttccaagaatatctgatgatagtataagctttgaaaattttacaatcttc
caaaaaagtgtataaaaattcaaaaattcatttctagggaaaatgcataaaatgccagatttctatatct
atactgcataggtgtaccaagtttcaagcaaaaagattgaaaactgtcaaagttattagccatcaaacac
atcaaccccctcttacagtgctttccatatca
>KolobokP-2_PhoLin-I
tataagaactcaacatatgcctagaatttgttgaaatttacctacatagtacatcaatgtatcaggattc
accatcccaaataccaaataagtcacttcacttacagcgaaatgacagtcattcttcaatacataccta
tgaccaatttgggcttcaggaaaagccggccgtccaagatttcgcacatgcgcaattcatcacagcagtg
tctcctgttgaaataaagaaacaaagtaaagttttcataaacagtggagtatcactctatcacagaatac
aagtttcctagaaataaacattaaatttgatattttagtaaaaaaaaatcaaaagtagtcatttttaccc
aaaaaaatttgatttaatacaaagttaccaaaattctttgattctcaacctgcaaaatttctattttcta
taaagaagccttttcagggattctaatgagctattacaccatggcacctgttcagtgggtatctcttaac
gaggccctaaattgtccctcctcacaggacagtatacaaacaaacaacatggcaagggaggagacagtct
gttatggcaggcaggggtcagatttgacagacatgggggtgttttcaaagcattacctacccaagggac
tcaaataactataaaagagaggtggaaaacttgagttatcattcccttccatgacttaccaccatgaaag
gcgacaagcgaagaaaagcaacacagatgaagagaggccatgcacagttaaagaagagtcaagattctaa
ctcagaagaaaccatggtacctccaagagaccaggtacaacggcagtatacacgacttagtccagaagaa
tctgccatggtcagcagatccacatcgggggcaccagtctatcggcggcgaacattgacggcaaagtct
catccatttgcctgctgcgtccaaggtctcgtaatcacccagaaaggcgagaaacaacaagacagagtga
acttgagggcaacagagtcattgatgtcaccaagatgaccgaggcggtcaattctgccctagaattgcac
cacacatcatcacccgactgccgagttccacaactagttgtgttccgggagagaaaatggggtctcgcct
ggaagataacactcaaatgcactgagtgtgatctaataacacccgaaatgaagttgtacaaggaggttga
gacaaaccaacccggtccaaatgcagcagcgcagaatgtcggccttgccgtagggttgcaagacacacca
atagggaacagcagagccagacttctgttggctaacatggatatccctcctccctgtcacagtgccatgc
agagaacatctaacaaggtctccaaagttgtcacacagctcaacacccaagacatggcagagaaagtgaa
cattgtgaaaaaaatcagtgagaagatgggaaacaatcctaatgaaatgaacattgcaatggacggtcgc
tacaactcaactacaattaccagtcgcaagaagccggggcagaatgcatcgcaagctattggtctagcct
gcgaaaccattacagaaaagaagtttatagtggccgcaagtttccaaaacaagctatgttggacaggagc
ctggttacggggcaagggaatagaagtcagatgtccggacggccatccagagtgcacagccaacatctct
gcatttgctccactctctgagctgaacatggggacggatatcggcaatcaacttacaaggcaagggattc
tcatcaagtatgctacaactgatggagatggaagatctgcagagggcttagacaaggcaatgagagtctt
agatcccatgtggaaggttcagagacaagcagaccctacccatctagcccagggacaattccgacaatgt
tataaggcgcagttcagtgctgacatgtttcccggggcgactacaagggatcagaagatggctgcaaaga
ggacgttaagtcaggatgtgaaggctcgatgcagtcttgtactcaaagagctgatgaaacaacatgctgg
tgatatgaagtcgctctcaagtatcttacctagggtgcttgagacaacactcaagtgttatgatggggac
tgctcactatgtggccgtcactcggtggtgtgtggtggtggggtgactaatagctggtggcaacgctcca
tgttcctagcctgcaatggaattgaacagttaaatatgaatgacagtgacagacatctacttcttgaaat
tctaaagatgaaactaagtgtacaggttgttgaaagcatgaaactttatacagacacacagaagtgtgag
gcttcaaatcgatcgttaagtgtgtcagtgccaaaaaatgtgaactttccaagaaatatgactggtcgtg
cttcatcagccattcatagactgaacaacaaccagggatcttctacaatagccaaatgtgaggccacagg
tgtcggactctcttcgcggtgtttgcgtacactgaccaagatggacagagaccaagagtatcagaaacaa
tatcaaaaggacccatccaatgttaaaagaaagttaattaagcatggcagagacattgtcaaacattgtg
aataccggaaaaacaatggggaaaagtctgactatcagaaaggacagctagatcccaaaccatctcacag
ccgagatcatccctactcccgatgagtctaaacaatattgtaaatatctattaatatattgtatatattt
atatatgtatatatcctaatacattctatgatatttattgagaaaagcaaacattgatgttgtaaaagag
caaaattattcttgtgaatgactgtagttcttgaatgaaattcaataaaatactattttatgctattttt
ttttttgcttatttattggttgaagaaagcaaagtgcagtggagtatgagtaatgaactgcttacaaaag
gttgaactgattttcctaagccttgtaatgtaaaaggcaaggtgggtagggcccatgagttgagcaatc
attccgattgacacaacgcaccctatcagcatttatggcggctgcctctgcggacaggtgttgcgcattc
acgcacaacctattgtggcacaggtgactgacatggagaccagcaaggttgtcagacctgctatttacca
tccatgccagcctatgagcatgaatggtgtgccaaccagtcactgggtgcttatatttaataacaccata
gccatctttacttgtacccctagtccagtatctacactgtccatgtggcatggaaaattcagtaattttt
gccaaatatttaagataaaattcctccatgatggtaacaataatgatgtgagtaataatacatttagaga
gtattccagtgctaactttggtatgaaaaggcatctaacaatcaaaatttacaaatttaaaaattttaca
gtgaaaatgcaataaaacatccaaaatatcataaactaagggattcaaattaaaataaagtgcatttcct
agtaatttccagtgtaactgacttacatacaaaattttgataatgtagttccgttagttcttgagttatt
gcaaaaatagaattcacatagccagttctatatca
>KolobokP-1_OwFu-I
ataatgagcgcatattttcgtatgcaacacgatccaatatgtttatagcaatttttgacaattccgatca
atacgatatgcagaaatatggtattatgaataaaaatggatataaaatgccccctgaaaatgcctacata
aattggattttgataattttggggacttgagaaaacatggggtaggggagtggaaggagcattgtactga
tagctgttttcaattggtttaagaaaatatatacatatttggtaccattttaaagcttatatatattcct
ttaatttggtactgatattatgacatcatatgacacagtgtgacgtcataatggtaccaaatgatgatta
tgtctattttctagcatttttaggcattatttgaatgtttccaatcatggcaacttggtatccatgagac

taattttaccagccagattctctgtatgacatcacataatatttcatatttgatatgtgtgacatcatat
taatacacatatgtcatcttcttgacaacagctactctcaaaacatccaaatatcaacaaaatgcctttt
gaaaaacagagaaaaaagtcagcattttccaagggacatgcatacatacctggtaggagatatggagatc
tcaaagaaaaacacactcttccatggaaaaggctcacatatgaggaccaggactgcattgttcagcagga
taaagatgtactgaggaccccattactggatggcacggatggcctaaaactactgaggccaaagccaaaa
tcaccagaccatgaaaatactgacaatgatatttgccatgttatgagtgttaaaaaagttgaagacctgt
tgtctcatgtagtaaggcatactaatgaaagcccaaaatgtcacaatccagccataagaatatacacgca
aaaatggggagttacgtgtacattaacgtatgagtgtactcgctgcggttttcaatcacaaaacttcaaa
atgtataacatcgtaaagactcagaaaagaggacgaaaagcagccgtgaataacattggacttcatgctg
gcattaatgaatcaacaattggcgtaaaaaaggtacagatagcatttgcggcctgtggaattgagcctcc
aaacacaagtagcatgtacaagacctcaaaccaagtaggatcagctattcttaccgaggcaaggagggac
atatttgagaaaatagaatatgtcaaaaagttgaataaccttaaaggcgaaagcgatactaatgcagtag
atatttctattgatgcaatgtataattcaaatcaaatcgcatcaagaaataaatacggccagagtgctac
acaagcatacacaacagttcgagagcacaacacgaagagaaactttataattggtgcccatgtagcaaac
aaattatgctgggtgggagcgtatttacgcggaaaagggtatgaaaatgtcaaatgtcctggtcattctg
gttgtacggcaaattatgatagagcaacaccactttcggaacgtcacctggcttatatgattggaagaaa
tttatcgcacgaaggcttgaaccctaatttggttatcagtgatggggactctcaatccataaatggtctc
aatgacgcgattcctgagaataaaatgattgcacaaaaagaccctattcattcagcccagtgccaatttc
gcgcagttcagcgagcagaattttctaacgacatgtttggtttgaacacgccaaatatatcggtaggtga
aatcaagaaggtactaggctccgatatcaaaatgcggtgtaatggggccatcacaatgatacgaaaacaa
ttcgaccaaaatttaacattcgatgagaatatcaatttaataagtggaaaaatagcatcatccgctagcg
ctatcgtgaaatgctacacaggcgattgttccagttgcggacagcagggtgtatacagccaatgcgacgg
agggaaaaatacatggtggaataaatcttaccatttcaaaaatgtaaacccaccgatcataaagggcagt
ttcagacccgataaaatagacattgaaattcttagagacattattgatattctaatgtcgcgagcttctt
tcgaattgatgaaatctgggaaatcaacaaattccaacgaagcagcaaatagggctatcaccgccagggt
cccaaaaaatgtaagatggtcacgaaatggtaccagccgtgtttttgggacaattcacactttgaacaat
gggccgggagtttcgatgtgcaaaaaattgaatgcaatcaatattcctatagcaggaaggatatcgaatt
ttttcaaacggatacagaaaaaagccctatacaaaagttcctggcaaaaaagagactctgtcaaagccca
acgggtaaaacgcagaaaatcgcaaatgtcagatttttataatggacggggaaaacggcaacggtcagac
tataaaaaatacaatgtacctacattaaaaaggaacaatgatcatacatatcaacaagtagaaataaagc
aaaaaagggctaggcttaataggcaaaaacatcagtagagggggtcttacggtaagtgaatctaagggca
ggtaggggtggggcttaccccatacctgcccttagactcacttacagattacactaagggaacctaagga
aagaaggaggggctactgtaaggggatctaaagggagggaagggggaggggtgtattgtgtatgactgca
tatttcaataaggcttttatatggaaaaatgtatttttgtacctgtatacacataataaataaatatagc
aaaatgtctattttaaaatgcaaaatggttcgtcatgatttttgctgcatcgcctgggcattaaaagttt
acagggctgcctggccttatttgttgaatgggactctaaaactaaatgtgttggattcacacaatgctta
acatggcacaaatgtgagcattcctttgaatcatcacttacctcaaacaaaaaatcaaatctttttaact
taaaaattactgaaacttgatgggcatataacgtcttctttggaatattggaactcaaaggaaatgaaat
ggaaaccctaccataccccccgtgtagtgggccctgccaaacccagcaaccattactgtcttcaattgat
cctgcttgcaatattttttggagatttttaaaccatgctggttcagccatcttgcaaaattcaaatttcc
cagaaaaaaactttaatctcaaaaccggaagtgaatttttggagcaaaaaaatatttttttgcttgtttg
gggtaggggttggtactgccatatcatattggctgcaattatgtctattttttagagaaaaatgttttgat
ttggtatactggatagcatacagagcaaacttcattttgaatttcattctatggaaatttgatgttctct
gtgatgtcacagagtgcttaccttgccagaatgggtccaaaacagctaaaataat
>KolobokP-1N1_OwFu-I
ataatggggacatgttttcttattcagcactagtgtaactgtttttaggtaattgagacatttctgataa
aaatggtatccagaaacatgatattttgaataaaacggggccaaaaaacgacccctgaagatgcctggat
tatttgagattttggtgtttttcactggcctgaaaaatggatggggtaggggagtgacacaccttttggg
cttattcaagttagcattttaatcaaatgaatggtcacatgtttggtaccatcttaaagcttgtcacgtc
cgcttcaatttggtacccatatcatgaggtcagacgctatattgtgacgtcacgatggtaccaaacgatt
ataaagtgaaaattaagcatcaaaatgcggcaaaataat
>KolobokP-2_OwFu-I
taatagtacaaccattttgtttgtttttcttcaaaatggtgctatttttattcttggcatttttctgaac
acaatgatatatgacaatgctatattgaagccaatacaagggggaaatcaatatttaaaggcacaggtc
ctttaagctaaattcatatatttttggtaacgaaatatcttgtattttctttggggtaggggtgcaccat
gtaaatttgggagatgtttccttacacttaatcaagttgaatatagtcatatagggtatcaatggaaagg
tcctttactcacctttataataatgcccatttttgggcatttgctaatatattatgacgtcacaattgcc
cttacaaaaaagttacactcaaaactgcccttgaaaactgaatttcacaggagacggaatggagggatat
tatgcaaggttgacacacaccaaaaaagcctaattaggtaaaacaatattatatgttgtcatatgtggtc
ctattgctccataattaagcctacctttacattctgcagcatcaatgatttcagaagcaggtgacatcta
taatgcagaaaatgaagaaattctcaaacctacctgttgtataaaaatgtactactgccaatggcctctc
taactgcaaggctctggatgtacactctggcaaaacagactctggcaacatgttctggcttcagggtctg
tctagatgcaggaatctagggaggtaatatagagataatattataaatatctaccaatgcatgccaatga
gcttaagattaaaaaaaatgagataaaagctcttcatagcatgcatacatagaccagattaataactagc
aatgctttccatttcttgtaactattgtattagtctggtaactattgtattagtttttttcaatttctcaa
tttcaactttcaactatgatgtcataatgatcaatacaaattgaatcagtttgtcccgagaggatttgaa
ataaagtgatttctggcaaatatatctgtgtgttatttcaatttggatatactacatattacacaatgtc
aagagatttaagtagtacacataaaggattggcttacaagaaaaggcaaagtcctgctggtaagaagtca
agatttacaaaaggacatgcacacatcaaaccatttaccaaatctaaacctttttcaggggatcattccc
attccataccggacacaatcgagagatttgatgaagatacattccggaatgtcgctacaaccagccgtga
taatcaacgctatgagaatctcgatgtgaatatgaactttggaaatgcccgcctgctaagaccgtgcaaa
agccggaaagcagatctcactaacaccatggccgaagcacctcctggtgcgacaccgaatgactgtatta
tcgtgagtacggcgcaaacacttatcatgataaacgatttaaacaatgaacacagaaaacatagtatcgc
gtgtgaagggaactataatatatcacgaatcgcttcatttggtttgggcacaaaattaaagtttgcttgt
tcatcctgccattttcaatcaaagttgtacaaaacatatgatgaagtagacaatagctattacaaaggta
acatcaagggccctagaccagcaaagacaaatgttgcattacaggtagcacttaagcaatcttccattga
ttccaagcaagttagaagaatttttgctgccctaaatatcaaacccccaagtaaaactcatatgtttcaa

acaagcaaaaaggtagatgaaagaataacagaactctgtcaacaagatatgagcaagctaagacaaaatc
tcaaggaaataaataaaatgagaggtgtagctgaacaagatcaaaataaaatagccgctgcatttgatgc
tcaatatggttccgtaaaatttggctctagaaacaaacttggtccagatgccaacagggctatagggtta
actgttgaaaccatgacagaaaatcttgtaatcattgatgccgttcttcaaaataaactttgttggaggg
gttcgtatatgcgtgcaaggggattcgatgttaagtgcccagggggcatgagggctgcacagcaaccat
cgagaaagcagcgtcattcaaagaacgtcaaatgggtcgatctacagcaaagacatgccaggaagacggg
attgttattcagtacctcacaacagatggagatgggaagggaatcatgggagtcaatgatcactatgcag
agacaggaatggaacacagagcagagcgcctcagcgaccgcattcacttgagtaatctccaattcaagca
ctccctaaagacctcattttcaagtcagatgtttccgggaaaaaacaagtcaactaaagaacactatcag
cgcctattttgtattgacctaacccaaagatgcaccgctatccttcgcaaactttccctcactgataggg
acctctctaaagtctccactttcaaaatcatcaaaatcactgattctctgatacaatgttaccaaggaaa
ccatacactctgtaagcaagacctcaatgaatgtaaggggaggaggagcaacaactggtggacgaactct
tacaatctaaaggcttgcaacctcaaagctactgacataaatatggacaagaaaaatgatgaaatgtatc
tccgccttctgatagaaatgagattaggtccagaagcccttgctagcggcaaattttacacccatacaaa
taaaaatgagagcgcgaaccatgttctatcaactgttttacccaagaatagagtctttccttcgacggtt
tcgggtagaatgtccagtgcaatcatgcaacttaacagggggcaacaaatggaactcacctgatgcttc
aatgtgttggtgctccagtatcagctggctcccgcgcagcaaaggaactgaaaaaaattcaaaaagacat
tgactatggaaagcaatacaaaaaaacaacatccgcaaagaaagcgcgtagataccatcggtcggtacag
ttaaatcaatactgcaaaatgaagtcatcagagaagaaacaagaatataagaagaatcaattagatgaag
taaggttaacaaggcaggggtgtgcaaacaggccagagttaaaagatcatgcatatgctgcagcaaaggg
aaagaaggtatgttattactacatgtattctacagaactacattttgagaagagtagttattgttattgt
gcctaccttctcagaacatatagagatgtatcatgcacaaaggcgcgtgaccccccaaacacaccccata
attaacacatgcagtcctaccattattgacccccctcccctctaaaactaagtgatctggattaatacaa
gaactattgtgacacaagtgagaaacatctaaagtactatctaaatctaatttatcccgagaaatcatat
acataaccctgtgaaccctcattgaaactgacctcccatccaatttttttactttaatcctcccataccc
tgatgaatctagacacccctcccacaataaacactgcccattgaccccctaaaaaacatctctcttgaatt
cttgctttgtacagtgtcttccactggtccggattcatgatgtgctgcttaaaattaaggaaaattttga
aataattatgaaataaaaatgaattaaaaatcttaccatctttttagtgggcgataata
>KolobokP-2N1_OwFu-I
taatagtaaactgaattagtttgttttctttcaacatggttatattagcattgtaggcattatcccgatc
acaatggtatataacaatgctatatttgagaaaatgcacgggggaagattcaatccttatagacacaggt
ccttttagcaaaattcaggaaatttgattttgagatttcttgaaatttctttggggtaggggtgcacctt
acagtttgcatcacagattgagtacacccaactgaggggaacatggccatatggggtatcattggaaagc
gatttctactatctttcgagtgatatccatgtcaaggcgttcaattgcatattatgacgtcataacgtta
gggagaaacaaaggttcattcaaaactgcccagaagacaattgaatggcacgggagaatggaattcgggg
tacttggcacaatggcctcgcccccaaaacactaaagttgcttcaaaatgtcacgattttggtaccattg
ttcagctaagatacagtcctttaatatgatatgcgctattaggcttcttaatgaacttaaaaacctcaaa
aggggacaaaacctacaaaataccccaaaaatgcgccttctaatagtaaactgaattagtttgttttctt
tcaacatggttatattagcattgtaggcattatcccgatcacaatggtatataacaatgctatatttgag
aaaatgcacgggggaagattcaatccttatagacacaggtccttttagcaaaattcaggaaatttgattt
tgagatttcttgaaatttctttggggtaggggtgcaccttacagtttgcatcacagattgagtacaccca
actgaggggaacatggccatatggggtatcattggaaagcgatttctactatctttcgagtgatatccat
gtcaaggcgttcaattgcatattatgacgtcataacgttagggagaaacaaaggttcattcaaaactgcc
cagaagacaattgaatggcacgggagaatggaattcggggtacttggcacaatggcctcgcccccaaaac
actaaagttgcttcaaaatgtcacgattttggtaccattgttcagctaagatacagtcctttaatatgat
atgcgctattaggcttcttaatgaacttaaaaacctcaaaaggggacaaaacctacaaaataccccaaaa
atgcgccttctaatagtaaactgaattagtttgttttctttcaacatggttatattagcattgtaggcat
tatcccgatcacaatggtatataacaatgctatatttgagaaaatgcacgggggaagattcaatccttat
agacacaggtccttttagcaaaattcaggaaatttgattttgagatttcttgaaatttctttggggtagg
ggtgcaccttacagtttgcatcacagattgagtacacccaactgaggggaacatggccatatggggtatc
attggaaagcgatttctactatctttcgagtgatatccatgtcaaggcgttcaattgcatattatgacgt
cataacgttagggagaaacaaaggttcattcaaaactgcccagaagacaattgaatggcacgggagaatg
gaattcggggtacttggcacaatggcctcgcccccaaaacactaaagttgcttcaaaatgtcacgatttt
ggtaccattgttcagctaagatacagtcctttaatatgatatgcgctattaggcttcttaatgaacttaa
aaacctcaaaaggggacaaaacctacaaaataccccaaaaatgcgccttctaataggttgaagaccacag
tagagtttcccataggtggccattataacattttaccctgtatcttattaacgattagtatccaagccta
gtgcaacataccacagtgttcgggagagactcgcctagactgctataacaatcttaccatgggttgtcca
ccgaatttttgagataaataaacttgaagacactaacccccctctatcgtcagatttctcagagaattctg
gtaaaatcaccaaggtcaaaatcactccgccgccagcttaaaatgagatttcccactgaactttggcatg
ttttcagataaatcaggacctaccttataaatatttagtcccaattcacttcccaaacgccaaatggcgc
tccaatggggtaggaaaggagaaaaatgactttttatacagaaaaccaaatcactaacggtaaccgctag
tgtccttactcaggactatatcctaatagtaaactgaattagtttgttttctttcaacatggttatatta
gcattgtaggcattatcccgatcacaatggtatataacaatgctatatttgagaaaatgcacgggggaag
attcaatccttatagacacaggtccttttagcaaaattcaggaaatttgattttgagatttcttgaaatt
tctttggggtaggggtgcaccttacagtttgcatcacagattgagtacacccaactgaggggaacatggc
catatggggtatcattggaaagcgatttctactatctttcaagtgatatccatgtcaaggcgttcaattg
catattatgacgtcataacgttagggagaaacaaaggttcattcaaaactgcccagaagacaattgaatg
gcacgggagaatggaattcggggtacttggcacaatggcctcgcccccaaaacactaaagttgcttcaaa
atgtcacgattttggtaccattgttcagctaagatacagtcctttaatatgatatgcgctattaggcttc
ttaatgaacttaaaaacctcaaaaggggacaaaacctacaaaataccccaaaaatgcgccttctaata
>KolobokP-2N2_OwFu-I
taatagtgacacggttgaatgattttgtgccagaaatcttgtttttcttaaacatwagagtgtttcaaga
ctttttgtgcaatttaagcccaatcgcaccaatagaagagcttttatagctgctttgttaaccccggggt
catttattttagggccatgcagttttatacctaactgaccatttttgcaatagaaatactgctactcgct
atggcttaacaaaaattcacaatatttagcatgtcaaaacgtttattcgcacttccataatgtttattgc
caaaatcactgaaccatgaaacaaaaaaatggtcaaatctagtcatttttgatcagagattaccccaaat
tagccattatttagtgattaattttcagtaaatgagcttctatattaaattatcatatcagttttgcata

gcaatgttcttgttttaattacatgcatgttagtaactgttttcagccatatttagtcagtctatkgggt
taaatgtgtcaawtttgtcatatattttcaagaaaaattcagacacaaaggaaaaagctgtattacatgc
tatgataatggctaaatccttaaattcggtaataatttgataaaagcctgtcaacaacctcttaaaacca
gcactaacagccctcttgtgctatcctgtaacaaaatacaggccaaaaatggtcaaaacctgtgattttt
aggtaaaaaatgaaaaatgctgaatcagcgaaaatctaaattgctaattcttcggataatttgatttttc
ttagagatgcaaggtacacattgctaagaaagtgtaatttttacaaaaataacgctatttcccaccctag
tactgtcaccaccctgttgctaata
>KolobokP-3_OwFu-I
taatagtcacacctggttgatccctggcaccaatgtcctgtgtgaggtgaatctactgtcttaccttaat
aaaagtccataatgcttttaatcctcctccatgtttatctatgtaaaaaaaattgaattctgacttaccc
tcacatggaatatttggttgtgctgactggttaaacactgattgtgctgctgttgtctcaacttctatgg
actctatcggtgtatgtactctcagttgagatgttgtctgtatgatgtgatgtaggaacaagtcacggct
actgaaaaacatgaaccgaaaaatgagttatgcaataactaattaatatgcgcataattaatgagtattg
tgtttttttcattaaaattgtgatatattcataaaaaagctgatctataaatcaggtaatttgaccaggtc
tgctttaaatgggacaacaatgccttatctagcaaataaatcaagatacagtttgtttacatatactgac
aatgtactgacatgccatcttttgatctaggggtgatttatctcaaactcaaacagtgaactatttattg
tgttaagactttatcattcttcagacactttttaaacataatctggaattattaattggtcagatggct
gcccaagtcacccagaaacacatctcatgtattatcaaaccactactttcacttgaaagactggacttag
agaggagaacatcattctgctttggattgttccaagttatcaagcaatgtcaactatgtcaaaaccacca
agaggatttggtcatttttattccaaaaataagtttcgtaaccagcaaacattttctgaaggacatacgc
agcatccggcatatgggacggtagcgaatcgcgaagaggaatcgtccccacctacaaaaattaggaggct
agatcaggagactcattcgttagttgtcaggaaaagtagaggtgggaatagttatgaagtacccgatgtc
cacatgcaacctggtagaggtaagattttaaggccaaaactttctgaaccaaaaatctcagataaataca
tcactgatgaacccctagatggcactgttggtggagatgaaactctaatagttagtcagaattctatgat
taattgtttcaacttggtagcaaaagatcatagggcccatagcaaaaaatgtagaggtgatttgaaggta
cataaaaaaattccatttggtttagtttgcaaaatgcagctttcatgtagtaaatgtgggtttctctcca
aaatgtgcaaattatatgaagaagtcaagggaaaaaagaaaggtggcaggaggaaagctaagatcaatgt
agcaatacacgtagctctacaatcctcctctattggtgttgaggcatttcgaaaaattttagctacttgc
aatgcaagggccccttcttcccaaggaatgcaaaaaaattctaaagcggtcagtggagatattgagaaaa
tgggtgaaaatgatttaaaaacgagacgtagcaaattaatagagktgaataaactgaggggtgtatcttc
aaaagatgaaaaaaaagtccaggtagcatttgatgggtgttacagtagtagcacattcggatcttggggt
aagcttggtcagcgcgctaccagagcagttggtgtatctgttgaaacaatgaccgatgccatgcagataa
tatctgtatgcttgtataataagctatgttggcgcggggcttacctaagggcgcgtgggtttgatgttaa
atgtcctggtggccatgatggttgtacatcaaatataaagaaatctgaagcttttcaagaattcaacatg
gggtacaaacttgcaaaagatttggatgcagacaatttgcaaatagaatacctgactacagatggtgatg
ctaaaggctcccttggtgctcagtcattttacgatgaaaaacagagcaatcacaaagttacaaaacttag
cgatagagtgcacaaagggcaacttcaatttaagcacagtcttaaagctagctttagtagacatatgttc
ccaggtaacaaagatctccagcagaaaatgttttccctagatataaaagagaggtgtgacaaaatcatgg
gtcagcttgccaaaaaatacaatgatttgggtgttgtatcagttcaatacatgaatagggtcatcacaag
tacagtgaactgttacgcggggaatcatgagcaatgtagaccccaccttaatagttgcaaaggggctaag
aaatcatggctagaaacatcccacaatctcaaggtgtgtggaattacaaagaatgacaaattgatgccat
ctcaggaagattgcaaattcatacgcgtacttttagaaatgaagttgggccccgaagctgttaatgacgg
gcgcttttacacgcatacaaacaaaaatgaagcatcaaataaggtaattgcagaatatttacctaaaaac
aaggtgttttcagccacatgtaatggcagattacattctgcagtattagatataaaccatggaagagaaa
atgccactcacctcaaattggaacttattggggccccaatatcatcagcaagtgctgcaaagcatctacg
tagcatgagtgaaaagaaaagatacatgagaaaatataaaaattccccacatgcaagaaaaaaacaagtct
tttcatagagcatactacactagacagtatttacacacaagattagaaaagcgaactagtgattataaga
aagagcaactagacaaaaaacccattttaaaaatgaaattgaggagatcaccaaggttcggtgatcacga
ttatgcccaagagattatatgaggtaagtgatctaatcataccatttacattttccattttaagaggcaa
tcagggtaggccccatgctgacggcattttccggcattgatacaacccattcgcacattatttacagaat
gtggttctaggctaaggtgatttatgttgatacatagcttgttatgacaaaggtgagataagtccagtgt
cgataaatcaagcgtatcgttaggatgccgtgtggcatacgacagccagtgagccgacacggtcacccat
ttttgactaccgcgcttctgtatgcacaactgcccatacccggtagccttgttacgatagcctgtccaaa
taatgcagttattggggtagactgttgcctcccggcccaatctctgggcatattttgcccccccaggcatc
cattttggcctattttacaagcaagaaacaacttatatgttatacaagtgagataagatagaatgaattc
tgaaaaaatcaaaaaaatcatttttcttaccaaattgtgtttccaaaaatctgaatatta
>KolobokP-4_OwFu-I
ttaatagattttggcatgtttccaattatcgagataattcatttttatggttgattgcactattctacac
ttgaaacattgattttaagtccaattttgatgtaggaaaaaaaaattttgtaaaagggggggtgtgaagc
gatttttgataccccgaaataccctaaaatatgctatcaaactttgcagcgatgacgtcacaaaagtaa
cctgactttctccgagattattgttgattattaagcccaaggtaagtatatttcaggatatgacatccat
taacactggtaagtgtttcatctgaacgaaattttaacttttaaatgcatcttgtctgactttttttccac
tatttcctccattctgaccaagtgctttgacgaagcatgcaaagctctgtgtagatgacgtcatacatac
ttatttagtcatatgtattatcaattaagtttctttggaaacctaaatcaataaactaaacagttcaagc
tgtaaaatggcgaagggaagtagaaaaaaacatacatttgagatcaataacaagcattgctacaacaata
gtggtcaggccagaactagaaatacccagccaaatgtcaaaaatagattcttcagattggacagtgatac
atctctaagggcttgcggtattccactcagcagcctgagaccagagaaaagagatgtgtttttcactaaa
ttgagaacaagtgtgaacagtcgcacagtcaagggaaacagactcgttaatatggagcagctaatggtaa
tgatcaatagaacaatcaaagatcatcaaaccgttagtccaaaatgtaaaacgcctgagttttgcttacc
tgccaacaaagaggagcaacaaggcttaggttatgcatacgcaatgaaatgcaagcagtgtcgattcaca
tcatgtacatttaagacttacctatcaatcggaaagacatcaaatcaagtacctggacgccaaaaagcaa
tgtgcaactaccagctggatacattcatgttgaaaacatcaataccaatggatgacatcagattattaat
gactatgttagatatgccacctatcggaaggtcgaccttgcaaagattagtagatgcatcatcaaaaacg
tactctgcccttaatgatgaccagaatgtgaaaaacggagagcttgtaaaacgtattacagagtcatcga
gcacaaaagagtgtgttgtacaactggacactgccttcaacaacaacccaaaaggccgtgctttttctca
accgggaacgcaaagctatacgccgttgctacatgaagagaccaatctaattctggcgaataaagcgtac
aataagctatgtagcaggcatgggctggcttgtaaagggaagatggatggatgttccagtgcaaatttcg

aaaaccagaaagcgataggtaattcagagagtatggctgcatcagttctcataacagaggcttctcaaga
cttaactatcactcacgctaccacagacggtaatacaagtgtgaaaaagacacttacaaacatcaacgtt
caacctctagaatgcgttgtgcacaaaggccgtggtttgaagagaaaattttattctcaaacatttagtc
aaataatgattggcaaaagcacggatcaaagttatggatacaaaagggctaaaatgtcagaatatctttc
aagtagatgtatctcagagctctacacagcaaggcgaaaattttctagaaaagatttcaaatttttttgag
ttgatcgaaaaaaaacgcacgcatctccttcgctgtataatgggtgaccacactcattgcaaaaaaacat
cactgcaatgcaatgtgaaaacgattctcaattacttaccttgtaatcaatatatacaagcaactcctgc
tgatatagcaaagctacaagcatgcattgactatatgttaaatgctgatacgataagacaaatgttcaaa
acaacaaacaccaacaagacagaggcttgtcatttgaggacacttaagacattgccaaaatcaaaaacgt
cttctaaaaatgtatatggtcgttttgacagtgcaatgcactcccattctatcggcactccaaagtccat
aatcatggcaaatgaaaaaatcggtgcaaaccttacttgtaaaaaggctgtgatcaatttggaggcaatg
catatagcaaatggaaagaaacttacccttcaaaggaagtgtaaaacaaagcaaaggagagtttctgcaa
gaaatgctaaaattggcctcaaaagactagcaactttaagtgttgaaggggcaagactagggaaaaatgg
aacaatttctgatcacaattatgtttagaccaataaatatctcccattaattctataatatatgtgttgt
tgttatttattctctagccttaccacaacgtagttgatcttaattgcccctatttctaaaaatagctaag
cacaagtgccaaagggaaaggatcaaaactgtaaagaaacaaagtgagaagaacaaaatataaatgcttt
taaatgtaacattggaaccctctttgacaaactgcgaattcactctaataaacattttgggccatcatga
ccaaagcaaatcccattcatgaagcaaacttttctgaggttgttattttgggggactcggcacagagat
ggtctatgttgatacacgttttagaatggcatagatgtgagatttgttcatttctagctaagggagtgtt
aaaatgtaaaatgtaaataagctgatgacaattcactggccgagcccccccaaccccgatacgtcactttt
atttggccataaccctttccacttttagcttttatccaatttttacaagtaccgacattaaccgttctgt
ctgggtgtgtcagtctctccagcaaggtatctctgtactctctgctgatattttctatcagttcatttat
acttgtgtgctaaaaaagaaagataaattaaatgaattttgttaatcaaataaaaatagatgttaatcaa
aaaaaaatcacataaaaaacatgaataaattaggaaacgtaagcttaatttggggtaatttaatataaa
aacgctatttctggataccaaagttactgatttttggccaattattaggttaaattaccatttttgggca
tgaaaacgtttcgatctatcaagaaatactgaaattatgagggttggaacaccactatacagtgtcagac
tcctgaagggaacattgaacgcaattcagaaaaattcgagaaaagcatgctatttttgctatctgagtgg
gtgttcaattgacttatattcactgtaaatctactattttgaagcatatataggggcaatggtcgacaaa
agacctttaagtcatcatataactgaatataaatggatacaatacgatacaagtcgggaaaatgtaaagt
tttgtacttacatccatgttgcagacagtttcaaggtcgaatttgggctcaattaa
>KolobokP-4N1_OwFu-I
ttaatgtgttatgacatcacactgatttttgtctctgcaactttttttggcagaaaatatcatttctagt
tcaaaaatgcataaaaacgtcggaactggctgaatatgtgaaaaatttatccatttaggctagaaagggt
taatttgccctaccccaaatacgtctaaatatgctagtctaactttgccactgtgacgtcacaaatctaa
cctgactatctctgagtttattgaagaaaatgatacgtctgacaaacagctttcattccctatatggcac
accttctactaagagttgcatcattgactaaatctaagacaaaggccctgtgtcccgaggattttgactg
aaatttggccatttcgcccaaatctaactaaattgcagtttccctcacacctgtattttgaaataaata
ttgctacaatgtagcaatttaaaaatcacacatgcttactgtttctgtcgacctgacacgtgtatctagc
ctgtaaaaatgaaaaatcgctacattgtagcaatttcactgagatatgggccctgaaaatcatgtaattt
tagcaaaaaatgaagaaataacctgcaattttgacggaatttcagagggctttctttcattccagaccct
ccacgggcctcttatgaccattcttatactcacagaatggttgaaagtgatttcaaatcattgtttgccc
tacgcagaacgaaaaaatcgattctgttctcaaattctgctagaatattggtctctggccaactagcaga
caattttaggcctcatattaa
>KolobokP-5_OwFu-I
tactagtgcccacttatagctaccacaccttaaatttacctcattctctatcactggctctttttttcagt
agaacacagcaaaccgttccccttttccacaaatatatgctgagatatggggttttgaagttactaacct
aaaatgacaacacttcccaagaatacaatgtcaggcttagatatatactggttggcttggtttctggtgg
accatggattggctataacttgctatgtcagctgctcccaaggtatgatgactcctatatatgcgtgtga
tctgaaaataataattaaaattcaattaattaacataattaatttatgcgcataattaattaggcgcagt
gtattttctaagtaaaatttgctttgtcagcatattttgggataattaatccattatcttattcaaacaa
agtttatacacaactgatattatcttaacaattaaatgataccattagataacatggaaatattctaaat
ctgatagcagagacatcttttaaacctataaaaacctgatttcaacatagtatcttactgttctagcata
tgttttatgtaagcattaattataacctatcatgcgacaaaaccaatttatccgcgtcgccactctgata
aaaattcaacattctttccatttttgttggacattaaacaaaaaatatataaaacactggtcattctgaa
gctagcttctaacaaagacacactgctctaagattctgttcaatctacaactttgtctaagttttggata
tttaattctttaaacaggtcaaagaatgaagggaaaaggaaagtcaggccattctaaatatgcaaatgct
aaatcatttaagcggcatcataaacgtcatgaaatacctcaggaagagtttgagattgagccacctttaa
aaatcatgagattgagtgagaccaatcataagctggttgtgaaggaaaagcaaggaaaatatgaacttgc
tgatattcatggtaaacaatgtagggctatgctgttacggccaaagaaagatgatcatttaccacttcat
gagcagtatggaacatcaaacccaacctcacccccagatgaggccctgattgtgagtcagttttccatga
tccacatgatgaatgaggttaatagggagcatagggtttataatgagggttgcatgggcaacctttccat
ttatgagaaaaaaccatttggactatgctggaggatgaaattgaaatgtgaggtatgtctatttatttct
gaaaattacaaactatatgaagaaattgaagcagaaggaagaaaaaggggtccaaaaccagcaaaaataa
atacacagattaatgtggcacttctgaacagttcaataggtattgagcaattccgtacaatattggctac
gtgtaatatcagacctccagctgtatcaggcatgcacaaaacagcagtaaaagtgagctcacaaattgaa
aaagtattagaaaatgatttgaagaatagatgtgacaatcttgtcagattaaataaattcaagggagtta
aagcatctgaacaaagcaaaatttcaatttcaacagatgcagcctatgacagcaatgtatatggtgatag
atccaagatggggcaaaattcaaccagagcttttacaatagctgttgagaatcacacccaagcccagcaa
gtcatagggtattgcctgcaggtgaaacactgttggagaggggcttatttgagagctttaggccttgatg
tgcaatgcccgggccatgaaaattgcactgccaacaagaacaaagcccattcttttacagaatttgaaat
gggtggagaaattgccaaacaactcaaggagtgtaatttaacagtagaatatttaaccactgatggggat
gccaaggggtctcaaggtgtacaggcgtattacaaagataataatattgaccaaaatgtatcaagattag
gggaccgtattcacaaagaacacctacaatataagaaatgcctgaaagaagaatttagtaggaaaatgtt
tcctggagcaacaaagaaagaaagaaaaacaatacagcaagtattttccagagatgttaaaacgagatgt
agcatacttgtccaaaaatatgctcaacttaacaatgacattacattagcaatatctacaattgaaatgt
tagaaattcaaaaatccattgttaactgttacaatgggtatcatgaatcatgtaaggtttctttcaatgg
gtgtaaaggccttccagataacaactgggtgtctaactctgcaaatttcagagcatgtaaattaaaaaat

ggggatatcaaaatgaccactgatgattctaagcgcttgatgattttattaggcttgagattaggaccag
aggccctctatgagactagattcaacactcataccaatacaaatgaatcagcaaatagtgttgtcaataa
ggtattaccaaagaggaacacttatggtgccacagcaggtggtagaatatcatcagcaatacaccaaatc
aataatggactaggggacagtactcaccttaagctccaatttgtccaggctccgctaccggttggctctt
ctgctagtgtggccctaaaaaagatgcaacagaagtcagtttacataaagaaatataaaacatcaaggaa
agcaaggagaaatagacagttccacaaacaatatcacatgagagaatatttcagagcgaaagaagaaaag
aaagcaatagactatcagaagaatcaactcgataaaacgactcgattagaacacaattacacaaagaaca
agaaatcaagacccaagaggctgtcaaggtatagggaccatgcatatgagaagaactagatgtacagtat
ataaggtaggcttcttatactaaactaaccttactcaaccttcagatctagtttgcacatgggatggggt
tcatgtccctgacattggttcatgtgaacgcaactcttgcgagaattgttcacccaatggggctccagtg
ataggtggcctatgacaacacaattcttattgaggcatatatgggatatatcatctgtacacctgatcaa
tgcagggttgtacatcataaatgccagtctatgaaccaatgacacagacttttatcttcatttggccac
ctaacactcacctgcttgtatccatcaggcctcaatgagcccccatcccataaccaacacacaccattgg
cactaggccttatatttcttctaaggcgttcttgttggtggttacaccagtcatctactactccagaaaa
gaatgaggtgtaaagtgattagctatatcaggcataaaatgtaaaagaacaagatatattttgaaaaaaa
gttttttttaatttttcgaaatgggcgataata
>KolobokP-5N1_OwFu-I
taatagtgccatggttcgatgatttttggtgaaaaatgatatttttatgaaagaatgaagagtttcaaga
ttgtttgtgcagtttaagaatgatatatagacaattgggcattttatgggtacttaactaaccttggggt
ggttcgaatttaggccaaactttttttcttgagaattcaacatttctgcatttaaatggttacattaactc
ggatctataaaattgagcttccaaagagtcgagcaaacttatttatattgatcaaagtgttttattggca
aaaatcactggaccatgacacattaatttggccaatttttgggcaaaattagccaaaagctgactaaatt
agtcagttttcactggttttctattgtttaacttcttcttaaatcaaaattatagattaattttggctaa
ttagataccattcgtgaaattattcccaattgtagccttaaaaaatcaaattcagccatttatttcgttc
aaatatgttgacatctctcggaaaaattaggctgagaaattctgacatctaaaaatgttacaatacatcc
tttaccaagcaaaatatggcaaaaaatattcaatattttatatgacagttcgcaggcctacaaacaatga
catttcaggcaactttccgatggttaatgccaaacagtgcctaataatcgtcaaaacccgtaattttgaa
gtcaaaaaaagaaaaatgctgattcagcattatttttttagtttctcccgatcagatccttatgggccatc
gtccaataaacatttcacgtcacatatctcttattctgagaggttttgagccaaataatagcagttttag
taatttcttatgttgctaata
>KolobokP-1_ParEch-I
aaatagatccgccgaatactgcaaatctttacaaaagtgacacagtgctgaagcagcacatacatcccga
ttaatggtctagggaaattgcgagtaagacaccgaatattaattgagatgtcttcgaaccaggcacccgt
ctgctgaggaacaatctctactgtgccatgaataacgtatatcaacaggaactcggacccatgcattagt
tgctgtgctcaaaatatatgaattctgatgtcagtgcctcactgtgtacactaagccgaccttgaaaacc
tcccctttgacaaatttagtgcaatttaaatattaaaaaaaaatattatccaatatgctgtctttttatc
acaaaatcttgaacatttgcctttttaagatgacatctgttcataatgggacttctaaacttcacgaat
tatagacagtgtgaagagaacatgtgtgttgttgcatttttggggtaaacaataaatgtcacatcaaaca
ctctgacagcatcccggtttcctattacgcacaggtacatgcgaattgcagccatcagagtgctggcctg
tctcaatattgaccttctacaagctctacccagcctgacagaaatcccaaatctggtgtacttcaaacag
tgagggttgtaaatcacaattcaaacatacatgggtgtactgttgcagccaatggctagcctggacaaaa
ccttgtgccctcctctcttaatttggggtggtcatattagggttagggttagcctgtcattacagtagag
atctctgctgagccaggttcaaagatttcttgcatctataccaactgtgatcatgtgtgatgtgttgga
taaacttcagccaaacgctatacccaagcatgacaggaaatgtggtttcaggaagggacatgctgtgtac
aaaacaaggacggaaagcagtgccagtgcattgggtgcagtgcaggaaagtggagaaaatttagctgtat
ggatgccaagactcactgaagccgatttcgagcaagtcacaaaagtgacaccaggtggcctcatcgagat
tcccgatgccgaaggtcgatctggcgaggcaaagttgctgaggccaaaacactgtgcacccaacgcgaat
gatgacgatgatgatttgtcattaacgtatctgaaagcagacgatacacatgaggaaatgcgcctttacg
ataagggcaagatgcaatacatgtacaatgaatgcatattacagcattcaacacagaaaaacatgtgtgc
tattcctgaatttaccattgccagagaggtcaaggttggattgtgttggcagtgctgtcttcgctgcaca
aaatgtggattcatgtctggcatttataagctgtataatgaaattgagacgggaaaacgaggtcgtaggc
cagcagcacctaatgttggcttacaggtaggacttcaagaatctacaacaggcaacgtgaaaggccgact
aatccttacttgcctgaatttacccgtgccaaatcgcagtgtgatgcagagaacagccaacaaagttgcc
actgcaacagcgactatgacgttagctgatctggcgaagaaacgtgaaaatgtgaaacgagtgaacaggc
tgcgtggccttcctgagaattcacctataaatatagcaatggacagtcgttacaactcagcgaccatcac
tggtgcatatcatgcagggcaaaatgcaagccaggcaattgctgttgctgtagagaaacagtcaggtcgc
agtgacattgtcggcataagcattcagaacaaactgtgcagtcttggtgcttcgctgcgaagacgagggc
aagacgtaacctgtccaggtcacgcaaactgcactgcgaccctaccagctgatgaaccattgtcagaata
tacagcaggagtagacattggacgacaatttgcatcacagaatgttggattacgatacgtggtcacagac
ggagatgctcgcagtgcagagggtgtgaaggctggcatgtcagaaacagcatgtgaagtcgaacgtcaag
ctgacacaacacacctggaacaatcattgttccgaaactcaatgaaagcacaatttagcgcgcgcatgtt
tcccgggtcgacagcagtgattagaaaggaacaaaaaaaaaatgttttcactggatgttaagaatcgatgc
cacagaattcacagtgaaatgcagaacatgcatgctggtgatagcaggaaagtggcctcacgcatgccac
gtgttatcgagacgacattagattgctatggtggagattgtggaaagtgcaggttcaactctgtcgtgtg
tgctggtgggaaaaaaaaaaaattggtggcacagatctatgtacctacaaacaggacgaattcaccacctg
aacatgactgatgcagacagggtcactctccgaaagctgattgaaatgagattaggcattcaagcactgg
caataacaaaactatgcctgaacactaatcgaaatgaggctctgaaccgctccttatcttcgactctccc
taagaatgtgaacttctcacgcaatgtcacaggccgtgcgtgtgcagccatagaccggctgaactacgga
gcagggacatctatcttgagaaaactggagttgaacaatgctcctataagcaaaggcggcagtgttgcaa
gagctacaagacagatacagcgtgaggcgttgtatcacaggacttacatgtaccgaagatcagtggtgag
gcgtctactgtataacaagagaaaacggtttgatgccatccgtgcagcaagacggcgaagagaggctaca
gctcagacaagggcagatgctcggaaacgaggctaccgtaagggacagctagaccaacaagtgaaaaaaa
aaatcaaaacaaaaaaaaagtgctgtcaagccaaacaaaacaatcagtgatcacccatattccctccgtga
cagatctatagatgaccatacatacccgtacgacatttgacatgtgtgtgcgctgactgcacaccatgca
ctgactaggcagttgagttgatcgatcaaataattacttgaaacatcaaccgcaatacatactccttgga
ttataccagagaggaatcaacaaaagaatgtcacacctgcgaacacattgaaagacacacagacagactg
agtggatgagagcactatacaagcagctcagacagacagcttatctaatggggtgagtactgacatgcta

attatagttaccttcatctcattttattacacaatttaaaaaaatatacaaggcggggattgaccgtggc
caaagcatctcttttctgtagcacaacgcctcctgttattgttcacaatgcatgtctctagggtcagatg
ttgtacattgacacatagagtactgtgacacagatggctgcagtgcaaatgttgaggaatttctgatcta
atatgcaacatgtatgctagacgatggacacgcatcacccgccatatatagctatcaccattcttcaatt
tcactgaaatcaccccataatttccagtaaccctacccccctcccgctttcttgatacagccagtccataa
tatgcacgtcccattggcagccggcactgtcctacaactaattctgtgctcatactctgttatgaagctg
tcatccatcttgtctgatgatatgttgtcaaggagacagaaaagtctaaaatctaatta
>KolobokP-2_ParEch-I
aaataggtatggcaatttttgagaaaaaatttgaaattactatattttagaggcactcatatctcaaaca
ctgtgcaatattttttttttttaattcatccacatgtttttttctatacacatttcaaaagcgccaccct
acttgaccgggcaccatatgagtaagcattttactgttggacatattctaatctggcataactctacact
gtacacactttctcatccggcatatatgaagacctaaccctgttttccagaaagtccagtatgttttctg
taaacatatcacatacaggcagttgttatgttgcatttatatttatattgatttttttacttttttccta
ttagacaactgtaattatacatgatgtatgaccttaaagtgtggattcttcaaaggccagacgtcttaat
gagttcaattgcacagtattacacaacccaattagtagacattcaatacactgtggcacttgcctgattg
agtactgatcaagtcaggtgactgtatgtcctggcagcctgggcctaggagctgattcccagtagtcact
ctgccaagatgtcattagggttgctcccaacctcttcacatgtgtttaattagctgaagtaaacaaaaaa
agattgtttaatcacccttctgcaatgtcattggtccatttatgatatagatttccaaatggaggcttca
ctaatgtgattctctccccacccccccttatcacacccaagttctgtcactgtgatttggactttcagact
cattagaagtctgtgatttcaaataacggtttgtccctttcaactgtgattctgccgggattattctgca
ttattacaatgaaggagactgcaaattatgagactggtaaacttagcaaacgtagtgataggaagacacc
gcctgttagaggaatgaggagaaatcggcgcggtttccaacaaggccacccacattttgaagcaatgcat
ggtgaatcaacagaaacaccacacagtcatgtatggatgccacgactcggcatggctgacttttcaagag
tcgtacaagaaacttctggtggactgttaacggtacccgatgctgacggcaggcctggcaatagcaaaat
tctacgaccgcggccataccgatgcccagatttaacagacagctacctccagccagacgaaaatcaagga
ggatccgagatgcgtctgctaaatatggaaaagagtgctcagatgtggaatgaatgttacactgagcacg
cctgcaagacgacgtgcacttctccacagtttcaaatataccatgaacaacagattggtctctgttggaa
gcaatcactccgatgtacaaattgtgactatcattcgcgcatgtacaaactatacactgaggtcagcact
ggacgtagtggtcagcgagctgctacaactaatgtcgcacttcacgtcggtctccaggattcgacaatag
ggaccacaaagatccgtcacattctagctgcaatggacacaccaccacctagtcgcacaggattacagag
aactgcaaacaaggtcgcaactgtcactgcacaagcaaccatggatgacttacacaggagaagacagaaa
acaaaagagaccaacactctgcgtggtctgccagaagatgctccgattaacataagcgtcgatgttcggt
acaactccacaaatctcaaaaacacttacagctgtggtgggcagaatgcgtcacaggctttgggaactgc
aatcagttggcaaactggtgaaaaggaaatcatagctttctggatggacagcaagctgtgtaaactagga
gcttcacttcggaacaagggtgtcaatgtcacctgtcctggccatgctggttgcacggccaatgtgcaag
caacagatccgctgtccgagtacagaattgggaaaaaaattggcgacagtttagcacaagatgacgttgc
aatcaagtatgttgctacagacggtgatgcattagcatcgcgaggtgtacaagatgcaatgccagcaggt
atggagacacagaggcaatctgatacgacgcatctgtctcaaacgcaatttcgtcatatcatgaaagcat
cattctcccccaggatgtttccaggagaaactgctgcaaggcgagcagaaaacagaaaaatgtttgcgga
agatgtgaaaacaagatgccagaagatctacacaagtgtccacatacttcacgacagtgacacgacagtt
ataactagacgtatgcccgacatcattcaagccacactcgactgctattctggctcgtgcagaaactgtc
gcagacatgcaattgtctgccgtggtggtcgcaagaattggtggaatacctcacagcatttgaaggcctg
cggcctcacgcgactaaatatgacagacagtgatcgagcaacacttcaaggactgattgaaatgcgtctt
ggcaatgccgcacttcaaatgacacgacgcagactaacaacaaatccaaatgaatctgcaaacagagctt
attccgcgtcggtacctaagaacgttaaattcagtcgaaatgcactgggacgtgtgtgttcagtcatcga
tcgtctaaactacggtgctggagattcaatgctacgaaaattggaaaatgtgcagtgtcccattacaaag
ggcggaagagttgctcacgctgttaaacggatccagcaagatgtagcttaccaacgacaatatgccagac
ggccatcggtgcgcctacacagacgagccaccaagtcaaagaggatgagagactacattagggcgaaacg
acagagatgccaagacgacatctacagaaagagtcaacttgatcccaaaccagcaagtcatacagtaaca
acagtagcagtgacagcaacggcagttacaccagtgagaaggaggcgccgacagccaccgggaaatcgta
ctgatcacccatattcgttaaggtcacgaaaatcggccgatcactcttacgcaagcagagagtagacaga
cacatagacacacatacacacgagcacaatgtgcatacacaacattataacaatacgacattctgtattt
ctgcagcgtctacttgggctgcaaactgaccatcatccatgcaaacacaatgtgaagacctagaccatcc
gacacacacgacaccatctgcctgccgacattcatgcgaacacaatccgactacgagcaccacgaacatc
ttgagtaccatgatcaatcgacacgatgcaatgctacatcagtgacagcaggggtgactgatgtcgacat
atatcagtgacagcaggggctgactgatgttgacatacatcagtgacagcaggggctgactgatgtcgac
atatatcagtgacagcaggggctgactgatgttgacatacatcagtgacagcaggggctgactgatgtcg
acatatatcagtgaccagttgtaacgatcacacatcggaaaagttcatcgtacacgaactcccgaaaaaa
tgtaagtgctcaccatttttttaatggatttaattttaaatctaacatgcatgcagggtaaatgccatgac
cacgacattcgcctgatttcacgcaagaaattctttcattgttgacaccgtgtggttccaggctgacatg
tgtggctgtcacacagagactattgtggcagagatgtgaagcatctagattcgctcttataaatgttctc
ctgtggtgcagcatatagcttaaccggtgtgccgtgatggtacgtctattgccacatctaaacttgcagc
taactgctccgtatttcaccccctgtgccttttgtgcagcctgtccacaatatacactgattgtttgttga
aatagacacattttcattaattttgtctgcatatttttcataaaacctgtcgtccatgatgcctcactgt
gtagcgtgtttccgttgctagggactttaatt
>KolobokP-3_ParEch-I
aagtaggcaaggcaattttggagaaaaatgttgaaactaacacattttatagcctctcctatctcataca
ctgtgtgatattttttttgtaattcatccacgtgtttgtttttttatacacatttcaaaagagccaccctac
ttgacagggcaccgtgtgaataagcatttcactgtccgacaatttctaatctggcatatctctacactgc
acactcattctcatgtggtacgtatsaactcctaacacagttttccagaaagtccaatatgtgctttata
agaatatccaattcagccagttgttatgataaattccaatttatcttgatttttttggctttttgccctat
ttacaaaggtaaaaatatcaacaaaaatcaccttagggtgtgaaatcatcaaaggccagacgtctcaagc
agttcaattgcataatattacacagcctaattagcagacatccagaagacaatagcacttgcctgattgg
ccagaaggcagagccagtagacagtatcacatagcagtcctcattatggtggctcacaccagtggctgtg
ctcaagtgtcattagggtcactgataaggtcttcacatgtgtttaattacctgaaaagtaaacaaaaaag
actgttgaattaccatgctgcaacatgattggtttattttcataaaaatttccaaactgaggcttaacta
atgggattatatccccacccccccttaattatcacacacacacacacacagatcttcattcactgtgattt

ggactgacagactagtgagaagtctgtaatttccaataacggtttgtccctttcaactgtaattttgcct
ggattattccgctgcgatattacaatgaaggagactacaaatgagattagtaaagttagtgataagaagc
caccgcctgttcgtggcaagacgataaatccatgtggttttcagcatggtcatcgatacttcaaaccaac
acgtgctgaaacatcagaaactcaagcaggtcgtgtgtggatgtcacgactacatatggacgactattcc
agagtggtacaggaaactgcgggtggactgttaacagtaccggatgccgatggagtatgtggcaatgcca
aaattctacgaccacggccagataagtgcctggatttaacagacagctatcttcaagcagacaaaaacca
taaagattctgagatgcgtctgctgaatatgaagaaaactgctgatatgtggaatgattgctacgcagag
catgcaaagacaacatgcacttctgcacattttgaagtacacgaggaacgacagatcggtctgtgctgga
agcaatcactccggtgtacacattgtgattatcattcacgcatgtacaaactatacactgaggtgaacac
tggacgctgtggtcagcgggccgcaacgactaatgtggcacttcacgtcggtctacaggattcaacgaca
ggaaccacaaagatccgtcacattctagctgcgacgaatacgcccccaccaagtcagtcagggttacaaa
gaaatgcaaacaaagttgcaactctcactgcccaggcaacaatggatgacttacatgagagaagacgaaa
aactaaagcaactaacatcctgcgtggtctgccagaagacgaccccattaacataagcgtcgatgttaga
tacaactcgacaaatctgaaaaacagttatagttgtggtggacaaaatgcgtcacaggcagtgggaactg
cagtcagctggcaaactggcaatcgggaaatcatagcgttccacctggacaacaagctctgcaaattagg
atcttcacttcgcagtcaaggcatggatgtaacctgtcctgggcatgccggttgcacagccaatgtgcaa
gcaacagaaccactgtctgagtacagaattgggaaaaagattggtgacagtcttacacaagacagtgttg
caatcaaatacgttgctaccgacggagatgcattagcatctcggggtatacaagatgccatgccagcagg
tacacagacagagagacaatccgatacgacacatctgtctcaaacacaatttcgtcacatcatgaaagcg
tcattctcctccagaatgttcccaggagagactgctgccgtgcgaacagaaaacagaagaatgtttgcgg
aagacgtgaagaccaggtgccagaagatttacacaagtgtacatatgcttcacgacagcgacgcgacagt
catagctagtcgtatgccggacatcattcaagccacgttagactgctattctggcacgtgcaaaaactgt
cgcagacatggaattgtctgccgtggtggtcgcaagaattggtggaatacctcacagcatttgaaggcat
gtggcctaaggcgagtaaatatgactgacagtgatcgagcaacacttcaaggactgatcgagatgcgtct
tggcaatcgcgcacttcaaatgacaagacgcaaattgacaactaattttaacgaaggagccaacagagca
atatctgcttctctccccaaaaattgtaaatttagccgaaatgcattgggacgtatgtcttcagtcatcg
atcgcttaaactacggtgctggaatttcaatgctacgaaagctggaaaacgttcagtgtccaattacaaa
gggtggcagagtggcacgtgctgtcaaacggattcagcaggacgtgctctaccagcgagagtattccaga
cagccatcggttcgcctacacagacgaagaaccaagtcaaagagaatgagagagtatctcgaggcaaaaa
aaaacagacgccaagacgacatatacaaaaagagtcagcttgatcccgaaccagagagtcgtaccagcac
gctgtcgagagatgtaacagcaacgacagtcaaaccaaatagaagaagacgcagacagcgaaagccacct
gtaaatcgcactgatcacccatattcattaaggtcacgccaatcggccgatcacccttacgcaagcagag
agtaggcgtacacggcattctaacaattgtgacattctgtatttctgcagcgtctacttgagctgcaaag
ctgactgtccgtgcgaacgcaatgagagaagacctacaccatccggtctgacatacacgcgacacaactc
cacctgcctgcctgcgaacacagccagaacacgagcaccacaaacaatcgacaatattacatcatatcag
ttagcatgggctgactgatatggacacatgactgcaaacgcaccccagtgaccagtgattacgacgataa
ttcggaaaagttcattctacatttactcccgaaaaaagtaagttcttacctatttttaaatctatcatgc
atgccgggtaagcgccatgaccagaacatttgtcaaaattactgcaacgtatcctctcattgttcacacg
gtgtggctcaaggctcacatgatcggctgttacacacaggctattgtgacagagatgggaagcatccagt
actgcttgtaggaatgtcgtacggtgctgcagcatgtaggctaaacggtgggctgttattgtgcgtctat
tgccacatgcaaatttacaattaattgcaccatactttacgtttgaccctttctgcatttcatacagcc
tgtccacaatatacacttattgtttgatgacacacacgcattttcattaattttgtgtgaatatttctca
taaaacgtgtcatccatgatgcctcactgtgtagctgtgtttccgttgccagggactttatt
>KolobokP-N1_ParEch-I
aaataggcatggtgattttggagaaaaatgttgaaaacaacacactttataggcaccaatgttacaagca
ctctgcaattttaattttgtaatccctccaggtgtttgtgttttatgcacatttcaaaggtgccaccaa
acttggcaaggcaccagaaaaatcagcacttcgctgttggacaatttgtaatccagcacaacgttctcag
gtggcactatgaactcctgatacagttttccagaaagtccaataagtgttttataagaaaattcaataca
ggcagttgttacgttacattcaaatgtatattgatttttttaccttgtgccctatttacaaaggtaaaca
tcaacaaaaagtcaccttagggtgtgaawtcctcaaaggccagacgtctcaagcagttcaattacataat
attacacagcttaattagcagacattcagaaaacaatgccacttgcttaattggccagaaggtccagtca
gtagacagatctgataacaggctccggctatggcctactccagctaatcactgtcctcagacttcattag
ggtcaatgacaacatattcacttgtgtttaattagctgaagtaaacaaaaaacgattgatgaatcaccct
gctgcaatgttattggttcattttcattcagattttcaaattgaggcttaactactggcatacgcacccc
accccccttataagacatgtgttccgtcactgtgattttgacttgcagactgatgagaagtctgtaatat
ccaaaaacggtttgtcccattcaactgtagttttgccatacacgtattttcagtaattgtttcaagatat
ttgaagtaaaacgcatcctccgacattgtgcaatgactatagcgtgctttcgttgccaggctctctactt
>KolobokP-N2_ParEch-I
aaatagatctcctgaatcttgcaaatcttcacgaaagttagatttatatcaagctatacatataactaga
ttaaccacaaagggaaatttacactgtctcagtaacaattaaaatatgcgtcatcaaactgtgcacacct
ccaagctaaacagcgccttgagttggtttaaacatgcttattcggtactttatattacatacgacgtttg
cctggtatgaatggtactgagcacagcatacaggccaacacagtatcttaccgatatgggaatcaaaata
tatagaatattaacgaaatatatttttttgtaatttttcataaaatgttgtatccttttgcaaaaaatac
acaaaatcattattttaagcagcattttattggacatgcactttgcaaatcttcacgaatttttgtatta
tgtaatgcccacaaactatgctaaataatctgatgcaatcatgaactatatgtcactcgtgtacgacacc
agtaggtgtcaaaagcacaagccaggcatttcaacaatattcacaatattgaamggcacagaggcgtctt
ttgatggtaactacaattatattgtagataacgggctaaaatgctgggtgtaaattgaggtatatacata
gtacattatgaaaccaaaactgagatgcccagtggaacgtgtatcttgcgtcacacaaattgttgtccta
accccccttaaaataaccatacttttacacaggcgcagatctctgcttgatacagagtgtccaaacatcg
ccacacgtcaattacttggccactatatatttcgctgatgtacacacattattggaaacaagagatacta
ctggcaaaatagttaatttcagaaagaaacaaacgtttgacaaggacatccaagacacgtgataaaggtt
gttgacagttgtggcacccagtatctctccaagtacatcaacgagtccattgtcctaatcatattttatt
caaacatcgccaaattcttacaagggtactaacaaacatttctgatgttaaatatacggatataacgtac
aaaataccagtgggwaaaaacagcttcttgtcagagtagttgacgtcagcctacaattagtttaagctgc
cttgccttgtgcaaacttcggtatgattcggttatattacagctgaaaagcacatgatcgttgaaaatga
tagcatgccagtccatgtacacaatttcaacatatgacccttctcttaggtgaccagttttaatttktat
ggctaaaaatgaactaatttggatcattgcggtcgctgcacatttggagagtcacacactgttatgatat

tctctgttacattcgtggtatatttatgtagattctcaattgttcttcttgtctcagttagtgtatggat
gttgctataaaaaataggtgctagatgcctgattagtagaagtgtgtggacaacttgcttcctatggtgg
ccaacatttcactaaaattagggccgcgttagggggggggtggaatcaaaatgttgcctgcttgcccatt
attgcttattgtgcgatccaagtatgatatatatmagtgccatataattaggaaggacaagttggaacag
cagaaacacccacgttagtattggggttatgaaacacgcatttatagatgtcgcttacaaaaatataaat
gatataactaaggcgaaattcatcctactggaaatatgtcaatattgttgcacatgatgtagatttaaaa
gaaactccacaccctgacacatatacacttttttacacaagctttcgtggtactcagtatcatagaacatg
cttggaatgtactttttaactttcacgccaccaaaattatcactttattttccatgcatatcacataata
actggcttattaattacttttaaaacttcttttagatgtaatactaacagtatgcaatagctgcacgata
ttctggtctacaccagcttccaaatacagccgaaaaaccatcacccgtcgctcaaatcacattttggcag
tttttgtgataaaacgggcgtatttctaattt
>KolobokP-1_LiLo-I
taataggaaggggttaatagcggatgggttaaaagtgggcatattctgaaagggttagacctcccatcca
acaggcatagggaagaaatttgatcaaggccctgcatggcctatgagatcaaattgtctattgtacttac
cattgcaacttcaaacggagttcacagggcggtcccttatattcaacaggtgctgtcttcaacaaacaca
gggttaaagcaatcaatcactatcagtcaatgactctgagaggccctcttctgaaatacaagaatgaaat
cattacacatgcagtttgtcacattaattcattgcataacatggccacacccatatatgggcggtacccg
cctaatattacgtaatcgtacgcaatagccggggacccttttttctgcaaattttagtatttttgatttca
aggatcctacccttcaagatgcatcaaaaagtttagtaggccatctcaagatgcaaaacatatcaagcgt
caaagatgttgtcaccccacaaggagttcatccagcaacaggcagaaggttaagaggacaatagaaatca
ttgtttattgatgcttcatgacagtttgatgacagagaaatgaattaattatgttagatattagttacaa
acaggtgtccaggtgtccagattaaattttttttaaatccccccatctttctcacaggttattggccgaagt
taagaagaatcagtatgtgttggggctaccaagccagcaggatggtgctcactaggcggcgtcgctatga
ctgtcaaaaaccagggattactaaacgaagggatacattttttcaaaaagggacacgttccgtacaagtcc
aaagtggattttcgagcctcggaccctggggagtggttacgtcttccactggatctttacaatcaagtga
ctacaaagggaccgggtggtacaaggattgttccggacattcacaacaggccttcaggtaggaccagact
tttgagacccattaagccgccatcagatcaagatgtacaaaatcatttcgaaccctcatcatataaccct
tcatgtagcatgaccgatgagaactatgtaatctctgtagaaaaaaatgatggacttaatgaactctgtag
ctgtgtcacacaatatgacaggttgtgttcgtccggagtttcaatatgaagataaagtaaaaaaagggtct
ttgcgtgaccgtgagggtcaaatgcaaaaattgcaagtacatttctgacaaaaataagttgtattcagag
ttggcatcggctaaggcagggggtaaaagtgccatacccaacatttccttacagtgcgcactgttggatt
catcaataggaaacttcaaggggagccagatcctgaacacaataggtttgccgacgccctgtctgagtag
catgcaggaacaagcaaaccgggtagggaaaaagattgttgaactcaatgtggaagacatggccaatatc
agaggttccttgagagaggttaatcgactgaggggtcaggaagacgtgaatgcattcacagcttcaatgg
acggtcgttacaacagccaacgaatcaccacaaggggcaaacttggccaggctgcatcccaggttgtaga
aatagccacagaaaatttaaccgattcgaagaaagttattgatgttgcatttttcaataagctatgctgg
aggggtgcatactgccgtgcacgtgggttacctattcattgtgggagcgctcccccaaagtcaaattcta
agtcaagtaaggcgagtaggcctaccacaaggagttctgctagggagtcaaatgagggagccgaacagtt
tcataagaattgcactgcaaattttccagaggtactcccttttttctgaatatgagttggccagaactatc
actgcaaaccttgccaaggagggcattcatcttgaatatgtctgcacagatggggacagttctgcttatc
gtggtgtcagggatgcaacagatatgattttttgggaccctgttcaatgtaaagcctcaaacagactctat
tcatttaggccaggcaatgtttcgtgctggtttaaagactgatttcagtaagaattgttttcctgggaga
acagcagatgatcgaaaaacatttcaaaaactgtttatgttagatgtaaagtcacgctccagcagggcat
tgaaactactcaggaatgatgtgggtggggacgtggtgaaaatgtcaaagaaactggtacccttggtgca
atgtttgatcgattgctactcaggtgattgctccaaatgtagaaataacaaatacaacctgtgcagcggt
ggaatcaccaattcttggaggaagaaatcagaatactttaaggcctatggtaaaaagtcccttgacattt
cattcaatgaaactgacagaaagctgctccaaatcctgttagaagttcgccttggggaatctgcattgga
gtctttgaaaacaggggttacaactaacaaacctgaagccacaaaccgtggcatttcaagcagcctaccc
aagaatattactttctccagaaatgcattgccaagggtacacagtgcaatccatcgcgtgaacaattcat
tggcaaagagtacaatgaccaagcttgataagggtctcaatgtaccgctgaaccccccagtctcatgctat
gaagaacttaggacggctccagaataggaaggcatacaatgtggcttacaaagagtctccggagttcaag
aagcaaaacaaacggcaaagaattaaacgcatgggagacttcctcacagataaaagggccagaaaggagg
gggactaccaaaagttcagccagctactgattccaatacccggagtagcagcagggtcatctaaatcacg
gggtcgcggtatgcctacaccaaccaacatcaccaaaccaaaaggcaagaaagacaaaaaaaatccacaag
gagcacccatacagtcaggcgccatctaacgagtcacggtccacagtcggagggtatgtcggagggggagg
accgtcgccgcagggaccacacatactccagtgatccagtccaatcaacgtcgtcacagggaccaactgt
caggaaaaagggaccaaagaaatatcaaacaaaaaaagtaagttggtttaaaatgtatgcatcagccagga
aggggttaattcgttaaaaattagcatggggtgggattagcttaataagcctgggtcttttgaacatttt
gtaacacaacagtagcatgtgcctaccttgaccagccccccatatgcaaattaagtatacatgcagggtat
gatgtgtgcccaatacacagtcctctggccaggcaacgctgccgactcttgtttatggcactgggctcca
gggtcaaatgttccaaattcacacacagcgaattgtgacaaagatgtgagacatcaccctgggggtcagt
gaccccagtattcaaaatgtacatcaacctgtgtacacgaaacttcttagacttctttgcatttggcagt
ttggcactcatttctccatatatcatacaccccagcttccgtttggccccccaagtccatatccggcatt
cacgattcttaccaacctggctacgggcctttattctttccttgtacgtgtgtttccaagcttcatcggc
cattttagctactgttgtgtcactgtgggagacaaaattcatgggtgtacggtccaactgaatgtaccta
ata
>KolobokP-2_LiLo-I
aatgaagaggggataatcttgagatatcaatgaaatatgccttttcccaagcatttgagatgtcttgacta
acataaccaagtttcatcaaaaaatgaacttgatttgagcgagctattaacgttttaaaattttgtccgat
acggacaacctaccttatatttcgtcttcaaattgtttacagactgtcttcaagttaagctcattaactc
gtagaattcttaatcgaatggtaccgaatcttcgacaaatcaccagtggtctacagtcaatctgaaatag
gaatttgaaacaattttaattcatgagaaatattcaaaaatttcaatttttttggaaaaaagttgtctttt
taaaggatgttccaaggttgtgacagtggtatcatgctgaaaatgtcaaaatttcaaatcgaacaatatt
aactttgtctgaaatttgaaaaacaacaaatatatgatgcattataagcatggaaagcttgttagtaagg
tgtgaaatgagaaaaagattaacccatatgttatctccctgtctgtcacatccaagtctgacacttccac
aacagcaaccattgtcatcgctattgtcctatcaacatcgaccctttttgtaatctcttgtcctttcttgt
gtattttcagtctgttgaaggaagcatcccgaaatagacatctcctaggagtacgattttaagcgaagaa

agacaagtatacgaaaagacaagacaagatgaggacaaaggcgaggagattgataggaaaacggggttct
ccgaggcgtgctagggcatcgaatgcagcaatgagaacaatgttcaagagcggacacgtgcggagtggag
gtgtcgaggtggtgagcgacgacgaacgagaagtaggcctacaacccgcatggagacgactcacgcccca
ggagtacggacaggtcacaacgatgggacacgacggcaaaagacacataccggatattaataaccgtact
gccaagaatgtcaacttactacggctgaacatgaaagaagacactgatgatgagatggagataccagatg
agaccatggagcatgatcacgagagtttcgttgttcagacaaccaaactgatggatgtgctcaacgacgt
taacagggcccatagcgaatatcacgtgaactgtcagccgatattcaaatatggagaggtacgtaagcag
ggggtgtgttgttcagtcactgtggtgtgtaaaaactgtggtttttattcgaaattgtataaacttttg
aggacatagacactggcaagcgcggacgaaaagccgctcttccaaatgttgcattacagattgcgttgca
ggacacaatgattggcaacgagagtatgagttacatactgagcatgatgggaatcccgccccctgtagg
agtatcatgcaaaagcaggcaaataaggttggtgatgaaacagttaaaatgaatgtggccgatatggcag
acatacgcgagaacttgaaaaggatactgcgcctaagaaatcaggacgacacaggaatcaatgtccaagc
tgatggacgctataatagtttacacctcacttcgaggaacaagatgggccgttgtgcgagcacaatgact
ggattatgtgttgaaaatgtgacagatgaacacaaaatcattgacattgaggttaaaacaaaactctgct
ggataggggctgcaatgcgggcgaaaggttttgaagttaaatgtggtgccactggcacaaacgttgacga
acgacactcgaagtgcacggctaactttcctgaagcagatgcattcagagaatatgatcttggggttgca
tttgccgagacttttgagaaacagggcctacatgtagaacatttgactacagatggtgattccagttcat
tcaaagggttaactgatgcaacaaaaaagctcttcggcggcttgttcacggtgaagcatcaacttgactc
tatacatttgcatcaagcccagacgcgtgcaggacatagagctgaattcagtaaaggtatgttcccggga
cgtactgctgaggagagaacactgttaaaaacaacgtttctgaatgatcttagggtaaggtcatccaaga
ttttgcaatctctattcaagcgctgcaacggtgatcttgacaggatcatgggatacctgtatgacactgt
tgcctgtgttgtgaactgttatggtggtgactgcactggttgtccacagaacgaattctcactgtgctca
ggcacaactggttcttggtggcacaaatcaacacgatggtcatcttacggtgagattcccaagtttaaaa
tgacagtgattgacaagaacttggtacatctcgttttggaggtatgccttagtaaaggagcgttgcaaca
gctgagtcttggcgtgacaacccaggggggtcgagtccaagaatcgaggtctgtcggccagcgatcctaag
attgtgaccaggagcaggaactcaacccctcgtatccattcagcagcccacagaatgaaccataacatag
ccgagagcgggcgacggaaactaaagtggttcaacatcgaagtcgatacttgttgccgcccaatgaaagc
actggacagaattgcaatgaacaggcagtacacagtcaggtaccaacggagtgctagacaccagaagtta
gctggtgttttgaaaaacatcagggcaacagattacttcaccaaaaagaaaaacaaaaccaggggtggtg
actacaagaaacatgtgatgataacaatgccggacttgcgaaggaaggctgtcaagaaggctgttgcacg
acgagacagaccgcctgtaccagagtggaaatgtctcgacccaaacagcagaaactacgaccactcctac
tttcggcgatccttcaagatccaaccagccaccaagacatccaagaggttgccatcgcggtaagaaaact
ttcaaatgtcaattcaatataatgatattttctttcaacttcaacatatagcttaccaggaaaaatgcat
ttagggcggttgccatggcctgcacagactttcttggcattgcacctttttctactttgattgacccttc
ctctttctattgtcaagtgctcgactttcacgcacagaggattatgacaaagatgacttatatgataacc
tgcgccagttttgaatttcaattcatcatggtcacacaaaaacattgcccggtgtgccgtcattgtgacc
ggcttatccaaaaaacttactttaacaaacccgtatgttacccccctctttttttgtttagcccccctccc
aattcaaacaaaaaccattacttttagtctttgctaatattcttgctcgtaattcagatacttttctctt
gaattcacctgctgaaataaaaaaattagtgaaagcttggtaccctgccacccagcgtgctctgtgcccg
acacctcccaaaatgaaaataacatgcaaaacaatcaacactttcattactattcacaattgatgattgt
tatgaaacatatctagcccaaaaaagtagaaaaatttgaaattatttgaaatttttgaaaatttcaatt
tttggcattttcacttaaaagccctgtaggccaggggggggggttaaatttgagaattttctttttttcc
acattttccaatttcaatgatatgtccacacattctcaacacatttcagccaattcaaacggtttttgtg
gatttcagagctcatttactatttttgacctgtaacccaaaaccctctaaata
>KolobokP-3_LiLo-I
tagtagagagccagtgttggccccaggtcaaatgttctgctgttttcagattcccctagatgttcacatt
actgtgcaaaaggaattttttgaattatattgaatagtaaaatttttattcgagttcatcttcttgctatg
tccggcatcaaattctatgttgaagtgatgtgggcgtggcaaattcttaaatacccaacatgtaatattt
ctgttaaaaattattaaatccctctaagcactgttagtttgaaataaaggatatatacattattagaaat
aagagtttttgagttcatatggtcattgctggatgtcccagggccaggtttctaagtttgggcagttacc
atgacaacggaatatagaaattgtagaatctctacaacactgagtctgaattttcaaaaagtcacaattc
cagattacttcagattcacacttgcctacctatcaagtttaaacttcatatgaccagcaaagagcttgtg
tggccaattcaaagtgaacccctttgaccccgagaaacctcatttactcacattctcaacttgaaagatc
aaaggacacatgtgtatcacatgtttgtttttcattaggatatgacttctgttatggctaaggtatttgt
ttatcttagcagctcatcaattcagagaagcacaatgatgatgaaccatcccaatatcctcaagtgtcaa
ctaggtatgctctggggggtcactttacatgtggactccacagagtaaacttcaactcaagatgcctaagc
taagcaaaaagccaagccgtggatttcaagttggtcactcttaccactcgccctacagcaggacaatgcc
tccagagaaaaccaatggtagctggtcgcgtttcagtaatgatgattatgacaatgttgtgcgtgagcgt
tcggatggcataatctatgcaccgacggtcgagaacacagtcagcccactgaagatacttcgccggggac
aggatgctgcaaaggacaatacgtatgttataccaactgaagatactaatattgttgccagcaaatttaa
aattcttgaaatgctgaatgggtgttcagagctccatttcaactttattggtcattgtgacattccaaag
tttgagatagaacatgacatcaagaagggtgctagtgctcgctgggcactgaggtgtgcaaattgtaatt
ttgcttgtgggaagcattattttaaactttataatgaaatagagaagcaaggtcgtggtgcaaagcaggc
tgaaataaatgttgcattggcactggcttctcaggaaattggtagtcaatcaaagactaggacattactg
tcaatcgcaaatgttccaccaatgtcaaaatcaagtttacaaaacaatgtcaatagggttgggatggaaa
ctgttgaactcaacaaggatgacttgaagagacaacagaattatctaaaagaggtgaaaagaatgagatg
tgatcaatcaggtgtcagtgttcaaatggacacagtctataatagttttactattgccagacgaagtagg
cccggtcaagccgcgactcaagcggtgggattagttcgtgaaaatgagacaccgtcccacaaaatcattg
cagtaggtatacttgacaaactgtgttataagggggcatggcttttaggaagagggttccaaccaaagtg
tggcacagatgaagcacatgatggatgcaaggcgaatcttccagattatcaagttctcagggaatatgac
attggtaatatggttgggttagagttggcagctaatcacttacttgttcattgctgtacaaccgacggtg
acggtaaatgtagtgctggcatggaggatgctttcagcacctttcttgggaaggccttaaaagtagaaag
aatgtcggatacatatcatttgggccaagcccaatttcgtaaaggcatgagagcccaattttcaaaggga
atgtttgttggtatgaaggcacctgatgcagcagaggcgaagaaatgtctctccagtgatattgcaatcc
gtgcatccacaattttcgataccctgtttaaaaagtatcatggggacactgaccaaattggtgaaaactt
agccacaatagtggacaatctgatcgcttgtttcgacggtgactgcagccagtgttcaatcgaaacaacc
gtatgcgatggccaaggttcttggtggagtgcatgcaaactaagcaacttggagttgtcagccaaggacc

tcaacatgagtgatgctgacaagcttcttgtgagaagcatactggagatgaagttgagtaaaacatgtct
aaaaaagatacgtcatgcgacaaatacacaggcctgtgagagtgccaatcggcaattgaatttgattgcg
ccaaaaaacgtgactcaccttaaaaactatcgcagtcgtgttcatgcagcaatccacaccatgaacaata
cacggggaaagtccattccactgaagctcaagagacttggtgcccgagtttcagcagcagcaatgaattg
tctgaaagaaatggacaataggaagtggtactataagaaatgggaaagcgagctgaaaactaggattaga
cgacgagttgtaaggacaattcgaacgcgtgcattctatcaagcaaagagaagggggtagttaaagaca
ttgactaccataaggacaggctagaactagagggggggaggaggagggtggattaaaggagagagctaa
gagactagcaagagattacaaccaccactttcgacaatacacagcaatttcatccagctcagaaacaatt
taagatatcactagcttcactttatacccactattatactatttacctcaaaacaagcagtctttttat
ctaagtgcccaaagcatttcttttcagccaggcaagtcttcctctggttgttaataccccctagcttctaa
actcaagtggtcaacattgacacacagcgcattgtggcatctgtgagagacatcaagtccaaaaggcaag
gggccaccataatatgcaatataagcaagcctatgggccctgtgctttttgctgcacccgtcccaaggta
gtttaaaccgtgtctccccataacgacgactatacccagtccataatatacagtttacacaattcgactt
cagatcagcttcgctgttccggtttattttaactttaaggtctgaatccatgattgctgctgaaaaggg
ttaatatgtacaataatgccataaatattggttcaactacaagaaaaaggactggaaaggacgtgcagaa
tagagaaaataagataaaatgtgcgtgcaagtgtcaaatttttcatacttggtcaaccattttgactact
a
>KolobokP-3N1_LiLo-I
tagtagagagccagtgttggccccaggtcaaatgttctgctgttttcagattcccctagatgtccacatt
actgtgcaaaaggaatttttgaattatattgaattgtaaaatttttattcgagttcatcttcttgctatg
tccggcctcaaattctatgttgaagtgatgtgggcgtggcaaatacccaacatgtaatatttctgttaaa
aattattaaatcctactaagcactgttagtttgaaataaaggatatatacattattagaaataagagttt
ttgagttcatatggtcattgctggatgtcccagggccaggtttctaagtttgggcagttaccatgacaac
ggattatagaaattgtagaatctctacaacactgagtctgaattttcaaaaagtcacaattccagattac
ttcagattcacacttgcctacctatcaagtttaaacttcatatggccagtaaagagcatgtgtggccaat
tcaaagtgaacccctttgaccctgagaaacctcatttctcacattctcaacttgaaagatcggaggaca
catgtgtaggcctaccaaatgtttgtttttcattaggatatgacttttgttatggctaaggtatttgttt
atctaagcagctcatcagtacagagaagcacaatgatgaaccatcccaatatcctcaagcttcaactaag
tatgccctgggggtcactttacatgtggactccacagagtaaacttcaactcaagatgcctataactaag
caaaaagccaagccgtggatttcaagttggtcactcttaccactcgccctacagcaggacaatgcctcca
gagaaaaccgatggtagctggtctcgtttcacttcagtaatgaggattacaacaattaatgttgtgcgtg
agcgatcggatggcatcatctttgcaccgacggtcgagaacacagtcagcccactgaggatacttcgccg
gaggggacaggatgctacaaaggacaatacggatgttatactaactgaggatactaacattgttgactat
acccagtccataatatacagtttacactactcgacttcagatcaagttcgctgttctggtttattttgag
ttttaaggtctgaatccatgattgcggctgaaaagggttaatatgtacaataatgccataagtattggtt
caactacaagaaaaagggatgaaaaggacgtgcagaatagagaaaataagataaaatgtgtgtgcaagtg
tcaaatttttcatacttggtcaaccattttgactacta
>KolobokP-N1_LiLo-I
aatgaaggtacatttcaggggttaacattttgagattttttcaaactaatgtagatgacatcccacaac
tctccaacaaaggaaagtcatatctggacatttttggagaaatgagagacatttcaaagagtggtacaac
tgtgccaaagcgacgaaagaggacaaaatcgacaaaaagtcatgattttgcagccaacagcaccaaattc
aaagacctgccctggccagaataagcaagctatggagctgaaaatttaggatgtgatttaggaatatctt
tgctgcttagggcacagctttcagaatcaaggcctaagtagtttcaaagaaattacaaaatgaatggcaa
cacaacaagacttgtaaaaatgaaaccgaacttagaccggggttaggttgactcttatttgggtcaaatt
aagtttttttctcattattttagcttgttttgaccttaaatcatcagcaatacaatattctaaatgaatt
agagtgatttgagcacattcaaatttttcactatattgacccaaaatgtagtgtaaata
>KolobokP-1_Memb-I
taatagtgagcatggcgattgccaatgacataaatatgctgtcctagtgagctagagcatgtgtcctaca
agaaaccgggattttatgacgatcagggcattacagacatgtccagacatgttttagacacaggcactcc
atgagtatttgcttactttttcagccatctattttcagtagtttcggaaatatgaccatctgataattgc
tctctcggtaacaacgtgccttcattcggaaacctatttggaaaaaagtaatactgaggtcaacagcaga
ctctaccatgtatgtttcaatatatattttgcctttaccacatgaggagttgcctgctcttaatacccga
gcaagcaactcccatttgcgggtaaccgtgggttgcatgagccattagaataccaagaataccgcaaaat
atgtaagaatgcgtttcgactgcaaggtatcttgcctataatcatgccacgtttaaataaaaatcaaatc
gcaaataaacttaagggatttcggccaggggatgatagaatagcgacacgaatgtttagcccacaatgca
gccgcaggacggtgagactcgagagcagccaatataggcgtatcattgaggagaaaaatgggtgagcttg
acttgacgcatgaaactgatgttttattggctttgaccagattttattcattcttaacgggtgtagtcac
atacacgaggtaataaattatatatatatatatatacaaatatatgtgagtgtagactgacatctgctgg
ttttctgtttgcagacgtctggtggcgagaacagctcgaggagagaatgctggtatgagagttcttcggc
ctcgctcaacctgcaaagacaaaaaattagagcagtcagataacggcaatgaggctaagggtgtgggaaa
ctttgttgtcaacatggacatgatgctgcatgcgctgcaggacttccagaatggccacaagacattggaa
acttgccaggagtgggtcatcggtatgggcgtcacaagaaagaaaggcctaacatgcagcatatcattaa
catgcgagtcaaaagggtgcagcttcactaaagtttatccgctctacaaagcagcgcaggagagcaggag
ggggccgaagagggcagctatcaacaaacaattcgggcatgccgtgcaggatagctgccttggcatgcac
ggggcatgtcagctgtttgcatcacttgacatgtccccgctggctcaatccaactgccagaagctggcca
agcttgtggacaatgagatgacagacataaataacagagatctgagcagactggcaggggaaaaattaaa
tgataacaaactatgtggcctgagcgggggagggaggggagattgccatatcagtggacagccgctac
aattcgtcaaggtttggcaataggaagaagatgggcctctgtgccacacaagccatcacgactgccatcg
acagctgtacggggagcgtattagactacgtcatcgagaataaggtctgctctaaggggtgcaggctgcg
ccagcacaacaacgcagcagcctgtgcccatgaacgcacgcagcatgactgcagtgccaccctgcccaaa
gaggagaacattctggagggaccgatgaataccaaattgggcgtgagcttgctgacacgtgggctcatcc
cggtgacggtgacatcggacaacgatgggcagggtgcaaagccctatcaggctttgttgagtgcgaggcc
agaaaagtggactgttacggcccagtccgacccatctcacctgggcaactcacaggttagagctgtagag
aaggtgaacttcaggaaagagacattcagttgcgccagggtaaagacaattgactcacttaagaagttgc
tggcaaaagacgtcaagtaccgttcgagtatcattgcgcagcagtcgtccaagctgggtttatcgataat
ccaagtaaatcatctggtcagtacggccgtggagtgcttcggcggcaatcatcagatgtgttctagactg
gttggcagcagctgctgcgggacagaagaggacaactggatgctcagtagctcactgttccgtgctgccg

gtctggggcatataaactgcaacgaagaagagaaaaaaaaactgttagatgttttcaatattcgccttca
tgccaacgcccttcaagcgacaaagcttggctttagtacacagcgcaacgaggcatttaacagggcattg
agtaaaaatatgccaaagaatgtcctattcagtaaaaactaccgcggcagagccagcagtgcagtttacc
gccacaacaatggacatagctctgtggcaaagaagcttgccgatgtcggctgcccgttaagccgaaatgc
catcaagcggcttcagtcgcagcaaaaaaatatcaaacgtgcaagagcactacaaataagcggacagtat
aagaagatgagggcaatgagccgcatgagagcggcccaagattggatcaaaagaaagatagccgttgaaa
gtcgtgaccatgactatatctgcagcaaaacgacaagcagagtcgccgctcgcctttcgcgctcagttga
gcatgattattgcaagggccaaaacgacccttttcagcctcagtgaacatcttgtttttttctttacata
cacacaatgttctatcatacaatacaatgctacacaacgtcacacagtaacatatgaatatatgaaataa
gcagtaaacatgtctggacatgtctgggcaggaatgagtaatgataatacatgcaagcggggtcttcatt
acaaaacccatatgtgagagtgtgtgtgcaatgagggattatagccagataatttattatatgagaaagc
aaacaccaaatcagatgggggggggggggggcagcagatgagtgaatacagtacagtacctgatagtagt
gataattattaaatcaagcagttcgggtagccagcagcatggccactgcacttgctgtcatgagagcacc
tcctgcgctgcatatttacccagtttggctctaggctcaggtggtggaccgaaaggcacagcttgttgtg
acacaagtgcgaggcctcgtagccaactggaagctgtgtggtgcggagggaacacatcatccttaccta
tgcacacccagcaccctactctccccatcactccattcaaccctcatcataccataacccctggatgatg
tgccaccagtccacaccaaacaacccccagaggcagaagcctgcactcgtgccatcaacttgctgtgcca
caaagtataaggtggccaagaagacatttgtttgttttgtttgttttgtttatcattcatttgtatcagc
tgattgtctgctaatgccactatta
>KolobokP-1_PhoAus-I
ttaaataaaaccacattttccaaattttttttcagaatagttcctctccacctgctgacctattttgcaa
agtaaaaacttgaagcgatgaccggttttgatttttcaagccaattgcatgtgcccacccaaaaactggg
atttttgaaatagggccattttaccggcaaagacaccaaatgcactttcttcttggtctgttttgcacta
aactgacttttgattgagggaattggagaaaattgataaattagcctatgaaaataacaataaaagttta
agttaatttcctgaccccacaagtggaacatgttctcagcaaggcccattgtgtatagcctaaggggaca
tatttattagagtcgatttgcataaccacaaaagtcatgagaacaagaaatttggcagccaagtttcatt
cacaacataactgttgagtggtgagcacacacaatgtatcctgaaaagacacgcagtttcactaggagca
gagggagaagaaaaagccaatttaaaaaaggacacagaccctccaacatggaccactgcagctccagcat
accaaggcagtcctaggcagcaatagacggttgcagggctgtgcgactcttcgaagctgaggatatcacg
tacatgtacctaactgacagtgatgctgctgtgacagcttacaggtttaggccaagtactgaaaaagaaa
gaaaaattcgtgacagcaagggactgagaatatcatcattaacgtgtgttgcttaacaggcctggtagga
catgtgcatcaggttgcatgcaaatttggaaaactagaactaactgttgaaaaaagactgggactttgtg
tctacgcatcattgcattgcaaagtttgcaaatataaatcgcccactgtgccattgtccgatgaggtcaa
gctgcagacgactgggccaccagcaggagccttgagcagccaggctgtactgcctgtgctaaagtcaaaa
gtgggaatcgaggatgcaatcaacattctgacatgcttgaatattgtgccaccatccaatcgcagcctgc
agaataaaatgaatgccatgagccaaacagtaactcaactgaacgaagaacaaatgattgagaaccagcg
ctacgttaagaaagtcaccactctggctggcggggaaggtgttgcagacgttcagtttgacgtttcctac
tccagtaggccacagggtggctgcaagaaagctaaacagagcttcgggggacttgtggagcacacgaccc
gaaaactttcactggcccttgccacggcgaacaaacactgcaggattcccgagtgcaagcatgaagactg
cagcaaaaactacccagctgaaaaatcaattgcatcaaatgaacgtacactgttacaagccagcctcaga
tcagtggaaaacagtggtgtggacctgaatatcaggagcattacgactgatgccagtagtcaagaggcga
aggccatccgtgatttctacagtgcaaaaacaaaaaacccaaacactacaaatgcttcattcacaaactg
aggcgaatgggaaagagtgtaaacgaagcggacttgaaaagtttgcccaggacacataacaagaccggtt
ataagaggaaactttcatcagctgtgcgatctagggtacaaaaagagctcatcaacctaaaaaacagaaa
aatcggaaaagacgaatttttaaaaattggagctgctgcagtgaacaatgttactagctgtttttcagga
cagcaccagcaatgtcgcgagctatcatctgtgtgcattgcacatcatagcaactatatgaagccatttg
ccttatgaccggcatctagaactcagtgaaaaagacttgaaaacaattcaaacaaaaatcaactcctact
tcaaccaggaacagctagcagatatgaaatatctattcaacacgaacattcgcgaaagtttaaatgcagc
agtttatgcatatgcaccaaaaacttcgcgatacacacggaatttcacggggctgtgccattcagccata
ccttccagaaccctgggacaagggaaatcgacggtgaagctggccgaggcatcaggactatccgtgcctg
aaaacagtgccatgaaaaaaagttgagaaatgtggactgtagaaagctttatcatgccacacgaaagtcg
agccaacaatacaaaaaggccaggtactacctaagggagaggatggccaacacgaatatgtttgacaaat
ctgtgtacagtacggaaaacatggcatctactaatgctactgaccacagctatggacttagttactaact
gtattttagccaaagcaaaaaccaaacgacaactggaaaaatggaacttaaaagcttggaagaatgctct
ggccatggcagagagagggttgttgtacctgtaaaagcatgttttgtatttgtttttaattgcactgtag
cacattgttgctgatgaacattaaattgtcatttgaaatgcattaatgagtgtctaaagttaatcgcaag
caccacatctacacccagctgcagggctcctgtgccaccacctggtccaagtacacagtcacacacaagt
ctttcttaaatttttacaaaaaaatgaaaaaaatattcaacaaaaactattgttctcactttgatttgtg
acagagagaagaattagtgccaaaactttgaaatatttcaacttgaaattttgccatttcttttaa
>KolobokP-1F_PhoAus-I
ttaaataaaaccacattttccaaattttgttcagaatagttcctctccacctgctgacctattttgcaaa
gtaaaaacttgaagcgatgaccggttttgatttttcaagccgattgcatgtgcccacccaaaaactggga
tttttgaactagggccatttaacagacaaagacaccaaatgcaatttcttgccctgttttgcactaaact
gacttttgattgagggaattggggaaaattgataaattagcatatgaaaataacaataaaagtttaattt
aagttaatttcctgaccccacaagtggaacatgttctcaccaaggcccattgtgtataggggacatattt
attagagtcgatttgcataaccacaaaagtcatgagaacaagaaatttggcagccaagtttcattcacaa
cataactgttgagtggtgagcacacacaatgtatcctgaaaagacacgcagtttcactaggagcagaggg
agaagaaaaagccaatttaaaaaaggacacagaccctccaacatggaccactgcagctccagcataccaa
ggcagcccgaggcagcaatagacggttgcagggctgtgcgtctcacacatttcgaagctgaggatgtcac
ctacctaactgacagtgatgctgctgcgacagcttacaggtttaggccaagtactgaaaaagaaagaaaa
attcgtgacagcaaaggactggatgagaatatcatcattaacgtgcgtcgcttaacaggcctggtgggac
atgtgcatcaggttgcatgcaaatttggaaaactagaactaattgttgaaaaaagactgggactgtgtgt
ctatgcatcattgcactgcagagtttgcaaatataaatcgcccactgtgccactgtcagatgaggtcaag
cagcagacgactgggccaccagctggagccttgaacagccaggctgtattgcctgtgctaaagtcaaaag
tgggaattgaggatgtaatcaacattctgacatgcttgaatattgtgccaccatccaatcgcagcctgca
gaataaaatgaatgccatgagccaaacagtaactcaactgaacgaagaacaaatgattgagaaccagcgc
tacgttaaaaaagtcaccactctggctggcggtgaaggtgttgcagacgttcagtttgacgtttcctact

ccagtaggccacagggtggctgcgagaaagctaagcagagcttcggtggacttgttgagcacacaacgac
ccgaaaacttccactggcccttgccacggcgaacaaacactgcaggattcccgggtgcaagcatgaacac
tgcagcaaaaactacccagctgagaaatcaattgcatcaagtgaacgcacactgttacaagccagcctca
gagcagtggaaaacagtggtgtggacctgaatatcaggagcattacgaccgatgccagcagccaagaggc
aaaggccatccgtgatttctacagtgcaaaaacaaaaaaacccaaacactacaaatgcttcattcacagg
ctgaggcgaatgggaaagagcgtaaacgaagcggacttgaaaagtttgcccaggacacataacaagaccg
gttacaagaggaaactttcatctgctgtgcgatccagggtgcgaaaagagctcatcaacctaaaaaacag
aaaaatcggaagagatgaatttttaaaaattggagctgctgcagtgaacaatgttactagctgtttttca
ggacagcaccagcaatgtcgcgagctatcatctgtgtgcattgcacatcatagcaactatacaacaagcc
acttgccttatgaccggcatctagaactcagtgaaaaagacttgaaaacaattcaaacaaaaatcaactc
ctacttcaaccaggaacagctagcagatatgaagtatctattcaacacgaacatttgcgaaagtttaaat
gcagcagtttatgcatacgcaccaaaaacttcgcgatacacacggaatttcacggggctgtgccattcag
ccatacattccagaaccctgggacaagggaaatccacggtgaagctggccgaggcatcaggactatccgt
gcctgaaaacagtgccatgaaaagaaagttgagaaatgtggactgcagaaagctttatcatgccacacga
aagtcgagccaacaatacaaaaaggccaggtactacctaagggagaggatggccaacacgaatatgtttg
acaaatctgtgtacagtacggaaaacatggcatctactagtgctactgaccacagctacggacttagtta
ctaactgtattttagccaaagcaaaaaccaaacgacaactggaaaaatggaacttaaaagctcttgatag
gattaccatattcagcaggcatgatttgtatccccaatgaccagtgcattccccattatttttgcaaatt
tgcctggagttgttaattttggggggttcataagaaaggtgagagaattttatacaggaagggttatgac
agaggtgggaaacgtggtgggactgggacagccgctggggcgtgccatgcaagtaataagacaagcgatg
agcctgtactctgatatatttccctcgaaatttaacacagtgaacaccgtatcccttcttatttacagga
cctgtccacagctgacactgtcccacagggtgagattttaaagagagggtttgttttagactatgggatt
cttggttatttaagggtgcttggaagaatgcactggccatggcagagagagggttgttgtacctgtaaaa
gcatattttgcagttgtttttaattgcactgtagcacactgttgctgatgaacattaaattgtcatttga
aatgcattaatgagtgtctaaagttaatcgcaagcaccacatccacacccagctgccgggctcctgtgcc
accagctggtccaagtacacagtcacacacaattctttcttcaatttttacaaaaaaatgaaaaaaatat
tcaacaaaaactattgttctcactttgatttgtggcagagagaagaattagtgccaaaactttgaaatat
ttcaaacttgaaatttcgccatttcttttaa
>KolobokP-1_BBe-I
tgtaagcagtccgaagttcgaaactgacaagtgacaacaccatcaaagaataaagagaatagttctacac
aacttgtccaagtttgaaagggtttcattgaacggtgactatgtagcactaactttccttgcaagtgtca
gaggcatgtattcaggcagtgatatagcattgacagacagggcagtctatgtagtagaatcatgtctgag
gacaagaaatggtgagaaaagatgcaagaaatgtcaaaaactggtcactctgatagagcaatctattatg
ctgtgtcagagacttcacttttctgaataaaagttgctaaacagcattttagggcatttcttgaggtgtt
tattgcaaaagtggttaacaggatgaaattggccatggtttttaagttatttagactatttgtggtggta
tgtcattattagaccctgaattcatggcctgacacttgctggtgttatttctaataaaaaaaattgaagg
aaaacaatgggaacaggagttgaactgccttctcatgagaaccaaacaagccccccccccccaagacac
acacaggtgagaagtggctctcaggccaagcaaaatccatatggtgtcatggtcactcccaacccccctcc
ctgcctccgggaaagtgatggctttataagggatggggacggtcaggtacagcacacttgattgtgacga
caggactgcctacatcaagacgccatcatgccacggaagaagaagaacaaggagaagcggggtaggccat
tcatccctggccacacaccatggaacaagggctgtgttgtcgagcgggaaaaggtcacctcttcatactg
caggccaactcaagatcaggaaggtctgtacatcaaccgagacaggaagggcaacatcctaccgaccgac
actgaaacccttaatgaggcaggaacactgatggtgcttcgtaccacacagctgccaggtagtaagattt
caaggcttctgggggacaaaacagacggggatgaagttgagggctaccggctctggcatgccaagacaac
tgtcaaagccctgtacagtctacagagagaacacgacgagctccggcctgactgcaagtgtatgcttcga
ctctcggcacagggagaaaggaaaaagggactggctactcaggagacactggtatgtgacaactgcggat
atgaatcacccaggacaaagttctacaccgaagtaccaatggagggacccggggccaagacagcagtccc
caaccgggcagtgcaggtcgccatgtttaacagcccagtaggacctaccgtagtaagagagatggcagca
gcccttgacatgccggtgccaacccgatctggactacaggaaggggcaaacaagtacagtgagttgatgg
tccaagagaatgaagcggacatgcagtcatggcgagagacagtggcggacatccatgtgcagaaaggaaa
cccacctgacagtggcattcccgtagaaggagacaccaagtatcagagccctctacgttctgcaaggact
aagaaaccaggtcaacctagctctaatagtgtcagcaccatcgcagaaaacgtgacgccaaagaagctag
ttatcgggactcatgtcaggaacaagatctgtcaaacacgcaactacaatgagggatgcggtaacccagt
cccaccccacaaatgcccagccaacatctcagaagacacagtgatcggggacgaacgccaagctgggagg
gacatggggaagaaactcctctctggacctgtaaagacacttgttagtgaaactacaacagatggagatg
gggattttgctgctggacttcaggaagtcatgatggaggaggcaggacagaaagtaagggccttctcgga
cacagtgcacctttgcaaagctattagaggcagggtttccagggcaacatggtccaagcaaatgtttcca
gggaacgatcaacaggagagaaaccgcaaccgggacagatttgggaatgacctcagcaaacgtctcaatg
ccgaacacaaagctgcacgtaagaagatcggggacaacaagcggaagatggagaaaaaaatgaagcgact
catgcaggcactgccctactgctatgaaggagatcattcgaggtgctacgagggatcactcatctgcagg
cacccaaggaaatggacatttccagacctacctgaaaaggtgaagggaaacatccacccaaacgatactg
atctcaaggtgttggcaggcataatggaggttcgtttaggaaagaaagctctgaaacggacacggaagtc
aagaaccacaaatcgagttgaaagtgtgaacagacagctggctaagacctgcccaaagaatgtagtgtac
tctagtaccttggaagggagggtagcctctgcagtacacgcgtcgaacaacggaacaggacggtcgatcg
cgctcaaaagggccgcagccaatatccccttctcaccagactctgctgttgtacctgcattagaggggat
ggagaagaaacaagaataccaccggtcatacaagatggaagtaacaaacaagaaaaggcagcaggctaag
gtaaagaaaagatatgctaactatgacattcgtaaagagcaaggcacttacaaaagtaagacagttgcag
ttgcagggcagtctaaaggcaagggtaaagccttaaggaagaaagtaaagaaaaggatgatagaacattc
atacagccgtgctgcagaagaggaatcctccagtgagtactcagaaggatgaagaaaaactgtgacttac
cttgacttgtcatctagaaaaatatacactgccgatagccatcatgccctctacactctcgtctagacag
gcaaacttttctgttgttgtttactagccttggttccagggacaaatgttctacatttacacgaaagggg
gtgtggcacaaatgggaaacgtctagttctccaagctgtagatttgcagagctgatcatgtatgctgccc
tatgcacgtaaaacttccgaggggtgaactgggggcgttggggaaccaggagacgttaacgacaccgta
gtttgacttaggcgacgactttccacccgtccaaagcagacacgatgttgtaggatggggagctactcta
gaggctatcttcttcctgtattcctcacaccagtgttgtatagaagccatgtatgccagaaaaacaacta
gaaactgaataaaatgctgtataaatgattccagatgtgtccttgtattctctgtgagtgtcttagggtg
tcaggtttcaagaagttgtgagaaaacttacctatttcgcgccattcctctgaacccaaatttgtgcag

>KolobokP-2_BBe-I
ttgaaccaacttgaaccctattttcttttaatttatacaagttaacaacatgtcacaaaggtgtgtgcca
aatatctgtattgtacggcggactttcgctgagatattccaacttttgtactttttttgaaaatcgcttcg
tcactcaatcaggccctgtccttggtgctataatgcaaggcaggcagaccacatgtactgatatcttgaa
gtcgtgtattaaacgttttcaaacggaaattaactgtctttgtgctttgttgacatccttgttagggaaa
ttctgcagtgtatacagtgttctttgagtagggacaggaagagtgagtgcgtgtatgtgcgtgtatttgt
gtgtatttcggtatgcatgtatgtatgcatgtgtgtatgtatgtatgtatgtatgtatgtatgtatacgt
gtgcgcgtgtttattcacttattcatgagaaaccatgggttcctggtggttttcaggtgtaatgattgaa
aagaggtgggtgcatatgggttaagactacagatctcattgcggtatatgcagctaggaggcccccatat
ggttcagggcccggtgtcatgggcgattctcacggccggcccctttcgcacgtgtttgtaacactttggt
tcacagcttgatgtggattaattagctaagatggtacaacaataagctttgatggatcccatgtctctga
gtggggcgggttgtaggcggagacggacatgtaaccaattatacgtctattaacacgcacattgcaaatc
ttgcacaagtttcgccattatcgttccacgctgtctcagaccacactacctctattgcattttccgccaa
agttacttcaactatggggagagtgtctggcaggcgagctgtaaaagctcggaaccgtcgcccccagacc
tttttacctgggaataccccttggaacaaaggtttggtagcagaggggggacatacggacgccggtgtct
cacccacggaggacggggaaccgacagtgcgaggggggacagacatcatccacgccaagtacttcgacgat
cgacttgaagaaaaggttggatgaaaagacgtattcgatgttacacagacgaggagaccacatcctcctc
ccctgccttcggaaacgaacgaagagagtggactacacagagccgaagcggccgtacaccaacaacaagg
tacttggaaacaggattgtgaacatggacaagttggcagaactggtcaatcttttcattgcgggccacag
aaaagcaaacggaaggtgtaagggcgagtgcaggttcttaaagaacttggaaagcaagcttggcttaggt
gttaaagaggcgctttcctgtaaggcctgtggctacaagacaacggccacggaactgttccagagggccg
acacgcgacgaccgggggcacgtggaccgcttgcagtgaagctcaacatccaggctgtgacacctggggt
gaaggacaggtatggtcctactgctctgaagcctctttttgcgtcgctggacattccagatcctggccgt
accaccatgcagaagaaaatgacggaagcaagtgaggtgtacgccgacctgaacaaggcgcagatggaac
agaactgcgctacattggcagaggtgaagcggcatcgtatcgaggcgggacttagcgaaacttctaccat
cattactgagagcgatgccgcctacaacaacccaccaaagggtaggtcaaggggacaaccgggaactcag
gcggaatgtcctacgtttgagaaagaaacgggccaggatatgctgctagcattgacaacaacaagtcagc
actgtaaggtctgcgaaaatcgacgacgcagagggctcccgaccggtcctggctcccatagaaattgtac
ccaaactttcgacaggacgaagaagatgggtgctgccgaatacgagatggcaaagaagaacacagaagct
gtgttgaagtgcagtggtctagctgccggcacgcactgtacggacaatgatagtaagaacataaaaggcg
tgaacgacgcacttcgcgaagccggtctccctcccgcagaaaagcaagactgcatccttcaccacaaaag
aaaccatcgcgccagagtgtttcccttggacctcgaagttttccaaggtgggaacgcgacacaagaggac
aggaagcagagaagtcgcagggtagggcactacattgttgacaggtgcgacactgcactacgcagggtta
ggaagctacaccgtgactgcgacacagactttttcgacgaggttgacaggtgtatccgccctactctttt
aaactgcattgcaggtgatcactctgagtgtacatatgagactgcgtgcccggagcacagcctagaacaa
catccagctccagacattccagcaggcggaaacttggccttgacgaagggcgaccgtgacgtcctccagg
ctctcgtggactatcggctagctccggagacagtgaaacgccaaaaaggcttaatgacgacgaataagag
tgaaagttttcacaaacgcgtaacaaaggcctgcccaaagtcaatgaattttccaaccaactaccacggt
cgggtcaatgccgctggcctggcggactctatcggctcgggcgccgctatccgacgggcgaatgagatga
tgggcgcgtctcattcagatgggagtccaggggcagctggacttgaggcgattgaccgggaggagaagta
ccaccaagacagaaagaagacatggaagtacaaaaacaggcgctacaagctgggaaaagccaaagcgcac
gccaaggtcaccaagtcgggagaaggatacagcacggactgcatgcatcctatggtcaggagtgaccatt
cctacaccaaaaactgatcgaatccaaagaatgagaaggacaacaaaggttgagaaacggcagaaaagac
tagcaaaagaactatcaaaagactatccttttccgtaaagtcgtgagggacaccgttaggtggggaggtg
tgtgttggaggggggggggtcatggtagaacgcaatccgggtacccttggtgggtaacacagccgccata
cgcattgttaacacaaacctgtcgagaattgttgacagaagctggctctagggacaagtgtgcctcagct
atacagtgtttctgatgacaaagatgagatacgtgtagagttgggtccaggaacccgtgaactaagaagt
aagcaaccctatgcagggctacgtccctgcatttcggccctatctgcacgctgattctgccatagccgtc
tctatctagacgaccttgccataaacggcatcccctaccacttgccgacactccttcagctagtctctgt
cttatcttagccttataaccttcgctccatgccgtaaagtcagccatgactgagtatgtaccgctatt
>KolobokP-3_BBe-I
ttctggtagagaagaatacctagaataaccgttttcaacacatcacacttaaggggaaagacttctaaga
cacatctgaaagtatcaaagctttctgacagataataagcatatgggagccataactctaacaggtgtct
gacagggccaggaggcagccatcatgggcctggctgtgtatgcactgcctgtgatacacacacagtagca
ccatttcttcagatttgagagcttaaatgtcaaaacctgatacatttcaataaaggtgaaaagactgag
cataaaaagccaccatgtattttaagtttgcacaccactttcgtaacatttgtaggcctttaaaagtttt
gtcctcaaaagtaggtcaaaccacttctctacacttctatgaatttttttagccatcaaagttcactttaa
tagaaagatgagccctactcgtggtcctgacacacacctgagttactgtggttcctggaaatctgaagga
acgttggaaatgacaggaaaatgacaggaatatccccattgcatgtccacttcctgtgccttcctggaat
ggtgacctcacaactccctctccccccctgggggccctggggcaattcattacccacccccaggccctgt
caagccccctataaacttgagtagccctctccaagcaagtcagacttcatcatgccgcgaggatcgaaga
accgtcagagctcccagttcaagaaaggacacacaccatggaaccgtggccagaagatgcatcgtgaaac
acagccggatccacccacctaccagagaccaacagaagaacaatatcatcttctggtgaatacagacaga
caaggagaaccaattgaagacctcgccaggatagccgtggaggaaagtcgacctcctctgcttcgcccca
agaaggaaccgagcaagatggacaggatcctgggcatagaggaagacgaggatgggagggatgaagaaga
tgaagaagatgacactatttccgggtacaggtactggctgggacatgctgccatgatggcttgcgccacc
gcccagagacagcatgacgacaggtcttcttgtaggaagctcatctacgcatcagcgaagagagaggaga
cgaggggaattgtgtcctcggaaactttcgtctgcaacgcctgtgagtacgtttcacaatcatataagtg
ctatgaggaggtggagacggggaagagaggcaagaaactagcagcccaaacatggcattacaaatagcac
tattcaataacccaataggaccaaagaactttatggagatcgctgccactctagatttaccagtacccag
cctgtctggcatgcagaagatggcgaaccgatatgcagaaatcattgtggaggagaacgacaaggacatg
aagagatgggggagatcgtgttggggaccaatgaagtcacaggaaaaccagcagacaggccaatcaaggg
gaggggggatgtgaggttccaaagcgccctcaattccggaagaggacgtaggccgggccagccatcatccc
atgcctttgcaacctttgcggaaaatgagactaacatgaaaaaaatcctggcaacatatctcagaaacaa
acaatgtgcgaagtgcaacctagggcacgtgggtcgaggagtgaagaggagccwcaattgttcagcaaac
atcgccaagcatgttgtgatagggggatgaggcagcactaggccgtgagatgggaaagaaacttgtcgaga
ataacatcagggtgtctcaccttgtacatgatggagatggacatatcttcaaggaatgtctgaggtgat

ggaagaggaaacaggtgagcccacaaagtccttgtctgacaatatccacctgtccagaagtattgccagg
gcagtgacaaaggctacatggagtgcccacatgtggccaggaaaaactcgggcggagaggatgcaagtaa
agaacaggtttggggatgaccttaaactacgtctagaagcagagcaccggcaggcacgcgagaaatatgg
caagaacaaggagaggatggaagaggccatgaacaaagcatcagatgccatagtgaattgctacttagat
ggagaccacaccctgtgtaggactcagtcacttgtatgcagtggaagcagtaaggtgtgggacttcagtt
tctttcctcatggcacaaaagaactcatctgccctgacgaggcagacagggaagagttgaggactatcat
aggaaagaggctggaccctgaggttctagaagccacaagatttggtcttaacacgaatcgtgcggaatct
gcaaatcgtcagtactctaaaagtgttccaaagaatcgtaccttgactacaaccttggctggtcactacg
cgtcagctgttcacagtgccaacaacgggacagctctgtctatcctgatgaagcgccaggcagccgggat
accgctgtctccgaggtcaccagcagtcaccgccctacgagcaatggacaaaatcgaaacttacaagaga
gcctaccacaaagaacctgctagaaggagtcgtcgcaaaaccaacagggtgaaagaattccaaacatata
atgaagccaaagacataggacactacaagagcaaaggtgaggaagaggaagagggcgaaaaagctggtcc
aagcgagccaaagaaaaggagagtttccaacaagcagcagaggggtaaacaggatcatgtatacagcagg
atggatggtgaatccctaccaacctggtccaaaaagaagaaggagtcacagtggtctggtgaggaggaca
actaaaagtacacccctacctcagtcctcaaagatacagtcagggtacccagggtgcttccgacaaacgc
ggttaaatctgcacctttgcctggaattgtttacagagtgcggttcagcagacaaatggtcaatattaac
acacagtgacgtatggcagcggtgagaaacatccagtcttcccaggtccagcctgcctgtcacagccatg
aagaacacacggtggactgtccacttgcttggcctgcgaaaaactctcccgggaaaccaagagacagaaa
tgatgccgtaaaggacttgactcctagtaactttggctccctgccatatattgcatgattttgtggaatg
tttgcgtagcctacgttgtattttagatgagtattctcttctccagtccctttctgccatttcgggcgca
aaaagtcgttgaaagtgtcgaaatttgtgaaactgatctgaataaagttgtcagaaggaatgtccaagtg
tttgtccatgtgttttagataaaaaaacggaaggaatttgcatagaaaagtcgtttagaaacagctatgc
tgaggggggggcagttca
>KolobokP-1_BJ-I
ttaatgtcttttgtttttttccttttggacttaaaataactcaagacactgtactttactcactgccaaag
aaaatatctggatacgattattctataccctgtaacaggtgcacctagaacgcatgccaagtggctgttt
tttgaggmcgcctagcggacaaactgcgcgctgcagstcatttggcgttgtcatgtatttgagtccttks
gcacttaamggcatctattagataaccmaaaagacattttacatgtcggaakgaaasaggagtttaattc
ctgttagatagatatccttgtggaawagtttttacgtcgcagcacacaaatgtatgtcatgtatacttga
agctgggcagttaatttcagcattacaacgggttagttggaatgtgttttccataagttcagtmcataaa
gacataamatwtacgcaagataatcwacgaacacatatgtatatccgtgttgtcccaaaaakgtgatttc
awtccgaaatttacaggacgagcggatacctacactkagcactacattgatkgattggaatggagkgccg
ggtacgacacggccccttctcgaacatggtgtggttagaacgtcacaatgaaacaattgttgatcaata
aaggagtaataaagatttggttgttgcgtgtgttctattttgaamattcgtgaaaatgtggtgtagctga
gttcmatccacgctagaacacggctgcaaagccgcagacatggccagccggcgtggccgtccttcmagga
aaaccctgagacatggcagagagggattcagggatgggcatccatactacccaccaaagagatccgagac
aacgacagataagtatgagcggcttaatcaacagatgtacaacatggcgcaggacgacgccaaatgccca
acaccaggcagactaaggccaacagaagacacacccagcctcctagaacgcatggctaaagtgcaggtgc
gaactgaaaacgtaccagaggcacgggacttctacgtcccatatttgatggacctgtttaacactttcat
tagagaacactacacaacacaaccacactgcacacaccctgtcgtcaccatgcccccgacactcgaaaag
aaatttggcacaggctacaaagaagcactccgctgtaacacctgcgggtatcacactaaacgacagaagc
tttacctggaagcagataggcagggaggtgccactagaggcccaaaaccaacgaggaaaggagtacaact
gtccatggcactctcaaagttgccaattggaataacaggagcgacgaccctcctgagtgccacagaaact
ggcactccgagtcagagcaaattgcaaaacataacgaacaaactaacagatctggtaatatcggaaatga
acgaatctctagctgataatagggcaaagcttaggaasgtttgtgaggtgcaggggggaagaagtaaagga
cggtgaagctgttctaaccacggccgcgatggacggcgcttacaacaatccttcgtatcgcacatacacc
caaaactccacacaggtaacagtaccggtataccagtcattgagaagaaaacagggcaagacatgtggat
agggtttgcgtttgcaagtaagttgtgtagctgtgggcgggatggaaaagacagggagagccatgatccg
agttgcatggccaactatcctcgggccgacccaatagggaaagcggaggaggtgttggcagggcgagccg
tcgaacagatgcaagagaaacacccagttttatttgacaaacttttaagcgacaatgatgcccacattat
aaacggtgttcgggccgcaaccaaaaagatttcccaaagtcttgaggtagaaaaactagactgtgacgta
cacgtctccaggggccagaaaaggaaaatgtttagaatgtctgaggtgctgagtaaccagctagtggcgg
gtgtgagtgatagttccgtcaaagtgatccaaaaaaaaagaacttggtttgccaccgcccttgggagagc
gatctccaaacgctgtagcatagagctacgccgggcacgaacaaaccacccgcacaatgatgatctcttt
aaacttgcagtacaacaggctaaggataatataataagttgtttcgctggtaatcacacaaagtgcaatg
aattgtccctcgtgtgccaggccggccccgaccataggcccacttatttaccgcgatgccagtacatctt
accaactgaagacgatatcaaacacttgcaaacggtgatagattataaattatcaaaagaaatggtccat
cgacagcgttatgtgatgtcaaccaacgcagtggaagctgcgcatagacggacacaacaatctttgccca
aaaacaatacatttaggtcgtcaggttccatgcgcgcaagcagcgccgcgcattcgggtgcagtcggtgg
gctaggagaatctctttatcgtctcaaccaagctgccggttcaggcttcatgcctggtggcacagcttgc
cgtgccttgctaggtatagatcggagagcagcttaccacaggaaacggaagcaaagcgtagcatacacaa
agaggagaaaggcccttgcaaaacggaaactaatgctgtccaaacaacaccttatgtatagccaggccac
gtacaagaccgatgttgaacacgaacaagagatggaattaaaatgagaaagctctgtattttttttcttg
tcttttgtgtgtgtgtgtgtgtgtgtgtgtgtgtgtgtgtgtgtgtgtgtgtgtgtgtgtgtgtgtgtgt
gtgggggggtgagtgagtgagtgagtgagtgagtgagtgagtgagtgagtgagtgagtgagtgagtgtga
gtgtgtgtgtgggggggggggggggtacctgtttgtcactctatgatgcagtcccgaaatggcttgtggc
tccccgtgcaagcaccatccgtccggcaccggttcctctgtgcgttgatggtgggtggctcatacgaaag
gtgctccatccggacacacagcttccgatggcatctgtggctgacgtggatgtgtgggtctagcgccggc
cagttggagttgacgaaatatgccagtcggtgaacctgaagagtgtattttccgccgatttcgggccgag
caacgtagcttccgtagccgtctttgtcaacatagcccgtccagacacgacagttgccaatatcctgact
tctttgagttaaaagttccttaagttgggccaagtagggtcctgtgggcctagagctgtagaattccgtc
attgttgatgtctgttttagaaatgagggttgtcctccgagctctccgcatgcgcaccatagtaaaaagt
ggaaatcggttgtttccgcaaaaattgatgacagctgtaccatccccagatgggcgcgagacgcatacca
cacggtatttagttacgattcgaattcaagctccccatccatcaaatttacagtcaacaccatatatcac
ggccctggacattagctagggaactattctggccttcctactttaatccgctctaacttttaatctatac
cttcctataaagatgacgtcattggacccagttatgaaaattggagaaattacaacggtttcagatggct
ccgacttcttttgtagccctatctacaaaaagaacttgaattttccccgtcggcttttattgggctgtga

ccaaaaactgtagtggaagacgatgacgtcatcgatattcatgtcacgtgatctacagacaattggcgaa
cttaacatggaggcaatgcttcgccaggggtgggagggggcggttgaaaaatctgaaatattaaaacat
gggaatttcgaaatttttcgacaggattacatttagtatatacactttcctagataaaacaccatttcc
gtggttgtaaccttatttttactccggcacagctccgtttaa
>KolobokP-2_BJ-I
ttaatcaacattgaactttttttttctttaattcgtttaagttgacaacaagtctggaatatgtgtgcca
aatttcacggcagtccattggaattccgctgagttattgccaactttgtactttgtacgtttcatgtcgt
catcctggtgctgtccttggtgctgtcaatcagggcgcatgctatgtagtatgatgtagaagttctgtat
taaaatgcttccaagaggaaatttacactgtctgtggtttcttgacatcattaaagggaaatatggtaga
aatatgtaccatacagtagctttgtcgggaggtggggtgacgtacgaaacaaggatgggtgcgtgcgggg
cgtcggaagtttgggttagggactttagggtgtaaagtaacagaaagaaatctcgaaatccgtatacttt
atcaagatagaaatcttaggtgcacgtggctacctggcgggcatatcaaagcgtaactttgttgacagtt
tacacacagcgtgacactaaacataatcagtatcacagacgtagcacgtggccacaattgttatgggaat
gactgtaccgtcgcctttcgtctcaaaggcggcgcattattgtttcgggtacaatgttacaagatttcta
aatttcaattgtcatgtaaggagccgttatgatcagttgtcacctgcgctctaaaacgcggcatccctta
gaagtgtaaagtgcgggctggacattatcaggtattccccgttattcttaattgtcgacttactactgag
cggacaacacacactagtattctatcgaagctactttcagtatggggagaatatctggcaaacgagcggc
caagcttaggcttcgccgccgcccacaatgctttcaacctggaaacactgttgcgaagaaagatggggac
gtgcgacgtgcgacgggcggacaaactagcactgcagttactgaagaaccgttgaacagtgacggctctg
tggacatcgagcacgcggccttgacaattgacccaaggaagaggttggaccaggacacataccgtatgct
gtacagacctggtgatcatgtcatcattccgcgctttcggatcaccaagaaaaagaactacagtgaacgg
aggccctacaccaaggacgaggtgacaggaaacagaattgtggacatgcgaaaggtggaggaacttatga
acctttacaacgccgggcacagaaaagcaaacggaaggaaagtgtgtaatggagagtgcaagttttctgg
gaaactggaaaggaaactgggctttggctgcaaagaggtactccgctgcaagacatgcgggtataagacc
acgccagcagaactgtttcagcgtgtagaccgcagacggcctggcacacgcgggccgctccctgtcaaga
tgaacatccaggctgtcacccctggagcaaaggaccggtatggtcccgccgctctaggaccgctctttgc
gtctctgaatgttcctggcccttcccgcacggtagcacagagaaagatgactgaggcgagcagtgccttt
cagaagttgaacaaggaacagatgcaaaagaactgcaacgtgcttgcggaggtgaagaagcttcgcctgg
ctgcgggtctgaaggaaagcgctggggtttcagcagagagtgatgtgggttataacaacccacccaaggg
aaggtctagaggacaaccaggcacacaagcagactgtcctcttttcgaacaagatacgcataagaagatg
ctcttgtccttgtggacagagagccagcactgtgccgggtgcgagcagcgacgccgacagggactaccta
cgggccctgaatatcatgacggctgcacacaaacatttgacagaactttaaagatggggaacagcgaggg
aaaaatggcgaagaagaacgcgacggctgtcctggagtccagcgggttggcgttagagggtcttactacc
gacaatgacagtaagatcgtgaatggaaccaacgaagcccttcgtgacgccaacctgccgcctgtccaga
agcaggactgcatgcagcataacaagaggaaccatcggaacagagttttccctctggatctcgaggtgtt
tcaaggagggaacaacacgaaggacagaagaaagaaagcaagccatgatgtagggaactacatcgtggag
acgtgtggtaaggctctgtataaagcgcgcgctcgaaaccccgacaacgacgaggccttctttggggaac
ttgagcatctccgcttgacgctagtggattgtcttgctggggaacactcacgctgcactgcggatttcgt
atgcccggcaaacagcccagatgatgacgggcctaccccagacctacccggaggtggaaacctggcactc
gagaaggctgaccgcggtgctctccaggcccttatagactaccgcctcgccccaaacgccgttgcacgac
agaagaagttaaggacaaccaacaaatgtgaagcattccacaacagagccctaaaggcagttccaaaatc
gttaaactttaaacacaactattcaggacgcgtgcacgccgctggtctggcggactcgatcgggtcttgc
gccgctatcgaccgtgtcaacagctttatgggcgcgagtcattcccctggcagccctgggcaagctgcat
ttgaagccattgacaaggaagaggattaccacaggaagaggaaacggagcattgggtaccgcatgcgacg
atgcaaccttcgcaaatcaaaaacagtaccaaaggatgcgaatgggcaagaatacagcacggattcaatg
caccctagtgtaaggaatgaccattcgtactccaaatgagagagaaacgaacgaacgaacgaacgaacga
acgaacgaacgaacgaacggcagaactggtacctacagggaggatagataggcaacattctgacacatcc
tgacgaactttggggggttggacacacattgggtggtcggcgggggggggggtgtcatcccagtacgcaat
ccatgtagccatcgtggtggctgcatttaccagaatgaacacagattcggcggctgtggttcacagccgc
cggttccagagagaggtgctggcttgccacgcagtttttacggtgacacacatgggaaacgtggctagaa
tggtccagatagccatgatacaggaagaaaaccacgcgatgcaggccagcgtccttgtttttggcgccga
cctggacactgattcgcccgtaaccgcacttgtctaggcgtccgtgccacagaatgcagcctctgtcagc
ctgagacactctatcagctattctttgtcgtaaaattgccctgtaggcctcgctccatgagttatacccc
tccatttgcaaagtttcgtaactgtattaa
>KolobokP-3_BJ-I
gggctgcagagctccgaacttcgaaactgacaagtgacaacaccatcaagcaataaagataagagttcta
cacaacttgtccaagtttgaaagagttttaataaagcatgcctgtgtagcaccaactttccttctaagtg
tcagggactgtattggagtactaaaatggcactcacaacacagggtaagccctgacagtggcatcatgtt
aaagggcaggaaatggtgagaaaagatgcaaccaatgccaaacactggtcattctgatagagaaattcac
tttgttttggtctgggacttcacttttctgaataaaaggtgatataacaacatttttgggcatttttttgg
aggtgttacttgcagatttagtggctacaggatgaagttggtcaacttagtcatgttttaagaagttgtt
tacaagatttgtggtgatatgccactgtcagaccctgaacttattccctgacacttgcttgatctcagta
ctaattaaaaaaaacccaaactgaagcaaaaacaatcttgggaactgcagttccctcccttctcatggga
accagtcaagccccccctcccacccccaacacacaaccaggtgagaaatggttcaggctggggccaagcca
tatggtttggctgtcactcccccacccccccacccccagcctcgggaaagggatagctttataagggtcgg
gaacgggaaggtagatcacactcgagacgcaacatcgtgaagacaggactgcctacccacatcaagacac
caccatgccacgaaagaagaagaagagactgagccacggttgccagttcagagctggccgtacaccttgg
aacaagggttataccctcgtgcagcaaaaggtcatctcttcatactgcaggccaactgaagacgaggaca
acctatatgtcaacagagataggaagggaaacatcctacagaacgacatcgaaaccctgaatgaggtagg
aacaacaatggtgcttcgtacgacaaagttgccaggtagcaagattgccaggtttctgggtgacaaaaca
gatggggatgaagtcgagggatatcgtatctggcacgccaagaaagccgtccaagcctgcgcaagtgctc
aaagacaacacgacgagctccacccagactgcaagggtcttgtcagactgtctgcacagggagagaggaa
gaaaggactggccaccctggagacactggtttgtgacgactgcagttatgctgctcctaagacaaagttt
tactccgaggtagaaacacagggtccaggtgccaagactgcagtacccaacctcgcagtgcaggtcgcca
tgttcaacagtccggtaggacctacagtaatcagagagatggcagctgccctggacataactgtgccgtc
ccgatctggacttcaggaaggggcaaacaagtacagtgacttgatggttacagaaaatgaagcagacatg
caagcttggcaagagacagtgcagggaatccatgaggagaagggaaatgaagccggcagtggcatcccca

tagaaggcgatggcaggtaccagagcccgctccgttctgcaagaggcaagaagccaggacaacccagttc
aaacagtatcaccactgtcgctgaaaacgtgactccaaagaagcttgttatcgccgcccatgtcagaaac
aagatctgcccaacacacaactataatgaaggatgtggaaaaccagtcccaccccacaaatgtccagcca
acatctcagaagaaacagtgatcggtgacgaacgacaagctggaaaggacattgggatgaaactcctctc
tggacctgtcaagacacttgttagtgagacgacaaatgatcaagatggtgactttgctgctggccttcaa
gaagtgatgatggagcaggcaggacaaaaagtcagagctttcacagacactgtacaccttggcaaaagta
ttagaaacagggtgaccagggccacgtggtccaaggagatgtttccaggggataaccaggaagagagaaa
ccgggtccgggacagatttgggaacgacctgagcaaacggctcagggcagaacgcaggaaaggacttaag
aagttccgtcagaagaggcaaattgaggaaagaatgaagcgagtaatgcagacactgccatactgttatt
caggaaaccatgagaggtgctacgagggatcactcatctgcagacatccaaggaaatggacatttccaga
cttacctgccaaggccaagggcaacatcaatccaaacgactctgacctcaaggtgttacgagacataatg
gaggttcggctaggcaagaaagctttggaaaaaacgagacgagggaggaccacgaaccgtgttgaaagcc
ttaaccgaacgctaagcaagaactgccccaaaaatatagttcattctcgtaccttagaggggagggtggc
ctctgcaattcactcttcaaacaacgggacggggatgtcaatagccctcaaacgtgctgcagcaaagatc
ccactgtcgcccgattcagctgtggtacctgcattggaagggatggagagaaagcagagctatcatcgat
cctacaagatggaagaagcaaacaagagaaggcagcaagccaaggtaaagcagagatatgctaagtatga
catacgtaaagaagaaggcacttacaaaagtaaaaaagtagcaggacagtctaaaggcaaaggcaaaggc
aagggcaaggcactgaggaagagaaaagacaagacaagtatggaacattcatacagccgggctccagaac
aggaatcccccaacgagtattcagacattgatcaggaagagaaactgtgtgacttaccttgactttttaa
aaaatacacggtggatggtctccatgccctgtgcaatgtaacctagacttgcaaccctttctctgattgt
tcaccactcttggctccagtgataggtgttgtacattaacacatagtggtgtgtgacaaagatgagacac
atctagattgccaagctggagattgcctgtatgggccatgtaagctgctctatggacgtaaaacttacgt
ggggtgaactgggggcgttgacaaaccaagagacattgacaacaccataatttgaagtggaagaacttt
ttccaccagtccaaatgaggcaagaagtacctgggtgagaaactactttagatcctatctttttcttgta
ctcctcacaccaagattgtaggtcatccatttgaggttcaaaactgcctgaacacgtgtacaaatgtgaa
taaaacggtatgtaactgatcccagatgtgtcctagtcttctcttagagtgtgttagggtgtcaggtttc
aagaagtcgtgaaaaaacttacctatttggcgccattctgatgatcccaaagggctgcaggcgacaggac
>KolobokP-4_BJ-I
tgttagggtcccgaacttgacttttgactgatgacaacaccatcaaacaatgcggggaagagttcttagt
atctcctccaagtttgaaacaatttcaatgaaatacgaccccgtagcaccaatttccgtgacaagtgtca
gtgactgtctgccattactggcatggcagaggtacacacagagatggcttaacagtagcaatctgtctgt
aggcagaaaatggtgaaaacagacaaaaataatgccaaacactgatcactctgatagggaaacctgcttt
gccctgtcaggcacaccaattttctgattaaaagatgaaaattaactttttgggcatttggaaaggtgt
atatgtgtataatacccaaaatggctgcaagacgaacttggcaatgtgttatgaaacatttctgtcagta
taccattgtcagactctgaaattaaaccttgacactcatctgatgctcttcatgatacaaaaactcaatt
aaaaccagtgagaaggacacctatctcctttctcatgggaagaagtaaaggcacaggcacacacagtgac
acacactcaggtgagaaatggctcagccaggaagttccagatggcgtggctgtcactccccccctgagtct
ctgggattatggttagctttataagggggggatgagaagatattacacactccgatccattcgagacact
gtgaagacagctccgagtacctatccacctcaagacgacatcatgccacagaagaaaaagaagaagaggc
tgagtcatgctggcctgttcagagctggcaacaaaccttggaacaagggtaagacccttatgaaggtaga
ggccttcagttcctaccataggccaacagaagatctggtccgtctgtatgtcaacacagacaggaaagga
aacattctacagaacgacgacgaaatcgtgaacgaggcaggaagaacaatggtgcttcgcccagcaaagc
tgccaggtagcaaaattgcaagatttctggggaagaaggaagacagcgatgaagtccaaggataccgaat
ctggcacgccaagcttgctgtccacgctgtggcacgtgcacagagagaacacgacaagcgccgaccagaa
tgcacgggcctcgtcaggctgtcagcccgtggagagaggaagaaaggactagccactttggagacgctgg
tatgcgacacttgtgagtacgaatcacctcagaggaagttttatggggaggtaaaatcaaagcgaccggg
tcccaaaattgcgatacccaacatggcagtccaaattgccatgtttaacagtccagtaggaccacaggtt
ctgagagagatggcagctgtcctggatttgccagtcccaagcagatctggactccagaagatggccaaca
agttcagtgaactcatggtacaggagaacgagagagacatgcaagcttggcgggagatagtgcaggacat
ccatgcgcagagaggaaacgaacctggcagcggcattccggtagaaaacgacacaaagtaccatagccca
ctgcgctctgcccgaggcaagaaaccaggtcagccgtccagcaatagtatcaccagtgtagctgagaatg
tgactgcaaggaagcttgtcatcagtacccacgtcaggaacaaagtctgtccaacctgtaactacaatga
agggcgtggagagcctgttcctccccacaaatgtgcagccaacattccacaagatgccgtgataggagac
gaagcagaggcagcaagggagattggcaaaaaacttatgtctggacctgtgaagacaccagtgggtgaga
gtgtgagtgatggagacagcagctgtaccaccggcctacaagaggtaatgatggaggagacaggacaaag
agttactgctttccaggatacagtgcaccttgacaaaagtataaggggcagagtaactagagggggcgtgg
tcaaaggggatgttcccagggcagaaccaggaggagagaaatcgggtgagagaccgcttcggaaatgacc
tgagcaaacggctcagtgccgaacacagaaaagggcgcaagaaattctcaacgaggaagaagatggtggc
aaaaatgaagaaagtgatgcagacaattccagactgctatggaggagaccactctagatgtcccaaaggt
tcacttgtgtgcaggcacccgaggacttggtcattcaaagacttacctgagaaggccaggggacacatca
acccaaatgacgctgaccgcaagttgttagcaaccataatggagaagaggttaggcgagcgagcacttcg
gagaacacggaaaggaagaaacaccaataaagttgaaagttttaacagacagctaagtaagacttgtcca
aaaaatgtcctattcactcgtaccttggagggcagggtagcttccgcagttcactcttccaacaacggga
cggggatgtcaatagcgctcaaacgggctgcagcaaatatccctctgtcaccggattcacctgccgtacc
tgcattggagggaatggagagaaaacaggattaccaccggtcgtacaaacaagaggtggcaaacaagaaa
agacagcaagccaagatagctcagaggtatgctacatatgacacaactaaagaacaaggacttacaaaa
gtaagaaagtggcaggacaatctaaaggtagaggtaaaggtaaaggtttgagaaagaagaagaagaagcc
agtgggcagcaaagaacattcgtattgtcgtcatccagatgaggaatcatcagatagctggtcaccagtt
gaggaggatggagaacaggatgaagaagttgaagaactgtaacttaccttgacttgacttgacttcactc
aaaaagacagtctgccttgtccccgtgccctgtacaattctgttgcaacttgcatactttcctactaata
ttgactgcccttgtctccaaggacagatgagcaggattaacacagagtccattatgacaaagatgtgaaa
cgtccagaccatgcagctcaaaatgtccagtgtaggccatataggcagccctgtgtacatggattttggc
acgtttgaagtctctagagtggggaaaccaggcaatgttaataaaaccataagcagaagatgtagatttc
ttgccaccagtccacagcaaacaagtagaagtaggatgaggctgtactctactctctattttggccttgt
attccaaacaccagttctgcccctcccccatgaagaaaactgtacataaaagtctgtgaaaggctggaaa
acgtgcacgtgtgaataaaaatggtgtataaatgactccagatgtgtccttgtcttgtcttaggctatca
tatttcaaaacgtttgagaaaaactcacctcattggttcatggtgaaatcctgt

>KolobokP-5_BJ-I
ttctaggtacgccaacgagaagattgacagatatcactcacatcaagtcttagggaaaagggttctgagc
aacatacccaaaaatcaaagtcctcccatgatttacaaagctatgacaggtgattatcctacaggtgtca
gtcaggcccttcttgcctgtaaatggcatccagcaatgaggaattgggagcagagcaggggggcagttac
acagctataatctgtcattagaggcaccctctatagtcagatcaggccagtttgtcaacagtgtcaatca
aaacactgacttaatcaccgagtgtctgaacgacacttaaatttgacactttgtgagtatattttgccgc
tgtcttgtaagaggagctgaaatttggaccagtattaggcaagaaagattaactcaagaaaactcaagaa
atgcatgcttattttggtttttcatacaatgattgacacctgtaggatgactactaccatattcctggaa
atcatggggggggggggggtccagaggtctacatacaccatttcttgggtccaaggcattacaagccctg
gttctcagaatgtagcaggggctggggttgacgtcagggctgcagactgcctcccctgggtcaatcaatc
accagcccccagcacttgtcaagaggccttttaataaagggcactaaaggtaacatctcagaaacttgcc
aaccatcaaccaacaaccatgggaaggaagagagccagcaaggacaggccaacattgtttcagaaaggtg
acacaccacacaacaagggcgtgaagaccgagtatgagaaggggggcgacacctgtctaccgcagggtgac
agaagaccagtacgagcttttgcaggacaccgaccgtaaaggtaaccctctggtatgtaagaagaaggta
gtccttgaaaagcgaagggtaatgttgctgaggccaaagaaggagtccgccagtaaggttgcgagggctg
tggctagagactgggatgctggagacaagaacccagatgatgtagttggttaccgcatctgggacgcacg
caaggcagctctggcctgtactactgcacagagggagcacgacgaccaacatccatgctgcagagcactt
gtacggctatcggccaagctagaaacgaaagtgggactagcaacagcagagacatttgtgtgtgacaggt
gcaccttccagtccaaaaagtataagttctacgacgaaatcaagtccaaaccaggaaaaccgggaaggaa
gatggcggtaccaaatatggcagtccaagtggccttagcaaatagctcgatgggagtgagggcatttcgg
gagttcgcagcagccatggacatacccatccctgctgaaagccagatgagggtcaatgccaagaagtaca
gagacatcatgcttgaagaagacaacagggacctgaaagaatggactgaaactgtaaaggagataaatgc
catcgttgggaatgatgcaggtagccctataatgggtgaaggagactcaaggtacaagagtgcactgtat
gctgggctaggaaggaagcctgggcaaccatcaccccatgctttcagcaccttctgtgaggacgtgactg
tacgcaggctcatcctggcacatttcctggcacagaagcagtgctcaacatgccagcggcccaaaaaacg
caaacgtggaaggcctgggagggtagagaagaggaagcacaggtgtcctgcaaatctgaggcaacatgat
gtgatagggaatgaagagctggctgggagggagatcgggaagaaactccttgcaaacaaggtcctggtca
gccacataataacagatggagatagccactttgcaaagggactctctgatgtcatgatggaagcacgagg
gaagcgcaccaaacacctcagagactttgtgcacataggcaggagcgtggccaaggctgtccgccgtgga
acatggagccgccaaatgttccccggcaaaacagccgtgcaaagaaacaaagtcaagtccaggtttgcag
aggagctgcgcgtcaggctcaatgcagagcatcacgccgccgtgttgagatttggcaaaagaagacacaa
gattgaaccagcaatggcaaaggcgatgttagctatacctgtatgctacatggggtcacatgagttgtgc
tcgactggttccctggtctgtaatggcaggagtagatggaaagcagacaggcggtacctacctgtgaaac
acttgaacccagacagctcggacgtgaaagagatgagaaggataatggagaagcgactcggaactcaagt
cttatcatccacgagatacctcatggactctaacaaatctgaatcggttaatcgacaatgttctaagaac
gtgcctaaaagtgtaaacttgatcgaaaccttacctgggaggttctgtacgactgttcacagttccaaca
atggcgtggccttgtcaatcgtgaagcgtagacgggcaaccggtatccccttgtctccaatgtcaccaac
cgtagcagtccttgaaagcatggagagaaatcgtcgttacaggagggcacacagacaggaaccagcatcc
aaagagagggccatggcaaaccgaatccgtcggttcaagacttatgatgagatgaaagaggaggggtacct
acaagtcaggtgatgttcctagtgctgaaaagaagattagactgtccaagaagcagccaagaggaagaat
tgaccatacttacaacagaatggaggggggaaggaccggcttccagcttagattctgatcagtggactgat
gatagtggagatgaatcagacaatgaatttctcttaccttactcttacactaagcgttgaggatgcatgc
cggatagggaccatgaccttgacactgtccccgagcatgacacctttgtctgttgttgttaatgttgtgt
ggttctgcagacagatgtgccggattgatacacagggaggtgtggcagagatgagaaatgtcaaggttct
gaatacggtcgaactggcccctccataccatgtaggcaacacgatgaacatgcatccttttcctcttctc
taaaaccttgccaggcatccaaggaacttggatgaccccgtagctaacwcctacttttctcacagaaagt
cccgtccaaatctgacatgatttggtttgcatgctgacgagtttttctagccagcttctgtttgaactccg
tttgccaatgtgcagcctgccctgccatgattttgtgtcaagagaagtgaaaatgccaattaaaaccaga
tgaaattaatggatacagtgtctgtcttcttcttggtgtcaatttccatgcataaattagccgcttaggg
cagctaacagccgaaacatgatagcacatgatagcccacatggtagttca
>KolobokP-N1_BJ-I
tgttaggggggttcaagttcataactgatagatttcaataatttatcataaaccggtgaagggttgtacac
tatcagccaaagtgtcaaagatttctgatcaggaatggtcccctgggaatacatttggtgacaggtgttt
tggcccttttcctggcctagggaactggtctgaggctattggtggggtccatattactaagaaaaaagta
agaaaaacagaaaaaaaagggaaaatttgctggtcctggcattaggggagtacctttcctctactaaggc
acctcaagttccatcttaaagctgttagatttacctattaaactgtttaatgacagtacatactgcactg
tcaataaaggaaacaaaatctaacaaaaatggtcaaaaacactgaaatcaaagaatttcctaacaaatct
gagcctggcgagtcagctggagcctgcttcctacccaaataactgatttcaggcaaatataagctctcac
tgttaatttgcccccccccccccaagtcaatgtca
>KolobokP-N2_BJ-I
ttaattgggtcaaattttaattaacattggtttttaatttamcccgtcatattctatcaccctgccaagt
ttgagttgtctgcgacttttccttcacgagtaacaggtggttgaaagtcaccatgtcgggaggctcctttt
tgcgcctccacgagcgcgttccattcacacacataatgggaattacgtcacaaagcccttattcttttga
agaggttttactccatgaaactatgtccaatgatcaagtatagccttgtaaaggaatggcagggtagtgt
tcttgcgatggagtagccatttcttttaattctgactacagtwaacggtgtaaaccacctaaaacgcagt
ttagctgatgacgtcacaatgcccacctctagccgatctctttaagtgggaacgacatggttttaagttc
cgtacgtcggtagsaaacagttagatttttaatttttggtcatatctggtagagaataaacctagctatc
tcacccaagcgtttctgctgccaagaktcattttgttttgcaggaaaatgaagctaaatgttgtatatkt
ggtcaggatggaatcctgctctgtccttctagcggctaggacggtataaatgtgcatcttttggggcaag
gctgtgcagtttggtgtcggtctcgmtgtgatatacaagccttgggatgtatcatctgattgctaggatt
tatttgtctgaaagttgaattctagtgcgaggccgaacccgaaacgtgctttggaaggaaagtgcgacag
tgacgtcatcaaggacatttttttcaccaaaawacgtctcctgtgcagtataggactcactgtcaagccca
cgtatgtgatttccaattccccmgggaataggaggaaacaaataacggcgtctcggtwaatatgacgtca
tcggtgtktagtgcagacgcttcgaaggttgtatgaccgtgttcggagggggggggggcgtggggagggg
ggcaaatgagcgcattatatgacattctataatcattttaaaacttgcgaaatccgaawtttttttggctc
gggctgggtagagtatatacactttgctggataaaatgcaatttgttggctgtttgagctaatatttttt
gcaggacgggtccscttaa

>KolobokP-N3_BJ-I
ttaagtacgtcagatcttaattaacattggtttttaattaaccatgtcatattctatcaccctgccaagt
ttgagttgtttgcgacttttccttcacgagtaacaggtggttgaaagttaccatgtcgggaggctctttt
tgcgcctccacgggtgcgttccattcacacacataatgggaattacgtcacaaagcccttattcttttga
agaggttttactccatgaaactatgtccaatgaacaagtatagccttgtaaaggaaaggcagagtggtat
tcttgcgatggagtagcaatttcttctaattctgactacaataaacggtgtaaaccacaaagaacgtagt
ttagctgatgacgtcacaatgcccacctctagccgatctctttaagtgagaacgacatggtttttagttc
cgtacgtcggcaggaaacagttagattttaatttttttggtcatatctggtagagaataaacctagctatc
tcatccaagcgtttctgctgccaagattcattttgttttgcagggaaatgaagctaaaatgttggatatg
tggtcaggatggaatcctgctctgtccttctagcggctaggacggtataaatgttcatcttttggcacaa
ggctgtgtagtttggtgtcggtctcgatgggatatacaagccttgggatgtatcttctgattgctgtgat
ttatttgtctgaaagttgaattctagtgcgataccgaacttgaaacgtgctttggaagggaattgcgaca
gtgacgtcatcgaggacatttttttcacctaaaaacgtctcctctccagtataggactcactggcaaaccc
acatatgtgatttccaattccccagggaataggaggatacaaataacggcgtcttggaaaatatgacgtc
atcggtgtttagtggaggcactttgaaggttgtatgaccgtgttcaggggggggggggcgtggggaggggg
gcaaatcagcgcattttatgacattgtatatacatttaaaaaattgcgaaatccgaattttttttttctgt
ggttggatgaagggtacacactttactggggaaaaatcaatttgtcggctttcattgccactaattttg
caggacgggtcggtttaa
>KolobokP-N4_BJ-I
tgcaatgcaggggtttaagttcaatgctgactgattttaatactttcacataaaccggggaggggttgt
aaactatcatcccaagtctcgaagagttctgaccagaaatggtcccctgggaatacttttggtgacaggt
gtctagggctctttctctggcctagggcactggtctgaggctattggagtggtccatattacctagcaaa
aagtaacaaaaacagagagaaaagtgaaaatttgctggtcctggcaccaggggagtacctatcctccacc
aagggaactaaagttgcatcttgaaattgttagattagccttttaagcagtttaaagacagtacatactg
cactatcaccaaaggatacaaaatctaacaaaaatggccaaaaacactaaaatcaaggaatttctgaaca
aatctgagaccagtgagtcagctgaaggctgctccttgcccaaataactgatttcagggaaaaataagcc
cttacggccagtctggccaccctccccaaagtgctgcaggggtttaagttcaatgctgactgattttaa
tactttcacataaaccggggaggggttgtaaactatcatcccaagtctcgaagagttctgaccagaaatg
gtcccctgggaatacttttggtgacaggtgtctagggctctttctctggcctagggcactggtctgaggc
tattggagtggtccatattacctagcaaaaagtaacaaaaacagagagaaaagtgaaaatttgctggtcc
tggcaccaggggagtacctatcctccaccaagggaactaaagttgcatcttgaaattgttagattagcct
tttaagcagtttaaagacagtacatactgcactatcaccaaaggatacaaaatctaacaaaaatggccaa
aaacactaaaatcaaggaatttctgaacaaatctgagaccagtgagtcagctgaaggctgctccttgccc
aaataactgatttcagggaaaaataagcccttacggccagtctggccaccctccccaaagtgctgca
>KolobokP-1_BF-I
ttcacagagcgaacgttgagaattaaacaataaaactaccatcatacaatggggacaagagttgtgatat
agtatcccagatatgaaggaatttgaatgatttacgaccccgtggcacacacttatatgacaggtgtagt
ggcatctaccacaggccaggagggctaggaaacagtgaatatactagcagaagctgacctgctgttatca
agcattgtggtagaaatagggctagaattgccaaaagctgtccatcctgacacacggtaactgtttctgc
agtgatagagggacacacnccccaccagtacctggctatgtttcatataacaatgcataacaataaactg
atataacaataaactgagagtgaaagggcagacattcacatgcaaaaattgacatgcaaatcaaatacct
gaaatcacacattgctggcgacaatcagtcagaaaagatagctttcagagaggaaggtcacatagcactg
tcaggacaagccatcaccatgccacgtagaaagctaagacatgacggccagttcaaggccaawaacaggc
catggaacaagggattgaagtttagagaacacaagggtacacgtcccacctacccacgacctaccgagga
tgatttcgcactgcttgaggagagagacaggcagggtaatgccaacatttgcaagagagatgtcatccga
gaggagagaaggcccatgctgctccgtcctactcctagccaggcaacttgcggtcagatgagcccctact
tggaggatggtccaggagaagatggagatcaggtactgggatataggatctggcatgcagggctagctgt
acaggcatgtgcgaaggcacagagagagcatgacagccaagagggggtactctgcaaggagctggtcagg
gcgtcttccactggtgagggtaagaaagggctcgctacatcacagactctagtgtgtgatggatgcgggt
acaaatcagccaaggagaacttctacgaggaagttgccagagatgggccaggggggcagagcagccgttcc
taacgcagcccttcaaattgcgctagctgacaaccccatgggcgtgactgcattcagggagatggcagca
gctatggacctgcccgtcccctccgaggcaagcttgcaggagggggctaaccggtacagcgatcttatga
cagccgtgaacgccaacgacgkkmgackctgggcaaaggtggtgawscscmcgamtgtkctgaaggggaa
cgaggcctgatatttgactctattttgtgtctatactctgcacaccacatgtgaagagccatgactagct
tcaaaggcagaatttgccaaaagaacgagaattactggagtttttgcaataaatttccgatgaatgaacc
ttccacgtgtttgtcctttaatctggggtcacagttcacacaggcagacgtaaacaacagctaggaaggg
tatgaatgggggagggggcgtggcacttca
>KolobokP-2_BF-I
tgtcacccccctgaacttgagaattaacagatgacaaccctaccaaacaatgatgaaagtagttttcaac
aacttctccaagtttgaaaaaatttcaatgaaatatgactgcgcagcaatgattttcctgacaagtgtca
atggctgttttcaaatgctgacaaggcagtgggtggggagggaagtggatcaaagtgccaacatggatga
ggtcaaaaattgtgaaaaaaagacacagacaatgtcaaccactggtaaccactggtaaccactggtcact
ctgatagatctacatgctttcctctgttttggacttaaaatttcttctttttttgaactttggaaatatgt
ataatacaaaaatgaataccagaatctcacctttatgtttttagaacacctttaatatgatatttctgcc
tagtatgccactgtcagtatctgaaaccaagtcatggcacttgcctggagctagtgctaatacacaaact
caattaaaatcactggaaagcatcctttctcatgagaaaatggcaagccacacagtcacacacccagaaa
cacaggcaggctgtgacacacactcaggtgagaaatggccaaggctaacaactccatgtggtacaaattg
tcactcccccctgagccggactatcttaaaagagctggtatgtgacaattcggcccataccctgtctgcct
gcctttctacctacctgcctacctacctacataccttctcacatcaacaccacatcatgccgaggagaag
aaagaagatgaggcttcgacatgccggccagttcagggcgggaaacaaaccgtggaacaagggtcttacg
atcacgaaggtaacacccatttcttcataccataggccaacacaagaccaggacagtctgtatgtcaacc
gagataggaagggaaacatcatacagaatgacattgaaactctgaatgacgtgggaacaacaatggtgct
ccgtcatacaaaactgcaaggaagccggattgcgagattcattggggagaagacagacagcgatgaagtg
gagggctaccgcatgtggcatgcgaagacagcggtccatgcctgtgtgagtgcacagagacaccacgaca
agatccaaacagactgcaagggactggtcagagtgtcagcccggaaggagcagaagaaaggcctgaccac
ttcggagacactggtgtgcaacgagtgcaactatgaatcgcctcacagtaagttttatggggtggtgccg
tcagacgaaccaggaaaacgaggacccaaggtcgcagaacctaacatggcagcacaagtcgccatgttta

acagtcccataggaccgcaagtgctcagagaagttgcagctgcactggatatatcagtgccaagcacggg
aggacttcagaacttagcaaataaatacagtgacaagatggtttccttaaacgagaaggacatggaagct
ttgcgagagacggtgaaggaagtccatgcagtgaaaggaaacgaaccggacagcggcatcccaatagaag
ccgactctcgctaccaaagcccgctccgttctggaaggggaagaaaccaggtcaaccgtctggcacctg
tgtcaccaatgtagctgaaaatgtgacaaaaaggaagtgggtcatctgcactcatgtcaagaacaagaac
tgtcaacaatgtagcagaggagaggggaagaaggaagtcccatcccacaagtgttcagcgaacatcccac
agtcagccgttataggggatgaaagacaggctgccatagagattgccaagaaactgatgtctgggcctag
caaaacaattgttgctgagttaactgaagatggtgatagcagcttctcctctggaatgaaagaagtcatg
gaggaagcaggactgaatgatctcaaagtcttcaaagacattgtgcacctttcaaaagctatcaggcgca
aggtgagcggaggcaagtggtccaagcagatgtttccaggaaaaacccaggaagagagaaagtggatcag
ggaccgcttcggcaatgacttggccataaggctaaatactgaacacatcctgggactaaagaagttccgt
aaccagagggtcatggctggaaaaatgaaacaggttatgcaagcaattccatattgctatagtggagatc
acagcagatgtaagtccggttccctcgtgtgcaggcacccatacaaatggaagtttcaagagttacaaga
gaaggcgaagggaaagctggacccgaccgacgctgacctgaaggagttaggaaaaatcatggagcacagg
ctaggaaaggaggctttgaaggtgacaagacgtgggcgaaccaccaacaaagtagagtctgttaatagac
agattagcaagacatgtccaaaaaatgtgaacagatctcgtacctttccgggcagagtgcactctggact
acactcctccaacaatggaaccgggatgtcgatagcccgcaaacgtgccgcagccaatataccgttttca
ccaaactccgcggtcgttcctgcattggaagggatggaaaagacacagaactatcaccgtgcgtacaaga
tggaggtagcaaaccagaaaagacaaaaagccaagacattgagaaggtttgcttcatatgacaaaagaaa
agagcagggaacttacaaaagcaagagagcagtgacaggtacaggtagggcaccaaggaagaagaagaag
gtagacaacagagaacattcatacacccgtcccgcagagcaggagtcaccaagtggatcagatgcagact
aaggactgatagaatgtagcttacctaagcacctacagaatacagtctggccaacctgtatgtgtccgac
atactccgcggcgtcggcaggactttctcccgttgttgacagactgaggctcgtaagataggtgtagata
gttcacacagagaggttgatgacagagatgagagacatgtatagctttgtcaaggtgcagctggccagaa
taaaccatgtaagacactctatgtacaggccacttcttagttgtaaaggcaggcgtatcagggaaccaac
agacattaatccctccgtagactgacaaagctgatttcaaacctcctttccaaaaaatacatgttgatcc
aggctctctacgtatgttagcctcaatctttcttctgtattccaagcgccaattatctgtttgactcatg
ggggagggggcgtgttacaaagtcagaaaaacagccaaaatgttgtataatttgaataaactggtcttc
aactaagccaccatgtgtccttgtactgtccttatcttgtctaagggtatcaagtatcaaaaagttgtga
aaaaactcacctcattggtgcaacccgtcggcatgca
>KolobokP-2N1_BF-I
tgtcacccccctgaacttgagaattgacaaatttcaatcatctcgcatgatgcgggtaagagttgtcaaa
cattatcccaagtttcaaagctttctgaccaagaatgctcccaggggagtagatctcatggcaggtgtca
gaggactttcctcaaggctttggcagtggtctgaggcatttgataggcgccatagcattcaccaaaaagt
gacacaaactgaagaaaaagttaaaagctgatctttctagcataaggagagtagtctctaaccatttaca
cacctaaagctgtattgaaaagcgctcaggttacactttgaagcattttatgagagtgcataatgcattg
tcaataaaaggtacagaaagtaagaaatcagcccccaaaacactaaaatggcagaatttcttaagaaatt
tcagtcagttgagtcaggtaaatcctttctattgcccacataaccaatttggggggaacacattagccccc
aaggatatataagcccccaagggcacacctacaccccccccccccattctgtgcactcccagtatctgct
ccaaagtgtggttgaacagcatttagatacaaattttggtgtctaaattgatggagagtagtctccttgt
gacagggtgagcagattttaccattttcccctgtttgtgtcactttttgctaagcaatatgaagtctaat
caaagccacaggccttaccacaggcctcctgacacctgtgctccctggggaccaatcctaggggtagggt
ctccgtgaagtctttgaaacttgtgacgatgcttgacaacccttcccgcatcacttggagtgtcttgaca
cttgccaattttgaactagaataccctaccgaaagcatgca
>KolobokP-3_BF-I
ttaagcaaacttgaactgtcatttcttttaattcatacaagagaacaactcttgctgaacatgtgtacca
aatatggttttgttccaatggactttcgctgagatattgcgaactttgtactttgttatctcgccgtctg
ccacgtgggtactgtccttggtactgtgcgtgccatgtacaggactgtcttgaagctgtgtgaataaaat
gtttgcgatacaaacctattctgcgctttattgaaatcattattgattggaagctttagttgagctaatt
tcaaggctttctttgttcttctttgttggaagaaggctagcaagggattgtttgtgtgtgttgagagctt
acgttaggggccaagaagagagtagcagatagatggagacagacaagtactaaggactttctccagactg
tggacagagaaaaagtagcagaaactgtagatcttgtaaccattcagtgcccttacgagtatagcttta
tcaaagtgtcactttagcgtgatttaggaatatcgcggggttacgtaatgtcggtctcccatgcaggtgc
agaccttatcattgacggtggtgggtgaaatgtgcctttatctttaatattaatccacttattattgttc
cgtgtcgacgattaaaagagttttacacgttcagactagatcatttgcatgtgcgccagttggaaagggg
aaggggggtgtaaggtagttcgcacctccgctctaaaatgcggtcgcgcttagaagaaacaataatgttt
attgcaagtgagaagaaaagtgccgacttgggcattattcctacacactgtctagtctgcaacaaccacg
gtcgcttcctgacaaagttcgtttaccatgggtcgtccagccggtagccgagctttgaaaaatcggatgg
ttcgcaacagcaagtttcaagctgggaacacccccctggaacaaagcttcactagcggaaagtgtagagaa
tacagggacggaagagccctctgcctctaccgtcgcgtcatgtagcagctcgagcggcgtggagcaggag
tcacagtcggagccgacacacactgcgtccgccatcgatgtaaaaaagaggctagatgaagacacgtaca
agatgctctgcaggcccagagatcgtgtcatcatccctagactccgcgttaccaagacgaagtgctacac
agagccgaagaggtcttacaagaagaaccaagtaagtggaagcagaatcatcgatttgcaaaagatggaa
gatttgatgaatctgttcaatgccgggcaccgaaaagcgaacggcaaaaggatctgcaaaggagactgga
agttttccagaaaactagaaaagaagatagggttcgggtgtatggaggcgcttacctgcaagacctgcgg
ctacaataccacacccgcagagctcttccgacacccgtcgcccgggcgcccgtggccccctccccgtgaa
aagcaacatccaggccgttactcccggtgccaaagacaggtacggtataagtgccataaagcctttaatg
gcttcgcttggcattcccgacccgtgccgcacaacactgcagagaaagatgaacgtggtgggtgaagcct
acgccgaattgaataagagacagatgaagaagagctgtgaaaccttggcggaggtgacgcgccatcgtgt
agaagcgggactgcaagaaagcgcctcagtggcagttgagatggatgttgggtataacaatccaccaaag
ggaaagtccagaggacaaccagggacacaagcggaatgccccatgtttgatggcgtgacgcagaagaaga
tgctattggccttaagtacagcaaaccagcattgtgtggattgcgataagctggaggagcaaggcctgcc
attcggacccggctctcatgaagggtgtacccggacttttgagaaacacattaagatgggtagcagtgag
agaaagatggcgaaggaaaacacaaagacagccttgaagaacggtataatcccgggaattgtcgaaactg
acaatgactctaagatttccggtggcaccaacgaggccctgcatgaagccggcttgcctcctgcagaaaa
acaagactgtatgcaacatatgagcagaaatcaccgaagtaaagcgtacaacctgtgtctggaagtgttt
gaaggaggaaacaacaccaaggtcacgaaacaggaggcaagtcggcgggtaggactatatattgtcgaca

gatgcgtgagggcactgtacaccgccagagcacaccatcctgatagtgacgaggactttttccgagaggt
ggaacgcgggcgcccgacaatagttagttgtgtatccggggaacattctgaatgcacttctgacttcggt
tgtccggagtacacccgagagggtcgccgccctgccccagacattccaggcggagggaacttggctctgt
ccgacaacgaccgacgcaccctgcaaaagttggtagactatagacttgcagaagaatcagtgaaaaggca
gcggacattcatgaataccaacaagagtgaagccttccacaatcgtgctttgaaggccgtccctaaggcc
gtaacgtggaagcgaaattacgagggtcgtgttcatgcagccggactagcggactccgtcggatcgggtt
ccgccatactgcgagccaacgaattcctgggtgcggcgcatccccaggggagttccgggactgctgcact
tgaaggcatcgacaaagaagaggaataccataagaaaaggaaggggacttttaaatataagatgaggcgc
tacattctgcgcaaaaggaaggtagaacagaaagataggaaaggggttggatatgagacggactctgtac
acgctatgtctaggggagaccattgttacgcgaagtaggcggactataaatcgtcttatgacaggaaaaa
ctgtctttagtgcaagacgcatccctgtcctgcttagggaaacgtggaaccctttaggtttgggggggg
ggggggttccgagctggggtgtgtgggctgaggtgtggggaggggggcgatgctgagaagtcaccgtaag
acgcagtttctgtattccccgtgtttctggcaatgtccacctataagacacgtctttcgctcattgttga
catcatgcggttctaaggacagatgttcttctgctacacatttcttttgatgacacagatgacttatatc
tcttccatcaataaatccgttgtacagaaaatatgcaaccctcggtagtctttcatccctacatttaggt
ccaatctgcacacttattctgccgtaaccatctccatcggtcctcccattccacaaaacacatcctttgt
catctgtagacacttcggcacggattctttgtagcacagatgccttgtacgcgtcgctccacgcgctgta
tcccgccattgctaatatataaaacagatgtgctgtggccccgtacaaggcatatctgggggccggcaat
tggtctcattatctaaacaaatttaatattccgggtgactttttcttctacaaggagcttgccagaccat
aatctaacgttctaagcggtggggggttggggggttgtctttttttagggcggaaaagaggggtgggggtcg
agcgggcttggggccacactaagtcctatacttaagcatagggagcggtcttggcggccgttgccatggt
aacggcgggtgtggcggtaaaaaaaatgttacatttcaagtcagccgacgtcattccctaacatatcagt
caaattcaatgccgtttaccgtccctagggaattaccactgtggttaaa
>KolobokP-3N1_BF-I
ttaagcaaacttgaccttggattttttttcgctcatacgataaaactagtcaagatacaaaaccctacca
aattttagaacagaaatccaaatggtkcctgagatatggccaactttgcacttcgacgcctagcacaggt
gtcggccccgtacaagggctatctgagggaccgctaattggtctcattatctcaacaaatgtaatattct
ggctgactttttcttctacaatgtgcttgccagaccttattctaacgttctgagtgattggggttggggt
ttagtcttttttttcggcgaaaaaaaggggggggggggtagagcgggtattggctcacactaagtcctata
cttaagcatagggcgcggtcwtggcggccgttgccatggtaacggcgggtctgacggtaacaaaaatgtt
acagttcaagtcagccgacgtcagtacctaacatatcagtcaaattcaacgctgttgcccgtccctgggg
aattcccactgtggttaaa
>KolobokP-N1_BF-I
ttgaacgaacttgaccttggattttttttcgttcagacaacaaaactagtaaagatgtaaacttctgcca
attaactttccccactttcaagcggttgctgagatttcggcaactttgtactttggcacctgccactagg
taggccccgtacagggcttatagaacgggccatttagtggtctcattatctgatcaaattcactattctg
gctgctcttttcattaaaacggagacaatctgtgtctgttgttacgttaggagtgatttagggttagatt
caaggttagttgccgcagaaaaaggggggggggggtgcgggtttcgggccaaaggcgctttcacactaag
tcccatagttaagcatagggaaaggcccaaagtggccgttgccatggcaacggccgtctgacggtagaac
aaacaggcttatttcaagggagtctacgccaggacctgacataccaagcaactttgcccaaatataacgc
ttttgtcagtttttggccaatacccgttgcggttgaa
>KolobokP-N2_BF-I
ttaatatatgcaaattatgattaatattgtcmatgactttaaaaagttatactctataatcatgccaagt
ttgacttgtctgcgacttttctttctcgagtaacaggtggttgaaagttgcggtgtcggaaggctgtttt
tgcgcctccaggatggtataccattcaatgcgcataatagcaattacgtcacaatgagcttgtcctatgg
aagaggttttaggctatcaatacgtgctaaataaagagttatggccatgaaaaggaatgacagaggggtg
ctcttgtgatggagcacactttcctcctatttctgactggaacaaacggtgtaaacctcttagaacatat
ttgagttcgtgacgtcacaatacctgccgctggcctatgttctgaagtgacaacgatgcagttttaagtt
ccgcgctttaacggaaagcacttgaaatgttagtttttggtcatatcgggtagagaatagacctagcttt
cccgtccaagcgtttccgttggcaagactccttttatttcgtagaaaaatgaagttaaatgttgtacctt
tggtaaagatggaggtctgtgctgcacttcttgcggctatgaaggtataaacgggcatctgctaagataa
ggctgtacagtttggtgtcggtctcggtgagataaagaagccttgggatgtgtcttcggattagtgcaac
ttatttgtgttaaggccgagaactagcgccacaccgaacttgacacttgattcggaaggaaagtgcgaaa
gtgacgtcatcggggcctttttttcattggaaaatgcctgcttctcaatgtaggagcaattgtgaaacgtt
caaatgcgatttcaaagtgcccaagaggtactgtagtttaaacaacaatgtttcggtaggttttatgcaa
tctatgcttagcacacacacttcgaaggctatgcgaccgtgtttgaggggggggggggcgtgggaggggg
gcaaatccacccattatatgacagtgtagaatcatatcaaaacctgcgaaatccggctttttttcagtca
gatacgatcggggctatacactttcatgggaaaaatgcaatttgttggctattatcactaatactttttt
caggatgggtgcttttaa
>KolobokP-N3_BF-I
ttcacagagcccaagttcagatttttacaaaaaaagttttatcatacaatggagacaggggttgtcacat
attatccaaagtatgaaagaatttcaatgatttatactcccctgggactcacttttataacaggtatcat
ggctgccatcagagtctgaagggggggggggggcagggcatatatatcaagagctgttttcaagaaacct
ggcatatgtaggccaagaaatgccaaaagctagtcactctgacacacagagactgcctctacagtgacag
agacctgaaatttggtcatgaagtgttgtttcagaggctttgtagggactttcagaaggtgtgtgctggg
catttaataaaagtaggtcaaagccaatttcactcaatgcacacaccactttcttaatcttttgtgctga
aaatggcactgacagactctggtatgctgtcatggtacctgccacatgctaacatcatgctaacatcccc
aaaaagttagcaaaacacaagataatggggcatagaaacacacctcctgtatccatgagctggaccgctt
tcgcggctcctcccctgggacgttgccaagtaacgtggttcaagtccaaccgaactcttttggactattc
ataatttactttcgcaattgccaatagaatacaggtcaaatggaagagtgatagactctagagtccatcc
tccctctcaagtcattttacaccaatggcaatttccattataagcagtgccctttagcacaccagtgaac
taaaaacagctaaaatataggaaattctcaacaaatttagcatggcatgtcccctgggaccatatttcac
acatatggaggtgtattcagccccaaaatagctaaaaaggggggagggcagggtgatacctggaacacttc
a
>KolobokP-N4_BF-I
ttaattaacacaaattataattaatatagagtatggctttagaaagtcatcttctatcatcatgccaagt
ttgaattgtctgtgacatttccttctcgaatagcaggtgtttgaaagttaccatctccgaaggctaattt

tgccacaccaggcattggtaccattcactgtacataatgcttctgacgtcacaatggttgtatactcttg
aagaggttttagctcgttaattcaggttaaacaaacacatattgccatagaaaaggtaggcataggccag
ttcttacgatgaagtagatttcctatccattttaggttggaatgagcgatataaaccacatggaacgtag
tttatataatgacgtcacaaagcccgccgcaagaagatgtatcaaagtgacaacgataccgttttaagtt
cctctcttcaacggaaagcatatatgatttcgtttttttggtcatatctggtaggtaagaaacctagcatt
cctgtccaagcgtttccgctgccgaaatgcatttcattttgcagaaaaacgcagccaaatgttgaatttg
tggctaggacggagtcccatgccgcacttctggcaactagggggggattaaatgggcatttgctgagagaa
gactgtacagttacgtgtcggtcacgatggattacacaagccttgggatatacctccaaattagagcgac
ttatttgtctgaacattgaataccctcgcgagcccggacctaaaactcgattttgctggaatgaaggaaa
gtgacgttatttgaagactttttcattaaaaaaagcattcccttcaatgcaggagtcgccattaatcaga
taaaagctatttacaagtggcccagagatgtagtggtactaaaaagatgttttggtaaacataacgccat
tgatgtctaagcacacacactttgaaggctatatggctatggtcaggggggggggcgtggggagggggc
aaatcatgcattttatcaacattatataatcatttcaaaagttgcgaattccgatttttttctatatcat
tgggtcaagggtacgcactttactggggaaaatgcaatttgttgactttctttgcttttagtttttgtag
gaggggccatattaa
>KolobokP-N5_BF-I
ttgtagggggcccgaacttgagaattgacaagtttcattcatttcgcatgatgcgggtaagagttttcaaa
cattatcccaagtttcaaagctttctgaccaagaatgctcccaggggaatagatctcatggcaggtgtca
gaggactttcctcagggctttggcagtggtttgaggcatttgataggctccatagcattgaccaaaaagt
gacacaaactgaagagaaagttaaaagctgatccttctagcataaggaaagtattctcccaccatttaca
cacctaaagttgtattgaaaagctgccaggttagactttgaagcattttatgagagtgcatattacatta
tcaataaaaggtacagaaagtaagaaatcaggcccaaaaccactaaaatggcagaatttctcaataaatt
tcagtcagttgagtcaggtaaagcctttcttttgcccatataaccaatttttgggaacatattggccccc
aaggatatataagccccaaaggacacacctaccccccccccctcattctatgcactctcatcatctgctc
caaagtgtggtctaacagcattaagatacaaattttggtgtctaaactgatggagagtagtccccttatg
acagggtgagcagattttaccattttcccctgtttgtgccactttttgctaaacagtatgaagtctaacc
aaagccacaaaccttaccacatccctcctgacacctgtgctccctggggaccaatccttggagtagggtc
tccgtgaagtctttgaaacttgtgacaatgcttgacaacccttcccgcatcacttgaagtgtcttaacac
ctgctaattttgaacttgaataccctgccgaacatggca
>KolobokP-N6_BF-I
ttgaaccaacttgaccttgggtttcttttcattcatacaataaaactagtcaagatataaaaccgtacca
agttttagaacagaaacccaaatggttcctgagatatggccaactttgcacttcggcgcctagcacaggt
gtcggccctgtacaaggactatctgaggggccgctaattggtctcattatctcaacaaatttaatattct
ggttgacttttttcttctacaagatgcttgccagaccacattctaccgttctgagtgatttggattggggg
ttagtctttttttagggcggaaaaggggggggggggggtcgagcgggctttagaccgcamtaagtcctata
cttaagcatagggwkcggtcttggcgsccgttgccatggtaacggcggttctgacggtaacaaaaacgtt
acagttcaagtcagccgaggtcaattcctaacatatcagtcgaattcaatgttattacccgtccctgggg
aattaacgctgtgattcaa
>KolobokP-1_BL-I
tgctaggccccaggctttgagaattaacaggtgacaacaccatcatacaatgatcaaacttattcttaac
aacctgtccaagcctggaacaatttgaacaaaatatgactctgtagtgctaatttccctggcgagtgtca
atggctgtattccagctgtattccaatactggcatggcagtgacagagaggaaatggcttacagaatcaa
catgatcgagggcaagaaattgagaaaaaagacacataaaatgtcaaccactggtcactctgataaattc
acaatgttttctctagtcagggaccgaaatctttcttttttggtctttggaaaggtgtatgaggcaaaa
atagctacatgtgatgaccttggcgaagttttagaatatcttaagaacatttctgtcagtatgccattgt
ctgagactgaaattatgccatgacacttgcttgaagctagtactaatacacaagtgacaaagtcaactaa
aatcttaacaaaaatctcctcctttctcgtgccgtgggaacaaggcaagccacacagtcacacacactca
gtctgacacaggcacactcaggtgagaaatgtttacaaggcagctccatatggtgaagttctcactcccc
ccaagctgagttagctttaaaagtgggggatttcaccagcatttcaccatttctcgatacgataaattgt
gaagacagctctgacaatcaacatctccaagtcaacttcatggcacggaagagaaagaagaggcttcaac
atggtggactgttcaggcctggtaacaaaccatggaacaaagggcttgccatctcgcaagcggagaccat
ctcttcatactgtcggccaactgaagatcaagacagcctctatgtcaatcgagacaggaggcgaaacatc
atacagaatgacatcgaaactctgaatgtgaggggtaaaagaatggtccttcgtccgacaaggcttgcag
gtcgcagcaagatctccaggtttctcggggagaaagaagaagacagggacgacgttgaagggtaccgcat
ctggcatgccaagatagctgtccacgcctgtgcgagtgcacagagagaacacgacaagcgccaaccagag
tgcaagggtctggtccgactatcagcccgaggagagaagaagaaaggcctggccactttggagactctgg
tgtgtgacacctgccagtacgcatcacctaaaagaaagttttatggtgaggttaagagagagggggcgagg
tcccaagatcgccgtgcctaacctggcagtgcaagttgcaacgttcaacagtccagtaggaccgacggtt
atcagagagatagcggctgccctagatcttacagtgccaagccgatctggactgcagaatatggccaaca
catacagcgacatgatggttcaggaaaacgagaaagacttacagagttggcgggaaacagtgcaggacat
gcacgtggagaagggcaacgctcctgggagtggcatcccggttcaaggggacacccgctaccagactccg
ctccgttcagctcggggaaagaaacccggacagccaagccctaattcagtcactagtgtggctgaaaatg
tgacgacgaggcaagttatcatcggtactcgtgttagaaacaagatttgtcaaccatgcatccaggctaa
gggttcgggcgggccccccccaccccacaaatgcccagcgaacatcccggaggaagccgtcataggtgat
gaaagacaagctgctgaggaccttgctcatgcattcctttctgggcccaacaagacgctagtgggcgagg
cattggaggatggggacagtggcttttcgggtggcatgaaaaaagtcatgatggaggaagcgggacagga
agttaaagtcttcaaagacattgtacaccttgcaaaaggtattagacacagggtgaccactgcctcctgg
tccaagcagatgtttccaggacatgaccaggatgagaagaacagagtaagagatcgcttcggcaacgact
tgagcaggcggctgaatgctgaacaccgtgcaggactgctgaagttcaggacgaagagagcaatggaaaa
gaaaatgaaacaagtcatgtcagcaataccctactgttatgaaggagaccaccgcaggtgccttgcacgt
tcactcatctgcagacatccaaagcattggaagtttaaggacttacctaagaaggcgaggggaaacatcc
aaccaagcagtgctgacctcaaggtgttaggaaaaataatggaacacagactaggaaagggagctctgtc
aaagacaagacgaggacgaacgacaaacaaagttgaatcttttaacaggcagcttagtaaatattgcccc
aaaaatgttgtcatatctcgtaccttagaaggcagagtggcttccgcggtgcactcctcgaacaacggca
cagggatgtcaatagcgatgaaacgtgccgcggcgaatattccgctgtcaccaaactcagcggtagttcc
tgcattagaaggaatggagagaagacaacagtatcaccgctcgtacaagatggaggtagcaaacaagaaa
agacaggaactcaagaaggcacaacggtttgctgactatgacacaagaaaagaacagggaacttataaaa

gtaagaaaccagcaggacagtccaaagctaaaggtaatggtagggcagcaaggaagaagaagacagtcgg
ggaacattcttatacccgtgctgcagagcaggaatcctccagtgagtacccatcagaccaggaatcagat
caggactgactgtgaaaaactgtagctaagtatatactcaccttgcctagcttattcaaaaatacagtcc
ggccggtccaagtgatgtgtacagtgacccaatctctggcacaccttcctcttattgttaaccgatgttg
gctccaaggtcaaatggacagggttcacgcacagacaacgatgacatagatgactcacacttaaatcacc
taagtctagtacaccatgatggatcctgtatgcgactctgtgtacatagaattttgtgggtgtaaactcg
tctctaggaatgtttggaaaccaagatacgttaataccaccatagggtggcgattttagactaccagtcc
aaataacacatgtagatgctggatcatgctgaatctttttctcgatcttcttcttgtattctaagaacca
atcttgtgcagaactcatgagcaaaatgtacggaaaacaagcaaaaaatgtccgacaatttgaataaatc
ttgcatcaactgactctcaactgtttccagatgtttccttgtctcctctaagggtatcagatgtcaagta
gttgcgaaaatactcacctcatatctgggtgggtgggggctgca
>KolobokP-2_BL-I
ttttacggctcatcccaggggaattgacagatgacaacccgttcaaacaatgcataaaatagttctacag
aacttctgtaagtttggaagaacttcaaagaagtatgactctgtagcactgattttcctgacaagtgtca
aagactgttgtttccttgtttacatggcagtgtttacattacatgcctcacactggcaacatgttttagg
caagaaaatgatggaaaaaagacaaaaacagttccaaacaatgaccactctgataaagttctttgctcta
gtccaccaagaacttaaattttctgttttaaaggatgagaaacaacattttggggcactttgggtggtgt
ataatgcaaaaatggctacaagatcatgtttttatggcatcttaagaagttttctgccaacatgccattg
caagacactgaaattagtccatgacacttgcttatactagttccaataaagacataaacaaacaaacagg
acaggacatctccctttccctgggaaccactgaagctacacagtcacacatgcacacgcacactcaggtg
agaaatggccctatctggcaagtccatatggtgacgttctcactcccccaagctgagaaggggggttaaa
agtagcagcatggcagctttgatcacactctgatctatacgagacatttcgaagacagctctagatatcc
gcatcaagaccacatcatggcagcgaggaagaagaggggggagaaagaagaatgggagaccgcatcgtgcc
ggccaattcaggaaaggccagaagccctggaataaaggccttgacctcatgcaggtaaaagccatttctt
cataccgtaggccaacagaagatcaggaaggtctctatgtcaacaaagacagggcgggaaacatcctcca
gaatgacaccgaaaccctaaatgaggtgggaaggactatggtacttcgtccaacaaagctagctggtagc
aggatcgcaaggtttctagggaagaaggtcgacagtgatgaagttgagggttatcgcatctggcatgcca
agacagctgtccgagcctgtgcaagcgcacagcgagaccacgataagcaccagccagactgtgagggtct
ggtcagactgtcattcaagggagagataaagaaaggcctggccacagtggagacacttgtgtgcgacacc
tgcaagtacgcatcgcccaaaagaaagttttatactgagacaaaaagaccaggaccaggtgccaagattg
cagtgcccaaccttgcagtgcaaattgccatgttcaacagtccgattggagtccgggtactcagagagat
ggcagctgctctagacctacccgtgccaagcaaatctggactccagaggatggcaaacagatacagcgac
ctgatggttaagaagaatgaggatgacatggagacttggcgagagacaatatatgacattcaccttcaga
agggaaaccctcctggcagcggcattccagcagagatagacactcgctatcaaaccccgcttcgttctgc
taggggaaagaaaccaggacagccaagcccaaacagtgtcaccagtttggctgaaaacgtgactccaaag
aaacttgctttggctgtgcatgtcaggaataagatctgtccaacacgcaacttaaacgaaggtcgcggga
agccggtcccagcacacaaatgccctgctaacatcccggaggaagcggttataggggatgagaaacaggc
cgccagagatgttgccaagaaaatgctgtctgggcggaggaagacacctgtgggtcagcttgtggaagat
ggagacagcagctcttccaaaggcttcaaagaagtcatgatggaggaaacaggacagaaggtgactgcct
ttaaggacattgtacaccttggcaaaggtgtaaggaacagagtgacaactaccaagtggtctaaggagat
gttcccaggacaagaccagctagagagaaacagggtcagggaccgctttgggaatgacctatgcaagaga
ttgaatactgaacacaaactggcgcgaaagcggttcccacagaagaagaagatggctgcaaaaatgaagc
aagtcatggaagccatcccctactgctatgcaggcgaccacgacagatgtgttgcaggttcgctggtatg
taggcatccacgtaaatggaagtttaaggacttacctgcaaaggcaagggaaaacatcaccccaagcagc
gctgacctcaaattgttacagaagataatggagcacaggttagggaagacagctttgaagaaaacacgtt
gtgggaggacaacgaacaaggtggaaagtttgaatagacagattggaaaggcttgccctaaaaatgtact
gaggtcacgtaccttcgcaggtagagtagcttctgccgtgcactcctcaaacaatggtacaggatgtca
atagctcagaaacgtgctgcggcgaatattccactgtcaccaaactctgcagtcgtttctggattggaag
gaatggacagagagcagaaatatcagcgggcatacaagatggaggtaccaaacaagataagacagcaggc
caagacaacgacaagatttgctgcatatgacataagaaaagagcagggaacttacaaaagcaaagtagtg
acaggaaagtctaaaggtaaaggtaaaggtagaggtaaaggtaaagcagtgaggaagaacacagaacatt
catacagccgggatgcagaagaagagtccccagacacttactcatcagatgaggagtaaagaaagaactg
tagtcttaaccttaccttctacgctgactcaaaaatacagtccggcaagcctctgtgcccttgacattca
cgcattcttcggcaaaccttccttcggttattcactgactgtggctccagggaaagatggcgaaagttta
cgcatttgctagtgtgacacagatgacttacaacttgtacacgtgggtctcctagttgtagttgtccaga
atagaccatgtaagcagctctatgtacatagaactggggagggtattcagggaaagggaaccaggagacg
ttgatgacaccgtaagactttttcttagcaccaatccaaagcaggcaagatgaactaggatgaggaagaa
ccctcgcctctatttcctcctgtactctgaacgccaatcttgttggtccttcatagggtagaaaacaga
gacaaacgtacactgtgtgaataaaataatcttcaactgacctctaattgtctccagatgtctccttgtg
ttgtacttgggtgtcaggtatccaaaagttagagagaactcaccttattggatgagccggaaagccttca
a
>KolobokP-3_BL-I
tattagaaaattcaacttgagaatttgcaaatttcaatagtatcatcaaatgcggggaagggttgtgaag
cactatccacaatatgaaagatgtctgttggttaatgaaccccctgggacccacttgtataacaggtgtca
tggcagctcttaaggcctgtgtacagcagtcttgaatagggcctagcttacagcagccaatatgttcagc
caggagatttggaaaaactaagcaaagaaatgtcagatattgtccattctaacacagaatggcatgcttt
actgtgacagggacctgaatatactcatcaaaagattctctggtgcattggggcacttttaaaaggtttg
ttctgtacagtctttaaaagtaggtcaaactcaagtctggaagattttacagactctacatgctactttc
tgaaaatatgctgtgacaaggcttccaagtatgatgccatggtacctgcttaatggtgatatcacccaaa
taaacaaggaaaacatcaggaaatgcactcccacacttgcatcatcacttattatttccttatttggctg
gccttgagtcagtggaaactaaagctagccacacacgtcatcaagggtggcagggccgggcatgtccctt
gagagctacctgtcacacatttccctgggtcaggtgctatataagagatgaggaaatgagttgtaggcca
cactttcttgaagacaattcagccgaagaacatccatccatcagaagacatcatgggaaagaggaagatg
cagcacgatgggcagttcaagcctggaaactatccatggaacaaaggggcactaagaaggcacgtgtaa
gacttcctacatacgtcagaccttcagaggaagactactctttgctggcggacagagacagggacggcaa
cgtcctccatgacaaaatggacatcatcagggagtctagaagacccatcctgctgcgtccacagaagtca
gactccagtgaaatgaaccagtatcttggggacgggagtgcagagggtgcagatgatgttgtcgggtaca

gaatctggcaggcgacagccgctgtccgagcatgtgccaaggcgcagcgggaccacgataagcgacgacc
cacctgcccagagcttgtcagggcctcggccagggaggagaagacgatgggactggcaacagctgaaaag
ctggtgtgcgatcgatgcggatatggctcatcctcccagaagttctatgacgagataatacgagatggac
caggagggagaaccccccgtcatcaacatggctgcgcaagtagccctggctgactgtccgatggctgtctc
tggattcagacagatgatggctaccatggacttacccgtaccgtctgagcctgcacttcagaacctggca
aataagtacagtgacataatggaagaagaaaacgaaaaggacatgaaacgctggattcaggtgatgaaaa
gggtgaatgcgctgaaggggtacaaaccagacagtccaattcgggggcaagcggacacaagataccatac
tccacttgggaacctcaggggaaggaagccgggacagccaagtgatagcagcaacgccacattcgtggaa
gaggtgacaacaaggaacaaagtcattgctaatcatctgaaaatcaagacctgtaggctgtgcacattct
accgaggccacggacttgagcctcctccccacaagtgctcagctaacactttgccgcaggccccgattgg
aaacgaagaggagtctggggaaaagatggcaagagacttgcttgagagcaacgtcagagtctcgggtctc
atcacagatggagacagtcaaactttcagagggatggccacggtaatgcgggaggaagccgtccgcccaa
tcagagtaaaacaggagaggtgcttggtacacatcagccgaaatgtgcgaactaaaatcagcagcactaa
atggtctcccaccatgtttccaggggcaaaagcagagagcagaagccgggtgaagcacagatttgctaac
gaccttgttcaacgcctgaatgctgagcatgaagcggccctgacacagcttggaatgcggacgagacacg
gagtggaaagagcgatggacaaggccatgcgggcaataccccactgctacgctggaaaccatgactggtg
tttggaatactccctcgtatgcagacacccatcacgatggaggtttcagaagataaatgctgaggcaaga
ggacaaatcaaccccaacccatctgactgtaaactcttacgtgtcgccatggagatgagactcggcaaga
aggcactagagttgactcggcattttgcgaatacgaacaaggccgaatctgttaacagacagataagtaa
aagtgcaccgaagaatattacccgcttccgcactcttgccggtaggatcgcctcagcccttcactcctcc
aacaatggcactggcctctccgtcgcgatgaagaggctcgctgcaggtatcccactctctccaaagtcca
aagctgtccgtgttctggaaaagatgagggagaggcaagattacaagagatcctacaagaaggaaccggc
aaacaagaaacgggaaaatgccaacataatgggaaaatttgtagtgtacgatgagggtcgagaaagaggc
acgtacaagagtgcagaggtggccaagccctccaagggacagcgtgagtcgccaaggaagcgagcacaac
aagacggcaccgaccacgggtacagccgggctggggacaaggaacctgaagattactacagtgactctga
agacgattcagacgaagatatggacttgtgttgagacttacctagctagttgaagatacaaggaggaaac
tgctcatggcggatgcaatacccacgggactggcattgtttacgactgttgttgatgtggggggttcta
gtgacaagtgctcgatccgaacgcacaatgggacactacacaaatgcgagacatgtagtccatcgatttc
tatgcgaccagtaaaagccatgtatgcaattctatgagcatgtaatctcgtacgattctcaggattcggg
aaggcccgatgtggcatcctcgcaacgttcaccgagccatattggactctagatgttctagtctttcccc
caatccacagtaaacaagatctggtggggtgggggagtactttttttagagattttggacctgtactctcg
gcaccaattgtcacaaatttgacaatgtctgtaatactgttccatgacagaaatgcctgaaaaggccaaa
aatgtaccgaatttctgaataaatgacagttgaaatatatcttcacgtgttgcagatgtcttcttgtgtc
aaaagtcaattagacaggggcgaaaatacaccgaattagcccgaacacagtgggaggtgggcggccagat
tttcctata
>KolobokP-4_BL-I
ttacaggtgatccagcaacgagatagacagatatcactaaaatcatgttgtacaggaaagggttcttagc
aatatccccaaaaattatagtcctcctatcatcaacgaagatatgacaggtagttatctaacaggtgtca
gctatgccctgagtgcttgcctgtactggcagtcagcacatgcctgttcagagaagctgggggagttaca
aggctacaaccggtcattagggacaagtcaattatcagatcatgatttttttgtcaacagtgacattcaa
gacacagacttgatcacttggtgtctgattgacacctattcttgacactttgaaaccaattttacactat
gacagcaaggggtagctggtactgggatttgggtggtgtcagtgtgaacagagtgacaatcttaccaaga
tttactcaagaaagacatcatattttggtccatcatacagttcacacctgtaagatgactagcatattcc
aggaaacggggggtgggggggggggtggtacaagccaatttctgggtccttcaagtctcagaaacttcc
cccagtcagcactaaacttcttggaattgggcaggcaggggtaaggggtgacgtcaggctctctcccctg
tgaccattcatcatcagcccacctcacttgtcaagaaagctttaaaatgaagccacaacaagtaaaatct
cagaaacacgccagcccagccatgggacgcaagaaatccagcaaggagaggccgaccgtcttcaagaaag
gttacacaccccacaacaagggcatgaagattcgacatgtcacgccagcaacacctgtctaccgcagggt
ttcggaagacaacttcgagctactacaggacacagaccgaagaggcaacctcttgatacgcccgaagaaa
gtagctcttgagaagaggagggtgatgcttctcaggccaaagaaggagtcatgcagcaagatcgcgagag
ctgtggcaagaggatacacccgggatgatgcaagagatgacaacttagacgacttggttggctatcgtat
ctggctggcacgagatgcagtaaaagcctgcaccaaggcacagagagaccatgatcaagaacatccaacc
tgcaaagcactggtccgtctatcagccaagttagagaggaaagtggggcttgccacatcagaaaccttcg
tatgcgacaagtgcaagtacaagtccaaaggaaaaaagttctatgacgagattgacaaggatgcaggaca
aggatgcaggcgaggaagaaaggtagctgtgcccaacatggctgttcaggtggcattggccaacagctcc
attggagtggctgccttcagacagtttgccgcagccatggacctacccgtgcctgtttccagccagatga
gaaaaaatgcacgcaagtacagcgacttgatggtgcgggagaacgaaagggacatgaaagcatggagtga
aaaggtcaaagagatgaactccatacttggcaacgcacctgacagccccataaagggtgaggcggactcc
agatacaagagtgccttgtatgcaggacttggaaaaaaacctggccagccatcaccccacgctcacagta
cgttctgtgagggtgtcactgcacgccgactaatcctggcccactttttagcgcagaagcagtgctcaac
ttgtcagcggcagcagctccgacgcaaatgtgtgagacctgggagagtgcagaagaaggcaaagaaacac
aaatgtactgcaaacttgaaacagcatgatgttatcgggaatgaggaacttgctggtagagagattggga
agaaactcctggccaacaaggtcctggtaagccacattctaacagatggagacagtcactttgccaaggg
cctgtctgaagtcatgatggaagcaagaggcaagcgcacaaaaccgctgagggactttgtgcacatgggc
aggagcgtggccaaagctgtccgccacggaacttggagcaaagaaatgttccctggcaacacagccgcaa
gaagaaacaaggtaaagtcccgatttgcagaggagctgagggtccgactgaatgcagaacacgacagtgc
cgtgctgaaatttggcaagaggagacaaaagatggaagctgcgatggaagacgtaatggacgctatacct
tcatgctacatgggcactcacgatttctgctcgacgcattccatggtctgcaatgccaggaggaggtgga
agacagacagacggtacctaccggtcaagtgcttacacccaaacagctcggacatcaaagaactcagacg
gatcatggagaagagactggggaagaaggccttagagtctacaagatactgcatggacagcaacaaggct
gaatctgtcaaccgccagtcaagtaagaatgtgcccaaaagtgggaactggatagagaccttacctggga
ggttttccacgactgtacacagctcgaacaatggtccggccttgtcaatcgtgaagcgtagagcggcagc
aggtattccgttatctccgatgtcgccgacagttaaagccctagaaagcatggaaagagataggctttac
cgcaggaagtataggaatgaaccggagtgcaaacagagggccttggcaaacagaattcatgggttcaaaa
catacgacgaactgcaggagatgggaacctacaagtcaggtaacgtgcctagtgcggagaagaagaggaa
ggtggtgaccaaggtgccgagaggaaaggaggaccatacttacaacagaatggaggggggaaggaccaact
ttagactcagactctccaggctggactgatactagtggtgatgaaacagactgacttgaacttaccttta

accttcaaaaatacaatccgggtagccaggatgtcccgtgcactcgcgctgagaatgacatctctgtctg
ttattgttgataatatgcggttctgctgacaagtgttcaggatttacacagagacgaacatgacagcgat
gcgatatgtctttgccagctatgtcaaactgacctgtccaagccatgtaggccactctatgaacattgat
tggtcttctatttcttttctgtaaaggtaattcttcacctggcatccagggcacatagatgcgaccatag
tttgttcttgcacgtacttgtccagtccaaatccagcaagatttagtcaggtgacgccttgtctttttag
taatttttctcttgaactctatctgccaagtgacattgtcaaattgctgtgccattttgtgtgaatagca
gttaaaatggtgaataaagttgaaaattctgaatggtaaccgtctctgtcatgtgttctggtgtcaatta
ctgatatagtttgcataaattagcagtttagagtgattagagggtaccaagtatatggcccatttccctc
acccctccccagtaca
>KolobokP-N1_BL-I
tattagagaaatcaatttcagaattgacagctgtcaatcatatcacacaatgcggggtagggttgtgagt
tagaatcctgagtatcaaagacctcagaccaaaaacagtcccctgggagttgatgacctaacaggtgtca
gggctgctctcctaagttaggctaggaatggccaagttacccattataggtggaaaattgtcagaaacag
gtaaataaagttagaatttggtcaccccagtacaggggggactttttttccctatagtaggcacctgaatt
tccttttgaggtacaacaaaagtcaatacaggggccctgacaggcctaaaagcaagacaagtgtgtagaa
accatcacaaaaagcagctataaacaaggcatagagcatagaaaaagagtttgacaaggtcaaaacacaa
tattcacctggtatgagaccccctcaccattagggacccccccctttcaattcacctttagaatactc
aaatcccatcctttgtacacatagcctagctgcagccaggcactgattggaggcggggttaaaaactcag
aaaaatggctaaaaataacaaaaatgggataatttattaataaaaatgagccaaatgaggtctgaccatt
gcttaggtaacaggtctaaggctaaaaccccatgaaatggaccaaaatggtctgcctctatcctatgtga
atttcctata
>KolobokP-N2_BL-I
ttctaggaaaatcaggttcaaagctgacagttgtcaatcatctcacacagtgcggtgtagggtagtaaat
taaaatcctgagtattaatgttctctgacaagaaccagtcccctgggacccgatcactcaacaggtgtca
gctatatagggttatgatggcaaactcataggtactaggtggaaaaattgacaggagaagaaaattaagt
caaaatatgatcacctgagtacagaagggctttgccaactatagtcctaccctgtatttcccctttgcaa
cctgtcaaagcagaaaaaagtccatctaaggggctctggccaccacacgggacaggtgtatgcaaggccc
agggcatagagatgaccttgacaatggcaaaacacaatgtttaccaggtcccggaccactcccgattacc
cgacacctactcccatacacccccccccccagcacctacccccctggcaggtagacacctacagttcacc
ccctagtatacaggatccattcctgaggagactgagtttggctgcagggttaaaaagtaaggaaaaaggc
ccaaaatgctaaaaatgagacaattcatgaatgaatatgagtcaacataagcctatcccttgcttagata
gcagattagaactgaaaaccccatagaaatagcagaaattgcttgcctacacttctacagattcccttca
>KolobokP-1_PaMi-I
tttaatagcaggcaactctcaaattttaacacaaaaccacattcaggagaagtagaatatcttcaaagtc
attattttgaaaaaaaaaatgaaaaatattgcccctcggagaaaccacaggggctaatgtgtagggtgtg
gctatggtgttttctactgttttgaataggaaaatacagcttgtgtgcatgcaatatgcacatggaagtt
gtttttctttccttctcacttcaaattttgccacatattcctttcttttacatttgcaatgtgtgtgaa
ctattttcttggttcatctggggtcactttgtggtggccataaccactttaggggcggatcaagggtgaa
aagaaggggggggggataaattaatttcagcacagctttaacattttgtatatgcaaatgtacagggtgc
cctgtattttaatggtggcatgtaacataagcaacattacaacagtagtattttggtcatttcttaaatt
attgcaagcaatgaacacccttggaagatcaaaaactgtacacaatgggccttgatgttgtgcactaatc
cctggctgtggctgccttttattctctgaaagtacatgctgaagatttattatatgaccctatttatgac
tccctggccctgtccccatcacagcccagcctctttagtactagctgggctaattactgtctccaaagat
tccattgtaaaacacctatcttccatccccccacccacccccccccccccccccccataaaataacaacag
aaataaactctctgtacagactggcttctgaattattaaccaaacttgtgctgtatagtcatcactctgg
cattgagagttagtgtgaccacatgcattctgtcacatgctcacacaggggaaaaacaatctcaattttg
caaaaccagctatttctgcagcagtttcagtatgggtcgattgggacgagcagtgtacaaaaggaagaaa
agctggttccagaatggccataaactattccctgcaaagtgtaaatccccaatcagtcattttcagatga
actcaccaacagtgtacacaaggccaacacaacagcagatgcaacttgctgttgaaagagatgagcgggg
gttcatctgtaccacgcccaatgcagtgccgacaaggaaagtcatgctgcttcgaccaaacgctgctgaa
actactccccttgacaaactccaactcaagaaggaagcagtaccacagcccctctgtagaagagaactcc
agcaccagagcccagctgatcagtcagatggagggaagcgagcagcctaccggttcgtcgacatcaattc
tgcagtcgacttgggagcgcaggctatcctagatcaccaacgcacacatcctgattgcagcggccggccc
acattgatgccagatgctgaggagcgacgaggtttggctgtgtccgaggtactgtcctgccaaaagtgtg
ggttctgcactgacaagacaaaactgttcaccgaaattccacagacggggagaggacgacggactgccac
tcccaacatggcgttccaggttggtctttacaacaccggcattgcagctgctggggctaggcggctgttg
tctgcaatggacacaactgttccctcgataagtagcctgcaaagtacatcaaacaaatgtggtgaaatta
tcaccacagaaaatgaaacggacatgtgcaacaagagaaaaatcgtgaaagacattcatgaactgcaggg
ttacgagagacacagcgcaattgcagtggaaactgacagacaatataataatccccttcgaaactgtcgc
aagaaaacacccttcgtcgcagccacccagacaagagatgttgtcgttgaaaatgtgacaaccagcaagt
atgttgtcatgtaccaccaggagaacaaactttgcaaaaaatgtgacattgacggagcgagggatcacaa
gcacgcagggaaatgcacggccactcgtccaccgaacttcaatatgggagatgagcgcaacgggggcatt
cagtgcgcccaaaaacttttaaactgtaaagaacctttgtttgtaaatagggtgacaacagatgccgacg
gttgtctggcacggggtatgagatcatatatgcaaagtaaggtggcaggtatggaaactgaacattcgtt
agatccgcctcatttaaaccgatcactctgtgcggccataagtcgtgcaaaattcagtaaagagatgttt
ccaggcaaaaataaaagagcaaagaaaaacattcaagatcgatttgccgatgacgtcagccacagggcac
aggccgaggctgctgctatactgaaaaaaagcggtgggaactcttcccaaatgacgaccatggctagaaa
tgcagcaagtgccattgttcaatgttacatggggaatcacggcctttgcaaaaagtggtcccaaatttgt
atgggtggtaaatatcggtttccatacctgccagtcgatgcccgtggcaaactgaaattagcaacttgtg
atgaacagactctcatcaaactcctaggcaaacgacttggccaagaagcccttgaaaaaactaaatatgg
caccagtactcaaaaagcagaatcaatgaaccatgcctttgcagtaaccaaccctaaggccacactaacc
ttctcaagaaatggttcttgtcgagaccatagcgcagtgcacttggtgaataacagacacgccaattcaa
tagtcaaaaagtgcgcagctgctggttgcccagtaactgcaggctccccagctgcaaaagcccttgctga
aatggataagaaacaaaaacagtatgttaaacgtgctaaaggtcaggagtacaaaacacgcagagcacaa
ttgcgttctcaaagattccagctgtattatgccacacggccactgtataaacgtggccaactggatccta
agccagacgatggtttgaaaaatgtctgccacatcatccaatctgaacacaattactgcaaacgtttta
aattatgtttttggaatgtaactgacaaatttattgttcattacgataccttaaagcaggcactcccggt

acccatcgtgcccttggcactgcttgagtcgtttgcagacttttctgctattatttacagccctaggttc
aagggacaaatgctcgaaacaaacgcacaatgaattctggcacaggtgacttacatcccactcccgtggc
aaatttacatttccggtgtgtgccatgtaagccgccctgtgcacagacttcagagtgcgtttgttcgagc
ctggcattttcattgaaatgatgccataggatttttttatgcatccccgccaaagccggcatcctttga
aggctgaaatctgctctttttttgcaatcgcccatttaattctggcgcaccacgactccatgtcggccatt
ttgtctcagtgtttttcaggagcccagaaatccattggctaaaagtttttttaccttggatgtgaatgtcc
acccttcaccatccatttattacaagtcaaaatttgatttgtacaagctcaaacaagtacaaacaatat
tattcacttatttttgtctgaataaagaaagaactgtgccttctctgtgttcgatttcaataataaccaa
ttaaaataccccgaaatggaaacaaaataagaacatgcacagactggggtgaatgactcattgtttacat
ttttttaacggttcacttacatgtaaatgctaacttccacagtgtgttttgaataaaaacctgaaaaatc
aaagatacagagtgtaaaatgtttgttttcatatttattttaactcaatttaagcttattttgtacttac
agcaataaaaagcacacgttgaaatcccccgtcataacaacatggccgccaccactcaggatctata
>KolobokP-2_PaMi-I
tttaaagaagcaaaaatttacattttatccccagaccgcatttaaggcaagaagaagtatccataaatca
tatttcattgaaaaattgaatgaaaaagtactctccggataattacaggcatgaatatgaaaactgtctt
taacagattatggcttgtctgccatatgtgcattgcagattggtgatgcccttttcccactgaagctgat
gaataacaacccctcttgcattggaaatatgtttcttggttcacaattagggttagaaattaccagttag
ggttaggatttgggttagggttaggtttagttagggttagggataggggttagggttaggggttaggggt
tagggttagggttagggggggagggaaggggagggggaatggaaaggggggggggtgatgcatatgccta
catatattcatttcatttgaaagaccagtttaaaacactgatatggttattggaaaatgtttattggcca
aaaaggacaagaaaagtaccccaaaaaatgaatatgggacatgattctccatgcatgcacttgttttcat
aaacagcaattaatcctgtttaatgattccactggttgcccctcaccaccattcaattcagaagtggcta
tggaagtttactgacttactgactgtcttcagttaattgccctctgtctgtgtcaatatcgcactacagt
ctgcactatagtctggctggggattaattacttcaaagaagcatccccatctaccatcatcaccacccct
ccccccccccccaataaaaatgaacaaggggcactaaagcatgtatccctactggtttactcattattgt
acacatcacttgtaagtgagcctgacaatttgtgtatgtgtaactacatgcactcctcacttttttcatcc
aagcagagggggaaattttgttttatcccatccatatacttctgtgtatgtaataacatggggcgatttg
ggcgtggtgtgtacaagaggaagaaggctggatttcagaagggcaacacttggtcacctcaaaaactaaa
tccacaaaaacgtcaattgcacacaggctcacctacagtctacaaaagaccaacgcatgagcaactggag
caggcggtcaccaaagatgcggagggattccactgtcaaactcccgtggatgagccaaccaggtccgtca
tgctgctccgacccaaagtccacgaaactaccccagtaggagagcttcagcatcgggaggttctgcaagg
acagagagaaacataccgattcgttagcattgatgcaaccgtcaagttaggcatgcaggctgcacttagt
catcagcaagcaaatcccgagtgcgaaagtcaacccgaacttgtgcatgatgccgaggtgcgcagaggtc
tgggtgtgtctgagatgttggcctgccagaagtgcggatttcacactgaccacacaaaactgtacagcga
aatacaacacactggaaaggggagaaagactgccactcccaatatggcattgcaagttggcctttacggc
accagcattgcaaccgcaggtgctcggcggctcctgtcagcgatggacacaaccgttcctgcaaaatctg
gtttgcaaaacctcgcagacaaatgcggaaaaatcatcactgctgaaaataaaagggacatgagtgataa
aagaagaattgtgaaagacattcacgaactacaaggtcatgacagggaaagccctgtggcagttgagatg
gacagacagtacaacaacccccctcagaaatgctcggaaaaaaacactgtttgtgccagcaacacaaacga
gagacgttgtttgtgaaaacgtgacaagtaagaagtacgtcgtgatgtttcaccacgaaaacaaactttg
caagaaatgtgacaatgccaactgcaatcaggaggagacgtgcacagctactcgtccagctaacttcaat
atgggcgatgaacaagaaggtggccgtcaatgtgcacaaaaactgataaactgtaacgagcctcttgtag
taaatagggttacaactgatgctgatgggtgtttggcaaaaggaatgaaatcatgtatgcaaagccaggc
aggtatggaaactgaacattcactagaccctcctcatttgaatcggtcactctgtagagccatgagtcgg
gcaaaatttagtgcaggcatgtttccagcaaaaacgaaaaaggaaaagaaaaaaattgcaaaatcgttttg
ctgacgatatcagccacagagcacaagccgaggctgctgcacttgtaaacaagttcagaagagactcctg
caagatggctgacatggctgaaaaagcagcaaaagccatcattcagtgctacatggggaaacatacccctt
tgtagaaagtggtcaatagtttgtggaggtggtagatataagtttccatacctgccagctgatgctcggg
gtaaattaaacatttcagcaactgatgaacaaactgttcttagactactcagcaaaagaattggcaatga
agctttggaaaaaaccaaatatggaacaagtacccagaaatctgaatcgatgaaccatgcatttgcggtt
acaaacccaaaaggaacacaaaccttctctagaaatggttctagtcgggaccacagtgccatccatatgg
tcaataatgggcatgccaattcaatcgtgaaaaagtgcaaagctgctagttgctcagttactccaggctc
tccagctgaaaaagccctcgctgaaatagaaaaggcacagaaatatcatgctgaaagaaaaaaggggggta
gcctacagaaaaagccgagcacagtgtagatctaagaggtttcattcctattattctacccaaaatatgt
ataagaagggccaaatgaatccgaagcacggtttcaaattccaaggagtgaatggaagtcacaatgttag
actcgaccataattattgcaagcagtttcctagcaagtaatttaatttaatttaactttgtggaaggcta
gtacagtagtaatgtaattttgtgccatacttacagtccactagttttttcacaggacacaacttttgta
tgttccatggccttgacattccttttgccttcggcaaactttacgctggttgttgatcagccttggctcc
agggaaaggtgctcgaagaggacacacaatgaattgtggcacaagtgacttacatcgtactcccggggca
aattgaaattctcggtatgtgcgatgtaagccactcgatgcacacttttcattgtgcgtctcttcgagcc
aggcattcggcatgaaatttgcccatattttgtttttctggaaccagtcttcgtggaccccgcccacact
aaacatccactcctgttcgttttgactcttctagaaattgcatattttatccttccacaccattgtttcg
tatcgttggcctccatttgacattaagaattgcttaccggtctattcattcatgccattggctgtaagt
ttccatccctaccctagtactgcaatgcctctcccagcctcgtatttactttaaatatgacagtaatgcc
gagcggtttttcagcaaagagcgaaaatactttaattaattaatattaatgaaattacagagacctgttt
tagccagaaaaggaaaatttaaccaacgaaaagaaactattcgaagcaccttaagactatggtgaatgga
gcagtgtttactcttgtgtttacataacaatccacacgaatgatatgttgcatgaattgtttttcaataa
aacacagccaaaaataagaacttcagaaaaatttgtcaagaaattgtctttaaaagtttttgggtgttta
tttcattttgtaatcaacaattttagacagcaattgccccttttcaatcccactttcctcacaacacgga
agccaccattttgaaactata
>KolobokP-2N1_PaMi-I
tatagagaagcgaggttttgaaaaatttccccaaaccacttgtacggcacgaggaaggatggatgactca
tatttcatcaaaaaattgcaagaaaaaaaatctctgactgcgatttctggcctgaaaacaagccctctgt
ttgcggctaccttactgtagggcgcccccaacgggcgttaattgagtttttgctccttccgaccccaaat
tccccccccccccccccttgttcgctcgaaattgctgtgatcggacgacggtccatcggccctattcaga
ttttgggcgcatttgttgcggaaccgccattggtgacccctttttgaaacgctgcccccccccaggttccg
ccccccctgaaaaaaaaactccgcaaacatttgacaacaaacgatggaaggcgccattttgaaactacaga

```
gaagcgaggttttgaaaaattcccccaaaccacttgtacggcacgaggaaggatggatgactcatatttc
atcaaaaaattgcaagaaaaaaaatctctgactgcgatttctggcctgaaaacagccctctgtttgcggc
taccttactgcagggcgcccccaacgggcgttaattgagtttttgctccttccgaccccaaattacccccc
cccccttgttcgctcgaattgctgtgatcggacgacggtccatcggccctattcagattttgggcgcatt
tgttgcggaaccgccattggtgacccttttgaaacgctgcccccccccccccccaggttccgcccccctg
aaaaaaaacactccgcaaacatttgacaacaaacgatggaaggcgccattttgaaactaca
>KolobokP-1_LyPi-I
tttattgtatgacgtaagaattttaaaaattatttcagcctccttggtgttctttcacatctgtacagtc
acatgattacaaaaaattgcactcaaaatacaaatataataagtaatgactcatttaattgactcatata
tgtacatgtttataggcactctacacaatatcctacctgcacactcataataaatgtattgtaagaactg
atatacttatgtaacataaaggtgtaaatatgtacaatttctgatcatatctgtatgctagaggccccaa
gacaatgtcctccctcattggtacatgatttgcataagaaggaaatgagaaaacaatgaaataatgtttc
tttttacatatattacaaatatttgtcttttcttcagtttattatcatggtataaagaatgagaaaggag
aatgagatgaagagaaagagaaaagagagatagatagaaagaaagatatagagggaaaggggggagacata
gagagaaggggggggagcaagaaaaaggacagagagagagagagagagaaaaagaaataaagaggaagtaa
gagatacactgaatggtagttgtgcaagtctattgagggtgattgaagaaatatgaacattgattctctg
tgcacatcttcccagtaaaagatttcaaatgtagaataaaatcataaccttattaattctaaatgtctgc
agttcaacagaacaaaggaaggattaactcactggtgcatacaagagtgtaaataaactacccttctaca
agattgagagagatgatgggggggagtggtgatgcacatctcatataaacatcatactgctcagtttgtt
cttgtcatcttgtagtgtaaagaaaattcagttggtgagatgacattgcatcatggctaggaggaaagat
tcatacaataaagtaaaaaagacacaatttaaagttaaacaccaaccctggaataaaggtcaatcatgtg
aaagggaattgctgaagtctaccagctacgtgcgattgacaagcaacgatatgaacagaacaacttctgt
ccatggtgatctacagtcaagcactatcgaaacacaagttaaaacagtaatgcttcttcgccctaaaatg
ggagaaagtttacctttgcaagaattcacagagccaatatctttatctggaaacgccactggcaatcgcc
ttgtggacatggagaaattagcagagtgcttcaacaacgtgatcagtcagcatcaagtcggtagtccttc
ttgttttgctccaaatctgttgataagtgataagggtgaaaatgtcagaggactagcagtaagtgagcgg
tttgagtgtaccaagtgcaattttaagagtacccaccaaaaactatacaaggagattgaaagcaacagga
gaggaaggaaggcagctgtaccaaacatggcgctccaagttggtctttaccaaaccagtattgctggtac
agctgtaacaaggatgcttacctctatgaataccccctgtccctgcaatgagctcccttcaaagaaatgct
aacaagtgtggacaaatcatgagagcagaaaacaaaaagtgatatggatcgaaagaagaagagggtgaag
gatatacttgaatggaggggaatggcaagggatactcccataaacattgaaattgatcgccaatacaata
ccccattaagccatgctcgacgaaagacccctttttgccccagcaagccagtccagggacacagtagctga
aaatgttacccatgaaaagtacattctagcatataatggaacaaataaactgtgtcgacatgggcagagg
cttgtcagggagggaaagaaacctttatgccccaatcatggaaagtgtactgcaactgtacctctctctg
caaacattggagatgaggaggatggaggagagcggtgtgccgagatgctactagatggatcagagagcat
aactgtgggagcagtgaccacagatagcgatggtcactttgcaagaggtataaagaaagtaatgtacaaa
agagcaaatatagacccagagccgctgctgtgcaacactcacctgaaccgaggcttggcacgaaggcttg
gtaaacttgaactcagcaagcaggcttttccagcaaagtatgtcacaggccggcacaaactgcagaggaa
ctttgctgatgacgtcgcataccgcgttgaggctgaaatgagagcagcaaaattgaaaaaaaggtgatata
tcaaaagtggattttagccaatgtgtgccagctatcattctctgcttcactggaaaccatgaattgtgtc
atataaatagtaatgtatgtagaggtgactttgactttgtgtactccagtctcagagttagacaaaccac
atgtttcacacagcttgatcgccaacgaattcagactgtcataatgcagcgcctgaacccatctactgtg
tccaaatcaaggctacttagcacaacccagaaatgtgagtcgatgaacagtgcatttaataccacccatc
ccaagcaaattaaaacctttaccagaaactatgaaggtagagaccactctgctatcagcttaaccaacaa
tggagcaggggcatccatcctctccaagactgcagcagcaaaggttccactgtctcagagtcccagagtc
gtgaagagactgaaagagatgcagagaagacgggattattggaaggccagatcaaagaggattcatgtca
agcatgcaagagctgttcatcgcatatataagttcagactgtacgagagaaagcgaatggacattaactc
caaagaatgttatttgaaaggacagttggaccaataccatagtcatatatatgtagatcatagctatgat
agaaccctgctgaacactgagagtgatagtgactagactctcccagaatagaggcaataagtgacattaa
tcaggtttctaggatgaatgattattttcaaggaatagtctcatgccagtgtaggcctacacctgatcta
ctttagatgtgagcctaactcatttttgttctgttggcccagtaactaacctgcactgtgtttagatctt
gactgttataggcctatgcctaaattagaattcttcattcaaattctaacataaaaatagaattaggcct
aacttaataatagacagcctaacttacttctagcctaccttaattggatcgggggaccttacttagtcca
actaagtctaagggaggagcaactaactataatgtaacttagtctaatttaaatatacatgatggtctgg
ttccatgccctagacatctttgcaaagaattgcaattccgtctagataggtttacttcatggctttcaag
gatcaaatgtgctggtctaacacaagaaggctgacagcacaaatgagatacttcaagctcaggaggcaag
gaccaagaccctgcaatactggtcatataggccaccctgtgtgctgcacgcacggtctggcctatccctg
gtattgtcacatccatacatttgtaattcgagcctttcttgctcctcccattgtatatccagcatataga
tagtgggtccccctcagaaccgttgtgcctttctctcaattttttttctgtagtgctcgcaccactcttct
tcagtcagatctttcaatagacttctccccattcctcttccaacttcagccatgtctctttttttcacaag
cacaagttatcaaacggtgttcctatcgaccgaacgtaagaatagcacagcattacaatcgtcggtagat
atggatgtagaacacgcacaggcactctaagttaggcctgcacacacaattacaagcatcggcctaacgt
taggttaggccctacagtatatttacctgcattatgcatatagccccattccgtcggtcgtacggccttc
aaattccactttttttgcgtcacctgatgatttaaacatggaaattagtgcaaaatattggctattgacga
gttttgacaggtatttcatcaacatccccaaaactgaaattttctcctattctccagtaga
>KolobokP-N1_LyPi-I
tatattatatgacctatgaattttattttttttaaatagctactttgctattcttttatatctgtacactc
acactagcacaaagaaatgcattgaaaatgcctattttctatgcaatgacccattttaatgtgttactca
tacatgttatattcactatatggatgtctacatatcctacctgcatatagcccaatgagtgaatgcaatg
acaatatatatataggcccctattctccttgtaatataaaggtgtatgtaaattttgtgtagatatcaggat
accagaactgccccaaacataattaccagatatacatgagattgaatgaggaaacaataaaagaatgaga
acatattactttagtacatttgcacttgacctattatatcaaaatcttcctagggctattgcctatcaga
tgtaagaaggaagagatacataaatagatagaaagagagagagagagagagagagaaagaaagaaagata
attttatagatggcatggcttataaagaggggggggctagactaagattggagcacacagccaccccccc
tccctccttagttgagcactattagataccttaccttactctgtgcctttttctaaataccttcttgaag
gtaccactctagttctcttcagtaaagttcacaaagttctatttcctctctaccccattccaaagaacaa
ttttaacatgagcaatttgtaaaggtctttctaacattaagwgggkgatsawacactwgcacaagccaat
```

tgtcaaagcagaaaaatcaaagaataagatcgccgaaagtttgagtaagatcggactagcaatacgagag
ttatgagcatttgaatattgagatcactaatgctatgtagatcctcccattggcaatgcgaccaagatct
atgatgtcacaacgacaactcccccctttgagcactgagataccccagatatccgtttttgtgtcactc
gatcataaaacattcctcaatgacatgatgtgctgaaatctcactccttcatgagaacagttcgcctgtg
atagaatgataaaagtgagattatagggtggaatgcagtactaagcaatgggggagttgtacgtgtgtga
catcacagatcttggtcgcattgccaatgggaggatctcatagcattagtgatctcaacattcaaatgct
cataacttccctattactcgttcgatcttcctcaaactgtcagtaatatggttctttgatttttctgctt
tgacaaaggctaactggttccaagggtttcattctcctttaagtaggtgatcatacactagcacaaacca
atgatgaatcagaacataaagagcctagactctcattgactagtctatatgcattcctaggcttacatac
acatacctactaactttacctgatatatagccttgctaagccattaaatccatttttttgcacccttgat
gacttaacatattgatatcagttagaaattgtttgctactgaggagtttgggcaggtatttcatccacta
caggggaattcgcctgtattacctatattatatgacctataaattttatttttttaaatagctactttgc
tattcctttatatctgtacactcacactagcacaaagaaatgcattgaaaatgcctattttctatgcaat
gacccattttaatgtgttactcatatatgttatattcactatatggatgtctacatatcctacctgcata
tagcccaacagtgaatgcaatgacaatatataaggcccctattctccttgtaatataaaggtgtatgtaa
attttgtgtagatatcaggataccagaactgccccaaacataattaccagatatacatgagattgaatga
ggaaacaataaaagaatgagaacatattactttagtacatttgcatttgacctattatatcaaatcttc
ctagggctattgcctatcagatgtaagaaggaagagatacataaatagatagaaagagagagagagagaa
agaaagataattttatagatgacatggcttataaagagggggggggggggctagactaagattggagcaca
cagccacccccctccctccttagttgagcactattagataccttaccttactctgtgccttttttctaaata
ccttcttgaaggtaccactctagttctcttcagtaaagttcacaaagttctatctcctctctaccccatt
ccaaagaacaattttaacatgagcaatttgtaaaggtctttctaacattaagtaggtgatcatacactag
cacaaaccaatgatgaatcagaacataaagaggcctagactctcattgaccagtctatatgcattcctag
gcttacatacacatacctactaactttacctgatatatagccttgctaagccattaaatcaatttttttgc
acccttgatgactaaacatattgatatcagttagaaattgtttgctactgaggagtttgggcaggtattc
catccactactaggggaattcgcctgtattacttata
>KolobokP-N2_LyPi-I
tatattatatgacctatgtattttattttttttgaatagctactttgctattcttttatatctgtacactc
acactagcacaaagaaatgcattgaaaatgcctattttctatgcaatgacccattttaatgtgttaccca
tacatgttattttcactatatatatggatgtatacaaatcctacctgcatatagcccaacagtgaatgca
atgacaatataggtccctatcaattgtaatataaaggtgtatgtaaattctgtgtagatatcaggatacc
agaactgccccaaacataattaccaaatatacatgagattgatgaggaaacaataaaagaatgagatact
acttattagtatatttgcatttgacctattcaaaatcttcctagggctgttgcctctcagatgtaagaag
gaagagatacataaatagagagatagagagagagagaaaaaataattttatatatggcatggcttaataagag
gggggggctatactaagattgcagcacacagccaccccccctccctccttagttgagcactattagatac
cttaccttactctgtgcccttttctaaataccttcttgaaggtaccactctagttctcttcagtaaagtt
ctcaaagttctatctcctctctacctaccccccttcctaagaacaattttaacatgagcaatttctaaagg
tctttctaatgctaagtaggtgatgtacactaacacaaaacaatgataaatcagaacataaagaggcctt
gactctatacattgaccaatctacatacattcctaggctacacagacctactaacctttacctgatgtag
cccctgctagccattaaatccatttttgcaccaatttgatgactaaacatattgatatcagtgagaaatt
gtttgctactgaagagtttggccaggtattccatccactactagggatttcccatattacttaca
>KolobokP-1_ApJa-I
ttaagtgaaagttgaaatttttaaactatactttaaacctctttttatgacagtttgagaagtcttgaaa
accagaaaatacataactttgacaatttgtttttatatctgtctatttggtgtctcttcatattgtgcct
catacaaattgtacaggccaaatgcacagtgcaatactgaatattaaagaagcaagtctccactggttag
catgttccctggtgttataaaaagccaaccataagcatgaatgttgtgtactgcatgtctaaggtgcttt
atggtttatagaatgtgttttaagcacagtaactcagtttggtgctacttaacccgccaggcgggagaaa
tgttcctgtgggacaggaaacttgacaaaactttgctttccaacctctcttcttccctctgaaatcttca
tcaatcccagcatccagtaaactttagaacatagctgtggccctgagttgaaagagaaaaatgtacttgt
tactttggcactatttttaaaaggaattttagcagattttggggccaaaaaagctcagtaaaacagtgtg
ctgtgctcagatctgaagtgagggcgctgttcaaacaacgttcccaaatcgttgtagtcgatcatattga
ctagtttctcagtgtaaatcttgttgatatcagtctatgcacagcttttcaactgcttagactttcaata
attgctaacaaacaaaactagtgaattagccactggaaactatttatttttaaaggataacataggaaat
actatttaaaagaatgggtagaaggaaaggcatgtatcgtaggcgtgcaacaacctttaaaacaggtaac
aggttcggttttacagcttgtacagctgatgaaactgatgccaattgcactggtgatatgcctagccctt
ctgatacaggtgtggcaactatgtcctcttgcaaacctgcatataaaaggctcagcaaggaagagtatga
gagggtagttaactctcctgtcaaaggacctgagatattttcatctcagtcccaacaaacaatgttacta
aggcctactaagcaccatgttggccagcttaataacatggctcctgctagtgttgcagaatctacaggca
acagggtagtgaatttgcaagtcacagtaagagcctatcatagtgctatgatggctcacagggaggcaag
caatggatgtgcagggctcctcatatctgtttctgatatggaggaatttgtgggacttggctcaaaggtt
gtgtttggatgtactctctgtaactacagaagtgagctcttgcccctctataaagttttaaaccaaagta
acaaacctggcaggaatccctcagagctaaacattgcattgcagttaggcttgcatcagacttccatcag
ttcaacagcagctcgtagattgctcagcagcatctctcttcccagtcctgctgtcagtacactgcaaaag
tcagccaaccagtttggacctgtaatgaaagaaattaatgaaaatgatatggctgaaaagaggggtataa
taaaggagacactgcaactgcgtggcctctcaagagattctcccatttttgcagaatatgatcggcagta
taataaccctctgcgtaattcaagaagtaagactccatttgctcctgcaagccaatgcagagatgtcatt
gtagagaatgtcaccatggacaagtttgttattggttaccatcacagtaacaagttgtgcctaatgggag
aaatgaaaagaagaaggggtcacaaggtacaatgtccaggccataagggctgttctgcaaatttaaggca
agatgccaatattggagatgaacagaatggtggaaagatttgtgcttcaaaattactagcaggtaaaaac
ccagtaaaggtcaagttcctcacaactgatgctgatggcaaggcatgtaaagggtttaatagtatcatgt
ctgaggctattcagtctgaaacagagaacctttagaccaggtgcatctaaacagatctctgcgcagagc
actcactggagcagaattctcctctgacatgtttcctgcaaagactgtgacgaagaagcggttgatacaa
aagcgatttgctgaggacttgacctaccgtgtacaggcagaggtgactgcttgcaggaaagtgcacaaat
atgaccactgcaaagttgcaaaagctttgtcaacatgtatggactgccttactaagtgctacagtggaga
tcattcagattgcccaaagcagtcatttgtatgtaagaggggcagatgctacaaatttccatttatgcca
aatgctgcaaagggttctctgataatatcaaacagtgacaatgcaaaacttatcgaattacttaaaacac
gcacctgctacaaacgattatggtccactcgttttgagacttccactcaaaaagcagaagcagtcaataa

cgctttcaaggtgactaatccaaaacatagcaccacatttgcacgtaacagtcagtacagggaccacagt
gccatacacatcaccaataatgggcctggtgagtccatagccagtaagatggaagctgctgggttaaagt
taagcacaaatagtccatgtatccagactctgaaacagatgcagagaaaacaagaatatgataaattacg
caagagaggcagagcctaccgaaggaggagagcacaactaagagagagaaagtacaagatacatggcaga
atcaaatacataaaccagcacagttgttataaaagagatcaactgatacatgaccacagttactctaagg
ggggatgtgacactgatgatgatgacaataattatcagtgatgcaagttatcatcaaagcaagtgataat
ccaaaacagtaatcactcactcactcactcattcactcagggaaccctaagcaatcagggtaatgaccat
gaccctggcaagcaccatcaaacttgcaggaccttctgctgttgttgatactgcgggattctgcagagag
gtgtgacacctccacacataggctattgtggcaaatgtgggagatgtcaaatgatctgctactaggacta
gttataacaagttttctctgcaaaatattctgaagagcatatgccagaatgtggactctgatggctcttc
tcttagctgccaggggtagcattgtgcggataatgccatatgctggggcccctggcttactggtggtgta
ggcacctgtccacattctgcatccccttgtgccacttgctgtatgtttggccatcttttcagtaatgcca
tcagcccatgccatccaatcttggtctgacattgtagagaagtttggagattgccagtactgtatacagg
gcctatgtttaatctcaatgcagcctagcacagtgcagctgcagtcacatcwaatactaatgtatatgta
aattctgaagtgtccgcttgtttttacactgtataaaagtatatgctactcacgtwtgtacaacttcagt
ccgtcaggttttagttgttctctatagcctaacatcgaaattagtggctccttataccagcctttacttt
caattactatacacatctcagtagcagacagctcaaaacaacatacacaggtggtactgtggcctataca
agccatgcagatgttcttcaagtccaaccattactcaccagtgcattttggagcacacaatgcaccaatt
tatttttcctcggcaatttcatgccatttttcgatattttgatgacattccgcaaaatttgaccccgaa
atcgcacaagcgtaaaattttagacgttttgtcatatttcattgttacacatcataccgacaccttcag
ctataataacgctcatagagtctaaaacggcaattttcacattttgcttaaggc

Supplemental Data S3. Multiple alignment of *KolobokP* DDD/E transposases (fasta format).

```
>Kolobok-5_TV1p
ME-------------YS-----------------------------------------------------------------------------
----------------------------------------IPIMEGYT----------------------------------------IEE--
------------------------------ILITYKADDC-----Q-K-RVYH--RT-------------------VYSN-------------
--K------------------------------------------------------------------------------------------
----K------------------------------------------------------P---------------------------------
-------------------------------------------------FSHFINLNQL-----NSKQDH-SQ-SNVK---------------
-----------------------------------E--ETIFNV--------K-F---K-P-G--------VPYSDV----------------
----VKNLHTYISLIS----GAGFSKHIELASL-LNFHCPTETTLLAHLKSILPDLE-KV------------ATASCEANIQEIL----
KHDSEYK-----F-------------------------------NVSLDCAWSSK----------RDALHAVITLIDINTNK-----------
----------------------------ILDFRIVSR-------HPELIK----------FKSP-----------------------------
---------KI--L-------------VEPKISPGLMESYA-LRDMLKSDLWKKIKVFCSDGDVKDSTMIENS--------------
GAQLSHVRDPNHVIKSA-FSKIVTKYPKFEKIFRILFAC---P----
TLKPEQKVQRWMGLINRYPGESPEHIEKREIITNIAPLFLRISKQYHTNFNEAFNSLKAHLVPKNLRWIIGFPARIFVSVFAYNEDNWKTKLR
R-LMGIDDSYFDEDILQVILNFENKSDYDH-------IRRTSE-----------
NYRNHNRCVHYIPDEIDPEFADLVPHKPSKDGSRSEKIEYTDDEDPPADEEPSESENLSLPTDYPK-----------------------FNK-
KSIFGSDIKVP-PDWKRFSF-FFKAK-------------------
EIPDDPQEFQNLLSKFKQDLYNTYSSLIRINEENISAVAVYDSQQAKEIMNSAFTDFHNFEKIKINAINYLNVLSHNPPSL-E----------
-MIADFHPDLIQLPQTLRKTHDKPKKQKLGIKLGKNLSFLAVIVQLITFRPLMHKLFDELFSNPNEQQLIYKEILSLTNRNFSEN------
ADVTYLYH------LYSKRQKIHQAD-------------------------------PIDAFKQLQESLA------
CEARQLDLIDYFKKFCWGFTDKIDIPLVNNNELQVTPTFSDYLFIDVAG---FTFPLDDYTWNNNPAVKSKLVAIITYHSRQYNI--
IILDENINSWILFC-NTKISYIRDEEL------------------------------------------------------------------
-------------------------------------ANNLNKG-AYNT-------------PKY-LIFEKI---------------------
----------------------------
>Kolobok-6_TV_1p
---------------------------------------------------------------------------------------------
-------------------------------------------MV------------------------------------------------
------------------------------TLFTVKKLGC-----KDG-TMITIIRP------SS-----------IFKS-----------
PLN-------------------------------------------------------------------------I---------S-
TFAEISRAEI-S------VPDGAI------------------------------------------P--------------------------
------------------X-----------EF--I---------------------YSRLYNFN--------------NN-ESTSSLQMCQ--
----CTFNSSKECTKMQNDEMICQQT-CEPIQDDYETGEELEE--EEIFEEEDL-----E-----N-E-G---------EKEKEKSKTFH---
--------EKY-TDQELDKHLFNCYIHGIGIQKYFDILTY-AGVKVPSVDTLNKRQAALGPSLE----------------EFCLNRIQKYI--
---------------------TDDM----------------------KAAYDCAWTGRRNAF-------------------------S-----
----------------------------SFSSLANIVLNKICGFRIV----TKY-KIDGYKTVISDP--------------------------
------------D---I-QANMMEITALN------------SII-NSD-----
IFPKIHTFVSDGDVKTNTALNHSKTSIQITKDSNHLIKNAFNKFKDQLHFLES--QFRIIFADN--------------------
TLTIEQKVQKWLDTKKKYVNRQ-
NYSEICETIDATAFLFRCIRKGLHTNFNESINALKARLIPKFSSWSIGFVIRMFVVVIAYNEPRWKELLRTEFFKFDISMYDEQIQNRIKRIE
SRSEHVRNHRAQKETKKRAIA------VRWARKHKANVEPG-----------------------------------AEAXKSPKKETN----
TIKIA------------------------HKKKDLIGN---IPIKDWKRKFF-FITNVALDNVDFQSAVLSIPYLYVRFSKDYKYAVILIK--
-----------DYNASMIIDSSIYLSMGLEREPLDDEVA---------------
QKVIENCPVDSGLVVFPTNKFVVSKSPTFNPGLDNLCRN---------------------CFLNVIIQLFTFNEFL--------------
KIEYTNILSMIQENYSTPDHPNQNLQLAQIFANCIHRPTDQISSAVHILD--TLIS------DTHYQPK-EDEGFVXL-
ENPIEALSLFVDKLSQIPVNNIDINSMXKGLFYIN-----PAQIEIEVCDGKVETQIDNFPPILFANVVNTNQFYLPL---
RFDEHPEFYYDLVAVISNPEDHFTI--YMKKTNS----IW-------YYFNDL---------------------------------NSD----
--F-------------------------------------------------------------VRA-DF--VYKLTRKEERVFLCVYQ----
-------------QRQC-----------------------------------IVQ
>KolobokP-1_SaGl_1p
---------------------------------------------------------------------------------------------
-------------------------------------------MP------------------------------------------------
----------------------------------KKGP-------K-W-L-----HQ-------H-----------QYKK--------
GSIPHN-K----------GQKK------------------------------------------------------IGTTSPVQSL---V-
PIRRLSKEEF-Q-DIVRPQKNG--------VFTVIDAK----SRSSEM----KFLR----------PGPS--EV-Q------PVDTYMES---
------------------V-SD-----DP-N---S------------K--------LYKVLHQEKTAKLWNKAFKEH-LQ-KFPD----
CDGPLDW--NQHGEEKRGLAWILSIRCKK--CHYTSH---------R--EKLYEEVR--R---S-----G-R-G---------RRAAQ-----
----------------LNVAMAVGHLGT---SLTTEGMRGMLLG-ANIEPASAANMQQTCNKVGKIIT-DV------------
NKQSMKKIRQNI-----QELNEKC----GL----PDTPI----------------------
RAEGDARYNNATFSAIGKTPFQAATQVTYTVSENVTQR-------------------------------------
KNVVAVFCGNKLCKKGAH----LR---SKG-KE-VTCP-------------------------------GHE----D---C-
TATIPPETTIG-DEKRWAANCVGELQ--SDD----RPLVISHFTSDGDSAAASGASEK-------QG-H-----VIENLKDLRHFFDS--
QRKQTAKAP----FSSHMFPG------R-TKAIRESMQRRFALD--------------------------LKLRCRTEYENCYKHYSG-----
-------------------DLPLMKRAMST-------------AVGSLINCYQ-GQCGKS------CQLYSF-----------------
SCRG--------------------------------------------------------------------------------LK-S---
NK--------W--KSS-VLPSD--------------FELN-MTEKDKCLLKDCIN-------------LTLGQSNLDRIKYHTCTQKSESVN-
------------------RAYLKSNPKCI-----------------------------------------------------------TS---
--------------------------SRNFES-----------------------RIHKVVHSLN--RGHG------NSTLELC-E-----
SV-GAPVVKGSRVVHHL----------KQQQNRQNYFK----------------------------------------------
MKQKLLKYKFQRKYYRSKRYQLYD---KKSKD--------------TYSKSLT---------------------------------DPK----
-------------L-------KRK------------------------------------L--------------------------------
-------------------------------------------------------
>KolobokP-2_CGi_1p
---------------------------------------------------------------------------------------------
-------------------------------------------MP------------------------------------------------
----------------------------------KKGP-------K-W-L-----HQ-------H-----------QYKK--------
```

GNIPHN-K----------GQKS--TGTTSPVQCL---C-
PIRRLSKEEF-Q-DVVQQQKDG--------IFTISDAE----RRGSQM----KILR----------PGPS--EV-Q------LVDSYTDA---
------------------E-SP-----NP-N---S------------R--------LYKVLHQEKTAKLWNHAFKEH-SQ-KFPD----
CDGSLDW--NQHGEEKRGLAWILSIRCKK--CHYTSH---------R--EKLYEEVS--R---S-----G-R-G---------RRAAQ-----
----------------LNVAMAVGHLGT---SLTTEGMRGMLLG-ANIEPASATNIHQTCNKVGKIIT-DV------------
NKNSMKKIRQDI-----QELNEKC----GL----ANTPI----------------------
RAEGDARYNNATFSAIGKTPFQAATQVTYTLSENVTKK-------------------------------------
KNVVAVFCGNKLCKKGTH----LR---AKG-KE-VTCP-------------------------------GHE----G---C-
TATIPPETTIG-DEKRWAAECISELQ--SDD----RPLVISHFTSDGDSAAASGASEK-------QG-H-----MIENLKDLRHFFDS--
QRKQTAKAP----FSSHMFPG------R-TKAMRESMQRRFALD--------------------------LKLRCRTEYENCYKHYSG-----
-------------------DLPLMKRAMST-------------AVRSIISCYQ-GQCGKS------CQLHSF-----------------
SCRG--LK-N---
NK--------W--NSS-VLPRD--------------FELS-MTEEDMCLLKDCIN-------------LTLGPNNLDRIKYHTCTQKSESVN-
------------------RAYLKSNPKCI---TS---
--------------------------SRNFES-----------------------RIHKVVHSLN--RGHG------NSTLELC-E-----
SV-GAPVVKGSRVVHHL----------KQQQNRQNYFK--
MKQKLLKYKFQE---

>KolobokP-5_CGi_1p

------------------------MTVNDAE----GRDSNM----MILR----------PRGR--EP-R------TIDLYGYSE---------
-----------A-AG-----SP-E---S------------E--------TYRLLHNKRLP-----SYGTI-HL-RIID-----PGSLDW--
NYSRDQKRGLAWITSLRCDN--CGYQSP---------P--RKLYEEVK--S---P-----G-P-G---------RKAAR--------------
-------LNLAMSVGRMGT---CLTTEGMRGMLLS-ANIHPGSASSMQETANKVGEKIL-ET------------NQQSMRGIRQKI-----
QENTTKC----GL---PATSTI----------------------
RAEGDARYNNSTFSAVGKTPFQAATQVTCTMCENVTSKKKSLQCFVETNCVKKERSCGGEGRMLPVQTTQTVLQTKKVIAVFCGNKLCKKGEV
----LR---RRG-AN-VTCP-------------------------------DHP----D---C-TANVAADTNIG-NEKRWAGECIREIQ--
RDP----HSLTVSHLTTDGDSAAATGASEI-------QG-K-----PIENLKDLRHFFES--QRKQTSKAP----FSASMFPG------R-
TKTDRESVQKRFAQD--------------------------LKMRCRSEYENAYDHYGG------------------------
DIRKLTRAMSY-------------AVDSILTCYR-GECGRP------CQKHSF-----------------ACKG-------------------
---LK-K---SH--------W--KSS-VLPKN---
-----------FKLN-MNDNDEHLLRDCVN-------------LTLGPKNLCRIRFHTSTQKCESVN-------------------
RAYLRSNSKCI---TS---------------------
--------SRNFEP-----------------------KIHRVVHSLN--EGYG------NSTLKLC-E-----AV-GAPVVKGGKVVHLL---
-------KQQEKRQAYFR--QRQQSKLYKSKRKYYRKVRYQLYD---
ERNEN--------------KYCKSLT---------------------------------DPK-----------------L-------
PKKRNS----------------------------------RA-ARV-EH--SY--HRK-----------------------------------
----
>KolobokP-1_DrRo_1p

---MR--
---------------------------------LSKRR-------A-H-L-----RK-------S-----------GFKK--------
GCVSPK-K----------GQTV---AF--PTLAD---N-
KFVRLEQKAF-D-SRVHT-SNN--------VLTFKDTD----GTDTTV----SPLR----------PRPN--AS-N------VVDEYSGC---
---------------DD-E-SI-----HP-D---L------------F--------TNKLMVQAKVQALFNSAFKEH-RH-DKPN----
CEGDLNF--DAAHAIKWGAGWRERLKCTK--CSYVSA---------Y--HKLYEEVE--N---K-----KGP-G---------RRAAK-----
----------------VNIGLQLGLCTT---PISNTGLQRIFNN-ANIIAPNLTSMQRLSNKVNEKIQ-SV------------
NIRDMREQRVTL-----VKENAMI----GQ---KNAQTV----------------------NVEGDSCYNNPMFNS-
DSTPFQAGSIATTTFCENNTKD-------------------------------------KKIIGVHIANKLCTIGAR----LR---NKG-ND-
VVCP------------------------------NHSG----H---C-SANVSESDPIG-NEGRWSEIVARNIS--NE-------
VQVTGYTGDGDSRSHAGVSKA-------SK-H-----TILHFKDIRHLGNS--LKRAVYAAN----FSSNMFKG----------
SSNTNLKNRFALS--------------------------IKQRCVAELKMAHKSYNG------------------------DINKIKQCMPK-
------------VADAIVACFG-GYCGEP------CKASSL-----------------VCCG-------------------------------
--N---YR--------Q--AKN-YMPMN--------------
VKLR-MTEGDKVLLLKCMD-------------IMLSPTALDKTKLLTSTQKCEAVN-------------------RSYQTVNPKMI-------
--TN-----------------------------PRNFAG----
-------------------RIHGQVHKLN--NGYA------VSVLIKT-K-----SL-GANLTKGSSVIRQI----------RTTDAISKKP-
--NKDVIQRRKITRYAARQRRYHLHE---R-----------------
LHYNKGLT---------------------------------DTV-----------------H-------GMEGFG------------------
-------------------QLR-DH--NY--V---

>KolobokP-1_DrPo_1p

---MG--
---------------------------------LSKRR-------A-H-L-----RK-------V-----------WFKK--------
GSVSPK-K----------GQTV---VF--PTVAD---N-
KYVRLEKKAF-E-SRVHI-NNN--------VLTFKDTD----GTDTTV----SALR----------PRPN--AS-N------VVDEYSGC---
---------------GD-E-SL-----HP-D---L------------Y--------TNKLMVQAKVQALFNSSFKKH-RH-DKPN----
CEGDLNF--DAANSVRWGMGWRERLKCTK--CSYMSD---------Y--HNLYEEVE--N---K-----KGP-G---------RRAAK-----
----------------VNIGLQLGLCTT---PISNTGVRRIFNN-ANIIAPNLAAMQRLSNKVNEKIQ-SV------------
NIRDMHEQRVTL-----VKENAII----GQ---KNAKTV----------------------NVEGDSCYNNPMFNS-
DSTPFQAGSIATTTFCENNTKN-------------------------------------KKIIGVHIANKLCTIGAR----LR---NKG-NN-
VVCP------------------------------NHKG----H---C-SANMSESDPIG-DEGRWSEIVARNIS--NE-------

```
VQVTGYTGDGDSRSHAGVSKA-------SE-H-----TILHFKDIRHLGNS--LKRAVYAAN----FSSNMFKG----------
SSNANLKNRFALS---------------------------IKHRCIAELNMAHKSYNG-------------------------DLNKIKQCMPK-
------------VADAIVACFG-GYCGEP------CKASSL-----------------VCCG---------------------------------
-----------------------------------------------------N---YR--------Q--AKN-YMPMT--------------
VKLR-MTEGDKVLLLKCMD-------------IMLSPIALDKTKLLTSTQKCEAVN--------------------RSYQAVNPKMI-------
-----------------------------------------------------TN-----------------------------PRNFAG----
-------------------RIHGQVLKLN--NGYA------VSVLIKT-K-----SL-GATLTKGSSVIRQI----------RKTDAISKRP-
-----------------------------------------------NNDVIQKRKITRYAARQRRYHLHE---R-----------------
LHYNKGLT---------------------------------DTV-----------------H-------GIRGVG------------------
-------------------QLQ-DH--NY--V-----------------------------------------------------------
----------
>KolobokP-2_SiCo_1p
----------------------------------------------------------------------------------------------------
-------------------------------------------MV---------------------------------------------------
---------------------------------LSRQR-------A-H-N-----RR-------V-----------GFTK--------
GHKSCL-K----------GKSF-----------------------------------------------------HFA-KPETP---V-
PYVRLPENEF-A-DRIYC-HDG--------VLTFRDTD----GRSTNV----KPLR----------PRPV--PV-D------SVDQVLCDT--
---------------CP-D-SP-----FP-V---S------------N--------CHKVYDLDKVSHLYDSGNRKH-GL-CSKG----
CDGRLLI--DSDNSKKWGLVWSERLKCTK--CSFVSD---------Y--CKLYEEVEG-K---S-----K-R-G---------RKAAT-----
-----------------VNVGLQLGLSTT---PISNTGICRVLNN-ANIVAPSISSMQRLSNKVLEEVI-SL------------
NQIDMQDKVRAV-----VKTNTLI----GQ---TNPLNI----------------------NVEGDTCYNNKIFNA-
DTTPFQAGTIATTTFCENNTRS---------------------------------------KRIIGVNIASKLCLVASR----MR---NKG-EE-
VTCP------------------------------HHAG----Y---C-TADISESSVIG-NEGTWSEKVAKNLT----------
GINIAGYTGDGDSTSHAGVQKA-------QP-C-----RVNMFKDVRHLGNS--VRRAVYSTK----FSSSMFSG----------
PSRCNLKNRFALS---------------------------IKQRCHAELKQAHKKYNG-------------------------HLPTIKFKMPM-
------------VIDTIVLCFN-GYCGQS------CKDNSL-----------------VCSG---------------------------------
-----------------------------------------------------N---YR--------L--AKN-FMPAN--------------
VKVR-MTEKDQDLLKQCIG-------------IMLGIEALEKTKLLTSTQKCEAVN--------------------RSYQAVNPKMV-------
-----------------------------------------------------TN-----------------------------PRTFKG----
-------------------RIHGQIHKLN--NGYT------RSMVMKA-R-----YL-GAPI-KNPAIIRQM----------
MAVDKRAARTK-----------------------------------------------DPGAIERRKVCRYSASRRRYNLHE---K--------
---------EHYSPGLT-----------------------------------DGK-----------------P-------AASSSK---------
-----------------------------HLK-DH--NY--V-----------------------------------------------------
--------------------
>KolobokP-1_MeMe_1p
----------------------------------------------------------------------------------------------------
----------------------------------------------------------------------------------------------------
----------------------------------------------------------------------------------------------------
P-K----------GT-------------------------------------------------------------------------------------
----------------------------------------------------------------------------------------------------
---------------------P-A---T------------I--------T--------------------------------------------
----------------------------------------------------------------------------------------------------
----------------------------------------------------------------------------------------------------
---------------------------------------------------PCLTP-KVRPFQAGTIVATTICENNTHR---------------
-----------------------KSIIGAFVGVKACTAASK----LR---NQGYKN-VKCP------------------------------
NHPG----H---C-TANLDEAQSIG-DEAKWNEMVSKDIT--KD-------LQISHHTGDGDSKGHSGVDKG-------QG-T-----
KTVHLKDVRHLANS--LKRQINRAP----FSDKMFRG----------KQKSNLKNRFALS---------------------------
IKSRCVSELSSAHKKYKS-------------------------NLKVIKHKMPN-------------IISAIVLCFK-GYCGTS------
CSKYSL------------------VCSG---------------------------------------------------------------------
-----------------N---YR--------K--AKN-YMPDN--------------VRLK-MTDDDETLLRKCIE-------------
VLLGPESIEKTKFLTSTQKCEAVN--------------------RSYNACLPKNV-----------------------------------------
--------------------TF------------------------------LRNCHG-----------------------RIHGQILRLN--
HGLA------DTVILKA-R-----VN-GVKLSHGSSVIRHL----------LRTEYYDKLRK--------------------------------
---------------TTRYISRARRSRFASRQYRYKLHT---D------------------LHYSKGLT--------------------------
--------DPK-----------------P-------DFSSTP---------------------------------------HLK-DH--AY--A-
---------------------------------------------------------------------------
>KolobokP-1_CorFlu_1p
----------------------------------------------------------------------------------------------------
-------------------------------------------MG---------------------------------------------------
---------------------------------RTWSRR-------N-L-C-----KR-------I-----------GFKK--------
GVASVN-K----------GTKF-----------------------------------------------------VFE-QHSTS---R-
KYVRLYKEQF-Q-NRIIT-SEG--------TLTFKDVD----GTDTGA----VPLR----------PLTE--ET-K------PIDHYVEP---
---------------ED-T-NV-----HP-D---L------------L--------TMKPYCPKLVQDMFNSEIQSH-ND-SNKS----
CTGRLEF--DGTGSRQRTLAFAERLKCTK--CTYVSK---------Y--YKLYDEIV--T---G-----K-R-G---------RRAAK-----
-----------------INVGLQLGLMTT---PISNTGAIRILAH-ANMIPPHRTSMQTMSNKVSASVE-TL------------
NKQNMHDIRQNL-----REQNEKC----GN---KNASKV----------------------NVEGDSCYNNPLFNS-
DSTPFQAGTIVTTTMCENNTKD---------------------------------------KKVIGVFVGVKLCKTASK----LK---NLG-NS-
VQCP------------------------------NHPG----T---C-TANIDQTQSIG-NEAKWNEHVTKDIN--AE-------
LSISSYTGDGDSKGHSGVDKG-------QN-D-----SVIHLKDIRHLGNT--LKRHLNKAP----FSNTMFSG----------
KLKSNLKNRFAMS---------------------------IKARCLSELKMAHKQYKG-------------------------DVNKINTKMPD-
------------VISTIVLCFK-GYCGES------CRKHSL-----------------VCYG---------------------------------
-----------------------------------------------------N---YR--------Q--VKY-YLPQN--------------
YKLK-MTASDENILYECIK-------------VFLGPQSVMNTRFLSSTQKCEAVN--------------------RAYQACMPKSV-------
-----------------------------------------------------TF-----------------------------SRNCHG----
-------------------RIHGQILRLN--HGFA------NSTVLKT-S-----AL-RCALTKGSSVIKRL----------
LKIDEQYKRIK-----------------------------------------------SERYINKQKATRFAYRMRNYQVHS---E--------
---------VHYKKGIS-----------------------------------DPK-----------------P-------DFSQIN---------
```

```
----------------------------HLK-DH--AY--V----------------------------------------------------
-------------------
>KolobokP-4_MeMe_1p
---------------------------------------------------------------------------------------------
-------------------------------------------MP------------------------------------------------
--------------------------------PASKRK-------K-I-G-----RQ-------H-----------GFQK--------
GMPNLSIK----------GKNF-------------------------------------------------------SYV--QNTG---R-
KFVRLQSDTF-Q-ARIHE-GND--------ILTFRDVD----GSETNT----TPLR----------PLSR--TQ-T------LAEEYAHS---
---------------DS-E-GIKN---HP-D---L------------L--------TNKLYRPLELSVMFNSEIKYH-ML-TYKS----
CNGDLYI--DTAASRKWGLGWYERLICTE--CGYCSK---------F--YRLFESVP--T---G-----K-K-G---------REAAK-----
----------------SNVGLQIGLMST---PISNTGAIRILAN-TDIIPPSPAGMQKQSNMVGEAIE-RL------------
NIEKMHEVRENI-----KAENEQL----GY---KNPRLV----------------------RAEGDSCYNNPLFNS-
ESTPFQAGTIVTSTICENNSKD-------------------------------------KKIIGVYVGNKLCKTASK----LQ---NQG-HD-
IKCP------------------------------NHKG----K---C-TSNLAESDSIG-NENKWSQNVVRDIN--QD-------
LDIAFFTGDGDSKSHAGVSAV-------QK-T-----HVNHLKDLRHMSNS--LKRELNKVP----FSKTMFTG----------
QFRANYRNRFSLS--------------------------VRARCVAELKKAHTIYRG------------------------DLSLIKQKMPN-
------------IISSIIMCFK-GYCGDF------CKKHSL-----------------VCTG-------------------------------
----------------------------------------------------N---YR--------R--VKY-FLPAN--------------
KKLK-MTKSDEEMLRACLQ-------------SFLGPSSLEQTRFLTSTQKCEAVN-------------------KSYQTCMPKNQ-------
----------------------------------------------------TF-----------------------------SRNCFG----
-------------------RIHGQILKLN--YGFA------DSVLIKC-K-----SL-GASLAKGTSVIKHL----------
LRTDSVHRRRK----------------------------------------------TSHYKLKAKLSRYASRTRRYKVHE---Q--------
---------LHYSKGLT---------------------------------DIK-----------------P-------DFSNIP---------
----------------------------HLS-DH--SY--AC---------------------------------------------------
-------------------
>KolobokP-6_MeMe_1p
---------------------------------------------------------------------------------------------
-------------------------------------------MV------------------------------------------------
--------------------------------RLKKQV-------K-H-C-----RS-------V-----------GFKK--------
GAPSTT-K----------GMKL-------------------------------------------------------CYN-TDKLE---Y-
RYVRLEKQVH-N-ATVHE-NSE--------VLTFKDVD----GNATMT----KPLR----------PKPD--SV-G------IIDKYGTA---
---------------QT-D-SV-----HP-D---L------------R--------TNKLYLPVNVQAMFNSEMRNH-RA-VHSE----
CEGDLVF--DGEASQKWGVVWRERLKCTK--CMFLSQ---------F--HNLYDEVD--T---G-----K-R-G---------RKAAT-----
----------------ANIGLQLGLYTT---PISNTGACRILNN-ANIISPCVASMHKTALKVGQGLQ-VL------------
NEQSMCDIQTNL-----KTVNEQI----GQ---KEPDLV----------------------NVEGDSCYNNPIFNS-
DSTPFQAGTIVATTLCENNTKS-------------------------------------KKIIGVHVGSKLCVVAAR----LR---NNG-RN-
VECP------------------------------NHEG----H---C-SANVPETDSIG-DEGRWSQKVASKLS-----------
MRIASYTGDGDSKSHAGIEKS-------QN-N-----KVTHLKDVRHLSNS--MRRELYKAP----FSSSMFKC--------
GSKQKSNLKNRFAIS--------------------------VKLRCVAELRKAHKVHNG------------------------
DISKIKKNMPK-------------VISAIVMCFK-GYCGQT------CKNDSL-----------------VCCG-------------------
----------------------------------------------------------------N---FK--------H--AYS-YMPAN---
-----------VKVR-MTECDENVLRKCIN--------------ILLGPDSISKTKLMTSTQKCEAVN-------------------
RSYQAVIPKQT-----------------------------------------------------------TF---------------------
--------ARSSSG-----------------------RIHGQITKLN--HGYA------ESVLLKT-E-----FF-KANISKGSKVVKQI---
-------VRTEKIRCTRL----------------------------------------------RSDMKQKAKRARYAARARRYKLHE---K-
----------------IHYSSGLT---------------------------------DPK-----------------P-------NYSAVK--
-----------------------------------HLQ-DH--CY--A---------------------------------------------
--------------------------
>KolobokP-4_CorFlu_1p
---------------------------------------------------------------------------------------------
-------------------------------------------MV------------------------------------------------
--------------------------------SYRKRK-------S-I-G-----AK-------T-----------GFKK--------
GQPSAM-K----------GKSY-------------------------------------------------------EYV-ETTSE---S-
KFARLAEQTY-Y-DRIVE-EGE--------ILTFKDVD----GSNTNV----KPLR----------SLSK--AK-T------LLEEYTHHT--
--------------VDS-G-YY-----HP-D---L------------L--------VNKIYRLLDVCLMFNSEMRKH-MS-LKPQ----
CNGYLMI--DAANSKAWGWAAIERLFCKE--CSYVSP---------Y--YKLYEEVE--T---D-----K-R-G---------RKAAQ-----
----------------INVGIQLGLMTT---PLSNTGLSRILAN-ANIPPPNLTSLQKMATHVSTSVT-EL------------
NEQSMSQIRQQI-----KADNKLC----GL---SDPSLV----------------------NTEGDSCYNNPLFSC-
DSTPFQGGTIAVTTMCDNNTRS-------------------------------------KPVIGVHVASKLCLVASR----LK---NKG-IN-
VECP------------------------------NHAG----H---C-TATIKESDSIG-NEARWNEAVTRQIN--QD-------
LNIACFTGDGDSKGHSGVDRG-------QI-K-----PVTHLKDIRHLANS--MKRAINKAP----FSKGMFNA--------
PPSHKTNMRNRFALS--------------------------IRSRCKAELKQCHKKYKG------------------------
DIHKVKKDMPK-------------IVSAIIMCYK-GYCGSS------CQKYSL-----------------VCNG-------------------
----------------------------------------------------------------Q---FH--------N--AKA-YMPAN---
-----------CKIK-MTVSDEAVLKSCIN--------------MLLGPDSIEKTKLLTSTQKCEAVN-------------------
RAYQFATPKLM-----------------------------------------------------------NF---------------------
--------ARNSTG-----------------------RIHSTILKLN--LGYA------DSVLLKS-E-----KT-GAPLKRGSSVIRFL---
-------RRTEQRAARLK----------------------------------------------SQQYKLRARQSRYATRKRRFSLHS---N-
----------------LHYAKGMA---------------------------------DPK-----------------P-------DFSAIP--
-----------------------------------HLS-DH--CY--SS--------------------------------------------
--------------------------
>KolobokP-3_DrPo_1p
---------------------------------------------------------------------------------------------
-------------------------------------------MP------------------------------------------------
--------------------------------RVSKQK-------A-C-L-----LK-------N-----------SYAK--------
GHTSVM-M----------GKSF-------------------------------------------------------GYE-SSVVQ---R-
SFVRLPGDDY-Q-DRISQ-LDE--------VLTFQDVD----GSSTSA----APLR----------PRSR--TR-S------RIEEYTNP---
```

---------------ES-G-KV-----HP-D---L------------L--------TNKIYRPLELGIMFNTEIRKH--I-EETS----
CHGFLMI--DTANSRNWGLGSSERLKCDS--CSYVSP---------H--YRLYEELE--T---G-----K-R-G---------RKPAK-----
----------------INVGLQTGLMTT---PISNTGMSRILAH-ANIAPPPQMYPRCIGLPVKCLRQWAL------------
NEKDMHDIREKI-----KQDNRLC----GL---KDGTKV----------------------
NVEGDTCYNNPLFNSGGHTPFQGGTIAVTTMCENNTRS--------------------------------------
KRIIGVHVANKLCMVASR----LR---NQG-IA-VDCP-------------------------------NHDG----K---C-
TANMSETGVIG-NEEKWNEQVARKIN--TD-------LNIASFTGDGDSKGHSGVDKA-------QV-Q-----QTVHFKDLRHLGNS--
LKRAINKAQ----FSSGMFAG--------PASKRANFQNRFALS--------------------------IRARCMSELTRAHKKYKG-----
-------------------NIKEIKNHMPK-------------VISSIILCYK-GYCGAY------CSKHSL-----------------
ACRG---SAGG---
KN--------K--AKL-YLPEN--------------CKLK-IAISDEALLKTCIQ-------------IVLGPESIDSTRLQTSTQKCEAVN-
------------------RAYQTAMPKTV---TF---
--------------------------SRNCTG-----------------------RIHSTILKLN--HGLA------DSAIVKS-E-----
FT-GAHLSKGSRVIAYL----------LKSKHNDMLKK---
TCAFQRRRKAARYLARKRRYALHS---E-----------------IHYSKGLT----------------------------------DPK----
-------------P-------DFSDIS--------------------------------------QLN-DH--SY--S---------------
----

>KolobokP-2_GarTel_1p

--M---
--------------------------------KKKTRN-------R-L-L-----RK-------I-----------GFKT--------
GTSNPSVK----------GKSR---NIF-TETEP---G-
QFIRTFKETF-D-SRIDEKENG--------SFGFVDVD----GQDTAV----RPLR----------PLKT--KP-K------LLDQYENK---
---------------SV-S-NL-----HP-D---L-----------F--------VNKLYCPFYVQGMFNSEIKKH-VQ-SNPT----
CNGDLVI--DVKHSMKWGLCWQERLVCSK--CTFTSG---------F--HKLYEEVE--S---K-----T-R-G---------RKSAK-----
----------------CNVGLQLGLMST---TISNTGACRVLAN-TNIIPPSRNSMRKQSNKVGEALV-NL------------
NNKSMRKVRQEL-----VEENRLC----GN---KNAKSV----------------------HIEGDSCYNNPIFNS-
ESTPFQAGTIVTSTFCENNSKS-------------------------------------KKIIGVFVGNKLCPIGSR----LK---NSG-QN-
VKCP------------------------------NHKG----K---C-TANVEESEPIG-NEEKWNSAVCSSIN--GD-------
IEIASFTGDGDSKGHKGVVKT-------VG-Y-----NIEQLKDLRHLGNS--VKRQINKTP----FSSNMFTG----------
AKRVNLKNRFAMS--------------------------VKARCIAELKRAHTVYRG-------------------------DLDKIAQKMPY-
------------VKNTILLCFN-GYCGQA------CSKHSL------------------VCGG-------------------------------
---N---YR--------R--EKY-FLPPN--------------
IKLR-MTESDEQLLLKSMD-------------ILLGTLNLPLTKRLSSTQKCEAVN-------------------RAYQSVNPKHS-------
---TF----------------------------PRNCTG----
-------------------RIHGQILKLN--MGSA------ESMLLKC-E-----SL-GAPLTKGSSVIKHL----------
AKLHARDLYRP---SKSSKARNRLRRYKTLARKYEMHA---S--------
---------IHYSKGLT----------------------------------DPR-----------------P-------DFSVHD---------
----------------------------HLQ-DH--KY--CT---

>KolobokP-6_DrPo_1p

---MG---
--------------------------------FLTKRK-------K-K-L-----LN-------T-----------AFKP--------
GGLSLN-K----------GRKY---HYERRSNSN---T-
TYVRLERSSF-E-ARVAY-NDN--------ILTFRDVD----GSKTPA----KPLR----------PRVN--RT-R------YVDMLRVP---
---------------AP-S-KV-----HP-D---L------------L--------TNKVYVPALLQSMMSSENRKH-RL-RSAK----
CKGDLVI--DGSASIKWGLGWKERLHCTR--CRYVGE---------H--YKLYNEVP--S---S-----T-R-G---------RKAAQ-----
----------------INVGSQIGVAST---SIGNTGFRRILNS-TNIIAPSPQAMQKQANKVNTALK-SL------------
NERSMCQIRKEL-----VAENAKI----GQ---KDPXLV----------------------NVEGDTCYNNPIFKS-
DATPFQAGTIAVSTMCENNTKK-------------------------------------KQIVGVLVANKLCRSSSM----LR---NKG-QS-
VQCP------------------------------NHRG----H---C-SANVSEREPIG-DEGKYNKVLGDLIS--QD-------
IKISNFTCDGDSRAFQGVRNS-------HG-H-----NVGHLRDLRHLGNS--LKRELNKAP----FSKGMLKG----------
HSKTNIRNRFALS--------------------------VKARCMAELKKAAHNKYQG------------------------DIKVLKKVMVD-
------------VISTIILCFN-GYCGTS------CAKYSY------------------VCAG-------------------------------
---T---NR--------Q--AKN-FMPNN--------------
VKVR-MVDSDQQVLKKCLE-------------MVLGPAALDATKLLTTTQKCEAAN-------------------RSYQAVNPKSV-------
---TF----------------------------SRNCVG----
-------------------RIHGQVHKLN--NSYA------DSVIAKT-M-----EL-HANLTQGSKVIKQL----------
AYEDRNELKRK---RLSATIKARALRARTRNYRYKLHE---E--------
---------LHYGKGIS----------------------------------DPK-----------------P-------DFDSLP---------
----------------------------HLK-HH--KY--A--

>KolobokP-1_BaPl_1p

---MG---
-----------------------------------RKRK-------Q-L-S-----KI-------F-----------GFKE--------
GNTPSN-R----------GKKL---EYE-QNSSS---E-
PFMRLKGGVF-E-SRVTQESDG--------VLTILDVD----QSPCPP----MLLR----------PRPK--SP-E------VLDDYLES---
-----------------T-VS-----DP-D---N------------H--------TYKHYVPSLVSVLWNSTIKEH-AI-ERNG----
CDGELEF--DSISSKKWGFAWKERLKCKK--CDFVGQ---------Y--HKLYYEVD--T---S-----G-P-G---------RKAAT-----
----------------INIGLQAGLMTT---PISNKSFRDITIN-CNIIPASLSSMQHLANKAGSVIV-KH------------
NRDDMKEIRETI-----VEENEMC----GF---ENPHLV----------------------NVESDARYNNPIYNS-
GVTPFQAGTQVVQTMCENNTKN-------------------------------------KKIVSAFLGNKLCNVASR----LR---NKG-IA-
VKCP------------------------------HHNG----H---C-TANLAEDAVIG-NETEYSRQCTTELN---D------
TLKIANITTDGDSKSFNGVNNA-------QG-K-----GATQLRDIRHLSNS--MKRAVQNCT----FSLSMFAG----------
KNKNNMKSRFAMD--------------------------LKARCVAELHQAFKAHKG------------------------QLIEVKKHMPN-

------------VIKTIVMCYK-GYCGIH------CQINSY-----------------VCAG-------------------------------
---LT-S---NH--------W--HKE-FIPGN--------------
TSLK-MTPDDEMSVEKCIG-------------VLLGSKSLELVRFLTSTQKCEAFN-------------------RTLQRCNPKNV-------
--TH-----------------------------SRNFPG----
-------------------RTHTAIHLRN--HWFG------NSTLLRT-K-----VL-GAALTPGSSVIKHL----------
KQEQYLEVYRS--KRKQLKESKQIRSMTRQRKYEMHA---AICYKSP--
---------IHYRKGIA---------------------------------DPK-----------------SS---------------------
----

>KolobokP-2_MiYe_1p

---MP--

-----------------------E--CQ---------
-----------------------------------H---
-------------------------------------HPPSYTGLQKTANHVGEKIV-TM------------SKASMESLREKI-----
VADNTTC----GL---EKPDLI----------------------NAEMDGRYNNPLFSG-DRTPYQGATQVTMTLCEQMTSD-----------
--------------------------KKILSVFTGSKLCKRAEY----LR---RCG-VQ-VTCP-----------------------------
-NHTG----I---C-TANVAEDAAIG-NEEAWATIVGEEIA---D------TLTINYLTTDGDSKTHTGMKKS-------HP-------
ACKNLKDPRHLVKS--VKREFWKST----FSADLFQK------F-RGLPYGTLKSRLATD--------------------------
VRNRCVTELERAHIVHKG------------------------NLDQIKKNMPM-------------TIEAMIQCYK-GNCAEM------
CAEFSY-----------------CCQG--
--------------LP-D---NH--------W--SKT-FLPQG--------------IVLC-LSAKDEHHFKQCVL-------------
KYLSTECLDSTKLLTSTQKCESFN-------------------RVLQKTNPKMM---------------------------------------
--------------------TC-----------------------------YRNFPA-----------------------RIHTAIHLCN--
FGIA------DSTVLRL-R-----AV-GSPLVSGSRVVSRI----------TNLRKRQLYHQ-------------------------------
---------------QRQRNIMFKLKRGYHRIRRYRMHA---KARVP------------TVTYKSGVD-------------------------
--------DPK--TIC-----------

>KolobokP-2_PeMa_1p

---------MQTALMGA---SVSNAAMIEICLT-ANIIPPSHTGLQNTANHVGEKIV-EM------------NTESMNNIRRNL-----
VEENRSL----GL---QDPECV----------------------NTEADGRYNNPLFSG-ERTPYQGATQVTYPICEQLSTK-----------
--------------------------KKILSVFTGNKLCRKAEI----LR---RQG-RH-IKCP-----------------------------
-DHEG----L---C-TANVPEEAAIG-NEETWATVVGEDIS---D------SLKVLHHTSDGDSKSFSGLRKS-------HP-------
GCASLKDTRHLAKG--IKKKMLSQK----FSPNMFRR------H-KGYTYGTLKSRLAND--------------------------
VKARCRAELIQAHKLHNG------------------------DMDKIQKHMPD-------------TIEAMLMCYK-GYCGDM------
CQRYSY-----------------CCKG--
--------------ER-Q---DH--------W--ERT-FLPEG--------------TVLS-MTEEDEKYFKVCVD-------------
TFLSTECLEQTKLLTDTQKTEAFN-------------------RKLQKTNPKSV---------------------------------------
--------------------TW-----------------------------YRNFPS-----------------------WVHTAVHMSN--
HGFA------DSTILRL-R-----SV-GSHITSGSSVVRRL----------VSVNRRQICHR-------------------------------
---------------LRQKKLAYKKRRGQLRIRRYLCHS---RNRAP------------VPTYRSGMD-------------------------
--------NPP--SVS-DH--TY--
SK--
>KolobokP-4_GiAe_1p

---MK--
-------------------------------PKKNPTE-------N-L-H-----NK-------G-----------AFQK--------
GHQCLF-KRTIG----VTKTKC--LKPEGDNI---P-
VTKRLSQEEF-N-AVVHHCDRQ--------SYIVKDAD----GIPSDI----RLLR----------PTRI--ST-GGG---TQAGEFLKA---
---------------------------KM-D---M------------N--------DSKIIHCGMLLKMFNDTNEDH-RL-ACPG----
CTGKYTW--NLDLCQKWGLCTRAGLRCQK--CSFTSK---------K--YKLYEETTQ-N---T-----R-R-G---------PKSAK-----
----------------PNVGVHVGLQST---SVGVTGFRGIMLC-AGVTPPATSGMQNTANEVGRVTT-SL------------
NKLDMSTRRADL-----VQLNINR----GF---SADHPI----------------------
AIEGDCRYNNPLSSGCGSTPFQPATQATYTMVENETGK-------------------------------------
KQIIGLYTGNKLCKAREL----QA---ARENRL-ILCP------------------------------DHTG----T---C-
TANLRLDDTIG-DEEHSAAQCSSDFS--SDV----SPLTIGFFTTDGDSRAVSGLQSS-------QS-KI-SKVKIKNLRDTRHLSES--
QRKFTERVK----FSDNMFPG------N-TKAEREKVKKRFAID--------------------------LRRRCHSEYTSAHNQLAG-----
-------------------NMNKLVSKLSY-------------TATSILYCVT-GKCGAT------CRKHSF-----------------
VCSG--VK-K---
GR--------S--NMT-FMPTG--------------TKLN-VTVEDEALIKQCID--------------IRLGVKAIEKTKLNTNTQKCESVN-
------------------RAYSRTNPKTV---TY---
--------------------------HRNFTS-----------------------RIHSAVHMLN--SGFS------SSVIQRC-T-----
AV-GAGVRDKTSVICHL----------KSIERIARRHA--
SRQRTQQFQARRASLRRKRYNMYD---RLHRT-------------ITYSKNLL---------------------------------DPE----
-------------L--NY--NSVGNKL----------

>KolobokP-7_GiAe_1p

```
----------------------------------------------------------------------------------
---------------------------------------MG-----------------------------------------
--------------------------KPPAKKKK----VLGRPP-N-----RK-------N-----------VYVI--------
GHKFHH-Q----------KEST-------------------------------------------------LLPNATKS---P-
TCTRLSREDF-D-AVAKPSTSRGAS-----SYVVTDSQ----GTTGNM----RLLR----------PHPA--VP-D------ALEDFLKQ---
----------------N--------NQ-D---L-----------D--------GYKLLHCGRVLNLFNESYREH-RN-NFPT----
CDGDLKY--DEQSYQQWGMCWRLGLKCEL--CHFRSK---------K--HKLYEEVTL-P---T-----A-R-G---------QKAAQ-----
--------------PNIGIHVGLMNT---SVGITGFRDSLSA-AGIPVGSKRGMQNSANYVGSKTV-YI-----------
NRQDMKERRSNL-----VRLNKLK----GF---TSDHKI---------------------
SVEGDCRYNNRLFGGNSSTPLQPATQAVYTIAENETPK-------------------------------
KEIIGIYTANKLCKKAEI----LR---SKG-EK-ITCP---------------------------DHGG----K---C-
TANLKTTDSIG-NEGLYAEKCVKEFQVDED------KLNIGYFTTDGDSHATDGATRA-------QT-G-----SIENLRDTRHFSKS--
QQKAIEKVS----FSLTMFPG------E-NKSDRQKVQRRFAID-------------------------IKQRCHLELDIAHRQLAG-----
------------------DITKIVRKMTY------------TIDSIVACVS-GNCSTL------CQRHSF-----------------
VCSG-----------------------------------------------------------------------RR-I---
KA--------W--KPA-YLPAD-------------SVLK-MKEGDKELLRKCLE-------------LRLGKTALLKTKLNTNTQKVESVN-
-----------------RAYTRTNPKQV-----------------------------------------------------TF---
-------------------------YRNFEA----------------------RIHSAAHMLN--KGIL------KSTVTRC-Q-----
LV-GAPLTGRIHALAEL----------KARERЕDRYHA-------------------------------------------
TRKKTTRYRSRRNYLRKVRYQQYD---DKHNSTA----------RTTYKKNVL---------------------------------DSE----
-------------V-------------------------------------------VPP-CLT-DH--TY--LSPRRKKQS--------
------------------L---------------------------------
>KolobokP-5_MoPh_1p
----------------------------------------------------------------------------------
---------------------------------------MP-----------------------------------------
--------------------------------KKGR-------Q-K-K-----RV-------Y-----------FFED--------
GHPXYK-KRT--P-------------------------------------------------------------SDEAVLD---V-
QSQRLPSNTF-Q-DAVQISADGN-------HFTVTDSD----GNPGNM----RFFR----------PIPE--DT-N---------EDHLDL---
------------------E-ND-----RT-D---L-----------Y--------GYKILHCGKLMDMINDFYSEH-LK-NSPR----
CKPLLRF--NPQDCTKWGFCWRIALKCEN--CNFSTE---------K--KKLYEESE--K---Q-----TPR-G---------QRYST-----
--------------VNLGIHVGLQSN---SIGIDGLRTLMLS-AGIPVPSCSGMHKAGIYVSDTTT-KL------------
NESDMQKRREKL-----TDLHKIR----GF---DASHPT---------------------
SVSGDCRYNNRLDSSVGHKLGQAGTQSVYCISENETKS-------------------------------
KDIISVNIDNKICVTGTR----LN----------ARCP---------------------------DHPG----I---C-
TANLDKDAVIG-DEGRSAERCALTMA--QDR----VPVIVGQFTSDGDSAASTGFERG-------QK-TH-SNVPVENLRDSRHFGES--
QRKVLKNIK----FSKTMFPG------R-NKATRDKVQQRFSIE--------------------------VTKRCNAEKNLAHKQYAG-----
------------------DINKIIRKLSY------------TVDSVLQCVT-GDCGQA------CKKHSF-----------------
VCSG-----------------------------------------------------------------------MK-K---
TA--------W--KPK-FLETG-------------TIFN-PTDNDLRLLRDCIV-------------LRLGAKGIRKTRLNTNTQKNESIN-
-----------------RTFTKTNPKST-----------------------------------------------------NY---
-------------------------CRTLSG----------------------RIHGAVHMRN--NGRS------NSICERL-R-----
AV-GSSVDSSTLVLRQL----------KNIQSKDTYHK-------------------------------------------
ARKNSTKYKARQSYLRRVRFNKSAV--EPHNT-------------GTYRKNIL---------------------------------DPE----
------------M---------------------------------------------N-DHTSLY--AKKNRRYRMKVE-----
----------------------------------------------------
>KolobokP-8_CGi_1p
----------------------------------------------------------------------------------
---------------------------------------M------------------------------------------
----------------------------------KGQ-------S-K-S-----KS-------A-----------
QFKAKDGRRRGPGHRGWG-K----------KRQP------------------------------------------------
--------S-KYLRP------Q-TRTQISKTG--------KCILKDVASAI-ETTGPI----MQLR----------PRTA--CT-V------
PEKD--------------------------N-PS-----KQ-E---Q-----------N--------SYRMLHMGKTCEMFNTAYQQH-KL-
QSPK----CPTYLEF--DFDAEQQKGVCWKETLKCRY--CNFRSG---------Q--NNLYEEMQ--T---D-----S-R-G---------
PKTAK---------------------PNISLWVALMDN---PIMGTSLQEIFLA-LNCPAPSYTGLQRNGGKVGPRMV-
EMVKKTGWSQDGGDGKEDLRRERTYL-----KDTLENC----GY---PRLTPI---------------------
PVEGDGRYNNPLYWSRDRNPFQPATQSTYTISENVTSD--------------------------------KKIIGVTAKNKLCRKR--
---------TKG----SRCP------------------------------EHPG----K---C-SASLTMDAPIG-CEHLATEEICHDFL--
TDR----EPTLISHMTTDGDSAAFRGVQKA-------MR-E--HGQTVEALRDTRHLAQS--QKKAADNAK----FSQNMFPG------R-
TATERQATKRKFSVD-------------------------LMKRCTAEYDIAQKKFCG-----------------------
DSEKLIHTLSF-------------ATDAIVECYS-GRCGGT------CTEHSL-----------------VCSG------------------
----------------------------------------------------LP-T---DC--------W--PKE-YLPPNS--
-----------RTLN-PTLEDEETIRRLVD------------FRFSRSTITTTRYGTNTQKSEALH------------------
RGYSKSNPKNV------------------------------------------------------TC-------------------
--------AVSFEP-----------------------QIYSAIHRMN--HGPG------KSTVLKC-A-----AL-GAPLPEDTRVTRQL---
-------ERKQEIYITDK-------------------------------------------KRKKSSVYRTRRRAFVKEKFEMYF---
QKKTGT-----------EAGYSKGMS-------------------------------------DPP------------------I-------
PKSVTK--------------------------------------NKS-EH--SY--NKQQK---------------------------------
--------------------------------
>KolobokP-1_CVi_1p
----------------------------------------------------------------------------------
---------------------------------------M------------------------------------------
----------------------------------RGK-------S-K-S-----KR-------G-----------
IFAPGDGRPRGSGHAGWL-K----------MQKK------------------------------------------------
--------S-KYIRG------E-AVKKVSKMG--------ASILRDHA----RSEGTT----MQLR----------PRSI--ST-Q------
SXLT--------------------------H-NP-----QT-E---S-----------N--------SYRFFHAGKVCELFNLAYHQH-LL-
TSPN----CPTRLEF--DYTREQQVGVCWTETLKCTY--CHFRSE---------K--TKLYEEAS--T---G-----K-R-G---------
KKPAK---------------------PNLALWTALMDN---PIMSKGLQEIFQA-LNCPAPSTTGLQHNANKVGPMIV-QM-----------
```

VQEDLKRERAHL-----KDIVEGC----GY---PRDTPI----------------------
PVEGDGRYNNPLYWSRDRNPFQPATQASYTISENITEE-------------------------------------KKIIGCTTRNKLCSKR--
---------NRG----ENCT------------------------------EHPG----E---C-AATLPYQHPIG-REDQAVEEICREFL--
SDP----EPTFISHITTDGDSTAFRGAQRA-------MA-E--REQTVEALRDTRHLAQS--QKKAADNAK----FSLQMFPG------R-
TAADRQATKRKFSVD--------------------------FMKRCTAEYDQAIKKYRG------------------------
DTEKLVNTLSF-------------ATDAIIDCYS-GRCGRT------CAQHSL-----------------VCSG-------------------
---AP-E---KC--------W--PKE-YLPPQS--
-----------RTLH-PTDDDEDVLRQLIN-------------FRFSRATLTSTRYGTNTQKSEATH-------------------
RGYSKSNPKNV---TC---------------------
--------ARNFQP-----------------------QIFSAIHRIN--HGPG------KSAAMKC-A-----AL-GAPITEGTRVCRQL---
-------QRKQKKHEQDR--ARKRSSKYRAKRQTLVKQKFQMYF---
QRAENET----------QAGYLKGMS---------------------------------NPP-----------------L-------PDSKK-
-------------------------------------QTG-EH--SY--SKMKKVK-------------------------------------
----
>KolobokP-4_MyEd_1p

---MT--
--------------------------------KFRKGN-------N------------------S-----------GFPT--------
GTVPYN-R----------QRKT--VKPSKSDTTQ---T-
VYKRLTRKMT-S-LVQNI--------------PYKDTDTQCVNDTKPA----VLLR----------PRDT--AP--------KIPKTKKS---
---------------AK-K-NQ-----RS-E---D------------S--------SNRTVNVRLNAIMWNSVFREH-QR-LHPN----
CNGFLQW--NMEREEKFGFVNREEAMCDK--CNYQSR---------K--FKLYEEVQ--T---K-----K-P-G---------RKAAK-----
----------------INVSAQAALSQT---PLGYTGLRKIVLG-CNMPAPSASGLQKRANKVLPEII-NI------------
NKEDMKARRKQL-----IEINTLR----GR---KDPGSV----------------------
SLQADGAYNNAIYSGIGKTPFQPATQVVYSVAEAETED-------------------------------------
KSIIGVVCKNKLCSIHPI----KS---G------EKCT---------------------------------S----S---C-
SSNLTFMKSIG-DEYTWAKEALQDLA--SD------GIEAKHLTTDPDSSAYRAADDL-------YL-ENTTSTEPEHFLDTRHFLNN--
HRKNIKNNK----ELGEIMPG------R-TKKDREKLLNNFALD--------------------------LAERCQSEFTQAMEKYGG-----
-------------------DFTKVKNKISF-------------TVDAIPACYT-GNHEL-------CRRHSF-----------------
VCKG--GK-K----
F--------WLSNRA-FLPNS--------------FKIR-KLDENLNAIRKCVL-------------YRLSPSALKKTRLNLNTQKVEGFN--
-----------------RSLRRSLPKNV---TY----
-------------------------TKNFEG-----------------------RVHSAIHSVN--LGPG------ESLLVIC-K-----QL-
GAEISPGSAAEKEL----------KAIQKTDRMQK--
AYKKSMKYKKERSNTRKHLYKIYE---KHQEE-------------KCYEKNKL---------------------------------MRP----
-------------V-------KRTKTQAE---------------------------------------DH--PY--AKKPR------------
VAVTKKC--
>KolobokP-1_PeVi_1p

---MV--
--SF---------------

---LIFRL---
-----------------------N---S------------D--------SHRVLCLYKSENMWNCAFREH-MM-KSPD----CDGILLY--
DLDKEEKRGFSSRFRLICAS--CKYFSK---------M--YTQYEELE--T---G-----R-P-G---------RKPSK--------------
-------LTTGVLVGLSQT---PIGPTSLRKIFLS-GNISTPAASSLQRRTNQVYKNIV-QV------------VKKDMKRRREEL-----
VEINRLR----GK---KNPRCI----------------------SVQMDGMYSNRPYSGIGRTPFQPANQVVYTSAENVTKK-----------
--------------------------HNIVNLVDKNKLCSEHSS----LDVDFDSGQLH-SECT-----------------------------
--------DE---C-SANIPMVKSIG-DEYAWARECLEDLL--KD------NIEVDYLVTDPDSSAYRAAVDL-------YE-
EGKSKTEPQHFIDTRHLSEN--MRKSVKKDK----SLLKIMPA------R-TQKQRQKLLNNFATD--------------------------
LTERCNSELAVCTKQFGG------------------------NFKRVKSKMSH-------------TVDAVIRCYM-AKHDL-------
CKKYSF-----------------VCGK--
---------------------SE--------WLKRRS-LLPKT--------------FKIM-HTHENMDFIRIAVN-------------
KRLGPKALEKTRLNMNTNFVEGVN-------------------RSIRRSLPSNV---------------------------------------
--------------------TY-----------------------------KKNYSG-----------------------RAHSAIHSVN--
LGPG------ESILELC-S-----AL-HCDIPVGSTAYQAL----------KSVQKLDILQK-------------------------------
---------------QHKQTMEYKKYRSEKREKLYKLYE---KLSEI-------------IAYEKNLL-------------------------
--------LKS-----------------E-------KEQKPSSRN----------------------------------KHT-DH--SY--
SVKSKKRR---------ISVRK--
>KolobokP-3_MyGa_1p

--MEEMWNAAFKEH-QL-RNPQ----CEGILLC--
DLENEEKRGFGTRQQLICTK--CDYKSK---------R--YTLYEELE--S---G-----K-P-G---------RKASK--------------
-------LDTAIHVGLSQS---PIAYSGIQKIFLS-GNILAPTTSSLQRRANTVMKQIE-QI------------NKQDMKRRRNDI-----
VEINKLR----GK---ENPHAI----------------------SVQMDGMYNNPLYSGVGRTPFQPATQTIYTAAENETSK-----------
--------------------------HNILALNIKNKLCSKHSS----LDVDNDSGRLH-EDCT-----------------------------
--------DD---C-SANIPMVKSIG-DEYTWARECLLDLK--ED------QLEIEHLVTDADSSAYKAAMDL-------HN-
EGINTVEPENFLDTRHLSDH--VRKGAKSDK----TLLKVMPA------T-TKLKRQKLLNNFSVD--------------------------
LTERCNRELLLAYKFYAG------------------------DFCKVKNKISH-------------TVDAIAYCYM-GDHAR-------
CRKNSF-----------------ACKG--
--------------FQ-----GS--------WLKGRP-FLPNS--------------FKIS-SCNENLDSLRKQIN-------------
KRLGSKVLEKTRLNMNTNFVEGFN-------------------RCLRRII---
--------------------TF-----------------------------KCYFQE------------------------------------

----

--KH--VW--
SCTCSSSQR---
>KolobokP-2_MaMa_1p

--MFSS-------------

--IHINR--
--------------------SG-E---E------------N--------TYRLVHTGKNAQMWNTMFHEH-QF-KHPQ----CNGFLSW--
DLRHEEKRGLVWRERAICST--CSYASK---------M--FNLYDEVQK-T---S-----T-K-G---------RKAAC--------------
-------VNSSIQIGLSQT---SIGNESLRTILLS-ANIPAPSTKGMQKSANSVCKEIE-GT------------NIRDMAEIRKGV-----
KEFNFMR----G----NDTNII----------------------DMECDSTYNNAIYSGVGRTPFQPATQCTYIQVEDTTPE-----------
--------------------------KKVINIITKNKLCTFGKM----HT---SDD-DA-KLC------------------------------
---------H---C-SANISLQRNIG-DEYSWCKEGLMYLK-KSD------NLEVHNITTDPDTASFRASLDL-------RR-
EGVTKTVPENHIDTRHLGQN--HTKFIKKQS----KLIDLMPG------R-TKKRREKMRNNFAHD--------------------------
LSKRCQAEFEKSFQKCGK------------------------CTPKLISNLSY-------------ATDAIVKCYQ-GDHSL-------
CYKQSN-----------------VCWV--
--------------DG-C----N--------WVYRST-FLPSY--------------FKLKISSESEESTLRMCIE-------------
YRLGKSTVLKTKLNKNTQKVESVN-------------------RRIKRSLPKNI---------------------------------------
--------------------TF-----------------------------KRNFHG-----------------------RAHSAVHNCN--
HGPG------ESLVKLC-K-----SI-GCPVSACSSVAQHL----------KTKQDVYLRDK-------------------------------
---------------MYKSSKVYKKKRSEKRAFLYKLYE---KYQEQ-------------KNYDKGKL-------------------------
--------LCA-----------------M-------ARKIKEKY---------------------------------------EH--SY--
HKPPP-------------------------------F-----------------------------------
>KolobokP-4_MaMa_1p

--MKRH------PP---------------
--N-----N-P-G---------RKAAM----------------
-----INKGIQIGLSQT---SIGNSSLRSILLS-ANVPAPSKSGMQKASNSAAHAIE-AE------------NRKDMDKIREEI-----
KEVNLLR----G----NTAGII----------------------DIQCDGMYNNAMYSGVGKTPFRPATQCSYIQVENITHT-----------
--------------------------NKVINVVTKNKLCNYGKF----HT---SQE-EV-DNCT-----------------------------
-------------C-SANITVQRDIG-DEKSWSKEGLLELK-LQS------DMEVKNMTTDPDSSSYRAAVEL-------HV-
AGTTNTFPKNFIDTRHLGQN--HRKFIKKSN----VLTNLMPG------K-NKVERERMKNNFSID--------------------------
LSKRCHAEFNAAFEKYCG------------------------DTSMIMRAMSR-------------AVDAIVLCYQ-GDHVL-------
CKTKSY-----------------VCTC--
--------------EN-A----N--------WVVKSS-YLPGT--------------FRIV-KSTHTESMIRMLVN-------------
YRLGKMTIEKTYLNTNTQKSECVN-------------------KVIRRSLPRNV---------------------------------------
--------------------TF-----------------------------KRNFHA-----------------------RCHSAVHYSN--
NGPG------ESIVKLC-Q-----AS-GCPIAANSSVAMQL----------KRRQKEHIIDKT------------------------------
---------------FYKKKKAYKHNKCKKRALLFRLYE---KYQEE-------------RNYAKGRL-------------------------
--------LCN-----------------I-------GKKLRAKY---------------------------------------DK--SV--
KDLP--
>KolobokP-4_MeNe_1p

---MWNTVFREH-AQ-ISPK----CLGFLQW--
DQLSEEKWGLGWRERAICNR--CIYKSE---------M--FNLFEEIY--N---Q-----N-P-G---------RKAAN--------------
-------INRGLQVGLTQV---SMGNAGLRKLLLS-ASIPAPSTKGMQKVSNKICKEII-QE------------NILDMRSRRQNL-----
REINIAR----G----NPPDII----------------------DVQGDGSYNNPLYSGVGKTPFQPATQVCYLQAENVTSK-----------
--------------------------NDIIALTTKNKLCSHHIQ----HQ---SDE----LNMT-----------------------------
-NKTC----D---C-TANISMETNIG-DEETWASECLLNLK--ED------NFEVRNITTDPDTSSYRAAVNL-------YN-
EGLTQTIPKNFIDTRHFSEN--HRKYIKRCP----NLVHMMPG------L-TKAARQKMRDRFAID--------------------------
LTQRCQVEFENAFNKLNG------------------------DTDRVKGCLSY-------------SVDAIVQCYQ-GDHSL-------
CTDNSY-----------------ACKG--
----------------------N--------WLEKSP-YLPFS--------------FKVH-VTKQSEGILRDCIN-------------
YRLGPLNLEKTKFNTNTQKVEAAN-------------------RVLRRSLPRNI---------------------------------------
--------------------TW-----------------------------TRNFPG-----------------------RAHSAVHSLN--
NGPG------ESILKLC-N-----AV-GCSISSGTRVAQTL----------AQEQKLFTRYK-------------------------------
---------------AYKKSKAFKRRRCEKRTYLYKLYE---EFQEK-------------KKLQERVI-------------------------
--------VAK-------------------------------------C--

>KolobokP-5_MyEd_1p

---MWNEVFIEH-RQ-ISPM----CTGFISW--

```
DLSAEQQRGAAWREKASCNE--CSYHSK---------M--FNLYNEVI--A---K-----K-R-G---------RRTAA--------------
-------INLSIQVALNHI---AISTTGLQKLFLG-SNIPAPSTSSMQHSANVVSEIIE-EY-----------NQKDLVQKRKLI-----
KEINILR----G----DNPNII----------------------NIQADGMYNNPIYSGMGKTPFQPATQCTYNMLENNTYK-----------
--------------------------HSIVSTRQVSKLCSNKSE----HK---TKT-----KVK-----------------------------
-SHKG----H---C-SANIEMHDSIG-SEERWAKDCLLDLK--ND------GLEVQEITTDPDSSAFRAAESL-------FK-
AGTTNTQPIHFLDTRHVSAN--HRNFIKKMS----GLKEIMPA------R-LVSEKDKMLKRFALD--------------------------
MAARCQAEYNAAFDKFNM------------------------DSVRVKVHLSY-------------VCHAIVSCYQ-GDHNG-------
CTKYSL-----------------VCSN------------------------------------------------------------------
--------------RN-S--LKT--------WTEKSA-YLKNN--------------FKLN-KSENTAALLRECVK-------------
YRLGPTMLNRTCKNTNTQKAEATN-------------------RAIRATVPSNV---------------------------------------
--------------------TF-----------------------------TRNYKG-----------------------RVHTAIHNVN--
NGPG------ESIVKLC-K-----AA-GVPIEPGSRAARGL----------KNIQQHNEKHK-------------------------------
---------------LYKQSKQYTDQRCSKKHELYKIYD---EYQEE-------------KDYEKNKL-------------------------
--------LPT-----------------K-------RLAAKQPQ---------------------------------------EY--SF--
AKKLR------------SRHVKNK------------------------------------------------
>KolobokP-1_PiIm_1p
---------------------------------------------------------------------------------------------
---------------------------------------------------------------------------------------------
---------------------------------------------------------------------------------------------
---------------------------------------------------------------------------------------------
---------------------------------------------------------------------------------------------
-------------------------------------------------------------MWYNVYKEH-SQ-TSPN----CDGMLVW--
HLASEERRGLGCREVAKCTK--CKYMSS---------M--HTLYTEVQ--T---P-----S-Q-G---------RKCAD--------------
-------LNLGLQVGLCLS---PIGPTSLRRICLS-TNMPAPSSKGLQLAANKVGKAIV-EE-----------NKKDMRRRSQRL-----
KQINILR----GI---QTPENI----------------------HVQSDAVYNNPLYSGVGKTPFQPATQVVYTVAENETKK-----------
--------------------------HEIISLVTKNKLCSKNKH----LD---KDH----DTCS-----------------------------
----P----S---C-SANIPFEHSIG-DEYSWAKEAYKDLE--KE------GLHVSYLCTDPDSSAFKAAEDL-------AI-
ESNHLFQVQHFIDTRHMSDN--HRKNIKKNE----NLMKIVNG------R-TKAEKKKVVNNLAID--------------------------
IADRCQAEINQAYKSHTG------------------------DVQIMQRKLTY-------------ATDAIIQCYQ-GCHAL-------
CRKKSF-----------------VCKG------------------------------------------------------------------
--------------GK-T---NN--------WLLKSN-FLGES--------------FTLL-RGKNTNEILSDCLK-------------
FRFSLAALKKTIIIANTQKVEGFN-------------------RSIRRSLPKTS---------------------------------------
--------------------TF-----------------------------CKNFTG-----------------------RAHAAAYAVN--
NGEG------DAISNLC-N-----AV-GCIIPKGGIVSKAL----------QKNQELDQMRK-------------------------------
---------------ANMKKIEFKRRKCQKKRKLFKLYE---EASEK-------------AEYEKGKL-------------------------
--------LKS-----------------L-------QIK----------------------------------------DYS-DH--TY--
SKKKK------------VNHNR--------------------------------------------------
>KolobokP-2_GiAe_1p
---------------------------------------------------------------------------------------------
-------------------------------------------MG------------------------------------------------
-----------------------------------------------------------------------------FYL--------G----
---------------------------------------------------------------------------------------------
---------------------------------------------FINR--------------------------------------------
-----------S-----------E---A-----------D--------TYRVLHMGKLEQMMNRTHNDH-RT-LAPE----CHGQLMW--
DFPAEERRGVCTRQRLCCNI--CTYHSG---------M--YNLYTEVD--NPERK-----G-R-G---------RKAAQ--------------
-------PNVSVQIGLTKM---PIGVQNFRLLCLC-MNIPAPCVAGCQKTANKVCDTII-NS-----------NKTDMQARRNNL-----
KTINTIR----G----CQPNAV----------------------NLQCDGMYNNPLYSGVGKTPFQPATQVVYTATEHNTSQ-----------
--------------------------KQVLAIATKNKLCSKRHG----IS----QS----HTCL-----------------------------
--NDP----T---C-SRNIPMTMSIG-DEKTWAKDCLTDLK--ND------GLDVHYMTTDPDAKGFKGAEEL-------YQ-
QNVTDIEPQMLIDTRHVSEN--HRKFVKGAK----FVENMMPG------R-TISDKKKLQIKFSND--------------------------
IAFRCQAEYEQAYRIHTG------------------------NIPKFMHALSY-------------TSDAIAACYQ-GNHKK-------
CMKHSF-----------------VCKS------------------------------------------------------------------
--------------AS-K----N--------WLVKST-FLNSH--------------FKI---ATNNDTDLRKCID-------------
YRLGPKMLQMTKFNTNTQKSEAVN-------------------KSIRKSLPRHT---------------------------------------
--------------------TF-----------------------------SRNFSG-----------------------RAHSAVHSIN--
NGPV------ESLLKLS-R-----DM-GCSFSSGSKVISAL----------HKVEHNRTLQL-------------------------------
---------------NRKKNNAI----------------------------------------------------------------------
---------------------------------------------------------------------------------------------
----------------------------------------------------------------------

>KolobokP-5_GiAe_1p
---------------------------------------------------------------------------------------------
---------------------------------------------------------------------------------------------
---------------------------------------------------------------------------------------------
---------------------------------------------------------------------------------------------
---------------------------------------------------------------------------------------------
-------------------------------------------------------MGRLEELINTSQVKH-AD-SSPN----CCGVFRF--
DFDREERRGICTRLNVFCEK--CSYLSN---------T--FTLYDEVE--KTEVR-----S-R-G---------RRAAQ--------------
-------PNVTVQVGLTKM---PIGAENFRLLCLC-MNIPAPSASGCQRTANKVCKNIV-TS-----------NTADMKARRETL-----
RDINKLR----G----SKPNTV----------------------NLQCDGMYNNPLYSGVGKTPYQPATQVVYTAVEHNTHK-----------
--------------------------KQVLAISTKNKLCSKHHN----IS----TS----ETCL-----------------------------
--QDP----T---C-SRNIPMSMSIG-DEKKWAKDCLAQLK--ND------SLEVQYMTTDPDAKGFKGALEM-------YN-
ENNSEIKPQMLLDTRHLSEN--HRKFVKNSK----FVENMMPG------R-TVSNRKKMQIRFSND--------------------------
IAMRCQAEFEQAYKQHAG------------------------NTKKIMHALRY-------------ISDAIAACYQ-GVHNK-------
CKRHSF-----------------VCKK------------------------------------------------------------------
-----------------K----N--------WLAKSS-FLNSD--------------FKIP-KSIQNNXNLRKCID-------------
YRLGPKMLHLTKFNTNTQKCEAVN-------------------KCIRKSLPRHT---------------------------------------
--------------------TF-----------------------------SRNFAA-----------------------RAHSAVHTIN--
```

NGPA------ESLLLLS-K-----DM-GCTFSAGSKVVADL----------SKVETLRTQQL-------------------------------
---------------NRKKKNTYKIKRCEKRKALYEMYE---RHQND-------------ELYEKNSL-------------------------
--------MRN-----------------L-------VPKTAQNSTKKKCN------------NKK----------------------------
----
>KolobokP-2_HaRuf_1p

---M---

--CKGDIVM--
DIDNEQQWGLNWIERLKCTD--CTYLSE---------Y--HKLYKEVE--G---S---GMR-R-G---------RRSST--------------
-------ANTGLQIGLSKC---PIGSESFRVLCMS-ADIPPPSHSGMQKAANRVCDNIK-SQ------------NTKDMRARRVEI-----
REINKYR----G----TNLNNI----------------------SLSTDAMYNNPLCSGVGKTPFQPATQVTYVATENQTSD-----------
--------------------------HQILALNTKSKLCSKHSL----VD---N------ISCV-----------------------------
--NNP----D---C-TSDLHMSESIG-NEKQLAAECFRDLK--KD------QIEVEFLTTDADSAAHTALSEM-------YE-
AGDMDVSPQTFLDTRHLSAS--HRKFIKNSK----FVVDMMPA------R-TKKDKQSLANTFSLD--------------------------
CASRCAAEFSAAYSSHAG------------------------NLTKLKTSLSC-------------TSDAIANCYM-GDHRK-------
CRRQSF-----------------VCKG--
--------------EK-K----N--------WLLQSK-YLDKN--------------FKIG-NNETNFQNLRKCTD-------------
FRLGPNMIEKTQFNLNSQKCEATN-------------------KALRRSLPRDT---------------------------------------
--------------------TF-----------------------------ARNFQG-----------------------RAHSAVHSVNC-
QSPV------KSIRNLR-L-----GL-GCAVTAKGKIDDEI----------SKSHNKVLKAK-------------------------------
---------------TYKKSQKSKDRRARKKLELFKLHHC-------R-----------KVHYSKDML-------------------------
--------LKR---------------------------------N--AY--
LRNLHPK---
>KolobokP-2_CySi_1p

---MD-------------------------------FC---------------
--SFSE-------------

---GYRTFHTGKLWEMFNKVTAEH-YN-HAGR---VCHGNLGF--
VKEAEQKWGFGWREKVQCDL--CSYSSG---------M--FNLYEEVK--N---Q-----K-A-G---------RKAAT--------------
-------INMGINIAMTQT---PTGPTSIRKIFHG-GNIPAPSRAGMQKTARKVSKILV-QA------------NMDDMKERREKL-----
KRIKRLR----N----SPEHEI----------------------AVQSDGIYNNALNSAATRNPFQPATQVVYTVAENETLQ-----------
--------------------------HEILAVETVNKCCSKYGF----HE---QED----EECD-----------------------------
-MKCA----P---C-SSTACMEVNIG-DEQRWAKYAFESLK--ED------GLEVKYITTDPDTSAHRALEEM-------YS-
SGVTKTKPEYQVDTRHLSRN--HGKFVRQSE----KVLQMMPA------R-LKSVRVKLRARFALD--------------------------
ISHRCHAEIQKIHLEERG------------------------IFSKISARVAK-------------CIDVMVKCYG-GDHGC-------
CAANST-----------------MCHG--
--------------TS-S---DN--------WLVKNV-YLQSN--------------FKID-ISK-HEETLRDCIN-------------
YRLGPERLKLTRLNTNSQKVEGTN-------------------RAIKRSLPKDV---------------------------------------
--------------------TY-----------------------------TRNFEG-----------------------RAHSAIHSVN--
NGPG------QSLITLL-D-----KA-GCSIPRGGQVCRAL----------ASEQRFSEAKK-------------------------------
---------------RREKSIDSKCKRKMRRLKLFNLYR---RHQEK-------------RNYIKGLL-------------------------
--------LRS-----------------K-------QSTKRARKVLTR--------------TPQA----------SR-SSI-DH--NY--
SK--
>KolobokP-6_CorFlu_1p

---MG--
-------------------KIRK-------GRAIRKGL-------Q-A-R-----KA-------T-----------QFKK--------
GHISPM-K----------GVNM---EREAFPDEK---V-
VYLRPSSSEY-A-AATSEPIETE-------ALALDDPSETTTATSTKY----KLLR----------PTKDK-KP--------TTDDVDEEF--
------------------S--------NL-EWAIK------------E--------GYRTFHKNKVMDMMNEVFREH-NNYGAAP----
CPGALQF--DTSEEIKWGFAWFEKAKCEK--CHYSSK---------V--YKLFEEVK--T---G-----K-R-G---------RRAAT-----
----------------INVGISIAMTQT---PIGPTSLRHIFHG-GNIPAPSRSGMQQTAKRVASTII-KT------------
NKADMKCRTEKT-----KRILRMR----N----RPDNEF----------------------
AVQSDSVYNNNLFSAIGKSPFQAGTQAVYTVAENVTCN-------------------------------------
HDIIEIETVNKLCSKEGY----HD---SNS-M--GKCN------------------------------ILHS----S---C-
SATHPMEDNIG-DEKRWAKSTFQRLK--EQ------AVEVKYLTTDPDTAAFKAVEEM-------HR-SGETTTKPIHQIDTRHLSKN--
HRKFIRNSE----KVLKMMPG------H-TVKLRKEQRNGFAFD--------------------------LSQRCYAEINQIHQQQKG-----
-------------------QFDKIRMKVEK-------------CIQAMLKCYA-GNHSD-------CKENSA-----------------
VCKG--ES-N---
NN--------WFCPSTIYLNAS--------------FKIN--TDIHKQTFLDCVN-------------YRLGPERLILTRLNTNSQKVEGTN-
------------------RAIKRSLPPNV---TF---
--------------------------TRCFES-----------------------RAHSAIHLVN--NGPG------RSLAKLL-V-----
SA-GCPICPTGKVARAL----------NYEQLGTEKKK--
DREKTLEYKQKRKLRKVKIYNLYK---ARKEI-------------KNYQRGML---------------------------------LHM----
-------------K-------AAKRSATNILKKC---------------------------KK-PRD-DH--SY--SRKPPTDNKATFNMQ--
-------------------------AAAPGQEQDPDLKHSCL-------------
>KolobokP-8_CorFlu_1p
MS---
---MALKRELQHCDTIYKGALKLFYLDNSSYHRNAPNCP--------
FLQPQYHLDAILS-------EKQFLKAKR-------KMGRNKGF----F----K-R-----RR-------T-----------TFKH-------
-GNSATI-R----------RVQD--CISNAEETEP---V-
KYVRLDAEQQ-A-MVENNPILPA-------AVAKHSASD---KMVPTF----KFLR----------PCRA--NP-A-----DVKPKLSKQ---

```
------------------Q-IQ-----SI-E---A------------D--------SYRVLHCGQLWTMINEVTREH-LD-RNPR----
CKGNLSF--DFENEMQWELCWRESVICDK--CQYKSK---------I--YNLYTEVE--T---G-----R-P-G---------RKAAT-----
----------------ANVGLHIAMSQT---PVGPTSVRKLLLA-SNIPAPCISGMQKTSNKVNKLIE-NT-----------
NKEDMQQRRENL-----SAINQLR----G----APQNEI----------------------
AIQSDGVYNNSLWSGVTKTPYQPATQMAYIVAENVTGK------------------------------------
HQIINVELVNKICSKHGY----HT---MED----DECD------------------------------IKSG----E---C-
SATASMETSIG-DEKRCAKLVLEGLL--ED------GLSVKYITTDQDTAAYLAATEL-------YH-ENKTETEPEHQIDTRHLACN--
HRKQIKNSS----DVQAMMPG------N-TIQYRQYLQGRFAID-------------------------ISKRCHKEYATIFKEEAG-----
-------------------DFKALSDRINL-------------AIGAIKRCYG-GDHSR-------CQRFST-----------------
ACKG---------------------------------------------------------------------------------ES-T---
NN--------WILRSA-YLPRN--------------FKINIQNEHNQEVLTQCIE-------------YRLGQPILEKTRLNTNSQKCEATN-
-------------------RSVRRSLPKNT---------------------------------------------------------------LF---
---------------------------GRNFPG----------------------RAHSAIHSVN--NGPG------DSIKKLC-L-----
RA-GCPIPSGSKVAQRL----------QKEQQLSEKYK------------------------------------------------
ERARSITAKSKRIARANKLYKLHE---KXREK-------------NRYIRAQI----------------------------------SKQ----
-------------L-------TVKNKKKTDVSKCN------------TPRP-------------STS-DH--HY--NRLSSQRKKIQGGL---
----------------------------------------------------------
>KolobokP-9_CGi_1p
----------------------------------------------------------------------------------------------------
---------------------------------------------IE-----------------------------------------------------
---------------------------------TKTRSLGCL------K-L-----KL-------H-----------QFEI--------VHV--
--K-----------------------------------------------------------------------------------P-VSLR---
----EVTLITA------------LMLCILVT----VSSKQI----IVTL---------PRLA-----------------------------
-----------------------D---Q------------N--------SHRLLHLGKTAEMWNEVLRLH-QQ-ETPE----CTGLLSW--
DLASEERRGLGSRMGLRCTK--CTYSSR---------R--YNLYEEVD--T---K-----S-P-G---------RKAAK-------------
-------INYGLQVGLSQT---PVGNDGMRKILLC-TNTPPPARRSLQKLPIKFC-------------------------------------
--------------PIPHAI-----------------------PVQCDGMYNNPLYSGVGETPFQPATQTVYSFAENVTSK-------------
-------------------------HQIIKMVTKNKICSKHGH----LL---DEGSRD-HQCN-------------------------------
-PG----V---C-GANLPMHHTIG-DEFTWAREGMAELL-CED------GLEVREVTTDPDSSAGRAADSL-------YK-
DGLLENKPCHYIDTRHLSES--IRKAIKRDQ----KLLEIMPK------R-TKAEKTKLLHNFALD---------------------------
AVDRCTAEINQAHALYAG------------------------NADKIKNKLSY-------------TKHAVVKCYT-GDHAL-------
CKNTRKTSSLTPNMQW-----LSVTQGIMP--------------------------Y-----AKTLVSVQ----------------------
--------------RRVN---NN--------WVRKSS-YLGQN--------------FSIP-SSDENETMLHACFS-------------
KRLSPAVLDKTIKTVILRRSKAST-ELCD--------------DHCLAMSPSQE----------------------------------------
---------------------TL-----------LAGLTVLLTPVTTVPAAPPRSL-------------------------
RRGWSSHTLWWPSGPDPRQRSRQLLRGLC-A-----GV-GAPIPSGGQVDQTL----------DKIQTDYELNK-------------------
----------------------------SYQKSLSYKAKRVTKRKKMYKLYE---KHQEE-------------IKYKKNMM-------------
---------------------LTE-------------------S-------RARSCKK-------------------------------------
THT-EH--NY--SKPDQFHDYILKT----SNYTKICILDGDGVPHCI-------PPAACNGSSPDSWHEAPVRGASG-------
>KolobokP-3_MeMe_1p
----------------------------------------------------------------------------------------------------
---------------------------------------------MAQKRLLL-------------------------------------------
------------TILPVFKQTASKSYINCIEFNMAKGK-------L-K-R-----RA-------AGG---------HFKP--------
GNKKSP-AYL------------------------------------------------------------------------------------
-----------LPSKAATDPGC------SSAVNEDQ----LDRGCPK--KPELR----------PRQL-----D-------------------
----------------L-KM-----EC-E---P------------L--------GNRVVNLDQLIYLMNSVYQQH----SSGK----CK-
QLNI--SLHKEEKYGLGSKLQFKCNS--CHFLSN---------K--MDTYKKRR-----------------D---------GKGAA--------
-------------INMLLASALQDT---AIGIEKVNLLLTS-MDIPPPARSNMQTLLNAASLNTV-KL------------NEEDMAAKRQLV-
----IQHNLEQ----GH---QNPQLL----------------------DLSFDGRYNATRMVS-SYKPGQAASQAYGVAIENHTSY-------
--------------------------------KYIVGLAVQNKLCWTGAY----LR---NKG-FD-ITCP------------------------
-----GGHA----E---C-TATISYMQPH--SERKMAYGIAEQLS--NE------DLLVRTLTTDGDTAAHLGMNDF-------YD-
KLGYAWSVNRQADPHHLGSR--QVRKARTSN----WSQTVFEGK-----KLTGSARQQAISALGKD---------------------------
IKARCSAIIEKLRTDGNG------------------------LTEEVVNRLPA-------------IRAATIQCYA-GNCSF-------
CPHDSI-----------------VCSGL--------------------------------------------------------------
--------------GG-V---GD--------WWYHSK-FLPTHGI------------HHLQ-LNKNDRELLSTILE-------------
IRLSECAIMSVKNNTSTQKCEGFN--------------------RAVLSTMPKDI---------------------------------------
---------------------NL----------------------------SRNFAG------------------------SLASKTLQLN--
NSLQ------ASVEKKV-K-----SITGLALSP--QAKRYL----------KTTSKRSQRHR-------------------------------
---------------NHQKTAKFKTRRKNNRAKLEYAYH---QARSSGQ---------YSDEYVKGQL------------------------
--------DEA-----------------P-------SAKQCF------------------------------------------------
-------------------------------------------------------------------------
>KolobokP-101_GarTel_1p
----------------------------------------------------------------------------------------------------
----------------------------------------------------------------------------------------------------
----------------------------------MVKGK-------F-K-R-----RA-------AGT---------QFNSL------------
--KE-----------------------------------------------------------------------------------E-
KFIRRKKAFI-RRVITRSSSKVNT------ATGLHEPTALR---------PGPTLR----------PREE-----D-----------------
---------------------------IQ-P---C------------L--------GNRIINLDMMLIMMNNLFNSH-RN-SSKH----CD-
QLTL--GLRKEQKHGLGSRLQFQCET--CNFVSE---------S--YRTYQPCV--N---G-------R-G------------AA--------
-------------VNKLLASALMDM---PIGVEKANVLLTC-LDIPPPCRSYLQELVNKASSDVE-QL------------NQCDMAEKRQLV-
----VKHNREQ----GL---RNPKHL----------------------DISFDGRYNATRMVS-SYKPGQAASQAYGVAIENHTSY-------
-----------------------------QYIVALAVENKLCWTGAY----LK---NRN-FR-VTCP-------------------------
-----GPHE----G---C-TATKEYFSPH--SERSMAFSIAEQLS--QE------DLLVRTLTTDGDAKAFLGMTDF-------YK-
KLDSTWKVSRQSDPYHLASR--QYRKARSAQ----FSQNMFPKR-----K-NKEARGIASTALARD---------------------------
IKARCSAVLDRLREIGHG------------------------DVSQQIERLPA-------------ICSATVECYS-GNCSF-------
CPHDSL-----------------VCSGL--------------------------------------------------------------
--------------RR-K---GD--------WWYKSA-FLQPHGI------------NSLK-MTTNDRELMSTILE-------------
```

IRLSEQAVLSVKSGTSTQKCEAFN-------------------RATLSTMPKEI---------------------------------------
--------------------NM-----------------------------SRNFAG-----------------------ALASKTLQLN--
NSLK------ASVQKKV-L-----GITGRELSP--RASQYL----------TATSRRANSHR-------------------------------
---------------NYQKTLTYKRRRRYRRARLENQYR---VMRNKGL---------IVEEYVKEKT-------------------------
---DG--TY--
EKM---
>KolobokP-3_GarTel_1p

---------------------------------MAKGD-------R-K-K-----RA-------AGI---------LYKK--------
NHPST--RH--
-----------AVEEHHQQPGQRFATEVQPEAPAKI----ISESRP-------R----------TRGLL----K-------------------
----------------M-EK-----SN-E---S------------L--------GNRIVSLNKMICLMNTVCRDH-QQ-FPAH----CE-
TMNL--QLHSEKKIGLGSDLKFACKT--CCFVSQ---------T--CQTYTPCA--G---K-----T-R-G------------AA--------
-------------INMLLASALQDM---SIGVGKANILLSS-MDIPPPSNSHLQTLAIKASENTV-QL------------NERDMAEKRQLV-
----IQHNQAK----GA---TDPRQL------------------DVSFDCRYNANRMVS-SYKPGQAASQTYGVAIENHTSF-------
------------------------------KYIVGLAIENKLCWYGAY----LR---NQG-FEGIECP-------------------------
--DTDNSHE--------C-TATLDYMEPH--SERRMAYDVASQMH--RE------DIIIRTLTTDGDTKSYLGMQDF-------YD-
QLGEAWSVSRQADPYHLGST--QVRRVRKAK----WSTGMFPSC-----A-NQQTRQQAMSAFAKD--------------------------
VKSRSSKVVERLRALGDG------------------------DITKHIKRLPG-------------VCAATVDCYS-GNCSL-------
CPHESL-----------------VCSGV---
--------------RG-Q---GD--------WWSTSE-FLSTHEI------------DRLR-MTENDKCLLKTILE-------------
VRLSEQAVYNVSSNTSTQKCEAFN-------------------RGALASLPKEV---------------------------------------
--------------------NF-----------------------------GKTFPG-----------------------RLASKTLQLN--
NSIQ------TSVQAKV-T-----AITGQKLSS--RTSRYL----------DFCARRTESRK-------------------------------
---------------QRQRSIRFKAKRRHNRARLELAYH---SARSGCKKQ------SGSNEYSKGQL-------------------------
--------D--

>KolobokP-4_GarTel_1p

---MS--
----------------------------------KKGK-------L-K-R-----RR-------AGH---------LFGS--------
NNKRTS-R---
-----------VTKHTSAEK----VTPVHIPVPVLH----INDGQQENLGPCLR----------PRGN-------------------------
------------------LE-----NT-G---N------------L--------GNRIISLDQLLEVISTVSNDH-GN-HPVN----CE-
SMKL--RLLSERKLGLGSDLTFACST--CQFVSK---------P--CQTYRPRA--G---K-----R-R-G------------AA--------
-------------VNMLLASGLQDT---CIGVEKGNILLSS-MDIPPPSKSHLQSLVNEMSASTI-SL------------NEDDMSKKRNWI-
----IQQNEAK----GA---NDPRQL------------------DLSFDCRYNATRMVS-SYKPGQSGSQAYGVAIENHSEY-------
------------------------------KFVLALAIENKLCLTGAL----LK---RKHKYEEVKCP-------------------------
-----GKHE----G---C-TANIPYMQPH--SERRMAHDVAQQLW--ED------DIVVRTLTTDGDTRSFLGMQDF-------YR-
ELGEAWDVTRQADPHHLGST--VVRHSRKAN----WSRDMFPN------KVTRLTKQQAIAAFAKD--------------------------
VKSRCSKIISKLRILGDG------------------------DLRRTLPLLPS-------------VCSATVECYS-GNCGL-------
CPHRSL-----------------VCNGV---
--------------GG-Q---GD--------WWFHSE-FLPTHNI------------NSLK-MTEQDKELLGSILQ-------------
IRLSEQAIINVASNTDTQKCEAFN-------------------RGVLSILPKEV---------------------------------------
--------------------NY-----------------------------CRNFAG-----------------------KLASKTLQLN--
NSLV------TAIEAKV-S-----LITGESLSA--KPKSYL----------RQRSRVSAARK-------------------------------
---------------RLQGTTAFKARRRKRRAELEHTYH---TARTGATAE-------RQDEYVKGLL-------------------------
--------DKM---------------G--
-----------------------C--
>KolobokP-2_MyGa_1p

---M---

--ARTTMQRITNNVCDKVV-KL------------ANEETKKVVENF-----
KRRNETL----GL---NKNSPI----------------------KLQMDGTYQSVYIKS-RHKMGQNASQAIGIACENETDS-----------
--------------------------HDVIGLHLINKLCWVGAW----LR---GKG-YD-VECP-----------------------------
--NHE----H---C-TANTNRYDPL--SEKDLGYEIGKKIA--TN------ELLVDYCTTDGDAKSVKGLQEA-------
MQEVFGPLWSVNRLADTIHRGQS--QFREGIRAN----FSVGMFPG------V-TKAQKNDIKTAFAND------------------------
--IKLRSHGIMKSLFMKYNG------------------------DRQQISKCLPG-------------IVRSVVNCYS-GNCGDT------
CRWSIT-----------------LCNG--
--------------GK-K---TS--------WWYKSI-NLNSHGLQN----------GSLI-PTDRDKLLIESLLE-------------
MKLSQSALDQMKFFSNTNKCESVN-------------------RTISTYLPKNK---------------------------------------
--------------------NF-----------------------------SRNAIG-----------------------RASAAVLKVN--
NNRD------VALVKTL-K-----AV-GCTMGKKSRAAVAL----------KKIRKLEMYDS-------------------------------
---------------AYQKSSKVKYRRLQARKRQAINFL---LHKSNR-----------NDGYKKHQL-------------------------
--------EPK-----------------L-------SQLTNCK--EF----
--VP--QPGCSFWPD---------
>KolobokP-7_CGi_1p

----------------------------------MRGE-------R-K-K-----HK-------F--V-----CGNSIRK--------
GRVGHG-H----------SKHE-VF---KS---V-

```
WLPRLTANQF-N-LITKVSPDGK-------SYTLSGAE----KSLGNV----KVLR----------PRPS--DP-N------PY-----S---
------------------R-VT-----RL-D---S------------R--------EMRVVDKGLTLDMINDLCVSH----ARCK----CQ-
LADF--EIKSETKWGLGWSWTFGCKR--CNFISK---------P--YKLYHEVP--S---E-----H-C-G---------RKAAE--------
-------------MNLGIQTGIYQT---PLGNDQARLILAC-TGIPPPARSAMYKSGNKVGEKIV-EI------------AEHDMNEKLKQL-
----KKKNEML----GL---PSSHPI----------------------NIQMDASYQSRGITS-RHKMGQGASQVIGVACEDETDQ-------
------------------------------HNVISYHMVNKLCWVGAW----LR---GEG-YD-VSCP-------------------------
-----NGHV----G---C-TANKNPAEPL--SERETGYKIGEKLA--KH------DLLVKYVTTDGDATSCAGLETA-------
LQNTLSPLWKTSRLADRIHRGQS--LFRQGIKAE----FSPKMFPA------H-TKTQKSDLQKMFAND------------------------
--IKERCHGIFQALFKKHNG------------------------DLNKISKCLPR-------------VVDRVIKCYS-GGCGEG------
CRWSVT-----------------LCKG------------------------------------------------------------------
--------------GK-K---TS--------WWHKSV-GLRAHNLEP----------GDLK-LNQKDKVILKSLLG-------------
MVLSISALNEMKLNTTTNKCESVN-------------------RTISVSLPKNR---------------------------------------
--------------------TY-----------------------------SRNAKS-----------------------RALSGLLRAN--
NGVD------MAVCKTL-A-----SL-GAPLGAQSQSLKAL----------QRIKQISEYSI-------------------------------
---------------KHAKSLNQKKKRAWARCRNAIQYV---KQKLNRP-V--------KTDYRKNQL-------------------------
--------EPS-----------------L-------SGLAKQ-----------------------------------R-CEN---------
LEN----------------------------------------------PDPD-QPGCSHW-----------
>KolobokP-3_SiCo_1p
---------------------------------------------------------------------------------------------
---------------------------------------------------------------------------------------------
----------------------------------MKGD-------K-A-R-----KS-------T-----------MFKA--------
RHVPWN-X----------GLKM-A----------------------------------------SSSS------------EGXRQQ---
PIITTRLTADEF-S-MVSKPSADGR-------GFTTPDCE----GNSGNI----RLLR----------PKSE--KT-S------DLA----S-
--------------------K-ED-----NE-S---I------------T--------GMRFIDIEKNSAVFNEVFQCH-QH-ESKD----
CI-QANM--TVAEERKVGVCVKFSLKCTN--CDYISP---------V--KKLYKEAS--S---S-----K-R-G---------PKPAA-----
----------------ANXALATALQDT---PMSNTKCRYLLAN-MDVPPPSRTSMQRTSNRVSKAIK-TL------------
NDRDMSAKVQHV-----KDVNKKR----G----HDENII----------------------NVGIDGRYNSACITS-
RKKAGQNASQSIGIACESVTGK-------------------------------------QYIIASALQNKLCWSGAW----LR---NQG-FK-
VTCP------------------------------GGHE----D---C-TANIHPQAPL--SEYELGREIGEQLG--VQ------
GVFIKYATTDGDSRSAAGVAQA-------MK-VLDPMWKVERLADPTHLGQA--QFRQCYSAK----FSSEMFSG------K-
TREKRQEQQRVLSQD--------------------------VKARCSLVLKELMKVLAG------------------------
DVRRIKVELPN-------------VLRATVSCYD-GDCSS-------CRRHSY-----------------VCSG-------------------
-------------------------------------------------------------GV-S---NS--------WWTRSM-FLATHRI-
-----------SNLQ-MNDNDRHLLEEILK-------------MKLSISALEEMKLGTSTQKCEAVN-------------------
RSISASLPKNV-----------------------------------------------------------NF---------------------
--------SRNVHG-----------------------RLSSTIHRLN--NGLC------NSAVEKL-G-----CM-GVTLSP--KPLRSL---
-------KRMQQEQEYQK-----------------------------------------------MYAKRPETTKRRLLCKGRQLREHL---
QYKKISK-L--------TSDYRKGRL---------------------------------DPV-----------------P-------STSRA-
-------------------------------------ENN-EH--SY--SQ------------------------------------------
----------------------------
>KolobokP-2_MeMe_1p
---------------------------------------------------------------------------------------------
---------------------------------------------------------------------------------------------
----------------------------------MKGD-------R-N-R-----KS-------S-----------RFSP--------
GSIPWN-K----------GLQL-S----------------------------------------SMNC------------GDTEQK---P-
STSRMTAEEF-S-LVARPTADG---------------G----ETSGNV----RVLR----------PKVT--ET-S------AL-----K---
------------------K-ED-----TT-S---C------------S--------GMRLVDCEKMSEAFNEAFQIH-QQ-EELD----CE-
QANI--IVAEERKVGVCWKFSLKCTN--CNYVTP---------L--KKLYQEAP--S---P-----R-R-G---------PNPAA--------
-------------PNLGLAAGLQDT---PLGNTRCRYLLAN-LDIPPPSKSGMQKTANKVGSAIK-EL------------NDSDMSEKVEML-
----KEVNRRR----G----DPENSI----------------------NIAMDGRYNSTTISS-RKKAGQNASQSIGIACETMTEK-------
------------------------------QYIIAAAVQNKLCWTGAW----LR---NKG-YD-VRCP-------------------------
-----DGHA----D---C-TASLHRNAPL--SEYQLGKSIGEQLG--VQ------EAYIKYVTTDGDARSAAGVGEA-------MR-
LLDPMWKVERQADPTHLGQA--QFRHCYQAN----FSAGMFTA------K-TREKRQEQQKVFSQD--------------------------
IKARCSLVFKELMRIHAG------------------------DITQIKKELPS-------------VLTATVNCYA-GDCSS-------
CRRHSY-----------------VCGG------------------------------------------------------------------
--------------GV-T---NS--------WWNRSM-YLASHKI------------TNLF-MEENDRHLVQEIIK-------------
MKLSDSAVEQMKLGTSTQKCEAVN-------------------RSISVSLPKNV---------------------------------------
--------------------NF-----------------------------SRNVHG-----------------------RLASTIHRLN--
NGNC------G----------------------------------------------------------------------------------
---------------------------------------------------------------------------------------------
---------------------------------------------------------------------------------------------
----------------------------------------------------------------------
>KolobokP-1_SiCo_1p
---------------------------------------------------------------------------------------------
---------------------------------------------------------------------------------------------
----------------------------------MRGD-------R-K-K-----KA-------F-----------GFKK--------
GCSPWN-K----------GLKS-D----------------------------------------SHFH------------VDAEPK---R-
SVIRLTEDEF-P-VSTRPTADG---------------S----ELRGDV----MLLR----------PRTS--RS-Q------PGA----K---
------------------Q-EE-----NS-N---C------------H--------GMRLVDKQEMASSINVALESH-RQ-KSTE----CH-
QPNL--IVADERKMGLCWKMSFKCNN--CSFITP---------L--LKLYKEAE--S---S-----K-R-G---------PKPAT--------
-------------TNLSLAVGLQDA---PMGNSKFRYILAS-MDIPPPAKSSMQKTSNTVGSAIK-EL------------NDRDMSKKICLV-
----KDISRKR----G----NPGNSI----------------------NIAVDGRYNSSVISS-RKKSGQNASQSIAVACETMTER-------
------------------------------QYILAAAVQNKLCWTGAW----LR---GKG-YH-VECP-------------------------
-----GGHA----G---C-TANTYRQAPL--SEYELGKSIGQTLA--AE------SALVRYVTTDGDSRSAAGVTEA-------MR-
LLDPMWKVERQADPIHLGQA--QFRYCYNAN----FSNGMFPGA-----K-TRDQRQEQQKGLSQD--------------------------
IKARCSCILKEMIKRHAG------------------------DMTFIKKTVTP--------------------------CS------------
---------------------------------------------------------------------------------------------
```


>KolobokP-1_HaRub_1p

--NVQ------------------

GGHP----D---C-TANMSAFTPF--SEFKMGEDIGNQLA--LQ------GVLIKYATTDGDSKSADGVDKA-------LK-
VLDPMWKVQRLADPTHLAQG--QFRQCYRAD----FNPDMFPG------T-TRTHKMEAKKVLSQD--------------------------
VKARCSLILKELMKDHAG------------------------DMKTLRSILPR-------------VLEATLKCYS-GDCSR-------
CARYSV-----------------VCRG--
--------------GV-T---NS--------WWQRSM-FLGCNSI------------THFN-MDDNDKHLLNEILK-------------
MKLSVEVVEQMKLYTDTQKCEAAN-------------------RSLSISLPKNV-----------------------------------
--------------------NF-----------------------------SRNMIG-----------------------RASSTIHRLN--
NGPG------TSAIDKC-D-----HS-GVKLSS--RAVRSL----------EQMDKEATYHK------------------------------
---------------DYQKSPSVVKRKLTQHGSKICEHL---KSKQTDK-Q--------SGDYRKGQL-------------------------
--------DPV------------------P-------CFSK--NH-EH--SY--
SKY---
>KolobokP-1_HaRuf_1p
-----------------------------------M------------------------------GYI-------------------------
---GTSCAQSS--
------------------------LVLSE-TI-TMKGD-------R-K-R-----IK-------S-----------QFSK--------
GHTPWN-K----------GLTL-E--SEETESD---------SPPQIP---R-
TVTRMSAEDF-S-LVTKSRLDGT-------GLSTPDCE----GISTSV----RLLR----------PTPA--SK-Q------NLK----Q---
------------------E-SS-----KD-D---L-----------E--------GMRLVDNEKVAEAWNTALRLH-RS-TSTD----CD-
DPQL--QIAKERKWGACWKVTLKCVV--CDFTAP---------E--MKLYKEIK--T---G-----K-P-G----------PNPAA--------
-------------PNVGLALGLQDT---PIGNTRARLLMAN-MDIPPPCRSSMQRTSNKVAIAVT-EL-----------NTQDMAEKVELV-
----KEVNRKR----G----TEPCEM----------------------NIAMDGRYNSTTIAS-RKKPGQNASQAIGIACETITDK-------
----------------------------KFIIGASFQNKLCWTGAW----LK---GKG-MD-VKCP-------------------------
-----GGHP----D---C-TANMSAFAPF--SEFNMGEDIGNQLA--LQ------GVLIKYATTDGDSKSADGIDKA-------LK-
VLDPMWKVQRLADPTHLAQG--QFRQCYRAD----FNPDMFPG------T-TRTQKLEAKKVLSQD--------------------------
VKARCSLILKELMKDHAG------------------------DMKTLRSILPR-------------VLEATLKCYS-GDCSR-------
CARYSV-----------------VCRG--
--------------GV-T---NS--------WWQRSM-FLGCNKI------------THFN-MDDNDKHLMNEILK-------------
MKLSVEVVEQMKLYTDTQKCEAVN-------------------RSMSISLPKNV-----------------------------------
--------------------NF-----------------------------SRNMIG-----------------------RASSTIHRLN--
NGPG------TSAIDKC-Q-----NS-GVELSS--RAVRSL----------EQMDKEAAYHK------------------------------
---------------EYQKSPSVVKRKLVQHGSKISEHL---KSKLSDK-Q--------RGDYKKGQL-------------------------
--------DPV------------------P-------CFSK--QA-DH--SY--
SKK---
>KolobokP-2_PhoLin_1p

----------------------------------MKGD-------K-R-R-----KA-------T-----------QMKR--------
GHAQLK-K----------SQDS-N--SEETMVP---------PRDQVQ---R-
QYTRLSPEES-A-MVSRSTSGGT-------SLSAANID----GKVSSI----CLLR----------PRSR--NH-P------ERR----E---
------------------T-TR-----QS-E---L-----------E--------GNRVIDVTKMTEAVNSALELH-HT-SSPD----CR-
VPQL--VVFRERKWGLAWKITLKCTE--CDLITP---------E--MKLYKEVE--T---N-----Q-P-G---------PNAAA--------
-------------QNVGLAVGLQDT---PIGNSRARLLLAN-MDIPPPCHSAMQRTSNKVSKVVT-QL-----------NTQDMAEKVNIV-
----KKISEKM----G----NNPNEM----------------------NIAMDGRYNSTTITS-RKKPGQNASQAIGLACETITEK-------
-----------------------------KFIVAASFQNKLCWTGAW----LR---GKG-IE-VRCP-------------------------
-----DGHP----E---C-TANISAFAPL--SELNMGTDIGNQLT--RQ------GILIKYATTDGDGRSAEGLDKA-------MR-
VLDPMWKVQRQADPTHLAQG--QFRQCYKAQ----FSADMFPGA-----T-TRDQKMAAKRTLSQD--------------------------
VKARCSLVLKELMKQHAG------------------------DMKSLSSILPR-------------VLETTLKCYD-GDCSL-------
CGRHSV------------------VCGG---
--------------GV-T---NS--------WWQRSM-FLACNGI------------EQLN-MNDSDRHLLLEILK-------------
MKLSVQVVESMKLYTDTQKCEASN-------------------RSLSVSVPKNV-----------------------------------
--------------------NF-----------------------------PRNMTG-----------------------RASSAIHRLN--
NNQG------SSTIAKC-E-----AT-GVGLSS--RCLRTL----------TKMDRDQEYQK------------------------------
---------------QYQKDPSNVKRKLIKHGRDIVKHC---EYRKNNG-E--------KSDYQKGQL-------------------------
--------DPK------------------P-------SHSR--DH--PY--
SR--
>KolobokP-3_SteCin_1p

----------------------------------MKGD-------K-R-R-----KA-------S-----------QMKK--------

GHPQFK-K----------SQDS-N--SEEPMPP---------PSDQVE---R-
QYTRLSPEES-E-MVSTSTSGGA-------SLSAANID----GKVSSI----CLLR----------PRSG--NH-P------ERQ----E---
------------------T-SG-----QS-E---L------------E--------GNRVIDVTKMTEAVNSALEMH-HT-SSPD----CR-
VPQL--VVHRERKWGLAWKITLKCTE--CHLISP---------E--MKMYKEVE--T---N-----Q-P-G---------PNAAA--------
-------------QNVGLAVGLQDT---PIGNSRARLLLAN-MDIPPPCHSAMQRQSNKVSKAVT-EL------------NTQDMAEKVKIV-
----KNISEKM----G----SNPNEM----------------------NIAMDGRYNSTTIVS-RKKPGQNASQAIGLACETMTEK-------
------------------------------KYIVAASFQNKLCWTGAW----LR---GKG-IE-VRCP-------------------------
-----DGHP----E---C-TANISAFAPL--SELNMGTDIGNQLT--RQ------GILIKYATTDGDARSAEGLDKA-------MK-
LLDPMWKVQRQADPTHLAQG--QFRQCYKAQ----FSADMFPGA-----T-TRDQKMAAKRTLSQD--------------------------
VKARCSLVLKELMKEHAG------------------------DMKSLSIILPK-------------VLETTLKCYD-GDCSQ-------
CGRHSV-----------------VCGG--
--------------GV-T---NS--------WWQRSM-FLACNGI------------EQLN-MNDSDRHLLLEILK-------------
MKLSVQVVESMKLYTNTQKCEASN-------------------RSLSVSVPKNV---------------------------------------
--------------------NF-----------------------------PRNMVG-----------------------RASSAIHRLN--
NNQG------ASTIAKC-E-----AM-GVDLSP--RCLRSL----------SKMDKDQEYQK-------------------------------
---------------RYQKNPSNVKRKLIKHGRDIVKHC---EXRKNNG-E--------KSDYQKGQL-------------------------
--------DPK-----------------P-------SHSR---DH--PY--
SR--
>KolobokP-1_SteCin_1p
-----------------------------------M----------------------------VYK--------------------------
---MSCCQQWC--
------------------------IISTL-LS-NMRGD-------K-K-R-----RT-------Q-----------GFQK--------
GNIPWN-Q----------GLTF-D--SDEESDL---------QQQTQP---K-
LVTRMSAEQF-E-LVARSRSEDS-------LLPTPDCE----GISSSV----RLLR----------PLPA--EK-S------KSQ--------
------------------D-GS-----SQ-V---S------------E--------GMRLVDSEEMAKCWNESIRLH-HS-TSPD----CE-
NPNL--VIHKERKWGVCWKVVLKCTA--CDFKTP---------E--MKLYKEVK--Q---D-----K-P-G---------PNPAA--------
-------------ANLALAVGLEDT---PMSTTRARLLMAT-MDIVPPAKSSMQRTANQVGKAVT-EL------------NTKDMADKVQLI-
----KQVNSDR----G----VEMSEV----------------------NIAMDGRYNSTTITS-RKKPGQNASQAIGLACETVTDK-------
------------------------------KYIIGASFQNKLCWTGAW----LR---GKG-LE-VTCP-------------------------
-----GGHA----E---C-TSTMSTFAPF--SEKNMGKDIGAQLA--LQ------GMLIKYVTTDGDSRSAEGVDSA-------MK-
TVDPMWRVERLADPTHLAQG--QFRHCYKAS----FSPEMFAG------T-TREQRKQQQRVLSQD--------------------------
VKARCSLILKELMKTHAG------------------------DMNALRNILPR-------------VLEATLLCYS-GDCSR-------
CARHSV-----------------VCSG--
--------------GV-S---RS--------WWTRSM-HLCKNKI------------SSFN-MNDSDKHLLQEILK-------------
MKLSVEVVEQMKFYTDTQKCEAAN-------------------RSLSVSLPKNV---------------------------------------
--------------------NF-----------------------------SRNYMG-----------------------RASSTIHRLN--
NGPG------TSAIEKT-E-----CV-GVHLSA--GSVRAL----------HQMDEQTEYQK-------------------------------
---------------KYNKDSAVVKRRLLQQGEHIGSYI---KAKASMK-D--------KCDYRKGQL-------------------------
--------DPK-----------------P-------STSQ--DR-DH--SY--
SK--
>KolobokP-6_GiAe_1p
MN-------------I------------------YI----------------------------RVD--------------------------
---MHTTISIS--
------------------------LFYTLRVT-SMEGN-------T-R-R-----KT-------------------QFKK--------
GHAPWN-K----------GATL-L--HEHASPE---------SHTEKL---R-
PVVRMNIDEF-A-LVTKSSSTSA-------TISTPDCE----GASQPV----RLLR----------PITSTCTA-S------EMK----E---
------------------E-QQ-----CK-D---K------------E--------GMRIMDNDRMVDAWNAAFRYH-ST-ASPE----CD-
EPEM--QILKEVKWGLCWKVTLHCVN--CDFTAP---------E--SKLYNEVK--T---N-----K-P-G---------PNAAK--------
-------------TNVGLAIGLQDT---PVGNTRARVLLAG-MDIPPPCRSSMQRTSYKVSTAVT-DL------------NEKDMAQKVELL-
----KDINMKR----G----NERDEI----------------------NIAMDGRYNSTTITS-RKKPGQNASQAIGLACETMTER-------
------------------------------KFIVSACFQNKLCWTGAW----LR---NKG-IH-VTCP-------------------------
-----GGHP----D---C-TANLPPFAPL--SEYEMGKDIGTDLA--LQ------GILIKYVTTDGDSRSSAGVGSA-------LK-
LLHPMWKVQRLADPTHLGQS--QFRKCYKAN----FSPDMFTG------Y-TREQKREAQKVFSQD--------------------------
MKARCSLILKELMKLHTG------------------------NIGVLKRNLPK-------------VLQSTLSCYG-GDCSK-------
CSRYSV-----------------VCSG--
--------------GI-T---NN--------WWHRSM-FLGTHRI------------TELN-MNENDKMLVLELLK-------------
IKLSISAVEQMKLYTDTQKCEAAN-------------------RSLSVSLPKNV---------------------------------------
--------------------NY-----------------------------SRTMTG-----------------------RAASTIHRLN--
NAPG------TSMIEKC-K-----YC-GIELSA--RVRRSL----------RQMDKESDYQK-------------------------------
---------------RYQNNPQVIKRHLVQQGSKIRDHL---RYKHLHRDQ--------QADYKKGLL-------------------------
--------DID-----------------S-------QRTD---------------------------------------NSC-DH--SY--
SK--
>KolobokP-1_MaMa_1p

----------------------------------MKGY-------T-K-K-----KK-------T-----------FFKP--------
GNIPHN-K----------GVRF-L--TDDTLPS---------SSAASA---Q-
VTRRMDADKF-S-LMAVTKPTGGGLQ----SVVAPDCN----GTPGSA----RILR----------PSSK--AR-L------TLK--------
------------------E-TE-----VN-Q---H------------E--------GTRMICMNKNVEMWNDIIHRH-GN-QDNR----CE-
RPDF--KIANETKWGLAWKHTMMCKN--CSFKSP---------E--YKLYDEIK--T---A-----K-P-G---------PNPAA--------
-------------VNMTFQAGLQDT---PMGNTRARYLLAA-XDIPPPSRTSMQRASNIVGQATV-QL------------NEMDMADKLKLV-
----RQVNVMR----GV---QDPEQI----------------------NVAFDARYNAISFGH-EKKPGQSSSQAVGIVCETVTEK-------
------------------------------QYIVGATVENKLCWTGAW----FK---GQG-LD-VQCP-------------------------
-----GGHV----D---C-TANLSPVAPH--SEHAMAREIGNKFA--LQ------EILIRHATTDGDAQAAAGFQDA-------YS-
ILYPMWKVXRLSDPTHLGRS--QFKRCNSAT----FSESMFPGI-----R-TREGKKLKQKILSQD--------------------------
VKARCSLIFKRMMEDTGG------------------------DVRLIQCQLPQ-------------VLDATLRCYS-GDCNK-------
CRHYSK-----------------VCGG--

--------------GS-T---NS--------WWIRSA-FLGPHQV------------THLN-MTESDKNILLEVLK-------------
MKLSCDVVQRLRLNTNTQKCEAVN-------------------RSISVSLPKNV---------------------------------------
--------------------NY-----------------------------SRNFEA-----------------------RVHSTIHRLN--
NRLG------SSLKTKV-K-----HL-GGILSP--RTLRSL----------NEMDQTCTYHQ-------------------------------
---------------LYQKRPETRKRILARRGNMIQAHL---RYRTQPGNR--------KSDYCKGLL-------------------------
--------DSH--SKG-DH--SY--
AQV---
>KolobokP-1_PoSt_1p

---MHCTRYIS--
---------------DREMIHLVLNFHAVIVTETMKGY-------K-K-R-----KT-------T-----------FFKP--------
GYIPHN-K----------GVRY-L--TDDTLPT---------SSAASV---P-
VTRRMDAVKF-S-LMAKTKPTNGDLQ----SVQALDCN----GTPGSA----NILR----------PSYK--AK-L------DLK--------
------------------E-TE-----LN-E---H------------D--------GTRIICMKKNVEMWNDIIHRH-GN-QVNM----CG-
RPEF--QIANERQWGLAWKHTMMCKN--CSFKSP---------E--YKLYDEIK--T---N-----K-P-G---------PNPAA--------
-------------VNMMFQVGLQDT---PMGNTRARYLLAA-TDIPPPSRTSMQIASHTVGKATV-QL------------NEMDMIDKLKLV-
----RQVNVMR----GV---QHPEQI----------------------NVAFDARYNAITFGH-EKKPGQSSSQAVGIACETLTEK-------
------------------------------QYIVGTAVENKLCWTGAW----LK---GKG-YD-VQCP-------------------------
-----GGHV----D---C-TANLSPVAPH--SELEMAREIGNKFV--LQ------EILIRHATTDGDAQAVAGFQAA-------CN-
ILHPMWKVERQSDPIHLGRL--QFKRCNSAV----FSEDMFPGI-----R-TREGKKLKQKIFSQD--------------------------
IKARCSLIFKQLMEDNSG------------------------DVTKIKCQLPQ-------------VLEATLRCYS-GDCSK-------
CRHHSK-----------------VCGG--
--------------GS-T---NS--------WWMRSA-YLGPHQV------------THLN-MTEDDKNILLEVLK-------------
MKLSCDAIERLRLNTNTQKCEAVN-------------------RSISVSLPKNV---------------------------------------
--------------------NY-----------------------------SRNFEA-----------------------RVHSTIHRLN--
NSLG------SSLKTKV-T-----HL-GGKLSA--RTLRSL----------KIMDKDCTYHK-------------------------------
---------------LYRKRPQTRQRILARRGRMIQAHL---RYRTQPGNR--------KSDYSKGQL-------------------------
--------D--TKG-DH--SY--
ANA---
>KolobokP-2_MeNe_1p

---------------------------------MKGY-------K-K-R-----KT-------T-----------FFKP--------
GYIPHN-K----------GVRH-L--TDDTLPS---------SSAASV---P-
VTRRMDADKF-S-LIAVTKPTNGGLQ----SVQAPDCN----GTPGSA----KILR----------PSSK--AK-L------DLK--------
------------------E-TE-----LN-E---H------------D--------GTRIICMKKNVEMWNDIIHRH-GN-QVNI----CD-
RPEF--QIANERKWGLAWKHTMMCKN--CSFKSP---------E--YKLYEEIK--T---N-----K-P-G---------PNPAA--------
-------------VNMMFQAGLQDT---PMGNTRARYLLAA-TDIPPPSRTSMRRASNTVGKATV-EL------------NEMDMIDKLKLV-
----RQVNVMR----GV---QHPEQI----------------------NVAFDARYNAITFGH-EKKPGQSSSQAVGIACETLTEK-------
------------------------------QYIVGTTVENKLCWTGAW----LK---GKG-FD-VQCP-------------------------
-----GGHV----D---C-TANLSPAAPH--SELEMAREIGKKFV--LQ------EILIRHATTDGDAQAAAGFQHA-------YN-
ILHPMWKVERLSDPIHLGRL--QFKRCNSAI----LSENMFPGI-----R-TREGKRLKQKIFSQD--------------------------
IKARCSLIFKQLMEDNSG------------------------DVTKIKCQLPQ-------------VLDATLRCYS-GDCSK-------
CRHYSK-----------------VCGG--
--------------GS-T---NS--------WWMRSA-YLGPHQV------------THLN-MTENDKNILLEVLK-------------
MKLSCDVVERLRLNTNTQKCEAVN-------------------RSISVSLPKNV---------------------------------------
--------------------NY-----------------------------SRNFEA-----------------------RVHSTIHRLN--
NSLG------SSLKTKV-T-----HL-GGKLST--RTLRSL----------EAMDKDCTYHQ-------------------------------
---------------LYQKRPQTRQRILARRGNMIQAHL---RYRTQQGNR--------KSDYSKGLL-------------------------
--------D--TKG-DH--SY--
AQV---
>KolobokP-1_PatPel_1p

---------------------------------MKGE-------R-K-R-----QA-------G-----------RFLS--------
GHKVLF-K----------RQCL-Q--ATS--------------------------
--GTQTDY-N-VPSTSKCHTEAAG---FNIHPLFDQ----DSPGSH----RKLR----------PRSE--KK-G------DN-----Y-----
----------------H-IE-----AL-P---I------------E--------GNRIVDIVKMLEMWNSVIRGH-VKQKPKS----CS-
LPKF--VLATEEKWGAAWKCSVKCTK--CGFISP---------V--HKLYSEVH--T---G-----K-R-G---------PKPAS--------
-------------VNLTLQSGLMDT---PISNSRARLLMAD-LDIPPPARNSMQKLSNYVNTKTT-RL------------NTLSMREKINEV-
----KSINARG----GA---VDPNVI----------------------NVAVDGRYNCITIGH-SKKPGQGASQAIALACETNTEH-------
------------------------------KYILAAVMQNKLCWIGAW----MK---GKG-MQ-VECP-------------------------
-----GGHE----G---C-TANLVTEAPL--SELDMGKALGTDFA--LQ------NVLIKFATTDGDGRAAQGIEES-------LQ-
HLHPMWKVERLADPTHLAAS--QFRQCSNAK----FSENMFPG------K-TKLEKGKFKKILSQD--------------------------
VKARCSLIVKNLLKDHCG------------------------NVQELKKRLPK-------------ILQATIMCYD-GDCSK-------
CKVHSV-----------------VCNG--
--------------NL-R---SN--------WWIRSM-FLAANKF------------TGLQ-MEDNDKFILNEILK-------------
IRLSEEAIIKTRFNTNTQKCEALN-------------------RSLSVSLPKNV---------------------------------------
--------------------NY-----------------------------GRNAMG-----------------------RLSSTILRNN--
IGIK------RSTEGKA-N-----YL-GAKLSS--KTRHSL----------TRMENERKYYS-------------------------------
---------------QYKNRPAVKKRNLFTRGRKLYSFA---KYKQSHS-V--------ESGYKKGQL-------------------------
--------DFHSR-----------------------DMTDN---------------------------------------SLE-DH--SY--
SQ--
>KolobokP-3_PatPel_1p
---MG--------------------------
---MVPTTFLT--
---------------KRNAKQSTYNQFML-PS-NMRGQ-------K-K-R-----NA-------F-----------FFKL--------

```
GNQVNP-K----------KQRL-Q----------------------------------------GSST-------------------------
--ATQTDY-D-LPSTSTGGTEIRDE--INMHHLSTQ----SSPESH----RVLR----------SRPEMLKL-D------ET-----E-----
----------------N-SE-----AN-A---I------------Q--------GNRIIDIQKMFEMWNSVIRLH-TQGQRKA----CA-
VPNF--QPTSEQKWGAGWIYSVKCTK--CTFISP---------V--YKLYKEIA--T---G-----K-P-G---------RKAAA--------
-------------INLTLQSGLLDM---PVGNTRARLLLTD-LDIPPPSRSGMQTLSNQVSAKMT-RL------------NTMNMREKIEAV-
----KTLNQKA----GA---VNPNII----------------------NVAVDGRYNSITIGH-SKKPGQAASQAIGIACETNTKH-------
------------------------------KYILSAVMQNKLCWQGAW----LK---GKG-FT-VNCP-------------------------
-----GGHE----G---C-TANLPVHAPL--SEYDMGKSIGTELA--LQ------NVLIKYATTDGDGRSAAGIEDS-------LK-
FLHPMWSVERLADPSHLAAT--QFRHCYNAK----FSDEMFPG------K-TRLDKGKVKKILSQD--------------------------
VKARCSLIMSTLFENHCG------------------------NTEEMRKRLPK-------------ILESTIMCYD-GDCSK-------
CRPHSV-----------------VCRG------------------------------------------------------------------
--------------GI-T---NN--------WWNRSM-YLASNKV------------DSLN-MNENDKSILSEILK-------------
IRISESALEKTKFQTNTQKCEAIN-------------------RSLSVSLPKNV---------------------------------------
--------------------NY-----------------------------GRNAMG-----------------------RLSSTILRSN--
DGIK------SATEQKA-K-----LL-GAPLSP--KTRYSL----------RQIERESLYHR-------------------------------
---------------EYQTRPEVRRQALLLRGRKLYDFC---EYKNYRA-V--------ESGYKKGHL-------------------------
--------DQPSKADDSDQPSKADDLDQP-------STADD--------------------------------------TLQ-DH--SY--
SK----------------------------------------------------------------------
>KolobokP-1_LG_1p
---------------------------------------------------------------------------------------------
---------------------------------------------------------------------------------------------
---------------------------------------------------------------------------------------------
---------------------------------------------------------------------------------------------
---------------------------------------------------------------------------------------------
----------------------------------------------------------MVEMWNIAIQTH-ADEEAEV----CA-VPQF--
KINTEEKWGIGWKYSLKCLN--CGYISP---------V--VKLYKEIT--T---G-----K-R-G---------RKAAA--------------
-------VNINLQAGLLDM---PLGNSRTRLLLAD-LDIPPPAESGMSKLSSYVSSKTT-DL------------NNESMRTKVEEV-----
KNVNRRR----GA---SNPNVI----------------------NVALDGRYNCLTIGH-SKKPGQGASQSIGIACETNTDK-----------
--------------------------KYIISAVLQNKLCWTGAW----LR---NTG-IT-VNCP-----------------------------
--GHE----G---C-TANLTRHAPL--SEREMGRIIGEDLS--IQ------GVLIKFATTDGDSTSATGIEEA-------MR-
TLHPMWKVERLADPAHLSSS--QFRQCFKAN----FSDGMFRG------K-TKVEKTNQKKTLSQD--------------------------
IKSRCSLVIKEMYSTYCG------------------------DVDKLKKQLPK-------------ILQATVMCYD-GDCSK-------
CRSNSV-----------------VCSG------------------------------------------------------------------
--------------GH-S---NN--------WWNRSM-FLASNNL------------TSLE-MNDNDKFLLNELLK-------------
IRLSEEAIDKTRYKTDTQKCESIN-------------------RSINISLPKSV---------------------------------------
--------------------KY-----------------------------GRNAMG-----------------------RLSSTILRSN--
IGIK------EATQTKA-E-----FL-GAKLSP--KSLQAL----------SSIQKKSVFHR-------------------------------
---------------DYETRPEVKRRKLLARGRNIHEFA---KYKKFNS-T--------DSGYRKGQL-------------------------
--------DKIL----------------P-------SALQQ--------------------------------------LDD-DH--SY--
SHSK--------------------------------------------------------------------
>KolobokP-2_TeGr_1p
---------------------------------------------------------------------------------------------
---------------------------------------------------------------------------------------------
----------------------------------MKGD-------K-G-R-----KV-------T-----------QFKK--------
NQTGPN-RRY------IANLDV-ME---------------------------------------TDSEPVP---------STSSGS---T-
GWKRLSSDMY-S-MVTKTGLDGE-------CVTVPDCE----GNTGTA----KILR----------PKSS--SC-D-----PDLK----Q---
------------------M-KK-----ED-E---I------------K--------GMRLVDNEELLKMMNDVYRYH-TD-EASD----CS-
NPDF--TIAKQTKWGICWKYTLMCKT--CKFQSA---------E--YKLYKEIK--T---N-----L-P-G---------SNPAA--------
-------------PNYSLAIGLQDT---PIGNTKARLLLAS-MDVPPPAIQSMQTTSNKVSRATT-EL------------NQRDMSAKIQQV-
----KEINVER----GV---EDPSQL----------------------NISMDVRYNSIQITS-RRKPGQNASQAIGLAVETTSDK-------
------------------------------KYIIGAAIQNKLCWKGQW----LR---NRG-YE-VTCP-------------------------
-----DGHE----D---C-TSNLSKPVPL--SEYEIGKTLAEQFL--MD------EIHIKFATTDSDGRGAEGLNDV-------FS-
EVNQDWSVERLADPTHVAQG--QFRKCQSAK----FSDQMFAWK-----R-TKAGQREAQLDLSKD--------------------------
VKSRCSMIYKKMMHDYAG------------------------NMTEIKKKLPK-------------VMEATLRCYA-GDCSM-------
CRRHAI-----------------VCEG------------------------------------------------------------------
--------------GD-Q---KN--------WWNRSF-HLATSKI------------NQLN-MTDKDKDLMLEILK-------------
MRLSEEAFMDMRLNTDTQKNEAVN-------------------RSLSVSLPKNV---------------------------------------
--------------------NF-----------------------------SRNLPG-----------------------RVASTIHRVN--
NTTG------KSTIEKC-T-----NV-GVDLSP--LAKHSL----------IQMEKAEEYKR-------------------------------
---------------EYNKRPEVKNRRIKQLGRDIHAHK---RYKESGG-I--------ETDYKKGQL-------------------------
-----------------------------------------------------------------------------------DH--TY--
SR----------------------------------------------------------------------
>KolobokP-2_MyEd_1p
---------------------------------------------------------------------------------------------
---------------------------------------------------------------------------------------------
----------------------------------MKGG-------K-N-R-----RK-------S-----------CFKA--------
GNRPHN-K----------GIKH-E----------------------------------------TQSSNS----------QPSSSN---T-
QWKRLDAEKF-S-LVAKSGQDGT-------SYSLPDVE----GNPGSA----KLLR----------PQPK--CL-K------VLK----E---
------------------E-AKSES--HD-E---TH--------VTDE--------SYRVVKMNKVTLLLNVVVKFH-NL-QDIK----CD-
NPNF--ELTKLIKYGSCVKCIYKCTE--CKFTSP---------C--VNLFDEIK--T---P-----K-R-G---------PNPGE--------
-------------LTRMLVSALQET---PIGIKRGRFLMAAGLNIPPPTKRTQQRHSNFVANEIK-EL------------NDNDMKKKLETV-
----KEVNRIR----GV---KEPSHI----------------------PVAIDTRYNSMHIVS-TKKPGQNASQAISLACEQVTDH-------
------------------------------KFIVASVFHNKLCWTGSW----LK---GKG-LN-VTCP-------------------------
-----DGHA---GT---C-TANVKPTNPL--SEYLMGKEIGLQID--RQ------NVLVKYAVTDGDGRGAEGINDA-------LK-
VLHPLWKVERQADPIHLGRS--QFRQSNSAN----FSLNMFYG------S-TREKKKQGQKVLSQD--------------------------
LKARCSLVFNEMWKENCG------------------------NLEKIKKCLPR-------------VLDATVRCYT-GDCSK-------
CPTSSY-----------------VCGG------------------------------------------------------------------
```

--------------GH-T---NC--------WWLRSM-YLGANKL------------TNLN-MDDNDKLIMQELLK-------------
MKLSEEALMKMKFNFNTQKCEAVN-------------------RALSVSLPKNV---------------------------------------
--------------------NF-----------------------------SRNFEG-----------------------RASAIIHTLN--
NGIA------NSWKQKA-E-----CI-GFKISQ--GSFKHL----------EQMQRECERNK-------------------------------
---------------VYQNTPERKKKNLQQTGQKIKDHV---EEKEQSK-F--------KDRYQKGQL-------------------------
--------DPK-----------------L-------NTAFVQ-----------------------------------S-TKD-DH--SY--
GRVMPAKKNIPTR--------------------------------SCTSKIPKSKKT---------------
>KolobokP-5_MyCo_1p

----------------------------------MKGE-------K-N-R-----RK-------S-----------CFKP--------
GNIPHN-K----------GIKY-E--TQSSNS----------QLSSSN---I-
QWKRLDAEKF-S-LVAKTSQDGT-------SYSLPDVE----GNPGSA----KLLR----------PHPK--SL-K------ILK----K---
------------------E-AIFES--HD-E---IH--------VKDD--------TYRAVKMKKITLLINVVVNLH-NL-QDIK----CE-
NPNF--ELTKVIKYGSGVKCIYKCTK--CKFTSP---------R--VNLFDEIK--T---L-----K-P-G---------PNPGE--------
-------------LTRMFVSALQET---PMGIKRGRFLMAAGLNIPPPTKRTLQRHSNVVANEIK-EL------------NDNDMKKKLETV-
----KDVNRIR----GV---KEPSHI----------------------PVAIDTRYNSMHIVS-TKKPGQNASQAISLACEQVTDH-------
------------------------------KFIVASVFHNKLCWTGAW----LK---GKG-LD-VTCP-------------------------
-----DGHA---GT---C-TANVKPTTPL--SEYLMGKEIGLQVD--RQ------NVLIKYAVTDGDGRGAEGINDA-------LK-
VLHPLWKVERQADPIHLGRS--QFRQSNSAN----FSLNMFSG------S-TREKKKQGQKVLSQD--------------------------
LKARCSLVFNEMWKENCG------------------------NLEKIKKCLPR-------------VLDATVRCYT-GDCSK-------
CPTSSY-----------------VCGG--
--------------GH-T---NC--------WWLRSM-YLGANKL------------TNLN-MDNNDKLIMQELLK-------------
MKLSEEALMKMKFNFNTQKCEAVN-------------------RALSVSLPKNV---------------------------------------
--------------------NF-----------------------------SRNFEG-----------------------RASAIIHTLN--
NGIG------NSWRQKA-E-----CI-GFKISQ--GSLKHL----------DQMQRECERNK-------------------------------
---------------VYQNTPERKKKNLQQTGQKIKDHV---EEKEQSK-F--------TDRYQKGQL-------------------------
--------DPK-----------------L-------DIAFVK-----------------------------------S-IKD-DH--SY--
GRVMPAKKNIPTR--------------------------------SCTSKVPKSKKI---------------
>KolobokP-3_MoPh_1p

--MFQV--------
GHVPVN-K----------DAV--LQ-KRKIPR-PGRFQKGNTAKR---------------AALDNSTNEEYA-------ASTSNTS---T-
IHASLETHNY-I------------------EDVTPEMH----DDTDEWK---LKLS---HRRC---PGSVN----S------NLK----N---
------------------E-EE-----WT-V---L------------S--------GMRFIDSEEMMKMMNTAYKNH-RA-TSTV----CC-
EPQM--EAYRQKKWGVCWTYYLRCLK--CTYWSQ---------P--FKLYKEVV--N---N-----K-P-G---------PNPGA--------
-------------PNVALATALQDS---PIGNTKTQEILAR-MNCPPPSRSGMNKMSNTVGKKTV-AL------------NKKDMGEKLEMV-
----KAVNKQR----G----TPENQI----------------------NVTVDGRYNSQTIVS-KKKPGQNASQAFSLAIETVTDM-------
------------------------------KFIVAAVWQNQLCWTGAW----LR---GQG-FD-VSCP-------------------------
-----GGHE----E---C-TANHHRAAPL--SEYTMGKEIGSQLA--LQ------NALVKYATTDGDGRSAAGIDDA-------MR-
ALHPMWKVNRLADHVHLGQT--QFRTALRAR----FSEQMFHG------K-TKEEKKELQKILSKD--------------------------
VKCRSAMVLSNLMTKYKK------------------------NIEEICKDLPA-------------VLEATLLCYS-GDCSL-------
CSQHSL-----------------VCDG--
--------------TD-T---SN--------WWLRSK-YLCSYNI------------TVLQ-MEKEDMLLLQEILK-------------
MKLSEXAIRSMKLYDTTNKNEAVH-------------------RSLSVNLPKNV---------------------------------------
--------------------NF-----------------------------SRNMDS-----------------------RLSSGVHRNN--
NRPG------TSALLKS-Q-----HL-GVNLCD--HSTAYL----------KKMDRDFEYKR-------------------------------
---------------RYEKKPEVKKRRLQLSAERLQEHK---AYKELHK-Q--------HPEYKKGQL-------------------------
--------DQV-----------------P-------AIVL---HQ--DY--
CKK--KMPPDAP----------------
>KolobokP-7_MoPh_1p

--MFTK--------
GCVPFN-K----------GVTG-LQ-KRKIPP-AGRFTKNHTRWK---------------GSQKTMTVTAED--------ESSNTS---T-
IYASPVLHEQ-S------------------SHVTPSIH----RDTDEWK---SRLR----------SGTP-----A------NLKREE-E---
------------------E-EE-----CT-V---L------------K--------GMRFVDSEEMMHMMNTVMKQH-RK-TSMT----CD-
EPEM--EACTQKKWGVCWTYQLRCVN--CSFRSE---------Q--FKLYKEVM--N---S-----K-R-G---------PNPAA--------
-------------PNVALATVLQDS---PIGNTKTQEILAR-MNCPPPSRTSMNRMSNTVGKMTV-DL------------NVKDMANKVEIV-
----KEVNRQR----G----APINEI----------------------DVTVDGRYNSQTISS-RKKPGLNASQAFSLAIETVTDM-------
------------------------------KYIISAVSQNQLCWKGAW----LR---GQG-FE-VKCP-------------------------
-----GGHE----E---C-TANHYRAAPL--SEYTMGKEIGNQLA--LQ------NVLVRYATTDGDGRSAAGIDDA-------TR-
ALHPLWKVNRLADHVHLGQT--QFRASMRAQ----YSDGMFSG------K-TKDEKQELKKIFCLD--------------------------
VKCRSSMVVDKLMERYNR------------------------DTEKICKCLPE-------------VLDATLRCYD-GDCSQ-------
CGQHSI-----------------VCDG--
--------------TD-S---SN--------WWXRSK-YLSVYNI------------TALR-MDQKDIVLLQEILK-------------
MKLSENAITNMKLYNSTNKNEAVH-------------------RAISVNLPKNV---------------------------------------
--------------------NF-----------------------------SRNMNA-----------------------RLSSGVHRNN--
NKPG------TSALKKS-E-----HF-GIDLCD--ESCNFL----------KKMDQTFIYKQ-------------------------------
---------------NYEKRPEVKKRPSNKVSRNCKSTR---HTKK---

>KolobokP-2_MyCo_1p

------------------------------MTKSIKTNTSMVCHPS-FFR-----RF-------TLQHQNI--CLNMYPK--------

GYWPFN-K----------GLAGRLQ-RRRVAP-VGRFQNGRIPWNVP------------VPVPVLELSTTP---------DQDINN---A-
PVVEMNVEQK-D---IKDEPTIT-------STSAVHND----GIKTRS----QLLV----------SS-------Y------DLKT---E---
------------------E-ED-----WS-S---L------------S--------GMRFVDHEEMMKMLNIVIKAH-TK-EFKY----CK-
NPEI--LAHRQVKWGVCWKYTLRCKN--CTYMSP---------V--IKLYKEVV--T---N-----K-P-G---------TNPGA--------
-------------PNVAVAAVLHDC---PIGSTKFQELFAR-MNCPPPSRTSMQRMSHNVGKELV-KL------------NRTDMSEKLEIV-
----KSVNRER----G----LQENVI----------------------NVTVDGRYNSQTITS-RKKPGLNATQAFTLAIETMTER-------
------------------------------KYIVASFAQNQMCWKGAW----LR---GKG-FD-VNCP-------------------------
-----NGHE----D---C-TANLYRAAPV--SEYQMGKEIGSQLA--LQ------DILVKNATTDGDGRAAKGIDDA-------TR-
ALHPMWKVERLADYVHLGQS--QFRASLMAQ----FSEGMFYG------R-TKEIKKEMKKAFSQD--------------------------
VKCRSSMIVGQLMEQYKR------------------------NTDDVCKDLPK-------------ALQATIRCYD-GDCSM-------
CKQHSV-----------------VCAG--
--------------DE-S---YN--------WWTRSK-YLGCYNI------------TILQ-MDEKDKLLLQEILK-------------
MKLSEDAISSMKLYNTTNKNEAVH-------------------RSLSVNLPKNV---------------------------------------
--------------------IF-----------------------------SRGMEA-----------------------RLASGIHRNN--
NMPG------TSAKQKC-E-----HL-GVNLSD--SSLKFL----------DSMDKQFTYKQ-------------------------------
---------------EYEKKPEVKRRRLTQSGEKIVEHR---EAKVKSK-E--------RDVYRKGQL-------------------------
--------DPV-----------------P-------ALLS---HS--TY--
CKR--KLPP-AP----------------
>KolobokP-7_MyEd_1p

---MIDSIIMP--
------------I----NN-----QHQSYIMQKSIKTNNFKVCHHS-SIR-----RF-------TL-FQNILFIIKMFPK--------
GSRPFN-K----------GLIG-LQ-RGRVAP-GYKYQHNRTPWN---------------KVPVSQLSTSL---------DPEENH---V-
PAVEMNVEPP-N-AILDESPCTS-------STSPADND----RKQTRS----QLLV----------PS-------A------DLK----T---
------------------E-EE-----WC-S---T------------S--------GMRFVDHEEMMKMLNTVITIH-TK-KFRH----CK-
NPEI--LAHKQEKWGVCWKYTLRCKN--CTYMSP---------L--MKLYKEIV--T---T-----K-P-G---------TNPGA--------
-------------PNVAVAAAIQDC---PMGSTKLQELLAR-MNCPPPSRTSMHRMTHNVGKELV-QL------------NTRDMAEKLEIV-
----KAKNQER----G----LAENEI----------------------NVTVDGRYNSQTITS-RKKPGLNATQGFALAMETTTEN-------
------------------------------KYIVASFAQNQMCWKGAW----LR---GKG-FK-VECP-------------------------
-----NGHE----D---C-TANLHRAAPL--SEYEMGKEIGTQLA--LQ------DILVKNATTDGDGRAAKGIDDA-------TR-
ALHPMWKVERLADYVHLGQS--QFRASLRAK----FSESMFYG------R-TKEIRKEMKKAFSQD--------------------------
VKCRSSMIIGQLMEKHNK------------------------VTEDVCKDLPK-------------VLEATLRCYD-GDCSM-------
CKQYSV-----------------VCTG--
--------------DE-G---YN--------WWTRSK-YLGCYNI------------TVLQ-MDEKDKLLLQEILK-------------
MKLSEQALNSMKLYDTTNKNEGVH-------------------RALSVNLPKNV---------------------------------------
--------------------IH-----------------------------SRGMQA-----------------------RLASGIHRNN--
NKPG------TSAKMKC-E-----HL-GVNLSE--SSLQFL----------SKMDSDYTYKQ-------------------------------
---------------EYEKKPEVKRRRLIQTGEKILEHR---EAKQSRK-K--------CDVYRKGQL-------------------------
--------DPV-----------------P-------AL---
CKR--APPP-AP----------------
>KolobokP-101_PeVi_1p

--VF---------------

---H-LH-ARVV----CR-ETPT--E-

--VMV-KQ------------NKDDMKDKVEQV-----
KAYNKKK----G----QPENVI----------------------DVTLDGRYPTATRSS-RKKPGLNASQAQMLAIEQNTPW-----------
--------------------------KFVVAASSLNQLCWKGAW----LR---GKG-FD-AECP-----------------------------
--GHE----G---C-TATLHRTIPI--SEYAMGKEIGNQLA--LQ------GMLIRYATTDGDARSAKGVEDA-------IH-
VLYPMWEVQRLADPVHLSQH--QFRAGMRAE----FSETMFHG------Q-TKEENKDLKKMFSLD--------------------------
LKCRSSMILNQLMNEYNK------------------------DTEKVCKKLPT-------------ILSTTVQCYG-GDCSS-------
CLKESV-----------------VCKG--
--------------TV-S---DN--------WWTRSF-YLSTYNI------------TVLK-IDEIDEIILLELLK-------------
MILSEEAVNSMRMFHTTNKNEGVH-------------------RALSVSLPKNV---------------------------------------
--------------------TH-----------------------------GKNMEG-----------------------RLHSAIHRTN--
NRPG------TSAKMKS-K-----EL-NIKVSE--DCETFF----------DKMDREYTYKR-------------------------------
---------------TYEQKPEVKTRRLQQAAVRLEEHK---IHKELHK-K--------RPDYVKGQL-------------------------
--------DPV-----------------P-------DELM---HD--AY--
CKK--KMPK-------------------
>KolobokP-1_MyCo_1p

---MPSVQVVIL---
---RIQL--NF-------K-----------MFTK--------
GYTPYS-K----------GKKR-SK-KDNSPRNPGWFPVGI-GGR-----------------SRSRTSIQIP--------DSASAS---T-
SYVR-------Q------------------TLQSPDCE----KATGHI----CNLR----------PQAN-----V------NLK----I---
------------------E-EN-----NA-L---H------------S--------GMRWIDSQLTLSLINTTYDKH-QV-ESPS----CP-
RFNV--DYHAQKKWGTCWQYTLKCIT--CGFIGD---------R--MKMYKEIA--N---G-----K-P-G---------PNPGQ--------
-------------PNVALASALQDC---PIGNTTVQQLLAG-VDTPPPCRSSMQRTSNRVAAEMV-KL------------NKTDMAQKLEQV-
----KEVNRKR----G----VPENEI----------------------NITVDARYNSNTIVS-KKKPGQNATQAFALGIETMTDR-------
------------------------------KFIVAAVVQNKMCWKGAW----LR---GKG-FP-IECP-------------------------
------GHE----E---C-TANLYRAAAL--SEYELGKEMGNQLG--LQ------KFLVRYVTTDGDGRSARGIEDA-------IK-
ALEPMWEVERLADPVHLGQS--QFRASNRAQ----YSAGMFHG------K-TKEENRQLKTVFSKD--------------------------
IKCRCSMIINKLMEKYDK------------------------NIYDMSKDLPK-------------VLDATLRCYD-GDCTL-------
CQEHSI-----------------VCKG--

--------------DA-A---LN--------WWSRSH-YLSTYQI------------TALQ-MDKTDKFLLQEILK-------------
MKLTKAAVESMKLYDNTNKNEGVH-------------------RAMSVNLPKNV---------------------------------------
--------------------IY-----------------------------SKNMEG-----------------------RLASGVHRNN--
NMPG------TSTQLKC-K-----NL-GVDLST--RSQLYL----------KKMDTNFKYSQ-------------------------------
---------------DYELRPAVQERRLKQNAEKLAEHK---TWREMNK-K--------HDHYCKGQL-------------------------
--------DPQ-----------------P-------ALQFQ--DHD-SY--
CKP--KLPRPAA---------------D
>KolobokP-6_MyEd_1p

--MFTK--------
GHTPYS-K----------GKKW-SK-KDNSPRNPGCFQAGKPSGR-----------------SRSQTSIQIP--------ESASAS---T-
SYVR-------Q------------------TFKSSDCKM---ETTGNI----CTLR----------PLDN-----A------DLK----R---
------------------E-EN-----KE-L---H------------S--------GMRWIDSQLNISLINTNINKH-QV-DSPS----CP-
MFIA--DYYEQKKWGTCWQFTLKCIA--CGFIGD---------R--MKMYKEIA--N---G-----K-P-G---------PNPGQ--------
-------------PNVALASALQDC---PIGNTTVQQLLAG-IDTPPPCRSSMQRTSSRVAAEMV-KL------------NKTDMAQKLELV-
----KEVNRRR----G----VPENEI----------------------NITVDARYNSNTIVS-KKKPGQNATQVFALGIETITDR-------
------------------------------KFIVAAVVQNKMCWKGAW----LR---GKG-FP-IECP-------------------------
------GHE----E---C-TANLYRAAAL--SEYELGKEMGNQIG--LQ------KCLVRYVTTDGDGRSAKGIEEA-------LA-
AVEPMWKVERLADHVHLGQS--QFRASLRAQ----YSPGMFHG------K-TKEENKQLKTVFSKD--------------------------
LKCRCSMILKKLMEKYDK------------------------NINEISKDLPK-------------VLDATLRCYD-GDCTQ-------
CPEYSI-----------------VCKG--
--------------DD-A---LN--------WWSRSH-YLSTYQI------------TALQ-MDKTDKFLLQEILK-------------
MKLTTAAVESMKLYDNTNKNEGVH-------------------RALSVNLPKNV---------------------------------------
--------------------IY-----------------------------SKNMEG-----------------------RLASGVHRNN--
NMPG------TSTQLKC-T-----NL-GVELSE--RSKRYL----------KKMDTDFTYNQ-------------------------------
---------------DYILRPEVQKRRLQQNAERLAEHK---TWRELNK-K--------HDYYCKGQL-------------------------
--------DPQ-----------------P-------ALQFQ--DHD-SY--
CKP--KLPRPAA---------------D
>KolobokP-4_CGi_1p

--MFQK--------
NQRPWN-K----------GREL----RQRKPPSRRFFRQGNTNR--------------------SKASSED---------QETTRK---R-
SFVRLSAGDH-A-LVTRPSLDCQ-------SYITPDCD----GESGSS----CILR----------PRSE--SC-R------YLQ----N---
------------------A-NS-----NE-Y---E------------G--------GQRVVSEEKMIDMMTSTAHIH-QT-TSST----CP-
MPNF--AVLEARKLGISWKYKIKCAN--CPFTSP---------T--FNLYHEIH--Q---E-----G-P-G---------RNPSA--------
-------------TNIALAYAIQDT---AIGAEKIIELLTC-LDIPAPSKSFMQTLLCKTSRKMT-EL------------NKLDMNDKIQKV-
----KEVNVTR----G----HPENII----------------------NISTDARYNSSVMFS-RKTPGQNASQAFSLAVETNTDR-------
------------------------------KYILACAVQNKLCWTGAW----LR---GKG-ME-VDCP-------------------------
-----GGHA----E---C-TANLSPAAPF--SEYEMGKDIGTQLG--LQ------DVLVRYVTTDGDAQGAKGVDDA-------MR-
ALHPLWKVERLADHVHLGQS--QFRASNRAV----YSDEMFHS------K-TKEEKKELQRVFSND--------------------------
LKSRCSMIVKKLFTKYDR------------------------DLEKMSSFLPK-------------VLEATVACYD-GDCSM-------
CAAYSL-----------------VCEG--
SGLQLVELAVSYPKSWRQLLHVTTETVLCVPPIPWSVRGV-V---SN--------WWNFSR-NLAIYRI------------TALQ-
MNDNDLRIVTEVLK-------------MKLSVEAISAMKLLTDTNANESYH-------------------RSASANMPKNV------------
---KF-----------------------------SRTMEG---------
--------------RLHSMIHRRN--NLPG------TSTRLKC-E-----SA-GVQYSD--SGKRRL----------ARIDQQFLYRQ-----
---EYHKKEKIKTRRNKQQGRKMEEHH---LYRQEHR-T--------
KSDYRKGQL---------------------------------DMNL----------------P-------AA---------------------
----------------------R-DH--PY--HRRGA---CQYHLRP-RPGC---
----------D
>KolobokP-1_CHo_1p

--MTRYK---R-S------

---K-----
KLINITR----G----HPENII----------------------NISTDARYNSSVMFS-RKTPGQNASQAFSLAVETNTDR-----------
--------------------------KYILACAVQNKLCWTGAW----LR---GKG-ME-VDCP-----------------------------
-GGHA----E---C-TANLSPAAPF--SEYEMGKDIGTQLG--LX------DVLVRYVTTDGDAQGAKGVDDA-------MR-
ALHPLWKVERLADPVHLGQS--QFRASNRAV----YSDEMFHS------K-TKEEKKELQRVFSND--------------------------
LKSRCSMIVKKLFTKYDR------------------------DLEKMSSFLPK-------------VLEATVACYD-GDCSM-------
CAAYSL-----------------VCDG--
--------------GV-V---SN--------WWNFSR-NLAIYRI------------TALQ-MNDNDLRIVTEVLK-------------
MKLSVEAISAMKLLTDTNANESYH-------------------RSASANMPKNV---------------------------------------
--------------------KF-----------------------------SRTMEG-----------------------RLHSMIHRRN--
NLPG------TSTRLKC-E-----SA-GVQYSD--SGKRRL----------ARIDQQFLYRQ-------------------------------
---------------EYNKKEKTKTRRNKQQGRKMEEHH---LYRQEHR-M--------KSDYQKGQL-------------------------
--------DMNL----------------P-------AA---R-DH--PY--
HRRGA---CQYRLRP-RPGC-------------D
>KolobokP-1_MoPh_1p


```
---------------------------------------------N-N-K-----RK-------I-----------MFKK--------
GGTPWN-K----------GRTG-LI-SMKIPK-KSLFVKNSTPW--------------------NKGTEVPW--------DKSEPE---N-
SYQRMESKEY-E-LVVKPKMDGK-------LLSTPDCD----GKSGPL----HLLR----------PKAP--PK-Q------DLK----V---
------------------D-MK------N-L---C------------E--------GMSYVDNEELMTMINSAIQLHIST-ESTS----CQ-
VPCF--NYYNIQKWGICWKCQLYCEN--CNLITP---------V--YKLYKTIP--S---N-----K-P-G---------PDPAA--------
-------------PNVGFALGLQET---AIGNTKAQHLLSN-INVPPPSRSSMQRTSNAVSLKTI-EM------------NKRDMSEKLEIV-
----KSVNRKR----G----VPENEI----------------------NVTVDARYSSNTIIS-KKKAGQNANQAIALGIETVTDR-------
------------------------------KYVVATASLNRMCWTGAW----LR---GKG-FD-IECP-------------------------
-----GGHE----E---C-TANLYRAAPL--SEYELGKQIGTQLA--VQ------GILVKYATTDGDGRSASGINDA-------IQ-
ALNPLWTVERLADPVHLGNS--QFRSAYHAC----FSENMFYS------R-TKEQKK------------------------------------
---------------------------------------------------------------------------------------------
---------------------------------------------------------------------------------------------
---------GN-A---KG--------IQPRY--------------------------------------------------------------
---------------------------------------------------------------------------------------------
---------------------------------------------------------------------------------------------
---------------------------------------------------------------------------------------------
---------------------------------------------------------------------------------------------
---------------------------------------------------------------------------------------------
------------------------------------------------------------
>KolobokP-2_MoPh_1p
---------------------------------------------------------------------------------------------
---------------------------------------------------------------------------------------------
----------------------------------------------------------------------------MFQK--------
GRTAWN-K----------GVSN-LTKSRKKHN-SSHFKKGSTPW--------------------NKDSKVAW--------DTTKPD---T-
TYERMESAEY-D-LVVKPKMDGM-------LLTTPDCD----GNLGSM----CVLR----------PKSS--PT-V------DLK----A---
------------------K-IN-----MN-L---A------------A--------GMSYVDNEELMSMINSAVQQH-AM-KSMS----CS-
IPSF--QYYNMKKWGVCWKYQLYCVH--CQFVTP---------E--YNLYKSIP--S---K-----K-P-G---------PNPAA--------
-------------PNIGLALGLQET---AIGNTRAQQLLSN-MNCSPPTKKSMQRMSNQVAKRTV-EL------------NKNDMAEKLELV-
----KAVNRKR----G----APENII----------------------NLTVDARYSSNSIVS-KKKPCQNPNQAIALGIETVTDR-------
------------------------------KYIVATAALNQMCWTGAW----LR---GKG-FE-VQCP-------------------------
-----DGHE----E---C-TANLHRTKPL--SEYDLGKQIGSQLA--VQ------GILVKYATTDGDGRSASGINDA-------IK-
AFHPMWEVERLADPIHLGQA--QFRASMRAN----FSTGMFHG------K-TKEQRKRLQKIFSQD--------------------------
LKARSSLVIDNLMKNHGN------------------------QIQKVAKDLPK-------------ALESTMRCYD-GDCSM-------
CASHSV-----------------VCAG------------------------------------------------------------------
--------------GE-T---KN--------WWNRSI-FLTSHQI------------TSLQ-MDENDKKLLLEILK-------------
MKLSLSALETMKLFDNTNKNEAVH-------------------RAISASLPKKC---------------------------------------
---I-----------------------------------------------------------------------------------------
---------------------------------------------------------------------------------------------
---------------------------------------------------------------------------------------------
---------------------------P-----------------------------------------------------------------
---------------------------------------------------------------------
>KolobokP-1_PeMa_1p
---------------------------------------------------------------------------------------------
---------------------------------------------------------------------------------------------
---------------------------------MKGR-------K-K-R-----LK-------S-----------AFQY--------
GNTPHN-K----------GKTT-A----------------------------------------GHGVSKV---------LTKNES---G-
TWKRLSPNVF-S-LVTVTRPGSN-------FPSTSDTE----GNPGNV----KLLR----------PRKT--KA-T------DLE----A---
------------------L-DL-----HE-N---MNKKPRLQTRIRNQ--------GNRLINTEKIGDLMQII--------SSHR----CD-
NPDV--DIYNEIKIGVTCKFQVSCKT--CGWVSD---------T--HKMYNEVQ--S---P-----K-R-G---------PNPAV--------
-------------PNLTMHAGLQET---PMGPAGARRLFNS-MNLVPPCRSGMQRNANMVGEKII-SL------------NKEDMANKAKEV-
----REICELR----G-----DPNMV----------------------NVQVDGRYNRSTITC-RNKPGTSAVQLALIAREDITDK-------
------------------------------NYILGIAVQSQICWKGKW----LR---LKG-FD-AKCP-------------------------
-----GGHI----D---C-SATIGEGTPL--SEFEAAKLVGEQLY--QQ------DLIVRYATSDGDARSVAGLDAA-------YK-
NFIPMWKVQRLADPTHLGQA--QFRKCIRAH----FSSDMFPGM-----S-TREGKTESQKVLSRD--------------------------
IKARCSLICKELTKDCTG------------------------ETKIMREKLPH-------------VLDATLRCYS-GDCSR-------
CKRYSY-----------------VCNG------------------------------------------------------------------
--------------GVGS---GT--------WWNRSI-FLSKHKL------------FSLN-PDENDKQLLLEILK-------------
MKLTDECITGMSLGANTQRCEAFF-------------------RLLNSRLPKNV---------------------------------------
--------------------LY-----------------------------SKNMNA-----------------------RFNGAVHTAN--
NGVG------NSLMKKM-E-----CL-GAAVAP--EVRRSL----------RQMQSEQNYHQ-------------------------------
---------------EYQKTPAFKKRTLELKAEAIQRHI---KNKG----A--------RSDYKKGQL-------------------------
--------DTY-----------------P-------DLSTSR---------------------------------LSS-NND-EH--NY--
HK----------------------------------------------------------------------
>KolobokP-1_MiYe_1p
---------------------------------------------------------------------------------------------
---------------------------------------------------------------------------------------------
---------------------------------MKGD-------T-K-R-----RK-------N-----------HFQP--------
GNIPKN-K----------GQTL-M----------------------------------------PVEDQAG---------HPNDRT---V-
FTRRLTTEEF-K-LVTKPSHDGR-------SLIASDTE----DRPCPA----RILR----------PSTV--PQ-E------D------S---
------------------D-SE-----PD-N---I------------E--------GNRIIDVLLMVKMWNSVFRLH-AEKHHET----CK-
IPEF--ELDTESKRALGWSCSVRCAK--CSYRSP---------L--HKLYREAD--T---N-----K-P-G---------PKPAL--------
-------------VNRYLPSALCGQ---SVSTKGARLLLGH-LNIPAGAKSGMQRQANLVSKEIT-AL------------NKIDMAEKTRQV-
----VEVNHLR----GD---ANPSTI----------------------GIALDGRYNSTSFGS-AKKPGMNASQGMSLGIETSTPK-------
------------------------------KWIVARTLQNKLCWTGAW----LR---GKG-YD-VNCP-------------------------
-----GGHP----D---C-TANTDRYDGL--SEHAMGKNIGDQLT--SQ------GILVKYATTDGDGTGAKGIEEA-------MQ-
ALDPMWKVERLADPTHLGKA--QFRKSNSAK----FSESMFPG------R-TRLMRAHSQKIFSQD--------------------------
LKARSSLIFKDLMKLHNG------------------------NMDIITKRLPA-------------VLDATVACYS-GDCSK-------
```

CKQHSV-----------------VCSG--
--------------GD-S---NN--------WWTRSM-FLSANKI------------HGLQ-MTSDDEMLVLELLK-------------
MKLSTETVQSMRLSTSTQKNEGCH-------------------RTLNVSLPKYQ---------------------------------------
--------------------NF-----------------------------GRNAEG-----------------------RLDNTILTIN--
NTAG------KATQQKV-D-----HL-GGTLSK--EVRHQL----------DQISSEEMYQR-------------------------------
---------------QRQYKPDVKKRNLELLVEQHREHA---NRSKSA--R--------KSDYRKGQL-------------------------
--------DPT-----------------L-------HRTRG-----------------------------------DN-QPQ-DH--LY----
----

>KolobokP-2_PaGe_1p

----------------------------------MKGE-------R-K-R-----KH-------A-----------MFKK--------
NCTPYN-K----------GLVR-LS---------------------------------------SASSSNS---------SDNMAA---E-
RSIRRSTTSL-S-LCSQTSTSTS--------------------QEADV----LVLR----------HRPE--TV-N------KTQ----D---
------------------I-NR-----EA-E---H------------D--------GNRIIDNDKMMEAFNDCTQSH-Q--ESAT----CT-
KPHF--AIHSNEKQGLAWKYTFRCVN--CTYKSK---------K--YKFFKEIV--T---R-----L-P-G---------PNAAA--------
-------------INVSFQAGLQDT---PIGVTRSRLLIAS-MDIPPPTKNAMQKTANKVARETV-KV------------TKLDMEEKLQLL-
----QTTLSDL----G-----LSGEI----------------------HGSFDGRYNSLTIVS-CTKPGHNSSQAIGVFAESMTEN-------
------------------------------HFILDYEIENKLCYVGAH----LR---SKG-YD-VNCP-------------------------
------GHA----D---C-TATLPNVAPH--SEYEMAKSIATRLG--QK------GVKVSILTTDQDAQAQKGFQEI-------YE-
TMFPGHKILQQADPAHIGRS--QVRKCISAT----FSKDMFVGC-----S-TREASKKAQTIFALD--------------------------
VKSRCSLIVKKVLELYCG------------------------DLPSMIKAVKK-------------ALKSTIQCYD-GDCTT-------
CRRNSF-----------------VCGG--
--------------GQ-K---SC--------WFERSA-FLAPHKI------------TTLR-MAESDKELLEDILK-------------
IRLCEKAIESTQFNYNTQSVESFN-------------------ATLSKSAPKNV---------------------------------------
--------------------NF-----------------------------SRNYGG-----------------------RCASAVLRHN--
NGQA------TALIKKL-E-----YM-GIYLSK--RTGQAV----------SKIGADMRSHR-------------------------------
---------------KYRKDKQYNRRIILRRGEMESRHR---WYRRLKNNR--------QSDT------------------------------
----

>KolobokP-1_ParEch_1p

---MC--
---------------DVLDKLQP------------------NAIPK-H-D-----RK-------C-----------GFRK--------
GHAVYK-T----------RTES---SASALGAV-------QESGENLA---V-
WMPRLTEADF-E-QVTKVTPGG--------LIEIPDAE----GRSGEA----KLLR----------PKHC--AP-NANDDDDDLSLTYLK---
------------------A--------DD-T---H------------E--------EMRLYDKGKMQYMYNECILQH-ST-QKNM----CA-
IPEF--TIAREVKVGLCWQCCLRCTK--CGFMSG---------I--YKLYNEIE--T---G-----K-R-G---------RRPAA--------
-------------PNVGLQVGLQES---TTGNVKGRLILTC-LNLPVPNRSVMQRTANKVATATA-TM------------TLADLAKKRENV-
----KRVNRLR----GL---PENSPI----------------------NIAMDSRYNSATITG-AYHAGQNASQAIAVAVEKQSGR-------
------------------------------SDIVGISIQNKLCSLGAS----LR---RRG-QD-VTCP-------------------------
------GHA----N---C-TATLPADEPL--SEYTAGVDIGRQFA--SQ------NVGLRYVVTDGDARSAEGVKAG-------MS---
ETACEVERQADTTHLEQS--LFRNSMKAQ----FSARMFPG------S-TAVIRKEQKKMFSLD--------------------------
VKNRCHRIHSEMQNMHAG------------------------DSRKVASRMPR-------------VIETTLDCYG-GDCGK-------
CRFNSV-----------------VCAG--
--------------GK-K---KN--------WWHRSM-YLQTGRI------------HHLN-MTDADRVTLRKLIE-------------
MRLGIQALAITKLCLNTNRNEALN-------------------RSLSSTLPKNV---------------------------------------
--------------------NF-----------------------------SRNVTG-----------------------RACAAIDRLN--
YGAG------TSILRKL-E-----LN-NAPISKGGSVARAT----------RQIQREALYHR-------------------------------
---------------TYMYRRSVVRRLLYNKRKRFDAIR---AARRRREATAQTRADARKRGYRKGQL-------------------------
--------DQQ-----------------V-------KKKIKTKKSAV---------------KPNKTISDHPYSLRDR-SID-DH--TY----
-----------------------------PYDI-------------------------------------
>KolobokP-2_ParEch_1p

---MKETANYET---
-----------GKLSKRSDRKTP----------PVRGM-------R---R-----NR-------R-----------GFQQ--------
GHPHFE-A---------MHGES---TETPHSH---V-
WMPRLGMADF-S-RVVQETSGG--------LLTVPDAD----GRPGNS----KILR----------PRPY--RC-P------DLTDSYLQ---
------------------P-DE-----NQ-G---G------------S--------EMRLLNMEKSAQMWNECYTEH-AC--KTT----CT-
SPQF--QIYHEQQIGLCWKQSLRCTN--CDYHSR---------M--YKLYTEVS--T---G-----R-S-G---------QRAAT--------
-------------TNVALHVGLQDS---TIGTTKIRHILAA-MDTPPPSRTGLQRTANKVATVTA-QA------------TMDDLHRRRQKT-
----KETNTLR----GL---PEDAPI----------------------NISVDVRYNSTNLKNTYSCGGQNASQALGTAISWQTGE-------
------------------------------KEIIAFWMDSKLCKLGAS----LR---NKG-VN-VTCP-------------------------
------GHA----G---C-TANVQATDPL--SEYRIGKKIGDSLA--QD------DVAIKYVATDGDALASRGVQDA-------MP----
AGMETQRQSDTTHLSQT--QFRHIMKAS----FSPRMFPG------E-TAARRAENRKMFAED--------------------------
VKTRCQKIYTSVHILHDS------------------------DTTVITRRMPD-------------IIQATLDCYS-GSCRN-------
CRRHAI-----------------VCRG--
--------------GR-K----N--------WWNTSQ-HLKACGL------------TRLN-MTDSDRATLQGLIE-------------
MRLGNAALQMTRRRLTTNPNESAN-------------------RAYSASVPKNV---------------------------------------
--------------------KF-----------------------------SRNALG-----------------------RVCSVIDRLN--
YGAG------DSMLRKL-E-----NV-QCPITKGGRVAHAV----------KRIQQDVAYQR-------------------------------
---------------QYARRPSVRLHRRATKSKRMRDYI---RAKRQRC-Q--------DDIYRKSQL-------------------------
--------DPK-----------------P-------ASHTVT-TVAVTATAVTPVRRRRRQ--PPGNRTDHPYSLRSR-KSA-DH--SY--
ASRE--
>KolobokP-3_ParEch_1p

---MKETTN--

------------EISKVSDKKPP----------PVRGK-------T---I-----NP-------C-----------GFQH--------
GHRYFK-P---------TRAET---SETQAGR---V-
WMSRLHMDDY-S-RVVQETAGG--------LLTVPDAD----GVCGNA----KILR----------PRPD--KC-L------DLTDSYLQ---
-----------------A-DK-----NH-K---D-----------S--------EMRLLNMKKTADMWNDCYAEH-A---KTT----CT-
SAHF--EVHEERQIGLCWKQSLRCTH--CDYHSR---------M--YKLYTEVN--T---G-----R-C-G---------QRAAT--------
-------------TNVALHVGLQDS---TTGTTKIRHILAA-TNTPPPSQSGLQRNANKVATLTA-QA------------TMDDLHERRRKT-
----KATNILR----GL---PEDDPI----------------------NISVDVRYNSTNLKNSYSCGGQNASQAVGTAVSWQTGN-------
------------------------------REIIAFHLDNKLCKLGSS----LR---SQG-MD-VTCP------------------------
------GHA----G---C-TANVQATEPL--SEYRIGKKIGDSLT--QD------SVAIKYVATDGDALASRGIQDA-------MP----
AGTQTERQSDTTHLSQT--QFRHIMKAS----FSSRMFPG------E-TAAVRTENRRMFAED--------------------------
VKTRCQKIYTSVHMLHDS-------------------------DATVIASRMPD-------------IIQATLDCYS-GTCKN-------
CRRHGI-----------------VCRG---
--------------GR-K----N--------WWNTSQ-HLKACGL------------RRVN-MTDSDRATLQGLIE-------------
MRLGNRALQMTRRKLTTNFNEGAN-------------------RAISASLPKNC---------------------------------------
--------------------KF----------------------------SRNALG------------------------RMSSVIDRLN--
YGAG------ISMLRKL-E-----NV-QCPITKGGRVARAV----------KRIQQDVLYQR-------------------------------
---------------EYSRQPSVRLHRRRTKSKRMREYL---EAKKNRR-Q--------DDIYKKSQL-------------------------
--------DPE-----------------P-------ESRTSTLSRDVTATTVKPNRRRRRQRKPPVNRTDHPYSLRSR-QSA-DH--PY--
ASRE---
>KolobokP-3_LiLo_1p
MT-------------S--VMAKVFV---------YL------------SSSSIQRS--------TMM--------------------------
-----MNH---P---NILKCQLGMLWGSLYM-----WTPQSKLQL---
---------------------------------KMPKL-------S-K-K-----PS-------R-----------GFQV--------
GHSYHS-P----------YSRT---MPPEKTN---G-
SWSRFSNDDY-D-NVVRERSDGI--------IYAPTVE----NTVSPL----KILR----------RGQD--AA-K------DNTYV------
-----------------------------------I--P------TED--------TNIVASKFKILEMLNGCSELH-FN-FIGH----CD-
IPKF--EIEHDIKKGASARWALRCAN--CNFACG---------KHYFKLYNEIE--K---Q-----G-R-G---------AKQAE--------
-------------INVALALASQEI---G-SQSKTRTLLSI-ANVPPMSKSSLQNNVNRVGMETV-EL------------NKDDLKRQQNYL-
----KEVKRMR----C-----DQSGV---------------------SVQMDTVYNSFTIAR-RSRPGQAATQAVGLVRENETPS-------
------------------------------HKIIAVGILDKLCYKGAW----LL---GRG-FQ-PKCGT------------------------
----DEAHD----G---C-KANLPDYQVL--REYDIGNMVGLELA--AN------HLLVHCCTTDGDGKCSAGMEDA-------
FSTFLGKALKVERMSDTYHLGQA--QFRKGMRAQ----FSKGMFVG------M-KAPDAAEAKKCLSSD------------------------
--IAIRASTIFDTLFKKYHG-------------------------DTDQIGENLAT-------------IVDNLIACFD-GDCSQ-------
CSIETT-----------------VCDG---
-----------------Q---GS--------WWSACK--LSNLELSA----------KDLN-MSDADKLLVRSILE-------------
MKLSKTCLKKIRHATNTQACESAN-------------------RQLNLIAPKNV---------------------------------------
--------------------TH----------------------------LKNYRS------------------------RVHAAIHTMN--
NTRG------KSIPLKL-K-----RL-GARVSA--AAMNCL----------KEMDNRKWYYK-------------------------------
---------------KWESELKTRIRRRVVRTIRTRAFY---QAKRRGVVK--------DIDYHKDRL-------------------------
--------ELEGGEEEG---------GLK-------ERAKRL--------------------------------------ARDYNH--HF--
RQYT--AISSSSETI--------------
>KolobokP-1_LiLo_1p
MC---
-----------------------------------WGYQASRMV---
-----------------LTRRRR-------YDCQKPGI-------T-K-R-----RD-------T-----------FFKK--------
GHVPYKSK---VDFRASDP---G-
EWLRLPLDLY-N-QVTTKGPGG--------TRIVPDIHN---RPSGRT----RLLR----------PIKP--PSDQ------DVQNHFEP---
---------------SSYN-PS-----CS-M---T------------D--------ENYVISVEKMMDLMNSVAVSH----NMTG----CV-
RPEF--QYEDKVKKGLCVTVRVKCKN--CKYISD---------K--NKLYSELA--S---A-----K-A-G---------GKSAI--------
-------------PNISLQCALLDS---SIGNFKGSQILNT-IGLPTPCLSSMQEQANRVGKKIV-EL------------NVEDMANIRGSL-
----REVNRLR----GQ---EDVNAF----------------------TASMDGRYNSQRITT-RGKLGQAASQVVEIATENLTDS-------
------------------------------KKVIDVAFFNKLCWRGAY----CR---ARG-LP-
IHCGSAPPKSNSKSSKASRPTTRSSARESNEGAEQFHK----N---C-TANFPEVLPF--SEYELARTITANLA--KE------
GIHLEYVCTDGDSSAYRGVRDA-------TDMIFGTLFNVKPQTDSIHLGQA--MFRAGLKTD----FSKNCFPG------R-
TADDRKTFQKLFMLD-------------------------VKSRSSRALKLLRNDVGG-----------------------
DVVKMSKKLVP-------------LVQCLIDCYS-GDCSK-------CRNNKY----------------NLCSG--------------------
--GI-T---NS--------WRKKSE-
YFKAYGKKS----------LDIS-FNETDRKLLQILLE-------------VRLGESALESLKTGVTTNKPEATN------------------
-RGISSSLPKNI--TF--------------------
---------SRNALP-----------------------RVHSAIHRVN--NSLA------KSTMTKLDK-----GL-NVPLNPQSHAMKNL--
--------GRLQNRKAYNV--AYKESPEFKKQNKRQRIKRMGDFL---
TDKRARK----------EGDYQKFSQLLIPIPGVAAGSSKSRGRGMPTPTNITKPKGKKDKKIHKEH------------P-
YSQAPSNESRSTVGGYVGG---------------------------EDR-RRR-DH--TY--SSDPVQSTSSQGP------------------
-------------TVRKKGPK-------------KYQTKK
>KolobokP-2_LiLo_1p

--MRTKAR---
------------------------------RLIGKRGS------PR---R----ARA-SNAAMRT-----------MFKS--------
GHVRSG------------GVEV--VSDDEREVGLQ---P-
AWRRLTPQEY-G-QVTTMGHDG--------KRHIPDINN---RTAKNV----NLLR----------LNMK--ED-T------DDEMEIPD---
--------------------ET-----MEHD----------------H--------ESFVVQTTKLMDVLNDVNRAH-SE-YHVN----CQ--
PIF--KYGEVRKQGVCCSVTVVCKN--CGFYSK---------L--YKLFEDID--T---G-----K-R-G---------RKAAL---------
------------PNVALQIALQDT---MIGNESMSYILSM-MGIPPPCRSIMQKQANKVGDETV-KM------------NVADMADIRENL--
---KRILRLR----N----QDDTGI----------------------NVQADGRYNSLHLTS-RNKMGRCASTMTGLCVENVTDE--------
------------------------------HKIIDIEVKTKLCWIGAA----MR---AKG-FE-VKCGA------------------------
TGTNVDERHS----K---C-TANFPEADAF--REYDLGVAFAETFE--KQ------GLHVEHLTTDGDSSSFKGLTDA-------
TKKLFGGLFTVKHQLDSIHLHQA--QTRAGHRAE----FSKGMFPG------R-TAEERTLLKTTFLND------------------------

--LRVRSSKILQSLFKRCNG-------------------------DLDRIMGYLYD-------------TVACVVNCYG-GDCTG-------
CPQNEF-----------------SLCSG---
---------------TT-----GS--------WWHKST-RWSSYGEI------------PKFK-MTVIDKNLVHLVLE-------------
VCLSKGALQQLSLGVTTQGVESKN-------------------RGLSASDPKIV---
---------------------TR-----------------------------SRNSTP------------------------RIHSAAHRMN--
HNIA------ESGRRKL-K-----WF-NIEVDTCCRPMKAL----------DRIAMNRQYTV--------------------------------
---------------RYQRSARHQKLAGVLKNIRATDYF---TKKKNKT-R--------GGDYKKHVM-------------------------
--------ITM-----------------P-------DLRRKAVKKAVA-----------RRDRPPVP----EWKCLDPNSRNYDH--SY--
FRRSFKIQPATKT-----------------------------------SKRLPS---------R---------
>KolobokP-2_OwFu_1p

---MS----RD---
-------------------------------LSSTHKGLAY-----K-K-RQSPAGKK-------S-----------RFTK--------
GHAHIK-P----------FTKS---KPFSGDHSHSIP---D-
TIERFDEDTF-R-NVATTSRDNQ-------RYENLDVN----MNFGNA----RLLR----------PCKS--RK-A------DLTNTMAE---
------------------A-PP-----GA-T---P------------N--------DCIIVSTAQTLIMINDLNNEH-RK-HSIA----CE--
GNY--NISRIASFGLGTKLKFACSS--CHFQSK---------L--YKTYDEVD--N---SYYKGNI-K-G---------PRPAK--------
------------TNVALQVALKQS---SIDSKQVRRIFAA-LNIKPPSKTHMFQTSKKVDERIT-EL------------CQQDMSKLRQNL--
---KEINKMR----GVA-EQDQNKI----------------------AAAFDAQYGSVKFGS-RNKLGPDANRAIGLTVETMTEN--------
-----------------------------LVIIDAVLQNKLCWRGSY----MR---ARG-FD-VKCP--------------------------
----GGHE----G---C-TATIEKAASF--KERQMGRSTAKTCQ--ED------GIVIQYLTTDGDGKGIMGVNDH-------YA-
ETGMEHRAERLSDRIHLSNL--QFKHSLKTS----FSSQMFPG------K-NKSTKEHYQRLFCID-------------------------
LTQRCTAILRKLSLTDRD------------------------LSKVSTFKIIK-------------ITDSLIQCYQ-GNHTL-------
CKQDLN-----------------ECKG---
--------------RR-S---NN--------WWTNSY-NLKACNLKA----------TDINMDKKNDEMYLRLLIE-------------
MRLGPEALASGKFYTHTNKNESAN-------------------HVLSTVLPKNR---
---------------------VF-----------------------------PSTVSG------------------------RMSSAIMQLN--
RGAT------NGTHLML-Q-----CV-GAPVSAGSRAAKEL----------KKIQKDIDYGK--------------------------------
---------------QYKKTTSAKKARRYHRSVQLNQYC---KMKSSEK----------KQEYKKNQL-------------------------
--------DEV-------------------------RLTRQG---------------------------------CANRPELK-DH--AY--
AAAKGKKVCYYYMYSTELHFEKSSYC------YCAYLLRTYRDVSCTKARDPPNTPHN--------------
>KolobokP-3_OwFu_1p

---MS---
-----------------------T------MSKPPRGFGHF--YSKNKFR-----NQ-------Q-----------TFSE--------
GHTQHP-AY---------GTVA---NREEESSPP---T-
KIRRLDQETH-S-LVVRKSRGGN-------SYEVPDVH----MQPGRG----KILR----------PKLS--EP-K------ISDKYITD---
---------------------EPLDG--TV-G---G------------D--------ETLIVSQNSMINCFNLVAKDH-RA-HSKK----CR--
GDL--KVHKKIPFGLVCKMQLSCSK--CGFLSK---------M--CKLYEEVK--G---K-----K-KGG---------RRKAK--------
------------INVAIHVALQSS---SIGVEAFRKILAT-CNARAPSSQGMQKNSKAVSGDIE-KM------------GENDLKTRRSKL--
---IEXNKLR----GVS-SKDEKKV----------------------QVAFDGCYSSSTFGS-WGKLGQRATRAVGVSVETMTDA--------
-----------------------------MQIISVCLYNKLCWRGAY----LR---ARG-FD-VKCP--------------------------
----GGHD----G---C-TSNIKKSEAF--QEFNMGYKLAKDLD--AD------NLQIEYLTTDGDAKGSLGAQSF-------YD-
EKQSNHKVTKLSDRVHKGQL--QFKHSLKAS----FSRHMFPG-----------NKDLQQKMFSLD-------------------------
IKERCDKIMGQLAKKYND------------------------LGVVSVQYMNR-------------VITSTVNCYA-GNHEQ-------
CRPHLN-----------------SCKG---
--------------AK-----KS--------WLETSH-NLKVCGITK---------NDKLM-PSQEDCKFIRVLLE-------------
MKLGPEAVNDGRFYTHTNKNEASN-------------------KVIAEYLPKNK---
---------------------VF-----------------------------SATCNG------------------------RLHSAVLDIN--
HGRE------NATHLKL-E-----LI-GAPISSA-SAAKHL----------RSMSEKKRYMR--------------------------------
---------------KYKNSPHARKNKSFHRAYYTRQYL---HTRLEKR----------TSDYKKEQL-------------------------
--------DKK-----------------P-------ILKMKL-----------------------------------RRSPRFG-DH--DY--
AQEII---
>KolobokP-5_OwFu_1p

---MK---
-----------------------------------GKGKSG-----HSKYA-----NA-------K-----------SFKR--------
HHKRHE--IPQEEFEIEPP---L-
KIMRLSETNH-K-LVVKEKQG---------KYELADIH----GKQCRA----MLLR----------PKKD--DH-L------PLHEQYGT---
------------------S-NP-----TS-P---P------------D--------EALIVSQFSMIHMMNEVNREH-RV-YNEG----CM--
GNL--SIYEKKPFGLCWRMKLKCEV--CLFISE---------N--YKLYEEIE--A---E---GRK-R-G---------PKPAK--------
------------INTQINVALLNS---SIGIEQFRTILAT-CNIRPPAVSGMHKTAVKVSSQIE-KV------------LENDLKNRCDNL--
---VRLNKFK----GVK-ASEQSKI----------------------SISTDAAYDSNVYGD-RSKMGQNSTRAFTIAVENHTQA--------
-----------------------------QQVIGYCLQVKHCWRGAY----LR---ALG-LD-VQCP--------------------------
-----GHE----N---C-TANKNKAHSF--TEFEMGGEIAKQLK--EC------NLTVEYLTTDGDAKGSQGVQAY-------YK-
DNNIDQNVSRLGDRIHKEHL--QYKKCLKEE----FSRKMFPG------A-TKKERKTIQQVFSRD-------------------------
VKTRCSILVQKYAQLNND----------I-------------TLAISTIEMLE-------------IQKSIVNCYN-GYHES-------
CKVSFN-----------------GCKG---
--------------LP-D---NN--------WVSNSA-NFRACKLKN----------GDIK-MTTDDSKRLMILLG-------------
LRLGPEALYETRFNTHTNTNESAN-------------------SVVNKVLPKRN---
---------------------TY-----------------------------GATAGG------------------------RISSAIHQIN--
NGLG------DSTHLKL-Q-----FV-QAPLPVGSSASVAL----------KKMQQKSVYIK--------------------------------
---------------KYKTSRKARRNRQFHKQYHMREYF---RAKEEKK----------AIDYQKNQL-------------------------
--------DKT---TRL-EH--NY--
TKNKKSRPKRLSRYRD-HAYEKN---
>KolobokP-1_OwFu_1p

```
--------------------------------------------------------------------------------
-------------------------------------MP-----------------FE-----------------------
---------------------------------------K-Q-R-----KK-------S-----------AFSK--------
GHAYIP------------GRRY------------------------------------------------GDLKEKHT---L-
PWKRLTYEDQ-D-CIVQQDKD---------VLRTPLLDG-----TDGL----KLLR----------PKPK--SP-D----------------
--------------------HE-----NT-D---N------------D--------ICHVMSVKKVEDLLSHVVR-H-TN-ESPK----CH-
NPAI--RIY-TQKWGVTCTLTYECTR--CGFQSQ---------N--FKMYNIVK--T---Q-----K-R-G---------RKAAV-------
-------------NNIGLHAGINES---TIGVKKVQIAFAA-CGIEPPNTSSMYKTSNQVGSAIL-TE------------ARRDIFEKIEYV-
----KKLNNLK----GE---SDTNAV---------------------DISIDAMYNSNQIAS-RNKYGQSATQAYTTVREHNTKR-------
------------------------------NFIIGAHVANKLCWVGAY----LR---GKG-YENVKCP------------------------
------GHS----G---C-TANYDRATPL--SERHLAYMIGRNLS--HE------GLNPNLVISDGDSQSINGLNDA-------IP-----
ENKMIAQKDPIHSAQC--QFRAVQRAE----FSNDMFGL------NTPNISVGEIKKVLGSD--------------------------
IKMRCNGAITMIRKQFDQNLTFDE------------------NINLISGKIAS-------------SASAIVKCYT-GDCSS-------
CGQQGV---------------YSQCDG---------------------------------------------------------------
--------------GK-----NT--------WWNKSY-HFKNVNPPII--------KGSFR-PDKIDIEILRDIID------------
ILMSRASFELMKSGKSTNSNEAAN-------------------RAITARVPKNV-------------------------------------
--------------------RW----------------------------SRNGTS----------------------RVFGTIHTLN--
NGPG------VSMCKKL-N-----AI-NIPIAG--RISNFF----------KRIQKKALYKS-----------------------------
---------------SWQKRDSVKAQRVKRRKSQMSDFY---NGRGKRQ----------RSDYKKYNV------------------------
---------------------------P---------------------------------------------TLK-RNN-DH--TY--
QQVEIKQKRARLN-----------------------------------------------------RQKHQ
>KolobokP-1_ApJa_1p
--------------------------------------------------------------------------------
-------------------------------------MG--------------------------------------------
---------------------------------RRKGM-------Y-R-R-----RA-------T-----------TFKT--------
GNRFGFTA--------CTADETDANCTGD--------------------------------MPSPSDTGV--------ATMSSCK---P-
AYKRLSKEEY-E-RVVNSPVKG------------PEIFS---SQSQQT----MLLR----------PTKH--HV-G------QLNNMAPA---
------------------S--------VA-E---S-----------T--------GNRVVNLQVTVRAYHSAMMAH-RE-ASNG----
CAGLLIS--VSDMEEFVGLGSKVVFGCTL--CNYRSE---------L--LPLYKVLNQ-S---N-----K-P-G---------RNPSE-----
----------------LNIALQLGLHQT---SISSTAARRLLSS-ISLPSPAVSTLQKSANQFGPVMK-EI------------
NENDMAEKRGII-----KETLQLR----GL---SRDSPI---------------------
FAEYDRQYNNPLRNSRSKTPFAPASQCRDVIVENVTMD-----------------------------------
KFVIGYHHSNKLCLMGEM----KR---RRG-HK-VQCP--------------------------------GHK----G---C-
SANLRQDANIG-DEQNGGKICASKLLAGKN------PVKVKFLTTDADGKACKGFNSI-------MS-EA-IQSETENLLDQVHLNRS--
LRRALTGAE----FSSDMFPA------K-TVTKKRLIQKRFAED--------------------------LTYRVQAEVTACRKVHKY-----
-------------------DHCKVAKALST-------------CMDCLTKCYS-GDHSD-------CPKQSF-----------------
VCKR------------------------------------------------------------------------GR-C-----
--------Y--KFP-FMPNAAK------------GSLI-ISNSDNAKLIELLK------------TRTCYKRLWSTRFETSTQKAEAVN---
----------------NAFKVTNPKHS--------T-------------------------------------------------TF-----
------------------------ARNSQY---------------------RDHSAIHITN--NGPG------ESIASKM-E-----AA-
GLKLSTNSPCIQTL----------KQMQRKQEYDK-------------------------------------------
LRKRGRAYRRRRAQLRERKYKIHG---RIKYINQ----------HSCYKRDQLIH-------------------------------------
---------------------------------------------------------------DH--SY--SKGGCDTDDDDNNYQ--
----------------------------------------------------
>KolobokP-1_LyPi_1p
--------------------------------------------------------------------------------
--------------------------------------------------------------------------------
--------------------------------------------------------------------------------
--------------------------------------------------------------------------------
--------------------------------------------------------------------------------
--------------------------------------------------------------------------------
--------------------------------------------------------------------------------
-------------------------------------------------------E-QK------------TKSDMDRKKKRV-----
KDILEWR----GM---ARDTPI---------------------NIEIDRQYNTPLSHARRKTPFAPASQSRDTVAENVTHE-----------
--------------------------KYILAYNGTNKLCRHGQR----LV---REG-KK-PLCP-----------------------------
--NHG----K---C-TATVPLSANIG-DEEDGGERCAEMLLDGSE------SITVGAVTTDSDGHFARGIKKV-------MY-KR-
ANIDPEPLLCNTHLNRG--LARRLGKLE----LSKQAFPA------K-YVTGRHKLQRNFADD--------------------------
VAYRVEAEMRAA-KLKKG------------------------DISKV--DFSQ-------------CVPAIILCFT-GNHEL-------
CHINSN------------------VCRG--------------------------------------------------------------
--------------D----------------F--DFV-YSSLRVR------------QTTC-FTQLDRQRIQTVIM-------------
QRLNPSTVSKSRLLSTTQKCESMN-------------------SAFNTTHPKQI--------K-----------------------------
--------------------TF-----------------------------TRNYEG-----------------------RDHSAISLTN--
NGAG------ASILSKT-A-----AA-KVPLSQSPRVVKRL----------KEMQRRRDYWK------------------------------
---------------ARSKRIHVKHARAVHRIYKFRLYE---RKRMDIN---------SKECYLKGQL-------------------------
--------DQY------------------------------------------------------------HSH-IYV-DH--SY--
DRTLLNTESDSD----------------------------------------------------
>KolobokP-1_PaMi_1p
--------------------------------------------------------------------------------
-------------------------------------MG--------------------------------------------
-------------------------------RLGRAV-------Y-K-R-----KK-------S-----------WFQN--------
GHKLFPAK--------CKSPIS----------------------------------------------------HFQMNSP---T-
VYTRPTQQQM-Q-LAVERDERG--------FICTTPNA----VPTRKV----MLLR----------PNAA--ET-T------
PLDKLQLKKEAVPQPLCRRELQHQSPAD-Q-SD-----GG-K---R------------A--------AYRFVDINSAVDLGAQAILDH-QR-
THPD----CSGRPTL--MPDAEERRGLAVSEVLSCQK--CGFCTD---------K--TKLFTEIP--Q---T-----G-R-G---------
RRTAT---------------------PNMAFQVGLYNT---GIAAAGARRLLSA-MDTTVPSISSLQSTSNKCGEIIT-TE------------
NETDMCNKRKIV-----KDIHELQ----GY---ERHSAI---------------------
AVETDRQYNNPLRNCRKKTPFVAATQTRDVVVENVTTS-----------------------------------
```

KYVVMYHQENKLCKKCDI----------------DGAR-------------------------------DHKHA-GK---C-
TATRPPNFNMG-DERNGGIQCAQKLLNCKE------PLFVNRVTTDADGCLARGMRSY-------MQ-SKVAGMETEHSLDPPHLNRS--
LCAAISRAK----FSKEMFPG------K-NKRAKKNIQDRFADD--------------------------VSHRAQAEAAAILKKSGG-----
-------------------NSSQMTTMARN-------------AASAIVQCYM-GNHGL-------CKKWSQ-----------------
ICMG--GK-------
--------Y--RFP-YLPVDAR------------GKLK-LATCDEQTLIKLLG-------------KRLGQEALEKTKYGTSTQKAESMN---
----------------HAFAVTNPKAT--------L--TF-----
------------------------SRNGSC-----------------------RDHSAVHLVN--NRHA------NSIVKKC-A-----AA-
GCPVTAGSPAAKAL----------AEMDKKQKQYV--
KRAKGQEYKTRRAQLRSQRFQLYY---ATRPL---------------YKRGQL---------------------------------DPK----
-------------P-----DDGLK----NV------------------------------CHI-IQS-EH--NY--CKRF-------------
----
>KolobokP-2_PaMi_1p

---MG--
--------------------------------RFGRGV-------Y-K-R-----KK-------A-----------GFQK--------
GNTWSPQK--------LNPQKR---QLHTGSP---T-
VYKRPTHEQL-E-QAVTKDAEG--------FHCQTPVD----EPTRSV----MLLR----------PKVH--ET-T------PVGELQHR---
------------------E------VLQG-Q---R------------E--------TYRFVSIDATVKLGMQAALSH-QQ-ANPE----
CESQPEL--VHDAEVRRGLGVSEMLACQK--CGFHTD---------H--TKLYSEIQ--H---T-----G-K-G---------RKTAT-----
----------------PNMALQVGLYGT---SIATAGARRLLSA-MDTTVPAKSGLQNLADKCGKIIT-AE------------
NKRDMSDKRRIV-----KDIHELQ----GH---DRESPV----------------------
AVEMDRQYNNPLRNARKKTLFVPATQTRDVVCENVTSK-------------------------------------
KYVVMFHHENKLCKKCDN----------------ANCN-------------------------------QEE----T---C-
TATRPANFNMG-DEQEGGRQCAQKLINCNE------PLVVNRVTTDADGCLAKGMKSC-------MQ-SQ-AGMETEHSLDPPHLNRS--
LCRAMSRAK----FSAGMFPA------K-TKKEKKKLQNRFADD--------------------------ISHRAQAEAAALVNKFRR-----
-------------------DSCKMADMAEK-------------AAKAIIQCYM-GKHTL-------CRKWSI-----------------
VCGG--GR-------
--------Y--KFP-YLPADAR------------GKLN-ISATDEQTVLRLLS-------------KRIGNEALEKTKYGTSTQKSESMN---
----------------HAFAVTNPKGT--------Q--TF-----
------------------------SRNGSS-----------------------RDHSAIHMVN--NGHA------NSIVKKC-K-----AA-
SCSVTPGSPAEKAL----------AEIEKAQKYHA--
ERKKGVAYRKSRAQCRSKRFHSYY---STQNM---------------YKKGQM---------------------------------NPK----
--------------------HGFKFQGVNG------------------------------SHN-VRL-DH--NY--CKQFPSK----------
----
>KolobokP-2_BF_1p
MAKANNSMWYKLSLPLSRTILKELVCDNSAHTLSACLSTYLPT--YLH----------------TFS--------------------------
----HQHHI----------------------------------MP--
--------------------------------RRRKKM-------R-L-R-----HA-------G-----------QFRA--------
GNKPWN-K----------GLTI--TKVTPI---S-
SYHRPTQDQD-S-LYVNRDRKGNI------IQNDIETL----NDVGTT----MVLR----------HTKL--QG-S------RIARFIGE---
------------------K-TD-----SD-E---V------------E--------GYRMWHAKTAVHACVSAQRHH-DK-IQTD----
CKGLVRV--SARKEQKKGLTTSETLVCNE--CNYESP---------H--SKFYGVVP--S---D--EPGK-R-G---------PKVAE-----
----------------PNMAAQVAMFNS---PIGPQVLREVAAA-LDISVPSTGGLQNLANKYSDKMV-SL------------
NEKDMEALRETV-----KEVHAVK----GN---EPDSGI----------------------
PIEADSRYQSPLRSGRGKKPGQPSGTCVTNVAENVTKR-------------------------------------
KWVICTHVKNKNCQQCSR----GE---GK-----KEVP-------------------------------SHK--------C-
SANIPQSAVIG-DERQAAIEIAKKLM--SGP----SKTIVAELTEDGDSSFSSGMKEV-------ME-EA-GLNDLKVFKDIVHLSKA--
IRRKVSGGK----WSKQMFPG------K-TQEERKWIRDRFGND--------------------------LAIRLNTEHILGLKKF-R-----
-------------------NQRVMAGKMKQ-------------VMQAIPYCYS-GDHSR-------CKSGSL-----------------
VCRH--PY-K-----
--------W--KFQ-ELQEKAK------------GKLD-PTDADLKELGKIME-------------HRLGKEALKVTRRGRTTNKVESVN---
----------------RQISKTCPKNV---NR-----
------------------------SRTFPG-----------------------RVHSGLHSSN--NGTG------MSIARKR-A-----AA-
NIPFSPNSAVVPAL----------EGMEKTQNYHR--
AYKMEVANQKRQKAKTLRRFASYD---KRKEQGT-------------YKSKRAV-------------------------------------
TGTG----------------RAPRKKKKV-------------------------------------DNR-EH--SY--TRPAEQESPSGSD--
--------------------------------AD-----------------------
>KolobokP-1_BBe_1p

---MP--
---------------------------------RKKKN-------K-E-K-----RG-------R-----------PFIP--------
GHTPWN-K----------GCVV--EREKVT---S-
SYCRPTQDQE-G-LYINRDRKGNI------LPTDTETL----NEAGTL----MVLR----------TTQL--PG-S------KISRLLGD---
------------------K-TD-----GD-E---V------------E--------GYRLWHAKTTVKALYSLQREH-DE-LRPD----
CKCMLRL--SAQGERKKGLATQETLVCDN--CGYESP---------R--TKFYTEVP--M---E-----G-P-G---------AKTAV-----
----------------PNRAVQVAMFNS---PVGPTVVREMAAA-LDMPVPTRSGLQEGANKYSELMV-QE------------
NEADMQSWRETV-----ADIHVQK----GN---PPDSGI----------------------
PVEGDTKYQSPLRSARTKKPGQPSSNSVSTIAENVTPK-------------------------------------
KLVIGTHVRNKICQTRNY----NE---GCG----NPVP-------------------------------PHK--------C-
PANISEDTVIG-DERQAGRDMGKKLL--SGP----VKTLVSETTTDGDGDFAAGLQEV-------MM-EE-AGQKVRAFSDTVHLCKA--
IRGRVSRAT----WSKQMFPG------N-DQQERNRNRDRFGND--------------------------LSKRLNAEHKAARKKIGD-----
-------------------NKRKMEKKMKR-------------LMQALPYCYE-GDHSR-------CYEGSL-----------------
ICRH--PR-K-----
--------W--TFP-DLPEKVK------------GNIH-PNDTDLKVLAGIME-------------VRLGKKALKRTRKSRTTNRVESVN---
----------------RQLAKTCPKNV---VY-----
------------------------SSTLEG-----------------------RVASAVHASN--NGTG------RSIALKR-A-----AA-

NIPFSPDSAVVPAL----------EGMEKKQEYHR--
SYKMEVTNKKRQQAKVKKRYANYD---IRKEQGT--------------YKSKTV--------------------------------
AVAGQSKGKG----------------KALRKKVKK-------------------------------------RMI-EH--SY--
SRAAEEESSSEYS---------------EG--
>KolobokP-3_BJ_1p

---MP--
---------------------------------RKKKK-------R-L-S-----HG-------C-----------QFRA--------
GRTPWN-K----------GYTL--VQQKVI---S-
SYCRPTEDED-N-LYVNRDRKGNI------LQNDIETL----NEVGTT----MVLR----------TTKL--PG-S------KIARFLGD---
------------------K-TD-----GD-E---V------------E--------GYRIWHAKKAVQACASAQRQH-DE-LHPD----
CKGLVRL--SAQGERKKGLATLETLVCDD--CSYAAP---------K--TKFYSEVE--T---Q-----G-P-G---------AKTAV-----
----------------PNLAVQVAMFNS---PVGPTVIREMAAA-LDITVPSRSGLQEGANKYSDLMV-TE------------
NEADMQAWQETV-----QGIHEEK----GN---EAGSGI----------------------
PIEGDGRYQSPLRSARGKKPGQPSSNSITTVAENVTPK-------------------------------------
KLVIAAHVRNKICPTHNY----NE---GCG----KPVP-------------------------------PHK--------C-
PANISEETVIG-DERQAGKDIGMKLL--SGP----VKTLVSETTNDQDGDFAAGLQEV-------MM-EQ-AGQKVRAFTDTVHLGKS--
IRNRVTRAT----WSKEMFPG------D-NQEERNRVRDRFGND--------------------------LSKRLRAERRKGLKKF-R-----
-------------------QKRQIEERMKR-------------VMQTLPYCYS-GNHER-------CYEGSL-----------------
ICRH--PR-K-----
--------W--TFP-DLPAKAK------------GNIN-PNDSDLKVLRDIME-------------VRLGKKALEKTRRGRTTNRVESLN---
----------------RTLSKNCPKNI---VH-----
------------------------SRTLEG-----------------------RVASAIHSSN--NGTG------MSIALKR-A-----AA-
KIPLSPDSAVVPAL----------EGMERKQSYHR--
SYKMEEANKRRQQAKVKQRYAKYD---IRKEEGT--------------YKSKKV--------------------------------
AGQSKGKGKG----------------KGKALRKRK----------------------------------DK-TSM-EH--SY--
SRAPEQESPNEYS---------------------------------DIDQEEK----LCD--LP--------
>KolobokP-4_BJ_1p
MR-------------RYYTLRS--------------------------IRDTVKTAP------------------------------------
STYPPQDDI----------------------------------MP--
--------------------------------QKKKKK-------R-L-S-----HA-------G-----------LFRA--------
GNKPWN-K----------GKTL--MKVEAF---S-
SYHRPTEDLV-R-LYVNTDRKGNI------LQNDDEIV----NEAGRT----MVLR----------PAKL--PG-S------KIARFLGK---
------------------K-ED-----SD-E---V------------Q--------GYRIWHAKLAVHAVARAQREH-DK-RRPE----
CTGLVRL--SARGERKKGLATLETLVCDT--CEYESP---------Q--RKFYGEVK--S---K-----R-P-G---------PKIAI-----
----------------PNMAVQIAMFNS---PVGPQVLREMAAV-LDLPVPSRSGLQKMANKFSELMV-QE------------
NERDMQAWREIV-----QDIHAQR----GN---EPGSGI----------------------
PVENDTKYHSPLRSARGKKPGQPSSNSITSVAENVTAR-------------------------------------
KLVISTHVRNKVCPTCNY----NE---GRG----EPVP-------------------------------PHK--------C-
AANIPQDAVIG-DEAEAAREIGKKLM--SGP----VKTPVGESVSDGDSSCTTGLQEV-------MM-EE-TGQRVTAFQDTVHLDKS--
IRGRVTRGA----WSKGMFPG------Q-NQEERNRVRDRFGND--------------------------LSKRLSAEHRKGRKKF-S-----
-------------------TRKKMVAKMKK-------------VMQTIPDCYG-GDHSR-------CPKGSL-----------------
VCRH--PR-T-----
--------W--SFK-DLPEKAR------------GHIN-PNDADRKLLATIME-------------KRLGERALRRTRKGRNTNKVESFN---
----------------RQLSKTCPKNV---LF-----
------------------------TRTLEG-----------------------RVASAVHSSN--NGTG------MSIALKR-A-----AA-
NIPLSPDSPAVPAL----------EGMERKQDYHR--
SYKQEVANKKRQQAKIAQRYATYD---TTKEQGT--------------YKSKKV--------------------------------
AGQSKGRGKG----------------KGLRKKKKK----------------------------------PV-GSK-EH--SY--
CRHPDEESSDSWS---------------------------------PVEEDGEQDEEVEE--L---------
>KolobokP-1_BL_1p

---MA--
---------------------------------RKRKK-------R-L-Q-----HG-------G-----------LFRP--------
GNKPWN-K----------GLAI--SQAETI---S-
SYCRPTEDQD-S-LYVNRDRRRNI------IQNDIETL----NVRGKR----MVLR----------PTRL--AGRS------KISRFLGE---
------------------KEED-----RD-D---V------------E--------GYRIWHAKIAVHACASAQREH-DK-RQPE----
CKGLVRL--SARGEKKKGLATLETLVCDT--CQYASP---------K--RKFYGEVK--R---E-----G-R-G---------PKIAV-----
----------------PNLAVQVATFNS---PVGPTVIREIAAA-LDLTVPSRSGLQNMANTYSDMMV-QE------------
NEKDLQSWRETV-----QDMHVEK----GN---APGSGI----------------------
PVQGDTRYQTPLRSARGKKPGQPSPNSVTSVAENVTTR-------------------------------------
QVIIGTRVRNKICQPCIQ----AK---GSG----GPPP-------------------------------PHK--------C-
PANIPEEAVIG-DERQAAEDLAHAFL--SGP----NKTLVGEALEDGDSGFSGGMKKV-------MM-EE-AGQEVKVFKDIVHLAKG--
IRHRVTTAS----WSKQMFPG------H-DQDEKNRVRDRFGND--------------------------LSRRLNAEHRAGLLKF-R-----
-------------------TKRAMEKKMKQ-------------VMSAIPYCYE-GDHRR-------CLARSL-----------------
ICRH--PK-H-----
--------W--KFK-DLPKKAR------------GNIQ-PSSADLKVLGKIME-------------HRLGKGALSKTRRGRTTNKVESFN---
----------------RQLSKYCPKNV---VI-----
------------------------SRTLEG-----------------------RVASAVHSSN--NGTG------MSIAMKR-A-----AA-
NIPLSPNSAVVPAL----------EGMERRQQYHR--
SYKMEVANKKRQELKKAQRFADYD---TRKEQGT--------------YKSKKP--------------------------------
AGQSKAKGNG----------------RAARKKK---------------------------------------TVG-EH--SY--
TRAAEQESSSEYP----------------------------------SDQESDQD-----------------
>KolobokP-2_BL_1p
MT-------------
LAYTSSNKDINKQTGQDISLSLGTTEATQSHMHTHTQVRNGPIWQVHMVTFSLPQAEKGGQHGSFDHTLIYTRHFEDSSRYPHQDHI------
----------------------------MA---

-----------------ARKKRG-------R-K-KNGRPHRA-------G-----------QFRK--------GQKPWN-K----------
GLDL--MQVKAI---S-SYRRPTEDQE-G-
LYVNKDRAGNI------LQNDTETL----NEVGRT----MVLR----------PTKL--AG-S------RIARFLGK----------------
-----K-VD-----SD-E---V------------E--------GYRIWHAKTAVRACASAQRDH-DK-HQPD----CEGLVRL--
SFKGEIKKGLATVETLVCDT--CKYASP---------K--RKFYTETK--R---P-----G-P-G---------AKIAV--------------
-------PNLAVQIAMFNS---PIGVRVLREMAAA-LDLPVPSKSGLQRMANRYSDLMV-KK------------NEDDMETWRETI-----
YDIHLQK----GN---PPGSGI----------------------PAEIDTRYQTPLRSARGKKPGQPSPNSVTSLAENVTPK-----------
--------------------------KLALAVHVRNKICPTRNL----NE---GRG----KPVP-----------------------------
--AHK--------C-PANIPEEAVIG-DEKQAARDVAKKML--SGR----RKTPVGQLVEDGDSSSSKGFKEV-------MM-EE-
TGQKVTAFKDIVHLGKG--VRNRVTTTK----WSKEMFPG------Q-DQLERNRVRDRFGND--------------------------
LCKRLNTEHKLARKRF-P------------------------QKKKMAAKMKQ-------------VMEAIPYCYA-GDHDR-------
CVAGSL-----------------VCRH--
--------------PR-K-------------W--KFK-DLPAKAR------------ENIT-PSSADLKLLQKIME-------------
HRLGKTALKKTRCGRTTNKVESLN-------------------RQIGKACPKNV---------------------------------------
--------------------LR-----------------------------SRTFAG-----------------------RVASAVHSSN--
NGTG------MSIAQKR-A-----AA-NIPLSPNSAVVSGL----------EGMDREQKYQR-------------------------------
---------------AYKMEVPNKIRQQAKTTTRFAAYD---IRKEQGT-------------YKSKVV-------------------------
-------TGKSKGKGKG----------------RGKGKAVRK--------------------------------------NT-EH--SY--
SRDAEEESPDTYS----------------------------------SDEE---------------------
>KolobokP-1_BF_1p
MQ-------------IKYLKSH-IAGDNQSEKI-------------------------------AFR--------------------------
----EEGHIA--------------------------LSGQAITMP--
------------------------------------RR-------K-L-R-----HD-------G-----------QFKA--------
XNRPWN-K----------GLKF---REHKGTR---P-
TYPRPTEDDF-A-LLEERDRQGNA------NICKRDVI----REERRP----MLLR----------PTPS--QA-TCG----QMSPYLED---
---------------GP-G-ED-----GD-Q---V------------L--------GYRIWHAGLAVQACAKAQREH-DS-QEGV---
LCKELVRA--SSTGEGKKGLATSQTLVCDG--CGYKSA---------K--ENFYEEVA--R---D-----G-P-G---------GRAAV----
-----------------PNAALQIALADN---PMGVTAFREMAAA-MDLPVPSEASLQEGANRYSDLMT-AV------------
NANDXRXWAKVV-----XXXXVLK----GN---

--EA-----------

>KolobokP-3_BL_1p

---MG--
------------------------------------KR-------K-M-Q-----HD-------G-----------QFKP--------
GNYPWN-K----------GGTK--KARVRL---P-
TYVRPSEEDY-S-LLADRDRDGNV------LHDKMDII----RESRRP----ILLR----------PQKS--DS-S------EMNQYLGD---
---------------GS-A-EG-----AD-D---V------------V--------GYRIWQATAAVRACAKAQRDH-DK-RRPT----
CPELVRA--SAREEKTMGLATAEKLVCDR--CGYGSS---------S--QKFYDEII--R---D-----G-P-G---------GRTPV-----
----------------INMAAQVALADC---PMAVSGFRQMMAT-MDLPVPSEPALQNLANKYSDIME-EE------------
NEKDMKRWIQVM-----KRVNALK----GY---KPDSPI----------------------
RGQADTRYHTPLGNLRGRKPGQPSDSSNATFVEEVTTR-------------------------------------
NKVIANHLKIKTCRLCTF----YR---GHG----LEPP-------------------------------PHK--------C-
SANTLPQAPIG-NEEESGEKMARDLL--ES------NVRVSGLITDGDSQTFRGMATV-------MREEAVRPIRVKQERCLVHISRN--
VRTKISSTK----WSPTMFPG------A-KAESRSRVKHRFAND--------------------------LVQRLNAEHEAALTQLGMR----
-------------------TRHGVERAMDK-------------AMRAIPHCYA-GNHDW-------CLEYSL-----------------
VCRH--PS-R-----
--------W--RFQ-KINAEAR------------GQIN-PNPSDCKLLRVAME-------------MRLGKKALELTRHFANTNKAESVN---
----------------RQISKSAPKNI---TR-----
------------------------FRTLAG-----------------------RIASALHSSN--NGTG------LSVAMKR-L-----AA-
GIPLSPKSKAVRVL----------EKMRERQDYKR--
SYKKEPANKKRENANIMGKFVVYD---EGRERGT-------------YKSAEV--------------------------------
AKPSKGQRES-----------P---------RKRA----------------------------------QQ-DGT-DH--GY--
SRAGDKEPEDYYS---------------------------------DSEDDSDEDMDLC-------------
>KolobokP-5_BJ_1p

---MG--
----------------------------------RKRA-------S-K-D-----RP-------T-----------LFQK--------
GDTPHN-K----------GVKT---EYEKGAT---P-
VYRRVTEDQY-E-LLQDTDRKGNP------LVCKKKVV----LEKRRV----MLLR----------PKKE--SA-S------KVARAVARDW-
--------------DAG-D-KN-----PD-D---V------------V--------GYRIWDARKAALACTTAQREH-DD-QHPC----
CRALVRL--SAKLETKVGLATAETFVCDR--CTFQSK---------K--YKFYDEIK--S---KPG---K-P-G---------RKMAV-----
----------------PNMAVQVALANS---SMGVRAFREFAAA-MDIPIPAESQMRVNAKKYRDIML-EE------------
DNRDLKEWTETV-----KEINAIV----GN---DAGSPI----------------------
MGEGDSRYKSALYAGLGRKPGQPSPHAFSTFCEDVTVR-------------------------------------
RLILAHFLAQKQCSTCQRPK--KR---KRG-RP-GRVEK-----------------------------RKHR--------C-
PANLRQHDVIG-NEELAGREIGKKLL--AN------KVLVSHIITDGDSHFAKGLSDV-------MM-EA-RGKRTKHLRDFVHIGRS--

VAKAVRRGT----WSRQMFPG------K-TAVQRNKVKSRFAEE--------------------------LRVRLNAEHHAAVLRFGK-----
-------------------RRHKIEPAMAK-------------AMLAIPVCYM-GSHEL-------CSTGSL-----------------
VCNG--RS-R-----
--------WKADRR-YLPV---------------KHLN-PDSSDVKEMRRIME--------------KRLGTQVLSSTRYLMDSNKSESVN---
----------------RQCSKNVPKSV---NL-----
------------------------IETLPG-----------------------RFCTTVHSSN--NGVA------LSIVKRR-R-----AT-
GIPLSPMSPTVAVL----------ESMERNRRYRR--
AHRQEPASKERAMANRIRRFKTYD---EMKEEGT-------------YKSGDV--------------------------------PSAEK---
-------------------KIRLSKKQ----------------------------------PR-GRI-DH--TY--NRMEGEGPASSLD----
------------------------------SDQWTDDSGDESDNEFLLPYSYTKR
>KolobokP-4_BL_1p
MK--PQQVK--------------------
----SQKHASPA-------------------------------MG--
----------------------------------RKKS-------S-K-E-----RP-------T-----------VFKK--------
GYTPHN-K----------GMKI---RHVTPAT---P-
VYRRVSEDNF-E-LLQDTDRRGNL------LIRPKKVA----LEKRRV----MLLR----------PKKE--SC-S------
KIARAVARGYT----------RD--DAR-D-DN-----LD-D---L------------V--------GYRIWLARDAVKACTKAQRDH-DQ-
EHPT----CKALVRL--SAKLERKVGLATSETFVCDK--CKYKSK---------G--KKFYDEID--K---DAGQGCR-R-G---------
RKVAV---------------------PNMAVQVALANS---SIGVAAFRQFAAA-MDLPVPVSSQMRKNARKYSDLMV-RE------------
NERDMKAWSEKV-----KEMNSIL----GN---APDSPI----------------------
KGEADSRYKSALYAGLGKKPGQPSPHAHSTFCEGVTAR-------------------------------------
RLILAHFLAQKQCSTCQRQQ--LR---RKCVRP-GRVQK---------------------------KAKKHK--------C-
TANLKQHDVIG-NEELAGREIGKKLL--AN------KVLVSHILTDGDSHFAKGLSEV-------MM-EA-RGKRTKPLRDFVHMGRS--
VAKAVRHGT----WSKEMFPG------N-TAARRNKVKSRFAEE--------------------------LRVRLNAEHDSAVLKFGK-----
-------------------RRQKMEAAMED-------------VMDAIPSCYM-GTHDF-------CSTHSM-----------------
VCNA--RR-R-----
--------WKTDRR-YLPV---------------KCLH-PNSSDIKELRRIME--------------KRLGKKALESTRYCMDSNKAESVN---
----------------RQSSKNVPKSG---NW-----
------------------------IETLPG-----------------------RFSTTVHSSN--NGPA------LSIVKRR-A-----AA-
GIPLSPMSPTVKAL----------ESMERDRLYRR--
KYRNEPECKQRALANRIHGFKTYD---ELQEMGT-------------YKSGNV--------------------------------PSAEK---
-------------------KRKVVTKV----------------------------------PR-GKE-DH--TY--NRMEGEGPTLDSD----
------------------------------SPGWTDTSGDETD------------
>KolobokP-3_BBe_1p

---MP--
-----------------------------------RGS-------K-N-R-----QS-------S-----------QFKK--------
GHTPWN-R----------GQKM--HRETQPDP---P-
TYQRPTEEQY-H-LLVNTDRQGEP------IEDLARIA----VEESRP----PLLR----------PKKE--PS--------
KMDRILGIEED----------EDGRDEE-D-EE-----DD-T---I------------S--------GYRYWLGHAAMMACATAQRQH-DD--
RSS----CRKLIYA--SAKREETRGIVSSETFVCNA--CEYVSQ---------S--YKCYEEVE--T---G-----K-R-G---------
KKLAA--QTWHYKGGGDGEERQETSSPNMALQIALFNN---PIGPKNFMEIAAT-LDLPVPSLSGMQKMANRYAEIIV-EE------------
NDKDMKRWGRSCWGPMKSQENQQT----GQ---
SRGGGCEVPKRPQFRKRTSHRKTSRQANQGEGDVRFQSALNSGRGRRPGQPSSHAFATFAENETNM---------------------------
----------KKILATYLRNKQCAKCNL----GHV--GRG-------------------------------------VKRSX----N---C-
SANIAKHVVIG-DEAALGREMGKKLV--EN------NIRVSHLVHDGDGHIFKGMSEV-------ME-EE-TGEPTKSLSDNIHLSRS--
IARAVTKAT----WSAHMWPG------K-TRAERMQVKNRFGDD--------------------------LKLRLEAEHRQAREKYGK-----
-------------------NKERMEEAMNK-------------ASDAIVNCYLDGDHTL-------CRTQSL-----------------
VCSG--SS-K-----
---V----W--DFS-FFPHGTK------------ELIC-PDEADREELRTIIG--------------KRLDPEVLEATRFGLNTNRAESAN---
----------------RQYSKSVPKNR---TL-----
------------------------TTTLAG-----------------------HYASAVHSAN--NGTA------LSILMKR-Q-----AA-
GIPLSPRSPAVTAL----------RAMDKIETYKR--
AYHKEPARRSRRKTNRVKEFQTYN---EAKDIGH-------------YKSKGE---------------------------------
EEEEGEKAG-----------PS-EPKKRRVSNKQ----------------------------------QR-GKQ-DH--VY--
SRMDGESLPTWSK----------------------------------KKKESQWSGEEDN------------
>KolobokP-3_BF_1p

---MG--
------------------------------RPAGSRALKN-----R-M-V-----RN-------S-----------KFQA--------
GNTPWN-KASLAESVENTGTEEPSASTV----------------------------------ASCSSSSGVEQESQSEPTHTASAI---D-
VKKRLDEDTY-K-MLCRP--------------------------RDRVI--IPRLRV-TKTKCYTEPKRS-----------------------
--------------------YK-----KN-Q---V------------S--------GSRIIDLQKMEDLMNLFNAGH-RK-ANGK--
RICKGDWKF--SRKLEKKIGFGCMEALTCKT--CGYNTT---------P--AELFRHPS--P---G--RP---R-G---------PLPVK---
------------------SNIQAVTPGAKD---RYGISAIKPLMAS-LGIPDPCRTTLQRKMNVVGEAYA-EL------------
NKRQMKKSCETL-----AEVTRHRVE-AGL---QESASV----------------------AVEMDVGYNNPPK---
GKSRGQPGTQAECPMFDGVTQK-------------------------------------KMLLALSTANQHCVDCDK----LE---EQG-LP-
FGPG-------------------------------SHE----G---C-TRTFEKHIKMGSSERKMAKENTKTAL--KN------
GIIPGIVETDNDSKISGGTNEA-------LH-EA-GLPPAEKQDCMQHMSRN--HRSKAYN------LCLEVFEGGN----N-
TKVTKQEASRRVGLY--------------------------IVDRCVRALYTARAHHPD------------------------
SDEDFFREVER-------------GRPTIVSCVS-GEHSE-------CTSDF------------------GCPE-------------------
---YTRE---GRR----------PAP-DIPGG---
-----------GNLA-LSDNDRRTLQKLVD-------------YRLAEESVKRQRTFMNTNKSEAFH-------------------
NRALKAVPKAV---TW---------------------
--------KRNYEG-----------------------RVHAAGLADS--VGSG------SAILRAN-E-----FL-GAAHPQGSSGTAAL---
-------EGIDKEEEYHK--KRKGTFKYKMRRYILRKRKVEQ-----
KDRKGVG-------------YETDSV---------------------------------HAM-------------------------------

-------------------------------------SRG-DH--CY--A--

>KolobokP-2_BJ_1p

---MG---
------------------------------RISGKRAAKL-----RLR-R-----RP-------Q-----------CFQP--------
GNTVAK-KD--GDVRRATGGQTSTAVTE-----------------------------------EPLNSDGSV------DIEHAALTI---D-
PRKRLDQDTY-R-MLYRP--------------------------GDHVI--IPRFRI-TKKKNYS-ERRP------------------------
--------------------YT-----KD-E---V------------T--------GNRIVDMRKVEELMNLYNAGH-RK-ANGR--
KVCNGECKF--SGKLERKLGFGCKEVLRCKT--CGYKTT---------P--AELFQRVD--R---R--RPGT-R-G---------PLPVK---
------------------MNIQAVTPGAKD---RYGPAALGPLFAS-LNVPGPSRTVAQRKMTEASSAFQ-KL------------
NKEQMQKNCNVL-----AEVKKLRLA-AGL---KESAGV------------------------SAESDVGYNNPPK---
GRSRGQPGTQADCPLFEQDTHK--------------------------------------KMLLSLWTESQHCAGCEQ----RR---RQG-LP-
TGPE-------------------------------YHD----G---C-TQTFDRTLKMGNSEGKMAKKNATAVL-ESS------
GLALEGLTTDNDSKIVNGTNEA-------LR-DA-NLPPVQKQDCMQHNKRN--HRNRVFP------LDLEVFQGGN----N-
TKDRRKKASHDVGNY--------------------------IVETCGKALYKARARNPD-------------------------
NDEAFFGELEH-------------LRLTLVDCLA-GEHSR-------CTADF-------------------VCPA-------------------
--NSPD---DDG----------PTP-DLPGG---
-----------GNLA-LEKADRGALQALID-------------YRLAPNAVARQKKLRTTNKCEAFH--------------------
NRALKAVPKSL--NF---------------------
--------KHNYSG-----------------------RVHAAGLADS--IGSC------AAIDRVN-S-----FM-GASHSPGSPGQAAF---
-------EAIDKEEDYHR---KRKRSIGYRMRRCNLRKSKTVP-----
KDANGQE-------------YSTDSM----------------------------------HPS-------------------------------
-------------------------------------VRN-DH--SY--SK---

>KolobokP-2_BBe_1p

---MG---
------------------------------RVSGRRAVKA-----R-N-R-----RP-------Q-----------TFLP--------
GNTPWN-KGLVAE-----GGHTDAGVSPT-------------------------------EDGEPTVRGGQT------SSTPSTSTI---D-
LKKRLDEKTY-S-MLHRR--------------------------GDHIL--LPCLRKRTKRVDYTEPKRP------------------------
--------------------YT-----NN-K---V------------L--------GNRIVNMDKLAELVNLFIAGH-RK-ANGR----
CKGECRF--LKNLESKLGLGVKEALSCKA--CGYKTT---------A--TELFQRAD--T---R--RPGA-R-G---------PLAVK-----
----------------LNIQAVTPGVKD---RYGPTALKPLFAS-LDIPDPGRTTMQKKMTEASEVYA-DL------------
NKAQMEQNCATL-----AEVKRHRIE-AGL---SETSTI----------------------ITESDAAYNNPPK---
GRSRGQPGTQAECPTFEKETGQ--------------------------------------DMLLALTTTSQHCKVCEN----RR---RRG-LP-
TGPG-------------------------------SHR----N---C-TQTFDRTKKMGAAEYEMAKKNTEAVL-KCS------
GLAAGTHCTDNDSKNIKGVNDA-------LR-EA-GLPPAEKQDCILHHKRN--HRARVFP------LDLEVFQGGN----A-
TQEDRKQRSRRVGHY--------------------------IVDRCDTALRRVRKLHRD-------------------------
CDTDFFDEVDRC------------IRPTLLNCIA-GDHSE-------CTYET-------------------ACPE-------------------
--HSLE---QH-----------PAP-DIPAG---
-----------GNLA-LTKGDRDVLQALVD-------------YRLAPETVKRQKGLMTTNKSESFH--------------------
KRVTKACPKSM--NF---------------------
--------PTNYHG-----------------------RVNAAGLADS--IGSG------AAIRRAN-E-----MM-GASHSDGSPGAAGL---
-------EAIDREEKYHQ---DRKKTWKYKNRRYKLGKAKAHA----
KVTKSGEG-------------YSTDCM----------------------------------HPM------------------------------
--------------------------------------VRS-DH--SY--TKN---

>KolobokP-3_CGi_1p
MI-------------FN---
---MP---
-------------------RTDG---------RRQGKK-------K-K-T-----RK-------T-----------TFLK--------
GHLPWN-KD----------QQPISSVPC--TESTSVN---I-
PVCRPEKAIY-D-ASILKYE-----------------------GPPNTL--PGKLR------------PKHV----------------RKE---
------------------D-EP-----LD-N---S-----------S--------ENIIVNFCKLKEFIKSSISHN------------
CRRQDSF--NVAIAKRKGLCVCLQASCTN--CGFDTG---------V--HKLYTEYT--R---N-----K-R-G---------PAAGC-----
----------------LNEGLSMATVKS---KMGGTDVQMLMAC-LDIRAPSLTIINNKINKQCEKMI-EL------------
NEKSMVENQKFA-----GQVATFI----GE-----GNNI-----------------------DVETDTCFNNRNQTG-----
YEAGTQSFCPVIEKTTGL-------------------------------------NLPIAMATASKLCSKI-------------------
NMCT--------------------------------HE----NN-KY-SLTYNPDESISSSESKLTTKNLQKIH-DLK------
IVSIKSLTSDASSQISKVVREV-------GK-K--NEIVIKYFQCFVHRLRT--IQKKVKSIQ----FLK-LPAG----------
YDQDIFRQKLATC--------------------------IRSRVRLELVRIKKKYPA-------------------------EGA-FVTRALM-
------------AMKNIVTCFS-GSHTY-------CAERSL------------------ACNA-------------------------------
--HL-E----S--------L--STQ-YLPYG--------------
SYLS-LVTEDRRKLESVMN-----------KSLTTEILNKIANLYTTNQCESLH----------------------HRVFTYAPKSV-------
---LF-----------------------------TRNFNG----
-------------------LCHSACHSST--FGTG------KSSVLLA-K-----SL-GLNFSKCAPFFRYM----------
TRKDILALYHS---QRKKTPKYKIQRYLAKCRKSN--R---KIRQGSL--
-----------YSNACN---
--------------------------E-LVQ-IH--NY--AINVNKH---

>KolobokP-1_MyEd_1p

---MS---
-------------------SYD---------RRKNRI-------Q-R-R-----RK-------S-----------KFVK--------
GYTPWN-D----------KLEYITSDTE--AEHQYIN---N-
RICRPEMKIY-K-TAVSQHT-----------------------KEDNTL--PTKLR----------PRHCF-ME-------------EEE---

------------------T-EA-----QE-D---T------------T--------ENIIVNFCKLNDFIKKCLKHR------------
CRNKSSM--NINISNRMGICISLRANCAK--CQFDTN---------S--CKVYTECE--K---K-----S-R-G---------PAAGY-----
----------------LHEAMTLAVLKT---KMGGSDLQMTLAC-LNIRVPSLQLISNNINKQCDKII-QL------------
NEEAMIENQRFV-----KEFNTCI----GK-----DNEV----------------------DVETDTAYNNRNQVG-----
YEAGTQSFCPLIEQNTGL-------------------------------------NLPISMSTANKLCSRK------------------
KNCN--------------------------------HS----DNQSC-KKNYNTDDTIASSEGKLAEKNLQKVN-DQN------
ILKVKSVTSDASAQLSKVVREF-------GK-K--SGNSIRYYQCFVHRMRT--VQKYIKNLN----FSK-LPPG----------
YDKDFFRQRLATC--------------------------IRSRVRLELVRIHK-IST------------------------EHT-FGTLAQS-
------------AIKNIVSCFS-GNHAN-------CKEKSE-----------------ACCA-------------------------------
---HL-D----T--------F--NTK-YLPYG--------------
VYLN-LNVIDKKKIESALN-------------KSLSLDVLNKISSLHTTNQCESLH-------------------HRVFTYAPKSV-------
--LH-----------------------------KRNFNG----
-------------------LCHSACHSST--FGTG------KSSILLA-K-----SL-GLNFSKFAPFFKFM----------
IRKDIIALYHS--QRKKTAKYKIQKFLAKCKKQN--R---RVRQGSM--
-----------YSGGST--
--------------------------D-VNE-IH--NY--GINENN---

>KolobokP-1B_MyEd_1p

---MP--
-------------------GQD----------RRKKKK-------L-W-R-----KR-------N-----------NFNK--------
GYTPWN-N----------KVQFELGVEA---DSSNAAN---I-
KVSRPKIEIY-E-AALTQHN-----------------------GAANTL--PTRLR----------PRRS--SH-------------KEK---
------------------T-EE-----QE-D---C------------S--------ENIIVNFSKLKDFIKKCLKHR------------
CRNNSKV--DVAIAKRMGLCISLQANCEK--CNFDTR---------A--CKVYTECE--K---K-----S-R-G---------PAAGY-----
----------------LHEAMTLAILKT---KMGGTDLQMALAC-LNIKVPSLQLISTNVNKQCDKMI-EL------------
NEAAMVKNQQFV-----QEFNQCI----GK-----DNEV----------------------DVETDTAYNNRNQVG-----
YEAGTQSFCPLIEKNTGL-------------------------------------NLLMTMSTANKLCNKK------------------
NECC--------------------------------HT----E---C-KKTYSSEETISSSEGKLAEKNLQKMN-DLK------
ILKVKSVTSDASAQLSKVVREF-------GK-K--SGNSIRYYQCFVHRMRT--VQKYIKNLT----FSK-LQTG----------
YDRDFFRQRLATC--------------------------IRSRVRLELVRIHK-IST------------------------QLN-FATLAQS-
------------AIKNIVSCFS-GNHAN-------CKENSV-----------------ACCA-------------------------------
---HL-D----T--------Y--NTK-FLPYG--------------
VYLN-LSVVDKKK--

>KolobokP-3_MyEd_1p

---MV--
---N-T-----KK-------S---A----CNWLLKP--------
GCIPWN-KSMDTGHDGTTSTDGTLSTDTDYTA------KNNSALKVSGVSCVSFNNLSLLSSSDSSVASDF---SDSPNLHPNSEP---I-
KFSRPDKTVY-D-EALKSEAKWAG----------------CKPGEITFL--PTKLR----------PKKM--EN-H--------FHDETE---
------------------I-DL-----AD-D---E------------N--------ENLIVSVNKLSAAISAFLPH--FQ-VRKK----CK-
SPNP--TIQVTERNGICVSITVSCRN--CTFKSS---------Q--IEMFKRIVN-N---N-----A-P-G---------PNPGE--------
-------------LNEALAIPVMKT---KCGPGDVVFLLSC-LNIRPPSLSLINRKVNKTCDKMV-SL------------NENSMIENQQYV-
----KKVNNLL----GH-----DNFI----------------------DVETDTSYNNRPQAG-----GESATQSFTPLVELNTTK-------
------------------------------KLTIALDVKNKLCKK-------------------RKCC-------------------------
-------HE----ND-YC-KKNYATEESISSSEAKSAKINLQKVN-SGG------ILKINSVTCDASAQLAKTIRET-------SE-N--
FGYTIRHYTCFIHKMRT--IQKNVKNLK----LTR-TPAN----------TNRDIFLQRLATC--------------------------
VRARVRLEIVRIKKNFPL------------------------EDT-FCDKAMA-------------AIENIVSCFS-GRHTN-------
CKKNSI-----------------VCIA--
--------------HL-A----M--------Y--NTN-FLPYG--------------RHLE-LCTEDELKLKNAIQ-------------
KSLSLETMQKISRLLNTNKSESLH-------------------HRVFTYAPKNT---------------------------------------
--------------------VW-----------------------------SRNFSA-----------------------LCHSAVHSAS--
IGTG------RSALLIA-K-----QT-GIKYTKLNTFFRYM----------VKRDDSDRYHL-------------------------------
---------------EKKQTPWYKKNRFINRKRKFM--R---TPLANEI--
---NI----

>KolobokP-2_PoSt_1p

---MA--
--------------------------------AGRPRK-------R-A-K-----RP-------Q-----------EFTQ--------
HSQPWN-Y----------GVKVSKSDQP--GSSLQSGSAP---P-
LIKRPRFDVY-Q-ESLSS-------------------------CGPNEV--PSKLR----------PEKE--RQ-N---K-EKFFVGSRE---
------------------A-TC-----VN-N---X------------S--------ENVIINISKLNMILENLRLMH-D--RLGN----CK-
RYCP--QINKSRTQGLCAFLTATCTS--CNLKES---------E--QAAFSEKC--QG-QT-----R-R-G---------PVTGN--------
-------------LNEMLALSCYKT---KVGVTDLRFIISC-LNILPPSSGYLYKKLNELSSTII-SV------------NECVLVENQKFV-
----QEVNMLT-----------YKGI----------------------HVQTDTAYNHRPQSG-----GESATQSVAIVMEHTTSR-------
------------------------------KLPLCVSIANKLCSK-------------------KTCI-------------------------
-------HL----N---C-KKNHRTEDSMSSTETKLAKENLNKLE-MLN------ILKIDSLTCDASAQLEKTVREH-------SA-K--
VQRPIRQYTCYVHAMRT--LQKHVRNVD----LTG-PFPG----------KDKHLFQQKLATA--------------------------
IRRRVHFELVRIQRLFSS------------------------EEV-FLAKAKA-------------AVQNIIQCFS-NEHGN-------
CRKHSL-----------------VCLA--

--------------HL-A----S--------Y--SSK-FLPYA--------------KHLN-LCSEDTEKLESVLR-------------
NFVSDVKLSKISRLLNTNKCESLH-------------------SRIFTYAPKTS---------------------------------------
--------------------VW-----------------------------SRNFDA-----------------------LCHSALHSSV--
FGTG------KSTLILA-E-----SR-GIVCNQSEPVFQHM----------VERDQRTAKDR-------------------------------
---------------ERKSSLPYKYTRYIKRKQKCN--R---TLIQNSV-------------FANSVN-------------------------
---NFVTN-EH--SY--
TLNPLN--
>KolobokP-1_MeNe_1p

---MA--
--------------------------------AGRPKK-------I-A-K-----RP-------Q-----------EFTQ--------
HSQPWN-Y----------GVRGSKSDQP--GSSLQSGSAY---P-
PIKRPRLDVY-E-ESLSS-------------------------CGPNQV--PSKLR----------PEKE--RK-N---E-EKFLAGSHE---
------------------A-TG-----VD-N---I------------N--------ENVIINISKLNMILETLRIMH-D--RVGN----CK-
RYCP--QMYASKTQGLCAFLTVKCTN--CNLKEA---------E--QAAFPEKC--QG-QR-----K-R-G---------PVTGN--------
-------------LNEMLALSCYKT---KVGVTDLRFIISC-LNIRPPSSGYLYKKLNELSSTII-SV------------NESVLVENQKFV-
----QEVNQLT-----------YKGI----------------------HVQTDTAYNHRPHSG-----GESATQSVAIVMEHCTSR-------
------------------------------KLPLCVSIANKLCGK-------------------KTCI-------------------------
-------HL----N---C-KKNHRTEDSMSSTETKLAKENLNKLE-MLN------ILKIDSLTCDASAQLEKTVREH-------SA-K--
VQRPIRQYTCYVHAMRT--LQKHVKNVD----LIG-PFPG----------KDKHLFQQKLATA--------------------------
IRRRVHFELVRIQRHFSS------------------------EEV-FLAKAKA-------------AVQNIIQCFS-NKHGN-------
CRKNSL-----------------VCLA--
--------------HL-A----S--------Y--SSK-FLPYG--------------KHLN-LCSEDIEKLECVLR-------------
NFVSDVKLSKISRLLNTNKCESLH-------------------NRIFTYAPKTS---------------------------------------
--------------------VW-----------------------------SRNFDA-----------------------LCHSALHSSV--
FGTG------KSTLLLA-E-----SR-GIVCNQSEPVFQHM----------VERDQRTVKDR-------------------------------
---------------ERKSSLPYKYTRYIKRKQKCN--R---TLIQNSV-------------FANSIN-------------------------
---NFVTN-EH--SY--
TLNPLN--
>KolobokP-3_MaMa_1p

---MA--
--------------------------------AGRHKK-------R-P-R-----RH-------Q-----------QFVQ--------
HSIPWN-R----------GVEVSTSDPP--SSSWQSGSVP---P-
LIKRPRFDVY-Q-ESLSS-------------------------CGQNEV--PSKLR----------PAKX--RK-N---E-EMFLSGSHE---
------------------V-NG-----VD-D---T------------N--------ENVILNIAKVNIILXDLHQMH-E--EIGN----CK-
RYSP--QMVVSKTQGLCAFVTVKCVK--CNLVNV---------E--QPVFKEQG--QE-WR-----K-R-G---------PVTGN--------
-------------LNEMLAFSCFKT---KVGVTDLRFILSC-LNIQPPSSGFLYKKVNELSSSII-SV------------NESVLLENQKFV-
----YEVNQLT-----------HTGI----------------------HVQTDTAYNHRPQSG-----GEAATQSVAIVTEHSTSR-------
------------------------------KLPLCVSIANKLCRK-------------------KACT-------------------------
-------HF----N---C-KKNHRTEDSMSSTETKLANENLTKLE-TAN------ILKIDSLTCDASAQLEKTVREH-------SA-K--
FKRPIRQYTCFVHVMRT--LQKHVKNVN----FIG-PFKG----------KDRHLFQQKLATA--------------------------
IRRRVYLELVCIQRYFNS------------------------EEI-FLAKAKA-------------AVQNVIPCFS-NRHEH-------
CRQNSL-----------------VCLA--
--------------HL-A----S--------Y--SPK-FLPYG--------------KHLN-LCHEDSEKLILVLR-------------
NFFSDVKLGKISRLLNTNKCESLH-------------------NRIFTYAPKTS---------------------------------------
--------------------VW-----------------------------SRNFDA-----------------------LCHSALHSSV--
FGTG------KSMLLLA-Q-----SH-GIVSNESDPVFQHM----------VERDRRTVKDR-------------------------------
---------------ERKSGLQYKYTRYIKRKQKCN--R---TLIQNSV-------------FANSTN-------------------------
---NVVTN-EH--SY--
SLNPLN--
>KolobokP-6_CGi_1p

---MK------T---
--------------------------------NSYKKN-------Q-S-RK----KK-------V-----------LFKA--------
GHTYLP-----------PRVSASGNEDQA---------------------------------STASGGKEV----------------HQP-
QWLRPSYEEY-W-DAVHMSA-----------------------DDDSLR--PTKLR----------PAKS--AN-------------FRL---
------------------E-ED-----II-S---C------------E--------ENIIVNIQSLGSLLQNSSDHH------------CE-
NPAM--NFYITKRQGLCITGRAECSY--CHFKST---------E--VKLHTTFK------K-----K-R-GLKPVPEKERPETGT--------
-------------LNDGLALALTKS---KLGVADAKLVMSC-LNINPPDGRGLQRKLNQMCDRVE-AI------------NEASMVENQQYV-
----RRVNTLR----GE-----GDAV----------------------DLETDTSYNNRPQAG-----FEAATQSFSPMVEASTPR-------
------------------------------KLVVSLQTANKLCCK-------------------RKCE-------------------------
------NHN----N---C-KNNYYTEDSISSSEAKLLRKNLDFIQ--------------TRISCLGPSE--KTIRDY-------SE-D--
HNLNIKHYHCFIHKLRT--LQKNLRSIR----LKTRLAAG----------QDREAFIQRLATC--------------------------
IRARIRYKLVKIRRLCRV------------------------DAT-FLRHAQN-------------AVLNVLPCFS-GIHDK-------
CRRSSF-----------------TCCA--
--------------HL-P----G--------Y--TT---------------------FRME--------------------------------
---------------------------------------NTWTWTLPTWM---
----------------PF--SPSSASISAM--MVSG-
--KS-ASWQPLT----------GV---
----------RTSTSVSSRTRPRTPRGPGTS--QPC-ATLQSTP---
----PR--MAP-AS--PW---------

>KolobokP-1_CGi_1p

---MP--
-------------------RSKR---------TQYKIN-------H-G-K-----RK-------G-----------YFQK--------

SHPYFS-----------SKVVPVGGCHD----------------------------------DDVGDADDS--------SRSVSAS---D-
QWMRPPQEAY-R-DALSSMS--------------SSCQ-----QSGAVL--PSRLR----------PVKP--TT-------------VKI---
------------------A-SE-----DR-S---D------------G--------ENIIVNVQSLTMLLQETAVHH-----------YCS-
SPKM--NFSISKRQGLCITGQSKCLT--CRFSSS---------Q--VQLYETHK------K-----S-R-G---------PETGT--------
-------------LNEALLLGCLKQ---RLECQTLASFSAA-LMLRHLIQGGMQRKLNSLCDRVE-EI------------NSESMRENQRYV-
----RRVNRLA----GR----EETEV----------------------DLETDTSYNNRPQAG-----FEAGTQSFSPLVENNTPR-------
------------------------------KLVLVLQTANKLCSK--------------------RSCD-------------------------
-------HR----N---C-KKNYESDETIASSEATLVRRNLDSLR-EEN------ILNVRSVTSDSSAQLDKTLRDY-------SA-A--
TGTKIIQYKCFIHKLRN--LQKNIRSIK----LKSPLPPG----------SNRDAFIQRLATC--------------------------
VRARVRYELVHIRKLCKS------------------------DEL-FIHKAEQ-------------AISNILPCFS-GVHDH-------
CRRMSF-----------------TCNA--
--------------HL-P----T--------Y--SAK-FLPYG--------------RHLD-LEPEDISSIQRVIN-------------
KQILGQNLPKICRLSTTNKCESLH-------------------QTVFTYAPKNT-----------------------------------
--------------------TW----------------------------ARNFTA------------------------LCHSAVHTST--
HGTG------SSSSLIA-N-----KV-GVRWDHAGPFHRKM----------TQLDRLRQYHS------------------------------
---------------LRKKTIKYKTTRHFGRKKKMN--R---KLRELSL-------------YNNERE-------------------------
---N-RAN-EHA-------

>KolobokP-4_DrPo_1p

---MA--
------------------KSNSR---------RSYRLN-------K-A-R-----KA-------F-----------WFRP--------
GNTFRQ-----------TGQNL--PSPTEDIAVP----------------SVP-
ATERLSAEEA-V-DVLHASS------------------------SGTTL--PYKLR----------PKAE-----------------IKD---
------------------I-PD-----CT-S---V------------D--------ENVIVNMQCLRNIIQQVHQQ--------N----CK-
NSLV--SVSVVHRRGLAISICAECRG--CHYKST---------P--MELSNTIK------K-----P-R-G---------PGAGV--------
-------------LNEMILLPVMKS---KMGMADVSLVLSC-LNIKPPSDSLMQKKMNLMSDKAT-TV------------NEDEMCNNQHYV-
----SRILTLS----GK-----DNSA----------------------DVQFDTSFSCRPQGG-----AEKAKQSFAPLIEHNTSK-------
------------------------------KFVLAASISSKFCKK-------------------KACT-------------------------
-------HD----N---C-SKTLATDQSISSTERSSLHTNLNSVL-KRR------VLNIRSVTTDASSQVAKALRDY-------NT-E--
NKTNMKHYHCFIHKLRT--LEKKIRNIN----LNSKLKAG----------QNKTIFLKSLASG--------------------------
IRTRARMELTNLKSVRSN------------------------EAE-FIERSTK-------------ALENIVPCFA-DSHVA-------
CSKQST-----------------VCQH--
--------------HL-V----H-----YGKY--SYK-HLPYG--------------QHLT-LSKDDQTTLKDAIM-------------
NTFNTETLRSVKELYSTNQCESMH-------------------STIFNYAPKFT-----------------------------------
--------------------CW----------------------------TRNFSG------------------------LCHSATHSRT--
LGRG------RATLILA-R-----AV-GINVKKNSQMYMNL----------NRIDQKSQYHS------------------------------
---------------RRKKSQAYKQSRYFLRKRVSN--R---PLLQESL-------------YSCEPS-------------------------
---T-SSQ-DH--SY--
GITY---
>KolobokP-5_DrPo_1p

---MA--
------------------NHKVR---------RSYKKS-------R-G-K-----SQ-------S-----------WFKR--------
GHVGFS-R--------QTPQEI--QNMSTSAAED------------------S-
QTKRLSVREA-E-DVRHAAE------------------------DINGATL--PYQLR----------PQPQ-----------------NEE---
------------------R-ED-----MA-E---L------------D--------ENIIVNIDCLHNLVREVHKQ--------S----CS-
KPCV--KVAVTKRAGLCVTVQMKCSS--CKYQAP---------P--VNLTDTMK------K-----S-R-G---------PAAGV--------
-------------LNDMLVLPVLKS---KLGLMDVSLVLSC-LNIKAPTQALLQRKLNNMSNTVS-RL------------NEDQMMHNQQYV-
----HRVLQLS----GK-----EAAA----------------------DVQFDTSYASRPQGG-----FEKAKQSFAAVIEHNTKQ-------
------------------------------KLVLSTAIANKHCAS-------------------KTCA-------------------------
-------HN----N---C-SKNFPSQQSIASSERHLLLENLNNLD-KKK------ILKIRSITTDASAQIGKALRDF-------NS-K--
KSSNIKHYHCFIHRLRT--LEKKIRNIK----LDSKMLGT----------QDKTNYIKMLASG--------------------------
VRTRVRMELQNLKTIKSN------------------------DGNYFVTRSSQ-------------ALANIIPCFS-NDHSK-------
CSKAST-----------------VCQH--
--------------HM-S----S-----IGKY--SVK-HLPYG--------------QHLH-LSNTDSSRLKDTIS-------------
STFDTETLQYVSNLYNTNRCESLH-------------------STIFNYAPKFT-----------------------------------
--------------------CW----------------------------KRNFSG------------------------LCHSATHSRT--
LGKG------RATVILA-R-----AF-GIKVKQHSQMYRNL----------QHIDKRSTYHS------------------------------
---------------RRKESFQYKQTRYFLRKRVSN--L---PLLKDSL-------------YSSQPS-------------------------
---S-SAG-DH--SY--
GISY---
>KolobokP-7_MeMe_1p
M--
---LK--
------------------GSTSK---------RSYXHA-------H-K-R-----RK-------S-----------YFKI--------
GNQLYK-N-----------RHKPCEQAE-------------------------------------VLTSGQD----------QVET-------
FTQRLSXSDA-Q-EILHISR--------------DD-------NPDLTL--PYRLR----------PKPE-----------------KDN---
------------------D-NN-----XN-K---L------------D--------ENVIINISNLQELVKSLNGH--------I----CD-
QPHL--ETQITDRTGLCITLSVNCHH--CHFSSG---------E--INMSEKVK------K-----S-R-G---------PPAGA--------
-------------LNEMLLLPVLKS---RMGLSDVLLVLSC-LNIKTPNRTTLQSKLNVLSDAAT-QI------------NKKQMTSNQRYL-
----ARITSLA----GK-----EKSV----------------------DTQYDTSFSSRPQAG-----SEKAQQSFSPLIEHTSLK-------
------------------------------KHVLSIAVANKHCMK-------------------KNCN-------------------------
-------HQ----S---C-KKNYPSDASISASESTLLHQNLNNIS-EAG------HIKVRSVTTDSSSQVAKALREY-------NA-E--
KKSNIKHYKCFIHKLRT--LNKHVKNXK----LSS-IPKK----------HDKNTYTQKLATS--------------------------
VRTRVRMELQNLRGLKPS------------------------TEK-FVEKGSL-------------SLKNIVPCFS-GHHKN-------
CRQHST-----------------VCSS--

```
--------------HL-K----S--------Y--NTN-FLPFG--------------VHLS-LSGSDQDKITKEIT-------------
TVLDKNELRSLSHLYNTNLCENLH-------------------STVYTYAPKFT----------------------------------------
--------------------CW----------------------------SRNFPA------------------------LCHSATHSRT--
MGSG------LATILLA-K-----SV-GIKTRGRSRMCLQL----------KKYDEQREYHQ-------------------------------
---------------RRKRTTKYKQTRFFSRKRKLF--R---TVYLKSM-------------YTDLPS-------------------------
---------TS-----------------------------------------------------------------S-MNX-DH--NY--
GLRV-----------------------------------------------------------------------
>KolobokP-7_CorFlu_1p
------------------------------------------------------------------------------------------
-------------------------------------------MG---------------------------------------------
--------------------YKK---------KSYKYS-------N-A-K-----KA-------T-----------WFKK--------
GHNVRK------------QIKLHLPNEEF-----------------------------------------------------LNPAVGS-TDI-
KSKRLTLSEA-C-EVQHLMEH---------------------QTQPNTL--PYRLR----------STAE------------------SEQ---
-----------------N-SDL----MC-N---S------------D--------ENIIVNFKKLQEFTKMIHSK--------C----CT-
RPQI--ELSMS-RTGLCVSLTAYCSR--CQYRSP---------S--MDMSEKIR------K-----A-R-G---------PAGGA--------
-------------LNEMLLLPVMKS---KMGIDDVLMVMAC-MNIKAPSRSVLQKKLNSLSDLMI-NM------------NEAQMIQNQEEV-
----KHLTEQA----GL-----PCEV----------------------DVQTDTAFASRPQSS-----HEKAQQSVAATIEHTTKK-------
------------------------------KRIIALSVVNKHCNI-------------------KDCN-------------------------
-------HQ----N---C-HKNFSKEKSITSSERTLLHENMSKLK-KTK------IVKVKSITTDGSSQLAKAVRDF-------NS-A--
KSENIIHYQCFIHKQRT--LEKHIKNLS----LKT-IPSR----------YNKSTYMQKLASC--------------------------
IRTRVRLELQSLHALKKN------------------------TNN-YIGCCIL-------------ALENIVPCFS-GNHVN-------
CKVKST-----------------VCKS-------------------------------------------------------------------
--------------HL-E----S--------Y--TTK-FLPWG--------------VHLE-IDGTDRKSVKDEIM-------------
KVYNIDALKKTVKLFNTNMCESFH-------------------STVFSYASKSI----------------------------------------
--------------------CW----------------------------ARNFAG------------------------MCHSAAHTRT--
LGRG------MSTVDLA-R-----AV-GIRVSKHSSMMQQL----------VKMDKRNKYDS--------------------------------
---------------HRKSTKEYRENRYFLRKKKSN--R---LLYQDSL-------------YSAEPS-------------------------
----------------------------------------------------------------------------T-SSL-EH--NY--
GLSF-----------------------------------------------------------------------
>KolobokP-1_CySi_1p
------------------------------------------------------------------------------------------
------------------------------------------------------------------------------------------
------------------------------------------------------------------------------------------
------------------------------------------------------------------------------------------
------------------------------------------------------------------------------------------
------------------------------------------------------------------------------------------
------------------------------------------------------------------------------------------
------------------------------------------------------------------------------------------
------------------------------------------------------------------------------------------
------------------------------------------------------------------------------------------
-----------------------------------------------------------------------------------NSIHYTA-
-----------------------------------------KKTYMQKLASS--------------------------
IRARVRLELQSLQARKRN------------------------END-YIGRCIL-------------ALENIVPCFS-GQHTT-------
CKLKST-----------------TCRS-------------------------------------------------------------------
--------------HL-E----T--------Y--TTK-FLPWG--------------THLE-LNSTDKRLLTDEIL-------------
KVYNTEALKKTAMHFNTNMCESFH-------------------STVFTYATKRV----------------------------------------
--------------------CW----------------------------ARNFAA------------------------MCHSAAHSRT--
LGRG------KATIQLA-K-----AA-SIKVPPKSRMANQL----------HSMDKRNQYHS--------------------------------
---------------KRKSSDKYREKSIF-----------------------------------------------------------------
------------------------------------------------------------------------------------------
------------------------------------------------------------------------
>KolobokP-1_GarTel_1p
MK-------------EP-----------------------------------------N-----WTFYL------------------
ICYEEDQNIC-TNSYFLCG---LSLTKKIFHK---EIGITYTK-------ME----------------------------------------
----------------------KRCKRRDR-------TMVSCNNS-------R-K-K-----RA-------T-----------QFKP------
--GRSAAT-------------TVEVLPETT-----------------------------------PQAVRSTCGG-----------------NES-
IRSRLTSQEA-K-DLDAISS-------------------------SPNL--PYTLR----------PKAV--MD-------------ECP---
-----------------G-IE-----NL-E---M------------D--------ENVIVNMRKVNELVNRVHGL--------T----CK-
NPRV--SVGIVDRKGLCVSFEVKCSA--CRLRLH---------P--VQMSETVK------K-----A-K-G---------PPAGA--------
-------------LNEMAVLPAVKT---KVGLADITTVLTC-LNIKAPCKRAMQSKFNSMCDKMT-QM------------NERQMERNQEYV-
----QHVTSLI----PT-----EAGI----------------------DAQYDVSYTSRPQGG-----SEKAEQSFAAIIEHTTTR-------
------------------------------KLPIAAGIANKHCTI-------------------KDCD-------------------------
-------HT----QA-RC-KKSYSSDETIASSERALLHKTLDDVK-RTG------LITLRSVTTDASTQLARALKDY-------NK-KD-
VNSNIAHYNCFIHKGRT--FEKHVRALQ----LRS-IPKR------H---TNKPKYRQKLASC--------------------------
IRSRIRIELKRARAITRT------------------------DDS-FIRAGSA-------------ATHNITQCFS-GHHRL-------
CRDVSH-----------------VCKS-------------------------------------------------------------------
--------------HL-T----A--------Y--TTD-HLPYG--------------VHLE-LSETDLCLVQKEID-------------
KMFDENGLKSISKLFNTNMVESLH-------------------SAVYRLAPKST----------------------------------------
--------------------CW----------------------------KRNFPA------------------------LCHSAVHSSS--
IGPS------RSTIYLA-Q-----AA-GIKIYKTSRLYSQL----------KLKDRQRQYDS--------------------------------
---------------KRSASSKFKQRRYFLRKRKLF--W---PLFTNSL-------------YSTGNE-------------------------
---------AS-----------------------------------------------------------------Q-SAS-EH--NY--
GLLS-----------------------------------------------------------------------
>KolobokP-8_DrPo_1p
------------------------------------------------------------------------------------------
-------------------------------------------MY---------------------------------------------
-----------------KRADK---------ISSNNS-------Y-K-K-----RK-------T-----------QFKK--------
```

GFGVMK-SDDANA--KSVHLSEPLPSTS-----------------------------------QCSDTVDA----------------VDS-
VKSRLN-QEA-A-DLKYMSC-------------------------TEYL--PYTLR----------PKAQ-----------------ESP---
------------------A-CE-----ND-V---V------------L--------ENVIVNVGKVAQLVSRVHEA--------S----CQ-
DPRV--KVDVVNRMGLCVSFAVKCNS--CNFSLE---------P--IQMSDTVE------K-----A-K-G---------PPAGA--------
-------------LNEMAVMPAVKT---KMGLTDITTVLTC-LNIKAPSKTAMQTKFNALCDKMA-AV------------NERQLIKNQEYL-
----RKVTSLL----PA-----ISGI----------------------ETQYDVSYSSRPQGG-----SEKAQQSFADIIEHTTTR-------
------------------------------KLPIAAAVANKHCRR-------------------KDCD-------------------------
-------HR----QD-KC-RKTYSSEKSIAASERSLLNETLNNVR-TAG------LITLRSVTTDASTQLAKALREY-------KA-E--
NNLNFSHYKCFIHRGRT--LEKHIRALQ----LCS-IPKT----------CDRQAYMQKLASC--------------------------
LRGRVRIELKNARALIKG------------------------DGS-FIRAGAA-------------AVRVITKCLS-GDHRH-------
CKGTSF-----------------VCRG--
--------------HL-L----T--------Y--STN-CLPYG--------------MHLQ-LNNKDQSLIEKELE-------------
KMFNEDGLKDMCKLFNTNMVESLH-------------------STVYRLAPKSL---------------------------------------
--------------------CY-----------------------------SRNFSA-----------------------LCHSAVHSTS--
VGPS------LSTLQLA-Q-----AA-GLNVKENSQMYKQL----------RLKDRQRKYDS-------------------------------
---------------KRRASADYKQRRYYLRKKKSN--R---SLFSNSL-------------YSIGQE-------------------------
---------AS--P-MAQ-EH--NY--
GLTS--
>KolobokP-1F_PhoAus_1p

---MY--
-------------------PEKT---------RSFTRS-------R-G-R-----RK-------S-----------QFKK--------
GHRPSN-MD---HCSSSIPR----------QPEAAI-DGC-
RAVRLTHFEA-E-DVTYLTD-----------------------SDAAAT--AYRFR----------PSTE-----------------KER---
------------------KIRD-----SK-G---L------------D--------ENIIINVRRLTGLVGHVHQV--------A----CK-
FGKL--ELIVEKRLGLCVYASLHCRV--CKYKSP---------T--VPLSDEVK--Q---Q-----T-T-G---------PPAGA--------
-------------LNSQAVLPVLKS---KVGIEDVINILTC-LNIVPPSNRSLQNKMNAMSQTVT-QL------------NEEQMIENQRYV-
----KKVTTLA----GG-----EGVA----------------------DVQFDVSYSSRPQGG-----CEKAKQSFGGLVEHTTTR-------
------------------------------KLPLALATANKHCRI-------------------PGCK-------------------------
-------HE----H---C-SKNYPAEKSIASSERTLLQASLRAVE-NSG-----VDLNIRSITTDASSQEAKAIRDF-------YS-A--
KTKKPKHYKCFIHRLRR--MGKSVNEAD----LKS-LPRT----------HNKTGYKRKLSSA--------------------------
VRSRVRKELINLKNRKIG------------------------RDE-FLKIGAA-------------AVNNVTSCFS-GQHQQ-------
CRELSS-----------------VCIA--
--------------HH-S----N--------Y--TTS-HLPYD--------------RHLE-LSEKDLKTIQTKIN-------------
SYFNQEQLADMKYLFNTNICESLN-------------------AAVYAYAPKTS---------------------------------------
--------------------RY-----------------------------TRNFTG-----------------------LCHSAIHSRT--
LGQG------KSTVKLA-E-----AS-GLSVPENSAMKRKL----------RNVDCRKLYHA-------------------------------
---------------TRKSSQQYKKARYYLRERMAN--T---NMFDKSV-------------YSTENM-------------------------
---------AS--T-SAT-DH--SY--
GLSY--
>KolobokP-7_DrPo_1p

---MP------------------F-----------------------------
---------------NYRKSAGN---------ISTRRS-------R-G-K-----KA-------T-----------QFKL--------
GHKPKW----------TTSTEI---------------------------------------HQPGVSSAS---------SEAPKAS---K-
RLVRLTDKEA-R-DSQFVRTTAG-------------------LAQADTL--AYTLR----------PKPE-----------------KEE---
------------------K-TD-----CECD---R------------N--------ENFIVNNEKMLALIKDVHAS--------G----CK-
KPNM--KMKVT-RAGLCVYICVKCSY--CGFNSQ---------T--LPMSDTIQ------T-----A-R-G---------PPAGF--------
-------------LNYLVAMPVLKS---KIGMDDVATVLTW-LNIKAPCKQTFQKKFNDLGQAQT-EL------------SDKQLELNQDYV-
----ANVMKKA----GF-----EPXV----------------------DIQFDVAYTCRPQGG-----CEKSTQSFGALVEHNTGQ-------
------------------------------KLPLAIAMANKHCRK-------------------RYCR-------------------------
-------HD----N---C-SKTFAAEASIASSERVLLHRSLDKVK--DG------PLAVRSITTDSGTQSAKAIRDYYELKKQTNH-L--
KKQTATHYKCFVHKLRA--LERHVRAAKSD--IKS-IPKK----------YNKDEYMRKLASC--------------------------
LRCRVRIELERLRKQSCS------------------------DEL-FLRSAKF-------------AIENIMHCFS-GDHKM-------
CKERSR-----------------VCTY--
--------------RV-T----S-----------SYK-HLPYG--------------EPLA-LQESDKKIVLGNIN-------------
KTFDATGLKEVAKLFNTNACESLN-------------------ASVFHYAPKTS---------------------------------------
--------------------FY-----------------------------ARNFAA-----------------------LCHSAVHTRS--
LGPS------KSSMKVA-E-----KVTGKKISLHSMIYNQL----------KAKDRRREYDS-------------------------------
---------------RRKASVRYKIVRFYLRKRHAN--R---ALYRDGL-------------YASESM-------------------------
---------PS--T-SAA-DH--SY--
GLKV--
>KolobokP-3_MeNe_1p
MY-------------GK------------------C------------SFTPLRRS-------------------------------------
----PLGHFHTGQLILLLKLLLFRQ---VFFPSYCN-------MP--
---------------TLNSKVKR---------KSCKRN-------Q-G-R-----RK-------T-----------QFKM--------
GHPFLA-SYQ--NSSEVDADT--------PVPAKAP---Q-
QLMRLTPDET-F-DVIQASN----------------------VSSASVL--PYTLR----------PRRN--EN-P--------ELQEEC---
------------------S-QS-----SE-E---S------------D--------ENVIINFKKLTHLFEAFIPHM------------CD-
VPKP--QVIIDKRMGLCITAAVTCTN--CSFKSM---------G--VELYETVK------K-----S-R-G---------PSAGV--------
-------------LNESLVLPVLKS---KVGIADVLLVLSC-MNIKVPDAHGLQRKLNILSDKMV-DL------------NEGHMEKNQQYV-
----HCINLIA----GS---IDDEAV----------------------DTEYDVFYASRPQAG-----CEASTQSFAPLIERNTRK-------
------------------------------KLVISLQVGNKLCRK--------------------LCD-------------------------
-------HK----NN-KC-QNKLKSEESIASAESKFLKRNLENIQ-SRK------ILRVRSVTSDASSQIAKALCQY-------ST-S--
QNHPIVHYRCFVHRLQI--IYKQIKNIK----LTS-CPKA----------YDKRLYCQKLVSC--------------------------
IQARIRLELVRQRSRCTT------------------------DDA-VVQYSKT-------------AIDNILYCFR-GQHTM-------
CSRLM------------------VCCS--

--------------HL-A----S--------Y--SPK-FLPYG--------------KHLQ-LSMSDIKKLQAKID-------------
NLCSQENLKQMSKLSITNKAESLH-------------------HHVFTYAPKNT---------------------------------------
--------------------VW-----------------------------S-NFSG-----------------------LCNSAAHSSS--
LGSG------QSTIHLA-K-----HM-GIEVKSQDPFYMQL----------NKIDMKAKYDS-------------------------------
---------------KRQMTFHYKRNRYFLHKKKNN--Q---TICKSSV-------------FTGLQE-------------------------
---IAD-EH--EY--
SLNPVS--
>KolobokP-2_DrPo_1p

---MR--
----------------SLHKKSQ---------HSYKNN-------R-A-RK---NKG-------A-----------LFKK--------
GVFPNY-NS--------SHVRCVEKQDQ----------------------------------DSIPLNLD----------------PDLE-
SVVRLGDSVA-N-DVLNACN--------------RS-------RDPLVL--PFKLR----------PRQT--VP-Q-----------PEK---
------------------Q-SA-----NI-S---D------------N--------ENIIVNISQFGNIFKCIGNH--------S----CD-
QPSL--DYNITDRKGLCVDIKVLCHN--CDFCSD---------E--IPLYTSIP------Q-----K-H-G---------KPLGV--------
-------------LNVSLLLPVLCS---KLGISDIQLLLAC-LNIQAPDKRGMQRKLNTVADQVE-DM------------GRQQLVKNQEYV-
----RRISNLA----GC-----SGET----------------------DVEFDVAYTSRPQAG-----CDTATQVFAPVIEKTTSK-------
------------------------------HLPVDLHIGNKLCSK-------------------PNCN-------------------------
-------HL----DR-SC-KKNYCDETSINQAEAIFLKKSLENIK-NQN------ILKVKSVTTDGSASLAKAMREH-------NA-K--
VQEKVQHFKCFIHNMRN--LHKHLRSAC----IKQ--PKG----------MDRLLYCKKLATA--------------------------
IRTRVRLELTRLRKLLRG------------------------DEL-YLARARE-------------AVTNVLDCFS-GSHDS-------
CKHKSV-----------------VCTA--
--------------HL-K----G--------H--STR-YLPYG--------------KNVV-LSAADKQKLQKVLD-------------
KYCGVQQLRDTVQLHNTNRCENMH-------------------SRLFSYAPKSM---------------------------------------
--------------------VW-----------------------------KRSFTA-----------------------LCYSATLGAS--
VGRG------LSLLQLA-G-----RC-GINIEASDPMYRYA----------MFTQNIARYHS-------------------------------
---------------DRKQKHEYKTSRYLSHRKRAN--R---AVLSQSL-------------YKA----------------------------
---------PS--K-VTK-EH--DY--
GINLGN--
>KolobokP-5_MeMe_1p

---MR--
----------------STKKKSE---------RKYKTV------SR-N-Q-----RA-------G-----------FFKK--------
GHKPYY---------LYTNTQCLSE-------------------------------------ESVVAGCSFD-------HSHQGGV---S-
TVTRLSASVA-A-DVLNATST---------------------DDTDLTV--PFKLR----------PAAA--PK-K---P-------------
--------------------PEPV---NK-NI-HL------------NLTRMRRKTMKILSNLDMLKCAFEMVSRHT------------CP-
VPDI--TLGRANWKGLLVEIDIVCRN--CDFHTG---------L--IPVTVPTT--V-----------------------MNSSL--------
-------------INTSLLLPVMLS---KLGINDLQLVLAA-LNIHAPDKRGMQRGLNFLADEIQ-RL------------SRKQLIKNQEYV-
----KQIEKHS----GK-----TDGT----------------------DVEYDVSYTSRPKAG-----SDTASQCFAPLLEKTTRQ-------
------------------------------HLPVDIQIGNKLCPK-------------------RNCG-------------------------
-------HK----SD-TC-KKNFDSDTSITNAEKILLKSSVKSVD-KSN------IITLNTITTDGSQQISKALREI-------NT-R--
RKKNITHYRCFVHNMRN--FHRHLKNAK----IKA--PSG----------QNRKVFTMKLASS--------------------------
LRMRIRLELKRLKTVCKG------------------------TAS-FIHKAHA-------------SVNNILDCFH-GNHTL-------
CRKKSV-----------------VCTA--
--------------HL-K----K-------RF--TMS-HLPYG---------------KLK-LSCKDKQIVMSYIE-------------
KYCGLDQLKHSAELYTTNSCENMH-------------------SRLFSYAPKTT---------------------------------------
--------------------VW-----------------------------SRNFPG-----------------------LCHSVSLGAS--
IGRG------KSLLQIV-Q-----HL-GIPFIQ-DPIYKYA----------LKAERVAMYNR-------------------------------
---------------SRKRSYKYKTSRYVKRRKYSN--R---IILENSI-------------YRSRTT-------------------------
---ASK-EH--TY--
AIDMLK--
>KolobokP-3_CorFlu_1p
MFL---IRKTSR--------------------------------
ADNEV---FCHYFRG-LCSKKKQNLTTN---------TNLVK----MR---
-------------------TNSSKSR---------LSYKNS-------L-K-R-----RK-------T-----------QFKK--------
CHKVWN-K----------SLKSDKN-------------------------------------EINEPGPSH------KQDLPNYAE-----
PVVRLEASVA-E-DVCNAQLG---------------------YSTLYNS--PVKLR----------PKKK--KE-S---------SKDVN---
------------------E-PQ-----HV-G---E------------N--------ENIIVNFKQLESLFQKFAAH--------T----CD-
NPNI--KLTLEDRQGLCVSLKVKCSN--CKFITD---------T--SKLFNSIK------S-----T-R-G---------PDAGC--------
-------------LNSALLLPVLLS---KVGINDILLMLSA-LNIQAPNKRGLQKKLNTLSDKIE-NL------------GKEQMIQNQHYV-
----KKIQSLS----GT------SGT----------------------DVEFDVAYTSRPQSG-----CETSSQSFAPIIEQTTSR-------
------------------------------HLPIYLSTANKLCLK-------------------QKCN-------------------------
-------HN----NR-NC-KKSYSTEDSIASTEAVFIKSAIESVE-SSK------ILKIRSVTTDASLQIAKSLREI-------NS-E--
RNSKINHYKCFVHNLRN--MNKHLKAIK----FNK-IPPG----------QNKKYFASKLASS--------------------------
IRVRVRLELTRLKICSKT------------------------YEE-FLQKAQV-------------SIENILPCFK-GLHIN-------
CRRDSV-----------------VCKS--
--------------HL-K----C--------F--KLS-YLPYG--------------KYLK-LPDSDISKIKSIIS-------------
KYCGPAMIKDISQLKTTNQCESFH-------------------NRVFSHVPKNT---------------------------------------
--------------------VW-----------------------------SRNFSG-----------------------LCHSATIRAS--
IGRG------STLLKIA-Q-----NL-GLPVSDHDPLQRYA----------LDTDKNDKYHM-------------------------------
---------------NRKKSFKYKTFLHLKRKRNSN--R---SIISQSA-------------YSDATD-------------------------
---LTE-EH--PY--
ALNPN---
>KolobokP-5_CorFlu_1p

---MR--
----------------TRSSKSR---------NCYKNS-------L-K-R-----RE-------T-----------SFKK--------

```
FNKACK-K----------TCNSGQN-------------------------------------ESNVQGPSNE----YEQILPDNSK---S-
VFTRLDASEF-N-DVCNANQS----------------------DAGCYTS--PIKLR----------PKKR--KE-S---------SPVVQ---
------------------Q-PG-----SV-G---E------------N--------ENFIVSLKKLESIFQAFAFH--------S----CD-
NPNV--TLSLEDRQGLCISIKVVCRK--CKFSTD---------V--TKMYDVIK------P-----S-R-G---------PSGGC--------
-------------LNSAILMPVMLS---KIGISDILLVLSA-LNIRAPDKRGLQRKLNHLSDNIE-NI------------GREQMLQNQQYV-
----QRIQSLA----GM------SGT----------------------EVEFDVAYTSRPKSG-----CETSSQSFAPLIEQTTTR-------
------------------------------HLPIAISTANKLCSK-------------------PACA-------------------------
-------HN----NA-QC-KKSYDADVSIGRTEATFIKSAIDSVE-SSN------IIKIRSVTTDASLQIAKALKEV-------NS-A--
RSIQIKHYKCFVHNLRN--MNKHLKAIK----FSK-LPQG----------QDKKFFALKLASS--------------------------
IRVRVRLELTRLKNSSKY------------------------NEV-FLQKAPM-------------SIDNILQCFQ-GKHIN-------
CRKHSL-----------------VCKA------------------------------------------------------------------
--------------HL-K----C--------F--KLN-YLPYG--------------KYLS-LPDSDISKIKSVLA-------------
KYCGPVILKDLCQLKTTNQCESFH-------------------NRVFSFAPKNT---------------------------------------
--------------------VW-----------------------------SRNFSG-----------------------LCHSVTIAAS--
VGRG------MTLLKVA-Q-----KL-GLPVTISDPLYHYA----------INTDKKEKYHM-------------------------------
---------------KRKKSFKYKTFLHLKRKRISH--H---AIISQSA-------------YSDDTD-------------------------
-------------------------------------------------------------------------------LME-EH--PY--
AINPN-------------------------------------------------------------------
>KolobokP-1_GiAe_1p
ME-------------------------------------------------------------------------------------------
-----LSHD---------KFLVTVA------------------MG-------------------------------------------IQ---
----VLSMNNWK---MAAPPKNA---------RNTKFS-------R-K-R-----RK--KG--TS-----------LFQK--------
GNCKWR-KTSEQP-------------------------------------------------QTRDKGQEK-----EQIQPMKTDT---Q-
RVKRIEIEIF-Q-SVST---------------------------SGNTL--PSKLR----------PKIK--TV-S-----------CKS---
------------------A-PR-----LN-N---F------------T--------GNVIVNINNLPLLVNKFCSIH-SA-HSNE----CK-
SPHC--VLRSIKYVGLCVSVSLGCQS--CNMNIT------------MDLTDKLPKPK---N-----K-P-G---------PPPST--------
-------------LNKALALSVLKT---KAGPSDVRYILSL-LDIKPPSMTQVQKDLKLLSNETV-HI------------NNVSMKKNQEFI-
----NEVAKML----NQVTSNSRGLI----------------------PVEADCSYNNRCQSG-----YEAGTQAFCPLVEQLTKR-------
------------------------------KLVLGLSTANKLCRK-------------------HQCS-------------------------
-------HT----K---C-ARTYNPTHSMASVEKKLISENITNVE-SQN------VVKISSLTTDACAQLGNIMQTSNPIHC--SS-H--
SNMFPLKYVCFVHRMRRVFINTKVAALK----LQY-KPIG----------LTKDAFANKLAVG--------------------------
MRCRLTRELSQLHKQCHG------------------------VKA-FVSKGAS-------------IRDNIVNCFC-GEHTH-------
CKTASL-----------------VCNA------------------------------------------------------------------
--------------KS-S----K--------YF-KPV-FLPNG--------------QYLQ-LNNTDRHSLMHTLN-------------
KTVSNIMLSQLVCLYNTNKSESLH-------------------HTVFTYAPKMS---------------------------------------
--------------------LY-----------------------------SCNFHG-----------------------LCHSAVHTNT--
HGRG------NSTLIIA-K-----KI-GIKYSNYGPMCDYM----------KKTDEKFQYDY-------------------------------
---------------KRQNTHRYKRDRYFSLLNRCH--R---KLYMHSS-------------YSSGSK-------------------------
-----------------------------------------------------------------------------H-INR-QH--NY--
AINLNHWQQQQQH---------------------------------------------------------QH
>KolobokP-1_TeGr_1p
MF-------------VN---------------------------------------H-------SFK-----------------------
SDDII---KQHD-------VISFCV---------------------MHKTV---------------FF---------
SKLCHQFLFLLEINIYK--H--FALSKPVAA--TAVMPKKSK-------HWYYFKINQF-----K-G-T-----RR-------H---------
--QFKI--------GNKLWN-RRTPN--------------------------------------------------NHSVVIPDN--------
VEPSSSS---N-QWVRPTQSLY-E-AAMYP--------------------------GNSCA--PTKLR----------PIPE-----------
------MKE---------------------T-ENLQRFEPN-S---L------------L--------GNGIIQFNKLPQLFNNFVSFH-S--
HNTE----CQ-KMHI--DIFKVVRKGLCIELQIYCVT--CGIKSE---------S--IKMYDEVH--KK-LS-----S-Y-G---------
PKPGS---------------------LNIALCLSILKT---KVGITDLQFIMSC-LDIKPPTRSSMQRLVNQLSNKVA-RM------------
NRDSMLDNQKFV-----KLVTEIR----GD-----PPTV----------------------DVETDVSYNNRLALG-----
FQSGTQAFCPLVENTTNK-------------------------------------KLVLNCQIANKL--------------------------
-------------------------------------NS---C-TQNVAPNTVLTTHEKTMTIRNVEEVN-ANN------
FVTVSSITTDGCPQMDSVASAI-------GV-AC-GKPPLRHFTCFVHKMRR--VYKNITSLP----LSL---PG------Y-
TTERRATFKKKLAIA--------------------------TRNRLTRELTRLKNNVSV------------------------ERQ-
FVEQAVR-------------IRENVLDCFS-GNHSQ-------CKKISL-----------------ICKP-----------------------
---------------------------------------------------------HL------------------PPI-HLPFG-------
-------RWLN-LCDSDKDRYTCTLR-------------KILNNKKLSEMTRLFNTNRSESIH-------------------
HRVFTYATKQV-----------------------------------------------------------TW---------------------
--------RRNFPG-----------------------LCHSAIHSDT--HGTG------KSTVLIG-K-----DV-GLNFHIQGPLMTQM---
-------VKVDTVAKYQS----------------------------------------------RYKAMLANKIKRYYARYRNNH--Q---
SILNNSM-------------YTANGT-------------------------------------------------------------------
-----------------------------------N-QGN-EH--NY--AINLT---------------------------------------
----------------------------
>KolobokP-2_SteCin_1p
---------------------------------------------------------------------------------------------
-------------------------------------------MK------------------------------------------------
--------------------------------KCGKSSGC-----K-G-RY---NYP-------G-----------RFRP--------
GVKHKP-KVSVLV-------------------------------------------------ESYDSA-----------DEDAVTD---N-
LRTRLR-----Q-DVVTCAE----------------------DRKSVLK--PSRLR----------PAKI--EG-N---Q-------------
------------------Q-QP-----SA-P---K------------A--------LNGLVLLQSIPELFNNFISIH-NT-ADIK----CQ-
NMLM--EIFDIAYVGLGIILTVQCAN--CKMHLS-------HKM--YKMCERIN------Q-----R-R-G---------PRQPE--------
-------------MNRQVGMALFKT---KMGISDIQFLFSC-MDVHPPCSATLQRVSNLIGETVA-DL------------NAESMVENQVFI-
----RDVINLQKQTYGD---DKKLIL----------------------DVETDVAYNNRPSGG-----GESGTQAFAPIIETVTNK-------
------------------------------HLVLGFSTAQKVCTRG------------------LTCS-------------------------
-------HK----N---C-EVNYDMKKSISSAEAVLAIENIVNVN-KNG------IVKVESITADACNQIDKVADKL----------------
KLNRNTCVVHRMRV--LQRRIQSLD----LSR--HTG------S-NKNERNLYIRKLSRA---------------------------
VRNRIRSELSALKCKRIS------------------------QTE-YVKIAKG-------------IRGNVLNCFS-GNHAV-------
```

CTYSG------------------GCKA--
--------------RK-N---SS--------F--KPV-FLPHG--------------SYLD-LTESDRSKINAVLN-------------
SVLSERLLQQMWRMKTTNHCESLN-------------------HQVFTYAPKMS---------------------------------------
--------------------LY-----------------------------AKNFKN-----------------------LCHSAVHSNT--
HSVG------QSTCLLA-K-----KL-KIKYAKQGCFKHFM----------LGKDRRALYDR-------------------------------
---------------TRQAGKPFLIRRHLRRKIRAY--D---KTLQSSL-------------YLSSSNV------------------------
---S-LSD-EH--NY--
AKNIF---
>KolobokP-4_OwFu_1p

---------------------------------MAKGS-----------R-----KK-------H-----------TFEINN------
KHCYNN-S----------GQAR---TRNTQPNVK---N-
RFFRLDSDTSLR--------------------------------ACGIP--LSSLR----------PEKR--DV--F---FTKLRT-------
------------------S-VN-----SR-T---V------------K--------GNRLVNMEQLMVMINRTIKDH-QT-VSPK----CK-
TPEFCLPANKEEQQGLGYAYAMKCKQ--CRFTSC---------T--FKTYLSIGK-TS-NQ-----V-P-G---------RQKAM--------
-------------CNYQLDTFMLKT---SIPMDDIRLLMTM-LDMPPIGRSTLQRLVDASSKTYS-AL------------NDDQNVKNGELV-
----KRITESS----S------TKEC----------------------VVQLDTAFNNNPK---GRAFSQPGTQSYTPLLHEET---------
------------------------------NLILANKAYNKLCSR-------------HG----LACK-------------------------
-----GKMD----G---CSSANFENQKAIG-NSESMAASVLITEA--SQ------DLTITHATTDGNTSVKKTLTNI----------------
NVQPLECVVHKGRG--LKRKFYSQT----FSQIMIGK------S-TDQSYGYKRAKMSEY--------------------------
LSSRCISELYTARRKFSR------------------------KDFKFFELIEK-------------KRTHLLRCIM-GDHTH-------
CKKTSL-----------------QCNV--
--------------KT------------------ILN-YLPCN--------------QYIQ-ATPADIAKLQACID-------------
YMLNADTIRQMFKTTNTNKTEACH-------------------LRTLKTLPKSK---------------------------------------
--------------------TS-----------------------------SKNVYG-----------------------RFDSAMHSHS--
IGTP------KSIIMAN-E-----KI-GANLTCKKAVIN-L----------EAMHIANGKKL-------------------------------
---------------TLQRKCKTKQRRVSARNAKIGLKR-LATLSVEGA-RL----------GKNGTI-------------------------
---S-DH--NY--V-

>KolobokP-1_Memb_1p

---M---

---RVLR----------PRSTC-KD-K------KLEQ--------------
-----------S-DN-----GN-E---A------------KG------VGNFVVNMDMMLHALQDFQNGH-KT------LETCQ-EWVI--
GMGVTRKKGLTCSISLTCESKGCSF-TK---------V--YPLYKAAQ--E---S-----R-R-G---------PKRAA--------------
-------INKQFGHAVQDS---CLGMHGACQLFAS-LDMSPLAQSNCQKLAKLVDNEMT-DI------------NNRDLSRLAGEK-----
LNDNKLC----GLS-GGREGEI----------------------AISVDSRYNSSRFGN-RKKMGLCATQAITTAIDSCTGS-----------
----------------------------VLDYVIENKVCSKGCR----LR---QHN-NA-AACA-----------------------------
---HERTQHD---C-SATLPKEENI--LEGPMNTKLGVSLL--TR------GLIPVTVTSDNDGQGAKPYQAL-------LS-
ARPEKWTVTAQSDPSHLGNS--QVRAVEKVN----FRKETFSC------A-RVKTIDSLKKLLAKD--------------------------
VKYRSSIIAQQSSKLGLS-----------------------------IIQVNH-------------LVSTAVECFG-GNHQM-------
CSRLVG----------------SSCCG--
--------------TE-E---DN--------WMLSSS-LFRAAGL------------GHIN-CNEEEKKKLLDVFN-------------
IRLHANALQATKLGFSTQRNEAFN-------------------RALSKNMPKNV---------------------------------------
--------------------LF-----------------------------SKNYRG-----------------------RASSAVYRHN--
NGHS-------SVAKKL-A-----DV-GCPLSR--NAIKRL----------QSQQKNIKRAR-------------------------------
---------------ALQISGQYKKMRAMSRMRAAQDWI---KRKIAVESR--------DHDYICSKT-------------------------
----------------------------------TSRVAARL-------------------------------------SRSVEH--DY--
CKGQNDPFQPQ---
>KolobokP-1_BJ_1p

---MA--
---------------------------------SRRGR-------P-S-R-----KTLRHG--RE-----------GFRD--------
GHPYYPPK--RSETTT---D-
KYERLNQQMY-N-MAQDD--------------------------AKCPT--PGRLR----------PTED--TP-S------LLERMAKV---
------------------Q-VR-----TE-N---V------------P--------EARDFYVPYLMDLFNTFIREH-YT-TQPH----CT-
HPVVTMPPTLEKKFGTGYKEALRCNT--CGYHTK---------R--QKLYLEAD--R---Q--GGAT-R-G---------PKPTR--------
-------------KGVQLSMALSKL---PIGITGATTLLSA-TETGTPSQSKLQNITNKLTDLVI-SE------------MNESLADNRAKL-
----RXVCEVQ----GEE-VKDGEAVL--------------------TTAAMDGAYNNPSY----RTYTQNSTQVTVPVYQSLRRKQG-----
------------------------------KTCGGFAFASKLCSCGRD---------GKD-RE------------------------------
------SHD---PS---C-MANYPRADPIGKAEEVLAGRAVEQMQ-EKH------PVLFDKLLSDNDAHIINGVRAA-------TK-
KISQSLEVEKLDCDVHVSRG--QKRKMFRMSE--VLSNQLVAGVSDSSVKVIQKKRTWFATALGRA--------------------------
ISKRCSIELRRARTNHPH------------------------NDDLFKLAVQQ-------------AKDNIISCFA-GNHTK-------
CNELSL-----------------VCQA--
--------------GP-D----H-----------RPT-YLPRC--------------QYIL-PTEDDIKHLQTVID-------------
YKLSKEMVHRQRYVMSTNAVEAAH-------------------RRTQQSLPKNN---------------------------------------
--------------------TF-----------------------------RSSGSM-----------------------RASSAAHSGAV-
GGLG------ESLYRLN-Q-----AA-GSGFMPGGTACRAL----------LGIDRRAAYHR-------------------------------
---------------KRKQSVAYTKRRKALAKRKLM------LSKQHLM-------------YSQATY-------------------------
---------KT--DVEH--EQ--
EMELK---

Supplemental Data S4. Multiple alignment of *KolobokP* HNH nucleases and related homing endonucleases. (fasta format)

```
>AF172595_1
GCHITDYVPA-------------------------ANGYAQVRW----------------CGTKYYCHILAAVMQMQRH------------
PL--------PGEEASHLCGN------------KNCVRPS--HMTFESGELN-KTRSYCTYF
>AAV35433_1
SCYRTNYAVV------------K------------PAGYVQLRL----------------HGRKYYGHVVACIFNAGRV------------
PV--------EGEQASHRCHR------------GACVNPQ--HLVFETERVN-KTRQYCDHF
>MBS0647523_1
SCLGVDLAAA------------K------------EKGYTQVRI----------------KGVKYHVHKLAAMLSTQQA--------------
-------PNGREASHLCHN------------PACVNPD--HLVFEDGEVN-KSRGCCLLY
>KAG9249353_1
WCKVSKLAPN------------K------------DGGYIQISA--------QG------CNKFAILQEVVLWAKGVTVP-------EP----
-------RHEWHISHLCDT------------PSCILPE--HMVLETPSMN-NSRKNCGQV
>AAL69647_1
WCRSSVLKPN------------K------------QDGYIQVSF--------KG------ANKIALLHELVLWADGLQC--------NP--I-
-------RNGQHISHLCAN------------PRCMTLG--HLVVEDAAIN-QSRKGCGKI
>NG_055332_1
GCWMYRGRTI----------N--------------TDGYVQIYV--KRDVTLTGRS----NQVACLLHIASYVLHRDDY--------------
-------DSELQVSHLCDN------------RGCFNPE--HLTLESRQAN-NSRRQCQGV
>KPM34462_1
GCWLSKSSAT-------------------------RDGYCQVTTLKNSQLGRRGQ-----RPVAFLLHKVSFVAHYGRN------------P-
-------TAGNHVSHLCDR------------RNCFNPE--HLIDESPQLN-NGRKNCIGP
>XP_035333862_1
GCHIPIGCRT-------------------------RDGYAQIWT-KSNAKAKKGLTGRK-ASRAYLLHIVAYAQLHKR---------------
-------NPNDHVSHLCDN------------PACFNPT--HLVDETASNN-NSRKGCPGP
>KolobokP_3_MyEd
NCHIWTGGNV----------T--------------ADGYGTFRI------VFRG------KRKRFSVHRLSFFMKVNH---------DL----
-------CQTFHVSHLCHT------------KLCLKFE--HLSYEPKYIN-DSRKICKNN
>KolobokP_2_PeVi
NCFIYRGEV--------------------------MGGYPSLRV------TFRG------RRIRLRVHRLTFYLLNNAE------------
PL-------NSSMHVSHLCHE------------KLCINIE--HLSYEPNAIN-CQRNLCYEE
>KolobokP_2_SteC
DCHMWEGSTD-------------------------KDGYPVLTT------TLQK------RRVRLRVHRAFYYMMNSE---------SF----
-------HSSLEISHLCNT------------KICMNIQ--HLNQETSCTN-SQRKACFQE
>KolobokP_1_CGi
FCHFWLGSLN-------------------------ESGYGVFRP------VINS------KQHKVLVHRFYFFIQGHHI--------------
-------DKNLHVSHLCHC------------KICINTQ--HLSIEPQSVN-NSRKTCKSL
>KolobokP_1_BJ
NCRVWTGYVD-------------------------KDGYGSYVA-------RPEIG----GKYTLQVHRLAYFVNSNWP--------AL----
-------DPHIHVSHRCHR------------KLCVRME--HLSYEPPTIN-AQRNRCRTD
>KolobokP_3_LiLo
NCILWTGYSR---------------------------RYGETRF------KLPWDG----CSKKHRAHRLAYIAYYGG-------------
PL-------PFGLDVSHRCHN------------ALCVNVD--HLSLEARGIN-NQRKTCLAE
>KolobokP_4_BJ
TCLLWTGGKK---------ST--------------SSAYGFINI-----AWFPHSRDF--KRAKIHVHRAAYMAYTGHF--------EL----
--------HGLDVSHLCHN------------GLCVNPA--HLSLETRAVN-ISRKVCKLQ
>KolobokP_3_BL
SCLLWIGGKT---------RTS-------------RVQYGSVNVARMPHRAFPNPE----NRTRLHAHRIAYMAFTGRI--------EI----
--------DGLHVSHLCSV------------PLCVRIE--HLSLEPPHIN-NSRKQCQSR
>KolobokP_1_ParE
TCILWTGCIK------------KAGGGRV------TGNYGVISV------KLKNGDSY--IWRVMRVHRLAYMLHIRS---------EI----
-------PQHLHCSHLCHS------------TLCVNVQ--HLTLETCIVN-NNRRRCATE
>KolobokP_1_PaMi
GCRLWRGCI--------------------------KKSYGIISM------KMPGSN----KRTLKSVHRAAYMAHTGNV--------NL----
-------PREWDVSHLCQN------------SLCVCFE--HLSLEPRAVN-NSRKVCKRL
>KolobokP_7_GiAe
GCVIWTGCQK--------WDKRKD-----------YNKYGVIHA------KIPGRP----KHQTFKVHRLQYMLHHNHF--------DI----
-------PRSMDVSHLCHH------------SLCVNIE--HLSLEPHYVN-NNRQHCLNI
>KolobokP_5_CGi
QCQIFNHNNT-------------------------FSKYIQTTV------LXPGAA----RKTRIYVHRLSYIVHTRNF--------DIF---
-------NRHMHVSHLCHN------------THCINIQ--HLSYEPQHVN-NNRQRCKIR
>KolobokP_4_CGi
QCRIWTGARS-----------S-------------NNKYGVVCY------KHPVKN----KWVTMHVHRLSVMLHHGYH--------EL----
-------DASIVASHLCHN------------RLCVVPE--HITLEPHGIN-NQRQTCRAR
>KolobokP_5_MyCo
KCLIWDQETT------------K------------DGKYGLISF------LDPISG----TWKKKKAHRFSFMVFNNYL-------------
VL------NKTFDASHLCHN------------SLCVNAA--HINLEPHHIN-NNRIYCKHR
>KolobokP_8_CGi_
GCDFFDNSLA-TNCR--RRGNNQ------------GPKYGRVKI------TLPDN-----TRKVFLAHRFMYMLHTNTL--------HI----
-------PHDKQISHICHN------------SLCINPL--HLSLEEAHVN-NERQLCKNL
>KolobokP_2_MeMe
GCHIWQGCVK------------K-G----------AIGYGVIKA------KFPDG-----DWHTMHAHRLRYMISRRIL--------SL----
-------EPGLDVSHLCHE------------PLCLNLE--HLSLEPHXVN-TERQKCVNR
>KolobokP_2_OwFu
```

```
QCLLWEGCLD-------------------------SSGYGRIKV-----KKLDG------RSVSMRVHRVMYMISRDKL--------DL----
-------DSTLDVSHLCHN------------SSCINPD--HLVLEGRGVN-NGRTACVNY
>KolobokP_1_Memb
GCLVWTGGTS-------------------------SRGYGMMRV------EWSDG-----ESRVLGVHRVRMMCSLRTT--------QL----
-------PVGYEASHLCHN------------KLCLSVH--HLSLEPNWVN-MQRRRCSHD
>KolobokP_9_CGi_
GCVLWKGGVG-------------------------RNGYGYIRV------LWPDE-----GSKLERVHRVALMVEMRLT------RSQF--P-
-------GGNLEVSHLCHE------------KLCVNPM--HLSLEPHATN-QERIHCMQQ
>KolobokP_5_MyEd
DCTVWVGALE-------------------------HGVYGKKVM------NWPDG-----TRTLERAHRLSYMLDKRIL------KENL--
PRFSPN---GDLLDISHLCHN------------PKCIKPE--HLILESHSIN-IERIFCRRT
>KolobokP_2_HaRu
GCKEWLGCRD-------------------------KQGYGRCFF------LWPSK-----ARTEERAHRIALCIVHKCLPDGFSNTDNF----
-------GNKVEVSHLCHN------------ASCVNVE--HLKVESHEIN-MERLHCRQQ
>KolobokP_3_BF_2
GCVLWNGRTD-------------------------GDGYGRISV-----QIGP-------KCRDERLPRVAYFLYNGFI--------------
--------DGRDISHLCHQ------------KKCVAEE--HLSLEPHDVN-NERKTCLIG
>KolobokP_3_GarT
KCMFWTGCIS-------------------------KQGYGQFRY------KDPRDLSSA-DHKTRTAHRVALMAKFQNF--------DV----
-------SAKQQASHLCNN------------KICVNAE--HIVFESSSTN-CQRRTCFRL
>KolobokP_4_OwFu
TCKNWIKAKS-------------------------GKGYGQIKV------TYRGW-----GARPVNCHQLIYILHFNT-------------
PL-------ARNEQISHLCHS------------KTCINID--HLCAESPQNN-NLRKVCFMN
>KolobokP_1_LyPi
ICWIYNGRSK------------K------------GSNYKCMDV------TIPGI-----GQTVRAAHRVAYMTSIAGS-------WSL----
-------PPELEVSHLCCQ------------PSCVRPA--HLILESHEVN-LSRRNCNSL
>KolobokP_1_OwFu
GCWVWQGPL--------------------------HGGYGRVSI------SFPLSSNI--PKKTLYAHQVSVIFKLKRF----DFLFEVS---
-------DDSKECSHLCHV------------KHCVNPT--HLVLESHSTN-KARQPCKLL
>I_CpiI
SCWAAKQESG-----------H-------------RNGYIKLNLRNTVGPTGQK------IGVSPWAHQLAAVAAGQGS--------QL--
PLTT-----KGEYHVSHLCHN------------AGCINPD--HVIIEPAWIN-EFRKFCAKA
>OBT43967_1
NCWMSGNAAA-----------H-------------PNDYTKINM---RGTYMPGSNPPRKFPAQPFGHQMGIVAGGYGH----MLLLTF--P-
-------GGDYEVSHLCHP------------TACFNPD--HLIVETKVQN-KMRNNCHGS
>BAB87243_1
NCWKPTTKPT-----------H-------------RNGYCQINL---RHTLLDASDKK--IGRCIYLHHLSLVAANKAH--------EL--
TRLNAG---KKRLQVSHLCHR------------GWCFNPE--HLVVESQRLN-AKRNNCKGN
>AAP73783_1
ACWTSPDKQP-----------SEP-----------GKPYRKLNW---RNTNHPNRPSEK-IGHVFYAHHIGAVAAGYGN------HLSLI---
-SGA---HAAYNVSHLCGN------------GECFNPD--HLVIETAGLN-QRRKTCQGH
>I_PchI
GCWESNLAGR-------------P-----------NSGYQRVNL---RNTVINGRK----AEVQIYLHQLTLIADNRRG--------
ELMAAVLES----ELAMECSHLCHX------------GRCFRPS--HLVVEPRAEN-KERQTCNGH
>CAC24704_1
GCWTLRKIRR-------TKTVKK-G----------TMTYPPGFK------TGKKLQYKI-EGRYVVAHRVWFYATEESR--------E----
VLG-----RPNATVSHRCHN------------EHCLNPD--HLVVEPLQVN-QSRNTCTGV
>I_PpoI
HCYEIPLAAP----YGVGFAKNGPT----------RWQYKRTIN--------Q-------VVHRWGSHTVPFLLEPDNI--------N-----
-------GKTCTASHLCHN------------TRCHNPL--HLCWESLDDN-KGRNWCPGP
>CAG8949560_1
DCWLHPSPPPARLKNGRLFARHI-ISKRFTFTC--PSPYAPYEL-QTHSLTIPYG-----IAHHLVYHSLTLQQQDGWI--------------
-------NQNWQNSHLCGN------------WTCLNPE--HLTVESRGIN-VSRNNCFSH
>I_NaaI
ECLIGAANKT-KSGFKVRFMNDK-G----------SDSYVHHVS------VFAN------STCENCIHSRKMLETVSSS------KKD-----
-------PDARTISHLCGN------------GGCARPG--HLRIEKKTVN-DERTHCHFL
>ABD62811_1
ECLLGASNKS-KSGFKCSFLSDK-G----------SDGYVHHVS------ILAN------SRSSDPIHKLALIKKVSQR------KKE-----
-------KNAYSVSHLCGN------------GGCARPG--HIIIEPKTVN-DERVACHRF
>I_DirI
MCLMSPRPHH-----QMTIQTSK-GR---------AGRFKIQAS------ALQAV-----LVNNPSSHDELVEQVKGLI------DRE-----
-------TTTFHSSHLCKG-----------DGSCMELK--HTLRVPAQTNLADHELCPAF
>MBK4161067_1
NCWEWSGTKY-------------------------PTGYTQLTS----------------GRKQVLGHRWAYEHYRGEI--------------
-------PSGMVIDHLCRN------------RGCVNPA--HMEVVTNEEN-LRRGAGYAI
>NUK07250_1
GCWDWNGHVK-------------------------PNGYGQFKV----------------EGRPQYAHRVAYTLTRGTI--------------
-------PEGLVIDHLCRR------------RHCVNPD--HLEVVSHRTN-ILRGVSPAA
>WP_138708849_1
GCWIWTGIVI-------------------------NSGYPRLGI------TRQN------KTKQYLAHRLSYQLFVGPI--------------
-------PEGMQIDHLCMV------------RRCVNPQ--HLEAVTPQIN-VLRSPNTIA
>YP_009301978_1
DCWLWLGYVR-------------------------SDGYGQCYT----------------RDGNRLAHRVAYMLDGNRL--------------
-------DPELTLDHLCRR------------RACVNPQ--HLEQVTAEVN-TARGEAGVA
>QBP30472_1
ECWPWTAYVD------------K------------SKGYGQFGV--------GGR-----DGGLESAHRMAYRLAVGPI--------------
-------GEGMHIDHTCHNGSGCPGGKECPHRRCCNPA--HLEQVTQEVN-KARGEAGAY
```

```
>WP_181272794_1
GCWEWGGSSW-------------------------GDGYGQMRV----------------NGKATLVHRLSFEWNSGPI--------------
-------PPGMVVDHMCWN------------RSCVRPD--HLRLATPSQN-QRSQSGPNR
>SEC89835_1
ACWEWNACIT-------------------------PDGYGQFGL----------------GGRVLLAHRVSYEFEHGAV--------------
-------PPGLFVDHICHN------------RACVRPS--HLRLVTQKQN-QENHAGAHR
>SKT75437_1
GCWLWSAGRD-------------------------SGGYGNFYL----------------NGREAKAHRVSYELSVGPI--------------
-------PPGMLIDHRCFN------------PSCVNPG--HLRVVTRKQN-NENLQGPRR
>MBG0586989_1
ECQVWTGAKL-------------------------KTGYGSIRT----------------GGKALRVHRFVWESVNGPV--------------
-------PDGMDVDHICRN------------RLCCNIN--HLRLASRSEN-NQNLGGAKK
>DAL20869_1
GCWNWLAYIN-------------------------PDGYGMFKH----------------KGMMTLAHKASYEILVKNV--------------
-------PDGFELDHLCHN------------RKCVNPK--HLRVVTHTVN-VWNRIKPVS
>WP_090685763_1
GCWIWMGAVN-------------------------AQGYGFMKA----------------NGRQGFAHRWSFEAHNGEI--------------
-------PKGMVICHRCDV------------PACVNPN--HLFLGTYLDN-AKDCVKKNR
>WP_112116992_1
ECWGWEGYLM-------------------------PNGYGQLKV----------------KERNVYAHRFSFKLHFGFL--------------
-------PDHLLVCHKCDN------------RNCVNPN--HLFLGTHKEN-TRDMDLKGR
>MQU93811_1
GCWEWTGPDS--------GKNGR------------GKGYPRMSL----------------DGQTVAVHIAMWTNEHGYI--------------
-------PGKKELDHACRN------------RLCVRPEKDHVEMVTRKEN-AKRREQAKR
>I_HmuI
YSIKSGKTLK-----------HQ-IP---------KDGYHRIGL-------FKGG-----KGKTFQVHRLVAIHFCEGY--------------
-------EEGLVVDHKDGN------------KDNNLST--NLRWVTQKIN-VENQMSRGT
>I_BasI
ASKRTGVIMA----------QYK-I----------NSGYLCIKF------TVNK------KRTSHLVHRLVAREFCEGY--------------
-------SPELDVNHKDTD------------RMNNNYD--NLEWLTRADN-LKDVRERGK
>NP_046715_1
ISKKLKKPRK-------TFIT--------------PHGYEMIGY------THPKK-----GTQNYLVHRLVAKYFIYDI--------------
-------PKGMFVNHIDGN------------KLNNHVR--NLEIVTPKEN-TLHAMKIGL
```

Supplemental Data S5. Multiple alignment of *Kolobok* DDD/E transposases. (fasta format)

```
>KolobokP_1_PaMi_1p
AIAVETDRQYNNPLRNCRKKTPFVAATQTRDVVVENVTTSK-----YVVMYHQENKLCKK--------------------C--DIDGARDH--
---------------KH-AGKCTA-------TRPPN-FNMGDE-RNGGIQCAQKLL-----NCKEPL-----------------------
FVNRVTTDADGCLARGMRSYMQSKVA---------GMETEH------------------SLDPPHLNR--------------
SLCAAISRAKFSKEMF--------PG-KNKRAKKNI----QDRFADDVSHRA---QAEAAAILKKSGGN-------
SSQMTTMARNAASAIVQCYMGN--------HGL-----------CK----------KWSQI-------CMG----------------------
-----------GKYRFPYLPVDAR---------------------------------------GKLKL-ATCDEQT-----LIKLLGK--
RLGQEALEKTKYGTSTQKAESMNH
>KolobokP_2_BF_1p
GIPIEADSRYQSPLRSGRGKKPGQPSGTCVTNVAENVTKRK-----WVICTHVKNKNCQQ--------------------CSRGEGKKEV---
---P-----------SH---KCSA-------NIPQS-AVIGDE-RQAAIEIAKKLM---SGPSKT-------------------------
IVAELTEDGDSSFSSGMKEVMEEAG----------LNDLKV------------------FKDIVHLSK--------------
AIRRKVSGGKWSKQMF--------PG-KTQEERKWI----RDRFGNDLAIRL---NTEHILGLKKFRN--------
QRVMAGKMKQVMQAIPYCYSGD--------HSR-----------CK----------SGSLV-------CRH----------------------
----------PYKWKFQELQEKAK---------------------------------------GKLDP-TDADLKE-----LGKIMEH--
RLGKEALKVTRRGRTTNKVESVNR
>KolobokP_7_GiAe_1p
KISVEGDCRYNNRLFGGNSSTPLQPATQAVYTIAENETPKK-----EIIGIYTANKLCKKAEIL-------RSKGEK-ITC------------
---P-----------DH-GGKCTA-------NLKTT-DSIGNE-GLYAEKCVKEFQ-----VDEDKL-----------------------
NIGYFTTDGDSHATDGATRAQTG--------------SIEN------------------LRDTRHFSK--------------
SQQKAIEKVSFSLTMF--------PG-ENKSDRQKV----QRRFAIDIKQRC---HLELDIAHRQLAGD-------
ITKIVRKMTYTIDSIVACVSGN--------CSTL----------CQ----------RHSFV-------CSG----------------------
--------RRIKAWKPAYLPADSV-----------------------------------------LKM-KEGDKEL-----LRKCLEL--
RLGKTALLKTKLNTNTQKVESVNR
>KolobokP_4_MyEd_1p
SVSLQADGAYNNAIYSGIGKTPFQPATQVVYSVAEAETEDK-----SIIGVVCKNKLCSIHPI---------KSGEK---C--------T---
----------------S---SCSS-------NLTFM-KSIGDE-YTWAKEALQDLA-------SDGI-----------------------
EAKHLTTDPDSSAYRAADDLYLENTT---------STEPEH------------------FLDTRHFLN--------------
NHRKNIKNNKELGEIM--------PG-RTKKDREKL----LNNFALDLAERC---QSEFTQAMEKYGGD-------
FTKVKNKISFTVDAIPACYTGN--------HEL-----------CR----------RHSFV-------CKG----------------------
-------GKKFWLSNRAFLPNSFK-----------------------------------------IRK-LDENLNA-----IRKCVLY--
RLSPSALKKTRLNLNTQKVEGFNR
>KolobokP_1_PoSt_1p
QINVAFDARYNAIT-FGHEKKPGQSSSQAVGIACETLTEKQ-----YIVGTAVENKLCWTGAWL-------KGKGYD-VQC------------
---PG----------GH--VDCTA-------NLSP--VAPHSE-LEMAREIGNKFV-------LQEI-----------------------
LIRHATTDGDAQAVAGFQAACNILHP---------MWKVER------------------QSDPIHLGR--------------
LQFKRCNSAVFSEDMF--------PGIRTREGKKLK----QKIFSQDIKARC---SLIFKQLMEDNSGD-------
VTKIKCQLPQVLEATLRCYSGD--------CSK-----------CR----------HHSKV-------CGG----------------------
------GSTNSWWMRSAYLGPHQV---------------------------------------THLNM-TEDDKNI-----LLEVLKM--
KLSCDAIERLRLNTNTQKCEAVNR
>KolobokP_2_OwFu_1p
KIAAAFDAQYGSVK-FGSRNKLGPDANRAIGLTVETMTENL-----VIIDAVLQNKLCWRGSYM-------RARGFD-VKC------------
---PG----------GH--EGCTA-------TIEK--AASFKE-RQMGRSTAKTCQ-------EDGI-----------------------
VIQYLTTDGDGKGIMGVNDHYAETGM---------EHRAER------------------LSDRIHLSN--------------
LQFKHSLKTSFSSQMF--------PG-KNKSTKEHY----QRLFCIDLTQRC---TAILRKLSLTDRD-------
LSKVSTFKIIKITDSLIQCYQGN--------HTL-----------CK----------QDLNE-------CKG----------------
RRSNN-------WWTNSYNLKACNLK------------------------------------------ATDINMDKKNDEMY-----
LRLLIEM--RLGPEALASGKFYTHTNKNESANH
>KolobokP_1_OwFu_1p
AVDISIDAMYNSNQ-IASRNKYGQSATQAYTTVREHNTKRN-----FIIGAHVANKLCWVGAYL-------RGKGYENVKC------------
---P-----------GH--SGCTA-------NYDR--ATPLSE-RHLAYMIGRNLS-------HEGL-----------------------
NPNLVISDGDSQSINGLNDAIPE-------------NKMIA------------------QKDPIHSAQ--------------
CQFRAVQRAEFSNDMFGLN----TPNISVGE----I----KKVLGSDIKMRC---NGAITMIRKQFDQN-
LTFDENINLISGKIASSASAIVKCYTGD--------CSS-----------CG--------QQGVYSQ-------CDG----------------
-GKNT-------WWNKSYHFKNVNPPII----------------------------------------KGSFRP-DKIDIEI-----
LRDIIDI--LMSRASFELMKSGKSTNSNEAANR
>KolobokP_2_LiLo_1p
GINVQADGRYNSLH-LTSRNKMGRCASTMTGLCVENVTDEH-----KIIDIEVKTKLCWIGAAM-------RAKGFE-VKC------
GATGTN--VDE----------RH--SKCTA-------NFPE--ADAFRE-YDLGVAFAETFE-------KQGL--------------------
---HVEHLTTDGDSSSFKGLTDATKKLFG--------GLFTVKH------------------QLDSIHLHQ--------------
AQTRAGHRAEFSKGMF--------PG-RTAEERTLL----KTTFLNDLRVRS---SKILQSLFKRCNGD-------
LDRIMGYLYDTVACVVNCYGGD--------CTG-----------CP---------QNEFSL-------CSG----------------------
-------TTGSWWHKSTRWSSYGE--------------------------------------IPKFKM-TVIDKNL-----VHLVLEV--
CLSKGALQQLSLGVTTQGVESKNR
>KolobokP_1_CGi_1p
EVDLETDTSYNNRP-----QAGFEAGTQSFSPLVENNTPRK-----LVLVLQTANKLCSK------------------RSC------------
---------------DH--RNCKK-------NYESDETIASSE-ATLVRRNLDSLR-------EENIL----------------------
NVRSVTSDSSAQLDKTLRDYSAATG-----------TKIIQ------------------YKCFIHKLR--------------
NLQKNIRSIKLKSPLP--------PG----SNRDAF----IQRLATCVRARV---RYELVHIRKLCKS--------
DELFIHKAEQAISNILPCFSGV--------HDH-----------CR----------RMSFT-------CNA----------------------
---------HLPTYSAKFLPYGRHLD-----------------------------------------L-EPEDISS-----IQRVINK--
QILGQNLPKICRLSTTNKCESLHQ
>KolobokP_1_GiAe_1p
LIPVEADCSYNNRC-----QSGYEAGTQAFCPLVEQLTKRK-----LVLGLSTANKLCRK------------------HQC------------
---------------SH--TKCAR-------TYNPTHSMASVE-KKLISENITNVE-------SQNVV----------------------
```

KISSLTTDACAQLGNIMQTSNP-------------IHCSSHSNMF-----------PLKYVCFVHRMR--------------
RVFINTKVAALKLQYKP------I-G-LTKD---AF----ANKLAVGMRCRL---TRELSQLHKQCHG--------
VKAFVSKGASIRDNIVNCFCGE--------HTH-----------CK----------TASLV-------CNA----------------------
--------KSSKYFKPVFLPNGQYLQ---L-NNTDRHS-----LMHTLNK--
TVSNIMLSQLVCLYNTNKSESLHH
>KolobokP_4_OwFu_1p
ECVVQLDTAFNNNP---KGRAFSQPGTQSYTPLLHEET-NL------ILANKAYNKLCSRHG----------------LAC---------
KGK---------------MD---GCSS------ANFENQKAIGNSE-SMAASVLITEAS--------QDL-----------------------
TITHATTDGNTSVKKTLTNI-----------------NVQP------------------LECVVHKGR--------------
GLKRKFYSQTFSQIMIGK---------STDQSYGYK----RAKMSEYLSSRC---ISELYTARRKFSRK-------
DFKFFELIEKKRTHLLRCIMGD--------HTH-----------CK----------KTSLQ-------CNV----------------------
------------KTILNYLPCNQYIQ---A-TPADIAK-----LQACIDY--
MLNADTIRQMFKTTNTNKTEACHL
>KolobokP_3_BF_1p
SVAVEMDVGYNNPP---KGKSRGQPGTQAECPMFDGVTQKK-----MLLALSTANQHCV--------------------DC----
DKLEEQGLPFGPG----------SH--EGCTR-------TFEKHIKMGSSE-RKMAKENTKTAL-------KNGI------------------
-----IPGIVETDNDSKISGGTNEALHEAG----------LPPAEK------------------QDCMQHMSR--------------
NHRSKAYNLCLEVFEGGN---------NTKVTKQEA----SRRVGLYIVDRC---VRALYTARAHHPDS-------
DEDFFREVERGRPTIVSCVSGE--------HSE-----------CT------------SDFG------CPE----------------------
-------YTREGRRPAPDIPG---GGNLAL-SDNDRRT-----LQKLVDY--
RLAEESVKRQRTFMNTNKSEAFHN
>KolobokE_3_LHu_1p
YITVVADGSWMKRS-----YGNAYDSLSGVGAIIGYRTRK-------VLFIGIRNKFCT--------------------VC----
DMAERNGF--EPR----------RH---KCYK-----NFDRKA--SSTKME-SDAIVEGFKSSL------EMHGL------------------
-----MYRTVIADGDSSVYQSIQNNAPYCEQ---------MVTVKK------------------IECTNHLLR--------------
NFCKKLKIVAETVQSKQHRT-RGFVQ--------------LRNVVKNNILKM---RKEMLEAATLRREEKV----
PHHSKATELQKDILNIPSHIFGE--------HKR-----------CK----------ERGRI-------CEE---------------------
-----------NHDKKQNYVPYL--K------AHGLYQK-----IESAVMY--
LSAY--SDSLLLNLTNNPAEWFNS
>KolobokE_1_TC_1p
LVPVVADGTWGTRS-----NKSNFNSLTGAAAIVGAHTGK-------VLYLGVRNKFCM--------------------IC----SRQKERS-
---PT----------DH---VCTK-------NHIG--SSGSME-SQIILQGFKTSV------EMYNI-----------------------
KYNTLIGDGDSSTYKKIIEGRPYNN-----------LTVEK------------------IECRNHLLR--------------
NLRGKLKSLVGDRKFPLQ----------------------NRKLLEGKIMRL---STGIRAAITYRKKEA-----
DKVSAAGRLRQDILNCAQHVFGE--------HKD-----------CA------------NYF-------CQR---------------------
--------------KTDTNTWDTI--STELKGQ-----IQNILRT--
LASH--SRSLLCDVDNNVVEGFNS
>KolobokE_1_SpEx_1p
LIDVIADGCWSKRS-----YKSNYAALSGAAAIVGRRFGQ-------ILFMSVKNKYCC--------------------IC----
ARSEKRNT--TPR----------AH---QCFK-------NYSG--TSTAME-SAILVEGFKQSV------EMHNL------------------
-----IYARFIADGDSSTYAKILESRPYPD-----------VTVEK------------------INCRNHILR--------------
NFCNKLQQIKTDTKFNLND---------------------RKKVTTAKILTA---RKYITDSITYHNKN------
RELDGIKSLHVDINQSMNHAFGK--------HGE-----------CN------------KNI-------CSR---------------------
-----------ENVSQECADFF--NSYIFNK-----LRFITTN--
VSSH--ARSLIENVDSNVVERFNG
>KolobokE_2_TC_1p
YITVVVDGAWSKRS-----YKSNYNASSGVATIVGARTKK-------ILYVGVKNKNCK--------------------KC---
MYYKKKNQT--TP-----------PH---KCFK-------NWKN--TSTSME-AAIILDGFKKSV------DMHNV-----------------
------RFKNLIGDGDSSVYKKIRNARPYGPN----------YFIKK------------------IECRNHILR--------------
NFCTKIREIAKTPRSDIN----------------------IKTFLRNNYLKF---RTAIVSAIKYRKNENC----
TFDQKAENLKNDILNGPAHIFGN--------HSK-----------CA------------TYF-------CKT---------------------
-----------VGQITNAVAYSDFK---NSSLFEL-----FMQALKR--
VANL--SSSLLYDVDNNSVESFNS
>KolobokE_1_LGig_1p
AITVVCDGGWSKRS-----HKHSYNALGGVGIIIGQVTRK-------LLHIGIRNKYCY--------------------TCSLAESRQKD---
---PG----------QH---ECFK-------NWDE--SSQAME-ADVIVEGFMNAE------KDHGV-----------------------
RYMRLVADGDSSVYARIQKTVPIWG-----------PHVTK------------------LECANHACK--------------
CLRSSLEKLVDRLPKLKG----------------------RGKLTLATRIRI---VSAVRCAIRMRSSEE-----
NRSLACKKLVHDIKNCIYHVFGD--------HTR-----------CP-------------EF-------CKK---------------------
---------SSGEIPSTESSPTSTCEDEQSESVISV-VGDQQDFWTDGSSLKDQEDSRLAVPGRGLSAA-DKEIINE-----VCQLLNR--
IASK--AERLLGNFTTNLAEAWMN
>Kolobok_1_KlNi_1p
AIAATFDGGWPKRG----TRSGGYNSLGGMAALMSALTGK-------VLQVEVLNTRCG--------------------IC----
DQAEDLKT--EPP----------DH---RCHR-------NYAD--SAKSME-PEGGVRMLERIT-------KFGC------------------
-----VVRDLIGDADSSVMAAVRERLPPWI----------ANVVEK------------------ALDIGHLKG--------------
NLYDKMTKLKASQFKKD-----------------------RKAFTDGQIRAV---SSYFAAAILSNRG-----------
DRAAVKQALESIVPHLFNE--------HDK-----------CK----KKRHPEDEEGW-------CKA-------------------------
---------GLEPDHVPNRLK-------------------------------------NHGGXFT-GDNFRKA-----LEDLIGH--
YVQPAMLDQIYKGYSSNQSESLHN
>Kolobok_1_AdSt_1p
NIKVSVDGTWLTRR--------GHSSLHGIATVCSTSDPPK------VLDFECLSRHCT--------------------TC-SGLLGIREHN-
---PE--MYEQLVE-EHIQDGCEA-------NHKG--SSGGME-AAGIVEVFRRSE------SKHLL-----------------------
RYTTYIGDGDANNERALRDAQPYXD-----------ITIKR------------------
LQCINHFSKRNIVIIMIIHLFFYRMRTALETLKKXYKGX-----------KLEDDKP--IGGRSGRLTDDKIHQL---TVYYGSAIRSHVN--
---------DLESMKAACWGMFHHYNST---RENPNHDY-----------CD------------PAK-------CHI----------------
-------------------YRYKSFDTSEH--TM-APDVMKA-----
IRPVYEK--MCSDDTLSKVVDGGTTNPNESYHS

>Kolobok_24_HMa_1p
NVTVSVDGTWQRRG---------FSSLNGVVTAISN--GK-------CVDTQTLIKDCK--------------------SC-QYWQRNKDI--
---PG---YNDWVE-SH---ICPI-------NHKG--SAGAME-AIGALQIFKRST------VFNKL----------------------
RYTKYCGDGDSKSYQNIESNNVYPG-----------YKIEK------------------SECVSHVQK--------------
RVGSRLRSLKVLYKGK-----------VLKDGKR--LTG-KGRMTDKVINTL---QNYYGMSIRQNKG-----------
NLYGMKKSIAGLIHHCSES--SSDEERHKY-----------CP---------RTSDSW-------CKY------------------------
------QRDKINQT-LTYKNSIN--I-PEAVCEI-----
IKPIFSHKDLGANKLLKRCLDGETQNANESLNN
>Kolobok_3_DeSi_1p
NIAVCYDGTWLTRG---------HSSHIGVGTVVELFTGY-------VLDYHVMSNFCL--------------------GC--------
ENGP--KPDSEGYDLWKL-SH---QCQK-------NTSC--KSGQME-VEAGKILFERSL------QRHNL----------------------
-RYTTMLCDGDSRTYNAIREAKVYGY-----------IEVEK------------------EDCVNHVRK--------------
RMGTALRNLLQKHKGEGKRS----------------LGG-KGRLTAELVDRL---AIYYGRALKSHVG-----------
DVEAMSRAVMATFYHVTST---DSCPNHAL-----------CP---------AGEQSW-------CPH------------------------
------NAAKAKGEPEPRHKYN---L-PSDVAAA-----LLPVYQR--
LSEKSLLQRCLRGRTQNSNESLHS
>Kolobok_9_CGi_1p
GIVVSFDGAWQKRG-----TGRAYNSLTGHASLIGNKTGK-------CVGYALKSKKCR--------------------IC----
SAAKEKNV--TPR----------KH---NCKK-------NWKG--SAKSME-PAMACEMLQSVL-------DQGE------------------
-----KVSTLVMDNDSTTIARVKSTVD--------------QNITK------------------RCDSNHTRK--------------
GFTASLIELSKAHKLLR---------------------------NTKVRSHI---ERCFTYSISQNKG-----------
QPEQLADALSSIVPHLYGE--------HSS-----------CG------------SW-------CRG------------------------
------NEEGYKHKALPYGKPLD--DRDLREA-----LQSLMEK--FSSK--
SSELAYMGSTQPNESFNY
>Kolobok_7_CGi_1p
KFSVSFDAGWQTRG-----SGRNYASLSGHASMIGEKTGK-------ILSYAVRCKKCR--------------------FC----DKAPVSA-
--EVN----------KH---DCRR-------NWEK--SSKAME-PDMALEMLHDLK-------ARDF-----------------------
HVKHLIMDNDSTTLAKAKMSFD--------------PNIQK------------------ISDFNHTKK--------------
NLASKLYDIKKEKK--------------------------YPLLGPKSIQHL---LKCFSYAVKSNE-----------
DTDTLKRNLDSIPLHVFGN--------HKK-----------CE------------KKW-------CKY------------------------
----LQDPENYKPINLPYGKYLS--GVDLLKD-----LQHLFSD--LAKN--
ASKLSNIGSTQANENFNK
>Kolobok_1_CGi_1p
SISASFDGGWQKRG-----SGKSYSSLSGHAAFIGHKTGK-------CIAFSTRNKYCR--------------------KC----
DSALKKGC--DVK----------EH---DCRK-------NWEG--SSKAME-SDMCISMLKNLE-------SNDV------------------
-----CVGTIIMDNDSTTISKARSEVK--------------ADLKK------------------QCDRNHLLK--------------
DFTNKLYDIRKAKN--------------------------FRELTPKTISHI---SKCFRYCVSQNQE-----------
DTEKMKKNLLALGKHVFGD--------HSY-----------CG------------SW-------CGY------------------------
----LQNPLKYKPKHLPYSRYLC--DEKLKIT-----LMDLLHK--YSSD--
ADKLTHLGSSQANESLNN
>Kolobok_1_ADi_1p
GIAVSYDMGWQKRG-------RGHNSSTGHGAAMGLTTGK-------VVSYSTRCKTCR--------------------VC----
SHNKVTGK--EK-----------KH---DCRK-------NHSG--SSKSME-RDVACELWSKAP-------QSGV------------------
-----KFSIYVGDDDSTTLADIKNKVP--------------YGVEK------------------WSDIVHTKR--------------
SLNSRLYKLRERFKGPN-----------------------CSVLSPKVINYL---TKCFSYCINQNVG-----------
DSNSLKKGLKNIVPHAFGD--------HSC-----------CD------------NAW-------CGY------------------------
----KQNPAAYKHTELPYGKDLF--GDSLKKA-----LTDILDE--
YSTDIVVNKLAPCANSQRNESLNS
>Kolobok_1_MicRcc299_1p
AIVVSADGSWAIKG---------YTSNCGQASLIFSCEDFDR----LVVAQDFRQKTCS--------------------TC----DWYGKNK-
---PS------WVVPPH---VCRK-------NYTD--TSKSME-QDILISLVDDVA----IYEHKTVDGTMEEKPMEERL----------
VIEAVCADEDSSFWNRITQDGALKNQ----------FAPRK------------------LSDVNHLSN--------------
CLMRRLAGQKAKKMKN------------------------TSLLSNAVCQKF---CTSFRWIVKQNTG-----------
DHQRAKAQLENMICHYFDD--------HTN-----------CGDFPKLNEDGTPKEQW-------CRA------------QVAAENE------
---AEEGDDHAGPTNFPRNKYLDKIVTVKRGKYEETIVDGATKKT------------------LV-GAEDYEINYFDDVKEVFKV--
FLTDQVIKAATSGYSSNVNESLHS
>Kolobok_1_FraCyl_2p
NIGVSMDAGWRKRS-----SGRTYDAQQAQFFAFGVETNK-------ILYFEQMSTRCR--------------------KC--------
EHKL---------------EHDPKLCSH-------NYTG--SAKGME-PHAALKCIQSIF------SKGDA----------------------
-FVGTIVTDDDSSMRARLKQNGREKIEAGLCRKEDLTPTIQLSNKNDHGALELNIPEPICKADANHRVR--------------
GFGNAVQKLVNMRNGE------------------------SGGVTGVDRDRL---KVNFAYARAKNVDK----------
DFNLFCAAFAPVIEHHFNN--------HEL-----------CG------------EW-------CAA------------------------
------KKLTDTKKSADHLHYRCK---------------------------------------VK-NKLMYDN-----LKEIHKR--
YTTEQQLREIHHKVNTNLSESANF
>Kolobok_1_LHu_1p
ELTVSGDGTWQKRG---------YSSLYGVFSVIGYYSGK-------VIDIIVKSRYCK--------------------SC----
EFWKSKTD--TQEF---EEWFE-TH-KESCSS-------NHEG--SSGKME-VDGILDIFKRSI------KQYAV------------------
-----KFINYIGDGDSKTYKGLVDGNPYEG-----------ITINK------------------KECIGHVQK--------------
RMGTRLREAKKKNKGLSG----------------------KGKLTGKVIDKL---TVYYGLSIRRHCN-----------
SVNDMKNAIWATFYHYSST---EAKPNHSK-----------CP---------PGPESW-------CEY----------------KRAE-----
---ANNNLKNYVQDYEP---L-PADVLKA-----IKPIYED--
LSKDELLERCLGGFTQNNNESFNQ
>Kolobok_3_Aplcal_1p
ATTISFDGTWHKRG---------HSSHFGVGVVIDCKTGF-------VLDYQVLSNYCH--------------------GC----
EVGLKSGD--EQ----YLLWKN-KH-QLKCQQ-------NFQG--SAKAME-AEAAVTIFRRSV------QHRGL------------------
-----VYSRMLCDGDARSHQLINTKGIYD-----------FEVIK------------------EDCINHISK--------------
RMFNALENTKNSNKKEL-----------------------NRKLTKTKIEKI---TNTYATNLKQNAP-----------

DTEQMQSDVYGGIYHMLST---DDNPQHHL-----------CP---------TGISSW-------CHF------------------------
-------QRALATKEEPRKHTPT---------------------------------------I-TEDVAKF-----VWPVVER--
LTRPDVLKRCASMQTQNANECFNS
>Kolobok_1_OwFu_1p
GITIMTDARHGCRK----------NAKDCDIVCIGEKTHQ-------VLHSIHVTKRD----------------------------------
--------------------------------DP--CSQRHE-TFGTRRMYDDFE-------RQNV-----------------------
SIGTHVHDCNGAVNCLVRDTQI---------------FTDN------------------QNDSWHACI-------------
ALKKQIQNVASGPKYKH--------G---------------QSWHAQLEDKP---EAIRKFAYTAMKNCGE--------
NAETLRYMLANMTEHFQNN--------HQN-----------CF----D-------QSR-------CRL------------------------
-----------DPNYEPSTI--VVRNDVAIRL-----LNDTIKK--
STIYKNAHNYTKALSTAYVESFNN
>Kolobok_16_CGi_2p
GIDIITDARHGWRK----------NAQDTSVVAIGERSHK-------VLNCAHVTKAE----------------------------------
--------------------------------DP--VSQRHE-TIGTRKIYEDFD-------NRDI-----------------------
SVNVHTHDRNMAINKYVREREH--------------GFTCN------------------QNDLWHGIK-------------
SIKKALTNISSGPRYKE--------G---------------KTWSEELVDKV---EPIATHFYWAAKHCEG--------
NSTTLRTLLDNIVDHYQND--------HKR-----------CH-----------PSSR-------CKQ------------------------
-----------DPNYEPSKV--VLENPVAIKL-----LKSVIVN--
STIYKYPDHFNLGRDTYFVESFNN
>Kolobok_1_MyEd_1p
GIDIVTDARHGTRK----------NSMYTDVVCLGARTHK-------VLRVETISKVD----------------------------------
--------------------------------CT--SAQKHE-LIGTERIYEYFK---NLRDEYEV-----------------------
KIRVHCHDRNTSVNKFIRINGI---------------DTES------------------TNDTWHATK-------------
NIAKEIKTICSGPRYKE--------G---------------QTWHPELSDKA---ASIKTHLYWAMKNCNK--------
DPVKLKLSLLNIVEHYKNN--------HEH-----------CS----E-------LSR-------CKT------------------------
-----------DSNYEPTKY--LIKDPKAEML-----LGRALMN--
TQVYKSPTDYVHCMDSYYVESFNN
>Kolobok_1_NaeGru_1p
FLDISVDARYSSRR----------NVYECTLVVFETKAKK-------TIERSHVIKKRASNRKSALQ-------------------------
------------------------------WFMG--ASKLME-PEACRLAITSLKHKSFPVMGYGILHF--------------------
KIGSFVHDKDSTVAAVIKKLEP---------------TALE------------------KLDPNHVIK-------------NLSKEVEEK--
-----------------------------APRIASIIVSSF---RKALKLANTTTNS------------NEKLQALLKAYPQHLQNN------
--HSL-----------CD------------SG-------CPH--------------------------------------TIKPEK------
-------------------------------------LI-TKEEAQV-----VKEIFDK--RIKYS-HKFCDIACNSQNNESIHS
>Kolobok_2_NaeGru_1p
PLDISVDARYSSRR----------NAHECTLIVFETKFKK-------IIERSHVIKKRTLKKRSIAL-------------------------
---------KGCLD-----------------IFVG--ASKLME-PEACKMAITSLQHKSFPLTGHGLLHF--------------------
KINSYVHDKDSTVSTIIKKLEP---------------TAEE------------------KLDPNHVVR-------------NVRKEAKER--
-----------------------------IPRVAVIVVESF---RKCIKMTSSLSDSE----------AEKKLKKLLTAYPYHLQND------
--HSK-----------CD------------EG-------CPG------------------------------------TTKSQK------
-------------------------------------LI-TKEEAII-----VQEIFDK--RIKYC-HKFCNSEYNSQTNESLHS
>KolobokP_1_TeGr_1p
TVDVETDVSYNNRL-----ALGFQSGTQAFCPLVENTTNK----K--LVLNCQIANKLN---------------------------------
----------SCTQ-----------------NVAPNTVLTHE-KTMTIRNVEEVN-------ANNFV----------------------
TVSSITTDGCPQMDSVASAIGVACG----------KPPLRH------------------FTCFVHKMR--------------
RVYKNITSLPLSL-----------PG-YTTERRATF----KKKLAIATRNRL---TRELTRLKNNVSV--------
ERQFVEQAVRIRENVLDCFSGN--------HSQ-----------CK----------KISLI-------CKP----------------------
--------HLPPIHLPFGRWLNL---CDSDKDRYTC-TLRKILNN--KK-
---LSEMTRLFNTNRSESIHH
>KolobokH_3_DiVe_1p
TLPVSFDCSWSHVR---NAQQASGEIIYDGTDI-KGYLYK-P-----IIAFYTVEKPRKINK--------------------GNEKE-IV---
--------------------YREG-------NFDA--SSRQME-HAILIGVIGKLT---------PILEKYD------------------
MLLNITIDGDLDSNKTLGNVAV---------------VNQI------------------FADLKHVTK-------------NIRKNLLNK-
YPQ---------------------------WREFEQHIMQYF---TSCVYAAGILKSDS----ENCHIQEKDIRHIQTEGLFQHLCNN-----
---HEI-----------CW------------PEV-------CWI------------------------------KNNPEIQLSEPTLR----
--------------------------------------SY-APQQREK-----FKSMLET--IFRLPINQGIGTKTRTSQNEAFNR
>KolobokH_1_RIr_1p
ALTIGFDCSWSHSR---NAKQASGEFIYLEELE--GYGHK-A-----VVAFHVVEKSRITTKKG------------KD---GTSEEK-VV---
--------------------IHQG-------NFDA--SSRQME-HAILIALLEQII---------PILEESD------------------
LLLEVCIDGDLDSNKTLANVPI---------------VSEI------------------YADLKHASK-------------
NIRKNLLKKQYAR---------------------------YHNFEQHIMRYF---NGCVFTAGLRKKNN----
DPNAPTNEELRYIQVEGLIQHLLNN--------HDL-----------CW------------KEV-------CWY------------------
------------KENEELQLQAPTLQ---SF-TKTEIEG-----FRQMLLT--
IFKLPIQQSLVTHYRTAYNEAFNR
>KolobokH_2_DiVe_1p
ILPIGFDCSWSHSR---NAHQASGEFIYLGNLP--GYNYQ-P-----VIGFYTVEHSRLSKL--------------------SDESL-KI---
--------------------LHKG-------NFDG--TSRQME-HAILIELLNNIM---------PILEEYD------------------
FTLHICVDGDLETNKTLACIPA---------------VSRI------------------FADLKHVSK-------------
NIRKNLLKKKYSR---------------------------WHSFEQHIMRYF---NSCIFVAGIQKKNN----
QNSAPTEEETRHIQVEGLIQHLHDN--------HNL-----------CW------------SDV-------CWI------------------
------------KDNPELQLQEPTLK---NY-TQTEIVN-----FRNVLMT--
IFRVPFGQGLVTTFRTSYNEAFNR
>KolobokH_3_RIr_1p
VLTVGFDTSWSHVR---NASQASGEFIYHG-IP-EGYTRK-P-----VVGYFVVEKSRVKKD--------------------KNGNK-TT---
--------------------VHQG-------NHEA--SSKQME-HAVLIGILEIVV---------PILEETD------------------
MLLDIVVDGDLDSNKTLRGVKC---------------VNKI------------------FPDLKHLTR-------------
NIRKKLNSKKWER---------------------------YSHYEDVILQYY---KKCIFIAAVQGENK----

KDLPLTTESVKYAQVYGLTKHLCGD--------HSE-----------CW------------PEV-------CWI-------------------
------------AQNPELALCEPNLL---NS-TSEERKK-----FTEMLGE--
VFQLNVGQSLITDARTSQNEAFNR
>Kolobok_1_CCri_1p
APDLSFDGAWSHGR---NASVGYGDLLAHLTEKPDGYVTRFP-----VVGFHVVQKSRHTGLRPVAV---ADSEDAEE---ESSAAD-VV--
YVSFEG---------------EH-G--------VAG--ASQTME-DSCFEAIMPDVN-------KRLVSAR--------------------
KQVTLVCDGDLRLGAKARELDC---------------VRDI------------------GLDLRHFSS--------------
TVKKRLLKAYGENRKTPS--------------------EEHVALIDNVQKHL---LWCYYIG------------
KQNKLSASQFDDAVVGCVDHWRGD--------HSQ-----------CP-----------PSSI-------CVK--------------------
------------SGLGSSYQVPANRSLAS--L-SENSAAL-----LKQRIRA--
ALRREEYQQVLVDANTSINESFHH
>KolobokH_1_GiRo_1p
NLEVSFDCQWSHVR---EAPAASGEFIYNGFLN--ENDHK-P-----IIGFHVAEKSRKYQK--------------------TDGQV-VT---
--------------------INEG-------NHNG--SSSTME-HLILITIIEQIS---------PVLETSE------------------
IVLDVGIDGDLNSNKTLGAQKI---------------VHKI------------------CADLKHKAK--------------NVRAKIAKN--
NK---------------------------WKHLESPIMKYY---VQCVYAATARANDP----NLPTPTEQDLFKMQTEGVIAHLQNN------
--HDD-----------CW------------NEV-------CWF-------------------------------TENPDMILPEPNLI-----
--------------------------------------LY-TKSQCEA-----LLKDLKQ--YMKLT-GQGLITTIRTSANEAVNR
>Kolobok_2_PPac_1p
RIHLCGDGSFDSRG---------YSAAWCRYFLLDAESGV-------ALHYVLIHKS------------------------------------
--------------------------------DTG--SSSTME-VAALERSLNELS--------LMIGGTE-------------------
GIASVVTDRHGSVIKMMRNKFP---------------GIEH------------------YFDPWHFIR--------------
NITLSLLKICKASYMTP-----------------------VRFWVKPIINRC---YDAIVSA---------------
QGNGELASEKFRAIPLCMQGI--------HKFDQDPSFKLFKECT----------------------HSPPTN--------------------
---------------PSIFIPK--GGKILKR-----LEALIFT--
ERNIEDIKSVSWLLQTSPCESINS
>Kolobok_1_AP_2p
DIWLAGDGQYDSPG---------FCAKYCIYSVMDLRSGK-------IVDFKLVQKGM-----------------------------------
-------------------------------------VKGDLE-RKGCELLLNDLT------KNQNF-----------------------
NIKLFLTDRHKGIRFYIRTQHP---------------EIQH------------------EFDVWHLSK--------------
SLMKKMKTLEKKHENA-------------------------YLWKSSVNNHL---WWASQNC---------------
KGDGQLLVEKFTSLLHHIKNE--------HEWEEN---GVTKTCD----------------------HDPLTDE-------------------
------------EINKKLWLKS--DDESYYA-----LKKIITA--
KDFIKDLPHAKHFVHTGRLESYHN
>Kolobok_6_TrFo_1p
---ILFDCAWITRR----------NSRAAFGAFISHGNNK-------VFDFEIAIKNI-----------------------------------
-------------------------------NYIG--TSQHME-AHIFELLSRRWK---------DVD----------------------
IHKYIVKDGDVHIESILKEINW---------------NIEI------------------KLDPNHAFG--------------
IIKKKVKNLAKLH----------------------------NTVFRGITNKI---IRYAQVLIHDTSI-----------
SSEKRIFLWENLLNHLKND--------HSK-----------CI----------------------HPEYR-----------------------
--------------ICWKQSG---DAFAENI-----ISNLINN--TKDN--
IKKIDPLYTTQPSESLHS
>Kolobok_4_TrFo_1p
---CFVDGSWSHPR----------YAKQHTTTIMNLEGK--------IVGQAFFTKPYP------------------------------
GLRGNV-----------------------------NTNV--SPNTLE-IVGIESLRDVIS-------DR------------------------
-KFTGFCSDLDASLWKHVKNMNQ----------------FNR------------------HIDPRHALS--------------
SIKRMLVKINNSTK--------------------------TKGVFSNLIPDF---IRYVKYLIKFEPND------------
QLRVDKYLNIVNHFSRN--------CTPL----------CN----------------------HKTEVD------------------------
-----------KGAYFDES---NPNTKLV-----FIKFLEN--TQKI--
IKFSSQAYSTQKLESFHH
>Kolobok_3_TrFo_1p
---ISFDSAWSHPR----------NAHECFGAFIDLATQK-------IIDYYVVSLK------------------------------------
-----SHWS---IG-----------------NCEG--FPQSLE-SEILNKLSSKWC-------SLS------------------------
KIDSITMDNDNNVGGIFTKLNW---------------KIIQ------------------FIDVNHAYK--------------
QFKNRFLKFNFDN----------------------------KNIFSDIIKSF---LKFVKVLMLDRDL-----------
PLITKKEQFFNITEHFSNN--------HQK-----------CL----------------------HENLIDF---------------------
------------KIPAFAK---NINLKNR-----LDTFLDD--NVII--
FEKTEPHKNTQCNESLHS
>Kolobok_2_TrFo_1p
NLVVSFDGTWDHPR----------FATRCFGALIELTKGK-------IIDFFLILRTNSPID-------------------------------
------HN--FHVS-----------------TTQR--YPQGLE-TEVLRKLAPIWF-------SDN------------------------
KISFFIQDSHNKNPSIFREYNN---------------NMYR------------------KIAINHQYK--------------
RLTNMIKKFISTNK-----------------------------FSKDIQQSL---LNFSRYILFLDASD------------
VKKQELWSNVVNHFAGD--------HTK-----------CI----------------------HSFNQ-------------------------
-------------SFPQL--NEQQISL-----LRQFITL--TFDE--
IICTTRLMHTQYNESLNH
>Kolobok_1_XT1p
AVVLAGDGQFDSPG---------HSAKYCTYTMMDIMTKK-------IVDFTIEQVCP-----------------------------------
---------------------------------GK--TSGQME-TIAFEKCLSSLE-------KKGI-----------------------
DIRVMATDRHSSIRKFMKTKSE---------------TINH------------------QFDVWHICK--------------
SLVKKLTAASKQRKCKD-----------------------IAHWIGPITNHL---WWCSQTC---------------
DQNVENLLDKWRSLLYHIANK--------HTFRNLKTY---KKCQ----------------------HKKITA--------------------
-----------EEMKDKKWITP--SHPAYST-----LVAILTN--
KLLIKDISQIEKFCHTGDLENFHS
>Kolobok_1_BF1p
QVTLLGDGRCDSPG---------HCAKYCSYTLMEEKTQF-------ILDFQLAQVTE-----------------------------------
---------------------------------TG--TSQAME-RHAFEKSLEFVR-------DNGI-----------------------

DVECIVTDRHRGIGASLKQRNN--------------RHINH------------------QYDVFHMAK--------------
SIQKKLSKSAKRKANRA-----------------------LGPWIKFIKNHL---WYSSSTC---------------
EGDDVLLQEKWLSLIDHIANR--------HTFRKN---QLFKKCA----------------------HHRLTP--------------------
-----------DEKENITWLRP---GSAPHRA-----MREIVSN--
KTFVKDMAHLTGFKHTGVLEVYHN
>Kolobok_1_TV1p
---LSIDARWTHRR----------NSPSCTVTALDAVTKR-------VLACVNINHIGGNR-------------------------------
-----QH-----------------------AQYSG--ASNNME-SAGTRIILKQLK-------KYNILK---------------------
DVKEIIKDRDNKSVSVFKEFGV---------------SHLE------------------RFDPGHVSK--------------
NISKDFTKFSASHKTVEV-----FND-KTQKIEK--IERPFWGLNASLSMWL---WSCF-----------------
AEENIDKRTKMWENCVYHYVGN--------HSF-----------CE------------P---------HNYKC--------------------
-----------------FEWQKGV---NSIQLQFM----LYDWVHK--
WTPI--VSKVSSIGSTCMNEAFNS
>Kolobok_2_TV1p
GTVITIDAHFSHVR----------NADECEFVAMGPKGD--------IVAKFVIVRSRKGKAG-----------------------------
-------------------------------NYTG--ASCQME-SVAADQALQILE-------DTVGKG---------------------
IVDGFCHDRDNKSRNIVHKHFP---------------DAVE------------------YQDGFHCKK--------------
KFELCWKRLTSNEYAKSD-----MPI----------V---DSKIFHGIKNRL---KIFFNTLLDKHISK------------
EQKLFYWLHAVDHLIGN--------HS---------PEYCL----------------------HSTK-------------------------
------DEEKEHFIWTAGL-------------------E-------------------------NENARNH-----LLEFLKD--SGSV--
FQIVNPDYATHINESYNA
>Kolobok_2_DR_1p
AVALAGDMRADTPG---------HSAKFGSYTLMDNETNK-------IVDLQLIQSNE----------------------------------
---------------------------------VG--GSYHME-KEGLKRCLDKLD-------ANGL-----------------------
AVDYIVTDRHPQIQKYLRERGI---------------TQ--------------------FYDVWHFEK--------------
GLSKKLEKLSKRKECEV-----------------------LKRWLKSIKNHV---YWSATSS----------------
VSGPEKVAKLTSLLNHIQNI--------HVHNNPLFP----KCE----------------------HPDVLS-R-------------------
-------------DRKKWFQPGIYPKKVYEFTQYWLNVELLF----------------VFHGCIIT-GSQALYK-----VEKVLNN--
KRVVKDVAKLSHHYQTSSLEAFHS
>Kolobok_1_NV1p
DVVLAGDGQCDSPG---------FSAKNLCYFLMEIVTGY-------ILEVEIMDKRH----------------------------------
--------------------------------VNM--KSATME-RKALDNALSRVK---------KVL----------------------
SVTEVCTDASSSIKKMIAEEFK---------------GIFH------------------SLDVWHKAK--------------
SIRKCLLKVSNTRENGK-----------------------VGKWTDHIIRHF---WHCCSVCGEGTST--------
DEEALERLKNQWISLLHHVCNT--------HEWP-------TGKCH----------------------HGDLPD-----E-------------
---------------HELPWFDR--RDKDFQA-----LQKVILE--
PSLLDSFKSYVRFRHTGALECANS
>Kolobok_1_PiPr_1p
DVVVLADGRNDTPG---------HCAQYCSYTTMENDSLE-------IISVVTVDKRQ----------------------------------
--------------------------------TNR--RSAIME-KEAFILTMDQLV--------TEL-----------------------
KLVEICTDAHSQIGALMDPVKGRYK----------DYRIHH------------------SLDMWHGAK--------------
NLAKKIAAAAQVKELSV-----------------------LLLWLKDIVNHF---WWCCKTA----------------
ESYDEFLTLWAGILHHVCNE--------HEWA-------MGSCK----------------------HGQLAD--------------------
-------------SEKQWIQR---DSKAHKA-----LVEIILK--
KRWLKDVHKYLRFRSTADLESFQN
>Kolobok_14_CGi_1p
PLSLAVDVRYDTPG---------FCANKSTAVFMDVNSKH-------IIHLEIGDSRE----------------------------------
--------------------------------VGR--HSPKME-RLLIERGLNYIL---------NVSPY--------------------
VVWEIISDASRNIISMMRTDPF--------------KHLQH------------------SLDIWHKAK--------------
KLAFLLGEIAKKAANKD-----------------------LLPWIRPIINHF---WYCCSAS---------------
KGNVEKLLKKWFGILHHVTNQ--------HIWP-------GGRCH----------------------HTENNL--------------------
----------ESLSSNRKWLHR---NSSALQE-----LRKAITN--
RDWCGSMAFYVNCRQTWAIENFFS
>Kolobok_1_CaNi_1p
DLDLAGDAMFDHPG---------HTAEHSRYAILDVDTNF-------VLEAELLRKTSKS--------------------------------
-----------------------------NIFLSE--SSKSLE-PKSLDLALTKLQ--------ESLKLPDG----TERI----------
GIGSITTDRDPSVAKLLAAKYP---------------QIRP------------------FYDGWHFAR--------------
NVKKSIWKVRFSXKLNQKQMS-P-----------------VKTWIRNLNNHL---YHSFSSS---------------
AGDGKLAVEKFVSFFLHMQGI--------HDNGFK--FHLINKCD---------------------HGALSN-K------------------
---------------RSDYVDLKNK---K-HXNAFNL-----LWKMVAT--
EKRLKDLEKVSPFHSTSEVESFNS
>Kolobok_5_TV1p
KFNVSLDCAWSSKR----------DALHAVITLIDINTNK-------ILDFRIVSRHPELIKF----------------------------
KSPK-ILV-------------------------EPKI--SPGLME-SYALRDMLKSDL------------WK--------------------
KIKVFCSDGDVKDSTMIENSGA---------------QLSH------------------VRDPNHVIK--------------
SAFSKIVTKYPKFE------------------------KI---FR-ILFACPT-------------------------LKPEQKVQRWMG-
LINRYPGE--
---SPEHIEK------REIITN--IAPL--
FLRISKQYHTNFNEAFNS
>Kolobok_6_TV_1p
--KAAYDCAWTGRR----------NAFSSFSSLANIVLNK-------ICGFRIVTKYKIDGYKT----------------------------
---VIS-------------------------DPDI--QANMME-ITALNSIINSDI---FP-----------------------------
KIHTFVSDGDVKTNTALNHSKT---------------SIQI------------------TKDSNHLIK--------------
NAFNKFKDQLHFLESQ----------------------------FR-IIFADNT-------------------------
LTIEQKVQKWLDTKKKYVNR--
---QNYSE-----ICETIDA--TAFL-
-FRCIRKGLHTNFNESINA

```
>Kolobok_5_TrFo_1p
---VAIDGSWSSRR----------NAQFCIVDAISIETKK-------IIDFEIVIKSTSKIK-------------------------------
------------------------------GNYHG--PSNLME-AEGFKRMFPRLL-----NSK--------------------------
KVSSIVIDGDVKIPKLVNQMSP---------------DVQV------------------LRDYTHYFK--------------
NLPNLFKKYNKNTKGK----------------LRGTKTQIINQFKSILFSESN------------------------
VILRYQRIQELKFSIINKIK-------------------------------------------------------------------------
-----------------------------------------------------------------NI-DRNTIDA-----ITSLFGE--
LLAF--SVNFHQGVTTNYCESYHN
```

Supplemental Data S6. KolX protein sequences encoded by *piggyBac* DNA transposons. (fasta format)

```
>piggyBac-2_GrCh_2p
MQAGNHILQENEQAPNMSHFQLCLCNSIKASHCACRSNESHTSKRQYETSQETKTGNMALEPTSDSSTVEVNAVLRFIPPITTHNEDIACHIC
GGTPCYWMQLGPTVVEKVAHCYPALGKTKEDNNHARKSAYKAFTYERHGFLGKGKRVQIPRCALEGIRRLWPDECGAYMGFKEQ
>piggyBac-14_PI_2p
LTVLLKIQNFKLVMVIGEAPSRTCRYCDDEPCEWIQYSRDIIGSRLRMLSCSHRRLTSRQVTRTLSQHYYYKKYGHLSKKTRIRLPFCVERGI
QRLNSTIRYIKRLLLCFLR
```
